# CTRP6 promotes the macrophage inflammatory response, and its deficiency attenuates LPS-induced inflammation

**DOI:** 10.1016/j.jbc.2023.105566

**Published:** 2023-12-14

**Authors:** Cheng Xu, Dylan C. Sarver, Xia Lei, Ageline Sahagun, Jun Zhong, Chan Hyun Na, Assaf Rudich, G. William Wong

**Affiliations:** 1 Department of Physiology, Johns Hopkins University School of Medicine, Baltimore, Maryland, USA; 2 Department of Biochemistry and Molecular Biology, Oklahoma State University, Stillwater, Oklahoma, USA; 3 Delta Omics Inc, Baltimore, Maryland, USA; 4 Department of Neurology, Institute for Cell Engineering, Johns Hopkins University School of Medicine, Baltimore, Maryland, USA; 5 Faculty of Health Sciences, Department of Clinical Biochemistry and Pharmacology, Ben-Gurion University of the Negev, Beer-Sheva, Israel

**Keywords:** CTRP, macrophage, inflammation, signaling, LPS, transcriptomics, phosphoproteomics

## Abstract

Macrophages play critical roles in inflammation and tissue homeostasis, and their functions are regulated by various autocrine, paracrine, and endocrine factors. We have previously shown that CTRP6, a secreted protein of the C1q family, targets both adipocytes and macrophages to promote obesity-linked inflammation. However, the gene programs and signaling pathways directly regulated by CTRP6 in macrophages remain unknown. Here, we combine transcriptomic and phosphoproteomic analyses to show that CTRP6 activates inflammatory gene programs and signaling pathways in mouse bone marrow-derived macrophages (BMDMs). Treatment of BMDMs with CTRP6 upregulated proinflammatory, and suppressed the antiinflammatory, gene expression. We also showed that CTRP6 activates p44/42-MAPK, p38-MAPK, and NF-κB signaling pathways to promote inflammatory cytokine secretion from BMDMs, and that pharmacologic inhibition of these signaling pathways markedly attenuated the effects of CTRP6. Pretreatment of BMDMs with CTRP6 also sensitized and potentiated the BMDMs response to lipopolysaccharide (LPS)-induced inflammatory signaling and cytokine secretion. Consistent with the metabolic phenotype of proinflammatory macrophages, CTRP6 treatment induced a shift toward aerobic glycolysis and lactate production, reduced oxidative metabolism, and elevated mitochondrial reactive oxygen species production in BMDMs. Importantly, in accordance with our *in vitro* findings, BMDMs from CTRP6-deficient mice were less inflammatory at baseline and showed a marked suppression of LPS-induced inflammatory gene expression and cytokine secretion. Finally, loss of CTRP6 in mice also dampened LPS-induced inflammation and hypothermia. Collectively, our findings suggest that CTRP6 regulates and primes the macrophage response to inflammatory stimuli and thus may have a role in modulating tissue inflammatory tone in different physiological and disease contexts.

Macrophages play important and critical roles in both the innate and adaptive immune response, as well as in tissue repair and homeostasis (1, 2, 3, 4). They express a wide variety of cell surface receptors that allow them to sense and respond to environmental cues derived from the host or pathogens (5, 6). Given their pleiotropic roles, macrophages can be functionally grouped into different subsets based on specific cell surface markers and the types of mediators they secrete (7, 8).

Among the different subsets based on initial studies, the classically activated M1 macrophages are generally thought to promote inflammation by secreting proinflammatory cytokines, whereas the alternatively activated M2 macrophages dampen inflammation by producing antiinflammatory cytokines (2, 7). While not mutually exclusive, M1 macrophages rely heavily on aerobic glycolysis whereas M2 macrophages primarily utilize mitochondrial oxidative metabolism to fuel their energetic needs (9, 10, 11). However, with the increasing recognition that there is a wide spectrum of macrophage functional phenotypes distinct from the classical M1 or M2, or along the continuum of M1 to M2, has led to a more nuanced appreciation of macrophage functional heterogeneity and plasticity (12, 13, 14).

In the contexts of murine models of obesity, 40 to 50% of cells within the adipose compartment are macrophages derived from both circulating monocytes and the locally proliferating resident macrophages (15, 16, 17). Although less dramatic, macrophage infiltration into different fat depots has also been noted in human obesity (18). These infiltrating and locally proliferating macrophages often promote chronic low-grade inflammation within the adipose compartment (15, 16, 17, 19). This could disrupt normal adipocyte function and dysregulate the adipokine secretory profile, contributing to impaired local and systemic metabolism (20, 21). However, a homeostatic role of the infiltrating macrophages has also been noted in the context of weight loss, where elevated lipid flux due to enhanced lipolysis in adipose tissue promotes the recruitment of macrophages, which then turn into lipid-laden macrophages to help buffer the rise in local lipid levels (22).

We have previously shown that C1q/tumor necrosis factor (TNF)-related protein 6 (CTRP6; also known as C1qtnf6)—a secreted protein of the C1q family (23, 24)—is one of the autocrine and paracrine factors that regulates macrophage function in obesity (25). CTRP6 expression is upregulated in obesity and acts on adipocytes and macrophages to promote inflammation within fat depots (25). While both adipocytes and macrophages produce CTRP6 in the fat pads of diet-induced obese mice, a proportionally higher transcript expression is found in the nonadipocyte fraction that comprises the infiltrated macrophages (25). Mice lacking CTRP6 have reduced adipose tissue inflammation and an improved systemic metabolic profile; conversely, overexpression of CTRP6 in mice impairs insulin sensitivity (25). In chronic obesity, CTRP6-mediated inflammation has a detrimental role; however, in the context of an acute short-term (3-days) caloric overload, upregulated CTRP6 expression promotes a homeostatic inflammatory response in the fat compartment to limit excess fat storage (26). Thus, the physiological outcomes of CTRP6-mediated inflammatory responses are context-dependent.

While we know macrophages are one of the cell targets of CTRP6, the gene programs and signaling pathways directly regulated by CTRP6 in macrophages to promote a proinflammatory response has not been determined and is the focus of the present study. Combining transcriptomic and phosphoproteomic analyses with cell signaling and respirometry studies, we directly demonstrated that CTRP6 promotes a proinflammatory phenotype in bone marrow-derived macrophages (BMDMs). Consistent with our *in vitro* findings, BMDMs derived from *Ctrp6* KO mice had lower basal and lipopolysaccharide (LPS)-stimulated inflammatory cytokine gene expression and secretion. Further, mice deficient in CTRP6 had lower circulating TNF-α levels and an attenuated hypothermic response induced by an acute LPS challenge. Collectively, these results highlight the role of CTRP6 as a physiologic modulator of tissue inflammatory tone by sensitizing and potentiating macrophage’s response to inflammatory stimuli.

## Results

### CTRP6 promotes a macrophage proinflammatory phenotype

**Figure 1:**
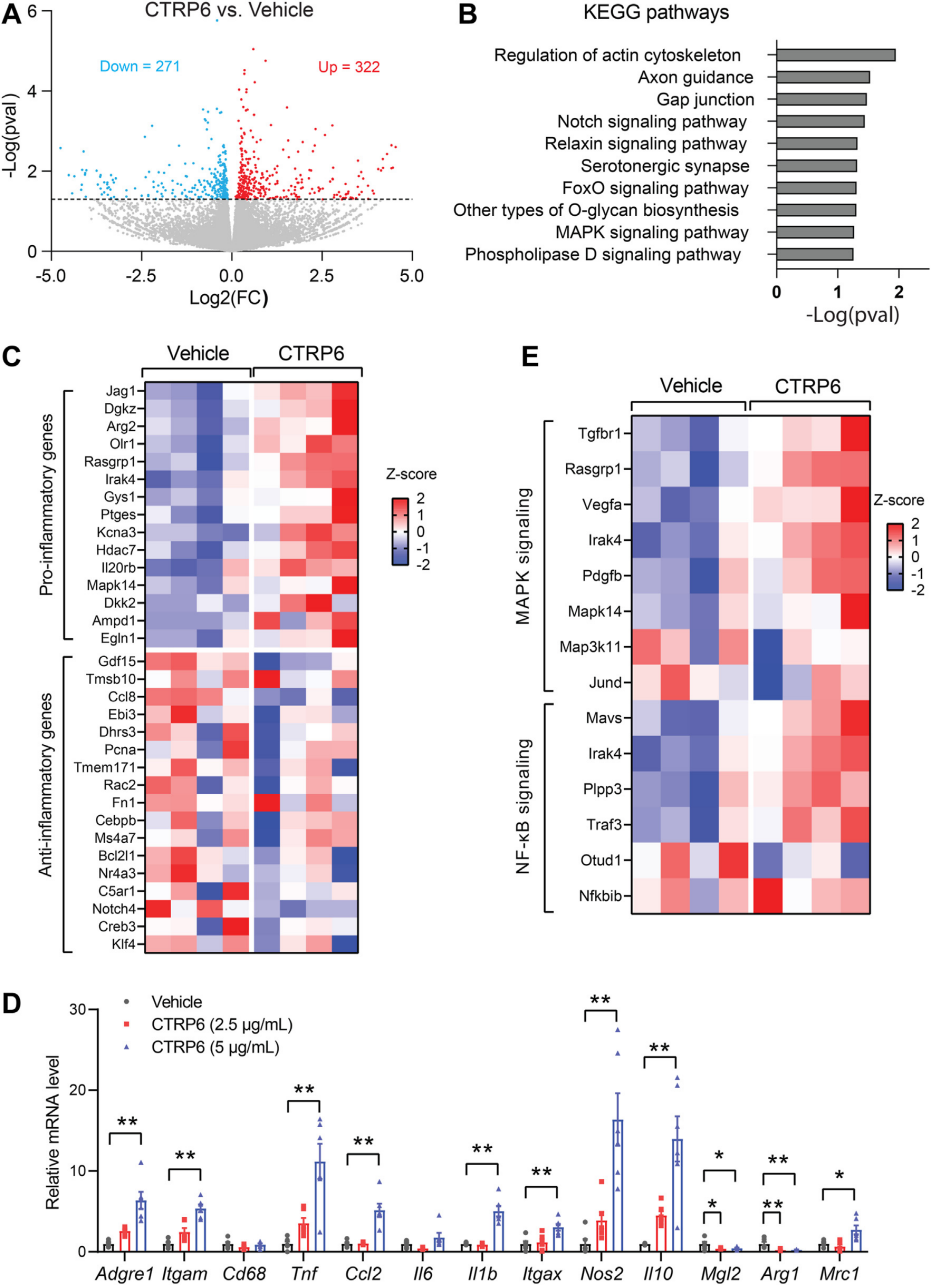
**CTRP6 promotes a macrophage proinflammatory phenotype.***A*, a volcano plot showing significantly upregulated and downregulated genes in BMDMs treated with CTRP6 or vehicle control. *B*, the top upregulated pathways in CTRP6-treated BMDMs, as indicated by the KEGG pathway analysis. *C*, a heatmap showing some of the differentially expressed proinflammatory and antiinflammatory genes obtained from RNA-seq in CTRP6-treated BMDMs. Color gradient reflects row Z-score values. *D*, relative mRNA expression of proinflammatory and antiinflammatory genes in BMDMs treated with CTRP6 for 6 h (n = 6). Data are shown as the mean ± SEM. Representative of three independent experiments. One-way ANOVA followed by Tukey’s post hoc test. ∗*p* < 0.05; ∗∗*p* < 0.01. *E*, a heatmap showing differentially expressed genes (from RNA-seq) involved in MAPK and NF-κB signaling pathways in CTRP6-treated BMDMs. Color gradient reflects row Z-score values. BMDM, bone marrow-derived macrophages; KEGG, Kyoto Encyclopedia of Genes and Genomes; MAPK, mitogen-activated protein kinase.

To better understand how CTRP6 regulates macrophage function, we performed an unbiased transcriptomics analysis of genes that are regulated by this secreted protein in BMDMs. Bulk RNA sequencing was carried out on BMDMs stimulated with vehicle control or recombinant CTRP6 for 6 h. The short time frame allowed us to focus on differentially expressed genes (DEGs) likely to be directly regulated by CTRP6. Of the DEGs (*p*-value < 0.05 and log2FoldChange >/<0), there were a total of 322 genes upregulated and 271 genes downregulated in BMDMs upon CTRP6 treatment (Fig. 1*A* and Table S1 and S2). Kyoto Encyclopedia of Genes and Genomes (KEGG) pathway enrichment analysis revealed that those DEGs were enriched for signaling pathways known to promote a proinflammatory macrophage phenotype, such as the Notch (27), FoxO (28), and mitogen-activated protein kinase (MAPK) (29, 30) signaling pathways (Fig. 1*B*).

Among the upregulated transcripts induced by CTRP6 were *Jag1*, *Dgkz*, *Arg2*, and *Olr1* which have been shown to be associated with proinflammatory macrophages (Fig. 1*C*) (31, 32, 33, 34). Conversely, some of the antiinflammatory genes associated with macrophages (*e.g.*, *Gdf15*, *Tmsb10*, and *Ccl8*) (35, 36, 37) were downregulated in CTRP6-treated BMDMs (Fig. 1*C*). In addition to RNA sequencing, we also performed real-time PCR to further confirm that many of the proinflammatory genes (*e.g.*, *Tnf*, *Ccl2*, *Il1b*, *Itgax*, and *Nos2*) were indeed upregulated, and antiinflammatory genes (*i.e.*, *Mgl2* and *Arg1*) downregulated, by CTRP6 treatment in BMDMs (Fig. 1*D*). Interestingly, some antiinflammatory genes (*e.g.*, *Il10*, *Mrc1*) were also being upregulated by CTRP6.

In addition to the classic inflammatory genes, transcripts that encode proteins involved in inflammatory MAPK signaling (*e.g.*, *Rasgrp1*, *Irak4*, *Mapk14*, and *Map3k11*) and NF-κB signaling (*e.g.*, *Mavs*, *Irak4*, *Plpp3*, and *Traf3*) were also upregulated in BMDMs by CTRP6 treatment (Fig. 1*E*). Together, these results indicate that CTRP6 treatment alters the expression of many immune-related genes, changes consistent with promoting a more proinflammatory profile in BMDMs.

### CTRP6 activates the MAPK and NF-κB signaling pathways to promote macrophage inflammatory response

**Figure 2:**
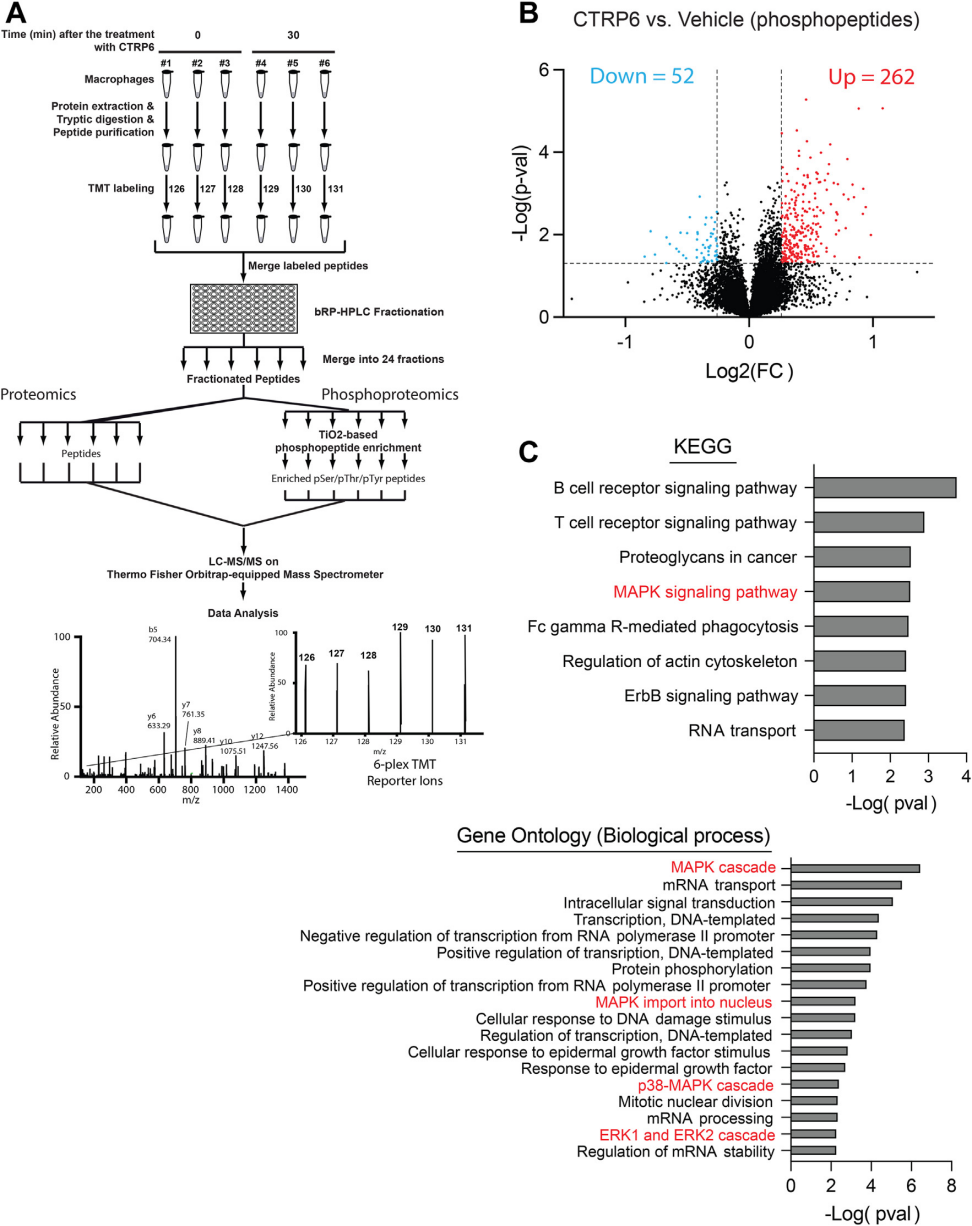
**Phosphoproteomic analysis reveals enrichment for MAPK signaling induced by CTRP6 in macrophages.***A*, schematic illustration of TMT 6 plex-based workflow for quantitative phosphoproteomic analysis [see details in Experimental procedures]. *B*, a volcano plot showing all the phosphopeptides that are upregulated or downregulated by CTRP6-treated BMDMs. Fold-change cutoff was placed on ±1.2-fold, as explained in Result section. *C*, Kyoto Encyclopedia of Genes and Genomes (KEGG) pathway enrichment analysis (*top*) and Gene Ontology biological process analysis (*bottom*) for phosphoproteins with significantly altered phosphorylation level. BMDM, bone marrow-derived macrophage; MAPK, Mitogen-activated protein kinase; TMT, tandem mass tag.

In parallel to our transcriptomic analysis, we also performed unbiased quantitative proteomic and phosphoproteomic analyses to determine what proximal signaling events are directly regulated by CTRP6 in BMDMs (Fig. 2*A*). As expected, we did not observe a significant change in BMDMs at the proteomic level after CTRP6 treatment for 30 min. Of the 6993 quantitated proteins, 6989 showed <1.2 fold change in CTRP6-treated BMDMs compared with vehicle-treated ones (Table S3) while only four proteins showed >1.2 fold change with significance of *p* <0.05. Thus, we chose 1.2 fold change as a cutoff for CTRP6-regulated phosphorylation. Out of 11,588 quantitated phosphopeptides, the phosphorylation levels on 314 peptides were significantly changed (*p* < 0.05) after CTRP6 treatment (Fig. 2*B*), among which 262 were upregulated and 52 were downregulated (Table S4). A partial list of the upregulated and downregulated phosphoproteins is shown in Tables 1 and 2. Mapk1-, MapK3-, MAPK-activated protein kinase 2 (Mapkapk2), and TNF receptor (TNFR) associated factor family member-associated NF-κB activator (Tank) were among the phosphoproteins upregulated by CTRP6 treatment in BMDMs (Table 1). Of the phosphoproteins that were significantly changed, KEGG pathway analysis revealed an enrichment for the MAPK signaling pathway (Fig. 2*C*). Gene Ontology analysis also confirmed that MAPK, extracellular regulated protein kinase (ERK1 and ERK2), and p38-MAPK pathways were among the top enriched biological processes (Fig. 2*C*).

**Table 1:** A partial list of CTRP6-upregulated phosphoproteins in BMDMs

Gene symbol	Protein	UniProt accession #	Phosphosite	Fold change	*p*-value
Ddx17	DEAD (Asp-Glu-Ala-Asp) box polypeptide 17, isoform CRA_a	Q3U741	S494	2.11	8.66E-06
Setd1a	Histone-lysine N-methyltransferase SETD1A	E9PYH6	T1103, S1109	1.98	0.0102
Lrrc41	Leucine-rich repeat-containing protein 41	Q8K1C9	S326	1.92	0.0021
Tank	TRAF family member-associated NF-kappa-B activator	P70347	T301, S302	1.89	0.0008
Elmsan1	ELM2 and Myb/SANT-like domain-containing 1	E9Q2I4	S564	1.89	0.0031
Dhx29	ATP-dependent RNA helicase DHX29	Q6PGC1	S69	1.85	0.0355
Mapk1	Mitogen-activated protein kinase 1	P63085	T183	1.85	0.0000
Nelfe	Negative elongation factor E	P19426	S51	1.80	0.0012
Nop9	Nucleolar protein 9	Q8BMC4	S631	1.78	0.0006
Trim47	E3 ubiquitin-protein ligase TRIM47	Q8C0E3	T100	1.75	0.0014
Gigyf2	GRB10-interacting GYF protein 2 (Fragment)	G3UZS9; Q6Y7W8	S4, S202	1.73	0.0001
Stac2	SH3 and cysteine-rich domain-containing protein 2	Q8R1B0	S268	1.72	0.0070
Mki67	Proliferation marker protein Ki-67	E9PVX6	S125	1.70	0.0011
Znf703	Zinc finger protein 703	P0CL69	S191	1.65	0.0080
Psmd11	26S proteasome non-ATPase regulatory subunit 11	Q8BG32	S14	1.64	0.0006
Pttg1	Securin	Q9CQJ7	S100	1.64	0.0032
Pnn	Pinin	Q3TUQ5	S66	1.63	0.0029
Pnn	Pinin	Q3TUQ5	S66	1.63	0.0029
Ankrd50	Ankyrin repeat domain 50 (Fragment)	F7BE84	S1100	1.63	0.0045
Gemin4	Gem (Nuclear organelle) associated protein 4	Q6P6L6	S84	1.63	0.0098
Dennd4b	DENN domain-containing protein 4B	A0A0R4J172	S727	1.61	0.0335
Ulk1	Serine/threonine-protein kinase	Q6PB82	S752, S766, T769	1.61	0.0006
Piezo1	Piezo-type mechanosensitive ion channel component 1	E2JF22	S1600	1.58	0.0018
Stmn1	Stathmin	P54227	S25	1.57	0.0001
Bod1l	Biorientation of chromosomes in cell division protein 1-like 1	E9Q6J5	T2890	1.57	0.0002
Vim	Vimentin	P20152	S430	1.56	0.0003
Rif1	Telomere-associated protein RIF1	Q6PR54	S1565	1.55	0.0008
Mapk3	Mitogen-activated protein kinase	D3Z3G6	T203, Y205	1.54	0.0259
Dcaf17	DDB1- and CUL4-associated factor 17	Q3TUL7	S412	1.54	0.0211
Dok1	Docking protein 1	P97465	S269	1.52	0.0030
Tbc1d5	TBC1 domain family member 5	A0A286YDB3	S600, T606	1.50	0.0166
Zdhhc5	Palmitoyltransferase ZDHHC5	Q8VDZ4	S636	1.50	0.0001
Sqstm1	Sequestosome-1	Q64337	S308	1.49	0.0026
Kank2	KN motif and ankyrin repeat domain-containing protein 2	Q8BX02	S246	1.49	0.0065
Ppp1r12a	Protein phosphatase 1 regulatory subunit 12A	Q9DBR7	S507	1.49	0.0005
Toe1	Target of EGR1 protein 1	Q9D2E2	S349	1.48	0.0005
Lrch4	Leucine-rich repeat and calponin homology domain-containing protein 4	Q921G6	S512, S517, S518	1.48	0.0334
Trafd1	TRAF-type zinc finger domain-containing protein 1	Q3UDK1	T277	1.47	0.0111
Ppp1r12a	Protein phosphatase 1 regulatory subunit 12A	Q9DBR7	S507	1.47	0.0003
Phf8	Histone lysine demethylase PHF8	Q80TJ7	S984	1.47	0.0022
Ppp1r9b	Neurabin-2	Q6R891	S100	1.46	0.0336
Slc9a7	Sodium/hydrogen exchanger 7	Q8BLV3	S694	1.46	0.0036
Fyb1	FYN-binding protein 1	O35601	S201	1.46	0.0096
Rhbdf2	Inactive rhomboid protein 2	Q80WQ6	S83	1.46	0.0004
Nucks1	Nuclear ubiquitous casein and cyclin-dependent kinase substrate 1	Q80XU3	S181	1.46	0.0086
Cdkn2aip	CDKN2A-interacting protein	Q8BI72	S124	1.46	0.0003
Usp5	Ubiquitin carboxyl-terminal hydrolase 5	P56399	T623	1.45	0.0255
Ncoa7	Nuclear receptor coactivator 7	Q6DFV7	S92	1.45	0.0010
Vamp4	Vesicle-associated membrane protein 4	O70480	S30	1.44	0.0457
Itpkb	Inositol 1,4,5-trisphosphate 3-kinase B	B2RXC2	S42, S48	1.44	0.0036
Ptpn21	Tyrosine-protein phosphatase nonreceptor type	G5E8J4	S799	1.44	0.0463
Fndc3a	Fibronectin type-III domain-containing protein 3A	Q8BX90	S244	1.44	0.0311
Slc12a6	Solute carrier family 12 member 6	Q924N4	S1029, S1032	1.43	0.0188
Riox1	Ribosomal oxygenase 1	Q9JJF3	S107	1.43	0.0025
Myo9b	Unconventional myosin-IXb	E9PZW8; A0A1D5RLD1	T1910, T1882	1.43	0.0053
Fndc3a	Fibronectin type-III domain-containing protein 3A	Q8BX90	S244	1.43	0.0274
Svil	Supervillin	A0A1B0GS91	S307, S314	1.42	0.0089
Osbpl3	Oxysterol-binding protein	D3YTT6	S33	1.42	0.0218
Mtdh	Protein LYRIC	Q80WJ7	S297	1.41	0.0069
Anks1	Ankyrin repeat and SAM domain containing 1	Q3UHP6	S640, T648	1.41	0.0459
Gle1	Nucleoporin GLE1	Q8R322	S352	1.41	0.0001
Cytip	Cytohesin-interacting protein	Q91VY6	S242	1.41	0.0131
Tpr	Nucleoprotein TPR	F6ZDS4	S2223	1.41	0.0006
Erbin	Erbb2ip protein	B7ZNX6	S1125	1.40	0.0023
Svil	Supervillin	A0A1B0GS91	S307	1.40	0.0451
Mepce	7SK snRNA methylphosphate capping enzyme	Q8K3A9	S126	1.40	0.0016
Tcf4	Transcription factor 4	E9Q8G4	S568	1.40	0.0220
Lrrfip1	Leucine-rich repeat flightless-interacting protein 1	Q3UZ39	S284	1.40	0.0307
Dennd5a	DENN domain-containing protein 5A	Q6PAL8	S193	1.39	0.0060
Fhod1	FH1/FH2 domain-containing protein 1	Q6P9Q4	T499, S502	1.39	0.0079
Iqgap2	Ras GTPase-activating-like protein IQGAP2	Q3UQ44	S16	1.39	0.0193
Reps1	RalBP1-associated Eps domain-containing protein 1	O54916	S273	1.39	0.0459
Herc1	HECT and RLD domain-containing E3 ubiquitin protein ligase family member 1	E9PZP8	S1491	1.39	0.0004
Zfp36	mRNA decay activator protein ZFP36	P22893	S52	1.39	0.0004
P2ry10b	Purinergic receptor P2Y, G-protein-coupled 10B	Q8BY68	S337	1.39	0.0420
Cdc42ep4	Cdc42 effector protein 4	Q9JM96	S302	1.39	0.0416
Golga5	Golgin subfamily A member 5	Q9QYE6	S150	1.38	0.0225
Ubash3b	Ubiquitin-associated and SH3 domain-containing protein B	Q8BGG7	S366	1.38	0.0063
Rhbdf2	Inactive rhomboid protein 2	Q80WQ6	S60	1.38	0.0028
Usp24	Ubiquitin carboxyl-terminal hydrolase 24	E9PV45	S1283	1.38	0.0001
Hnrnph1	Heterogeneous nuclear ribonucleoprotein H	Q8C2Q7	S23	1.38	0.0246
Mapk3	Mitogen-activated protein kinase	D3Z3G6	Y205	1.35	0.0006
Ppp1r12a	Protein phosphatase 1 regulatory subunit 12A	Q9DBR7	S507	1.35	0.0011
Mapkapk2	MAP kinase-activated protein kinase 2	P49138	T320	1.29	0.0075
Tulp4	Tubby-related protein 4	Q9JIL5	S1444	1.38	0.0000

Abbreviation: TRAF, TNF receptor (TNFR) associated factor.

**Table 2:** A partial list of CTRP6-downregulated phosphoproteins in BMDMs

Gene symbol	Protein	UniProt accession #	Phosphosite	Fold change	*p*-value
Ogfr	Opioid growth factor receptor	Q99PG2	S616	0.557	0.0342
Tuba1b	Tubulin alpha-1B chain	P05213	S439	0.576	0.0083
Sec16a	Protein transport protein sec16	A2AIX1	S422	0.590	0.0305
Abr	Active breakpoint cluster region-related protein	Q5SSL4	S53	0.629	0.0486
Trim35	Tripartite motif-containing 35	A0A0R4J031	S23	0.629	0.0117
Brca1	Breast cancer type 1 susceptibility protein homolog	P48754	S1479	0.642	0.0169
Abhd2	Monoacylglycerol lipase ABHD2	Q9QXM0	S415	0.665	0.0221
Mef2a	Myocyte-specific enhancer factor 2A	Q60929	T413	0.703	0.0374
Arhgef11	Rho guanine nucleotide exchange factor (GEF) 11	Q68FM7	S1321	0.707	0.0091
Rps6kc1	Ribosomal protein S6 kinase delta-1	E9QMX4	S492, S493	0.717	0.0038
Brd1	Bromodomain-containing 1	E9PZ26	S1186	0.746	0.0233
Tcf20	Transcription factor 20	Q9EPQ8	S567	0.746	0.0358
Kmt2d	Histone-lysine N-methyltransferase 2D	A0A0A0MQ73	S1136	0.747	0.0132
Med13 l	Mediator of RNA polymerase II transcription subunit 13	A0A0J9YUA8	S918	0.749	0.0088
Rsf1	Remodeling and spacing factor 1	E9PWW9	S1286	0.758	0.0012
Samd4b	Protein Smaug homolog 2	G5E8A7	S271	0.763	0.0470
Rasal2	RAS protein activator-like 2	E9PW37	T131	0.766	0.0281
Htt	Huntingtin	G3X9H5	S2917	0.771	0.0437
Map4	Microtubule-associated protein 4	P27546	S345	0.773	0.0176
Psmd4	26S proteasome non-ATPase regulatory subunit 4	O35226	T250	0.773	0.0439
Map4k4	Mitogen-activated protein kinase kinase kinase kinase 4	A0A0A6YW53; A0A0A6YWM8; F8VPL5	S684, S607, S638	0.776	0.0360
Pitpna	Phosphatidylinositol transfer protein alpha isoform	J3QPW1	Y142	0.780	0.0406
Cic	Protein capicua homolog	Q924A2	S2311	0.782	0.0208
Cic	Protein capicua homolog	Q924A2	S2311	0.783	0.0057
Igf2bp1	Insulin-like growth factor 2 mRNA-binding protein 1	O88477	S181	0.786	0.0039
Skil	Ski-like protein	Q60665	S507	0.791	0.0135
Tanc1	Protein TANC1	Q0VGY8	S267	0.794	0.0464
Cbl	E3 ubiquitin-protein ligase CBL	P22682	S907	0.796	0.0253
Golga2	Golgin subfamily A member 2 (Fragment)	Z4YJU8	S748	0.797	0.0309
Gabpb2	GA-binding protein subunit beta-2	P81069	S218	0.797	0.0455
Lrch4	Leucine-rich repeat and calponin homology domain-containing protein 4	Q921G6	S313	0.797	0.0201
Arhgap31	Rho GTPase-activating protein 31	A6X8Z5	S1413	0.800	0.0210
Mef2c	Myocyte-specific enhancer factor 2C	A0A0H2UH28	S118	0.805	0.0185
Znf609	Zinc finger protein 609	Q8BZ47	S358	0.807	0.0112
Tomm34	Mitochondrial import receptor subunit TOM34	Q9CYG7	S186	0.808	0.0093
Aggf1	Angiogenic factor with G patch and FHA domains 1	Q7TN31	S308	0.810	0.0039
Map4	Microtubule-associated protein 4	P27546	S345	0.811	0.0428
Dcbld2	Discoidin, CUB and LCCL domain-containing protein 2	Q91ZV3	S719, S722	0.815	0.0097
Numa1	Nuclear mitotic apparatus protein 1	E9Q7G0	S167	0.829	0.0256

**Figure 3:**
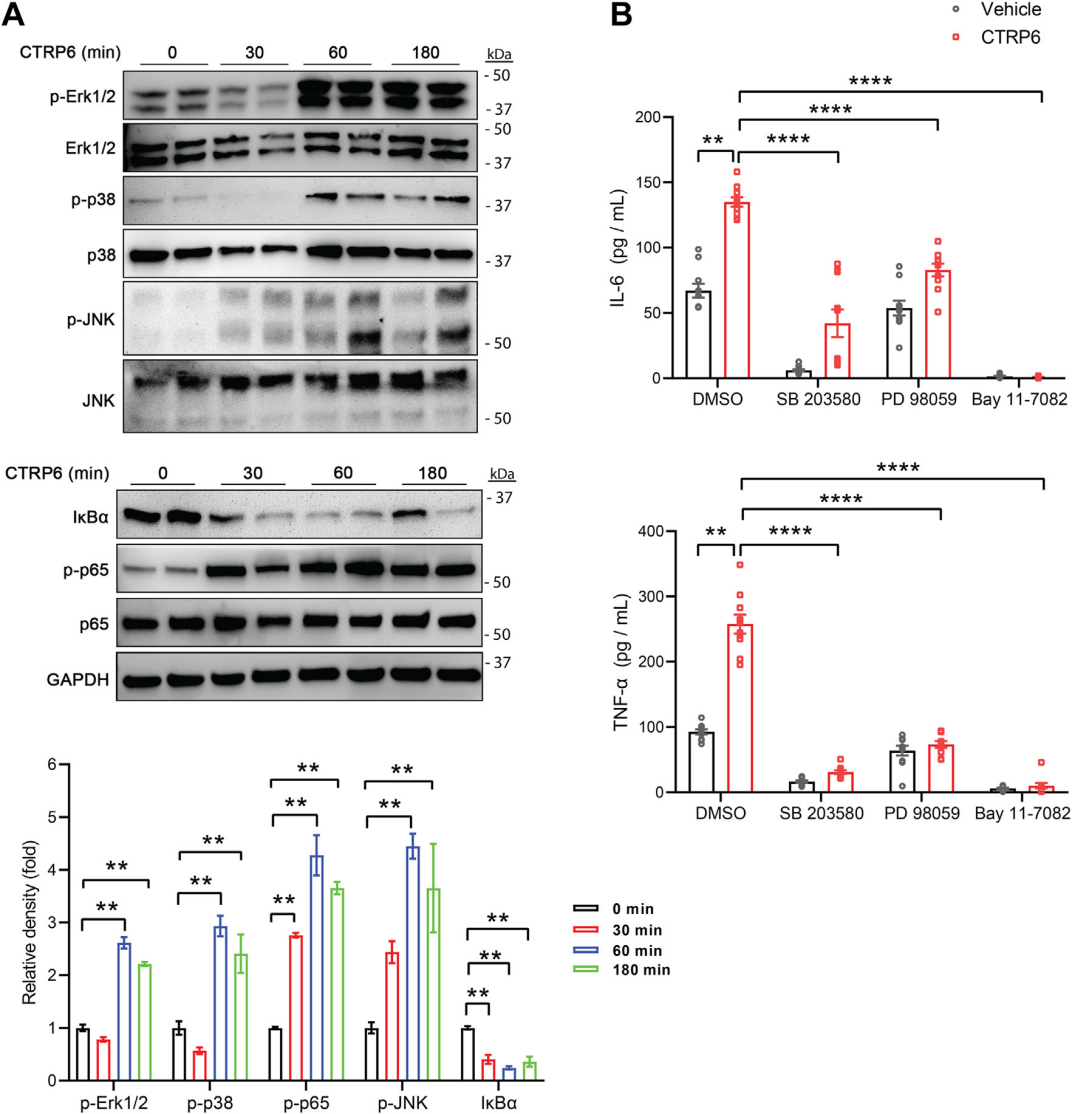
**CTRP6 activates the MAPK and NF-κB signaling pathways to promote a proinflammatory response in macrophages.***A*, Western blot analysis of MAPK and NF-κB signaling pathways activated by CTRP6 in BMDMs over a 180-min time course. Phospho (P) and total Erk1/2, p38-MAPK, JNK, IκB, p65 subunit of NF-κB are indicated. The band densities of phospho-Erk1/2, p38-MAPK, JNK, and p65 were first normalized to their corresponding total protein. In the case of IκB, it was normalized to GAPDH. The relative band density shown at 30, 60, and 180 min was normalized to the band density at time 0 (which was set to 1). Data are shown as the mean ± SEM. Representative of two independent experiments. One-way ANOVA followed by Tukey’s post hoc test. ∗∗*p* < 0.01. *B*, secretion of cytokines from BMDMs pretreated with p38 MAPK inhibitor SB203580, MEK/Erk1/2 inhibitor PD98059, and NF-κB inhibitor Bay 11-7082 for 1 h, followed by cotreatment with CTRP6 for 24 h (n = 10). Data are shown as the mean ± SEM. Representative of three independent experiments. Two-tailed unpaired Student’s *t* test. ∗∗*p* < 0.01; ∗∗∗∗*p* < 0.0001. BMDM, bone marrow-derived macrophage; MAPK, mitogen-activated protein kinase. JNK, c-Jun N-terminal kinase.

MAPK and NF-κB are the two major signaling pathways involved in macrophage proinflammatory activation (38, 39, 40, 41). We therefore performed immunoblot analyses to further determine whether and to what extent these signaling pathways contribute to the CTRP6-induced inflammatory response in macrophages. We observed that CTRP6 induced a rapid and robust phosphorylation and activation of ERK1/2, p38-MAPK, and c-Jun N-terminal kinase signalings in BMDMs (Fig. 3*A*). We also noted a rapid activation of the NF-κB signaling pathway, as shown by the time-dependent degradation and reduction of inhibitor of κB (IκBα) and the corresponding increase in p65 (RelA, a subunit of NF-κB) phosphorylation (Fig. 3*A*).

Next, we determined whether the activation of MAPK and NF-κB signaling by CTRP6 is directly linked to inflammatory cytokine (TNF-α and interleukin 6 [IL-6]) secretion by BMDMs. Treatment of BMDMs with CTRP6 resulted in a robust and significantly higher amount of TNF-α and IL-6 being secreted into the conditioned medium (Fig. 3*B*). However, pretreating BMDMs with inhibitors specific for MEK/Erk1/2 (PD98059), p38-MAPK (SB203580), and NF-kB (Bay 11-7082) abolished TNF-α and markedly reduced IL-6 secretion (Fig. 3*B*). Together, these results indicate that CTRP6 engages the MAPK and NF-κB signaling pathways to promote inflammatory cytokine secretion from macrophages.

### CTRP6 augments LPS-stimulated inflammatory response in macrophages

**Figure 4:**
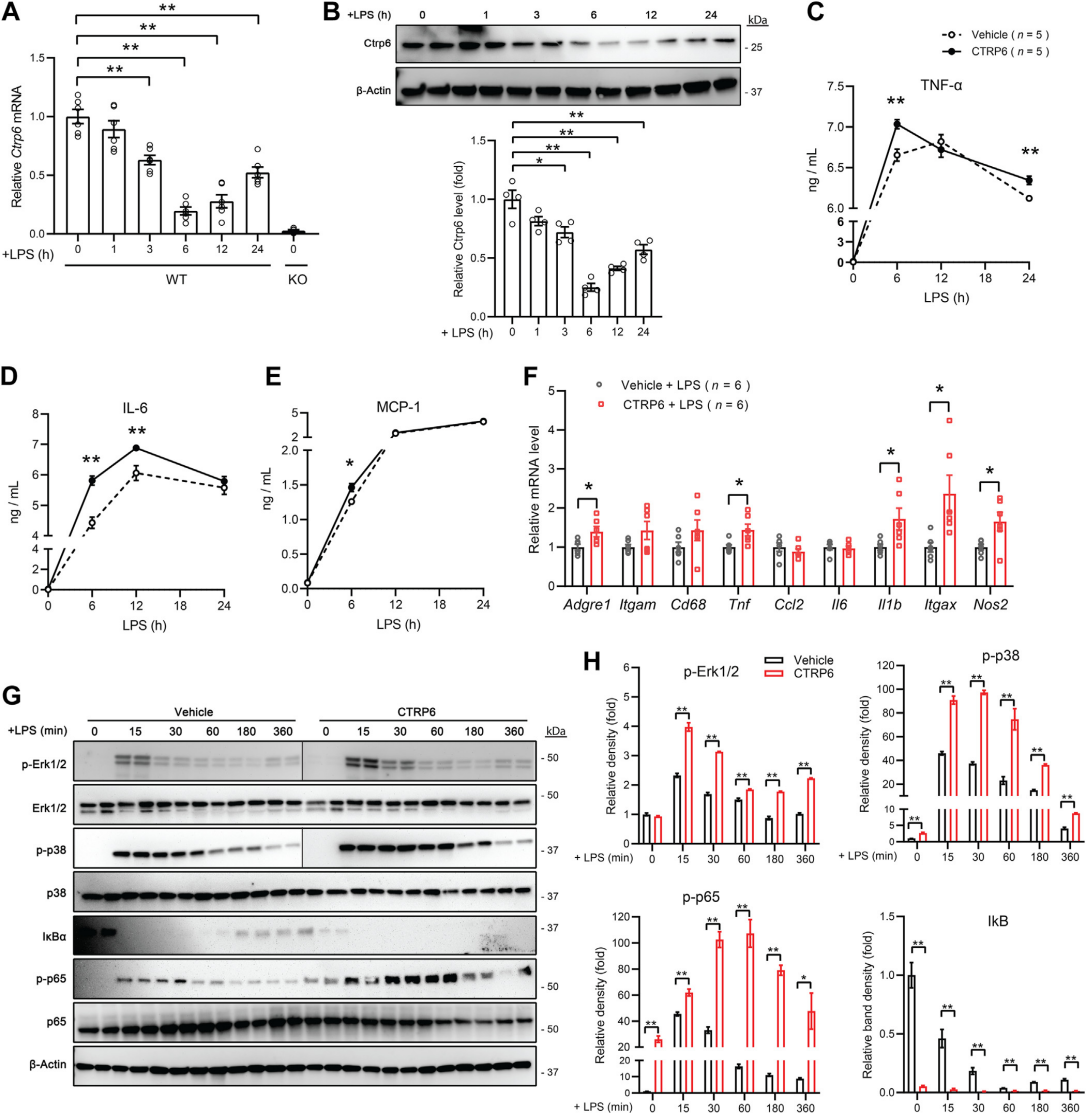
**CTRP6 augments LPS-stimulated inflammatory response in macrophages.***A*, *Ctrp6* expression in BMDMs stimulated with LPS (100 ng/ml) for 0 to 24 h (n = 6). Data are shown as the mean ± SEM. Representative of two independent experiments. One-way ANOVA followed by Tukey’s post hoc test. ∗∗*p* < 0.01. BMDMs from *Ctrp6* KO mice were used as a negative control. *B*, Western blot analysis of Ctrp6 in BMDMs stimulated with LPS (100 ng/ml) for 0 to 24 h (*top panel*). Band intensities were quantified and shown on the *bottom panel* (n = 4). Data are shown as the mean ± SEM. Representative of two independent experiments. One-way ANOVA followed by Tukey’s post hoc test. ∗*p* < 0.05, ∗∗*p* < 0.01. *C*–*E*, secretion of TNF-α (*C*), IL-6 (*D*), and MCP-1 (*E*) from BMDMs pretreated with CTRP6 (5 μg/ml) for 1 h, followed by LPS (100 ng/ml) stimulation for 0 to 24 h in the presence of CTRP6. Data are shown as the mean ± SEM. Representative of two independent experiments with five samples per group. Two-tailed unpaired Student’s *t* test. ∗*p* < 0.05, ∗∗*p* < 0.01. *F*, expression of proinflammatory genes in BMDMs pretreated with CTRP6 for 1 h, followed by LPS stimulation for 24 h in the presence of CTRP6 (n = 6). Data are shown as the mean ± SEM. Representative of three independent experiments. Two-tailed unpaired Student’s *t* test. ∗*p* < 0.05. *G*, Western blot analysis of MAPK and NF-κB signaling pathways activated in BMDMs by LPS alone, or LPS in combination with CTRP6 (pretreatment for 1 h and thereafter). Phospho (P) and total Erk1/2, p38-MAPK, IκB, p65 subunit of NF-κB are indicated. For the phospho-Erk1/2 and phospho-p-38 blots, original sample loading was in reverse order. For consistency, we switched the group such that vehicle-treated samples came before the CTRP6-treated samples. We inserted a line on the gel images to indicate where splicing occurred. *H*, the band densities of phospho-Erk1/2, p38-MAPK and p65 were first normalized to their corresponding total protein. In the case of IκB, it was normalized to β-actin. The relative band density shown at 15, 30, 60, 180, and 360 min was normalized to the band density at time 0 (which was set to 1). Data are shown as the mean ± SEM. Representative of two independent experiments with two samples per group. Two-tailed unpaired Student’s *t* test. ∗*p* < 0.05, ∗∗*p* < 0.01. BMDM, bone marrow-derived macrophage; IL, interleukin; LPS, lipopolysaccharide; MAPK, mitogen-activated protein kinase; MCP-1, monocyte chemoattractant protein-1; TNF, tumor necrosis factor.

Macrophages express toll-like receptor 4 and respond to bacterial LPS stimulation. We first assessed whether *Ctrp6* gene and protein expression are positively regulated by toll-like receptor 4 signaling, and thus may constitute a feed-forward loop; alternatively, LPS may reduce the expression of *Ctrp6* in a temporal manner to limit excessive inflammatory response in macrophages. We observed that LPS stimulation caused a time-dependent decline in *Ctrp6* transcript and protein expression, followed by a gradual rebound, in BMDMs (Fig. 4, *A* and *B*). Specifically, the expression of *Ctrp6* transcript and protein began to drop at 1 h post LPS stimulation, reached the lowest level by 6 h, followed by a gradual rebound over the next 18 h.

Next, we asked whether CTRP6 can further augment inflammatory cytokine secretion, gene expression, and signaling in macrophages stimulated with LPS. Pretreating BMDMs with CTRP6 for 1 h before stimulation with LPS significantly augmented IL-6 secretion, whereas the effects on TNF-α and monocyte chemoattractant protein-1 (MCP-1) were marginally and not biologically significant (Fig. 4, *C*–*E*). At the level of gene expression, CTRP6 pretreatment further enhanced the expression of inflammatory genes (*e.g.*, *Adgre1*, *Tnf*, *Il1b*, *Itgax*, and *Nos2*) induced by LPS (Fig. 4*F*). At the level of signaling, we observed a significantly greater LPS-induced reduction in IκB and phosphorylation of ERK1/2, p38-MAPK, and p65 (RelA) in CTRP6-pretreated BMDMs than the LPS stimulation alone (Fig. 4, *G* and *H*). These results suggest that CTRP6 can potentiate LPS-induced activation of MAPK and NF-κB signaling pathways in macrophages. Therefore, the observed time-dependent reduction in CTRP6 expression induced by LPS (Fig. 4, *A* and *B*) suggests a potential negative feedback mechanism to prevent prolonged hyperactivation of macrophages during the course of inflammation.

### CTRP6 promotes aerobic glycolysis and ROS production in macrophages

**Figure 5:**
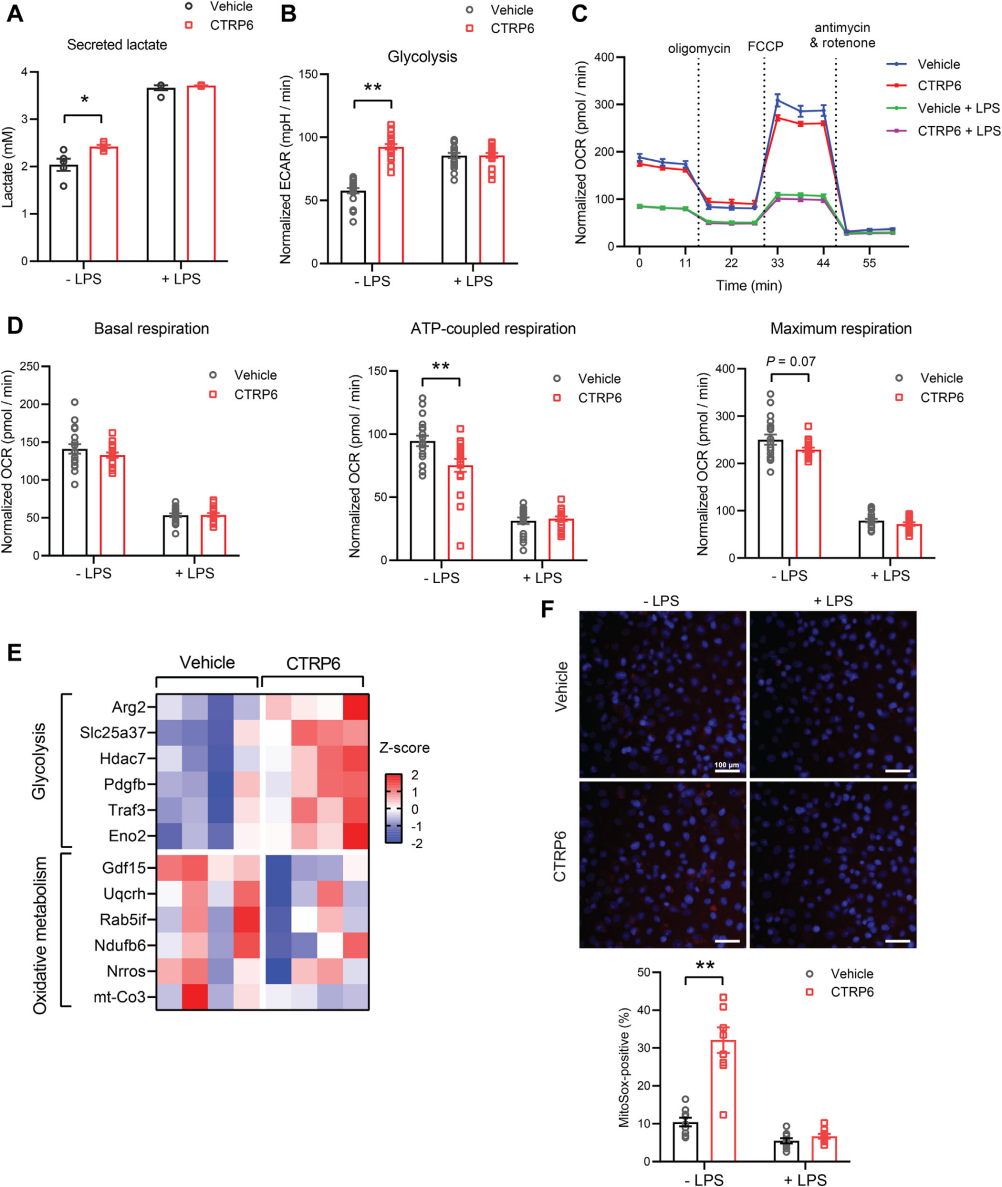
**CTRP6 promotes a metabolic shift toward aerobic glycolysis and ROS production in macrophages.***A*, secreted lactate levels in BMDMs treated with CTRP6, with or without LPS, for 6 h (n = 5). *B*, seahorse analysis of extracellular acidification rate (ECAR) after glucose addition in BMDMs treated with CTRP6, with or without LPS, for 6 h (n = 20). *C*–*D*, seahorse analysis of oxygen consumption rate (OCR) in BMDMs treated with CTRP6, with or without LPS, for 6 h after sequential addition of oligomycin, FCCP, and antimycin A/rotenone. Basal respiration, ATP-coupled respiration, and maximal respirations were shown in (*D*) (n = 18). *E*, a heatmap showing the differentially expressed glycolytic and mitochondrial oxidative metabolism genes (from RNA-seq) in CTRP6-treated BMDMs. Color gradient reflects row Z-score values. *F*, mitochondrial ROS production. MitoSOX staining of BMDMs treated with CTRP6, with or without LPS, for 6 h. Bar graph shows the quantification of MitoSOX-positive cells (n = 9 images). The *white* scale bar represents 100 μm. Data are shown as the mean ± SEM. Representative of three independent experiments. Two-tailed unpaired Student’s *t* test. ∗*p* < 0.05, ∗∗*p* < 0.01. BMDM, bone marrow-derived macrophage; FCCP, carbonyl cyanide-4-(trifluoromethoxy)phenylhydrazone; LPS, lipopolysaccharide; ROS, reactive oxygen species.

A now well-established immunometabolism paradigm is that proinflammatory macrophage activation shifts the cell metabolism toward aerobic glycolysis (10, 42, 43). Because CTRP6 promotes a proinflammatory macrophage phenotype, we therefore assessed whether CTRP6 also induces a shift in energy metabolism toward aerobic glycolysis in BMDMs. To help regenerate NAD^+^ needed for ongoing aerobic glycolysis, pyruvate (the end product of glycolysis) gets reduced to lactate and excreted from cells (44). Treatment of resting BMDMs with CTRP6 resulted in greater lactate secretion (Fig. 5*A*). LPS stimulation appeared to maximally increase lactate secretion from BMDMs, and the cotreatment with CTRP6 did not further elevate lactate secretion (Fig. 5*A*).

The rate of glycolysis can be inferred from the measurement of extracellular acidification rate (ECAR) (45). Consistent with greater lactate secretion, CTRP6 treatment significantly increased ECAR in resting BMDMs, and was not further elevated by costimulation with LPS (Fig. 5*B*). In contrast to increased rate of glycolysis, mitochondrial oxidative metabolism was reduced in CTRP6-treated BMDMs (Fig. 5, *C* and *D*). LPS stimulation maximally reduced mitochondrial oxidative metabolism in BMDMs, and cotreatment with CTRP6 did not further suppress oxidative metabolism (Fig. 5, *C* and *D*). Consistent with the functional data, genes involved in glycolytic flux were upregulated whereas genes involved in mitochondrial oxidative metabolism downregulated in BMDMs treated with CTRP6 (Fig. 5*E*).

In addition to secreting inflammatory cytokines, proinflammatory macrophages also generate reactive oxygen species (ROS) which serves as microbicidal agents in host defense. In addition to the NADPH oxidase system (46), it is known that proinflammatory macrophages also have elevated mitochondrial ROS production (47, 48). We observed that CTRP6 treatment significantly increased mitochondrial ROS production in BMDMs as measured by MitoSOX staining (Fig. 5*F*). Since LPS maximally suppressed mitochondrial oxidative metabolism (Fig. 5*D*), the ability of CTRP6 to promote mitochondrial ROS production was lost in the presence of LPS (Fig. 5*F*). Collectively, these results indicate that CTRP6 induces a metabolic shift from oxidative metabolism toward aerobic glycolysis and increases mitochondrial ROS production in macrophages, both of which are consistent with CTRP6 promoting a proinflammatory macrophage phenotype.

### CTRP6 deficiency attenuates LPS-induced inflammation and hypothermia

**Figure 6:**
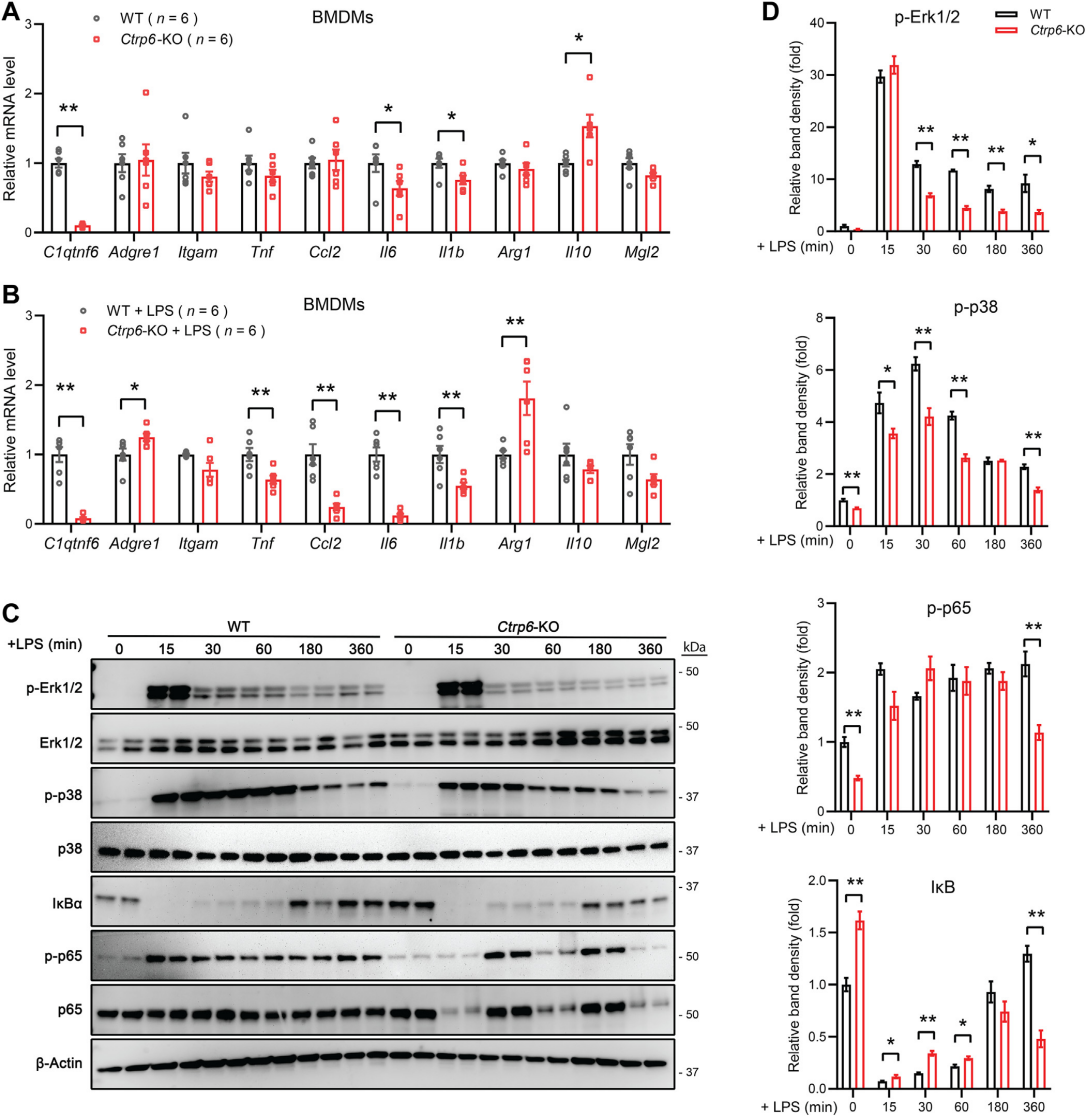
**CTRP6-deficient BMDMs have reduced LPS-induced inflammation gene expression and signaling.***A* and *B*, expression of proinflammatory and antiinflammatory genes in WT and *Ctrp6* KO BMDMs at baseline (*A*), and after LPS treatment (*B*) for 24 h (n = 6). Data are shown as the mean ± SEM. Representative of three independent experiments. Two-tailed unpaired Student’s *t* test. ∗*p* < 0.05, ∗∗*p* < 0.01. *C*, Western blot analysis of MAPK and NF-κB signaling pathways activated before (time 0) and after LPS stimulation in WT and CTRP6-deficient BMDMs. Phospho (P) and total Erk1/2, p38-MAPK, IκB, p65 subunit of NF-κB are indicated. *D*, the band densities of phospho-Erk1/2, p38-MAPK and p65 were first normalized to their corresponding total protein. In the case of IκB, it was normalized to β-actin. The relative band density shown at 15, 30, 60, 180, and 360 min was normalized to the band density at time 0 (which was set to 1). Data are shown as the mean ± SEM. Representative of two independent experiments with two samples per group. Two-tailed unpaired Student’s *t* test. ∗*p* < 0.05, ∗∗*p* < 0.01. BMDM, bone marrow-derived macrophage; LPS, lipopolysaccharide; MAPK, mitogen-activated protein kinase.

Next, we used BMDMs and mice deficient in CTRP6 to further confirm and extend our findings. At resting state, BMDMs derived from *Ctrp6* KO mice had lower expression of proinflammatory genes, *Il6* and *Il1b*, and higher expression of the antiinflammatory gene *Il10* (Fig. 6*A*). In response to LPS stimulation, CTRP6-deficient BMDMs had markedly reduced expression of proinflammatory genes (*e.g.*, *Tnf*, *Ccl2*, *Il6*, and *Il1b*) and increased expression of the antiinflammatory gene *Arg1* relative to WT BMDMs (Fig. 6*B*). Similar to CTRP6-deficient BMDMs, we also observed an attenuated proinflammatory gene expression at baseline and in response to LPS stimulation in alveolar macrophages (AMs) isolated from the lung and Kupffer cells isolated from the liver of *Ctrp6*-KO mice (Fig. S1). In accordance, CTRP6-deficient BMDMs also showed an attenuated LPS-induced phosphorylation of Erk1/2 and p38-MAPK, and to a much lesser extent of p65 (Fig. 6, *C* and *D*). The protein levels of IκB were higher before LPS stimulation, and remained higher for the first 60 min after LPS stimulation in CTRP6-deficient BMDMs.

**Figure 7:**
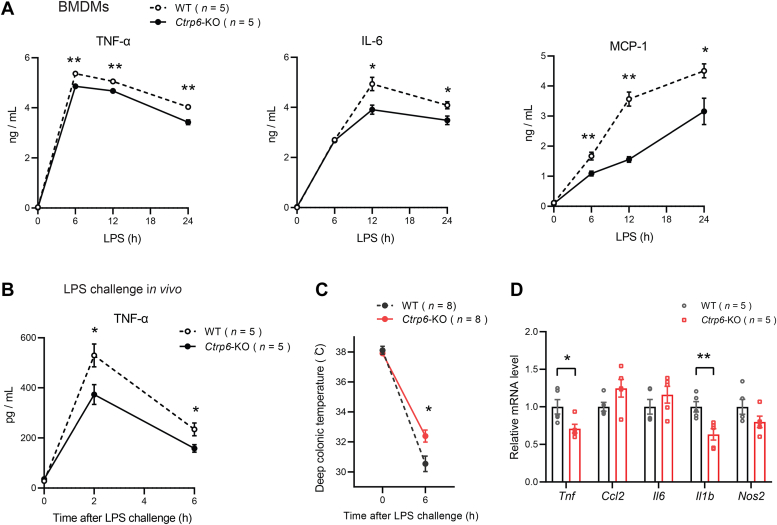
**Mice lacking CTRP6 have attenuated LPS-induced inflammation and hypothermia.***A*, secretion of TNF-α, IL-6, and MCP-1 from WT and *Ctrp6* KO BMDMs treated with LPS for 0 to 24 h. Data are shown as the mean ± SEM. Representative of two independent experiments with five samples per group. Two-tailed unpaired Student’s *t* test. ∗*p* < 0.05, ∗∗*p* < 0.01. *B*, serum TNF-α levels in WT and *Ctrp6* KO mice after intraperitoneal LPS injection at a dose of 5 mg/kg. *C*, changes in body (rectal) temperature before and after LPS injection (n = 8 per group). *D*, expression of proinflammatory genes in the liver of WT and *Ctrp6* KO mice at 6 h after LPS injection (n = 5 per group). Data are shown as the mean ± SEM. Representative of three independent experiments. Two-tailed unpaired Student’s *t* test. ∗*p* < 0.05, ∗∗*p* < 0.01. IL, interleukin; LPS, lipopolysaccharide; MCP-1, monocyte chemoattractant protein-1; TNF, tumor necrosis factor.

Secretion of inflammatory cytokines (TNF-α, IL-6, and MCP-1) was also significantly reduced in CTRP6-deficient BMDMs compared to WT BMDMs upon LPS stimulation (Fig. 7*A*). Thus, deletion of *Ctrp6* gene reduces the macrophage inflammatory response whereas recombinant CTRP6 treatment promotes the opposite phenotype. Lastly, we sought to determine whether CTRP6 deficiency protects mice from LPS-induced inflammation and hypothermia. Mice lacking CTRP6 had significantly lower serum TNF-α levels relative to WT controls after an LPS challenge (Fig. 7*B*). Serum IL-1β, IL-6, and MCP-1 levels, however, were not significantly different between genotypes (data not shown). In addition, loss of CTRP6 also significantly attenuated hypothermia induced by LPS challenge (Fig. 7*C*). Consistent with the physiological data, transcript expression of *Tnf* and *Il1b* was also significantly lower in the liver of *Ctrp6* KO mice relative to WT controls after an LPS challenge (Fig. 7*D*). Together, these results indicate that CTRP6 deficiency dampens LPS-induced systemic inflammation in mice.

## Discussion

Using a combination of unbiased and targeted approaches, we provided new insights concerning the direct action of CTRP6 on macrophages. We showed that CTRP6 engages the MAPK and NF-κB signaling pathways in macrophages to drive the expression of proinflammatory gene expression and cytokine production and secretion. In resting BMDMs, CTRP6 treatment alone was sufficient to promote the expression of proinflammatory genes, and induce a metabolic shift from oxidative metabolism toward aerobic glycolysis, and enhanced mitochondrial ROS production. Pretreatment with CTRP6 also sensitized and potentiated BMDMs response to LPS. In contrast, BMDMs lacking CTRP6 had significantly reduced LPS-induced proinflammatory gene expression and cytokine secretion. Crucially, mice lacking CTRP6 had attenuated systemic inflammation in response to endotoxemia, as indicated by reduced serum TNF-α and attenuated LPS-induced hypothermia. Collectively, these results along with our previous studies (25, 26) extend and affirm the autocrine and paracrine role of CTRP6 in regulating macrophage function. Our findings also suggest a potential role for CTRP6 in modulating the inflammatory tone in local tissue environment under various physiological and pathophysiological states (*e.g.*, short-term caloric surplus, infection, and chronic obesity).

In a short-term caloric overload model (3-days high-fat feeding), the *Ctrp6* transcript is rapidly upregulated in the adipose tissue (26). This occurred prior to any significant infiltration of macrophages into the fat compartment. In this physiological context, the inflammatory response mediated by the locally produced CTRP6 presumably acts on adipocytes and stromal vascular cells (including resident adipose tissue macrophages) to limit excess fat accumulation, thus serving a homeostatic role (26). Such homeostatic, rather than pathological, inflammatory response in the early phase of caloric surplus is well-documented (49, 50). However, in the setting of obesity induced by chronic high-fat feeding, close to half of the cells in the adipose compartment in mice are comprised of macrophages (7, 15, 17). In this context, the direct action of CTRP6 on adipose tissue macrophages represents an important axis contributing to low-grade chronic inflammation in the fat pads. In addition, obesity is known to induce gut dysbiosis and leakiness, leading to increased circulating LPS (51, 52). Based on the data presented in this study, it is likely that in obesity CTRP6 acts in concert with LPS derived from the gut bacteria to further exacerbate adipose tissue dysfunction, underscoring its potential modulatory role in priming adipose tissue macrophages to inflammatory stimuli. Consistent with this, mice lacking CTRP6 have reduced adipose tissue inflammation and improved systemic insulin sensitivity (25).

We showed that pretreatment of BMDMs with CTRP6 enhances LPS-stimulated proinflammatory signaling and cytokine secretion. Conversely, CTRP6-deficient BMDMs had a significantly dampened LPS-induced proinflammatory response. Interestingly, we also noted that acute LPS treatment caused a time-dependent suppression of CTRP6 expression in BMDMs, followed by a gradual rebound at 24 h. Since CTRP6 alone or in combination with LPS promotes the macrophage proinflammatory response, the temporal suppression of CTRP6 expression by LPS suggests a potential negative feedback mechanism to prevent prolonged macrophage hyperactivation, as an uncontrolled inflammatory response is detrimental. In contrast to this potential negative feedback mechanism by LPS, we previously showed that CTRP6 treatment of macrophages can upregulate *Ctrp6* expression, resulting in positive reinforcement (25). However, what signal(s) terminates this positive feedback once CTRP6 action is initiated remains to be identified. This signal(s)—in the form of a secreted factor or metabolite—likely exists, as we have previously noted that *Ctrp6* expression in obesity can be downregulated back to normal levels if mice are subjected to a diet reversal (*i.e.*, switching from a high-fat to a low-fat diet after obesity induction) (26). Collectively, these observations indicate that *Ctrp6* expression in macrophages is subjected to both positive and negative regulations, and dysregulated CTRP6 expression may contribute to pathology in diseased settings.

The CTRP6-induced metabolic shift from oxidative metabolism toward aerobic glycolysis in BMDMs is consistent with the metabolic phenotype of proinflammatory macrophages reported in the literatures (9, 10, 11, 42, 43, 53, 54, 55, 56, 57, 58). Proinflammatory macrophages are known to upregulate their primary glucose transporter (GLUT1) leading to increased glucose uptake (10). Despite the presence of oxygen, upon proinflammatory activation, macrophages shift to rely on glycolysis rather than on mitochondrial oxidative metabolism to fuel their energetic needs (44, 59, 60). This “Warburg-like” metabolism with elevated lactate secretion (61) has been extensively documented in M1-polarized macrophages (42, 60). While glucose flux through glycolysis and the pentose phosphate pathway are greatly elevated in proinflammatory macrophages, the mitochondrial oxidative metabolism is correspondingly downregulated (9, 10, 11, 42, 43, 53, 54, 55, 56, 57, 58). The mechanisms underpinning the “Warburg-like” metabolism in proinflammatory macrophages are being actively investigated (44, 59, 60). Recent studies have suggested that the tricarboxylic acid cycle within mitochondria that supplies the reducing equivalents (NADH2 and FADH2) to the electron transport chain is broken at two places, leading to elevated citrate and succinate (42, 44). Succinate stabilizes hypoxia-inducible factor 1-alpha, leading to the upregulated expression of glycolytic enzymes and glucose transporters (11). Increased succinate oxidation by succinate dehydrogenase (part of complex II) without the corresponding increase in ATP synthesis results in reverse electron transport within the electron transport chain that enhances mitochondrial ROS production (48). These mechanisms are also likely responsible for the observed increase in glycolytic rate, lactate secretion, and the corresponding reduction in oxidative metabolism in BMDMs treated with CTRP6.

CTRP6 belongs to the C1q family with over 30 members, united by a shared globular C1q domain (62, 63). Interestingly, several members of this family have also been demonstrated to play a role in regulating macrophage function and polarization. For example, complement C1q (64, 65, 66), adiponectin (67, 68, 69), CTRP4 (70), and CTRP9 (71) have been shown to polarize macrophages toward the antiinflammatory M2 phenotype. In contrast, CTRP1 appears to polarize macrophages toward the proinflammatory phenotype (72). Depending on the context, CTRP3 has been shown to polarize macrophages toward either the proinflammatory or antiinflammatory phenotype (73, 74). Thus, in a broader context, our current findings along with previous studies by others suggest the C1q family to be an important class of secreted regulators of macrophage function across different physiological and disease states.

Two limitations of the study are noted. First, the dose of CTRP6 we used in our *in vitro* studies was empirically determined. We do not know the concentration of CTRP6 in the adipose tissue microenvironment, and the lack of a validated ELISA specific for mouse CTRP6 precluded such measurement. Given CTRP6 is an autocrine and paracrine factor markedly upregulated in the fat compartment in obesity, the local concentration of CTRP6 that adipose tissue macrophages potentially exposed to could be high and thus the dose we used is likely within the physiological range encountered *in vivo*. Importantly, our loss-of-function studies in BMDMs and in mice corroborated our gain-of-function studies using recombinant CTRP6. Second, we do not know the receptor on macrophages that mediates the biological action of CTRP6. While it has been shown that CTRP6 can bind to soluble complement C3, collectin-11, and certain sugar such as I-fucose (75, 76), no plasma membrane receptor on macrophages has been identified that binds CTRP6. Since CTRP6 alone can rapidly and directly activate BMDMs, we presumed that a CTRP6-specific receptor is expressed by macrophages, the identity of which remains to be discovered.

In summary, our data highlighted the direct mechanistic action of CTRP6 on promoting macrophage proinflammatory response. Since mice lacking CTRP6 have reduced LPS-induced systemic inflammation, as well as decreased adipose tissue inflammation in obesity (25), approaches to neutralize the activity of CTRP6 in pathological settings may prove beneficial.

## Experimental procedures

### Animals

C57BL/6J WT mice were purchased from The Jackson Laboratory. The *Ctrp6* whole-body KO mice (129S5-C1qtnf6^tm1Lex^/Mmucd; stock number 031616-UCD) were obtained from Mutant Mouse Regional Resource Center, and backcrossed for >10 generations onto a C57BL/6J genetic background (25). Heterozygous mice were intercrossed to obtain WT (+/+) and *Ctrp6* KO (−/−) mice. All mice were housed in polycarbonate cages under a 12-h/12-h light/dark photocycle and fed a standard chow (9% fat; Lab Diet) with free access to water. All mouse protocols were approved by the Institutional Animal Care and Use Committee of the Johns Hopkins University School of Medicine. All animal experiments were conducted in accordance with the National Institute of Health guidelines and followed the standards established by the Animal Welfare Acts.

### Recombinant mouse CTRP6 production

Recombinant full-length mouse CTRP6 containing a C-terminal FLAG epitope tag (DYKDDDDK) was produced in suspension FreeStyle 293-F cells (Thermo Fisher Scientific) at the Mammalian Cell Expression Core of The Johns Hopkins University School of Medicine. For 1 l expression, 1 mg of purified CTRP6 expression plasmid (in pCDNA3.1 vector) were transfected with PEI (Polysciences, Inc) at a ratio of 1:3 into FreeStyle 293-F cells at a concentration of 2 × 10^6^ to 2.4 × 10^6^ cells per ml and incubated at 37 °C incubator for 3 days. Cells were cultured in FreeStyle 293 expression medium (Thermo Fisher Scientific). The 1 l medium was harvested *via* centrifugation, sterile filtered, and subjected to affinity chromatography using an anti-FLAG M2 affinity gel (Sigma-Aldrich, A2220) according to the manufacturer’s protocol. Purified protein was dialyzed against 20 mM Hepes (pH 8.0) containing 135 mM NaCl using a 10-kDa molecular weight cutoff dialysis cassette (Thermo Fisher Scientific, 66810) and concentrated with a 10-kDa cutoff Amicon Ultra-15 centrifugal filter unit (Millipore, UFC901024). Protein concentration was determined using the bicinchoninic acid (BCA) assay kit (Thermo Fisher Scientific, 23227) and samples were aliquoted and stored at −80 °C.

### Macrophage cell culture

Primary mouse BMDMs were cultured as previously described (25). Briefly, BMDMs were isolated from the femurs and tibias of C57BL/6J WT mice or *Ctrp6* KO mice between 8 and 12 weeks of age and cultured on Petri dishes in RPMI 1640 medium supplemented with 10% fetal bovine serum (FBS), 1% penicillin/streptomycin, 1% Hepes, 1% sodium pyruvate plus 10 ng/ml recombinant macrophage colony-stimulating factor (M-CSF; R&D Systems, 416-ML-010). Differentiated cells were refed on day 3. On day 7, differentiated BMDMs were lifted by trypsinization, collected, counted, and replated in complete RPMI 1640 medium in culture plates. After an overnight incubation, BMDMs were treated accordingly. The purity of BMDMs was assessed by flow cytometry. Approximately 80% of the cells were positive for F4/80 and Cd11b (Fig. S2). For CTRP6 treatment, cells were treated with vehicle control (Hepes) or purified recombinant CTRP6 in RPMI 1640 with 0.2% bovine serum album for varying periods of time. For pretreatment experiments, BMDMs were pretreated with 5 μg/ml CTRP6 for 1 h, followed by stimulation with 100 ng/ml LPS (Sigma-Aldrich *Escherichia coli* 055: B5, L2880) in the presence of CTRP6 for varying periods of time. For cotreatment experiments, BMDMs were treated with 5 μg/ml CTRP6 in the presence of 100 ng/ml LPS for 6 h. In experiments involving pharmacologic inhibitors, BMDMs were pretreated for 1 h with dimethyl sulfoxide (vehicle control) or p38-MAPK inhibitor SB203580 (10 μM, Selleckchem, S1076), MEK/Erk1/2 inhibitor PB98059 (20 μM, Tocris Bioscience, 1213), or NF-κB inhibitor Bay 11-7082 (Bay11, 5 μM, Selleckchem, S2913), and subsequently treated with 5 μg/ml CTRP6 or vehicle control (Hepes) for an additional 24 h in the presence of each inhibitor. For WT and *Ctrp6-*KO BMDMs, cells were stimulated with 100 ng/ml LPS for varying periods of time as indicated. After treatment, cells were washed and harvested for immunoblotting and quantitative real-time PCR (qRT-PCR). Supernatants were collected to measure cytokine production by ELISA.

Primary AMs were isolated from the bronchoalveolar lavage (BAL) fluid of C57BL/6J WT mice or *Ctrp6*-KO mice as previously described (77). Briefly, the thoracic cavity of anesthetized mice was opened to expose the airway, then BAL fluid was collected by cannulating the trachea and lavaging the lung using prechilled PBS with 1 mM EDTA. Multiple flushes of BAL fluid were pooled and centrifuged at 250*g* at 4 °C for 10 min. The pelleted AMs were resuspended and cultured in Dulbecco's modified Eagle's medium supplemented with 10% FBS, 1% penicillin/streptomycin, 10 mM Hepes plus 25 ng/ml M-CSF. After 24 h, the adherent AMs were stimulated with 200 ng/ml LPS or 20 ng/ml IL-4 for 24 h for the M1 macrophage polarization and M2 macrophage polarization, respectively.

Kupffer cells were isolated from the liver of *Ctrp6*-KO mice by a modified *in situ* perfusion procedure as previously described (78). Briefly, the inferior vena cava was cannulated and the liver was perfused with liver perfusion media (Thermo Fisher Scientific, 17701) followed by 0.1% collagenase IV digest (Thermo Fisher Scientific, 17104019). The disassociated liver was then minced by spatula, filtered through a 100 μM strainer, and centrifuged at 100*g* for 3 min. The supernatant enriched in Kupffer cells was loaded onto a Percoll gradient (GE HealthCare, 17-0891-01) of 25% and 50% for density gradient centrifugation at 2300*g* for 30 min at 4 °C without a break. The interphase ring was collected, resuspended, and cultured in RPMI 1640 medium supplemented with 10% FBS, 1% penicillin/streptomycin. After 2 h, nonadherent cells were removed by changing the culture medium. The adherent fraction was washed and stimulated with 100 ng/ml LPS for 24 h.

### Flow cytometry

Day-6 differentiated BMDMs were collected and washed, then incubated in fluorescence activated cell sorting (FACS) buffer (0.5% BSA and 2 mM EDTA in PBS) with Fc-block (CD16/32) (BioLegend, 101319) on ice for 10 min to reduce nonspecific binding. Cells were stained with F4/80 and CD11b on ice for 30 min in the dark. After multiple washes to remove excess antibodies, cells were assayed on an Attune N × T Acoustic Focusing Cytometer (Invitrogen) and data were analyzed using FlowJo software v.10.8.0 (Tree Star Inc). The antibody information is provided in Table S6.

### Quantitative real-time PCR (qRT-PCR)

Total RNA was extracted from liver tissues or cells using TRIzol reagent (Invitrogen, 15596018) according to the manufacturer’s protocol. The first-strand complementary DNAs were reverse transcribed using iScript cDNA Synthesis Kit (Bio-Rad, 1708891). qRT-PCR was performed using the Universal SYBR Green Supermix (Bio-Rad, 1725124) on a CFX Connect system (Bio-Rad). Results were normalized to *36B4* and expressed as fold changes using the 2^−△△CT^ method (79). The sequences of all primers are listed in Table S5.

### Immunoblotting

Proteins from tissues or cells were extracted in RIPA buffer (Sigma-Aldrich, R0278) freshly supplemented with protease inhibitor cocktails (Roche, 11836170001) and phosphatase inhibitor cocktails (Roche, PhosSTOP, 04906837001). Protein concentrations were quantified using the Pierce BCA Protein Assay Kit (Thermo Fisher Scientific, 23227). Equal amounts of total protein lysates were analyzed by standard Western blot procedures using either the Mini-Protean tris-glycine extended gels or the Criterion tris-glycine extended Midi Protein gels (Bio-Rad). Protein bands were developed using the ECL substrate (Millipore, WBLUC0500) and visualized by FluorChem Q Imager (Alpha Innotech) followed by densitometry quantification using the ImageJ software (80). Antibody information is provided in Table S6.

### Cytokine measurements

Mouse IL-6 and MCP-1 in cell culture supernatants were measured by commercially available ELISA kit (R&D Systems, M6000B and MJE00B) according to the manufacturer’s instructions. For quantifying TNF-α level in cell culture supernatants and mouse serum, mouse TNF-α ELSIA kit (R&D Systems MTA00B) was used according to the manufacturer’s instructions.

### Lactate assay

The levels of lactate in cell culture supernatants were measured with the Lactate Assay Kit (Abcam, ab65330) according to the manufacturer's instructions.

### Mitochondrial ROS measurement

For measuring mitochondrial ROS generation, BMDMs were treated with 5 μg/ml CTRP6 with or without 100 ng/ml LPS for 6 h, followed by incubation with 5 μM MitoSOX (Thermo Fisher Scientific, M36008) in Hanks' balanced salt solution plus calcium and magnesium for 15 min at 37 °C, protected from light. After staining, cells were washed and counterstained with Hoechst 33258 (Sigma-Aldrich, 94403) and imaged using a fluorescence microscope. The positive cells were counted and normalized to total cell number.

### Seahorse respirometry assay

Oxygen consumption rate and ECAR were measured using an XF96 Extracellular Flux Analyzer (Seahorse Bioscience). Briefly, BMDMs were seeded at 8 × 10^4^ cells per well in Seahorse XF96 tissue culture plates (Agilent Technologies) and cultured overnight before treated with 5 μg/ml CTRP6 with or without 100 ng/ml LPS for 6 h. The ECAR in BMDMs were measured under basal conditions and following the addition of 20 mM glucose. Changes in oxygen consumption rate were detected under basal conditions and following the sequential addition of 2.5 μM oligomycin, 2 μM carbonyl cyanide-4-(trifluoromethoxy)phenylhydrazone and 0.5 μM antimycin A/rotenone (all the chemical compounds were from Agilent Technologies). Results were collected with Wave software version 2.6 (Agilent Technologies). Data were normalized to the absorbance at 450 nm after incubation with water-soluble tetrazolium salts cell proliferation reagent (Roche, 05015944001) as a measure of cell number in each well.

### LPS-induced inflammation and hypothermia

For endotoxemia model, 8 to 12-week-old male *Ctrp6* KO mice and age-matched WT littermates were administered with LPS (*E. coli* 055: B5, L2880, Sigma-Aldrich) at 5 mg/kg by intraperitoneal injection. The mice had normal access to food and water and the deep colonic temperature was monitored with a digital thermocouple thermometer (BAT-12, Physitemp Instruments) before and 6 h after LPS injection. Blood was collected before and at 2 and 6 h after LPS injection, placed at room temperature (RT) for 2 h and then centrifuged for 5 min at 10,000*g*. Serum samples were collected and stored at −80 °C. Mice were euthanized at 6 h after LPS injection and tissues were snap-frozen and stored at −80 °C.

### RNA sequencing and analysis

Bulk RNA sequencing was performed on total RNA isolated from BDMDs treated with 5 μg/ml CTRP6 or vehicle control (Hepes) for 6 h. There were four biological replicates for each group. Library was prepared and sequenced at Novogene on an Illumina NovaSeq 6000 platform with total raw reads of ∼20 million per sample. The RNA-seq base call files were converted to fastq files by using the bcl2fastq (version 2.15.0.4) program. RNA-seq reads were then aligned to the Ensembl release with STAR version 2.0.4b. Gene counts were derived from the number of uniquely aligned unambiguous reads by Subread feature count version 1.4.5. Quantification, normalization, and DEGs were determined with the Cufflinks package (version 2.2.1). DEGs were used as an input for pathway analysis through Ingenuity Pathway Analysis suite (www.ingenuity.com). The R/Bioconductor package heatmap3 was used to display heatmaps or annotated KEGG graphs across groups of samples for each KEGG pathway with a Benjamini–Hochberg false-discovery rate adjusted *p*-value less than or equal to 0.05. DEGs were defined as ones with at least 0.5 fragments per kilobase of transcript per million mapped reads level of expression in at least 1 of the conditions and a Q-value <0.05. The RNA-seq data was deposited in the NCBI database, with the Sequence Read Archive (SRA) accession # PRJNA947594.

### Reagents for proteomics analysis

Titanspheres (TiO2, 5 μm beads) were from GL Sciences Inc. L-1-tosylamide-2-phenylethyl chloromethyl ketone (TPCK) treated trypsin was from Worthington Biochemical Corp. All other reagents used in this study were from Thermo Fisher Scientific.

### Cell lysis, protein digestion, and TMT labeling

BMDM were treated with 5 μg/ml CTRP6 or vehicle control (Hepes) for 30 min. The treatments were carried out in triplicates. Cells were washed with ice-cold PBS, collected and lysed in lysis buffer (8 M urea, 20 mM Hepes, pH 8.0, 2.5 mM sodium pyrophosphate, 1 mM disodium β-glycerophosphate, 1 mM sodium orthovanadate, and 10 mM sodium fluoride) by sonication. After centrifugation at 17,000*g* at 15 °C for 20 min, the protein lysates were collected and the protein concentration was determined using BCA assay (Pierce). An equal amount of protein from each sample was reduced by DTT at a final concentration of 5 mM at 60 ˚C for 20 min and alkylated using 10 mM iodoacetamide for 20 min at RT in the dark. For tryptic digestion, protein extracts were diluted in 20 mM Hepes pH 8.0 to a final concentration of 2 M urea and incubated with TPCK-treated trypsin at 25 °C overnight. Protein digests were acidified by 1% TFA and subjected to centrifugation at 2000*g* at RT for 5 min. The supernatant of protein digests was loaded onto a Sep-Pak C18 column (Waters, WAT051910) equilibrated with 0.1% TFA. Columns were washed with 12 ml of 0.1% TFA and peptides were eluted in 6 ml of 40% acetonitrile (ACN) with 0.1% TFA. Eluted peptides were lyophilized and subjected to tandem mass tag (TMT) labeling. TMT labeling was carried out according to the manufacturer’s instructions. Briefly, 250 μg of tryptic peptides from each sample was reconstituted in 100 μl of 50 mM triethylammonium bicarbonate buffer (TEABC) buffer and mixed with a 6-plex TMT reagent reconstituted in 41 μl of anhydrous ACN and incubated at RT for 1 h. All the labeled peptides from each sample were equally mixed, dried completely in a vacuum concentrator, and kept at −80 °C.

### Fractionation of peptides by basic reversed-phase liquid chromatography

TMT-labeled peptide mixtures were resuspended in 1 ml of 10 mM TEABC, pH 8.0 and loaded on a XBridge BEH C18 Column, 130 Å, 5 μm, 4.6 mm × 250 mm (Waters, Cat # 186003117), and fractionated on an Agilent 1100 Series HPLC system by basic reversed-phase chromatography at a flow rate of 400 μl/min. Mobile phase consisted of 10 mM TEABC, pH 8.0 (buffer A) and 10 mM TEABC, 90% ACN, pH 8.0 (buffer B). After loading 1 ml of sample (6.4 mg) onto the column, the peptides were separated using the following gradient: 5 min isocratic hold at 2% B, 5 to 7% solvent B in 2 min; 5 to 40% solvent B in 63 min; 40 to 100% solvent B in 2 min; hold at 100% solvent B for 3 min, 100 to 2% solvent B in 1 min, hold at 2% solvent B for 4 min for a total gradient time of 80 min. Using 96 × 1 ml well plates (Thermo Fisher Scientific, #7701-5200), fractions were collected for a total of 96 fractions through the elution profile of the separation. A total of 5% of collection from each well were merged into 12 fractions and dried by vacuum centrifugation for the liquid chromatography tandem mass spectrometry (LC-MS/MS) analysis of the proteomic changes in cells. The rest of the collection from each well were merged into 12 fractions and dried by vacuum centrifugation for TiO_2_-based phosphopeptide enrichment.

### Phosphopeptide enrichment strategy

The 12 fractions of TMT-labeled peptides were subjected to TiO2-based phosphopeptide enrichment as described by Larsen *et al.* (81) with minor modification. Briefly, TiO2 beads were pretreated by incubation with 2,5-dihydroxybenzoic acid (DHB) solution (80% v/v ACN, 3% v/v TFA, and 5% w/v DHB) for 20 min at RT. Each fraction was resuspended in DHB solution and incubated with pretreated TiO_2_ beads (Peptides:TiO2 = 1:1). Phosphopeptide-bound TiO2 beads were washed twice with 400 μl of washing solution (80% v/v ACN, 3% v/v TFA). Peptides were eluted three times with 20 μl of 4% v/v ammonia into 20 μl of 20% v/v TFA and dried completely by vacuum centrifugation. The dried peptides were resuspended in 50 μl 0.15% TFA, and desalted using C18 Stage Tips (82).

### Liquid chromatography tandem mass spectrometry

LC-MS/MS analysis of peptides and phosphopeptides was carried out using a reversed phase liquid chromatography system interfaced with an Orbitrap Fusion Lumos mass spectrometer. The mass spectrometer was operated in the “high-high” mode, where mass spectra of both precursor and product ions were acquired in the high resolution Orbitrap analyzer (Thermo Fisher Scientific). The peptides were loaded onto a trap column (100 μm I.D. × 2 cm nanoViper column packed with Acclaim PepMap RSLC C18, 5 μm 100 Å; Thermo Fisher Scientific, 164564) by 0.1% v/v formic acid and eluted to the mass spectrometer by an analytical column (EASY-spray column, 75 μm I.D. × 50 cm nanoViper column packed with Acclaim PepMap RSLC C18, 2 μm 100 Å; Thermo Fisher Scientific, ES803) using an ACN gradient (0–90% v/v) containing 0.1% v/v formic acid. The mass spectrometer settings were the following: (a) fourier transform-based mass spectrometry (FTMS) precursor scans from 400 to 1600 m/z (Maximum Injection Time (ms) = 50, AGC Target = 200000) at 120,000 resolution; and (b) MS2 scan (FTMS) of higher-energy collisional dissociation fragmentation of the most intense ions (isolation mode: quadrupole; isolation window: 1.60 m/z; Isolation m/z offset: 0.5; collision energy (%): 32; activation Q = 0.25; FT first mass value: 110.00 (fixed); data type: centroid; AGC Target = 50,000) at 30,000 resolution; and c) MS3 scan (FTMS) of higher-energy collisional dissociation fragmentation of the most intense ions (isolation mode: IonTrap; MS2 isolation window: 2 m/z; stepped collision energy (%): 5; collision energy (%): 65; activation Q = 0.25; FT first mass value: 100.00 (fixed); data type: centroid; AGC Target = 100000) at 60,000 resolution.

### Mass spectrometry data analysis

The tandem mass spectra were searched using the Andromeda search algorithm SEQUEST HT (83) against a mouse UniProt database (released on May 2018) embedded in the Proteome Discoverer platform (Thermo Fisher Scientific, version 2.2). The search parameters were set as follows: a maximum of two missed cleavages, carbamidomethylation at cysteine and TMT at lysine and peptide N terminus as a fixed modification and oxidation at methionine, acetylation at protein N terminus, and phosphorylation at serine, threonine, and tyrosine as variable modifications. The mass tolerances for MS and MS/MS were set to 10 ppm and 0.02 Da, respectively. The reverse type of the target–decoy analysis was chosen. False discovery rates for both peptide and protein level filterings were set to 0.01. PhosphoRS (84) was used to calculate phosphorylation site probability. The minimum peptide length was set to six amino acids. The minimum number of peptides for protein identification was set to 1. The TMT reporter ion intensities were used to calculate the abundance changes of proteins and phosphopeptides in CTRP6- *versus* control vehicle-treated BMDMs.

### Statistical analysis

All results are expressed as mean ± standard error of the mean (SEM). Statistical analysis was performed with Prism 9 software (GraphPad). Data were analyzed with two-tailed Student’s *t*-tests or by One-way ANOVA. Values were considered to be significant at *p* <0.05.

## Data availability

All data described in this manuscript is contained in the main text or Supplementary materials. RNA-seq data has been deposited in NCBI database, with the Sequence Read Archive (SRA) accession # PRJNA947594. The mass spectrometry proteomics data have been deposited to the ProteomeXchange Consortium *via* the PRIDE partner repository (http://www.ebi.ac.uk/pride) with the dataset identifier PXD042268.

## Supporting information

This article contains supporting information.

## Conflict of interest

The authors declare that they have no conflicts of interest with the contents of this article.
