# Supplementary material for: CTRP6 promotes the macrophage inflammatory response, and its deficiency attenuates LPS-induced inflammation
Source: J Biol Chem. 2023 Dec 14;300(1):105566. doi: 10.1016/j.jbc.2023.105566 (PMC10789631; doi:10.1016/j.jbc.2023.105566)
Supplement: Supporting Tables S1–S6 [file mmc1.pdf]

Supplemental Table S1

Gene Symbol	Gene Name	log2(FC)	*P* -value
Gm11465	predicted gene 11465	4.5425	0.00249
AI464131	expressed sequence AI464131	4.4644	0.00816
1700095J12Rik	RIKEN cDNA 1700095J12 gene	4.4240	0.00225
Gm43823	predicted gene 43823	4.4239	0.00846
Rtkn	rhotekin	4.3022	0.00374
Radil	Ras association and DIL domains	4.1939	0.00823
Ube4bos2	ubiquitination factor E4B, opposite strand 2	4.1862	0.00501
Gm37900	predicted gene, 37900	4.1386	0.00962
Cfap161	cilia and flagella associated protein 161	4.0604	0.00884
Wfikkn2	WAP, follistatin/kazal, immunoglobulin, kunitz and netrin domain containing 2	3.9368	0.03606
Gm44639	predicted gene 44639	3.9017	0.03087
Nek2	NIMA (never in mitosis gene a)-related expressed kinase 2	3.7792	0.04935
Gm7972	predicted gene 7972	3.7723	0.02291
Shank1	SH3 and multiple ankyrin repeat domains 1	3.6407	0.01530
Lime1	Lck interacting transmembrane adaptor 1	3.5956	0.03282
AC113006.1	novel transcript	3.5853	0.01059
Crlf1	cytokine receptor-like factor 1	3.5281	0.03803
Fam131b	family with sequence similarity 131, member B	3.5179	0.01328
Gm11592	predicted gene 11592	3.4972	0.00547
Capn12	calpain 12	3.4912	0.02444
Gm17794	predicted gene, 17794	3.4477	0.04704
Catsperz	cation channel sperm associated auxiliary subunit zeta	3.3561	0.04829
Gm15560	predicted gene 15560	3.3323	0.04601
Ampd1	adenosine monophosphate deaminase 1	3.2906	0.04541
Zfp93	zinc finger protein 93	3.2321	0.03227
A430027C01Rik	RIKEN cDNA A430027C01 gene	3.2193	0.03618
Gm13163	predicted gene 13163	3.1724	0.03733
Gm42686	predicted gene 42686	3.1601	0.04596
Gm48102	predicted gene, 48102	3.1590	0.03769
Gm48294	predicted gene, 48294	3.1506	0.02079
Gm8188	predicted gene 8188	3.0979	0.04650
Gm13038	predicted gene 13038	3.0831	0.04728
Dkk2	dickkopf WNT signaling pathway inhibitor 2	3.0058	0.04250
Gm14168	predicted gene 14168	2.9706	0.03349
BC030343	cDNA sequence BC030343	2.9124	0.03876
Gm4240	predicted gene 4240	2.8411	0.03917
Foxl2os	forkhead box L2, opposite strand	2.8286	0.04497
Gnb5	guanine nucleotide binding protein (G protein), beta 5	2.8071	0.00815
1700012D14Rik	RIKEN cDNA 1700012D14 gene	2.7956	0.01592
Shpk	sedoheptulokinase	2.7892	0.01539
Neil2	nei like 2 (E. coli)	2.7863	0.00072
Gm38293	predicted gene, 38293	2.7263	0.01808
1700123M08Rik	RIKEN cDNA 1700123M08 gene	2.5824	0.00125
Snord73a	small nucleolar RNA, C/D box U73A	2.5202	0.02694
Adgrg1	adhesion G protein-coupled receptor G1	2.4493	0.02808
Gm38171	predicted gene, 38171	2.4111	0.03099
Gm22151	predicted gene, 22151	2.4032	0.02560
Carmil2	capping protein regulator and myosin 1 linker 2	2.3193	0.00088
Gapdh	glyceraldehyde-3-phosphate dehydrogenase	2.2999	0.00228
Gm15793	predicted gene 15793	2.2936	0.03460
Gm6710	predicted gene 6710	2.2469	0.00316
Gm20900	predicted gene, 20900	2.2229	0.02777
Zfp763	zinc finger protein 763	2.1962	0.00965
Fkbp14	FK506 binding protein 14	2.1652	0.03590
Lpar5	lysophosphatidic acid receptor 5	2.1552	0.00935
7330423F06Rik	RIKEN cDNA 7330423F06 gene	2.1351	0.01508
4932438H23Rik	RIKEN cDNA 4932438H23 gene	1.9652	0.02563
Ftx	Ftx transcript, Xist regulator (non-protein coding)	1.9649	0.00188
Gm48137	predicted gene, 48137	1.9433	0.01620
Ctxn1	cortexin 1	1.9367	0.01885
Abcg4	ATP binding cassette subfamily G member 4	1.9198	0.02678
Gm26964	predicted gene, 26964	1.9004	0.01257
Ndrg4	N-myc downstream regulated gene 4	1.8614	0.04379
Bcl7a	B cell CLL/lymphoma 7A	1.8446	0.01938
Gm26516	predicted gene, 26516	1.8371	0.00325
Slc29a2	solute carrier family 29 (nucleoside transporters), member 2	1.8177	0.01939
Gm609	predicted gene 609	1.8173	0.04421
Pomgnt2	protein O-linked mannose beta 1,4-N-acetylglucosaminyltransferase 2	1.8072	0.04997
Gm15190	predicted gene 15190	1.7857	0.01548

Gm17249	predicted gene, 17249	1.7806	0.03911
9330020H09Rik	RIKEN cDNA 9330020H09 gene	1.7733	0.04193
Gm42986	predicted gene 42986	1.7538	0.02463
Dnmt3l-ps1	DNA methyltransferase 3-like, pseudogene 1	1.7524	0.00863
Zfp109	zinc finger protein 109	1.7465	0.02943
Gm48621	predicted gene, 48621	1.7327	0.03626
Proser3	proline and serine rich 3	1.6549	0.03723
Mrip-ps	Mom radiation induced polyposis, pseudogene	1.6446	0.02330
Tsen2	tRNA splicing endonuclease subunit 2	1.5818	0.00448
Gm43062	predicted gene 43062	1.5771	0.03006
Art2a-ps	ADP-ribosyltransferase 2a, pseudogene	1.5665	0.04905
Gm43858	predicted gene 43858	1.5664	0.00171
Gm16096	predicted gene 16096	1.5327	0.00025
Gm2511	predicted gene 2511	1.5157	0.04309
Ccdc28b	coiled coil domain containing 28B	1.4451	0.04477
Agbl3	ATP/GTP binding protein-like 3	1.4433	0.03033
Rgs11	regulator of G-protein signaling 11	1.4236	0.00290
Gm45847	predicted gene 45847	1.3509	0.04522
Gm20342	predicted gene, 20342	1.2661	0.01988
Tacc2	transforming, acidic coiled-coil containing protein 2	1.2658	0.01776
Gm7901	predicted gene 7901	1.2625	0.00749
Six5	sine oculis-related homeobox 5	1.2586	0.04359
1700086O06Rik	RIKEN cDNA 1700086O06 gene	1.2406	0.01073
Pstk	phosphoseryl-tRNA kinase	1.2253	0.04389
Fam57a	family with sequence similarity 57, member A	1.2097	0.02111
Gm49528	predicted gene, 49528	1.1908	0.00872
Dnah2	dynein, axonemal, heavy chain 2	1.1687	0.04732
Rab11fip4	RAB11 family interacting protein 4 (class II)	1.1342	0.04640
Acot6	acyl-CoA thioesterase 6	1.1295	0.01248
Ppp1r12b	protein phosphatase 1, regulatory subunit 12B	1.0873	0.00071
Efna2	ephrin A2	1.0658	0.04727
Ablim2	actin-binding LIM protein 2	1.0548	0.01299
Gm15701	predicted gene 15701	1.0452	0.02051
Boc	biregional cell adhesion molecule-related/down-regulated by oncogenes (Cdon) binding protein	1.0349	0.02866
8430429K09Rik	RIKEN cDNA 8430429K09 gene	1.0072	0.04951
Gm5150	predicted gene 5150	0.9940	0.01513
Msantd1	Myb/SANT-like DNA-binding domain containing 1	0.9751	0.01011
2810013P06Rik	RIKEN cDNA 2810013P06 gene	0.9589	0.01653
Spats2	spermatogenesis associated, serine-rich 2	0.9560	0.02728
AI504432	expressed sequence AI504432	0.9374	0.00002
Ipp	IAP promoted placental gene	0.9279	0.03769
Smarcd3	SWI/SNF related, matrix associated, actin dependent regulator of chromatin, subfamily d, member 3	0.9223	0.03366
1500015A07Rik	RIKEN cDNA 1500015A07 gene	0.8965	0.03009
2900076A07Rik	RIKEN cDNA 2900076A07 gene	0.8878	0.04232
2610037D02Rik	RIKEN cDNA 2610037D02 gene	0.8774	0.02673
Gm5417	predicted gene 5417	0.8754	0.02424
AC110534.5	NLR family, pyrin domain containing (Nlrp) pseudogene	0.8721	0.01414
Ankmy2	ankyrin repeat and MYND domain containing 2	0.8473	0.01225
Jade1	jade family PHD finger 1	0.8430	0.01132
Gm41077	predicted gene, 41077	0.8410	0.02648
AC154378.1	novel transcript	0.8156	0.02470
Kcna3	potassium voltage-gated channel, shaker-related subfamily, member 3	0.8125	0.01495
Fam217b	family with sequence similarity 217, member B	0.8067	0.03600
Cxcr6	chemokine (C-X-C motif) receptor 6	0.7999	0.04124
Arg2	arginase type II	0.7953	0.00164
Gm26510	predicted gene, 26510	0.7931	0.02047
Rps26-ps1	ribosomal protein S26, pseudogene 1	0.7839	0.02910
Gm23722	predicted gene, 23722	0.7803	0.03445
Gm26280	predicted gene, 26280	0.7798	0.02994
Gm40723	predicted gene, 40723	0.7738	0.03481
Carf	calcium response factor	0.7541	0.01989
S1pr1	sphingosine-1-phosphate receptor 1	0.7415	0.03455
Ccdc66	coiled-coil domain containing 66	0.7361	0.00492
Miga1	mitoguardin 1	0.7284	0.02884
Slc18a2	solute carrier family 18 (vesicular monoamine), member 2	0.7151	0.04718
Gtf2ird1	general transcription factor II I repeat domain-containing 1	0.7026	0.01037
Rpusd1	RNA pseudouridylate synthase domain containing 1	0.6892	0.00188
Thumpd2	THUMP domain containing 2	0.6855	0.04683
Trmu	tRNA 5-methylaminomethyl-2-thiouridylate methyltransferase	0.6832	0.01024
Gcdh	glutaryl-Coenzyme A dehydrogenase	0.6765	0.02708
Spic	Spi-C transcription factor (Spi-1/PU.1 related)	0.6706	0.01814
Ercc5	excision repair cross-complementing rodent repair deficiency, complementation group 5	0.6650	0.00814

Pcgf2	polycomb group ring finger 2	0.6567	0.04613
Klhl36	kelch-like 36	0.6515	0.00701
Sema6b	sema domain, transmembrane domain (TM), and cytoplasmic domain, (semaphorin) 6B	0.6512	0.04496
Tsfm	Ts translation elongation factor, mitochondrial	0.6381	0.01455
Elk3	ELK3, member of ETS oncogene family	0.6360	0.00006
Zfp946	zinc finger protein 946	0.6325	0.01950
Dhodh	dihydroorotate dehydrogenase	0.6285	0.02370
2610008E11Rik	RIKEN cDNA 2610008E11 gene	0.6280	0.04302
Eme2	essential meiotic structure-specific endonuclease subunit 2	0.6213	0.00444
Polrmt	polymerase (RNA) mitochondrial (DNA directed)	0.6180	0.00039
Aph1b	aph1 homolog B, gamma secretase subunit	0.6141	0.02305
Carnmt1	carnosine N-methyltransferase 1	0.6128	0.01569
Susd2	sushi domain containing 2	0.6128	0.00039
Mir5125	microRNA 5125	0.6124	0.00326
AW209491	expressed sequence AW209491	0.6082	0.03131
Fam120aos	family with sequence similarity 120A, opposite strand	0.6017	0.02577
Spata13	spermatogenesis associated 13	0.5990	0.00001
Rbbp9	retinoblastoma binding protein 9, serine hydrolase	0.5979	0.01627
Xxylt1	xyloside xylosyltransferase 1	0.5870	0.00142
Ccdc84	coiled-coil domain containing 84	0.5712	0.01595
Fcho1	FCH domain only 1	0.5600	0.01074
Mtfmt	mitochondrial methionyl-tRNA formyltransferase	0.5532	0.01028
Homez	homeodomain leucine zipper-encoding gene	0.5489	0.03789
Kdelc1	KDEL (Lys-Asp-Glu-Leu) containing 1	0.5391	0.03009
Creg2	cellular repressor of E1A-stimulated genes 2	0.5329	0.00984
Htr2b	5-hydroxytryptamine (serotonin) receptor 2B	0.5297	0.02039
Trmt5	TRM5 tRNA methyltransferase 5	0.5265	0.00097
Emc9	ER membrane protein complex subunit 9	0.5202	0.04972
Cdk5rap1	CDK5 regulatory subunit associated protein 1	0.5138	0.03659
Ado	2-aminoethanethiol (cysteamine) dioxygenase	0.5136	0.01169
Usp30	ubiquitin specific peptidase 30	0.5038	0.00493
Ndor1	NADPH dependent diflavin oxidoreductase 1	0.4962	0.01696
Dph1	diphthamide biosynthesis 1	0.4922	0.03704
Gys1	glycogen synthase 1, muscle	0.4888	0.01388
Cacna1b	calcium channel, voltage-dependent, N type, alpha 1B subunit	0.4871	0.01843
Cgref1	cell growth regulator with EF hand domain 1	0.4863	0.01841
Il20rb	interleukin 20 receptor beta	0.4756	0.02947
Mthfsd	methenyltetrahydrofolate synthetase domain containing	0.4747	0.02659
Ccdc163	coiled-coil domain containing 163	0.4729	0.02568
Zfp704	zinc finger protein 704	0.4631	0.03447
Igfbp7	insulin-like growth factor binding protein 7	0.4625	0.00501
Jade3	jade family PHD finger 3	0.4623	0.01585
Klhdc2	kelch domain containing 2	0.4619	0.01951
Gt(ROSA)26Sor	gene trap ROSA 26	0.4533	0.00647
Gm14325	predicted gene 14325	0.4497	0.00606
Gm37795	predicted gene, 37795	0.4480	0.04200
Fancm	Fanconi anemia, complementation group M	0.4464	0.04429
Tmeff1	transmembrane protein with EGF-like and two follistatin-like domains 1	0.4403	0.00915
Neurl4	neuralized E3 ubiquitin protein ligase 4	0.4362	0.02214
Brd3	bromodomain containing 3	0.4362	0.00029
Zdhhc24	zinc finger, DHHC domain containing 24	0.4322	0.00918
Gm10419	predicted gene 10419	0.4281	0.02021
Tmem237	transmembrane protein 237	0.4279	0.03755
Spaca6	sperm acrosome associated 6	0.4254	0.02521
Gm44165	predicted gene, 44165	0.4231	0.02726
Xpc	xeroderma pigmentosum, complementation group C	0.4222	0.04143
Gm12940	predicted gene 12940	0.4148	0.01354
Abca1	ATP-binding cassette, sub-family A (ABC1), member 1	0.4071	0.00009
Mylip	myosin regulatory light chain interacting protein	0.4034	0.00496
Neat1	nuclear paraspeckle assembly transcript 1 (non-protein coding)	0.4027	0.00011
Akip1	A kinase (PRKA) interacting protein 1	0.3955	0.01518
Tram2	translocating chain-associating membrane protein 2	0.3953	0.00337
Mob3b	MOB kinase activator 3B	0.3943	0.03228
Mkrn2	makorin, ring finger protein, 2	0.3942	0.01411
Cfap97	cilia and flagella associated protein 97	0.3931	0.02607
Clmp	CXADR-like membrane protein	0.3924	0.00030
4632415L05Rik	RIKEN cDNA 4632415L05 gene	0.3918	0.03323
Il1rl2	interleukin 1 receptor-like 2	0.3900	0.04107
Hdac7	histone deacetylase 7	0.3889	0.01807
Gm12404	predicted gene 12404	0.3749	0.04828
Tmem181b-ps	transmembrane protein 181B, pseudogene	0.3749	0.04927
Poglut1	protein O-glucosyltransferase 1	0.3734	0.00738

Usp53	ubiquitin specific peptidase 53	0.3727	0.03273
Gm37121	predicted gene, 37121	0.3716	0.03778
Olr1	oxidized low density lipoprotein (lectin-like) receptor 1	0.3686	0.00314
Mapk14	mitogen-activated protein kinase 14	0.3666	0.03925
1700071M16Rik	RIKEN cDNA 1700071M16 gene	0.3662	0.03671
Phka2	phosphorylase kinase alpha 2	0.3633	0.01505
5830432E09Rik	RIKEN cDNA 5830432E09 gene	0.3599	0.00718
Atxn1	ataxin 1	0.3594	0.04923
Slc36a1	solute carrier family 36 (proton/amino acid symporter), member 1	0.3566	0.01287
Nlrc4	NLR family, CARD domain containing 4	0.3558	0.01753
Ulk3	unc-51-like kinase 3	0.3536	0.04454
Tgfbr1	transforming growth factor, beta receptor I	0.3509	0.00118
Jag1	jagged 1	0.3507	0.00003
Acvr1b	activin A receptor, type 1B	0.3488	0.00004
Twistnb	twist basic helix-loop-helix transcription factor 1 neighbor	0.3449	0.01291
Hmgn2	high mobility group nucleosomal binding domain 2	0.3441	0.00111
Orai2	ORAI calcium release-activated calcium modulator 2	0.3398	0.01869
Ints11	integrator complex subunit 11	0.3376	0.01107
Fam117b	family with sequence similarity 117, member B	0.3358	0.01707
Acd	adrenocortical dysplasia	0.3351	0.02041
Fbxl19	F-box and leucine-rich repeat protein 19	0.3350	0.04073
Eif2ak4	eukaryotic translation initiation factor 2 alpha kinase 4	0.3323	0.01652
Nop56	NOP56 ribonucleoprotein	0.3300	0.02734
Ppp1r8	protein phosphatase 1, regulatory subunit 8	0.3291	0.02412
Emilin1	elastin microfibril interfacer 1	0.3279	0.03378
Lhfpl2	lipoma HMGIC fusion partner-like 2	0.3190	0.00025
Acpp	acid phosphatase, prostate	0.3182	0.01695
Rnf166	ring finger protein 166	0.3174	0.04246
Vav2	vav 2 oncogene	0.3166	0.03642
Wrnip1	Werner helicase interacting protein 1	0.3151	0.03116
Leng8	leukocyte receptor cluster (LRC) member 8	0.3137	0.01871
Arhgap35	Rho GTPase activating protein 35	0.3132	0.03701
Ints5	integrator complex subunit 5	0.3118	0.00707
AI597479	expressed sequence AI597479	0.3118	0.01399
Vegfa	vascular endothelial growth factor A	0.3108	0.00917
Cnst	consortin, connexin sorting protein	0.3084	0.04009
Mavs	mitochondrial antiviral signaling protein	0.2998	0.00510
Plekha1	pleckstrin homology domain containing, family A (phosphoinositide binding specific) member 1	0.2991	0.04280
AC160637.1	novel transcript	0.2975	0.01970
Cspp1	centrosome and spindle pole associated protein 1	0.2974	0.04784
Tmem268	transmembrane protein 268	0.2941	0.00148
Txndc15	thioredoxin domain containing 15	0.2940	0.04249
Strada	STE20-related kinase adaptor alpha	0.2937	0.03898
B4galt7	xylosylprotein beta1,4-galactosyltransferase, polypeptide 7 (galactosyltransferase I)	0.2920	0.00758
Cdc42bpa	CDC42 binding protein kinase alpha	0.2918	0.03078
Nr2c2	nuclear receptor subfamily 2, group C, member 2	0.2904	0.04707
Tuba1a	tubulin, alpha 1A	0.2894	0.04047
Irak4	interleukin-1 receptor-associated kinase 4	0.2871	0.00956
Rasgrp1	RAS guanyl releasing protein 1	0.2869	0.00377
Prpf39	pre-mRNA processing factor 39	0.2820	0.00689
Zcchc4	zinc finger, CCHC domain containing 4	0.2816	0.00423
Plk3	polo like kinase 3	0.2790	0.03181
Agap1	ArfGAP with GTPase domain, ankyrin repeat and PH domain 1	0.2768	0.01314
Sdc1	syndecan 1	0.2767	0.02271
Zbtb18	zinc finger and BTB domain containing 18	0.2767	0.00273
Golga1	golgi autoantigen, golgin subfamily a, 1	0.2760	0.04881
Sgk1	serum/glucocorticoid regulated kinase 1	0.2755	0.00307
Cd33	CD33 antigen	0.2754	0.00379
Top3b	topoisomerase (DNA) III beta	0.2737	0.02509
Taf1d	TATA-box binding protein associated factor, RNA polymerase I, D	0.2731	0.01045
Skil	SKI-like	0.2705	0.00185
Dgkz	diacylglycerol kinase zeta	0.2696	0.00134
Ago3	argonaute RISC catalytic subunit 3	0.2679	0.01013
Dsel	dermatan sulfate epimerase-like	0.2674	0.01589
Fam193b	family with sequence similarity 193, member B	0.2666	0.04655
Epb41l2	erythrocyte membrane protein band 4.1 like 2	0.2637	0.00016
Wdr13	WD repeat domain 13	0.2603	0.00866
B3gnt3	UDP-GlcNAc:betaGal beta-1,3-N-acetylglucosaminyltransferase 3	0.2601	0.03000
AU020206	expressed sequence AU020206	0.2585	0.03250
Ogt	O-linked N-acetylglucosamine (GlcNAc) transferase	0.2581	0.00966
Rai14	retinoic acid induced 14	0.2557	0.00086
Plpp3	phospholipid phosphatase 3	0.2535	0.01101

Sh2b2	SH2B adaptor protein 2	0.2525	0.02715
Sema4a	semaphorin 4A	0.2496	0.01610
Slc38a6	solute carrier family 38, member 6	0.2489	0.00606
Slc25a37	solute carrier family 25, member 37	0.2480	0.00273
Zfp445	zinc finger protein 445	0.2453	0.00808
Alkbh3	alkB homolog 3, alpha-ketoglutarate-dependent dioxygenase	0.2419	0.04504
Fblim1	filamin binding LIM protein 1	0.2374	0.01693
Gde1	glycerophosphodiester phosphodiesterase 1	0.2309	0.00204
Slc27a1	solute carrier family 27 (fatty acid transporter), member 1	0.2243	0.04658
Amz2	archaelysin family metallopeptidase 2	0.2227	0.03707
Adcy7	adenylate cyclase 7	0.2197	0.02073
Eno2	enolase 2, gamma neuronal	0.2158	0.04358
Dock7	dedicator of cytokinesis 7	0.2149	0.02984
Pdgfb	platelet derived growth factor, B polypeptide	0.2147	0.02248
Thumpd3	THUMP domain containing 3	0.2141	0.02550
Ncoa4	nuclear receptor coactivator 4	0.2076	0.01081
Ptges	prostaglandin E synthase	0.2075	0.01426
Egln1	egl-9 family hypoxia-inducible factor 1	0.2063	0.04876
Rrn3	RRN3 RNA polymerase I transcription factor homolog (yeast)	0.2057	0.03985
Rnf149	ring finger protein 149	0.2050	0.00009
Smc6	structural maintenance of chromosomes 6	0.1987	0.04000
Dtx4	deltex 4, E3 ubiquitin ligase	0.1960	0.03622
Fosl2	fos-like antigen 2	0.1934	0.02589
Itgav	integrin alpha V	0.1889	0.00056
Hnrnpdl	heterogeneous nuclear ribonucleoprotein D-like	0.1882	0.02028
Fbxl5	F-box and leucine-rich repeat protein 5	0.1876	0.04442
Parg	poly (ADP-ribose) glycohydrolase	0.1861	0.03961
Top2b	topoisomerase (DNA) II beta	0.1766	0.04710
Fkbp15	FK506 binding protein 15	0.1746	0.03972
Ssh3	slingshot protein phosphatase 3	0.1742	0.04841
Inf2	inverted formin, FH2 and WH2 domain containing	0.1707	0.04393
Traf3	TNF receptor-associated factor 3	0.1706	0.03271
Camta2	calmodulin binding transcription activator 2	0.1672	0.03207
Klhl24	kelch-like 24	0.1457	0.04807
Sh3tc1	SH3 domain and tetratricopeptide repeats 1	0.1397	0.04077
Plec	plectin	0.1331	0.04786
Rab32	RAB32, member RAS oncogene family	0.1076	0.04300

Supplemental Table S2

Gene Symbol	Gene Name	log2(FC)	*P* -value
Nacad	NAC alpha domain containing	-4.7482	0.00264
Gm17796	predicted gene, 17796	-4.5169	0.01258
4930534H03Rik	RIKEN cDNA 4930534H03 gene	-4.4240	0.03585
Mir146b	microRNA 146b	-4.3618	0.01334
Gm13171	predicted gene 13171	-4.1751	0.02899
Gfy	golgi-associated olfactory signaling regulator	-4.1570	0.02181
A730063M14Rik	RIKEN cDNA A730063M14 gene	-4.1472	0.00926
Slc9b1	solute carrier family 9, subfamily B (NHA1, cation proton antiporter 1), member 1	-4.1105	0.00322
Gm28277	predicted gene 28277	-4.0713	0.01223
Smim17	small integral membrane protein 17	-4.0638	0.01118
Gm18666	predicted gene, 18666	-4.0461	0.04334
Gm43810	predicted gene 43810	-4.0327	0.00952
Gm13940	predicted gene 13940	-3.7482	0.00971
Gm16095	predicted gene 16095	-3.7154	0.02144
Gm37216	predicted gene, 37216	-3.6474	0.01375
Epha2	Eph receptor A2	-3.6370	0.02696
Gm12164	predicted gene 12164	-3.6369	0.01250
Gm26571	predicted gene, 26571	-3.6026	0.03779
Tmem240	transmembrane protein 240	-3.5387	0.01886
Gm43275	predicted gene 43275	-3.5335	0.03375
4833427F10Rik	RIKEN cDNA 4833427F10 gene	-3.4505	0.02118
C030010L15Rik	RIKEN cDNA C030010L15 gene	-3.4473	0.01924
Mir210	microRNA 210	-3.4468	0.02635
Abcc2	ATP-binding cassette, sub-family C (CFTR/MRP), member 2	-3.4344	0.03869
Atp8b5	ATPase, class I, type 8B, member 5	-3.4066	0.02518
4921522P10Rik	RIKEN cDNA 4921522P10 gene	-3.3666	0.02853
Cd200r3	CD200 receptor 3	-3.3458	0.04630
Gm29488	predicted gene 29488	-3.3457	0.01253
Gm22766	predicted gene, 22766	-3.2958	0.01641
Gm45464	predicted gene 45464	-3.2704	0.01915
Gm44101	predicted gene, 44101	-3.2642	0.04384
Gm29284	predicted gene 29284	-3.2134	0.01500
Gm20517	predicted gene 20517	-2.9215	0.04289
Gm43566	predicted gene 43566	-2.9210	0.02986
Gm43289	predicted gene 43289	-2.6824	0.02676
Notch4	notch 4	-2.4947	0.04115
Serpini1	serine (or cysteine) peptidase inhibitor, clade I, member 1	-2.4059	0.00139
Gm16201	predicted gene 16201	-2.3844	0.01406
Cmya5	cardiomyopathy associated 5	-2.3146	0.03896
Gm16273	predicted gene 16273	-2.3116	0.04144
Gm8034	predicted gene 8034	-2.2924	0.01046
Tlr11	toll-like receptor 11	-2.2092	0.00073
Fat1	FAT atypical cadherin 1	-2.1670	0.01838
D530018E20Rik	RIKEN cDNA D530018E20 gene	-2.1645	0.02501
Gm44573	predicted gene 44573	-2.1025	0.04924
Gm43070	predicted gene 43070	-2.0611	0.03215
Gm43112	predicted gene 43112	-2.0355	0.02284
4930539J05Rik	RIKEN cDNA 4930539J05 gene	-2.0269	0.01062
Ccdc162	coiled-coil domain containing 162	-1.9460	0.03707
AC122901.1	TEC	-1.9409	0.01998
Gm9905	predicted gene 9905	-1.9167	0.04243
Taf9	TATA-box binding protein associated factor 9	-1.8955	0.02530
Gm28791	predicted gene 28791	-1.8736	0.01098
Prss16	protease, serine 16 (thymus)	-1.8354	0.03011
Gemin6	gem nuclear organelle associated protein 6	-1.7286	0.00704
Rapgef3os2	Rap guanine nucleotide exchange factor (GEF) 3, opposite strand 2	-1.6491	0.03519
Nfatc2	nuclear factor of activated T cells, cytoplasmic, calcineurin dependent 2	-1.6391	0.01725
Gm45479	predicted gene 45479	-1.6285	0.02941
Gm10076	predicted gene 10076	-1.5682	0.04292
Gm38236	predicted gene, 38236	-1.5024	0.02044
Ccl8	chemokine (C-C motif) ligand 8	-1.4660	0.00231
Llph-ps2	LLP homolog, pseudogene 2	-1.4109	0.02172
Gm10484	predicted gene 10484	-1.3797	0.03668
Abcb6	ATP-binding cassette, sub-family B (MDR/TAP), member 6	-1.3738	0.03432
Tagln3	transgelin 3	-1.3028	0.02927
Pheta2	PH domain containing endocytic trafficking adaptor 2	-1.2936	0.00652

Gm26780	predicted gene, 26780	-1.2768	0.04126
9030025P20Rik	RIKEN cDNA 9030025P20 gene	-1.2756	0.01318
Gm13342	predicted gene 13342	-1.2455	0.04391
Unc93a	unc-93 homolog A	-1.2448	0.01361
Sparc	secreted acidic cysteine rich glycoprotein	-1.1804	0.03241
Gm47586	predicted gene, 47586	-1.1608	0.04839
Gm14719	predicted gene 14719	-1.1118	0.03267
Mroh8	maestro heat-like repeat family member 8	-1.0914	0.02618
Gm38048	predicted gene, 38048	-1.0618	0.00636
Gm17812	predicted gene, 17812	-1.0342	0.02624
AC168220.3	novel transcript, antisense to Dopey2 and Morc3	-1.0163	0.02229
Cdc6	cell division cycle 6	-1.0135	0.03808
Lyl1	lymphoblastomic leukemia 1	-0.9504	0.02922
Gm15501	predicted pseudogene 15501	-0.9194	0.04399
Gm43813	predicted gene 43813	-0.9141	0.04652
Apol9a	apolipoprotein L 9a	-0.9057	0.01685
Rusc1	RUN and SH3 domain containing 1	-0.8917	0.01874
Gm47920	predicted gene, 47920	-0.8517	0.04360
Gm8797	predicted pseudogene 8797	-0.8505	0.02183
Gm14010	predicted gene 14010	-0.8148	0.00862
Dmtn	dematin actin binding protein	-0.8078	0.03239
Gm7536	predicted gene 7536	-0.7958	0.00029
Zfp97	zinc finger protein 97	-0.7660	0.01700
1700001K19Rik	RIKEN cDNA 1700001K19 gene	-0.7613	0.02566
Gm8129	predicted pseudogene 8129	-0.7479	0.00051
Dnmt3c	DNA methyltransferase 3C	-0.7466	0.02454
Lrmp	lymphoid-restricted membrane protein	-0.7440	0.02919
Rpl34-ps1	ribosomal protein L34, pseudogene 1	-0.7231	0.00054
Gm15559	predicted gene 15559	-0.7136	0.03720
Rpl21	ribosomal protein L21	-0.6903	0.00090
Rsph9	radial spoke head 9 homolog (Chlamydomonas)	-0.6787	0.01796
Gm23935	predicted gene, 23935	-0.6646	0.00061
Gm26917	predicted gene, 26917	-0.6582	0.00033
Timm10b	translocase of inner mitochondrial membrane 10B	-0.6513	0.03971
Rpl10-ps1	ribosomal protein L10, pseudogene 1	-0.6501	0.01999
Gm24270	predicted gene, 24270	-0.6395	0.00264
Gm26532	predicted gene, 26532	-0.6337	0.03917
Gm15682	predicted gene 15682	-0.6165	0.00885
1110065P20Rik	RIKEN cDNA 1110065P20 gene	-0.6096	0.02114
Upp1	uridine phosphorylase 1	-0.5992	0.02345
Il12rb1	interleukin 12 receptor, beta 1	-0.5658	0.01146
Rps13-ps2	ribosomal protein S13, pseudogene 2	-0.5625	0.04241
CT010467.1	18s RNA, related sequence 5	-0.5614	0.00158
Igsf3	immunoglobulin superfamily, member 3	-0.5578	0.01850
4930523C07Rik	RIKEN cDNA 4930523C07 gene	-0.5438	0.04818
Gm18852	predicted gene, 18852	-0.5428	0.02996
Klrg2	killer cell lectin-like receptor subfamily G, member 2	-0.5355	0.04341
2310001H17Rik	RIKEN cDNA 2310001H17 gene	-0.5307	0.02589
Ptx3	pentraxin related gene	-0.5195	0.02631
Fn1	fibronectin 1	-0.4960	0.02377
Tmem171	transmembrane protein 171	-0.4882	0.01398
Lars2	leucyl-tRNA synthetase, mitochondrial	-0.4860	0.01174
Nol4l	nucleolar protein 4-like	-0.4798	0.02670
Lta	lymphotoxin A	-0.4712	0.02284
Il27	interleukin 27	-0.4702	0.03469
Adamts4	a disintegrin-like and metallopeptidase (reprolysin type) with thrombospondin type 1 motif, 4	-0.4603	0.00885
Kif5c	kinesin family member 5C	-0.4592	0.04799
Lyz1	lysozyme 1	-0.4566	0.03061
Csf3	colony stimulating factor 3 (granulocyte)	-0.4542	0.01299
Rnf219	ring finger protein 219	-0.4488	0.03245
Ccl2	chemokine (C-C motif) ligand 2	-0.4326	0.01854
Gm10443	predicted pseudogene 10443	-0.4315	0.01480
Hdc	histidine decarboxylase	-0.4283	0.04786
Bambi	BMP and activin membrane-bound inhibitor	-0.4269	0.01747
Raet1d	retinoic acid early transcript delta	-0.4179	0.03811
Phactr2	phosphatase and actin regulator 2	-0.4126	0.00028
Pttg1	pituitary tumor-transforming gene 1	-0.4112	0.02121
Gdf15	growth differentiation factor 15	-0.4101	0.00000
Nr4a3	nuclear receptor subfamily 4, group A, member 3	-0.3968	0.03815

mt-Co3	mitochondrially encoded cytochrome c oxidase III	-0.3956	0.04283
Cd80	CD80 antigen	-0.3938	0.01049
Bcl2l1	BCL2-like 1	-0.3829	0.02914
Gm9843	predicted gene 9843	-0.3824	0.03103
Il12b	interleukin 12b	-0.3768	0.02129
Gm10053	predicted gene 10053	-0.3723	0.01628
Anapc13	anaphase promoting complex subunit 13	-0.3695	0.00609
Lrrc32	leucine rich repeat containing 32	-0.3651	0.00426
Tecpr2	tectonin beta-propeller repeat containing 2	-0.3645	0.03283
Fam53a	family with sequence similarity 53, member A	-0.3619	0.03286
Bbc3	BCL2 binding component 3	-0.3472	0.02034
Hhex	hematopoietically expressed homeobox	-0.3404	0.01792
Jade2	jade family PHD finger 2	-0.3357	0.02761
Ebi3	Epstein-Barr virus induced gene 3	-0.3314	0.00509
Lsp1	lymphocyte specific 1	-0.3271	0.00035
Otud1	OTU domain containing 1	-0.3258	0.00398
Gm7730	predicted gene 7730	-0.3230	0.03798
Bcl2a1b	B cell leukemia/lymphoma 2 related protein A1b	-0.3213	0.04219
Phc2	polyhomeotic 2	-0.3207	0.02333
Slc26a2	solute carrier family 26 (sulfate transporter), member 2	-0.3177	0.00717
Basp1	brain abundant, membrane attached signal protein 1	-0.3171	0.01309
1110038F14Rik	RIKEN cDNA 1110038F14 gene	-0.3162	0.01247
Rpl19-ps11	ribosomal protein L19, pseudogene 11	-0.3143	0.00590
Irf1	interferon regulatory factor 1	-0.3133	0.01163
Klf4	Kruppel-like factor 4 (gut)	-0.3102	0.04394
Jund	jun D proto-oncogene	-0.3051	0.01885
Uqcrh	ubiquinol-cytochrome c reductase hinge protein	-0.3017	0.00723
Rras2	related RAS viral (r-ras) oncogene 2	-0.3000	0.02558
Hmgn5	high-mobility group nucleosome binding domain 5	-0.2918	0.04278
Tmsb10	thymosin, beta 10	-0.2910	0.00034
Fut11	fucosyltransferase 11	-0.2901	0.04397
Commd1	COMM domain containing 1	-0.2892	0.03066
Junb	jun B proto-oncogene	-0.2843	0.02476
Ndufb6	NADH:ubiquinone oxidoreductase subunit B6	-0.2740	0.01978
Zfp513	zinc finger protein 513	-0.2680	0.02635
S100a11	S100 calcium binding protein A11	-0.2674	0.01557
Fam49a	family with sequence similarity 49, member A	-0.2632	0.01822
Hmga1	high mobility group AT-hook 1	-0.2616	0.00708
Nuak2	NUAK family, SNF1-like kinase, 2	-0.2600	0.03604
Mtmr14	myotubularin related protein 14	-0.2561	0.04700
Ccl3	chemokine (C-C motif) ligand 3	-0.2493	0.04268
Gm4332	predicted gene 4332	-0.2442	0.02501
Trim13	tripartite motif-containing 13	-0.2439	0.00237
Tnf	tumor necrosis factor	-0.2439	0.00656
Limd2	LIM domain containing 2	-0.2438	0.04492
Pdzd11	PDZ domain containing 11	-0.2429	0.02012
Ahsa1	AHA1, activator of heat shock protein ATPase 1	-0.2417	0.00342
Ptgs2	prostaglandin-endoperoxide synthase 2	-0.2407	0.04009
Cebpd	CCAAT/enhancer binding protein (C/EBP), delta	-0.2382	0.04002
Cd99l2	CD99 antigen-like 2	-0.2382	0.03720
Nlrp3	NLR family, pyrin domain containing 3	-0.2381	0.01976
Dhrs3	dehydrogenase/reductase (SDR family) member 3	-0.2372	0.00651
Rab5if	RAB5 interacting factor	-0.2358	0.01151
Cpq	carboxypeptidase Q	-0.2341	0.03554
Psme2b	protease (prosome, macropain) activator subunit 2B	-0.2338	0.02315
Sdc4	syndecan 4	-0.2336	0.03161
Stim1	stromal interaction molecule 1	-0.2278	0.01389
Bahd1	bromo adjacent homology domain containing 1	-0.2268	0.01885
Atp6v1b2	ATPase, H+ transporting, lysosomal V1 subunit B2	-0.2266	0.04478
Chmp2b	charged multivesicular body protein 2B	-0.2263	0.00224
Pmpcb	peptidase (mitochondrial processing) beta	-0.2258	0.02557
Rps21	ribosomal protein S21	-0.2253	0.01553
Arl5c	ADP-ribosylation factor-like 5C	-0.2242	0.02205
Vps4a	vacuolar protein sorting 4A	-0.2236	0.04247
Igbp1	immunoglobulin (CD79A) binding protein 1	-0.2228	0.04998
Map3k11	mitogen-activated protein kinase kinase kinase 11	-0.2214	0.01150
Elob	elongin B	-0.2199	0.02926
Arhgap22	Rho GTPase activating protein 22	-0.2194	0.04963
Rasgef1b	RasGEF domain family, member 1B	-0.2192	0.00252

Dio2	deiodinase, iodothyronine, type II	-0.2181	0.02762
Ssr4	signal sequence receptor, delta	-0.2179	0.01423
Adgrg6	adhesion G protein-coupled receptor G6	-0.2174	0.00962
Cd2bp2	CD2 antigen (cytoplasmic tail) binding protein 2	-0.2159	0.01381
Ankrd33b	ankyrin repeat domain 33B	-0.2143	0.01295
Znhit1	zinc finger, HIT domain containing 1	-0.2118	0.01945
Nfkbib	nuclear factor of kappa light polypeptide gene enhancer in B cells inhibitor, beta	-0.2110	0.00664
CAAA01118383.1	dehydrogenase/reductase (SDR family) X chromosome	-0.2109	0.01720
Ier5	immediate early response 5	-0.2090	0.04393
Snx6	sorting nexin 6	-0.2087	0.01086
Siah2	siah E3 ubiquitin protein ligase 2	-0.2079	0.03945
Nrros	negative regulator of reactive oxygen species	-0.2078	0.03594
Ppp4r2	protein phosphatase 4, regulatory subunit 2	-0.2070	0.01043
Sap30	sin3 associated polypeptide	-0.2044	0.00704
Rac2	Rac family small GTPase 2	-0.2017	0.02309
Slc2a6	solute carrier family 2 (facilitated glucose transporter), member 6	-0.1996	0.03776
Tspan3	tetraspanin 3	-0.1987	0.01651
Ddit3	DNA-damage inducible transcript 3	-0.1986	0.03642
Snx20	sorting nexin 20	-0.1959	0.00750
Spp1	secreted phosphoprotein 1	-0.1935	0.00332
Rhoc	ras homolog family member C	-0.1930	0.02956
Pxn	paxillin	-0.1922	0.04762
Creb3	cAMP responsive element binding protein 3	-0.1918	0.04340
Ccl5	chemokine (C-C motif) ligand 5	-0.1885	0.02219
Il2rg	interleukin 2 receptor, gamma chain	-0.1879	0.03347
Bcl2a1a	B cell leukemia/lymphoma 2 related protein A1a	-0.1870	0.02758
C5ar1	complement component 5a receptor 1	-0.1862	0.04049
Rps20	ribosomal protein S20	-0.1860	0.03193
Bcl2a1d	B cell leukemia/lymphoma 2 related protein A1d	-0.1857	0.01280
Pcna	proliferating cell nuclear antigen	-0.1843	0.00811
Aggf1	angiogenic factor with G patch and FHA domains 1	-0.1834	0.00771
Cebpb	CCAAT/enhancer binding protein (C/EBP), beta	-0.1822	0.02558
Atp6v0a1	ATPase, H+ transporting, lysosomal V0 subunit A1	-0.1770	0.00317
Nsfl1c	NSFL1 (p97) cofactor (p47)	-0.1765	0.03682
Fcer1g	Fc receptor, IgE, high affinity I, gamma polypeptide	-0.1736	0.00366
Swap70	SWA-70 protein	-0.1699	0.00888
Pcbp1	poly(rC) binding protein 1	-0.1695	0.00510
Fam129a	family with sequence similarity 129, member A	-0.1693	0.01624
Mocos	molybdenum cofactor sulfurase	-0.1691	0.04295
Mrpl30	mitochondrial ribosomal protein L30	-0.1685	0.04166
Rps24	ribosomal protein S24	-0.1664	0.00988
Riox2	ribosomal oxygenase 2	-0.1637	0.04736
Ccl9	chemokine (C-C motif) ligand 9	-0.1637	0.00963
Rtf2	replication termination factor 2	-0.1636	0.03567
Maea	macrophage erythroblast attacher	-0.1627	0.03634
Szrd1	SUZ RNA binding domain containing 1	-0.1623	0.01774
Keap1	kelch-like ECH-associated protein 1	-0.1585	0.03551
Mmadhc	methylmalonic aciduria (cobalamin deficiency) cblD type, with homocystinuria	-0.1546	0.04679
Ms4a7	membrane-spanning 4-domains, subfamily A, member 7	-0.1519	0.02881
Trp53inp2	transformation related protein 53 inducible nuclear protein 2	-0.1507	0.04025
Akr1a1	aldo-keto reductase family 1, member A1 (aldehyde reductase)	-0.1479	0.01500
Cdkn1a	cyclin-dependent kinase inhibitor 1A (P21)	-0.1474	0.04554
Rusc2	RUN and SH3 domain containing 2	-0.1473	0.01571
Necap2	NECAP endocytosis associated 2	-0.1459	0.02820
Cap1	CAP, adenylate cyclase-associated protein 1 (yeast)	-0.1450	0.01957
Spi1	spleen focus forming virus (SFFV) proviral integration oncogene	-0.1407	0.03455
Rilpl2	Rab interacting lysosomal protein-like 2	-0.1373	0.04321
Stk4	serine/threonine kinase 4	-0.1354	0.03597
Psma2	proteasome (prosome, macropain) subunit, alpha type 2	-0.1348	0.04555
Tmsb4x	thymosin, beta 4, X chromosome	-0.1307	0.01714
Fxyd5	FXYD domain-containing ion transport regulator 5	-0.1279	0.04466
Ets2	E26 avian leukemia oncogene 2, 3' domain	-0.1254	0.02949
Bag6	BCL2-associated athanogene 6	-0.1243	0.04615
Clptm1	cleft lip and palate associated transmembrane protein 1	-0.1219	0.04109
Ehd1	EH-domain containing 1	-0.1176	0.03771
Capg	capping protein (actin filament), gelsolin-like	-0.1149	0.04693
Psmd8	proteasome (prosome, macropain) 26S subunit, non-ATPase, 8	-0.1091	0.04977

Supplementary Table S3. A list of proteins in Ctrp6-treated BMDMs quantitated by TMT

Gene Symbol	UniProt Accession Number	Protein	Fold Change (Ctrp6- vs Vehicle-treated BMDMs)	p-value
Pmf1	Q9CPV5	Polyamine-modulated factor 1	0.79	0.08
Cenpe	Q6RT24	Centromere-associated protein E	0.79	0.01
A2m	Q6GQT1	Alpha-2-macroglobulin-P	0.80	0.03
Epc2	Q8C0I4	Enhancer of polycomb homolog 2	0.80	0.30
Vsir	A0A171EBK7	RIKEN cDNA 4632428N05, isoform CRA_b	0.82	0.01
Klhl11	Q8CE33	Kelch-like protein 11	0.82	0.18
Polr3e	Q9CZT4	DNA-directed RNA polymerase III subunit RPC5	0.82	0.31
Tmem115	Q9WUH1	Transmembrane protein 115	0.83	0.14
Polr1c	G3UX92	DNA-directed RNA polymerases I and III subunit RPAC1	0.84	0.28
Rac3	P60764	Ras-related C3 botulinum toxin substrate 3	0.84	0.44
Sirt5	A0A1Y7VM56	NAD-dependent protein deacylase sirtuin-5, mitochondrial	0.85	0.38
Chst11	Q9JME2	Carbohydrate sulfotransferase 11	0.85	0.16
Clcn3	P51791	H(+)/Cl(-) exchange transporter 3	0.85	0.05
Slc26a11	Q80ZD3	Sodium-independent sulfate anion transporter	0.86	0.29
Rbx1	P62878	E3 ubiquitin-protein ligase RBX1	0.86	0.40
Acap3	Q6NXL5	ArfGAP with coiled-coil, ankyrin repeat and PH domains 3	0.86	0.03
Clec4d	Q9Z2H6	C-type lectin domain family 4 member D	0.86	0.29
Nipsnap3b	Q9CQE1	Protein NipSnap homolog 3B	0.86	0.63
Foxred2	Q3USW5	FAD-dependent oxidoreductase domain-containing protein 2	0.86	0.01
Dbndd2	Q9CRD4	Dysbindin domain-containing protein 2	0.87	0.07
Mt2	P02798	Metallothionein-2	0.87	0.13
Mt1	P02802	Metallothionein-1	0.87	0.09
Tulp3	O88413	Tubby-related protein 3	0.87	0.15
Fhl2	O70433	Four and a half LIM domains protein 2	0.87	0.18
Kat5	Q8CHK4	Histone acetyltransferase KAT5	0.87	0.37
Rspry1	Q8BVR6	RING finger and SPRY domain-containing protein 1	0.87	0.00
Armc7	Q3UJZ3	Armadillo repeat-containing protein 7	0.87	0.06
Cytip	Q91VY6	Cytohesin-interacting protein	0.87	0.04
Fam234b	Q8BYI8	Protein FAM234B	0.88	0.51
Kctd17	E0CYQ0	MCG13350, isoform CRA_b	0.88	0.29
Kif1b	A2AH75	Kinesin family member 1B, isoform CRA_a	0.88	0.31
Hist1h2ae	C0HKE4	Histone H2A type 1-E	0.88	0.46
Snx21	Q3UR97	Sorting nexin-21	0.88	0.49
Kxd1	E9QNP0	KxDL motif-containing protein 1	0.88	0.47
Ptrhd1	D3Z4S3	Putative peptidyl-tRNA hydrolase PTRHD1	0.88	0.32
Med7	Q9CZB6	Mediator of RNA polymerase II transcription subunit 7	0.88	0.35
Lysmd4	Q8CC84	LysM and putative peptidoglycan-binding domain-containing protein 4	0.89	0.30
Nepro	Q8R2U2	Nucleolus and neural progenitor protein	0.89	0.36
Ccl6	P27784	C-C motif chemokine 6	0.89	0.45
Atp5f1c	Q91VR2	ATP synthase subunit gamma, mitochondrial	0.89	0.20
Ninj1	D3YW25	Ninjurin 1, isoform CRA_a	0.89	0.21
Senp5	Q6NXL6	Sentrin-specific protease 5	0.89	0.12
Chpf2	Q3UU43	Hexosyltransferase	0.89	0.10
Rnf220	Q6PDX6	E3 ubiquitin-protein ligase Rnf220	0.89	0.55
Ogfrl1	Q8VE52	Opioid growth factor receptor-like protein 1	0.89	0.28
Rpf1	Q7TND5	Ribosome production factor 1	0.90	0.37
Chchd7	Q8K2Q5	Coiled-coil-helix-coiled-coil-helix domain-containing protein 7	0.90	0.46
Usp36	B1AQJ2	Ubiquitin carboxyl-terminal hydrolase 36	0.90	0.06
Tusc1	Q673H1	Tumor suppressor candidate gene 1 protein homolog	0.90	0.40
Sec61a2	Q9JLR1	Protein transport protein Sec61 subunit alpha isoform 2	0.90	0.41
Sirpa	E0CYM8	Tyrosine-protein phosphatase non-receptor type substrate 1	0.90	0.08
Prrc2c	S4R2E2	Protein PRRC2C (Fragment)	0.90	0.37
Hcfc2	G5E837	Host cell factor 2	0.90	0.33
Napsa	O09043	Napsin-A	0.90	0.32
Polg	P54099	DNA polymerase subunit gamma-1	0.90	0.38
Tk2	Q8BN51	Thymidine kinase 2, mitochondrial	0.91	0.15
Abca7	E9Q6G4	ATP-binding cassette sub-family A member 7	0.91	0.14
Ddit3	P35639	DNA damage-inducible transcript 3 protein	0.91	0.32
Pgam2	O70250	Phosphoglycerate mutase 2	0.91	0.19
N4bp2l2	Q8JZS6	NEDD4-binding protein 2-like 2	0.91	0.19
Prmt9	F8WIU7	Protein arginine N-methyltransferase 9	0.91	0.15
Herpud2	Q9JJC9	Homocysteine-responsive endoplasmic reticulum-resident ubiquitin-like domain member 2 protein	0.91	0.41
Dnajb9	Q9QYI6	DnaJ homolog subfamily B member 9	0.91	0.09
Hook2	Q7TMK6	Protein Hook homolog 2	0.91	0.40
Cbll1	Q9JIY2	E3 ubiquitin-protein ligase Hakai	0.91	0.42
Arf3	P61205	ADP-ribosylation factor 3	0.91	0.26
Ddt	O35215	D-dopachrome decarboxylase	0.91	0.16
Chrac1	Q9JKP8	Chromatin accessibility complex protein 1	0.91	0.00
Casp2	P29594	Caspase-2	0.91	0.26

Pcbp2	A0A2R8VHL8	Poly(rC)-binding protein 2 (Fragment)	0.91	0.34
Pot1	Q91WC1	Protection of telomeres protein 1	0.91	0.28
Kiaa1109	A2AAE1	Transmembrane protein KIAA1109	0.91	0.30
Plg	P20918	Plasminogen	0.91	0.02
Pold4	Q9CWP8	DNA polymerase delta subunit 4	0.92	0.22
Dhrs13	Q5SS80	Dehydrogenase/reductase SDR family member 13	0.92	0.32
Phykpl	Q8R1K4	5-phosphohydroxy-L-lysine phospho-lyase	0.92	0.27
Asap2	E9PX52	Arf-GAP with SH3 domain, ANK repeat and PH domain-containing protein 2	0.92	0.54
Rfxank	Q9Z205	DNA-binding protein RFXANK	0.92	0.37
Naip5	Q9R016	Baculoviral IAP repeat-containing protein 1e	0.92	0.61
P2ry6	Q9ERK9	P2Y purinoceptor 6	0.92	0.16
Haus2	Q9CQS9	HAUS augmin-like complex subunit 2	0.92	0.30
Samd1	D3YXK1	Atherin	0.92	0.34
	Q5EBG8	Uncharacterized protein C1orf50 homolog	0.92	0.19
Chdh	Q8BJ64	Choline dehydrogenase, mitochondrial	0.92	0.20
Rps28	P62858	40S ribosomal protein S28	0.92	0.37
Pms1	Q8K119	PMS1 homolog 1, mismatch repair system component	0.92	0.08
Clk2	E9Q5Y1	Dual-specificity protein kinase CLK2	0.92	0.30
Nabp1	Q8BGW5	SOSS complex subunit B2	0.92	0.37
Ppp6r2	G3X9K4	SAPS domain family, member 2, isoform CRA_a	0.92	0.49
Hacl1	Q9QXE0	2-hydroxyacyl-CoA lyase 1	0.92	0.05
Ptprs	B0V2N1	Receptor-type tyrosine-protein phosphatase S	0.92	0.20
Hace1	Q3U0D9	E3 ubiquitin-protein ligase HACE1	0.92	0.01
Hpgd	Q8VCC1	15-hydroxyprostaglandin dehydrogenase [NAD(+)]	0.92	0.41
Dhdds	Q99KU1	Dehydrodolichyl diphosphate synthase complex subunit Dhdds	0.92	0.59
Lsp1	A2A6J7	Lymphocyte-specific protein 1	0.92	0.41
Ttr	P07309	Transthyretin	0.92	0.07
Gab1	A0A1B0GS41	GRB2-associated-binding protein 1	0.92	0.12
Dolk	Q8R2Y3	Dolichol kinase	0.92	0.44
Npm3	Q9CPP0	Nucleoplasmin-3	0.92	0.50
Pop4	Q9CR08	Ribonuclease P protein subunit p29	0.92	0.45
Rps6kb1	Q8BSK8	Ribosomal protein S6 kinase beta-1	0.93	0.34
Rab11a	P62492	Ras-related protein Rab-11A	0.93	0.69
Fam126a	Q6P9N1	Hyccin	0.93	0.15
Zmym2	Q9CU65	Zinc finger MYM-type protein 2	0.93	0.17
Qtrt1	Q9JMA2	Queuine tRNA-ribosyltransferase catalytic subunit 1	0.93	0.12
Mcm8	Q9CWV1	DNA helicase MCM8	0.93	0.42
Slmap	F6WY34	Sarcolemmal membrane-associated protein (Fragment)	0.93	0.42
Lpin1	E9QKQ5	Phosphatidate phosphatase LPIN1	0.93	0.50
S100a10	P08207	Protein S100-A10	0.93	0.39
Crlf2	A0A0R4J0F5	Cytokine receptor-like factor 2	0.93	0.34
Phospho1	Q8R2H9	Phosphoethanolamine/phosphocholine phosphatase	0.93	0.31
Nfkbia	Q9Z1E3	NF-kappa-B inhibitor alpha	0.93	0.03
Lrrk2	Q5S006	Leucine-rich repeat serine/threonine-protein kinase 2	0.93	0.06
Kif13a	F8VQ75	Kinesin-like protein KIF13A	0.93	0.39
Atp6v0c	P63082	V-type proton ATPase 16 kDa proteolipid subunit	0.93	0.15
Fbxw17	Q8CFE8	F-box and WD-40 domain protein 17	0.93	0.81
Myo5a	D3Z4J3	Unconventional myosin-Va	0.93	0.46
Dennd4a	E9Q8V6	DENN/MADD domain-containing 4A	0.93	0.43
Ly6a	P05533	Lymphocyte antigen 6A-2/6E-1	0.93	0.57
Uri1	A0A0U1RNX4	Unconventional prefoldin RPB5 interactor (Fragment)	0.93	0.03
Med1	Q925J9	Mediator of RNA polymerase II transcription subunit 1	0.93	0.22
Abcb4	P21440	Phosphatidylcholine translocator ABCB4	0.93	0.14
Laptm5	Q61168	Lysosomal-associated transmembrane protein 5	0.93	0.50
Gm49359	Q14DI0	Predicted gene, 49359	0.93	0.26
Ift57	Q8BXG3	Intraflagellar transport protein 57 homolog	0.93	0.65
Daxx	Q3UKR0	Death domain-associated protein 6	0.93	0.20
Mblac2	Q8BL86	Metallo-beta-lactamase domain-containing protein 2	0.93	0.47
Rhbdd2	Q8VEK2	Rhomboid domain-containing protein 2	0.93	0.26
Nacc2	Q9DCM7	Nucleus accumbens-associated protein 2	0.93	0.05
Trim41	Q5NCC3	E3 ubiquitin-protein ligase TRIM41	0.93	0.02
Folr1	P35846	Folate receptor alpha	0.93	0.55
Mlh1	Q9JK91	DNA mismatch repair protein Mlh1	0.93	0.28
Serpinb1b	Q8VHP7	Leukocyte elastase inhibitor B	0.93	0.40
Foxp1	D3Z6Q3	Forkhead box protein P1	0.93	0.02
Terf2ip	Q91VL8	Telomeric repeat-binding factor 2-interacting protein 1	0.93	0.12
Fam118a	Q91YN1	Protein FAM118A	0.94	0.50
Tmem208	Q9CR96	Transmembrane protein 208	0.94	0.38
Trim11	Q99PQ2	E3 ubiquitin-protein ligase TRIM11	0.94	0.33
Thbs1	Q80YQ1	Thrombospondin-1	0.94	0.35
Dhps	Q3TXU5	Deoxyhypusine synthase	0.94	0.59
Gtf2a1	Q99PM3	Transcription initiation factor IIA subunit 1	0.94	0.45
Pkig	O70139	cAMP-dependent protein kinase inhibitor gamma	0.94	0.26

Itih3	Q61704	Inter-alpha-trypsin inhibitor heavy chain H3	0.94	0.47
Hmg20a	Q9DC33	High mobility group protein 20A	0.94	0.38
Tmem59	Q9QY73	Transmembrane protein 59	0.94	0.61
Commd6	Q3V4B5	COMM domain-containing protein 6	0.94	0.04
Cdk7	Q03147	Cyclin-dependent kinase 7	0.94	0.02
Mis12	Q9CY25	Protein MIS12 homolog	0.94	0.22
Ift140	E9PY46	Intraflagellar transport protein 140 homolog	0.94	0.36
Lamtor4	Q8CF66	Ragulator complex protein LAMTOR4	0.94	0.27
Cpeb2	E9Q969	Cytoplasmic polyadenylation element-binding protein 2	0.94	0.55
Ess2	Q3UFM6	Expressed sequence 2 embryonic lethal, isoform CRA_a	0.94	0.15
Mroh4	G3X8W1	MCG2797	0.94	0.36
Mbnl1	G3X9Q0	Muscleblind-like 1 (Drosophila), isoform CRA_a	0.94	0.42
Ints15	Q8BGA7	Integrator complex subunit 15	0.94	0.36
Poli	E9QJU6	DNA polymerase iota	0.94	0.08
Cpped1	Q8BFS6	Serine/threonine-protein phosphatase CPPED1	0.94	0.27
Slc31a2	Q9CPU9	Probable low affinity copper uptake protein 2	0.94	0.45
8030462N17Rik	Q0VAW6	RIKEN cDNA 8030462N17 gene	0.94	0.03
Ccnh	Q3UUW5	Cyclin-H	0.94	0.50
Tysnd1	Q9DBA6	Peroxisomal leader peptide-processing protease	0.94	0.62
Rapgef6	Q5NCJ1	Rap guanine nucleotide exchange factor (GEF) 6	0.94	0.49
Ptcd1	Q8C2E4	Pentatricopeptide repeat-containing protein 1, mitochondrial	0.94	0.39
Tmem160	Q9D938	Transmembrane protein 160	0.94	0.29
Zbed3	Q9D0L1	Zinc finger BED domain-containing protein 3	0.94	0.53
Cdk5rap2	Q8K389	CDK5 regulatory subunit-associated protein 2	0.94	0.08
Inpp5k	Q8C5L6	Inositol polyphosphate 5-phosphatase K	0.94	0.37
Znrf1	Q91V17	E3 ubiquitin-protein ligase ZNRF1	0.94	0.52
Ehbp1	Q69ZW3	EH domain-binding protein 1	0.94	0.37
Asah2	Q9JHE3	Neutral ceramidase	0.94	0.33
Alkbh8	Q80Y20	Alkylated DNA repair protein alkB homolog 8	0.95	0.16
Pigl	Q5SX19	N-acetylglucosaminyl-phosphatidylinositol de-N-acetylase	0.95	0.07
Cyth1	Q3TZ02	Cytohesin-1	0.95	0.16
Pias2	Q8C5D8	E3 SUMO-protein ligase PIAS2	0.95	0.48
Pigo	Q562G0	GPI ethanolamine phosphate transferase 3	0.95	0.50
Vnn1	Q9Z0K8	Pantetheinase	0.95	0.56
Actbl2	Q8BFZ3	Beta-actin-like protein 2	0.95	0.64
Scnm1	Q8K136	Sodium channel modifier 1	0.95	0.36
Cop1	Q9R1A8	E3 ubiquitin-protein ligase COP1	0.95	0.46
Pdxp	P60487	Pyridoxal phosphate phosphatase	0.95	0.64
Gm11639	A0A1D5RLM8	Predicted gene 11639	0.95	0.32
Ogfod2	Q9CQ04	2-oxoglutarate and iron-dependent oxygenase domain-containing protein 2	0.95	0.36
Tdp2	Q9JJX7	Tyrosyl-DNA phosphodiesterase 2	0.95	0.40
Tbc1d22b	Q80VE5	TBC1 domain family, member 22B	0.95	0.48
Eef1d	F6ZFU0	Elongation factor 1-delta (Fragment)	0.95	0.90
Csf2rb	P26955	Cytokine receptor common subunit beta	0.95	0.66
Clic5	Q8BXK9	Chloride intracellular channel protein 5	0.95	0.58
Wbp2	P97765	WW domain-binding protein 2	0.95	0.31
Anapc16	S4R2B6	Anaphase-promoting complex subunit 16	0.95	0.48
C2cd2	E9Q3C1	C2 domain-containing protein 2	0.95	0.26
Rcbtb1	A0A0R4J025	RCC1 and BTB domain-containing protein 1	0.95	0.53
Men1	A0A0R4J1I3	Menin	0.95	0.49
Tanc2	A2A690	Protein TANC2	0.95	0.24
Tldc1	Q8K0P3	TLD domain-containing protein 1	0.95	0.36
Gps1	A0A140LJB7	COP9 signalosome complex subunit 1	0.95	0.10
Sh3tc1	G3X9F6	SH3 domain and tetratricopeptide repeats 1	0.95	0.50
Polr2e	Q80UW8	DNA-directed RNA polymerases I, II, and III subunit RPABC1	0.95	0.35
Clp1	Q99LI9	Polyribonucleotide 5'-hydroxyl-kinase Clp1	0.95	0.32
Ttl	P38585	Tubulin--tyrosine ligase	0.95	0.13
Cdkn1c	P49919	Cyclin-dependent kinase inhibitor 1C	0.95	0.47
Smug1	Q6P5C5	Single-strand selective monofunctional uracil DNA glycosylase	0.95	0.51
Rpusd2	Q149F1	RNA pseudouridylate synthase domain-containing protein 2	0.95	0.42
Wdr53	Q9DB94	WD repeat-containing protein 53	0.95	0.52
Smurf2	A2A5Z6	E3 ubiquitin-protein ligase SMURF2	0.95	0.68
Rnf34	Q99KR6	E3 ubiquitin-protein ligase RNF34	0.95	0.16
	Q9CWB7	Glutaredoxin-like protein C5orf63 homolog	0.95	0.28
G6pc3	Q6NSQ9	Glucose-6-phosphatase 3	0.95	0.52
Eri1	A0A0R4J0C8	3'-5' exoribonuclease 1	0.95	0.35
Uqcc3	Q8K2T4	Ubiquinol-cytochrome-c reductase complex assembly factor 3	0.95	0.67
Mysm1	Q69Z66	Histone H2A deubiquitinase MYSM1	0.95	0.14
Alyref2	G3X9I4	Aly/REF export factor 2	0.95	0.54
Eif4enif1	Q9EST3	Eukaryotic translation initiation factor 4E transporter	0.95	0.41
Fth1	P09528	Ferritin heavy chain	0.95	0.56
Rpp21	Q8R040	Ribonuclease P protein subunit p21	0.95	0.40
Vsir	Q9D659	V-type immunoglobulin domain-containing suppressor of T-cell activation	0.95	0.00

Slfn1	Q9Z0I7	Schlafen 1	0.95	0.40
Heca	Q3V1N5	Hdc homolog, cell cycle regulator	0.95	0.30
Tra2b	P62996	Transformer-2 protein homolog beta	0.95	0.42
C4b	P01029	Complement C4-B	0.95	0.61
Mtrr	A0A0R4J0G9	Methionine synthase reductase	0.95	0.33
Cldnd1	Q9CQX5	Claudin domain-containing protein 1	0.95	0.34
Rapgef5	Q8C0Q9	Rap guanine nucleotide exchange factor 5	0.95	0.26
Msra	Q9D6Y7	Mitochondrial peptide methionine sulfoxide reductase	0.95	0.24
Tmem219	G3X9Z3	Insulin-like growth factor-binding protein 3 receptor	0.95	0.14
Endog	O08600	Endonuclease G, mitochondrial	0.95	0.56
Nfatc2ip	O09130	NFATC2-interacting protein	0.95	0.34
Arhgap15	Q811M1	Rho GTPase-activating protein 15	0.95	0.40
Rusc2	Q3V1Z0	Iporin	0.95	0.62
Zfp36l2	P23949	mRNA decay activator protein ZFP36L2	0.95	0.47
Arhgap24	G3X9N1	Rho GTPase activating protein 24, isoform CRA_b	0.95	0.68
Ero1b	Q8R2E9	ERO1-like protein beta	0.95	0.09
Polr3b	P59470	DNA-directed RNA polymerase III subunit RPC2	0.95	0.44
Pias1	O88907	E3 SUMO-protein ligase PIAS1	0.95	0.38
Calhm2	Q8VEC4	Calcium homeostasis modulator protein 2	0.95	0.65
Pask	Q8CEE6	PAS domain-containing serine/threonine-protein kinase	0.95	0.52
Map3k3	Q61084	Mitogen-activated protein kinase kinase kinase 3	0.95	0.31
Usp38	Q8BW70	Ubiquitin carboxyl-terminal hydrolase 38	0.95	0.28
Cuta	Q9CQ89	Protein CutA	0.95	0.25
Lims2	Q91XD2	LIM and senescent cell antigen-like-containing domain protein 2	0.95	0.25
Dapp1	Q9QXT1	Dual adapter for phosphotyrosine and 3-phosphotyrosine and 3-phosphoinositide	0.95	0.64
Tia1	P52912	Nucleolysin TIA-1	0.95	0.09
Yju2	Q9D6J3	YJU2 splicing factor homolog	0.95	0.45
Stx16	Q8BVI5	Syntaxin-16	0.95	0.29
Ctsk	P55097	Cathepsin K	0.95	0.47
Cnot6	Q8K3P5	CCR4-NOT transcription complex subunit 6	0.95	0.50
Man2a2	Q8BRK9	Alpha-mannosidase 2x	0.95	0.49
Frrs1	A0A0G2JFP4	Ferric-chelate reductase 1	0.95	0.09
Slc36a1	Q8K4D3	Proton-coupled amino acid transporter 1	0.95	0.27
Cited2	O35740	Cbp/p300-interacting transactivator 2	0.96	0.19
Arhgap39	G3X932	DNA segment, Chr 15, Wayne State University 169, expressed, isoform CRA_a	0.96	0.30
Vps54	Q5SPW0	Vacuolar protein sorting-associated protein 54	0.96	0.53
Samhd1	F6TVP2	Deoxynucleoside triphosphate triphosphohydrolase SAMHD1 (Fragment)	0.96	0.69
Ptma	P26350	Prothymosin alpha	0.96	0.31
Phactr2	F7D4H5	Phosphatase and actin regulator	0.96	0.51
Gls	F6RDM4	Glutaminase kidney isoform, mitochondrial (Fragment)	0.96	0.48
Timmdc1	Q8BUY5	Complex I assembly factor TIMMDC1, mitochondrial	0.96	0.57
Yrdc	Q3U5F4	YrdC domain-containing protein, mitochondrial	0.96	0.36
Gpn3	Q9D3W4	GPN-loop GTPase 3	0.96	0.72
Iba57	Q8CAK1	Putative transferase CAF17 homolog, mitochondrial	0.96	0.23
Asb6	Q91ZU1	Ankyrin repeat and SOCS box protein 6	0.96	0.09
Fzr1	Q9R1K5	Fizzy-related protein homolog	0.96	0.71
Hdac10	Q6P3E7	Histone deacetylase 10	0.96	0.35
Abcg2	A0A0R4J0B6	ATP-binding cassette sub-family G member 2	0.96	0.38
Gan	F6TZU3	Gigaxonin (Fragment)	0.96	0.49
Ear10	Q923L6	Eosinophil-associated ribonuclease 10	0.96	0.37
Nr1h2	Q60644	Oxysterols receptor LXR-beta	0.96	0.23
Wdsub1	Q9D0I6	WD repeat, SAM and U-box domain-containing protein 1	0.96	0.35
Ndor1	A0A2I3BQN1	NADPH-dependent diflavin oxidoreductase 1	0.96	0.60
Helz	A0A0R4J0Y3	Probable helicase with zinc finger domain	0.96	0.52
Ifrd1	P19182	Interferon-related developmental regulator 1	0.96	0.39
H2-K1	P04223	H-2 class I histocompatibility antigen, K-K alpha chain	0.96	0.52
Dhrs9	Q58NB6	Dehydrogenase/reductase SDR family member 9	0.96	0.39
Neil2	Q6R2P8	Endonuclease 8-like 2	0.96	0.56
Srxn1	A2AQU8	Sulfiredoxin	0.96	0.23
Nxt1	Q9QZV9	NTF2-related export protein 1	0.96	0.30
Efhd2	Q8C845	EF-hand domain-containing protein D2	0.96	0.30
Fastkd1	Q6DI86	FAST kinase domain-containing protein 1, mitochondrial	0.96	0.67
Mkrn2	Q9ERV1	Probable E3 ubiquitin-protein ligase makorin-2	0.96	0.44
Cbx3	Q9DCC5	Cbx3 protein	0.96	0.23
Med19	Q8C1S0	Mediator of RNA polymerase II transcription subunit 19	0.96	0.45
Edem2	Q8BJT9	ER degradation-enhancing alpha-mannosidase-like protein 2	0.96	0.69
Dgkz	D6RHK9	Diacylglycerol kinase zeta	0.96	0.45
Dazap2	Q9DCP9	DAZ-associated protein 2	0.96	0.46
Gpsm3	Q3U1Z5	G-protein-signaling modulator 3	0.96	0.50
Znf516	Q7TSH3	Zinc finger protein 516	0.96	0.38
Snrpe	P62305	Small nuclear ribonucleoprotein E	0.96	0.39
2210016L21Rik	A0A0R4J099	RIKEN cDNA 2210016L21 gene	0.96	0.54
Pdss1	Q33DR2	Decaprenyl-diphosphate synthase subunit 1	0.96	0.72

Mprip	Q5SWZ5	Myosin phosphatase Rho-interacting protein	0.96	0.52
Jund	P15066	Transcription factor jun-D	0.96	0.48
S100a4	A0A0G2JGD2	Protein S100-A4 (Fragment)	0.96	0.57
Orai1	Q8BWG9	Calcium release-activated calcium channel protein 1	0.96	0.63
Mark3	A0A1Y7VNZ6	Non-specific serine/threonine protein kinase	0.96	0.22
Aar2	Q9D2V5	Protein AAR2 homolog	0.96	0.71
Lrif1	Q8CDD9	Ligand-dependent nuclear receptor-interacting factor 1	0.96	0.74
Tti2	Q8BGV4	TELO2-interacting protein 2	0.96	0.41
Nutf2	P61971	Nuclear transport factor 2	0.96	0.13
Arpc4	P59999	Actin-related protein 2/3 complex subunit 4	0.96	0.35
Mcee	Q9D1I5	Methylmalonyl-CoA epimerase, mitochondrial	0.96	0.48
Ankrd27	Q3UMR0	Ankyrin repeat domain-containing protein 27	0.96	0.46
Lyz2	P08905	Lysozyme C-2	0.96	0.42
Cd200r5	Q8BTP3	Cell surface glycoprotein CD200 receptor 5	0.96	0.35
Ndufv3	Q8BK30	NADH dehydrogenase [ubiquinone] flavoprotein 3, mitochondrial	0.96	0.13
Pole3	A0A0R4J091	DNA polymerase epsilon subunit 3	0.96	0.29
Naca	P70670	Nascent polypeptide-associated complex subunit alpha, muscle-specific form	0.96	0.36
Atxn1	J3QPR1	Ataxin-1	0.96	0.12
Nupl2	E9QL43	Nucleoporin-like protein 2	0.96	0.55
Dtd1	Q9DD18	D-aminoacyl-tRNA deacylase 1	0.96	0.59
Map2k5	Q9WVS7	Dual specificity mitogen-activated protein kinase kinase 5	0.96	0.35
Alkbh7	Q9D6Z0	Alpha-ketoglutarate-dependent dioxygenase alkB homolog 7, mitochondrial	0.96	0.45
Mad2l1bp	Q9DCX1	MAD2L1-binding protein	0.96	0.62
0610012G03Rik	A0A0J9YTR2	RIKEN cDNA 0610012G03 gene	0.96	0.36
Wrb	Q8K0D7	Tail-anchored protein insertion receptor WRB	0.96	0.27
Rnf216	P58283	E3 ubiquitin-protein ligase RNF216	0.96	0.67
Krtcap2	Q5RL79	Keratinocyte-associated protein 2	0.96	0.62
Swi5	A0A0A0MQ99	DNA repair protein SWI5 homolog	0.96	0.56
Susd3	Q9D176	Sushi domain-containing protein 3	0.96	0.44
Rmdn2	Q8BSE0	Regulator of microtubule dynamics protein 2	0.96	0.30
Mapkap1	Q8BKH7	Target of rapamycin complex 2 subunit MAPKAP1	0.96	0.49
Gm49391	A0A286YD17	Predicted gene, 49391	0.96	0.18
Tprkb	Q8QZZ7	EKC/KEOPS complex subunit Tprkb	0.96	0.46
Bbc3	Q99ML1	Bcl-2-binding component 3	0.96	0.42
Cdkal1	Q91WE6	Threonylcarbamoyladenosine tRNA methylthiotransferase	0.96	0.17
Gsta3	P30115	Glutathione S-transferase A3	0.96	0.61
Ogt	Q8CGY8	UDP-N-acetylglucosamine--peptide N-acetylglucosaminyltransferase 110 kDa subunit	0.96	0.37
Tsen2	Q6P7W5	tRNA-splicing endonuclease subunit Sen2	0.96	0.61
Ifi30	Q9ESY9	Gamma-interferon-inducible lysosomal thiol reductase	0.96	0.40
Sptan1	A3KGU5	Spectrin alpha chain, non-erythrocytic 1	0.96	0.27
Fpgs	P48760	Folylpolyglutamate synthase, mitochondrial	0.96	0.43
Psmb4	P99026	Proteasome subunit beta type-4	0.96	0.42
Rps16	P14131	40S ribosomal protein S16	0.96	0.49
Ccdc61	Q3UJV1	Coiled-coil domain-containing protein 61	0.96	0.56
Myo10	F8VQB6	Unconventional myosin-X	0.96	0.69
Ccnb2	P30276	G2/mitotic-specific cyclin-B2	0.96	0.24
Fam206a	Q80ZQ9	Protein Simiate	0.96	0.16
Lsm12	Q9D0R8	Protein LSM12 homolog	0.96	0.27
Fbxl15	Q91W61	F-box/LRR-repeat protein 15	0.96	0.44
Abracl	E9QMV2	Costars family protein ABRACL	0.96	0.25
N4bp2	F8VQG7	NEDD4-binding protein 2	0.96	0.57
Fam222b	Q6P539	Protein FAM222B	0.96	0.50
Tdp1	B8JJC1	Tyrosyl-DNA phosphodiesterase 1	0.96	0.54
Ints5	Q8CHT3	Integrator complex subunit 5	0.96	0.29
Tmsb10	Q6ZWY8	Thymosin beta-10	0.96	0.42
Trpc4ap	Q9JLV2	Short transient receptor potential channel 4-associated protein	0.96	0.39
Llgl1	A0A0R4J0S4	Lethal(2) giant larvae protein homolog 1	0.96	0.50
Snrpd1	P62315	Small nuclear ribonucleoprotein Sm D1	0.96	0.77
Tmem186	Q9CR76	Transmembrane protein 186	0.96	0.31
Mlf2	Q99KX1	Myeloid leukemia factor 2	0.96	0.36
Kyat1	Q8BTY1	Kynurenine--oxoglutarate transaminase 1	0.96	0.57
Rbm3	Q8BG13	RNA-binding protein 3	0.96	0.70
Hmox1	P14901	Heme oxygenase 1	0.96	0.04
Cux1	H3BJQ9	Homeobox protein cut-like	0.96	0.45
Thg1l	Q9CY52	Probable tRNA(His) guanylyltransferase	0.96	0.43
Klhl18	A0A0G2JGN5	Kelch-like 18	0.96	0.60
Tubal3	Q3UX10	Tubulin alpha chain-like 3	0.96	0.30
Cd274	Q9EP73	Programmed cell death 1 ligand 1	0.96	0.58
Eps8l1	E9Q4X5	Epidermal growth factor receptor kinase substrate 8-like protein 1	0.96	0.73
Trappc13	J3QNW4	Trafficking protein particle complex subunit 13	0.96	0.31
Dstyk	Q6XUX1	Dual serine/threonine and tyrosine protein kinase	0.96	0.33
S100a6	P14069	Protein S100-A6	0.96	0.35
Wdr34	A2BE91	WD repeat domain 34	0.96	0.59

Uxt	Q9WTZ0	Protein UXT	0.96	0.49
Ctdspl2	Q8BG15	CTD small phosphatase-like protein 2	0.96	0.51
Mras	O08989	Ras-related protein M-Ras	0.96	0.63
Ctsc	P97821	Dipeptidyl peptidase 1	0.96	0.39
Slc2a6	Q3UDF0	Solute carrier family 2 (Facilitated glucose transporter), member 6, isoform CRA_a	0.97	0.22
Khnyn	Q80U38	Protein KHNYN	0.97	0.68
Rad54l2	E9QKL0	Helicase ARIP4	0.97	0.58
Phf10	K4DI61	PHD finger protein 10	0.97	0.49
Irak1	B1AUW6	Interleukin-1 receptor-associated kinase 1	0.97	0.55
Ercc6	F8VPZ5	Excision repair cross-complementing rodent repair deficiency, complementation group 6	0.97	0.56
Gle1	Q8R322	Nucleoporin GLE1	0.97	0.59
Atm	Q62388	Serine-protein kinase ATM	0.97	0.56
Yeats2	Q3TUF7	YEATS domain-containing protein 2	0.97	0.54
Kif23	E9Q5G3	Kinesin-like protein KIF23	0.97	0.57
Il6st	Q00560	Interleukin-6 receptor subunit beta	0.97	0.50
Glmp	Q9JHJ3	Glycosylated lysosomal membrane protein	0.97	0.50
Papss2	O88428	Bifunctional 3'-phosphoadenosine 5'-phosphosulfate synthase 2	0.97	0.53
Yipf6	Q8BR70	Protein YIPF6	0.97	0.34
Cox6a1	Q9DCW5	Cytochrome c oxidase subunit 6A, mitochondrial	0.97	0.48
Taf5	F8VPY2	Transcription initiation factor TFIID subunit 5	0.97	0.61
Rnf41	Q8BH75	E3 ubiquitin-protein ligase NRDP1	0.97	0.70
Tha1	Q6XPS7	L-threonine aldolase	0.97	0.64
Ighmbp2	A0A0B4J1E3	DNA-binding protein SMUBP-2	0.97	0.63
Tyrobp	A0A140LHP7	TYRO protein tyrosine kinase-binding protein (Fragment)	0.97	0.72
Wsb2	E9Q8J9	WD repeat and SOCS box-containing protein 2	0.97	0.56
Ncdn	Q9Z0E0	Neurochondrin	0.97	0.43
Trps1	V9GX74	Zinc finger transcription factor Trps1	0.97	0.38
Tbc1d9	Q3UYK3	TBC1 domain family member 9	0.97	0.52
Klhl26	Q8BGY4	Kelch-like protein 26	0.97	0.63
Szrd1	Q6NXN1	SUZ domain-containing protein 1	0.97	0.61
Txndc17	Q9CQM5	Thioredoxin domain-containing protein 17	0.97	0.53
Notch1	Q01705	Neurogenic locus notch homolog protein 1	0.97	0.24
Dvl1	P51141	Segment polarity protein dishevelled homolog DVL-1	0.97	0.65
Hmgn1	P18608	Non-histone chromosomal protein HMG-14	0.97	0.60
Vps37a	Q8CHS8	Vacuolar protein sorting-associated protein 37A	0.97	0.35
B3galt6	Q91Z92	Beta-1,3-galactosyltransferase 6	0.97	0.32
Nfkbib	Q60778	NF-kappa-B inhibitor beta	0.97	0.11
Cux1	H3BJL7	Protein CASP (Fragment)	0.97	0.77
Ube2a	Q9Z255	Ubiquitin-conjugating enzyme E2 A	0.97	0.49
Tmem230	Q8CIB6	Transmembrane protein 230	0.97	0.47
Fam168a	A0A140LIJ1	Protein FAM168A	0.97	0.62
Cct6b	B1AT05	T-complex protein 1 subunit zeta-2	0.97	0.36
Sec14l1	A8Y5H7	SEC14-like protein 1	0.97	0.21
Lmbrd1	Q8K0B2	Probable lysosomal cobalamin transporter	0.97	0.53
Dhtkd1	A2ATU0	Probable 2-oxoglutarate dehydrogenase E1 component DHKTD1, mitochondrial	0.97	0.68
Gm49339	A0A1W2P7X1	Predicted gene, 49339	0.97	0.62
Zcrb1	Q9CZ96	Zinc finger CCHC-type and RNA-binding motif-containing protein 1	0.97	0.62
Ptpn22	P29352	Tyrosine-protein phosphatase non-receptor type 22	0.97	0.66
Kdm1b	Q8CIG3	Lysine-specific histone demethylase 1B	0.97	0.57
Rcsd1	Q3UZA1	CapZ-interacting protein	0.97	0.45
Gusb	P12265	Beta-glucuronidase	0.97	0.45
Eif4ebp2	P70445	Eukaryotic translation initiation factor 4E-binding protein 2	0.97	0.51
Uqcr11	Q9CPX8	Cytochrome b-c1 complex subunit 10	0.97	0.74
Ercc2	O08811	General transcription and DNA repair factor IIH helicase subunit XPD	0.97	0.58
Atp13a3	Q5XF89	Probable cation-transporting ATPase 13A3	0.97	0.53
Eral1	Q9CZU4	GTPase Era, mitochondrial	0.97	0.45
Fastkd5	A0A0R4J291	FAST kinase domain-containing protein 5, mitochondrial	0.97	0.57
Dph7	Q9CYU6	Diphthine methyltransferase	0.97	0.63
Dctpp1	Q9QY93	dCTP pyrophosphatase 1	0.97	0.56
Commd6	B7ZNP3	COMM domain-containing protein 6	0.97	0.40
Pcbp1	P60335	Poly(rC)-binding protein 1	0.97	0.23
Med28	Q920D3	Mediator of RNA polymerase II transcription subunit 28	0.97	0.35
Itprid2	Q922B9	Protein ITPRID2	0.97	0.67
Papolg	Q6PCL9	Poly(A) polymerase gamma	0.97	0.36
Tk1	P04184	Thymidine kinase, cytosolic	0.97	0.59
Hira	Q61666	Protein HIRA	0.97	0.66
Lcmt2	Q8BYR1	tRNA wybutosine-synthesizing protein 4	0.97	0.67
Srsf10	Q9R0U0	Serine/arginine-rich splicing factor 10	0.97	0.47
Nav1	Q8CH77	Neuron navigator 1	0.97	0.69
Tmem237	Q3V0J1	Transmembrane protein 237	0.97	0.48
Cnot4	A0A0R4J1K1	CCR4-NOT transcription complex subunit 4	0.97	0.35
Trit1	Q80UN9	tRNA dimethylallyltransferase	0.97	0.34
Ntmt1	Q8R2U4	N-terminal Xaa-Pro-Lys N-methyltransferase 1	0.97	0.20

Ncaph2	Q8BSP2	Condensin-2 complex subunit H2	0.97	0.64
Zbtb7a	O88939	Zinc finger and BTB domain-containing protein 7A	0.97	0.16
BC037034	E9Q2V5	cDNA sequence BC037034	0.97	0.62
Bop1	P97452	Ribosome biogenesis protein BOP1	0.97	0.38
Gatad1	Q920S3	GATA zinc finger domain-containing protein 1	0.97	0.75
Gid4	Q9CPY6	Glucose-induced degradation protein 4 homolog	0.97	0.79
Phactr4	Q501J7	Phosphatase and actin regulator 4	0.97	0.25
Tuba1c	P68373	Tubulin alpha-1C chain	0.97	0.23
Myo9b	A0A1D5RLD1	Unconventional myosin-IXb	0.97	0.20
Tti1	Q91V83	TELO2-interacting protein 1 homolog	0.97	0.66
Rbm7	Q9CQT2	RNA-binding protein 7	0.97	0.17
Zgpat	Q8VDM1	Zinc finger CCCH-type with G patch domain-containing protein	0.97	0.33
Plscr4	P58196	Phospholipid scramblase 4	0.97	0.63
Taf9	Q8VI33	Transcription initiation factor TFIID subunit 9	0.97	0.65
Trappc10	F8VQF9	Trafficking protein particle complex subunit 10	0.97	0.69
Asb3	A0A0N4SV07	Ankyrin repeat and SOCS box protein 3	0.97	0.72
Cdc42bpg	Q80UW5	Serine/threonine-protein kinase MRCK gamma	0.97	0.64
Dab2	A0A0R4J104	Disabled homolog 2	0.97	0.42
Psen1	P49769	Presenilin-1	0.97	0.43
Gnl1	P36916	Guanine nucleotide-binding protein-like 1	0.97	0.56
Erh	G3UW85	Enhancer of rudimentary homolog	0.97	0.74
Oxr1	Q4KMM3	Oxidation resistance protein 1	0.97	0.40
G6pdx	A3KG36	Glucose-6-phosphate 1-dehydrogenase (Fragment)	0.97	0.54
Unc119	Q9Z2R6	Protein unc-119 homolog A	0.97	0.42
Cep152	A2AUM9	Centrosomal protein of 152 kDa	0.97	0.72
Eif2b2	Q99LD9	Translation initiation factor eIF-2B subunit beta	0.97	0.22
Osbpl7	A2A716	Oxysterol-binding protein	0.97	0.53
Tor1aip1	E9PWW2	Torsin-1A-interacting protein 1	0.97	0.42
Dpy30	Q99LT0	Protein dpy-30 homolog	0.97	0.26
Ran	P62827	GTP-binding nuclear protein Ran	0.97	0.45
Cops5	O35864	COP9 signalosome complex subunit 5	0.97	0.12
Kti12	Q9D1R2	Protein KTI12 homolog	0.97	0.61
Anxa5	A0A0G2JGQ0	Annexin A5 (Fragment)	0.97	0.52
Ssh2	A0A0R4J2A0	Protein phosphatase Slingshot homolog 2	0.97	0.68
Ccnl2	Q9JJA7	Cyclin-L2	0.97	0.66
Fam160b2	Q80YR2	Protein FAM160B2	0.97	0.75
Wdr82	Q8BFQ4	WD repeat-containing protein 82	0.97	0.36
Psmb3	Q9R1P1	Proteasome subunit beta type-3	0.97	0.38
Papd5	E9QJT6	PAP-associated domain-containing 5	0.97	0.40
Ppih	A2BGI8	Peptidyl-prolyl cis-trans isomerase (Fragment)	0.97	0.12
Gba2	Q69ZF3	Non-lysosomal glucosylceramidase	0.97	0.61
Hdac4	Q6NZM9	Histone deacetylase 4	0.97	0.57
Arpc1b	Q91Z25	Actin-related protein 2/3 complex subunit	0.97	0.44
Tjap1	I7H459	Protein incorporated later into tight junctions	0.97	0.40
Csnk2b	P67871	Casein kinase II subunit beta	0.97	0.51
Ube2q2	Q8K2Z8	Ubiquitin-conjugating enzyme E2 Q2	0.97	0.52
Srgap1	D3YZW1	SLIT-ROBO Rho GTPase-activating protein 1	0.97	0.60
Alg13	E9Q161	Putative bifunctional UDP-N-acetylglucosamine transferase and deubiquitinase ALG13	0.97	0.56
Dock4	P59764	Dedicator of cytokinesis protein 4	0.97	0.53
Retsat	Q64FW2	All-trans-retinol 13,14-reductase	0.97	0.43
Tbc1d14	G3UVU5	TBC1 domain family member 14	0.97	0.56
Tpm3-rs7	D3Z2H9	Tropomyosin 3, related sequence 7	0.97	0.21
Minpp1	Q9Z2L6	Multiple inositol polyphosphate phosphatase 1	0.97	0.25
Med24	Q99K74	Mediator of RNA polymerase II transcription subunit 24	0.97	0.39
Baz1a	G3UWZ0	Bromodomain adjacent to zinc finger domain protein 1A	0.97	0.84
Mctp1	E9PV86	Multiple C2 and transmembrane domain-containing protein 1	0.97	0.75
Arhgdib	Q61599	Rho GDP-dissociation inhibitor 2	0.97	0.42
Dnmt1	J3QNW0	DNA (cytosine-5)-methyltransferase	0.97	0.40
Nup43	P59235	Nucleoporin Nup43	0.97	0.62
Nbr1	K3W4P1	Next to BRCA1 gene 1 protein	0.97	0.70
Rps6kb2	Q9Z1M4	Ribosomal protein S6 kinase beta-2	0.97	0.43
Psmg1	Q9JK23	Proteasome assembly chaperone 1	0.97	0.35
Tbkbp1	A2A9T0	TANK-binding kinase 1-binding protein 1	0.97	0.74
Ttc7b	A0A1Y7VL44	Tetratricopeptide repeat protein 7B	0.97	0.44
Tsen54	Q8C2A2	tRNA-splicing endonuclease subunit Sen54	0.97	0.77
Il7r	P16872	Interleukin-7 receptor subunit alpha	0.97	0.51
Znf706	Q9D115	Zinc finger protein 706	0.97	0.63
Bcl10	Q9Z0H7	B-cell lymphoma/leukemia 10	0.97	0.11
Slco2b1	Q3V1K7	Solute carrier organic anion transporter family member	0.97	0.25
Hnrnpll	Q921F4	Heterogeneous nuclear ribonucleoprotein L-like	0.97	0.32
Gns	Q8BFR4	N-acetylglucosamine-6-sulfatase	0.97	0.39
Cdt1	Q8R4E9	DNA replication factor Cdt1	0.97	0.50
Glrx	Q9QUH0	Glutaredoxin-1	0.97	0.50

Cnbp	A0A0N4SVS6	Cellular nucleic acid-binding protein	0.97	0.53
Cln3	Q61124	Battenin	0.97	0.05
Tcp11l2	Q8K1H7	T-complex protein 11-like protein 2	0.97	0.78
Nudt5	Q9JKX6	ADP-sugar pyrophosphatase	0.97	0.50
Ap4b1	Q9WV76	AP-4 complex subunit beta-1	0.97	0.50
Sarnp	Q9D1J3	SAP domain-containing ribonucleoprotein	0.97	0.40
Txnip	Q8BG60	Thioredoxin-interacting protein	0.97	0.11
Ttn	A2ASS6	Titin	0.97	0.45
Fbxo28	Q8BIG4	F-box only protein 28	0.97	0.68
Svbp	A2A7P9	Coiled-coil domain containing 23, isoform CRA_c	0.97	0.38
Pde4a	O89084	cAMP-specific 3',5'-cyclic phosphodiesterase 4A	0.97	0.75
	Q9D9H8	Mitochondrial protein C2orf69 homolog	0.97	0.44
Tsen34	Q8BMZ5	tRNA-splicing endonuclease subunit Sen34	0.97	0.65
Prdx2	Q61171	Peroxiredoxin-2	0.97	0.48
Atf7ip	Q7TT18	Activating transcription factor 7-interacting protein 1	0.97	0.31
Palld	D3Z1J5	Palladin (Fragment)	0.97	0.68
Fcrls	Q9EQY5	Fc receptor-like S, scavenger receptor	0.97	0.58
Oxld1	Q9CR10	Oxidoreductase-like domain-containing protein 1	0.97	0.63
Fgfr1op	Q66JX5	FGFR1 oncogene partner	0.97	0.65
Gtpbp8	Q9CY28	GTP-binding protein 8	0.97	0.62
Slfn5	Q8CBA2	Schlafen family member 5	0.97	0.43
Otud3	B1AZ99	OTU domain-containing protein 3	0.97	0.78
Virma	E9PZY8	Protein virilizer homolog	0.97	0.70
Ascc1	Q9D8Z1	Activating signal cointegrator 1 complex subunit 1	0.97	0.56
Set	Q9EQU5	Protein SET	0.97	0.25
L2hgdh	Q91YP0	L-2-hydroxyglutarate dehydrogenase, mitochondrial	0.97	0.60
Camsap3	Q80VC9	Calmodulin-regulated spectrin-associated protein 3	0.97	0.60
Ccdc71l	E9Q4T4	Coiled-coil domain-containing 71-like	0.97	0.49
Diexf	Q8BTT6	Digestive organ expansion factor homolog	0.97	0.59
Uimc1	A0A0R4J073	BRCA1-A complex subunit RAP80	0.97	0.79
Mafb	P54841	Transcription factor MafB	0.97	0.38
Ids	Q08890	Iduronate 2-sulfatase	0.97	0.55
Gmnn	O88513	Geminin	0.97	0.89
	Q8CIL4	Uncharacterized protein C1orf131 homolog	0.97	0.83
Dpp8	Q80YA7	Dipeptidyl peptidase 8	0.97	0.52
Gclm	O09172	Glutamate--cysteine ligase regulatory subunit	0.97	0.57
Gng5	Q80SZ7	Guanine nucleotide-binding protein G(I)/G(S)/G(O) subunit gamma-5	0.97	0.66
Timm8b	P62077	Mitochondrial import inner membrane translocase subunit Tim8 B	0.97	0.30
Cdc73	Q8JZM7	Parafibromin	0.97	0.47
Cd2bp2	Q9CWK3	CD2 antigen cytoplasmic tail-binding protein 2	0.97	0.51
	Q8WUR0	Protein C19orf12 homolog	0.97	0.41
Traf6	P70196	TNF receptor-associated factor 6	0.97	0.57
Gmfb	Q9CQI3	Glia maturation factor beta	0.97	0.41
Tfcp2	A0A2R8VKI7	Alpha-globin transcription factor CP2	0.97	0.65
Jun	P05627	Transcription factor AP-1	0.97	0.39
Ppp2ca	P63330	Serine/threonine-protein phosphatase 2A catalytic subunit alpha isoform	0.97	0.40
Ifrd2	Q9D8U0	Interferon-related developmental regulator 2	0.97	0.80
Ppp2r2d	Q925E7	Serine/threonine-protein phosphatase 2A 55 kDa regulatory subunit B delta isoform	0.97	0.48
Elmo2	F6XD63	Engulfment and cell motility protein 2 (Fragment)	0.97	0.60
Snx18	Q8C788	Sorting nexin	0.97	0.38
Srsf9	Q9D0B0	Serine/arginine-rich splicing factor 9	0.97	0.26
Myo1b	Q7TQD7	Myo1b protein	0.97	0.69
Fmc1	Q9CR13	Protein FMC1 homolog	0.97	0.69
Rwdd4	Q9CPR1	RWD domain-containing protein 4	0.97	0.39
Tbl3	Q8C4J7	Transducin beta-like protein 3	0.97	0.64
Dhx37	Q6NZL1	DEAH (Asp-Glu-Ala-His) box polypeptide 37	0.97	0.59
Ifnar2	O35664	Interferon alpha/beta receptor 2	0.97	0.68
Pole	Q9WVF7	DNA polymerase epsilon catalytic subunit A	0.97	0.68
Fbxl8	Q8CIG9	F-box/LRR-repeat protein 8	0.97	0.77
Gda	Q9R111	Guanine deaminase	0.97	0.61
Cmpk2	Q3U5Q7	UMP-CMP kinase 2, mitochondrial	0.97	0.42
Akirin2	B1AXD8	Akirin-2	0.97	0.45
Tyw1	Q8BJM7	S-adenosyl-L-methionine-dependent tRNA 4-demethylwyosine synthase	0.97	0.65
Sec22a	Q8BH47	Vesicle-trafficking protein SEC22a	0.97	0.57
Khdrbs1	Q60749	KH domain-containing, RNA-binding, signal transduction-associated protein 1	0.97	0.52
Anapc7	Q9WVM3	Anaphase-promoting complex subunit 7	0.97	0.55
COA4	Q8BT51	Cytochrome c oxidase assembly factor 4 homolog, mitochondrial	0.97	0.43
Hebp1	Q9R257	Heme-binding protein 1	0.98	0.36
Tgm2	P21981	Protein-glutamine gamma-glutamyltransferase 2	0.98	0.49
Kdm5a	Q3UXZ9	Lysine-specific demethylase 5A	0.98	0.48
Xylt1	F8VPK6	Xylosyltransferase 1	0.98	0.35
Trip13	Q3UA06	Pachytene checkpoint protein 2 homolog	0.98	0.46
Ehmt1	Q5DW34	Histone-lysine N-methyltransferase EHMT1	0.98	0.39

Nrde2	Q80XC6	Protein NRDE2 homolog	0.98	0.71
Ptk7	Q8BKG3	Inactive tyrosine-protein kinase 7	0.98	0.68
Arhgap21	B7ZCJ1	Rho GTPase-activating protein 21	0.98	0.59
Cyth2	P63034	Cytohesin-2	0.98	0.85
Eif4e	P63073	Eukaryotic translation initiation factor 4E	0.98	0.38
Gstm5	P48774	Glutathione S-transferase Mu 5	0.98	0.64
Nap1l4	B7ZNL2	Nap1l4 protein	0.98	0.65
2700097O09Rik	Q6PGK3	RIKEN cDNA 2700097O09	0.98	0.52
Pef1	Q8BFY6	Peflin	0.98	0.37
Numbl	O08919	Numb-like protein	0.98	0.66
Myl6	A0A1W2P6G5	Myosin light polypeptide 6	0.98	0.57
Eef1akmt4-Ece2	P0DPD9	EEF1AKMT4-ECE2 readthrough transcript protein	0.98	0.39
Cope	O89079	Coatomer subunit epsilon	0.98	0.61
Klhl1	Q9JI74	Kelch-like protein 1	0.98	0.58
Smarcc1	P97496	SWI/SNF complex subunit SMARCC1	0.98	0.55
Ubr7	Q8BU04	Putative E3 ubiquitin-protein ligase UBR7	0.98	0.48
Tomm6	Q9CQN3	Mitochondrial import receptor subunit TOM6 homolog	0.98	0.77
H2-Q7	P14429	H-2 class I histocompatibility antigen, Q7 alpha chain	0.98	0.62
Fbxw11	Q5SRY7	F-box/WD repeat-containing protein 11	0.98	0.38
Tuba4a	A0A0A0MQA5	Tubulin alpha chain (Fragment)	0.98	0.43
Lamp2	P17047	Lysosome-associated membrane glycoprotein 2	0.98	0.44
Hps3	Q91VB4	Hermansky-Pudlak syndrome 3 protein homolog	0.98	0.56
Kdm3a	Q6PCM1	Lysine-specific demethylase 3A	0.98	0.60
Katnal1	Q8K0T4	Katanin p60 ATPase-containing subunit A-like 1	0.98	0.56
Hspbap1	Q8BK58	HSPB1-associated protein 1	0.98	0.63
Klhdc3	A0A0R4J0W0	Kelch domain containing 3, isoform CRA_a	0.98	0.71
Xrcc4	A0A0R4J024	DNA repair protein XRCC4	0.98	0.54
Tubgcp5	Q8BKN5	Gamma-tubulin complex component 5	0.98	0.69
Kdm4a	Q8BW72	Lysine-specific demethylase 4A	0.98	0.64
Ccrl2	O35457	C-C chemokine receptor-like 2	0.98	0.68
Scaf8	Q6DID3	Protein SCAF8	0.98	0.66
Mettl1	Q9Z120	tRNA (guanine-N(7)-)-methyltransferase	0.98	0.61
Mrps17	Q9CQE3	28S ribosomal protein S17, mitochondrial	0.98	0.60
Wwc2	Q6NXJ0	Protein WWC2	0.98	0.83
Sod2	P09671	Superoxide dismutase [Mn], mitochondrial	0.98	0.36
Pdp2	Q504M2	MCG53395	0.98	0.39
Mkrn1	Q8C5V4	E3 ubiquitin-protein ligase makorin-1	0.98	0.57
Ankrd13d	Q6PD24	Ankyrin repeat domain-containing protein 13D	0.98	0.24
Asf1b	Q9DAP7	Histone chaperone ASF1B	0.98	0.79
Tmem68	Q9D850	Transmembrane protein 68	0.98	0.79
Aptx	Q7TQC5	Aprataxin	0.98	0.60
1700074P13Rik	Q9D9G7	1700074P13Rik protein	0.98	0.59
Noc4l	Q8BHY2	Nucleolar complex protein 4 homolog	0.98	0.68
Fbxo7	Q3U7U3	F-box only protein 7	0.98	0.55
Ddx55	Q6ZPL9	ATP-dependent RNA helicase DDX55	0.98	0.67
Rtn4	Q8BH78	Reticulon	0.98	0.25
Usp34	F6WJB7	Ubiquitin carboxyl-terminal hydrolase 34 (Fragment)	0.98	0.73
Mpnd	Q3TV65	MPN domain-containing protein	0.98	0.29
Orc4	O88708	Origin recognition complex subunit 4	0.98	0.60
Serac1	Q3U213	Protein SERAC1	0.98	0.54
Dsn1	Q9CYC5	Kinetochore-associated protein DSN1 homolog	0.98	0.77
Tbcb	Q9D1E6	Tubulin-folding cofactor B	0.98	0.56
Slc7a6os	Q7TPE5	Probable RNA polymerase II nuclear localization protein SLC7A6OS	0.98	0.62
Fermt2	Q8CIB5	Fermitin family homolog 2	0.98	0.85
Ifit3	Q64345	Interferon-induced protein with tetratricopeptide repeats 3	0.98	0.47
Rhoc	Q62159	Rho-related GTP-binding protein RhoC	0.98	0.67
Cit	D3YU89	Citron Rho-interacting kinase	0.98	0.82
Atxn2l	Q3TGG2	Ataxin-2-like protein	0.98	0.54
Gcat	O88986	2-amino-3-ketobutyrate coenzyme A ligase, mitochondrial	0.98	0.59
Sidt2	Q8CIF6	SID1 transmembrane family member 2	0.98	0.78
Tatdn2	B7ZNL9	TatD DNase domain-containing 2	0.98	0.73
Sesn1	E9PXR3	Sestrin-1	0.98	0.72
Mettl6	Q8BVH9	Methyltransferase-like protein 6	0.98	0.45
Dnajc25	A2ALW5	DnaJ homolog subfamily C member 25	0.98	0.84
Asrgl1	Q8C0M9	Isoaspartyl peptidase/L-asparaginase	0.98	0.54
Nfkbie	O54910	NF-kappa-B inhibitor epsilon	0.98	0.25
Cdk20	Q9JHU3	Cyclin-dependent kinase 20	0.98	0.87
Rps27	Q6ZWU9	40S ribosomal protein S27	0.98	0.73
Abhd14b	E9QN99	Protein ABHD14B	0.98	0.58
Setx	A2AKX3	Probable helicase senataxin	0.98	0.65
Ezh2	Q61188	Histone-lysine N-methyltransferase EZH2	0.98	0.75
Tubb4b	P68372	Tubulin beta-4B chain	0.98	0.57
Tagln2	Q9WVA4	Transgelin-2	0.98	0.37

Skp1	Q9WTX5	S-phase kinase-associated protein 1	0.98	0.52
Sdhd	Q9CXV1	Succinate dehydrogenase [ubiquinone] cytochrome b small subunit, mitochondrial	0.98	0.64
Mcat	Q8R3F5	Malonyl-CoA-acyl carrier protein transacylase, mitochondrial	0.98	0.24
Nhej1	Q3KNJ2	Non-homologous end-joining factor 1	0.98	0.64
Galm	Q8K157	Aldose 1-epimerase	0.98	0.33
	Q3TEI4	Uncharacterized protein C15orf39 homolog	0.98	0.84
Slc29a1	Q9JIM1	Equilibrative nucleoside transporter 1	0.98	0.53
Tubg1	P83887	Tubulin gamma-1 chain	0.98	0.50
4931406P16Rik	Q8C5X1	RIKEN cDNA 4931406P16 gene	0.98	0.51
Mrpl55	Q9CZ83	39S ribosomal protein L55, mitochondrial	0.98	0.55
Cdc37	Q61081	Hsp90 co-chaperone Cdc37	0.98	0.56
Cotl1	Q9CQI6	Coactosin-like protein	0.98	0.38
Plcg1	Q62077	1-phosphatidylinositol 4,5-bisphosphate phosphodiesterase gamma-1	0.98	0.77
Aif1	O70200	Allograft inflammatory factor 1	0.98	0.56
Arhgdia	Q99PT1	Rho GDP-dissociation inhibitor 1	0.98	0.62
Chd2	E9PZM4	Chromodomain-helicase-DNA-binding protein 2	0.98	0.53
Rbpms	Q9WVB0	RNA-binding protein with multiple splicing	0.98	0.48
Ly86	O88188	Lymphocyte antigen 86	0.98	0.21
Fuom	F6SJM7	Fucose mutarotase (Fragment)	0.98	0.46
Pphln1	G3X959	Periphilin 1, isoform CRA_a	0.98	0.65
Hdhd2	Q3UGR5	Haloacid dehalogenase-like hydrolase domain-containing protein 2	0.98	0.40
Prmt3	Q922H1	Protein arginine N-methyltransferase 3	0.98	0.52
Ngdn	Q9DB96	Neuroguidin	0.98	0.72
Erich1	E9PY43	Glutamate-rich 1	0.98	0.52
Abl1	P00520	Tyrosine-protein kinase ABL1	0.98	0.55
Nsdhl	Q9R1J0	Sterol-4-alpha-carboxylate 3-dehydrogenase, decarboxylating	0.98	0.46
Idi1	G3XA48	Isopentenyl-diphosphate Delta-isomerase 1	0.98	0.31
Dynlt3	P56387	Dynein light chain Tctex-type 3	0.98	0.70
Ppp1r14b	Q62084	Protein phosphatase 1 regulatory subunit 14B	0.98	0.63
Nqo2	Q9JI75	Ribosyldihydronicotinamide dehydrogenase [quinone]	0.98	0.54
Actr2	P61161	Actin-related protein 2	0.98	0.41
Hnrnpab	Q20BD0	Heterogeneous nuclear ribonucleoprotein A/B	0.98	0.36
Cnot3	Q8K0V4	CCR4-NOT transcription complex subunit 3	0.98	0.70
Igbp1	Q61249	Immunoglobulin-binding protein 1	0.98	0.63
Tinagl1	Q99JR5	Tubulointerstitial nephritis antigen-like	0.98	0.75
7-Sep	O55131	Septin-7	0.98	0.46
Ndufa10	Q99LC3	NADH dehydrogenase [ubiquinone] 1 alpha subcomplex subunit 10, mitochondrial	0.98	0.65
Slc7a6	Q8BGK6	Y+L amino acid transporter 2	0.98	0.65
Pnpo	Q91XF0	Pyridoxine-5'-phosphate oxidase	0.98	0.87
Mia3	J3KMH5	Transport and Golgi organization protein 1 homolog	0.98	0.73
Serpinc1	P32261	Antithrombin-III	0.98	0.86
Brms1l	Q3U1T3	Breast cancer metastasis-suppressor 1-like protein	0.98	0.72
Mif	P34884	Macrophage migration inhibitory factor	0.98	0.63
Atg10	Q8R1P4	Ubiquitin-like-conjugating enzyme ATG10	0.98	0.71
Rap1gds1	E9Q6Q4	RAP1, GTP-GDP dissociation stimulator 1	0.98	0.65
Cox17	P56394	Cytochrome c oxidase copper chaperone	0.98	0.49
Unc119b	Q8C4B4	Protein unc-119 homolog B	0.98	0.53
Lztr1	Q9CQ33	Leucine-zipper-like transcriptional regulator 1	0.98	0.72
Nip7	Q9CXK8	60S ribosome subunit biogenesis protein NIP7 homolog	0.98	0.56
Ppp1cb	P62141	Serine/threonine-protein phosphatase PP1-beta catalytic subunit	0.98	0.24
Sub1	P11031	Activated RNA polymerase II transcriptional coactivator p15	0.98	0.28
Ctnnd1	E9Q8Z5	Catenin delta-1	0.98	0.73
Rnf2	Q9CQJ4	E3 ubiquitin-protein ligase RING2	0.98	0.63
Higd1a	Q8R472	HIG1 domain family member 1A, mitochondrial	0.98	0.84
Nxn	P97346	Nucleoredoxin	0.98	0.58
Cox5a	P12787	Cytochrome c oxidase subunit 5A, mitochondrial	0.98	0.45
Oat	P29758	Ornithine aminotransferase, mitochondrial	0.98	0.52
Mrpl4	Q9DCU6	39S ribosomal protein L4, mitochondrial	0.98	0.65
Tada2b	D3Z4Z0	Transcriptional adapter	0.98	0.72
Adi1	Q99JT9	1,2-dihydroxy-3-keto-5-methylthiopentene dioxygenase	0.98	0.49
Wdr76	A6PWY4	WD repeat-containing protein 76	0.98	0.53
Btbd9	Q8C726	BTB/POZ domain-containing protein 9	0.98	0.75
Hdgf	P51859	Hepatoma-derived growth factor	0.98	0.38
Gtf3c3	Q3TMP1	General transcription factor IIIC, polypeptide 3	0.98	0.42
Gemin4	Q6P6L6	Gem (Nuclear organelle) associated protein 4	0.98	0.62
Pld4	Q8BG07	Phospholipase D4	0.98	0.51
Hnrnpd	G3X9W0	Heterogeneous nuclear ribonucleoprotein D, isoform CRA_a	0.98	0.78
Mrpl38	Q8K2M0	39S ribosomal protein L38, mitochondrial	0.98	0.57
Rpsa	P14206	40S ribosomal protein SA	0.98	0.38
Prpf18	Q8BM39	Pre-mRNA-splicing factor 18	0.98	0.69
Mgrn1	Q9D074	E3 ubiquitin-protein ligase MGRN1	0.98	0.61
Gtf2a2	Q80ZM7	Transcription initiation factor IIA subunit 2	0.98	0.74
Ufm1	P61961	Ubiquitin-fold modifier 1	0.98	0.36

Gatm	Q9D964	Glycine amidinotransferase, mitochondrial	0.98	0.64
Gcsh	Q91WK5	Glycine cleavage system H protein, mitochondrial	0.98	0.48
Atp6v0d1	P51863	V-type proton ATPase subunit d 1	0.98	0.59
Fam234a	Q8C0Z1	Protein FAM234A	0.98	0.59
Trmt61a	Q80XC2	tRNA (adenine(58)-N(1))-methyltransferase catalytic subunit TRMT61A	0.98	0.77
Slc31a1	Q8K211	High affinity copper uptake protein 1	0.98	0.55
Mfhas1	Q3V1N1	Malignant fibrous histiocytoma-amplified sequence 1 homolog	0.98	0.82
Ssbp1	Q8R2K3	Single-stranded DNA binding protein 1	0.98	0.54
Irgq	Q8VIM9	Immunity-related GTPase family Q protein	0.98	0.63
Ganc	A2AQJ8	Neutral alpha-glucosidase C	0.98	0.61
Bag4	Q8CI61	BAG family molecular chaperone regulator 4	0.98	0.62
Plxna2	P70207	Plexin-A2	0.98	0.83
Yae1d1	Q9DAY6	MCG16556, isoform CRA_a	0.98	0.74
Dhx57	Q6P5D3	Putative ATP-dependent RNA helicase DHX57	0.98	0.74
Gca	Q8VC88	Grancalcin	0.98	0.67
Gpcpd1	Q8C0L9	Glycerophosphocholine phosphodiesterase GPCPD1	0.98	0.71
Fdx2	Q9CPW2	Ferredoxin-2, mitochondrial	0.98	0.68
Lmnb2	A0A0R4J0Q5	Lamin-B2	0.98	0.39
Psmg4	P0C7N9	Proteasome assembly chaperone 4	0.98	0.62
Rit1	P70426	GTP-binding protein Rit1	0.98	0.78
Med15	Q924H2	Mediator of RNA polymerase II transcription subunit 15	0.98	0.58
Psmg2	Q9EST4	Proteasome assembly chaperone 2	0.98	0.47
Nfx1	B1AY10	Transcriptional repressor NF-X1	0.98	0.72
Nudt2	P56380	Bis(5'-nucleosyl)-tetraphosphatase [asymmetrical]	0.98	0.49
Dnmbp	A0A0R4J055	Dynamin-binding protein	0.98	0.61
Mff	F6VAL0	Mitochondrial fission factor (Fragment)	0.98	0.61
Cpox	P36552	Oxygen-dependent coproporphyrinogen-III oxidase, mitochondrial	0.98	0.68
Cfap20	Q8BTU1	Cilia- and flagella-associated protein 20	0.98	0.57
Hnrnpa3	Q8BG05	Heterogeneous nuclear ribonucleoprotein A3	0.98	0.74
	Q9D7E4	UPF0449 protein C19orf25 homolog	0.98	0.59
Mmab	Q9D273	Cob(I)yrinic acid a,c-diamide adenosyltransferase, mitochondrial	0.98	0.67
U2af2	P26369	Splicing factor U2AF 65 kDa subunit	0.98	0.45
Haus4	Q8BFT2	HAUS augmin-like complex subunit 4	0.98	0.51
Ppp6c	Q9CQR6	Serine/threonine-protein phosphatase 6 catalytic subunit	0.98	0.46
Lsm7	Q9CQQ8	U6 snRNA-associated Sm-like protein LSm7	0.98	0.59
Exosc4	Q921I9	Exosome complex component RRP41	0.98	0.79
Ddx28	Q9CWT6	Probable ATP-dependent RNA helicase DDX28	0.98	0.59
Prodh	Q9WU79	Proline dehydrogenase 1, mitochondrial	0.98	0.60
Med31	Q9CXU1	Mediator of RNA polymerase II transcription subunit 31	0.98	0.57
Isyna1	Q9JHU9	Inositol-3-phosphate synthase 1	0.98	0.53
Msn	P26041	Moesin	0.98	0.48
Habp4	E9QKB2	Intracellular hyaluronan-binding protein 4	0.98	0.84
S100a11	P50543	Protein S100-A11	0.98	0.73
Tnip2	Q80VZ7	TNFAIP3-interacting protein 2	0.98	0.73
Polr2m	Q6P6I6	DNA-directed RNA polymerase II subunit GRINL1A	0.98	0.79
Pdcd6	P12815	Programmed cell death protein 6	0.98	0.44
Dus1l	B1ATU5	tRNA-dihydrouridine(16/17) synthase [NAD(P)(+)]-like	0.98	0.58
Ivns1abp	Q920Q8	Influenza virus NS1A-binding protein homolog	0.98	0.74
Csrp1	P97315	Cysteine and glycine-rich protein 1	0.98	0.68
Dph1	Q5NCQ5	2-(3-amino-3-carboxypropyl)histidine synthase subunit 1	0.98	0.70
Phf6	Q9D4J7	PHD finger protein 6	0.98	0.52
Marf1	Q8BJ34	Meiosis regulator and mRNA stability factor 1	0.98	0.69
Gmfg	Q9ERL7	Glia maturation factor gamma	0.98	0.54
Gabpb1	Q00420	GA-binding protein subunit beta-1	0.98	0.77
Stard9	Q80TF6	StAR-related lipid transfer protein 9	0.98	0.59
Myl6	Q60605	Myosin light polypeptide 6	0.98	0.42
Knop1	H7BX94	Lysine-rich nucleolar protein 1	0.98	0.55
Dhx15	O35286	Pre-mRNA-splicing factor ATP-dependent RNA helicase DHX15	0.98	0.34
Gdi2	Q61598	Rab GDP dissociation inhibitor beta	0.98	0.45
Wdr45b	Q9CR39	WD repeat domain phosphoinositide-interacting protein 3	0.98	0.54
Rnf13	O54965	E3 ubiquitin-protein ligase RNF13	0.98	0.56
Cdc27	A2A6Q5	Cell division cycle protein 27 homolog	0.98	0.27
P4ha1	E9Q7B0	Prolyl 4-hydroxylase subunit alpha-1	0.98	0.81
Eloc	A0A087WNT1	Elongin-C	0.98	0.88
Rab11fip5	A0A0N4SW73	Rab11 family-interacting protein 5	0.98	0.60
Trim28	Q62318	Transcription intermediary factor 1-beta	0.98	0.39
Ube2h	P62257	Ubiquitin-conjugating enzyme E2 H	0.98	0.50
Nhlrc3	Q8CCH2	NHL repeat-containing protein 3	0.98	0.53
Klhl9	Q6ZPT1	Kelch-like protein 9	0.98	0.62
Kctd12	A0A0R4J2B2	BTB/POZ domain-containing protein KCTD12	0.98	0.45
Nudcd2	Q9CQ48	NudC domain-containing protein 2	0.98	0.66
Eif2ak4	A2AUM0	eIF-2-alpha kinase GCN2	0.98	0.59
Ppp2r1b	G3UWS4	Serine/threonine-protein phosphatase 2A 65 kDa regulatory subunit A beta isoform	0.98	0.47

Rps7	P62082	40S ribosomal protein S7	0.98	0.51
Tlr1	Q9EPQ1	Toll-like receptor 1	0.98	0.70
Gsdme	Q9Z2D3	Gasdermin-E	0.98	0.61
Pars2	A8Y5T6	Probable proline--tRNA ligase, mitochondrial	0.98	0.60
Bckdk	O55028	[3-methyl-2-oxobutanoate dehydrogenase [lipoamide]] kinase, mitochondrial	0.98	0.68
Syncrip	A0A0R4J259	Heterogeneous nuclear ribonucleoprotein Q	0.98	0.79
Dok3	Q9QZK7	Docking protein 3	0.98	0.52
Carmil1	Q6EDY6	F-actin-uncapping protein LRRC16A	0.98	0.77
Apba3	O88888	Amyloid-beta A4 precursor protein-binding family A member 3	0.98	0.70
Arrb1	J3QNU6	Beta-arrestin-1	0.98	0.56
Apobec3	Q3U5C5	Apolipoprotein B mRNA editing enzyme, catalytic polypeptide 3	0.98	0.63
Rab5c	P35278	Ras-related protein Rab-5C	0.98	0.62
Cct4	P80315	T-complex protein 1 subunit delta	0.98	0.39
Emg1	O35130	Ribosomal RNA small subunit methyltransferase NEP1	0.98	0.45
Kdm6a	O70546	Lysine-specific demethylase 6A	0.98	0.69
Cox20	Q9D7J4	Cytochrome c oxidase assembly protein COX20, mitochondrial	0.98	0.59
Cd68	A0A0R4J1C8	Macrosialin	0.98	0.49
Ints10	A0A0R4J0V1	Integrator complex subunit 10	0.98	0.64
Oasl2	Q9Z2F2	2'-5'-oligoadenylate synthase-like protein 2	0.98	0.57
Cnbp	P53996	Cellular nucleic acid-binding protein	0.98	0.63
Spryd3	E9Q9B3	SPRY domain-containing 3	0.98	0.69
Dpm3	Q9D1Q4	Dolichol-phosphate mannosyltransferase subunit 3	0.98	0.82
Zc3hc1	Q80YV2	Nuclear-interacting partner of ALK	0.98	0.74
Ampd2	A2AE27	AMP deaminase	0.98	0.40
Pik3r2	O08908	Phosphatidylinositol 3-kinase regulatory subunit beta	0.98	0.56
Pxn	F8VQ28	Paxillin	0.98	0.60
Dlg1	E9Q9H0	Disks large homolog 1	0.98	0.62
Hat1	Q8BY71	Histone acetyltransferase type B catalytic subunit	0.98	0.57
Nudt21	Q9CQF3	Cleavage and polyadenylation specificity factor subunit 5	0.98	0.46
Ddx6	P54823	Probable ATP-dependent RNA helicase DDX6	0.98	0.45
Rnf130	Q8VEM1	E3 ubiquitin-protein ligase RNF130	0.98	0.69
Hprt1	P00493	Hypoxanthine-guanine phosphoribosyltransferase	0.98	0.51
Desi1	Q9CQT7	Desumoylating isopeptidase 1	0.98	0.77
Dr1	Q91WV0	Protein Dr1	0.98	0.70
Cenpi	Q8K1K4	Centromere protein I	0.98	0.76
Capza2	P47754	F-actin-capping protein subunit alpha-2	0.98	0.46
	Q9CRC3	UPF0235 protein C15orf40 homolog	0.98	0.65
Fdps	Q920E5	Farnesyl pyrophosphate synthase	0.98	0.25
Tollip	Q9QZ06	Toll-interacting protein	0.98	0.51
Ndufa5	Q9CPP6	NADH dehydrogenase [ubiquinone] 1 alpha subcomplex subunit 5	0.98	0.58
Gstm1	P10649	Glutathione S-transferase Mu 1	0.98	0.50
Adgre5	E9QJS7	Adhesion G protein-coupled receptor E5	0.98	0.70
Arg1	Q61176	Arginase-1	0.98	0.63
Pcna	P17918	Proliferating cell nuclear antigen	0.98	0.39
Pak1	G5E884	Non-specific serine/threonine protein kinase	0.98	0.65
Hsp90ab1	P11499	Heat shock protein HSP 90-beta	0.98	0.55
Dcaf13	Q6PAC3	DDB1- and CUL4-associated factor 13	0.98	0.66
Atp5f1a	Q03265	ATP synthase subunit alpha, mitochondrial	0.98	0.64
Mdh1	P14152	Malate dehydrogenase, cytoplasmic	0.98	0.28
Eef2	P58252	Elongation factor 2	0.98	0.47
Plin3	Q9DBG5	Perilipin-3	0.98	0.61
Ibtk	Q6ZPR6	Inhibitor of Bruton tyrosine kinase	0.98	0.34
Mnda	P0DOV1	Interferon-activable protein 205-B	0.98	0.49
Sh3bgrl3	Q91VW3	SH3 domain-binding glutamic acid-rich-like protein 3	0.98	0.61
Psme1	P97371	Proteasome activator complex subunit 1	0.98	0.52
Med27	Q9DB40	Mediator of RNA polymerase II transcription subunit 27	0.98	0.68
Rnf20	Q5DTM8	E3 ubiquitin-protein ligase BRE1A	0.98	0.66
Plin2	P43883	Perilipin-2	0.98	0.34
Smap	Q9R0P4	Small acidic protein	0.98	0.49
Ddx10	Q80Y44	Probable ATP-dependent RNA helicase DDX10	0.98	0.77
Rab11b	P46638	Ras-related protein Rab-11B	0.98	0.45
Haus1	Q8BHX1	HAUS augmin-like complex subunit 1	0.98	0.51
Dph2	Q9CR25	2-(3-amino-3-carboxypropyl)histidine synthase subunit 2	0.98	0.73
Clasrp	Q8CFC7	CLK4-associating serine/arginine rich protein	0.98	0.41
Oas2	E9Q9A9	2'-5'-oligoadenylate synthase 2	0.98	0.52
Atp5po	Q9DB20	ATP synthase subunit O, mitochondrial	0.98	0.60
Sh2b1	Q91ZM2	SH2B adapter protein 1	0.98	0.75
Fgr	P14234	Tyrosine-protein kinase Fgr	0.98	0.86
Elavl1	P70372	ELAV-like protein 1	0.98	0.58
Pebp1	P70296	Phosphatidylethanolamine-binding protein 1	0.98	0.43
Nedd1	P33215	Protein NEDD1	0.98	0.68
Shq1	Q7TMX5	Protein SHQ1 homolog	0.98	0.71
Mapkapk3	Q3UMW7	MAP kinase-activated protein kinase 3	0.98	0.61

Dpcd	Q8BPA8	Protein DPCD	0.98	0.61
Wdr74	Q8VCG3	WD repeat-containing protein 74	0.98	0.52
Snx3	Q78ZM0	Sorting nexin 3, isoform CRA_b	0.98	0.59
Adat2	Q6P6J0	tRNA-specific adenosine deaminase 2	0.98	0.70
Ctsl	P06797	Cathepsin L1	0.98	0.67
Dopey1	H7BWZ9	Protein dopey-1	0.98	0.76
Adssl1	P28650	Adenylosuccinate synthetase isozyme 1	0.98	0.63
Lcorl	Q3U285	Ligand-dependent nuclear receptor corepressor-like protein	0.98	0.76
Rps15a	P62245	40S ribosomal protein S15a	0.98	0.74
Cetn2	Q9R1K9	Centrin-2	0.98	0.66
Trim27	Q62158	Zinc finger protein RFP	0.98	0.86
Kank3	Q9Z1P7	KN motif and ankyrin repeat domain-containing protein 3	0.98	0.73
Tmem143	G3X9F4	Transmembrane protein 143	0.98	0.75
Uqcrh	P99028	Cytochrome b-c1 complex subunit 6, mitochondrial	0.98	0.73
Snx16	Q8C080	Sorting nexin-16	0.98	0.64
Pdap1	Q3UHX2	28 kDa heat- and acid-stable phosphoprotein	0.98	0.46
Armc1	Q9D7A8	Armadillo repeat-containing protein 1	0.98	0.52
Rab11fip1	E9Q8L9	Rab11 family-interacting protein 1	0.98	0.52
Eef1d	A0A0R4J1E2	Elongation factor 1-delta	0.98	0.48
Pfdn4	Q3UWL8	Prefoldin subunit 4	0.98	0.50
Engase	Q8BX80	Cytosolic endo-beta-N-acetylglucosaminidase	0.98	0.58
Zfyve21	Q8VCM3	Zinc finger FYVE domain-containing protein 21	0.98	0.80
Chil3	O35744	Chitinase-like protein 3	0.98	0.40
Rabl3	Q9D4V7	Rab-like protein 3	0.98	0.63
Mpc1	P63030	Mitochondrial pyruvate carrier 1	0.98	0.58
Gdi1	P50396	Rab GDP dissociation inhibitor alpha	0.98	0.54
Ca2	P00920	Carbonic anhydrase 2	0.98	0.71
Pyhin1	Q8BV49	Pyrin and HIN domain-containing protein 1	0.98	0.74
Spag9	Z4YMB8	C-Jun-amino-terminal kinase-interacting protein 4 (Fragment)	0.98	0.92
Cwc15	Q9JHS9	Spliceosome-associated protein CWC15 homolog	0.98	0.63
Ywhaq	F6VW30	14-3-3 protein theta (Fragment)	0.98	0.46
9930111J21Rik1	Q5SVP0	RIKEN cDNA 9930111J21 gene 1	0.98	0.65
Sirt6	P59941	NAD-dependent protein deacetylase sirtuin-6	0.98	0.79
Nap1l1	E9PW66	Nucleosome assembly protein 1-like 1	0.98	0.67
Got1	P05201	Aspartate aminotransferase, cytoplasmic	0.98	0.69
Ube2m	P61082	NEDD8-conjugating enzyme Ubc12	0.98	0.57
Ggact	Q923B0	Gamma-glutamylaminecyclotransferase	0.98	0.58
Serpinb6a	F8WIV2	Serine (or cysteine) peptidase inhibitor, clade B, member 6a	0.98	0.35
Rngtt	O55236	mRNA-capping enzyme	0.98	0.66
Gorab	Q8BRM2	RAB6-interacting golgin	0.98	0.61
Rnps1	Q99M28	RNA-binding protein with serine-rich domain 1	0.98	0.44
Dxo	A0A0R4J288	DOM-3 homolog Z (C. elegans), isoform CRA_a	0.98	0.83
Golph3l	H3BJ07	Golgi phosphoprotein 3-like	0.98	0.55
Cars2	Q8BYM8	Probable cysteine--tRNA ligase, mitochondrial	0.98	0.70
St3gal5	O88829	Lactosylceramide alpha-2,3-sialyltransferase	0.98	0.75
Rwdd1	Q9CQK7	RWD domain-containing protein 1	0.98	0.74
Fip1l1	D3Z3F1	Pre-mRNA 3'-end-processing factor FIP1 (Fragment)	0.98	0.84
Acot13	Q9CQR4	Acyl-coenzyme A thioesterase 13	0.98	0.74
Snrpd3	P62320	Small nuclear ribonucleoprotein Sm D3	0.98	0.54
Paics	Q9DCL9	Multifunctional protein ADE2	0.98	0.51
Ncl	P09405	Nucleolin	0.98	0.52
Pex6	Q99LC9	Peroxisome assembly factor 2	0.98	0.78
Prkar1a	Q9DBC7	cAMP-dependent protein kinase type I-alpha regulatory subunit	0.98	0.51
Mtmr2	Q9Z2D1	Myotubularin-related protein 2	0.98	0.50
Dynll2	Q9D0M5	Dynein light chain 2, cytoplasmic	0.98	0.84
Col4a1	P02463	Collagen alpha-1(IV) chain	0.98	0.77
Smpd3	Q9JJY3	Sphingomyelin phosphodiesterase 3	0.98	0.77
Actr1a	P61164	Alpha-centractin	0.98	0.65
Pnp	Q543K9	Purine nucleoside phosphorylase	0.98	0.62
Kctd21	B2RTJ2	BTB/POZ domain-containing protein KCTD21	0.98	0.56
Psmb9	A0A0R4J256	Proteasome subunit beta type	0.98	0.67
Cenpt	Q3TJM4	Centromere protein T	0.98	0.81
Hsd17b4	P51660	Peroxisomal multifunctional enzyme type 2	0.98	0.47
Tom1	Q3UDC3	Target of Myb protein 1	0.98	0.59
Usf2	Q64705	Upstream stimulatory factor 2	0.98	0.75
Fgd2	Q8BY35	FYVE, RhoGEF and PH domain-containing protein 2	0.98	0.79
Zbtb8os	Q505B7	Protein archease	0.98	0.52
Aldoart1	A6ZI46	Fructose-bisphosphate aldolase	0.98	0.94
Map3k1	F8VQ72	Mitogen-activated protein kinase kinase kinase 1	0.98	0.69
Traf7	F8WJF7	E3 ubiquitin-protein ligase TRAF7	0.98	0.60
Srcap	A0A087WQ44	Snf2-related CREBBP activator protein	0.98	0.85
Mpo	P11247	Myeloperoxidase	0.98	0.88
Chkb	O55229	Choline/ethanolamine kinase	0.98	0.12

Ddi2	A2ADY9	Protein DDI1 homolog 2	0.98	0.50
Eif3l	Q8QZY1	Eukaryotic translation initiation factor 3 subunit L	0.98	0.44
Memo1	Q91VH6	Protein MEMO1	0.98	0.73
Eef1a1	P10126	Elongation factor 1-alpha 1	0.98	0.67
Pkib	F7D7A9	cAMP-dependent protein kinase inhibitor beta	0.98	0.82
Ext2	P70428	Exostosin-2	0.98	0.78
Oscp1	A0ZV96	Organic solute carrier protein 1 isoform	0.98	0.85
Ass1	P16460	Argininosuccinate synthase	0.98	0.74
Ranbp1	P34022	Ran-specific GTPase-activating protein	0.98	0.55
Tmod1	P49813	Tropomodulin-1	0.98	0.85
Ywhag	P61982	14-3-3 protein gamma	0.98	0.71
Hmgb1	A0A0J9YUZ4	High mobility group protein B1 (Fragment)	0.98	0.61
Phpt1	Q9DAK9	14 kDa phosphohistidine phosphatase	0.98	0.61
Snta1	Q61234	Alpha-1-syntrophin	0.98	0.89
Fam213b	Q9DB60	Prostamide/prostaglandin F synthase	0.98	0.61
Hps5	P59438	Hermansky-Pudlak syndrome 5 protein homolog	0.98	0.75
Snx6	Q6P8X1	Sorting nexin-6	0.98	0.62
Zbtb14	Q08376	Zinc finger and BTB domain-containing protein 14	0.98	0.79
Psma7	Q9Z2U0	Proteasome subunit alpha type-7	0.98	0.63
Gls	F6U529	Glutaminase kidney isoform, mitochondrial (Fragment)	0.98	0.73
Snx33	Q4VAA7	Sorting nexin-33	0.98	0.77
Apip	Q9WVQ5	Methylthioribulose-1-phosphate dehydratase	0.98	0.55
Atic	Q9CWJ9	Bifunctional purine biosynthesis protein PURH	0.98	0.53
Lypla2	Q9WTL7	Acyl-protein thioesterase 2	0.98	0.61
Lage3	Q9CR70	EKC/KEOPS complex subunit Lage3	0.98	0.83
Picalm	A0A140LHQ8	Phosphatidylinositol-binding clathrin assembly protein (Fragment)	0.98	0.43
Stmn1	P54227	Stathmin	0.98	0.68
Dnal4	Q8CDT8	Dynein light chain	0.98	0.69
Gdap2	Q9DBL2	Ganglioside-induced differentiation-associated protein 2	0.98	0.59
Hnrnpf	Q9Z2X1	Heterogeneous nuclear ribonucleoprotein F	0.98	0.47
Washc1	Q8VDD8	WASH complex subunit 1	0.98	0.60
Rrp36	Q3UFY0	Ribosomal RNA processing protein 36 homolog	0.98	0.52
Ppp2r2a	Q6P1F6	Serine/threonine-protein phosphatase 2A 55 kDa regulatory subunit B alpha isoform	0.98	0.73
Pgpep1	Q9ESW8	Pyroglutamyl-peptidase 1	0.98	0.69
Cep55	Q8BT07	Centrosomal protein of 55 kDa	0.98	0.74
Mpst	Q99J99	3-mercaptopyruvate sulfurtransferase	0.98	0.71
Cct2	P80314	T-complex protein 1 subunit beta	0.98	0.62
Hnrnpd	Q60668	Heterogeneous nuclear ribonucleoprotein D0	0.98	0.53
Epg5	Q80TA9	Ectopic P granules protein 5 homolog	0.98	0.69
Slc39a11	Q8BWY7	Zinc transporter ZIP11	0.98	0.69
Akr1a1	Q9JII6	Alcohol dehydrogenase [NADP(+)]	0.98	0.71
Zfyve19	Q9DAZ9	Abscission/NoCut checkpoint regulator	0.98	0.55
Arpc3	Q9JM76	Actin-related protein 2/3 complex subunit 3	0.98	0.44
Ap4m1	Q9JKC7	AP-4 complex subunit mu-1	0.98	0.86
Commd10	Q8JZY2	COMM domain-containing protein 10	0.98	0.62
Copz1	P61924	Coatomer subunit zeta-1	0.98	0.76
Zyg11b	Q3UFS0	Protein zyg-11 homolog B	0.98	0.72
Capg	Q99LB4	Capping protein (Actin filament), gelsolin-like	0.98	0.59
Anp32e	P97822	Acidic leucine-rich nuclear phosphoprotein 32 family member E	0.98	0.57
Ttpal	Q9D3D0	Alpha-tocopherol transfer protein-like	0.98	0.80
Taf9b	A2AP82	Transcription initiation factor TFIID subunit 9B	0.98	0.81
Vps13b	Q80TY5	Vacuolar protein sorting-associated protein 13B	0.98	0.89
Prdx5	P99029	Peroxiredoxin-5, mitochondrial	0.98	0.47
Hgh1	Q8C3I8	Protein HGH1 homolog	0.98	0.80
Stat5b	P42232	Signal transducer and activator of transcription 5B	0.98	0.58
Dhx35	A2ACQ1	DEAH (Asp-Glu-Ala-His) box polypeptide 35	0.98	0.82
Terf2	O35144	Telomeric repeat-binding factor 2	0.98	0.61
Myg1	Q9JK81	UPF0160 protein MYG1, mitochondrial	0.98	0.41
Alg11	Q3TZM9	GDP-Man:Man(3)GlcNAc(2)-PP-Dol alpha-1,2-mannosyltransferase	0.99	0.72
Cbr2	P08074	Carbonyl reductase [NADPH] 2	0.99	0.82
Ctss	F6WR04	Cathepsin S	0.99	0.70
Bin1	Q6P1B9	Bin1 protein	0.99	0.68
Tspan31	Q9CQ88	Tetraspanin-31	0.99	0.79
Itpr3	P70227	Inositol 1,4,5-trisphosphate receptor type 3	0.99	0.85
Cct3	P80318	T-complex protein 1 subunit gamma	0.99	0.50
Rps12	A0A1W2P7A1	40S ribosomal protein S12	0.99	0.56
Nol10	Q5RJG1	Nucleolar protein 10	0.99	0.80
Psmd12	Q9D8W5	26S proteasome non-ATPase regulatory subunit 12	0.99	0.53
Hist1h3a	P68433	Histone H3.1	0.99	0.75
Rhbdd1	Q8BHC7	Rhomboid-related protein 4	0.99	0.72
2410002F23Rik	Q3TE80	RIKEN cDNA 2410002F23 gene	0.99	0.83
Dcps	Q9DAR7	m7GpppX diphosphatase	0.99	0.45
Aff4	Q9ESC8	AF4/FMR2 family member 4	0.99	0.89

Mthfr	A2A7F7	Methylenetetrahydrofolate reductase	0.99	0.71
2010005H15Rik	Q9D8D6	2010005H15Rik protein	0.99	0.90
Pcbp2	A0A2R8W6U6	Poly(rC)-binding protein 2 (Fragment)	0.99	0.73
Capza1	Q5RKN9	Capping protein (Actin filament) muscle Z-line, alpha 1	0.99	0.62
Lacc1	Q8BZT9	Laccase domain-containing protein 1	0.99	0.79
Thnsl1	Q8BH55	Threonine synthase-like 1	0.99	0.83
Unk	Q8BL48	RING finger protein unkempt homolog	0.99	0.71
Npepl1	Q6NSR8	Probable aminopeptidase NPEPL1	0.99	0.68
Dpysl2	O08553	Dihydropyrimidinase-related protein 2	0.99	0.69
Raver1	Q9CW46	Ribonucleoprotein PTB-binding 1	0.99	0.74
Ppp1r2	Q9DCL8	Protein phosphatase inhibitor 2	0.99	0.72
Trim24	Q64127	Transcription intermediary factor 1-alpha	0.99	0.87
Mnat1	P51949	CDK-activating kinase assembly factor MAT1	0.99	0.71
Rfc5	Q9D0F6	Replication factor C subunit 5	0.99	0.69
Tgtp2	Q3T9E4	T-cell-specific guanine nucleotide triphosphate-binding protein 2	0.99	0.94
Srm	Q64674	Spermidine synthase	0.99	0.52
Vat1	Q62465	Synaptic vesicle membrane protein VAT-1 homolog	0.99	0.53
Anxa1	P10107	Annexin A1	0.99	0.69
Anxa4	P97429	Annexin A4	0.99	0.64
Hint1	P70349	Histidine triad nucleotide-binding protein 1	0.99	0.55
Trim30a	P15533	Tripartite motif-containing protein 30A	0.99	0.75
Pdia6	Q3TML0	Protein disulfide-isomerase A6	0.99	0.63
Prdx6	Q6GT24	Peroxiredoxin-6	0.99	0.29
Mydgf	Q9CPT4	Myeloid-derived growth factor	0.99	0.57
Pyurf	Q9D1C3	Protein preY, mitochondrial	0.99	0.53
Commd2	Q8BXC6	COMM domain-containing protein 2	0.99	0.60
Rgl3	Q3UYI5	Ral guanine nucleotide dissociation stimulator-like 3	0.99	0.78
Cmpk1	Q9DBP5	UMP-CMP kinase	0.99	0.69
Twf2	Q9Z0P5	Twinfilin-2	0.99	0.64
Cwc27	Q3TKY6	Spliceosome-associated protein CWC27 homolog	0.99	0.67
Nabp2	E9Q199	SOSS complex subunit B1	0.99	0.82
Eef1b	O70251	Elongation factor 1-beta	0.99	0.36
N6amt1	Q6SKR2	Methyltransferase N6AMT1	0.99	0.67
Anxa5	P48036	Annexin A5	0.99	0.61
Zbp1	A2APF7	Z-DNA-binding protein 1	0.99	0.77
Iah1	Q9DB29	Isoamyl acetate-hydrolyzing esterase 1 homolog	0.99	0.64
Dynlrb1	A2AVR9	Dynein light chain roadblock	0.99	0.56
Rab20	P35295	Ras-related protein Rab-20	0.99	0.70
Ywhaz	P63101	14-3-3 protein zeta/delta	0.99	0.49
Hmbox1	H3BKF8	Homeobox-containing protein 1 (Fragment)	0.99	0.72
1700037H04Rik	F8WIU1	RIKEN cDNA 1700037H04 gene	0.99	0.90
Gsap	Q3TCV3	Gamma-secretase-activating protein	0.99	0.78
Mtnd4l	P03903	NADH-ubiquinone oxidoreductase chain 4L	0.99	0.80
Cct5	P80316	T-complex protein 1 subunit epsilon	0.99	0.57
Trmt112	Q9DCG9	Multifunctional methyltransferase subunit TRM112-like protein	0.99	0.81
Cyb5a	P56395	Cytochrome b5	0.99	0.69
Txndc12	Q9CQU0	Thioredoxin domain-containing protein 12	0.99	0.68
Anxa2	P07356	Annexin A2	0.99	0.80
Zhx1	P70121	Zinc fingers and homeoboxes protein 1	0.99	0.75
Cstb	Q62426	Cystatin-B	0.99	0.60
Twistnb	Q78WZ7	DNA-directed RNA polymerase I subunit RPA43	0.99	0.79
Pkm	P52480	Pyruvate kinase PKM	0.99	0.53
Disc1	D3YYC9	Disrupted in schizophrenia 1 homolog	0.99	0.84
Cnpy2	Q9QXT0	Protein canopy homolog 2	0.99	0.71
Ppil1	Q9D0W5	Peptidyl-prolyl cis-trans isomerase-like 1	0.99	0.60
Akr1b8	P45377	Aldose reductase-related protein 2	0.99	0.73
Ube2n	P61089	Ubiquitin-conjugating enzyme E2 N	0.99	0.51
Ubfd1	Q78JW9	Ubiquitin domain-containing protein UBFD1	0.99	0.59
Mapre1	Q61166	Microtubule-associated protein RP/EB family member 1	0.99	0.67
Agap1	Q8BXK8	Arf-GAP with GTPase, ANK repeat and PH domain-containing protein 1	0.99	0.76
Aoah	O35298	Acyloxyacyl hydrolase	0.99	0.75
Eif2ak2	Q03963	Interferon-induced, double-stranded RNA-activated protein kinase	0.99	0.69
Kank2	Q8BX02	KN motif and ankyrin repeat domain-containing protein 2	0.99	0.77
Rwdd2b	Q99M03	RWD domain-containing protein 2B	0.99	0.93
Tmbim6	Q9D2C7	Bax inhibitor 1	0.99	0.80
Acsl3	Q9CZW4	Long-chain-fatty-acid--CoA ligase 3	0.99	0.60
Snx5	Q9D8U8	Sorting nexin-5	0.99	0.67
Depdc5	P61460	GATOR complex protein DEPDC5	0.99	0.78
Ep400	Q8CHI8	E1A-binding protein p400	0.99	0.71
Fry	E9Q8I9	Protein furry homolog	0.99	0.86
Hs1bp3	Q3TC93	HCLS1-binding protein 3	0.99	0.47
Hist1h2bf	P10853	Histone H2B type 1-F/J/L	0.99	0.69
Relch	E9QM90	RAB11-binding protein RELCH	0.99	0.77

Sh3pxd2a	O89032	SH3 and PX domain-containing protein 2A	0.99	0.73
Herc6	F2Z461	E3 ISG15--protein ligase Herc6	0.99	0.72
Zfp428	H3BLD9	Zinc finger protein 428	0.99	0.87
Scaf1	Q5U4C3	Splicing factor, arginine/serine-rich 19	0.99	0.66
Ctsz	Q9WUU7	Cathepsin Z	0.99	0.33
Dcun1d2	Q8BZJ7	DCN1-like protein 2	0.99	0.72
Psma6	Q9QUM9	Proteasome subunit alpha type-6	0.99	0.77
Asah1	Q9WV54	Acid ceramidase	0.99	0.59
Hexa	P29416	Beta-hexosaminidase subunit alpha	0.99	0.66
Elac1	Q8VEB6	Zinc phosphodiesterase ELAC protein 1	0.99	0.70
Ypel5	P62700	Protein yippee-like 5	0.99	0.66
Ripk1	Q60855	Receptor-interacting serine/threonine-protein kinase 1	0.99	0.59
Lgmn	O89017	Legumain	0.99	0.43
Tuba1a	P68369	Tubulin alpha-1A chain	0.99	0.67
Kdm2a	F6YRW4	F-box and leucine-rich repeat protein 11	0.99	0.77
Lmna	P48678	Prelamin-A/C	0.99	0.46
Ptges3	Q9R0Q7	Prostaglandin E synthase 3	0.99	0.36
Psmb2	Q9R1P3	Proteasome subunit beta type-2	0.99	0.47
Snx12	Q3V2H3	Sorting nexin-12	0.99	0.48
Uba3	Q8C878	NEDD8-activating enzyme E1 catalytic subunit	0.99	0.59
Csnk1g1	Q8BTH8	Casein kinase I isoform gamma-1	0.99	0.42
Ciao2a	Q9DCL2	Cytosolic iron-sulfur assembly component 2A	0.99	0.63
Pura	P42669	Transcriptional activator protein Pur-alpha	0.99	0.63
Ppp1ca	P62137	Serine/threonine-protein phosphatase PP1-alpha catalytic subunit	0.99	0.59
Gcdh	A0A0A0MQ68	Glutaryl-CoA dehydrogenase, mitochondrial	0.99	0.78
Mrpl50	Q8VDT9	39S ribosomal protein L50, mitochondrial	0.99	0.57
Eif4a1	P60843	Eukaryotic initiation factor 4A-I	0.99	0.57
Cep41	Q99NF3	Centrosomal protein of 41 kDa	0.99	0.59
Cib1	Q9Z0F4	Calcium and integrin-binding protein 1	0.99	0.84
Dctn3	Q9Z0Y1	Dynactin subunit 3	0.99	0.57
Ctsf	Q9R013	Cathepsin F	0.99	0.76
Setdb1	D3YYC3	Histone-lysine N-methyltransferase	0.99	0.84
Htra2	Q9JIY5	Serine protease HTRA2, mitochondrial	0.99	0.69
Ehhadh	Q9DBM2	Peroxisomal bifunctional enzyme	0.99	0.75
Tfg	Q9Z1A1	TFG protein	0.99	0.50
Dnm2	P39054	Dynamin-2	0.99	0.69
B3galnt1	Q920V1	UDP-GalNAc:beta-1,3-N-acetylgalactosaminyltransferase 1	0.99	0.75
Sod1	P08228	Superoxide dismutase [Cu-Zn]	0.99	0.63
Hnrnpl	G5E924	Heterogeneous nuclear ribonucleoprotein L (Fragment)	0.99	0.70
Eif3m	Q99JX4	Eukaryotic translation initiation factor 3 subunit M	0.99	0.66
Grb2	Q60631	Growth factor receptor-bound protein 2	0.99	0.67
Ddx19b	Q8BZY3	DEAD (Asp-Glu-Ala-Asp) box polypeptide 19b	0.99	0.90
Dhrs1	Q99L04	Dehydrogenase/reductase SDR family member 1	0.99	0.74
	Q8C708	Transmembrane protein C16orf54 homolog	0.99	0.76
Mtrf1l	Q8BJU9	Peptide chain release factor 1-like, mitochondrial	0.99	0.88
Banf1	O54962	Barrier-to-autointegration factor	0.99	0.68
Pdxk	Q8K183	Pyridoxal kinase	0.99	0.83
Mrps21	P58059	28S ribosomal protein S21, mitochondrial	0.99	0.57
Nab2	Q61127	NGFI-A-binding protein 2	0.99	0.58
Hnrnpa2b1	O88569	Heterogeneous nuclear ribonucleoproteins A2/B1	0.99	0.59
Lsm1	Q8VC85	U6 snRNA-associated Sm-like protein LSm1	0.99	0.69
Cic	Q924A2	Protein capicua homolog	0.99	0.79
Tom1l2	Q5SRX1	TOM1-like protein 2	0.99	0.83
Cep250	A3KGJ7	Centrosome-associated protein CEP250	0.99	0.87
Arglu1	Q3UL36	Arginine and glutamate-rich protein 1	0.99	0.75
F13a1	Q8BH61	Coagulation factor XIII A chain	0.99	0.69
Rin3	P59729	Ras and Rab interactor 3	0.99	0.66
Stam2	O88811	Signal transducing adapter molecule 2	0.99	0.54
Fcer1g	P20491	High affinity immunoglobulin epsilon receptor subunit gamma	0.99	0.84
Rplp0	P14869	60S acidic ribosomal protein P0	0.99	0.56
Nop16	D3Z7M5	Nucleolar protein 16	0.99	0.75
Prkar2a	Q8K1M3	Protein kinase, cAMP dependent regulatory, type II alpha	0.99	0.56
Taf1	D3YZK4	Transcription initiation factor TFIID subunit	0.99	0.81
Rasa2	P58069	Ras GTPase-activating protein 2	0.99	0.71
Bin2	S4R270	Bridging integrator 2	0.99	0.45
Dpp3	Q99KK7	Dipeptidyl peptidase 3	0.99	0.55
Tiprl	Q8BH58	TIP41-like protein	0.99	0.70
Trim65	Q8BFW4	Tripartite motif-containing protein 65	0.99	0.78
Gyg	K3W4S6	Glycogenin	0.99	0.83
Phyh	O35386	Phytanoyl-CoA dioxygenase, peroxisomal	0.99	0.72
Arpc2	A0A087WRT2	Actin-related protein 2/3 complex subunit 2 (Fragment)	0.99	0.91
Chordc1	Q9D1P4	Cysteine and histidine-rich domain-containing protein 1	0.99	0.54
Zmat2	Q9CPW7	Zinc finger matrin-type protein 2	0.99	0.64

Saal1	Q9D2C2	Protein SAAL1	0.99	0.80
Sptb	Q3UGX2	Spectrin beta chain	0.99	0.85
Ugt1a6	Q64435	UDP-glucuronosyltransferase 1-6	0.99	0.84
Acvrl1	Q61288	Serine/threonine-protein kinase receptor R3	0.99	0.85
Trex1	Q91XB0	Three-prime repair exonuclease 1	0.99	0.64
Dhx32	Q8BZS9	Putative pre-mRNA-splicing factor ATP-dependent RNA helicase DHX32	0.99	0.73
Hsp90aa1	P07901	Heat shock protein HSP 90-alpha	0.99	0.57
Med4	Q9CQA5	Mediator of RNA polymerase II transcription subunit 4	0.99	0.78
Scfd2	Q8BTY8	Sec1 family domain-containing protein 2	0.99	0.80
Cacybp	Q9CXW3	Calcyclin-binding protein	0.99	0.62
Idh1	O88844	Isocitrate dehydrogenase [NADP] cytoplasmic	0.99	0.57
Nucks1	Q80XU3	Nuclear ubiquitous casein and cyclin-dependent kinase substrate 1	0.99	0.63
B3GNT2	Q9Z222	N-acetyllactosaminide beta-1,3-N-acetylglucosaminyltransferase 2	0.99	0.80
Akap8l	Q9R0L7	A-kinase anchor protein 8-like	0.99	0.87
Clpp	O88696	ATP-dependent Clp protease proteolytic subunit, mitochondrial	0.99	0.79
Glul	P15105	Glutamine synthetase	0.99	0.51
Ap2s1	P62743	AP-2 complex subunit sigma	0.99	0.59
Tbc1d8	Q9Z1A9	TBC1 domain family member 8	0.99	0.81
Gorasp2	Q99JX3	Golgi reassembly-stacking protein 2	0.99	0.62
Samd4b	G5E8A7	Protein Smaug homolog 2	0.99	0.80
Ctsh	P49935	Pro-cathepsin H	0.99	0.67
Rogdi	Q3TDK6	Protein rogdi homolog	0.99	0.90
Erp29	P57759	Endoplasmic reticulum resident protein 29	0.99	0.73
Actr3	Q99JY9	Actin-related protein 3	0.99	0.69
Plec	A0A0R4J221	Plectin (Fragment)	0.99	0.77
Tarbp1	E9Q368	TAR RNA-binding protein 1	0.99	0.77
Mcrs1	Q99L90	Microspherule protein 1	0.99	0.84
Psmd14	O35593	26S proteasome non-ATPase regulatory subunit 14	0.99	0.59
Ptbp1	Q922I7	MCG13402, isoform CRA_c	0.99	0.76
Hspa8	P63017	Heat shock cognate 71 kDa protein	0.99	0.46
Hddc2	Q3SXD3	HD domain-containing protein 2	0.99	0.78
Jpt1	P97825	Jupiter microtubule associated homolog 1	0.99	0.43
Sacs	E9QNY8	Sacsin	0.99	0.81
Brca1	P48754	Breast cancer type 1 susceptibility protein homolog	0.99	0.84
Cndp2	Q9D1A2	Cytosolic non-specific dipeptidase	0.99	0.80
Txn2	P97493	Thioredoxin, mitochondrial	0.99	0.44
Stat5a	P42230	Signal transducer and activator of transcription 5A	0.99	0.63
Nvl	Q9DBY8	Nuclear valosin-containing protein-like	0.99	0.73
Vta1	Q9CR26	Vacuolar protein sorting-associated protein VTA1 homolog	0.99	0.82
Arl14ep	Q8BIX3	ARL14 effector protein	0.99	0.82
AI837181	E9QNR6	Expressed sequence AI837181	0.99	0.19
Kiaa0513	Q8R0A7	Uncharacterized protein KIAA0513	0.99	0.69
Uba1	Q02053	Ubiquitin-like modifier-activating enzyme 1	0.99	0.58
Stxbp2	F8WGM5	Syntaxin-binding protein 2 (Fragment)	0.99	0.84
Sec61g	G3UWH0	MCG7641, isoform CRA_b	0.99	0.66
Eif6	O55135	Eukaryotic translation initiation factor 6	0.99	0.59
Casp3	P70677	Caspase-3	0.99	0.68
Add3	Q9QYB5	Gamma-adducin	0.99	0.75
Mob2	A0A1B0GR18	MOB kinase activator 2	0.99	0.70
Igf1	Q8CAR0	Insulin-like growth factor I	0.99	0.79
Tor1a	Q9ER39	Torsin-1A	0.99	0.73
Ube2o	Q6ZPJ3	(E3-independent) E2 ubiquitin-conjugating enzyme UBE2O	0.99	0.68
Mdh2	P08249	Malate dehydrogenase, mitochondrial	0.99	0.69
Nsrp1	Q5NCR9	Nuclear speckle splicing regulatory protein 1	0.99	0.84
Ppt1	O88531	Palmitoyl-protein thioesterase 1	0.99	0.76
Polr3a	B2RXC6	DNA-directed RNA polymerase subunit	0.99	0.51
Cct7	P80313	T-complex protein 1 subunit eta	0.99	0.67
Dgkh	D3YXJ0	Diacylglycerol kinase	0.99	0.84
Nmral1	Q8K2T1	NmrA-like family domain-containing protein 1	0.99	0.42
Bud31	Q6PGH1	Protein BUD31 homolog	0.99	0.75
Fkbp1a	P26883	Peptidyl-prolyl cis-trans isomerase FKBP1A	0.99	0.93
Sf1	D3YZC9	Splicing factor 1	0.99	0.71
Gatad2b	Q8VHR5	Transcriptional repressor p66-beta	0.99	0.75
Khsrp	Q3U0V1	Far upstream element-binding protein 2	0.99	0.55
Man2b1	O09159	Lysosomal alpha-mannosidase	0.99	0.51
Tpm3	E9Q7Q3	Tropomyosin alpha-3 chain	0.99	0.44
Mic13	Q8R404	MICOS complex subunit MIC13	0.99	0.82
Sra1	Q80VJ2	Steroid receptor RNA activator 1	0.99	0.70
Pgd	Q9DCD0	6-phosphogluconate dehydrogenase, decarboxylating	0.99	0.45
St13	Q99L47	Hsc70-interacting protein	0.99	0.53
Ifitm3	Q9CQW9	Interferon-induced transmembrane protein 3	0.99	0.85
Lrrc25	Q8K1T1	Leucine-rich repeat-containing protein 25	0.99	0.81
Pus1	Q9WU56	tRNA pseudouridine synthase A	0.99	0.64

Dazap1	Q3UGB5	DAZ associated protein 1, isoform CRA_b	0.99	0.65
Rpa1	Q8VEE4	Replication protein A 70 kDa DNA-binding subunit	0.99	0.74
Clic4	Q9QYB1	Chloride intracellular channel protein 4	0.99	0.72
Sfswap	Q3USH5	Splicing factor, suppressor of white-apricot homolog	0.99	0.69
Snrpf	P62307	Small nuclear ribonucleoprotein F	0.99	0.55
Pan2	Q8BGF7	PAN2-PAN3 deadenylation complex catalytic subunit Pan2	0.99	0.73
Ilk	O55222	Integrin-linked protein kinase	0.99	0.40
Pggt1b	Q8BUY9	Geranylgeranyl transferase type-1 subunit beta	0.99	0.74
Als2	Q920R0	Alsin	0.99	0.74
Lypla1	P97823	Acyl-protein thioesterase 1	0.99	0.57
Inip	Q3TXT3	SOSS complex subunit C	0.99	0.59
Gpc1	Q9QZF2	Glypican-1	0.99	0.74
Fblim1	Q71FD7	Filamin-binding LIM protein 1	0.99	0.70
Commd5	A0A0R4J0U7	COMM domain-containing protein 5	0.99	0.70
Nudcd3	Q8R1N4	NudC domain-containing protein 3	0.99	0.62
Phf5a	P83870	PHD finger-like domain-containing protein 5A	0.99	0.65
Hmgb2	P30681	High mobility group protein B2	0.99	0.76
Tcea1	P10711	Transcription elongation factor A protein 1	0.99	0.70
F8a	Q9JJQ6	Factor 8-associated gene A	0.99	0.74
Sfpq	Q8VIJ6	Splicing factor, proline- and glutamine-rich	0.99	0.58
Rab28	Q99KL7	Ras-related protein Rab-28	0.99	0.87
Ppp2r1a	Q76MZ3	Serine/threonine-protein phosphatase 2A 65 kDa regulatory subunit A alpha isoform	0.99	0.59
Kdm2b	Q6P1G2	Lysine-specific demethylase 2B	0.99	0.75
Eif1a	Q60872	Eukaryotic translation initiation factor 1A	0.99	0.70
Pus7	Q91VU7	Pseudouridylate synthase 7 homolog	0.99	0.75
Rhot2	Q8JZN7	Mitochondrial Rho GTPase 2	0.99	0.76
Scaf11	E9PZM7	SR-related CTD-associated factor 11	0.99	0.75
Wdfy2	Q8BUB4	WD repeat and FYVE domain-containing protein 2	0.99	0.78
Rrm1	P07742	Ribonucleoside-diphosphate reductase large subunit	0.99	0.69
Gnb1	P62874	Guanine nucleotide-binding protein G(I)/G(S)/G(T) subunit beta-1	0.99	0.69
Raf1	Q99N57	RAF proto-oncogene serine/threonine-protein kinase	0.99	0.76
Trappc4	Q9ES56	Trafficking protein particle complex subunit 4	0.99	0.62
Glb1	P23780	Beta-galactosidase	0.99	0.62
Nudt8	Q9CR24	Nucleoside diphosphate-linked moiety X motif 8	0.99	0.83
Mrps2	Q924T2	28S ribosomal protein S2, mitochondrial	0.99	0.74
Mrpl12	Q9DB15	39S ribosomal protein L12, mitochondrial	0.99	0.64
Dnase2	P56542	Deoxyribonuclease-2-alpha	0.99	0.41
Rpl11	Q9CXW4	60S ribosomal protein L11	0.99	0.69
Rcor1	Q8CFE3	REST corepressor 1	0.99	0.73
Tbc1d8b	A3KGB4	TBC1 domain family member 8B	0.99	0.73
Hypk	Q9CR41	Huntingtin-interacting protein K	0.99	0.65
Clns1a	Q923F1	Chloride channel, nucleotide-sensitive, 1A	0.99	0.44
Ppm1h	Q3UYC0	Protein phosphatase 1H	0.99	0.69
Gpd1l	Q3ULJ0	Glycerol-3-phosphate dehydrogenase 1-like protein	0.99	0.49
Rsbn1l	D3Z0K6	MCG120108, isoform CRA_a	0.99	0.83
Napa	Q9DB05	Alpha-soluble NSF attachment protein	0.99	0.69
Eno2	P17183	Gamma-enolase	0.99	0.92
Cemip2	Q5FWI3	Cell surface hyaluronidase	0.99	0.82
Prkar2b	P31324	cAMP-dependent protein kinase type II-beta regulatory subunit	0.99	0.65
Ecd	Q9CS74	Protein ecdysoneless homolog	0.99	0.72
Dus2	Q9D7B1	tRNA-dihydrouridine(20) synthase [NAD(P)+]-like	0.99	0.87
Ciao1	Q99KN2	Probable cytosolic iron-sulfur protein assembly protein CIAO1	0.99	0.58
Cltc	Q5SXR6	Clathrin heavy chain	0.99	0.66
Sf3b6	P59708	Splicing factor 3B subunit 6	0.99	0.71
Gpalpp1	Q69ZC8	GPALPP motifs-containing protein 1	0.99	0.82
Hdac1	O09106	Histone deacetylase 1	0.99	0.62
Lcp1	Q61233	Plastin-2	0.99	0.56
Atpif1	E9PV44	ATPase inhibitory factor 1	0.99	0.67
Stat1	Q99K94	Signal transducer and activator of transcription	0.99	0.81
Prmt7	Q922X9	Protein arginine N-methyltransferase 7	0.99	0.83
Usp5	Q3U4W8	Ubiquitin carboxyl-terminal hydrolase	0.99	0.61
Ocrl	Q6NVF0	Inositol polyphosphate 5-phosphatase OCRL-1	0.99	0.81
Cdk1	P11440	Cyclin-dependent kinase 1	0.99	0.78
Vrk3	Q8K3G5	Inactive serine/threonine-protein kinase VRK3	0.99	0.88
Gabarapl2	P60521	Gamma-aminobutyric acid receptor-associated protein-like 2	0.99	0.62
Bloc1s4	Q8VED2	Biogenesis of lysosome-related organelles complex 1 subunit 4	0.99	0.69
Fam221a	Q8C790	Protein FAM221A	0.99	0.81
Ssr4	Q9D8L3	Signal sequence receptor, delta	0.99	0.90
Lsm11	Q8BUV6	U7 snRNA-associated Sm-like protein LSm11	0.99	0.82
Vps29	Q9QZ88	Vacuolar protein sorting-associated protein 29	0.99	0.53
Actg1	F8WGM8	Actin, cytoplasmic 2 (Fragment)	0.99	0.95
Nt5dc3	Q3UHB1	5'-nucleotidase domain-containing protein 3	0.99	0.82
Psma5	Q9Z2U1	Proteasome subunit alpha type-5	0.99	0.62

Picalm	Q7M6Y3	Phosphatidylinositol-binding clathrin assembly protein	0.99	0.56
Tubb2a	Q7TMM9	Tubulin beta-2A chain	0.99	0.70
Ppm1g	Q61074	Protein phosphatase 1G	0.99	0.71
Tardbp	Q921F2	TAR DNA-binding protein 43	0.99	0.58
Actb	E9Q5F4	Actin, cytoplasmic 1 (Fragment)	0.99	0.74
Pdlim7	Q3TJD7	PDZ and LIM domain protein 7	0.99	0.79
Ints7	A0A0R4J0E4	Integrator complex subunit 7	0.99	0.75
Psmd8	Q9CX56	26S proteasome non-ATPase regulatory subunit 8	0.99	0.79
Card9	A2AIV8	Caspase recruitment domain-containing protein 9	0.99	0.71
Gm43738	A0A0G2JEA5	Predicted gene 43738	0.99	0.83
Naglu	O88325	Alpha-N-acetylglucosaminidase	0.99	0.61
Gstp1	P19157	Glutathione S-transferase P 1	0.99	0.81
Psmd13	Q9WVJ2	26S proteasome non-ATPase regulatory subunit 13	0.99	0.61
Cops3	O88543	COP9 signalosome complex subunit 3	0.99	0.65
Polr1e	Q8K202	DNA-directed RNA polymerase I subunit RPA49	0.99	0.88
Gamt	O35969	Guanidinoacetate N-methyltransferase	0.99	0.80
Dph5	Q9CWQ0	Diphthine methyl ester synthase	0.99	0.85
Ube2d2	P62838	Ubiquitin-conjugating enzyme E2 D2	0.99	0.70
Rnaseh2b	Q80ZV0	Ribonuclease H2 subunit B	0.99	0.87
Pstpip1	A0A0R4J0P5	Proline-serine-threonine phosphatase-interacting protein 1	0.99	0.52
Rbks	Q8R1Q9	Ribokinase	0.99	0.65
Fau	Q642K5	40S ribosomal protein S30	0.99	0.60
Tango2	P54797	Transport and Golgi organization 2 homolog	0.99	0.88
Alox5ap	A0A0J9YV38	Arachidonate 5-lipoxygenase-activating protein (Fragment)	0.99	0.91
G6pdx	Q00612	Glucose-6-phosphate 1-dehydrogenase X	0.99	0.66
Cnot6l	Q8VEG6	CCR4-NOT transcription complex subunit 6-like	0.99	0.84
Npc2	Q9Z0J0	NPC intracellular cholesterol transporter 2	0.99	0.78
Dvl3	E9Q967	Segment polarity protein dishevelled homolog DVL-3	0.99	0.68
Pfkl	P12382	ATP-dependent 6-phosphofructokinase, liver type	0.99	0.77
Clic1	Q9Z1Q5	Chloride intracellular channel protein 1	0.99	0.73
Slain2	Q8CI08	SLAIN motif-containing protein 2	0.99	0.77
Prrc1	Q3UPH1	Protein PRRC1	0.99	0.76
Isg15	Q64339	Ubiquitin-like protein ISG15	0.99	0.65
Eef1g	Q9D8N0	Elongation factor 1-gamma	0.99	0.59
Tubb5	P99024	Tubulin beta-5 chain	0.99	0.68
Cdkn1b	P46414	Cyclin-dependent kinase inhibitor 1B	0.99	0.51
Mocs2	A0A0R4J206	Molybdopterin synthase catalytic subunit	0.99	0.52
Gnpnat1	Q9JK38	Glucosamine 6-phosphate N-acetyltransferase	0.99	0.74
Mbd3	Q9Z2D8	Methyl-CpG-binding domain protein 3	0.99	0.44
Mcm4	P49717	DNA replication licensing factor MCM4	0.99	0.75
Dhx9	E9QNN1	ATP-dependent RNA helicase A	0.99	0.63
Tlr8	P58682	Toll-like receptor 8	0.99	0.79
Atp6v1a	P50516	V-type proton ATPase catalytic subunit A	0.99	0.74
Acta1	P68134	Actin, alpha skeletal muscle	0.99	0.80
Shmt1	P50431	Serine hydroxymethyltransferase, cytosolic	0.99	0.82
Tkt	P40142	Transketolase	0.99	0.57
Ubl5	E0CZB3	Ubiquitin-like protein 5	0.99	0.85
Ankrd13a	Q80UP5	Ankyrin repeat domain-containing protein 13A	0.99	0.73
Phka2	Q8BWJ3	Phosphorylase b kinase regulatory subunit alpha, liver isoform	0.99	0.74
Cstf2	Q8BIQ5	Cleavage stimulation factor subunit 2	0.99	0.82
Oard1	Q8R5F3	O-acetyl-ADP-ribose deacetylase 1	0.99	0.77
Hist2h2aa1	Q6GSS7	Histone H2A type 2-A	0.99	0.86
Sdhb	Q9CQA3	Succinate dehydrogenase [ubiquinone] iron-sulfur subunit, mitochondrial	0.99	0.64
Gsr	P47791	Glutathione reductase, mitochondrial	0.99	0.65
Ahsa1	Q8BK64	Activator of 90 kDa heat shock protein ATPase homolog 1	0.99	0.65
Dhx16	G3X8X0	DEAH (Asp-Glu-Ala-His) box polypeptide 16	0.99	0.68
Ap2a2	P17427	AP-2 complex subunit alpha-2	0.99	0.57
Mrgbp	Q9DAT2	MRG/MORF4L-binding protein	0.99	0.85
Irf5	P56477	Interferon regulatory factor 5	0.99	0.60
Csnk2a1	Q60737	Casein kinase II subunit alpha	0.99	0.83
Nmt1	O70310	Glycylpeptide N-tetradecanoyltransferase 1	0.99	0.67
Cpne3	Q8BT60	Copine-3	0.99	0.60
Copb2	O55029	Coatomer subunit beta'	0.99	0.60
Rnf181	D3YUJ1	E3 ubiquitin-protein ligase RNF181	0.99	0.72
Tmed8	Q3UHI4	Protein TMED8	0.99	0.76
Flcn	Q8QZS3	Folliculin	0.99	0.88
Eif2b3	B1AUN2	Eukaryotic translation initiation factor 2B, subunit 3	0.99	0.75
Med16	G3UW74	Mediator of RNA polymerase II transcription subunit 16	0.99	0.81
Usp14	Q9JMA1	Ubiquitin carboxyl-terminal hydrolase 14	0.99	0.67
Tpt1	P63028	Translationally-controlled tumor protein	0.99	0.43
Ncbp2	Q9CQ49	Nuclear cap-binding protein subunit 2	0.99	0.71
Senp3	Q9EP97	Sentrin-specific protease 3	0.99	0.83
Atxn2	E9QM77	Ataxin-2	0.99	0.63

Eng	Q63961	Endoglin	0.99	0.87
Gm16286	Q9CQX6	MCG141091, isoform CRA_a	0.99	0.88
Snrnp27	Q8K194	U4/U6.U5 small nuclear ribonucleoprotein 27 kDa protein	0.99	0.69
Wdr1	O88342	WD repeat-containing protein 1	0.99	0.81
Rpl27	P61358	60S ribosomal protein L27	0.99	0.74
Map3k4	O08648	Mitogen-activated protein kinase kinase kinase 4	0.99	0.79
D6Wsu163e	Q91YN0	Protein C12orf4 homolog	0.99	0.76
Prune2	Q52KR3	Protein prune homolog 2	0.99	0.82
Gspt1	Q8R050	Eukaryotic peptide chain release factor GTP-binding subunit ERF3A	0.99	0.66
Pdcd6ip	Q9WU78	Programmed cell death 6-interacting protein	0.99	0.71
Pdlim4	P70271	PDZ and LIM domain protein 4	0.99	0.69
Pfdn1	Q9CQF7	Prefoldin 1	0.99	0.76
Dnajb1	Q9QYJ3	DnaJ homolog subfamily B member 1	0.99	0.80
Aldh2	A0A0G2JEU1	Aldehyde dehydrogenase, mitochondrial	0.99	0.88
Mapk8	G3X8U9	Mitogen-activated protein kinase	0.99	0.85
Edem3	A0A087WR24	alpha-1,2-Mannosidase	0.99	0.86
Dhx58	Q99J87	Probable ATP-dependent RNA helicase DHX58	0.99	0.73
Coq4	Q8BGB8	Ubiquinone biosynthesis protein COQ4 homolog, mitochondrial	0.99	0.75
Mcm7	Q61881	DNA replication licensing factor MCM7	0.99	0.74
Scpep1	Q920A5	Retinoid-inducible serine carboxypeptidase	0.99	0.60
Pik3r1	P26450	Phosphatidylinositol 3-kinase regulatory subunit alpha	0.99	0.75
Dcxr	Q91X52	L-xylulose reductase	0.99	0.73
Limd2	Q8BGB5	LIM domain-containing protein 2	0.99	0.85
Mcm3	P25206	DNA replication licensing factor MCM3	0.99	0.71
Actn4	P57780	Alpha-actinin-4	0.99	0.82
Eif2s3y	Q9Z0N2	Eukaryotic translation initiation factor 2 subunit 3, Y-linked	0.99	0.73
Rtn4ip1	A0A0R4J0S3	Reticulon 4 interacting protein 1	0.99	0.80
Smyd5	Q3TYX3	SET and MYND domain-containing protein 5	0.99	0.78
Npm1	Q61937	Nucleophosmin	0.99	0.66
Ddx39b	Q9Z1N5	Spliceosome RNA helicase Ddx39b	0.99	0.42
Asna1	O54984	ATPase Asna1	0.99	0.77
Srsf7	Q8BL97	Serine/arginine-rich splicing factor 7	0.99	0.87
Smpdl3a	P70158	Acid sphingomyelinase-like phosphodiesterase 3a	0.99	0.74
Lpar6	Q8BMC0	Lysophosphatidic acid receptor 6	0.99	0.81
Lamtor2	Q9JHS3	Ragulator complex protein LAMTOR2	0.99	0.80
Arsg	Q3TYD4	Arylsulfatase G	0.99	0.88
Snrpg	P62309	Small nuclear ribonucleoprotein G	0.99	0.76
Lsm4	Q9CY46	U6 snRNA-associated Sm-like protein LSm4	0.99	0.84
Phf11	A6H5X4	PHD finger protein 11	0.99	0.87
	Q9D727	Uncharacterized protein C6orf226 homolog	0.99	0.78
Nudt16l1	Q8VHN8	Tudor-interacting repair regulator protein	0.99	0.69
Rubcn	D3Z7B1	Run domain Beclin-1-interacting and cysteine-rich domain-containing protein	0.99	0.83
Wiz	F6ZBR8	Protein Wiz	0.99	0.81
Lxn	P70202	Latexin	0.99	0.78
Pnn	Q3TUQ5	Pinin	0.99	0.73
Crbn	Q8C7D2	Protein cereblon	0.99	0.79
Lrrfip2	A0A0G2JEP4	Leucine-rich repeat flightless-interacting protein 2	0.99	0.66
Il3ra	P26952	Interleukin-3 receptor subunit alpha	0.99	0.89
Ip6k1	Q6PD10	Inositol hexakisphosphate kinase 1	0.99	0.73
Fgfr1op2	Q9CRA9	FGFR1 oncogene partner 2 homolog	0.99	0.92
Rps15	P62843	40S ribosomal protein S15	0.99	0.70
Washc3	Q9CR27	WASH complex subunit 3	0.99	0.70
Cacul1	Q8R0X2	CDK2-associated and cullin domain-containing protein 1	0.99	0.80
Rpl32	P62911	60S ribosomal protein L32	0.99	0.84
Lpxn	Q99N69	Leupaxin	0.99	0.83
Mcfd2	Q8K5B2	Multiple coagulation factor deficiency protein 2 homolog	0.99	0.87
Naa30	E9QMB7	N-alpha-acetyltransferase 30	0.99	0.81
Ptgr1	Q91YR9	Prostaglandin reductase 1	0.99	0.63
Vps37b	Q8R0J7	Vacuolar protein sorting-associated protein 37B	0.99	0.90
Tubb6	Q922F4	Tubulin beta-6 chain	0.99	0.76
Braf	P28028	Serine/threonine-protein kinase B-raf	0.99	0.83
Ccnl1	Q52KE7	Cyclin-L1	0.99	0.86
Paat	Q9D2Q3	ATPase PAAT	0.99	0.90
Ywhab	Q9CQV8	14-3-3 protein beta/alpha	0.99	0.78
Lgals3bp	Q07797	Galectin-3-binding protein	0.99	0.80
Bclaf1	Q8K019	Bcl-2-associated transcription factor 1	0.99	0.82
Nans	Q99J77	Sialic acid synthase	0.99	0.82
Fabp4	P04117	Fatty acid-binding protein, adipocyte	0.99	0.83
Haus3	Q8QZX2	HAUS augmin-like complex subunit 3	0.99	0.86
Ikbkb	O88351	Inhibitor of nuclear factor kappa-B kinase subunit beta	0.99	0.64
Ptbp3	G3UXA6	Polypyrimidine tract-binding protein 3	0.99	0.69
Ist1	Q9CX00	IST1 homolog	0.99	0.72
Eif3f	Q9DCH4	Eukaryotic translation initiation factor 3 subunit F	0.99	0.72

Fam151b	D3YUE4	Family with sequence similarity 151, member B	0.99	0.89
Brd3	Q8K2F0	Bromodomain-containing protein 3	0.99	0.84
Myl12a	Q6ZWQ9	MCG5400	0.99	0.65
Hspe1	Q64433	10 kDa heat shock protein, mitochondrial	0.99	0.80
Dtd2	Q8BHA3	D-aminoacyl-tRNA deacylase 2	0.99	0.73
Psmc5	P62196	26S proteasome regulatory subunit 8	0.99	0.67
Psma2	P49722	Proteasome subunit alpha type-2	0.99	0.79
Cfl1	P18760	Cofilin-1	0.99	0.68
Xiap	Q60989	E3 ubiquitin-protein ligase XIAP	0.99	0.76
Tbca	P48428	Tubulin-specific chaperone A	0.99	0.60
Tpd52	E9PUA7	Tumor protein D52	0.99	0.70
Mon1b	Q8BMQ8	Vacuolar fusion protein MON1 homolog B	0.99	0.88
Uba2	Q9Z1F9	SUMO-activating enzyme subunit 2	0.99	0.62
Prepl	Q8C167	Prolyl endopeptidase-like	0.99	0.84
Tlr2	G3X8Y8	Toll-like receptor 2	0.99	0.86
Plekhf2	Q91WB4	Pleckstrin homology domain-containing family F member 2	0.99	0.67
Rbm22	Q8BHS3	Pre-mRNA-splicing factor RBM22	0.99	0.72
Ap1m1	P35585	AP-1 complex subunit mu-1	0.99	0.74
Sec24d	Q6NXL1	Sec24-related gene family, member D (S. cerevisiae)	0.99	0.79
Thoc5	A0A0R4J0J6	THO complex subunit 5 homolog	0.99	0.78
Acp1	Q9D358	Low molecular weight phosphotyrosine protein phosphatase	0.99	0.68
Sh3glb1	A0A0G2JEC4	Endophilin-B1	0.99	0.74
Plbd2	Q3TCN2	Putative phospholipase B-like 2	0.99	0.72
Fam193a	M0QWZ1	Protein FAM193A	0.99	0.77
Pgk1	P09411	Phosphoglycerate kinase 1	0.99	0.81
Mtr	A6H5Y3	Methionine synthase	0.99	0.88
Tcp1	P11983	T-complex protein 1 subunit alpha	0.99	0.78
Cwc25	Q9DBF7	Pre-mRNA-splicing factor CWC25 homolog	0.99	0.88
Slc36a4	A0A0R4J0Q3	Proton-coupled amino acid transporter 4	0.99	0.81
Cdv3	Q4VAA2	Protein CDV3	0.99	0.75
Tlr3	Q99MB1	Toll-like receptor 3	0.99	0.84
Nsfl1c	Q9CZ44	NSFL1 cofactor p47	0.99	0.76
Snx2	Q9CWK8	Sorting nexin-2	0.99	0.78
Copg1	Q9QZE5	Coatomer subunit gamma-1	0.99	0.74
Snrpc	Q62241	U1 small nuclear ribonucleoprotein C	0.99	0.67
Calu	Q6XLQ8	Calumenin	0.99	0.84
Srsf6	Q3TWW8	Serine/arginine-rich splicing factor 6	0.99	0.75
Bccip	Q9CWI3	BRCA2 and CDKN1A-interacting protein	0.99	0.75
Ap2b1	Q9DBG3	AP-2 complex subunit beta	0.99	0.69
Ogfr	Q99PG2	Opioid growth factor receptor	0.99	0.75
Manf	Q3TMX5	Arginine-rich, mutated in early stage tumors, isoform CRA_b	0.99	0.74
Gps1	G3UXW9	COP9 signalosome complex subunit 1	0.99	0.81
Vps13a	Q5H8C4	Vacuolar protein sorting-associated protein 13A	0.99	0.84
Babam1	Q3UI43	BRISC and BRCA1-A complex member 1	0.99	0.74
Ddx3y	Q62095	ATP-dependent RNA helicase DDX3Y	0.99	0.61
Lactb2	Q99KR3	Endoribonuclease LACTB2	0.99	0.73
Pnkp	G5E8N7	Bifunctional polynucleotide phosphatase/kinase	0.99	0.82
Brd9	A0A0R4J175	Bromodomain-containing protein 9	0.99	0.83
Isca2	Q9DCB8	Iron-sulfur cluster assembly 2 homolog, mitochondrial	0.99	0.90
Zmym3	Q9JLM4	Zinc finger MYM-type protein 3	0.99	0.90
Chil4	Q91Z98	Chitinase-like protein 4	0.99	0.90
Rfc3	Q8R323	Replication factor C subunit 3	0.99	0.86
Cat	P24270	Catalase	0.99	0.72
Etfb	Q9DCW4	Electron transfer flavoprotein subunit beta	0.99	0.80
Polr1c	P52432	DNA-directed RNA polymerases I and III subunit RPAC1	0.99	0.85
Sap18	E9Q317	Histone deacetylase complex subunit SAP18	0.99	0.78
Parp3	Q3ULW8	Poly [ADP-ribose] polymerase	0.99	0.74
Adss	P46664	Adenylosuccinate synthetase isozyme 2	0.99	0.71
Ndufb4	Q9CQC7	NADH dehydrogenase [ubiquinone] 1 beta subcomplex subunit 4	0.99	0.81
Atp5f1b	P56480	ATP synthase subunit beta, mitochondrial	0.99	0.83
Vps29	D3YYD5	Vacuolar protein sorting-associated protein 29 (Fragment)	0.99	0.83
Glrx2	B7ZC40	Glutaredoxin 2 (Thioltransferase), isoform CRA_a	0.99	0.92
Aldh5a1	Q8BWF0	Succinate-semialdehyde dehydrogenase, mitochondrial	0.99	0.73
Sash3	Q8K352	SAM and SH3 domain-containing protein 3	0.99	0.75
Ywhah	P68510	14-3-3 protein eta	0.99	0.72
Hbp1	E9Q1A8	HMG box-containing protein 1	0.99	0.88
Arpc2	Q9CVB6	Actin-related protein 2/3 complex subunit 2	0.99	0.80
Trmt2a	E9PUQ7	tRNA (uracil-5-)-methyltransferase homolog A	0.99	0.78
Ctso	Q8BM88	Cathepsin O	0.99	0.87
Txnl1	Q8CDN6	Thioredoxin-like protein 1	0.99	0.70
Hnrnpm	Q9D0E1	Heterogeneous nuclear ribonucleoprotein M	0.99	0.76
Prkab2	Q6PAM0	5'-AMP-activated protein kinase subunit beta-2	0.99	0.89
Bag5	Q8CI32	BAG family molecular chaperone regulator 5	0.99	0.75

Taldo1	A0A1B0GR11	Transaldolase	0.99	0.61
Rab3il1	Q8VDV3	Guanine nucleotide exchange factor for Rab-3A	0.99	0.78
Kars	Q8R2P8	Lysine--tRNA ligase	0.99	0.77
Prpf31	Q8CCF0	U4/U6 small nuclear ribonucleoprotein Prp31	0.99	0.81
Cdyl	Q9WTK2	Chromodomain Y-like protein	0.99	0.89
B4galt7	Q8R087	Beta-1,4-galactosyltransferase 7	0.99	0.81
Tubgcp4	Q9D4F8	Gamma-tubulin complex component 4	0.99	0.91
Tnfaip3	Q60769	Tumor necrosis factor alpha-induced protein 3	0.99	0.85
Psma4	Q9R1P0	Proteasome subunit alpha type-4	0.99	0.71
Arcn1	Q5XJY5	Coatomer subunit delta	0.99	0.69
Mtnd2	P03893	NADH-ubiquinone oxidoreductase chain 2	0.99	0.90
Rabggtb	P53612	Geranylgeranyl transferase type-2 subunit beta	0.99	0.90
Bag2	Q91YN9	BAG family molecular chaperone regulator 2	0.99	0.86
Glrx5	Q80Y14	Glutaredoxin-related protein 5, mitochondrial	0.99	0.81
Casp7	P97864	Caspase-7	0.99	0.81
Casp6	O08738	Caspase-6	0.99	0.66
Pdxdc1	Q99K01	Pyridoxal-dependent decarboxylase domain-containing protein 1	0.99	0.88
Psmb6	Q60692	Proteasome subunit beta type-6	0.99	0.79
Tpi1	P17751	Triosephosphate isomerase	0.99	0.78
Hist3h2bb	Q8CGP0	Histone H2B type 3-B	0.99	0.96
Caskin2	Q8VHK1	Caskin-2	0.99	0.92
Cyhr1	Q9QXA1	Cysteine and histidine-rich protein 1	0.99	0.81
Atxn7l3b	Q3UD01	Ataxin-7-like protein 3B	0.99	0.87
Oas3	Q8VI93	2'-5'-oligoadenylate synthase 3	0.99	0.82
Foxred1	Q3TQB2	FAD-dependent oxidoreductase domain-containing protein 1	0.99	0.92
Prune1	Q8BIW1	Exopolyphosphatase PRUNE1	0.99	0.65
Tmcc3	Q8R310	Transmembrane and coiled-coil domain protein 3	0.99	0.87
Bcl2l1	Q64373	Bcl-2-like protein 1	0.99	0.77
Selenom	Q8VHC3	Selenoprotein M	0.99	0.94
Ctbp2	P56546	C-terminal-binding protein 2	0.99	0.82
Traf3ip3	G3X949	TRAF3 interacting protein 3	0.99	0.86
Exd2	Q8VEG4	Exonuclease 3'-5' domain-containing protein 2	0.99	0.90
Ubac1	Q8VDI7	Ubiquitin-associated domain-containing protein 1	0.99	0.82
Dyrk1a	Q61214	Dual specificity tyrosine-phosphorylation-regulated kinase 1A	0.99	0.76
Exosc1	Q9DAA6	Exosome complex component CSL4	0.99	0.76
Rnpep	Q8VCT3	Aminopeptidase B	0.99	0.75
Pgm1	Q9D0F9	Phosphoglucomutase-1	0.99	0.79
Nop10	Q9CQS2	H/ACA ribonucleoprotein complex subunit 3	0.99	0.83
Stard5	Q9EPQ7	StAR-related lipid transfer protein 5	0.99	0.73
Isoc1	Q91V64	Isochorismatase domain-containing protein 1	0.99	0.83
Pde8a	O88502	High affinity cAMP-specific and IBMX-insensitive 3',5'-cyclic phosphodiesterase 8A	0.99	0.84
Tnpo1	Q8BFY9	Transportin-1	0.99	0.73
Tfdp1	Q08639	Transcription factor Dp-1	0.99	0.87
Eno1	P17182	Alpha-enolase	0.99	0.80
Ifit2	Q6GTM0	Ifit2 protein	0.99	0.77
Ccdc115	Q8VE99	Coiled-coil domain-containing protein 115	0.99	0.80
Cops8	Q8VBV7	COP9 signalosome complex subunit 8	0.99	0.86
Ipo11	Q8K2V6	Importin-11	0.99	0.80
Commd7	Q8BG94	COMM domain-containing protein 7	0.99	0.76
Sephs1	Q8BH69	Selenide, water dikinase 1	0.99	0.75
Mat2a	Q3THS6	S-adenosylmethionine synthase isoform type-2	0.99	0.76
Pip4k2c	Q91XU3	Phosphatidylinositol 5-phosphate 4-kinase type-2 gamma	0.99	0.77
Rbm6	S4R1W5	RNA-binding motif protein 6	0.99	0.86
Anxa11	P97384	Annexin A11	0.99	0.75
Gapdh	A0A0A0MQF6	Glyceraldehyde-3-phosphate dehydrogenase	0.99	0.77
Rfc2	Q9WUK4	Replication factor C subunit 2	0.99	0.83
Rgs10	Q9CQE5	Regulator of G-protein signaling 10	0.99	0.78
Nat2	P50295	Arylamine N-acetyltransferase 2	0.99	0.90
Clta	Q6PFA2	Clathrin light chain	0.99	0.63
Parp10	Q8CIE4	Poly [ADP-ribose] polymerase	0.99	0.90
Cd9	P40240	CD9 antigen	0.99	0.91
Fuk	Q7TMC8	L-fucose kinase	0.99	0.88
Rbm19	Q8R3C6	Probable RNA-binding protein 19	0.99	0.89
Pcnp	Q6P8I4	PEST proteolytic signal-containing nuclear protein	0.99	0.82
Mrpl42	Q9CPV3	39S ribosomal protein L42, mitochondrial	0.99	0.87
Tpm4	Q6IRU2	Tropomyosin alpha-4 chain	0.99	0.74
N4bp1	Q6A037	NEDD4-binding protein 1	0.99	0.91
Vps13d	B1ART2	Vacuolar protein sorting 13D	0.99	0.91
Psmc6	P62334	26S proteasome regulatory subunit 10B	0.99	0.80
Trim21	Q3U7K7	E3 ubiquitin-protein ligase TRIM21	0.99	0.85
Gab2	Q3ZB57	GRB2-associated-binding protein 2	0.99	0.80
Stard8	Q8K031	StAR-related lipid transfer protein 8	0.99	0.85
Rps3	P62908	40S ribosomal protein S3	0.99	0.86

Arhgap4	Q80Z68	Rho GTPase activating protein 4, isoform CRA_f	0.99	0.82
Abhd4	Q3U7M5	Protein ABHD4	0.99	0.87
Kif3c	O35066	Kinesin-like protein KIF3C	0.99	0.74
Fermt3	Q8K1B8	Fermitin family homolog 3	0.99	0.82
Hspd1	P63038	60 kDa heat shock protein, mitochondrial	0.99	0.82
Usp10	P52479	Ubiquitin carboxyl-terminal hydrolase 10	0.99	0.81
Fip1l1	Q9D824	Pre-mRNA 3'-end-processing factor FIP1	0.99	0.77
Trappc6a	Q78XR0	Trafficking protein particle complex subunit 6A	0.99	0.90
Coq6	Q8R1S0	Ubiquinone biosynthesis monooxygenase COQ6, mitochondrial	0.99	0.93
Aldoa	A6ZI44	Fructose-bisphosphate aldolase	0.99	0.76
Rnaseh1	E9QLN8	Ribonuclease H1	0.99	0.87
Smox	Q99K82	Spermine oxidase	0.99	0.93
Ppia	P17742	Peptidyl-prolyl cis-trans isomerase A	0.99	0.75
Tal1	P22091	T-cell acute lymphocytic leukemia protein 1 homolog	0.99	0.86
Gpam	Q61586	Glycerol-3-phosphate acyltransferase 1, mitochondrial	0.99	0.88
Sphk2	Q9JIA7	Sphingosine kinase 2	0.99	0.80
As3mt	Q91WU5	Arsenite methyltransferase	0.99	0.84
Zcchc24	B2RVL6	Zinc finger CCHC domain-containing protein 24	0.99	0.85
Stard4	Q80SX0	StAR-related lipid transfer protein 4	0.99	0.93
Cops4	O88544	COP9 signalosome complex subunit 4	0.99	0.80
Ptrh1	Q8BW00	Probable peptidyl-tRNA hydrolase	0.99	0.93
Psmc3	O88685	26S proteasome regulatory subunit 6A	0.99	0.81
Map2k1	P31938	Dual specificity mitogen-activated protein kinase kinase 1	0.99	0.70
Gsto1	O09131	Glutathione S-transferase omega-1	0.99	0.80
Yif1a	Q91XB7	Protein YIF1A	0.99	0.74
Natd1	Q9DBW3	Protein NATD1	0.99	0.85
Ddx19a	Q61655	ATP-dependent RNA helicase DDX19A	0.99	0.69
Fam49b	Q921M7	Protein FAM49B	0.99	0.78
Rgs1	Q9JL25	Regulator of G-protein signaling 1	0.99	0.84
Ubp1	Q811S7	Upstream-binding protein 1	0.99	0.88
Pygb	Q8CI94	Glycogen phosphorylase, brain form	0.99	0.75
Rmnd1	Q8CI78	Required for meiotic nuclear division protein 1 homolog	0.99	0.89
Osgep	A0A0R4J1Y3	Probable tRNA N6-adenosine threonylcarbamoyltransferase	0.99	0.85
Ssu72	Q9CY97	RNA polymerase II subunit A C-terminal domain phosphatase SSU72	0.99	0.83
Bst1	A0A0R4J190	ADP-ribosyl cyclase/cyclic ADP-ribose hydrolase 2	0.99	0.81
Vwa5a	Q99KC8	von Willebrand factor A domain-containing protein 5A	0.99	0.76
Matr3	Q8K310	Matrin-3	0.99	0.74
Dbi	Q4VWZ5	Acyl-CoA-binding protein	0.99	0.89
Atp6v1b2	P62814	V-type proton ATPase subunit B, brain isoform	0.99	0.86
Samhd1	Q60710	Deoxynucleoside triphosphate triphosphohydrolase SAMHD1	0.99	0.77
Vav1	P27870	Proto-oncogene vav	0.99	0.77
Rps27a	P62983	Ubiquitin-40S ribosomal protein S27a	0.99	0.84
Fli1	P26323	Friend leukemia integration 1 transcription factor	0.99	0.84
Ncor2	F8VQL9	Nuclear receptor corepressor 2	0.99	0.94
Rfk	Q8CFV9	Riboflavin kinase	0.99	0.73
Lpin2	E9PWN0	Phosphatidate phosphatase LPIN2	0.99	0.90
Uba7	Q9DBK7	MCG18845, isoform CRA_d	0.99	0.67
Adcy9	P51830	Adenylate cyclase type 9	0.99	0.89
Etv3	Q8R4Z4	ETS translocation variant 3	0.99	0.89
Ctbs	Q8R242	Di-N-acetylchitobiase	0.99	0.88
Pdhx	Q8BKZ9	Pyruvate dehydrogenase protein X component, mitochondrial	0.99	0.82
Telo2	Q9DC40	Telomere length regulation protein TEL2 homolog	0.99	0.91
Coq5	Q9CXI0	2-methoxy-6-polyprenyl-1,4-benzoquinol methylase, mitochondrial	0.99	0.89
Ethe1	Q9DCM0	Persulfide dioxygenase ETHE1, mitochondrial	0.99	0.73
Dhx8	A2A4P0	ATP-dependent RNA helicase DHX8	0.99	0.89
Plbd1	A0A0R4J0B2	Phospholipase B-like	0.99	0.88
Plk1	Q07832	Serine/threonine-protein kinase PLK1	0.99	0.89
Rps21	Q9CQR2	40S ribosomal protein S21	0.99	0.84
Lrch1	P62046	Leucine-rich repeat and calponin homology domain-containing protein 1	0.99	0.89
Ripk3	Q9QZL0	Receptor-interacting serine/threonine-protein kinase 3	0.99	0.84
Septin2	P42208	Septin-2	0.99	0.70
Lap3	Q9CPY7	Cytosol aminopeptidase	0.99	0.86
Qsox2	Q3TMX7	Sulfhydryl oxidase 2	0.99	0.88
Srp68	Q8BMA6	Signal recognition particle subunit SRP68	0.99	0.78
Eif2s3x	Q9Z0N1	Eukaryotic translation initiation factor 2 subunit 3, X-linked	0.99	0.72
Nfu1	A0A0N4SUH8	NFU1 iron-sulfur cluster scaffold homolog, mitochondrial	0.99	0.91
Coil	E9Q284	Coilin	0.99	0.86
Lgals8	Q9JL15	Galectin-8	0.99	0.83
Tubgcp3	P58854	Gamma-tubulin complex component 3	0.99	0.91
Ptpre	A0A1B0GRT6	Receptor-type tyrosine-protein phosphatase	0.99	0.86
Coro7	Q9D2V7	Coronin-7	0.99	0.75
Wdtc1	Q80ZK9	WD and tetratricopeptide repeats protein 1	0.99	0.83
Tlk1	Q8C0V0	Serine/threonine-protein kinase tousled-like 1	0.99	0.81

Sympk	F8WJD4	Symplekin	0.99	0.85
Map2k3	O09110	Dual specificity mitogen-activated protein kinase kinase 3	0.99	0.81
Ube2s	A0A0U1RQ37	MCG20927, isoform CRA_b	0.99	0.91
Stxbp2	Q64324	Syntaxin-binding protein 2	0.99	0.78
Coq9	Q8K1Z0	Ubiquinone biosynthesis protein COQ9, mitochondrial	0.99	0.90
Acp1	Q561M1	Acp1 protein	0.99	0.94
Mvp	E9Q3X0	Major vault protein	0.99	0.79
Cstf1	Q99LC2	Cleavage stimulation factor subunit 1	0.99	0.88
Ube2k	P61087	Ubiquitin-conjugating enzyme E2 K	0.99	0.78
	P10404	MLV-related proviral Env polyprotein	0.99	0.82
Ints14	Q8R3P6	Integrator complex subunit 14	0.99	0.84
Fam126b	Q8C729	Protein FAM126B	0.99	0.93
Snrpn	P63163	Small nuclear ribonucleoprotein-associated protein N	0.99	0.82
Pira2	F8VQ94	Paired-Ig-like receptor A2	0.99	0.92
Psmd10	Q9Z2X2	26S proteasome non-ATPase regulatory subunit 10	0.99	0.82
Prpf40a	Q9R1C7	Pre-mRNA-processing factor 40 homolog A	0.99	0.76
Camsap2	H7BX08	Calmodulin-regulated spectrin-associated protein 2	0.99	0.87
Tgs1	Q923W1	Trimethylguanosine synthase	0.99	0.84
Nubp1	Q9R060	Cytosolic Fe-S cluster assembly factor NUBP1	0.99	0.83
Anxa7	A0A2C9F2D2	Annexin	0.99	0.85
Ntan1	Q64311	Protein N-terminal asparagine amidohydrolase	0.99	0.91
Slc25a30	Q9CR58	Kidney mitochondrial carrier protein 1	0.99	0.86
Nudt3	Q9JI46	Diphosphoinositol polyphosphate phosphohydrolase 1	0.99	0.71
Dnaja1	P63037	DnaJ homolog subfamily A member 1	0.99	0.84
Tmem259	Q8CIV2	Membralin	0.99	0.94
Qrich1	G3X8R5	Glutamine-rich protein 1	0.99	0.84
Srsf1	H7BX95	Serine/arginine-rich-splicing factor 1	0.99	0.84
Frmd8	Q3UFK8	FERM domain-containing protein 8	0.99	0.87
Pak2	Q8CIN4	Serine/threonine-protein kinase PAK 2	0.99	0.80
Capns1	O88456	Calpain small subunit 1	0.99	0.85
Taf4	E9QAP7	TATA-box-binding protein-associated factor 4	0.99	0.89
Chmp4b	Q9D8B3	Charged multivesicular body protein 4b	0.99	0.80
Stat2	E9QJX9	Signal transducer and activator of transcription	0.99	0.88
Lrrc40	A0A0R4J0W6	Leucine rich repeat containing 40, isoform CRA_a	0.99	0.85
Park7	Q99LX0	Protein/nucleic acid deglycase DJ-1	0.99	0.86
Fnip2	D3YUC5	Folliculin-interacting protein 2	0.99	0.93
	Q3TQI7	Splicing factor C9orf78 homolog	0.99	0.90
Qpctl	Q8BH73	Glutaminyl-peptide cyclotransferase-like protein	0.99	0.93
Ahcy	P50247	Adenosylhomocysteinase	0.99	0.85
Lamtor3	O88653	Ragulator complex protein LAMTOR3	0.99	0.93
Bnip2	O54940	BCL2/adenovirus E1B 19 kDa protein-interacting protein 2	0.99	0.83
Mocos	Q14CH1	Molybdenum cofactor sulfurase	0.99	0.85
Lbh	Q9CX60	Protein LBH	0.99	0.88
Insr	P15208	Insulin receptor	0.99	0.86
Arl1	P61211	ADP-ribosylation factor-like protein 1	0.99	0.74
Itih4	A6X935	Inter alpha-trypsin inhibitor, heavy chain 4	0.99	0.96
Ahcyl1	Q80SW1	S-adenosylhomocysteine hydrolase-like protein 1	0.99	0.82
Tmco3	Q8BH01	Transmembrane and coiled-coil domain-containing protein 3	0.99	0.91
Uqcrc1	Q9CZ13	Cytochrome b-c1 complex subunit 1, mitochondrial	0.99	0.87
Rbl2	Q64700	Retinoblastoma-like protein 2	0.99	0.93
Rsu1	Q9D031	Ras suppressor protein 1	0.99	0.81
Nlrc4	Q3UP24	NLR family CARD domain-containing protein 4	0.99	0.88
Neurl3	Q8CJC5	E3 ubiquitin-protein ligase NEURL3	0.99	0.93
Rpap1	Q80TE0	RNA polymerase II-associated protein 1	0.99	0.92
Thoc7	Q7TMY4	THO complex subunit 7 homolog	0.99	0.85
Pgam1	Q9DBJ1	Phosphoglycerate mutase 1	0.99	0.92
Sgsh	Q9EQ08	Heparan N-sulfatase	0.99	0.91
Rnf126	Q91YL2	E3 ubiquitin-protein ligase RNF126	0.99	0.87
Trio	Q0KL02	Triple functional domain protein	0.99	0.87
Eif4a2	P10630	Eukaryotic initiation factor 4A-II	0.99	0.88
Siae	P70665	Sialate O-acetylesterase	0.99	0.91
Pcif1	P59114	Phosphorylated CTD-interacting factor 1	0.99	0.92
U2af1	Q9D883	Splicing factor U2AF 35 kDa subunit	0.99	0.88
Ctc1	E0CXE7	CST complex subunit CTC1	0.99	0.95
Ssna1	Q9JJ94	Sjoegren syndrome nuclear autoantigen 1 homolog	0.99	0.89
Gnb2	E9QKR0	Guanine nucleotide-binding protein G(I)/G(S)/G(T) subunit beta-2	0.99	0.84
Ddx5	Q8BTS0	DEAD (Asp-Glu-Ala-Asp) box polypeptide 5	0.99	0.77
Abr	Q5SSL4	Active breakpoint cluster region-related protein	0.99	0.81
Uchl5	Q9WUP7	Ubiquitin carboxyl-terminal hydrolase isozyme L5	0.99	0.76
Cops6	O88545	COP9 signalosome complex subunit 6	0.99	0.87
Pgrmc1	O55022	Membrane-associated progesterone receptor component 1	0.99	0.87
Mtmr3	Q8K296	Myotubularin-related protein 3	0.99	0.87
Gatd1	Q8BFQ8	Glutamine amidotransferase-like class 1 domain-containing protein 1	0.99	0.84

Aimp2	Q8R010	Aminoacyl tRNA synthase complex-interacting multifunctional protein 2	0.99	0.88
Retreg3	Q9CQV4	Reticulophagy regulator 3	0.99	0.86
Snx1	Q6NZD2	Sorting nexin-1	0.99	0.76
Zfp217	Q3U0X6	Zinc finger protein 217	0.99	0.87
Caprin1	Q60865	Caprin-1	0.99	0.87
Pum1	Q80U78	Pumilio homolog 1	0.99	0.78
Spryd4	Q91WK1	SPRY domain-containing protein 4	0.99	0.79
5730455P16Rik	J3KMQ6	RIKEN cDNA 5730455P16 gene	0.99	0.86
Uqcrq	Q9CQ69	Cytochrome b-c1 complex subunit 8	0.99	0.95
Dap	Q91XC8	Death-associated protein 1	0.99	0.90
Dnpep	Q3TVK3	Aspartyl aminopeptidase	0.99	0.81
Sephs2	P97364	Selenide, water dikinase 2	0.99	0.92
Paf1	Q8K2T8	RNA polymerase II-associated factor 1 homolog	0.99	0.84
Washc2	Q6PGL7	WASH complex subunit 2	0.99	0.88
Prmt6	Q6NZB1	Protein arginine N-methyltransferase 6	0.99	0.90
Dnaaf5	B9EJR8	Dynein assembly factor 5, axonemal	0.99	0.90
Nono	Q99K48	Non-POU domain-containing octamer-binding protein	0.99	0.87
Arsa	P50428	Arylsulfatase A	0.99	0.81
Msh2	P43247	DNA mismatch repair protein Msh2	0.99	0.85
Apobec1	P51908	C->U-editing enzyme APOBEC-1	0.99	0.85
Nbdy	A0A0N4SUI7	Negative regulator of P-body association	0.99	0.90
Parg	O88622	Poly(ADP-ribose) glycohydrolase	0.99	0.93
Kmt2d	A0A0A0MQ73	Histone-lysine N-methyltransferase 2D	0.99	0.95
Gm11273	Q9D881	Cytochrome c oxidase subunit 5B, mitochondrial	0.99	0.80
Pacsin2	Q9WVE8	Protein kinase C and casein kinase substrate in neurons protein 2	0.99	0.84
Polr1d	Q9D1M1	DNA-directed RNA polymerases I and III subunit RPAC2	0.99	0.89
Ybx1	P62960	Nuclease-sensitive element-binding protein 1	0.99	0.96
Pkn1	P70268	Serine/threonine-protein kinase N1	0.99	0.86
Qki	Q9QYS9	Protein quaking	0.99	0.85
C1qbp	Q8R5L1	Complement component 1 Q subcomponent-binding protein, mitochondrial	0.99	0.82
Cnot11	Q9CWN7	CCR4-NOT transcription complex subunit 11	0.99	0.92
Tmem106b	Q80X71	Transmembrane protein 106B	0.99	0.79
Hikeshi	Q9DD02	Protein Hikeshi	0.99	0.86
Fahd2a	A0A0R4J094	Fumarylacetoacetate hydrolase domain-containing 2A	0.99	0.86
Nenf	Q9CQ45	Neudesin	0.99	0.87
Coro1c	Q9WUM4	Coronin-1C	0.99	0.83
Rnh1	A0A1B0GSG5	Ribonuclease inhibitor	0.99	0.83
Polr1b	P70700	DNA-directed RNA polymerase I subunit RPA2	1.00	0.89
Zdhhc5	Q8VDZ4	Palmitoyltransferase ZDHHC5	1.00	0.91
Psmb1	O09061	Proteasome subunit beta type-1	1.00	0.96
Taco1	Q8K0Z7	Translational activator of cytochrome c oxidase 1	1.00	0.92
Txnl4b	Q8BUH1	Thioredoxin-like protein 4B	1.00	0.87
Psmg3	Q9CZH3	Proteasome assembly chaperone 3	1.00	0.75
Tlk2	B1ASU9	Serine/threonine-protein kinase tousled-like 2	1.00	0.87
Dap3	G3X9M0	28S ribosomal protein S29, mitochondrial	1.00	0.85
Oxct1	Q9D0K2	Succinyl-CoA:3-ketoacid coenzyme A transferase 1, mitochondrial	1.00	0.88
Prkdc	P97313	DNA-dependent protein kinase catalytic subunit	1.00	0.89
Itsn1	E9Q3I4	Intersectin-1	1.00	0.86
Oasl1	Q8VI94	2'-5'-oligoadenylate synthase-like protein 1	1.00	0.94
Stambp	Q9CQ26	STAM-binding protein	1.00	0.88
Pcp4l1	Q6W8Q3	Purkinje cell protein 4-like protein 1	1.00	0.94
Ap5b1	Q3TAP4	AP-5 complex subunit beta-1	1.00	0.90
Ugp2	Q91ZJ5	UTP--glucose-1-phosphate uridylyltransferase	1.00	0.82
Rin2	Q9D684	Ras and Rab interactor 2	1.00	0.87
Arhgap6	O54834	Rho GTPase-activating protein 6	1.00	0.93
Oxsm	Q9D404	3-oxoacyl-[acyl-carrier-protein] synthase, mitochondrial	1.00	0.89
Tpm1	G5E8R1	Tropomyosin 1, alpha, isoform CRA_j	1.00	0.86
Galns	Q571E4	N-acetylgalactosamine-6-sulfatase	1.00	0.87
Irf2bp2	E9Q1P8	Interferon regulatory factor 2-binding protein 2	1.00	0.91
Try10	Q792Z1	MCG140784	1.00	0.91
Epn1	Q80VP1	Epsin-1	1.00	0.87
Dusp28	Q8BTR5	Dual specificity phosphatase 28	1.00	0.91
Ing3	Q8VEK6	Inhibitor of growth protein 3	1.00	0.95
Impdh1	F7DEU6	Inosine-5'-monophosphate dehydrogenase	1.00	0.88
Pmvk	Q9D1G2	Phosphomevalonate kinase	1.00	0.91
Blvrb	Q923D2	Flavin reductase (NADPH)	1.00	0.84
Tsc2	Q3UHB2	Tuberin	1.00	0.88
Cdk17	Q8K0D0	Cyclin-dependent kinase 17	1.00	0.89
Atf7	Q3TZR9	Activating transcription factor 7, isoform CRA_a	1.00	0.89
Fis1	Q9CQ92	Mitochondrial fission 1 protein	1.00	0.88
Pgm2l1	Q8CAA7	Glucose 1,6-bisphosphate synthase	1.00	0.88
Psma1	Q9R1P4	Proteasome subunit alpha type-1	1.00	0.81
Polr2i	P60898	DNA-directed RNA polymerase II subunit RPB9	1.00	0.82

Pfas	Q5SUR0	Phosphoribosylformylglycinamidine synthase	1.00	0.89
Dlst	Q9D2G2	Dihydrolipoyllysine-residue succinyltransferase component of 2-oxoglutarate dehydrogenase complex, mitochondria	1.00	0.85
Ada	P03958	Adenosine deaminase	1.00	0.87
Ncor1	Q5RIM6	Nuclear receptor co-repressor 1, isoform CRA_a	1.00	0.94
Ctsa	G3X8T3	Carboxypeptidase	1.00	0.94
Gm10334	Q792Y8	MCG15081	1.00	0.88
Psme2	P97372	Proteasome activator complex subunit 2	1.00	0.85
Tmem9b	Q9JJR8	Transmembrane protein 9B	1.00	0.88
Sdcbp	O08992	Syntenin-1	1.00	0.93
Vars2	Q3U2A8	Valine--tRNA ligase, mitochondrial	1.00	0.93
Mapk1	P63085	Mitogen-activated protein kinase 1	1.00	0.84
Ube2r2	Q6ZWZ2	Ubiquitin-conjugating enzyme E2 R2	1.00	0.86
Rab19	P35294	Ras-related protein Rab-19	1.00	0.91
Mtfr1l	Q9CWE0	Mitochondrial fission regulator 1-like	1.00	0.94
Hspa2	P17156	Heat shock-related 70 kDa protein 2	1.00	0.88
Abce1	P61222	ATP-binding cassette sub-family E member 1	1.00	0.83
Cox6b1	P56391	Cytochrome c oxidase subunit 6B1	1.00	0.90
Psmd3	P14685	26S proteasome non-ATPase regulatory subunit 3	1.00	0.85
Dusp12	Q9D0T2	Dual specificity protein phosphatase 12	1.00	0.92
Abhd10	Q6PE15	Mycophenolic acid acyl-glucuronide esterase, mitochondrial	1.00	0.91
Fads1	Q920L1	Fatty acid desaturase 1	1.00	0.91
Sdhaf2	Q8C6I2	Succinate dehydrogenase assembly factor 2, mitochondrial	1.00	0.96
Herc4	Q6PAV2	Probable E3 ubiquitin-protein ligase HERC4	1.00	0.86
Tor1aip2	Q9ER81	Torsin-1A-interacting protein 2, isoform IFRG15	1.00	0.95
Mrpl10	Q3TBW2	39S ribosomal protein L10, mitochondrial	1.00	0.91
Apbb1ip	Q8R5A3	Amyloid beta A4 precursor protein-binding family B member 1-interacting protein	1.00	0.88
Gnpda1	O88958	Glucosamine-6-phosphate isomerase 1	1.00	0.84
Nlrp1a	Q2LKU9	NACHT, LRR and PYD domains-containing protein 1a	1.00	0.90
Mettl26	Q9DCS2	Methyltransferase-like 26	1.00	0.86
Nbas	E9Q411	Neuroblastoma-amplified sequence	1.00	0.92
Rab14	Q91V41	Ras-related protein Rab-14	1.00	0.88
G3bp1	P97855	Ras GTPase-activating protein-binding protein 1	1.00	0.81
Nop9	Q8BMC4	Nucleolar protein 9	1.00	0.90
Arl6ip1	Q9JKW0	ADP-ribosylation factor-like protein 6-interacting protein 1	1.00	0.92
Aars2	Q14CH7	Alanine--tRNA ligase, mitochondrial	1.00	0.92
Tsta3	P23591	GDP-L-fucose synthase	1.00	0.87
Dok2	O70469	Docking protein 2	1.00	0.90
Bbx	Q8VBW5	HMG box transcription factor BBX	1.00	0.95
Sf3b4	Q8QZY9	Splicing factor 3B subunit 4	1.00	0.89
Acox1	Q9R0H0	Peroxisomal acyl-coenzyme A oxidase 1	1.00	0.87
Akr1c13	Q8VC28	Aldo-keto reductase family 1 member C13	1.00	0.88
Ctu2	Q3U308	Cytoplasmic tRNA 2-thiolation protein 2	1.00	0.94
Paip2	Q9D6V8	Polyadenylate-binding protein-interacting protein 2	1.00	0.89
Cdk9	Q99J95	Cyclin-dependent kinase 9	1.00	0.88
Dctn2	Q99KJ8	Dynactin subunit 2	1.00	0.89
Parvg	E9PYG5	Gamma-parvin	1.00	0.89
Pafah1b3	Q61205	Platelet-activating factor acetylhydrolase IB subunit gamma	1.00	0.80
Lrrc14	Q8VC16	Leucine-rich repeat-containing protein 14	1.00	0.92
Pdia3	P27773	Protein disulfide-isomerase A3	1.00	0.87
Rbbp7	Q60973	Histone-binding protein RBBP7	1.00	0.87
Hbs1l	L7N209	HBS1-like protein	1.00	0.93
Btf3	Q64152	Transcription factor BTF3	1.00	0.87
Acot7	E9PYH2	Cytosolic acyl coenzyme A thioester hydrolase	1.00	0.90
Sh3gl1	Q62419	Endophilin-A2	1.00	0.90
Gys1	Q9Z1E4	Glycogen [starch] synthase, muscle	1.00	0.89
Glud1	P26443	Glutamate dehydrogenase 1, mitochondrial	1.00	0.88
Wdr48	Q8BH57	WD repeat-containing protein 48	1.00	0.90
Eif3d	O70194	Eukaryotic translation initiation factor 3 subunit D	1.00	0.85
Imp3	Q921Y2	U3 small nucleolar ribonucleoprotein protein IMP3	1.00	0.96
Ptpn6	P29351	Tyrosine-protein phosphatase non-receptor type 6	1.00	0.84
Zc3h13	A0A2I3BPQ5	Zinc finger CCCH domain-containing protein 13	1.00	0.94
Crat	P47934	Carnitine O-acetyltransferase	1.00	0.88
2210016F16Rik	G3X8U3	Queuosine salvage protein	1.00	0.89
Srp14	P16254	Signal recognition particle 14 kDa protein	1.00	0.87
Tigar	Q8BZA9	Fructose-2,6-bisphosphatase TIGAR	1.00	0.83
Cherp	A0A1D5RL92	Calcium homeostasis endoplasmic reticulum protein	1.00	0.88
Polr2c	P97760	DNA-directed RNA polymerase II subunit RPB3	1.00	0.84
Capzb	A2AMW0	Capping protein (Actin filament) muscle Z-line, beta, isoform CRA_a	1.00	0.84
Pla2g15	Q8VEB4	Group XV phospholipase A2	1.00	0.77
Wdr59	D3Z1F9	WD repeat-containing protein 59	1.00	0.95
Usp48	Q3V0C5	Ubiquitin carboxyl-terminal hydrolase 48	1.00	0.87
Hpse	Q6YGZ1	Heparanase	1.00	0.90
Dars	Q922B2	Aspartate--tRNA ligase, cytoplasmic	1.00	0.85

Mycbp	Q9EQS3	c-Myc-binding protein	1.00	0.94
Rnf114	Q9ET26	E3 ubiquitin-protein ligase RNF114	1.00	0.94
Eif2s1	Q6ZWX6	Eukaryotic translation initiation factor 2 subunit 1	1.00	0.86
Serpinb9	O08797	SPI6	1.00	0.88
Anp32b	Q9EST5	Acidic leucine-rich nuclear phosphoprotein 32 family member B	1.00	0.87
Tatdn1	Q6P8M1	Putative deoxyribonuclease TATDN1	1.00	0.92
Cdk2	P97377	Cyclin-dependent kinase 2	1.00	0.89
Kansl2	Q8BQR4	KAT8 regulatory NSL complex subunit 2	1.00	0.93
Capn1	O35350	Calpain-1 catalytic subunit	1.00	0.80
Wdr36	Q3TAQ9	WD repeat domain 36	1.00	0.93
Sik2	F8VPT7	Serine/threonine-protein kinase SIK2	1.00	0.93
Sf3b3	Q921M3	Splicing factor 3B subunit 3	1.00	0.86
Anp32a	O35381	Acidic leucine-rich nuclear phosphoprotein 32 family member A	1.00	0.86
Pin4	Q9CWW6	Peptidyl-prolyl cis-trans isomerase NIMA-interacting 4	1.00	0.85
Yy1	Q00899	Transcriptional repressor protein YY1	1.00	0.88
Pcbp2	Q61990	Poly(rC)-binding protein 2	1.00	0.87
Arl3	Q9WUL7	ADP-ribosylation factor-like protein 3	1.00	0.89
Pspc1	Q8R326	Paraspeckle component 1	1.00	0.87
Aarsd1	Q3THG9	Alanyl-tRNA editing protein Aarsd1	1.00	0.91
Acly	Q3V117	ATP-citrate synthase	1.00	0.82
Tnrc6a	Q3UHK8	Trinucleotide repeat-containing gene 6A protein	1.00	0.92
Psmd4	O35226	26S proteasome non-ATPase regulatory subunit 4	1.00	0.90
Cd3eap	Q76KJ5	DNA-directed RNA polymerase I subunit RPA34	1.00	0.89
Rack1	P68040	Receptor of activated protein C kinase 1	1.00	0.83
Pdcl	Q9DBX2	Phosducin-like protein	1.00	0.94
Scp2	P32020	Non-specific lipid-transfer protein	1.00	0.90
Unc45a	Q99KD5	Protein unc-45 homolog A	1.00	0.92
Wipi2	Q80W47	WD repeat domain phosphoinositide-interacting protein 2	1.00	0.91
Tubb2b	Q9CWF2	Tubulin beta-2B chain	1.00	0.98
Celf2	E9QA47	CUGBP Elav-like family member 2	1.00	0.85
Ddb1	Q3U1J4	DNA damage-binding protein 1	1.00	0.86
Unc13d	B2RUP2	Protein unc-13 homolog D	1.00	0.92
Hcls1	P49710	Hematopoietic lineage cell-specific protein	1.00	0.93
Kpna3	O35344	Importin subunit alpha-4	1.00	0.81
Dock2	Q8C3J5	Dedicator of cytokinesis protein 2	1.00	0.86
Lims1	E9QP62	LIM and senescent cell antigen-like-containing domain protein	1.00	0.89
Thoc6	Q5U4D9	THO complex subunit 6 homolog	1.00	0.90
Umps	P13439	Uridine 5'-monophosphate synthase	1.00	0.89
Rps27l	Q6ZWY3	40S ribosomal protein S27-like	1.00	0.99
Itch	Q8C863	E3 ubiquitin-protein ligase Itchy	1.00	0.90
Ndufab1	Q9CR21	Acyl carrier protein, mitochondrial	1.00	0.95
Nanp	Q9CPT3	N-acylneuraminate-9-phosphatase	1.00	0.93
Crls1	Q80ZM8	Cardiolipin synthase (CMP-forming)	1.00	0.89
Herc1	E9PZP8	HECT and RLD domain-containing E3 ubiquitin protein ligase family member 1	1.00	0.95
Rbm47	Q91WT8	RNA-binding protein 47	1.00	0.85
Homer3	Q99JP6	Homer protein homolog 3	1.00	0.92
Skap2	Q3UND0	Src kinase-associated phosphoprotein 2	1.00	0.91
Luc7l2	Q7TNC4	Putative RNA-binding protein Luc7-like 2	1.00	0.89
Txn	P10639	Thioredoxin	1.00	0.66
Mapk14	P47811	Mitogen-activated protein kinase 14	1.00	0.90
Pwwp2b	E9Q9M8	PWWP domain-containing 2B	1.00	0.93
Tfe3	A2AEW1	Transcription factor E3	1.00	0.90
Esd	H3BKH6	S-formylglutathione hydrolase	1.00	0.96
Tln1	P26039	Talin-1	1.00	0.88
Creg1	O88668	Protein CREG1	1.00	0.94
Thyn1	Q91YJ3	Thymocyte nuclear protein 1	1.00	0.91
Slc9a3r1	P70441	Na(+)/H(+) exchange regulatory cofactor NHE-RF1	1.00	0.73
Mast3	E9Q8S5	Microtubule-associated serine/threonine-protein kinase 3	1.00	0.96
Aven	Q9D9K3	Cell death regulator Aven	1.00	0.93
Guk1	Q564G0	Guanylate kinase	1.00	0.92
Ddx41	A0A1S6GWJ4	DEAD (Asp-Glu-Ala-Asp) box polypeptide 41	1.00	0.93
Naga	Q9QWR8	Alpha-N-acetylgalactosaminidase	1.00	0.88
Prep	Q9QUR6	Prolyl endopeptidase	1.00	0.93
Sdha	Q8K2B3	Succinate dehydrogenase [ubiquinone] flavoprotein subunit, mitochondrial	1.00	0.90
Babam2	Q8K3W0	BRISC and BRCA1-A complex member 2	1.00	0.87
Nt5c3a	Q9D020	Cytosolic 5'-nucleotidase 3A	1.00	0.85
Mto1	G5E889	Mitochondrial translation optimization 1 homolog (S. cerevisiae), isoform CRA_b	1.00	0.95
Cct8	P42932	T-complex protein 1 subunit theta	1.00	0.89
Akr1b1	P45376	Aldose reductase	1.00	0.87
Hcfc1	Q61191	Host cell factor 1	1.00	0.89
Trdmt1	O55055	tRNA (cytosine(38)-C(5))-methyltransferase	1.00	0.94
Aprt	P08030	Adenine phosphoribosyltransferase	1.00	0.91
Slc23a2	Q9EPR4	Solute carrier family 23 member 2	1.00	0.93

Trerf1	Q8BXJ2	Transcriptional-regulating factor 1	1.00	0.92
Plrg1	Q922V4	Pleiotropic regulator 1	1.00	0.88
Rpl9	P51410	60S ribosomal protein L9	1.00	0.90
Pola1	P33609	DNA polymerase alpha catalytic subunit	1.00	0.91
Ipo4	Q8VI75	Importin-4	1.00	0.92
Slc28a2	O88627	Sodium/nucleoside cotransporter 2	1.00	0.96
Ca13	Q9D6N1	Carbonic anhydrase 13	1.00	0.84
Pepd	Q11136	Xaa-Pro dipeptidase	1.00	0.92
Mtap	Q9CQ65	S-methyl-5'-thioadenosine phosphorylase	1.00	0.92
Golt1b	Q9CR60	Vesicle transport protein GOT1B	1.00	0.95
Psmb10	O35955	Proteasome subunit beta type-10	1.00	0.93
Tmem141	A2AJB2	Transmembrane protein 141	1.00	0.96
Becn1	O88597	Beclin-1	1.00	0.91
Trmt11	E9QKG3	tRNA (guanine(10)-N2)-methyltransferase homolog	1.00	0.95
Rnaseh2c	Q9CQ18	Ribonuclease H2 subunit C	1.00	0.93
Sars	P26638	Serine--tRNA ligase, cytoplasmic	1.00	0.93
Gga3	Q8BMI3	ADP-ribosylation factor-binding protein GGA3	1.00	0.87
Ppwd1	Q8CEC6	Peptidylprolyl isomerase domain and WD repeat-containing protein 1	1.00	0.95
Tomm34	Q9CYG7	Mitochondrial import receptor subunit TOM34	1.00	0.88
Ahcyl2	F8WGT1	Adenosylhomocysteinase	1.00	0.87
Spen	Q62504	Msx2-interacting protein	1.00	0.93
Jmjd6	Q9ERI5	Bifunctional arginine demethylase and lysyl-hydroxylase JMJD6	1.00	0.93
Wdr18	Q4VBE8	WD repeat-containing protein 18	1.00	0.93
Hexb	P20060	Beta-hexosaminidase subunit beta	1.00	0.88
Ssh3	Q8K330	Protein phosphatase Slingshot homolog 3	1.00	0.95
Cltb	Q6IRU5	Clathrin light chain B	1.00	0.85
Rnf31	Q924T7	E3 ubiquitin-protein ligase RNF31	1.00	0.94
Ubr5	A0A2I3BQS6	E3 ubiquitin-protein ligase UBR5	1.00	0.94
Mdn1	A2ANY6	Midasin	1.00	0.94
R3hcc1	E9PUE1	R3H and coiled-coil domain-containing protein 1	1.00	0.96
Arl4c	P61208	ADP-ribosylation factor-like protein 4C	1.00	0.97
Ube2l3	P68037	Ubiquitin-conjugating enzyme E2 L3	1.00	0.88
Prkcd	P28867	Protein kinase C delta type	1.00	0.88
Mycbp2	Q7TPH6	E3 ubiquitin-protein ligase MYCBP2	1.00	0.95
Nisch	Q80TM9	Nischarin	1.00	0.90
F2	P19221	Prothrombin	1.00	0.97
Appbp2	Q9DAX9	Amyloid protein-binding protein 2	1.00	0.97
Cmc1	Q9CPZ8	COX assembly mitochondrial protein homolog	1.00	0.90
Casp8	O89110	Caspase-8	1.00	0.91
Dnttip2	Q8R2M2	Deoxynucleotidyltransferase terminal-interacting protein 2	1.00	0.94
Usp40	Q8BWR4	Ubiquitin carboxyl-terminal hydrolase 40	1.00	0.95
Mrtfa	Q8K4J6	Myocardin-related transcription factor A	1.00	0.90
Tdrd3	Q91W18	Tudor domain-containing protein 3	1.00	0.89
Hdgfl2	Q3UMU9	Hepatoma-derived growth factor-related protein 2	1.00	0.94
Cbx1	P83917	Chromobox protein homolog 1	1.00	0.94
Abca9	Q8K449	ATP-binding cassette sub-family A member 9	1.00	0.94
Scoc	Q78YZ6	Short coiled-coil protein	1.00	0.91
Chtf18	A0A0R4J0I4	CTF18, chromosome transmission fidelity factor 18 homolog (S. cerevisiae), isoform CRA_a	1.00	0.97
Rbmx	Q9WV02	RNA-binding motif protein, X chromosome	1.00	0.96
Btbd2	E9PUS2	BTB (POZ) domain-containing 2	1.00	0.96
Dennd2a	Q8C4S8	DENN domain-containing protein 2A	1.00	0.97
Mtmr14	Q8VEL2	Myotubularin-related protein 14	1.00	0.97
Sik3	E9PU87	Serine/threonine-protein kinase SIK3	1.00	0.95
Naf1	E9QJT2	H/ACA ribonucleoprotein complex non-core subunit NAF1	1.00	0.97
Tm7sf3	A0A0R4J0K4	Transmembrane 7 superfamily member 3	1.00	0.97
Gmppa	Q922H4	Mannose-1-phosphate guanyltransferase alpha	1.00	0.88
Scyl1	Q9EQC5	N-terminal kinase-like protein	1.00	0.93
Cpq	Q9WVJ3	Carboxypeptidase Q	1.00	0.84
Ddx39a	Q8VDW0	ATP-dependent RNA helicase DDX39A	1.00	0.88
Fubp1	Q3TUE1	Far upstream element-binding protein 1	1.00	0.84
Acbd6	Q9D061	Acyl-CoA-binding domain-containing protein 6	1.00	0.94
Mms19	Q9D071	MMS19 nucleotide excision repair protein homolog	1.00	0.93
Metap1	Q8BP48	Methionine aminopeptidase 1	1.00	0.91
Nfatc1	B5B2N2	Nuclear factor of activated T-cells c1 isoform IA-IXL	1.00	0.94
Lrp1	A0A0R4J0I9	Low density lipoprotein receptor-related protein 1	1.00	0.90
Timm13	P62075	Mitochondrial import inner membrane translocase subunit Tim13	1.00	0.90
Msrb2	Q78J03	Methionine-R-sulfoxide reductase B2, mitochondrial	1.00	0.96
Pym1	Q8CHP5	Partner of Y14 and mago	1.00	0.90
Nup160	Q9Z0W3	Nuclear pore complex protein Nup160	1.00	0.94
Epb41l2	O70318	Band 4.1-like protein 2	1.00	0.89
Urb2	E9Q7L1	URB2 ribosome biogenesis 2 homolog (S. cerevisiae)	1.00	0.97
Hsp90b1	P08113	Endoplasmin	1.00	0.91
Exosc5	Q9CRA8	Exosome complex component RRP46	1.00	0.93

Farsb	Q9WUA2	Phenylalanine--tRNA ligase beta subunit	1.00	0.89
Isoc2a	P85094	Isochorismatase domain-containing protein 2A	1.00	0.93
Hnrnpc	Q9Z204	Heterogeneous nuclear ribonucleoproteins C1/C2	1.00	0.91
Ccdc127	Q3TC33	Coiled-coil domain-containing protein 127	1.00	0.92
Cap1	P40124	Adenylyl cyclase-associated protein 1	1.00	0.96
B430306N03Rik	Q6QX36	CRKD-binding protein	1.00	0.93
Erp44	Q9D1Q6	Endoplasmic reticulum resident protein 44	1.00	0.93
Ccdc43	E9Q2S9	Coiled-coil domain-containing protein 43	1.00	0.92
Wdr11	G5E8J3	Bromodomain and WD repeat domain containing 2, isoform CRA_a	1.00	0.94
Psmd5	Q8BJY1	26S proteasome non-ATPase regulatory subunit 5	1.00	0.94
Heatr6	Q6P1G0	HEAT repeat-containing protein 6	1.00	0.96
Pde12	Q3TIU4	2',5'-phosphodiesterase 12	1.00	0.95
Wdr20	Q3UWE6	MCG14935, isoform CRA_a	1.00	0.93
Npl	Q9DCJ9	N-acetylneuraminate lyase	1.00	0.93
Ftl1	Q9CPX4	Ferritin	1.00	0.97
Ipo5	Q8BKC5	Importin-5	1.00	0.89
IntS13	Q8QZV7	Integrator complex subunit 13	1.00	0.97
RTRAF	Q9CQE8	RNA transcription, translation and transport factor protein	1.00	0.94
Cog1	Q9Z160	Conserved oligomeric Golgi complex subunit 1	1.00	0.95
Psma3	O70435	Proteasome subunit alpha type-3	1.00	0.92
Spr	Q91XH5	Sepiapterin reductase	1.00	0.93
Eif3a	P23116	Eukaryotic translation initiation factor 3 subunit A	1.00	0.91
Ap3m1	Q9JKC8	AP-3 complex subunit mu-1	1.00	0.92
Arih1	Q9Z1K5	E3 ubiquitin-protein ligase ARIH1	1.00	0.92
Atp6v1e1	P50518	V-type proton ATPase subunit E 1	1.00	0.92
Lipe	E9Q4M2	Hormone-sensitive lipase	1.00	0.96
Purb	O35295	Transcriptional activator protein Pur-beta	1.00	0.93
Ptpn21	G5E8J4	Tyrosine-protein phosphatase non-receptor type	1.00	0.97
Gdf3	Q07104	Growth/differentiation factor 3	1.00	0.96
Prps1l3	G3UXL2	Phosphoribosyl pyrophosphate synthetase 1-like 3	1.00	0.94
Zmynd8	A2A484	Zinc finger, MYND-type-containing 8	1.00	0.95
Camk1d	Q8BW96	Calcium/calmodulin-dependent protein kinase type 1D	1.00	0.93
Tns1	E9Q0S6	Tensin 1	1.00	0.94
Dnm2	G3X9G4	Dynamin-2	1.00	0.91
Srsf5	Q9D8S5	MCG7614, isoform CRA_c	1.00	0.94
Gphn	A0JNY3	Gephyrin	1.00	0.94
Ccdc186	Q8C9S4	Coiled-coil domain-containing protein 186	1.00	0.96
Plpbp	A0A1B0GRP7	Pyridoxal phosphate homeostasis protein (Fragment)	1.00	0.89
Rnf135	Q9CWS1	E3 ubiquitin-protein ligase RNF135	1.00	0.94
Slc30a9	Q5IRJ6	Zinc transporter 9	1.00	0.97
Vav2	Q60992	Guanine nucleotide exchange factor VAV2	1.00	0.95
Wars	P32921	Tryptophan--tRNA ligase, cytoplasmic	1.00	0.94
Stk4	Q9JI11	Serine/threonine-protein kinase 4	1.00	0.92
Csde1	Q91W50	Cold shock domain-containing protein E1	1.00	0.91
Aga	Q64191	N(4)-(beta-N-acetylglucosaminyl)-L-asparaginase	1.00	0.97
Sipa1l1	Q8C0T5	Signal-induced proliferation-associated 1-like protein 1	1.00	0.97
Nudt16	Q6P3D0	U8 snoRNA-decapping enzyme	1.00	0.95
Septin10	A0A1W2P6J7	Septin-10	1.00	0.89
Hyou1	Q9JKR6	Hypoxia up-regulated protein 1	1.00	0.92
Nmt2	O70311	Glycylpeptide N-tetradecanoyltransferase 2	1.00	0.93
Rc3h1	Q4VGL6	Roquin-1	1.00	0.97
Nacc1	Q7TSZ8	Nucleus accumbens-associated protein 1	1.00	0.89
Noct	O35710	Nocturnin	1.00	0.96
Uaca	A0A0R4J0S7	Uveal autoantigen with coiled-coil domains and ankyrin repeats	1.00	0.96
Gm10073	E9Q3T0	Predicted pseudogene 10073	1.00	0.90
Elp5	Q99L85	Elongator complex protein 5	1.00	0.97
Txndc9	Q9CQ79	Thioredoxin domain-containing protein 9	1.00	0.95
Polr3c	Q9D483	DNA-directed RNA polymerase III subunit RPC3	1.00	0.98
Riok3	Q9DBU3	Serine/threonine-protein kinase RIO3	1.00	0.93
Ldlrap1	Q8C142	Low density lipoprotein receptor adapter protein 1	1.00	0.91
Rps6ka1	E9PWV3	Ribosomal protein S6 kinase	1.00	0.95
Eif3k	Q9DBZ5	Eukaryotic translation initiation factor 3 subunit K	1.00	0.95
Arfrp1	Q8BXL7	ADP-ribosylation factor-related protein 1	1.00	0.98
Ppp1cc	A0A0G2JFF1	Serine/threonine-protein phosphatase (Fragment)	1.00	0.94
Slc17a5	Q8BN82	Sialin	1.00	0.96
Ranbp9	E9Q5D6	Ran-binding protein 9	1.00	0.95
Cryl1	Q99KP3	Lambda-crystallin homolog	1.00	0.96
Syn1	O88935	Synapsin-1	1.00	0.94
Ndufs7	Q9DC70	NADH dehydrogenase [ubiquinone] iron-sulfur protein 7, mitochondrial	1.00	0.93
Rragc	Q99K70	Ras-related GTP-binding protein C	1.00	0.94
Adprh	P54923	[Protein ADP-ribosylarginine] hydrolase	1.00	0.94
Ifit1	Q64282	Interferon-induced protein with tetratricopeptide repeats 1	1.00	0.97
Gart	Q64737	Trifunctional purine biosynthetic protein adenosine-3	1.00	0.93

Cyth4	Q80YW0	Cytohesin-4	1.00	0.94
Chst14	Q80V53	Carbohydrate sulfotransferase 14	1.00	0.96
Lmnb1	P14733	Lamin-B1	1.00	0.94
Ttc5	Q99LG4	Tetratricopeptide repeat protein 5	1.00	0.97
Pald1	A0A0R4J007	Paladin	1.00	0.97
Actn1	Q7TPR4	Alpha-actinin-1	1.00	0.95
Edf1	Q9JMG1	Endothelial differentiation-related factor 1	1.00	0.91
Ddx17	Q3U741	DEAD (Asp-Glu-Ala-Asp) box polypeptide 17, isoform CRA_a	1.00	0.93
Phkg2	Q9DB30	Phosphorylase b kinase gamma catalytic chain, liver/testis isoform	1.00	0.93
Cnot7	Q60809	CCR4-NOT transcription complex subunit 7	1.00	0.97
Cmtr1	Q9DBC3	Cap-specific mRNA (nucleoside-2'-O-)-methyltransferase 1	1.00	0.95
Gdpd1	Q9CRY7	Lysophospholipase D GDPD1	1.00	0.95
Arl6ip4	Q9JM93	ADP-ribosylation factor-like protein 6-interacting protein 4	1.00	0.97
Ehd4	Q9EQP2	EH domain-containing protein 4	1.00	0.93
Wdr70	G3X934	MCG115964	1.00	0.88
Mrpl19	Q9D338	39S ribosomal protein L19, mitochondrial	1.00	0.95
Dera	Q91YP3	Deoxyribose-phosphate aldolase	1.00	0.93
Tmpo	Q61033	Lamina-associated polypeptide 2, isoforms alpha/zeta	1.00	0.95
Ndufa4	Q62425	Cytochrome c oxidase subunit NDUFA4	1.00	0.96
Impdh2	P24547	Inosine-5'-monophosphate dehydrogenase 2	1.00	0.94
Cd2ap	Q9JLQ0	CD2-associated protein	1.00	0.94
Ehd1	Q9WVK4	EH domain-containing protein 1	1.00	0.95
Hfe	P70387	Hereditary hemochromatosis protein homolog	1.00	0.96
Cdkn2c	Q60772	Cyclin-dependent kinase 4 inhibitor C	1.00	0.97
Ppig	A2AR02	Peptidyl-prolyl cis-trans isomerase G	1.00	0.95
Ppif	Q99KR7	Peptidyl-prolyl cis-trans isomerase F, mitochondrial	1.00	0.94
Rcn1	Q05186	Reticulocalbin-1	1.00	0.97
Txnl4a	P83877	Thioredoxin-like protein 4A	1.00	0.91
Ppp2r5e	Q61151	Serine/threonine-protein phosphatase 2A 56 kDa regulatory subunit epsilon isoform	1.00	0.96
Ankfy1	Q810B6	Rabankyrin-5	1.00	0.94
Gla	Q8BGZ6	Alpha-galactosidase	1.00	0.92
Nudt9	Q8BVU5	ADP-ribose pyrophosphatase, mitochondrial	1.00	0.94
Apex1	P28352	DNA-(apurinic or apyrimidinic site) lyase	1.00	0.96
Apeh	Q8R146	Acylamino-acid-releasing enzyme	1.00	0.94
Pik3r4	Q8VD65	Phosphoinositide 3-kinase regulatory subunit 4	1.00	0.95
Elp1	Q7TT37	Elongator complex protein 1	1.00	0.96
Dnajc8	Q6NZB0	DnaJ homolog subfamily C member 8	1.00	0.95
Sumo3	Q9Z172	Small ubiquitin-related modifier 3	1.00	0.96
Naa10	Q9QY36	N-alpha-acetyltransferase 10	1.00	0.95
Man2b2	O54782	Epididymis-specific alpha-mannosidase	1.00	0.94
Nudt14	Q9D142	Uridine diphosphate glucose pyrophosphatase	1.00	0.97
Zmym4	A2A791	Zinc finger MYM-type protein 4	1.00	0.98
Ddx59	Q9DBN9	Probable ATP-dependent RNA helicase DDX59	1.00	0.98
Zpr1	Q62384	Zinc finger protein ZPR1	1.00	0.96
Ccm2	Q8K2Y9	Cerebral cavernous malformations protein 2 homolog	1.00	0.98
Fam114a2	Q8VE88	Protein FAM114A2	1.00	0.94
C2cd5	A0A0N4SW93	C2 domain-containing protein 5	1.00	0.98
Thrap3	Q569Z6	Thyroid hormone receptor-associated protein 3	1.00	0.96
Atp6v1f	Q9D1K2	V-type proton ATPase subunit F	1.00	0.96
Usp47	A0A1L1SV73	Ubiquitin carboxyl-terminal hydrolase 47	1.00	0.95
Atg2b	Q80XK6	Autophagy-related protein 2 homolog B	1.00	0.97
Anxa6	P14824	Annexin A6	1.00	0.93
Ubap1	Q8BH48	Ubiquitin-associated protein 1	1.00	0.96
Anxa6	F8WIT2	Annexin	1.00	0.97
Trim36	Q80WG7	E3 ubiquitin-protein ligase Trim36	1.00	0.99
Snd1	Q78PY7	Staphylococcal nuclease domain-containing protein 1	1.00	0.95
Prim1	P20664	DNA primase small subunit	1.00	0.98
Lrrc47	E9PV22	Leucine-rich repeat-containing protein 47	1.00	0.95
Wdr44	Q6NVE8	WD repeat-containing protein 44	1.00	0.95
Pabpc1	P29341	Polyadenylate-binding protein 1	1.00	0.96
Rpia	P47968	Ribose-5-phosphate isomerase	1.00	0.98
Cebpa	P53566	CCAAT/enhancer-binding protein alpha	1.00	0.99
Ncapg2	Q6DFV1	Condensin-2 complex subunit G2	1.00	0.98
Aldh9a1	Q9JLJ2	4-trimethylaminobutyraldehyde dehydrogenase	1.00	0.95
Elp3	A0A286YDB8	Elongator complex protein 3	1.00	0.99
	Q91V76	Ester hydrolase C11orf54 homolog	1.00	0.95
Ripor1	Q68FE6	Rho family-interacting cell polarization regulator 1	1.00	0.98
Wdr55	Q9CX97	WD repeat-containing protein 55	1.00	0.99
Ttc4	Q8R3H9	Tetratricopeptide repeat protein 4	1.00	0.97
Ctsd	P18242	Cathepsin D	1.00	0.97
Apbb2	E9QPX0	Amyloid-beta A4 precursor protein-binding family B member 2	1.00	0.97
Itpa	Q9D892	Inosine triphosphate pyrophosphatase	1.00	0.92
Isoc2b	Q9DCC7	Isochorismatase domain-containing protein 2B	1.00	0.97

Ufd1	P70362	Ubiquitin recognition factor in ER-associated degradation protein 1	1.00	0.96
Tbcc	A0A0R4J0M1	Tubulin-specific Chaperone C	1.00	0.95
Smarca2	H3BLH0	Probable global transcription activator SNF2L2	1.00	0.99
Fto	Q8BGW1	Alpha-ketoglutarate-dependent dioxygenase FTO	1.00	0.97
Entpd6	Q3U0P5	Ectonucleoside triphosphate diphosphohydrolase 6	1.00	0.99
Hdac5	B7ZDF5	Histone deacetylase	1.00	0.99
Hnrnpk	P61979	Heterogeneous nuclear ribonucleoprotein K	1.00	0.96
Fabp7	P51880	Fatty acid-binding protein, brain	1.00	0.97
Capn2	O08529	Calpain-2 catalytic subunit	1.00	0.96
Thoc3	Q8VE80	THO complex subunit 3	1.00	0.97
Crlf3	Q9Z2L7	Cytokine receptor-like factor 3	1.00	0.97
Shmt2	Q9CZN7	Serine hydroxymethyltransferase, mitochondrial	1.00	0.97
Clcn6	A2A7F6	Chloride channel protein	1.00	0.95
Atg7	A0A0A0MQN4	Ubiquitin-like modifier-activating enzyme ATG7	1.00	0.95
Mthfsl	L7N466	5-formyltetrahydrofolate cyclo-ligase	1.00	0.98
Ikzf1	Q5SWT9	DNA-binding protein Ikaros	1.00	0.96
Akr1b10	G5E895	Aldo-keto reductase family 1, member B10 (aldose reductase)	1.00	0.96
Strap	Q9Z1Z2	Serine-threonine kinase receptor-associated protein	1.00	0.96
Mtx2	O88441	Metaxin-2	1.00	0.98
Galk1	Q9R0N0	Galactokinase	1.00	0.97
Eci2	Q9WUR2	Enoyl-CoA delta isomerase 2, mitochondrial	1.00	0.96
Pdf	S4R2K0	Peptide deformylase	1.00	0.99
Rad23b	P54728	UV excision repair protein RAD23 homolog B	1.00	0.95
Prpf4	Q9DAW6	U4/U6 small nuclear ribonucleoprotein Prp4	1.00	0.97
Ndufa7	Q9Z1P6	NADH dehydrogenase [ubiquinone] 1 alpha subcomplex subunit 7	1.00	0.97
Coa6	Q8BGD8	Cytochrome c oxidase assembly factor 6 homolog	1.00	0.97
Cdc42	P60766	Cell division control protein 42 homolog	1.00	0.96
Ndufs8	Q8K3J1	NADH dehydrogenase [ubiquinone] iron-sulfur protein 8, mitochondrial	1.00	0.97
Wasf2	Q8BH43	Wiskott-Aldrich syndrome protein family member 2	1.00	0.97
Stat3	P42227	Signal transducer and activator of transcription 3	1.00	0.97
Fxr1	A0A0G2JEP0	Fragile X mental retardation syndrome-related protein 1	1.00	0.99
Ercc1	P07903	DNA excision repair protein ERCC-1	1.00	0.99
Pdha1	P35486	Pyruvate dehydrogenase E1 component subunit alpha, somatic form, mitochondrial	1.00	0.98
Pml	Q60953	Protein PML	1.00	0.96
Stoml2	Q99JB2	Stomatin-like protein 2, mitochondrial	1.00	0.98
Cbr3	Q8K354	Carbonyl reductase [NADPH] 3	1.00	0.97
Rpp40	Q8R1F9	Ribonuclease P protein subunit p40	1.00	0.97
Gstz1	Q9WVL0	Maleylacetoacetate isomerase	1.00	0.98
Il1rn	P25085	Interleukin-1 receptor antagonist protein	1.00	0.99
Stag1	Q9D3E6	Cohesin subunit SA-1	1.00	0.99
Tyw5	A2RSX7	tRNA wybutosine-synthesizing protein 5	1.00	0.99
Agps	A2AL50	Alkylglycerone-phosphate synthase	1.00	0.97
Hadhb	Q99JY0	Trifunctional enzyme subunit beta, mitochondrial	1.00	0.98
Mtpn	P62774	Myotrophin	1.00	0.98
Osbp	Q3B7Z2	Oxysterol-binding protein 1	1.00	0.97
Fntb	Q8K2I1	Protein farnesyltransferase subunit beta	1.00	0.98
Polr2h	Q923G2	DNA-directed RNA polymerases I, II, and III subunit RPABC3	1.00	0.98
Grpel1	Q99LP6	GrpE protein homolog 1, mitochondrial	1.00	0.98
Lta4h	P24527	Leukotriene A-4 hydrolase	1.00	0.98
Elp6	Q8BK75	Elongator complex protein 6	1.00	0.99
Ddx58	Q6Q899	Probable ATP-dependent RNA helicase DDX58	1.00	0.98
Aim2	Q91VJ1	Interferon-inducible protein AIM2	1.00	0.98
Clk3	O35492	Dual specificity protein kinase CLK3	1.00	0.99
Fam129b	Q8R1F1	Niban-like protein 1	1.00	0.99
Mcm6	P97311	DNA replication licensing factor MCM6	1.00	0.98
Slc30a1	Q60738	Zinc transporter 1	1.00	0.99
Lamtor5	G3UW70	MCG21719	1.00	0.97
Nt5c	Q9JM14	5'(3')-deoxyribonucleotidase, cytosolic type	1.00	0.97
Txnrd1	Q9JMH6	Thioredoxin reductase 1, cytoplasmic	1.00	0.98
Vps26a	P40336	Vacuolar protein sorting-associated protein 26A	1.00	0.98
Dguok	Q9QX60	Deoxyguanosine kinase, mitochondrial	1.00	0.98
Nt5c2	E9Q9M1	Cytosolic purine 5'-nucleotidase	1.00	0.99
Stat1	A0A087WSP5	Signal transducer and activator of transcription	1.00	0.99
Trim32	Q8CH72	E3 ubiquitin-protein ligase TRIM32	1.00	0.99
Sowahc	Q8C0J6	Ankyrin repeat domain-containing protein SOWAHC	1.00	0.99
Mrpl43	Q99N89	39S ribosomal protein L43, mitochondrial	1.00	0.99
Nolc1	A0A286YDV7	Nucleolar and coiled-body phosphoprotein 1 (Fragment)	1.00	0.94
Cd300lb	A0A0R3P9D2	CMRF35-like molecule 7	1.00	1.00
Gba	P17439	Glucosylceramidase	1.00	0.99
Psmc4	P54775	26S proteasome regulatory subunit 6B	1.00	0.98
Fahd1	Q8R0F8	Acylpyruvase FAHD1, mitochondrial	1.00	0.99
Colgalt1	Q8K297	Procollagen galactosyltransferase 1	1.00	0.99
Sirt7	Q8BKJ9	NAD-dependent protein deacetylase sirtuin-7	1.00	1.00

Prpsap2	Q8R574	Phosphoribosyl pyrophosphate synthase-associated protein 2	1.00	0.99
Exosc9	Q9JHI7	Exosome complex component RRP45	1.00	0.99
Wdr73	Q9CWR1	WD repeat-containing protein 73	1.00	0.99
Smc2	Q8CG48	Structural maintenance of chromosomes protein 2	1.00	0.99
Pdlim2	Q8R1G6	PDZ and LIM domain protein 2	1.00	0.99
Apoo	Q9DCZ4	MICOS complex subunit Mic26	1.00	0.99
Gmpr2	Q99L27	GMP reductase 2	1.00	0.99
Tsc22d1	E9QLZ1	TSC22 domain family protein 1	1.00	0.99
AB124611	A0A0B4J1G2	cDNA sequence AB124611	1.00	0.99
Prmt1	Q9JIF0	Protein arginine N-methyltransferase 1	1.00	0.99
Supt4h1a	P63271	Transcription elongation factor SPT4-A	1.00	0.99
Rpa3	Q9CQ71	Replication protein A 14 kDa subunit	1.00	0.99
Psat1	Q99K85	Phosphoserine aminotransferase	1.00	0.99
Ncf4	P97369	Neutrophil cytosol factor 4	1.00	0.99
Akr1c12	Q9JLI0	Aldo-keto reductase a	1.00	1.00
Tbc1d13	Q8R3D1	TBC1 domain family member 13	1.00	0.99
Snapin	Q9Z266	SNARE-associated protein Snapin	1.00	1.00
Fbxo22	Q78JE5	F-box only protein 22	1.00	1.00
Kctd9	E9PUA6	BTB/POZ domain-containing protein KCTD9	1.00	1.00
Kdelc1	Q9JHP7	KDEL motif-containing protein 1	1.00	1.00
	Q6PIU9	Uncharacterized protein FLJ45252 homolog	1.00	0.99
Coasy	Q9DBL7	Bifunctional coenzyme A synthase	1.00	1.00
Rheb	Q921J2	GTP-binding protein Rheb	1.00	1.00
Nup50	Q9JIH2	Nuclear pore complex protein Nup50	1.00	0.99
Cbr4	Q91VT4	Carbonyl reductase family member 4	1.00	1.00
Ppp4c	P97470	Serine/threonine-protein phosphatase 4 catalytic subunit	1.00	1.00
Nipbl	Q6KCD5	Nipped-B-like protein	1.00	1.00
Cox16	Q9CR63	Cytochrome c oxidase assembly protein COX16 homolog, mitochondrial	1.00	0.99
Entpd7	Q3TCT4	Ectonucleoside triphosphate diphosphohydrolase 7	1.00	1.00
Brd8	Q8R3B7	Bromodomain-containing protein 8	1.00	1.00
Dlat	Q8BMF4	Dihydrolipoyllysine-residue acetyltransferase component of pyruvate dehydrogenase complex, mitochondrial	1.00	1.00
Gm11214	V9GX06	Predicted gene 11214 (Fragment)	1.00	1.00
Nampt	Q99KQ4	Nicotinamide phosphoribosyltransferase	1.00	1.00
Znf592	Q8BHZ4	Zinc finger protein 592	1.00	1.00
Ppp4r2	A0A0R4J0U2	Serine/threonine-protein phosphatase 4 regulatory subunit 2	1.00	1.00
Mtrex	Q9CZU3	Exosome RNA helicase MTR4	1.00	1.00
Rpp38	A2AJG0	Ribonuclease P protein subunit p38	1.00	1.00
Cryzl2	Q3UNZ8	Quinone oxidoreductase-like protein 2	1.00	1.00
Meaf6	Q2VPQ9	Chromatin modification-related protein MEAF6	1.00	1.00
Fam118b	Q8C569	Protein FAM118B	1.00	1.00
Gvin1	L7N451	Interferon-induced very large GTPase 1	1.00	1.00
Agfg1	A0A087WR52	Arf-GAP domain and FG repeat-containing protein 1 (Fragment)	1.00	1.00
Slc35a4	A0A087WQH8	Probable UDP-sugar transporter protein SLC35A4	1.00	1.00
Tpp2	Q64514	Tripeptidyl-peptidase 2	1.00	1.00
Jpt2	Q6PGH2	Jupiter microtubule associated homolog 2	1.00	1.00
Echdc1	Q9D9V3	Ethylmalonyl-CoA decarboxylase	1.00	1.00
Mocs3	A2BDX3	Adenylyltransferase and sulfurtransferase MOCS3	1.00	1.00
Dennd1c	Q8CFK6	DENN domain-containing protein 1C	1.00	1.00
Pacs2	A0A1Y7VLZ7	Phosphofurin acidic cluster sorting protein 2	1.00	1.00
Parn	A0A0R4J0P6	Poly(A)-specific ribonuclease PARN	1.00	1.00
Coq10a	E9Q3H6	Coenzyme Q10A	1.00	1.00
Thada	A8C756	Thyroid adenoma-associated protein homolog	1.00	1.00
Ss18	Q62280	Protein SSXT	1.00	1.00
Ercc4	Q9QZD4	DNA repair endonuclease XPF	1.00	1.00
Ldha	A0A1B0GSX0	L-lactate dehydrogenase	1.00	1.00
Fkbp3	Q62446	Peptidyl-prolyl cis-trans isomerase FKBP3	1.00	1.00
Pram1	Q6BCL1	PML-RARA-regulated adapter molecule 1	1.00	1.00
Plaa	P27612	Phospholipase A-2-activating protein	1.00	1.00
Pdhb	Q9D051	Pyruvate dehydrogenase E1 component subunit beta, mitochondrial	1.00	1.00
Cad	B2RQC6	CAD protein	1.00	1.00
Vcp	Q01853	Transitional endoplasmic reticulum ATPase	1.00	0.99
Wdhd1	A0A2K6EDP7	WD repeat and HMG-box DNA-binding protein 1	1.00	0.99
Rpl12	P35979	60S ribosomal protein L12	1.00	0.99
Pfkp	Q8C605	ATP-dependent 6-phosphofructokinase	1.00	1.00
Carhsp1	Q9CR86	Calcium-regulated heat stable protein 1	1.00	0.99
Snx27	Q3UHD6	Sorting nexin-27	1.00	0.99
Selenbp1	P17563	Methanethiol oxidase	1.00	0.99
Serpinf1	P97298	Pigment epithelium-derived factor	1.00	0.99
Asl	Q91YI0	Argininosuccinate lyase	1.00	0.99
Pip4k2b	Q80XI4	Phosphatidylinositol 5-phosphate 4-kinase type-2 beta	1.00	0.99
Cdan1	Q8CC12	Codanin-1	1.00	0.99
Camk1	Q91YS8	Calcium/calmodulin-dependent protein kinase type 1	1.00	0.99
Zfand5	O88878	AN1-type zinc finger protein 5	1.00	0.99

Ndufs3	Q9DCT2	NADH dehydrogenase [ubiquinone] iron-sulfur protein 3, mitochondrial	1.00	0.99
Hnrnph2	P70333	Heterogeneous nuclear ribonucleoprotein H2	1.00	0.99
Mcmbp	Q8R3C0	Mini-chromosome maintenance complex-binding protein	1.00	0.99
Qars	Q8BML9	Glutamine--tRNA ligase	1.00	0.99
Ndel1	Q9ERR1	Nuclear distribution protein nudE-like 1	1.00	0.99
Psmd1	Q3TXS7	26S proteasome non-ATPase regulatory subunit 1	1.00	0.99
Rcc2	Q8BK67	Protein RCC2	1.00	0.99
Sipa1	E9Q0Y4	Signal-induced proliferation-associated protein 1	1.00	0.99
Wdr77	Q99J09	Methylosome protein 50	1.00	1.00
Pdcd5	P56812	Programmed cell death protein 5	1.00	0.99
Rnaset2a	C0HKG5	Ribonuclease T2-A	1.00	0.99
Phrf1	A6H619	PHD and RING finger domain-containing protein 1	1.00	0.99
Fkbp11	Q9D1M7	Peptidyl-prolyl cis-trans isomerase FKBP11	1.00	0.99
Nudt12	Q9DCN1	Peroxisomal NADH pyrophosphatase NUDT12	1.00	0.99
Chmp1a	Q921W0	Charged multivesicular body protein 1a	1.00	0.98
Lrsam1	Q80ZI6	E3 ubiquitin-protein ligase LRSAM1	1.00	0.99
Vbp1	P61759	Prefoldin subunit 3	1.00	0.99
Gpx1	P11352	Glutathione peroxidase 1	1.00	0.99
Chmp7	Q8R1T1	Charged multivesicular body protein 7	1.00	0.99
Med18	Q9CZ82	Mediator of RNA polymerase II transcription subunit 18	1.00	0.99
Map3k11	Q80XI6	Mitogen-activated protein kinase kinase kinase 11	1.00	1.00
Apba1	B2RUJ5	Amyloid-beta A4 precursor protein-binding family A member 1	1.00	1.00
Usp25	P57080	Ubiquitin carboxyl-terminal hydrolase 25	1.00	0.99
Jak2	G5E852	Tyrosine-protein kinase	1.00	0.99
Gripap1	Q8VD04	GRIP1-associated protein 1	1.00	0.99
Trim14	Q8BVW3	Tripartite motif-containing protein 14	1.00	0.99
Papss1	Q60967	Bifunctional 3'-phosphoadenosine 5'-phosphosulfate synthase 1	1.00	0.99
Ppid	Q9CR16	Peptidyl-prolyl cis-trans isomerase D	1.00	0.98
Dnajb14	Q149L6	DnaJ homolog subfamily B member 14	1.00	0.99
Rfc4	Q99J62	Replication factor C subunit 4	1.00	0.99
Mtnd5	P03921	NADH-ubiquinone oxidoreductase chain 5	1.00	0.99
Prpf38b	Q80SY5	Pre-mRNA-splicing factor 38B	1.00	0.99
Akap11	E9Q774	A kinase (PRKA) anchor protein 11	1.00	0.99
Chmp2a	Q9DB34	Charged multivesicular body protein 2a	1.00	0.99
Oas1a	P11928	2'-5'-oligoadenylate synthase 1A	1.00	0.99
Glt8d1	Q6NSU3	Glycosyltransferase 8 domain-containing protein 1	1.00	0.99
Fadd	Q61160	FAS-associated death domain protein	1.00	0.99
Pwp2	Q8BU03	Periodic tryptophan protein 2 homolog	1.00	0.99
Rilpl1	Q9JJC6	RILP-like protein 1	1.00	0.99
Pmpca	Q9DC61	Mitochondrial-processing peptidase subunit alpha	1.00	0.98
Coq8b	E9QLB8	Atypical kinase COQ8B, mitochondrial	1.00	0.99
Pfdn5	Q9WU28	Prefoldin subunit 5	1.00	0.98
Mblac1	Q8BWY4	Metallo-beta-lactamase domain-containing protein 1	1.00	0.98
Rpp30	O88796	Ribonuclease P protein subunit p30	1.00	0.99
Sash1	F8VQK5	SAM and SH3 domain-containing protein 1	1.00	0.98
Tpd52l2	A2AUD5	Tumor protein D54	1.00	0.97
Gins3	Q9CY94	DNA replication complex GINS protein PSF3	1.00	0.99
Tbc1d22a	Q8R5A6	TBC1 domain family member 22A	1.00	0.98
Atad2b	E9Q166	ATPase family, AAA domain-containing 2B	1.00	0.99
Hspa4	Q3U2G2	Heat shock 70 kDa protein 4	1.00	0.98
Cd44	A2APM2	CD44 antigen	1.00	0.98
Mak16	Q8BGS0	Protein MAK16 homolog	1.00	0.98
Psmd9	Q9CR00	26S proteasome non-ATPase regulatory subunit 9	1.00	0.98
Lsm2	O35900	U6 snRNA-associated Sm-like protein LSm2	1.00	0.99
Dus3l	A0A0R4IZY9	tRNA-dihydrouridine(47) synthase [NAD(P)(+)]	1.00	0.98
Rplp2	P99027	60S acidic ribosomal protein P2	1.00	0.98
Kpna6	O35345	Importin subunit alpha-7	1.00	0.98
Fkbp15	Q6P9Q6	FK506-binding protein 15	1.00	0.98
Elmo1	Q8BPU7	Engulfment and cell motility protein 1	1.00	0.98
Sqor	Q9R112	Sulfide:quinone oxidoreductase, mitochondrial	1.00	0.98
Plek	Q9JHK5	Pleckstrin	1.00	0.98
Rtcb	Q99LF4	tRNA-splicing ligase RtcB homolog	1.00	0.96
Acsf2	Q8VCW8	Acyl-CoA synthetase family member 2, mitochondrial	1.00	0.97
Kiaa2013	Q91X21	Uncharacterized protein KIAA2013	1.00	0.99
Borcs6	Q9D6W8	BLOC-1-related complex subunit 6	1.00	0.98
Sgta	Q8BJU0	Small glutamine-rich tetratricopeptide repeat-containing protein alpha	1.00	0.97
Tra2a	E9QP00	Transformer-2 protein homolog alpha	1.00	0.98
Pea15	Q62048	Astrocytic phosphoprotein PEA-15	1.00	0.97
Clcn4	Q61418	H(+)/Cl(-) exchange transporter 4	1.00	0.99
Mrpl18	Q9CQL5	39S ribosomal protein L18, mitochondrial	1.00	0.99
Gne	Q3UW64	Bifunctional UDP-N-acetylglucosamine 2-epimerase/N-acetylmannosamine kinase	1.00	0.97
Igtp	Q9DCE9	Interferon gamma-induced GTPase	1.00	0.99
Cln5	Q3UMW8	Ceroid-lipofuscinosis neuronal protein 5 homolog	1.00	0.98

Slc16a3	P57787	Monocarboxylate transporter 4	1.00	0.98
Emc2	Q9CRD2	ER membrane protein complex subunit 2	1.00	0.98
Hdac6	Q9Z2V5	Histone deacetylase 6	1.00	0.98
Fmr1	E9QAT0	Synaptic functional regulator FMR1	1.00	0.99
Plcg2	Q8CIH5	1-phosphatidylinositol 4,5-bisphosphate phosphodiesterase gamma-2	1.00	0.97
Gpx4	O70325	Phospholipid hydroperoxide glutathione peroxidase	1.00	0.96
Edem1	Q925U4	ER degradation-enhancing alpha-mannosidase-like protein 1	1.00	0.99
Arhgap25	Q8BYW1	Rho GTPase-activating protein 25	1.00	0.98
Pgls	Q9CQ60	6-phosphogluconolactonase	1.00	0.97
Smc4	Q8CG47	Structural maintenance of chromosomes protein 4	1.00	0.98
Gbp6	A0A0G2JDV3	Guanylate-binding protein 6	1.00	0.99
Trim25	Q61510	E3 ubiquitin/ISG15 ligase TRIM25	1.00	0.97
Elob	P62869	Elongin-B	1.00	0.96
Eif3c	Q8R1B4	Eukaryotic translation initiation factor 3 subunit C	1.00	0.97
Rnf25	Q9QZR0	E3 ubiquitin-protein ligase RNF25	1.00	0.97
Fdxr	Q61578	NADPH:adrenodoxin oxidoreductase, mitochondrial	1.00	0.97
Polr2g	P62488	DNA-directed RNA polymerase II subunit RPB7	1.00	0.97
Cryz	P47199	Quinone oxidoreductase	1.00	0.97
Phc2	Q9QWH1	Polyhomeotic-like protein 2	1.00	0.95
Nat9	Q3UG98	N-acetyltransferase 9	1.00	0.98
Mrps25	Q9D125	28S ribosomal protein S25, mitochondrial	1.00	0.98
Syk	P48025	Tyrosine-protein kinase SYK	1.00	0.98
Slc35a2	A2AER4	UDP-galactose translocator	1.00	0.97
Dok1	P97465	Docking protein 1	1.00	0.97
Kpna4	A0A0B4J1E7	Importin subunit alpha-3	1.00	0.96
Rac2	Q05144	Ras-related C3 botulinum toxin substrate 2	1.00	0.97
Tvp23b	Q9D8T4	Golgi apparatus membrane protein TVP23 homolog B	1.00	0.98
Rpa2	Q3TE40	Replication protein A 32 kDa subunit	1.00	0.98
Gbp7	Q91Z40	Gbp6 protein	1.00	0.99
Abi2	Q6AXD2	Abi2 protein	1.00	0.98
Tmem173	Q3TBT3	Stimulator of interferon genes protein	1.00	0.96
Srsf4	Q542V3	Serine/arginine-rich-splicing factor 4	1.00	0.98
Ivd	Q9JHI5	Isovaleryl-CoA dehydrogenase, mitochondrial	1.00	0.97
Dph6	Q9CQ28	Diphthine--ammonia ligase	1.00	0.96
Tmod3	Q9JHJ0	Tropomodulin-3	1.00	0.96
Med30	Q9CQI9	Mediator of RNA polymerase II transcription subunit 30	1.00	0.98
Adsl	P54822	Adenylosuccinate lyase	1.00	0.95
Gon7	P0C8B4	EKC/KEOPS complex subunit GON7	1.00	0.99
Trnt1	Q8K1J6	CCA tRNA nucleotidyltransferase 1, mitochondrial	1.00	0.97
Kif15	Q6P9L6	Kinesin-like protein KIF15	1.00	0.97
Srsf11	E9Q6E5	Serine/arginine-rich-splicing factor 11	1.00	0.96
Atg101	Q9D8Z6	Autophagy-related protein 101	1.00	0.97
Gopc	K3W4Q9	Golgi-associated PDZ and coiled-coil motif-containing protein	1.00	0.96
Pik3ap1	Q9EQ32	Phosphoinositide 3-kinase adapter protein 1	1.00	0.98
Mvb12b	Q6KAU4	Multivesicular body subunit 12B	1.00	0.98
Scly	A0A0R4J069	Selenocysteine lyase	1.00	0.96
Plec	Q9QXS1	Plectin	1.00	0.97
Sec24c	G3X972	SEC24 related gene family, member C (S. cerevisiae), isoform CRA_a	1.00	0.97
Hadha	Q8BMS1	Trifunctional enzyme subunit alpha, mitochondrial	1.00	0.96
Mrpl40	Q9Z2Q5	39S ribosomal protein L40, mitochondrial	1.00	0.98
Snx19	Q6P4T1	Sorting nexin-19	1.00	0.99
Nkap	Q9D0F4	NF-kappa-B-activating protein	1.00	0.93
Ap1s3	Q7TN05	AP-1 complex subunit sigma-3	1.00	0.98
Gfm1	Q8K0D5	Elongation factor G, mitochondrial	1.00	0.96
Amz1	Q8BVF9	Archaemetzincin-1	1.00	0.97
Nup214	Q80U93	Nuclear pore complex protein Nup214	1.00	0.97
Pwp1	Q99LL5	Periodic tryptophan protein 1 homolog	1.00	0.96
Haus6	Q6NV99	HAUS augmin-like complex, subunit 6	1.00	0.98
Commd3	Q63829	COMM domain-containing protein 3	1.00	0.97
Aldh2	P47738	Aldehyde dehydrogenase, mitochondrial	1.00	0.96
Sec23a	Q01405	Protein transport protein Sec23A	1.00	0.96
Setd7	Q8VHL1	Histone-lysine N-methyltransferase SETD7	1.00	0.97
Llph	D3YXW1	MCG19223	1.00	0.98
Gna11	P21278	Guanine nucleotide-binding protein subunit alpha-11	1.00	0.98
Sirt2	Q8VDQ8	NAD-dependent protein deacetylase sirtuin-2	1.00	0.96
Hyal1	Q91ZJ9	Hyaluronidase-1	1.00	0.98
Trim56	A0A0R4J0Q6	E3 ubiquitin-protein ligase TRIM56	1.00	0.97
Spg21	Q9CQC8	Maspardin	1.00	0.96
Snx9	Q91VH2	Sorting nexin-9	1.00	0.95
Septin6	Q9R1T4	Septin-6	1.00	0.96
Coro1a	O89053	Coronin-1A	1.00	0.95
Nfkb1	P25799	Nuclear factor NF-kappa-B p105 subunit	1.00	0.95
Mgat2	Q921V5	Alpha-1,6-mannosyl-glycoprotein 2-beta-N-acetylglucosaminyltransferase	1.00	0.97

Nsd2	Q8BVE8	Histone-lysine N-methyltransferase NSD2	1.00	0.98
Arf2	Q8BSL7	ADP-ribosylation factor 2	1.00	0.91
Abi1	J3QNK8	Abl interactor 1	1.00	0.97
Ckb	Q04447	Creatine kinase B-type	1.00	0.96
Gpr89a	Q8BS95	Golgi pH regulator	1.00	0.98
Gtf2f1	Q3THK3	General transcription factor IIF subunit 1	1.00	0.97
Tes	Q921W7	Testin	1.00	0.97
Padi2	Q08642	Protein-arginine deiminase type-2	1.00	0.95
Sh3glb2	Q8R3V5	Endophilin-B2	1.00	0.94
Sumf2	Q8BPG6	Inactive C-alpha-formylglycine-generating enzyme 2	1.00	0.97
Septin11	A0A0J9YUL3	Septin 11, isoform CRA_b	1.00	0.95
Samd9l	E9PX59	Sterile alpha motif domain-containing protein 9-like	1.00	0.97
Ptms	Q9D0J8	Parathymosin	1.00	0.94
Ggct	Q9D7X8	Gamma-glutamylcyclotransferase	1.00	0.97
Dennd4b	A0A0R4J172	DENN domain-containing protein 4B	1.00	0.97
Myh9	Q8VDD5	Myosin-9	1.00	0.93
Slc35e1	Q8CD26	Solute carrier family 35 member E1	1.00	0.99
Nol11	Q8BJW5	Nucleolar protein 11	1.00	0.97
Son	H9KV01	Protein SON	1.00	0.98
Snrnp40	Q6PE01	U5 small nuclear ribonucleoprotein 40 kDa protein	1.00	0.93
Fmnl3	Q6ZPF4	Formin-like protein 3	1.00	0.95
Hk3	Q3TRM8	Hexokinase-3	1.00	0.97
Lcp2	Q60787	Lymphocyte cytosolic protein 2	1.00	0.97
Lig1	Q3U4X8	DNA ligase	1.00	0.96
Srpk2	A0A0R4J124	SRSF protein kinase 2	1.00	0.96
Septin9	Q80UG5	Septin-9	1.00	0.97
Ogfod3	Q9D136	2-oxoglutarate and iron-dependent oxygenase domain-containing protein 3	1.00	0.99
Ccs	Q9WU84	Copper chaperone for superoxide dismutase	1.00	0.97
Msh6	P54276	DNA mismatch repair protein Msh6	1.00	0.97
Anxa3	O35639	Annexin A3	1.00	0.96
Hmgcs1	Q8JZK9	Hydroxymethylglutaryl-CoA synthase, cytoplasmic	1.00	0.96
Srr	Q9QZX7	Serine racemase	1.00	0.96
Csnk2a2	O54833	Casein kinase II subunit alpha'	1.00	0.96
Nit1	Q8VDK1	Deaminated glutathione amidase	1.00	0.95
Aldh4a1	Q8CHT0	Delta-1-pyrroline-5-carboxylate dehydrogenase, mitochondrial	1.00	0.95
Eif1b	Q9CXU9	Eukaryotic translation initiation factor 1b	1.00	0.96
Ifi35	Q9D8C4	Interferon-induced 35 kDa protein homolog	1.00	0.96
Plekhg1	F6S200	Pleckstrin homology domain-containing, family G (with RhoGef domain) member 1 (Fragment)	1.00	0.96
Calr	P14211	Calreticulin	1.00	0.90
Snrpd2	P62317	Small nuclear ribonucleoprotein Sm D2	1.00	0.97
Pafah1b1	P63005	Platelet-activating factor acetylhydrolase IB subunit alpha	1.00	0.93
Fh	P97807	Fumarate hydratase, mitochondrial	1.00	0.92
Aldh1b1	Q9CZS1	Aldehyde dehydrogenase X, mitochondrial	1.00	0.95
Vps45	P97390	Vacuolar protein sorting-associated protein 45	1.00	0.94
Rcbtb2	Q99LJ7	RCC1 and BTB domain-containing protein 2	1.00	0.97
Bub3	Q9WVA3	Mitotic checkpoint protein BUB3	1.00	0.93
Dhdh	Q9DBB8	Trans-1,2-dihydrobenzene-1,2-diol dehydrogenase	1.00	0.95
Pla2g4a	P47713	Cytosolic phospholipase A2	1.00	0.97
Nubpl	Q9CWD8	Iron-sulfur protein NUBPL	1.00	0.95
Smc5	Q8CG46	Structural maintenance of chromosomes protein 5	1.00	0.96
Ube2g2	P60605	Ubiquitin-conjugating enzyme E2 G2	1.00	0.96
Supt5h	O55201	Transcription elongation factor SPT5	1.00	0.94
Tkfc	Q8VC30	Triokinase/FMN cyclase	1.00	0.96
Otud6b	A0A0A0MQF5	Deubiquitinase OTUD6B	1.00	0.95
Hpcal1	P62748	Hippocalcin-like protein 1	1.00	0.96
Siglec1	G3X8X6	Sialic acid binding Ig-like lectin 1, sialoadhesin, isoform CRA_b	1.00	0.97
Lsp1	P19973	Lymphocyte-specific protein 1	1.00	0.96
Pofut2	Q8VHI3	GDP-fucose protein O-fucosyltransferase 2	1.00	0.94
Rps6ka3	P18654	Ribosomal protein S6 kinase alpha-3	1.00	0.91
Camkk2	Q8C078	Calcium/calmodulin-dependent protein kinase kinase 2	1.00	0.98
Dennd1b	Q3U1T9	DENN domain-containing protein 1B	1.00	0.96
Stk24	Q99KH8	Serine/threonine-protein kinase 24	1.00	0.95
Snrpa1	P57784	U2 small nuclear ribonucleoprotein A'	1.00	0.93
Evl	P70429	Ena/VASP-like protein	1.00	0.95
Sdf4	Q61112	45 kDa calcium-binding protein	1.00	0.94
Gtf2h2	Q91YN8	General transcription factor IIH subunit	1.00	0.96
Glod4	Q9CPV4	Glyoxalase domain-containing protein 4	1.00	0.93
Parp12	Q8BZ20	Poly [ADP-ribose] polymerase 12	1.00	0.95
Atxn3	Q9CVD2	Ataxin-3	1.00	0.91
Dscr3	O35075	Down syndrome critical region protein 3 homolog	1.00	0.92
Crkl	P47941	Crk-like protein	1.00	0.89
Hmgcl	P38060	Hydroxymethylglutaryl-CoA lyase, mitochondrial	1.00	0.94
Mrpl23	O35972	39S ribosomal protein L23, mitochondrial	1.00	0.96

Acadsb	Q9DBL1	Short/branched chain specific acyl-CoA dehydrogenase, mitochondrial	1.00	0.95
Rdx	P26043	Radixin	1.00	0.91
Prkab1	Q9R078	5'-AMP-activated protein kinase subunit beta-1	1.00	0.96
Cops2	P61202	COP9 signalosome complex subunit 2	1.00	0.91
Gtf2b	P62915	Transcription initiation factor IIB	1.00	0.97
Sp140	Q6NSQ5	Sp140 nuclear body protein	1.00	0.97
Fbxo3	Q9DC63	F-box only protein 3	1.00	0.94
Dynlt1	P51807	Dynein light chain Tctex-type 1	1.00	0.96
Mars	E9QB02	Methionine--tRNA ligase, cytoplasmic	1.00	0.94
Secisbp2	Q3U1C4	MCG1271	1.00	0.97
Arhgef10l	A2AWP8	Rho guanine nucleotide exchange factor 10-like protein	1.00	0.94
Bcl2l11	O54918	Bcl-2-like protein 11	1.00	0.93
Snx17	Q8BVL3	Sorting nexin-17	1.00	0.93
Sh3bp5l	Q99LH9	SH3 domain-binding protein 5-like	1.00	0.97
Tars	Q9D0R2	Threonine--tRNA ligase, cytoplasmic	1.00	0.90
Prkci	Q62074	Protein kinase C iota type	1.00	0.94
Trim34a	E9PYZ4	Tripartite motif-containing protein 34A	1.00	0.94
Fkbp8	O35465	Peptidyl-prolyl cis-trans isomerase FKBP8	1.00	0.93
Ap1s2	Q3TIV9	AP complex subunit sigma	1.00	0.93
Tor1b	Q9ER41	Torsin-1B	1.00	0.93
Rictor	Q6QI06	Rapamycin-insensitive companion of mTOR	1.00	0.97
Rbm39	Q8VH51	RNA-binding protein 39	1.00	0.95
Ipo13	Q8K0C1	Importin-13	1.00	0.96
Mrps35	A0A0R4J0L6	28S ribosomal protein S35, mitochondrial	1.00	0.94
Adh5	P28474	Alcohol dehydrogenase class-3	1.00	0.92
Fkbp4	P30416	Peptidyl-prolyl cis-trans isomerase FKBP4	1.00	0.93
Cdk5	P49615	Cyclin-dependent-like kinase 5	1.00	0.94
Mtf2	Q02395	Metal-response element-binding transcription factor 2	1.00	0.97
Acaa1a	Q921H8	3-ketoacyl-CoA thiolase A, peroxisomal	1.00	0.94
Tsc22d3	Q9Z2S7	TSC22 domain family protein 3	1.00	0.95
Pdcd4	Q61823	Programmed cell death protein 4	1.00	0.96
Eef2k	O08796	Eukaryotic elongation factor 2 kinase	1.00	0.95
Mrpl28	Q9D1B9	39S ribosomal protein L28, mitochondrial	1.00	0.96
Vps4a	Q8VEJ9	Vacuolar protein sorting-associated protein 4A	1.00	0.93
Mzt1	Q8BUR9	Mitotic-spindle organizing protein 1	1.00	0.94
Phb2	O35129	Prohibitin-2	1.00	0.94
	Q80WR5	UPF0688 protein C1orf174 homolog	1.00	0.96
Syvn1	A0A0R4J1R1	E3 ubiquitin-protein ligase synoviolin	1.00	0.93
Pstk	Q8BP74	L-seryl-tRNA(Sec) kinase	1.00	0.98
Vps35	Q9EQH3	Vacuolar protein sorting-associated protein 35	1.00	0.90
Smarcc2	Q6PDG5	SWI/SNF complex subunit SMARCC2	1.00	0.90
Nek7	Q9ES74	Serine/threonine-protein kinase Nek7	1.00	0.93
Calu	O35887	Calumenin	1.00	0.95
Rnf146	Q9CZW6	E3 ubiquitin-protein ligase RNF146	1.00	0.98
Ppp1r7	Q3UM45	Protein phosphatase 1 regulatory subunit 7	1.00	0.88
Flna	B7FAU9	Filamin, alpha	1.00	0.93
Tnfaip8l2	Q9D8Y7	Tumor necrosis factor alpha-induced protein 8-like protein 2	1.00	0.93
Gga1	Q8R0H9	ADP-ribosylation factor-binding protein GGA1	1.00	0.93
Nipa2	Q9JJC8	Magnesium transporter NIPA2	1.00	0.99
Mbd1	A0A286YDI1	Methyl-CpG-binding domain protein 1	1.00	0.96
Aco2	Q99KI0	Aconitate hydratase, mitochondrial	1.00	0.91
Rad21	Q61550	Double-strand-break repair protein rad21 homolog	1.00	0.91
Arih2	Q9Z1K6	E3 ubiquitin-protein ligase ARIH2	1.00	0.92
Card6	E9PWH2	Caspase recruitment domain family, member 6	1.00	0.94
Washc4	Q3UMB9	WASH complex subunit 4	1.00	0.91
Ptk2b	F7CCX1	Protein-tyrosine kinase 2-beta (Fragment)	1.00	0.86
Selplg	Q3TA56	P-selectin glycoprotein ligand 1	1.00	0.97
Acadl	A0A0R4J083	Long-chain-specific acyl-CoA dehydrogenase, mitochondrial	1.00	0.94
Aqr	Q8CFQ3	RNA helicase aquarius	1.00	0.95
Fuca1	Q99LJ1	Tissue alpha-L-fucosidase	1.00	0.91
Sf3b2	Q3UJB0	Splicing factor 3b, subunit 2	1.00	0.94
Fen1	Q91Z50	Flap endonuclease 1	1.00	0.93
Tsg101	Q61187	Tumor susceptibility gene 101 protein	1.00	0.92
Safb	S4R1M2	Scaffold attachment factor B1	1.00	0.92
Cbwd1	Q8VEH6	COBW domain-containing protein 1	1.00	0.92
Hdlbp	Q8VDJ3	Vigilin	1.00	0.92
Snrnp70	Q62376	U1 small nuclear ribonucleoprotein 70 kDa	1.00	0.90
Anks1	Q3UHP6	Ankyrin repeat and SAM domain containing 1	1.00	0.96
Dut	Q8VCG1	Deoxyuridine triphosphatase	1.00	0.91
Uba5	Q8VE47	Ubiquitin-like modifier-activating enzyme 5	1.00	0.95
Dda1	Q9D9Z5	DET1- and DDB1-associated protein 1	1.00	0.95
Strn3	B2RQS1	Striatin-3	1.00	0.92
Tp53bp1	P70399	TP53-binding protein 1	1.00	0.92

Aco1	P28271	Cytoplasmic aconitate hydratase	1.00	0.92
Acat1	Q8QZT1	Acetyl-CoA acetyltransferase, mitochondrial	1.00	0.91
Ints9	A0A0R4J0J5	Integrator complex subunit 9	1.00	0.96
Gtpbp10	Q8K013	GTP-binding protein 10	1.00	0.94
Psme3	P61290	Proteasome activator complex subunit 3	1.00	0.90
Inpp5d	Q9ES52	Phosphatidylinositol 3,4,5-trisphosphate 5-phosphatase 1	1.00	0.92
Ywhae	P62259	14-3-3 protein epsilon	1.00	0.58
Fxr1	Q61584	Fragile X mental retardation syndrome-related protein 1	1.00	0.90
Smarcd1	Q61466	SWI/SNF-related matrix-associated actin-dependent regulator of chromatin subfamily D member 1	1.00	0.96
Dstn	Q9R0P5	Destrin	1.00	0.89
Ppp1r12a	Q9DBR7	Protein phosphatase 1 regulatory subunit 12A	1.00	0.88
Cnpy3	Q9DAU1	Protein canopy homolog 3	1.00	0.90
Tank	P70347	TRAF family member-associated NF-kappa-B activator	1.00	0.94
Mbnl2	A0A2K6EDM5	Muscleblind-like protein 2	1.00	0.93
Mbnl1	A0A0A6YVV8	Muscleblind-like protein 1	1.00	0.95
Tifa	Q793I8	TRAF-interacting protein with FHA domain-containing protein A	1.00	0.92
Stx17	Q9D0I4	Syntaxin-17	1.00	0.95
Map4k3	E9QNE9	Mitogen-activated protein kinase kinase kinase kinase	1.00	0.92
Puf60	Q3UEB3	Poly(U)-binding-splicing factor PUF60	1.00	0.93
Trappc8	A0A286YCX6	Trafficking protein particle complex 8	1.00	0.92
Pcm1	Q9R0L6	Pericentriolar material 1 protein	1.00	0.93
Psmd6	Q99JI4	26S proteasome non-ATPase regulatory subunit 6	1.00	0.84
Limd1	Q9QXD8	LIM domain-containing protein 1	1.00	0.91
Dgkd	E9PUQ8	Diacylglycerol kinase	1.00	0.96
Gsdmdc1	Q9D8T2	Gasdermin-D	1.00	0.90
Atg13	Q91YI1	Autophagy-related protein 13	1.00	0.97
Ifi204	P0DOV2	Interferon-activable protein 204	1.00	0.91
Otub1	Q7TQI3	Ubiquitin thioesterase OTUB1	1.00	0.95
Eif3h	Q91WK2	Eukaryotic translation initiation factor 3 subunit H	1.00	0.92
Trpm7	Q923J1	Transient receptor potential cation channel subfamily M member 7	1.00	0.97
Brcc3	P46737	Lys-63-specific deubiquitinase BRCC36	1.00	0.90
Dhrs11	Q3U0B3	Dehydrogenase/reductase SDR family member 11	1.00	0.96
Slc38a9	Q8BGD6	Sodium-coupled neutral amino acid transporter 9	1.00	0.97
Rps6ka4	Q9Z2B9	Ribosomal protein S6 kinase alpha-4	1.00	0.95
Fgd3	Q3TNB8	FYVE, RhoGEF and PH domain containing 3, isoform CRA_b	1.00	0.95
Dbnl	Q62418	Drebrin-like protein	1.00	0.90
Atp5f1e	P56382	ATP synthase subunit epsilon, mitochondrial	1.00	0.93
Aldh16a1	A0A1B0GSU0	Aldehyde dehydrogenase family 16 member A1	1.00	0.92
Nsun2	Q1HFZ0	tRNA (cytosine(34)-C(5))-methyltransferase	1.00	0.92
Ep300	B2RWS6	Histone acetyltransferase p300	1.00	0.95
Decr1	Q9CQ62	2,4-dienoyl-CoA reductase, mitochondrial	1.00	0.90
Ndufaf2	Q59J78	NADH dehydrogenase [ubiquinone] 1 alpha subcomplex assembly factor 2	1.00	0.92
Phospho2	Q9D9M5	Pyridoxal phosphate phosphatase PHOSPHO2	1.00	0.93
Mvb12a	Q78HU3	Multivesicular body subunit 12A	1.00	0.95
Gbp9	Q8BTS3	Guanylate-binding protein 9	1.00	0.90
Mecr	Q9DCS3	Enoyl-[acyl-carrier-protein] reductase, mitochondrial	1.00	0.93
Smpdl3b	P58242	Acid sphingomyelinase-like phosphodiesterase 3b	1.00	0.92
Prdx3	P20108	Thioredoxin-dependent peroxide reductase, mitochondrial	1.00	0.94
Arhgap18	Q8K0Q5	Rho GTPase-activating protein 18	1.00	0.90
Retreg2	Q6NS82	Reticulophagy regulator 2	1.00	0.92
Tmlhe	Q91ZE0	Trimethyllysine dioxygenase, mitochondrial	1.00	0.93
Mapk3	Q63844	Mitogen-activated protein kinase 3	1.00	0.87
Sec13	Q9D1M0	Protein SEC13 homolog	1.00	0.92
Pole4	Q9CQ36	DNA polymerase epsilon subunit 4	1.00	0.93
Gcfc2	Q8BKT3	GC-rich sequence DNA-binding factor 2	1.00	0.95
Ankmy2	Q3TPE9	Ankyrin repeat and MYND domain-containing protein 2	1.00	0.91
Map2k7	Q8CE90	Dual specificity mitogen-activated protein kinase kinase 7	1.00	0.96
Napg	Q9CWZ7	Gamma-soluble NSF attachment protein	1.00	0.93
Gtf2e1	Q9D0D5	General transcription factor IIE subunit 1	1.00	0.95
Pikfyve	Q9Z1T6	1-phosphatidylinositol 3-phosphate 5-kinase	1.00	0.92
Naa20	Q9DB82	N-acetyltransferase 5 (ARD1 homolog, S. cerevisiae), isoform CRA_a	1.00	0.94
Kpnb1	P70168	Importin subunit beta-1	1.00	0.85
Nckipsd	Q9ESJ4	NCK-interacting protein with SH3 domain	1.00	0.93
Syf2	Q9D198	Pre-mRNA-splicing factor SYF2	1.00	0.80
Tbc1d17	Q8BYH7	TBC1 domain family member 17	1.00	0.94
Nr2c2ap	Q3TV70	Nuclear receptor 2C2-associated protein	1.00	0.80
Eftud2	O08810	116 kDa U5 small nuclear ribonucleoprotein component	1.00	0.91
Tgfbrap1	Q3UR70	Transforming growth factor-beta receptor-associated protein 1	1.00	0.95
Hectd3	Q3U487	E3 ubiquitin-protein ligase HECTD3	1.00	0.92
Abcf3	Q8K268	ATP-binding cassette sub-family F member 3	1.00	0.92
Abhd11	Q8K4F5	Protein ABHD11	1.00	0.93
Kansl3	A2RSY1	KAT8 regulatory NSL complex subunit 3	1.00	0.96
Wwox	Q91WL8	WW domain-containing oxidoreductase	1.00	0.96

Copb1	Q9JIF7	Coatomer subunit beta	1.00	0.88
Cstf3	Q99LI7	Cleavage stimulation factor subunit 3	1.00	0.92
Pctp	Q5SV41	Phosphatidylcholine transfer protein	1.00	0.88
Zfand2b	Q91X58	AN1-type zinc finger protein 2B	1.00	0.87
Adk	P55264	Adenosine kinase	1.00	0.85
Txndc16	Q7TN22	Thioredoxin domain-containing protein 16	1.00	0.96
Wdyhv1	Q80WB5	Protein N-terminal glutamine amidohydrolase	1.00	0.93
Maco1	Q7TQE6	Macoilin	1.00	0.97
Abcc3	B2RX12	Canalicular multispecific organic anion transporter 2	1.00	0.85
Uqcc2	Q9CQY6	Ubiquinol-cytochrome-c reductase complex assembly factor 2	1.00	0.90
Xrn2	Q9DBR1	5'-3' exoribonuclease 2	1.00	0.90
Mpp1	P70290	55 kDa erythrocyte membrane protein	1.00	0.92
Tsfm	Q9CZR8	Elongation factor Ts, mitochondrial	1.00	0.92
Bid	P70444	BH3-interacting domain death agonist	1.00	0.92
B2m	P01887	Beta-2-microglobulin	1.00	0.91
Scyl2	Q8CFE4	SCY1-like protein 2	1.00	0.89
Suds3	A0A0R4J243	Sin3 histone deacetylase corepressor complex component SDS3	1.00	0.86
Eif4e2	D3YUV9	Eukaryotic translation initiation factor 4E type 2	1.00	0.94
Taf12	E9QNT5	Transcription initiation factor TFIID subunit 12	1.00	0.96
Iars2	Q8BIJ6	Isoleucine--tRNA ligase, mitochondrial	1.00	0.88
Ctdp1	Q7TSG2	RNA polymerase II subunit A C-terminal domain phosphatase	1.00	0.95
Aatk	B1AZF3	Serine/threonine-protein kinase LMTK1	1.00	0.94
Ccdc91	Q9D8L5	Coiled-coil domain-containing protein 91	1.00	0.94
Echs1	Q8BH95	Enoyl-CoA hydratase, mitochondrial	1.00	0.92
Psmd11	Q8BG32	26S proteasome non-ATPase regulatory subunit 11	1.00	0.87
Gnpda2	Q9CRC9	Glucosamine-6-phosphate isomerase 2	1.00	0.90
Exoc7	O35250	Exocyst complex component 7	1.00	0.90
Nudcd1	Q6PIP5	NudC domain-containing protein 1	1.00	0.91
Brk1	Q91VR8	Protein BRICK1	1.00	0.88
Rps24	P62849	40S ribosomal protein S24	1.00	0.86
Gale	Q8R059	UDP-glucose 4-epimerase	1.00	0.93
Abhd5	Q9DBL9	1-acylglycerol-3-phosphate O-acyltransferase ABHD5	1.00	0.92
Etf1	Q8BWY3	Eukaryotic peptide chain release factor subunit 1	1.00	0.79
Kctd12b	Q8C7J6	Potassium channel tetramerisation domain containing 12b, isoform CRA_a	1.00	0.82
Sec23b	Q9D662	Protein transport protein Sec23B	1.00	0.92
Dync1i2	O88487	Cytoplasmic dynein 1 intermediate chain 2	1.00	0.90
Ggh	Q9Z0L8	Gamma-glutamyl hydrolase	1.00	0.84
Elof1	P60003	Transcription elongation factor 1 homolog	1.00	0.90
Cops9	D3Z159	COP9 signalosome complex subunit 9	1.00	0.96
Nmnat3	Q99JR6	Nicotinamide/nicotinic acid mononucleotide adenylyltransferase 3	1.00	0.92
Ctnnd1	P30999	Catenin delta-1	1.00	0.88
Carm1	D3YUP1	Histone-arginine methyltransferase CARM1	1.00	0.92
Bckdha	Q3U3J1	2-oxoisovalerate dehydrogenase subunit alpha	1.00	0.92
Calm1	Q3UKW2	Calmodulin-1	1.00	0.78
Ugdh	O70475	UDP-glucose 6-dehydrogenase	1.00	0.79
Zdhhc18	Q5Y5T2	Palmitoyltransferase ZDHHC18	1.00	0.93
Morc3	F7BJB9	MORC family CW-type zinc finger protein 3	1.00	0.88
Nme7	Q3UMG6	Non-metastatic cells 7, protein expressed in, isoform CRA_c	1.00	0.94
Aatf	Q9JKX4	Protein AATF	1.00	0.91
Mrpl39	Q9JKF7	39S ribosomal protein L39, mitochondrial	1.00	0.88
Prkcb	P68404	Protein kinase C beta type	1.00	0.90
Arrb2	Q91YI4	Beta-arrestin-2	1.00	0.88
Trim26	J3QN94	Tripartite motif-containing protein 26	1.00	0.91
Wdr61	Q9ERF3	WD repeat-containing protein 61	1.00	0.85
Nln	Q91YP2	Neurolysin, mitochondrial	1.00	0.83
Itgam	G5E8F1	Integrin alpha-M	1.00	0.87
Limk1	P53668	LIM domain kinase 1	1.00	0.93
Ppil4	Q9CXG3	Peptidyl-prolyl cis-trans isomerase-like 4	1.00	0.93
Chek2	Q9Z265	Serine/threonine-protein kinase Chk2	1.00	0.93
Ncaph	Q8C156	Condensin complex subunit 2	1.00	0.94
Cab39l	Q9DB16	Calcium-binding protein 39-like	1.00	0.80
Ndufb10	Q9DCS9	NADH dehydrogenase [ubiquinone] 1 beta subcomplex subunit 10	1.00	0.90
Ube2z	Q3UE37	Ubiquitin-conjugating enzyme E2 Z	1.00	0.87
Kcnab2	Q3UPV6	Voltage-gated potassium channel subunit beta-2	1.00	0.92
Eci1	P42125	Enoyl-CoA delta isomerase 1, mitochondrial	1.00	0.90
Sec24b	Q80ZX0	Sec24-related gene family, member B (S. cerevisiae)	1.00	0.87
Pygm	Q9WUB3	Glycogen phosphorylase, muscle form	1.00	0.92
Ppp2r5c	A0A1Y7VIR0	Serine/threonine-protein phosphatase 2A 56 kDa regulatory subunit gamma isoform (Fragment)	1.00	0.89
Uap1l1	Q3TW96	UDP-N-acetylhexosamine pyrophosphorylase-like protein 1	1.00	0.90
Dtymk	P97930	Thymidylate kinase	1.00	0.92
Nasp	B1AU75	Nuclear autoantigenic sperm protein	1.00	0.86
Zfp748	Q7TPL6	Zinc finger protein 748	1.00	0.98
Dnaja2	Q9QYJ0	DnaJ homolog subfamily A member 2	1.00	0.94

Slc26a2	Q62273	Sulfate transporter	1.00	0.93
Kat7	Q5SVQ0	Histone acetyltransferase KAT7	1.00	0.92
Csf1r	P09581	Macrophage colony-stimulating factor 1 receptor	1.00	0.88
Ralgapa1	A0A2I3BRX9	Ral GTPase-activating protein subunit alpha-1	1.00	0.97
Nelfe	P19426	Negative elongation factor E	1.00	0.93
Mrpl57	Q9CQF8	Ribosomal protein 63, mitochondrial	1.00	0.95
Dnajc7	Q9QYI3	DnaJ homolog subfamily C member 7	1.00	0.75
Eif3e	P60229	Eukaryotic translation initiation factor 3 subunit E	1.00	0.83
Cgas	Q8C6L5	Cyclic GMP-AMP synthase	1.00	0.89
Slc7a8	Q9QXW9	Large neutral amino acids transporter small subunit 2	1.00	0.93
Fnbp1	Q80TY0	Formin-binding protein 1	1.00	0.87
Eya3	Q6P4T3	Eyes absent homolog	1.00	0.96
Mmp12	P34960	Macrophage metalloelastase	1.00	0.93
Eif5	P59325	Eukaryotic translation initiation factor 5	1.00	0.83
Pitpna	P53810	Phosphatidylinositol transfer protein alpha isoform	1.00	0.90
Evi2b	Q8VD58	Protein EVI2B	1.00	0.96
P4hb	P09103	Protein disulfide-isomerase	1.00	0.81
Helz2	A2AS03	Helicase with zinc finger domain 2	1.00	0.94
Ppm1f	Q8CGA0	Protein phosphatase 1F	1.00	0.90
Aamp	J3QN89	Angio-associated migratory protein	1.00	0.92
Gemin5	E9PUU4	Gem-associated protein 5	1.00	0.92
Dcun1d1	Q9QZ73	DCN1-like protein 1	1.00	0.83
Bclaf3	A2AG58	BCLAF1 and THRAP3 family member 3	1.00	0.90
Txlng	Q8BHN1	Gamma-taxilin	1.00	0.97
Crybg3	A0A338P6N4	Beta/gamma crystallin domain-containing protein 3	1.00	0.83
Dek	Q7TNV0	Protein DEK	1.00	0.90
Derl1	Q99J56	Derlin-1	1.00	0.96
Tufm	Q8BFR5	Elongation factor Tu, mitochondrial	1.00	0.87
Nfs1	Q9Z1J3	Cysteine desulfurase, mitochondrial	1.00	0.90
Fam172a	Q3TNH5	Cotranscriptional regulator FAM172A	1.00	0.92
Ezr	P26040	Ezrin	1.00	0.89
Arhgef1	E9PUF7	Rho guanine nucleotide exchange factor 1	1.00	0.90
Pank2	Q3U4S0	Pantothenate kinase 2	1.00	0.90
Lin7c	O88952	Protein lin-7 homolog C	1.00	0.91
Ythdc2	B2RR83	3'-5' RNA helicase YTHDC2	1.00	0.89
Ube2b	P63147	Ubiquitin-conjugating enzyme E2 B	1.00	0.90
Dennd1a	Q8K382	DENN domain-containing protein 1A	1.00	0.90
Mfn1	Q811U4	Mitofusin-1	1.00	0.94
Mgmt	P26187	Methylated-DNA--protein-cysteine methyltransferase	1.00	0.96
Ncoa7	Q6DFV7	Nuclear receptor coactivator 7	1.00	0.93
Wdr12	Q9JJA4	Ribosome biogenesis protein WDR12	1.00	0.92
Zbtb21	E9Q444	Zinc finger and BTB domain-containing 21	1.00	0.96
Copg2	Q9QXK3	Coatomer subunit gamma-2	1.00	0.76
Larp1b	F6U5V1	La ribonucleoprotein domain family, member 1B	1.00	0.95
Mfsd1	Q9DC37	Major facilitator superfamily domain-containing protein 1	1.00	0.90
Pdpk1	Q9Z2A0	3-phosphoinositide-dependent protein kinase 1	1.00	0.87
Arhgap45	G3X9Q3	Histocompatibility (Minor) HA-1, isoform CRA_a	1.00	0.78
Impa1	Q924B0	Inositol-1-monophosphatase	1.00	0.83
Chm	A2AD03	Rab proteins geranylgeranyltransferase component A	1.00	0.89
Lasp1	Q61792	LIM and SH3 domain protein 1	1.00	0.92
Nol7	Q9D7Z3	Nucleolar protein 7	1.00	0.93
Nfkb2	Q9WTK5	Nuclear factor NF-kappa-B p100 subunit	1.00	0.89
Ppt2	O35448	Lysosomal thioesterase PPT2	1.00	0.93
Socs6	Q9JLY0	Suppressor of cytokine signaling 6	1.00	0.90
Wdr3	Q8BHB4	WD repeat-containing protein 3	1.00	0.88
Rbsn	Q80Y56	Rabenosyn-5	1.00	0.89
Cox19	Q8K0C8	Cytochrome c oxidase assembly protein COX19	1.00	0.90
Rnaseh2a	Q9CWY8	Ribonuclease H2 subunit A	1.00	0.94
Tmem135	Q9CYV5	Transmembrane protein 135	1.00	0.95
Usb1	A0A0R4J0E0	U6 snRNA phosphodiesterase	1.00	0.92
Cabin1	G3X8Q1	Calcineurin binding protein 1, isoform CRA_a	1.00	0.95
Grk2	Q99MK8	Beta-adrenergic receptor kinase 1	1.00	0.87
Ttc37	F8VPK0	Tetratricopeptide repeat domain 37	1.00	0.87
Irf2bpl	Q8K3X4	Interferon regulatory factor 2-binding protein-like	1.00	0.89
Ndufa6	Q9CQZ5	NADH dehydrogenase [ubiquinone] 1 alpha subcomplex subunit 6	1.00	0.85
Igf2bp2	Q5SF07	Insulin-like growth factor 2 mRNA-binding protein 2	1.00	0.88
Prpf6	Q91YR7	Pre-mRNA-processing factor 6	1.00	0.89
Me2	Q99KE1	NAD-dependent malic enzyme, mitochondrial	1.00	0.90
Nrd1	A2A9Q2	Nardilysin, N-arginine dibasic convertase, NRD convertase 1	1.00	0.70
Rbpj	P31266	Recombining binding protein suppressor of hairless	1.00	0.88
Bap1	Q99PU7	Ubiquitin carboxyl-terminal hydrolase BAP1	1.00	0.96
Vars	Q9Z1Q9	Valine--tRNA ligase	1.00	0.89
Gmppb	Q8BTZ7	Mannose-1-phosphate guanyltransferase beta	1.00	0.72

Nadk2	Q8C5H8	NAD kinase 2, mitochondrial	1.00	0.90
Eif3b	Q8JZQ9	Eukaryotic translation initiation factor 3 subunit B	1.00	0.85
Ccndbp1	Q3TVC7	Cyclin-D1-binding protein 1	1.00	0.95
Cpne1	Q8C166	Copine-1	1.00	0.89
Sirpb1b	A0A0A6YXN8	Signal-regulatory protein beta 1B	1.00	0.93
Adar	Q99MU3	Double-stranded RNA-specific adenosine deaminase	1.00	0.89
Fabp5	Q05816	Fatty acid-binding protein 5	1.00	0.88
Gm7324	A0A2I3BRL8	Predicted gene 7324	1.00	0.82
Cdc42se2	Q8BGH7	CDC42 small effector protein 2	1.00	0.97
Blvra	Q9CY64	Biliverdin reductase A	1.00	0.82
Efcab14	Q6PCQ6	EF-hand calcium-binding domain-containing protein 14	1.00	0.93
Cnih4	Q9CX13	Protein cornichon homolog 4	1.00	0.95
Smu1	Q3UKJ7	WD40 repeat-containing protein SMU1	1.00	0.90
Pithd1	Q8BWR2	PITH domain-containing protein 1	1.00	0.85
Ppfia1	B2RXQ2	Ppfia1 protein	1.00	0.89
Hars	Q61035	Histidine--tRNA ligase, cytoplasmic	1.00	0.82
Timm8a1	Q9WVA2	Mitochondrial import inner membrane translocase subunit Tim8 A	1.00	0.89
Ring1	O35730	E3 ubiquitin-protein ligase RING1	1.00	0.93
Lgals3	P16110	Galectin-3	1.00	0.91
Dusp3	B1AQF4	Dual-specificity protein phosphatase 3	1.00	0.92
Zc3h18	G3X8T2	RIKEN cDNA 5830416A07, isoform CRA_c	1.00	0.85
Cops7b	Q8BV13	COP9 signalosome complex subunit 7b	1.00	0.91
Slfn9	B1ARD6	Schlafen family member 9	1.00	0.91
Nfyc	P70353	Nuclear transcription factor Y subunit gamma	1.00	0.81
Blmh	Q8R016	Bleomycin hydrolase	1.00	0.80
Shcbp1	Q9Z179	SHC SH2 domain-binding protein 1	1.00	0.95
Trmt5	Q9D0C4	tRNA (guanine(37)-N1)-methyltransferase	1.00	0.92
Mettl13	Q91YR5	Methyltransferase-like protein 13	1.00	0.89
Urgcp	Q5NCI0	Up-regulator of cell proliferation	1.00	0.92
Arsb	A0A0R4J138	Arylsulfatase B	1.00	0.78
Pold1	P52431	DNA polymerase delta catalytic subunit	1.00	0.89
Snx8	Q8CFD4	Sorting nexin-8	1.00	0.88
Oxsr1	Q6P9R2	Serine/threonine-protein kinase OSR1	1.00	0.86
Prpf19	Q99KP6	Pre-mRNA-processing factor 19	1.00	0.88
Rps6kc1	E9QMX4	Ribosomal protein S6 kinase delta-1	1.00	0.88
Cpsf7	Q8BTV2	Cleavage and polyadenylation specificity factor subunit 7	1.00	0.84
Stau1	A2A5S3	Double-stranded RNA-binding protein Staufen homolog 1	1.00	0.87
Got2	P05202	Aspartate aminotransferase, mitochondrial	1.00	0.89
Srp72	F8VQC1	Signal recognition particle subunit SRP72	1.00	0.81
Chchd5	Q9CQP3	Coiled-coil-helix-coiled-coil-helix domain-containing protein 5	1.00	0.90
	Q8BGC1	UPF0489 protein C5orf22 homolog	1.00	0.95
Tarsl2	Q8BLY2	Probable threonine--tRNA ligase 2, cytoplasmic	1.00	0.96
Hnrnpul2	Q00PI9	Heterogeneous nuclear ribonucleoprotein U-like protein 2	1.00	0.77
Csk	P41241	Tyrosine-protein kinase CSK	1.00	0.86
Epb41l1	A2AUK7	Band 4.1-like protein 1	1.00	0.85
Casp9	Q8C3Q9	Caspase-9	1.00	0.84
Ppfibp1	Q8C8U0	Liprin-beta-1	1.00	0.86
Vps11	Q91W86	Vacuolar protein sorting-associated protein 11 homolog	1.00	0.88
Hspa5	P20029	Endoplasmic reticulum chaperone BiP	1.00	0.82
Vps18	Q8R307	Vacuolar protein sorting-associated protein 18 homolog	1.00	0.83
Ggnbp2	Q5SV77	Gametogenetin-binding protein 2	1.00	0.96
Heatr5b	Q8C547	HEAT repeat-containing protein 5B	1.00	0.89
Nhlrc2	Q8BZW8	NHL repeat-containing protein 2	1.00	0.89
Nup37	Q9CWU9	Nucleoporin Nup37	1.00	0.85
Hectd4	E9Q2E4	HECT domain E3 ubiquitin protein ligase 4	1.00	0.95
Ucp2	P70406	Mitochondrial uncoupling protein 2	1.00	0.97
Cd84	E9Q9E8	SLAM family member 5	1.00	0.87
Ythdf1	A2AWN8	YTH domain family 1, isoform CRA_a	1.00	0.90
Mrpl58	Q8R035	Peptidyl-tRNA hydrolase ICT1, mitochondrial	1.00	0.89
Cyc1	Q9D0M3	Cytochrome c1, heme protein, mitochondrial	1.00	0.84
Syne2	Q6ZWQ0	Nesprin-2	1.00	0.96
Ndufb8	Q9D6J5	NADH dehydrogenase [ubiquinone] 1 beta subcomplex subunit 8, mitochondrial	1.00	0.92
Dhfr	P00375	Dihydrofolate reductase	1.00	0.92
Trappc2l	Q9JME7	Trafficking protein particle complex subunit 2-like protein	1.00	0.92
Cpsf1	Q9EPU4	Cleavage and polyadenylation specificity factor subunit 1	1.00	0.87
Tcf25	Q8R3L2	Transcription factor 25	1.00	0.86
Mapre2	Q8R001	Microtubule-associated protein RP/EB family member 2	1.00	0.86
Aldoc	P05063	Fructose-bisphosphate aldolase C	1.00	0.86
Acap2	A0A338P6P6	Arf-GAP with coiled-coil, ANK repeat and PH domain-containing protein 2	1.00	0.88
Phgdh	Q61753	D-3-phosphoglycerate dehydrogenase	1.00	0.92
Plxnb2	B2RXS4	Plexin-B2	1.00	0.81
Plaur	P35456	Urokinase plasminogen activator surface receptor	1.00	0.83
Camk2g	Q923T9	Calcium/calmodulin-dependent protein kinase type II subunit gamma	1.00	0.84

Ppil2	Q9D787	RING-type E3 ubiquitin-protein ligase PPIL2	1.00	0.86
Ctif	E9Q1U6	CBP80/20-dependent translation initiation factor	1.00	0.93
Tmem181a	A0A338P7C9	Transmembrane protein 181A	1.00	0.85
Ttf2	Q5NC05	Transcription termination factor 2	1.00	0.91
Grwd1	Q810D6	Glutamate-rich WD repeat-containing protein 1	1.00	0.89
Rcor3	A0A0A6YXM5	REST corepressor 3	1.00	0.95
Hibadh	Q99L13	3-hydroxyisobutyrate dehydrogenase, mitochondrial	1.00	0.90
Hsph1	Q61699	Heat shock protein 105 kDa	1.00	0.87
Polr2f	P61219	DNA-directed RNA polymerases I, II, and III subunit RPABC2	1.00	0.81
Fasn	P19096	Fatty acid synthase	1.00	0.76
Ints11	Q9CWS4	Integrator complex subunit 11	1.00	0.85
Actr10	Q9QZB7	Actin-related protein 10	1.00	0.87
Sucla2	Q9Z2I9	Succinate--CoA ligase [ADP-forming] subunit beta, mitochondrial	1.00	0.77
Rangap1	P46061	Ran GTPase-activating protein 1	1.00	0.81
Nek9	Q8K1R7	Serine/threonine-protein kinase Nek9	1.00	0.83
Nagk	Q9D997	N-acetyl-D-glucosamine kinase	1.00	0.87
Klc4	Q9DBS5	Kinesin light chain 4	1.00	0.76
Il16	O54824	Pro-interleukin-16	1.00	0.91
Fam173a	Q501J2	Protein N-lysine methyltransferase FAM173A	1.00	0.92
Uckl1	Q91YL3	Uridine-cytidine kinase-like 1	1.00	0.85
Psmb7	P70195	Proteasome subunit beta type-7	1.00	0.73
Crot	Q9DC50	Peroxisomal carnitine O-octanoyltransferase	1.00	0.85
Mipep	A6H611	Mitochondrial intermediate peptidase	1.00	0.88
0610010K14Rik	D3Z687	RIKEN cDNA 0610010K14 gene	1.00	0.89
Rpl5	P47962	60S ribosomal protein L5	1.00	0.89
Actr6	A0A0R4J009	Actin-related protein 6	1.00	0.93
Mib1	Q80SY4	E3 ubiquitin-protein ligase MIB1	1.00	0.91
Bach1	P97302	Transcription regulator protein BACH1	1.00	0.95
Ythdc1	E9Q5K9	YTH domain-containing protein 1	1.00	0.85
Prkacb	P68181	cAMP-dependent protein kinase catalytic subunit beta	1.00	0.73
Etfdh	Q921G7	Electron transfer flavoprotein-ubiquinone oxidoreductase, mitochondrial	1.00	0.83
Stk3	Q9JI10	Serine/threonine-protein kinase 3	1.01	0.89
Otulin	Q3UCV8	Ubiquitin thioesterase otulin	1.01	0.74
Golga3	E9QP99	Golgin subfamily A member 3	1.01	0.84
Zfyve26	Q5DU37	Zinc finger FYVE domain-containing protein 26	1.01	0.91
Mcm2	P97310	DNA replication licensing factor MCM2	1.01	0.86
Emb	P21995	Embigin	1.01	0.85
Recql	Q9Z129	ATP-dependent DNA helicase Q1	1.01	0.88
Kif16b	E7FLY0	Kinesin superfamily protein 16B	1.01	0.89
Gmps	Q3THK7	GMP synthase [glutamine-hydrolyzing]	1.01	0.85
Fus	P56959	RNA-binding protein FUS	1.01	0.80
Sae1	Q9R1T2	SUMO-activating enzyme subunit 1	1.01	0.80
Chst12	Q99LL3	Carbohydrate sulfotransferase 12	1.01	0.88
Creld2	Q9CYA0	Cysteine-rich with EGF-like domain protein 2	1.01	0.87
Gtf2h4	O70422	General transcription factor IIH subunit 4	1.01	0.95
Uevld	Q3U1V6	Ubiquitin-conjugating enzyme E2 variant 3	1.01	0.80
Uchl1	Q9R0P9	Ubiquitin carboxyl-terminal hydrolase isozyme L1	1.01	0.85
Rgs14	P97492	Regulator of G-protein signaling 14	1.01	0.86
Kcmf1	Q80UY2	E3 ubiquitin-protein ligase KCMF1	1.01	0.90
Gtf2i	Q9ESZ8	General transcription factor II-I	1.01	0.89
Ppat	Q8CIH9	Amidophosphoribosyltransferase	1.01	0.91
Ubxn6	Q99PL6	UBX domain-containing protein 6	1.01	0.88
Kdm1a	A3KG93	Lysine-specific histone demethylase 1A	1.01	0.87
Dis3l	Q8C0S1	DIS3-like exonuclease 1	1.01	0.90
Chmp5	Q9D7S9	Charged multivesicular body protein 5	1.01	0.82
Comt	O88587	Catechol O-methyltransferase	1.01	0.82
Igsf8	A0A0R4J117	Immunoglobulin superfamily member 8	1.01	0.94
Atp6v1h	Q8BVE3	V-type proton ATPase subunit H	1.01	0.91
Kif4	P33174	Chromosome-associated kinesin KIF4	1.01	0.92
Cyfip1	Q7TMB8	Cytoplasmic FMR1-interacting protein 1	1.01	0.76
Idh3a	A0A1L1STE6	Isocitrate dehydrogenase [NAD] subunit, mitochondrial	1.01	0.87
Drg2	Q9QXB9	Developmentally-regulated GTP-binding protein 2	1.01	0.86
Dhrs4	Q99LB2	Dehydrogenase/reductase SDR family member 4	1.01	0.82
Slc9a9	Q8BZ00	Sodium/hydrogen exchanger 9	1.01	0.84
Gbp4	Q61107	Guanylate-binding protein 4	1.01	0.94
Anapc4	Q91W96	Anaphase-promoting complex subunit 4	1.01	0.86
Gmeb1	Q9JL60	Glucocorticoid modulatory element-binding protein 1	1.01	0.90
Tusc3	Q8BTV1	Tumor suppressor candidate 3	1.01	0.82
Cbr1	P48758	Carbonyl reductase [NADPH] 1	1.01	0.89
Smcr8	Q3UMB5	Guanine nucleotide exchange protein SMCR8	1.01	0.84
Dip2a	F8WI56	Disco-interacting protein 2 homolog A	1.01	0.92
Lpp	Q8BFW7	Lipoma-preferred partner homolog	1.01	0.92
Parp9	Q8CAS9	Poly [ADP-ribose] polymerase 9	1.01	0.86

Pdia4	A0A0R4J0Z1	Protein disulfide-isomerase A4	1.01	0.81
Dock8	Q8C147	Dedicator of cytokinesis protein 8	1.01	0.77
Ifih1	Q8R5F7	Interferon-induced helicase C domain-containing protein 1	1.01	0.88
Rbm33	Q9CXK9	RNA-binding protein 33	1.01	0.91
Nsl1	E9QME3	Kinetochore-associated protein NSL1 homolog	1.01	0.95
Celf1	A0A0R4J0T5	CUG triplet repeat, RNA binding protein 1, isoform CRA_b	1.01	0.88
Gm3839	S4R1W1	Glyceraldehyde-3-phosphate dehydrogenase	1.01	0.91
Emilin1	Q99K41	EMILIN-1	1.01	0.96
Cryzl1	Q921W4	Quinone oxidoreductase-like protein 1	1.01	0.81
Upf2	A2AT37	UPF2 regulator of nonsense transcripts homolog (Yeast)	1.01	0.87
Nploc4	P60670	Nuclear protein localization protein 4 homolog	1.01	0.77
Prps2	Q9CS42	Ribose-phosphate pyrophosphokinase 2	1.01	0.86
Ptpa	P58389	Serine/threonine-protein phosphatase 2A activator	1.01	0.75
Cog6	A0A0R4J0L5	Conserved oligomeric Golgi complex subunit 6	1.01	0.82
Sipa1l3	G3X9J0	Signal-induced proliferation-associated 1-like protein 3	1.01	0.93
Padi4	Q9Z183	Protein-arginine deiminase type-4	1.01	0.93
Qtrt2	B8ZXI1	Queuine tRNA-ribosyltransferase accessory subunit 2	1.01	0.94
Tgfbr2	Q62312	TGF-beta receptor type-2	1.01	0.94
Blnk	Q9QUN3	B-cell linker protein	1.01	0.84
Cox7c	P17665	Cytochrome c oxidase subunit 7C, mitochondrial	1.01	0.90
Smn1	P97801	Survival motor neuron protein	1.01	0.88
Usp24	E9PV45	Ubiquitin carboxyl-terminal hydrolase 24	1.01	0.90
Fcgr1	P26151	High affinity immunoglobulin gamma Fc receptor I	1.01	0.84
Ndufs4	E9QPX3	NADH dehydrogenase [ubiquinone] iron-sulfur protein 4, mitochondrial	1.01	0.80
Hnrnpa1	Q5EBP8	Heterogeneous nuclear ribonucleoprotein A1	1.01	0.88
Prcp	Q7TMR0	Lysosomal Pro-X carboxypeptidase	1.01	0.88
Tbck	E9Q1W7	TBC domain-containing protein kinase-like protein	1.01	0.91
Rprd2	Q6NXI6	Regulation of nuclear pre-mRNA domain-containing protein 2	1.01	0.83
Ppp5c	Q60676	Serine/threonine-protein phosphatase 5	1.01	0.83
Enpp1	A0A0R4J1Q7	Ectonucleotide pyrophosphatase/phosphodiesterase 1, isoform CRA_a	1.01	0.78
Bcas2	Q9D287	Pre-mRNA-splicing factor SPF27	1.01	0.87
Pitpnc1	Q8K4R4	Cytoplasmic phosphatidylinositol transfer protein 1	1.01	0.89
Ppm1a	P49443	Protein phosphatase 1A	1.01	0.80
Ppp4r3a	E9Q481	Serine/threonine-protein phosphatase 4 regulatory subunit 3A	1.01	0.85
Pdlim5	Q8CI51	PDZ and LIM domain protein 5	1.01	0.83
Cwf19l1	Q8CI33	CWF19-like protein 1	1.01	0.81
Eif3i	Q9QZD9	Eukaryotic translation initiation factor 3 subunit I	1.01	0.82
Pter	Q60866	Phosphotriesterase-related protein	1.01	0.79
Wapl	Q65Z40	Wings apart-like protein homolog	1.01	0.80
Nars	Q8BP47	Asparagine--tRNA ligase, cytoplasmic	1.01	0.75
Mroh1	E0CZ22	Maestro heat-like repeat family member 1	1.01	0.83
Sos1	Q62245	Son of sevenless homolog 1	1.01	0.92
Axl	Q00993	Tyrosine-protein kinase receptor UFO	1.01	0.84
Rab5b	P61021	Ras-related protein Rab-5B	1.01	0.83
Zwilch	G3X9Z0	Protein zwilch homolog	1.01	0.87
Alg2	Q9DBE8	Alpha-1,3/1,6-mannosyltransferase ALG2	1.01	0.80
Lrch3	Q8BVU0	Leucine-rich repeat and calponin homology domain-containing protein 3	1.01	0.87
Spout1	Q3UHX9	Putative methyltransferase C9orf114 homolog	1.01	0.89
Maz	A0A0U1RNL9	Myc-associated zinc finger protein	1.01	0.92
Snap47	Q8R570	Synaptosomal-associated protein 47	1.01	0.88
Itpripl2	Q3UV16	Inositol 1,4,5-trisphosphate receptor-interacting protein-like 2	1.01	0.96
Ankrd40	Q5SUE8	Ankyrin repeat domain-containing protein 40	1.01	0.82
Arl2	Q9D0J4	ADP-ribosylation factor-like protein 2	1.01	0.85
Atp6v0d2	Q80SY3	V-type proton ATPase subunit d 2	1.01	0.91
Glyr1	D3YYT1	Putative oxidoreductase GLYR1	1.01	0.72
Dbt	P53395	Lipoamide acyltransferase component of branched-chain alpha-keto acid dehydrogenase complex, mitochondrial	1.01	0.84
Zc3h7b	F8VPP8	Zinc finger CCCH type-containing 7B	1.01	0.90
Crtc1	Q68ED7	CREB-regulated transcription coactivator 1	1.01	0.85
Rassf5	Q5EBH1	Ras association domain-containing protein 5	1.01	0.91
Txndc5	Q91W90	Thioredoxin domain-containing protein 5	1.01	0.79
Btaf1	E9QAE3	B-TFIID TATA-box-binding protein-associated factor 1	1.01	0.88
Acaca	Q5SWU9	Acetyl-CoA carboxylase 1	1.01	0.85
Dctn1	E9Q586	Dynactin subunit 1	1.01	0.74
Vps4b	P46467	Vacuolar protein sorting-associated protein 4B	1.01	0.70
Gnas	Q6R0H7	Guanine nucleotide-binding protein G(s) subunit alpha isoforms XLas	1.01	0.71
Fnta	Q61239	Protein farnesyltransferase/geranylgeranyltransferase type-1 subunit alpha	1.01	0.78
Vps25	Q9CQ80	Vacuolar protein-sorting-associated protein 25	1.01	0.82
Acp2	P24638	Lysosomal acid phosphatase	1.01	0.67
Prex1	Q69ZK0	Phosphatidylinositol 3,4,5-trisphosphate-dependent Rac exchanger 1 protein	1.01	0.87
Slirp	Q9D8T7	SRA stem-loop-interacting RNA-binding protein, mitochondrial	1.01	0.93
Supt6h	Q62383	Transcription elongation factor SPT6	1.01	0.83
Spata5	Q3UMC0	ATPase family protein 2 homolog	1.01	0.84
Appl1	Q8K3H0	DCC-interacting protein 13-alpha	1.01	0.85

Tmub1	E9QN77	Transmembrane and ubiquitin-like domain-containing protein 1	1.01	0.90
Amdhd2	Q8JZV7	N-acetylglucosamine-6-phosphate deacetylase	1.01	0.79
Vdac2	Q60930	Voltage-dependent anion-selective channel protein 2	1.01	0.85
Fam129a	Q3UW53	Protein Niban	1.01	0.78
Apaf1	O88879	Apoptotic protease-activating factor 1	1.01	0.85
Traf3ip2	Q8N7N6	TRAF3-interacting protein 2	1.01	0.94
Acyp1	E9QJT5	Acylphosphatase	1.01	0.77
Tbc1d1	Q60949	TBC1 domain family member 1	1.01	0.83
Eif5a	P63242	Eukaryotic translation initiation factor 5A-1	1.01	0.95
Rbms1	E9PZ21	RNA-binding motif, single-stranded-interacting protein 1	1.01	0.77
Stip1	Q60864	Stress-induced-phosphoprotein 1	1.01	0.81
Ddhd2	Q80Y98	Phospholipase DDHD2	1.01	0.88
Pelo	Q80X73	Protein pelota homolog	1.01	0.87
Glo1	Q9CPU0	Lactoylglutathione lyase	1.01	0.76
Arid4a	F8VPQ2	AT-rich interactive domain-containing protein 4A	1.01	0.87
Ints1	A0A0G2JH17	Integrator complex subunit 1	1.01	0.88
Txnrd2	A0A0M3HEQ0	Thioredoxin reductase 2, mitochondrial	1.01	0.86
Naa15	G3X8Y3	N-alpha-acetyltransferase 15, NatA auxiliary subunit	1.01	0.80
Arpc5	Q9CPW4	Actin-related protein 2/3 complex subunit 5	1.01	0.78
Pcgf5	Q3UK78	Polycomb group RING finger protein 5	1.01	0.91
G3bp2	P97379	Ras GTPase-activating protein-binding protein 2	1.01	0.83
Hspa4l	P48722	Heat shock 70 kDa protein 4L	1.01	0.72
Rassf2	Q8BMS9	Ras association domain-containing protein 2	1.01	0.77
Psmc2	Q8BVQ9	26S proteasome regulatory subunit 7	1.01	0.74
Zranb3	Q6NZP1	DNA annealing helicase and endonuclease ZRANB3	1.01	0.90
Pls3	Q99K51	Plastin-3	1.01	0.80
Smc3	Q9CW03	Structural maintenance of chromosomes protein 3	1.01	0.82
Dnmt3a	O88508	DNA (cytosine-5)-methyltransferase 3A	1.01	0.82
Msr1	P30204	Macrophage scavenger receptor types I and II	1.01	0.86
Lsm6	P62313	U6 snRNA-associated Sm-like protein LSm6	1.01	0.82
Alg3	Q8K2A8	Dol-P-Man:Man(5)GlcNAc(2)-PP-Dol alpha-1,3-mannosyltransferase	1.01	0.91
Abcd4	O89016	ATP-binding cassette sub-family D member 4	1.01	0.89
Nckap1l	Q8K1X4	Nck-associated protein 1-like	1.01	0.69
Smarcb1	Q9Z0H3	SWI/SNF-related matrix-associated actin-dependent regulator of chromatin subfamily B member 1	1.01	0.79
Ech1	O35459	Delta(3,5)-Delta(2,4)-dienoyl-CoA isomerase, mitochondrial	1.01	0.78
Cox11	Q6P8I6	Cytochrome c oxidase assembly protein COX11, mitochondrial	1.01	0.83
Chmp1b1	Q99LU0	Charged multivesicular body protein 1b-1	1.01	0.82
Gars	Q9CZD3	Glycine--tRNA ligase	1.01	0.74
Utp18	Q5SSI6	U3 small nucleolar RNA-associated protein 18 homolog	1.01	0.83
Trappc11	B2RXC1	Trafficking protein particle complex subunit 11	1.01	0.83
Atg12	Q9CQY1	Ubiquitin-like protein ATG12	1.01	0.64
Eps15l1	Q60902	Epidermal growth factor receptor substrate 15-like 1	1.01	0.84
Hagh	G5E8T9	Hydroxyacyl glutathione hydrolase	1.01	0.80
Yipf5	Q9EQQ2	Protein YIPF5	1.01	0.87
Ensa	P60840	Alpha-endosulfine	1.01	0.75
Snw1	A0A0B4J1E2	SNW domain-containing protein 1	1.01	0.75
Acp5	Q05117	Tartrate-resistant acid phosphatase type 5	1.01	0.74
Nfatc2	Q60591	Nuclear factor of activated T-cells, cytoplasmic 2	1.01	0.79
Bcs1l	Q9CZP5	Mitochondrial chaperone BCS1	1.01	0.89
Ecpas	Q6PDI5	Proteasome adapter and scaffold protein ECM29	1.01	0.71
Synrg	V9GX40	Synergin gamma	1.01	0.81
Prkag1	O54950	5'-AMP-activated protein kinase subunit gamma-1	1.01	0.79
Wdr13	Q91V09	WD repeat-containing protein 13	1.01	0.85
Pmm2	Q9Z2M7	Phosphomannomutase 2	1.01	0.69
Ctbp1	O88712	C-terminal-binding protein 1	1.01	0.81
Rbm43	Q99J64	RNA-binding protein 43	1.01	0.95
Inppl1	Q6P549	Phosphatidylinositol 3,4,5-trisphosphate 5-phosphatase 2	1.01	0.81
Dglucy	E9QMK9	D-glutamate cyclase, mitochondrial	1.01	0.81
Akt1s1	E9QKI4	Proline-rich AKT1 substrate 1	1.01	0.83
Pcyox1	Q9CQF9	Prenylcysteine oxidase	1.01	0.70
Thop1	A0A0R4IZY0	Thimet oligopeptidase	1.01	0.82
Snf8	Q9CZ28	Vacuolar-sorting protein SNF8	1.01	0.74
Eny2	Q9JIX0	Transcription and mRNA export factor ENY2	1.01	0.80
Nup107	Q8BH74	Nuclear pore complex protein Nup107	1.01	0.76
Gpd1	P13707	Glycerol-3-phosphate dehydrogenase [NAD(+)], cytoplasmic	1.01	0.93
Atg2a	Q6P4T0	Autophagy-related protein 2 homolog A	1.01	0.91
Rad50	P70388	DNA repair protein RAD50	1.01	0.84
Golga1	Q9CW79	Golgin subfamily A member 1	1.01	0.89
Psmc1	P62192	26S proteasome regulatory subunit 4	1.01	0.70
Eif1ax	Q8BMJ3	Eukaryotic translation initiation factor 1A, X-chromosomal	1.01	0.80
Neu3	Q9JMH7	Sialidase-3	1.01	0.96
Gas7	Q60780	Growth arrest-specific protein 7	1.01	0.66
Mgst3	Q9CPU4	Microsomal glutathione S-transferase 3	1.01	0.85

Ado	Q6PDY2	2-aminoethanethiol dioxygenase	1.01	0.75
Eea1	Q8BL66	Early endosome antigen 1	1.01	0.79
Sf3a3	Q9D554	Splicing factor 3A subunit 3	1.01	0.80
Marcks	P26645	Myristoylated alanine-rich C-kinase substrate	1.01	0.80
Eif4ebp1	Q60876	Eukaryotic translation initiation factor 4E-binding protein 1	1.01	0.85
Pdcd10	Q8VE70	Programmed cell death protein 10	1.01	0.78
Med29	Q9DB91	Mediator of RNA polymerase II transcription subunit 29	1.01	0.81
Pgs1	Q8BHF7	CDP-diacylglycerol--glycerol-3-phosphate 3-phosphatidyltransferase, mitochondrial	1.01	0.86
Rnf213	E9Q555	E3 ubiquitin-protein ligase RNF213	1.01	0.81
Pgp	Q8CHP8	Glycerol-3-phosphate phosphatase	1.01	0.72
Nsf	P46460	Vesicle-fusing ATPase	1.01	0.63
Qrsl1	Q9CZN8	Glutamyl-tRNA(Gln) amidotransferase subunit A, mitochondrial	1.01	0.87
Pccb	Q99MN9	Propionyl-CoA carboxylase beta chain, mitochondrial	1.01	0.84
Cbfb	Q08024	Core-binding factor subunit beta	1.01	0.89
Ahnak	E9Q616	AHNAK nucleoprotein (desmoyokin)	1.01	0.68
Ppp1r12c	Q3UMT1	Protein phosphatase 1 regulatory subunit 12C	1.01	0.79
Ruvbl2	Q9WTM5	RuvB-like 2	1.01	0.77
Ap1b1	O35643	AP-1 complex subunit beta-1	1.01	0.73
Eprs	Q8CGC7	Bifunctional glutamate/proline--tRNA ligase	1.01	0.81
Rbm45	Q8BHN5	RNA-binding protein 45	1.01	0.84
Pcyt1a	P49586	Choline-phosphate cytidylyltransferase A	1.01	0.56
Heatr5a	Q5PRF0	HEAT repeat-containing protein 5A	1.01	0.79
U2surp	Q6NV83	U2 snRNP-associated SURP motif-containing protein	1.01	0.78
Vps13c	Q8BX70	Vacuolar protein sorting-associated protein 13C	1.01	0.79
Nelfa	Q8BG30	Negative elongation factor A	1.01	0.79
Smad2	Q62432	Mothers against decapentaplegic homolog 2	1.01	0.82
Akr7a2	Q8CG76	Aflatoxin B1 aldehyde reductase member 2	1.01	0.85
Gmip	Q6PGG2	GEM-interacting protein	1.01	0.79
Iars	Q8BU30	Isoleucine--tRNA ligase, cytoplasmic	1.01	0.75
Kpna1	Q60960	Importin subunit alpha-5	1.01	0.71
Epdr1	Q99M71	Mammalian ependymin-related protein 1	1.01	0.76
Wwp1	Q8BZZ3	NEDD4-like E3 ubiquitin-protein ligase WWP1	1.01	0.89
Btd	A0A0R4J131	Biotinidase	1.01	0.82
H3f3c	P02301	Histone H3.3C	1.01	0.85
Grsf1	Q8C5Q4	G-rich sequence factor 1	1.01	0.83
Nit2	Q9JHW2	Omega-amidase NIT2	1.01	0.80
Erlin2	Q8BFZ9	Erlin-2	1.01	0.82
Sars2	Q9JJL8	Serine--tRNA ligase, mitochondrial	1.01	0.81
Ppa2	Q91VM9	Inorganic pyrophosphatase 2, mitochondrial	1.01	0.81
Fyb1	O35601	FYN-binding protein 1	1.01	0.79
Pnpla8	Q8K1N1	Calcium-independent phospholipase A2-gamma	1.01	0.86
Bysl	O54825	Bystin	1.01	0.85
Hspa9	P38647	Stress-70 protein, mitochondrial	1.01	0.72
Rbbp9	O88851	Putative hydrolase RBBP9	1.01	0.85
Lancl1	O89112	LanC-like protein 1	1.01	0.87
Psmb8	P28063	Proteasome subunit beta type-8	1.01	0.71
Vps33b	P59016	Vacuolar protein sorting-associated protein 33B	1.01	0.82
Htatsf1	Q8BGC0	HIV Tat-specific factor 1 homolog	1.01	0.72
Srrt	Q99MR6	Serrate RNA effector molecule homolog	1.01	0.76
Nrbp1	Q99J45	Nuclear receptor-binding protein	1.01	0.74
Chac2	Q9CQG1	Putative glutathione-specific gamma-glutamylcyclotransferase 2	1.01	0.93
Galk2	Q68FH4	N-acetylgalactosamine kinase	1.01	0.75
Dync1h1	Q9JHU4	Cytoplasmic dynein 1 heavy chain 1	1.01	0.68
Bckdhb	Q6P3A8	2-oxoisovalerate dehydrogenase subunit beta, mitochondrial	1.01	0.92
Uhrf2	Q7TMI3	E3 ubiquitin-protein ligase UHRF2	1.01	0.78
Lrrc57	Q8JZX5	Leucine rich repeat containing 57	1.01	0.87
Cog7	A0A0R4J0Q9	Conserved oligomeric Golgi complex subunit 7	1.01	0.77
Nle1	Q8VEJ4	Notchless protein homolog 1	1.01	0.75
Acads	Q07417	Short-chain specific acyl-CoA dehydrogenase, mitochondrial	1.01	0.75
Gfm2	Q8R2Q4	Ribosome-releasing factor 2, mitochondrial	1.01	0.85
Ccdc12	Q8R344	Coiled-coil domain-containing protein 12	1.01	0.77
Srp9	P49962	Signal recognition particle 9 kDa protein	1.01	0.73
Ppp1r10	Q80W00	Serine/threonine-protein phosphatase 1 regulatory subunit 10	1.01	0.64
Tyms	P07607	Thymidylate synthase	1.01	0.61
Wars2	Q9CYK1	Tryptophan--tRNA ligase, mitochondrial	1.01	0.83
Irak3	Q8K4B2	Interleukin-1 receptor-associated kinase 3	1.01	0.79
Ubr3	F6QC57	E3 ubiquitin-protein ligase UBR3	1.01	0.86
Ap5m1	A0A0R4J0K9	AP-5 complex subunit mu-1	1.01	0.90
Tsn	Q62348	Translin	1.01	0.73
Rab10	P61027	Ras-related protein Rab-10	1.01	0.78
Znf593	Q9DB42	Zinc finger protein 593	1.01	0.87
Exoc3	Q6KAR6	Exocyst complex component 3	1.01	0.66
Dhodh	O35435	Dihydroorotate dehydrogenase (quinone), mitochondrial	1.01	0.84

Rab34	B1AQD4	Ras-related protein Rab-34 (Fragment)	1.01	0.78
Gm9774	A0A0A6YVU8	MCG119397	1.01	0.63
Cct6a	P80317	T-complex protein 1 subunit zeta	1.01	0.78
Tmem41a	Q9D8U2	Transmembrane protein 41A	1.01	0.92
Ddrgk1	Q80WW9	DDRGK domain-containing protein 1	1.01	0.79
Wdr37	Q8CBE3	WD repeat-containing protein 37	1.01	0.79
Sptbn1	Q62261	Spectrin beta chain, non-erythrocytic 1	1.01	0.78
Arhgef11	Q68FM7	Rho guanine nucleotide exchange factor (GEF) 11	1.01	0.83
Bri3bp	Q8BXV2	BRI3-binding protein	1.01	0.84
Znf148	Q61624	Zinc finger protein 148	1.01	0.74
Ikbkg	Q7TSS3	Inhibitor of kappaB kinase gamma	1.01	0.74
Creb1	Q01147	Cyclic AMP-responsive element-binding protein 1	1.01	0.80
Mrpl46	Q9EQI8	39S ribosomal protein L46, mitochondrial	1.01	0.87
Slc15a3	Q8BPX9	Solute carrier family 15 member 3	1.01	0.82
Iqsec1	E9PUA3	IQ motif and SEC7 domain-containing protein 1	1.01	0.85
Esf1	Q3V1V3	ESF1 homolog	1.01	0.79
Lrrfip2	E9QN52	Leucine-rich repeat flightless-interacting protein 2	1.01	0.80
Actr1b	Q8R5C5	Beta-centractin	1.01	0.88
Anapc1	P53995	Anaphase-promoting complex subunit 1	1.01	0.81
Nedd4l	Q8CFI0	E3 ubiquitin-protein ligase NEDD4-like	1.01	0.78
Rbck1	Q9WUB0	RanBP-type and C3HC4-type zinc finger-containing protein 1	1.01	0.80
Rtfdc1	Q99K95	Protein RTF2 homolog	1.01	0.79
Srp54	P14576	Signal recognition particle 54 kDa protein	1.01	0.77
Hnrnpu	Q8VEK3	Heterogeneous nuclear ribonucleoprotein U	1.01	0.72
Dld	O08749	Dihydrolipoyl dehydrogenase, mitochondrial	1.01	0.61
Uqcrc2	Q9DB77	Cytochrome b-c1 complex subunit 2, mitochondrial	1.01	0.84
Nosip	Q9D6T0	Nitric oxide synthase-interacting protein	1.01	0.83
Grcc10	O35127	Protein C10	1.01	0.84
Mitf	Q08874	Microphthalmia-associated transcription factor	1.01	0.81
Ttc38	A3KMP2	Tetratricopeptide repeat protein 38	1.01	0.83
Akap10	O88845	A-kinase anchor protein 10, mitochondrial	1.01	0.75
Yars	A2A7S7	Tyrosine--tRNA ligase	1.01	0.77
Trim16	Q5SVT2	Tripartite motif-containing protein 16	1.01	0.92
Desi2	Q9D291	Deubiquitinase DESI2	1.01	0.91
Vps33a	Q9D2N9	Vacuolar protein sorting-associated protein 33A	1.01	0.77
Ranbp3	Q9CT10	Ran-binding protein 3	1.01	0.67
Birc6	O88738	Baculoviral IAP repeat-containing protein 6	1.01	0.76
Cyp51a1	Q8K0C4	Lanosterol 14-alpha demethylase	1.01	0.90
Naxd	K3W4M4	ATP-dependent (S)-NAD(P)H-hydrate dehydratase	1.01	0.84
Lamp1	P11438	Lysosome-associated membrane glycoprotein 1	1.01	0.80
Dhx38	Q80X98	DEAH (Asp-Glu-Ala-His) box polypeptide 38	1.01	0.81
Flad1	Q8R123	FAD synthase	1.01	0.74
Bag3	Q9JLV1	BAG family molecular chaperone regulator 3	1.01	0.77
Mpi	Q924M7	Mannose-6-phosphate isomerase	1.01	0.77
Dpp9	Q8BVG4	Dipeptidyl peptidase 9	1.01	0.76
Rbbp4	Q60972	Histone-binding protein RBBP4	1.01	0.97
Bloc1s6	Q9R0C0	Biogenesis of lysosome-related organelles complex 1 subunit 6	1.01	0.69
Adnp	Q9Z103	Activity-dependent neuroprotector homeobox protein	1.01	0.81
Nup88	Q8CEC0	Nuclear pore complex protein Nup88	1.01	0.78
Flii	Q9JJ28	Protein flightless-1 homolog	1.01	0.72
Pank3	Q8R2W9	Pantothenate kinase 3	1.01	0.71
Commd8	Q9CZG3	COMM domain-containing protein 8	1.01	0.72
Nudt13	Q8JZU0	Nucleoside diphosphate-linked moiety X motif 13	1.01	0.86
Anln	Q8K298	Anillin	1.01	0.72
Pmm1	O35621	Phosphomannomutase 1	1.01	0.85
Cs	Q9CZU6	Citrate synthase, mitochondrial	1.01	0.74
Rps29	P62274	40S ribosomal protein S29	1.01	0.87
Osbpl3	D3YTT6	Oxysterol-binding protein	1.01	0.87
Pld2	Q6NV49	Phospholipase	1.01	0.89
Stx11	Q3U5V8	MCG49559	1.01	0.94
Ap1g1	P22892	AP-1 complex subunit gamma-1	1.01	0.67
Ptk2b	E9Q2A6	Protein-tyrosine kinase 2-beta	1.01	0.78
Acot9	Q9R0X4	Acyl-coenzyme A thioesterase 9, mitochondrial	1.01	0.77
Tut1	Q8R3F9	Speckle targeted PIP5K1A-regulated poly(A) polymerase	1.01	0.92
Grn	Q3U9N4	Granulins	1.01	0.62
Phf8	Q80TJ7	Histone lysine demethylase PHF8	1.01	0.93
Pik3c2a	F8VPL2	Phosphatidylinositol 4-phosphate 3-kinase C2 domain-containing subunit alpha	1.01	0.85
Srgap2	Q91Z67	SLIT-ROBO Rho GTPase-activating protein 2	1.01	0.74
Vps16	G3X8X7	Vacuolar protein sorting 16 (Yeast)	1.01	0.76
Arpp19	E9Q827	cAMP-regulated phosphoprotein 19	1.01	0.82
Gpi	P06745	Glucose-6-phosphate isomerase	1.01	0.81
Mib2	Q8R516	E3 ubiquitin-protein ligase MIB2	1.01	0.88
Crnkl1	P63154	Crooked neck-like protein 1	1.01	0.83

Ptprj	E9Q4S7	Receptor-type tyrosine-protein phosphatase eta	1.01	0.74
Sgk3	Q9ERE3	Serine/threonine-protein kinase Sgk3	1.01	0.83
Slc9a6	A1L3P4	Sodium/hydrogen exchanger	1.01	0.87
Map2k6	P70236	Dual specificity mitogen-activated protein kinase kinase 6	1.01	0.86
Aida	Q8C4Q6	Axin interactor, dorsalization-associated protein	1.01	0.84
Coro1b	Q9WUM3	Coronin-1B	1.01	0.58
Gmpr	Q9DCZ1	GMP reductase 1	1.01	0.88
Cd151	O35566	CD151 antigen	1.01	0.76
Aldh1l1	Q8R0Y6	Cytosolic 10-formyltetrahydrofolate dehydrogenase	1.01	0.76
Oxnad1	Q8VE38	Oxidoreductase NAD-binding domain-containing protein 1	1.01	0.80
Pfn1	P62962	Profilin-1	1.01	0.73
Ndufa3	Q9CQ91	NADH dehydrogenase [ubiquinone] 1 alpha subcomplex subunit 3	1.01	0.80
Pcx	E9QPD7	Pyruvate carboxylase	1.01	0.93
Kdm4b	Q91VY5	Lysine-specific demethylase 4B	1.01	0.85
Plekha1	Q8BUL6	Pleckstrin homology domain-containing family A member 1	1.01	0.87
Cpsf2	O35218	Cleavage and polyadenylation specificity factor subunit 2	1.01	0.81
Ranbp2	Q9ERU9	E3 SUMO-protein ligase RanBP2	1.01	0.75
Mta3	Q924K8	Metastasis-associated protein MTA3	1.01	0.67
Man2c1	Q91W89	Alpha-mannosidase 2C1	1.01	0.81
Nufip2	Q5F2E7	Nuclear fragile X mental retardation-interacting protein 2	1.01	0.65
Pgm2	Q7TSV4	Phosphoglucomutase-2	1.01	0.64
Ap3b1	Q9Z1T1	AP-3 complex subunit beta-1	1.01	0.76
Pa2g4	P50580	Proliferation-associated protein 2G4	1.01	0.76
Vhl	P40338	von Hippel-Lindau disease tumor suppressor	1.01	0.89
Cand1	Q6ZQ38	Cullin-associated NEDD8-dissociated protein 1	1.01	0.63
Ago2	Q8CJG0	Protein argonaute-2	1.01	0.75
Itgb7	P26011	Integrin beta-7	1.01	0.89
Commd9	Q8K2Q0	COMM domain-containing protein 9	1.01	0.72
Ear2	P97425	Eosinophil cationic protein 2	1.01	0.89
Chd4	E9QAS5	Chromodomain-helicase-DNA-binding protein 4	1.01	0.80
Ppp4r1	E9QPR5	Serine/threonine-protein phosphatase 4 regulatory subunit 1	1.01	0.75
Rpl38	Q9JJI8	60S ribosomal protein L38	1.01	0.81
Elp2	Q91WG4	Elongator complex protein 2	1.01	0.86
Parp14	Q2EMV9	Poly [ADP-ribose] polymerase 14	1.01	0.82
Arhgap1	A2AH25	Rho GTPase-activating protein 1	1.01	0.65
Xdh	Q00519	Xanthine dehydrogenase/oxidase	1.01	0.68
Stxbp1	O08599	Syntaxin-binding protein 1	1.01	0.81
Rybp	Q8CCI5	RING1 and YY1-binding protein	1.01	0.73
Fam120b	Q6RI63	Constitutive coactivator of peroxisome proliferator-activated receptor gamma	1.01	0.67
Exosc7	Q9D0M0	Exosome complex exonuclease RRP42	1.01	0.66
Pqbp1	Q91VJ5	Polyglutamine-binding protein 1	1.01	0.75
Iqgap3	F8VQ29	IQ motif-containing GTPase-activating protein 3	1.01	0.85
Rbm15	Q0VBL3	RNA-binding protein 15	1.01	0.73
Cnrip1	Q5M8N0	CB1 cannabinoid receptor-interacting protein 1	1.01	0.84
Alpl	P09242	Alkaline phosphatase, tissue-nonspecific isozyme	1.01	0.94
Atxn2l	A0A0U1RPL0	Ataxin-2-like protein	1.01	0.67
Sf3a2	G3UVU2	Splicing factor 3A subunit 2	1.01	0.72
Bphl	Q8R164	Valacyclovir hydrolase	1.01	0.74
Supt16	G3X956	Suppressor of Ty 16	1.01	0.75
Ubxn7	G5E8R8	UBX domain-containing protein 7	1.01	0.83
Ttll12	Q3UDE2	Tubulin--tyrosine ligase-like protein 12	1.01	0.68
Arpc1a	Q9R0Q6	Actin-related protein 2/3 complex subunit 1A	1.01	0.71
Ascc3	E9PZJ8	Activating signal cointegrator 1 complex subunit 3	1.01	0.69
Alkbh5	Q3TSG4	RNA demethylase ALKBH5	1.01	0.76
Stub1	Q9WUD1	STIP1 homology and U box-containing protein 1	1.01	0.61
Ldhb	P16125	L-lactate dehydrogenase B chain	1.01	0.72
Syap1	Q9D5V6	Synapse-associated protein 1	1.01	0.75
Nostrin	Q6WKZ7	Nostrin	1.01	0.80
Slc35a1	Q61420	CMP-sialic acid transporter	1.01	0.83
Cbx5	Q61686	Chromobox protein homolog 5	1.01	0.75
Tab2	Q99K90	TGF-beta-activated kinase 1 and MAP3K7-binding protein 2	1.01	0.86
Ptpn11	P35235	Tyrosine-protein phosphatase non-receptor type 11	1.01	0.63
Snx4	Q91YJ2	Sorting nexin-4	1.01	0.66
Mthfd1l	Q3V3R1	Monofunctional C1-tetrahydrofolate synthase, mitochondrial	1.01	0.75
Cdc123	Q8CII2	Cell division cycle protein 123 homolog	1.01	0.86
Abi3	Q8BYZ1	ABI gene family member 3	1.01	0.84
Gnai3	Q9DC51	Guanine nucleotide-binding protein G(k) subunit alpha	1.01	0.89
Pfkfb3	A7UAK5	6-phosphofructo-2-kinase/fructose-2, 6-biphosphatase 3 splice variant 2	1.01	0.74
Nup54	Q8BTS4	Nuclear pore complex protein Nup54	1.01	0.74
Nudc	O35685	Nuclear migration protein nudC	1.01	0.65
Rbm12	Q8R4X3	RNA-binding protein 12	1.01	0.53
Ndufaf6	A2AIL4	NADH dehydrogenase (ubiquinone) complex I, assembly factor 6	1.01	0.82
Glipr2	Q9CYL5	Golgi-associated plant pathogenesis-related protein 1	1.01	0.80

Ctps1	P70698	CTP synthase 1	1.01	0.74
Arfgap1	Q9EPJ9	ADP-ribosylation factor GTPase-activating protein 1	1.01	0.77
Gm20390	E9PZF0	Nucleoside diphosphate kinase	1.01	0.73
Psd3	A0A1D5RMH7	PH and SEC7 domain-containing protein 3	1.01	0.88
Gclc	P97494	Glutamate--cysteine ligase catalytic subunit	1.01	0.70
Clybl	Q8R4N0	Citramalyl-CoA lyase, mitochondrial	1.01	0.78
Slc16a1	P53986	Monocarboxylate transporter 1	1.01	0.81
Ppp3ca	P63328	Serine/threonine-protein phosphatase 2B catalytic subunit alpha isoform	1.01	0.74
Mindy1	Q76LS9	Ubiquitin carboxyl-terminal hydrolase MINDY-1	1.01	0.78
Pcbd2	Q9CZL5	Pterin-4-alpha-carbinolamine dehydratase 2	1.01	0.77
Lsm8	Q6ZWM4	U6 snRNA-associated Sm-like protein LSm8	1.01	0.81
Lipa	Q9Z0M5	Lysosomal acid lipase/cholesteryl ester hydrolase	1.01	0.85
Mob4	Q6PEB6	MOB-like protein phocein	1.01	0.57
Tapbp	Q3TCU5	Tapasin	1.01	0.82
Aamdc	D3YZD8	Mth938 domain-containing protein	1.01	0.89
Capn7	Q9R1S8	Calpain-7	1.01	0.74
Acad10	Q8K370	Acyl-CoA dehydrogenase family member 10	1.01	0.79
Pfdn6	Q03958	Prefoldin subunit 6	1.01	0.61
Lrrfip1	A0A087WSF5	Leucine-rich repeat flightless-interacting protein 1 (Fragment)	1.01	0.82
Mrpl17	Q9D8P4	39S ribosomal protein L17, mitochondrial	1.01	0.76
Trafd1	Q3UDK1	TRAF-type zinc finger domain-containing protein 1	1.01	0.81
Ubxn1	Q922Y1	UBX domain-containing protein 1	1.01	0.71
Nlrp3	Q8R4B8	NACHT, LRR and PYD domains-containing protein 3	1.01	0.83
Slc4a1ap	E9PX68	Solute carrier family 4 (anion exchanger), member 1, adaptor protein	1.01	0.66
Tns3	Q5SSZ5	Tensin-3	1.01	0.64
Vps26b	Q8C0E2	Vacuolar protein sorting-associated protein 26B	1.01	0.60
Grhpr	Q91Z53	Glyoxylate reductase/hydroxypyruvate reductase	1.01	0.74
Vsp35l	I1E4X5	VPS35 endosomal protein sorting factor-like	1.01	0.59
Nipsnap1	O55125	Protein NipSnap homolog 1	1.01	0.75
Slc25a22	Q9D6M3	Mitochondrial glutamate carrier 1	1.01	0.75
Taf6l	H3BK01	TAF6-like RNA polymerase II p300/CBP-associated factor-associated factor 65 kDa subunit 6L	1.01	0.88
Czib	Q8BHG2	CXXC motif containing zinc binding protein	1.01	0.75
Gbe1	Q9D6Y9	1,4-alpha-glucan-branching enzyme	1.01	0.79
Stxbp3	Q60770	Syntaxin-binding protein 3	1.01	0.68
Utrn	E9Q6R7	Utrophin	1.01	0.81
Papola	Q61183	Poly(A) polymerase alpha	1.01	0.80
Ndufaf7	Q9CWG8	Protein arginine methyltransferase NDUFAF7, mitochondrial	1.01	0.86
Ddx46	Q569Z5	Probable ATP-dependent RNA helicase DDX46	1.01	0.77
Bsg	P18572	Basigin	1.01	0.69
Prim2	P33610	DNA primase large subunit	1.01	0.84
Mogs	Q80UM7	Mannosyl-oligosaccharide glucosidase	1.01	0.68
Osbpl2	Q8BX94	Oxysterol-binding protein-related protein 2	1.01	0.85
Stk25	Q9Z2W1	Serine/threonine-protein kinase 25	1.01	0.78
Smg8	Q8VE18	Protein SMG8	1.01	0.77
Atp5s	Q9CRA7	ATP synthase subunit s, mitochondrial	1.01	0.86
Iigp1	Q9QZ85	Interferon-inducible GTPase 1	1.01	0.98
Acsl4	Q9QUJ7	Long-chain-fatty-acid--CoA ligase 4	1.01	0.71
Fam219b	Q14DQ1	Protein FAM219B	1.01	0.91
Xpnpep1	Q3UE92	X-prolyl aminopeptidase (Aminopeptidase P) 1, soluble, isoform CRA_b	1.01	0.69
Rraga	Q80X95	Ras-related GTP-binding protein A	1.01	0.68
Dnm1l	Q8K1M6	Dynamin-1-like protein	1.01	0.58
Fcgr2b	A0A0B4J1E6	Fc receptor, IgG, low affinity IIb	1.01	0.80
Tmed5	Q9CXE7	Transmembrane emp24 domain-containing protein 5	1.01	0.80
Ptgr2	Q8VDQ1	Prostaglandin reductase 2	1.01	0.58
Cttn	Q60598	Src substrate cortactin	1.01	0.90
Ghdc	Q99J23	GH3 domain-containing protein	1.01	0.87
Nup210	Q9QY81	Nuclear pore membrane glycoprotein 210	1.01	0.82
Twf1	Q91YR1	Twinfilin-1	1.01	0.87
Pld3	O35405	Phospholipase D3	1.01	0.78
Smarcd2	Q99JR8	SWI/SNF-related matrix-associated actin-dependent regulator of chromatin subfamily D member 2	1.01	0.73
Brd4	Q3UH70	Bromodomain-containing protein 4	1.01	0.69
Itpk1	Q8BYN3	Inositol-tetrakisphosphate 1-kinase	1.01	0.87
Glce	Q9EPS3	D-glucuronyl C5-epimerase	1.01	0.85
Galnt2	Q6PB93	Polypeptide N-acetylgalactosaminyltransferase 2	1.01	0.71
Rexo2	Q9D8S4	Oligoribonuclease, mitochondrial	1.01	0.65
Rars	Q9D0I9	Arginine--tRNA ligase, cytoplasmic	1.01	0.67
Pck2	A0A0R4J0G0	Phosphoenolpyruvate carboxykinase [GTP], mitochondrial	1.01	0.68
Klhdc4	G3X961	Kelch domain containing 4, isoform CRA_a	1.01	0.74
Gsn	P13020	Gelsolin	1.01	0.80
Ankrd49	Q8VE42	Ankyrin repeat domain-containing protein 49	1.01	0.81
Abcf2	Q99LE6	ATP-binding cassette sub-family F member 2	1.01	0.72
Dnttip1	Q99LB0	Deoxynucleotidyltransferase terminal-interacting protein 1	1.01	0.76
Cdk11b	P24788	Cyclin-dependent kinase 11B	1.01	0.81

Gak	A0A0R4J0F6	Cyclin-G-associated kinase	1.01	0.66
Hdac3	Q3UM33	Histone deacetylase	1.01	0.81
Lrrc8c	Q8R502	Volume-regulated anion channel subunit LRRC8C	1.01	0.88
Exosc6	Q8BTW3	Exosome complex component MTR3	1.01	0.80
Setd3	Q91WC0	Histone-lysine N-methyltransferase setd3	1.01	0.74
Cfdp1	O88271	Craniofacial development protein 1	1.01	0.85
Dnajc9	Q91WN1	DnaJ homolog subfamily C member 9	1.01	0.78
Dmxl2	B0V2P5	DmX-like protein 2	1.01	0.66
Ankzf1	J3QM81	Ankyrin repeat and zinc finger domain-containing protein 1	1.01	0.89
Chd3	B1AR17	Chromodomain helicase DNA-binding protein 3	1.01	0.67
Larp4	G3X9Q6	La-related protein 4	1.01	0.75
Usp7	E9PXY8	Ubiquitin carboxyl-terminal hydrolase 7	1.01	0.63
H2-D1	P01899	H-2 class I histocompatibility antigen, D-B alpha chain	1.01	0.77
Uba6	Q8C7R4	Ubiquitin-like modifier-activating enzyme 6	1.01	0.67
Copz2	Q9JHH9	Coatomer subunit zeta-2	1.01	0.82
Hmga1	P17095	High mobility group protein HMG-I/HMG-Y	1.01	0.83
Bcar3	Q9QZK2	Breast cancer anti-estrogen resistance protein 3	1.01	0.90
Ndufv1	Q91YT0	NADH dehydrogenase [ubiquinone] flavoprotein 1, mitochondrial	1.01	0.63
Mat2b	Q99LB6	Methionine adenosyltransferase 2 subunit beta	1.01	0.66
Tbc1d15	Q9CXF4	TBC1 domain family member 15	1.01	0.60
Adprhl2	Q8CG72	Poly(ADP-ribose) glycohydrolase ARH3	1.01	0.69
Dnajb4	Q9D832	DnaJ homolog subfamily B member 4	1.01	0.49
Incenp	Q9WU62	Inner centromere protein	1.01	0.79
Pold3	Q9EQ28	DNA polymerase delta subunit 3	1.01	0.73
Ddx51	Q6P9R1	ATP-dependent RNA helicase DDX51	1.01	0.87
Cerk	Q8K4Q7	Ceramide kinase	1.01	0.71
Sap30bp	Q02614	SAP30-binding protein	1.01	0.77
Commd1	Q8K4M5	COMM domain-containing protein 1	1.01	0.77
Ago1	Q8CJG1	Protein argonaute-1	1.01	0.80
Diaph2	Q6W4W7	DIA3	1.01	0.71
Oplah	Q8K010	5-oxoprolinase	1.01	0.92
Smap1	Q91VZ6	Stromal membrane-associated protein 1	1.01	0.70
Atp5me	Q06185	ATP synthase subunit e, mitochondrial	1.01	0.81
Svil	A0A1B0GS91	Supervillin	1.01	0.69
Slc44a1	A2AMH4	Choline transporter-like protein 1	1.01	0.74
Srek1	Q8BZX4	Splicing regulatory glutamine/lysine-rich protein 1	1.01	0.75
Tradd	Q3U0V2	Tumor necrosis factor receptor type 1-associated DEATH domain protein	1.01	0.68
Mtco2	P00405	Cytochrome c oxidase subunit 2	1.01	0.55
Cars	Q9ER72	Cysteine--tRNA ligase, cytoplasmic	1.01	0.68
Trmu	Q9DAT5	Mitochondrial tRNA-specific 2-thiouridylase 1	1.01	0.76
	Q8C5K5	Uncharacterized protein CXorf38 homolog	1.01	0.65
Eif4a3	Q91VC3	Eukaryotic initiation factor 4A-III	1.01	0.62
Aagab	Q8R2R3	Alpha- and gamma-adaptin-binding protein p34	1.01	0.77
Osbpl9	A2A8Z1	Oxysterol-binding protein-related protein 9	1.01	0.67
Blzf1	Q8R2X8	Golgin-45	1.01	0.94
Slc3a2	P10852	4F2 cell-surface antigen heavy chain	1.01	0.70
Lrpap1	P55302	Alpha-2-macroglobulin receptor-associated protein	1.01	0.70
Melk	Q61846	Maternal embryonic leucine zipper kinase	1.01	0.82
Faim	D3Z3C1	Fas apoptotic inhibitory molecule 1	1.01	0.77
Parp4	E9PYK3	Poly [ADP-ribose] polymerase	1.01	0.73
Pi4ka	E9Q3L2	Phosphatidylinositol 4-kinase alpha	1.01	0.75
Pus10	Q9D3U0	Putative tRNA pseudouridine synthase Pus10	1.01	0.77
Ppm1b	Q99NF7	Ppm1b protein	1.01	0.80
Ciao2b	Q9D187	Cytosolic iron-sulfur assembly component 2B	1.01	0.77
Clec7a	Q6QLQ4	C-type lectin domain family 7 member A	1.01	0.88
Srrm2	Q8BTI8	Serine/arginine repetitive matrix protein 2	1.01	0.71
Atp11c	F6Q8D3	Phospholipid-transporting ATPase	1.01	0.80
Mpeg1	E9QN37	Macrophage-expressed gene 1 protein	1.01	0.65
Trap1	Q9CQN1	Heat shock protein 75 kDa, mitochondrial	1.01	0.75
Ncf2	O70145	Neutrophil cytosol factor 2	1.01	0.64
Rab29	E9QLQ7	Ras-related protein Rab-7L1	1.01	0.77
Wdr43	Q6ZQL4	WD repeat-containing protein 43	1.01	0.70
Pex19	Q8VCI5	Peroxisomal biogenesis factor 19	1.01	0.76
Urod	P70697	Uroporphyrinogen decarboxylase	1.01	0.81
Gnptab	Q69ZN6	N-acetylglucosamine-1-phosphotransferase subunits alpha/beta	1.01	0.67
Usp4	P35123	Ubiquitin carboxyl-terminal hydrolase 4	1.01	0.60
Arhgap17	Q3UIA2	Rho GTPase-activating protein 17	1.01	0.46
Mrpl52	Q9D0Y8	39S ribosomal protein L52, mitochondrial	1.01	0.85
Ezh1	A0A0R4J1C0	Histone-lysine N-methyltransferase EZH1	1.01	0.87
Alad	P10518	Delta-aminolevulinic acid dehydratase	1.01	0.74
Rab33b	O35963	Ras-related protein Rab-33B	1.01	0.93
Ndufs5	Q99LY9	NADH dehydrogenase [ubiquinone] iron-sulfur protein 5	1.01	0.51
Lin9	A0A0A6YVZ7	Protein lin-9 homolog	1.01	0.75

Ddah2	Q99LD8	N(G),N(G)-dimethylarginine dimethylaminohydrolase 2	1.01	0.59
Selenot	P62342	Thioredoxin reductase-like selenoprotein T	1.01	0.90
Hgsnat	Q3UDW8	Heparan-alpha-glucosaminide N-acetyltransferase	1.01	0.81
Snx10	Q9CWT3	Sorting nexin-10	1.01	0.77
Sec23ip	G3X928	SEC23-interacting protein	1.01	0.60
Arap1	Q4LDD4	Arf-GAP with Rho-GAP domain, ANK repeat and PH domain-containing protein 1	1.01	0.69
Themis2	Q91YX0	Protein THEMIS2	1.01	0.75
Dock5	B2RY04	Dedicator of cytokinesis protein 5	1.01	0.91
Micall1	Q8BGT6	MICAL-like protein 1	1.01	0.76
Cep350	E9Q309	Centrosome-associated protein 350	1.01	0.66
Pcmtd1	P59913	Protein-L-isoaspartate O-methyltransferase domain-containing protein 1	1.01	0.82
Gsk3a	Q2NL51	Glycogen synthase kinase-3 alpha	1.01	0.78
Ssrp1	Q08943	FACT complex subunit SSRP1	1.01	0.67
Aars	Q8BGQ7	Alanine--tRNA ligase, cytoplasmic	1.01	0.69
Map3k20	Q9ESL4	Mitogen-activated protein kinase kinase kinase 20	1.01	0.76
Cnot1	Q6ZQ08	CCR4-NOT transcription complex subunit 1	1.01	0.69
Atg14	Q8CDJ3	Beclin 1-associated autophagy-related key regulator	1.01	0.83
Tpm1	G5E8R2	Tropomyosin 1, alpha, isoform CRA_k	1.01	0.30
Rab3gap1	Q80UJ7	Rab3 GTPase-activating protein catalytic subunit	1.01	0.72
Ankhd1	E9PUR0	Ankyrin repeat and KH domain-containing 1	1.01	0.83
Mrps27	Q8BK72	28S ribosomal protein S27, mitochondrial	1.01	0.75
Sipa1l2	Q80TE4	Signal-induced proliferation-associated 1-like protein 2	1.01	0.87
Plekho2	Q8K124	Pleckstrin homology domain-containing family O member 2	1.01	0.59
Arpc5l	Q9D898	Actin-related protein 2/3 complex subunit 5-like protein	1.01	0.76
Med9	Q8VCS6	Mediator of RNA polymerase II transcription subunit 9	1.01	0.72
Wdfy3	G3UYW1	WD repeat and FYVE domain-containing protein 3	1.01	0.68
Smap2	Q7TN29	Stromal membrane-associated protein 2	1.01	0.67
Crtc2	Q3U182	CREB-regulated transcription coactivator 2	1.01	0.93
Rcc1l	Q9CYF5	RCC1-like G exchanging factor-like protein	1.01	0.89
Dock7	A2A9M4	Dedicator of cytokinesis protein 7	1.01	0.74
Vipas39	Q8BGQ1	Spermatogenesis-defective protein 39 homolog	1.01	0.63
Cul2	Q9D4H8	Cullin-2	1.01	0.70
Ddx1	Q91VR5	ATP-dependent RNA helicase DDX1	1.01	0.67
Vps50	Q8CI71	Syndetin	1.01	0.69
Vamp2	B0QZN5	Vesicle-associated membrane protein 2	1.01	0.93
Npepps	Q11011	Puromycin-sensitive aminopeptidase	1.01	0.58
Lgals1	P16045	Galectin-1	1.01	0.43
Smg1	Q8BKX6	Serine/threonine-protein kinase SMG1	1.01	0.82
Sec31a	Q3UPL0	Protein transport protein Sec31A	1.01	0.68
Cpsf3	Q9QXK7	Cleavage and polyadenylation specificity factor subunit 3	1.01	0.76
Gpaa1	Q9WTK3	Glycosylphosphatidylinositol anchor attachment 1 protein	1.01	0.84
Psd4	Q8BLR5	PH and SEC7 domain-containing protein 4	1.01	0.73
Tspan14	Q8QZY6	Tetraspanin-14	1.01	0.76
Trmt1	A0A0R4IZW7	tRNA (guanine(26)-N(2))-dimethyltransferase	1.01	0.83
Srbd1	Q497V5	S1 RNA-binding domain-containing protein 1	1.01	0.80
Galt	Q03249	Galactose-1-phosphate uridylyltransferase	1.01	0.86
Prpf39	E9QJV4	Pre-mRNA-processing factor 39	1.01	0.85
Slamf9	A0A0R4J072	SLAM family member 9	1.01	0.91
Drg1	P32233	Developmentally-regulated GTP-binding protein 1	1.01	0.66
Coro2a	B1AVH5	Coronin	1.01	0.56
Tma7	Q8K003	Translation machinery-associated protein 7	1.01	0.67
Eif4b	Q8BGD9	Eukaryotic translation initiation factor 4B	1.01	0.74
Guf1	Q8C3X4	Translation factor Guf1, mitochondrial	1.01	0.82
Myl6b	Q8CI43	Myosin light chain 6B	1.01	0.82
Scyl3	Q9DBQ7	Protein-associating with the carboxyl-terminal domain of ezrin	1.01	0.66
Uso1	Q9Z1Z0	General vesicular transport factor p115	1.01	0.51
Afdn	E9Q9C3	Afadin	1.01	0.75
Ak2	Q9WTP6	Adenylate kinase 2, mitochondrial	1.01	0.76
Gm2a	Q60648	Ganglioside GM2 activator	1.01	0.68
Tmub2	G8JL73	Transmembrane and ubiquitin-like domain-containing protein 2 (Fragment)	1.01	0.79
Efl1	Q8C0D5	Elongation factor-like GTPase 1	1.01	0.79
Casp1	P29452	Caspase-1	1.01	0.69
Trappc9	Q3U0M1	Trafficking protein particle complex subunit 9	1.01	0.66
Dkc1	Q9ESX5	H/ACA ribonucleoprotein complex subunit DKC1	1.01	0.74
Tmx3	Q8BXZ1	Protein disulfide-isomerase TMX3	1.01	0.51
Acin1	Q9JIX8	Apoptotic chromatin condensation inducer in the nucleus	1.01	0.65
Spsb2	O88838	SPRY domain-containing SOCS box protein 2	1.01	0.81
Ube2f	Q9CY34	NEDD8-conjugating enzyme UBE2F	1.01	0.88
Arhgef12	F8VQN6	Rho guanine nucleotide exchange factor 12	1.01	0.84
Vps28	Q9D1C8	Vacuolar protein sorting-associated protein 28 homolog	1.01	0.60
Cul5	G3X914	Cullin-5	1.01	0.70
Lars	Q8BMJ2	Leucine--tRNA ligase, cytoplasmic	1.01	0.74
Nlrp10	Q8CCN1	NACHT, LRR and PYD domains-containing protein 10	1.01	0.92

Dync1li1	Q8R1Q8	Cytoplasmic dynein 1 light intermediate chain 1	1.01	0.40
Uchl3	Q9JKB1	Ubiquitin carboxyl-terminal hydrolase isozyme L3	1.01	0.67
Pnkd	A0A0R4J1J1	MCG114807, isoform CRA_c	1.01	0.89
C3	P01027	Complement C3	1.01	0.82
Xpo1	Q6P5F9	Exportin-1	1.01	0.46
Rpl31	P62900	60S ribosomal protein L31	1.01	0.67
Usp15	Q8R5H1	Ubiquitin carboxyl-terminal hydrolase 15	1.01	0.58
Aip	O08915	AH receptor-interacting protein	1.01	0.63
Mios	Q8VE19	GATOR complex protein MIOS	1.01	0.59
Ubap2l	Q80X50	Ubiquitin-associated protein 2-like	1.01	0.60
Lss	Q8BLN5	Lanosterol synthase	1.01	0.78
Ube4b	Q9ES00	Ubiquitin conjugation factor E4 B	1.01	0.71
Ppip5k2	E9Q9J4	Inositol hexakisphosphate and diphosphoinositol-pentakisphosphate kinase	1.01	0.71
Polr2d	Q9D7M8	DNA-directed RNA polymerase II subunit RPB4	1.01	0.70
Slu7	Q8BHJ9	Pre-mRNA-splicing factor SLU7	1.01	0.79
Pcyox1l	Q8C7K6	Prenylcysteine oxidase-like	1.01	0.73
Pih1d1	Q9CQJ2	PIH1 domain-containing protein 1	1.01	0.74
Myo18a	E9QAX2	Unconventional myosin-XVIIIa	1.01	0.70
Avl9	Q80U56	Late secretory pathway protein AVL9 homolog	1.01	0.77
Tubgcp2	Q921G8	Gamma-tubulin complex component 2	1.01	0.74
Abcc5	Q9R1X5	Multidrug resistance-associated protein 5	1.01	0.83
Iws1	Q8C1D8	Protein IWS1 homolog	1.01	0.68
Nub1	A0A0G2JGQ4	NEDD8 ultimate buster 1	1.01	0.53
Ube2d1	P61080	Ubiquitin-conjugating enzyme E2 D1	1.01	0.76
Strn	O55106	Striatin	1.01	0.68
Hadh	Q61425	Hydroxyacyl-coenzyme A dehydrogenase, mitochondrial	1.01	0.67
Pygl	Q9ET01	Glycogen phosphorylase, liver form	1.01	0.63
Plekha2	Q9ERS5	Pleckstrin homology domain-containing family A member 2	1.01	0.66
Nmrk1	Q91W63	Nicotinamide riboside kinase 1	1.01	0.83
Kin	Q8K339	DNA/RNA-binding protein KIN17	1.01	0.87
Gaa	P70699	Lysosomal alpha-glucosidase	1.01	0.70
Def8	G3X9U5	Differentially expressed in FDCP 8, isoform CRA_c	1.01	0.81
Skiv2l	Q6NZR5	Superkiller viralicidic activity 2-like (S. cerevisiae)	1.01	0.79
Cox15	Q8BJ03	Cytochrome c oxidase assembly protein COX15 homolog	1.01	0.78
Arhgef6	F6WMJ3	Rho guanine nucleotide exchange factor 6	1.01	0.66
Pou2f1	A0A0R4J1Z4	POU domain protein	1.01	0.77
Ufsp2	Q99K23	Ufm1-specific protease 2	1.01	0.75
Mphosph10	Q810V0	U3 small nucleolar ribonucleoprotein protein MPP10	1.01	0.59
Katnb1	Q8BG40	Katanin p80 WD40 repeat-containing subunit B1	1.01	0.81
Serpinb12	Q9D7P9	Serpin B12	1.01	0.81
Enpp4	Q8BTJ4	Bis(5'-adenosyl)-triphosphatase enpp4	1.01	0.84
Lhpp	Q9D7I5	Phospholysine phosphohistidine inorganic pyrophosphate phosphatase	1.01	0.76
Cdipt	Q8VDP6	CDP-diacylglycerol--inositol 3-phosphatidyltransferase	1.01	0.74
Smchd1	Q6P5D8	Structural maintenance of chromosomes flexible hinge domain-containing protein 1	1.01	0.77
Gipc1	Q9Z0G0	PDZ domain-containing protein GIPC1	1.01	0.77
Mpp7	G5E8S8	MAGUK p55 subfamily member 7	1.01	0.84
Cd14	P10810	Monocyte differentiation antigen CD14	1.01	0.76
Sgf29	Q9DA08	SAGA-associated factor 29	1.01	0.90
Atp6v0a1	Q9Z1G4	V-type proton ATPase 116 kDa subunit a isoform 1	1.01	0.92
Ublcp1	Q8BGR9	Ubiquitin-like domain-containing CTD phosphatase 1	1.01	0.58
Lrmp	G5E880	Lymphoid-restricted membrane protein	1.01	0.70
Hectd1	F8WIE5	E3 ubiquitin-protein ligase HECTD1	1.01	0.73
Cpeb4	Q7TN98	Cytoplasmic polyadenylation element-binding protein 4	1.01	0.53
Ints8	Q80V86	Integrator complex subunit 8	1.01	0.76
Itgax	Q9QXH4	Integrin alpha-X	1.01	0.81
Gstt3	Q99L20	Glutathione S-transferase theta-3	1.01	0.74
Tecpr1	Q80VP0	Tectonin beta-propeller repeat-containing protein 1	1.01	0.60
Mrps7	Q80X85	28S ribosomal protein S7, mitochondrial	1.01	0.72
Ltf	P08071	Lactotransferrin	1.01	0.78
Larp4b	Q6A0A2	La-related protein 4B	1.01	0.58
Hip1	Q8VD75	Huntingtin-interacting protein 1	1.01	0.48
Psmd2	Q8VDM4	26S proteasome non-ATPase regulatory subunit 2	1.01	0.49
Epm2aip1	Q8VEH5	EPM2A-interacting protein 1	1.01	0.84
Mesd	Q9ERE7	LRP chaperone MESD	1.01	0.65
Cdk5rap3	Q99LM2	CDK5 regulatory subunit-associated protein 3	1.01	0.65
Rxrb	Q3TWJ1	Retinoic acid receptor RXR-beta	1.01	0.79
Nrbf2	Q8VCQ3	Nuclear receptor-binding factor 2	1.01	0.78
Tprg1l	Q9DBS2	Tumor protein p63-regulated gene 1-like protein	1.01	0.86
Nup98	A0A1B0GSX7	Nuclear pore complex protein Nup98-Nup96	1.01	0.68
Pex16	Q91XC9	Peroxisomal membrane protein PEX16	1.01	0.62
Mrps28	Q9CY16	28S ribosomal protein S28, mitochondrial	1.01	0.86
Rae1	Q8C570	mRNA export factor	1.01	0.61
Nab1	Q61122	NGFI-A-binding protein 1	1.01	0.70

Abcb6	Q9DC29	ATP-binding cassette sub-family B member 6, mitochondrial	1.01	0.84
Hscb	A0A0R4J0T0	Iron-sulfur cluster co-chaperone protein HscB, mitochondrial	1.01	0.79
Dhrsx	Q8VBZ0	Dehydrogenase/reductase SDR family member on chromosome X homolog	1.01	0.76
Fam32a	Q9CR80	Protein FAM32A	1.01	0.69
Kif1b	Q60575	Kinesin-like protein KIF1B	1.01	0.71
Ncoa3	Q05BA5	Nuclear receptor coactivator	1.01	0.70
Klc1	Q8CD76	Kinesin light chain 1	1.01	0.50
Brox	Q8K2Q7	BRO1 domain-containing protein BROX	1.01	0.67
Ide	F6RPJ9	Insulin-degrading enzyme (Fragment)	1.01	0.85
Rap1gds1	Q3TU36	RAP1, GTP-GDP dissociation stimulator 1	1.01	0.64
Cast	P51125	Calpastatin	1.01	0.73
Fggy	A2AJL3	FGGY carbohydrate kinase domain-containing protein	1.01	0.74
Riox2	Q8CD15	Ribosomal oxygenase 2	1.01	0.63
Arid2	E9Q7E2	AT-rich interactive domain-containing protein 2	1.01	0.69
Ap1g2	O88512	AP-1 complex subunit gamma-like 2	1.01	0.66
H2-K1	P01901	H-2 class I histocompatibility antigen, K-B alpha chain	1.01	0.76
Atg4b	A0A0R4J065	Cysteine protease	1.01	0.75
Gcc2	B2RSU7	GRIP and coiled-coil domain containing 2	1.01	0.69
Strn4	P58404	Striatin-4	1.01	0.75
Pycr3	Q9DCC4	Pyrroline-5-carboxylate reductase 3	1.01	0.77
Psip1	Q99JF8	PC4 and SFRS1-interacting protein	1.01	0.72
Mrpl21	Q9D1N9	39S ribosomal protein L21, mitochondrial	1.01	0.80
Vps41	Q5KU39	Vacuolar protein sorting-associated protein 41 homolog	1.01	0.57
Tars2	Q3UQ84	Threonine--tRNA ligase, mitochondrial	1.01	0.75
Ipo9	Q91YE6	Importin-9	1.01	0.58
Gadd45gip1	Q9CR59	Growth arrest and DNA damage-inducible proteins-interacting protein 1	1.01	0.74
Gm20431	E9PY39	Predicted gene 20431	1.01	0.63
Rap2b	P61226	Ras-related protein Rap-2b	1.01	0.53
Scfd1	Q8BRF7	Sec1 family domain-containing protein 1	1.01	0.67
Hnrnpul1	Q8VDM6	Heterogeneous nuclear ribonucleoprotein U-like protein 1	1.01	0.62
Eif5b	Q05D44	Eukaryotic translation initiation factor 5B	1.01	0.60
Map4k4	F8VPL5	Mitogen-activated protein kinase kinase kinase kinase 4	1.01	0.71
Sp100	Q8C405	Nuclear autoantigen Sp-100	1.01	0.76
Upf1	Q9EPU0	Regulator of nonsense transcripts 1	1.01	0.57
Dpp7	Q9ET22	Dipeptidyl peptidase 2	1.01	0.67
Aldh7a1	Q9DBF1	Alpha-aminoadipic semialdehyde dehydrogenase	1.01	0.60
Bcas3	Q8CCN5	Breast carcinoma-amplified sequence 3 homolog	1.01	0.79
Ndufa2	Q9CQ75	NADH dehydrogenase [ubiquinone] 1 alpha subcomplex subunit 2	1.01	0.73
Ccdc28a	Q8CEI3	Coiled-coil domain-containing 28A	1.01	0.74
Glmn	Q8BZM1	Glomulin	1.01	0.74
Sec24a	A2AA71	Protein transport protein Sec24A	1.01	0.76
Mrpl14	Q9D1I6	39S ribosomal protein L14, mitochondrial	1.01	0.73
Wdr26	Q8C6G8	WD repeat-containing protein 26	1.01	0.56
Acbd3	A0A0R4J079	Acyl-Coenzyme A binding domain containing 3, isoform CRA_b	1.01	0.50
Tfeb	Q3UKG7	Transcription factor EB	1.01	0.59
Atpaf2	A0A0R4J1C5	ATP synthase mitochondrial F1 complex assembly factor 2	1.01	0.89
Adck1	Q9D0L4	Uncharacterized aarF domain-containing protein kinase 1	1.01	0.70
Pus7l	Q8CE46	Pseudouridylate synthase 7 homolog-like protein	1.01	0.62
Gnai2	P08752	Guanine nucleotide-binding protein G(i) subunit alpha-2	1.01	0.58
Stam	P70297	Signal transducing adapter molecule 1	1.01	0.66
Tnpo3	Q6P2B1	Transportin-3	1.01	0.58
Spart	Q8R1X6	Spartin	1.01	0.66
Fkbp1b	Q9Z2I2	Peptidyl-prolyl cis-trans isomerase FKBP1B	1.01	0.82
Tyk2	E9QJS1	Tyrosine-protein kinase	1.01	0.87
Dctn6	Q9WUB4	Dynactin subunit 6	1.01	0.53
Arl8a	Q8VEH3	ADP-ribosylation factor-like protein 8A	1.01	0.62
Lyrm9	E9PX24	LYR motif-containing protein 9 (Fragment)	1.01	0.54
Ncoa5	Q91W39	Nuclear receptor coactivator 5	1.01	0.73
Arfgap2	Q99K28	ADP-ribosylation factor GTPase-activating protein 2	1.01	0.73
Glb1l	Q8VC60	Beta-galactosidase-1-like protein	1.01	0.78
Ddx42	Q810A7	ATP-dependent RNA helicase DDX42	1.01	0.66
Hspbp1	Q99P31	Hsp70-binding protein 1	1.01	0.48
Pnpt1	Q8K1R3	Polyribonucleotide nucleotidyltransferase 1, mitochondrial	1.01	0.69
Pfdn2	O70591	Prefoldin subunit 2	1.01	0.70
Degs1	O09005	Sphingolipid delta(4)-desaturase DES1	1.01	0.88
Etfa	Q99LC5	Electron transfer flavoprotein subunit alpha, mitochondrial	1.01	0.72
Rab1a	Q5SW88	RAB1A, member RAS oncogene family	1.01	0.64
Osbpl11	G5E8A0	Oxysterol-binding protein	1.01	0.54
Ola1	Q9CZ30	Obg-like ATPase 1	1.01	0.64
Pdcl3	Q8BVF2	Phosducin-like protein 3	1.01	0.63
Fkbpl	O35450	FK506-binding protein-like	1.01	0.62
Yif1b	Q9CX30	Protein YIF1B	1.01	0.86
Spi1	P17433	Transcription factor PU.1	1.01	0.71

Plod1	Q9R0E2	Procollagen-lysine,2-oxoglutarate 5-dioxygenase 1	1.01	0.75
Acin1 SV=8	B8JJ89	Apoptotic chromatin condensation inducer in the nucleus (Fragment)	1.01	0.84
Acad11	A0A0R4J0I6	Acyl-CoA dehydrogenase family member 11	1.01	0.79
Ate1	A0A1L1SQ41	Arginyl-tRNA--protein transferase 1	1.01	0.50
Usp45	Q8K387	Ubiquitin carboxyl-terminal hydrolase 45	1.01	0.83
Emilin2	Q8K482	EMILIN-2	1.01	0.85
Gk	B1ASZ3	Glycerol kinase	1.01	0.69
Rreb1	Q3UH06	Ras-responsive element-binding protein 1	1.01	0.85
Tsc22d2	E9Q7M2	TSC22 domain family, member 2	1.01	0.65
Galnt1	O08912	Polypeptide N-acetylgalactosaminyltransferase 1	1.01	0.55
Fam136a	Q9CR98	Protein FAM136A	1.01	0.65
Naa35	Q6PHQ8	N-alpha-acetyltransferase 35, NatC auxiliary subunit	1.01	0.70
Zfyve16	Q80U44	Zinc finger FYVE domain-containing protein 16	1.01	0.76
Nkiras2	Q9CR56	NF-kappa-B inhibitor-interacting Ras-like protein 2	1.01	0.74
Cab39	Q06138	Calcium-binding protein 39	1.01	0.46
Sptan1	P16546	Spectrin alpha chain, non-erythrocytic 1	1.01	0.54
Appl2	Q8K3G9	DCC-interacting protein 13-beta	1.01	0.61
Tmem86a	Q9D8N3	Lysoplasmalogenase-like protein TMEM86A	1.01	0.79
Hnrnpdl	D3YTQ3	Heterogeneous nuclear ribonucleoprotein D-like	1.01	0.64
Ccar2	Q8VDP4	Cell cycle and apoptosis regulator protein 2	1.01	0.75
Ophn1	Q99J31	Oligophrenin-1	1.01	0.61
Klhl25	Q8R2P1	Kelch-like protein 25	1.01	0.83
Rnf113a2	Q14B01	Ring finger protein 113A2	1.01	0.64
Swap70	Q6A028	Switch-associated protein 70	1.01	0.64
Bin3	Q9JI08	Bridging integrator 3	1.01	0.84
Camk2d	A0A0G2JGS4	Calcium/calmodulin-dependent protein kinase type II subunit delta	1.01	0.60
Aspscr1	Q8VBT9	Tether containing UBX domain for GLUT4	1.01	0.72
Smndc1	Q8BGT7	Survival of motor neuron-related-splicing factor 30	1.01	0.63
Arhgap17	E9QAJ9	Rho GTPase-activating protein 17	1.01	0.89
Gorasp1	Q91X51	Golgi reassembly-stacking protein 1	1.01	0.70
Ltv1	Q6NSQ7	Protein LTV1 homolog	1.01	0.74
Idh3g	P70404	Isocitrate dehydrogenase [NAD] subunit gamma 1, mitochondrial	1.01	0.64
Ostc	Q78XF5	Oligosaccharyltransferase complex subunit OSTC	1.01	0.71
Ctnnbl1	Q9CWL8	Beta-catenin-like protein 1	1.01	0.63
Utp4	Q8R2N2	U3 small nucleolar RNA-associated protein 4 homolog	1.01	0.77
Clock	O08785	Circadian locomoter output cycles protein kaput	1.01	0.89
Ddx56	Q9D0R4	Probable ATP-dependent RNA helicase DDX56	1.01	0.74
Pold2	O35654	DNA polymerase delta subunit 2	1.01	0.84
Sugt1	Q9CX34	Protein SGT1 homolog	1.01	0.50
Znf830	Q8R1N0	Zinc finger protein 830	1.01	0.87
Drap1	D3YY09	Dr1-associated corepressor	1.01	0.49
Acox3	Q9EPL9	Peroxisomal acyl-coenzyme A oxidase 3	1.01	0.69
Psmb5	O55234	Proteasome subunit beta type-5	1.01	0.88
Tjp2	Q9Z0U1	Tight junction protein ZO-2	1.01	0.80
Acadm	P45952	Medium-chain specific acyl-CoA dehydrogenase, mitochondrial	1.01	0.48
Tfip11	Q9ERA6	Tuftelin-interacting protein 11	1.01	0.80
Hspb1	P14602	Heat shock protein beta-1	1.01	0.82
Frmd4b	Q920B0	FERM domain-containing protein 4B	1.01	0.76
Tmed9	Q99KF1	Transmembrane emp24 domain-containing protein 9	1.01	0.65
Mtor	Q9JLN9	Serine/threonine-protein kinase mTOR	1.01	0.71
Fech	Q544X6	Ferrochelatase	1.01	0.53
Rbm17	Q8JZX4	Splicing factor 45	1.01	0.71
Sbds	P70122	Ribosome maturation protein SBDS	1.01	0.48
Mfsd5	Q921Y4	Molybdate-anion transporter	1.01	0.66
Toe1	Q9D2E2	Target of EGR1 protein 1	1.01	0.75
Ppox	P51175	Protoporphyrinogen oxidase	1.01	0.58
Bfar	A0A0R4J040	Bifunctional apoptosis regulator	1.01	0.73
H2-T23	P06339	H-2 class I histocompatibility antigen, D-37 alpha chain	1.01	0.80
Mlkl	Q9D2Y4	Mixed lineage kinase domain-like protein	1.01	0.64
Col18a1	E9QPX1	Collagen alpha-1(XVIII) chain	1.01	0.84
Plcl2	Q8K394	Inactive phospholipase C-like protein 2	1.01	0.77
Enoph1	Q8BGB7	Enolase-phosphatase E1	1.01	0.77
Atp6v1c1	Q9Z1G3	V-type proton ATPase subunit C 1	1.01	0.56
Btf3l4	Q9CQH7	Transcription factor BTF3 homolog 4	1.01	0.72
Wdr6	Q99ME2	WD repeat-containing protein 6	1.01	0.78
Farsa	Q8C0C7	Phenylalanine--tRNA ligase alpha subunit	1.01	0.71
Rel	A4QPD3	Proto-oncogene c-Rel	1.01	0.67
Tmf1	B9EKI3	TATA element modulatory factor	1.01	0.64
Ccdc167	Q9D162	Coiled-coil domain-containing protein 167	1.01	0.89
Vim	P20152	Vimentin	1.01	0.68
Rlim	Q9WTV7	E3 ubiquitin-protein ligase RLIM	1.01	0.89
Ptgs1	P22437	Prostaglandin G/H synthase 1	1.01	0.51
Vps36	Q91XD6	Vacuolar protein-sorting-associated protein 36	1.01	0.66

Chmp3	Q9CQ10	Charged multivesicular body protein 3	1.01	0.56
Ripk2	P58801	Receptor-interacting serine/threonine-protein kinase 2	1.01	0.75
Ticam2	Q8BJQ4	TIR domain-containing adapter molecule 2	1.01	0.92
Serpinb8	O08800	Serpin B8	1.01	0.52
Shkbp1	Q6P7W2	SH3KBP1-binding protein 1	1.01	0.78
0610009B22Rik	Q8R3W2	MCG6979, isoform CRA_a	1.01	0.37
Eefsec	Q9JHW4	Selenocysteine-specific elongation factor	1.01	0.78
Bag6	Q9Z1R2	Large proline-rich protein BAG6	1.01	0.71
Pcca	Q91ZA3	Propionyl-CoA carboxylase alpha chain, mitochondrial	1.01	0.65
Dock10	E9QM99	Dedicator of cytokinesis protein 10	1.01	0.62
Naa50	Q6PGB6	N-alpha-acetyltransferase 50	1.01	0.49
Slc27a1	Q60714	Long-chain fatty acid transport protein 1	1.01	0.63
Eif3g	Q9Z1D1	Eukaryotic translation initiation factor 3 subunit G	1.01	0.49
Pla2g7	Q60963	Platelet-activating factor acetylhydrolase	1.01	0.75
Ptpmt1	Q66GT5	Phosphatidylglycerophosphatase and protein-tyrosine phosphatase 1	1.01	0.80
Med10	Q9CXU0	Mediator of RNA polymerase II transcription subunit 10	1.01	0.78
Sco1	Q5SUD5	Protein SCO1 homolog, mitochondrial	1.01	0.67
Kifap3	P70188	Kinesin-associated protein 3	1.01	0.58
Srsf3	P84104	Serine/arginine-rich splicing factor 3	1.01	0.55
Mcm5	Q52KC3	DNA helicase	1.01	0.69
Pxk	Q8BX57	PX domain-containing protein kinase-like protein	1.01	0.82
Hibch	Q8QZS1	3-hydroxyisobutyryl-CoA hydrolase, mitochondrial	1.01	0.61
Ticrr	Q8BQ33	Treslin	1.01	0.91
Ruvbl1	P60122	RuvB-like 1	1.01	0.58
Ptpn9	O35239	Tyrosine-protein phosphatase non-receptor type 9	1.01	0.53
Cdc16	Q8R349	Cell division cycle protein 16 homolog	1.01	0.60
Tbrg4	Q91YM4	FAST kinase domain-containing protein 4	1.01	0.64
Ncf1	Q09014	Neutrophil cytosol factor 1	1.01	0.58
Sh3kbp1	Q8R550	SH3 domain-containing kinase-binding protein 1	1.01	0.42
Asns	Q61024	Asparagine synthetase [glutamine-hydrolyzing]	1.01	0.73
Dynll1	P63168	Dynein light chain 1, cytoplasmic	1.01	0.63
Armc10	Q9D0L7	Armadillo repeat-containing protein 10	1.01	0.63
Nkapd1	E9PUQ3	NKAP domain-containing 1	1.01	0.85
Pdxdc1	A0A0R4J034	MCG129810, isoform CRA_c	1.01	0.60
Cyp4f13	Q99N19	Cytochrome P450 CYP4F13	1.01	0.77
Psph	Q99LS3	Phosphoserine phosphatase	1.01	0.58
Rint1	Q8BZ36	RAD50-interacting protein 1	1.01	0.84
Manba	A0A0R4J092	Beta-mannosidase	1.01	0.40
Gnb4	P29387	Guanine nucleotide-binding protein subunit beta-4	1.01	0.78
Pacs1	Q8K212	Phosphofurin acidic cluster sorting protein 1	1.01	0.67
Col4a3bp	Q9EQG9	Collagen type IV alpha-3-binding protein	1.01	0.40
Stk11ip	Q3TAA7	Serine/threonine-protein kinase 11-interacting protein	1.01	0.77
Edc3	Q8K2D3	Enhancer of mRNA-decapping protein 3	1.01	0.78
Rbm42	Q91V81	RNA-binding protein 42	1.01	0.80
Nras	P08556	GTPase NRas	1.01	0.45
Apmap	Q9D7N9	Adipocyte plasma membrane-associated protein	1.01	0.49
Ube3c	Q80U95	Ubiquitin-protein ligase E3C	1.01	0.72
Arhgap22	Q8BL80	Rho GTPase-activating protein 22	1.01	0.61
Phf14	G5E8S0	PHD finger protein 14	1.01	0.80
Elac2	Q80Y81	Zinc phosphodiesterase ELAC protein 2	1.01	0.72
Supv3l1	Q80YD1	ATP-dependent RNA helicase SUPV3L1, mitochondrial	1.01	0.55
Havcr2	Q8VIM0	Hepatitis A virus cellular receptor 2 homolog	1.01	0.81
Gmeb2	P58929	Glucocorticoid modulatory element-binding protein 2	1.01	0.72
Gss	P51855	Glutathione synthetase	1.01	0.65
Pgam5	Q8BX10	Serine/threonine-protein phosphatase PGAM5, mitochondrial	1.01	0.62
Fcho2	Q3UQN2	F-BAR domain only protein 2	1.01	0.68
Gm20425	E9Q035	Predicted gene 20425	1.01	0.79
Denr	Q9CQJ6	Density-regulated protein	1.01	0.41
Bzw1	A0A087WQS2	Basic leucine zipper and W2 domain-containing protein 1	1.01	0.53
Nbeal2	E9Q9L6	Neurobeachin-like protein 2	1.01	0.76
Nmes1	Q810Q5	Normal mucosa of esophagus-specific gene 1 protein	1.01	0.79
Ints12	Q9D168	Integrator complex subunit 12	1.01	0.68
Mfn2	Q80U63	Mitofusin-2	1.01	0.78
Cox7a1	A0A140LIU4	Cytochrome c oxidase subunit 7A1, mitochondrial	1.01	0.84
Uros	P51163	Uroporphyrinogen-III synthase	1.01	0.82
Neu1	O35657	Sialidase-1	1.01	0.76
Sord	Q64442	Sorbitol dehydrogenase	1.01	0.51
Zeb2	A0A0M3HEP2	Zinc finger E-box-binding homeobox 2	1.01	0.52
Rufy2	Q8R4C2	RUN and FYVE domain-containing protein 2	1.01	0.54
Brat1	E9QLK3	BRCA1-associated ATM activator 1	1.01	0.79
Tial1	P70318	Nucleolysin TIAR	1.01	0.71
Gid8	Q9D7M1	Glucose-induced degradation protein 8 homolog	1.01	0.60
Arfgef2	A2A5R2	Brefeldin A-inhibited guanine nucleotide-exchange protein 2	1.01	0.61

Ube3a	O08759	Ubiquitin-protein ligase E3A	1.01	0.61
Pin1	Q9QUR7	Peptidyl-prolyl cis-trans isomerase NIMA-interacting 1	1.01	0.57
Orc3	Q9JK30	Origin recognition complex subunit 3	1.01	0.93
Tmed10	Q9D1D4	Transmembrane emp24 domain-containing protein 10	1.01	0.76
Nup93	Q8BJ71	Nuclear pore complex protein Nup93	1.01	0.54
Ctr9	Q62018	RNA polymerase-associated protein CTR9 homolog	1.01	0.83
Glrx3	Q9CQM9	Glutaredoxin-3	1.01	0.40
Ube2v2	Q9D2M8	Ubiquitin-conjugating enzyme E2 variant 2	1.01	0.65
Eps15	P42567	Epidermal growth factor receptor substrate 15	1.01	0.60
Ipo7	Q9EPL8	Importin-7	1.01	0.35
Bola2	Q8BGS2	BolA-like protein 2	1.01	0.71
Sms	P97355	Spermine synthase	1.01	0.64
Xpo6	Q924Z6	Exportin-6	1.01	0.86
Ankrd50	F7BE84	Ankyrin repeat domain 50 (Fragment)	1.01	0.61
Gbp2	Q9Z0E6	Guanylate-binding protein 2	1.01	0.93
Cdk4	P30285	Cyclin-dependent kinase 4	1.01	0.70
Gpd2	Q64521	Glycerol-3-phosphate dehydrogenase, mitochondrial	1.01	0.66
Os9	Q8K2C7	Protein OS-9	1.01	0.61
Ufl1	Q8CCJ3	E3 UFM1-protein ligase 1	1.01	0.58
Hsbp1	Q9CQZ1	Heat shock factor-binding protein 1	1.01	0.27
Ap3d1	O54774	AP-3 complex subunit delta-1	1.01	0.51
Ppp6r3	G5E8R4	SAPS domain family, member 3, isoform CRA_c	1.01	0.67
Cpsf6	H3BJW3	Cleavage and polyadenylation-specificity factor subunit 6	1.01	0.50
Tmem168	Q91VX9	Transmembrane protein 168	1.01	0.73
Tnks	Q6PFX9	Tankyrase-1	1.01	0.54
Ptk2	P34152	Focal adhesion kinase 1	1.01	0.81
Timm44	O35857	Mitochondrial import inner membrane translocase subunit TIM44	1.01	0.53
Idh2	P54071	Isocitrate dehydrogenase [NADP], mitochondrial	1.01	0.55
Gskip	Q8BGR8	GSK3B-interacting protein	1.01	0.82
Nup35	Q8R4R6	Nucleoporin NUP35	1.01	0.70
Il10rb	Q8VHM7	Interleukin 10 receptor 2	1.01	0.86
Atl2	Q6PA06	Atlastin-2	1.01	0.75
Atg16l1	Q8C0J2	Autophagy-related protein 16-1	1.01	0.56
Wipf1	Q8K1I7	WAS/WASL-interacting protein family member 1	1.01	0.71
Wdr5	P61965	WD repeat-containing protein 5	1.01	0.61
Ifi47	Q61635	GTP-binding protein	1.01	0.84
Snap29	Q9ERB0	Synaptosomal-associated protein 29	1.01	0.56
Dcaf5	Q80T85	DDB1- and CUL4-associated factor 5	1.01	0.89
Mtmr6	L8AZD2	Myotubularin-related protein 6	1.01	0.44
Med14	A2ABV5	Mediator of RNA polymerase II transcription subunit 14	1.01	0.67
Prdx1	P35700	Peroxiredoxin-1	1.01	0.73
Fibp	Q8K2D8	Acidic fibroblast growth factor intracellular-binding protein	1.01	0.81
Nmi	O35309	N-myc-interactor	1.01	0.56
Hspa1a	Q61696	Heat shock 70 kDa protein 1A	1.01	0.66
Hmgn5	Q9JL35	High mobility group nucleosome-binding domain-containing protein 5	1.01	0.77
Baiap2	B1AZ46	Brain-specific angiogenesis inhibitor 1-associated protein 2	1.01	0.42
Dnajc13	G3X922	DnaJ heat shock protein family (Hsp40) member C13	1.01	0.54
Sssca1	P56873	Sjoegren syndrome/scleroderma autoantigen 1 homolog	1.01	0.61
Pcmt1	E0CYV0	Protein-L-isoaspartate O-methyltransferase	1.01	0.46
Trappc3	O55013	Trafficking protein particle complex subunit 3	1.01	0.38
Vdac1	Q60932	Voltage-dependent anion-selective channel protein 1	1.01	0.66
Nsmaf	O35242	Protein FAN	1.01	0.83
Eif2a	Q8BJW6	Eukaryotic translation initiation factor 2A	1.01	0.57
Bloc1s1	O55102	Biogenesis of lysosome-related organelles complex 1 subunit 1	1.01	0.76
Dnajb11	Q99KV1	DnaJ homolog subfamily B member 11	1.01	0.49
Epn2	Q8CHU3	Epsin-2	1.01	0.86
Diaph1	E9PV41	Protein diaphanous homolog 1	1.01	0.43
Mlst8	Q9DCJ1	Target of rapamycin complex subunit LST8	1.01	0.75
Spag9	Q58A65	C-Jun-amino-terminal kinase-interacting protein 4	1.01	0.49
Rnf40	Q3U319	E3 ubiquitin-protein ligase BRE1B	1.01	0.78
Lyrm4	Q8K215	LYR motif-containing protein 4	1.01	0.93
Trim33	Q99PP7	E3 ubiquitin-protein ligase TRIM33	1.01	0.66
Phaf1	Q922R1	Phagosome assembly factor 1	1.01	0.80
Nup85	Q8R480	Nuclear pore complex protein Nup85	1.01	0.71
Fgd4	Q91ZT5	FYVE, RhoGEF and PH domain-containing protein 4	1.01	0.53
Clcn5	B1ATV0	Chloride channel protein	1.01	0.57
Asap1	E9QN63	Arf-GAP with SH3 domain, ANK repeat and PH domain-containing protein 1	1.01	0.60
Prpf4b	Q61136	Serine/threonine-protein kinase PRP4 homolog	1.01	0.63
Nif3l1	Q9EQ80	NIF3-like protein 1	1.01	0.64
Pola2	P33611	DNA polymerase alpha subunit B	1.01	0.67
Ogdh	Q60597	2-oxoglutarate dehydrogenase, mitochondrial	1.01	0.53
Erc1	F8VPM7	ELKS/Rab6-interacting/CAST family member 1	1.01	0.72
Me1	P06801	NADP-dependent malic enzyme	1.01	0.76

Gstm2	P15626	Glutathione S-transferase Mu 2	1.01	0.67
Uvrag	Q8K245	UV radiation resistance associated protein	1.01	0.61
Etv6	P97360	Transcription factor ETV6	1.01	0.63
Gpkow	Q56A08	G-patch domain and KOW motifs-containing protein	1.01	0.80
Ap5s1	A2ANC6	AP-5 complex subunit sigma-1	1.01	0.74
Ltn1	Q6A009	E3 ubiquitin-protein ligase listerin	1.01	0.47
Zbed5	B2RPU8	MCG130675	1.01	0.64
Rab2a	P53994	Ras-related protein Rab-2A	1.01	0.63
Uqcrb	Q9CQB4	Cytochrome b-c1 complex subunit 7	1.01	0.66
Rcc1	Q6PFB2	Rcc1 protein	1.01	0.58
Hus1	Q8BQY8	Checkpoint protein HUS1	1.01	0.81
Slc48a1	Q9D8M3	Heme transporter HRG1	1.01	0.78
Ddx23	D3Z0M9	DEAD (Asp-Glu-Ala-Asp) box polypeptide 23	1.01	0.52
Phf23	Q8BSN5	PHD finger protein 23	1.01	0.75
Shoc2	O88520	Leucine-rich repeat protein SHOC-2	1.01	0.65
Stab1	G3X973	Stabilin 1, isoform CRA_a	1.01	0.75
Ap3s1	Q9DCR2	AP-3 complex subunit sigma-1	1.01	0.67
Pik3cd	Q3T9Y0	Phosphatidylinositol 4,5-bisphosphate 3-kinase catalytic subunit	1.01	0.75
Rb1	P13405	Retinoblastoma-associated protein	1.01	0.75
Ostf1	Q62422	Osteoclast-stimulating factor 1	1.01	0.67
Necap2	Q9D1J1	Adaptin ear-binding coat-associated protein 2	1.01	0.70
Ptpn23	Q6PB44	Tyrosine-protein phosphatase non-receptor type 23	1.01	0.58
Cactin	Q9CS00	Cactin	1.01	0.72
Anapc2	Q8BZQ7	Anaphase-promoting complex subunit 2	1.01	0.61
Sart3	Q9JLI8	Squamous cell carcinoma antigen recognized by T-cells 3	1.01	0.58
Cep192	E9Q4Y4	Centrosomal protein 192	1.01	0.85
Med25	A0A140LHG7	Mediator of RNA polymerase II transcription subunit 25	1.01	0.78
Lonp1	Q8CGK3	Lon protease homolog, mitochondrial	1.01	0.51
Mrps30	Q9D0G0	28S ribosomal protein S30, mitochondrial	1.01	0.66
Dtnbp1	Q91WZ8	Dysbindin	1.01	0.60
Ercc6l	Q8BHK9	DNA excision repair protein ERCC-6-like	1.01	0.66
Il1rap	Q61730	Interleukin-1 receptor accessory protein	1.01	0.78
Clint1	Q5SUH7	Clathrin interactor 1	1.01	0.71
Ptdss2	Q9Z1X2	Phosphatidylserine synthase 2	1.01	0.43
Irf9	E9PZJ2	Interferon regulatory factor 9	1.01	0.83
Nfrkb	Q6PIJ4	Nuclear factor related to kappa-B-binding protein	1.01	0.79
Rida	P52760	2-iminobutanoate/2-iminopropanoate deaminase	1.01	0.61
Cul3	Q9JLV5	Cullin-3	1.01	0.49
Kdm3b	B9EKS2	Jumonji domain containing 1B	1.01	0.70
Znf385a	Q8VD12	Zinc finger protein 385A	1.01	0.66
Polr1a	O35134	DNA-directed RNA polymerase I subunit RPA1	1.01	0.77
Ubtf	P25976	Nucleolar transcription factor 1	1.01	0.59
Gspt2	Q149F3	Eukaryotic peptide chain release factor GTP-binding subunit ERF3B	1.01	0.79
Ppie	Q9QZH3	Peptidyl-prolyl cis-trans isomerase E	1.01	0.64
Mrps9	Q9D7N3	28S ribosomal protein S9, mitochondrial	1.01	0.52
Cstf2t	Q8C7E9	Cleavage stimulation factor subunit 2 tau variant	1.01	0.62
Gigyf2	Q6Y7W8	GRB10-interacting GYF protein 2	1.01	0.55
Cul1	Q9WTX6	Cullin-1	1.01	0.54
H2afz	P0C0S6	Histone H2A.Z	1.01	0.60
Med13	Q5SWW4	Mediator of RNA polymerase II transcription subunit 13	1.01	0.78
Ppib	P24369	Peptidyl-prolyl cis-trans isomerase B	1.01	0.43
Arhgap12	A0A0A0MQ95	Rho GTPase-activating protein 12	1.01	0.64
Hk2	O08528	Hexokinase-2	1.01	0.57
Map2k4	P47809	Dual specificity mitogen-activated protein kinase kinase 4	1.01	0.53
Inf2	E9QLA5	Inverted formin-2	1.01	0.67
Prcc	Q9EQC8	Papillary Renal Cell carcinoma (Translocation-associated)	1.01	0.75
Rsbn1	Q80T69	Lysine-specific demethylase 9	1.01	0.85
Zw10	O54692	Centromere/kinetochore protein zw10 homolog	1.01	0.73
Ankle2	Q6P1H6	Ankyrin repeat and LEM domain-containing protein 2	1.01	0.63
Rab3a	P63011	Ras-related protein Rab-3A	1.01	0.77
Myadm	O35682	Myeloid-associated differentiation marker	1.01	0.75
Fryl	F8VQ05	FRY-like transcription coactivator	1.01	0.62
Smg9	Q9DB90	Protein SMG9	1.01	0.63
Pnpla6	Q3TRM4	Neuropathy target esterase	1.01	0.85
Ecsit	A0A0R4J174	Evolutionarily conserved-signaling intermediate in Toll pathway, mitochondrial	1.01	0.67
Pex5	O09012	Peroxisomal targeting signal 1 receptor	1.01	0.63
Maf1	Q9D0U6	Repressor of RNA polymerase III transcription MAF1 homolog	1.01	0.82
Kif5b	Q61768	Kinesin-1 heavy chain	1.01	0.47
Hpf1	Q8CFE2	Histone PARylation factor 1	1.01	0.60
Ampd3	A0A1L1SRX2	AMP deaminase	1.01	0.65
Get4	Q9D1H7	Golgi to ER traffic protein 4 homolog	1.01	0.69
Irf3	P70671	Interferon regulatory factor 3	1.01	0.69
Irf2	P23906	Interferon regulatory factor 2	1.01	0.66

Tbc1d5	A0A286YDB3	TBC1 domain family member 5	1.01	0.72
Naaa	Q9D7V9	N-acylethanolamine-hydrolyzing acid amidase	1.01	0.79
Aak1	Q3UHJ0	AP2-associated protein kinase 1	1.01	0.54
Ccar1	Q8CH18	Cell division cycle and apoptosis regulator protein 1	1.01	0.64
Galc	P54818	Galactocerebrosidase	1.01	0.58
Ncapd2	A0A0R4J0H7	Condensin complex subunit 1	1.01	0.74
Dcp1a	Q91YD3	mRNA-decapping enzyme 1A	1.01	0.72
Cttnbp2nl	Q99LJ0	CTTNBP2 N-terminal-like protein	1.01	0.57
Ccnk	Q3U3M5	Cyclin-K	1.01	0.53
Clip2	Q9Z0H8	CAP-Gly domain-containing linker protein 2	1.01	0.50
Eipr1	Q8K0G5	EARP and GARP complex-interacting protein 1	1.01	0.37
Gpt2	Q8BGT5	Alanine aminotransferase 2	1.01	0.63
Rilpl2	Q99LE1	RILP-like protein 2	1.01	0.47
Rela	Q04207	Transcription factor p65	1.01	0.49
Mrpl37	Q921S7	39S ribosomal protein L37, mitochondrial	1.01	0.67
Mrpl16	Q99N93	39S ribosomal protein L16, mitochondrial	1.01	0.72
Tmed7	D3YZZ5	Transmembrane p24-trafficking protein 7	1.01	0.67
Plekha3	Q9ERS4	Pleckstrin homology domain-containing family A member 3	1.01	0.77
Eif1ad	Q3THJ3	Probable RNA-binding protein EIF1AD	1.01	0.57
Vav3	Q9R0C8	Guanine nucleotide exchange factor VAV3	1.01	0.56
Hsd17b10	Q99N15	17beta-hydroxysteroid dehydrogenase type 10/short chain L-3-hydroxyacyl-CoA dehydrogenase	1.01	0.64
Ddx60	E9PZQ1	DEAD (Asp-Glu-Ala-Asp) box polypeptide 60	1.01	0.82
Sf3a1	Q8K4Z5	Splicing factor 3A subunit 1	1.01	0.55
Pkn2	Q8BWW9	Serine/threonine-protein kinase N2	1.01	0.83
Cnot10	Q8BH15	CCR4-NOT transcription complex subunit 10	1.01	0.75
Ears2	Q9CXJ1	Probable glutamate--tRNA ligase, mitochondrial	1.01	0.77
Chd1	P40201	Chromodomain-helicase-DNA-binding protein 1	1.01	0.67
Aimp1	Q3UZG4	Aminoacyl tRNA synthase complex-interacting multifunctional protein 1	1.01	0.54
Rabgef1	D3Z2L2	Rab5 GDP/GTP exchange factor (Fragment)	1.01	0.59
RtcA	Q9D7H3	RNA 3'-terminal phosphate cyclase	1.01	0.78
Aftph	Q80WT5	Aftiphilin	1.01	0.61
Ppp1r9b	Q6R891	Neurabin-2	1.01	0.48
Ifi207	E9Q3L4	Interferon-activated gene 207	1.01	0.60
Leo1	Q5XJE5	RNA polymerase-associated protein LEO1	1.01	0.70
Serpinb9b	Q9DAV6	R86	1.01	0.81
Actc1	P68033	Actin, alpha cardiac muscle 1	1.01	0.65
Acss1	Q99NB1	Acetyl-coenzyme A synthetase 2-like, mitochondrial	1.01	0.72
Ppp1r21	Q3TDD9	Protein phosphatase 1 regulatory subunit 21	1.01	0.61
Metap2	Q3UI33	Methionine aminopeptidase 2	1.01	0.53
Impa2	Q91UZ5	Inositol monophosphatase 2	1.01	0.64
Nup155	Q99P88	Nuclear pore complex protein Nup155	1.01	0.34
Slc35e4	A0A0R4J0N3	Solute carrier family 35 member E4	1.01	0.86
Gm49342	A0A2I3BQH2	Predicted gene, 49342 (Fragment)	1.01	0.76
Cc2d1b	Q8BRN9	Coiled-coil and C2 domain-containing protein 1B	1.01	0.59
Tmem120b	Q3TA38	Transmembrane protein 120B	1.01	0.82
Irgm2	A0A140LIF8	Immunity-related GTPase family M member 2	1.01	0.79
Mrpl32	Q9DCI9	39S ribosomal protein L32, mitochondrial	1.01	0.80
Tcerg1	Q8CGF7	Transcription elongation regulator 1	1.01	0.48
Srsf2	Q62093	Serine/arginine-rich splicing factor 2	1.01	0.72
Agl	F8VPN4	Amylo-1,6-glucosidase, 4-alpha-glucanotransferase	1.01	0.64
Snrnp200	Q6P4T2	U5 small nuclear ribonucleoprotein 200 kDa helicase	1.01	0.47
Ap2a1	P17426	AP-2 complex subunit alpha-1	1.01	0.31
Edc4	G5E896	Enhancer of mRNA decapping 4, isoform CRA_b	1.01	0.64
Ncoa1	P70365	Nuclear receptor coactivator 1	1.01	0.78
Nipsnap2	Q7TMG8	Glioblastoma amplified sequence	1.01	0.70
Nck1	Q99M51	Cytoplasmic protein NCK1	1.01	0.62
Nat10	Q8K224	RNA cytidine acetyltransferase	1.01	0.68
Dctn4	Q8CBY8	Dynactin subunit 4	1.01	0.50
Dcaf11	Q91VU6	DDB1- and CUL4-associated factor 11	1.01	0.59
Tnks1bp1	P58871	182 kDa tankyrase-1-binding protein	1.01	0.65
Ppa1	Q9D819	Inorganic pyrophosphatase	1.01	0.33
Osbpl1a	Q91XL9	Oxysterol-binding protein-related protein 1	1.01	0.62
Ptp4a2	O70274	Protein tyrosine phosphatase type IVA 2	1.01	0.54
Rala	P63321	Ras-related protein Ral-A	1.01	0.72
Nxf1	Q99JX7	Nuclear RNA export factor 1	1.01	0.47
Ahnak	G5E8K8	AHNAK nucleoprotein (desmoyokin)	1.01	0.79
Ercc3	P49135	General transcription and DNA repair factor IIH helicase subunit XPB	1.01	0.56
Sh3kbp1	B0R0Y8	SH3 domain-containing kinase-binding protein 1 (Fragment)	1.01	0.50
Sh3bp1	P55194	SH3 domain-binding protein 1	1.01	0.66
Xylb	Q3TNA1	Xylulose kinase	1.01	0.55
Uxs1	Q91XL3	UDP-glucuronic acid decarboxylase 1	1.01	0.64
Zc3h7a	E9PWW6	Zinc finger CCCH type-containing 7 A	1.01	0.69
Ctc1	Q5SUQ9	CST complex subunit CTC1	1.01	0.73

Fhl3	Q9R059	Four and a half LIM domains protein 3	1.01	0.40
	Q8R092	Uncharacterized protein C1orf43 homolog	1.01	0.68
Tbc1d20	Q9D9I4	TBC1 domain family member 20	1.01	0.81
Map4k2	Q61161	Mitogen-activated protein kinase kinase kinase kinase 2	1.01	0.62
Ttc7a	Q8BGB2	Tetratricopeptide repeat protein 7A	1.01	0.78
Cfl2	P45591	Cofilin-2	1.01	0.58
Mfap1b	C0HKD9	Microfibrillar-associated protein 1B	1.01	0.62
Cdkn2aip	Q8BI72	CDKN2A-interacting protein	1.01	0.52
Arfgef1	G3X9K3	Brefeldin A-inhibited guanine nucleotide-exchange protein 1	1.01	0.50
Sbf2	E9PXF8	SET-binding factor 2	1.01	0.84
Cks1brt	Q3UNC9	Cyclin-dependent kinases regulatory subunit	1.01	0.73
Pank4	Q80YV4	Pantothenate kinase 4	1.01	0.60
Serhl	Q3U3G8	Serine hydrolase-like protein	1.01	0.74
Lyst	P97412	Lysosomal-trafficking regulator	1.01	0.76
Gtf3c1	Q8K284	General transcription factor 3C polypeptide 1	1.01	0.68
Gng11	P61953	Guanine nucleotide-binding protein G(I)/G(S)/G(O) subunit gamma-11	1.01	0.77
Pten	O08586	Phosphatidylinositol 3,4,5-trisphosphate 3-phosphatase and dual-specificity protein phosphatase PTEN	1.01	0.66
Ndst1	Q3UHN9	Bifunctional heparan sulfate N-deacetylase/N-sulfotransferase 1	1.01	0.78
Tut4	A2A8R7	Terminal uridylyltransferase 4	1.01	0.71
Wwp2	Q9DBH0	NEDD4-like E3 ubiquitin-protein ligase WWP2	1.01	0.54
Nbn	Q9R207	Nibrin	1.01	0.66
Serpinb6b	O08804	NK13	1.01	0.43
Eri3	Q8C460	ERI1 exoribonuclease 3	1.01	0.57
Rhog	P84096	Rho-related GTP-binding protein RhoG	1.01	0.34
Rdh13	Q8CEE7	Retinol dehydrogenase 13	1.01	0.63
Fkbp2	P45878	Peptidyl-prolyl cis-trans isomerase FKBP2	1.01	0.52
Eed	Q921E6	Polycomb protein EED	1.01	0.58
Zcchc8	Q9CYA6	Zinc finger CCHC domain-containing protein 8	1.01	0.63
Washc5	Q8C2E7	WASH complex subunit 5	1.01	0.57
Ndufs1	Q91VD9	NADH-ubiquinone oxidoreductase 75 kDa subunit, mitochondrial	1.01	0.47
Arf6	P62331	ADP-ribosylation factor 6	1.01	0.39
Dbr1	Q923B1	Lariat debranching enzyme	1.01	0.53
Fam91a1	Q3UVG3	Protein FAM91A1	1.01	0.43
Tcof1	H3BL37	Treacle protein	1.01	0.57
Lancl2	Q9JJK2	LanC-like protein 2	1.01	0.43
Fyn	P39688	Tyrosine-protein kinase Fyn	1.01	0.65
Tnrc6b	Q8BKI2	Trinucleotide repeat-containing gene 6B protein	1.01	0.62
Trmt1l	A0A0R4J0U8	RIKEN cDNA 1190005F20, isoform CRA_a	1.01	0.63
Zmynd11	D3YXX1	Zinc finger MYND domain-containing protein 11	1.01	0.88
Tsc22d4	Q9EQN3	TSC22 domain family protein 4	1.01	0.65
Pgm3	Q9CYR6	Phosphoacetylglucosamine mutase	1.01	0.47
Ascc2	Q91WR3	Activating signal cointegrator 1 complex subunit 2	1.01	0.52
Rsrc1	Q9DBU6	Serine/Arginine-related protein 53	1.01	0.74
Ganab	Q8BHN3	Neutral alpha-glucosidase AB	1.01	0.25
Gatd3a	Q9D172	Glutamine amidotransferase-like class 1 domain-containing protein 3A, mitochondrial	1.01	0.63
Huwe1	A2AFQ0	E3 ubiquitin-protein ligase HUWE1	1.01	0.52
Khdc4	Q3TCX3	KH homology domain-containing protein 4	1.01	0.73
Lsm14a	Q8K2F8	Protein LSM14 homolog A	1.01	0.62
Riok2	Q9CQS5	Serine/threonine-protein kinase RIO2	1.01	0.63
Hnmt	Q91VF2	Histamine N-methyltransferase	1.01	0.59
Clasp2	A0A1L1ST22	CLIP-associating protein 2	1.01	0.43
Gdpgp1	Q3TLS3	GDP-D-glucose phosphorylase 1	1.01	0.78
Hgs	Q3UMA3	Hepatocyte growth factor-regulated tyrosine kinase substrate	1.01	0.52
Pdk3	Q922H2	[Pyruvate dehydrogenase (acetyl-transferring)] kinase isozyme 3, mitochondrial	1.01	0.69
Tm9sf2	P58021	Transmembrane 9 superfamily member 2	1.01	0.62
Htt	G3X9H5	Huntingtin	1.01	0.61
Ubl7	Q91W67	Ubiquitin-like protein 7	1.01	0.61
Anapc5	Q8BTZ4	Anaphase-promoting complex subunit 5	1.01	0.84
Ttc1	Q91Z38	Tetratricopeptide repeat protein 1	1.01	0.47
Ubr2	Q6WKZ8	E3 ubiquitin-protein ligase UBR2	1.01	0.47
Tmem260	A0A2I3BQ91	Transmembrane protein 260	1.01	0.74
Lrrfip1	Q3UZ39	Leucine-rich repeat flightless-interacting protein 1	1.01	0.36
Ythdf3	Q8BYK6	YTH domain-containing family protein 3	1.01	0.49
Mef2a	Q60929	Myocyte-specific enhancer factor 2A	1.01	0.55
Gstk1	Q9DCM2	Glutathione S-transferase kappa 1	1.01	0.65
Snx15	Q91WE1	Sorting nexin-15	1.01	0.67
Abca3	Q8R420	ATP-binding cassette sub-family A member 3	1.01	0.72
Tmem11	Q8BK08	Transmembrane protein 11, mitochondrial	1.01	0.75
Ubap2	Q91VX2	Ubiquitin-associated protein 2	1.01	0.25
Ndufs2	Q91WD5	NADH dehydrogenase [ubiquinone] iron-sulfur protein 2, mitochondrial	1.01	0.51
Selenon	D3Z2R5	Selenoprotein N	1.01	0.84
Mkln1	O89050	Muskelin	1.02	0.60
Phb	P67778	Prohibitin	1.02	0.44

Tmem199	Q5SYH2	Transmembrane protein 199	1.02	0.80
Ccdc22	Q9JIG7	Coiled-coil domain-containing protein 22	1.02	0.61
Preb	Q9WUQ2	Prolactin regulatory element-binding protein	1.02	0.71
Slc10a7	Q5PT53	Sodium/bile acid cotransporter 7	1.02	0.63
Rps10	P63325	40S ribosomal protein S10	1.02	0.31
Exosc2	Q8VBV3	Exosome complex component RRP4	1.02	0.82
Immt	E9Q800	MICOS complex subunit MIC60	1.02	0.77
Rabif	Q91X96	Guanine nucleotide exchange factor MSS4	1.02	0.30
R3hdm1	E9Q9Q2	R3H domain-containing 1	1.02	0.64
Tbc1d23	A0A2I3BRD1	TBC1 domain family member 23	1.02	0.55
Pmpcb	Q9CXT8	Mitochondrial-processing peptidase subunit beta	1.02	0.51
Eif2s2	Q99L45	Eukaryotic translation initiation factor 2 subunit 2	1.02	0.45
Gatc	Q8CBY0	Glutamyl-tRNA(Gln) amidotransferase subunit C, mitochondrial	1.02	0.90
Dck	P43346	Deoxycytidine kinase	1.02	0.90
Ddx3x	Q62167	ATP-dependent RNA helicase DDX3X	1.02	0.36
Fkbp5	Q64378	Peptidyl-prolyl cis-trans isomerase FKBP5	1.02	0.60
Tlr6	Q3UV88	Toll-like receptor 6	1.02	0.83
Ap1s1	P61967	AP-1 complex subunit sigma-1A	1.02	0.33
Rbm14	Q8C2Q3	RNA-binding protein 14	1.02	0.47
Mrps31	Q61733	28S ribosomal protein S31, mitochondrial	1.02	0.50
Itga6	Q61739	Integrin alpha-6	1.02	0.54
Acot8	P58137	Acyl-coenzyme A thioesterase 8	1.02	0.49
Dgcr8	Q9EQM6	Microprocessor complex subunit DGCR8	1.02	0.63
Eif3j2	Q66JS6	Eukaryotic translation initiation factor 3 subunit J-B	1.02	0.32
Trabd	Q99JY4	TraB domain-containing protein	1.02	0.67
Gemin2	Q9CQQ4	Gem-associated protein 2	1.02	0.62
Mars2	Q499X9	Methionine--tRNA ligase, mitochondrial	1.02	0.80
Pik3c3	Q6PF93	Phosphatidylinositol 3-kinase catalytic subunit type 3	1.02	0.48
Inpp5f	Q8CDA1	Phosphatidylinositide phosphatase SAC2	1.02	0.52
Ash2l	Q91X20	Set1/Ash2 histone methyltransferase complex subunit ASH2	1.02	0.63
Ppp1r11	A5A4Y9	E3 ubiquitin-protein ligase PPP1R11	1.02	0.52
Exoc6	Q3U9D6	Exocyst complex component	1.02	0.63
Chaf1b	Q9D0N7	Chromatin assembly factor 1 subunit B	1.02	0.78
Ctps2	P70303	CTP synthase 2	1.02	0.46
Daam1	Q8BPM0	Disheveled-associated activator of morphogenesis 1	1.02	0.82
Lrpprc	Q6PB66	Leucine-rich PPR motif-containing protein, mitochondrial	1.02	0.46
Ppfibp2	G3X957	Liprin-beta-2	1.02	0.68
Ndufaf5	A2APY7	Arginine-hydroxylase NDUFAF5, mitochondrial	1.02	0.83
Pam	P97467	Peptidyl-glycine alpha-amidating monooxygenase	1.02	0.87
Nus1	Q99LJ8	Dehydrodolichyl diphosphate synthase complex subunit Nus1	1.02	0.79
Mrps10	G5E8U5	28S ribosomal protein S10, mitochondrial	1.02	0.64
Polr2b	Q8CFI7	DNA-directed RNA polymerase II subunit RPB2	1.02	0.51
Zcchc2	Q69ZB8	Zinc finger CCHC domain-containing protein 2	1.02	0.88
Prmt5	A0A0R4J049	Protein arginine N-methyltransferase 5	1.02	0.57
Kif21b	Q9QXL1	Kinesin-like protein KIF21B	1.02	0.67
Rsf1	E9PWW9	Remodeling and spacing factor 1	1.02	0.79
Usp3	Q91W36	Ubiquitin carboxyl-terminal hydrolase 3	1.02	0.66
Angptl2	Q9R045	Angiopoietin-related protein 2	1.02	0.66
Fbh1	Q8K2I9	F-box DNA helicase 1	1.02	0.82
Nmd3	Q99L48	60S ribosomal export protein NMD3	1.02	0.53
Abraxas2	Z4YJY0	BRISC complex subunit Abraxas 2	1.02	0.59
Rars2	Q3U186	Probable arginine--tRNA ligase, mitochondrial	1.02	0.68
Cops7a	Q9CZ04	COP9 signalosome complex subunit 7a	1.02	0.60
Chmp1b2	Q9CQD4	Charged multivesicular body protein 1b-2	1.02	0.63
Elmo2	Q8BHL5	Engulfment and cell motility protein 2	1.02	0.56
Adam17	E9PXU2	Disintegrin and metalloproteinase domain-containing protein 17	1.02	0.76
2610301B20Rik	B1AV75	RIKEN cDNA 2610301B20 gene	1.02	0.74
Sdf2	A0A0R4IZW9	Stromal cell-derived factor 2	1.02	0.76
Eif2d	Q61211	Eukaryotic translation initiation factor 2D	1.02	0.75
Zswim8	Q3UHH1	Zinc finger SWIM domain-containing protein 8	1.02	0.74
Arfip2	Q8K221	Arfaptin-2	1.02	0.66
Kdelc2	G5E897	KDEL (Lys-Asp-Glu-Leu) containing 2, isoform CRA_b	1.02	0.69
Triobp	Q99KW3	TRIO and F-actin-binding protein	1.02	0.67
Prkra	Q9WTX2	Interferon-inducible double-stranded RNA-dependent protein kinase activator A	1.02	0.58
Mrpl53	Q9D1H8	39S ribosomal protein L53, mitochondrial	1.02	0.65
Pld1	D6RH77	Phospholipase	1.02	0.63
Vps8	D3YUP0	Vacuolar protein sorting-associated protein 8 homolog	1.02	0.58
Cog4	Q8R1U1	Conserved oligomeric Golgi complex subunit 4	1.02	0.71
Stk17b	Q8BG48	Serine/threonine-protein kinase 17B	1.02	0.69
Panx1	Q9JIP4	Pannexin-1	1.02	0.73
Gsn	A0A0J9YUQ8	Gelsolin (Fragment)	1.02	0.53
Yars2	Q8BYL4	Tyrosine--tRNA ligase, mitochondrial	1.02	0.77
Upf3b	Q3ULL6	UPF3 regulator of nonsense transcripts homolog B (yeast)	1.02	0.56

Tor3a	Q9ER38	Torsin-3A	1.02	0.44
Bloc1s2	Q9CWG9	Biogenesis of lysosome-related organelles complex 1 subunit 2	1.02	0.65
Klhl42	Q8BFQ9	Kelch-like protein 42	1.02	0.73
Ccdc134	Q8C7V8	Coiled-coil domain-containing protein 134	1.02	0.57
Parvb	Q9ES46	Beta-parvin	1.02	0.32
Ncbp3	Q8BZR9	Nuclear cap-binding protein subunit 3	1.02	0.69
Phyhd1	A0A0R4J137	Phytanoyl-CoA dioxygenase domain-containing protein 1	1.02	0.70
Tnpo2	Q99LG2	Transportin-2	1.02	0.64
Tmem62	Q8BXJ9	Transmembrane protein 62	1.02	0.73
Spast	Q9QYY8	Spastin	1.02	0.73
Erap1	Q9EQH2	Endoplasmic reticulum aminopeptidase 1	1.02	0.53
Mink1	Q5SXG3	Misshapen-like kinase 1	1.02	0.64
Aldh18a1	Q9Z110	Delta-1-pyrroline-5-carboxylate synthase	1.02	0.54
Slc25a40	Q8BGP6	Solute carrier family 25 member 40	1.02	0.60
Sf3b1	G5E866	Splicing factor 3B subunit 1	1.02	0.44
Rbbp5	Q8BX09	Retinoblastoma-binding protein 5	1.02	0.73
Slc35b2	Q91ZN5	Adenosine 3'-phospho 5'-phosphosulfate transporter 1	1.02	0.48
Pid1	Q3UBG2	PTB-containing, cubilin and LRP1-interacting protein	1.02	0.62
Lgals9	G3X9T7	Galectin	1.02	0.71
Taok3	Q8BYC6	Serine/threonine-protein kinase TAO3	1.02	0.38
Dapk1	Q80YE7	Death-associated protein kinase 1	1.02	0.68
Haus5	Q9D786	HAUS augmin-like complex subunit 5	1.02	0.84
Rmnd5a	Q80YQ8	E3 ubiquitin-protein ligase RMND5A	1.02	0.53
Sumf1	Q8R0F3	Formylglycine-generating enzyme	1.02	0.52
Frg1	P97376	Protein FRG1	1.02	0.54
Kif2c	Q922S8	Kinesin-like protein KIF2C	1.02	0.92
Scrn2	Q8VCA8	Secernin-2	1.02	0.76
Ccdc97	Q9DBT3	Coiled-coil domain-containing protein 97	1.02	0.78
Dpep2	Q8C255	Dipeptidase 2	1.02	0.78
Mrps23	Q8VE22	28S ribosomal protein S23, mitochondrial	1.02	0.63
Atpaf1	H3BLL2	ATP synthase mitochondrial F1 complex assembly factor 1	1.02	0.66
Zfp326	A0A0R4J098	Zinc finger protein 326	1.02	0.47
Triap1	Q9D8Z2	TP53-regulated inhibitor of apoptosis 1	1.02	0.58
Trim47	Q8C0E3	E3 ubiquitin-protein ligase TRIM47	1.02	0.57
Ehmt2	Q9Z148	Histone-lysine N-methyltransferase EHMT2	1.02	0.78
Ndrg3	Q9QYF9	Protein NDRG3	1.02	0.72
Chtf8	P0CG14	Chromosome transmission fidelity protein 8 homolog isoform 2	1.02	0.66
Thoc2	B1AZI6	THO complex subunit 2	1.02	0.60
Man2a1	P27046	Alpha-mannosidase 2	1.02	0.49
Crtap	Q9CYD3	Cartilage-associated protein	1.02	0.65
Polb	Q8K409	DNA polymerase beta	1.02	0.50
Lonp2	Q9DBN5	Lon protease homolog 2, peroxisomal	1.02	0.75
Lman2	Q9DBH5	Vesicular integral-membrane protein VIP36	1.02	0.47
Dgkz	A2AHK0	Diacylglycerol kinase	1.02	0.56
Tab1	Q8CF89	TGF-beta-activated kinase 1 and MAP3K7-binding protein 1	1.02	0.68
Crk	Q64010	Adapter molecule crk	1.02	0.50
Epb41	A2A841	Protein 4.1	1.02	0.70
Rab7a	P51150	Ras-related protein Rab-7a	1.02	0.62
Speg	E9QQ25	Striated muscle-specific serine/threonine-protein kinase	1.02	0.72
Rpl39	P62892	60S ribosomal protein L39	1.02	0.81
Znf207	Q9JMD0	BUB3-interacting and GLEBS motif-containing protein ZNF207	1.02	0.50
Larp1	Q6ZQ58	La-related protein 1	1.02	0.50
Fchsd1	Q6PFY1	F-BAR and double SH3 domains protein 1	1.02	0.58
Armh3	Q6PD19	Armadillo-like helical domain-containing protein 3	1.02	0.49
Atg3	Q9CPX6	Ubiquitin-like-conjugating enzyme ATG3	1.02	0.56
Pip4k2a	O70172	Phosphatidylinositol 5-phosphate 4-kinase type-2 alpha	1.02	0.32
Pip5k1c	F8WHW6	Phosphatidylinositol 4-phosphate 5-kinase type-1 gamma	1.02	0.54
Pcf11	G3X9Z4	Cleavage and polyadenylation factor subunit homolog (S. cerevisiae)	1.02	0.81
Tfam	P40630	Transcription factor A, mitochondrial	1.02	0.54
Mvd	Q99JF5	Diphosphomevalonate decarboxylase	1.02	0.18
Cyfip2	Q5SQX6	Cytoplasmic FMR1-interacting protein 2	1.02	0.51
Med6	Q921D4	Mediator of RNA polymerase II transcription subunit 6	1.02	0.54
Bcat2	O35855	Branched-chain-amino-acid aminotransferase, mitochondrial	1.02	0.24
Alkbh3	A2AKV6	Alpha-ketoglutarate-dependent dioxygenase alkB homolog 3	1.02	0.53
Evi5l	H3BKQ3	Ecotropic viral integration site 5-like	1.02	0.73
Akt3	Q9WUA6	RAC-gamma serine/threonine-protein kinase	1.02	0.70
Tssc4	Q9JHE7	Protein TSSC4	1.02	0.79
Nt5dc1	Q8C5P5	5'-nucleotidase domain-containing protein 1	1.02	0.38
Prkcsh	O08795	Glucosidase 2 subunit beta	1.02	0.41
Exoc1	Q6P1Y9	Exocyst complex component 1	1.02	0.51
Med23	E9QNV2	Mediator of RNA polymerase II transcription subunit 23	1.02	0.66
Usp9x	P70398	Probable ubiquitin carboxyl-terminal hydrolase FAF-X	1.02	0.40
Hspa13	Q8BM72	Heat shock 70 kDa protein 13	1.02	0.56

Rbm25	B2RY56	RNA-binding protein 25	1.02	0.41
Kif13b	A0A286YCV9	Kinesin family member 13B	1.02	0.30
Imp4	Q8VHZ7	U3 small nucleolar ribonucleoprotein protein IMP4	1.02	0.86
Smc1a	Q9CU62	Structural maintenance of chromosomes protein 1A	1.02	0.47
Tle3	Q08122	Transducin-like enhancer protein 3	1.02	0.69
Emc8	O70378	ER membrane protein complex subunit 8	1.02	0.57
Lrba	Q9ESE1	Lipopolysaccharide-responsive and beige-like anchor protein	1.02	0.73
Thumpd3	P97770	THUMP domain-containing protein 3	1.02	0.58
Pak1ip1	Q9DCE5	p21-activated protein kinase-interacting protein 1	1.02	0.64
Rab18	P35293	Ras-related protein Rab-18	1.02	0.65
Ak6	Q8VCP8	Adenylate kinase isoenzyme 6	1.02	0.51
	Q9CXL3	Uncharacterized protein C7orf50 homolog	1.02	0.67
Dis3l2	Q8CI75	DIS3-like exonuclease 2	1.02	0.61
Pcnt	F8VPV0	Pericentrin	1.02	0.69
Irak4	Q8R4K2	Interleukin-1 receptor-associated kinase 4	1.02	0.54
Hsd17b8	P50171	Estradiol 17-beta-dehydrogenase 8	1.02	0.60
Gstt2	Q61133	Glutathione S-transferase theta-2	1.02	0.82
Mrc1	Q61830	Macrophage mannose receptor 1	1.02	0.65
Mrps6	P58064	28S ribosomal protein S6, mitochondrial	1.02	0.71
Aacs	Q9D2R0	Acetoacetyl-CoA synthetase	1.02	0.61
Mast2	E9Q1Q1	Microtubule-associated serine/threonine-protein kinase 2	1.02	0.73
Specc1	A0A0J9YTU3	Cytospin-B	1.02	0.40
Man1b1	A2AJ15	Endoplasmic reticulum mannosyl-oligosaccharide 1,2-alpha-mannosidase	1.02	0.62
Rassf4	Q8CB96	Ras association domain-containing protein 4	1.02	0.56
Lsm3	P62311	U6 snRNA-associated Sm-like protein LSm3	1.02	0.92
Atp11b	Q6DFW5	Phospholipid-transporting ATPase	1.02	0.62
Cyb5r1	Q9DB73	NADH-cytochrome b5 reductase 1	1.02	0.56
Slk	O54988	STE20-like serine/threonine-protein kinase	1.02	0.28
Pstpip2	Q99M15	Proline-serine-threonine phosphatase-interacting protein 2	1.02	0.52
Rnf14	Q9JI90	E3 ubiquitin-protein ligase RNF14	1.02	0.51
Fxr2	Q6P5B5	Fragile X mental retardation syndrome-related protein 2	1.02	0.32
Serf1	O88892	Small EDRK-rich factor 1	1.02	0.61
Xab2	Q9DCD2	Pre-mRNA-splicing factor SYF1	1.02	0.76
Ak3	Q9WTP7	GTP:AMP phosphotransferase AK3, mitochondrial	1.02	0.31
Sh3bp4	Q921I6	SH3 domain-binding protein 4	1.02	0.83
Spred1	Q924S8	Sprouty-related, EVH1 domain-containing protein 1	1.02	0.71
Rpl37a	P61514	60S ribosomal protein L37a	1.02	0.92
Mthfs	Q9D110	5-formyltetrahydrofolate cyclo-ligase	1.02	0.74
Fnbp1l	E9PUI5	Formin-binding protein 1-like	1.02	0.69
Necap1	Q9CR95	Adaptin ear-binding coat-associated protein 1	1.02	0.43
Marcksl1	P28667	MARCKS-related protein	1.02	0.76
Xpr1	Q9Z0U0	Xenotropic and polytropic retrovirus receptor 1	1.02	0.57
Armt1	A6H630	Protein-glutamate O-methyltransferase	1.02	0.48
Usp16	Q99LG0	Ubiquitin carboxyl-terminal hydrolase 16	1.02	0.70
Rps6ka1	F6Q8A4	Ribosomal protein S6 kinase alpha-1 (Fragment)	1.02	0.56
Mut	P16332	Methylmalonyl-CoA mutase, mitochondrial	1.02	0.65
Slc9a7	Q8BLV3	Sodium/hydrogen exchanger 7	1.02	0.58
Pdk1	Q8BFP9	[Pyruvate dehydrogenase (acetyl-transferring)] kinase isozyme 1, mitochondrial	1.02	0.64
Mthfd1	Q922D8	C-1-tetrahydrofolate synthase, cytoplasmic	1.02	0.42
Wbp4	Q61048	WW domain-binding protein 4	1.02	0.45
Arf4	P61750	ADP-ribosylation factor 4	1.02	0.26
Bmp2k	Q91Z96	BMP-2-inducible protein kinase	1.02	0.60
Cnot2	Q8C5L3	CCR4-NOT transcription complex subunit 2	1.02	0.56
Csnk1d	Q9DC28	Casein kinase I isoform delta	1.02	0.33
Ptgfrn	Q9WV91	Prostaglandin F2 receptor negative regulator	1.02	0.87
Gm27029	A2A4P4	Predicted gene, 27029	1.02	0.65
Alox5ap	P30355	Arachidonate 5-lipoxygenase-activating protein	1.02	0.60
Fam98a	Q3TJZ6	Protein FAM98A	1.02	0.39
Aph1c	Q9DCZ9	Putative gamma-secretase subunit APH-1C	1.02	0.69
Tubgcp6	G5E8P0	Gamma-tubulin complex component 6	1.02	0.69
Dock1	Q8BUR4	Dedicator of cytokinesis protein 1	1.02	0.50
Nsmce4a	G3XA30	MCG1618, isoform CRA_c	1.02	0.78
Capn5	O08688	Calpain-5	1.02	0.57
Cog8	Q9JJA2	Conserved oligomeric Golgi complex subunit 8	1.02	0.52
Naa25	Q8BWZ3	N-alpha-acetyltransferase 25, NatB auxiliary subunit	1.02	0.52
Clpx	Q9JHS4	ATP-dependent Clp protease ATP-binding subunit clpX-like, mitochondrial	1.02	0.63
Slc12a6	Q924N4	Solute carrier family 12 member 6	1.02	0.75
Ubash3b	Q8BGG7	Ubiquitin-associated and SH3 domain-containing protein B	1.02	0.40
Sco2	Q8VCL2	Protein SCO2 homolog, mitochondrial	1.02	0.73
Ryden	Q8CAK3	Repressor of yield of DENV protein homolog	1.02	0.70
Mre11	Q61216	Double-strand break repair protein MRE11	1.02	0.61
Nelfcd	Q3TW27	Negative elongation factor D	1.02	0.76
Mdp1	Q9D967	Magnesium-dependent phosphatase 1	1.02	0.66

Prkaa1	Q5EG47	5'-AMP-activated protein kinase catalytic subunit alpha-1	1.02	0.41
Wnk1	P83741	Serine/threonine-protein kinase WNK1	1.02	0.40
Flnc	Q8VHX6	Filamin-C	1.02	0.67
Runx1	Q3UM65	Runt-related transcription factor	1.02	0.84
Utp15	Q8C7V3	U3 small nucleolar RNA-associated protein 15 homolog	1.02	0.47
Cdk18	Q04899	Cyclin-dependent kinase 18	1.02	0.20
Thoc1	Q8R3N6	THO complex subunit 1	1.02	0.47
Cnot9	Q9JKY0	CCR4-NOT transcription complex subunit 9	1.02	0.58
Tmem175	Q9CXY1	Endosomal/lysosomal potassium channel TMEM175	1.02	0.80
Tmem126a	Q9D8Y1	Transmembrane protein 126A	1.02	0.81
Fam50a	Q9WV03	Protein FAM50A	1.02	0.47
Sptlc2	P97363	Serine palmitoyltransferase 2	1.02	0.43
Etfrf1	Q91V16	Electron transfer flavoprotein regulatory factor 1	1.02	0.64
Ino80b	Q99PT3	INO80 complex subunit B	1.02	0.75
Tlr7	P58681	Toll-like receptor 7	1.02	0.70
Naa38	Q9D2U5	N-alpha-acetyltransferase 38, NatC auxiliary subunit	1.02	0.65
Slc2a8	Q9JIF3	Solute carrier family 2, facilitated glucose transporter member 8	1.02	0.71
Wtap	E0CYH0	MCG16685, isoform CRA_d	1.02	0.65
Cd74	P04441	H-2 class II histocompatibility antigen gamma chain	1.02	0.88
Fam98b	Q80VD1	Protein FAM98B	1.02	0.46
Actl6a	Q9Z2N8	Actin-like protein 6A	1.02	0.40
Arf5	P84084	ADP-ribosylation factor 5	1.02	0.43
Cmc4	Q61908	Cx9C motif-containing protein 4	1.02	0.67
Pbdc1	Q9D0B6	Protein PBDC1	1.02	0.59
Itgb2	P11835	Integrin beta-2	1.02	0.45
Mpc2	Q9D023	Mitochondrial pyruvate carrier 2	1.02	0.43
Inpp4a	D3Z230	Type I inositol 3,4-bisphosphate 4-phosphatase	1.02	0.76
Gfer	P56213	FAD-linked sulfhydryl oxidase ALR	1.02	0.69
Actr5	A0A0A0MQ89	Actin-related protein 5	1.02	0.81
Zmiz1	Q6P1E1	Zinc finger MIZ domain-containing protein 1	1.02	0.71
Vps39	Q8R5L3	Vam6/Vps39-like protein	1.02	0.47
Mtmr12	Q80TA6	Myotubularin-related protein 12	1.02	0.76
Jmjd1c	G3UZM1	Probable JmjC domain-containing histone demethylation protein 2C	1.02	0.66
Uggt1	Q6P5E4	UDP-glucose:glycoprotein glucosyltransferase 1	1.02	0.34
Tbc1d2b	Q3U0J8	TBC1 domain family member 2B	1.02	0.48
Rufy3	A0A0G2JFT8	Protein RUFY3	1.02	0.47
Rnmt	Q9D0L8	mRNA cap guanine-N7 methyltransferase	1.02	0.48
Hck	F6UND7	Tyrosine-protein kinase	1.02	0.49
Znf276	Q8CE64	Zinc finger protein 276	1.02	0.85
Kntc1	Q8C3Y4	Kinetochore-associated protein 1	1.02	0.64
Acadvl	P50544	Very long-chain specific acyl-CoA dehydrogenase, mitochondrial	1.02	0.49
Sec22b	O08547	Vesicle-trafficking protein SEC22b	1.02	0.53
Psme4	Q5SSW2	Proteasome activator complex subunit 4	1.02	0.70
Psmd7	P26516	26S proteasome non-ATPase regulatory subunit 7	1.02	0.43
Btk	P35991	Tyrosine-protein kinase BTK	1.02	0.39
Srp19	Q9D104	Signal recognition particle 19 kDa protein	1.02	0.52
Maged1	Q9QYH6	Melanoma-associated antigen D1	1.02	0.60
Dmxl1	Q6PNC0	DmX-like protein 1	1.02	0.46
Mrpl47	Q8K2Y7	39S ribosomal protein L47, mitochondrial	1.02	0.37
Fam49a	Q8BHZ0	Protein FAM49A	1.02	0.55
Tnfaip8	D3Z325	Tumor necrosis factor alpha-induced protein 8	1.02	0.29
Eif4g2	G3XA17	Eukaryotic translation initiation factor 4 gamma 2	1.02	0.33
Ccdc25	Q78PG9	Coiled-coil domain-containing protein 25	1.02	0.54
Ighm	A0A075B6A0	Immunoglobulin heavy constant mu (Fragment)	1.02	0.91
Gins4	Q99LZ3	DNA replication complex GINS protein SLD5	1.02	0.52
Eogt	Q8BYW9	EGF domain-specific O-linked N-acetylglucosamine transferase	1.02	0.44
Cdc40	Q9DC48	Pre-mRNA-processing factor 17	1.02	0.71
C330007P06Rik	Z4YLD0	RIKEN cDNA C330007P06 gene	1.02	0.52
Myl12b	Q3THE2	Myosin regulatory light chain 12B	1.02	0.48
Cog2	Q921L5	Conserved oligomeric Golgi complex subunit 2	1.02	0.60
Slc25a19	Q9DAM5	Mitochondrial thiamine pyrophosphate carrier	1.02	0.85
Myd88	P22366	Myeloid differentiation primary response protein MyD88	1.02	0.54
Wdr91	Q7TMQ7	WD repeat-containing protein 91	1.02	0.50
Peak1	Q69Z38	Inactive tyrosine-protein kinase PEAK1	1.02	0.25
Fosl2	P47930	Fos-related antigen 2	1.02	0.62
Suclg1	Q9WUM5	Succinate--CoA ligase [ADP/GDP-forming] subunit alpha, mitochondrial	1.02	0.43
Fbxl18	E9PYR1	F-box and leucine-rich repeat protein 18	1.02	0.55
Ubqln4	Q99NB8	Ubiquilin-4	1.02	0.73
Ttc33	Q9D6K7	Tetratricopeptide repeat protein 33	1.02	0.70
Madd	A2AGQ8	MAP kinase-activating death domain protein	1.02	0.57
Abcd2	A0A0R4J0U5	ATP-binding cassette sub-family D member 2	1.02	0.63
Fmnl2	A2APV2	Formin-like protein 2	1.02	0.57
Atg5	Q99J83	Autophagy protein 5	1.02	0.07

Dip2c	E9PWR4	Disco-interacting protein 2 homolog C	1.02	0.71
Rap1b	Q99JI6	Ras-related protein Rap-1b	1.02	0.30
Akap8	Q9DBR0	A-kinase anchor protein 8	1.02	0.64
Cxxc1	Q9CWW7	CXXC-type zinc finger protein 1	1.02	0.70
Cdc23	A0A0R4J1W7	CDC23 (Cell division cycle 23, yeast, homolog), isoform CRA_c	1.02	0.78
Xrcc1	Q60596	DNA repair protein XRCC1	1.02	0.71
Rabep2	Q91WG2	Rab GTPase-binding effector protein 2	1.02	0.48
Itgav	P43406	Integrin alpha-V	1.02	0.42
Rap2c	Q8BU31	Ras-related protein Rap-2c	1.02	0.36
Cyld	Z4YJJ5	Ubiquitin carboxyl-terminal hydrolase CYLD	1.02	0.71
Dhx30	Q99PU8	ATP-dependent RNA helicase DHX30	1.02	0.62
Ints4	Q8CIM8	Integrator complex subunit 4	1.02	0.73
Ubqln2	Q9QZM0	Ubiquilin-2	1.02	0.66
Coa7	Q921H9	Cytochrome c oxidase assembly factor 7	1.02	0.55
Clasp2	Q08EB5	CLIP-associating protein 2	1.02	0.50
Atg4c	Q811C2	Cysteine protease ATG4C	1.02	0.73
Qdpr	Q8BVI4	Dihydropteridine reductase	1.02	0.53
Eif4h	Q9WUK2	Eukaryotic translation initiation factor 4H	1.02	0.30
Hnrnph3	D3Z3N4	Heterogeneous nuclear ribonucleoprotein H3	1.02	0.52
Golph3	Q9CRA5	Golgi phosphoprotein 3	1.02	0.59
Xrn1	F8VQ87	5'-3' exoribonuclease 1	1.02	0.41
Adap1	E9PY16	ArfGAP with dual PH domains 1	1.02	0.43
Zranb2	Q9R020	Zinc finger Ran-binding domain-containing protein 2	1.02	0.41
H2afx	P27661	Histone H2AX	1.02	0.55
Tbc1d9b	Q5SVR0	TBC1 domain family member 9B	1.02	0.52
Ttc39b	Q8BYY4	Tetratricopeptide repeat protein 39B	1.02	0.37
Ppp3r1	Q63810	Calcineurin subunit B type 1	1.02	0.33
Add1	Q9QYC0	Alpha-adducin	1.02	0.59
Myef2	Q8C854	Myelin expression factor 2	1.02	0.55
Ranbp10	A0A0R4J0G4	Ran-binding protein 10	1.02	0.59
Rufy1	Q8BIJ7	RUN and FYVE domain-containing protein 1	1.02	0.40
Ints3	Q7TPD0	Integrator complex subunit 3	1.02	0.54
Mmp19	Q9JHI0	Matrix metalloproteinase-19	1.02	0.83
Stk38	Q91VJ4	Serine/threonine-protein kinase 38	1.02	0.43
Tbl1x	Q9QXE7	F-box-like/WD repeat-containing protein TBL1X	1.02	0.45
Plod3	Q9R0E1	Procollagen-lysine,2-oxoglutarate 5-dioxygenase 3	1.02	0.50
Hspg2	E9PZ16	Basement membrane-specific heparan sulfate proteoglycan core protein	1.02	0.72
Rnf214	Q8BFU3	RING finger protein 214	1.02	0.38
Cd40	P27512	Tumor necrosis factor receptor superfamily member 5	1.02	0.65
Tpr	F6ZDS4	Nucleoprotein TPR	1.02	0.56
Mical1	Q8VDP3	[F-actin]-monooxygenase MICAL1	1.02	0.50
Rpl36	Q6ZWZ4	60S ribosomal protein L36	1.02	0.50
Ppp4r3b	Q922R5	Serine/threonine-protein phosphatase 4 regulatory subunit 3B	1.02	0.70
Cav2	Q9WVC3	Caveolin-2	1.02	0.77
Nae1	Q8VBW6	NEDD8-activating enzyme E1 regulatory subunit	1.02	0.37
Cnot8	Q9D8X5	CCR4-NOT transcription complex subunit 8	1.02	0.84
Gps2	Q921N8	G protein pathway suppressor 2	1.02	0.68
Hmgn2	F6W687	Non-histone chromosomal protein HMG-17 (Fragment)	1.02	0.65
Rab23	P35288	Ras-related protein Rab-23	1.02	0.83
Tnfrsf26	P83626	Tumor necrosis factor receptor superfamily member 26	1.02	0.71
Fxyd5	P97808	FXYD domain-containing ion transport regulator 5	1.02	0.42
Fuca2	Q99KR8	Plasma alpha-L-fucosidase	1.02	0.49
Sh3bp2	E9QJU7	SH3 domain-binding protein 2	1.02	0.69
Phc3	Q8CHP6	Polyhomeotic-like protein 3	1.02	0.59
Utp14a	Q640M1	U3 small nucleolar RNA-associated protein 14 homolog A	1.02	0.54
Rabgap1	A2AWA9	Rab GTPase-activating protein 1	1.02	0.33
Chmp2b	Q8BJF9	Charged multivesicular body protein 2b	1.02	0.40
Zc3hav1	D3Z5I1	Zinc finger CCCH-type antiviral protein 1	1.02	0.45
Cd180	Q62192	CD180 antigen	1.02	0.61
2900026A02Rik	A0A1B0GR85	RIKEN cDNA 2900026A02 gene	1.02	0.78
Stim2	I1E4X8	Stromal interaction molecule 2	1.02	0.73
Ric1	E9QPA1	RAB6A-GEF complex partner protein 1	1.02	0.86
Mrpl24	Q9CQ06	39S ribosomal protein L24, mitochondrial	1.02	0.52
	Q8K039	Uncharacterized protein KIAA1143 homolog	1.02	0.67
Tlr9	Q9EQU3	Toll-like receptor 9	1.02	0.57
Rps6ka5	Q8C050	Ribosomal protein S6 kinase alpha-5	1.02	0.68
Lamb2	Q61292	Laminin subunit beta-2	1.02	0.86
Pitpnb	P53811	Phosphatidylinositol transfer protein beta isoform	1.02	0.30
Tfrc	Q62351	Transferrin receptor protein 1	1.02	0.63
Cluh	A0A0R4J140	Clustered mitochondria protein homolog	1.02	0.64
Mepce	Q8K3A9	7SK snRNA methylphosphate capping enzyme	1.02	0.56
Eif4g1	Q6NZJ6	Eukaryotic translation initiation factor 4 gamma 1	1.02	0.18
Prkch	P23298	Protein kinase C eta type	1.02	0.66

Alg8	Q6P8H8	Probable dolichyl pyrophosphate Glc1Man9GlcNAc2 alpha-1,3-glucosyltransferase	1.02	0.69
Ilvbl	Q8BU33	Acetolactate synthase-like protein	1.02	0.62
Ube4a	G3X9Y5	Ubiquitin conjugation factor E4 A	1.02	0.60
Ubl4a	P21126	Ubiquitin-like protein 4A	1.02	0.53
Sh3bgrl	Q9JJU8	SH3 domain-binding glutamic acid-rich-like protein	1.02	0.46
Reps1	O54916	RalBP1-associated Eps domain-containing protein 1	1.02	0.39
Kif3a	B1AQZ2	Kinesin-like protein	1.02	0.41
Lima1	Q9ERG0	LIM domain and actin-binding protein 1	1.02	0.46
Rad1	Q9QWZ1	Cell cycle checkpoint protein RAD1	1.02	0.64
Gm21992	F7BGR7	Predicted gene 21992	1.02	0.74
Ube2q1	Q7TSS2	Ubiquitin-conjugating enzyme E2 Q1	1.02	0.47
Gtf2f2	Q8R0A0	General transcription factor IIF subunit 2	1.02	0.39
Rab6a	P35279	Ras-related protein Rab-6A	1.02	0.55
Pgghg	Q8BP56	Protein-glucosylgalactosylhydroxylysine glucosidase	1.02	0.75
Mmtag2	Q99LX5	Multiple myeloma tumor-associated protein 2 homolog	1.02	0.53
Pitrm1	Q8K411	Presequence protease, mitochondrial	1.02	0.49
Safb2	Q80YR5	Scaffold attachment factor B2	1.02	0.43
Vcpip1	A0A0R4J0M9	Deubiquitinating protein VCIP135	1.02	0.35
Rad9a	Q9Z0F6	Cell cycle checkpoint control protein RAD9A	1.02	0.79
Slbp	P97440	Histone RNA hairpin-binding protein	1.02	0.70
Dicer1	F8VQ54	Endoribonuclease Dicer	1.02	0.56
Hspa14	Q99M31	Heat shock 70 kDa protein 14	1.02	0.39
Rcl1	Q9JJT0	RNA 3'-terminal phosphate cyclase-like protein	1.02	0.26
Fbxo30	Q8BJL1	F-box only protein 30	1.02	0.61
Ubqln1	Q8R317	Ubiquilin-1	1.02	0.49
Ankrd44	B2RXR6	Serine/threonine-protein phosphatase 6 regulatory ankyrin repeat subunit B	1.02	0.38
Cyhr1	H3BIV6	Cysteine and histidine-rich protein 1	1.02	0.58
Dip2b	Q3UH60	Disco-interacting protein 2 homolog B	1.02	0.64
Naxe	Q8K4Z3	NAD(P)H-hydrate epimerase	1.02	0.59
Oxr1	E9Q0A7	Oxidation resistance protein 1	1.02	0.47
Gpnmb	Q99P91	Transmembrane glycoprotein NMB	1.02	0.23
Timm29	Q8BGX2	Mitochondrial import inner membrane translocase subunit Tim29	1.02	0.64
Pex11b	Q9Z210	Peroxisomal membrane protein 11B	1.02	0.66
Nol6	Q8R5K4	Nucleolar protein 6	1.02	0.68
Ehbp1l1	E9QP49	EH domain-binding protein 1-like protein 1	1.02	0.54
Keap1	Q9Z2X8	Kelch-like ECH-associated protein 1	1.02	0.63
Mtif3	Q9CZD5	Translation initiation factor IF-3, mitochondrial	1.02	0.30
Fubp3	Q3TIX6	Far upstream element (FUSE)-binding protein 3	1.02	0.40
Wdr7	Q920I9	WD repeat-containing protein 7	1.02	0.52
Slc25a17	O70579	Peroxisomal membrane protein PMP34	1.02	0.31
Ilf3	A0A1L1STE4	Interleukin enhancer-binding factor 3	1.02	0.28
Arid4b	A2CG63	AT-rich interactive domain-containing protein 4B	1.02	0.90
Rhbdf2	Q80WQ6	Inactive rhomboid protein 2	1.02	0.72
Cycs	P62897	Cytochrome c, somatic	1.02	0.44
Gpatch11	A0A0R4J215	G patch domain-containing protein 11	1.02	0.44
Usp19	J3KMM1	Ubiquitin carboxyl-terminal hydrolase 19	1.02	0.47
Afg1l	Q3V384	AFG1-like ATPase	1.02	0.65
Cdc34	Q8CFI2	Ubiquitin-conjugating enzyme E2 R1	1.02	0.44
AK157302	I3ITR1	MCG50313	1.02	0.58
Auh	A0A0R4J023	Methylglutaconyl-CoA hydratase, mitochondrial	1.02	0.52
Ciao3	Q7TMW6	Cytosolic iron-sulfur assembly component 3	1.02	0.67
Tbc1d24	Q3UUG6	TBC1 domain family member 24	1.02	0.53
Slfn2	Q9Z0I6	Schlafen 2	1.02	0.70
Tacc1	F8VQ95	Transforming acidic coiled-coil-containing protein 1	1.02	0.44
Kiaa1522	A2A7S8	Uncharacterized protein KIAA1522	1.02	0.64
Elp4	Q9ER73	Elongator complex protein 4	1.02	0.63
Lat2	Q9JHL0	Linker for activation of T-cells family member 2	1.02	0.69
Gfpt1	P47856	Glutamine--fructose-6-phosphate aminotransferase [isomerizing] 1	1.02	0.52
Ppfibp1	F6S1C4	Liprin-beta-1 (Fragment)	1.02	0.04
Ralgapb	A2ACC6	Ral GTPase-activating protein subunit beta	1.02	0.55
Brap	Q99MP8	BRCA1-associated protein	1.02	0.56
Pan3	H3BKF3	PAN2-PAN3 deadenylation complex subunit PAN3	1.02	0.62
Snx30	Q8CE50	Sorting nexin-30	1.02	0.43
Cntrl	A2AL36	Centriolin	1.02	0.76
Son	H9KV00	Protein SON	1.02	0.49
Myo1c	Q9WTI7	Unconventional myosin-Ic	1.02	0.44
Irf8	P23611	Interferon regulatory factor 8	1.02	0.55
Ndufs6	P52503	NADH dehydrogenase [ubiquinone] iron-sulfur protein 6, mitochondrial	1.02	0.30
Mob1b	Q3UDM0	MOB kinase activator 1B	1.02	0.47
Xpo5	Q924C1	Exportin-5	1.02	0.68
Ermp1	Q3UVK0	Endoplasmic reticulum metallopeptidase 1	1.02	0.52
Ephx1	Q9D379	Epoxide hydrolase 1	1.02	0.70
Wdfy4	E9Q2M9	WD repeat and FYVE domain-containing 4	1.02	0.47

Fbxo4	Q8CHQ0	F-box only protein 4	1.02	0.51
Ctu1	Q99J10	Cytoplasmic tRNA 2-thiolation protein 1	1.02	0.75
Nfya	Q9DBV7	Nfya protein	1.02	0.36
Mtch1	Q791T5	Mitochondrial carrier homolog 1	1.02	0.53
Rbm27	Q5SFM8	RNA-binding protein 27	1.02	0.70
Mrps22	Q9CXW2	28S ribosomal protein S22, mitochondrial	1.02	0.53
Fnbp4	A0A0R4IZZ6	Formin-binding protein 4	1.02	0.44
Sart1	Q9Z315	U4/U6.U5 tri-snRNP-associated protein 1	1.02	0.54
Api5	O35841	Apoptosis inhibitor 5	1.02	0.43
Rab3gap2	E9QKE4	Rab3 GTPase-activating protein non-catalytic subunit	1.02	0.45
Mad2l1	Q9Z1B5	Mitotic spindle assembly checkpoint protein MAD2A	1.02	0.68
Slc1a5	Q9ESU7	Amino acid transporter	1.02	0.76
Sumo2	P61957	Small ubiquitin-related modifier 2	1.02	0.46
Atp5c1	A2AKU9	ATP synthase subunit gamma	1.02	0.47
Mtatp8	P03930	ATP synthase protein 8	1.02	0.46
Gbf1	Q6DFZ1	Golgi-specific brefeldin A-resistance factor 1	1.02	0.55
Mrpl13	Q9D1P0	39S ribosomal protein L13, mitochondrial	1.02	0.74
Otud4	B2RRE7	OTU domain-containing protein 4	1.02	0.30
Arhgap9	Q1HDU4	ArhGAP9	1.02	0.47
Ylpm1	D3YWX2	YLP motif-containing protein 1	1.02	0.56
Cant1	Q8VCF1	Soluble calcium-activated nucleotidase 1	1.02	0.68
Trip11	E9Q512	Thyroid hormone receptor interactor 11	1.02	0.58
Naa40	Q8VE10	N-alpha-acetyltransferase 40	1.02	0.61
Lcmt1	A0A0U1RNF2	Leucine carboxyl methyltransferase 1	1.02	0.44
Cyba	Q61462	Cytochrome b-245 light chain	1.02	0.41
Ndufa11	G5E814	MCG5603	1.02	0.68
Rpl23a	P62751	60S ribosomal protein L23a	1.02	0.34
Mrpl11	Q9CQF0	39S ribosomal protein L11, mitochondrial	1.02	0.49
Apobr	Q8VBT6	Apolipoprotein B receptor	1.02	0.25
	Q8VE95	UPF0598 protein C8orf82 homolog OS=Mus musculus OX=10090	1.02	0.71
Gatb	Q99JT1	Glutamyl-tRNA(Gln) amidotransferase subunit B, mitochondrial	1.02	0.61
Nup205	A0A0J9YUD5	Nucleoporin 205	1.02	0.49
Ppil3	Q9D6L8	Peptidyl-prolyl cis-trans isomerase-like 3	1.02	0.63
Zc3h12d	E9QNR7	Probable ribonuclease ZC3H12D	1.02	0.58
Mrpl44	Q9CY73	39S ribosomal protein L44, mitochondrial	1.02	0.47
Atp6ap1	Q9R1Q9	V-type proton ATPase subunit S1	1.02	0.52
Clcn7	O70496	H(+)/Cl(-) exchange transporter 7	1.02	0.65
Arfgap3	A0A0R4J0T8	ADP-ribosylation factor GTPase-activating protein 3	1.02	0.38
Golga2	E9PUQ5	Golgin subfamily A member 2	1.02	0.39
Gtf3c2	Q8BL74	General transcription factor 3C polypeptide 2	1.02	0.42
Adck5	E9PUK2	Uncharacterized aarF domain-containing protein kinase 5	1.02	0.79
Itpkb	B2RXC2	Kinase	1.02	0.51
Sri	Q6P069	Sorcin	1.02	0.21
Wbp11	Q923D5	WW domain-binding protein 11	1.02	0.43
Ero1a	Q8R180	ERO1-like protein alpha	1.02	0.31
Dgke	Q9R1C6	Diacylglycerol kinase epsilon	1.02	0.58
Ptpro	E9Q4I1	Receptor-type tyrosine-protein phosphatase O	1.02	0.48
Col4a2	P08122	Collagen alpha-2(IV) chain	1.02	0.73
Dync1li2	Q6PDL0	Cytoplasmic dynein 1 light intermediate chain 2	1.02	0.31
Asph	Q8BSY0	Aspartyl/asparaginyl beta-hydroxylase	1.02	0.30
Armc6	Q8BNU0	Armadillo repeat-containing protein 6	1.02	0.58
Mettl16	Q5SW15	U6 small nuclear RNA (adenine-(43)-N(6))-methyltransferase	1.02	0.55
Ppp1r18	Q8BQ30	Phostensin	1.02	0.23
Map3k2	G5E8L8	Mitogen-activated protein kinase kinase kinase 2	1.02	0.08
Ndufaf3	Q9JKL4	NADH dehydrogenase [ubiquinone] 1 alpha subcomplex assembly factor 3	1.02	0.72
Ddx27	Q921N6	Probable ATP-dependent RNA helicase DDX27	1.02	0.27
Gm10320	E9PW43	Protein transport protein Sec61 subunit beta	1.02	0.69
Spcs3	Q6ZWQ7	Signal peptidase complex subunit 3	1.02	0.67
Sgpl1	Q8R0X7	Sphingosine-1-phosphate lyase 1	1.02	0.51
Fcgrt	Q6PKB0	Fcgrt protein	1.02	0.51
Septin8	B1AQZ0	Septin-8	1.02	0.53
Rabl6	Q5U3K5	Rab-like protein 6	1.02	0.51
Calcoco1	Q8CGU1	Calcium-binding and coiled-coil domain-containing protein 1	1.02	0.54
Stard3	Q61542	StAR-related lipid transfer protein 3	1.02	0.70
Rpl10a	Q5XJF6	Ribosomal protein	1.02	0.46
Ap2m1	Q3TWV4	AP-2 complex subunit mu	1.02	0.55
Lemd3	D3YU56	Inner nuclear membrane protein Man1	1.02	0.71
Ddx49	Q4FZF3	Probable ATP-dependent RNA helicase DDX49	1.02	0.86
Nfic	A0A1Y7VK55	Nuclear factor 1	1.02	0.47
Chchd3	Q9CRB9	MICOS complex subunit Mic19	1.02	0.64
Ik	Q9Z1M8	Protein Red	1.02	0.55
Nt5dc2	A0A2I3BR81	5'-nucleotidase domain-containing 2	1.02	0.60
Tbc1d25	A1A5B6	TBC1 domain family member 25	1.02	0.85

Tpp1	O89023	Tripeptidyl-peptidase 1	1.02	0.84
Gp49a	Q61450	Mast cell surface glycoprotein Gp49A	1.02	0.72
Inpp5b	Q8K337	Type II inositol 1,4,5-trisphosphate 5-phosphatase	1.02	0.53
Med11	G3UZ31	Mediator of RNA polymerase II transcription subunit 11	1.02	0.51
Znrf2	Q71FD5	E3 ubiquitin-protein ligase ZNRF2	1.02	0.33
Renbp	P82343	N-acylglucosamine 2-epimerase	1.02	0.18
Ankrd17	Q99NH0	Ankyrin repeat domain-containing protein 17	1.02	0.70
Itgal	D3Z627	Integrin alpha-L	1.02	0.61
Fmnl1	A0A1W2P6X3	Formin-like protein 1	1.02	0.58
Ubr4	A2AN08	E3 ubiquitin-protein ligase UBR4	1.02	0.45
Nucb2	P81117	Nucleobindin-2	1.02	0.19
Pde4dip	H3BJ38	Myomegalin	1.02	0.68
Hsdl2	Q2TPA8	Hydroxysteroid dehydrogenase-like protein 2	1.02	0.59
Bag1	A0A1L1SRT0	BAG family molecular chaperone regulator 1	1.02	0.55
Ptcd3	Q14C51	Pentatricopeptide repeat domain-containing protein 3, mitochondrial	1.02	0.52
Dennd6a	Q8BH65	Protein DENND6A	1.02	0.59
LRWD1	Q8BUI3	Leucine-rich repeat and WD repeat-containing protein 1	1.02	0.81
Ccdc6	D3YZP9	Coiled-coil domain-containing protein 6	1.02	0.57
Kif1c	E9Q9B0	Kinesin-like protein KIF1C	1.02	0.54
Paip1	F6Y616	Polyadenylate-binding protein-interacting protein 1	1.02	0.62
Rpain	Q9CWY9	RPA-interacting protein	1.02	0.46
Rptor	A2ACM0	Regulatory-associated protein of mTOR	1.02	0.58
Gtpbp3	E9PWV7	tRNA modification GTPase GTPBP3, mitochondrial	1.02	0.73
Prag1	Q571I4	Inactive tyrosine-protein kinase PRAG1	1.02	0.86
Hmbs	P22907	Porphobilinogen deaminase	1.02	0.58
Rdh10	Q8VCH7	Retinol dehydrogenase 10	1.02	0.78
Stx7	O70439	Syntaxin-7	1.02	0.60
5031439G07Rik	B1APX2	RIKEN cDNA 5031439G07 gene	1.02	0.51
Hs2st1	Q8R3H7	Heparan sulfate 2-O-sulfotransferase 1	1.02	0.58
Copa	F8WHL2	Coatomer subunit alpha	1.02	0.28
Ilf2	Q9CXY6	Interleukin enhancer-binding factor 2	1.02	0.50
Rmdn3	Q3UJU9	Regulator of microtubule dynamics protein 3	1.02	0.48
Ogfod1	Q3U0K8	Prolyl 3-hydroxylase OGFOD1	1.02	0.71
Shtn1	Q8K2Q9	Shootin-1	1.02	0.32
Reep3	A0A1W2P8A8	Receptor expression-enhancing protein	1.02	0.38
Rpl22	P67984	60S ribosomal protein L22	1.02	0.61
Cpt2	P52825	Carnitine O-palmitoyltransferase 2, mitochondrial	1.02	0.31
Golga4	Q91VW5	Golgin subfamily A member 4	1.02	0.53
Atg9a	A0A087WP33	Autophagy-related protein 9 (Fragment)	1.02	0.52
Ints6	Q6PCM2	Integrator complex subunit 6	1.02	0.58
Pf4	Q9Z126	Platelet factor 4	1.02	0.89
Eif2b5	Q8CHW4	Translation initiation factor eIF-2B subunit epsilon	1.02	0.46
Cebpb	P28033	CCAAT/enhancer-binding protein beta	1.02	0.55
Gsn	A6PWS5	Gelsolin (Fragment)	1.02	0.54
Emsy	Q8BMB0	BRCA2-interacting transcriptional repressor EMSY	1.02	0.58
Kansl1	A2A5Y4	KAT8 regulatory NSL complex subunit 1	1.02	0.77
Acad9	Q8JZN5	Acyl-CoA dehydrogenase family member 9, mitochondrial	1.02	0.44
Luzp1	Q8R4U7	Leucine zipper protein 1	1.02	0.58
Ubr1	O70481	E3 ubiquitin-protein ligase UBR1	1.02	0.75
Tsnax	Q9QZE7	Translin-associated protein X	1.02	0.38
Haus7	Q8BKT8	HAUS augmin-like complex subunit 7	1.02	0.46
Cebpz	A0A0R4J046	CCAAT/enhancer-binding protein zeta	1.02	0.69
Ppp2cb	P62715	Serine/threonine-protein phosphatase 2A catalytic subunit beta isoform	1.02	0.72
Vasp	P70460	Vasodilator-stimulated phosphoprotein	1.02	0.50
Dpagt1	P42867	UDP-N-acetylglucosamine--dolichyl-phosphate N-acetylglucosaminephosphotransferase	1.02	0.53
Cln6	Q3U466	Ceroid-lipofuscinosis, neuronal 6	1.02	0.47
Synj1	D3Z656	Synaptojanin-1	1.02	0.52
Cul4a	Q3TCH7	Cullin-4A	1.02	0.35
Pdcd2l	Q8C5N5	Programmed cell death protein 2-like	1.02	0.83
Rabgef1	Q9JM13	Rab5 GDP/GTP exchange factor	1.02	0.38
P3h1	A2A7Q5	Prolyl 3-hydroxylase 1	1.02	0.37
Akt2	Q60823	RAC-beta serine/threonine-protein kinase	1.02	0.60
Acat2	Q8CAY6	Acetyl-CoA acetyltransferase, cytosolic	1.02	0.71
Ulk2	Q9QY01	Serine/threonine-protein kinase ULK2	1.02	0.75
Ptpn1	P35821	Tyrosine-protein phosphatase non-receptor type 1	1.02	0.38
Cept1	Q8BGS7	Choline/ethanolaminephosphotransferase 1	1.02	0.51
Pfkm	P47857	ATP-dependent 6-phosphofructokinase, muscle type	1.02	0.57
Aktip	D3Z2J4	AKT-interacting protein	1.02	0.42
Akt1	P31750	RAC-alpha serine/threonine-protein kinase	1.02	0.41
Aldh3b1	Q80VQ0	Aldehyde dehydrogenase family 3 member B1	1.02	0.33
Rps14	P62264	40S ribosomal protein S14	1.02	0.38
Exoc5	Q3TPX4	Exocyst complex component 5	1.02	0.50
Spg11	Q3UHA3	Spatacsin	1.02	0.64

Arfip1	G5E8V9	ADP-ribosylation factor-interacting protein 1	1.02	0.64
Gtf2h1	E9QKD9	General transcription factor IIH subunit 1	1.02	0.81
Prg4	E0CZ58	Proteoglycan 4	1.02	0.82
Kbtbd2	G3X9X1	Kelch repeat and BTB (POZ) domain-containing 2	1.02	0.70
Msto1	E9PUB7	Protein misato homolog 1	1.02	0.45
Prkaca	P05132	cAMP-dependent protein kinase catalytic subunit alpha	1.02	0.44
Vwa8	Q8CC88	von Willebrand factor A domain-containing protein 8	1.02	0.47
Ppp2r5c	Q60996	Serine/threonine-protein phosphatase 2A 56 kDa regulatory subunit gamma isoform	1.02	0.41
Phip	F8VQ93	PH-interacting protein	1.02	0.70
Was	P70315	Wiskott-Aldrich syndrome protein homolog	1.02	0.48
Rxra	P28700	Retinoic acid receptor RXR-alpha	1.02	0.60
Vps51	Q3UVL4	Vacuolar protein sorting-associated protein 51 homolog	1.02	0.44
Slc35b1	P97858	Solute carrier family 35 member B1	1.02	0.29
Smarce1	O54941	SWI/SNF-related matrix-associated actin-dependent regulator of chromatin subfamily E member 1	1.02	0.34
Pi4k2a	Q2TBE6	Phosphatidylinositol 4-kinase type 2-alpha	1.02	0.50
Rps17	P63276	40S ribosomal protein S17	1.02	0.49
Comtd1	Q8BIG7	Catechol O-methyltransferase domain-containing protein 1	1.02	0.56
Pik3cg	Q9JHG7	Phosphatidylinositol 4,5-bisphosphate 3-kinase catalytic subunit gamma isoform	1.02	0.48
Fam133b	Q9CVI2	Protein FAM133B	1.02	0.66
Esyt1	Q3U7R1	Extended synaptotagmin-1	1.02	0.38
Stk10	O55098	Serine/threonine-protein kinase 10	1.02	0.16
Trmt6	Q8CE96	tRNA (adenine(58)-N(1))-methyltransferase non-catalytic subunit TRM6	1.02	0.39
Rtf1	A2AQ19	RNA polymerase-associated protein RTF1 homolog	1.02	0.30
Stx6	Q9JKK1	Syntaxin-6	1.02	0.50
Ptrh2	Q8R2Y8	Peptidyl-tRNA hydrolase 2, mitochondrial	1.02	0.53
Ccdc50	Q810U5	Coiled-coil domain-containing protein 50	1.02	0.52
Zzef1	A0A140LJ04	Zinc finger ZZ-type and EF-hand domain-containing protein 1	1.02	0.44
Pbrm1	F8VQD1	Protein polybromo-1	1.02	0.38
Nup62	Q63850	Nuclear pore glycoprotein p62	1.02	0.40
Cog3	E9QL65	Conserved oligomeric Golgi complex subunit 3	1.02	0.48
Dop1b	Q3UHQ6	Protein dopey-2	1.02	0.51
Ints2	Q80UK8	Integrator complex subunit 2	1.02	0.66
Cd47	A0A2R8VK70	Leukocyte surface antigen CD47	1.02	0.62
Pgap3	A2A559	Post-GPI attachment to proteins factor 3	1.02	0.64
Cse1l	Q9ERK4	Exportin-2	1.02	0.27
Uqcrfs1	Q9CR68	Cytochrome b-c1 complex subunit Rieske, mitochondrial	1.02	0.41
Stap1	Q9JM90	Signal-transducing adaptor protein 1	1.02	0.20
Ntpcr	Q9CQA9	Cancer-related nucleoside-triphosphatase homolog	1.02	0.58
Itsn2	B2RR82	Intersectin-2	1.02	0.40
Atp5f1d	Q9D3D9	ATP synthase subunit delta, mitochondrial	1.02	0.44
Mgll	D3YYS6	Monoglyceride lipase	1.02	0.68
Znf598	Q80YR4	E3 ubiquitin-protein ligase ZNF598	1.02	0.61
Bsdc1	Q80Y55	BSD domain-containing protein 1	1.02	0.60
Arid1b	A0A338P6U8	AT-rich interactive domain-containing protein 1B	1.02	0.48
Mpp6	Q9JLB0	MAGUK p55 subfamily member 6	1.02	0.65
Dtx3l	Q3UIR3	E3 ubiquitin-protein ligase DTX3L	1.02	0.62
Abcf1	Q6P542	ATP-binding cassette sub-family F member 1	1.02	0.26
Smarcad1	Q04692	SWI/SNF-related matrix-associated actin-dependent regulator of chromatin subfamily A containing DEAD/H box 1	1.02	0.48
Col6a3	E9PWQ3	Collagen, type VI, alpha 3	1.02	0.72
Mff	E0CYB9	Mitochondrial fission factor	1.02	0.56
Rsrc2	S4R2L4	Arginine/serine-rich coiled-coil protein 2	1.02	0.47
Git2	E9PVA6	ARF GTPase-activating protein GIT2	1.02	0.35
Cog5	Q8C0L8	Conserved oligomeric Golgi complex subunit 5	1.02	0.50
Emc1	Q8C7X2	ER membrane protein complex subunit 1	1.02	0.37
Wdr92	Q8BGF3	WD repeat-containing protein 92	1.02	0.50
Ppp2r5d	Q91V89	Serine/threonine-protein phosphatase 2A 56 kDa regulatory subunit	1.02	0.20
Prorsd1	Q9D820	Prolyl-tRNA synthetase associated domain-containing protein 1	1.02	0.55
Nomo1	Q6GQT9	Nodal modulator 1	1.02	0.37
Cdc26	Q99JP4	Anaphase-promoting complex subunit CDC26	1.02	0.63
Micu2	Q8CD10	Calcium uptake protein 2, mitochondrial	1.02	0.55
Maea	Q4VC33	E3 ubiquitin-protein transferase MAEA	1.02	0.37
Lamtor1	Q9CQ22	Ragulator complex protein LAMTOR1	1.02	0.41
Fbl	P35550	rRNA 2'-O-methyltransferase fibrillarin	1.02	0.27
Slc12a9	Q99MR3	Solute carrier family 12 member 9	1.02	0.44
Gmds	Q8K0C9	GDP-mannose 4,6 dehydratase	1.02	0.34
Pon3	Q62087	Serum paraoxonase/lactonase 3	1.02	0.54
Tut7	Q5BLK4	Terminal uridylyltransferase 7	1.02	0.42
Mphosph6	Q9D1Q1	M-phase phosphoprotein 6	1.02	0.44
Mrps18b	Q99N84	28S ribosomal protein S18b, mitochondrial	1.02	0.42
Dnajc24	A2A4A1	DnaJ homolog subfamily C member 24	1.02	0.36
Spc25	Q3UA16	Kinetochore protein Spc25	1.02	0.67
Plcb3	P51432	1-phosphatidylinositol 4,5-bisphosphate phosphodiesterase beta-3	1.02	0.49
Lin37	Q9D8N6	Protein lin-37 homolog	1.02	0.47

Atp5pf	P97450	ATP synthase-coupling factor 6, mitochondrial	1.02	0.36
Chd8	Q09XV5	Chromodomain-helicase-DNA-binding protein 8	1.02	0.66
Dnajc10	Q9DC23	DnaJ homolog subfamily C member 10	1.02	0.36
Zwint	Q9CQU5	ZW10 interactor	1.02	0.70
Zcchc9	Q8R1J3	Zinc finger CCHC domain-containing protein 9	1.02	0.54
Ppm1b	P36993	Protein phosphatase 1B	1.02	0.70
Def6	A0A0R4IZX1	Differentially-expressed in FDCP 6	1.02	0.59
Ak1	Q9R0Y5	Adenylate kinase isoenzyme 1	1.02	0.59
Xpo7	A0A2I3BQV5	Exportin-7	1.02	0.62
Arhgef2	Q60875	Rho guanine nucleotide exchange factor 2	1.02	0.44
Igf2bp3	Q9CPN8	Insulin-like growth factor 2 mRNA-binding protein 3	1.02	0.73
Ptpn12	P35831	Tyrosine-protein phosphatase non-receptor type 12	1.02	0.43
Rnf7	Q9WTZ1	RING-box protein 2	1.02	0.54
Mtss1	G3X9H7	Metastasis suppressor 1, isoform CRA_e	1.02	0.31
Raph1	F2Z3U3	Ras association (RalGDS/AF-6) and pleckstrin homology domains 1	1.02	0.53
Rnf149	Q3U2C5	E3 ubiquitin-protein ligase RNF149	1.02	0.65
Pogz	Q8BZH4	Pogo transposable element with ZNF domain	1.02	0.36
Sh2b3	O09039	SH2B adapter protein 3	1.02	0.70
Sec16a	A2AIX1	Protein transport protein sec16	1.02	0.60
Araf	P04627	Serine/threonine-protein kinase A-Raf	1.02	0.67
Pik3ca	P42337	Phosphatidylinositol 4,5-bisphosphate 3-kinase catalytic subunit alpha isoform	1.02	0.63
Rab4b	Q91ZR1	Ras-related protein Rab-4B	1.02	0.39
Rasa4	Q6PFQ7	Ras GTPase-activating protein 4	1.02	0.50
Aldh6a1	Q9EQ20	Methylmalonate-semialdehyde dehydrogenase [acylating], mitochondrial	1.02	0.45
Pnpla2	Q8BJ56	Patatin-like phospholipase domain-containing protein 2	1.02	0.49
Znf280c	Q6P3Y5	Zinc finger protein 280C	1.02	0.69
Fscn1	Q61553	Fascin	1.02	0.78
Pcyt2	Q922E4	Ethanolamine-phosphate cytidylyltransferase	1.02	0.45
Sar1a	Q99JZ4	GTP-binding protein SAR1a	1.02	0.38
Dnaja3	Q99M87	DnaJ homolog subfamily A member 3, mitochondrial	1.02	0.62
Zyx	Q62523	Zyxin	1.02	0.28
Katna1	E9PZI6	Katanin p60 ATPase-containing subunit A1	1.02	0.32
Csad	Q9DBE0	Cysteine sulfinic acid decarboxylase	1.02	0.47
Mindy2	Q6PDI6	Ubiquitin carboxyl-terminal hydrolase MINDY-2	1.02	0.36
Ctdnep1	Q3TP92	CTD nuclear envelope phosphatase 1	1.02	0.68
Wdr75	Q3U821	WD repeat-containing protein 75	1.02	0.37
Card11	Q8CIS0	Caspase recruitment domain-containing protein 11	1.02	0.34
Mrps33	Q9D2R8	28S ribosomal protein S33, mitochondrial	1.02	0.52
Phax	Q9JJT9	Phosphorylated adapter RNA export protein	1.02	0.50
Cep135	Q6P5D4	Centrosomal protein of 135 kDa	1.02	0.75
Mtx1	F7C846	Metaxin-1	1.02	0.39
Bicd2	Q921C5	Protein bicaudal D homolog 2	1.02	0.45
Tax1bp3	Q9DBG9	Tax1-binding protein 3	1.02	0.06
Cd93	O89103	Complement component C1q receptor	1.02	0.74
Snx13	E9QNG6	Sorting nexin-13	1.02	0.63
Lyar	Q08288	Cell growth-regulating nucleolar protein	1.02	0.43
Eif1	P48024	Eukaryotic translation initiation factor 1	1.02	0.53
S100a13	A0A0A0MQ90	Protein S100-A13	1.02	0.41
Luc7l	Q9CYI4	Putative RNA-binding protein Luc7-like 1	1.02	0.30
Znfx1	Q8R151	NFX1-type zinc finger-containing protein 1	1.02	0.54
Suclg2	Q9Z2I8	Succinate--CoA ligase [GDP-forming] subunit beta, mitochondrial	1.02	0.43
Kidins220	E9Q9B7	Kinase D-interacting substrate 220	1.02	0.48
Hars2	Q99KK9	Probable histidine--tRNA ligase, mitochondrial	1.02	0.48
Mtm1	Q9Z2C5	Myotubularin	1.02	0.32
Dnase1l1	Q9D7J6	Deoxyribonuclease-1-like 1	1.02	0.62
Gabpa	Q00422	GA-binding protein alpha chain	1.02	0.52
Pip5k1a	P70182	Phosphatidylinositol 4-phosphate 5-kinase type-1 alpha	1.02	0.50
Vmp1	Q99KU0	Vacuole membrane protein 1	1.02	0.59
Lsg1	Q3UM18	Large subunit GTPase 1 homolog	1.02	0.58
Ggta1	Q9DBU1	Glycoprotein galactosyltransferase alpha 1, 3, isoform CRA_e	1.02	0.36
Znf703	P0CL69	Zinc finger protein 703	1.02	0.77
Cmss1	Q9CZT6	Protein CMSS1	1.02	0.70
Nup133	Q8R0G9	Nuclear pore complex protein Nup133	1.02	0.42
Pds5a	E9QPI5	Sister chromatid cohesion protein PDS5 homolog A	1.02	0.27
Txlna	Q6PAM1	Alpha-taxilin	1.02	0.37
Pnpla7	A2AJ88	Patatin-like phospholipase domain-containing protein 7	1.02	0.38
Mylk	B1B1A8	Myosin light chain kinase, smooth muscle	1.02	0.80
Sat2	Q6P8J2	Diamine acetyltransferase 2	1.02	0.60
Srrm1	Q52KI8	Serine/arginine repetitive matrix protein 1	1.02	0.40
Cybb	Q61093	Cytochrome b-245 heavy chain	1.02	0.39
Mccc2	Q3ULD5	Methylcrotonoyl-CoA carboxylase beta chain, mitochondrial	1.02	0.44
Gtf3c4	Q8BMQ2	General transcription factor 3C polypeptide 4	1.02	0.54
Tdrd7	A0A0A0MQD1	Tudor domain-containing protein 7	1.02	0.48

Syncrip	Q7TMK9	Heterogeneous nuclear ribonucleoprotein Q	1.02	0.25
Rrp9	Q91WM3	U3 small nucleolar RNA-interacting protein 2	1.02	0.57
Cbl	P22682	E3 ubiquitin-protein ligase CBL	1.02	0.12
Rad23a	P54726	UV excision repair protein RAD23 homolog A	1.02	0.41
Cenpv	Q9CXS4	Centromere protein V	1.02	0.90
Pomp	Q9CQT5	Proteasome maturation protein	1.02	0.64
Pigs	Q6PD26	GPI transamidase component PIG-S	1.02	0.45
Rab8b	P61028	Ras-related protein Rab-8B	1.02	0.38
Ly9	Q01965	T-lymphocyte surface antigen Ly-9	1.02	0.26
Ckap5	A2AGT5	Cytoskeleton-associated protein 5	1.02	0.49
Dock11	A2AF47	Dedicator of cytokinesis protein 11	1.02	0.57
Chchd1	Q9CQA6	Coiled-coil-helix-coiled-coil-helix domain-containing protein 1	1.02	0.72
Pum2	Q80U58	Pumilio homolog 2	1.02	0.30
Cfap36	Q8C6E0	Cilia- and flagella-associated protein 36	1.02	0.47
Ccdc88a	Q5SNZ0	Girdin	1.02	0.45
Rnasel	Q05921	2-5A-dependent ribonuclease	1.02	0.58
Uprt	B1AVZ0	Uracil phosphoribosyltransferase homolog	1.02	0.25
Map1s	Q8C052	Microtubule-associated protein 1S	1.02	0.46
Pdp1	A2AJQ0	[Pyruvate dehydrogenase [acetyl-transferring]]-phosphatase 1, mitochondrial	1.02	0.61
Grk6	O70293	G protein-coupled receptor kinase 6	1.02	0.68
Hsd17b12	O70503	Very-long-chain 3-oxoacyl-CoA reductase	1.02	0.47
Rcan1	Q9JHG6	Calcipressin-1	1.02	0.67
Itpkc	Q7TS72	Inositol-trisphosphate 3-kinase C	1.02	0.67
Trove2	O08848	60 kDa SS-A/Ro ribonucleoprotein	1.02	0.54
Ralbp1	Q62172	RalA-binding protein 1	1.02	0.54
Inpp1	P49442	Inositol polyphosphate 1-phosphatase	1.02	0.33
Tor1aip2	Q8BYU6	Torsin-1A-interacting protein 2	1.02	0.39
Plp2	Q9R1Q7	Proteolipid protein 2	1.02	0.42
Ndufb9	Q9CQJ8	NADH dehydrogenase [ubiquinone] 1 beta subcomplex subunit 9	1.02	0.52
Dpf2	D3Z5N6	Zinc finger protein ubi-d4	1.02	0.31
Cers6	H3BL08	Ceramide synthase 6	1.02	0.56
Rps26	P62855	40S ribosomal protein S26	1.02	0.37
Ahnak2 SV=8	E9PYB0	AHNAK nucleoprotein 2 (Fragment)	1.02	0.39
Hnrnpa0	Q9CX86	Heterogeneous nuclear ribonucleoprotein A0	1.02	0.86
Thtpa	Q8JZL3	Thiamine-triphosphatase	1.02	0.50
Plekha5	E9Q6H8	Pleckstrin homology domain-containing, family A member 5	1.02	0.61
Hexim1	Q8R409	Protein HEXIM1	1.02	0.46
Ece1	Q4PZA2	Endothelin-converting enzyme 1	1.02	0.65
Git1	Q5F258	ARF GTPase-activating protein GIT1	1.02	0.45
Cul4b	A2A432	Cullin-4B	1.02	0.38
Sbno1	B2RRI2	Protein strawberry notch homolog 1	1.02	0.53
Ociad1	Q9CRD0	OCIA domain-containing protein 1	1.02	0.33
Iqsec2	E9QAD8	IQ motif and SEC7 domain-containing protein 2	1.02	0.61
Clec2d	Q91V08	C-type lectin domain family 2 member D	1.02	0.57
Pusl1	A2ADA5	tRNA pseudouridine synthase-like 1	1.02	0.64
Ctnna1	P26231	Catenin alpha-1	1.02	0.40
Raly	Q64012	RNA-binding protein Raly	1.02	0.28
Ergic1	Q9DC16	Endoplasmic reticulum-Golgi intermediate compartment protein 1	1.02	0.78
Impact	O55091	Protein IMPACT	1.02	0.46
Tlr4	Q9QUK6	Toll-like receptor 4	1.02	0.74
Paip2b	Q91W45	Polyadenylate-binding protein-interacting protein 2B	1.02	0.71
Ndufa9	Q9DC69	NADH dehydrogenase [ubiquinone] 1 alpha subcomplex subunit 9, mitochondrial	1.02	0.35
1700021F05Rik	D3Z316	Protein 1700021F05Rik	1.02	0.30
Rps19	Q9CZX8	40S ribosomal protein S19	1.02	0.25
Dis3	Q9CSH3	Exosome complex exonuclease RRP44	1.02	0.44
Ralb	Q9JIW9	Ras-related protein Ral-B	1.02	0.30
Pdpr	Q7TSQ8	Pyruvate dehydrogenase phosphatase regulatory subunit, mitochondrial	1.02	0.35
Mocs1	Q5RKZ7	Molybdenum cofactor biosynthesis protein 1	1.02	0.40
Rab5a	Q9CQD1	Ras-related protein Rab-5A	1.02	0.49
Acot11	A2AVR6	Acyl-coenzyme A thioesterase 11	1.02	0.32
Ccdc93	Q7TQK5	Coiled-coil domain-containing protein 93	1.02	0.33
Nob1	Q8BW10	RNA-binding protein NOB1	1.02	0.47
Thumpd1	Q99J36	THUMP domain-containing protein 1	1.02	0.17
Ino80c	Q8BHA0	INO80 complex subunit C	1.02	0.54
Josd2	Q9CR30	Josephin-2	1.02	0.84
Vamp4	O70480	Vesicle-associated membrane protein 4	1.02	0.61
Mtnd4	P03911	NADH-ubiquinone oxidoreductase chain 4	1.02	0.53
Naip2	Q9QUK4	Baculoviral IAP repeat-containing protein 1b	1.02	0.48
Rhoa	Q9QUI0	Transforming protein RhoA	1.02	0.26
Borcs5	E9Q2W8	BLOC-1-related complex subunit 5	1.02	0.52
Cd300ld	Q8VCH2	CMRF35-like molecule 5	1.02	0.54
Ndufv2	Q9D6J6	NADH dehydrogenase [ubiquinone] flavoprotein 2, mitochondrial	1.02	0.47
Dohh	Q99LN9	Deoxyhypusine hydroxylase	1.02	0.53

Pom121	Q8K3Z9	Nuclear envelope pore membrane protein POM 121	1.02	0.52
Gga2	Q6P5E6	ADP-ribosylation factor-binding protein GGA2	1.02	0.45
Fam208a	Q69ZR9	Protein TASOR	1.02	0.82
Ttc39c	G3X8X1	RIKEN cDNA 2810439F02	1.02	0.51
Polr1d	P97304	DNA-directed RNA polymerases I and III subunit RPAC2	1.02	0.67
Bscl2	A0A0R4J225	Bernardinelli-Seip congenital lipodystrophy 2 homolog (Human), isoform CRA_a	1.02	0.58
Znf330	Q922H9	Zinc finger protein 330	1.02	0.47
Gls	D3Z7P3	Glutaminase kidney isoform, mitochondrial	1.02	0.31
Ankrd52	Q8BTI7	Serine/threonine-protein phosphatase 6 regulatory ankyrin repeat subunit C	1.02	0.61
Ifitm2	Q99J93	Interferon-induced transmembrane protein 2	1.02	0.59
Rras	P10833	Ras-related protein R-Ras	1.02	0.56
Fnip1	Q68FD7	Folliculin-interacting protein 1	1.02	0.58
Mkl2	G3X8R8	MCG123888	1.02	0.39
Mier1	Q5UAK0	Mesoderm induction early response protein 1	1.02	0.52
Faf1	P54731	FAS-associated factor 1	1.02	0.42
Cep170	H7BX26	Centrosomal protein of 170 kDa	1.02	0.40
Tf	Q921I1	Serotransferrin	1.02	0.27
Uck1	A2AN37	Uridine-cytidine kinase	1.02	0.45
Specc1l	A0A0R4J0J8	Cytospin-A	1.02	0.61
Hdac2	A0A0R4J008	Histone deacetylase	1.02	0.08
Slc43a2	Q8CGA3	Large neutral amino acids transporter small subunit 4	1.02	0.37
Seh1l	Q8R2U0	Nucleoporin SEH1	1.02	0.37
Surf6	P70279	Surfeit locus protein 6	1.02	0.51
Basp1	Q91XV3	Brain acid soluble protein 1	1.02	0.32
Rmc1	Q8VC42	Regulator of MON1-CCZ1 complex	1.02	0.57
Exoc6b	A6H5Z3	Exocyst complex component 6B	1.02	0.36
Ppme1	Q8BVQ5	Protein phosphatase methylesterase 1	1.02	0.43
Hnrnph1	Q8C2Q7	Heterogeneous nuclear ribonucleoprotein H	1.02	0.49
Slc9a1	Q61165	Sodium/hydrogen exchanger 1	1.02	0.69
Vma21	Q78T54	Vacuolar ATPase assembly integral membrane protein Vma21	1.02	0.72
Gpn1	Q8VCE2	GPN-loop GTPase 1	1.02	0.50
Synj2	F8WHD8	Synaptojanin-2	1.02	0.46
Cyp27a1	Q9DBG1	Sterol 26-hydroxylase, mitochondrial	1.02	0.80
Kyat3	Q71RI9	Kynurenine--oxoglutarate transaminase 3	1.02	0.51
C3ar1	O09047	C3a anaphylatoxin chemotactic receptor	1.02	0.21
Crocc	Q8CJ40	Rootletin	1.02	0.61
Slc12a7	Q9WVL3	Solute carrier family 12 member 7	1.02	0.55
Mrto4	Q9D0I8	mRNA turnover protein 4 homolog	1.02	0.54
Wdr4	E9Q156	tRNA (guanine-N(7)-)-methyltransferase non-catalytic subunit WDR4	1.02	0.65
Prpf8	Q99PV0	Pre-mRNA-processing-splicing factor 8	1.02	0.21
Rock2	A0A1Y7VMN0	Rho-associated protein kinase 2 (Fragment)	1.02	0.27
Card19	Q9D1I2	Caspase recruitment domain-containing protein 19	1.02	0.60
Reep4	A0A2I3BQJ3	Receptor expression-enhancing protein	1.02	0.50
Fam177a1	Q8BR63	Protein FAM177A1	1.02	0.35
Gapvd1	Q6PAR5	GTPase-activating protein and VPS9 domain-containing protein 1	1.02	0.41
Nucb1	Q02819	Nucleobindin-1	1.02	0.32
Ugt1a7c	Q6ZQM8	UDP-glucuronosyltransferase 1-7C	1.02	0.22
Riox1	Q9JJF3	Ribosomal oxygenase 1	1.02	0.70
Plekhm1	Q7TSI1	Pleckstrin homology domain-containing family M member 1	1.02	0.53
Mtrfr	Q80VP5	Mitochondrial translation release factor in rescue	1.02	0.37
Vps53	Q8CCB4	Vacuolar protein sorting-associated protein 53 homolog	1.02	0.41
Gosr2	O35166	Golgi SNAP receptor complex member 2	1.02	0.64
Dirc2	Q8BFQ6	Disrupted in renal carcinoma protein 2 homolog	1.02	0.68
Cox7a2	P48771	Cytochrome c oxidase subunit 7A2, mitochondrial	1.02	0.31
Acot2	Q9QYR9	Acyl-coenzyme A thioesterase 2, mitochondrial	1.02	0.21
Kctd10	F8WGQ9	BTB/POZ domain-containing adapter for CUL3-mediated RhoA degradation protein 3	1.02	0.53
Dcaf8	Q8N7N5	DDB1- and CUL4-associated factor 8	1.02	0.33
Gins1	Q9CZ15	DNA replication complex GINS protein PSF1	1.02	0.66
Rgl2	Q61193	Ral guanine nucleotide dissociation stimulator-like 2	1.02	0.71
Nedd4	P46935	E3 ubiquitin-protein ligase NEDD4	1.02	0.82
Fhod1	Q6P9Q4	FH1/FH2 domain-containing protein 1	1.02	0.38
Ppp1r37	Q8BKR5	Protein phosphatase 1 regulatory subunit 37	1.02	0.68
Map3k5	O35099	Mitogen-activated protein kinase kinase kinase 5	1.02	0.73
Tacc3	Q99LH8	Tacc3 protein	1.02	0.38
Bcl7b	Q921K9	B-cell CLL/lymphoma 7 protein family member B	1.02	0.68
Mtco1	P00397	Cytochrome c oxidase subunit 1	1.02	0.94
Cd22	Q3UP36	B-cell receptor CD22	1.02	0.56
Stat6	P52633	Signal transducer and transcription activator 6	1.02	0.43
Tsr1	Q5SWD9	Pre-rRNA-processing protein TSR1 homolog	1.02	0.62
Cmas	A0A0R4J0B4	Cytidine monophospho-N-acetylneuraminic acid synthetase	1.02	0.24
Ubxn4	A0A0R4J078	UBX domain containing 2, isoform CRA_a	1.02	0.29
Cdc42bpb	Q7TT50	Serine/threonine-protein kinase MRCK beta	1.02	0.46
Map1lc3b	M0QWC2	MCG14171, isoform CRA_b	1.02	0.50

Iqgap1	Q9JKF1	Ras GTPase-activating-like protein IQGAP1	1.02	0.29
Cnih1	D6RGU4	Protein cornichon homolog 1	1.02	0.82
Gsk3b	E9QAQ5	Glycogen synthase kinase-3 beta	1.02	0.37
Ahnak2	F7DBB3	AHNAK nucleoprotein 2 (Fragment)	1.02	0.31
Cdk12	Q14AX6	Cyclin-dependent kinase 12	1.02	0.11
Arhgap5	E9PYT0	Rho GTPase-activating protein 5	1.02	0.51
Ccdc124	Q9D8X2	Coiled-coil domain-containing protein 124	1.02	0.25
Pdcd2	A0A0R4J0N5	Programmed cell death 2, isoform CRA_b	1.02	0.47
Wac	Q924H7	WW domain-containing adapter protein with coiled-coil	1.02	0.70
Parp1	Q921K2	Poly [ADP-ribose] polymerase	1.02	0.38
Mapkapk2	P49138	MAP kinase-activated protein kinase 2	1.02	0.32
Tnfrsf23	Q9ER63	Tumor necrosis factor receptor superfamily member 23	1.02	0.33
Dnajc19	Q9CQV7	Mitochondrial import inner membrane translocase subunit TIM14	1.02	0.46
Macf1	E9PVY8	Microtubule-actin cross-linking factor 1	1.02	0.29
Wdr81	K4DI77	WD repeat-containing protein 81 (Fragment)	1.02	0.38
Dlgap5	A0A0R4J0G7	Disks large-associated protein 5	1.02	0.74
Hk1	P17710	Hexokinase-1	1.02	0.31
Rabggta	Q9JHK4	Geranylgeranyl transferase type-2 subunit alpha	1.02	0.38
Nagpa	Q8BJ48	N-acetylglucosamine-1-phosphodiester alpha-N-acetylglucosaminidase	1.02	0.49
Slc49a3	Q8CE47	Solute carrier family 49 member A3	1.02	0.47
Map4k5	E9PX30	Mitogen-activated protein kinase kinase kinase kinase	1.02	0.82
Lyn	P25911	Tyrosine-protein kinase Lyn	1.02	0.35
Dpy19l4	A2AJQ3	Probable C-mannosyltransferase DPY19L4	1.02	0.83
Nop53	Q8BK35	Ribosome biogenesis protein NOP53	1.02	0.66
Rabep1	O35551	Rab GTPase-binding effector protein 1	1.02	0.28
Iscu	Q9D7P6	Iron-sulfur cluster assembly enzyme ISCU, mitochondrial	1.02	0.32
Rpn2	Q9DBG6	Dolichyl-diphosphooligosaccharide--protein glycosyltransferase subunit 2	1.02	0.43
Nsmce3	Q9CPR8	Non-structural maintenance of chromosomes element 3 homolog	1.02	0.69
Rpl22l1	Q9D7S7	60S ribosomal protein L22-like 1	1.02	0.33
Fez2	Q6TYB5	Fasciculation and elongation protein zeta-2	1.02	0.51
Igf2r	Q07113	Cation-independent mannose-6-phosphate receptor	1.02	0.11
C1qa	P98086	Complement C1q subcomponent subunit A	1.02	0.43
Cybc1	Q3TYS2	Cytochrome b-245 chaperone 1	1.02	0.30
Adcy7	P51829	Adenylate cyclase type 7	1.02	0.41
Exoc8	Q6PGF7	Exocyst complex component 8	1.02	0.32
Ccl9	P51670	C-C motif chemokine 9	1.02	0.59
Sap30	O88574	Histone deacetylase complex subunit SAP30	1.02	0.61
Idh3b	Q91VA7	Isocitrate dehydrogenase [NAD] subunit, mitochondrial	1.02	0.58
Vdac3	Q60931	Voltage-dependent anion-selective channel protein 3	1.02	0.47
Fyco1	Q8VDC1	FYVE and coiled-coil domain-containing protein 1	1.02	0.59
Cd36	Q08857	Platelet glycoprotein 4	1.02	0.30
Chd5	E9PYL1	Chromodomain-helicase-DNA-binding protein 5	1.02	0.53
Abcb1b	P06795	Multidrug resistance protein 1B	1.02	0.54
Sap130	J3QNK5	Histone deacetylase complex subunit SAP130	1.02	0.75
Gpatch8	A2A6A1	G patch domain-containing protein 8	1.02	0.23
Sfr1	Q8BP27	Swi5-dependent recombination DNA repair protein 1 homolog	1.02	0.55
Mcrip1	Q3UGS4	Mapk-regulated corepressor-interacting protein 1	1.02	0.37
Vac14	Q80WQ2	Protein VAC14 homolog	1.02	0.23
Ube3b	Q9ES34	Ubiquitin-protein ligase E3B	1.02	0.39
Hmgb3	O54879	High mobility group protein B3	1.02	0.58
Zfpl1	Q9DB43	Zinc finger protein-like 1	1.02	0.50
Rrp1	P56183	Ribosomal RNA processing protein 1 homolog A	1.02	0.29
Itgb5	Q6PE70	Integrin beta	1.02	0.22
Csnk1g2	Q99K78	Casein kinase 1, gamma 2	1.02	0.19
Zc3h15	Q3TIV5	Zinc finger CCCH domain-containing protein 15	1.02	0.20
Smyd3	Q9CWR2	Histone-lysine N-methyltransferase SMYD3	1.02	0.37
Arl6	O88848	ADP-ribosylation factor-like protein 6	1.02	0.67
Rab22a	P35285	Ras-related protein Rab-22A	1.02	0.38
Ankrd28	A0A2I3BQ07	Serine/threonine-protein phosphatase 6 regulatory ankyrin repeat subunit A	1.02	0.64
Dipk2a	Q3USZ8	Divergent protein kinase domain 2A	1.02	0.57
Tfb1m	Q8JZM0	Dimethyladenosine transferase 1, mitochondrial	1.02	0.68
Immt	A0A0U1RP81	MICOS complex subunit MIC60	1.02	0.54
Trp53rkb	Q543M9	MCG14605, isoform CRA_c	1.02	0.38
Leng8	D3YWS8	Leukocyte receptor cluster (LRC) member 8, isoform CRA_a	1.02	0.54
Atp1a1	Q8VDN2	Sodium/potassium-transporting ATPase subunit alpha-1	1.02	0.42
Arhgap30	E9QMX7	Rho GTPase-activating protein 30	1.02	0.20
Dnm1l	E9PUD2	Dynamin-1-like protein	1.02	0.33
Utp6	Q8VCY6	U3 small nucleolar RNA-associated protein 6 homolog	1.02	0.47
Dse	Q8BLI4	Dermatan-sulfate epimerase	1.02	0.57
Cux1	P53564	Homeobox protein cut-like 1	1.02	0.56
Sqstm1	Q64337	Sequestosome-1	1.02	0.29
Rhob	P62746	Rho-related GTP-binding protein RhoB	1.02	0.33
Ncbp1	Q3UYV9	Nuclear cap-binding protein subunit 1	1.02	0.10

Sdad1	A0A0R4J0B7	Protein SDA1 homolog	1.02	0.63
Snu13	Q9D0T1	NHP2-like protein 1	1.02	0.71
Dennd4c	A6H8H2	DENN domain-containing protein 4C	1.02	0.30
Paxbp1	P58501	PAX3- and PAX7-binding protein 1	1.02	0.73
Ccdc88b	Q4QRL3	Coiled-coil domain-containing protein 88B	1.02	0.47
Btbd1	P58544	BTB/POZ domain-containing protein 1	1.02	0.47
Lrch4	H3BLL3	Leucine-rich repeat and calponin homology domain-containing protein 4	1.02	0.48
Zfand1	Q8BFR6	AN1-type zinc finger protein 1	1.02	0.35
R3hcc1l	Q8BJM3	Coiled-coil domain-containing protein R3HCC1L	1.02	0.53
Setd2	E9Q5F9	Histone-lysine N-methyltransferase SETD2	1.02	0.54
Dhx29	Q6PGC1	ATP-dependent RNA helicase DHX29	1.02	0.35
Kiaa0391	Q8JZY4	Mitochondrial ribonuclease P catalytic subunit	1.02	0.44
Pabpc4	Q6PHQ9	Polyadenylate-binding protein	1.02	0.29
Mcts1	Q9DB27	Malignant T-cell-amplified sequence 1	1.02	0.17
Cep97	Q9CZ62	Centrosomal protein of 97 kDa	1.02	0.66
Fndc3b	A0A0R4J0H8	Fibronectin type III domain-containing protein 3B	1.02	0.51
Cetn3	O35648	Centrin-3	1.02	0.68
Kif2a	E0CZ72	Kinesin-like protein	1.02	0.57
Snx14	G3UX33	Sorting nexin-14	1.02	0.57
Cggbp1	Q8BHG9	CGG triplet repeat-binding protein 1	1.02	0.59
Fastkd2	Q922E6	FAST kinase domain-containing protein 2, mitochondrial	1.02	0.71
Helb	Q6NVF4	DNA helicase B	1.02	0.52
Srpk1	O70551	SRSF protein kinase 1	1.02	0.48
Tamm41	G5E881	Phosphatidate cytidylyltransferase, mitochondrial	1.02	0.42
Lrrc41	Q8K1C9	Leucine-rich repeat-containing protein 41	1.02	0.34
Tmed2	Q9R0Q3	Transmembrane emp24 domain-containing protein 2	1.02	0.41
Lztfl1	Q9JHQ5	Leucine zipper transcription factor-like protein 1	1.02	0.37
Stag2	A2AFF6	Cohesin subunit SA-2	1.02	0.41
Tax1bp1	Q3UKC1	Tax1-binding protein 1 homolog	1.02	0.43
Mon2	B9EKJ3	Mon2 protein	1.02	0.32
Myo1g	Q5SUA5	Unconventional myosin-Ig	1.02	0.26
Arhgef7	Q9ES28	Rho guanine nucleotide exchange factor 7	1.03	0.23
Idnk	Q8R0J8	Probable gluconokinase	1.03	0.58
Ppp6r1	Q7TSI3	Serine/threonine-protein phosphatase 6 regulatory subunit 1	1.03	0.23
Slc25a15	Q9WVD5	Mitochondrial ornithine transporter 1	1.03	0.56
Tmed4	Q8R1V4	Transmembrane emp24 domain-containing protein 4	1.03	0.54
Eif2b4	D3Z780	Translation initiation factor eIF-2B subunit delta	1.03	0.14
Tbl1xr1	Q8BHJ5	F-box-like/WD repeat-containing protein TBL1XR1	1.03	0.36
Eef1a2	P62631	Elongation factor 1-alpha 2	1.03	0.69
Abcc1	O35379	Multidrug resistance-associated protein 1	1.03	0.43
Nqo1	Q64669	NAD(P)H dehydrogenase [quinone] 1	1.03	0.54
Por	P37040	NADPH--cytochrome P450 reductase	1.03	0.29
Mrpl45	Q9D0Q7	39S ribosomal protein L45, mitochondrial	1.03	0.30
Magohb	A0A023T778	Mago nashi protein	1.03	0.48
Efr3b	Q6ZQ18	Protein EFR3 homolog B	1.03	0.59
M6pr	P24668	Cation-dependent mannose-6-phosphate receptor	1.03	0.43
Nup58	Q8R332	Nucleoporin p58/p45	1.03	0.47
Strip1	Q8C079	Striatin-interacting protein 1	1.03	0.45
Fam107b	A0A2I3BR29	Protein FAM107B	1.03	0.18
Pum1	E9Q6M7	Pumilio homolog 1	1.03	0.52
Aasdhppt	Q9CQF6	L-aminoadipate-semialdehyde dehydrogenase-phosphopantetheinyl transferase	1.03	0.67
Eef1akmt1	A0A0D2X7Z2	EEF1A lysine methyltransferase 1	1.03	0.60
Dnajc3	Q91YW3	DnaJ homolog subfamily C member 3	1.03	0.19
Ddx20	Q9JJY4	Probable ATP-dependent RNA helicase DDX20	1.03	0.51
Phkb	Q7TSH2	Phosphorylase b kinase regulatory subunit beta	1.03	0.43
Mcub	Q810S1	Calcium uniporter regulatory subunit MCUb, mitochondrial	1.03	0.44
Tomm20	Q9DCC8	Mitochondrial import receptor subunit TOM20 homolog	1.03	0.30
Psmf1	Q8BHL8	Proteasome inhibitor PI31 subunit	1.03	0.49
Itga5	P11688	Integrin alpha-5	1.03	0.31
Rrm2	P11157	Ribonucleoside-diphosphate reductase subunit M2	1.03	0.52
Emc3	Q99KI3	ER membrane protein complex subunit 3	1.03	0.24
Rab8a	P55258	Ras-related protein Rab-8A	1.03	0.20
Cblb	Q3TTA7	E3 ubiquitin-protein ligase CBL-B	1.03	0.30
Fanci	Q8K368	Fanconi anemia group I protein homolog	1.03	0.70
	Q9D937	Uncharacterized protein C11orf98 homolog	1.03	0.51
Eml2	E9QK48	Echinoderm microtubule-associated protein-like 2	1.03	0.35
Ldah	A0A0F6AIX5	Lipid droplet-associated hydrolase	1.03	0.29
Ulk3	Q3U3Q1	Serine/threonine-protein kinase ULK3	1.03	0.67
Chuk	E9QNL4	Inhibitor of nuclear factor kappa-B kinase subunit alpha	1.03	0.24
Akr1e2	Q9DCT1	1,5-anhydro-D-fructose reductase	1.03	0.29
Cyb5b	Q9CQX2	Cytochrome b5 type B	1.03	0.56
Ggps1	Q9WTN0	Geranylgeranyl pyrophosphate synthase	1.03	0.35
P33monox	Q9DBN4	Putative monooxygenase p33MONOX	1.03	0.46

Ap1s2	Q8BW87	AP complex subunit sigma	1.03	0.23
Dnph1	Q80VJ3	2'-deoxynucleoside 5'-phosphate N-hydrolase 1	1.03	0.41
Tmem206	Q9D771	Transmembrane protein 206	1.03	0.69
Ap3m2	Q8R2R9	AP-3 complex subunit mu-2	1.03	0.44
Cwc22	Q8C5N3	Pre-mRNA-splicing factor CWC22 homolog	1.03	0.49
Cutc	Q9D8X1	Copper homeostasis protein cutC homolog	1.03	0.38
Znf24	Q91VN1	Zinc finger protein 24	1.03	0.65
Aifm2	Q8BUE4	Apoptosis-inducing factor 2	1.03	0.44
Bola1	Q9D8S9	BolA-like protein 1	1.03	0.58
Irgm1	Q60766	Immunity-related GTPase family M protein 1	1.03	0.37
Ndufb1	P0DN34	NADH dehydrogenase [ubiquinone] 1 beta subcomplex subunit 1	1.03	0.41
Alcam	Q61490	CD166 antigen	1.03	0.24
Bub1b	Q9Z1S0	Mitotic checkpoint serine/threonine-protein kinase BUB1 beta	1.03	0.57
Trim3	Q9R1R2	Tripartite motif-containing protein 3	1.03	0.37
Fbxl20	Q9CZV8	F-box/LRR-repeat protein 20	1.03	0.52
Nt5c3b	Q3UFY7	7-methylguanosine phosphate-specific 5'-nucleotidase	1.03	0.53
Aifm1	Q9Z0X1	Apoptosis-inducing factor 1, mitochondrial	1.03	0.33
Erbin	B7ZNX6	Erbb2ip protein	1.03	0.37
Myo5a	Q99104	Unconventional myosin-Va	1.03	0.33
Anpep	P97449	Aminopeptidase N	1.03	0.41
Luc7l3	Q5SUF2	Luc7-like protein 3	1.03	0.25
Nup188	Q6ZQH8	Nucleoporin NUP188 homolog	1.03	0.30
Clpb	Q8CCN6	Caseinolytic peptidase B protein homolog	1.03	0.46
Mark2	E9QMP6	Non-specific serine/threonine protein kinase	1.03	0.25
Gcn1	E9PVA8	eIF-2-alpha kinase activator GCN1	1.03	0.41
Cdca8	Q8BHX3	Borealin	1.03	0.63
Trir	Q9D735	Telomerase RNA component interacting RNase	1.03	0.27
Mtmr10	Q7TPM9	Myotubularin-related protein 10	1.03	0.65
Bax	Q07813	Apoptosis regulator BAX	1.03	0.46
Mlxip	G5E8D8	MLX interacting protein, isoform CRA_a	1.03	0.70
Paox	Q8C0L6	Peroxisomal N(1)-acetyl-spermine/spermidine oxidase	1.03	0.26
Serinc3	Q9QZI9	Serine incorporator 3	1.03	0.65
Pigg	D3Z3Y1	Phosphatidylinositol glycan anchor biosynthesis, class G	1.03	0.40
Hnrnpr	Q8VHM5	Heterogeneous nuclear ribonucleoprotein R	1.03	0.25
P4ha1	Q60715	Prolyl 4-hydroxylase subunit alpha-1	1.03	0.21
Mrps26	Q80ZS3	28S ribosomal protein S26, mitochondrial	1.03	0.61
Sergef	Q80YD6	Secretion-regulating guanine nucleotide exchange factor	1.03	0.36
Eif4g3	A0A0N4SVL0	Eukaryotic translation initiation factor 4 gamma 3	1.03	0.42
Wdr41	Q3UDP0	WD repeat-containing protein 41	1.03	0.58
Crtc3	Q91X84	CREB-regulated transcription coactivator 3	1.03	0.67
B4galt1	P15535	Beta-1,4-galactosyltransferase 1	1.03	0.25
Vcl	Q64727	Vinculin	1.03	0.45
Rif1	Q6PR54	Telomere-associated protein RIF1	1.03	0.43
Timm9	Q9WV98	Mitochondrial import inner membrane translocase subunit Tim9	1.03	0.73
Pts	Q9R1Z7	6-pyruvoyl tetrahydrobiopterin synthase	1.03	0.21
Riok1	Q922Q2	Serine/threonine-protein kinase RIO1	1.03	0.21
Vapb	Q8BH80	Vesicle-associated membrane protein, associated protein B and C	1.03	0.43
Prpf38a	Q4FK66	Pre-mRNA-splicing factor 38A	1.03	0.43
Rmdn1	Q9DCV4	Regulator of microtubule dynamics protein 1	1.03	0.32
Nrros	Q8BMT4	Transforming growth factor beta activator LRRC33	1.03	0.64
Parp2	O88554	Poly [ADP-ribose] polymerase 2	1.03	0.45
Serpinb2	P12388	Plasminogen activator inhibitor 2, macrophage	1.03	0.46
Tor1aip1	Q921T2	Torsin-1A-interacting protein 1	1.03	0.33
	Q9CR55	UPF0547 protein C16orf87 homolog	1.03	0.65
Psen2	Q3U4P5	Presenilin	1.03	0.39
Ptpra	Q91V35	Receptor-type tyrosine-protein phosphatase	1.03	0.48
Dock6	A0A1L1SQR4	Dedicator of cytokinesis protein 6	1.03	0.19
Cd200r1	Q9ES57	Cell surface glycoprotein CD200 receptor 1	1.03	0.57
Homer1	Q9Z2Y3	Homer protein homolog 1	1.03	0.82
Zadh2	Q8BGC4	Prostaglandin reductase-3	1.03	0.30
Ptcd2	Q8R3K3	Pentatricopeptide repeat-containing protein 2, mitochondrial	1.03	0.61
Trappc12	Q8K2L8	Trafficking protein particle complex subunit 12	1.03	0.21
Ccdc90b	Q8C3X2	Coiled-coil domain-containing protein 90B, mitochondrial	1.03	0.40
Csf2rb2	P26954	Interleukin-3 receptor class 2 subunit beta	1.03	0.44
B3glct	Q8BHT6	Beta-1,3-glucosyltransferase	1.03	0.51
Polr3d	Q91WD1	DNA-directed RNA polymerase III subunit RPC4	1.03	0.47
Git2	Q9JLQ2	ARF GTPase-activating protein GIT2	1.03	0.62
Tm9sf3	Q9ET30	Transmembrane 9 superfamily member 3	1.03	0.51
Champ1	A0A140T8S5	Chromosome alignment-maintaining phosphoprotein 1	1.03	0.35
Coq7	Q3TYT1	5-demethoxyubiquinone hydroxylase, mitochondrial	1.03	0.63
Htatip2	Q3U816	Oxidoreductase HTATIP2	1.03	0.14
Arf1	P84078	ADP-ribosylation factor 1	1.03	0.25
Atp5mf	P56135	ATP synthase subunit f, mitochondrial	1.03	0.37

Rab3d	P35276	Ras-related protein Rab-3D	1.03	0.44
Xylt2	Q9EPL0	Xylosyltransferase 2	1.03	0.12
Myo9b	E9PZW8	Unconventional myosin-IXb	1.03	0.31
Mri1	Q9CQT1	Methylthioribose-1-phosphate isomerase	1.03	0.28
Fra10ac1	Q8BP78	Protein FRA10AC1 homolog	1.03	0.55
Fcgr4	A0A0B4J1G0	Low affinity immunoglobulin gamma Fc region receptor IV	1.03	0.51
Sin3a	Q60520	Paired amphipathic helix protein Sin3a	1.03	0.46
Ak4	Q9WUR9	Adenylate kinase 4, mitochondrial	1.03	0.61
Znf622	Q91VY9	Zinc finger protein 622	1.03	0.46
Arid3a	Q62431	AT-rich interactive domain-containing protein 3A	1.03	0.34
Tm6sf1	P58749	Transmembrane 6 superfamily member 1	1.03	0.42
H6pd	A2A7A7	GDH/6PGL endoplasmic bifunctional protein	1.03	0.38
Vezt	D3Z4E6	Vezatin	1.03	0.65
Fgd6	Q69ZL1	FYVE, RhoGEF and PH domain-containing protein 6	1.03	0.01
Mcu	Q3UMR5	Calcium uniporter protein, mitochondrial	1.03	0.31
Cdc42ep4	Q9JM96	Cdc42 effector protein 4	1.03	0.32
Pycard	Q9EPB4	Apoptosis-associated speck-like protein containing a CARD	1.03	0.25
Mrps5	Q99N87	28S ribosomal protein S5, mitochondrial	1.03	0.55
Naa80	Q9R123	N-alpha-acetyltransferase 80	1.03	0.65
Sp110	Q8BVK9	Sp110 nuclear body protein	1.03	0.32
Sdhaf4	Q8BTE0	Succinate dehydrogenase assembly factor 4, mitochondrial	1.03	0.24
Tbcel	Q8C5W3	Tubulin-specific chaperone cofactor E-like protein	1.03	0.51
Palld	Q9ET54	Palladin	1.03	0.45
Pecr	Q99MZ7	Peroxisomal trans-2-enoyl-CoA reductase	1.03	0.50
Cdkn1a	P39689	Cyclin-dependent kinase inhibitor 1	1.03	0.67
Klc2	Q91YS4	Kinesin light chain 2	1.03	0.12
Ccdc58	Q8R3Q6	Coiled-coil domain-containing protein 58	1.03	0.52
Spats2	Q8K1N4	Spermatogenesis-associated serine-rich protein 2	1.03	0.62
Ovca2	Q9D7E3	Esterase OVCA2	1.03	0.45
Stk16	O88697	Serine/threonine-protein kinase 16	1.03	0.50
Kdm5c	P41230	Lysine-specific demethylase 5C	1.03	0.35
Dnajc11	Q5U458	DnaJ homolog subfamily C member 11	1.03	0.40
Alyref	O08583	THO complex subunit 4	1.03	0.43
Atad2	G3X963	ATPase family AAA domain-containing protein 2	1.03	0.53
Ythdf2	Q91YT7	YTH domain-containing family protein 2	1.03	0.22
Cdk14	O35495	Cyclin-dependent kinase 14	1.03	0.29
Tbpl1	P62340	TATA box-binding protein-like protein 1	1.03	0.61
Rbm5	Q91YE7	RNA-binding protein 5	1.03	0.66
Agfg2	Q3U2K8	Arf-GAP domain and FG repeat-containing protein 2	1.03	0.63
Ndufb6	Q3UIU2	NADH dehydrogenase [ubiquinone] 1 beta subcomplex subunit 6	1.03	0.38
Optn	Q8K3K8	Optineurin	1.03	0.26
Cuedc2	Q9CXX9	CUE domain-containing protein 2	1.03	0.22
Bpnt1	Q9Z0S1	3'(2'),5'-bisphosphate nucleotidase 1	1.03	0.43
Patl1	Q3TC46	Protein PAT1 homolog 1	1.03	0.55
Nr3c1	E9PYV1	Glucocorticoid receptor	1.03	0.47
Exoc2	Q9D4H1	Exocyst complex component 2	1.03	0.30
Nol9	Q3TZX8	Polynucleotide 5'-hydroxyl-kinase NOL9	1.03	0.22
Pigt	Q8BXQ2	GPI transamidase component PIG-T	1.03	0.30
Ireb2	Q811J3	Iron-responsive element-binding protein 2	1.03	0.39
Cst3	P21460	Cystatin-C	1.03	0.23
Acad8	A0A0R4J0P1	Acyl-Coenzyme A dehydrogenase family, member 8	1.03	0.46
Rnf141	Q99MB7	RING finger protein 141	1.03	0.56
Tmem87a	A2AQJ6	Transmembrane protein 87A	1.03	0.28
Slc15a4	Q91W98	Solute carrier family 15 member 4	1.03	0.63
Egln1	Q91YE3	Egl nine homolog 1	1.03	0.28
C9orf72	Q6DFW0	Guanine nucleotide exchange C9orf72 homolog	1.03	0.02
Pigu	Q3TAA8	Phosphatidylinositol glycan anchor biosynthesis class U protein	1.03	0.29
Nf2	P46662	Merlin	1.03	0.51
Psenen	Q9CQR7	Gamma-secretase subunit PEN-2	1.03	0.55
Slc11a2	P49282	Natural resistance-associated macrophage protein 2	1.03	0.62
Hccs	P53702	Cytochrome c-type heme lyase	1.03	0.13
Usp8	A2AI52	Ubiquitin carboxyl-terminal hydrolase 8	1.03	0.26
Tmem63a	Q91YT8	CSC1-like protein 1	1.03	0.33
Zfp91	Q62511	E3 ubiquitin-protein ligase ZFP91	1.03	0.59
Galnt7	Q80VA0	N-acetylgalactosaminyltransferase 7	1.03	0.35
Rap2a	Q80ZJ1	Ras-related protein Rap-2a	1.03	0.28
Abca1	P41233	ATP-binding cassette sub-family A member 1	1.03	0.31
Med8	Q9D7W5	Mediator of RNA polymerase II transcription subunit 8	1.03	0.52
H2-Aa	P14434	H-2 class II histocompatibility antigen, A-B alpha chain	1.03	0.76
Nop58	Q6DFW4	Nucleolar protein 58	1.03	0.47
Cep131	J3QMP9	Centrosomal protein of 131 kDa	1.03	0.55
Lig3	K4DI59	DNA ligase	1.03	0.78
Xpot	Q9CRT8	Exportin-T	1.03	0.36

Rchy1	Q9CR50	RING finger and CHY zinc finger domain-containing protein 1	1.03	0.30
Myof	Q69ZN7	Myoferlin	1.03	0.31
Flnb	Q80X90	Filamin-B	1.03	0.20
Daglb	Q91WC9	Sn1-specific diacylglycerol lipase beta	1.03	0.55
Mad1l1	Q9WTX8	Mitotic spindle assembly checkpoint protein MAD1	1.03	0.45
Cpsf4	B2LVG5	Cleavage and polyadenylation specific factor 4 isoform 1	1.03	0.36
Acsl5	Q8JZR0	Long-chain-fatty-acid--CoA ligase 5	1.03	0.24
Rps20	P60867	40S ribosomal protein S20	1.03	0.16
Tex10	Q3URQ0	Testis-expressed protein 10	1.03	0.37
Lnpep	Q8C129	Leucyl-cystinyl aminopeptidase	1.03	0.30
Etnk1	A0A0N4SVC4	Ethanolamine kinase 1	1.03	0.39
Mtmr1	I7HJQ9	Myotubularin-related protein 1	1.03	0.50
Rbms2	Q8VC70	RNA-binding motif, single-stranded-interacting protein 2	1.03	0.64
Sugp1	Q8CH02	SURP and G-patch domain-containing protein 1	1.03	0.41
Adap2	Q8R2V5	Arf-GAP with dual PH domain-containing protein 2	1.03	0.34
Foxo3	Q9WVH4	Forkhead box protein O3	1.03	0.38
Clec10a	J3QPR7	C-type lectin domain family 10 member A	1.03	0.60
Lipt1	Q8VCM4	Lipoyltransferase 1, mitochondrial	1.03	0.81
Stn1	Q8K2X3	CST complex subunit STN1	1.03	0.35
Rtn3	Q9ES97	Reticulon-3	1.03	0.50
Arrb1	F7DF62	Beta-arrestin-1 (Fragment)	1.03	0.79
Ngly1	Q9JI78	Peptide-N(4)-(N-acetyl-beta-glucosaminyl)asparagine amidase	1.03	0.62
Sigmar1	O55242	Sigma non-opioid intracellular receptor 1	1.03	0.27
Gtf2e2	Q9D902	General transcription factor IIE subunit 2	1.03	0.19
Ppcdc	Q8BZB2	Phosphopantothenoylcysteine decarboxylase	1.03	0.57
Psap	Q8BFQ1	Prosaposin	1.03	0.57
Tep1	P97499	Telomerase protein component 1	1.03	0.59
Fam20c	Q5MJS3	Extracellular serine/threonine protein kinase FAM20C	1.03	0.44
Parl	Q5XJY4	Presenilins-associated rhomboid-like protein, mitochondrial	1.03	0.27
Cc2d1a	Q8K1A6	Coiled-coil and C2 domain-containing protein 1A	1.03	0.53
Prrc2b	F8WHT3	Protein PRRC2B	1.03	0.37
Rex1bd	Q9CYZ6	Required for excision 1-B domain-containing protein	1.03	0.69
Eml3	Q8VC03	Echinoderm microtubule-associated protein-like 3	1.03	0.38
Abcc4	E9Q236	ATP-binding cassette, sub-family C (CFTR/MRP), member 4	1.03	0.72
Eif4e3	Q9DBB5	Eukaryotic translation initiation factor 4E type 3	1.03	0.61
BC017158	A0A0R4J0B5	cDNA sequence BC017158	1.03	0.44
Exosc8	Q9D753	Exosome complex component RRP43	1.03	0.36
Tmsb4x	P20065	Thymosin beta-4	1.03	0.70
Polr2a	P08775	DNA-directed RNA polymerase II subunit RPB1	1.03	0.31
Scrn3	Q3TMH2	Secernin-3	1.03	0.39
Yeats4	Q9CR11	YEATS domain-containing protein 4	1.03	0.73
Endod1	Q8C522	Endonuclease domain-containing 1 protein	1.03	0.43
Slc25a3	Q8VEM8	Phosphate carrier protein, mitochondrial	1.03	0.41
Ppp1r13l	Q5I1X5	RelA-associated inhibitor	1.03	0.60
Slc29a3	Q99P65	Equilibrative nucleoside transporter 3	1.03	0.55
Nsun5	Q8K4F6	Probable 28S rRNA (cytosine-C(5))-methyltransferase	1.03	0.58
Dars2	Q8BIP0	Aspartate--tRNA ligase, mitochondrial	1.03	0.18
Stard3nl	A0A1Y7VJA7	STARD3 N-terminal-like protein	1.03	0.53
Nhp2	Q9CRB2	H/ACA ribonucleoprotein complex subunit 2	1.03	0.12
Ddost	O54734	Dolichyl-diphosphooligosaccharide--protein glycosyltransferase 48 kDa subunit	1.03	0.28
Arpin	Q9D0A3	Arpin	1.03	0.48
Oga	Q9EQQ9	Protein O-GlcNAcase	1.03	0.24
Cd38	P56528	ADP-ribosyl cyclase/cyclic ADP-ribose hydrolase 1	1.03	0.64
Mgst1	E9QJW0	Microsomal glutathione S-transferase 1	1.03	0.41
Pop5	Q9DB28	Ribonuclease P/MRP protein subunit POP5	1.03	0.52
Slc41a3	G3X937	Solute carrier family 41 member 3	1.03	0.76
Tm9sf4	Q8BH24	Transmembrane 9 superfamily member 4	1.03	0.36
Oraov1	Q3TF33	Oral cancer overexpressed 1, isoform CRA_a	1.03	0.54
Rbm12b2	Q66JV4	RNA-binding protein 12B-B	1.03	0.83
Zfand6	Q9DCH6	AN1-type zinc finger protein 6	1.03	0.40
Atf1	P81269	Cyclic AMP-dependent transcription factor ATF-1	1.03	0.61
Dlgap4	B1AZP2	Disks large-associated protein 4	1.03	0.17
Dnajc5	P60904	DnaJ homolog subfamily C member 5	1.03	0.54
Pop7	Q9DCH2	Ribonuclease P protein subunit p20	1.03	0.57
Hip1	A0A0J9YUX9	Huntingtin-interacting protein 1 (Fragment)	1.03	0.15
Rprd1b	Q9CSU0	Regulation of nuclear pre-mRNA domain-containing protein 1B	1.03	0.48
C5ar1	P30993	C5a anaphylatoxin chemotactic receptor 1	1.03	0.41
Entpd5	Q3TQC7	Ectonucleoside triphosphate diphosphohydrolase 5	1.03	0.56
Letm1	Q9Z2I0	Mitochondrial proton/calcium exchanger protein	1.03	0.39
Cyp4v2	Q9DBW0	Cytochrome P450 4V2	1.03	0.42
Mccc1	Q99MR8	Methylcrotonoyl-CoA carboxylase subunit alpha, mitochondrial	1.03	0.30
Golim4	Q8BXA1	Golgi integral membrane protein 4	1.03	0.39
Afg3l1	Q920A7	AFG3-like protein 1	1.03	0.51

Mtg1	Q8R2R6	Mitochondrial ribosome-associated GTPase 1	1.03	0.84
Rock1	P70335	Rho-associated protein kinase 1	1.03	0.27
Zmpste24	Q80W54	CAAX prenyl protease 1 homolog	1.03	0.46
Mien1	Q9CQ86	Migration and invasion enhancer 1	1.03	0.17
Ptpn2	Q06180	Tyrosine-protein phosphatase non-receptor type 2	1.03	0.47
Ap5z1	Q3U829	AP-5 complex subunit zeta-1	1.03	0.24
Pycr2	Q922Q4	Pyrroline-5-carboxylate reductase 2	1.03	0.35
Rac1	P63001	Ras-related C3 botulinum toxin substrate 1	1.03	0.30
Cpd	O89001	Carboxypeptidase D	1.03	0.28
Mapk8ip3	K3W4S4	C-Jun-amino-terminal kinase-interacting protein 3	1.03	0.68
Rpap3	Q9D706	RNA polymerase II-associated protein 3	1.03	0.46
Cnnm3	Q32NY4	Metal transporter CNNM3	1.03	0.61
Pitpnm1	O35954	Membrane-associated phosphatidylinositol transfer protein 1	1.03	0.44
Shpk	Q9D5J6	Sedoheptulokinase	1.03	0.52
Gpr107	Q8BUV8	Protein GPR107	1.03	0.28
Camk2g	A0A286YDL6	Calcium/calmodulin-dependent protein kinase type II subunit gamma (Fragment)	1.03	0.40
Cacfd1	Q8BG21	Calcium channel flower homolog	1.03	0.57
Dusp23	Q6NT99	Dual specificity protein phosphatase 23	1.03	0.57
Crip1	A0A0G2JEK2	Cysteine-rich protein 1	1.03	0.79
Lrrc8d	Q8BGR2	Volume-regulated anion channel subunit LRRC8D	1.03	0.45
Milr1	Q3TB92	Allergin-1	1.03	0.31
Spns1	Q8R0G7	Protein spinster homolog 1	1.03	0.40
Ankhd1	F6RUI8	Ankyrin repeat and KH domain-containing 1 (Fragment)	1.03	0.74
Ilkap	Q8R0F6	Integrin-linked kinase-associated serine/threonine phosphatase 2C	1.03	0.42
Atp13a1	Q9EPE9	Manganese-transporting ATPase 13A1	1.03	0.35
Cmbl	Q8R1G2	Carboxymethylenebutenolidase homolog	1.03	0.29
Mrps15	Q9DC71	28S ribosomal protein S15, mitochondrial	1.03	0.49
Szt2	A2A9C3	KICSTOR complex protein SZT2	1.03	0.55
Ufc1	Q9CR09	Ubiquitin-fold modifier-conjugating enzyme 1	1.03	0.46
Hmox2	O70252	Heme oxygenase 2	1.03	0.30
Ear6	Q923L7	Ear6 protein	1.03	0.30
Sdhc	Q9CZB0	Succinate dehydrogenase cytochrome b560 subunit, mitochondrial	1.03	0.40
Arap3	Q8R5G7	Arf-GAP with Rho-GAP domain, ANK repeat and PH domain-containing protein 3	1.03	0.59
Slc25a13	Q9QXX4	Calcium-binding mitochondrial carrier protein Aralar2	1.03	0.43
Phf21a	A2AHG2	PHD finger protein 21A	1.03	0.64
Ralgapa2	A0A2I3BPN8	Ral GTPase-activating protein subunit alpha-2	1.03	0.36
Oxa1l	Q8BGA9	Mitochondrial inner membrane protein OXA1L	1.03	0.50
Arl8b	Q9CQW2	ADP-ribosylation factor-like protein 8B	1.03	0.38
Tbc1d10a	Q5SPX8	TBC1 domain family member 10A	1.03	0.05
Lars2	Q8VDC0	Probable leucine--tRNA ligase, mitochondrial	1.03	0.29
Rab7b	Q8VEA8	Ras-related protein Rab-7b	1.03	0.23
Sos2	Q02384	Son of sevenless homolog 2	1.03	0.23
Tpcn2	Q8BWC0	Two pore calcium channel protein 2	1.03	0.40
Rasa3	Q60790	Ras GTPase-activating protein 3	1.03	0.31
Zdhhc21	Q9D270	Probable palmitoyltransferase ZDHHC21	1.03	0.58
Mtpap	Q9D0D3	Poly(A) RNA polymerase, mitochondrial	1.03	0.35
Sltm	Q8CH25	SAFB-like transcription modulator	1.03	0.50
Mrps34	Q9JIK9	28S ribosomal protein S34, mitochondrial	1.03	0.57
Clip1	Q922J3	CAP-Gly domain-containing linker protein 1	1.03	0.11
Fn3krp	Q8K274	Ketosamine-3-kinase	1.03	0.33
Gpatch1	Q9DBM1	G patch domain-containing protein 1	1.03	0.60
Maip1	Q8BHE8	m-AAA protease-interacting protein 1, mitochondrial	1.03	0.37
Ciapin1	F8WIK0	Anamorsin	1.03	0.32
Mrps24	Q9CQV5	28S ribosomal protein S24, mitochondrial	1.03	0.70
Arid1a	E9QAQ7	AT-rich interactive domain-containing protein 1A	1.03	0.37
Scrib	Q80U72	Protein scribble homolog	1.03	0.59
Polr2j	O08740	DNA-directed RNA polymerase II subunit RPB11	1.03	0.46
Nceh1	Q8BLF1	Neutral cholesterol ester hydrolase 1	1.03	0.65
Ano6	A0A2I3BPX3	Anoctamin	1.03	0.26
Plch1	Q4KWH5	1-phosphatidylinositol 4,5-bisphosphate phosphodiesterase eta-1	1.03	0.42
Ctdsp1	P58466	Carboxy-terminal domain RNA polymerase II polypeptide A small phosphatase 1	1.03	0.52
Ndrg1	Q62433	Protein NDRG1	1.03	0.39
Zc3h11a	Q6NZF1	Zinc finger CCCH domain-containing protein 11A	1.03	0.47
Leprotl1	Q9CQ74	Leptin receptor overlapping transcript-like 1	1.03	0.62
Gins2	Q9D600	DNA replication complex GINS protein PSF2	1.03	0.70
Pir	Q9D711	Pirin	1.03	0.43
Nectin2	P32507	Nectin-2	1.03	0.71
Gpr108	Q91WD0	Protein GPR108	1.03	0.54
Ptafr	Q62035	Platelet-activating factor receptor	1.03	0.52
Pakap	F7AA26	Paralemmin A kinase anchor protein (Fragment)	1.03	0.60
Arhgap27	A2AB59	Rho GTPase-activating protein 27	1.03	0.59
Tsen15	G3X8S8	MCG14499	1.03	0.46
Zfp106	R4GML0	Zinc finger protein 106	1.03	0.33

Tbcd	Q8BYA0	Tubulin-specific chaperone D	1.03	0.23
Gatad2a	E9QMN5	Transcriptional repressor p66 alpha	1.03	0.54
Cdk6	Q64261	Cyclin-dependent kinase 6	1.03	0.20
Ceacam1	P31809	Carcinoembryonic antigen-related cell adhesion molecule 1	1.03	0.66
Golga5	Q9QYE6	Golgin subfamily A member 5	1.03	0.16
Rasal3	Q8C2K5	RAS protein activator like-3	1.03	0.06
Atp2b1	G5E829	Plasma membrane calcium-transporting ATPase 1	1.03	0.30
Ppp1r8	Q8R3G1	Nuclear inhibitor of protein phosphatase 1	1.03	0.63
Bloc1s5	Q8R015	Biogenesis of lysosome-related organelles complex 1 subunit 5	1.03	0.37
Itga4	Q00651	Integrin alpha-4	1.03	0.47
Ssr1	Q9CY50	Translocon-associated protein subunit alpha	1.03	0.45
Heatr3	Q8BQM4	HEAT repeat-containing protein 3	1.03	0.29
Slc25a5	P51881	ADP/ATP translocase 2	1.03	0.31
Sptlc1	O35704	Serine palmitoyltransferase 1	1.03	0.39
Ndufa8	Q9DCJ5	NADH dehydrogenase [ubiquinone] 1 alpha subcomplex subunit 8	1.03	0.23
Wdr33	Q8K4P0	pre-mRNA 3' end processing protein WDR33	1.03	0.40
Itih2	G3X977	Inter-alpha trypsin inhibitor, heavy chain 2	1.03	0.63
Aaas	P58742	Aladin	1.03	0.56
Nck2	O55033	Cytoplasmic protein NCK2	1.03	0.47
Arl5a	Q80ZU0	ADP-ribosylation factor-like protein 5A	1.03	0.41
Tpmt	A0A0R4J018	Thiopurine S-methyltransferase	1.03	0.46
Rasa1	E9PYG6	RAS p21 protein activator 1	1.03	0.27
Shc1	P98083	SHC-transforming protein 1	1.03	0.25
Atp1b3	P97370	Sodium/potassium-transporting ATPase subunit beta-3	1.03	0.54
Rab13	Q9DD03	Ras-related protein Rab-13	1.03	0.51
Retreg1	Q8VE91	Reticulophagy regulator 1	1.03	0.17
Cask	O70589	Peripheral plasma membrane protein CASK	1.03	0.56
Tomm22	Q9CPQ3	Mitochondrial import receptor subunit TOM22 homolog	1.03	0.28
Rab32	Q9CZE3	Ras-related protein Rab-32	1.03	0.40
Macf1	B1ARU1	Microtubule-actin cross-linking factor 1	1.03	0.09
Nubp2	Q9R061	Cytosolic Fe-S cluster assembly factor NUBP2	1.03	0.53
Nes	Q6P5H2	Nestin	1.03	0.49
	Q922M7	Ashwin	1.03	0.45
Dhx36	Q8VHK9	ATP-dependent DNA/RNA helicase DHX36	1.03	0.41
Sntb2	B7ZNU9	Beta-2-syntrophin	1.03	0.56
Mtmr9	Q9Z2D0	Myotubularin-related protein 9	1.03	0.40
Map2k2	Q63932	Dual specificity mitogen-activated protein kinase kinase 2	1.03	0.38
Tmx4	Q8C0L0	Thioredoxin-related transmembrane protein 4	1.03	0.28
Cyp4f16	Q99N17	Cytochrome P450 CYP4F16	1.03	0.31
Cyp20a1	Q8BKE6	Cytochrome P450 20A1	1.03	0.42
Atp5md	Q78IK2	Up-regulated during skeletal muscle growth protein 5	1.03	0.77
Stxbp5	Q8K400	Syntaxin-binding protein 5	1.03	0.40
Rab3ip	A0A1W2P7K6	RAB3A interacting protein, isoform CRA_b	1.03	0.36
Mta1	F8WHY8	Metastasis-associated protein MTA1	1.03	0.34
Smarca5	Q91ZW3	SWI/SNF-related matrix-associated actin-dependent regulator of chromatin subfamily A member 5	1.03	0.32
Hsd17b11	Q9EQ06	Estradiol 17-beta-dehydrogenase 11	1.03	0.34
Nsun4	Q9CZ57	5-methylcytosine rRNA methyltransferase NSUN4	1.03	0.63
Erlin1	A0A0R4J1G5	Erlin-1	1.03	0.27
Sestd1	Q80UK0	SEC14 domain and spectrin repeat-containing protein 1	1.03	0.34
Ccny	Q8BGU5	Cyclin-Y	1.03	0.58
Rpn1	Q91YQ5	Dolichyl-diphosphooligosaccharide--protein glycosyltransferase subunit 1	1.03	0.22
Rras2	P62071	Ras-related protein R-Ras2	1.03	0.45
Galnt10	Q6P9S7	Polypeptide N-acetylgalactosaminyltransferase 10	1.03	0.53
Trrap	A0A1D5RLL4	Transformation/transcription domain-associated protein	1.03	0.39
Mlec	Q6ZQI3	Malectin	1.03	0.45
Pik3cb	Q8BTI9	Phosphatidylinositol 4,5-bisphosphate 3-kinase catalytic subunit beta isoform	1.03	0.26
Ipo8	Q7TMY7	Importin-8	1.03	0.29
Nifk	Q91VE6	MKI67 FHA domain-interacting nucleolar phosphoprotein	1.03	0.09
Vamp3	P63024	Vesicle-associated membrane protein 3	1.03	0.39
Mthfd2l	D3YZG8	Probable bifunctional methylenetetrahydrofolate dehydrogenase/cyclohydrolase 2	1.03	0.78
Polr2l	P62876	DNA-directed RNA polymerases I, II, and III subunit RPABC5	1.03	0.67
Tbce	Q8CIV8	Tubulin-specific chaperone E	1.03	0.29
Mrpl1	Q99N96	39S ribosomal protein L1, mitochondrial	1.03	0.07
Lpcat1	Q3TFD2	Lysophosphatidylcholine acyltransferase 1	1.03	0.41
Ell2	Q3UKU1	RNA polymerase II elongation factor ELL2	1.03	0.74
Arhgap10	Q6Y5D8	Rho GTPase-activating protein 10	1.03	0.38
Pitpnb	Q8JZZ5	Phosphatidylinositol transfer protein beta isoform	1.03	0.41
Acaa2	Q8BWT1	3-ketoacyl-CoA thiolase, mitochondrial	1.03	0.40
Brpf1	A0A0N4SUT9	Peregrin	1.03	0.75
Mob3a	Q8BSU7	MOB kinase activator 3A	1.03	0.14
Nde1	Q9CZA6	Nuclear distribution protein nudE homolog 1	1.03	0.22
Tbc1d2	B1AVH7	TBC1 domain family member 2A	1.03	0.24
Vcpkmt	Q8C436	Protein-lysine methyltransferase METTL21D	1.03	0.42

Cox6c	Q9CPQ1	Cytochrome c oxidase subunit 6C	1.03	0.44
Cpne2	P59108	Copine-2	1.03	0.33
Trip12	G5E870	E3 ubiquitin-protein ligase TRIP12	1.03	0.22
Atp9b	D3YV00	Phospholipid-transporting ATPase	1.03	0.23
Casc3	Q8K3W3	Protein CASC3	1.03	0.73
	Q99M08	Uncharacterized protein C4orf3 homolog	1.03	0.70
Jak1	B1ASP2	Tyrosine-protein kinase	1.03	0.34
Wdr45	Q91VM3	WD repeat domain phosphoinositide-interacting protein 4	1.03	0.48
Trappc1	Q5NCF2	Trafficking protein particle complex subunit 1	1.03	0.57
Tbxas1	P36423	Thromboxane-A synthase	1.03	0.39
Rab35	Q6PHN9	Ras-related protein Rab-35	1.03	0.24
Chid1	A0A0R4J242	Chitinase domain-containing protein 1	1.03	0.25
Xpc	P51612	DNA repair protein complementing XP-C cells homolog	1.03	0.84
Pex1	Q5BL07	Peroxisome biogenesis factor 1	1.03	0.53
Slc4a7	F8VQC9	Anion exchange protein	1.03	0.41
Mthfd2	P18155	Bifunctional methylenetetrahydrofolate dehydrogenase/cyclohydrolase, mitochondrial	1.03	0.49
Clasp1	E9QKH0	CLIP-associating protein 1	1.03	0.64
Mocs2	F6ZGI7	Molybdopterin synthase sulfur carrier subunit	1.03	0.31
Smdt1	Q9DB10	Essential MCU regulator, mitochondrial	1.03	0.62
Hacd4	A2AKM2	Very-long-chain (3R)-3-hydroxyacyl-CoA dehydratase 4	1.03	0.40
Tec	P24604	Tyrosine-protein kinase Tec	1.03	0.35
Smpd2	O70572	Sphingomyelin phosphodiesterase 2	1.03	0.52
Atp6v0a1	K3W4T3	V-type proton ATPase subunit a	1.03	0.29
Brd1	E9PZ26	Bromodomain-containing 1	1.03	0.41
Gxylt1	D3Z6W7	Glucoside xylosyltransferase 1	1.03	0.41
Gcc1	Q9D4H2	GRIP and coiled-coil domain-containing protein 1	1.03	0.43
Prkx	Q922R0	cAMP-dependent protein kinase catalytic subunit PRKX	1.03	0.43
Adpgk	A0A1L1SSF2	ADP-dependent glucokinase	1.03	0.20
Selenof	A0A0R4J0K1	Selenoprotein F	1.03	0.38
Ncoa4	Q5U4H9	Nuclear receptor coactivator 4	1.03	0.55
Mtnd3	P03899	NADH-ubiquinone oxidoreductase chain 3	1.03	0.24
Med12	A2AGH6	Mediator of RNA polymerase II transcription subunit 12	1.03	0.53
Elf1	Q60775	ETS-related transcription factor Elf-1	1.03	0.29
Lman1	Q9D0F3	Protein ERGIC-53	1.03	0.20
Zdhhc17	Q80TN5	Palmitoyltransferase ZDHHC17	1.03	0.73
Nme4	Q9WV84	Nucleoside diphosphate kinase, mitochondrial	1.03	0.44
Gnl3	Q8CI11	Guanine nucleotide-binding protein-like 3	1.03	0.16
Ptges2	Q8BWM0	Prostaglandin E synthase 2	1.03	0.12
Timm17b	Q9Z0V7	Mitochondrial import inner membrane translocase subunit Tim17-B	1.03	0.47
Stt3a	P46978	Dolichyl-diphosphooligosaccharide--protein glycosyltransferase subunit STT3A	1.03	0.33
Kcnn4	O89109	Intermediate conductance calcium-activated potassium channel protein 4	1.03	0.36
Ssb	P32067	Lupus La protein homolog	1.03	0.31
Usf1	Q61069	Upstream stimulatory factor 1	1.03	0.62
Ttc12	Q8BW49	Tetratricopeptide repeat protein 12	1.03	0.45
Ttc13	A0A1L1SSC7	Tetratricopeptide repeat domain 13	1.03	0.68
Syne3	Q4FZC9	Nesprin-3	1.03	0.52
Ctcf	Q61164	Transcriptional repressor CTCF	1.03	0.17
Hirip3	Q8BLH7	HIRA-interacting protein 3	1.03	0.66
Fer	P70451	Tyrosine-protein kinase Fer	1.03	0.50
Coa5	Q99M07	Cytochrome c oxidase assembly factor 5	1.03	0.59
Ttc27	Q8CD92	Tetratricopeptide repeat protein 27	1.03	0.53
Fbxo6	Q9QZN4	F-box only protein 6	1.03	0.35
Cnpy4	Q8BQ47	Protein canopy homolog 4	1.03	0.24
Snx29	Q9D3S3	Sorting nexin-29	1.03	0.54
Adam10	O35598	Disintegrin and metalloproteinase domain-containing protein 10	1.03	0.37
Cmtm7	Q9ESD6	CKLF-like MARVEL transmembrane domain-containing protein 7	1.03	0.65
Naprt	Q8CC86	Nicotinate phosphoribosyltransferase	1.03	0.58
Tor4a	Q8BH02	Torsin-4A	1.03	0.12
Trem2	Q99NH8	Triggering receptor expressed on myeloid cells 2	1.03	0.29
Myh10	Q3UH59	Myosin-10	1.03	0.71
D17Wsu92e	B2KF52	Uncharacterized protein C6orf106 homolog	1.03	0.45
Mtch2	Q791V5	Mitochondrial carrier homolog 2	1.03	0.39
Pbxip1	Q3TVI8	Pre-B-cell leukemia transcription factor-interacting protein 1	1.03	0.54
Slc38a7	Q8BWH0	Putative sodium-coupled neutral amino acid transporter 7	1.03	0.56
Rnf185	Q91YT2	E3 ubiquitin-protein ligase RNF185	1.03	0.43
Xpo4	A0A0R4J254	Exportin-4	1.03	0.45
Mrpl41	Q9CQN7	39S ribosomal protein L41, mitochondrial	1.03	0.28
Ptprc	P06800	Receptor-type tyrosine-protein phosphatase C	1.03	0.26
Cd300a	Q6SJQ0	CMRF35-like molecule 8	1.03	0.52
Ndc80	Q9D0F1	Kinetochore protein NDC80 homolog	1.03	0.56
Pak4	Q8BTW9	Serine/threonine-protein kinase PAK 4	1.03	0.45
Adam15	O88839	Disintegrin and metalloproteinase domain-containing protein 15	1.03	0.36
Armc8	G3X920	Armadillo repeat containing 8, isoform CRA_b	1.03	0.19

Ppfia1	S4R1D4	Protein tyrosine phosphatase, receptor type, f polypeptide (PTPRF),-interacting protein (liprin), alpha 1 (Fragment)	1.03	0.62
Poglut1	Q8BYB9	Protein O-glucosyltransferase 1	1.03	0.42
Gigyf1	Q99MR1	GRB10-interacting GYF protein 1	1.03	0.36
Ykt6	Q9CQW1	Synaptobrevin homolog YKT6	1.03	0.21
Rbm26	Q6NZN0	RNA-binding protein 26	1.03	0.29
Mlycd	Q99J39	Malonyl-CoA decarboxylase, mitochondrial	1.03	0.48
Rcn2	Q8BP92	Reticulocalbin-2	1.03	0.20
Micu1	Q8VCX5	Calcium uptake protein 1, mitochondrial	1.03	0.32
Fam160b1	Q8CDM8	Protein FAM160B1	1.03	0.14
Taf5l	Q91WQ5	TAF5-like RNA polymerase II p300/CBP-associated factor-associated factor 65 kDa subunit 5L	1.03	0.46
Ubac2	Q8R1K1	Ubiquitin-associated domain-containing protein 2	1.03	0.47
Eef1e1	Q9D1M4	Eukaryotic translation elongation factor 1 epsilon-1	1.03	0.22
Smarca4	A0A0R4J170	Transcription activator BRG1	1.03	0.33
P2rx4	Q9Z257	P2X purinoceptor	1.03	0.37
Iqgap2	Q3UQ44	Ras GTPase-activating-like protein IQGAP2	1.03	0.15
Nelfb	A0A0X1KG62	Negative elongation factor B	1.03	0.60
Tbc1d10b	Q8BHL3	TBC1 domain family member 10B	1.03	0.24
Sfxn3	Q91V61	Sideroflexin-3	1.03	0.32
Cnnm2	Q3TWN3	Metal transporter CNNM2	1.03	0.52
Plekhm2	Z4YJW6	Pleckstrin homology domain-containing family M member 2	1.03	0.12
Yaf2	Q99LW6	YY1-associated factor 2	1.03	0.67
Folr2	Q05685	Folate receptor beta	1.03	0.20
Max	P28574	Protein max	1.03	0.56
Gltp	Q9JL62	Glycolipid transfer protein	1.03	0.35
Uqcr10	Q8R1I1	Cytochrome b-c1 complex subunit 9	1.03	0.53
Uhrf1bp1l	A2RSJ4	UHRF1-binding protein 1-like	1.03	0.29
Phf3	B2RQG2	PHD finger protein 3	1.03	0.57
Churc1	Q6DG52	Protein Churchill	1.03	0.68
Lactb	Q9EP89	Serine beta-lactamase-like protein LACTB, mitochondrial	1.03	0.34
Stx12	Q9ER00	Syntaxin-12	1.03	0.31
Mef2d	Q63943	Myocyte-specific enhancer factor 2D	1.03	0.60
Idua	Q8BMG0	Alpha-L-iduronidase	1.03	0.36
Reps2	B9EI38	RalBP1-associated Eps domain-containing protein 2	1.03	0.42
Tgfb1	P04202	Transforming growth factor beta-1 proprotein	1.03	0.48
Gnpat	P98192	Dihydroxyacetone phosphate acyltransferase	1.03	0.14
Exoc4	O35382	Exocyst complex component 4	1.03	0.12
Mfsd12	Q3U481	Major facilitator superfamily domain-containing protein 12	1.03	0.49
Fam3c	G5E911	DNA segment, Chr 6, Wayne State University 176, expressed, isoform CRA_f	1.03	0.45
Ankib1	Q6ZPS6	Ankyrin repeat and IBR domain-containing protein 1	1.03	0.28
Agpat5	Q9D1E8	1-acyl-sn-glycerol-3-phosphate acyltransferase epsilon	1.03	0.40
Iqcb1	Q8BP00	IQ calmodulin-binding motif-containing protein 1	1.03	0.73
Fam114a1	Q9D281	Protein Noxp20	1.03	0.57
Farp2	Q91VS8	FERM, ARHGEF and pleckstrin domain-containing protein 2	1.03	0.56
Tmem106a	Q8VC04	Transmembrane protein 106A	1.03	0.55
Igsf6	P0C6B7	Immunoglobulin superfamily member 6	1.03	0.26
Gon4l	Q9DB00	GON-4-like protein	1.03	0.53
Flvcr1	B2RXV4	Feline leukemia virus subgroup C receptor-related protein 1	1.03	0.38
Hdhd2	D6RI20	Haloacid dehalogenase-like hydrolase domain-containing protein 2	1.03	0.59
Tecpr2	Q3UH45	Tectonin beta-propeller repeat-containing 2	1.03	0.60
Wfs1	P56695	Wolframin	1.03	0.53
Prrc2c	S4R2J9	Protein PRRC2C	1.03	0.38
Soat1	Q61263	Sterol O-acyltransferase 1	1.03	0.26
Tbl2	Q9R099	Transducin beta-like protein 2	1.03	0.39
Pafah2	E9QNW6	Platelet-activating factor acetylhydrolase	1.03	0.47
Tbc1d4	Q8BYJ6	TBC1 domain family member 4	1.03	0.50
Xpnpep3	B7ZMP1	Xaa-Pro aminopeptidase 3	1.03	0.31
Surf4	Q64310	Surfeit locus protein 4	1.03	0.33
Pias4	Q9JM05	E3 SUMO-protein ligase PIAS4	1.03	0.52
Rftn1	Q6A0D4	Raftlin	1.03	0.12
Trim35	A0A0R4J031	Tripartite motif-containing 35	1.03	0.66
Ccr5	P51682	C-C chemokine receptor type 5	1.03	0.08
Plekhb2	Q9QZC7	Pleckstrin homology domain-containing family B member 2	1.03	0.66
Tyw3	Q8BSA9	tRNA wybutosine-synthesizing protein 3 homolog	1.03	0.76
Bloc1s3	Q5U5M8	Biogenesis of lysosome-related organelles complex 1 subunit 3	1.03	0.32
Cnn2	Q08093	Calponin-2	1.03	0.28
Scamp1	Q8K021	Secretory carrier-associated membrane protein 1	1.03	0.29
Chp1	P61022	Calcineurin B homologous protein 1	1.03	0.16
Abcb8	Q9CXJ4	ATP-binding cassette sub-family B member 8, mitochondrial	1.03	0.63
Bud13	Q8R149	BUD13 homolog	1.03	0.57
Pigk	Q8BL63	GPI-anchor transamidase	1.03	0.44
Agpat2	Q8K3K7	1-acyl-sn-glycerol-3-phosphate acyltransferase beta	1.03	0.22
Nup153	E9Q3G8	Nucleoporin 153	1.03	0.22
Map3k7	Q923A8	Mitogen-activated protein kinase kinase kinase 7	1.03	0.43

MINDY3	Q9CV28	Ubiquitin carboxyl-terminal hydrolase MINDY-3	1.03	0.34
Prpsap1	B1AT82	MCG6846, isoform CRA_c	1.03	0.31
Ptpn7	Q8BUM3	Tyrosine-protein phosphatase non-receptor type 7	1.03	0.53
Ube2e3	P52483	Ubiquitin-conjugating enzyme E2 E3	1.03	0.51
Mtarc2	Q922Q1	Mitochondrial amidoxime reducing component 2	1.03	0.33
Armcx3	Q8BHS6	Armadillo repeat-containing X-linked protein 3	1.03	0.37
Atp5pb	Q9CQQ7	ATP synthase F(0) complex subunit B1, mitochondrial	1.03	0.35
Rhot1	Q8BG51	Mitochondrial Rho GTPase 1	1.03	0.18
Gpat4	Q8K2C8	Glycerol-3-phosphate acyltransferase 4	1.03	0.68
Clec4a3	Q8JZX6	C-type lectin domain family 4, member a3	1.03	0.70
Flot2	Q60634	Flotillin-2	1.03	0.23
Atp1a3	A0A0G2JGX4	Sodium/potassium-transporting ATPase subunit alpha	1.03	0.31
Elmod2	Q8BGF6	ELMO domain-containing protein 2	1.03	0.12
Exog	E9PZS5	Nuclease EXOG, mitochondrial	1.03	0.25
Ndufaf4	Q9D1H6	NADH dehydrogenase [ubiquinone] 1 alpha subcomplex assembly factor 4	1.03	0.27
Hmga1	A0A338P6G6	High mobility group protein HMG-I/HMG-Y	1.03	0.25
Pofut1	Q91ZW2	GDP-fucose protein O-fucosyltransferase 1	1.03	0.41
Tor2a	Q8R1J9	Torsin-2A	1.03	0.72
Fes	P16879	Tyrosine-protein kinase Fes/Fps	1.03	0.15
Ncapd3	K4DI67	Condensin-2 complex subunit D3	1.03	0.35
Steap3	E9QN92	Metalloreductase STEAP3	1.03	0.30
Top2b	Q64511	DNA topoisomerase 2-beta	1.03	0.17
Crybg1	A0A0G2JG52	Crystallin beta-gamma domain-containing 1	1.03	0.44
Napepld	Q8BH82	N-acyl-phosphatidylethanolamine-hydrolyzing phospholipase D	1.03	0.67
Ergic3	Q9CQE7	Endoplasmic reticulum-Golgi intermediate compartment protein 3	1.03	0.40
Mrpl9	Q99N94	39S ribosomal protein L9, mitochondrial	1.03	0.48
Pcdh7	A0A0A6YY83	Protocadherin 7	1.03	0.51
Slc33a1	Q99J27	Acetyl-coenzyme A transporter 1	1.03	0.44
Dcun1d3	Q8K0V2	DCN1-like protein 3	1.03	0.40
Trappc6b	Q9D289	Trafficking protein particle complex subunit 6B	1.03	0.03
Ncstn	P57716	Nicastrin	1.03	0.06
Micall2	Q3TN34	MICAL-like protein 2	1.03	0.48
Mfge8	P21956	Lactadherin	1.03	0.35
Man1a2	P39098	Mannosyl-oligosaccharide 1,2-alpha-mannosidase IB	1.03	0.65
Tomm70	Q9CZW5	Mitochondrial import receptor subunit TOM70	1.03	0.28
Agpat4	Q8K4X7	1-acyl-sn-glycerol-3-phosphate acyltransferase delta	1.03	0.20
Fam160a2	A0A1C7CYU5	FTS and Hook-interacting protein	1.03	0.62
Esyt2	Q3TZZ7	Extended synaptotagmin-2	1.03	0.35
Las1l	A2BE28	Ribosomal biogenesis protein LAS1L	1.03	0.55
Hbs1l	Q69ZS7	HBS1-like protein	1.03	0.23
Mrrf	Q9D6S7	Ribosome-recycling factor, mitochondrial	1.03	0.58
Slc25a51	A2AKW0	Solute carrier family 25 member 51	1.03	0.19
Pde6d	O55057	Retinal rod rhodopsin-sensitive cGMP 3',5'-cyclic phosphodiesterase subunit delta	1.03	0.53
Sppl2a	Q9JJF9	Signal peptide peptidase-like 2A	1.03	0.35
Ccz1	Q8C1Y8	Vacuolar fusion protein CCZ1 homolog	1.03	0.37
Gm20521	D3Z5F7	Predicted gene 20521	1.03	0.06
Vamp7	P70280	Vesicle-associated membrane protein 7	1.03	0.48
Smim14	Q91VT8	Small integral membrane protein 14	1.03	0.13
Extl3	Q6P1H4	Exostoses (Multiple)-like 3	1.03	0.30
Il18	K3W4N2	Interleukin-18	1.03	0.81
Slc30a6	J3QMX8	Zinc transporter 6	1.03	0.53
Slc25a12	Q8BH59	Calcium-binding mitochondrial carrier protein Aralar1	1.03	0.13
Ddx50	Q99MJ9	ATP-dependent RNA helicase DDX50	1.03	0.38
Sort1	Q6PHU5	Sortilin	1.03	0.36
Uqcc1	Q9CWU6	Ubiquinol-cytochrome-c reductase complex assembly factor 1	1.03	0.31
Ndufaf1	A0A0R4J081	Complex I intermediate-associated protein 30, mitochondrial	1.03	0.42
Cux1	H3BJN3	Protein CASP	1.03	0.05
Ino80	Q6ZPV2	Chromatin-remodeling ATPase INO80	1.03	0.42
Spin1	Q61142	Spindlin-1	1.03	0.25
Hps1	Q3U309	Hermansky-Pudlak syndrome 1 protein homolog	1.03	0.51
Tpk1	Q9R0M5	Thiamin pyrophosphokinase 1	1.03	0.52
Caap1	Q8VDY9	Caspase activity and apoptosis inhibitor 1	1.03	0.57
Xaf1	Q5NBU8	XIAP-associated factor 1	1.03	0.29
Rpl37	Q9D823	60S ribosomal protein L37	1.03	0.57
Bzw2	Q91VK1	Basic leucine zipper and W2 domain-containing protein 2	1.03	0.13
Rab9	A2AFP5	RAB9, member RAS oncogene family (Fragment)	1.03	0.09
Fam192a	Q91WE2	PSME3-interacting protein	1.03	0.09
Colec12	Q8K4Q8	Collectin-12	1.03	0.26
Sh3pxd2b	A2AAY5	SH3 and PX domain-containing protein 2B	1.03	0.19
Trip4	Q9QXN3	Activating signal cointegrator 1	1.03	0.59
Elmsan1	E9Q2I4	ELM2 and Myb/SANT-like domain-containing 1	1.03	0.33
Rpe	B2KGF0	Ribulose-phosphate 3-epimerase	1.03	0.06
Apoe	P08226	Apolipoprotein E	1.03	0.39

Trim12c	D3Z3L3	Tripartite motif-containing 12C	1.03	0.19
Zfyve1	Q810J8	Zinc finger FYVE domain-containing protein 1	1.03	0.42
Atrx	Q61687	Transcriptional regulator ATRX	1.03	0.49
Rab1b	Q9D1G1	Ras-related protein Rab-1B	1.03	0.32
Brd2	Q7JJ13	Bromodomain-containing protein 2	1.03	0.17
Abhd12	Q99LR1	Monoacylglycerol lipase ABHD12	1.03	0.17
Fkbp9	Q9Z247	Peptidyl-prolyl cis-trans isomerase FKBP9	1.03	0.39
Rps3a	P97351	40S ribosomal protein S3a	1.03	0.22
Erlec1	Q8VEH8	Endoplasmic reticulum lectin 1	1.03	0.06
Spag7	Q7TNE3	Sperm-associated antigen 7	1.03	0.06
Entr1	A2AIW0	Endosome-associated-trafficking regulator 1	1.03	0.68
Ftsj3	Q9DBE9	pre-rRNA processing protein FTSJ3	1.03	0.25
Opa1	H7BX01	Dynamin-like 120 kDa protein, mitochondrial	1.03	0.19
Rab6b	P61294	Ras-related protein Rab-6B	1.03	0.51
Rbbp6	P97868	E3 ubiquitin-protein ligase RBBP6	1.03	0.18
Leprot	O89013	Leptin receptor gene-related protein	1.03	0.60
App	P12023	Amyloid-beta A4 protein	1.03	0.41
Acp6	Q8BP40	Lysophosphatidic acid phosphatase type 6	1.03	0.28
Odr4	Q4PJX1	Protein odr-4 homolog	1.03	0.20
Stt3b	A0A0R4J0D3	Dolichyl-diphosphooligosaccharide--protein glycosyltransferase subunit STT3B	1.03	0.28
Rassf8	Q8CJ96	Ras association domain-containing protein 8	1.03	0.26
Hax1	O35387	HCLS1-associated protein X-1	1.03	0.16
Mul1	Q8VCM5	Mitochondrial ubiquitin ligase activator of NFKB 1	1.03	0.37
Serinc1	Q9QZI8	Serine incorporator 1	1.03	0.37
Rpl30	P62889	60S ribosomal protein L30	1.03	0.51
Pcmtd2	Q8BHD8	Protein-L-isoaspartate O-methyltransferase domain-containing protein 2	1.03	0.64
Vps52	Q8C754	Vacuolar protein sorting-associated protein 52 homolog	1.03	0.38
Cox14	Q8BH51	Cytochrome c oxidase assembly protein COX14	1.03	0.10
Dym	Q8CHY3	Dymeclin	1.03	0.23
Spcs2	Q9CYN2	Signal peptidase complex subunit 2	1.03	0.38
Spp1	F8WIP8	Osteopontin	1.03	0.08
Ndufa12	A0A0R4J275	NADH dehydrogenase [ubiquinone] 1 alpha subcomplex subunit 12	1.03	0.22
Decr2	Q9WV68	Peroxisomal 2,4-dienoyl-CoA reductase	1.03	0.55
Stim1	A0A1B0GRA5	Stromal interaction molecule 1	1.03	0.19
Tcirg1	Q9JHF5	V-type proton ATPase subunit a	1.03	0.17
Rpl34	Q9D1R9	60S ribosomal protein L34	1.03	0.51
Nrm	Q8VC65	Nurim	1.03	0.56
Mdc1	E9QK89	Mediator of DNA damage checkpoint protein 1	1.03	0.62
Tap2	P36371	Antigen peptide transporter 2	1.03	0.18
Fig4	Q91WF7	Polyphosphoinositide phosphatase	1.03	0.10
Snx7	F8WI30	Sorting nexin-7	1.03	0.11
Numb	Q9QZS3	Protein numb homolog	1.03	0.30
Rbm8a	Q9CWZ3	RNA-binding protein 8A	1.03	0.29
Rpl26	P61255	60S ribosomal protein L26	1.03	0.51
Tpcn1	Q9EQJ0	Two pore calcium channel protein 1	1.03	0.49
Pafah1b2	Q61206	Platelet-activating factor acetylhydrolase IB subunit beta	1.03	0.35
Nsun6	Q7TS68	Putative methyltransferase NSUN6	1.03	0.38
Tmem167a	Q9CR64	Protein kish-A	1.03	0.55
Sec11a	D3YTS1	Signal peptidase complex catalytic subunit SEC11	1.03	0.46
Tarbp2	P97473	RISC-loading complex subunit TARBP2	1.03	0.33
Serpinb1a	Q9D154	Leukocyte elastase inhibitor A	1.03	0.45
Far1	Q922J9	Fatty acyl-CoA reductase 1	1.03	0.35
Commd4	Q9CQ02	COMM domain-containing protein 4	1.03	0.14
Ppcs	Q8VDG5	Phosphopantothenate--cysteine ligase	1.03	0.31
Cpt1a	P97742	Carnitine O-palmitoyltransferase 1, liver isoform	1.03	0.19
Surf1	P09925	Surfeit locus protein 1	1.03	0.41
Golga7	Q91W53	Golgin subfamily A member 7	1.03	0.31
Tdrkh	Q80VL1	Tudor and KH domain-containing protein	1.03	0.31
Sfxn2	Q925N2	Sideroflexin-2	1.03	0.47
Tmem120a	Q8C1E7	Transmembrane protein 120A	1.03	0.29
Pvr	Q8K094	Poliovirus receptor	1.03	0.04
Itpr2	Q9Z329	Inositol 1,4,5-trisphosphate receptor type 2	1.03	0.09
Lrrc20	Q8CI70	Leucine-rich repeat-containing protein 20	1.03	0.20
Srd5a3	Q9WUP4	Polyprenol reductase	1.03	0.47
Yipf4	Q8C407	Protein YIPF4	1.03	0.52
Ppp2r5a	Q6PD03	Serine/threonine-protein phosphatase 2A 56 kDa regulatory subunit alpha isoform	1.03	0.29
Scarb2	O35114	Lysosome membrane protein 2	1.03	0.27
Mitd1	Q8VDV8	MIT domain-containing protein 1	1.03	0.60
Ikbke	Q9R0T8	Inhibitor of nuclear factor kappa-B kinase subunit epsilon	1.03	0.35
Maged2	Q9ER67	Maged2 protein	1.03	0.32
Mbp	P04370	Myelin basic protein	1.03	0.42
Fam98c	E9PYD1	Family with sequence similarity 98, member C	1.03	0.60
Atl3	Q91YH5	Atlastin-3	1.03	0.25

Ube2l6	Q9QZU9	Ubiquitin/ISG15-conjugating enzyme E2 L6	1.03	0.34
Azi2	Q9QYP6	5-azacytidine-induced protein 2	1.03	0.32
L3hypdh	Q9CXA2	Trans-L-3-hydroxyproline dehydratase	1.03	0.51
Dpm1	O70152	Dolichol-phosphate mannosyltransferase subunit 1	1.03	0.22
Soga1	A2ACV6	Protein SOGA1	1.03	0.42
Bnip1	Q6QD59	Vesicle transport protein SEC20	1.03	0.65
Med17	Q8VCD5	Mediator of RNA polymerase II transcription subunit 17	1.03	0.59
Zdhhc13	Q9CWU2	Palmitoyltransferase ZDHHC13	1.03	0.74
Arl11	Q6P3A9	ADP-ribosylation factor-like protein 11	1.03	0.13
Atxn10	P28658	Ataxin-10	1.03	0.60
Gemin8	Q8BHE1	Gem-associated protein 8	1.03	0.14
Man1a	A0A1W2P788	alpha-1,2-Mannosidase (Fragment)	1.04	0.26
Acbd5	E9QNH7	Acyl-CoA-binding domain-containing protein 5	1.04	0.16
Ostm1	Q8BGT0	Osteopetrosis-associated transmembrane protein 1	1.04	0.58
Pqlc3	Q8C6U2	PQ-loop repeat-containing protein 3	1.04	0.48
Med22	Q62276	Mediator of RNA polymerase II transcription subunit 22	1.04	0.32
Sil1	Q9EPK6	Nucleotide exchange factor SIL1	1.04	0.15
Foxk1	P42128	Forkhead box protein K1	1.04	0.43
Nin	Q61043	Ninein	1.04	0.44
Tmx2	Q9D710	Thioredoxin-related transmembrane protein 2	1.04	0.46
Zbtb2	Q3V3W4	Zinc finger and BTB domain-containing 2	1.04	0.80
Ell	O08856	RNA polymerase II elongation factor ELL	1.04	0.36
Myo1a	O88329	Unconventional myosin-Ia	1.04	0.08
Hook3	Q8BUK6	Protein Hook homolog 3	1.04	0.30
Tmem189	Q99LQ7	Transmembrane protein 189	1.04	0.70
Zfp638	E9QML5	Zinc finger protein 638	1.04	0.11
Cers5	Q9D6K9	Ceramide synthase 5	1.04	0.40
Mrpl15	Q9CPR5	39S ribosomal protein L15, mitochondrial	1.04	0.32
Ube2j1	Q9JJZ4	Ubiquitin-conjugating enzyme E2 J1	1.04	0.10
Mtnd1	P03888	NADH-ubiquinone oxidoreductase chain 1	1.04	0.31
Cdc5l	Q6A068	Cell division cycle 5-like protein	1.04	0.08
Usp39	Q3TIX9	U4/U6.U5 tri-snRNP-associated protein 2	1.04	0.21
Rab6a	D3YV69	Ras-related protein Rab-6A	1.04	0.67
Hyal2	O35632	Hyaluronidase-2	1.04	0.15
Pi4kb	E9Q8A3	Phosphatidylinositol 4-kinase beta	1.04	0.51
Sec62	Q8BU14	Translocation protein SEC62	1.04	0.47
Yipf3	Q3UDR8	Protein YIPF3	1.04	0.43
Lmf2	Q8C3X8	Lipase maturation factor 2	1.04	0.22
Itfg1	Q99KW9	T-cell immunomodulatory protein	1.04	0.49
Glg1	Q61543	Golgi apparatus protein 1	1.04	0.16
Ecm1	Q61508	Extracellular matrix protein 1	1.04	0.52
Ap4s1	Q9WVL1	AP-4 complex subunit sigma-1	1.04	0.41
Atp5pd	Q9DCX2	ATP synthase subunit d, mitochondrial	1.04	0.28
Tomm40	Q9QYA2	Mitochondrial import receptor subunit TOM40 homolog	1.04	0.23
Usp30	Q3UN04	Ubiquitin carboxyl-terminal hydrolase 30	1.04	0.39
Unc93b1	E9PYK0	Protein unc-93 homolog B1	1.04	0.22
Syne1	A0A1L1STC6	Nesprin-1	1.04	0.38
Txnrd3	Q99MD6	Thioredoxin reductase 3	1.04	0.62
Rgs2	O08849	Regulator of G-protein signaling 2	1.04	0.39
Gm10273	Q9CXU4	Mitochondrial import inner membrane translocase subunit TIM23	1.04	0.21
Lrrc8a	Q80WG5	Volume-regulated anion channel subunit LRRC8A	1.04	0.56
Bud23	Q9CY21	Probable 18S rRNA (guanine-N(7))-methyltransferase	1.04	0.70
Tmem38b	Q9DAV9	Trimeric intracellular cation channel type B	1.04	0.41
Stx2	Q00262	Syntaxin-2	1.04	0.21
Rp2	Q9EPK2	Protein XRP2	1.04	0.08
Vrk1	Q80X41	Serine/threonine-protein kinase VRK1	1.04	0.15
Ddx21	Q9JIK5	Nucleolar RNA helicase 2	1.04	0.17
Tst	P52196	Thiosulfate sulfurtransferase	1.04	0.47
Emd	O08579	Emerin	1.04	0.26
Lbr	Q3U9G9	Lamin-B receptor	1.04	0.30
Slc25a4	P48962	ADP/ATP translocase 1	1.04	0.40
Flot1	O08917	Flotillin-1	1.04	0.18
Rab5if	Q9CQT9	Uncharacterized protein RAB5IF homolog	1.04	0.37
Mettl2	Q8BMK1	Methyltransferase-like protein 2	1.04	0.30
Fam76b	Q80XP8	Protein FAM76B	1.04	0.57
Ccdc9	D3YW42	Coiled-coil domain-containing protein 9	1.04	0.29
Irf2bp1	Q8R3Y8	Interferon regulatory factor 2-binding protein 1	1.04	0.29
Pinx1	Q9CZX5	PIN2/TERF1-interacting telomerase inhibitor 1	1.04	0.45
Rab31	Q3TXV4	Rab22B	1.04	0.08
Myo7a	P97479	Unconventional myosin-VIIa	1.04	0.73
Cc2d1a	E9PX94	Coiled-coil and C2 domain-containing protein 1A	1.04	0.43
Prdm2	A2A7B5	PR domain-containing 2, with ZNF domain	1.04	0.60
Vapa	Q9WV55	Vesicle-associated membrane protein-associated protein A	1.04	0.26

Slc30a5	Q8R4H9	Zinc transporter 5	1.04	0.38
Ccdc86	Q9JJ89	Coiled-coil domain-containing protein 86	1.04	0.11
AU040320	Z4YK56	Expressed sequence AU040320	1.04	0.42
Slc25a20	Q9Z2Z6	Mitochondrial carnitine/acylcarnitine carrier protein	1.04	0.23
Sh3bp5	Q9Z131	SH3 domain-binding protein 5	1.04	0.64
Stx4	P70452	Syntaxin-4	1.04	0.28
Ndc1	Q8VCB1	Nucleoporin NDC1	1.04	0.26
Rpp25l	Q99JH1	Ribonuclease P protein subunit p25-like protein	1.04	0.52
FAM120A	Q6A0A9	Constitutive coactivator of PPAR-gamma-like protein 1	1.04	0.30
Pltp	P55065	Phospholipid transfer protein	1.04	0.28
Tagln	P37804	Transgelin	1.04	0.29
Bms1	Q6PGF5	BMS1 homolog, ribosome assembly protein (Yeast)	1.04	0.48
Snap23	Q9D3L3	Synaptosomal-associated protein	1.04	0.25
Nf1	Q04690	Neurofibromin	1.04	0.25
Tmppe	D3Z286	Transmembrane protein with metallophosphoesterase domain	1.04	0.29
Acsl1	D3Z041	Long-chain-fatty-acid--CoA ligase 1	1.04	0.13
Tsr2	H7BWY8	Pre-rRNA-processing protein TSR2 homolog	1.04	0.53
Fam3a	I7HJS7	Novel protein	1.04	0.32
Tbk1	Q9WUN2	Serine/threonine-protein kinase TBK1	1.04	0.29
Ap4e1	Q80V94	AP-4 complex subunit epsilon-1	1.04	0.50
Sap30l	Q5SQF8	Histone deacetylase complex subunit SAP30L	1.04	0.47
Clec4a2	Q923C7	C-type lectin domain family 4, member a2	1.04	0.13
Traf5	P70191	TNF receptor-associated factor 5	1.04	0.61
Ewsr1	Q5SUS9	RNA-binding protein EWS	1.04	0.36
Abhd17b	Q7M759	Alpha/beta hydrolase domain-containing protein 17B	1.04	0.22
Atp13a2	Q9CTG6	Cation-transporting ATPase 13A2	1.04	0.55
Atp8a1	A0A0M3HEP7	Phospholipid-transporting ATPase	1.04	0.25
Rrs1	Q9CYH6	Ribosome biogenesis regulatory protein homolog	1.04	0.34
Nek6	Q9ES70	Serine/threonine-protein kinase Nek6	1.04	0.23
	Q9CWU4	UPF0690 protein C1orf52 homolog	1.04	0.43
Ndufv3	Q3U422	NADH dehydrogenase [ubiquinone] flavoprotein 3, mitochondrial	1.04	0.21
Ebna1bp2	Q9D903	Probable rRNA-processing protein EBP2	1.04	0.25
Ddx47	Q9CWX9	Probable ATP-dependent RNA helicase DDX47	1.04	0.35
Urb1	E9PU96	Nucleolar pre-ribosomal-associated protein 1	1.04	0.15
Mau2	A0A1D5RLR7	MAU2 chromatid cohesion factor homolog	1.04	0.60
Traf2	P39429	TNF receptor-associated factor 2	1.04	0.39
Ccnyl1	D3YUJ3	Cyclin Y-like 1	1.04	0.44
Cd72	Q3UZ35	B-cell differentiation antigen CD72	1.04	0.22
Fam45a	Q9D8N2	Protein FAM45A	1.04	0.10
Gm11127	A7VMS2	MHC classIb T15	1.04	0.26
Ift22	Q9DAI2	Intraflagellar transport protein 22 homolog	1.04	0.20
Lpgat1	E9QL80	Acyl-CoA:lysophosphatidylglycerol acyltransferase 1	1.04	0.56
Zbtb11	G5E8B9	MCG130893	1.04	0.46
Spg7	Q3ULF4	Paraplegin	1.04	0.37
Entpd1	Q8CDV7	Ectonucleoside triphosphate diphosphohydrolase 1	1.04	0.19
Eno3	P21550	Beta-enolase	1.04	0.22
Mbd2	Q9Z2E1	Methyl-CpG-binding domain protein 2	1.04	0.30
Mospd2	B1AU74	Motile sperm domain-containing protein 2	1.04	0.02
Ddx31	Q6NZQ2	Probable ATP-dependent RNA helicase DDX31	1.04	0.30
Gm21985	Q6P6P5	Predicted gene 21985	1.04	0.23
Tpd52	D3Z125	Tumor protein D52 (Fragment)	1.04	0.80
Mgat1	P27808	Alpha-1,3-mannosyl-glycoprotein 2-beta-N-acetylglucosaminyltransferase	1.04	0.43
Usp33	Q8R5K2	Ubiquitin carboxyl-terminal hydrolase 33	1.04	0.46
Phf2	Q9WTU0	Lysine-specific demethylase PHF2	1.04	0.48
Isy1	Q69ZQ2	Pre-mRNA-splicing factor ISY1 homolog	1.04	0.24
Tlr13	Q6R5N8	Toll-like receptor 13	1.04	0.11
Suox	Q8R086	Sulfite oxidase, mitochondrial	1.04	0.27
Rpl35	Q6ZWV7	60S ribosomal protein L35	1.04	0.38
Gabarap	Q9DCD6	Gamma-aminobutyric acid receptor-associated protein	1.04	0.02
Numa1	E9Q7G0	Nuclear mitotic apparatus protein 1	1.04	0.34
Eml4	F8WJ93	Echinoderm microtubule-associated protein-like 4	1.04	0.44
Fxn	O35943	Frataxin, mitochondrial	1.04	0.25
Fam207a	P58468	Protein FAM207A	1.04	0.31
Cd81	P35762	CD81 antigen	1.04	0.60
Akap9	E9QQ10	A-kinase anchor protein 9	1.04	0.45
Chchd6	Q91VN4	MICOS complex subunit Mic25	1.04	0.26
Thbd	P15306	Thrombomodulin	1.04	0.14
Samm50	Q8BGH2	Sorting and assembly machinery component 50 homolog	1.04	0.13
Nop56	Q9D6Z1	Nucleolar protein 56	1.04	0.10
Lmf1	Q3U3R4	Lipase maturation factor 1	1.04	0.51
Tmem192	Q9CXT7	Transmembrane protein 192	1.04	0.43
Rab24	P35290	Ras-related protein Rab-24	1.04	0.24
Pet117	P0DJF2	Protein PET117 homolog, mitochondrial	1.04	0.47

C2cd2l	Q80X80	Phospholipid transfer protein C2CD2L	1.04	0.48
Taf15	Q8BQ46	TAF15 RNA polymerase II, TATA box binding protein (TBP)-associated factor	1.04	0.30
Mrpl27	Q99N92	39S ribosomal protein L27, mitochondrial	1.04	0.42
Slc27a4	Q91VE0	Long-chain fatty acid transport protein 4	1.04	0.20
Mical2	Q8BML1	[F-actin]-monooxygenase MICAL2	1.04	0.28
Sh3bgrl2	Q8BG73	SH3 domain-binding glutamic acid-rich-like protein 2	1.04	0.24
Cebpzos	Q8BTE5	Protein CEBPZOS	1.04	0.21
Otud7b	B2RUR8	OTU domain-containing protein 7B	1.04	0.41
Rfx1	P48377	MHC class II regulatory factor RFX1	1.04	0.41
Ptpn18	Q61152	Tyrosine-protein phosphatase non-receptor type 18	1.04	0.60
Scamp4	Q9JKV5	Secretory carrier-associated membrane protein 4	1.04	0.16
Srpra	Q9DBG7	Signal recognition particle receptor subunit alpha	1.04	0.30
Ndufc2	Q9CQ54	NADH dehydrogenase [ubiquinone] 1 subunit C2	1.04	0.07
Stx8	O88983	Syntaxin-8	1.04	0.22
Abcd1	P48410	ATP-binding cassette sub-family D member 1	1.04	0.26
Trmt10c	Q3UFY8	tRNA methyltransferase 10 homolog C	1.04	0.32
Mertk	Q60805	Tyrosine-protein kinase Mer	1.04	0.42
Strada	Q3UUJ4	STE20-related kinase adapter protein alpha	1.04	0.28
Tap1	P21958	Antigen peptide transporter 1	1.04	0.28
Uck2	Q99PM9	Uridine-cytidine kinase 2	1.04	0.51
Mrpl49	Q9CQ40	39S ribosomal protein L49, mitochondrial	1.04	0.21
Slc25a11	Q9CR62	Mitochondrial 2-oxoglutarate/malate carrier protein	1.04	0.11
Echdc3	Q9D7J9	Enoyl-CoA hydratase domain-containing protein 3, mitochondrial	1.04	0.46
Syngr1	O55100	Synaptogyrin-1	1.04	0.13
Rrp7a	Q9D1C9	Ribosomal RNA-processing protein 7 homolog A	1.04	0.29
Lpcat2	Q8BYI6	Lysophosphatidylcholine acyltransferase 2	1.04	0.42
Kifc5b	E9PUA5	Kinesin-like protein	1.04	0.44
Malt1	Q2TBA3	Mucosa-associated lymphoid tissue lymphoma translocation protein 1 homolog	1.04	0.07
Elovl1	Q9JLJ5	Elongation of very long chain fatty acids protein 1	1.04	0.42
Isg20l2	A0A0R4J0R3	Interferon-stimulated 20 kDa exonuclease-like 2	1.04	0.22
Lasp1	A2A6H1	LIM and SH3 domain protein 1 (Fragment)	1.04	0.67
Bak1	O08734	Bcl-2 homologous antagonist/killer	1.04	0.51
Gramd2b	Q6PEM6	GRAM domain-containing protein 2B	1.04	0.49
Cd28	P31041	T-cell-specific surface glycoprotein CD28	1.04	0.62
P2rx7	Q9Z1M0	P2X purinoceptor 7	1.04	0.14
Gas6	Q61592	Growth arrest-specific protein 6	1.04	0.40
Sf3b5	Q923D4	Splicing factor 3B subunit 5	1.04	0.22
Zc3h4	Q6ZPZ3	Zinc finger CCCH domain-containing protein 4	1.04	0.22
Emc4	Q9CZX9	ER membrane protein complex subunit 4	1.04	0.10
Tefm	Q5SSK3	Transcription elongation factor, mitochondrial	1.04	0.26
Impad1	Q80V26	Inositol monophosphatase 3	1.04	0.55
Fgg	Q3UER8	Fibrinogen gamma chain	1.04	0.68
Ftsj1	Q8CBC7	Putative tRNA (cytidine(32)/guanosine(34)-2'-O)-methyltransferase	1.04	0.69
Dpysl3	E9PWE8	Dihydropyrimidinase-related protein 3	1.04	0.56
Bod1l	E9Q6J5	Biorientation of chromosomes in cell division protein 1-like 1	1.04	0.16
Epb41	A0A1D5RLV1	Protein 4.1 (Fragment)	1.04	0.39
Mmgt1	Q8K273	Membrane magnesium transporter 1	1.04	0.42
Letmd1	Q924L1	LETM1 domain-containing protein 1	1.04	0.29
Rdh14	Q9ERI6	Retinol dehydrogenase 14	1.04	0.33
Sike1	Q9CPR7	Suppressor of IKBKE 1	1.04	0.27
Ppp3cb	P48453	Serine/threonine-protein phosphatase 2B catalytic subunit beta isoform	1.04	0.14
Junb	P09450	Transcription factor jun-B	1.04	0.14
Actr8	Q8R2S9	Actin-related protein 8	1.04	0.73
Tmsb15l	Q8C0W0	Thymosin beta 15b-like	1.04	0.09
Rab12	A2CG35	Ras-related protein Rab-12	1.04	0.23
Notch2	G5E8J0	Neurogenic locus notch homolog protein 2	1.04	0.17
Sft2d2	Q8VD57	Vesicle transport protein SFT2B	1.04	0.28
Snx24	Q9CRB0	Sorting nexin-24	1.04	0.41
Ric8a	Q3TIR3	Synembryn-A	1.04	0.22
Sdf2l1	Q9ESP1	Stromal cell-derived factor 2-like protein 1	1.04	0.28
Atp7a	A2AG68	Copper-transporting ATPase 1	1.04	0.20
Deptor	Q570Y9	DEP domain-containing mTOR-interacting protein	1.04	0.63
Slc4a2	A0A0R4J101	Anion exchange protein	1.04	0.48
Rps9	Q6ZWN5	40S ribosomal protein S9	1.04	0.24
Sts	P50427	Steryl-sulfatase	1.04	0.34
Top1	Q04750	DNA topoisomerase 1	1.04	0.23
Mpdu1	Q8R0J2	Mannose-P-dolichol utilization defect 1	1.04	0.44
Ano10	Q8BH79	Anoctamin-10	1.04	0.29
Pik3r5	Q5SW28	Phosphoinositide 3-kinase regulatory subunit 5	1.04	0.62
Mvk	Q9R008	Mevalonate kinase	1.04	0.82
Dmac2	Q9D7K5	Distal membrane-arm assembly complex protein 2	1.04	0.50
Wipi1	Q8R3E3	WD repeat domain phosphoinositide-interacting protein 1	1.04	0.49
Ddx52	Q8K301	Probable ATP-dependent RNA helicase DDX52	1.04	0.37

Gabarapl1	Q8R3R8	Gamma-aminobutyric acid receptor-associated protein-like 1	1.04	0.18
Acsf3	Q3URE1	Acyl-CoA synthetase family member 3, mitochondrial	1.04	0.19
Cd99l2	Q8BIF0	CD99 antigen-like protein 2	1.04	0.45
Sirpa	Q6P6I8	Signal-regulatory protein alpha	1.04	0.18
Fam20b	Q8VCS3	Glycosaminoglycan xylosylkinase	1.04	0.39
Rps2	P25444	40S ribosomal protein S2	1.04	0.10
Tmem87b	Q8BKU8	Transmembrane protein 87B	1.04	0.20
Ckap4	Q8BMK4	Cytoskeleton-associated protein 4	1.04	0.26
Dusp9	Q7TNL7	Dual specificity protein phosphatase	1.04	0.19
Tmem104	Q3TB48	Transmembrane protein 104	1.04	0.24
Eepd1	Q3TGW2	Endonuclease/exonuclease/phosphatase family domain-containing protein 1	1.04	0.33
Pnisr	A2AJT5	Arginine/serine-rich protein PNISR	1.04	0.75
Tmtc3	G5E8C4	MCG142017, isoform CRA_a	1.04	0.38
Tecr	Q52L67	Gpsn2 protein	1.04	0.35
Carnmt1	Q80UY1	Carnosine N-methyltransferase	1.04	0.22
Atp6v1d	P57746	V-type proton ATPase subunit D	1.04	0.30
Cdkn2aipnl	Q9D211	CDKN2AIP N-terminal-like protein	1.04	0.48
Dido1	Q8C9B9	Death-inducer obliterator 1	1.04	0.21
Tnfaip2	D3Z4L9	Tumor necrosis factor alpha-induced protein 2	1.04	0.02
Tmed3	Q78IS1	Transmembrane emp24 domain-containing protein 3	1.04	0.53
Itgb1	P09055	Integrin beta-1	1.04	0.11
Tmem101	Q91VP7	Transmembrane protein 101	1.04	0.62
Ubxn8	Q3TTF2	UBX domain-containing protein 8	1.04	0.58
Morc2a	Q69ZX6	MORC family CW-type zinc finger protein 2A	1.04	0.31
Snrpb2	Q9CQI7	U2 small nuclear ribonucleoprotein B''	1.04	0.06
Arsk	A0A0R4J1N2	Arylsulfatase K	1.04	0.42
Mtif2	Q91YJ5	Translation initiation factor IF-2, mitochondrial	1.04	0.25
B3gat3	P58158	Galactosylgalactosylxylosylprotein 3-beta-glucuronosyltransferase 3	1.04	0.54
Scamp3	Q3UXS0	Secretory carrier-associated membrane protein	1.04	0.19
Aph1a	Q8BVF7	Gamma-secretase subunit APH-1A	1.04	0.40
Eps8	Q08509	Epidermal growth factor receptor kinase substrate 8	1.04	0.37
Slc12a4	F8WIJ0	Solute carrier family 12 member 4	1.04	0.10
Slc25a24	Q8BMD8	Calcium-binding mitochondrial carrier protein SCaMC-1	1.04	0.12
Ndufb5	Q9CQH3	NADH dehydrogenase [ubiquinone] 1 beta subcomplex subunit 5, mitochondrial	1.04	0.27
	Q8BHN7	Uncharacterized protein C12orf29 homolog	1.04	0.41
Cyb5r4	Q3TDX8	Cytochrome b5 reductase 4	1.04	0.13
Plcb2	A3KGF7	1-phosphatidylinositol 4,5-bisphosphate phosphodiesterase beta-2	1.04	0.42
Ergic2	Q9CR89	Endoplasmic reticulum-Golgi intermediate compartment protein 2	1.04	0.48
Nol8	E9QKD1	Nucleolar protein 8	1.04	0.49
Acer3	Q9D099	Alkaline ceramidase 3	1.04	0.47
Taok1	Q5F2E8	Serine/threonine-protein kinase TAO1	1.04	0.24
Coq3	Q8BMS4	Ubiquinone biosynthesis O-methyltransferase, mitochondrial	1.04	0.47
Mrpl3	Q99N95	39S ribosomal protein L3, mitochondrial	1.04	0.21
Haus8	Q99L00	HAUS augmin-like complex subunit 8	1.04	0.05
Scaf4	Q6PFF0	SR-related CTD-associated factor 4	1.04	0.57
Cnn3	Q9DAW9	Calponin-3	1.04	0.50
Stk26	Q99JT2	Serine/threonine-protein kinase 26	1.04	0.62
Taf6	Q62311	Transcription initiation factor TFIID subunit 6	1.04	0.47
Mrpl22	Q8BU88	39S ribosomal protein L22, mitochondrial	1.04	0.39
Mcm3ap	Q9WUU9	Germinal-center associated nuclear protein	1.04	0.61
Gramd1b	A0A1D5RLT6	GRAM domain-containing protein 1B	1.04	0.35
Nop14	Q8R3N1	Nucleolar protein 14	1.04	0.26
Ctsb	P10605	Cathepsin B	1.04	0.56
Mrm1	Q99J25	rRNA methyltransferase 1, mitochondrial	1.04	0.53
Dcaf1	Q80TR8	DDB1- and CUL4-associated factor 1	1.04	0.27
Dcakd	Q8BHC4	Dephospho-CoA kinase domain-containing protein	1.04	0.23
Ddx24	F8WJA0	ATP-dependent RNA helicase DDX24	1.04	0.49
Rps4x	P62702	40S ribosomal protein S4, X isoform	1.04	0.17
Smad4	P97471	Mothers against decapentaplegic homolog 4	1.04	0.10
Mettl7a1	Q8C6B0	MCG20149, isoform CRA_a	1.04	0.31
Irak2	Q8CFA1	Interleukin-1 receptor-associated kinase-like 2	1.04	0.26
Sbf1	Q6ZPE2	Myotubularin-related protein 5	1.04	0.40
Plscr3	Q9JIZ9	Phospholipid scramblase 3	1.04	0.56
Pcid2	Q8BFV2	PCI domain-containing protein 2	1.04	0.25
Pip4p1	Q3TWL2	Type 1 phosphatidylinositol 4,5-bisphosphate 4-phosphatase	1.04	0.49
Gosr1	O88630	Golgi SNAP receptor complex member 1	1.04	0.06
Marchf5	Q3KNM2	E3 ubiquitin-protein ligase MARCH5	1.04	0.49
Amacr	O09174	Alpha-methylacyl-CoA racemase	1.04	0.71
Hgf	Q08048	Hepatocyte growth factor	1.04	0.29
Uhrf1	Q8VDF2	E3 ubiquitin-protein ligase UHRF1	1.04	0.36
Smpd4	Q6ZPR5	Sphingomyelin phosphodiesterase 4	1.04	0.21
Trpv2	Q9WTR1	Transient receptor potential cation channel subfamily V member 2	1.04	0.07
Cox4i1	P19783	Cytochrome c oxidase subunit 4 isoform 1, mitochondrial	1.04	0.25

Kif1bp	Q6ZPU9	KIF1-binding protein	1.04	0.22
Adam9	A0A140LHU0	Disintegrin and metalloproteinase domain-containing protein 9	1.04	0.10
Slc37a2	Q9WU81	Glucose-6-phosphate exchanger SLC37A2	1.04	0.25
Slc25a1	Q8JZU2	Tricarboxylate transport protein, mitochondrial	1.04	0.26
Prkrip1	Q9CWV6	PRKR-interacting protein 1	1.04	0.18
Sypl1	O09117	Synaptophysin-like protein 1	1.04	0.23
Fam122a	Q9DB52	Protein FAM122A	1.04	0.16
Bad	Q61337	Bcl2-associated agonist of cell death	1.04	0.26
Tcp11l1	Q8BTG3	T-complex protein 11-like protein 1	1.04	0.28
Ube2g1	P62254	Ubiquitin-conjugating enzyme E2 G1	1.04	0.48
Wfdc17	Q5SSJ1	Activated macrophage/microglia WAP domain protein	1.04	0.42
Birc5	O70201	Baculoviral IAP repeat-containing protein 5	1.04	0.27
Sharpin	Q91WA6	Sharpin	1.04	0.25
Yipf1	Q91VU1	Protein YIPF1	1.04	0.43
Sel1l	Q9Z2G6	Protein sel-1 homolog 1	1.04	0.26
Cfp	P11680	Properdin	1.04	0.20
Rai1	Q61818	Retinoic acid-induced protein 1	1.04	0.37
Fam162a	Q9D6U8	Protein FAM162A	1.04	0.26
Agap3	F8VQE9	Arf-GAP with GTPase, ANK repeat and PH domain-containing protein 3	1.04	0.21
Snrpa	Q62189	U1 small nuclear ribonucleoprotein A	1.04	0.13
Pfkfb4	Q6DTY7	6-phosphofructo-2-kinase/fructose-2,6-bisphosphatase 4	1.04	0.23
Golgb1	E9PVZ8	Golgi autoantigen, golgin subfamily b, macrogolgin 1	1.04	0.20
Rprd1a	Q8VDS4	Regulation of nuclear pre-mRNA domain-containing protein 1A	1.04	0.14
Suz12	Q80U70	Polycomb protein Suz12	1.04	0.44
Uhrf1bp1	B2KF50	UHRF1 (ICBP90)-binding protein 1	1.04	0.63
Sec63	Q8VHE0	Translocation protein SEC63 homolog	1.04	0.12
Bola3	E9PX89	BolA-like protein 3	1.04	0.26
Tmbim1	Q3U717	Protein lifeguard 3	1.04	0.09
Ttyh3	Q6P5F7	Protein tweety homolog 3	1.04	0.17
Trip10	Q8CJ53	Cdc42-interacting protein 4	1.04	0.18
Atox1	O08997	Copper transport protein ATOX1	1.04	0.73
Mettl27	Q8BGM4	Methyltransferase-like 27	1.04	0.44
Ubtd1	Q91WB7	Ubiquitin domain-containing protein 1	1.04	0.25
Zc3h14	Q8BJ05	Zinc finger CCCH domain-containing protein 14	1.04	0.22
Atad1	Q9D5T0	ATPase family AAA domain-containing protein 1	1.04	0.31
Cyb5r3	Q9DCN2	NADH-cytochrome b5 reductase 3	1.04	0.22
Sar1b	Q9CQC9	GTP-binding protein SAR1b	1.04	0.21
Sde2	Q8K1J5	Replication stress response regulator SDE2	1.04	0.27
Fars2	A0A286YCZ5	Phenylalanine--tRNA ligase, mitochondrial	1.04	0.42
Acly	Q91V92	ATP-citrate synthase	1.04	0.18
Pisd	E9PX91	Phosphatidylserine decarboxylase proenzyme, mitochondrial	1.04	0.21
Smad5	P97454	Mothers against decapentaplegic homolog 5	1.04	0.61
Pgrmc2	Q80UU9	Membrane-associated progesterone receptor component 2	1.04	0.21
Cd84	A0A0R4J0K5	SLAM family member 5	1.04	0.15
Immt	Q8CAQ8	MICOS complex subunit Mic60	1.04	0.16
Rgs3	Q9DC04	Regulator of G-protein signaling 3	1.04	0.37
Efr3a	A0A1D5RLL3	Protein EFR3 homolog A	1.04	0.15
Bcat1	Q8CBC8	Branched-chain-amino-acid aminotransferase	1.04	0.58
Rab43	Q8CG50	Ras-related protein Rab-43	1.04	0.24
Angel2	Q8K1C0	Protein angel homolog 2	1.04	0.36
Fbxo38	Q8BMI0	F-box only protein 38	1.04	0.23
Ndufb3	Q9CQZ6	NADH dehydrogenase [ubiquinone] 1 beta subcomplex subunit 3	1.04	0.43
Ptdss1	Q99LH2	Phosphatidylserine synthase 1	1.04	0.35
Grpel2	O88396	GrpE protein homolog 2, mitochondrial	1.04	0.61
Gng12	A0A0N4SW28	Guanine nucleotide-binding protein subunit gamma	1.04	0.12
Jak3	A0A0R4J0R7	Tyrosine-protein kinase	1.04	0.46
Rai14	Q9EP71	Ankycorbin	1.04	0.15
Usp46	P62069	Ubiquitin carboxyl-terminal hydrolase 46	1.04	0.29
Stx5a	I1E4X1	Syntaxin 5A	1.04	0.19
Rps19bp1	Q8C6B9	Active regulator of SIRT1	1.04	0.50
C1qc	Q02105	Complement C1q subcomponent subunit C	1.04	0.61
Atp2a2	O55143	Sarcoplasmic/endoplasmic reticulum calcium ATPase 2	1.04	0.27
Hint2	Q9D0S9	Histidine triad nucleotide-binding protein 2, mitochondrial	1.04	0.30
Faf2	Q3TDN2	FAS-associated factor 2	1.04	0.26
Gnptg	A0A0R4J0H5	N-acetylglucosamine-1-phosphotransferase subunit gamma	1.04	0.08
Raet1d	Q9JI58	Retinoic acid early-inducible protein 1-delta	1.04	0.38
Zfr	O88532	Zinc finger RNA-binding protein	1.04	0.10
Pelp1	Q9DBD5	Proline-, glutamic acid- and leucine-rich protein 1	1.04	0.12
Rps5	Q91V55	40S ribosomal protein S5	1.04	0.13
Klf13	Q9JJZ6	Krueppel-like factor 13	1.04	0.44
Fga	E9PV24	Fibrinogen alpha chain	1.04	0.71
Alas1	Q8VC19	5-aminolevulinate synthase, nonspecific, mitochondrial	1.04	0.26
Xxylt1	Q3U4G3	Xyloside xylosyltransferase 1	1.04	0.18

Hmmr	Q00547	Hyaluronan mediated motility receptor	1.04	0.34
Ticam1	Q80UF7	TIR domain-containing adapter molecule 1	1.04	0.48
Exosc3	Q7TQK4	Exosome complex component RRP40	1.04	0.24
Alg1	Q921Q3	Chitobiosyldiphosphodolichol beta-mannosyltransferase	1.04	0.33
Dgkq	Q6P5E8	Diacylglycerol kinase theta	1.04	0.37
Wasl	Q91YD9	Neural Wiskott-Aldrich syndrome protein	1.04	0.27
Ndufb11	O09111	NADH dehydrogenase [ubiquinone] 1 beta subcomplex subunit 11, mitochondrial	1.04	0.33
Fn1	P11276	Fibronectin	1.04	0.11
Lman2l	D3Z4P2	Lectin, mannose-binding 2-like, isoform CRA_b	1.04	0.20
Cd48	P18181	CD48 antigen	1.04	0.14
Hif1an	Q8BLR9	Hypoxia-inducible factor 1-alpha inhibitor	1.04	0.21
Lilrb3	P97484	Leukocyte immunoglobulin-like receptor subfamily B member 3	1.04	0.27
Mrps36	Q9CQX8	28S ribosomal protein S36, mitochondrial	1.04	0.07
Fndc3a	Q8BX90	Fibronectin type-III domain-containing protein 3A	1.04	0.15
Stk38l	Q7TSE6	Serine/threonine-protein kinase 38-like	1.04	0.30
Plxna1	P70206	Plexin-A1	1.04	0.20
Hacd3	Q8K2C9	Very-long-chain (3R)-3-hydroxyacyl-CoA dehydratase 3	1.04	0.27
Mapk7	Q9WVS8	Mitogen-activated protein kinase 7	1.04	0.21
Fcgr3	A0A0B4J1M6	Low affinity immunoglobulin gamma Fc region receptor III	1.04	0.74
Nfam1	Q8R4V1	NFAT activation molecule 1	1.04	0.11
Rrp1b	Q91YK2	Ribosomal RNA processing protein 1 homolog B	1.04	0.17
Dram2	Q9CR48	DNA damage-regulated autophagy modulator protein 2	1.04	0.61
Mon1a	A0A0R4J0D5	Vacuolar fusion protein MON1 homolog A	1.04	0.44
Tmpo	Q61029	Lamina-associated polypeptide 2, isoforms beta/delta/epsilon/gamma	1.04	0.23
Pde3b	Q61409	cGMP-inhibited 3',5'-cyclic phosphodiesterase B	1.04	0.13
Mrpl30	Q9D7N6	39S ribosomal protein L30, mitochondrial	1.04	0.24
Rnf115	Q9D0C1	E3 ubiquitin-protein ligase RNF115	1.04	0.62
	Q3U6N9	UPF0488 protein C8orf33 homolog	1.04	0.29
Ubl3	Q9Z2M6	Ubiquitin-like protein 3	1.04	0.41
Csnk1a1	E9PWB2	Casein kinase I isoform alpha	1.04	0.34
Gtf3c5	Q8R2T8	General transcription factor 3C polypeptide 5	1.04	0.35
Macrod1	Q922B1	O-acetyl-ADP-ribose deacetylase MACROD1	1.04	0.37
Emc7	Q9EP72	ER membrane protein complex subunit 7	1.04	0.22
Brd7	O88665	Bromodomain-containing protein 7	1.04	0.73
Phlda3	Q9WV95	Pleckstrin homology-like domain family A member 3	1.04	0.39
Tnfrsf1b	P25119	Tumor necrosis factor receptor superfamily member 1B	1.04	0.53
Sfxn1	Q99JR1	Sideroflexin-1	1.04	0.04
Pex3	Q9QXY9	Peroxisomal biogenesis factor 3	1.04	0.27
Dffa	O54786	DNA fragmentation factor subunit alpha	1.04	0.21
Npc1	O35604	NPC intracellular cholesterol transporter 1	1.04	0.20
Ppic	P30412	Peptidyl-prolyl cis-trans isomerase C	1.04	0.49
Tex2	Q6ZPJ0	Testis-expressed protein 2	1.04	0.29
Tapt1	Q4VBD2	Transmembrane anterior posterior transformation protein 1	1.04	0.19
Atp6ap2	Q9CYN9	Renin receptor	1.04	0.07
Lsm14b	Q8CGC4	Protein LSM14 homolog B	1.04	0.31
Tprn	A2AI08	Taperin	1.04	0.56
Cald1	E9QA16	Caldesmon 1	1.04	0.33
Fam120c	Q8C3F2	Constitutive coactivator of PPAR-gamma-like protein 2	1.04	0.63
Secisbp2l	A2AQE2	SECIS binding protein 2-like	1.04	0.61
Abcb10	Q9JI39	ATP-binding cassette sub-family B member 10, mitochondrial	1.04	0.56
Rab21	P35282	Ras-related protein Rab-21	1.04	0.15
Pclaf	Q9CQX4	PCNA-associated factor	1.04	0.15
Aplp2	Q60709	Amyloid-like protein 2	1.04	0.06
Cgnl1	Q6AW69	Cingulin-like protein 1	1.04	0.63
Tex264	E9Q137	Testis-expressed gene 264	1.04	0.11
Adam8	Q05910	Disintegrin and metalloproteinase domain-containing protein 8	1.04	0.14
Rad17	Q6NXW6	Cell cycle checkpoint protein RAD17	1.04	0.10
Sgpp1	Q9JI99	Sphingosine-1-phosphate phosphatase 1	1.04	0.38
Slc44a2	A0A1L1SVG6	Choline transporter-like protein 2	1.04	0.38
Sft2d3	E9QJT3	Vesicle transport protein	1.04	0.46
Slmap	D3Z7V3	Sarcolemmal membrane-associated protein	1.04	0.24
Mavs	Q8VCF0	Mitochondrial antiviral-signaling protein	1.04	0.09
Rcan3	Q9JKK0	Calcipressin-3	1.04	0.55
Hsdl1	Q8BTX9	Inactive hydroxysteroid dehydrogenase-like protein 1	1.04	0.18
Rgs18	Q99PG4	Regulator of G-protein signaling 18	1.04	0.34
Smc6	Q924W5	Structural maintenance of chromosomes protein 6	1.04	0.57
Mapre3	Q6PER3	Microtubule-associated protein RP/EB family member 3	1.04	0.40
Bst2	Q8R2Q8	Bone marrow stromal antigen 2	1.04	0.25
Pam16	Q9CQV1	Mitochondrial import inner membrane translocase subunit TIM16	1.04	0.23
Hpgds	Q9JHF7	Hematopoietic prostaglandin D synthase	1.04	0.05
Ncapg	E9PWG6	Non-SMC condensin I complex, subunit G	1.04	0.51
Mtdh	F6QHD1	Protein LYRIC (Fragment)	1.04	0.22
Nlrx1	Q3TL44	NLR family member X1	1.04	0.33

Rbmx2	Q8R0F5	RNA-binding motif protein, X-linked 2	1.05	0.41
Reep5	G3X8R0	Receptor expression-enhancing protein	1.05	0.20
Clec4a1	Q80UI7	C-type lectin domain family 4, member a1	1.05	0.40
Agk	Q9ESW4	Acylglycerol kinase, mitochondrial	1.05	0.08
Nudt19	P11930	Nucleoside diphosphate-linked moiety X motif 19	1.05	0.34
Gpr137b	A0A1Y7VLJ5	Integral membrane protein GPR137B	1.05	0.13
Dnajc21	E9Q8D0	DnaJ homolog subfamily C member 21	1.05	0.43
Pi4k2b	Q8CBQ5	Phosphatidylinositol 4-kinase type 2-beta	1.05	0.17
Timm50	Q9D880	Mitochondrial import inner membrane translocase subunit TIM50	1.05	0.32
Src	P05480	Neuronal proto-oncogene tyrosine-protein kinase Src	1.05	0.26
Ppp2r5b	Q6PD28	Serine/threonine-protein phosphatase 2A 56 kDa regulatory subunit beta isoform	1.05	0.72
Pomk	Q3TUA9	Protein O-mannose kinase	1.05	0.25
Cox7a2l	E9PZS8	Cytochrome c oxidase subunit 7A-related protein, mitochondrial	1.05	0.34
Fmn1	Q05860	Formin-1	1.05	0.17
Arl2bp	Q9D385	ADP-ribosylation factor-like protein 2-binding protein	1.05	0.06
Lrmda	Q9D9B4	Leucine-rich melanocyte differentiation-associated protein	1.05	0.28
Chmp6	P0C0A3	Charged multivesicular body protein 6	1.05	0.12
Bbip1	J3QMK2	BBSome-interacting protein 1	1.05	0.30
Mia3	Q8BI84	Transport and Golgi organization protein 1 homolog	1.05	0.15
Ulk1	Q6PB82	Serine/threonine-protein kinase	1.05	0.56
Pum3	A0A0N4SUH4	Pumilio homolog 3	1.05	0.17
Arnt	P53762	Aryl hydrocarbon receptor nuclear translocator	1.05	0.31
Dhrs7b	Q99J47	Dehydrogenase/reductase SDR family member 7B	1.05	0.20
Pon2	Q62086	Serum paraoxonase/arylesterase 2	1.05	0.18
Ppan	Q91YU8	Suppressor of SWI4 1 homolog	1.05	0.42
Cnp	P16330	2',3'-cyclic-nucleotide 3'-phosphodiesterase	1.05	0.24
Ncln	Q8VCM8	Nicalin	1.05	0.04
Mfsd10	Q9D2V8	Major facilitator superfamily domain-containing protein 10	1.05	0.17
Timm10	P62073	Mitochondrial import inner membrane translocase subunit Tim10	1.05	0.09
Rimoc1	Q8BR90	RAB7A-interacting MON1-CCZ1 complex subunit 1	1.05	0.04
Cds2	Q99L43	Phosphatidate cytidylyltransferase 2	1.05	0.45
Fmo5	P97872	Dimethylaniline monooxygenase [N-oxide-forming] 5	1.05	0.15
Gna12	P27600	Guanine nucleotide-binding protein subunit alpha-12	1.05	0.25
Atp6v0a2	P15920	V-type proton ATPase 116 kDa subunit a isoform 2	1.05	0.53
Acad12	D3Z7X0	Acyl-Coenzyme A dehydrogenase family, member 12	1.05	0.53
Hdhd3	Q9CYW4	Haloacid dehalogenase-like hydrolase domain-containing protein 3	1.05	0.39
Mia2	H3BJS0	Melanoma inhibitory activity protein 2	1.05	0.12
Plekho1	Q9JIY0	Pleckstrin homology domain-containing family O member 1	1.05	0.19
Aup1	Q3U3K9	Ancient ubiquitous protein 1	1.05	0.30
Canx	P35564	Calnexin	1.05	0.27
Icam1	P13597	Intercellular adhesion molecule 1	1.05	0.36
Top3b	Q9Z321	DNA topoisomerase 3-beta-1	1.05	0.30
Mta2	Q9R190	Metastasis-associated protein MTA2	1.05	0.23
Atp2c1	Q3UZR5	Calcium-transporting ATPase	1.05	0.25
Slc25a45	Q8CFJ7	Solute carrier family 25 member 45	1.05	0.22
Mrpl20	Q9CQL4	39S ribosomal protein L20, mitochondrial	1.05	0.30
Lpcat3	Q91V01	Lysophospholipid acyltransferase 5	1.05	0.20
Gkap1	Q9JMB0	G kinase-anchoring protein 1	1.05	0.53
Stambpl1	Q76N33	AMSH-like protease	1.05	0.27
Eif4g2	F7CBP1	Eukaryotic translation initiation factor 4 gamma 2	1.05	0.51
Rrp15	Q9CYX7	RRP15-like protein	1.05	0.25
Hnrnpc	A0A2I3BRM6	Heterogeneous nuclear ribonucleoproteins C1/C2 (Fragment)	1.05	0.59
Cdc42se1	Q8BHL7	CDC42 small effector protein 1	1.05	0.22
Nme3	Q9WV85	Nucleoside diphosphate kinase 3	1.05	0.13
Zfp36l1	P23950	mRNA decay activator protein ZFP36L1	1.05	0.50
Ktn1	A0A087WS29	Kinectin	1.05	0.32
Dnajc2	P54103	DnaJ homolog subfamily C member 2	1.05	0.20
Slc8a1	G3X9J1	Sodium/calcium exchanger 1	1.05	0.32
Lhfpl2	Q8BGA2	LHFPL tetraspan subfamily member 2 protein	1.05	0.75
Cadm1	E9PYN1	Cell adhesion molecule 1	1.05	0.11
Osbpl8	B9EJ86	Oxysterol-binding protein-related protein 8	1.05	0.17
Chaf1a	Q9QWF0	Chromatin assembly factor 1 subunit A	1.05	0.44
Abcg1	Q64343	ATP-binding cassette sub-family G member 1	1.05	0.15
Stom	P54116	Erythrocyte band 7 integral membrane protein	1.05	0.09
Dnajb12	Q8C4C9	DnaJ (Hsp40) homolog, subfamily B, member 12, isoform CRA_d	1.05	0.31
Ccnd1	P25322	G1/S-specific cyclin-D1	1.05	0.49
Map7d1	A8Y5P4	MAP7 domain-containing protein 1	1.05	0.18
Man1c1	Q6NXK9	alpha-1,2-Mannosidase	1.05	0.26
Tm7sf2	Q71KT5	Delta(14)-sterol reductase	1.05	0.49
Utp3	Q9JI13	Something about silencing protein 10	1.05	0.27
Pttg1ip	Q8R143	Pituitary tumor-transforming gene 1 protein-interacting protein	1.05	0.16
Trnau1ap	Q80VC6	tRNA selenocysteine 1-associated protein 1	1.05	0.88
Fitm2	P59266	Fat storage-inducing transmembrane protein 2	1.05	0.59

Casd1	Q7TN73	N-acetylneuraminate 9-O-acetyltransferase	1.05	0.61
Ahctf1	Q8CJF7	Protein ELYS	1.05	0.40
Atp5mg	Q9CPQ8	ATP synthase subunit g, mitochondrial	1.05	0.28
Rpl23	P62830	60S ribosomal protein L23	1.05	0.18
Mndal	D0QMC3	Myeloid cell nuclear differentiation antigen-like protein	1.05	0.13
Eaf1	Q9D4C5	ELL-associated factor 1	1.05	0.42
Pgap1	Q3UUQ7	GPI inositol-deacylase	1.05	0.28
Gm20509	G3UZF1	Predicted gene 20509 (Fragment)	1.05	0.24
Evi5	F8VPT6	Ecotropic viral integration site 5 protein	1.05	0.59
Cr1l	Q64735	Complement component receptor 1-like protein	1.05	0.19
Ybx3	Q9JKB3	Y-box-binding protein 3	1.05	0.04
Gar1	Q9CY66	H/ACA ribonucleoprotein complex subunit 1	1.05	0.05
Abcb7	Q61102	ATP-binding cassette sub-family B member 7, mitochondrial	1.05	0.03
Kri1	F6WIU1	Protein KRI1 homolog	1.05	0.16
Rasgef1b	A0A1B0GSM5	Ras-GEF domain-containing family member 1B	1.05	0.23
Lclat1	Q3UN02	Lysocardiolipin acyltransferase 1	1.05	0.19
Poldip2	Q91VA6	Polymerase delta-interacting protein 2	1.05	0.25
Rpl7l1	Q9D8M4	60S ribosomal protein L7-like 1	1.05	0.18
Bet1	O35623	BET1 homolog	1.05	0.29
Rbm10	Q99KG3	RNA-binding protein 10	1.05	0.34
Epb41l3	Q9WV92	Band 4.1-like protein 3	1.05	0.21
Lrrc58	Q3UGP9	Leucine-rich repeat-containing protein 58	1.05	0.27
Gna13	P27601	Guanine nucleotide-binding protein subunit alpha-13	1.05	0.04
Spryd7	Q3TFQ1	SPRY domain-containing protein 7	1.05	0.07
Alg10b	Q3UGP8	Putative Dol-P-Glc:Glc(2)Man(9)GlcNAc(2)-PP-Dol alpha-1,2-glucosyltransferase	1.05	0.13
Rb1cc1	Q9ESK9	RB1-inducible coiled-coil protein 1	1.05	0.06
Pdia5	Q921X9	Protein disulfide-isomerase A5	1.05	0.24
Nsa2	Q9CR47	Ribosome biogenesis protein NSA2 homolog	1.05	0.21
Pbk	Q9JJ78	Lymphokine-activated killer T-cell-originated protein kinase	1.05	0.61
Lnpk	Q7TQ95	Endoplasmic reticulum junction formation protein lunapark	1.05	0.24
Erg28	Q9ERY9	Probable ergosterol biosynthetic protein 28	1.05	0.24
Hs6st1	Q9QYK5	Heparan-sulfate 6-O-sulfotransferase 1	1.05	0.36
Gm11361	A0A1Y7VKY1	MCG116671	1.05	0.13
Mnt	O08789	Max-binding protein MNT	1.05	0.28
B3gnt8	Q8R3I9	UDP-GlcNAc:betaGal beta-1,3-N-acetylglucosaminyltransferase 8	1.05	0.11
Gnaq	P21279	Guanine nucleotide-binding protein G(q) subunit alpha	1.05	0.06
Hvcn1	Q3U2S8	Voltage-gated hydrogen channel 1	1.05	0.29
Tomm5	B1AXP6	Mitochondrial import receptor subunit TOM5 homolog	1.05	0.26
Apool	Q78IK4	MICOS complex subunit Mic27	1.05	0.16
Scamp2	Q9ERN0	Secretory carrier-associated membrane protein 2	1.05	0.15
Tm2d2	Q8R0I4	TM2 domain-containing protein 2	1.05	0.66
Arhgap31	A6X8Z5	Rho GTPase-activating protein 31	1.05	0.49
Tma16	Q9CR02	Translation machinery-associated protein 16	1.05	0.53
Rdh11	Q9QYF1	Retinol dehydrogenase 11	1.05	0.20
Fam213a	Q3U125	Redox-regulatory protein FAM213A	1.05	0.41
Ms4a7	E9Q9V5	Membrane-spanning 4-domains, subfamily A, member 7	1.05	0.14
Rfc1	G3UWX1	Replication factor C subunit 1	1.05	0.08
Dnajc1	Q61712	DnaJ homolog subfamily C member 1	1.05	0.33
Yme1l1	O88967	ATP-dependent zinc metalloprotease YME1L1	1.05	0.22
Snip1	Q8BIZ6	Smad nuclear-interacting protein 1	1.05	0.46
Rapgef1	Q3UHC1	Rap guanine nucleotide exchange factor (GEF) 1	1.05	0.43
Map4	P27546	Microtubule-associated protein 4	1.05	0.18
Praf2	Q9JIG8	PRA1 family protein 2	1.05	0.17
Mtfmt	Q9D799	Methionyl-tRNA formyltransferase, mitochondrial	1.05	0.02
Gngt2	Q61017	Guanine nucleotide-binding protein G(I)/G(S)/G(O) subunit gamma-T2	1.05	0.13
Tpra1	Q99MU1	Transmembrane protein adipocyte-associated 1	1.05	0.04
Lsm5	P62322	U6 snRNA-associated Sm-like protein LSm5	1.05	0.62
Snx11	Q91WL6	Sorting nexin-11	1.05	0.76
Tpst2	Q3TQN1	Protein-tyrosine sulfotransferase	1.05	0.16
Mov10	D3YVL0	Putative helicase MOV-10	1.05	0.24
Galnt6	Q8C7U7	Polypeptide N-acetylgalactosaminyltransferase 6	1.05	0.38
Pag1	Q3U1F9	Phosphoprotein associated with glycosphingolipid-enriched microdomains 1	1.05	0.63
Tada3	Q8R0L9	Transcriptional adapter 3	1.05	0.48
Slc39a14	A0A0R4J1V1	Solute carrier family 39 (Zinc transporter), member 14, isoform CRA_a	1.05	0.75
Piezo1	E2JF22	Piezo-type mechanosensitive ion channel component 1	1.05	0.19
Use1	E9Q496	Vesicle transport protein USE1	1.05	0.16
Atad3	Q925I1	ATPase family AAA domain-containing protein 3	1.05	0.17
Ahnak2	F7CVJ5	AHNAK nucleoprotein 2 (Fragment)	1.05	0.13
Dnajc16	Q80TN4	DnaJ homolog subfamily C member 16	1.05	0.11
Tsc1	Q9EP53	Hamartin	1.05	0.30
Ccdc47	Q9D024	Coiled-coil domain-containing protein 47	1.05	0.11
Mettl3	A0A0R4J041	N6-adenosine-methyltransferase subunit METTL3	1.05	0.31
Trappc5	Q9CQA1	Trafficking protein particle complex subunit 5	1.05	0.08

Zdhhc20	Q5Y5T1	Palmitoyltransferase ZDHHC20	1.05	0.21
Maoa	Q64133	Amine oxidase [flavin-containing] A	1.05	0.27
Lemd2	Q6DVA0	LEM domain-containing protein 2	1.05	0.21
Mapk9	Q9WTU6	Mitogen-activated protein kinase 9	1.05	0.33
Hm13	Q9D8V0	Minor histocompatibility antigen H13	1.05	0.16
Tm9sf1	Q9DBU0	Transmembrane 9 superfamily member 1	1.05	0.38
Hilpda	Q9JLS0	Hypoxia-inducible lipid droplet-associated protein	1.05	0.17
Rell1	Q8K2J7	RELT-like protein 1	1.05	0.18
Tmem33	Q9CR67	Transmembrane protein 33	1.05	0.22
Mknk1	O08605	MAP kinase-interacting serine/threonine-protein kinase 1	1.05	0.04
Plxnc1	Q9QZC2	Plexin-C1	1.05	0.44
Tmem30a	Q8VEK0	Cell cycle control protein 50A	1.05	0.20
Tmem165	P52875	Transmembrane protein 165	1.05	0.35
Pex12	Q8VC48	Peroxisome assembly protein 12	1.05	0.64
Rps13	P62301	40S ribosomal protein S13	1.05	0.20
Focad	A2AKG8	Focadhesin	1.05	0.38
Tmem43	Q9DBS1	Transmembrane protein 43	1.05	0.23
Mcur1	Q9CXD6	Mitochondrial calcium uniporter regulator 1	1.05	0.07
Pds5b	Q4VA53	Sister chromatid cohesion protein PDS5 homolog B	1.05	0.17
Nktr	P30415	NK-tumor recognition protein	1.05	0.09
Mmp9	P41245	Matrix metalloproteinase-9	1.05	0.50
Cln8	Q9QUK3	Protein CLN8	1.05	0.29
Exosc10	P56960	Exosome component 10	1.05	0.10
Nsmce1	A0A0R4J0C0	Non-SMC element 1 homolog (S. cerevisiae), isoform CRA_b	1.05	0.40
Slc38a10	J3QNE8	Putative sodium-coupled neutral amino acid transporter 10	1.05	0.35
Hsd17b7	O88736	3-keto-steroid reductase	1.05	0.10
Smim12	Q78RX3	Small integral membrane protein 12	1.05	0.31
Fbxo21	Q8VDH1	F-box only protein 21	1.05	0.24
Mrpl51	Q9CPY1	39S ribosomal protein L51, mitochondrial	1.05	0.32
Ndufa13	Q9ERS2	NADH dehydrogenase [ubiquinone] 1 alpha subcomplex subunit 13	1.05	0.35
Tmem129	Q8K304	E3 ubiquitin-protein ligase TM129	1.05	0.23
Rbm28	Q8CGC6	RNA-binding protein 28	1.05	0.21
Slc25a46	Q9CQS4	Solute carrier family 25 member 46	1.05	0.23
E2f4	Q8R0K9	Transcription factor E2F4	1.05	0.69
Vkorc1l1	Q6TEK5	Vitamin K epoxide reductase complex subunit 1-like protein 1	1.05	0.23
Sp1	O89090	Transcription factor Sp1	1.05	0.25
Akap13	A0A140LJJ5	A-kinase anchor protein 13	1.05	0.19
Slc35f6	Q8VE96	Solute carrier family 35 member F6	1.05	0.37
Vamp8	A0A0R4J0R1	Vesicle-associated membrane protein 8	1.05	0.77
Gpsm1	Q6IR34	G-protein-signaling modulator 1	1.05	0.14
Serbp1	Q9CY58	Plasminogen activator inhibitor 1 RNA-binding protein	1.05	0.21
Hps6	Q8BLY7	Hermansky-Pudlak syndrome 6 protein homolog	1.05	0.48
Rpusd4	Q9CWX4	Mitochondrial RNA pseudouridine synthase Rpusd4	1.05	0.50
Tox4	Q8BU11	TOX high mobility group box family member 4	1.05	0.24
Matk	A0A0R4J1N6	Tyrosine-protein kinase	1.05	0.16
Dag1	Q62165	Dystroglycan	1.05	0.59
Scarf1	Q5ND28	Scavenger receptor class F member 1	1.05	0.50
Tram1	Q91V04	Translocating chain-associated membrane protein 1	1.05	0.20
Dennd5a	Q6PAL8	DENN domain-containing protein 5A	1.05	0.10
Dctn5	Q9QZB9	Dynactin subunit 5	1.05	0.07
Amfr	Q9R049	E3 ubiquitin-protein ligase AMFR	1.05	0.55
S100a1	P56565	Protein S100-A1	1.05	0.07
Mmaa	Q8C7H1	Methylmalonic aciduria type A homolog, mitochondrial	1.05	0.52
Pno1	Q9CPS7	RNA-binding protein PNO1	1.05	0.50
Mrpl34	Q99N91	39S ribosomal protein L34, mitochondrial	1.05	0.38
Gnl3l	Q6PGG6	Guanine nucleotide-binding protein-like 3-like protein	1.05	0.61
Mea1	Q64327	Male-enhanced antigen 1	1.05	0.08
Morf4l1	P60762	Mortality factor 4-like protein 1	1.05	0.16
Vti1a	O89116	Vesicle transport through interaction with t-SNAREs homolog 1A	1.05	0.08
Lrrc59	Q922Q8	Leucine-rich repeat-containing protein 59	1.05	0.19
Cd300ld4	A2A7W1	CD300 molecule-like family member D4	1.05	0.13
Pptc7	Q6NVE9	Protein phosphatase PTC7 homolog	1.05	0.78
Tspo	P50637	Translocator protein	1.05	0.81
Abcd3	P55096	ATP-binding cassette sub-family D member 3	1.05	0.26
Slc6a6	O35316	Sodium- and chloride-dependent taurine transporter	1.05	0.14
Naa16	Q9DBB4	N-alpha-acetyltransferase 16, NatA auxiliary subunit	1.05	0.34
Bcap31	Q61335	B-cell receptor-associated protein 31	1.05	0.27
Traf3	Q60803	TNF receptor-associated factor 3	1.05	0.21
Creld1	Q91XD7	Cysteine-rich with EGF-like domain protein 1	1.05	0.37
Zdhhc14	Q8BQQ1	Probable palmitoyltransferase ZDHHC14	1.05	0.42
Glipr1	Q9CWG1	Glioma pathogenesis-related protein 1	1.05	0.17
Nptn	P97300	Neuroplastin	1.05	0.10
Clptm1l	Q8BXA5	Cleft lip and palate transmembrane protein 1-like protein	1.05	0.17

Sp3	O70494	Transcription factor Sp3	1.05	0.14
Diablo	Q9JIQ3	Diablo homolog, mitochondrial	1.05	0.16
Prrc2a	Q7TSC1	Protein PRRC2A	1.05	0.36
Pyroxd1	Q3TMV7	Pyridine nucleotide-disulfide oxidoreductase domain-containing protein 1	1.05	0.75
Ghitm	Q91VC9	Growth hormone-inducible transmembrane protein	1.05	0.18
Col6a1	Q04857	Collagen alpha-1(VI) chain	1.05	0.24
Kct2	Q8K201	Keratinocyte-associated transmembrane protein 2	1.05	0.20
Dpy19l1	A6X919	Probable C-mannosyltransferase DPY19L1	1.05	0.01
Tmem214	Q8BM55	Transmembrane protein 214	1.05	0.06
Pet100	P0DJE0	Protein PET100 homolog, mitochondrial	1.05	0.17
Wdr46	Q9Z0H1	WD repeat-containing protein 46	1.05	0.59
Icam2	P35330	Intercellular adhesion molecule 2	1.05	0.60
Hltf	Q6PCN7	Helicase-like transcription factor	1.05	0.28
Rtn4	Q99P72	Reticulon-4	1.05	0.45
Arrdc1	Q99KN1	Arrestin domain-containing protein 1	1.05	0.27
Acy1	A0A0R4J050	Aminoacylase-1	1.05	0.35
Noc2l	J3QK52	Nucleolar complex protein 2 homolog	1.05	0.21
Poldip3	Q8BG81	Polymerase delta-interacting protein 3	1.05	0.16
Nmnat1	Q9EPA7	Nicotinamide/nicotinic acid mononucleotide adenylyltransferase 1	1.05	0.42
Tmem126b	Q9D1R1	Complex I assembly factor TMEM126B, mitochondrial	1.05	0.17
Rps6	P62754	40S ribosomal protein S6	1.05	0.11
Hdhd5	Q91WM2	Haloacid dehalogenase-like hydrolase domain-containing 5	1.05	0.25
Mrps18a	Q5U5I3	28S ribosomal protein S18a, mitochondrial	1.05	0.13
Pign	G3X9F1	GPI ethanolamine phosphate transferase 1	1.05	0.13
Gm56451	A0A338P6I9	Predicted gene, 56451	1.05	0.45
Fdx1	P46656	Adrenodoxin, mitochondrial	1.05	0.08
Numa1	F6ZQA3	Nuclear mitotic apparatus protein 1 (Fragment)	1.05	0.35
Dcaf7	P61963	DDB1- and CUL4-associated factor 7	1.05	0.17
Cirbp	P60824	Cold-inducible RNA-binding protein	1.05	0.27
Nsd3	D3Z357	Histone-lysine N-methyltransferase NSD3	1.05	0.16
Cdc37l1	Q9CZP7	Hsp90 co-chaperone Cdc37-like 1	1.05	0.19
Selenoo	Q9DBC0	Selenoprotein O	1.05	0.07
Utp11	Q9CZJ1	Probable U3 small nucleolar RNA-associated protein 11	1.05	0.20
Larp7	Q05CL8	La-related protein 7	1.05	0.12
Rps25	P62852	40S ribosomal protein S25	1.05	0.12
Nars2	Q8BGV0	Probable asparagine--tRNA ligase, mitochondrial	1.05	0.48
Fam105a	Q3TVP5	Inactive ubiquitin thioesterase FAM105A	1.05	0.08
Dnmt1	P13864	DNA (cytosine-5)-methyltransferase 1	1.05	0.16
Mboat7	Q8CHK3	Lysophospholipid acyltransferase 7	1.05	0.14
Lzic	Q8K3C3	Protein LZIC	1.05	0.03
Clcc1	A2AEM2	Chloride channel CLIC-like 1, isoform CRA_a	1.05	0.14
Gtpbp1	O08582	GTP-binding protein 1	1.05	0.17
Sun1	F6RMJ1	SUN domain-containing protein 1 (Fragment)	1.05	0.41
Nusap1	Q9ERH4	Nucleolar and spindle-associated protein 1	1.05	0.19
Ahsa2	Q8N9S3	Activator of 90 kDa heat shock protein ATPase homolog 2	1.05	0.39
Bcl2l13	P59017	Bcl-2-like protein 13	1.05	0.14
Lrrfip1	A0A087WPT0	Leucine-rich repeat flightless-interacting protein 1 (Fragment)	1.05	0.56
Itgb3	O54890	Integrin beta-3	1.05	0.74
Gstm4	Q8R5I6	Glutathione S-transferase mu 4	1.05	0.33
Rab2b	P59279	Ras-related protein Rab-2B	1.05	0.23
Gm45837	F7D3W5	Phosphodiesterase	1.05	0.27
Rtl8b	Q9D1F0	CAAX box 1 homolog A (Human)	1.05	0.50
Atp11a	P98197	Probable phospholipid-transporting ATPase IH	1.06	0.10
Tmem109	Q3UBX0	Transmembrane protein 109	1.06	0.11
Ramac	Q9CQY2	RNA guanine-N7 methyltransferase activating subunit	1.06	0.30
Dhrs7	Q9CXR1	Dehydrogenase/reductase SDR family member 7	1.06	0.19
Cdk13	Q69ZA1	Cyclin-dependent kinase 13	1.06	0.33
Agpat3	Q9D517	1-acyl-sn-glycerol-3-phosphate acyltransferase gamma	1.06	0.24
Stk11	Q9WTK7	Serine/threonine-protein kinase STK11	1.06	0.56
Prdx4	O08807	Peroxiredoxin-4	1.06	0.65
Sacm1l	Q9EP69	Phosphatidylinositide phosphatase SAC1	1.06	0.05
Arl6ip5	Q8R5J9	PRA1 family protein 3	1.06	0.20
Aldh3a2	B1ATI0	Aldehyde dehydrogenase	1.06	0.06
Znf281	Q99LI5	Zinc finger protein 281	1.06	0.11
Alg5	Q9DB25	Dolichyl-phosphate beta-glucosyltransferase	1.06	0.19
Mfsd8	Q8BH31	Major facilitator superfamily domain-containing protein 8	1.06	0.50
Nme1	P15532	Nucleoside diphosphate kinase A	1.06	0.75
Mpv17	G3UVW1	Mpv17 transgene, kidney disease mutant, isoform CRA_b	1.06	0.09
Vti1b	Q91XH6	Vesicle transport through interaction with t-SNAREs 1B homolog	1.06	0.14
Zfc3h1	B2RT41	Zinc finger, C3H1-type-containing	1.06	0.28
Kif11	Q6P9P6	Kinesin-like protein KIF11	1.06	0.25
Zdhhc3	Q8R173	Palmitoyltransferase ZDHHC3	1.06	0.25
Wdr62	E9QK36	WD repeat-containing protein 62	1.06	0.17

Hint3	Q9CPS6	Histidine triad nucleotide-binding protein 3	1.06	0.59
Galnt4	O08832	Polypeptide N-acetylgalactosaminyltransferase 4	1.06	0.23
Sepsecs	Q6P6M7	O-phosphoseryl-tRNA(Sec) selenium transferase	1.06	0.37
4930433I11Rik	A0A0U1RPT6	RIKEN cDNA 4930433I11 gene	1.06	0.27
Atp2a3	Q64518	Sarcoplasmic/endoplasmic reticulum calcium ATPase 3	1.06	0.25
Psmd4	F7AI87	26S proteasome non-ATPase regulatory subunit 4 (Fragment)	1.06	0.41
Aurkb	O70126	Aurora kinase B	1.06	0.26
Fut11	Q8BHC9	Alpha-(1,3)-fucosyltransferase 11	1.06	0.25
Rer1	Q9CQU3	Protein RER1	1.06	0.09
Cers2	Q924Z4	Ceramide synthase 2	1.06	0.33
Vps37c	Q8R105	Vacuolar protein sorting-associated protein 37C	1.06	0.59
Mgat5	Q8R4G6	Alpha-1,6-mannosylglycoprotein 6-beta-N-acetylglucosaminyltransferase A	1.06	0.12
Antxr2	Q6DFX2	Anthrax toxin receptor 2	1.06	0.04
Dhrs3	O88876	Short-chain dehydrogenase/reductase 3	1.06	0.02
Cisd2	Q9CQB5	CDGSH iron-sulfur domain-containing protein 2	1.06	0.16
Gng2	P63213	Guanine nucleotide-binding protein G(I)/G(S)/G(O) subunit gamma-2	1.06	0.15
Prpf3	Q922U1	U4/U6 small nuclear ribonucleoprotein Prp3	1.06	0.13
Cdca3	Q99M54	Cell division cycle-associated protein 3	1.06	0.36
Slc25a31	Q3V132	ADP/ATP translocase 4	1.06	0.27
Zfx	P17012	Zinc finger X-chromosomal protein	1.06	0.08
Coa3	Q9D2R6	Cytochrome c oxidase assembly factor 3 homolog, mitochondrial	1.06	0.08
Sun1	Q9D666	SUN domain-containing protein 1	1.06	0.21
Mrpl54	Q9CPW3	39S ribosomal protein L54, mitochondrial	1.06	0.12
Afg3l2	Q8JZQ2	AFG3-like protein 2	1.06	0.08
Tmem205	Q91XE8	Transmembrane protein 205	1.06	0.13
Pes1	Q5SQ20	Pescadillo homolog	1.06	0.01
Pros1	Q08761	Vitamin K-dependent protein S	1.06	0.16
Lilrb4	Q64281	Leukocyte immunoglobulin-like receptor subfamily B member 4	1.06	0.14
Slc16a7	O70451	Monocarboxylate transporter 2	1.06	0.11
Mcl1	P97287	Induced myeloid leukemia cell differentiation protein Mcl-1 homolog	1.06	0.03
Plau	P06869	Urokinase-type plasminogen activator	1.06	0.50
Tmcc1	F8WJ98	Transmembrane and coiled-coil domains protein 1 (Fragment)	1.06	0.23
Rrp12	Q6P5B0	RRP12-like protein	1.06	0.11
Csrp2	A0A1W2P845	Cysteine and glycine-rich protein 2	1.06	0.07
2310022A10Rik	G5E8E3	RIKEN cDNA 2310022A10 gene	1.06	0.23
Sec61a1	P61620	Protein transport protein Sec61 subunit alpha isoform 1	1.06	0.17
Kdsr	Q6GV12	3-ketodihydrosphingosine reductase	1.06	0.33
Bptf	A2A654	Bromodomain PHD finger transcription factor	1.06	0.20
Kras	P32883	GTPase KRas	1.06	0.54
Tnc	Q80YX1	Tenascin	1.06	0.37
Zfyve27	Q3TXX3	Protrudin	1.06	0.01
Vrk2	Q8BN21	Serine/threonine-protein kinase VRK2	1.06	0.13
Kpna2	P52293	Importin subunit alpha-1	1.06	0.11
Prc1	G3UWQ7	Protein regulator of cytokinesis 1	1.06	0.15
Rnf123	Q5XPI3	E3 ubiquitin-protein ligase RNF123	1.06	0.30
Cmip	Q9D486	C-Maf-inducing protein	1.06	0.11
Tapbpl	Q8VD31	Tapasin-related protein	1.06	0.22
Timm10b	Q9WV96	Mitochondrial import inner membrane translocase subunit Tim10 B	1.06	0.28
Pdcd11	Q6NS46	Protein RRP5 homolog	1.06	0.12
Tmem65	Q4VAE3	Transmembrane protein 65	1.06	0.28
Camsap1	A0A0A0MQE5	Calmodulin-regulated spectrin-associated protein 1	1.06	0.33
Gpr180	Q8BPS4	Integral membrane protein GPR180	1.06	0.36
Pdzd8	B9EJ80	PDZ domain-containing protein 8	1.06	0.25
Utp20	E9QK83	Small subunit processome component 20 homolog	1.06	0.20
Agtpbp1	Q641K1	Cytosolic carboxypeptidase 1	1.06	0.36
Mphosph8	Q3TYA6	M-phase phosphoprotein 8	1.06	0.63
Lpl	P11152	Lipoprotein lipase	1.06	0.17
Rcn3	Q8BH97	Reticulocalbin-3	1.06	0.09
Mrps16	Q9CPX7	28S ribosomal protein S16, mitochondrial	1.06	0.16
Sun2	Q8BJS4	SUN domain-containing protein 2	1.06	0.23
Spcs1	A0A2I3BRW0	Signal peptidase complex subunit 1	1.06	0.21
D17h6s53e	Q9Z1R4	Uncharacterized protein C6orf47 homolog	1.06	0.13
Cd5l	Q9QWK4	CD5 antigen-like	1.06	0.20
Csf2ra	Q00941	Granulocyte-macrophage colony-stimulating factor receptor subunit alpha	1.06	0.16
Tmem19	Q91W52	Transmembrane protein 19	1.06	0.27
Zfand2a	Q9JII7	AN1-type zinc finger protein 2A	1.06	0.46
Dcp1b	B9EIX0	DCP1 decapping enzyme homolog b (S. cerevisiae)	1.06	0.43
Chek1	O35280	Serine/threonine-protein kinase Chk1	1.06	0.52
Atxn1l	P0C7T6	Ataxin-1-like	1.06	0.20
Slc25a25	A2ASZ8	Calcium-binding mitochondrial carrier protein SCaMC-2	1.06	0.29
Col6a2	Q02788	Collagen alpha-2(VI) chain	1.06	0.32
Nemp1	Q6ZQE4	Nuclear envelope integral membrane protein 1	1.06	0.19
Cd37	Q3U429	Tetraspanin	1.06	0.12

Nom1	Q3UFM5	Nucleolar MIF4G domain-containing protein 1	1.06	0.66
Stx3	Q64704	Syntaxin-3	1.06	0.07
Slc25a16	Q8C0K5	Graves disease carrier protein homolog	1.06	0.43
Uap1	Q3UHZ7	UDP-N-acetylhexosamine pyrophosphorylase	1.06	0.21
Sccpdh	Q8R127	Saccharopine dehydrogenase-like oxidoreductase	1.06	0.09
Ncs1	Q8BNY6	Neuronal calcium sensor 1	1.06	0.59
Tacc2	E9Q8T1	Transforming acidic coiled-coil-containing protein 2	1.06	0.78
Pex13	Q9D0K1	Peroxisomal membrane protein PEX13	1.06	0.21
Rps23	P62267	40S ribosomal protein S23	1.06	0.12
Ggcx	Q9QYC7	Vitamin K-dependent gamma-carboxylase	1.06	0.13
Fundc2	Q9D6K8	FUN14 domain-containing protein 2	1.06	0.09
Arl6ip6	Q8BH07	ADP-ribosylation factor-like protein 6-interacting protein 6	1.06	0.42
Tomm40l	Q9CZR3	Mitochondrial import receptor subunit TOM40B	1.06	0.10
Rap1a	P62835	Ras-related protein Rap-1A	1.06	0.01
Mrps11	Q3U8Y1	28S ribosomal protein S11, mitochondrial	1.06	0.13
Mt-Cyb	P00158	Cytochrome b	1.06	0.06
Med21	Q9CQ39	Mediator of RNA polymerase II transcription subunit 21	1.06	0.57
Myo1f	Q8CG29	Myosin IF	1.06	0.06
Slc30a7	Q9JKN1	Zinc transporter 7	1.06	0.13
Sema4d	O09126	Semaphorin-4D	1.06	0.13
Lmbrd2	Q8C561	LMBR1 domain-containing protein 2	1.06	0.10
Aldoart2	A6ZI47	Fructose-bisphosphate aldolase	1.06	0.08
Emc6	Q9CQW0	ER membrane protein complex subunit 6	1.06	0.02
Rps8	P62242	40S ribosomal protein S8	1.06	0.18
Ccr1	P51675	C-C chemokine receptor type 1	1.06	0.76
Mp68	P56379	6.8 kDa mitochondrial proteolipid	1.06	0.29
Zdhhc6	Q9CPV7	Palmitoyltransferase ZDHHC6	1.06	0.04
Nrf1	Q99K73	Nrf1 protein	1.06	0.36
Rab4a	P56371	Ras-related protein Rab-4A	1.06	0.25
Abhd16a	Q9Z1Q2	Protein ABHD16A	1.06	0.43
Setd1a	E9PYH6	Histone-lysine N-methyltransferase SETD1A	1.06	0.31
Nemf	Q8CCP0	Nuclear export mediator factor Nemf	1.06	0.08
Ssr3	Q9DCF9	Translocon-associated protein subunit gamma	1.06	0.22
Bmt2	Q8BXK4	S-adenosylmethionine sensor upstream of mTORC1	1.06	0.24
Rpl10	Q6ZWV3	60S ribosomal protein L10	1.06	0.15
Ammecr1	Q9JHT5	AMME syndrome candidate gene 1 protein homolog	1.06	0.04
Fabp3	P11404	Fatty acid-binding protein, heart	1.06	0.01
Tmco1	A0A0A6YVS2	Calcium load-activated calcium channel (Fragment)	1.06	0.51
Rpl35a	O55142	60S ribosomal protein L35a	1.06	0.11
Mief1	Q8BGV8	Mitochondrial dynamics protein MID51	1.06	0.11
Rrbp1	A2AVJ7	Ribosome-binding protein 1	1.06	0.11
Snx20	A0A0R4J0D0	Sorting nexin-20	1.06	0.16
Tgoln1	Q62313	Trans-Golgi network integral membrane protein 1	1.06	0.11
Rpl18a	P62717	60S ribosomal protein L18a	1.06	0.14
Atf3	Q60765	Cyclic AMP-dependent transcription factor ATF-3	1.06	0.03
Nop2	E9QN31	Probable 28S rRNA (cytosine-C(5))-methyltransferase	1.06	0.17
Pip4p2	Q9CZX7	Type 2 phosphatidylinositol 4,5-bisphosphate 4-phosphatase	1.06	0.26
Jagn1	Q5XKN4	Protein jagunal homolog 1	1.06	0.04
Tpm1	E9Q454	Tropomyosin alpha-1 chain	1.06	0.22
Mfap2	Q99PM0	Microfibril-associated glycoprotein 1	1.06	0.32
Cisd1	Q91WS0	CDGSH iron-sulfur domain-containing protein 1	1.06	0.11
Slc39a7	Q31125	Zinc transporter SLC39A7	1.06	0.21
Pfkfb2	A0A087WRM7	6-phosphofructo-2-kinase/fructose-2,6-bisphosphatase 2	1.06	0.24
Polr3k	Q9CQZ7	DNA-directed RNA polymerase III subunit RPC10	1.06	0.35
Itm2b	O89051	Integral membrane protein 2B	1.06	0.07
Adnp2	Q8CHC8	Activity-dependent neuroprotector homeobox protein 2	1.06	0.47
Adgre1	Q61549	Adhesion G protein-coupled receptor E1	1.06	0.00
Nrp2	O35375	Neuropilin-2	1.06	0.13
Senp8	A0A1L1SRH8	Sentrin-specific protease 8	1.06	0.10
Usp9x	G3UY52	Probable ubiquitin carboxyl-terminal hydrolase FAF-X (Fragment)	1.06	0.36
Ccdc51	Q3URS9	Coiled-coil domain-containing protein 51	1.06	0.20
Spast	A0A286YE25	Spastin	1.06	0.26
Slc35c1	Q8BLX4	GDP-fucose transporter 1	1.06	0.14
Rpl36a	P83882	60S ribosomal protein L36a	1.07	0.08
S100a9	P31725	Protein S100-A9	1.07	0.50
P4ha2	Q5SX75	Procollagen-proline, 2-oxoglutarate 4-dioxygenase (Proline 4-hydroxylase), alpha II polypeptide, isoform CRA_f	1.07	0.12
Nadk	P58058	NAD kinase	1.07	0.15
Alg12	Q8VDB2	Dol-P-Man:Man(7)GlcNAc(2)-PP-Dol alpha-1,6-mannosyltransferase	1.07	0.47
Znf512	Q69Z99	Zinc finger protein 512	1.07	0.08
Txndc11	Q8K2W3	Thioredoxin domain-containing protein 11	1.07	0.30
Plxdc1	Q91ZV7	Plexin domain-containing protein 1	1.07	0.67
Tmed1	Q3V009	Transmembrane emp24 domain-containing protein 1	1.07	0.04
Sfxn5	Q925N0	Sideroflexin-5	1.07	0.17

Kiaa0100	Q5SYL3	Protein KIAA0100	1.07	0.14
Cenpc	P49452	Centromere protein C	1.07	0.25
Mrpl48	E9QPQ8	39S ribosomal protein L48, mitochondrial	1.07	0.00
Borcs7	Q9CRC6	BLOC-1-related complex subunit 7	1.07	0.25
Bcap29	Q61334	B-cell receptor-associated protein 29	1.07	0.11
Fchsd2	Q3USJ8	F-BAR and double SH3 domains protein 2	1.07	0.28
Zc2hc1a	Q8BJH1	Zinc finger C2HC domain-containing protein 1A	1.07	0.29
Akap1	O08715	A-kinase anchor protein 1, mitochondrial	1.07	0.03
Romo1	P60603	Reactive oxygen species modulator 1	1.07	0.08
Rabac1	Q9Z0S9	Prenylated Rab acceptor protein 1	1.07	0.35
Dmac1	Q9CQ00	Distal membrane-arm assembly complex protein 1	1.07	0.07
Dennd5b	A2RSQ0	DENN domain-containing protein 5B	1.07	0.47
Pex14	Q9R0A0	Peroxisomal membrane protein PEX14	1.07	0.12
Fam84b	D3YXJ5	Family with sequence similarity 84, member B	1.07	0.56
Rab39a	Q8BHD0	Ras-related protein Rab-39A	1.07	0.47
H2afy	Q9QZQ8	Core histone macro-H2A.1	1.07	0.02
Mafg	O54790	Transcription factor MafG	1.07	0.04
Eloa	Q8CB77	Elongin-A	1.07	0.19
Gtpbp4	Q99ME9	Nucleolar GTP-binding protein 1	1.07	0.08
Tpm2	A2AIM4	Tropomyosin beta chain	1.07	0.02
Msmo1	Q9CRA4	Methylsterol monooxygenase 1	1.07	0.07
Tnfrsf22	Q9ER62	Tumor necrosis factor receptor superfamily member 22	1.07	0.11
Clec12a	A0A0R4J0T2	C-type lectin domain family 12 member A	1.07	0.04
Rpl13	P47963	60S ribosomal protein L13	1.07	0.05
Dad1	P61804	Dolichyl-diphosphooligosaccharide--protein glycosyltransferase subunit DAD1	1.07	0.08
Bet1l	A0A1B0GR52	BET1-like protein (Fragment)	1.07	0.00
Septin7	E9Q1G8	Septin-7	1.07	0.10
Rilp	Q5ND29	Rab-interacting lysosomal protein	1.07	0.13
Nrp1	P97333	Neuropilin-1	1.07	0.09
Top2a	Q01320	DNA topoisomerase 2-alpha	1.07	0.08
Jtb	O88824	Protein JTB	1.07	0.32
Pop1	Q8K205	Processing of 1, ribonuclease P/MRP family, (S. cerevisiae)	1.07	0.15
Heatr1	G3X9B1	HEAT repeat-containing 1	1.07	0.08
Ociad2	Q9D8W7	OCIA domain-containing protein 2	1.07	0.11
Rgs19	B7ZCT1	Regulator of G-protein-signaling 19	1.07	0.04
Hmga2	P52927	High mobility group protein HMGI-C	1.07	0.53
Plxnd1	Q3UH93	Plexin-D1	1.07	0.15
Slamf7	A0A0R4J2D6	SLAM family member 7	1.07	0.06
Wls	Q6DID7	Protein wntless homolog	1.07	0.06
Golm1	Q91XA2	Golgi membrane protein 1	1.07	0.26
Katnbl1	Q9CWJ3	KATNB1-like protein 1	1.07	0.22
Sec11c	Q9D8V7	Signal peptidase complex catalytic subunit SEC11C	1.07	0.09
Samsn1	P57725	SAM domain-containing protein SAMSN-1	1.07	0.24
Itsn2	A0A1W2P775	Intersectin-2 (Fragment)	1.07	0.19
Stx18	Q8VDS8	Syntaxin-18	1.07	0.09
Plekhm3	Q8BM47	Pleckstrin homology domain-containing family M member 3	1.07	0.30
Mrpl2	Q9D773	39S ribosomal protein L2, mitochondrial	1.07	0.16
Pde4dip	G3X9L9	Myomegalin	1.07	0.48
Ints6l	Q8BND4	Integrator complex subunit 6-like	1.07	0.59
Stau2	Q8CJ67	Double-stranded RNA-binding protein Staufen homolog 2	1.07	0.11
Dhcr7	A0A140LIT2	7-dehydrocholesterol reductase	1.07	0.11
Plgrkt	Q9D3P8	Plasminogen receptor (KT)	1.07	0.09
Tmx1	Q8VBT0	Thioredoxin-related transmembrane protein 1	1.07	0.08
Ctnnbip1	Q9JJN6	Beta-catenin-interacting protein 1	1.07	0.53
Rft1	Q8C3B8	Protein RFT1 homolog	1.07	0.32
Topbp1	Q6ZQF0	DNA topoisomerase 2-binding protein 1	1.07	0.45
Smad3	Q8BUN5	Mothers against decapentaplegic homolog 3	1.07	0.38
Ltbp3	F8VQ06	Latent-transforming growth factor beta-binding protein 3	1.07	0.59
Fkbp10	Q61576	Peptidyl-prolyl cis-trans isomerase FKBP10	1.07	0.27
Rexo4	Q6PAQ4	RNA exonuclease 4	1.07	0.26
Slc50a1	Q9CXK4	Sugar transporter SWEET1	1.07	0.13
Lair1	Q8BG84	Leukocyte-associated immunoglobulin-like receptor 1	1.07	0.14
Slc25a10	Q9QZD8	Mitochondrial dicarboxylate carrier	1.07	0.00
Rps11	P62281	40S ribosomal protein S11	1.07	0.07
Acot6	Q32Q92	Acyl-coenzyme A thioesterase 6	1.07	0.82
Prelid1	Q8R107	PRELI domain-containing protein 1, mitochondrial	1.07	0.17
Fam241a	Q9CZL2	Uncharacterized protein FAM241A	1.07	0.32
Tmem222	Q8BVA2	Transmembrane protein 222	1.07	0.04
Lamc1	F8VQJ3	Laminin subunit gamma-1	1.07	0.49
Dmap1	Q9JI44	DNA methyltransferase 1-associated protein 1	1.07	0.19
Chchd4	Q8VEA4	Mitochondrial intermembrane space import and assembly protein 40	1.07	0.08
Slc2a1	P17809	Solute carrier family 2, facilitated glucose transporter member 1	1.07	0.21
Fam83f	A0A0R4J033	Expressed sequence AW544981	1.07	0.26

Rnf128	Q9D304	E3 ubiquitin-protein ligase RNF128	1.07	0.39
Oma1	Q9D8H7	Metalloendopeptidase OMA1, mitochondrial	1.07	0.11
Rpl24	Q8BP67	60S ribosomal protein L24	1.07	0.07
Tnip1	D3Z2W0	TNFAIP3-interacting protein 1	1.07	0.19
Cnnm4	Q69ZF7	Metal transporter CNNM4	1.07	0.25
Ormdl2	Q9CQZ0	ORM1-like protein 2	1.07	0.07
Tmem245	D3YWD3	Transmembrane protein 245	1.07	0.05
Tmem209	Q8BRG8	Transmembrane protein 209	1.07	0.19
Smg7	Q5RJH6	Protein SMG7	1.07	0.44
Mybbp1a	Q7TPV4	Myb-binding protein 1A	1.07	0.03
Mki67	E9PVX6	Proliferation marker protein Ki-67	1.07	0.28
Mrm3	Q5ND52	rRNA methyltransferase 3, mitochondrial	1.08	0.51
Znf800	Q0VEE6	Zinc finger protein 800	1.08	0.11
Tbc1d7	Q9D0K0	TBC1 domain family member 7	1.08	0.56
Derl2	Q8BNI4	Derlin-2	1.08	0.02
Rpl15	Q9CZM2	60S ribosomal protein L15	1.08	0.10
Clptm1	Q8VBZ3	Cleft lip and palate transmembrane protein 1 homolog	1.08	0.08
Zcchc17	Q9ESX4	Nucleolar protein of 40 kDa	1.08	0.21
Fbn1	Q61554	Fibrillin-1	1.08	0.16
Ndufb7	Q9CR61	NADH dehydrogenase [ubiquinone] 1 beta subcomplex subunit 7	1.08	0.01
Mecp2	Q9Z2D6	Methyl-CpG-binding protein 2	1.08	0.08
Nfxl1	E9Q8I7	Nuclear transcription factor, X-box-binding-like 1	1.08	0.20
Capzb	P47757	F-actin-capping protein subunit beta	1.08	0.13
Rprd2	A0A0G2JFN1	Regulation of nuclear pre-mRNA domain-containing protein 2	1.08	0.35
Nudt7	Q99P30	Peroxisomal coenzyme A diphosphatase NUDT7	1.08	0.08
S100a8	P27005	Protein S100-A8	1.08	0.32
Ccnt1	Q9QWV9	Cyclin-T1	1.08	0.02
Eif2b1	Q99LC8	Translation initiation factor eIF-2B subunit alpha	1.08	0.04
Rpl21	O09167	60S ribosomal protein L21	1.08	0.03
Map4k4	B2RUE8	Map4k4 protein	1.08	0.13
Filip1l	E0CYH7	Filamin A-interacting protein 1-like	1.08	0.23
Flrt2	Q8BLU0	Leucine-rich repeat transmembrane protein FLRT2	1.08	0.04
Dgat1	Q9Z2A7	Diacylglycerol O-acyltransferase 1	1.08	0.20
Tmem167b	Q80X45	Protein kish-B	1.08	0.29
Zfp53	Q9Z117	KRAB-containing zinc-finger protein KRAZ1	1.08	0.32
Ddx18	Q8K363	ATP-dependent RNA helicase DDX18	1.08	0.00
Fam219a	A2ANP1	2310028H24Rik protein	1.08	0.07
Slc40a1	Q9JHI9	Solute carrier family 40 member 1	1.08	0.24
Mzt2	Q9CQ25	Mitotic-spindle organizing protein 2	1.08	0.40
Kif3b	Q61771	Kinesin-like protein KIF3B	1.08	0.39
Lipt2	Q9D009	Putative lipoyltransferase 2, mitochondrial	1.08	0.25
Dnajb6	O54946	DnaJ homolog subfamily B member 6	1.08	0.20
Racgap1	Q9WVM1	Rac GTPase-activating protein 1	1.08	0.13
Nudt18	Q3U2V3	8-oxo-dGDP phosphatase NUDT18	1.08	0.27
Rpl19	P84099	60S ribosomal protein L19	1.08	0.03
Slc16a10	Q3U9N9	Monocarboxylate transporter 10	1.08	0.02
Hist1h1a	P43275	Histone H1.1	1.08	0.15
Fam210b	Q9D8B6	Protein FAM210B, mitochondrial	1.08	0.12
Morf4l2	Q9R0Q4	Mortality factor 4-like protein 2	1.08	0.09
Ddx54	Q8K4L0	ATP-dependent RNA helicase DDX54	1.08	0.14
Baz1b	Q9Z277	Tyrosine-protein kinase BAZ1B	1.08	0.05
Saraf	A0A0R4J0D1	Store-operated calcium entry-associated regulatory factor	1.08	0.12
Nol12	Q8BG17	Nucleolar protein 12	1.08	0.25
Camlg	P49070	Calcium signal-modulating cyclophilin ligand	1.08	0.02
Noc3l	Q8VI84	Nucleolar complex protein 3 homolog	1.08	0.44
Inpp5a	E9QAS7	Inositol polyphosphate-5-phosphatase A	1.08	0.30
Tnfrsf11a	O35305	Tumor necrosis factor receptor superfamily member 11A	1.08	0.27
S1pr2	P52592	Sphingosine 1-phosphate receptor 2	1.08	0.01
Crebbp	F8VPR5	CREB-binding protein	1.08	0.63
Timm17a	Q9Z0V8	Mitochondrial import inner membrane translocase subunit Tim17-A	1.08	0.28
Myo1e	E9Q634	Unconventional myosin-Ie	1.08	0.06
Hist1h4a	P62806	Histone H4	1.08	0.12
Srfbp1	Q9CZ91	Serum response factor-binding protein 1	1.08	0.26
Gnl2	Q99LH1	Nucleolar GTP-binding protein 2	1.08	0.02
Rbm34	B2RUP0	RNA binding motif protein 34	1.08	0.19
Hmgn3	Q9DCB1	High mobility group nucleosome-binding domain-containing protein 3	1.08	0.22
Plscr1	Q9JJ00	Phospholipid scramblase 1	1.08	0.03
Gde1	Q9JL56	Glycerophosphodiester phosphodiesterase 1	1.08	0.06
Slc39a6	Q8C145	Zinc transporter ZIP6	1.08	0.09
Tceanc2	Q8R2M0	Transcription elongation factor A N-terminal and central domain-containing protein 2	1.08	0.20
Rrp8	E9PVA2	Ribosomal RNA-processing protein 8	1.08	0.39
Fam69a	Q9D6I7	Protein FAM69A	1.08	0.52
Ccdc137	Q8R0K4	Coiled-coil domain-containing protein 137	1.08	0.14

B4galnt1	Q09200	Beta-1,4 N-acetylgalactosaminyltransferase 1	1.08	0.28
Magt1	A2ADH1	Magnesium transporter protein 1	1.08	0.02
A630001G21Rik	Q3UTB2	RIKEN cDNA A630001G21 gene	1.08	0.00
Lrrk1	Q3UHC2	Leucine-rich repeat serine/threonine-protein kinase 1	1.08	0.68
Ebpl	Q9D0P0	Emopamil-binding protein-like	1.08	0.12
Emc10	A0A0X1KG67	ER membrane protein complex subunit 10	1.09	0.01
Gm45713	A0A1B0GS68	Predicted gene 45713	1.09	0.03
Acot1	O55137	Acyl-coenzyme A thioesterase 1	1.09	0.53
Mtdh	Q80WJ7	Protein LYRIC	1.09	0.11
Slc7a5	Q9Z127	Large neutral amino acids transporter small subunit 1	1.09	0.04
Rpl27a	P14115	60S ribosomal protein L27a	1.09	0.01
Tmem164	Q6PHN7	Transmembrane protein 164	1.09	0.30
Serpinh1	P19324	Serpin H1	1.09	0.14
Tmod2	Q9JKK7	Tropomodulin-2	1.09	0.48
Atg4a	Q8C9S8	Cysteine protease ATG4A	1.09	0.54
Fam111a	Q9D2L9	Protein FAM111A	1.09	0.27
Ifngr1	P15261	Interferon gamma receptor 1	1.09	0.12
Tln2	A0A1L1SQ51	Talin-2	1.09	0.03
Rpl17	Q6ZWZ7	60S ribosomal protein L17	1.09	0.02
Haspin	A0A0R4J0P2	Germ cell-specific gene 2	1.09	0.16
C1qb	P14106	Complement C1q subcomponent subunit B	1.09	0.03
Macf1	F6XCT0	Microtubule-actin cross-linking factor 1 (Fragment)	1.09	0.18
Kdelr3	Q8R1L4	ER lumen protein-retaining receptor 3	1.09	0.23
Slfn8	B1ARD8	Schlafen family member 8	1.09	0.58
Timm21	Q8CCM6	Mitochondrial import inner membrane translocase subunit Tim21	1.09	0.06
Pigx	F7DFD7	Phosphatidylinositol-glycan biosynthesis class X protein	1.09	0.12
Atraid	Q6PGD0	All-trans retinoic acid-induced differentiation factor	1.09	0.17
Vkorc1	Q9CRC0	Vitamin K epoxide reductase complex subunit 1	1.09	0.11
Nbeal1	E9PYP2	Neurobeachin-like 1	1.09	0.50
Macf1	F6RL59	Microtubule-actin cross-linking factor 1 (Fragment)	1.09	0.15
Hist2h3c1	A0A1W2P768	Histone H3.2	1.09	0.08
Alg9	Q8VDI9	Alpha-1,2-mannosyltransferase ALG9	1.09	0.04
Zfand3	Q497H0	AN1-type zinc finger protein 3	1.09	0.50
Cnep1r1	Q3UJ81	Nuclear envelope phosphatase-regulatory subunit 1	1.09	0.45
Tgfbr1	Q64729	TGF-beta receptor type-1	1.09	0.18
Vsig8	Q6P3A4	V-set and immunoglobulin domain-containing protein 8	1.09	0.49
Kif22	Q3V300	Kinesin-like protein KIF22	1.09	0.45
Mdfic	A0A087WRH9	MyoD family inhibitor domain-containing protein	1.09	0.04
Brix1	Q9DCA5	Ribosome biogenesis protein BRX1 homolog	1.09	0.02
Kcnj2	P35561	Inward rectifier potassium channel 2	1.09	0.03
Znf22	Q9ERU3	Zinc finger protein 22	1.09	0.03
Slc12a2	E9QM38	Solute carrier family 12 member 2	1.09	0.37
Ubald1	Q6P3B2	UBA-like domain-containing protein 1	1.09	0.50
Mrps14	Q9CR88	28S ribosomal protein S14, mitochondrial	1.09	0.07
Synj2bp	Q9D6K5	Synaptojanin-2-binding protein	1.09	0.32
Krr1	Q8BGA5	KRR1 small subunit processome component homolog	1.09	0.00
Col1a2	Q01149	Collagen alpha-2(I) chain	1.10	0.20
Slc37a4	A0A1L1SUI3	Solute carrier family 37 (glucose-6-phosphate transporter), member 4	1.10	0.40
Rpl8	P62918	60S ribosomal protein L8	1.10	0.03
Smg6	P61406	Telomerase-binding protein EST1A	1.10	0.00
Cdk19	Q8BWD8	Cyclin-dependent kinase 19	1.10	0.16
Sorl1	O88307	Sortilin-related receptor	1.10	0.22
Metrnl	Q8VE43	Meteorin-like protein	1.10	0.34
Smarcal1	Q8BJL0	SWI/SNF-related matrix-associated actin-dependent regulator of chromatin subfamily A-like protein 1	1.10	0.47
Surf2	P09926	Surfeit locus protein 2	1.10	0.65
Sec24a	Q3U2P1	Protein transport protein Sec24A	1.10	0.29
Tmem263	Q9DAM7	Transmembrane protein 263	1.10	0.15
Cry1	P97784	Cryptochrome-1	1.10	0.46
Mfsd14a	P70187	Hippocampus abundant transcript 1 protein	1.10	0.59
Mrpl35	Q9CQL6	39S ribosomal protein L35, mitochondrial	1.10	0.02
Ebag9	Q9D0V7	Receptor-binding cancer antigen expressed on SiSo cells	1.10	0.07
H1f0	P10922	Histone H1.0	1.10	0.01
Calcrl	Q9R1W5	Calcitonin gene-related peptide type 1 receptor	1.10	0.05
Rpl18	P35980	60S ribosomal protein L18	1.10	0.05
Sp100	F6WL90	Nuclear autoantigen Sp-100 (Fragment)	1.10	0.06
Cep120	Q7TSG1	Centrosomal protein of 120 kDa	1.10	0.73
Senp1	M0QWX4	Sentrin-specific protease 1	1.10	0.41
Kmt2b	F8WJ40	Histone-lysine N-methyltransferase	1.10	0.32
Rsl1d1	Q8BVY0	Ribosomal L1 domain-containing protein 1	1.10	0.03
Gas2l3	A0A0R4J1A6	GAS2-like protein 3	1.10	0.05
Atp6v1g1	Q9CR51	V-type proton ATPase subunit G 1	1.11	0.66
Fam76a	Q922G2	Protein FAM76A	1.11	0.31
	Q91WE4	UPF0729 protein C18orf32 homolog OS=Mus musculus OX=10090	1.11	0.13

Rpl28	P41105	60S ribosomal protein L28	1.11	0.08
Cd82	P40237	CD82 antigen	1.11	0.03
Ei24	A0A0R4J250	Etoposide induced 2.4 mRNA	1.11	0.00
Slc39a10	Q6P5F6	Zinc transporter ZIP10	1.11	0.07
Rpf2	Q9JJ80	Ribosome production factor 2 homolog	1.11	0.01
Tmem97	Q8VD00	Sigma intracellular receptor 2	1.11	0.10
Myo9a	D3Z3A8	Unconventional myosin-IXa	1.11	0.50
Agpat1	A0A0R4J263	1-acyl-sn-glycerol-3-phosphate acyltransferase	1.11	0.26
Evi2a	P20934	Protein EVI2A	1.11	0.05
Rpl3	P27659	60S ribosomal protein L3	1.11	0.07
Myh14	K3W4R2	Myosin-14	1.11	0.03
Hp1bp3	Q3TEA8	Heterochromatin protein 1-binding protein 3	1.11	0.09
Chtop	Q9CY57	Chromatin target of PRMT1 protein	1.11	0.04
Dnajb2	Q9QYI5	DnaJ homolog subfamily B member 2	1.11	0.09
Tomm7	Q9D173	Mitochondrial import receptor subunit TOM7 homolog	1.12	0.05
Fyttd1	Q91Z49	UAP56-interacting factor	1.12	0.10
Rpl29	P47915	60S ribosomal protein L29	1.12	0.02
Zfp871	G5E905	RIKEN cDNA 9030612M13	1.12	0.28
Ifi203	O35368	Interferon-activable protein 203	1.12	0.61
Cd63	P41731	CD63 antigen	1.12	0.12
Hist1h1b	P43276	Histone H1.5	1.12	0.01
Smim7	Q5RKS2	Small integral membrane protein 7	1.12	0.05
Ebp	P70245	3-beta-hydroxysteroid-Delta(8),Delta(7)-isomerase	1.12	0.20
Rpl7	P14148	60S ribosomal protein L7	1.12	0.01
Tanc1	Q0VGY8	Protein TANC1	1.12	0.30
Hist1h1c	P15864	Histone H1.2	1.12	0.06
Senp7	Q8BUH8	Sentrin-specific protease 7	1.12	0.30
Herc2	Q4U2R1	E3 ubiquitin-protein ligase HERC2	1.12	0.60
Rpl6	P47911	60S ribosomal protein L6	1.12	0.02
Zfp2	P08043	Zinc finger protein 2	1.12	0.07
Hist1h1e	P43274	Histone H1.4	1.12	0.10
D8Ertd738e	Q8R1F0	Leydig cell tumor 10 kDa protein homolog	1.13	0.06
Rpl7a	P12970	60S ribosomal protein L7a	1.13	0.06
Hba-a1	Q91VB8	Alpha globin 1	1.13	0.51
Tnip3	A0A1D5RMN0	TNFAIP3-interacting protein 3	1.13	0.05
Gm5814	J3QP80	MCG15559	1.13	0.09
Col1a1	P11087	Collagen alpha-1(I) chain	1.13	0.03
Scamp5	Q9JKD3	Secretory carrier-associated membrane protein 5	1.14	0.03
Rpl4	Q9D8E6	60S ribosomal protein L4	1.14	0.00
Rpl14	Q9CR57	60S ribosomal protein L14	1.15	0.02
Apoa1	Q00623	Apolipoprotein A-I	1.15	0.21
Taok2	Q6ZQ29	Serine/threonine-protein kinase TAO2	1.15	0.38
Gfap	P03995	Glial fibrillary acidic protein	1.15	0.58
Wdr83	Q9DAJ4	WD repeat domain-containing protein 83	1.15	0.50
Alg14	Q9D081	UDP-N-acetylglucosamine transferase subunit ALG14 homolog	1.16	0.00
Atf6	F6VAN0	Cyclic AMP-dependent transcription factor ATF-6 alpha	1.16	0.04
Hic2	Q9JLZ6	Hypermethylated in cancer 2 protein	1.17	0.00
Tpx2	A2APB8	Targeting protein for Xklp2	1.17	0.05
Prg2	Q61878	Bone marrow proteoglycan	1.17	0.08
Diaph3	F8WIG5	Protein diaphanous homolog 3	1.18	0.03
Smpd1	Q04519	Sphingomyelin phosphodiesterase	1.18	0.28
Tbp	P29037	TATA-box-binding protein	1.20	0.43
Cip2a	D3Z7B5	Protein CIP2A	1.20	0.46
Tmem50a	Q9CXL1	Transmembrane protein 50A	1.22	0.02
Hbb-bs	A8DUK4	Beta-globin	1.25	0.34
	Q5XFZ0	UPF0711 protein C18orf21 homolog	1.25	0.00
Hells	Q60848	Lymphocyte-specific helicase	1.25	0.39
Bcl7c	A0A0U1RNX8	B-cell CLL/lymphoma 7 protein family member C	1.25	0.29
C1qtnf6	Q6IR41	Complement C1q tumor necrosis factor-related protein 6	1.33	0.01
Des	P31001	Desmin	1.34	0.42
Gpn2	Q8VEJ1	GPN-loop GTPase 2	1.34	0.51
Smyd2	Q8R5A0	N-lysine methyltransferase SMYD2	1.46	0.01

Supplementary Table S4. A list of phosphosites in CTRP6-treated BMDMs quantitated by TMT

Gene Symbol	Protein	UniProt Accession Number	Phosphosite(s) in Protein	Phosphopeptide Sequence	Phosphorylation Location in Peptide	Fold Change (Ctrp6-vs Vehicle-treated BMDMs)	p-value
0610010K14Rik	RIKEN cDNA 0610010K14 gene	D3Z687	S96	VYEDSGIPLPAESPK	S13	1.069	0.539
0610010K14Rik	RIKEN cDNA 0610010K14 gene	D3Z687	S96	KVYEDSGIPLPAESPK	S14	0.978	0.819
1110008P14Rik	UPF0184 protein C9orf16 homolog	P58686	S82	LEFQQQLGEAPGDASP	S15	0.984	0.926
1110038F14Rik	UPF0488 protein C8orf33 homolog	Q3U6N9	S31	SASGASEPPLR	S1	1.064	0.311
1110059G10Rik	Uncharacterized protein KIAA1143 homolog	Q8K039	S150	NSSLLSFDSEDENE	S9	1.006	0.940
1700017B05Rik	Uncharacterized protein C15orf39 homolog	Q3TEI4	S471	TSPALHPCAK	S2	1.017	0.862
1700017B05Rik	Uncharacterized protein C15orf39 homolog	Q3TEI4	S912	TLSPSEPTVTR	S3	1.004	0.940
1700017B05Rik	Uncharacterized protein C15orf39 homolog	Q3TEI4	Ambiguous	AEAATGEGSRNPSPSSGASTSPPGPTLR	S/T	1.095	0.391
1700021F05Rik	Protein 1700021F05Rik	D3Z316	S106	EADEEDSDEETSYPER	S7	1.020	0.863
1700021F05Rik	Protein 1700021F05Rik	D3Z316	S106	EADEEDSDEETSYPERSEQEEELESEPGVAK	S7	1.018	0.779
1700025G04Rik	Uncharacterized protein C1orf21 homolog	Q8K207	S115	DYCSEEEDIT	S4	0.922	0.576
1700030K09Rik	Uncharacterized protein C19orf44 homolog	Q922C1	Ambiguous	ESASDEEEEMLLLR	S	0.990	0.946
1810037I17Rik	Uncharacterized protein C4orf3 homolog	Q99M08	S19	GSFEAGRR	S2	1.141	0.347
1810037I17Rik	Uncharacterized protein C4orf3 homolog	Q99M08	S19	RGSFEAGR	S3	1.106	0.279
2010111I01Rik	RIKEN cDNA 2010111I01 gene	Q3UQZ7	S368	ASPPDDLMTEHSLPLSPSEADLR	S16	0.697	0.068
2010300C02Rik	RIKEN cDNA 2010300C02 gene	E9Q3M9	S113	VFSQENVCDR	S3	1.064	0.359
2310011J03Rik	UPF0449 protein C19orf25 homolog	Q9D7E4	S49	GAPAQDPVFTALAPEDPPDPSPR	S21	1.131	0.491
2310022A10Rik	RIKEN cDNA 2310022A10 gene	G5E8E3	S141	MIANSLNHDSPPHTPTR	S10	1.004	0.907
2310022A10Rik	RIKEN cDNA 2310022A10 gene	G5E8E3	T317	ATSSATTTATTPK	T11	1.039	0.675
2310022B05Rik	Uncharacterized protein C1orf198 homolog	Q8C3W1	S176	SSSLDALGPAR	S3	1.035	0.631
2310039H08Rik	Uncharacterized protein C6orf226 homolog	Q9D727	Ambiguous	SRGSPAAQLDGGPR	S	1.081	0.431
2410004B18Rik	UPF0690 protein C1orf52 homolog	Q9CWU4	S156	LLPEGEETVESDDDKDER	S11	1.026	0.507
2410004B18Rik	RIKEN cDNA 2410004B18 gene (Fragment)	A0A087WQ28	Ambiguous	LLPEGEETVESEN	T/S	0.949	0.643
2810004N23Rik	Uncharacterized protein C1orf131 homolog	Q8CIL4	S67	DSETIEDVAVEPLPLPGSPVR	S18	0.827	0.172
2900026A02Rik	RIKEN cDNA 2900026A02 gene	A0A1B0GR85	S1132	DAPQTSPVLR	S6	0.990	0.706
2900026A02Rik	RIKEN cDNA 2900026A02 gene	A0A1B0GR85	S1152	TEVVSETFPGK	S5	0.955	0.705
2900026A02Rik	RIKEN cDNA 2900026A02 gene	A0A1B0GR85	S1194	TQQQQDSPPAEPSSSPQER	S7	0.975	0.809
2900026A02Rik	RIKEN cDNA 2900026A02 gene	A0A1B0GR85	S1331	YPVSPGSPPSDR	S7	1.003	0.963
2900026A02Rik	RIKEN cDNA 2900026A02 gene	A0A1B0GR85	S1331	SQEPGSRYPVSPGSPPSDRK	S14	0.941	0.445
2900026A02Rik	RIKEN cDNA 2900026A02 gene	A0A1B0GR85	S1454	QVSPVASVPR	S3	0.938	0.288
2900026A02Rik	RIKEN cDNA 2900026A02 gene	A0A1B0GR85	S1664	RDESDEEPPRVER	S4	0.929	0.576
2900026A02Rik	RIKEN cDNA 2900026A02 gene	A0A1B0GR85	S1747	SEECSPQWLK	S5	0.950	0.572
2900026A02Rik	RIKEN cDNA 2900026A02 gene	A0A1B0GR85	S600	SAGDAGPSEVSPEDDGSFQK	S11	1.023	0.809
2900026A02Rik	RIKEN cDNA 2900026A02 gene	A0A1B0GR85	S880	ANIEPCDSQVQTGPDSLSPK	S18	0.970	0.689
2900026A02Rik	RIKEN cDNA 2900026A02 gene	A0A1B0GR85	Ambiguous	SQEPGSRYPVSPGSPPSDR	S/Y	1.070	0.403
3110082I17Rik	Uncharacterized protein C7orf50 homolog	Q9CXL3	S177	LDEAEPEDSGGSPGKVQR	S9	0.983	0.532
3110082I17Rik	Uncharacterized protein C7orf50 homolog	Q9CXL3	S52	TSEAGPDKETASTLVQEASPELSPEER	S23	1.002	0.958
3110082I17Rik	Uncharacterized protein C7orf50 homolog	Q9CXL3	Ambiguous	LDEAEPEDSGGSPGK	S	0.986	0.903
4931414P19Rik	Uncharacterized protein C14orf93 homolog	Q8K2W9	S226	QLSPATQLAIQR	S3	1.256	0.256
4931414P19Rik	Uncharacterized protein C14orf93 homolog	Q8K2W9	T118	ARTPPPEEPGVLK	T3	1.034	0.692
4932438A13Rik	RIKEN cDNA 4932438A13 gene (Fragment)	F6RPM4; A2AAE1	S576;S3835	SLDQDSPSK	S6	1.021	0.545
4932438A13Rik	RIKEN cDNA 4932438A13 gene (Fragment)	F6RPM4; A2AAE1	S830;S4124	AGMPGSSGLGSPLGR	S11	1.081	0.251
4932438A13Rik	RIKEN cDNA 4932438A13 gene (Fragment)	F6RPM4; A2AAE1	T702;T3996	ALETIPITPIER	T8	0.881	0.737
4932438A13Rik	RIKEN cDNA 4932438A13 gene (Fragment)	F6RPM4; A2AAE1	Ambiguous	ASSRIGETDELPEIR	S/T	1.140	0.134
4933427D14Rik	RIKEN cDNA 4933427D14 gene	A0A0R4J1C3	S182	ADLTVPNSPPTHDPGLQPQPR	S8	1.010	0.796
4933427D14Rik	RIKEN cDNA 4933427D14 gene	A0A0R4J1C3	S868	EAPLPLSREDLHQR	S7	1.152	0.383
5031439G07Rik	RIKEN cDNA 5031439G07 gene	B1APX2	S15	ACASASAPAGLR	S6	1.100	0.146
5031439G07Rik	RIKEN cDNA 5031439G07 gene	B1APX2	S30	AGPAASRESVDSSEAEER	S9	0.991	0.898
5031439G07Rik	RIKEN cDNA 5031439G07 gene	B1APX2	S311	VSTGDTSPCGTEDSSPASPMHER	S7	1.055	0.515
5031439G07Rik	RIKEN cDNA 5031439G07 gene	B1APX2	S322	VSTGDTSPCGTEDSSPASPMHER	S18	1.068	0.151
5031439G07Rik	RIKEN cDNA 5031439G07 gene	B1APX2	S367	SHSANDSEEFFREDDSGADLHNATNLR	S3	1.089	0.541
5031439G07Rik	RIKEN cDNA 5031439G07 gene	B1APX2	T336	VTSFSTPPTPER	T9	0.986	0.571
5031439G07Rik	RIKEN cDNA 5031439G07 gene	B1APX2	S311,S322	VSTGDTSPCGTEDSSPASPMHER	S7,S18	1.215	0.087
5031439G07Rik	RIKEN cDNA 5031439G07 gene	B1APX2	S322,T/S	VSTGDTSPCGTEDSSPASPMHER	S18,T/S	1.104	0.447
5031439G07Rik	RIKEN cDNA 5031439G07 gene	B1APX2	S332,T336	VTSFSTPPTPER	S5,T9	1.039	0.342
8030462N17Rik	RIKEN cDNA 8030462N17 gene	Q0VAW6	S66	RDSSESQLASTESDKPTTGR	S3	1.054	0.089
8030462N17Rik	RIKEN cDNA 8030462N17 gene	Q0VAW6	Ambiguous	SRSESETSTMAAK	S/T	1.222	0.201
A430005L14Rik	UPF0688 protein C1orf174 homolog	Q80WR5	S22	SYSSASLASAR	S3	1.328	0.014
A430005L14Rik	UPF0688 protein C1orf174 homolog	Q80WR5	T119	EPPQGCSTPASEEPSVK	T8	1.059	0.056
A430005L14Rik	UPF0688 protein C1orf174 homolog	Q80WR5	Ambiguous	AENGLSTEPSSAAAAQEPDDSSAGQAEPVPR	S/T	1.091	0.655
A630001G21Rik	RIKEN cDNA A630001G21 gene	Q3UTB2	S115	SLGTELQDGYSPQK	S11	0.871	0.090
Aaas	Aladin	P58742	S495	FSPVLGR	S2	0.865	0.014
Aaed1	Thioredoxin-like protein AAED1	Q9D1A0	S10	QVSGCAGRVPSPAGSVTER	S3	1.158	0.089
Aak1	AP2-associated protein kinase 1	Q3UHJ0	S18	REQGSSGLGSGSSGGGGSSSGLGSGYIGR	S10	1.023	0.326
Aak1	AP2-associated protein kinase 1	Q3UHJ0	S18	EQGSSGLGSGSSGGGGSSSGLGSGYIGR	S9	0.964	0.667
Aak1	AP2-associated protein kinase 1	Q3UHJ0	S676	SATTTPSGSPR	S9	0.985	0.741
Aak1	AP2-associated protein kinase 1	Q3UHJ0	T389	AGQTQPNPGILPIQPALTPR	T18	0.787	0.119
Aak1	AP2-associated protein kinase 1	Q3UHJ0	S621,S622	VGSLTPPSSPKTQR	S8,S9	0.860	0.397
Aak1	AP2-associated protein kinase 1	Q3UHJ0	S676,T/S	SATTTPSGSPR	S9,T/S	1.035	0.675
Aak1	AP2-associated protein kinase 1	Q3UHJ0	T618,S622	VGSLTPPSSPK	T5,S9	0.989	0.851
Aak1	AP2-associated protein kinase 1	Q3UHJ0	S668,T670,S676	SKSATTTPSGSPR	S3,T5,S11	0.995	0.877
Aak1	Uncharacterized protein FLJ45252 homolog	Q6PIU9	S115	ASDDLGEPDVFATAPFR	S2	1.372	0.018
Aak1	Uncharacterized protein FLJ45252 homolog	Q6PIU9	S292	HYSPEDELSAEAQPIAAYK	S3	0.961	0.757
Aak1	AP2-associated protein kinase 1	Q3UHJ0	Ambiguous	VQTTPPPTIQGQK	T	1.031	0.310
Aars	Alanine--tRNA ligase, cytoplasmic	Q8BGQ7	S399	IQSLGDCK	S3	1.108	0.349
Aatk	Serine/threonine-protein kinase LMTK1	B1AZF3	S1012	DSAYFSDLDAESEPTFGPEK	S6	1.115	0.558
Aatk	Serine/threonine-protein kinase LMTK1	B1AZF3	S1044	SPPSPGHQSVQAFPR	S4	1.142	0.143
Aatk	Serine/threonine-protein kinase LMTK1	B1AZF3	S1187	CYSVQEPSEDSEEEPPAVPVVVAESQSAR	S11	1.069	0.593
Aatk	Serine/threonine-protein kinase LMTK1	B1AZF3	S1273	ESLPTFLEGGPSSPSATGLPLR	S13	0.899	0.348
Aatk	Serine/threonine-protein kinase LMTK1	B1AZF3	S1340	FTVSPTPASR	S4	0.978	0.695
Aatk	Serine/threonine-protein kinase LMTK1	B1AZF3	S727	ASPEVGHLLSQEDPR	S2	0.966	0.485
Aatk	Serine/threonine-protein kinase LMTK1	B1AZF3	Ambiguous	WEAGCGAEEYPPSGAASSPGSAAR	S/Y	1.087	0.198
AB124611	cDNA sequence AB124611	A0A0B4J1G2	T198	MTQEDSVATLDSGPR	T9	0.999	0.990
AB124611	cDNA sequence AB124611	A0A0B4J1G2	S211,T/S	KRPTSASSSPEPPEFSTFR	S7,T/S	0.941	0.249
Abca1	ATP-binding cassette sub-family A member 1	P41233	S1296	QSCLHPFTEDDAVDPNDSDIDPESR	S18	1.112	0.050
Abca1	ATP-binding cassette sub-family A member 1	P41233	T1265	VAEESGVDAETSDGTLPARR	T15	1.082	0.249
Abca1	ATP-binding cassette sub-family A member 1	P41233	Ambiguous	NSSSTVSCLK	S/T	0.906	0.320
Abca1	ATP-binding cassette sub-family A member 1	P41233	Ambiguous	DVESSLSSCR	S	1.061	0.173
Abca2	ATP-binding cassette sub-family A member 2	P41234	S1238	QPAEPGTSQEPGLASSPSGCPR	S16	0.973	0.716
Abca2	ATP-binding cassette sub-family A member 2	P41234	S1327	VSEEDQSLENSEADVK	S7	0.973	0.700
Abca2	ATP-binding cassette sub-family A member 2	P41234	S2427	AQLSFNTDTLC	S4	1.022	0.867
Abca2	ATP-binding cassette sub-family A member 2	P41234	T2411	ALVADEPEDLDTEDEGLISFEEER	T12	0.918	0.434
Abca2	ATP-binding cassette sub-family A member 2	P41234	S1327,S1331	VSEEDQSLENSEADVKESR	S7,S11	0.979	0.700
Abca2	ATP-binding cassette sub-family A member 2	P41234	S1327,S1331	VSEEDQSLENSEADVK	S7,S11	0.956	0.862
Abcb1a	Multidrug resistance protein 1A	P21447	S667	SICGPHDQDRK	S1	0.959	0.636
Abcb1b	Multidrug resistance protein 1B	P06795	S659,Y/S/T	GNEIEPGNNAYGSQSDTDASELTSEESKSPLIR	S29,Y/S/T	0.939	0.701
Abcb1b	Multidrug resistance protein 1B	P06795	Ambiguous	GNEIEPGNNAYGSQSDTDASELTSEESKSPLIRR	S/T/Y	1.107	0.593
Abcb1b	Multidrug resistance protein 1B	P06795	Ambiguous	GNEIEPGNNAYGSQSDTDASELTSEESKSPLIR	T/S/Y	1.114	0.059
Abcc1	Multidrug resistance-associated protein 1	O35379	S884	TYANAEQDLASEDDSVSGSGK	S17	1.060	0.282
Abcc1	Multidrug resistance-associated protein 1	O35379	S878,S882	TYANAEQDLASEDDSVSGSGK	S11,S15	1.204	0.065
Abcc3	Canalicular multispecific organic anion transporter 2	B2RX12	S907	EMSSLSSEGEVQNR	S7	1.062	0.592
Abcc3	Canalicular multispecific organic anion transporter 2	B2RX12	Ambiguous	EMSSLSSEGEVQNR	S	1.175	0.126
Abcc4	ATP-binding cassette, sub-family C (CFTR/MRP), member 4	E9Q236	T646	ENEEAEPSTAPGTPTLR	T13	0.999	0.985
Abcc4	ATP-binding cassette, sub-family C (CFTR/MRP), member 4	E9Q236	T646	KENEEAEPSTAPGTPTLR	T14	1.021	0.702
Abcc4	ATP-binding cassette, sub-family C (CFTR/MRP), member 4	E9Q236	T642,T646	ENEEAEPSTAPGTPTLR	T9,T13	1.207	0.385
Abcc4	ATP-binding cassette, sub-family C (CFTR/MRP), member 4	E9Q236	T642,T646	KENEEAEPSTAPGTPTLR	T10,T14	0.982	0.760
Abcc5	Multidrug resistance-associated protein 5	Q9R1X5	S14	EYIIPSPGYR	S6	1.123	0.039
Abcc5	Multidrug resistance-associated protein 5	Q9R1X5	S43	SLECQDALETAAR	S1	1.085	0.359
Abcc5	Multidrug resistance-associated protein 5	Q9R1X5	S43	TRSLECQDALETAAR	S3	1.089	0.235
Abcc5	Multidrug resistance-associated protein 5	Q9R1X5	S553	GHLLLDSDERPSPEEEEGK	S7	0.989	0.793
Abcd2	ATP-binding cassette sub-family D member 2	A0A0R4J0U5	S58	AEAYSPAENR	S5	1.022	0.670
Abcf1	ATP-binding cassette sub-family F member 1	Q6P542	S107	QLSVPASDEEDEVPAPIPR	S7	1.045	0.076
Abcf1	ATP-binding cassette sub-family F member 1	Q6P542	S138	GGNVFEALIQDDSEEEEEEENR	S13	1.566	0.502
Abcf1	ATP-binding cassette sub-family F member 1	Q6P542	S194	SKPAAADSEGEEEEEDTAK	S8	1.083	0.147
Abcf1	ATP-binding cassette sub-family F member 1	Q6P542	S194	SKPAAADSEGEEEEEDTAKEKEPPQQGK	S8	0.982	0.789
Abcf1	ATP-binding cassette sub-family F member 1	Q6P542	S194	SKPAAADSEGEEEEEDTAKEK	S8	1.014	0.810
Abcf1	ATP-binding cassette sub-family F member 1	Q6P542	S225	EAEQGSGEEKEEKEGDLK	S6	1.059	0.457
Abcf1	ATP-binding cassette sub-family F member 1	Q6P542	S225	EAEQGSGEEKEEK	S6	1.084	0.582
Abcf1	ATP-binding cassette sub-family F member 1	Q6P542	S90	DVDDDSDERVLMER	S6	1.054	0.214
Abcf1	ATP-binding cassette sub-family F member 1	Q6P542	S90	DVDDDSDERVLMER	S6	1.015	0.809
Abcf1	ATP-binding cassette sub-family F member 1	Q6P542	S103,S107	LKQLSVPASDEEDEVPAPIPR	S5,S9	1.007	0.918
Abhd12	Monoacylglycerol lipase ABHD12	Q99LR1	S20	CAASGSSSSGSAAAALDADCSLK	S6	1.117	0.250
Abhd2	Monoacylglycerol lipase ABHD2	Q9QXM0	S415	SQCSDTEQMEAELE	S4	0.665	0.022
Abhd2	Monoacylglycerol lipase ABHD2	Q9QXM0	S415	SQCSDTEQMEAELE	S4	0.998	0.979
Abhd2	Monoacylglycerol lipase ABHD2	Q9QXM0	S415	NKSQCSDTEQMEAELE	S6	0.979	0.751
Abhd2	Monoacylglycerol lipase ABHD2	Q9QXM0	S415	NKSQCSDTEQMEAELE	S6	0.998	0.967
Abi1	Abl interactor 1	J3QNK8	S183	TNPPTQKPPSPPVSGR	S10	0.998	0.957
Abi1	Abl interactor 1	J3QNK8	S216	TLEPVKPPTVPNDYMTSPAR	S17	1.007	0.872
Abi1	Abl interactor 1	J3QNK8	S225	LGSQHSPGR	S6	0.967	0.694
Abi1	Abl interactor 1	J3QNK8	S231	TASLNQRPR	S3	1.046	0.355
Abi1	Abl interactor 1	J3QNK8	S183,S187	TNPPTQKPPSPPVSGRGTGLGR	S10,S/T	0.936	0.498
Abi2	Abi2 protein	Q6AXD2	S227	NMAPSQQSPVR	S8	0.980	0.878
Abi2	Abi2 protein	Q6AXD2	S368	HTPPTIGGSLPYR	S9	1.015	0.853
Abi3	ABI gene family member 3	Q8BYZ1	S164	DLSTQLSR	S7	1.091	0.372

Abl1	Tyrosine-protein kinase ABL1	P00520	S569	GLGESDALDSEPAVSPLLPR	S15	1.018	0.768
Abl1	Tyrosine-protein kinase ABL1	P00520	S909	AGKPAQSPSQEAGEAGGPTK	S7	1.091	0.109
Abl2	Tyrosine-protein kinase	F8VQH0	S621	STQASSGSPALPR	S8	1.004	0.858
Abl2	Tyrosine-protein kinase	F8VQH0	S632	DKSPSSLLEDAK	S3	1.066	0.286
Abl2	Tyrosine-protein kinase	F8VQH0	S785	SNSTSSMSSGLPEQDR	S3	1.092	0.329
Abl2	Tyrosine-protein kinase	F8VQH0	S822	TVSTSSQPEENVDR	S6	1.170	0.027
Abl2	Tyrosine-protein kinase	F8VQH0	T820	TVSTSSQPEENVDRANDMLPK	T4	1.014	0.904
Abl2	Tyrosine-protein kinase	F8VQH0; P00520	Y439;Y393	LMTGDTYTAHAGAK	Y7	0.890	0.120
Abl2	Tyrosine-protein kinase	F8VQH0	S819,S822	TVSTSSQPEENVDR	S3,S6	1.115	0.240
Abl2	Tyrosine-protein kinase	F8VQH0	S819,T/S	TVSTSSQPEENVDRANDMLPK	S3,T/S	1.025	0.816
Abl2	Tyrosine-protein kinase	F8VQH0	Ambiguous	SSSFREMENQPHK	S	0.971	0.639
Abr	Active breakpoint cluster region-related protein	Q5SSL4	S53	GPPEGSETMPYIDESPTMSPQLSAR	S15	1.042	0.488
Abr	Active breakpoint cluster region-related protein	Q5SSL4	S53	GPPEGSETMPYIDESPTMSPQLSAR	S15	0.629	0.049
Abr	Active breakpoint cluster region-related protein	Q5SSL4	S53	GPPEGSETMPYIDESPTMSPQLSAR	S15	0.976	0.360
Abr	Active breakpoint cluster region-related protein	Q5SSL4	S72	SQGGGDSVSPTPPEGLAPGVEAGK	S9	1.002	0.976
Abr	Active breakpoint cluster region-related protein	Q5SSL4	S64,S/T	SQGGGDSVSPTPPEGLAPGVEAGK	S1,S/T	0.944	0.566
Abraxas1	BRCA1-A complex subunit Abraxas 1	Q8BPZ8	S48	NSITDSQMDNVK	S6	0.973	0.514
Abraxas2	BRISC complex subunit Abraxas 2	Z4YJY0	S289	QMPSESLEPAFSPR	S12	1.044	0.379
Abraxas2	BRISC complex subunit Abraxas 2	Z4YJY0	S289	QMPSESLEPAFSPR	S12	1.078	0.567
Abraxas2	BRISC complex subunit Abraxas 2	Z4YJY0	S293	MSYSGFSAEGR	S2	0.906	0.219
Acaca	Acetyl-CoA carboxylase 1	Q5SWU9	S2342	ILSTMDSPST	S7	1.106	0.373
Acap1	Arf-GAP with coiled-coil, ANK repeat and PH domain-containing protein 1	Q8K2H4	T739	SHDLHTL	T6	1.035	0.661
Acap2	Arf-GAP with coiled-coil, ANK repeat and PH domain-containing protein 2	A0A338P6P6	S379	KSSPSTGSLDSGNESK	S3	1.065	0.132
Acap2	Arf-GAP with coiled-coil, ANK repeat and PH domain-containing protein 2	A0A338P6P6	S384	SSPSTGSLDSGNESKEK	S7	0.963	0.575
Acap2	Arf-GAP with coiled-coil, ANK repeat and PH domain-containing protein 2	A0A338P6P6	S384	SSPSTGSLDSGNESK	S7	1.050	0.252
Acap2	Arf-GAP with coiled-coil, ANK repeat and PH domain-containing protein 2	A0A338P6P6	Y769	ESEGLYGQPGDETYQDIFR	Y6	0.943	0.448
Acap2	Arf-GAP with coiled-coil, ANK repeat and PH domain-containing protein 2	A0A338P6P6	S381,S384	SSPSTGSLDSGNESKEK	S4,S7	0.964	0.337
Acap2	Arf-GAP with coiled-coil, ANK repeat and PH domain-containing protein 2	A0A338P6P6	S381,S384	SSPSTGSLDSGNESK	S4,S7	1.061	0.041
Acap3	ArfGAP with coiled-coil, ankyrin repeat and PH domains 3	Q6NXL5	S383	TASPSTSSIDSTTDSR	S3	1.067	0.400
Acbd4	Acyl-Coenzyme A-binding domain-containing 4	E9Q289	S162	EDQAAPEPSCVPKEPVPPSPESRPPR	S19	1.182	0.069
Acbd5	Acyl-CoA-binding domain-containing protein 5	A0A2I3BQ50	S202	AESSDSGAESEEEEAQEELKGAEQSGSDDKK	S10	0.936	0.447
Acbd5	Acyl-CoA-binding domain-containing protein 5	A0A2I3BQ50	S198,S202	AESSDSGAESEEEEAQEELK	S6,S10	0.964	0.713
Acbd5	Acyl-CoA-binding domain-containing protein 5	A0A2I3BQ50	S198,S202	AESSDSGAESEEEEAQEELKGAEQSGSDDKK	S6,S10	1.004	0.956
Acd	Adrenocortical dysplasia protein	Q5EE38	S24	ELILGSETLSSPR	S10	1.043	0.711
Acin1	Apoptotic chromatin condensation inducer in the nucleus (Fragment)	F6RJ39	S162	SSSFSEEKGESDDEKPR	S11	0.973	0.675
Acin1	Apoptotic chromatin condensation inducer in the nucleus (Fragment)	F6RJ39	S328	SSQSLEEK	S4	1.054	0.343
Acin1	Apoptotic chromatin condensation inducer in the nucleus (Fragment)	F6RJ39	S335	SQSPSPPPLPEDLEK	S3	1.044	0.390
Acin1	Apoptotic chromatin condensation inducer in the nucleus (Fragment)	F6RJ39	S414	EPLASPHPAQLLR	S5	0.934	0.455
Acin1	Apoptotic chromatin condensation inducer in the nucleus (Fragment)	F6RJ39	S425	SLSPLSGTTDTK	S3	0.937	0.384
Acin1	Apoptotic chromatin condensation inducer in the nucleus (Fragment)	F6RJ39	S541	SPSPDSVASRPQSSPGSK	S3	1.052	0.122
Acin1	Apoptotic chromatin condensation inducer in the nucleus (Fragment)	F6RJ39	S656	HLSHPEPEQQHVIQR	S3	1.090	0.536
Acin1	Apoptotic chromatin condensation inducer in the nucleus (Fragment)	F6RJ39	S675	LQPEQGSPK	S7	0.924	0.496
Acin1	Apoptotic chromatin condensation inducer in the nucleus (Fragment)	F6RJ39	S784	GVQAGNSDTEGGQPGRK	S7	0.905	0.218
Acin1	Apoptotic chromatin condensation inducer in the nucleus (Fragment)	F6RJ39	S784	GVQAGNSDTEGGQPGR	S7	1.010	0.846
Acin1	Apoptotic chromatin condensation inducer in the nucleus (Fragment)	F6RJ39	S832	EAVVDLHADDSRISEDETER	S14	0.981	0.687
Acin1	Apoptotic chromatin condensation inducer in the nucleus (Fragment)	F6RJ39	S923	SGVSITIDDPVR	S4	1.232	0.145
Acin1	Apoptotic chromatin condensation inducer in the nucleus (Fragment)	F6RJ39	S937	TAQVPSPPR	S6	1.024	0.554
Acin1	Apoptotic chromatin condensation inducer in the nucleus (Fragment)	F6RJ39	S937	TAQVPSPPRGK	S6	0.973	0.606
Acin1	Apoptotic chromatin condensation inducer in the nucleus (Fragment)	F6RJ39	S335,S337	SQSPSPPPLPEDLEK	S3,S5	1.066	0.300
Acin1	Apoptotic chromatin condensation inducer in the nucleus (Fragment)	F6RJ39	S425,S437	SLSPLSGTTDTKAESPAGR	S3,S15	1.007	0.931
Acin1	Apoptotic chromatin condensation inducer in the nucleus (Fragment)	F6RJ39	S428,S/T	SLSPLSGTTDTK	S6,S/T	0.782	0.289
Acin1	Apoptotic chromatin condensation inducer in the nucleus (Fragment)	F6RJ39	Ambiguous	EAVVDLHADDSRISEDETERNGDDGTHDK	S/T	1.034	0.593
Acly	ATP-citrate synthase	Q3V117	S455	TASFSESR	S3	1.059	0.062
Acly	ATP-citrate synthase	Q3V117	S455	TASFSESRADEVAPAK	S3	0.912	0.428
Acly	ATP-citrate synthase	Q3V117	S481	AKPAMPQDSVPSPR	S12	0.995	0.911
Acly	ATP-citrate synthase	Q3V117	S481	AKPAMPQDSVPSPR	S12	0.887	0.101
Aco1	Cytoplasmic aconitate hydratase	P28271	S711	EFNSYGSRR	S7	1.068	0.484
Aco2	Aconitate hydratase, mitochondrial	Q99KI0	S559	VDVSPTSQR	S4	1.013	0.731
Acot1	Acyl-coenzyme A thioesterase 1	O55137	S416	QLGSECLHVSPK	S10	1.048	0.536
Acot1	Acyl-coenzyme A thioesterase 1	O55137	S56	YRADSHGELDLAR	S5	1.028	0.527
Acot11	Acyl-coenzyme A thioesterase 11	A2AVR6	S45	SISHPESGDPPTMAEGEGYR	S1	1.019	0.842
Acss2	Acetyl-coenzyme A synthetase, cytoplasmic	A2AQN4	S263	AELGMNDSPSQSPPVK	S8	1.066	0.317
Acss2	Acetyl-coenzyme A synthetase, cytoplasmic	A2AQN4	S30	GWSPPPEVR	S3	1.003	0.957
Acss2	Acetyl-coenzyme A synthetase, cytoplasmic	A2AQN4	S30	VRGWSPPPEVR	S5	1.109	0.415
Acss2	Acetyl-coenzyme A synthetase, cytoplasmic	A2AQN4	Ambiguous	AELGMNDSPSQSPPVK	S	1.380	0.059
Actb	Actin, cytoplasmic 1	P60710	S199	GYSFTTTAER	S3	1.040	0.540
Actb	Actin, cytoplasmic 1	P60710; P62737	S239;S241	SYELPDGQVITIGNER	S1	0.940	0.445
Actb	Actin, cytoplasmic 1	P60710	S368	QEYDESGPSIVHR	S9	0.968	0.680
Actb	Actin, cytoplasmic 1	P60710	S368	QEYDESGPSIVHRK	S9	0.948	0.380
Actb	Actin, cytoplasmic 1	P60710; P62737	S52;S54	DSYVGDEAQSK	S2	1.045	0.416
Actb	Actin, cytoplasmic 1	P60710; P62737	S60;S62	DSYVGDEAQSKR	S10	0.986	0.732
Actb	Actin, cytoplasmic 1	P60710; P62737	T318;T320	EITALAPSTMK	T3	1.030	0.542
Actb	Actin, cytoplasmic 1	P60710	Y294	DLYANTVLSGGTTMYPGIADR	Y3	0.837	0.621
Actl6a	Actin-like protein 6A	Q9Z2N8	S233	EGSPANWK	S3	0.970	0.784
Actn4	Alpha-actinin-4	P57780; Q7TPR4	S160;S140	FAIQDISVEETSAK	S7	1.034	0.832
Actr2	Actin-related protein 2	P61161	S207	GYAFNHSADFETVR	S7	0.943	0.401
Actr2	Actin-related protein 2	P61161	Y325	QLYLER	Y3	1.033	0.275
Actr3	Actin-related protein 3	Q99JY9	S220	DREVGIPPEQSLETAK	S11	0.974	0.505
Actr3	Actin-related protein 3	Q99JY9	Y109	AEPEDHYFLLTEPPLNTPENR	Y7	0.845	0.152
Actr3	Actin-related protein 3	Q99JY9	Y231	YSYVCPDLVK	Y1	1.141	0.407
Actr8	Actin-related protein 8	Q8R2S9	S132	IPVSPEQTR	S4	0.991	0.920
Actr8	Actin-related protein 8	Q8R2S9	S132	RIPVSPEQTR	S5	1.005	0.944
Acvr1b	Activin receptor type-1B	Q61271	S168	QRLDMEDPSCEMCLSK	S9	1.229	0.010
Adam17	Disintegrin and metalloproteinase domain-containing protein 17	E9PXU2	S813	SFEDLTDHPVTR	S1	0.994	0.836
Adar	Double-stranded RNA-specific adenosine deaminase	Q99MU3	S231	GEPGLDSEDGDPASDLEGPSEPLDMAEIK	S7	1.020	0.681
Adar	Double-stranded RNA-specific adenosine deaminase	Q99MU3	Ambiguous	ALQEEAASSADDQSGGANTDSLDESMAPNK	S/T	1.021	0.732
Adcy2	Adenylate cyclase	Q3V1Q3	S1089	SLSQSNLAS	S3	1.036	0.523
Adcy7	Adenylate cyclase type 7	P51829	T513	ESVSSSETPISNGR	T8	1.006	0.927
Adcy9	Adenylate cyclase type 9	P51830	S1259	CTDNGVVPQHQLSISPDIR	S15	1.076	0.513
Add1	Alpha-adducin	Q9QYC0; F8WGR0	S465;S465	GDDASEEGQNGSSPK	S13	0.951	0.583
Add1	Alpha-adducin	Q9QYC0; F8WGR0	S465;S465	WLHSGRGDDASEEGQNGSSPK	S19	0.992	0.924
Add1	Alpha-adducin	Q9QYC0; F8WGR0	S586;S617	QKGSEENLDETR	S4	1.123	0.187
Add1	Alpha-adducin	Q9QYC0	S605	SPPDQSAVPNTPPSTPVKLEEDLPQEPTSR	S6	0.946	0.067
Add1	Alpha-adducin	Q9QYC0; F8WGR0	T358;T358	SPGTPAGEGSGSPPK	T4	1.064	0.181
Add1	Alpha-adducin	Q9QYC0; F8WGR0	T610;T641	SPPDQSAVPNTPPSTPVK	T11	0.968	0.420
Add1	Alpha-adducin	Q9QYC0	S613,T614	EKSPPDQSAVPNTPPSTPVKLEEDLPQEPTSR	S16,T17	0.949	0.553
Add1	Alpha-adducin	Q9QYC0	T610,S/T	SPPDQSAVPNTPPSTPVKLEEDLPQEPTSR	T11,S/T	1.177	0.005
Add1	Alpha-adducin	Q9QYC0; F8WGR0	Ambiguous	YSDVEVPASVTGHSFASDGDSGTCSPLR	S/T/Y	0.929	0.639
Add1	Alpha-adducin	Q9QYC0; F8WGR0	Ambiguous	AAVVTSPPPTTAPHKER	S/T	1.043	0.657
Add1	Alpha-adducin	F8WGR0	Ambiguous	SPPDQSAVPNTPPSTPVKLEEAGDGCAK	S/T	0.954	0.788
Add1	Alpha-adducin	Q9QYC0; F8WGR0	Ambiguous	AAVVTSPPPTTAPHK	T/S	1.188	0.014
Add3	Gamma-adducin	Q9QYB5	S681	SPERTEEVLSPDGSPSKSPSK	S18	0.954	0.184
Add3	Gamma-adducin	Q9QYB5	S677,S681	SPERTEEVLSPDGSPSKSPSK	S14,S18	0.922	0.276
Add3	Gamma-adducin	Q9QYB5	S677,S/T	TEEVLSPDGSPSKSPSK	S10,S/T	1.021	0.913
Add3	Gamma-adducin	Q9QYB5	Ambiguous	TEEVLSPDGSPSKSPSK	S/T	0.984	0.876
Adgrl2	Adhesion G protein-coupled receptor L2	A0A0G2JGM8	S1458	GNSDGYIIPINK	S3	1.150	0.275
Adgrl2	Adhesion G protein-coupled receptor L2	A0A0G2JGM8	S1486	EGQMQLVTSL	S9	1.435	0.115
Adgrl2	Adhesion G protein-coupled receptor L2	A0A0G2JGM8	Ambiguous	DSLYTSMPNLR	T/S/Y	1.005	0.969
Adnp	Activity-dependent neuroprotector homeobox protein	Q9Z103	S904	IPSDNLEEPVPK	S3	0.949	0.236
Adnp2	Activity-dependent neuroprotector homeobox protein 2	Q8CHC8	S1036	DGEEPQLTLDADASSGSEK	S15	1.103	0.290
Adra1a	Alpha-1A adrenergic receptor	P97718	S458	IHTISLGENGEEV	S5	1.084	0.708
Aebp2	Zinc finger protein AEBP2	Q9Z248	S131	SLSPGAASSSSGDGDGKEGLEEPK	S3	1.078	0.081
Aebp2	Zinc finger protein AEBP2	Q9Z248	S131	SLSPGAASSSSGDGDGK	S3	1.096	0.069
Aebp2	Zinc finger protein AEBP2	Q9Z248	S199	RGSLEMSSDGEPLSR	S3	1.038	0.263
Aebp2	Zinc finger protein AEBP2	Q9Z248	S199	RGSLEMSSDGEPLSR	S3	0.984	0.870
Aebp2	Zinc finger protein AEBP2	Q9Z248	S24	LSPLSPGSPGPAAR	S8	1.023	0.476
Aes	Amino-terminal enhancer of split	P63002	S196	EDKNGHDGDTHQEDDGEKSD	S19	1.037	0.718
Afap1	Actin filament-associated protein 1	Q80YS6	S549	TASNAEQYK	S3	1.016	0.608
Afap1	Actin filament-associated protein 1	Q80YS6	S669	SGTSSPQSPVFR	S8	1.031	0.272
Afdn	Afadin	E9Q9C3	S1189	SSPNVANQPPSPGGKGPYTSGTAAK	S11	0.963	0.587
Afdn	Afadin	E9Q9C3	S1189	SSPNVANQPPSPGGK	S11	1.024	0.642
Afdn	Afadin	E9Q9C3	S1282	SQEELREEK	S1	1.035	0.700
Afdn	Afadin	E9Q9C3	S1282	VTRSQEELREEK	S4	0.955	0.369
Afdn	Afadin	E9Q9C3	S1777	SQDADLPGSSGAPENLTFK	S1	1.060	0.511
Afdn	Afadin	E9Q9C3	S1802	LFSQGQDVSDK	S3	1.163	0.020
Afdn	Afadin	E9Q9C3	S216	TISNPEVVMK	S3	0.973	0.456
Afdn	Afadin	E9Q9C3	S557	LDSDRVSSASSTAER	S3	1.002	0.970
Afdn	Afadin	E9Q9C3	T1494	ELQPQQQPRTIER	T10	1.033	0.739
Afdn	Afadin	E9Q9C3	S1179,S1189	SSPNVANQPPSPGGK	S1,S11	1.020	0.520
Afdn	Afadin	E9Q9C3	S1189,S	ADHRSSPNVANQPPSPGGK	S15,S	1.062	0.463
Afdn	Afadin	E9Q9C3	S1218,S/T/Y	ITSVSTGNLCTEEQSPPPRPEAYPIPTQTYTR	S15,T/S/Y	1.333	0.490
Afdn	Afadin	E9Q9C3	Ambiguous	TSSVVTLEVAK	S/T	1.003	0.946
Afdn	Afadin	E9Q9C3	Ambiguous	TISNPEVVMK	T/S	1.034	0.864
Aff1	AF4/FMR2 family member 1	E9Q921	S1106	VAQAPSPCTAR	S6	1.042	0.426
Aff1	AF4/FMR2 family member 1	E9Q921	Ambiguous	GSTESPQWR	T/S	1.036	0.522
Aff4	AF4/FMR2 family member 4	Q9ESC8	S1040	NSYSNSQAPSPGLGSK	S10	0.983	0.863
Aff4	AF4/FMR2 family member 4	Q9ESC8	S831	TVSQSSSLK	S3	1.227	0.050
Aff4	AF4/FMR2 family member 4	Q9ESC8	T516	EQGTASNYTDPGGTKETSSATPGRDSK	T14	0.903	0.382

Aff4	AF4/FMR2 family member 4	Q9ESC8	Ambiguous	SKSPRDPDANWDSPSR	S	1.229	0.273
Aftph	Aftiphilin	Q80WT5	S151	SFSPGDFR	S3	1.016	0.733
Aftph	Aftiphilin	Q80WT5	S412	SPDPDPTGQNALDDSAASMK	S18	1.037	0.298
Aftph	Aftiphilin	Q80WT5	S506	QTSDGLSEECPLAGESGGK	S7	0.944	0.064
Agap1	Arf-GAP with GTPase, ANK repeat and PH domain-containing protein 1	Q8BXK8; F8VQE9	S101;S156	YLTGTYVQEESPEGGR	S11	1.033	0.362
Agap1	Arf-GAP with GTPase, ANK repeat and PH domain-containing protein 1	Q8BXK8; F8VQE9	S315;S371	SNIFTSR	S1	0.904	0.050
Agap1	Arf-GAP with GTPase, ANK repeat and PH domain-containing protein 1	Q8BXK8	S422	ATSACAPISSPK	S10	0.993	0.897
Agap1	Arf-GAP with GTPase, ANK repeat and PH domain-containing protein 1	Q8BXK8	S521	LDPPPSPHANR	S6	1.024	0.335
Agap1	Arf-GAP with GTPase, ANK repeat and PH domain-containing protein 1	Q8BXK8	T303	IDVPPTANTPTPVR	T9	0.988	0.714
Agap2	Arf-GAP with GTPase, ANK repeat and PH domain-containing protein 2	Q3UHD9	S802	ALSTDCTPSGDLSPLSR	S3	1.222	0.210
Agap3	Arf-GAP with GTPase, ANK repeat and PH domain-containing protein 3	F8VQE9	S573	LEPPPSPHSNRK	S6	1.014	0.949
Agap3	Arf-GAP with GTPase, ANK repeat and PH domain-containing protein 3	F8VQE9	S573	LEPPPSPHSNR	S6	1.001	0.987
Agap3	Arf-GAP with GTPase, ANK repeat and PH domain-containing protein 3	F8VQE9	T359	IETIAASSTPTPIR	T9	0.993	0.884
Agap3	Arf-GAP with GTPase, ANK repeat and PH domain-containing protein 3	F8VQE9	Ambiguous	ATPTTAPGTSPR	T/S	1.041	0.092
Agfg1	Arf-GAP domain and FG repeat-containing protein 1 (Fragment)	A0A087WR52	S178	GTPSQSPVVGR	S6	1.014	0.455
Agfg1	Arf-GAP domain and FG repeat-containing protein 1 (Fragment)	A0A087WR52	T174,S178	GTPSQSPVVGR	T2,S6	1.009	0.786
Aggf1	Angiogenic factor with G patch and FHA domains 1	Q7TN31	S308	VCSVEYINCSEDEHSGNVK	S10	0.810	0.004
Ago2	Protein argonaute-2	Q8CJG0	S388	SASFNTDPYVR	S3	1.134	0.185
Agps	Alkylglycerone-phosphate synthase	A2AL50	S78	RAASAAGASPAATPAAPESGTIPK	S4	1.006	0.880
Agps	Alkylglycerone-phosphate synthase	A2AL50	S83	AASAAGASPAATPAAPESGTIPK	S8	0.989	0.843
Agps	Alkylglycerone-phosphate synthase	A2AL50	S78,S83	RAASAAGASPAATPAAPESGTIPK	S4,S9	0.826	0.199
Agps	Alkylglycerone-phosphate synthase	A2AL50	S83,T/S	AASAAGASPAATPAAPESGTIPK	S8,T/S	1.066	0.816
Agtpbp1	Cytosolic carboxypeptidase 1	Q641K1	S217,T226	NSGLMKVALDTLAALLK	S2,T11	0.974	0.601
Agtpbp1	Cytosolic carboxypeptidase 1	Q641K1	Ambiguous	VTSPTTYVLDEDEPR	T/S/Y	1.000	1.000
Ahctf1	Protein ELYS	Q8CJF7	S1507	ENVGLPEESPR	S9	1.090	0.076
Ahctf1	Protein ELYS	Q8CJF7	S1792	VAPENQLTAQSPPAPR	S11	0.964	0.460
Ahctf1	Protein ELYS	Q8CJF7	S1928	EVSVSSVTEEPK	S3	1.054	0.510
Ahctf1	Protein ELYS	Q8CJF7	T1824	DVSQGTLPSSGAVEPEPEPQGTPGR	T22	0.892	0.396
Ahctf1	Protein ELYS	Q8CJF7	T2067	DGLLAAAALTPSAQGTR	T10	1.203	0.552
Ahctf1	Protein ELYS	Q8CJF7	S1214,S1218	TTPLASPSLSPGR	S6,S10	1.219	0.195
Ahctf1	Protein ELYS	Q8CJF7	S1218,S/T	SGFRTTPLASPSLSPGR	S14,S/T	1.165	0.035
Ahctf1	Protein ELYS	Q8CJF7	Ambiguous	TTPLASPSLSPGR	S/T	1.032	0.367
Ahcyl1	S-adenosylhomocysteine hydrolase-like protein 1	Q80SW1	S68	SISQSSTDSYSSAASYTDSSDDEVSPR	S3	0.850	0.402
Ahcyl2	Putative adenosylhomocysteinase 3	Q68FL4	S109	HRDGGEALVSPDGTVTEAPR	S10	1.039	0.512
Ahcyl2	Putative adenosylhomocysteinase 3	Q68FL4	S109	DGGEALVSPDGTVTEAPR	S8	1.048	0.528
Ahcyl2	Putative adenosylhomocysteinase 3	Q68FL4	S151	SISQSSTDSYSSAASYTDSSDDETSPR	S3	0.883	0.438
Ahdc1	AT-hook DNA-binding motif-containing protein 1	Q6PAL7	S1060	ASTVSPGGYMVPK	S5	0.895	0.247
Ahdc1	AT-hook DNA-binding motif-containing protein 1	Q6PAL7	S593	LASPQPSYAADANDSK	S3	1.077	0.483
Ahdc1	AT-hook DNA-binding motif-containing protein 1	Q6PAL7	S892	ASPVAVGSSGAGADPSFQPVLPSR	S2	1.000	1.000
Ahnak	AHNAK nucleoprotein (desmoyokin)	E9Q616	S116	SSEVVLSGDDEDYQR	S7	1.030	0.517
Ahnak	AHNAK nucleoprotein (desmoyokin)	E9Q616	S1169	LDIETSDVSLEGPEGK	S9	1.105	0.296
Ahnak	AHNAK nucleoprotein (desmoyokin)	E9Q616	S1283;S2686;S4536;S6789;S9311;S11961;S14737;S17641;S21125;S24737;S28603;S32595;S36715;S4	ADLDVSGPK	S6	1.065	0.485
Ahnak	AHNAK nucleoprotein (desmoyokin)	E9Q616	S136	LRSEDGVEGDLGETQSR	S3	1.315	0.000
Ahnak	AHNAK nucleoprotein (desmoyokin)	E9Q616	S217	LPSGSGPASPTTGSAVDIR	S9	1.028	0.517
Ahnak	AHNAK nucleoprotein (desmoyokin)	E9Q616	S232	AGAISASGPELEGAGHSK	S5	1.015	0.487
Ahnak	AHNAK nucleoprotein (desmoyokin)	E9Q616	S42	DDGVFVQEVMQNSPAAR	S13	1.012	0.781
Ahnak	AHNAK nucleoprotein (desmoyokin)	E9Q616	S4857	AEVKSPK	S5	1.006	0.945
Ahnak	AHNAK nucleoprotein (desmoyokin)	E9Q616	S4890	AEAPLPSPK	S7	1.059	0.489
Ahnak	AHNAK nucleoprotein (desmoyokin)	E9Q616	S4890	FKAEAPLPSPK	S9	1.169	0.349
Ahnak	AHNAK nucleoprotein (desmoyokin)	E9Q616	S5099	GDLGASSPSMK	S7	1.060	0.611
Ahnak	AHNAK nucleoprotein (desmoyokin)	E9Q616	S5099	GDLGASSPSMK	S7	0.987	0.628
Ahnak	AHNAK nucleoprotein (desmoyokin)	E9Q616	S5504	GGVTGSPEASISGSK	S6	1.063	0.100
Ahnak	AHNAK nucleoprotein (desmoyokin)	E9Q616	S5504	GKGGVTGSPEASISGSK	S8	0.981	0.560
Ahnak	AHNAK nucleoprotein (desmoyokin)	E9Q616	S5525	ASLGSLEGEVEAEASSPK	S5	1.067	0.348
Ahnak	AHNAK nucleoprotein (desmoyokin)	E9Q616	S5536	ASLGSLEGEVEAEASSPKGK	S16	0.934	0.684
Ahnak	AHNAK nucleoprotein (desmoyokin)	E9Q616	S5555	SNSFSDER	S3	1.009	0.853
Ahnak	AHNAK nucleoprotein (desmoyokin)	E9Q616	S5607	SKGHYEVTGSDDEAGK	S10	0.933	0.796
Ahnak	AHNAK nucleoprotein (desmoyokin)	E9Q616	S893	AESPEMEVNLPK	S3	1.110	0.032
Ahnak	AHNAK nucleoprotein (desmoyokin)	E9Q616	S893	AESPEMEVNLPK	S3	1.097	0.289
Ahnak	AHNAK nucleoprotein (desmoyokin)	E9Q616	S94	SPEPGQTWTHEVFSSR	S1	0.963	0.812
Ahnak	AHNAK nucleoprotein (desmoyokin)	E9Q616	S94	GDRSPEPGQTWTHEVFSSR	S4	1.089	0.489
Ahnak	AHNAK nucleoprotein (desmoyokin)	E9Q616	T159	RVTAYTVDVTGR	T3	1.007	0.911
Ahnak	AHNAK nucleoprotein (desmoyokin)	E9Q616	T3094	VQTPEVDVK	T3	0.998	0.965
Ahnak	AHNAK nucleoprotein (desmoyokin)	E9Q616	T4950	VQANLDTPDINIEGPEAK	T7	1.344	0.004
Ahnak	AHNAK nucleoprotein (desmoyokin)	E9Q616	S211,S217	TVIRLPSGSGPASPTTGSAVDIR	S7,S13	1.201	0.090
Ahnak	AHNAK nucleoprotein (desmoyokin)	E9Q616	S213,S217	LPSGSGPASPTTGSAVDIR	S5,S9	1.058	0.162
Ahnak	AHNAK nucleoprotein (desmoyokin)	E9Q616	S5504,S5508	GGVTGSPEASISGSK	S6,S10	0.969	0.721
Ahnak	AHNAK nucleoprotein (desmoyokin)	E9Q616	S5504,S5512	GGVTGSPEASISGSKGDLK	S6,S14	1.076	0.384
Ahnak	AHNAK nucleoprotein (desmoyokin)	E9Q616	S5525,S5536	ASLGSLEGEVEAEASSPK	S5,S16	1.070	0.511
Ahnak	AHNAK nucleoprotein (desmoyokin)	E9Q616	S5525,S5536	ASLGSLEGEVEAEASSPKGK	S5,S16	0.993	0.954
Ahnak	AHNAK nucleoprotein (desmoyokin)	E9Q616	S5563,S/T	SNSFSDEREFSAPSTPTGTLEFAGGDAK	S11,S/T	0.912	0.072
Ahnak	AHNAK nucleoprotein (desmoyokin)	E9Q616	S5563,S/T	EFSAPSTPTGTLEFAGGDAK	S3,S/T	1.065	0.214
Ahnak	AHNAK nucleoprotein (desmoyokin)	E9Q616	S5563,S/T	SNSFSDEREFSAPSTPTGTLEFAGGDAK	S11,S/T	0.908	0.064
Ahnak	AHNAK nucleoprotein (desmoyokin)	E9Q616	Ambiguous	EFSAPSTPTGTLEFAGGDAK	S/T	0.925	0.484
Ahnak	AHNAK nucleoprotein (desmoyokin)	E9Q616	Ambiguous	LSSSSSNDSGTK	S/T	1.211	0.233
Ahnak	AHNAK nucleoprotein (desmoyokin)	E9Q616	Ambiguous	SNSFSDEREFSAPSTPTGTLEFAGGDAK	S/T	1.019	0.534
Ahnak	AHNAK nucleoprotein (desmoyokin)	E9Q616	Ambiguous	GHYEVTGSDDEAGK	T/S/Y	1.028	0.720
Ahnak	AHNAK nucleoprotein (desmoyokin)	E9Q616	Ambiguous	GHYEVTGSDDEAGKLQGSGVSLASK	T/S/Y	1.026	0.771
Ahnak2	AHNAK nucleoprotein 2 (Fragment)	E9PYB0	S286	DMSPTSTDTEVHR	S3	0.982	0.635
Ahnak2	AHNAK nucleoprotein 2 (Fragment)	E9PYB0	S286	DMSPTSTDTEVHR	S3	0.972	0.726
Ahnak2	AHNAK nucleoprotein 2 (Fragment)	E9PYB0	S326	FGMTSGQSPDTSEQK	S8	1.020	0.780
Ahnak2	AHNAK nucleoprotein 2 (Fragment)	E9PYB0; F7DBB3	2;S2290;S3389;S4661;S6106;S228;S	VQMPSLK	S5	1.035	0.444
Ahnak2	AHNAK nucleoprotein 2 (Fragment)	E9PYB0	S289,T290	DMSPTSTDTEVHR	S6,T7	1.024	0.925
Ahnak2	AHNAK nucleoprotein 2 (Fragment)	F7DBB3	S1152	TGSLPSQPR	S3	1.031	0.788
Ahnak2	AHNAK nucleoprotein 2 (Fragment)	F7DBB3	S1261	MPPASLEQSLEASPISK	S13	0.989	0.873
Ahnak2	AHNAK nucleoprotein 2 (Fragment)	F7DBB3	S1544	ESFSPEEEEEAKSEGTSARPGSEAMVASSAR	S22	0.867	0.217
Ahnak2	AHNAK nucleoprotein 2 (Fragment)	F7DBB3	S907	DVTTELHVRSPSTTAELVR	S10	0.830	0.179
Ahnak2	AHNAK nucleoprotein 2 (Fragment)	F7DBB3	S909	SPSTTAELVR	S3	0.976	0.589
Ahnak2	AHNAK nucleoprotein 2 (Fragment)	F7DBB3	Ambiguous	DTTQRSPVHVQPTAR	T/S	0.950	0.473
Ahsa2	Activator of 90 kDa heat shock protein ATPase homolog 2	Q8N9S3	S189	LQASPVALGVR	S4	0.920	0.423
AI467606	Transmembrane protein C16orf54 homolog	Q8C708	S195	QGSLEPDWGLQPR	S3	1.032	0.699
AI467606	Transmembrane protein C16orf54 homolog	Q8C708	T113	APGPPTPGGTLEGR	T6	1.030	0.318
AI467606	Transmembrane protein C16orf54 homolog	Q8C708	T113,T117	APGPPTPGGTLEGR	T6,T10	1.039	0.363
AI837181	Expressed sequence AI837181	E9QNR6	S36	ARSWVGAER	S3	1.048	0.198
Aifm1	Apoptosis-inducing factor 1, mitochondrial	Q9Z0X1	S115	AIASATEGGSVPQIR	S4	0.861	0.497
Aimp2	Aminoacyl tRNA synthase complex-interacting multifunctional protein 2	Q8R010	Ambiguous	TTSPATDAGHVQETSEPSLQALESR	T/S	0.556	0.422
Ajuba	LIM domain-containing protein ajuba	Q91XC0	S129	LETTAPALSPR	S9	0.966	0.705
Ak3	GTP:AMP phosphotransferase AK3, mitochondrial	Q9WTP7	S224	VPETTQKASVTP	S9	0.964	0.674
Akap1	A-kinase anchor protein 1, mitochondrial	O08715	S101	RRSESSGNLPSVADTR	S3	1.010	0.815
Akap1	A-kinase anchor protein 1, mitochondrial	O08715	S103	SESSGNLPSVADTR	S3	0.985	0.810
Akap1	A-kinase anchor protein 1, mitochondrial	O08715	S378	TGATASPSAEALPPK	S6	1.137	0.039
Akap1	A-kinase anchor protein 1, mitochondrial	O08715	Ambiguous	RSESSGNLPSVADTR	S/T	1.147	0.367
Akap10	A-kinase anchor protein 10, mitochondrial	O88845	S173	AHSLNTVK	S3	1.046	0.534
Akap10	A-kinase anchor protein 10, mitochondrial	O88845	S187	QSSLAEPVSPSK	S9	1.061	0.180
Akap10	A-kinase anchor protein 10, mitochondrial	O88845	S187	QSSLAEPVSPSKR	S9	1.011	0.748
Akap10	A-kinase anchor protein 10, mitochondrial	O88845	S52	ASVAVHSPQK	S7	0.956	0.292
Akap10	A-kinase anchor protein 10, mitochondrial	O88845	Ambiguous	NSCSSPLRELSEK	S	0.948	0.769
Akap11	A kinase (PRKA) anchor protein 11	E9Q774	S1163	SLSEEVESSEGEEHPGMHVK	S3	1.104	0.049
Akap11	A kinase (PRKA) anchor protein 11	E9Q774	S1163	SLSEEVESSEGEEHPGMHVK	S3	1.000	1.000
Akap11	A kinase (PRKA) anchor protein 11	E9Q774	S133	VSPTLPGLR	S2	1.003	0.979
Akap11	A kinase (PRKA) anchor protein 11	E9Q774	S244	VLVSYGSPK	S7	0.944	0.155
Akap11	A kinase (PRKA) anchor protein 11	E9Q774	S910	ACSPSLCDQAAPQENK	S3	0.977	0.409
Akap11	A kinase (PRKA) anchor protein 11	E9Q774	S987	FLDFSDNSPHR	S8	1.019	0.916
Akap11	A kinase (PRKA) anchor protein 11	E9Q774	T272	TSVTTSVSEPWTQR	T5	0.848	0.115
Akap12	A-kinase anchor protein 12	Q9WTQ5	S584	GPSEAPQEAEAEEGATSDGEK	S17	0.987	0.921
Akap12	A-kinase anchor protein 12	Q9WTQ5	S637	SATLSSTESTASGMQDEVR	S12	1.074	0.481
Akap13	A-kinase anchor protein 13	A0A140LJJ5	S1253	VETQNPSLSSPETK	S10	0.954	0.427
Akap13	A-kinase anchor protein 13	A0A140LJJ5	S1631	NSLMSLSEEHLEPDQR	S7	0.988	0.633
Akap13	A-kinase anchor protein 13	A0A140LJJ5	S1631	NSLMSLSEEHLEPDQR	S7	1.020	0.746
Akap13	A-kinase anchor protein 13	A0A140LJJ5	S1838	GSLQAHDTSSLPTVIMR	S10	1.212	0.182
Akap13	A-kinase anchor protein 13	A0A140LJJ5	S1857	SAVLLADEATAAPMFTNRR	S1	0.870	0.017
Akap13	A-kinase anchor protein 13	A0A140LJJ5	S1857	SAVLLADEATAAPMFTNR	S1	0.964	0.756
Akap13	A-kinase anchor protein 13	A0A140LJJ5	S1857	ERPRSAVLLADEATAAPMFTNR	S5	1.034	0.527
Akap13	A-kinase anchor protein 13	A0A140LJJ5	S1876	SQQSVSLSK	S1	0.970	0.558
Akap13	A-kinase anchor protein 13	A0A140LJJ5	S1910	FLSHSTDSLNK	S5	0.928	0.580
Akap13	A-kinase anchor protein 13	A0A140LJJ5	S1926	VNESTESLTDEGVGTDMNEGQLMGDFESDSK	S7	0.856	0.005
Akap13	A-kinase anchor protein 13	A0A140LJJ5	S2379	DMTECSTPLPEDCSPTHSPR	S14	0.996	0.957
Akap13	A-kinase anchor protein 13	A0A140LJJ5	S2480	RTESDSGLK	S4	0.987	0.812
Akap13	A-kinase anchor protein 13	A0A140LJJ5	S2545	SASRPSSLIEQEK	S3	1.057	0.167
Akap13	A-kinase anchor protein 13	A0A140LJJ5	S2710	SGSLDSELSVSPK	S11	0.929	0.375
Akap13	A-kinase anchor protein 13	A0A140LJJ5	T1338	ISAASEEEAVGNGAATPK	T16	0.990	0.809
Akap13	A-kinase anchor protein 13	A0A140LJJ5	T2392	SNTEEALK	T3	1.174	0.022
Akap13	A-kinase anchor protein 13	A0A140LJJ5	S1626,S1631	NPFGGEEQRNSLMSLSEEHLEPDQR	S11,S16	0.932	0.218
Akap13	A-kinase anchor protein 13	A0A140LJJ5	S2545,S2549	SASRPSSLIEQEK	S3,S7	0.934	0.446
Akap13	A-kinase anchor protein 13	A0A140LJJ5	S579,S/T	GCEGGTTSDQSSPVLPAAIENK	S8,S/T	1.003	0.984
Akap13	A-kinase anchor protein 13	A0A140LJJ5	T2372,S2383	DMTECSTPLPEDCSPTHSPR	T7,S18	0.651	0.294

Akap13	A-kinase anchor protein 13	A0A140LJJ5	Ambiguous	GCEGGTTSDQSSPVLPAAAIENK	S/T	1.099	0.328
Akap17b	A-kinase anchor protein 17B	A2A3V1	Ambiguous	DIASETDEHLSPTDGESYPVEK	T/S/Y	1.013	0.966
Akap7	A-kinase anchor protein 7 isoform alpha (Fragment)	H3BJV2	S54	LDSGTCEHISGEEEVAR	S10	0.956	0.336
Akap8	A-kinase anchor protein 8	Q9DBR0	S325	ADSDGDLSENDDGAGDLR	S8	0.947	0.600
Akap8	A-kinase anchor protein 8	Q9DBR0	S336	SGDEEFRGEDDLCDSR	S1	1.038	0.445
Akap8	A-kinase anchor protein 8	Q9DBR0	S320,S325	LARADSDGDLSENDDGAGDLR	S6,S11	1.022	0.820
Akap8l	A kinase (PRKA) anchor protein 8-like	Q5RL57	S284	QGGSPDEPDSK	S4	1.029	0.659
Akap8l	A kinase (PRKA) anchor protein 8-like	Q5RL57	S284	KQGGSPDEPDSK	S5	1.089	0.130
Akap8l	A kinase (PRKA) anchor protein 8-like	Q5RL57	S347	EGKEDSEKGALTAQDESSQAK	S6	0.981	0.723
Akap9	A-kinase anchor protein 9	E9QQ10	S1289	QVQELQSPVAAGQLQLTETEANHR	S7	0.955	0.081
Akip1	A-kinase-interacting protein 1	Q9JJR5	S41	AVDWHSPER	S6	0.986	0.666
Akna	AT-hook-containing transcription factor	Q80VW7	S1149	LYLSSESESPAPR	S9	1.022	0.890
Akna	AT-hook-containing transcription factor	Q80VW7	S302	FTGSVSPLSTR	S6	1.033	0.587
Akna	AT-hook-containing transcription factor	Q80VW7	S825	DATQEDEEQMGPMKSPDFRPSMAR	S15	1.042	0.770
Akna	AT-hook-containing transcription factor	Q80VW7	T244	NGGDVAPATPTEFR	T9	0.911	0.484
Akna	AT-hook-containing transcription factor	Q80VW7	T838	DTYTPVLDTAEVAQR	T4	1.104	0.614
Akr1a1	Alcohol dehydrogenase [NADP(+)]	Q9JII6	S38	HALSAGYR	S4	0.975	0.759
Akr1a1	Alcohol dehydrogenase [NADP(+)]	Q9JII6	Y210	GLEVTAYSPLGSSDR	Y7	0.916	0.525
Akt1	RAC-alpha serine/threonine-protein kinase	P31750	S124	SGSPSDNSGAEEMEVSLAKPK	S3	0.974	0.341
Akt1	RAC-alpha serine/threonine-protein kinase	P31750	S129	SGSPSDNSGAEEMEVSLAKPK	S8	1.012	0.767
Akt1s1	Proline-rich AKT1 substrate 1	E9QKI4	S284	SSDEENGPPSSPDLDR	S11	1.026	0.553
Akt1s1	Proline-rich AKT1 substrate 1	E9QKI4	S284	SSDEENGPPSSPDLDRIAASMR	S11	1.012	0.867
Akt1s1	Proline-rich AKT1 substrate 1	E9QKI4	T318	LNTSDFQK	T3	0.952	0.189
Akt1s1	Proline-rich AKT1 substrate 1	E9QKI4	S274,S/T	TEARSSDEENGPPSSPDLDRIAASMR	S5,S/T	1.005	0.956
Akt1s1	Proline-rich AKT1 substrate 1	E9QKI4	S275,S	SSDEENGPPSSPDLDR	S2,S	1.074	0.229
Akt1s1	Proline-rich AKT1 substrate 1	E9QKI4	S275,S/T	TEARSSDEENGPPSSPDLDR	S6,S/T	0.996	0.762
Akt1s1	Proline-rich AKT1 substrate 1	E9QKI4	Ambiguous	TEARSSDEENGPPSSPDLDR	S/T	0.960	0.636
Akt1s1	Proline-rich AKT1 substrate 1	E9QKI4	Ambiguous	SSDEENGPPSSPDLDRIAASMR	S	0.869	0.143
Akt2	RAC-beta serine/threonine-protein kinase	Q60823	S126	CGSPSDSSTSEMMEVAVNK	S3	1.054	0.460
Akt2	RAC-beta serine/threonine-protein kinase	Q60823	S126	CGSPSDSSTSEMMEVAVNK	S3	0.968	0.328
Akt2	RAC-beta serine/threonine-protein kinase	Q60823	S126	CGSPSDSSTSEMMEVAVNK	S3	1.286	0.104
Akt3	RAC-gamma serine/threonine-protein kinase	Q9WUA6	S120	MNCSPTSQIDNIGEEEMDASTTHHK	S4	1.050	0.686
Akt3	RAC-gamma serine/threonine-protein kinase	Q9WUA6	S34	TDGSFIGYK	S4	0.962	0.615
Alad	Delta-aminolevulinic acid dehydratase	P10518	S215	DAAQSSPAFGDR	S6	1.079	0.419
Alad	Delta-aminolevulinic acid dehydratase	P10518	S215	DAAQSSPAFGDRR	S6	0.970	0.587
Aldh3b1	Aldehyde dehydrogenase family 3 member B1	Q80VQ0	S279	FYGDNPQTSPNLGR	S9	0.976	0.895
Aldoa	Fructose-bisphosphate aldolase	A6ZI44	S100	LQSIGTENTEENRR	S3	1.041	0.029
Aldoa	Fructose-bisphosphate aldolase	A6ZI44	S100	LQSIGTENTEENR	S3	1.010	0.833
Aldoa	Fructose-bisphosphate aldolase	A6ZI44	S100	RLQSIGTENTEENRR	S4	1.046	0.232
Aldoa	Fructose-bisphosphate aldolase	A6ZI44	S100	RLQSIGTENTEENR	S4	0.979	0.309
Aldoa	Fructose-bisphosphate aldolase	A6ZI44	S186	GVVPLAGTNGETTTQGLDGLSER	S21	0.903	0.695
Aldoa	Fructose-bisphosphate aldolase	A6ZI44	S414	YTPSGQSGAAASESLFISNHAY	S18	1.256	0.334
Aldoa	Fructose-bisphosphate aldolase	A6ZI44	S93	GILAADESTGSIAK	S11	0.905	0.035
Aldoa	Fructose-bisphosphate aldolase	A6ZI44	S93	GILAADESTGSIAKR	S11	0.960	0.529
Alkbh5	RNA demethylase ALKBH5	Q3TSG4	S362	RGSFSSENYWR	S3	1.167	0.289
Alkbh5	RNA demethylase ALKBH5	Q3TSG4	S384,S/T/Y	KSYESSEDCPEAASSPTR	S14,S/T/Y	0.943	0.253
Alkbh5	RNA demethylase ALKBH5	Q3TSG4	Ambiguous	KSYESSEDCPEAASSPTR	S/T/Y	1.049	0.339
Alkbh5	RNA demethylase ALKBH5	Q3TSG4	Ambiguous	SYESSEDCPEAASSPTRK	S/Y/T	0.977	0.681
Alkbh5	RNA demethylase ALKBH5	Q3TSG4	Ambiguous	SYESSEDCPEAASSPTR	T/S/Y	1.058	0.308
Alms1	Alstrom syndrome protein 1 homolog	A0A1D5RMI8	S2257	SIEQSNPCFK	S1	1.082	0.674
Alox5ap	Arachidonate 5-lipoxygenase-activating protein	P30355	T45	TGTLAFER	T1	1.097	0.008
Alpl	Alkaline phosphatase, tissue-nonspecific isozyme	P09242	Ambiguous	TYNTNAQVPDSAGTATAYLCGVK	S/T/Y	1.180	0.357
Als2	Alsin	Q920R0	S460	SSSLMDIREEELEGGSR	S3	0.988	0.906
Alyref	THO complex subunit 4	O08583	S237	QQLSAEELDAQLDAYNAR	S4	1.024	0.841
Amer1	APC membrane recruitment protein 1	Q7TS75	S239,S246	MSPAQVHFSPTTEK	S2,S9	1.117	0.346
Amfr	E3 ubiquitin-protein ligase AMFR	Q9R049	S509	SDSLRPALNSPVERPSPDLEEGEASVQTER	S3	1.175	0.307
Amfr	E3 ubiquitin-protein ligase AMFR	Q9R049	S542	VPLDLSPR	S6	0.973	0.701
Ammecr1l	AMMECR1-like protein	Q8JZZ6	S74	ENVSDLTLGPGNSPITR	S13	1.065	0.694
Ampd2	AMP deaminase	A2AE27	S135	TDSDSDLQLYK	S3	0.974	0.781
Ampd2	AMP deaminase	A2AE27	S38	SGLGASPLQSAR	S6	0.925	0.237
Ampd2	AMP deaminase	A2AE27	Y90	SAPYEFPEESPIEQLEER	Y4	1.023	0.921
Ampd3	AMP deaminase	A0A1L1SRX2	S94	SQSLSLQMPTQQDWK	S3	1.229	0.017
Anapc1	Anaphase-promoting complex subunit 1	P53995	Ambiguous	VTSTPQKPQAEQEENR	T/S	1.168	0.073
Anapc2	Anaphase-promoting complex subunit 2	Q8BZQ7	T325	VFLQDNPTRPTSPEAGNTLR	T8	0.768	0.121
Ankfy1	Rabankyrin-5	Q810B6	S861	RESGAAEQVDNK	S3	1.004	0.912
Ankhd1	Ankyrin repeat and KH domain-containing 1	E9PUR0	S1592	GGGAGGNSDSDNIDSTDCNSESSSGGK	S8	1.068	0.170
Ankhd1	Ankyrin repeat and KH domain-containing 1 (Fragment)	F6RUI8	S614	GHNSPSSTTLQVGVAEGTEADR	S4	0.994	0.972
Ankhd1	Ankyrin repeat and KH domain-containing 1	E9PUR0	Ambiguous	ANVVTTPSTNR	T/S	1.016	0.761
Ankib1	Ankyrin repeat and IBR domain-containing protein 1	Q6ZPS6	S305	SGVQMPTPPPSGYNAWDTLPSPR	S21	1.134	0.463
Ankib1	Ankyrin repeat and IBR domain-containing protein 1	Q6ZPS6	S738	GVAPADSPDAPR	S7	1.066	0.114
Ankib1	Ankyrin repeat and IBR domain-containing protein 1	Q6ZPS6	S738	GVAPADSPDAPRR	S7	0.925	0.267
Ankib1	Ankyrin repeat and IBR domain-containing protein 1	Q6ZPS6	S938,T/S	LGADSDPFSTDTLSSRPLSETR	S19,S/T	1.056	0.759
Ankrd11	Ankyrin repeat domain-containing protein 11	E9Q4F8	S1697	EVLPASPR	S6	1.040	0.291
Ankrd11	Ankyrin repeat domain-containing protein 11	E9Q4F8	S859	FSLSDDQQER	S4	1.044	0.657
Ankrd12	Ankyrin repeat domain 12	G5E893	S1324	ETCLSPKPEVSSAVPER	S5	1.013	0.712
Ankrd12	Ankyrin repeat domain 12	G5E893	S561	ACISPGSSEMSLQPDLTR	S4	0.831	0.290
Ankrd12	Ankyrin repeat domain 12	G5E893	Ambiguous	SRSSELTDVYSK	S/T/Y	0.999	0.980
Ankrd12	Ankyrin repeat domain 12	G5E893	Ambiguous	DNSPDSTPSHPSQATPAQK	S/T	0.959	0.791
Ankrd12	Ankyrin repeat domain 12	G5E893	Ambiguous	GLSHVSDLHPNCTGSPTR	T/S	0.989	0.959
Ankrd13d	Ankyrin repeat domain-containing protein 13D	Q6PD24	S552	GPESPQKTPPSPAPPSFEEQLR	S4	1.058	0.842
Ankrd13d	Ankyrin repeat domain-containing protein 13D	Q6PD24	S552,T556	GPESPQKTPPSPAPPSFEEQLR	S4,T8	0.939	0.431
Ankrd17	Ankyrin repeat domain-containing protein 17	Q99NH0	S2043	EHYPVSSPSSPSPPAQPGGVSR	S12	1.042	0.208
Ankrd17	Ankyrin repeat domain-containing protein 17	Q99NH0	S2063	NSPLDCGSASPNK	S10	0.930	0.462
Ankrd17	Ankyrin repeat domain-containing protein 17	Q99NH0	S2401	APSPAPSSVPLGSEKPSSVSQDR	S3	0.952	0.108
Ankrd17	Ankyrin repeat domain-containing protein 17	Q99NH0	T1557	NNTITTTSSK	T3	1.016	0.943
Ankrd17	Ankyrin repeat domain-containing protein 17	Q99NH0	S1692,S	SGPSPLSSPNGK	S4,S	1.058	0.461
Ankrd17	Ankyrin repeat domain-containing protein 17	Q99NH0	S2041,S2043	EHYPVSSPSSPSPPAQPGGVSR	S10,S12	1.059	0.213
Ankrd33b	Ankyrin repeat domain-containing protein 33B	Q3U0L2	S21	CLSPQSPTPPR	S6	1.097	0.290
Ankrd34a	Ankyrin repeat domain 34A	B2RW11	S290	RHSTEGPEDPPPWAEK	S3	1.037	0.591
Ankrd34a	Ankyrin repeat domain 34A	B2RW11	S460	APSLPAPPPSGAPGSPR	S15	1.051	0.716
Ankrd50	Ankyrin repeat domain 50 (Fragment)	F7BE84	S1100	SNSSGGTGGGDLQPSLR	S3	1.628	0.005
Ankrd50	Ankyrin repeat domain 50 (Fragment)	F7BE84	S1146	SSLSNNSLK	S7	1.050	0.760
Ankrd50	Ankyrin repeat domain 50 (Fragment)	F7BE84	Ambiguous	SGSSPPKQTPAESQCK	S/T	0.961	0.262
Ankrd52	Serine/threonine-protein phosphatase 6 regulatory ankyrin repeat subunit C	Q8BTI7	S1075	HGAIGLDGCYSE	S11	1.010	0.839
Ankrd55	Ankyrin repeat domain-containing protein 55	Q8BLD6	S474	SRSEQDLLNNR	S3	1.034	0.365
Anks1	Ankyrin repeat and SAM domain containing 1	Q3UHP6	S461	IQSSAPQEEEEHPYELLLTAETK	S3	1.594	0.075
Anks1	Ankyrin repeat and SAM domain containing 1	Q3UHP6	S642	SLSKSDSDLLTCSPTEDATMGSR	S5	0.912	0.059
Anks1	Ankyrin repeat and SAM domain containing 1	Q3UHP6	S650	SDSDLLTCSPTEDATMGSR	S9	1.069	0.660
Anks1	Ankyrin repeat and SAM domain containing 1	Q3UHP6	S663	SESLSNCSIGK	S3	0.922	0.177
Anks1	Ankyrin repeat and SAM domain containing 1	Q3UHP6	S679	SPSFASEWDEIEK	S3	0.911	0.355
Anks1	Ankyrin repeat and SAM domain containing 1	Q3UHP6	S903	HDSLPDPGTASR	S3	0.992	0.863
Anks1	Ankyrin repeat and SAM domain containing 1	Q3UHP6	S903	RHDSLPDPGTASR	S4	1.088	0.385
Anks1	Ankyrin repeat and SAM domain containing 1	Q3UHP6	S903	RRHDSLPDPGTASR	S5	1.024	0.788
Anks1	Ankyrin repeat and SAM domain containing 1	Q3UHP6	S640,T648	SLSKSDSDLLTCSPTEDATMGSR	S3,T11	1.411	0.046
Anks1	Ankyrin repeat and SAM domain containing 1	Q3UHP6	Ambiguous	SLSKSDSDLLTCSPTEDATMGSR	S/T	1.024	0.678
Anks3	Ankyrin repeat and SAM domain-containing protein 3	Q9CZK6	Ambiguous	AQGLSSEASIESNEDSDHAR	S	1.139	0.135
Anks6	Ankyrin repeat and SAM domain-containing protein 6	Q6GQX6	Ambiguous	STSPTLTPSPSPK	T/S	1.019	0.770
Anks6	Ankyrin repeat and SAM domain-containing protein 6	Q6GQX6	Ambiguous	STSPTLTPSPSPK	T/S	0.979	0.850
Anln	Anillin	Q8K298	S180	AASPSKPPISNASATPVGR	S3	1.108	0.123
Anln	Anillin	Q8K298	Ambiguous	ASSPVTAATFITENR	S/T	0.974	0.854
Anln	Anillin	Q8K298	Ambiguous	DSNLSAASPKPGK	S	1.062	0.642
Anp32b	Acidic leucine-rich nuclear phosphoprotein 32 family member B	Q9EST5	T265	KRETDDEGEDD	T4	1.006	0.853
Antxr1	Anthrax toxin receptor 1	Q9CZ52	S360	EVPPPPVEESEEEDDDGLPK	S10	1.001	0.994
Anxa1	Annexin A1	P10107	S193	CQDLSVNQDLADTDAR	S5	0.760	0.056
Anxa1	Annexin A1	P10107	Ambiguous	DITSDTSGDFRK	T/S	1.030	0.528
Anxa1	Annexin A1	P10107	Ambiguous	DITSDTSGDFR	T/S	1.021	0.706
Anxa2	Annexin A2	P07356	S184	AEDGSVIDYELIDQDAR	S5	0.977	0.877
Anxa2	Annexin A2	P07356	S184	RAEDGSVIDYELIDQDAR	S6	0.917	0.004
Anxa2	Annexin A2	P07356	Y24	LSLEGDHSTPPSAYGSVKPYTNFDAER	Y14	0.982	0.961
Anxa2	Annexin A2	P07356	Y318	SLYYYIQQDTK	Y5	0.920	0.522
Anxa3	Annexin A3	O35639	S19	DYPGFSPSVDAEAIR	S6	1.092	0.234
Anxa3	Annexin A3	O35639	T267	GAGTDEFTLNR	T4	1.005	0.927
Anxa3	Annexin A3	O35639	Ambiguous	SLGDDISSETSGDFR	S/T	1.059	0.466
Anxa4	Annexin A4	P97429	S12	AASGFNATEDAQTLR	S3	1.050	0.462
Anxa5	Annexin A5	P48036; 155	Y92;Y93	LYDAYELK	Y5	0.970	0.684
Anxa6	Annexin	F8WIT2	S235	GELSGDFEK	S4	0.984	0.761
Ap1ar	AP1AR	E9PYF7	T201	STSGNDDSTSLDLEWEDEEGMNR	T9	0.918	0.555
Ap1ar	AP1AR	E9PYF7	T225	SKTEEDILR	T3	0.990	0.828
Ap1ar	AP1AR	E9PYF7	T225	ERSKTEEDILR	T5	1.082	0.347
Ap1b1	AP-1 complex subunit beta-1	O35643; H3BIY9	Y136;Y136	CLKDEPYVR	Y8	0.996	0.955
Ap1s1	AP-1 complex subunit sigma-1A	P61967	S147	AIEQADLLQEEDESPR	S14	0.944	0.697
Ap1s2	AP complex subunit sigma	Q3TIV9	T146	AIEQADLLQEEAETPR	T14	1.185	0.433
Ap2a1	AP-2 complex subunit alpha-1	P17426	S655	DTSSNDINGGVEPTPSTVSTPSPSADLLGLR	S22	1.116	0.273
Ap2a1	AP-2 complex subunit alpha-1	P17426	S655	RDTSSNDINGGVEPTPSTVSTPSPSADLLGLR	S23	0.964	0.889
Ap2m1	AP-2 complex subunit mu	P84091	T156	EEQSQITSQVTGQIGWR	T11	1.032	0.613
Ap3b1	AP-3 complex subunit beta-1	Q9Z1T1	S276	NFYESEEEEEKEK	S5	1.131	0.500
Ap3b1	AP-3 complex subunit beta-1	Q9Z1T1	Ambiguous	EDGGLEDNEKNFYESEEEEEKEK	S/Y	0.976	0.779
Ap3d1	AP-3 complex subunit delta-1	O54774	S760	RHSSLPTESDEDIAPAQR	S9	1.042	0.535

Ap3d1	AP-3 complex subunit delta-1	O54774	S760	HSSLPTESDEDIAPAQR	S8	1.049	0.208
Ap3d1	AP-3 complex subunit delta-1	O54774	S784	VDIITEEMPENALPSDEDDKDPNDPYR	S15	0.969	0.578
Ap3d1	AP-3 complex subunit delta-1	O54774	S784	VDIITEEMPENALPSDEDDKDPNDPYR	S15	1.030	0.688
Ap3d1	AP-3 complex subunit delta-1	O54774	S754,S760	RHSSLPTESDEDIAPAQR	S3,S9	1.017	0.666
Ap3d1	AP-3 complex subunit delta-1	O54774	T758,S760,S	HSSLPTESDEDIAPAQR	T6,S8,S	0.974	0.783
Ap3d1	AP-3 complex subunit delta-1	O54774	T758,S760,S	RHSSLPTESDEDIAPAQR	T7,S9,S	1.199	0.231
Ap3d1	AP-3 complex subunit delta-1	O54774	Ambiguous	HSSLPTESDEDIAPAQR	S/T	1.024	0.420
Ap4e1	AP-4 complex subunit epsilon-1	Q80V94	S962	IIEQPECSSPVIETER	S9	0.883	0.105
Apba1	Amyloid-beta A4 precursor protein-binding family A member 1	B2RUJ5	S246	LHHYDERSDGESDSPEKEAEFAPYPR	S8	0.953	0.081
Apba1	Amyloid-beta A4 precursor protein-binding family A member 1	B2RUJ5	S267	MDSYEQEEDIDQIVAEVK	S3	1.129	0.728
Apba1	Amyloid-beta A4 precursor protein-binding family A member 1	B2RUJ5	S573	SNSQENVEASHPSQDGK	S3	1.028	0.886
Apbb1ip	Amyloid beta A4 precursor protein-binding family B member 1-interacting protein	Q8R5A3; F2Z3U3	S340;S424	ASGIYYVPK	S2	1.063	0.398
Apbb1ip	Amyloid beta A4 precursor protein-binding family B member 1-interacting protein	Q8R5A3	S507	QGLGNHSPGATR	S7	1.031	0.276
Apbb1ip	Amyloid beta A4 precursor protein-binding family B member 1-interacting protein	Q8R5A3	S519	SSLPPPPPPVR	S1	0.969	0.548
Apbb1ip	Amyloid beta A4 precursor protein-binding family B member 1-interacting protein	Q8R5A3	S520	SSLPPPPPPVRR	S2	0.954	0.614
Apbb1ip	Amyloid beta A4 precursor protein-binding family B member 1-interacting protein	Q8R5A3	S537	SSDTCGSPALPSK	S7	0.966	0.228
Apbb1ip	Amyloid beta A4 precursor protein-binding family B member 1-interacting protein	Q8R5A3	S624	SSPLPPK	S2	1.034	0.514
Apbb1ip	Amyloid beta A4 precursor protein-binding family B member 1-interacting protein	Q8R5A3	S531,T534	RSSDTCGSPALPSKVK	S2,T5	1.046	0.639
Apbb1ip	Amyloid beta A4 precursor protein-binding family B member 1-interacting protein	Q8R5A3	S532,S537	RSSDTCGSPALPSK	S3,S8	1.046	0.186
Apbb1ip	Amyloid beta A4 precursor protein-binding family B member 1-interacting protein	Q8R5A3	S537,S/T	SSDTCGSPALPSK	S7,S/T	1.005	0.922
Apbb1ip	Amyloid beta A4 precursor protein-binding family B member 1-interacting protein	Q8R5A3	Ambiguous	RSSDTCGSPALPSK	S/T	1.059	0.111
Apbb2	Amyloid-beta A4 precursor protein-binding family B member 2	E9QPX0	S409	NAPHGDDDDSCSINSDPEAK	S	0.953	0.208
Apbb2	Amyloid-beta A4 precursor protein-binding family B member 2	E9QPX0	T34	NSPATPPNTLNLR	T5	0.866	0.244
Apc	Adenomatous polyposis coli protein	Q61315	S2449	EAPSPTLR	S4	1.052	0.168
Apc	Adenomatous polyposis coli protein	Q61315	S2674	SGRSPTGNTPPVIDSVSEK	S4	1.016	0.742
Apc	Adenomatous polyposis coli protein	Q61315	S2713	QSVGSGSPVQTVGLETR	S7	0.999	0.991
Apc	Adenomatous polyposis coli protein	Q61315	S2837	RHSGSYLVTSV	S3	1.105	0.225
Apc	Adenomatous polyposis coli protein	Q61315	S1036,S1040	YSDEQLNSGRQSPSQNER	S8,S12	1.093	0.402
Apc	Adenomatous polyposis coli protein	Q61315	Ambiguous	CTSVSSLDSFESR	S/T	0.967	0.869
Apc	Adenomatous polyposis coli protein	Q61315	Ambiguous	SPSEGPGATTSPR	T/S	1.226	0.244
Apeh	Acylamino-acid-releasing enzyme	Q8R146	S187	ALDVSASDEEMARPK	S7	1.020	0.654
Apeh	Acylamino-acid-releasing enzyme	Q8R146	S187	ALDVSASDEEMARPK	S7	1.002	0.958
Apex1	DNA-(apurinic or apyrimidinic site) lyase	P28352	S18	AAADDGEEPKSEPETKK	S11	0.955	0.573
Apex1	DNA-(apurinic or apyrimidinic site) lyase	P28352	S18	AAADDGEEPKSEPETK	S11	0.998	0.979
Apex1	DNA-(apurinic or apyrimidinic site) lyase	P28352	S18	KAAADDGEEPKSEPETK	S12	1.091	0.176
Api5	Apoptosis inhibitor 5	O35841	S464	TSEDTSSGSPPK	S9	1.024	0.743
Api5	Apoptosis inhibitor 5	O35841	S464	TSEDTSSGSPPKK	S9	1.052	0.445
Aplf	Aprataxin and PNK-like factor	Q9D842	S149	TTGASQLQGSPEITK	S10	1.032	0.803
Aplf	Aprataxin and PNK-like factor	Q9D842	S343,S344	SQGCHPESSSAPSSPDALHTDTADPVLGCSEESK	S9,S10	0.936	0.505
Aplf	Aprataxin and PNK-like factor	Q9D842	Ambiguous	SQGCHPESSSAPSSPDALHTDTADPVLGCSEESK	S/T	1.333	0.488
Apoa2	Apolipoprotein A-II	P09813	Ambiguous	LLAMVALLVTICSLEGALVK	T/S	0.989	0.942
Apobr	Apolipoprotein B receptor	Q8VBT6	S114	QDTGSWKAAEDAR	S5	1.114	0.307
Apobr	Apolipoprotein B receptor	Q8VBT6	S364	TGEMTEGHIAEEEAMGEQETEGSFEDEER	S23	1.147	0.091
Apobr	Apolipoprotein B receptor	Q8VBT6	S484	GQEETSGAPDLSPER	S12	1.009	0.881
Apobr	Apolipoprotein B receptor	Q8VBT6	S484	QLEEGQKGQEETSGAPDLSPER	S19	1.004	0.902
Apobr	Apolipoprotein B receptor	Q8VBT6	S520	RNSQEVK	S3	0.929	0.461
Apobr	Apolipoprotein B receptor	Q8VBT6	S699	QSGQEVGLVGSAEK	S11	1.107	0.160
Apobr	Apolipoprotein B receptor	Q8VBT6	S741	GTDGVTLGSQAER	S9	1.066	0.143
Apobr	Apolipoprotein B receptor	Q8VBT6	S862	SRPSFHR	S4	0.975	0.930
Apobr	Apolipoprotein B receptor	Q8VBT6	T440	SEAAQMTPEVLR	T7	1.065	0.423
Apobr	Apolipoprotein B receptor	Q8VBT6	T440	SEAAQMTPEVLR	T7	1.009	0.807
Apobr	Apolipoprotein B receptor	Q8VBT6	T751	AEGSITPMETEGLLR	T6	1.043	0.245
Apobr	Apolipoprotein B receptor	Q8VBT6	T751	AEGSITPMETEGLLR	T6	0.964	0.713
Apobr	Apolipoprotein B receptor	Q8VBT6	Ambiguous	DRSSNTAQEIWEHGEEEASSGEPLR	S/T	1.087	0.248
Apobr	Apolipoprotein B receptor	Q8VBT6	Ambiguous	GTDGVTLGSQAERAEGSITPMETEGLLR	T/S	0.963	0.791
Apobr	Apolipoprotein B receptor	Q8VBT6	Ambiguous	EGADSLGVQGTEYGSDPGDNFPGTTGR	Y/T/S	1.137	0.174
Apoe	Apolipoprotein E	P08226	S139	NEVHTMLGQSTEEIR	S10	0.856	0.110
Apoe	Apolipoprotein E	P08226	Ambiguous	NEVHTMLGQSTEEIR	T/S	1.007	0.912
Apool	MICOS complex subunit Mic27	Q78IK4	S219	HSVSLPK	S4	0.902	0.520
Appl1	DCC-interacting protein 13-alpha	Q8K3H0	S401	VNQSALEAVTPSPSFQQR	S12	1.022	0.650
Appl1	DCC-interacting protein 13-alpha	Q8K3H0	Ambiguous	QIYLSENPEETAAR	Y/S/T	1.062	0.517
Appl2	DCC-interacting protein 13-beta	Q8K3G9	S651	YVLLNDQADDTGGSPSENRGAESEA	S14	1.133	0.374
Appl2	DCC-interacting protein 13-beta	Q8K3G9	Ambiguous	YVLLNDQADDTGGSPSENR	S/T/Y	1.073	0.505
Aprt	Adenine phosphoribosyltransferase	P08030	S66	IDYIAGLDSR	S9	0.990	0.907
Aptx	Aprataxin	Q7TQC5	Ambiguous	KRSDCDSEEMEAESGTGLAPGSSPSQCSVSPK	S/T	1.080	0.022
Araf	Serine/threonine-protein kinase A-Raf	P04627	S198	DQEHFSFPAPANPPLQR	S6	0.849	0.304
Araf	Serine/threonine-protein kinase A-Raf	P04627	S255	GSPSPASVSSGR	S2	1.037	0.449
Araf	Serine/threonine-protein kinase A-Raf	P04627	S255	GGDGAPRGSPSPASVSSGR	S9	0.952	0.451
Araf	Serine/threonine-protein kinase A-Raf	P04627	T181	QQEAPSNLSVNELLTPQGPSPFTQQR	T15	1.533	0.132
Araf	Ras-binding protein DA-Raf	Q8CAD1; P04627	S157;S157	QFYHSIQDLSGGSR	S5	1.004	0.937
Araf	Ras-binding protein DA-Raf	Q8CAD1	T181	QQEAPSNLSVNELLTPQGPR	T15	1.144	0.157
Arap1	Arf-GAP with Rho-GAP domain, ANK repeat and PH domain-containing protein 1	Q4LDD4	S183	EELLLPSVSPR	S9	1.032	0.649
Arap1	Arf-GAP with Rho-GAP domain, ANK repeat and PH domain-containing protein 1	Q4LDD4	S448	GSEQPDRAGSLELR	S10	0.960	0.324
Arap1	Arf-GAP with Rho-GAP domain, ANK repeat and PH domain-containing protein 1	Q4LDD4	S499	SFDLTTPYR	S1	1.006	0.893
Arcn1	Coatomer subunit delta	Q5XJY5	S493	VTQVDGNSPVR	S8	0.960	0.645
Arel1	Apoptosis-resistant E3 ubiquitin protein ligase 1	Q8CHG5	S337	RPSTAIEEDDEDSPSECHTPEK	S13	0.914	0.399
Arf4	ADP-ribosylation factor 4	P61750	S147	LGLQSLR	S5	1.147	0.271
Arfgap1	ADP-ribosylation factor GTPase-activating protein 1	V9GXM1	S352	AKSPSSDSWTCADASTGR	S3	1.146	0.183
Arfgap1	ADP-ribosylation factor GTPase-activating protein 1	V9GXM1	S370	SSDSWDVWGSGSASNNK	S2	1.274	0.021
Arfgap1	ADP-ribosylation factor GTPase-activating protein 1	V9GXM1	S394	NSNSDGWESWEGASGEGR	S9	0.909	0.628
Arfgap1	ADP-ribosylation factor GTPase-activating protein 1	V9GXM1	S413	AAPSTADEGWDNQNW	S4	0.785	0.060
Arfgap1	ADP-ribosylation factor GTPase-activating protein 1	V9GXM1	Ambiguous	RSSDSWDVWGSGSASNNK	S	1.125	0.139
Arfgap2	ADP-ribosylation factor GTPase-activating protein 2	Q99K28	S239	VSNQSFTEIER	S5	0.921	0.196
Arfgap3	ADP-ribosylation factor GTPase-activating protein 3	A0A0R4J0T8	Ambiguous	LTNTSFTEIEK	T/S	0.922	0.187
Arfgef1	Brefeldin A-inhibited guanine nucleotide-exchange protein 1	G3X9K3	S1566	SVDIHDSIQPR	S1	0.924	0.305
Arfgef1	Brefeldin A-inhibited guanine nucleotide-exchange protein 1	G3X9K3	S234	QQQHLLQSPVSHHEPESPHLR	S8	1.046	0.541
Arfgef1	Brefeldin A-inhibited guanine nucleotide-exchange protein 1	G3X9K3	S286,S/T	SLQDDTEPENGSDISSAENEQTEADQATAAETLSK	S12,S/T	1.106	0.618
Arfgef1	Brefeldin A-inhibited guanine nucleotide-exchange protein 1	G3X9K3	Ambiguous	SLQDDTEPENGSDISSAENEQTEADQATAAETLSK	T/S	1.014	0.755
Arfgef2	Brefeldin A-inhibited guanine nucleotide-exchange protein 2	A2A5R2	S1535	GQSQLSNPTDDSWK	S6	1.074	0.363
Arfgef2	Brefeldin A-inhibited guanine nucleotide-exchange protein 2	A2A5R2	S356	QSLSSADNLEPDVQGHQVAAR	S5	0.972	0.399
Arfgef2	Brefeldin A-inhibited guanine nucleotide-exchange protein 2	A2A5R2	S1532,S1535	GQSQLSNPTDDSWK	S3,S6	0.977	0.718
Arfgef2	Brefeldin A-inhibited guanine nucleotide-exchange protein 2	A2A5R2	Ambiguous	QSLSSIDRNASER	S	0.944	0.575
Arfgef3	Brefeldin A-inhibited guanine nucleotide-exchange protein 3	Q3UGY8	S2090	SGSTGSSLSVSVR	S3	1.061	0.227
Arfgef3	Brefeldin A-inhibited guanine nucleotide-exchange protein 3	Q3UGY8	S593	YSESNFSVDDQDLSR	S7	1.040	0.727
Arfip1	ADP-ribosylation factor-interacting protein 1	G5E8V9	S79	EGVTEAGAPQGSSAPPLPCVLSPSR	S22	0.965	0.811
Arglu1	Arginine and glutamate-rich protein 1	Q3UL36	S75	ASSPPDRIDIFGR	S3	0.991	0.784
Arglu1	Arginine and glutamate-rich protein 1	Q3UL36	Ambiguous	ERASSPPDRIDIFGR	S	0.989	0.925
Arhgap1	Rho GTPase-activating protein 1	A2AH25	S91	SSSPEPVTHLK	S3	1.055	0.130
Arhgap1	Rho GTPase-activating protein 1	A2AH25	Ambiguous	SSSPEPVTHLKWDDPYYDIAR	S/T/Y	1.178	0.391
Arhgap1	Rho GTPase-activating protein 1	A2AH25	Ambiguous	SDDSKSSSPEPVTHLK	S/T	1.016	0.789
Arhgap10	Rho GTPase-activating protein 10	Q6Y5D8	S591	TSPDTTFAEPTCLSASPPNAPPR	S16	1.017	0.702
Arhgap11a	Rho GTPase-activating protein 11A	Q80Y19	Ambiguous	SVSCDSALPSCVQNTSK	S/T	1.117	0.387
Arhgap12	Rho GTPase-activating protein 12	S4R221	S199	TSFSQEQSCDSAGEGSER	S11	1.061	0.167
Arhgap12	Rho GTPase-activating protein 12	S4R221	S211	IQQDSESGDELSSSSTEQMR	S5	1.205	0.065
Arhgap12	Rho GTPase-activating protein 12	S4R221	S213	IQQDSESGDELSSSSTEQMR	S7	0.956	0.322
Arhgap12	Rho GTPase-activating protein 12	S4R221	S444	LCLPENESPPTSSK	S8	0.986	0.852
Arhgap12	Rho GTPase-activating protein 12	S4R221	S199,S204	TSFSQEQSCDSAGEGSER	S11,S16	0.845	0.157
Arhgap12	Rho GTPase-activating protein 12	S4R221	T229,S238	ATTPPNQGRPDSPVYANLQELK	T3,S12	0.975	0.354
Arhgap15	Rho GTPase-activating protein 15	Q811M1	S51	SMILTDAGK	S1	0.959	0.522
Arhgap15	Rho GTPase-activating protein 15	Q811M1	S51	SMILTDAGK	S1	1.078	0.337
Arhgap15	Rho GTPase-activating protein 15	Q811M1	Ambiguous	NPSCGSLELFNLQR	S	1.152	0.564
Arhgap17	Rho GTPase-activating protein 17	Q3UIA2	S671	SPSPPQQQQQQQQQQQQQQQQQQTPGMR	S3	1.011	0.860
Arhgap17	Rho GTPase-activating protein 17	Q3UIA2	S671	SPSPPQQQQQQQQQQQQQQQQQQTPGMR	S3	0.998	0.971
Arhgap17	Rho GTPase-activating protein 17	Q3UIA2	T730	LGEQGPEPGPTPPQTPTPPSTPPLAK	T11	1.045	0.214
Arhgap17	Rho GTPase-activating protein 17	Q3UIA2	S671,T691	SPSPPQQQQQQQQQQQQQQQQQQTPGMR	S3,T23	0.764	0.141
Arhgap17	Rho GTPase-activating protein 17	Q3UIA2	S739,T740	LGEQGPEPGPTPPQTPTPPSTPPLAK	S20,T21	0.970	0.429
Arhgap17	Rho GTPase-activating protein 17	Q3UIA2	T730,S739,T740	LGEQGPEPGPTPPQTPTPPSTPPLAK	T11,S20,T21	0.969	0.505
Arhgap17	Rho GTPase-activating protein 17	Q3UIA2	Ambiguous	CSSSLPPIQAPSHPPPQPPTQPR	S/T	1.091	0.538
Arhgap18	Rho GTPase-activating protein 18	Q8K0Q5	S263	ISSNDSLPSFR	S9	1.079	0.248
Arhgap21	Rho GTPase-activating protein 21	B7ZCJ1	S1666	RNSEGSEASCTEGSLTPSLDSR	S3	1.233	0.232
Arhgap21	Rho GTPase-activating protein 21	B7ZCJ1	S1710	FKSDSGSPGDTR	S3	1.370	0.001
Arhgap21	Rho GTPase-activating protein 21	B7ZCJ1	S1739	STGSLLTPSR	S1	1.062	0.329
Arhgap21	Rho GTPase-activating protein 21	B7ZCJ1	S42	DQGEPVSPSEDEPFSWPGPK	S7	1.117	0.735
Arhgap21	Rho GTPase-activating protein 21	B7ZCJ1	S861	QLSHDQESVGPPSLDGQHSSK	S3	0.888	0.484
Arhgap21	Rho GTPase-activating protein 21	B7ZCJ1	S885	SKSYDEGLDDYREDAK	S3	1.086	0.156
Arhgap21	Rho GTPase-activating protein 21	B7ZCJ1	S928	KGSSSEVFSDAAR	S3	1.202	0.002
Arhgap21	Rho GTPase-activating protein 21	B7ZCJ1	T1953	QSESPDTKAETPP	T11	1.032	0.721
Arhgap22	Rho GTPase-activating protein 22	Q8BL80	S387	GEPGSPGLPTHR	S5	1.014	0.701
Arhgap22	Rho GTPase-activating protein 22	Q8BL80	S387	DHRGEPGSPGLPTHR	S8	0.978	0.626
Arhgap22	Rho GTPase-activating protein 22	Q8BL80	S397	TSSLDGPAAAVLSR	S3	1.054	0.148
Arhgap23	Rho GTPase-activating protein 23	B1AQY2	S377	SAEALGPGALVSPR	S12	1.033	0.745
Arhgap23	Rho GTPase-activating protein 23	B1AQY2	S426	SYSPSFQR	S3	0.987	0.840
Arhgap23	Rho GTPase-activating protein 23	B1AQY2	S520	QMNLGFGDESPEPEAR	S10	1.058	0.755
Arhgap23	Rho GTPase-activating protein 23	B1AQY2	S599	HYSQDCSSIK	S3	0.972	0.732
Arhgap23	Rho GTPase-activating protein 23	B1AQY2	S624	SKSCDDGLNTFRDEGR	S3	1.021	0.443
Arhgap23	Rho GTPase-activating protein 23	B1AQY2	S366,S377	SAEALGPGALVSPR	S1,S12	1.032	0.707
Arhgap24	Rho GTPase activating protein 24, isoform CRA_b	G3X9N1	S390	SSVDNGSPTALSGSK	S7	1.006	0.927
Arhgap25	Rho GTPase-activating protein 25	Q8BYW1	S363	DAPISPPAQK	S5	1.047	0.386

Arhgap25	Rho GTPase-activating protein 25	Q8BYW1	S363	SKDAPISPPAQK	S7	1.039	0.376
Arhgap25	Rho GTPase-activating protein 25	Q8BYW1	S380	SSVGWDATEDPPLSR	S2	1.027	0.479
Arhgap25	Rho GTPase-activating protein 25	Q8BYW1	S396	TDSFSNTASSPDATSPTGPLPSDQHQEDSGKAPR	S3	1.025	0.839
Arhgap25	Rho GTPase-activating protein 25	Q8BYW1	S509	TSTYDNVPTSPQSQGNPAGALSPPASDSKR	S10	1.137	0.115
Arhgap25	Rho GTPase-activating protein 25	Q8BYW1	S509	TSTYDNVPTSPQSQGNPAGALSPPASDSK	S10	1.120	0.064
Arhgap25	Rho GTPase-activating protein 25	Q8BYW1	S553	NSGEDDLDSLQR	S9	0.964	0.659
Arhgap25	Rho GTPase-activating protein 25	Q8BYW1	T152	VAGTPSGAVFGQR	T4	1.053	0.242
Arhgap25	Rho GTPase-activating protein 25	Q8BYW1	T535	DALVSTDSEMEAGSK	T6	1.013	0.814
Arhgap25	Rho GTPase-activating protein 25	Q8BYW1	T535	DALVSTDSEMEAGSKNSGEDDLDSLQR	T6	0.980	0.874
Arhgap25	Rho GTPase-activating protein 25	Q8BYW1	T535	DALVSTDSEMEAGSK	T6	0.982	0.530
Arhgap25	Rho GTPase-activating protein 25	Q8BYW1	S396,T400	TDSFSNTASSPDATSPTGPLPSDQHQEDSGKAPR	S3,T7	1.073	0.411
Arhgap25	Rho GTPase-activating protein 25	Q8BYW1	S521,T/S/Y	TSTYDNVPTSPQSQGNPAGALSPPASDSKR	S22,T/S/Y	1.055	0.438
Arhgap25	Rho GTPase-activating protein 25	Q8BYW1	S521,T/S/Y	TSTYDNVPTSPQSQGNPAGALSPPASDSK	S22,T/S/Y	1.100	0.358
Arhgap25	Rho GTPase-activating protein 25	Q8BYW1	S534,T535	DALVSTDSEMEAGSK	S5,T6	1.020	0.773
Arhgap25	Rho GTPase-activating protein 25	Q8BYW1	T400,S/T	TDSFSNTASSPDATSPTGPLPSDQHQEDSGK	T7,S/T	1.131	0.287
Arhgap25	Rho GTPase-activating protein 25	Q8BYW1	Ambiguous	TDSFSNTASSPDATSPTGPLPSDQHQEDSGK	T/S	1.124	0.102
Arhgap25	Rho GTPase-activating protein 25	Q8BYW1	Ambiguous	DALVSTDSEMEAGSKNSGEDDLDSLQR	T/S	0.926	0.417
Arhgap25	Rho GTPase-activating protein 25	Q8BYW1	Ambiguous	DALVSTDSEMEAGSK	S/T	1.089	0.759
Arhgap27	Rho GTPase-activating protein 27	A2AB59	S118	ASSLCGPAR	S3	1.014	0.641
Arhgap27	Rho GTPase-activating protein 27	A2AB59	S215	AESPKQVDDPPEPVYANVER	S3	1.028	0.644
Arhgap27	Rho GTPase-activating protein 27	A2AB59	S215	CPPRAESPKQVDDPPEPVYANVER	S7	0.892	0.083
Arhgap27	Rho GTPase-activating protein 27	A2AB59	S469	SSQDSDTPAQASPPEEK	S12	0.950	0.450
Arhgap27	Rho GTPase-activating protein 27	A2AB59	S632	QNAASPSLSPGGLESDLSR	S5	1.015	0.671
Arhgap27	Rho GTPase-activating protein 27	A2AB59	S663	RPTLQSLR	S6	0.888	0.443
Arhgap27	Rho GTPase-activating protein 27	A2AB59	Y227	QVDDPPEPVYANVER	Y10	1.034	0.221
Arhgap27	Rho GTPase-activating protein 27	A2AB59	S215,Y227	AESPKQVDDPPEPVYANVER	S3,Y15	0.966	0.705
Arhgap27	Rho GTPase-activating protein 27	A2AB59	S215,Y227	CPPRAESPKQVDDPPEPVYANVER	S7,Y19	0.958	0.473
Arhgap27	Rho GTPase-activating protein 27	A2AB59	S459,T464	KSSQDSDTPAQASPPEEK	S3,T8	0.956	0.107
Arhgap27	Rho GTPase-activating protein 27	A2AB59	S462,S469,S/T	KSSQDSDTPAQASPPEEK	S6,S13,S/T	1.119	0.209
Arhgap27	Rho GTPase-activating protein 27	A2AB59	Ambiguous	KSSQDSDTPAQASPPEEK	S/T	1.062	0.260
Arhgap28	Rho GTPase-activating protein 28	Q8BN58	T160	YNTYTQTLR	T3	0.916	0.215
Arhgap30	Rho GTPase-activating protein 30	Q640N3	S1067	SRLSLPPR	S4	0.974	0.398
Arhgap30	Rho GTPase-activating protein 30	Q640N3	S669	STSDNREEAEATPETEMEAGKADAEGGEAER	S1	1.093	0.069
Arhgap30	Rho GTPase-activating protein 30	Q640N3	S669	STSDNREEAEATPETEMEAGKADAEGGEAER	S1	1.030	0.283
Arhgap30	Rho GTPase-activating protein 30	Q640N3	S669	STSDNREEAEATPETEMEAGK	S1	1.065	0.310
Arhgap30	Rho GTPase-activating protein 30	Q640N3	S875	VDSTEEINEQTSEMK	S3	0.999	0.990
Arhgap30	Rho GTPase-activating protein 30	Q640N3	S875	VDSTEEINEQTSEMK	S3	1.058	0.422
Arhgap30	Rho GTPase-activating protein 30	Q640N3	S966	IHVAPASPCPRPGR	S7	1.032	0.729
Arhgap30	Rho GTPase-activating protein 30	Q640N3	Ambiguous	STSDNREEAEATPETEMEAGK	S/T	1.067	0.257
Arhgap31	Rho GTPase-activating protein 31	A6X8Z5	S1163	RNSAPVSVSAVR	S3	1.006	0.808
Arhgap31	Rho GTPase-activating protein 31	A6X8Z5	S1242	STKDDSPSSLGSPEEEQPKQETGASASR	S12	1.122	0.143
Arhgap31	Rho GTPase-activating protein 31	A6X8Z5	S1242	DDSPSSLGSPEEEQPKQETGASASR	S9	1.095	0.198
Arhgap31	Rho GTPase-activating protein 31	A6X8Z5	S1242	STKDDSPSSLGSPEEEQPK	S12	1.186	0.184
Arhgap31	Rho GTPase-activating protein 31	A6X8Z5	S1242	DDSPSSLGSPEEEQPK	S9	1.080	0.145
Arhgap31	Rho GTPase-activating protein 31	A6X8Z5	S1413	SVILDGR	S1	0.800	0.021
Arhgap31	Rho GTPase-activating protein 31	A6X8Z5	S563	AECSQSPPLDPGTQVEK	S6	1.119	0.100
Arhgap31	Rho GTPase-activating protein 31	A6X8Z5	S563	AECSQSPPLDPGTQVEKK	S6	0.968	0.779
Arhgap31	Rho GTPase-activating protein 31	A6X8Z5	S850	GPGLPSPTK	S6	1.191	0.233
Arhgap31	Rho GTPase-activating protein 31	A6X8Z5	S941	QSHSLDSK	S4	1.063	0.421
Arhgap31	Rho GTPase-activating protein 31	A6X8Z5	T1224	SQEEPGSTPEIPQK	T8	1.045	0.579
Arhgap31	Rho GTPase-activating protein 31	A6X8Z5	Ambiguous	MHASSTGSSCDLSK	S/T	0.993	0.980
Arhgap31	Rho GTPase-activating protein 31	A6X8Z5	Ambiguous	AATEDTKPEPEVPGRAECSQSPPLDPGTQVEK	S/T	0.902	0.529
Arhgap31	Rho GTPase-activating protein 31	A6X8Z5	Ambiguous	SMDSLCSVPVEGK	S	0.910	0.202
Arhgap31	Rho GTPase-activating protein 31	A6X8Z5	Ambiguous	TDVYTEQPTPKESPGIPTPCQR	T/S/Y	0.987	0.906
Arhgap35	Rho GTPase-activating protein 35	Q91YM2	S1106	NEEENIYSVPHDSTQGK	S8	1.031	0.430
Arhgap35	Rho GTPase-activating protein 35	Q91YM2	S1134	AQSNGSGNGSDSEMDTSSLER	S10	1.067	0.616
Arhgap35	Rho GTPase-activating protein 35	Q91YM2	S1179	TSFSVGSDDELGPIR	S7	1.003	0.950
Arhgap39	DNA segment, Chr 15, Wayne State University 169, expressed, isoform CRA_a	G3X932	S271	RPSPFLR	S3	0.854	0.102
Arhgap39	DNA segment, Chr 15, Wayne State University 169, expressed, isoform CRA_a	G3X932	S364	QTPTSPCQQLMR	S5	1.009	0.861
Arhgap39	DNA segment, Chr 15, Wayne State University 169, expressed, isoform CRA_a	G3X932	S405	QLVYVEQAGSSPK	S11	0.982	0.810
Arhgap39	DNA segment, Chr 15, Wayne State University 169, expressed, isoform CRA_a	G3X932	S599	AFSEDEALAQQDSK	S3	1.036	0.400
Arhgap39	DNA segment, Chr 15, Wayne State University 169, expressed, isoform CRA_a	G3X932	T424	YAPNPGGGTYSLQPSPCLLR	T9	1.025	0.856
Arhgap39	DNA segment, Chr 15, Wayne State University 169, expressed, isoform CRA_a	G3X932	S280,S284	RAELSGNCSPLLIQPR	S5,S9	0.976	0.853
Arhgap39	DNA segment, Chr 15, Wayne State University 169, expressed, isoform CRA_a	G3X932	Ambiguous	ASADNSPGRGSRDGSTGSSLEPELEER	S/T	1.070	0.460
Arhgap39	DNA segment, Chr 15, Wayne State University 169, expressed, isoform CRA_a	G3X932	Ambiguous	QTPTSPCQQLMR	T/S	1.096	0.058
Arhgap4	Rho GTPase-activating protein 4	B1AUY3	S949	GPGAPISPSTSHPQGPDSTR	S7	1.172	0.006
Arhgap4	Rho GTPase-activating protein 4	B1AUY3	S949	GPGAPISPSTSHPQGPDSTRKPV	S7	1.023	0.759
Arhgap45	Histocompatibility (Minor) HA-1, isoform CRA_a	G3X9Q3	S23	AGSPNPQSSSGELPR	S3	0.989	0.775
Arhgap45	Histocompatibility (Minor) HA-1, isoform CRA_a	G3X9Q3	S23	AGSPNPQSSSGELPRK	S3	0.936	0.182
Arhgap45	Histocompatibility (Minor) HA-1, isoform CRA_a	G3X9Q3	S23	NRAGSPNPQSSSGELPR	S5	0.921	0.588
Arhgap45	Histocompatibility (Minor) HA-1, isoform CRA_a	G3X9Q3	S568	YDFEPYVSNNSWSPIMR	S13	0.934	0.347
Arhgap45	Histocompatibility (Minor) HA-1, isoform CRA_a	G3X9Q3	S577	GSFNPGDASGPEAAGSPPEEGGTSEAAPNKDHR	S2	0.976	0.593
Arhgap45	Histocompatibility (Minor) HA-1, isoform CRA_a	G3X9Q3	S577	KGSFNPGDASGPEAAGSPPEEGGTSEAAPNKDHR	S3	1.148	0.301
Arhgap45	Histocompatibility (Minor) HA-1, isoform CRA_a	G3X9Q3	S577	KGSFNPGDASGPEAAGSPPEEGGTSEAAPNK	S3	1.028	0.777
Arhgap45	Histocompatibility (Minor) HA-1, isoform CRA_a	G3X9Q3	S875	QGGSESEAATLAMVGR	S4	1.033	0.461
Arhgap45	Histocompatibility (Minor) HA-1, isoform CRA_a	G3X9Q3	S875	GRQGGSESEAATLAMVGR	S6	1.051	0.299
Arhgap45	Histocompatibility (Minor) HA-1, isoform CRA_a	G3X9Q3	S875	GRQGGSESEAATLAMVGR	S6	1.028	0.763
Arhgap45	Histocompatibility (Minor) HA-1, isoform CRA_a	G3X9Q3	S875	QGGSESEAATLAMVGR	S4	0.989	0.932
Arhgap45	Histocompatibility (Minor) HA-1, isoform CRA_a	G3X9Q3	S1020,S1022	ESHAASNDSDSELEDASDPLSSSDASALHR	S9,S11	1.065	0.445
Arhgap45	Histocompatibility (Minor) HA-1, isoform CRA_a	G3X9Q3	S23,S30	AGSPNPQSSSGELPR	S3,S10	0.988	0.686
Arhgap45	Histocompatibility (Minor) HA-1, isoform CRA_a	G3X9Q3	S577,S584	KGSFNPGDASGPEAAGSPPEEGGTSEAAPNK	S3,S10	0.964	0.822
Arhgap45	Histocompatibility (Minor) HA-1, isoform CRA_a	G3X9Q3	Ambiguous	ESHAASNDSDSELEDASDPLSSSDASALHR	S	1.025	0.830
Arhgap45	Histocompatibility (Minor) HA-1, isoform CRA_a	G3X9Q3	Ambiguous	GSFNPGDASGPEAAGSPPEEGGTSEAAPNK	T/S	1.349	0.412
Arhgap5	Rho GTPase-activating protein 5	E9PYT0	S1125	IRNSFVNNTQGDEENGFSDR	S4	1.228	0.032
Arhgap5	Rho GTPase-activating protein 5	E9PYT0	S1177	THSDASDDEAFTTSK	S6	0.956	0.234
Arhgap5	Rho GTPase-activating protein 5	E9PYT0	S1219	GGIDNPAITSDQEVDDK	S10	1.006	0.920
Arhgap5	Rho GTPase-activating protein 5	E9PYT0	Y1109	GYSDEIYVVPDDSQNR	Y7	0.900	0.658
Arhgap5	Rho GTPase-activating protein 5	E9PYT0	S1174,S1177	RTHSDASDDEAFTTSK	S4,S7	1.055	0.341
Arhgap5	Rho GTPase-activating protein 5	E9PYT0	S1177,T/S	THSDASDDEAFTTSK	S6,T/S	1.114	0.007
Arhgap5	Rho GTPase-activating protein 5	E9PYT0	Ambiguous	GGIDNPAITSDQEVDDKK	T/S	1.032	0.679
Arhgap5	Rho GTPase-activating protein 5	E9PYT0	Ambiguous	RTHSDASDDEAFTTSK	T/S	1.058	0.550
Arhgap6	Rho GTPase-activating protein 6	O54834	S332	ELSSSNSSLSSTSETPNESTSPNTPEPAPR	S5	1.005	0.969
Arhgap6	Rho GTPase-activating protein 6	O54834	S365	GAMSVDSITDLDDNQSR	S4	1.033	0.375
Arhgap6	Rho GTPase-activating protein 6	O54834	S365	GAMSVDSITDLDDNQSR	S4	0.969	0.731
Arhgap6	Rho GTPase-activating protein 6	O54834	S365	RGAMSVDSITDLDDNQSR	S5	0.994	0.968
Arhgap6	Rho GTPase-activating protein 6	O54834	S669	ASSGDISPYDNNSPVLSER	S7	1.021	0.678
Arhgap6	Rho GTPase-activating protein 6	O54834	S790	CQGSPTGLDSGTQVIR	S4	1.016	0.805
Arhgap6	Rho GTPase-activating protein 6	O54834	S331,S332	QNKELSSSNSSLSSTSETPNESTSPNTPEPAPR	S7,S8	1.148	0.382
Arhgap6	Rho GTPase-activating protein 6	O54834	S669,S675	ASSGDISPYDNNSPVLSER	S7,S13	0.989	0.898
Arhgap6	Rho GTPase-activating protein 6	O54834	Ambiguous	VCSTPHIQDGSR	S/T	0.940	0.161
Arhgap6	Rho GTPase-activating protein 6	O54834	Ambiguous	ELSSSNSSLSSTSETPNESTSPNTPEPAPR	S/T	1.070	0.875
Arhgap9	ArhGAP9	Q1HDU4	S294	AGSRPESSVDLR	S7	1.053	0.431
Arhgap9	ArhGAP9	Q1HDU4	S372	LSGSGPAELAELSAGEDDELESEPVSK	S13	0.966	0.665
Arhgdia	Rho GDP-dissociation inhibitor 1	Q99PT1	S47	SIQEIQELDKDDESLR	S14	1.007	0.914
Arhgef1	Rho guanine nucleotide exchange factor 1	E9PUF7	S383	SESLRVSDR	S3	1.039	0.570
Arhgef1	Rho guanine nucleotide exchange factor 1	E9PUF7	S409	SRSDVDMDPGSATAVLGPTRR	S3	0.934	0.391
Arhgef1	Rho guanine nucleotide exchange factor 1	E9PUF7	S409	SRSDVDMDPGSATAVLGPTRR	S3	1.002	0.966
Arhgef1	Rho guanine nucleotide exchange factor 1	E9PUF7	S409	SRSDVDMDPGSATAVLGPTR	S3	1.003	0.900
Arhgef1	Rho guanine nucleotide exchange factor 1	E9PUF7	S409	SRSDVDMDPGSATAVLGPTR	S3	1.091	0.522
Arhgef1	Rho guanine nucleotide exchange factor 1	E9PUF7	S841	EPLLSSSENGTGGAEMAPADAR	S5	1.188	0.006
Arhgef1	Rho guanine nucleotide exchange factor 1	E9PUF7	T429	RATPEPGDDGEPGR	T3	1.016	0.770
Arhgef1	Rho guanine nucleotide exchange factor 1	E9PUF7	T750	TLTPTPDGK	T3	0.878	0.176
Arhgef1	Rho guanine nucleotide exchange factor 1	E9PUF7	Ambiguous	QILLSTEEDSGAGPPR	S/T	1.130	0.449
Arhgef1	Rho guanine nucleotide exchange factor 1	E9PUF7	Ambiguous	QSSDPMLSEFK	S	0.976	0.581
Arhgef10	Rho guanine nucleotide exchange factor 10	Q8C033	S34	CDTSNNEEEGELFDFDSGDEVPEADR	S17	0.750	0.381
Arhgef11	Rho guanine nucleotide exchange factor (GEF) 11	Q68FM7	S1125	HPGDAPVLNHPSPPGSQEPAYQGSTSSR	S12	1.514	0.199
Arhgef11	Rho guanine nucleotide exchange factor (GEF) 11	Q68FM7	S1198	ALPCPPPSLDGENR	S8	0.778	0.291
Arhgef11	Rho guanine nucleotide exchange factor (GEF) 11	Q68FM7	S1321	NSGIWDSPELDRNPAEEASSSEPAGSYK	S2	0.707	0.009
Arhgef11	Rho guanine nucleotide exchange factor (GEF) 11	Q68FM7	S1321	TRNSGIWDSPELDRNPAEEASSSEPAGSYK	S4	1.046	0.862
Arhgef11	Rho guanine nucleotide exchange factor (GEF) 11	Q68FM7	S14	LSSLSSLGDSTPER	S3	1.091	0.300
Arhgef11	Rho guanine nucleotide exchange factor (GEF) 11	Q68FM7; P61021; Q8C266	S1446;S35;S36	SSLVLR	S2	1.006	0.904
Arhgef11	Rho guanine nucleotide exchange factor (GEF) 11	Q68FM7	S256	LSVDSQEADSGLDSGTER	S5	0.950	0.252
Arhgef11	Rho guanine nucleotide exchange factor (GEF) 11	Q68FM7	S35	QPSDTSETTGLVQR	S3	1.082	0.232
Arhgef11	Rho guanine nucleotide exchange factor (GEF) 11	Q68FM7	S665	SRSDVDMDAAAEATR	S3	0.909	0.193
Arhgef11	Rho guanine nucleotide exchange factor (GEF) 11	Q68FM7	S291,S295	NSVLSDPGLDSPQTSPVILAR	S11,S15	0.962	0.698
Arhgef11	Rho guanine nucleotide exchange factor (GEF) 11	Q68FM7	S693,T698	SLENPTPPFTPK	S1,T6	1.031	0.182
Arhgef11	Rho guanine nucleotide exchange factor (GEF) 11	Q68FM7	Ambiguous	ELLNSLGGESSGGTTPVGSFHTEAAR	S/T	1.005	0.988
Arhgef12	Rho guanine nucleotide exchange factor 12	F8VQN6	S342	SYLEEAPERSEGVQDAQEPQSLVGSPSTR	S25	1.096	0.458
Arhgef12	Rho guanine nucleotide exchange factor 12	Q8R4H2; F8VQN6	S1327;S1328	TGTGDIATCDSPR	S11	1.084	0.407
Arhgef12	Rho guanine nucleotide exchange factor 12	Q8R4H2; F8VQN6	S1389;S1390	EAHSDDNPSEGDGAVK	S4	1.065	0.421
Arhgef12	Rho guanine nucleotide exchange factor 12	Q8R4H2	S341	SEGVQDAEPQSLVGSPSTR	S15	1.054	0.632
Arhgef12	Rho guanine nucleotide exchange factor 12	Q8R4H2	Ambiguous	SYLEEAPERSEGVQDAEPQSLVGSPSTR	S/T/Y	0.987	0.864
Arhgef12	Rho guanine nucleotide exchange factor 12	F8VQN6	Ambiguous	SEGVQDAQEPQSLVGSPSTR	S/T	1.047	0.495
Arhgef12	Rho guanine nucleotide exchange factor 12	Q8R4H2; F8VQN6	Ambiguous	SSSHDFDPTDSSSK	S/T	0.946	0.702
Arhgef12	Rho guanine nucleotide exchange factor 12	Q8R4H2; F8VQN6	Ambiguous	HLSTPSSVSPEPQDPAK	S/T	0.997	0.977
Arhgef12	Rho guanine nucleotide exchange factor 12 (Fragment)	A0A1L1SUP5	Ambiguous	SSSHDFDPTGLVQR	S/T	0.887	0.205
Arhgef2	Rho guanine nucleotide exchange factor 2	Q60875	S109	NNTALQSVSLR	S9	0.993	0.885
Arhgef2	Rho guanine nucleotide exchange factor 2	Q60875	S174	QILSQSTDSLNMR	S6	0.941	0.184

Arhgef2	Rho guanine nucleotide exchange factor 2	Q60875	S174	QILSQSTDSLNMR	S6	0.940	0.447
Arhgef2	Rho guanine nucleotide exchange factor 2	Q60875	S646	LESFESLRGER	S3	1.192	0.008
Arhgef2	Rho guanine nucleotide exchange factor 2	Q60875	S646	LESFESLR	S3	1.201	0.010
Arhgef2	Rho guanine nucleotide exchange factor 2	Q60875	S885	SLPAGDALYLSFNPPQPSR	S1	1.009	0.976
Arhgef2	Rho guanine nucleotide exchange factor 2	Q60875	S931	EAQELGSPEDR	S7	1.192	0.082
Arhgef2	Rho guanine nucleotide exchange factor 2	Q60875	S955	LSPPHSPR	S2	1.003	0.954
Arhgef2	Rho guanine nucleotide exchange factor 2	Q60875	S174,S177	QILSQSTDSLNMR	S6,S9	1.081	0.232
Arhgef2	Rho guanine nucleotide exchange factor 2	Q60875	S177,S/T	QILSQSTDSLNMR	S9,S/T	1.008	0.958
Arhgef2	Rho guanine nucleotide exchange factor 2	Q60875	S955,S959	LSPPHSPR	S2,S6	1.065	0.260
Arhgef2	Rho guanine nucleotide exchange factor 2	Q60875	S955,S959	LQDSSDPDTGSEEEVSSRLSPPHSPR	S20,S24	1.052	0.309
Arhgef2	Rho guanine nucleotide exchange factor 2	Q60875	Ambiguous	ERPTSAIYPSDSFR	S/T/Y	0.897	0.071
Arhgef2	Rho guanine nucleotide exchange factor 2	Q60875	Ambiguous	LQDSSDPDTGSEEEVSSR	S/T	1.193	0.311
Arhgef2	Rho guanine nucleotide exchange factor 2	Q60875	Ambiguous	LQDSSDPDTGSEEEVSSRLSPPHSPR	S/T	0.990	0.858
Arhgef40	Rho guanine nucleotide exchange factor 40	S4R189	S968	IQQQLGEEASPR	S10	1.028	0.600
Arhgef40	Rho guanine nucleotide exchange factor 40	S4R189	S968	RIQQQLGEEASPR	S11	1.093	0.467
Arhgef5	Rho guanine nucleotide exchange factor 5	E9Q7D5	Ambiguous	EKEEEETSPDTSIPR	T/S	1.087	0.405
Arhgef6	Rho guanine nucleotide exchange factor 6	F6WMJ3	S511	MSGFMYQGK	S2	0.895	0.225
Arhgef6	Rho guanine nucleotide exchange factor 6	F6WMJ3	S511	MSGFMYQGK	S2	0.995	0.884
Arhgef6	Rho guanine nucleotide exchange factor 6	F6WMJ3	S578	GPTSCGSLSK	S7	0.978	0.796
Arhgef6	Rho guanine nucleotide exchange factor 6	F6WMJ3	S663	KASEEEYVIR	S3	1.125	0.265
Arhgef6	Rho guanine nucleotide exchange factor 6	F6WMJ3	S703	KDSVPQVLLPEEEK	S3	1.034	0.389
Arhgef6	Rho guanine nucleotide exchange factor 6	F6WMJ3	Ambiguous	SSSLSAATSSQTNPQVAVPSTAPEQHSEEK	S/T	0.900	0.434
Arhgef7	Rho guanine nucleotide exchange factor 7	Q9ES28	S155	SLDMTDNTNSQLVVR	S1	0.938	0.668
Arhgef7	Rho guanine nucleotide exchange factor 7	Q9ES28	S155	SLDMTDNTNSQLVVR	S1	1.000	0.994
Arhgef7	Rho guanine nucleotide exchange factor 7	Q9ES28	S497	MSGFIYQGK	S2	1.002	0.969
Arhgef7	Rho guanine nucleotide exchange factor 7	Q9ES28	S497	MSGFIYQGK	S2	0.834	0.118
Arhgef7	Rho guanine nucleotide exchange factor 7	Q9ES28	S655	EDLSKSPK	S6	1.018	0.835
Arhgef7	Rho guanine nucleotide exchange factor 7	Q9ES28	S673	KPSDEEFAVR	S3	1.073	0.136
Arhgef7	Rho guanine nucleotide exchange factor 7	Q9ES28; F6WMJ3	S682;S672	STAALEEDAQILK	S1	0.987	0.849
Arhgef7	Rho guanine nucleotide exchange factor 7	Q9ES28	S776	KESAPQVLLPEEEK	S3	0.989	0.864
Arhgef7	Rho guanine nucleotide exchange factor 7	Q9ES28	S155,S164	SLDMTDNTNSQLVVR	S1,S10	0.725	0.092
Arid1a	AT-rich interactive domain-containing protein 1A	E9QAQ7	S1184	SNSVGIQDAFPDGSDPTFQK	S3	1.088	0.605
Arid1a	AT-rich interactive domain-containing protein 1A	E9QAQ7	S1760	VYSPAHTEEEEEEHLDPK	S3	1.059	0.765
Arid1a	AT-rich interactive domain-containing protein 1A	E9QAQ7	S233	SAYPPPPQAYALSSPR	S13	0.981	0.856
Arid1a	AT-rich interactive domain-containing protein 1A	E9QAQ7	S365	SHHAPMSPGSSGGGGQPLAR	S7	0.898	0.542
Arid1a	AT-rich interactive domain-containing protein 1A	E9QAQ7	S384	TPQSSSPMDQMGK	S6	1.033	0.692
Arid1a	AT-rich interactive domain-containing protein 1A	E9QAQ7	S697	GPSPSPVGSPASVAQSR	S3	1.020	0.397
Arid1a	AT-rich interactive domain-containing protein 1A	E9QAQ7	S770	NPQMPQYTSPQPGSALSPR	S14	1.173	0.351
Arid1a	AT-rich interactive domain-containing protein 1A	E9QAQ7	T1890	HLTTVEGTPGTTEQEGPPPDGLPEKR	T8	1.024	0.772
Arid1a	AT-rich interactive domain-containing protein 1A	E9QAQ7	S699,S703	GPSPSPVGSPASVAQSR	S5,S9	1.051	0.248
Arid1a	AT-rich interactive domain-containing protein 1A	E9QAQ7	Ambiguous	TEPGIGPEGNMGTGAPQPNLMPSTPDSGMYSPSR	S/T/Y	1.107	0.676
Arid1a	AT-rich interactive domain-containing protein 1A	E9QAQ7	Ambiguous	TPQSSSPMDQMGK	S/T	1.196	0.112
Arid1a	AT-rich interactive domain-containing protein 1A	E9QAQ7	Ambiguous	NPQMPQYTSPQPGSALSPR	S/Y/T	1.005	0.929
Arid1a	AT-rich interactive domain-containing protein 1A	E9QAQ7	Ambiguous	ELQDGAESNGGGGGGGAGSGGGPGAEPDLK	S	0.846	0.393
Arid1b	AT-rich interactive domain-containing protein 1B	A0A338P6U8	S1602	APSPASFQR	S3	1.008	0.716
Arid1b	AT-rich interactive domain-containing protein 1B	A0A338P6U8	S1809	SSPALAAPDASVDPK	S2	0.902	0.264
Arid2	AT-rich interactive domain-containing protein 2	E9Q7E2	S1385	TSNHVGNGEISPVEPQGTSGATQQDTAK	S11	1.049	0.741
Arid3a	AT-rich interactive domain-containing protein 3A	Q62431	S102	EGTLSSPALHGSVLEGAGHAEGDR	S6	1.032	0.167
Arid3a	AT-rich interactive domain-containing protein 3A	Q62431	S127	HLMDVGSDDDDTK	S7	1.026	0.719
Arid3a	AT-rich interactive domain-containing protein 3A	Q62431	S127	HLMDVGSDDDDTK	S7	1.043	0.469
Arid3a	AT-rich interactive domain-containing protein 3A	Q62431	S78	AAAAGLGHPSSPGGSEDGPPISGDEDTAR	S11	0.958	0.496
Arid3a	AT-rich interactive domain-containing protein 3A	Q62431	Ambiguous	GLSSPNELQAAIDSNR	S	1.016	0.893
Arid3b	AT-rich interactive domain-containing protein 3B	Q9Z1N7	S87	MSEQSNVNSEPEEEEGGLEDEDGDDDVAEVAEK	S9	2.562	0.081
Arid4a	AT-rich interactive domain-containing protein 4A	F8VPQ2	S564	CDSEGEDEEDDTEPCLTGTK	S3	0.889	0.488
Arid4a	AT-rich interactive domain-containing protein 4A	F8VPQ2	S843	NFSSTEDDIDPYEK	S3	0.960	0.677
Arid4a	AT-rich interactive domain-containing protein 4A	F8VPQ2	S867	ILGQQSPEK	S6	0.807	0.069
Arid4a	AT-rich interactive domain-containing protein 4A	F8VPQ2	Ambiguous	NGAGQSSDSEDLPAMDSSSNCTPVKR	S/T	1.067	0.414
Arid5a	AT-rich interactive domain-containing protein 5A	D3Z0W0	S53	GNTEQSEEGDLPQLPVSPKPDDEQSR	S17	1.003	0.977
Arid5b	AT-rich interactive domain-containing protein 5B	Q8BM75	S1032	AVSPLDPAK	S3	0.990	0.900
Arih1	E3 ubiquitin-protein ligase ARIH1	Q9Z1K5	S515	DISQDSLQDIK	S6	1.048	0.864
Arl13b	ADP-ribosylation factor-like protein 13B	Q640N2	S328	EALSQQLDSEDEQDQR	S9	1.093	0.137
Arl13b	ADP-ribosylation factor-like protein 13B	Q640N2	S328	EALSQQLDSEDEQDQRGSESGENSK	S9	1.092	0.415
Arl13b	ADP-ribosylation factor-like protein 13B	Q640N2	S328,S337	EALSQQLDSEDEQDQRGSESGENSK	S9,S18	0.970	0.752
Arl14ep	ARL14 effector protein	Q8BIX3	S182,T/S	ATSAASDRQIIPAK	S6,T/S	0.926	0.661
Arl6ip4	ADP-ribosylation factor-like protein 6-interacting protein 4	Q9JM93	S140	SAGEDNDGPVLTDEQK	S1	1.066	0.206
Arl6ip6	ADP-ribosylation factor-like protein 6-interacting protein 6	Q8BH07	S80	VGDGSPVLPDKR	S5	1.039	0.561
Armcx3	Armadillo repeat-containing X-linked protein 3	Q8BHS6	S113	ASPNSDDTVLSPQELQK	S5	1.064	0.664
Armcx3	Armadillo repeat-containing X-linked protein 3	Q8BHS6	S61	YNDWSDDDDDSNESK	S5	1.005	0.938
Arnt	Aryl hydrocarbon receptor nuclear translocator	P53762	S77	FARSDDEQSSADKER	S4	1.012	0.832
Arpc1b	Actin-related protein 2/3 complex subunit	Q91Z25	S314	ASSEGGAATGAGLDSLHK	S2	0.989	0.946
Arpc3	Actin-related protein 2/3 complex subunit 3	Q9JM76	S172	SLSGPGQ	S3	1.063	0.535
Arrb1	Beta-arrestin-1	J3QNU6	S413	DDKDEEDDGTGSPHLNNR	S12	1.121	0.538
Arrb1	Beta-arrestin-1	J3QNU6	S413	GMKDDKDEEDDGTGSPHLNNR	S15	1.031	0.351
Arrb1	Beta-arrestin-1	J3QNU6	S413	GMKDDKDEEDDGTGSPHLNNR	S15	0.991	0.756
Asap1	Arf-GAP with SH3 domain, ANK repeat and PH domain-containing protein 1	E9QN63	S1045	SHTGDLSPNVQSR	S7	1.032	0.692
Asap1	Arf-GAP with SH3 domain, ANK repeat and PH domain-containing protein 1	E9QN63	S1059	QASEDSNDLTPTLPETPVPLPR	S3	1.201	0.002
Asap1	Arf-GAP with SH3 domain, ANK repeat and PH domain-containing protein 1	E9QN63	S448	GEQSTGENSLEDLTK	S9	1.002	0.981
Asap1	Arf-GAP with SH3 domain, ANK repeat and PH domain-containing protein 1	E9QN63	S732	QDEMDESDDDLDDKPSPIKK	S7	1.098	0.224
Asap1	Arf-GAP with SH3 domain, ANK repeat and PH domain-containing protein 1	E9QN63	S732	QDEMDESDDDLDDKPSPIK	S7	1.067	0.565
Asap1	Arf-GAP with SH3 domain, ANK repeat and PH domain-containing protein 1	E9QN63	S732	QDEMDESDDDLDDKPSPIK	S7	0.995	0.916
Asap1	Arf-GAP with SH3 domain, ANK repeat and PH domain-containing protein 1	E9QN63	S732	QDEMDESDDDLDDKPSPIKK	S7	0.987	0.906
Asap1	Arf-GAP with SH3 domain, ANK repeat and PH domain-containing protein 1	E9QN63	S854	RTLSDPPSPLPHGPPNK	S4	1.071	0.667
Asap1	Arf-GAP with SH3 domain, ANK repeat and PH domain-containing protein 1	E9QN63	S858	TLSDPPSPLPHGPPNK	S7	0.884	0.502
Asap1	Arf-GAP with SH3 domain, ANK repeat and PH domain-containing protein 1	E9QN63	S854,S858	TLSDPPSPLPHGPPNK	S3,S7	1.135	0.012
Asap1	Arf-GAP with SH3 domain, ANK repeat and PH domain-containing protein 1	E9QN63	S858,T/S	RTLSDPPSPLPHGPPNK	S8,T/S	1.101	0.092
Asap2	Arf-GAP with SH3 domain, ANK repeat and PH domain-containing protein 2	E9PX52	S701	LLHEDLDESDDDVDEK	S9	1.029	0.713
Ascc3	Activating signal cointegrator 1 complex subunit 3	E9PZJ8	S2197	DSDVFTDLSV	S9	0.942	0.430
Ascc3	Activating signal cointegrator 1 complex subunit 3	E9PZJ8	S361	AGEDGEVSGEGVLPFDPK	S8	1.366	0.018
Ash2l	Set1/Ash2 histone methyltransferase complex subunit ASH2	Q91X20	S618	SPPWEP	S1	0.921	0.657
Asxl1	Putative Polycomb group protein ASXL1	P59598	S201	HADGESGSPSSSSSGSLALGNSAIR	S8	0.985	0.889
Asxl1	Putative Polycomb group protein ASXL1	P59598	S500	DNLACASASPDRIPTLPQDTVDQETK	S9	1.004	0.944
Asxl2	Putative Polycomb group protein ASXL2	D6REV1	S1121	NTTGEISSKEDESDEDRVGDEQEPISVK	S13	1.060	0.390
Asxl2	Putative Polycomb group protein ASXL2	D6REV1	S1254	LYGSPTQIGPSYR	S4	0.947	0.378
Asxl2	Putative Polycomb group protein ASXL2	D6REV1	S373	SILPSEASPVR	S8	1.077	0.070
Asxl2	Putative Polycomb group protein ASXL2	D6REV1	S477	SENQEALAISPSK	S10	1.052	0.320
Asxl2	Putative Polycomb group protein ASXL2	D6REV1	S765	ATATAASPCHLQDPR	S7	1.043	0.551
Asxl2	Putative Polycomb group protein ASXL2	D6REV1	S1121,S/T	NTTGEISSKEDESDEDRVGDEQEPISVK	S13,S/T	1.038	0.731
Asxl2	Putative Polycomb group protein ASXL2 (Fragment)	D6RI62	Ambiguous	QSDGQSSSPQNSNSSFSSSVK	S	1.005	0.970
Atad1	ATPase family AAA domain-containing protein 1	Q9D5T0	S317	EYVNSTSEESHDEDEIRPVQQQDLHR	S5	0.983	0.831
Atad2	ATPase family AAA domain-containing protein 2	G3X963	S1216	NNSSSSNIENELEEPKETTEGTELRK	S4	1.068	0.644
Atat1	Alpha-tubulin N-acetyltransferase 1	Q8K341	S315	LLLATDPGGSPAQR	S10	0.891	0.129
Ate1	Arginyl-tRNA--protein transferase 1	A0A1L1SQ41	S519	SAMPYGVYK	S1	0.855	0.016
Atf2	Cyclic AMP-dependent transcription factor ATF-2	P16951	T53	NDSVIVADQTPTPTR	T12	1.106	0.184
Atf2	Cyclic AMP-dependent transcription factor ATF-2	P16951	T51,T53	NDSVIVADQTPTPTR	T10,T12	0.985	0.826
Atf4	Cyclic AMP-dependent transcription factor ATF-4	Q06507	S247	APPDNLPSPGGSR	S8	0.937	0.629
Atf6	Cyclic AMP-dependent transcription factor ATF-6 alpha	F6VAN0	S82	AEPQPLSPASSSCSISSPR	S7	1.026	0.823
Atf7	Activating transcription factor 7, isoform CRA_a	Q3TZR9	S423	ESSEPTGSPAPVIQHSSASAPSNGLSVR	S8	1.033	0.844
Atf7	Activating transcription factor 7, isoform CRA_a	Q3TZR9	S44	TDSVIIADQTPTPTR	S3	1.093	0.259
Atf7	Activating transcription factor 7, isoform CRA_a	Q3TZR9	T101,S	GAAGPLDMSLPSTPDIK	T13,S	1.147	0.303
Atf7	Activating transcription factor 7, isoform CRA_a	Q3TZR9	T51,T53	TDSVIIADQTPTPTR	T10,T12	1.042	0.733
Atf7ip	Activating transcription factor 7-interacting protein 1	Q7TT18	S593	SKSEDMDSVESK	S3	1.008	0.673
Atf7ip	Activating transcription factor 7-interacting protein 1	Q7TT18	S707	RQESPPNPPISPGKPANDTNSNNNMTYR	S11	1.082	0.183
Atf7ip	Activating transcription factor 7-interacting protein 1	Q7TT18	S951	IPISTFSPPSSAEQNSSATPR	S7	0.956	0.831
Atf7ip	Activating transcription factor 7-interacting protein 1	Q7TT18	Ambiguous	QESPPNPPISPGKPANDTNSNNNMTYR	S/T/Y	0.884	0.130
Atf7ip	Activating transcription factor 7-interacting protein 1	Q7TT18	Ambiguous	SKSEDMDSVESK	S	1.069	0.204
Atg14	Beclin 1-associated autophagy-related key regulator	Q8CDJ3	S232	TSGRDPADVSSETDSAMTSSMVSK	S20	1.119	0.244
Atg16l1	Autophagy-related protein 16-1	Q8C0J2	S287	SVSSIPVPQDIMDTHPASGK	S1	0.897	0.285
Atg16l2	Autophagy-related protein 16-2	Q6KAU8	S282	SASATSLTLSR	S3	1.010	0.662
Atg2b	Autophagy-related protein 2 homolog B	Q80XK6	S1571	DFATAPPTSPAK	S9	1.072	0.307
Atg2b	Autophagy-related protein 2 homolog B	Q80XK6	S496	SASVDESRPEFICR	S3	1.155	0.022
Atg2b	Autophagy-related protein 2 homolog B	Q80XK6	S885	IAAEEEEENDGHYQEEEDGGAHSLK	S23	1.162	0.166
Atg2b	Autophagy-related protein 2 homolog B	Q80XK6	Ambiguous	DSLSMGVSSEK	S	0.997	0.983
Atg2b	Autophagy-related protein 2 homolog B	Q80XK6	Ambiguous	AALSSRSASVDESRPEFICR	S	1.138	0.003
Atg4c	Cysteine protease ATG4C	Q811C2	Ambiguous	DFDFTSTAASEEDLFSEDER	S/T	0.961	0.915
Atg4d	Cysteine protease ATG4D	Q8BGV9	S184	WVEGTGLASSEMPGPASPSR	S17	1.354	0.005
Atg9a	Autophagy-related protein 9	Q3ZAQ4	S656	SFSPLQPGAAPQGR	S3	1.044	0.272
Atg9a	Autophagy-related protein 9	Q3ZAQ4	S675	VPSTMTGSGVDAR	S8	0.980	0.765
Atg9a	Autophagy-related protein 9	Q3ZAQ4	S828	HPEPVPEEGSEDELPPQVHKV	S10	0.954	0.041
Atg9a	Autophagy-related protein 9	Q3ZAQ4	S828	HPEPVPEEGSEDELPPQVHK	S10	1.013	0.736
Atm	Serine-protein kinase ATM	Q62388	T1891	SATPANSDSESENFLR	T3	0.991	0.859
Atn1	Atrophin-1	O35126	S101	TEQELPRPQSPSDLDSLDGR	S10	1.072	0.229
Atn1	Atrophin-1	O35126	S34	GRASPGGVSTSSSDGK	S4	0.957	0.212
Atn1	Atrophin-1	O35126	S34	GRASPGGVSTSSSDGKAEK	S4	0.836	0.462
Atn1	Atrophin-1	O35126	S617	TASPPGPPQYSK	S3	1.017	0.558
Atn1	Atrophin-1	O35126	S630	RAPSPGSYK	S4	0.968	0.306
Atn1	Atrophin-1	O35126	S646	TATPPGYKPGSPPSFR	S11	1.048	0.441
Atn1	Atrophin-1	O35126	T654	TGTPPGYR	T3	0.960	0.779
Atn1	Atrophin-1	O35126	S101,S107	TEQELPRPQSPSDLDSLDGR	S10,S16	1.002	0.984

Atn1	Atrophin-1	O35126	T638,S646	TATPPGYKPGSPPSFR	T3,S11	0.969	0.837
Atp11a	Probable phospholipid-transporting ATPase IH	P98197	S1146	LLAAQCSSPSGR	S8	1.132	0.071
Atp11c	Phospholipid-transporting ATPase	F6Q8D3	S1123	TFSDESNIL	S3	1.192	0.016
Atp13a1	Manganese-transporting ATPase 13A1	Q9EPE9	S896	DSPVLSNSGPR	S2	1.033	0.427
Atp13a2	Cation-transporting ATPase 13A2	Q9CTG6	Ambiguous	SQAAVGVTPEGTWQDTSELHRQEEAK	T/S	1.026	0.610
Atp13a3	Probable cation-transporting ATPase 13A3	Q5XF89	S1212	ENGSCQIITIA	S4	1.087	0.154
Atp13a3	Probable cation-transporting ATPase 13A3	Q5XF89	S163	GLDEGVSCASLYEK	S7	0.896	0.221
Atp13a3	Probable cation-transporting ATPase 13A3	Q5XF89	S813	LAHDSLEDLEVTR	S5	1.058	0.598
Atp1a1	Sodium/potassium-transporting ATPase subunit alpha-1	Q8VDN2	Y55	YGTDLSR	Y1	1.037	0.750
Atp1a3	Sodium/potassium-transporting ATPase subunit alpha	Q8VCE0	S456	CIELSSGSVK	S8	0.927	0.329
Atp2a2	Sarcoplasmic/endoplasmic reticulum calcium ATPase 2	O55143	S663	AFTGREFDELSPSAQR	S13	0.870	0.157
Atp2a2	Sarcoplasmic/endoplasmic reticulum calcium ATPase 2	O55143	S663	EFDELSPSAQR	S8	1.063	0.241
Atp2b1	Plasma membrane calcium-transporting ATPase 1	G5E829	S1140	SSIHNFMTHPEFR	S2	0.899	0.173
Atp2b1	Plasma membrane calcium-transporting ATPase 1	G5E829	S1155	IEDSEPHIPLIDDTDAEDDAPTK	S4	1.101	0.260
Atp2b1	Plasma membrane calcium-transporting ATPase 1	G5E829	S1155	IEDSEPHIPLIDDTDAEDDAPTKR	S4	1.058	0.510
Atp2b1	Plasma membrane calcium-transporting ATPase 1	G5E829	S1177	RNSSPPPSPNK	S3	1.063	0.795
Atp2b1	Plasma membrane calcium-transporting ATPase 1	G5E829	S1178	NSSPPPSPNK	S3	1.056	0.388
Atp2b1	Plasma membrane calcium-transporting ATPase 1	G5E829	S1211	SATSSSPGSPLHSLETSL	S9	1.081	0.488
Atp2b1	Plasma membrane calcium-transporting ATPase 1	G5E829	S1155,T1165	IEDSEPHIPLIDDTDAEDDAPTK	S4,T14	1.183	0.001
Atp2b1	Plasma membrane calcium-transporting ATPase 1	G5E829	S1177,S1178	RNSSPPPSPNK	S3,S4	1.094	0.232
Atp2b1	Plasma membrane calcium-transporting ATPase 1	G5E829	S1211,S/T	SATSSSPGSPLHSLETSL	S9,S/T	0.964	0.774
Atp5f1a	ATP synthase subunit alpha, mitochondrial	Q03265	S53	TGTAEMSSILEER	S8	0.834	0.475
Atp5f1a	ATP synthase subunit alpha, mitochondrial	Q03265	S53	TGTAEMSSILEER	S8	1.105	0.229
Atp5me	ATP synthase subunit e, mitochondrial	Q06185	S68	ELAEAQDDSILK	S9	0.977	0.840
Atp6v0a2	V-type proton ATPase 116 kDa subunit a isoform 2	P15920	S695	KDSEEEVSLLGNQDIEEGNSR	S3	1.004	0.926
Atp6v0a2	V-type proton ATPase 116 kDa subunit a isoform 2	P15920	S695,S700	KDSEEEVSLLGNQDIEEGNSR	S3,S8	0.994	0.949
Atp6v0d2	V-type proton ATPase subunit d 2	Q80SY3	S345	INSYIPIL	S3	0.704	0.140
Atp6v1b2	V-type proton ATPase subunit B, brain isoform	P62814	Y468	NFITQGPYENR	Y8	0.935	0.742
Atp6v1c1	V-type proton ATPase subunit C 1	Q9Z1G3	S141	SRASAYNNLK	S4	0.972	0.710
Atp6v1h	V-type proton ATPase subunit H	Q8BVE3	S483	QLQSEQPQTAAARS	S14	0.992	0.824
Atp6v1h	V-type proton ATPase subunit H	Q8BVE3	Ambiguous	RSSEDKQEMLQTEGSQCAK	S/T	0.927	0.205
Atp7a	Copper-transporting ATPase 1	A2AG68	T327	YNASLVTPEMLR	T7	0.704	0.148
Atp7a	Copper-transporting ATPase 1	A2AG68	Ambiguous	VSIASEVESTASSPSSSSLQK	S/T	0.971	0.671
Atp8a1	Phospholipid-transporting ATPase	A0A0M3HEP7	S29	TDDVSEKTSLADQEEVR	S9	1.011	0.761
Atp8a1	Phospholipid-transporting ATPase	A0A0M3HEP7	Ambiguous	AEGYEKTDDVSEKTSLADQEEVR	S/T/Y	1.051	0.478
Atp8a1	Phospholipid-transporting ATPase	A0A0M3HEP7	Ambiguous	TDDVSEKTSLADQEEVR	S/T	1.106	0.361
Atr	Serine/threonine-protein kinase ATR	A0A1L1SSL9	S431	IQCQAQQENLSGNNDEVSPK	S18	1.004	0.955
Atrx	Transcriptional regulator ATRX	Q61687	S1090	RPECSSSDTEK	S7	1.079	0.698
Atrx	Transcriptional regulator ATRX	Q61687	S111	AATENSENDITMQSLPK	S6	0.938	0.378
Atrx	Transcriptional regulator ATRX	Q61687	S111	AATENSENDITMQSLPK	S6	0.970	0.638
Atrx	Transcriptional regulator ATRX	Q61687	S1182	QVDITSSSSDIGDDDQNSAGEESSDEQK	S6	1.242	0.047
Atrx	Transcriptional regulator ATRX	Q61687	S1290	QSEESPADDGELR	S5	1.087	0.170
Atrx	Transcriptional regulator ATRX	Q61687	S1290	QSEESPADDGELRR	S5	1.154	0.200
Atrx	Transcriptional regulator ATRX	Q61687	S1290	IGKQSEESPADDGELRR	S8	0.915	0.408
Atrx	Transcriptional regulator ATRX	Q61687	S1290	IGKQSEESPADDGELR	S8	0.925	0.488
Atrx	Transcriptional regulator ATRX	Q61687	S1931	DSSSSGSGSDNDVEVIK	S9	1.097	0.016
Atrx	Transcriptional regulator ATRX	Q61687	S212	YYMSDDISRDSDGMDEQCR	S11	0.990	0.865
Atrx	Transcriptional regulator ATRX	Q61687	S665	QTESNPAMSNSDEESNGTMK	S11	1.050	0.409
Atrx	Transcriptional regulator ATRX	Q61687	S766	KNSTSGSDFDTK	S3	1.019	0.731
Atrx	Transcriptional regulator ATRX	Q61687	S801	QNYSESSNYDSELEREIK	S11	0.781	0.073
Atrx	Transcriptional regulator ATRX	Q61687	S801	QNYSESSNYDSELER	S11	0.996	0.907
Atrx	Transcriptional regulator ATRX	Q61687	S801	RQNYSESSNYDSELER	S12	0.958	0.636
Atrx	Transcriptional regulator ATRX	Q61687	S876	QGGSCSIAGGSIEK	S11	1.007	0.961
Atrx	Transcriptional regulator ATRX	Q61687	S912	EENVNSPEDKR	S6	1.182	0.435
Atrx	Transcriptional regulator ATRX	Q61687	S92	YVESDDEKPTDENVNEK	S4	0.978	0.478
Atrx	Transcriptional regulator ATRX	Q61687	S940	GGSSDGTDRFPK	S3	1.086	0.153
Atrx	Transcriptional regulator ATRX	Q61687	S854,S855	AKTTQEGSSADDTGDTEGR	S8,S9	1.034	0.685
Atrx	Transcriptional regulator ATRX	Q61687	Ambiguous	YYMSDDISRDSDGMDEQCR	S/Y	0.936	0.206
Atrx	Transcriptional regulator ATRX	Q61687	Ambiguous	SASSSSDAEGSSEDNKK	S	1.142	0.265
Atrx	Transcriptional regulator ATRX	Q61687	Ambiguous	TTSTSNPSSPAPDWYK	S/T/Y	1.327	0.077
Atrx	Transcriptional regulator ATRX	Q61687	Ambiguous	QVDITSSSSDIGDDDQNSAGEESSDEQK	S/T	1.124	0.643
Atxn1	Ataxin-1	J3QPR1	S213	AAGLVNPGSPPPPTQQNQYIHISSSPQSSGR	S9	0.945	0.678
Atxn1	Ataxin-1	J3QPR1	S382	EASPSTLNDK	S3	0.994	0.843
Atxn1	Ataxin-1	J3QPR1	S87	ALSAGLDYSPPSAPR	S9	0.897	0.493
Atxn1	Ataxin-1	J3QPR1	S213,S/T/Y	AAGLVNPGSPPPPTQQNQYIHISSSPQSSGR	S9,S/T/Y	0.994	0.971
Atxn1	Ataxin-1	J3QPR1	Ambiguous	GSVMVLPNSSTPSADLEAQQTTHR	S/T	1.417	0.580
Atxn10	Ataxin-10	P28658	S430	NLTEDNSQNQDVIAK	S7	1.165	0.257
Atxn1l	Ataxin-1-like	P0C7T6	S282	RESEALDSASSK	S3	1.309	0.004
Atxn1l	Ataxin-1-like	P0C7T6	S359	DEPSPLNLSHHNLDHQGEGR	S4	0.849	0.060
Atxn1l	Ataxin-1-like	P0C7T6	S359	KDEPSPLNLSHHNLDHQGEGR	S5	1.251	0.107
Atxn1l	Ataxin-1-like	P0C7T6	T328	VEVAAPAHRGTPDTDLEVQR	T11	0.978	0.462
Atxn2	Ataxin-2	E9QM77	S435	VALENDDRSEEEKYTAVQR	S9	0.963	0.322
Atxn2	Ataxin-2	E9QM77	S593	MSSEGPPRMSPK	S10	0.938	0.714
Atxn2	Ataxin-2	E9QM77	S741	ASETSPSFSK	S5	0.950	0.233
Atxn2	Ataxin-2	E9QM77	S832	TNSPSISPSMLSNAEHK	S3	1.041	0.764
Atxn2	Ataxin-2	E9QM77	T710	ALTPSIEAK	T3	0.944	0.384
Atxn2	Ataxin-2	E9QM77	S832,S834	TNSPSISPSMLSNAEHK	S3,S5	1.020	0.692
Atxn2	Ataxin-2	E9QM77	Ambiguous	RGPEVTSQGVQTSSPACK	S/T	0.850	0.434
Atxn2	Ataxin-2	E9QM77	Ambiguous	TNSPSISPSMLSNAEHK	T/S	1.085	0.545
Atxn2l	Ataxin-2-like protein	A0A0U1RPL0	S109	GQTTGKGPPQSPVFEGVYNNSR	S11	1.010	0.901
Atxn2l	Ataxin-2-like protein	A0A0U1RPL0	S109	GPPQSPVFEGVYNNSR	S5	1.097	0.044
Atxn2l	Ataxin-2-like protein	A0A0U1RPL0	S303	EIESSPQYR	S4	1.000	1.000
Atxn2l	Ataxin-2-like protein	A0A0U1RPL0	S337	QGSGRESPSLVSR	S7	0.986	0.843
Atxn2l	Ataxin-2-like protein	A0A0U1RPL0	S556	LQPSSSPETGLDPFPSR	S6	1.142	0.163
Atxn2l	Ataxin-2-like protein	A0A0U1RPL0	S591	EVDGLLTSDPMGSPVSSK	S13	1.072	0.030
Atxn2l	Ataxin-2-like protein	A0A0U1RPL0	S681	STSTPTSPGPR	S7	1.009	0.871
Atxn2l	Ataxin-2-like protein	A0A0U1RPL0	S333,S337	QGSGRESPSLVSR	S3,S7	0.984	0.791
Atxn2l	Ataxin-2-like protein	A0A0U1RPL0	S681,T/S	STSTPTSPGPR	S7,S/T	0.968	0.220
Atxn2l	Ataxin-2-like protein	A0A0U1RPL0	Ambiguous	TLSSPSNRPSGEASVPPTSAVGR	S/T	1.023	0.513
Atxn3	Ataxin-3	Q9CVD2	S219	VLEAADGSGIFDEDEDDLQR	S8	1.059	0.545
Atxn3	Ataxin-3	Q9CVD2	S273	SMCENSPQTSSPDLSSEELRR	S11	1.025	0.506
Atxn3	Ataxin-3	Q9CVD2	S273	SMCENSPQTSSPDLSSEELR	S11	0.918	0.249
Atxn3	Ataxin-3	Q9CVD2	S273	SMCENSPQTSSPDLSSEELR	S11	0.994	0.518
Atxn3	Ataxin-3	Q9CVD2	S273,T/S	SMCENSPQTSSPDLSSEELR	S11,T/S	0.989	0.895
Atxn3	Ataxin-3	Q9CVD2	Ambiguous	SMCENSPQTSSPDLSSEELRR	S/T	0.972	0.721
Atxn3	Ataxin-3	Q9CVD2	Ambiguous	SMCENSPQTSSPDLSSEELR	S/T	0.894	0.478
Atxn7	Ataxin-7	A0A286YDW9	Ambiguous	QVSASSSPPSTPSGLSSVPSSPLSR	S/T	0.993	0.962
Atxn7l1	Ataxin-7-like protein 1	F7C0E4	Ambiguous	DNLLTSSSTQHTVFSAKGPR	S/T	1.011	0.914
Atxn7l3	Ataxin-7-like protein 3 (Fragment)	Z4YN62	Ambiguous	LQWDGSSDLSPSDSGSSK	S	1.004	0.972
Atxn7l3b	Ataxin-7-like protein 3B	Q3UD01	S92	LPLCSLPGDPGDGPQTELQRSPPEFQ	S21	1.126	0.613
Aven	Cell death regulator Aven	Q9D9K3	S82	VEEDSDSETYGEENDEQGNFSR	S5	1.009	0.957
Avl9	Late secretory pathway protein AVL9 homolog	Q80U56	S368	DSLPSDSPPITVQPQANNR	S7	1.065	0.208
AW554918	Expressed sequence AW554918	E9QAK8	S20	DFSDEDQSVAYVPGISTEGNTR	S3	1.082	0.725
AW554918	Expressed sequence AW554918	E9QAK8	S228	SANQVSESFTELR	S1	0.933	0.589
Axin1	Axin-1	E9QMJ8	S82	RSDLDLGYEPEGSASPTPPYLR	S15	0.968	0.620
Axin1	Axin-1	E9QMJ8	S490,S497	TPGCQSPGPGHRSPDSGHVAK	S6,S13	1.047	0.282
Axin1	Axin-1	E9QMJ8	Ambiguous	SDLDLGYEPEGSASPTPPYLR	S/Y/T	1.000	1.000
Axl	Tyrosine-protein kinase receptor UFO	Q00993	Y860	YVLCPSTAPGPTLSADR	Y1	0.859	0.406
Azi2	5-azacytidine-induced protein 2	Q9QYP6	S314	APCYAPSSPLPGDR	S8	1.036	0.064
Azi2	5-azacytidine-induced protein 2	Q9QYP6	S314	APCYAPSSPLPGDRK	S8	1.001	0.980
Azi2	5-azacytidine-induced protein 2	Q9QYP6	Ambiguous	LVPNDGADFPEHSSYGR	S/Y	0.891	0.142
B3gat3	Galactosylgalactosylxylosylprotein 3-beta-glucuronosyltransferase 3	P58158	S329	QGQGSDPAIEV	S5	1.032	0.695
B430306N03Rik	CRKD-binding protein	Q6QX36	S269	LGSSKPDYPRGEDQQLR	S3	1.038	0.648
B430306N03Rik	CRKD-binding protein	Q6QX36	S269	LGSSKPDYPR	S3	0.981	0.829
B630019K06Rik	RIKEN cDNA B630019K06 gene	Q7TNS5	Ambiguous	DGAYATSPLQHLEPR	T/S/Y	0.765	0.300
Bach1	Transcription regulator protein BACH1	P97302	S448	ISESPEPGQR	S4	0.967	0.390
Bach1	Transcription regulator protein BACH1	P97302	S701	GSSAASQELVQESPPTTAAAPEQATLLEPCR	S13	1.060	0.459
Bad	Bcl2-associated agonist of cell death	Q61337	S112	HSSYPAGTEEDEGMEEELSPFR	S3	1.130	0.066
Bad	Bcl2-associated agonist of cell death	Q61337	S112	HSSYPAGTEEDEGMEEELSPFR	S3	0.987	0.921
Bad	Bcl2-associated agonist of cell death	Q61337	S136	SAPPNLWAAQR	S1	1.139	0.016
Bad	Bcl2-associated agonist of cell death	Q61337	S136	SRSAPPNLWAAQR	S3	0.977	0.756
Bad	Bcl2-associated agonist of cell death	Q61337	S155	MSDEFEGSFK	S2	0.892	0.220
Bad	Bcl2-associated agonist of cell death	Q61337	S155	RMSDEFEGSFK	S3	0.910	0.226
Bad	Bcl2-associated agonist of cell death	Q61337	S170	SAGTATQMR	S1	0.997	0.958
Bag2	BAG family molecular chaperone regulator 2	Q91YN9	S73	QISDGEREELNLTANR	S3	1.036	0.717
Bag3	BAG family molecular chaperone regulator 3	Q9JLV1	S138	SQSPLRGGMTEAAQTDK	S3	0.951	0.575
Bag3	BAG family molecular chaperone regulator 3	Q9JLV1	S138	SQSPLRGGMTEAAQTDK	S3	1.015	0.393
Bag3	BAG family molecular chaperone regulator 3	Q9JLV1	S179	SQSPAASDCSSSSSSASLPSSGR	S3	1.005	0.915
Bag3	BAG family molecular chaperone regulator 3	Q9JLV1	S270	AASPFRSPVR	S3	1.007	0.806
Bag3	BAG family molecular chaperone regulator 3	Q9JLV1	S297	SGTPVHCPSPIR	S9	1.037	0.225
Bag3	BAG family molecular chaperone regulator 3	Q9JLV1	S404	AAPSPAPAEPAAPK	S4	0.943	0.157
Bag3	BAG family molecular chaperone regulator 3	Q9JLV1	T560	AATPPNPSNPADSAGNLVAP	T3	0.370	0.361
Bag3	BAG family molecular chaperone regulator 3	Q9JLV1	S270,S274	AASPFRSPVR	S3,S7	0.999	0.979
Bag3	BAG family molecular chaperone regulator 3	Q9JLV1	S297,S/T	SGTPVHCPSPIR	S9,S/T	1.030	0.838
Bag6	Large proline-rich protein BAG6	Q9Z1R2	S1139	LQEDPNYSPQR	S8	1.029	0.426
Bag6	Large proline-rich protein BAG6	Q9Z1R2	S1139	LQEDPNYSPQRFPNAHR	S8	1.016	0.539
Bag6	Large proline-rich protein BAG6	Q9Z1R2	S986	VGDPPQTLPEEPMEVQGAERTSPEPQR	S22	1.074	0.311
Bag6	Large proline-rich protein BAG6	Q9Z1R2	S986	VGDPPQTLPEEPMEVQGAERTSPEPQR	S22	1.051	0.489

Bag6	Large proline-rich protein BAG6	Q9Z1R2	S986	RVGDPPQTLPEEPMEVQGAERTSPEPQR	S23	1.130	0.033
Bag6	Large proline-rich protein BAG6	Q9Z1R2	S995	TSPEPQRENASPAPGTTAEEAMSR	S11	0.990	0.858
Bag6	Large proline-rich protein BAG6	Q9Z1R2	S995	TSPEPQRENASPAPGTTAEEAMSR	S11	1.021	0.892
Bag6	Large proline-rich protein BAG6	Q9Z1R2	S995	ENASPAPGTTAEEAMSR	S4	1.076	0.253
Bag6	Large proline-rich protein BAG6	Q9Z1R2	S995	ENASPAPGTTAEEAMSR	S4	1.050	0.531
Bag6	Large proline-rich protein BAG6	Q9Z1R2	T1102	AAGARPLTSPESLSR	T8	1.186	0.152
Bahd1	Bromo adjacent homology domain-containing 1 protein	Q497V6	S101	SADEADELPPDLPKPPSPTSSSEDAGLVQPR	S17	0.872	0.066
Bahd1	Bromo adjacent homology domain-containing 1 protein	Q497V6	S182	LGDLGEGSRDLSPELAPDEGAR	S12	0.890	0.180
Bahd1	Bromo adjacent homology domain-containing 1 protein	Q497V6	S182	DLSPELAPDEGAR	S3	1.011	0.774
Bahd1	Bromo adjacent homology domain-containing 1 protein	Q497V6	S182	LGDLGEGSRDLSPELAPDEGARR	S12	0.992	0.925
Bahd1	Bromo adjacent homology domain-containing 1 protein	Q497V6	S44	GEPLQMEDSNMEQGTEDVEPGMPESPGHLTGR	S25	1.130	0.156
Baiap2	Brain-specific angiogenesis inhibitor 1-associated protein 2	B1AZ46	S326	SLSPPQSQSK	S3	1.022	0.530
Baiap2	Brain-specific angiogenesis inhibitor 1-associated protein 2	B1AZ46	S367	SSSMAAGLER	S3	0.944	0.416
Baiap2	Brain-specific angiogenesis inhibitor 1-associated protein 2	B1AZ46	S367	SSSMAAGLER	S2	0.991	0.794
Baiap2	Brain-specific angiogenesis inhibitor 1-associated protein 2	B1AZ46	S512	SVSSADVEVARF	S3	1.072	0.676
Baiap2	Brain-specific angiogenesis inhibitor 1-associated protein 2	B1AZ46	T341	LSDSYSNTLPVR	T8	0.964	0.773
Baiap2	Brain-specific angiogenesis inhibitor 1-associated protein 2	B1AZ46	T341	LSDSYSNTLPVRK	T8	0.981	0.681
Baiap2	Brain-specific angiogenesis inhibitor 1-associated protein 2	B1AZ46	T361	NSYATTENKTLPR	T10	1.084	0.498
Baiap2	Brain-specific angiogenesis inhibitor 1-associated protein 2	B1AZ46	Ambiguous	SSSTGNLLDKDDLALPPPDYGTSSR	S/T/Y	1.063	0.765
Bap1	Ubiquitin carboxyl-terminal hydrolase BAP1	Q99PU7	S327	TPVASECTQTDGAEEVAGSCPQTTTHSPPSK	S27	1.133	0.004
Bap1	Ubiquitin carboxyl-terminal hydrolase BAP1	Q99PU7	S395	VPVRAPQQYSEDEDDYEDEDEDVQNTNPAIR	S10	1.101	0.732
Bap1	Ubiquitin carboxyl-terminal hydrolase BAP1	Q99PU7	S395	SRVPVRAPQQYSEDEDDYEDEDEDVQNTNPAIR	S12	1.130	0.454
Bap1	Ubiquitin carboxyl-terminal hydrolase BAP1	Q99PU7	S395	APQQYSEDEDDYEDEDEDVQNTNPAIR	S6	1.006	0.970
Basp1	Brain acid soluble protein 1	Q91XV3	S131	AGEASAESTGAADGAAPEEGEAK	S8	0.979	0.526
Basp1	Brain acid soluble protein 1	Q91XV3	S192	ETPAASEAPSSAAK	S11	1.102	0.531
Basp1	Brain acid soluble protein 1	Q91XV3	S218	APAPAAPAAAEPQAEAPAAAASSEQSVAVKE	S23	1.030	0.655
Basp1	Brain acid soluble protein 1	Q91XV3	T31	AEGAGTEEEGTPK	T6	1.024	0.643
Basp1	Brain acid soluble protein 1	Q91XV3	T31	KAEGAGTEEEGTPK	T7	1.021	0.835
Basp1	Brain acid soluble protein 1	Q91XV3	T36	AEGAGTEEEGTPKESEPQAAADATEVK	T11	0.982	0.704
Basp1	Brain acid soluble protein 1	Q91XV3	S173,S	SDAAPAASDSKPSSAEPAPSSK	S14,S	0.981	0.462
Basp1	Brain acid soluble protein 1	Q91XV3	T31,T36	KAEGAGTEEEGTPKESEPQAAADATEVK	T7,T12	1.055	0.343
Basp1	Brain acid soluble protein 1	Q91XV3	Ambiguous	SDAAPAASDSKPSSAEPAPSSK	S	1.018	0.589
Batf3	Basic leucine zipper transcriptional factor ATF-like 3	Q9D275	S24	SVDAPGNQPQSPKDDDRK	S11	0.875	0.157
Batf3	Basic leucine zipper transcriptional factor ATF-like 3	Q9D275	S24	SVDAPGNQPQSPKDDDR	S11	1.050	0.346
Baz1a	Bromodomain adjacent to zinc finger domain protein 1A	O88379	T1366	GADHTPEHSPSFTNFR	T5	0.984	0.667
Baz1a	Bromodomain adjacent to zinc finger domain protein 1A	O88379	T732	DLDPDVVTEDEDDPGSHKR	T8	1.014	0.717
Baz1b	Tyrosine-protein kinase BAZ1B	Q9Z277	S1338	RQSLELQK	S3	1.054	0.240
Baz1b	Tyrosine-protein kinase BAZ1B	Q9Z277	S1464	FPDRLADDEGDSDSESVGQSR	S12	1.089	0.354
Baz1b	Tyrosine-protein kinase BAZ1B	Q9Z277	S1464	KFPDRLADDEGDSDSESVGQSR	S13	1.085	0.254
Baz1b	Tyrosine-protein kinase BAZ1B	Q9Z277	S1464	LADDEGDSDSESVGQSR	S8	1.079	0.222
Baz1b	Tyrosine-protein kinase BAZ1B	Q9Z277	S158	SDGACDSPSSDKENSSQMAQDLQK	S7	1.119	0.007
Baz1b	Tyrosine-protein kinase BAZ1B	Q9Z277	S1464,S1468	LADDEGDSDSESVGQSR	S8,S12	1.005	0.929
Baz1b	Tyrosine-protein kinase BAZ1B	Q9Z277	S1464,S1472	FPDRLADDEGDSDSESVGQSR	S12,S20	1.037	0.435
Baz1b	Tyrosine-protein kinase BAZ1B	Q9Z277	Ambiguous	DSSSLSSPLNPK	S	0.915	0.311
Baz1b	Tyrosine-protein kinase BAZ1B	Q9Z277	Ambiguous	SDGACDSPSSDKENSSQMAQDLQK	S	1.065	0.281
Baz2a	Bromodomain adjacent to zinc finger domain protein 2A	A0A1W2P6X9	S1175	ELTGSNASTSPAR	S10	1.052	0.154
Baz2a	Bromodomain adjacent to zinc finger domain protein 2A	A0A1W2P6X9	Ambiguous	LSGDSEEMSQSPTGLGQPK	S/T	0.992	0.915
Baz2a	Bromodomain adjacent to zinc finger domain protein 2A	A0A1W2P6X9	Ambiguous	IMEETSGIEEEEEEENTTAVHGR	T/S	1.074	0.828
Baz2b	Bromodomain adjacent to zinc finger domain, 2B	A2AUY4	T1200	DASGGIDLGEEQHPLGTPTPGR	T17	1.031	0.143
Baz2b	Bromodomain adjacent to zinc finger domain, 2B	A2AUY4	T1969	GNVAGDTEDEDSASTSSSLKR	T7	1.064	0.045
Baz2b	Bromodomain adjacent to zinc finger domain, 2B	A2AUY4	T1969	GNVAGDTEDEDSASTSSSLK	T7	1.011	0.901
Baz2b	Bromodomain adjacent to zinc finger domain, 2B	A2AUY4	T1969	KGNVAGDTEDEDSASTSSSLK	T8	0.890	0.259
Bbc3	Bcl-2-binding component 3	Q99ML1	S10	ARQEGSSPEPVEGLAR	S7	1.054	0.577
Bbx	HMG box transcription factor BBX	Q8VBW5	S811	TADGRVSPAGGTLDDKPK	S7	0.959	0.388
Bbx	HMG box transcription factor BBX	Q8VBW5	T576	AEPLTPTEDALPPSLPGQAKPEDSDCHR	T5	1.109	0.335
BC005624	Telomere length and silencing protein 1 homolog	Q3TQI7	S261	VGDTEKPEPERSPPNR	S12	0.946	0.118
BC005624	Telomere length and silencing protein 1 homolog	Q3TQI7	S15,S17	RADSESEEDEQESEEVR	S4,S6	0.997	0.972
BC030867	Uncharacterized protein C17orf53 homolog	Q32P12	S242	GSPAPAPQYLPVAGR	S2	0.988	0.945
BC037034	cDNA sequence BC037034	E9Q2V5	S495	SSPSSPAVR	S2	1.184	0.186
BC037034	cDNA sequence BC037034	E9Q2V5	S517	SQSFSHQQPSR	S3	1.056	0.553
Bcar3	Breast cancer anti-estrogen resistance protein 3	Q9QZK2	S370	TGSEPTLSPALVR	S8	0.910	0.101
Bcar3	Breast cancer anti-estrogen resistance protein 3	Q9QZK2	S466	NSGINYMILDGDDQAR	S2	1.220	0.324
Bcar3	Breast cancer anti-estrogen resistance protein 3	Q9QZK2	S370,T/S	TGSEPTLSPALVR	S8,S/T	1.059	0.756
Bcar3	Breast cancer anti-estrogen resistance protein 3	Q9QZK2	Ambiguous	LPPQSPSMDTSPCPSSPVFR	S/T	0.844	0.233
Bcar3	Breast cancer anti-estrogen resistance protein 3	Q9QZK2	Ambiguous	QSSPLTQDIIQENPLQDR	S/T	0.899	0.772
Bcas3	Breast carcinoma-amplified sequence 3 homolog	Q8CCN5	S503	LTSQDSYNNFTNNNPGNPR	S6	1.145	0.229
Bcas3	Breast carcinoma-amplified sequence 3 homolog	Q8CCN5	S570	SPPQISPSK	S6	1.000	1.000
Bcas3	Breast carcinoma-amplified sequence 3 homolog	Q8CCN5	S823	GVSTVTDAASGTFDR	S10	1.074	0.099
Bcas3	Breast carcinoma-amplified sequence 3 homolog	Q8CCN5	S869	LADAMAESPSRDVVGSGTELQR	S8	1.035	0.704
Bcas3	Breast carcinoma-amplified sequence 3 homolog	Q8CCN5	S886	DVVGSGTELQREGSIETLSNSSGSTSGSIPR	S14	0.976	0.701
Bcas3	Breast carcinoma-amplified sequence 3 homolog	Q8CCN5	S886	EGSIETLSNSSGSTSGSIPR	S3	0.979	0.487
Bcas3	Breast carcinoma-amplified sequence 3 homolog	Q8CCN5	S480,S	CSPVPGLSSSPSGSPLHGK	S2,S	0.848	0.189
Bcas3	Breast carcinoma-amplified sequence 3 homolog	Q8CCN5	S480,S486	QGGRCSPVPGLSSSPSGSPLHGK	S6,S12	1.030	0.641
Bcas3	Breast carcinoma-amplified sequence 3 homolog	Q8CCN5	S869,S877	LADAMAESPSRDVVGSGTELQR	S8,S16	0.999	0.990
Bcas3	Breast carcinoma-amplified sequence 3 homolog	Q8CCN5	S886,S900	EGSIETLSNSSGSTSGSIPR	S3,S17	1.042	0.664
Bcas3	Breast carcinoma-amplified sequence 3 homolog	Q8CCN5	Ambiguous	LADAMAESPSRDVVGSGTELQR	S/T	0.956	0.630
Bckdha	2-oxoisovalerate dehydrogenase subunit alpha	Q3U3J1	S348	SVDEVNYWDKQDHPISR	S1	0.996	0.930
Bckdha	2-oxoisovalerate dehydrogenase subunit alpha	Q3U3J1	S348	SVDEVNYWDK	S1	0.994	0.933
Bckdk	[3-methyl-2-oxobutanoate dehydrogenase [lipoamide]] kinase, mitochondrial	O55028	S31	STSATDTHHVELAR	S1	0.963	0.370
Bcl10	B-cell lymphoma/leukemia 10	Q9Z0H7	S138	CNSDESNLSEK	S3	1.314	0.003
Bcl10	B-cell lymphoma/leukemia 10	Q9Z0H7	S134,S138	CSSCEPFAAGATNNLSRCNSDESNLSEK	S16,S20	1.142	0.071
Bcl2l11	Bcl-2-like protein 11	O54918	Ambiguous	SSSGYFSFDTDRSPAPMSCDK	S/Y/T	1.032	0.790
Bcl2l12	BCL2-like 12 (Proline rich), isoform CRA_b	Q9D3J3	S194	ESSSSPNCSSPSLPCPGPPPPSPDPLAR	S22	1.047	0.732
Bcl3	B-cell lymphoma 3 protein homolog	Q9Z2F6	S377	AASGSQPEPSPDQSATNSPESSSR	S18	1.096	0.447
Bcl6	B-cell lymphoma 6 protein homolog	P41183	S334	GLVSPQSPQK	S7	1.178	0.003
Bcl6	B-cell lymphoma 6 protein homolog	P41183	S344	SDCQPNSPTESCSSK	S7	1.105	0.489
Bcl6	B-cell lymphoma 6 protein homolog	P41183	S458	SLAGSPR	S5	1.223	0.005
Bcl6	B-cell lymphoma 6 protein homolog	P41183	T172	EVVENNMPLRNTPGCESR	T12	1.097	0.495
Bcl6	B-cell lymphoma 6 protein homolog	P41183	S344,S/T	GLVSPQSPQKSDCQPNSPTESCSSK	S17,S/T	1.029	0.738
Bcl7a	B-cell CLL/lymphoma 7 protein family member A	Q9CXE2	Ambiguous	QENSSNSSPAPETNPPVPSDGTEAK	S/T	1.225	0.112
Bcl7b	B-cell CLL/lymphoma 7 protein family member B	Q921K9	S122	VDSSTNSSPSPQQSESLSPAHTSDFR	S18	1.151	0.180
Bcl7b	B-cell CLL/lymphoma 7 protein family member B	Q921K9	S180	EEPVPVETQTTEEEEDSGAPPLKR	S17	1.010	0.913
Bcl7b	B-cell CLL/lymphoma 7 protein family member B	Q921K9	S118,S122	VDSSTNSSPSPQQSESLSPAHTSDFR	S14,S18	0.995	0.849
Bcl7b	B-cell CLL/lymphoma 7 protein family member B	Q921K9	Ambiguous	FCVDQPVVPQTTSES	S/T	0.947	0.516
Bcl9	B-cell CLL/lymphoma 9 protein	Q9D219	S104	SISADSFDQRDPGTPNDDSDIKECNSADHIK	S3	1.353	0.112
Bcl9	B-cell CLL/lymphoma 9 protein	Q9D219	S291	LIPPVGSPGSSTPLPPDGTGPNSTPNNR	S7	0.955	0.390
Bcl9	B-cell CLL/lymphoma 9 protein	Q9D219	T315	AVTPVSQGSNSSSADPK	T3	1.052	0.480
Bcl9	B-cell CLL/lymphoma 9 protein	Q9D219	Ambiguous	IPVEGPLSPSR	S	0.928	0.124
Bcl9	B-cell CLL/lymphoma 9 protein	Q9D219	Ambiguous	SSTPSHGQTPAPEPISAQK	T/S	0.919	0.712
Bcl9l	B-cell CLL/lymphoma 9-like protein	Q67FY2	S118	SVSVDSGEQR	S3	1.294	0.054
Bcl9l	B-cell CLL/lymphoma 9-like protein	Q67FY2	S21,S25	EAPGSPPLSPR	S5,S9	0.990	0.859
Bclaf1	Bcl-2-associated transcription factor 1	Q8K019	S177	KAEGEPQEESPLK	S10	1.036	0.583
Bclaf1	Bcl-2-associated transcription factor 1	Q8K019	S177	KAEGEPQEESPLKSK	S10	0.954	0.633
Bclaf1	Bcl-2-associated transcription factor 1	Q8K019	S177	AEGEPQEESPLK	S9	1.056	0.083
Bclaf1	Bcl-2-associated transcription factor 1	Q8K019	S177	AEGEPQEESPLKSK	S9	1.012	0.724
Bclaf1	Bcl-2-associated transcription factor 1	Q8K019	S183	SQEEPKDTFEHDPSESIDEFNK	S1	0.936	0.080
Bclaf1	Bcl-2-associated transcription factor 1	Q8K019	S221	SATSGDIWPGLSAYDNSPR	S17	1.018	0.814
Bclaf1	Bcl-2-associated transcription factor 1	Q8K019	S267	NTPSQHSHSIQHSPER	S13	0.968	0.018
Bclaf1	Bcl-2-associated transcription factor 1	Q8K019	S289	YSPSQNSPIHHIPSR	S7	1.050	0.577
Bclaf1	Bcl-2-associated transcription factor 1	Q8K019	S289	YSPSQNSPIHHIPSRR	S7	1.198	0.227
Bclaf1	Bcl-2-associated transcription factor 1	Q8K019	S395	FHDSEGDDTEETEDYR	S4	1.033	0.784
Bclaf1	Bcl-2-associated transcription factor 1	Q8K019	S395	QKFHDSEGDDTEETEDYR	S6	1.020	0.654
Bclaf1	Bcl-2-associated transcription factor 1	Q8K019	S412	SVLADQGK	S1	1.060	0.092
Bclaf1	Bcl-2-associated transcription factor 1	Q8K019	S494	EVQSPEQVKSEK	S4	0.923	0.321
Bclaf1	Bcl-2-associated transcription factor 1	Q8K019	S494	EVQSPEQVK	S4	1.037	0.438
Bclaf1	Bcl-2-associated transcription factor 1	Q8K019	S494	KEVQSPEQVK	S5	0.998	0.979
Bclaf1	Bcl-2-associated transcription factor 1	Q8K019	S510	ELFDYSPPLHK	S6	0.979	0.687
Bclaf1	Bcl-2-associated transcription factor 1	Q8K019	S529	SIFREESPLR	S7	0.989	0.788
Bclaf1	Bcl-2-associated transcription factor 1	Q8K019	S656	RIDISPSALR	S5	0.988	0.773
Bclaf1	Bcl-2-associated transcription factor 1	Q8K019	S656	IDISPSALR	S4	1.032	0.405
Bclaf1	Bcl-2-associated transcription factor 1	Q8K019	S759	SSSSSASPSSPSSREEK	S10	1.014	0.945
Bclaf1	Bcl-2-associated transcription factor 1	Q8K019	T305	TITPQNAPR	T3	1.048	0.283
Bclaf1	Bcl-2-associated transcription factor 1	Q8K019	S284,S/Y	YSPSQNSPIHHIPSRR	S2,S/Y	1.028	0.737
Bclaf1	Bcl-2-associated transcription factor 1	Q8K019	S284,S289	YSPSQNSPIHHIPSR	S2,S7	1.062	0.326
Bclaf1	Bcl-2-associated transcription factor 1	Q8K019	S284,S289	SGSGSVGNGSSRYSPSQNSPIHHIPSR	S14,S19	1.090	0.425
Bclaf1	Bcl-2-associated transcription factor 1	Q8K019	S395,T400	FHDSEGDDTEETEDYR	S4,T9	0.967	0.782
Bclaf1	Bcl-2-associated transcription factor 1	Q8K019	S395,T400	QKFHDSEGDDTEETEDYR	S6,T11	1.006	0.857
Bclaf1	Bcl-2-associated transcription factor 1	Q8K019	S284,S289,S/Y	YSPSQNSPIHHIPSRRSPAK	S2,S7,S/Y	0.950	0.484
Bclaf3	BCLAF1 and THRAP3 family member 3	A2AG58	S205	VQEESYPQSPR	S9	0.875	0.021
Bclaf3	BCLAF1 and THRAP3 family member 3	A2AG58	S600	NHQSPSFSK	S4	0.961	0.569
Bclaf3	BCLAF1 and THRAP3 family member 3	A2AG58	S78,S80	SYVNRSPSPNVKPVEK	S6,S8	0.923	0.472
Bclaf3	BCLAF1 and THRAP3 family member 3	A2AG58	Ambiguous	LQNEDENIFHMASPTER	S/T	1.110	0.611
Bcor	BCL-6 corepressor	Q8CGN4	S1348	TDVLCTGEDEDCQAASPLQK	S16	1.023	0.726
Bcor	BCL-6 corepressor	Q8CGN4	S423	DGGSPPLLEK	S4	0.957	0.404
Bcorl1	BCL-6 corepressor-like protein 1	A2AQH4	T1314	QMWDTNEEEEDDEEEGLVKR	T5	0.972	0.651
Bcr	Breakpoint cluster region protein	Q6PAJ1	Ambiguous	GRSSESSCGLDGDYEDAELNPR	S/Y	1.076	0.184
Bhlhe41	Class E basic helix-loop-helix protein 41	Q99PV5	S248	QEPPGDSSPAPK	S8	1.034	0.328
Bhlhe41	Class E basic helix-loop-helix protein 41	Q99PV5	Ambiguous	AAVKQEPPGDSSPAPK	S	0.983	0.814

Bhlhe41	Class E basic helix-loop-helix protein 41	Q99PV5	Ambiguous	TQPGTEPEHDTDTDSGYGGEAEQGR	T/S/Y	0.909	0.730
Bicd2	Protein bicaudal D homolog 2	Q921C5	S578	SPVLLPK	S1	1.097	0.402
Bicd2	Protein bicaudal D homolog 2	Q921C5	Ambiguous	ADGGTGDNSPSPSSSLPSPLSDPR	S/T	1.107	0.145
Bicra	BRD4-interacting chromatin-remodeling complex-associated protein	F8VPZ9	S1427	GAGSPTPLPTK	S4	1.015	0.860
Bicra	BRD4-interacting chromatin-remodeling complex-associated protein	F8VPZ9	T751	GAGLGAQTPDSR	T8	1.001	0.933
Bicra	BRD4-interacting chromatin-remodeling complex-associated protein	F8VPZ9	T931	SPTPPPALHMVPEPTAPPPPPPR	T3	0.899	0.330
Bid	BH3-interacting domain death agonist	P70444	S76	IEPDSESQEEIIHNIAR	S5	0.924	0.707
Bin1	Myc box-dependent-interacting protein 1	O08539	S296	GNKSPSPPPDGSPAATPEIR	S4	0.992	0.816
Bin1	Myc box-dependent-interacting protein 1	O08539	S298	SPSPPPDGSPAATPEIR	S3	1.005	0.847
Bin1	Myc box-dependent-interacting protein 1	O08539	S304	AQPSDNAPEKGNKSPSPPPDGSPAATPEIR	S22	0.923	0.364
Bin1	Myc box-dependent-interacting protein 1	O08539	S324	VNHEPEPASGASPGATIPK	S12	0.989	0.537
Bin1	Myc box-dependent-interacting protein 1	O08539	S296,S298	AQPSDNAPEKGNKSPSPPPDGSPAATPEIR	S14,S16	0.988	0.871
Bin1	Myc box-dependent-interacting protein 1	O08539	S296,S298	GNKSPSPPPDGSPAATPEIR	S4,S6	1.030	0.502
Bin1	Myc box-dependent-interacting protein 1	O08539	S298,S304	SPSPPPDGSPAATPEIR	S3,S9	1.004	0.856
Bin1	Myc box-dependent-interacting protein 1	O08539	S332,S/T	VNHEPEPASGASPGATIPKSPSQLR	S20,S/T	0.995	0.955
Bin1	Myc box-dependent-interacting protein 1	O08539	S296,S298,S304	GNKSPSPPPDGSPAATPEIR	S4,S6,S12	1.027	0.469
Bin1	Myc box-dependent-interacting protein 1	O08539	S296,S304,T308	AQPSDNAPEKGNKSPSPPPDGSPAATPEIR	S14,S22,T26	1.112	0.030
Bin1	Myc box-dependent-interacting protein 1	O08539	S298,S304,T308	SPSPPPDGSPAATPEIR	S3,S9,T13	1.027	0.557
Bin2	Bridging integrator 2	S4R270	S397	DTPSPGDVVLR	S4	1.035	0.506
Bin2	Bridging integrator 2	S4R270	S397	GQTGKDTPSPGDVVLR	S9	1.000	1.000
Bin2	Bridging integrator 2	S4R270	S409	ASSEGAEQSKR	S3	0.996	0.897
Bin2	Bridging integrator 2	S4R270	S425	TSAPPSRPPPPR	S2	1.000	0.998
Bin2	Bridging integrator 2	S4R270	S447	ASGSGSCNAPGSPEGSSQLCSPR	S12	1.023	0.177
Bin2	Bridging integrator 2	S4R270	S460	ASPDASSNPEPAETR	S2	1.088	0.178
Bin2	Bridging integrator 2	S4R270	S493	SATQVFTSENTEL	S1	1.073	0.193
Bin2	Bridging integrator 2	S4R270	S441,S447	ASGSGSCNAPGSPEGSSQLCSPR	S6,S12	1.012	0.787
Bin2	Bridging integrator 2	S4R270	Ambiguous	ASSEGAEQSK	S	0.987	0.845
Bin2	Bridging integrator 2	S4R270	Ambiguous	EKEGAGSSGPEEPR	S	1.043	0.240
Birc6	Baculoviral IAP repeat-containing protein 6	O88738	S3601	LSMTDDSK	S2	0.920	0.509
Birc6	Baculoviral IAP repeat-containing protein 6	O88738	S483	LEGDSDDLLEDSDSEEHSR	S12	0.974	0.572
Birc6	Baculoviral IAP repeat-containing protein 6	O88738	S623	TQGESISEQGSTDNESCTNSELNSPLVR	S24	1.231	0.185
Blm	Bloom syndrome protein homolog	E9PZ97	S1304	YSEWTVPAEDGSPGAR	S12	1.189	0.059
Blm	Bloom syndrome protein homolog	E9PZ97	T1314	GAPEDTEEEEEEAPVSSHYFANQTR	T6	0.969	0.617
Blnk	B-cell linker protein	Q9QUN3	S129	SSQRHSPPFSK	S6	1.028	0.651
Blnk	B-cell linker protein	Q9QUN3	S196	ESSPPPAEK	S3	1.056	0.282
Blnk	B-cell linker protein	Q9QUN3	S220	HMSPPGTVAGR	S3	1.069	0.156
Blnk	B-cell linker protein	Q9QUN3	S220	HMSPPGTVAGR	S3	0.924	0.335
Blnk	B-cell linker protein	Q9QUN3	S230	NSGVWDSK	S2	0.942	0.409
Blnk	B-cell linker protein	Q9QUN3	S244	SSLPAAPSPLPR	S8	0.921	0.370
Blnk	B-cell linker protein	Q9QUN3	Y178	DLLEDEADYVVPVEDNDENYIHPR	Y9	0.894	0.398
Blnk	B-cell linker protein	Q9QUN3	Ambiguous	ESSPPPAEKAPMVNR	S	0.976	0.570
Blnk	B-cell linker protein	Q9QUN3	Ambiguous	ESSPPPAEKAPMVNR	S3	0.917	0.423
Bloc1s4	Biogenesis of lysosome-related organelles complex 1 subunit 4	Q8VED2	T164	AEAELGTFPR	T7	0.895	0.406
Blvra	Biliverdin reductase A	Q9CY64	S236	SGSLEEVPNVGVNK	S3	0.869	0.013
Blvra	Biliverdin reductase A	Q9CY64	S49	ELGSLDNVR	S4	1.117	0.189
Blvrb	Flavin reductase (NADPH)	E9PZC3	S38	DSSRLPSEGPQPAHVVVGDVR	S3	1.002	0.962
Bmi1	Polycomb complex protein BMI-1	P25916	S314	KSSLNGSSATSSG	S/T	1.032	0.743
Bmp2k	BMP-2-inducible protein kinase	Q91Z96	S1087	QNSVHGSFHSAETLR	S3	1.090	0.648
Bmp2k	BMP-2-inducible protein kinase	Q91Z96	S13	SEGSGGGAAAGGAAGGGLGGGFASSSMGVR	S4	0.966	0.180
Bmp2k	BMP-2-inducible protein kinase	Q91Z96	S702	NGGSSPLCK	S5	1.089	0.423
Bmp2k	BMP-2-inducible protein kinase	Q91Z96	S733	GHDDSESDFESDPPSPK	S7	1.053	0.417
Bmp2k	BMP-2-inducible protein kinase	Q91Z96	T644	LGASTPSDK	T5	1.028	0.699
Bmp2k	BMP-2-inducible protein kinase	Q91Z96	S1010,S/T	RDSQSSNEFLTISDSK	S3,S/T	1.089	0.371
Bmp2k	BMP-2-inducible protein kinase	Q91Z96	Ambiguous	DSQSSNEFLTISDSK	S/T	0.845	0.092
Bmpr2	Bone morphogenetic protein receptor type-2	O35607	S515	SVSPTVNPMSTAMQNER	S3	1.088	0.133
Bms1	BMS1 homolog, ribosome assembly protein (Yeast)	Q6PGF5	T817,S820	GKPGPDTQSEDIEEEVKEETDPSEEESAR	T21,S24	0.908	0.319
Bnip2	BCL2/adenovirus E1B 19 kDa protein-interacting protein 2	G5E8U9	S114	GSITEYTATEEK	S2	1.123	0.044
Bnip2	BCL2/adenovirus E1B 19 kDa protein-interacting protein 2	G5E8U9	S114	GSITEYTATEEKGDGR	S2	1.053	0.218
Bnip2	BCL2/adenovirus E1B 19 kDa protein-interacting protein 2	G5E8U9	S114	GSITEYTATEEKGDGRR	S2	1.006	0.944
Bnip2	BCL2/adenovirus E1B 19 kDa protein-interacting protein 2	G5E8U9	S114	KGSITEYTATEEK	S3	1.087	0.154
Bnip2	BCL2/adenovirus E1B 19 kDa protein-interacting protein 2	G5E8U9	S114	KGSITEYTATEEKGDGR	S3	0.952	0.244
Bnip3	BCL2/adenovirus E1B 19 kDa protein-interacting protein 3	O55003	S79	AEIDSHSFGEK	S7	0.986	0.885
Bnip3	BCL2/adenovirus E1B 19 kDa protein-interacting protein 3	O55003	S88	NSTLSEEDYIER	S5	1.022	0.740
Bnip3	BCL2/adenovirus E1B 19 kDa protein-interacting protein 3	O55003	Ambiguous	AEIDSHSFGEKNSTLSEEDYIER	S/T/Y	1.194	0.187
Bnip3l	BCL2/adenovirus E1B 19 kDa protein-interacting protein 3-like	Q9Z2F7	S117	DHSSQSEEEVVEGEK	S4	0.913	0.541
Bnip3l	BCL2/adenovirus E1B 19 kDa protein-interacting protein 3-like	Q9Z2F7	S117	DHSSQSEEEVVEGEKEVEALK	S4	0.914	0.161
Bnip3l	BCL2/adenovirus E1B 19 kDa protein-interacting protein 3-like	Q9Z2F7	S61,S	NGGLEHVPSSSSIHNGDMEK	S9,S	1.007	0.945
Bnip3l	BCL2/adenovirus E1B 19 kDa protein-interacting protein 3-like	Q9Z2F7	Ambiguous	NGGLEHVPSSSSIHNGDMEK	S	0.892	0.491
Bnip3l	BCL2/adenovirus E1B 19 kDa protein-interacting protein 3-like	Q9Z2F7	Ambiguous	NGGLEHVPSSSSIHNGDMEK	S	1.025	0.810
Bnip3l	BCL2/adenovirus E1B 19 kDa protein-interacting protein 3-like	Q9Z2F7	Ambiguous	NGGLEHVPSSSSIHNGDMEK	S	1.010	0.852
Bod1l	Biorientation of chromosomes in cell division protein 1-like 1	E9Q6J5	S1685	AGSLSSEDVDGSQENR	S12	0.993	0.747
Bod1l	Biorientation of chromosomes in cell division protein 1-like 1	E9Q6J5	S1794	EGCAISSETEESAESAMDSTEAK	S7	1.028	0.605
Bod1l	Biorientation of chromosomes in cell division protein 1-like 1	E9Q6J5	S2001	GSDDVLVSGEVPECEVGHMSPR	S20	0.956	0.144
Bod1l	Biorientation of chromosomes in cell division protein 1-like 1	E9Q6J5	S2554	SDHSGTWTSEGSPEK	S12	1.052	0.457
Bod1l	Biorientation of chromosomes in cell division protein 1-like 1	E9Q6J5	S2751	QYSETEPHDTKEENSGDVEEFSSVTSK	S15	0.922	0.805
Bod1l	Biorientation of chromosomes in cell division protein 1-like 1	E9Q6J5	S2777	TNSSTGLEDRDEFSSSEGTGEK	S14	1.099	0.450
Bod1l	Biorientation of chromosomes in cell division protein 1-like 1	E9Q6J5	S2841	TETDITTVEQSSPSGK	S12	1.045	0.343
Bod1l	Biorientation of chromosomes in cell division protein 1-like 1	E9Q6J5	S2841	EDSKTETDITTVEQSSPSGK	S16	0.962	0.628
Bod1l	Biorientation of chromosomes in cell division protein 1-like 1	E9Q6J5	S2841	SKEDSKTETDITTVEQSSPSGK	S18	1.029	0.782
Bod1l	Biorientation of chromosomes in cell division protein 1-like 1	E9Q6J5	S2898	SLTSSDDAESSEPER	S11	1.119	0.134
Bod1l	Biorientation of chromosomes in cell division protein 1-like 1	E9Q6J5	S2920	SVSETSEDKKDEESDEEEEEEEEEPLGATTR	S14	0.908	0.279
Bod1l	Biorientation of chromosomes in cell division protein 1-like 1	E9Q6J5	S3000	AQLSPSVK	S4	1.047	0.387
Bod1l	Biorientation of chromosomes in cell division protein 1-like 1	E9Q6J5	S3010	EVSPPGAR	S3	1.047	0.185
Bod1l	Biorientation of chromosomes in cell division protein 1-like 1	E9Q6J5	S480	YYSDSDDELTVEQR	S3	1.095	0.037
Bod1l	Biorientation of chromosomes in cell division protein 1-like 1	E9Q6J5	T2890	SLTSSDDAESSEPERK	T3	1.565	0.000
Bod1l	Biorientation of chromosomes in cell division protein 1-like 1	E9Q6J5	T2966	LGIPETISPR	T6	0.848	0.130
Bod1l	Biorientation of chromosomes in cell division protein 1-like 1	E9Q6J5	T657	TSTPVILEGAQEETDTR	T3	0.970	0.429
Bod1l	Biorientation of chromosomes in cell division protein 1-like 1	E9Q6J5	S480,S482	YYSDSDDELTVEQR	S3,S5	0.800	0.133
Bod1l	Biorientation of chromosomes in cell division protein 1-like 1	E9Q6J5	Ambiguous	KGSTEALSGCSVEADPEEVEEEEK	S/T	1.062	0.592
Bod1l	Biorientation of chromosomes in cell division protein 1-like 1	E9Q6J5	Ambiguous	ATSDSGGDGLEAALK	S/T	0.986	0.920
Bod1l	Biorientation of chromosomes in cell division protein 1-like 1	E9Q6J5	Ambiguous	RTSTPVILEGAQEETDTR	S/T	1.369	0.003
Bod1l	Biorientation of chromosomes in cell division protein 1-like 1	E9Q6J5	Ambiguous	RRTSTPVILEGAQEETDTR	T/S	0.980	0.791
Bola1	BolA-like protein 1	Q9D8S9	S23	GSAGSAAAGPVEAAIR	S2	1.081	0.236
Bola1	BolA-like protein 1	Q9D8S9	S81	FEGMSPLQR	S5	0.893	0.093
Bola1	BolA-like protein 1	Q9D8S9	S81	FEGMSPLQR	S5	0.972	0.616
Bop1	Ribosome biogenesis protein BOP1	P97452	S84	AEETSEELAQAAPLCSR	S5	0.955	0.761
Borcs6	BLOC-1-related complex subunit 6	Q9D6W8	S130	GSPGGVEMNVELPQQEGDDDDDEDEEAAGR	S2	0.780	0.618
Borcs6	BLOC-1-related complex subunit 6	Q9D6W8	S130	RGSPGGVEMNVELPQQEGDDDDDEDEEAAGR	S3	0.867	0.788
Borcs6	BLOC-1-related complex subunit 6	Q9D6W8	S173	SLDGLSGACGGGGSSSSGETGAGGGR	S1	1.104	0.053
Borcs6	BLOC-1-related complex subunit 6	Q9D6W8	S173	SLDGLSGACGGGGSSSSGETGAGGGRR	S1	0.944	0.128
Borcs6	BLOC-1-related complex subunit 6	Q9D6W8	S173	LQDSRSLDGLSGACGGGGSSSSGETGAGGGR	S6	0.986	0.880
Borcs6	BLOC-1-related complex subunit 6	Q9D6W8	S43	TLSGEEEAESVGVSSR	S3	1.024	0.482
Borcs6	BLOC-1-related complex subunit 6	Q9D6W8	T201	RATISSPLELEGTVSR	T3	0.920	0.332
Borcs6	BLOC-1-related complex subunit 6	Q9D6W8	Ambiguous	ATISSPLELEGTVSR	S/T	0.761	0.550
Bptf	Bromodomain PHD finger transcription factor	A2A654	S1302	DQVLDDVSIQSPGPNCQR	S11	0.980	0.821
Bptf	Bromodomain PHD finger transcription factor	A2A654	S1312	QNSVESDLDAR	S3	1.153	0.016
Bptf	Bromodomain PHD finger transcription factor	A2A654	S1388	STLPASVPKSPR	S10	1.013	0.829
Bptf	Bromodomain PHD finger transcription factor	A2A654	S2468	VQSPPLTR	S3	1.020	0.485
Braf	Serine/threonine-protein kinase B-raf	P28028	S483	RDSSDDWEIPDGQITVGQR	S3	0.984	0.823
Braf	Serine/threonine-protein kinase B-raf	P28028	S766	SASEPSLNR	S3	1.009	0.759
Brap	BRCA1-associated protein	Q99MP8	S116	EQSESVNTAPESPSK	S12	1.125	0.280
Brap	BRCA1-associated protein	Q99MP8	S116	SREQSESVNTAPESPSK	S14	1.152	0.166
Brat1	BRCA1-associated ATM activator 1	E9QLK3	S788	EAGDSPNSASVEAALQR	S5	0.861	0.122
Brca1	Breast cancer type 1 susceptibility protein homolog	P48754	S1479	RNSPSQEELLQPAGSEASSEPHNSTGQSCLPR	S3	0.642	0.017
Brca1	Breast cancer type 1 susceptibility protein homolog	P48754	S686	RASDAFPEEK	S3	1.221	0.007
Brca1	Breast cancer type 1 susceptibility protein homolog	P48754	S706	AGLLTSCSSPR	S9	1.000	1.000
Brca1	Breast cancer type 1 susceptibility protein homolog	P48754	T1199	ISNTPELTR	T4	0.991	0.752
Brca1	Breast cancer type 1 susceptibility protein homolog	P48754	Ambiguous	NSPSQEELLQPAGSEASSEPHNSTGQSCLPR	S/T	1.024	0.876
Brca1	Breast cancer type 1 susceptibility protein homolog	P48754	Ambiguous	VGPNADSLSDREK	S	1.050	0.580
Brd1	Bromodomain-containing 1	E9PZ26	S1186	VHGEPASDLSDID	S10	0.746	0.023
Brd1	Bromodomain-containing 1	E9PZ26	S128	IVEYSPPSAPR	S5	1.164	0.012
Brd1	Bromodomain-containing 1	E9PZ26	S906	NTETQPTSPQLGTK	S8	1.068	0.078
Brd1	Bromodomain-containing 1	E9PZ26	S934	SRSTCGDSEVEEESPGKR	S3	1.150	0.003
Brd1	Bromodomain-containing 1	E9PZ26	S939	STCGDSEVEEESPGKR	S6	1.270	0.032
Brd1	Bromodomain-containing 1	E9PZ26	S983	CASESSICSSNSPLCDSSFSTPK	S3	1.064	0.759
Brd1	Bromodomain-containing 1	E9PZ26	S1183,S1186	VHGEPASDLSDID	S7,S10	1.074	0.296
Brd1	Bromodomain-containing 1	E9PZ26	S939,S/T	SRSTCGDSEVEEESPGK	S8,S/T	0.995	0.939
Brd1	Bromodomain-containing 1	E9PZ26	Ambiguous	SRSTCGDSEVEEESPGK	S/T	1.117	0.183
Brd2	Bromodomain-containing protein 2	Q7JJ13	S631	TAPPVLPTGYDSEEEEESRPMSYDEK	S12	0.972	0.813
Brd2	Bromodomain-containing protein 2	Q7JJ13	S631	TAPPVLPTGYDSEEEEESRPMSYDEK	S12	1.009	0.880
Brd2	Bromodomain-containing protein 2	Q7JJ13	S631	TAPPVLPTGYDSEEEEESRPMSYDEKR	S12	0.941	0.476
Brd2	Bromodomain-containing protein 2	Q7JJ13	Ambiguous	TAPPVLPTGYDSEEEEESRPMSYDEKR	T/S/Y	1.025	0.866
Brd3	Bromodomain-containing protein 3	Q8K2F0	S262	SESPPPLSEPK	S3	1.016	0.514
Brd3	Bromodomain-containing protein 3	Q8K2F0	S564	QASASYDSEEEEEGLPMSYDEKR	S8	0.903	0.410
Brd3	Bromodomain-containing protein 3	Q8K2F0	S564	QASASYDSEEEEEGLPMSYDEKR	S8	0.940	0.318
Brd3	Bromodomain-containing protein 3	Q8K2F0	S564	QASASYDSEEEEEGLPMSYDEK	S8	1.057	0.470
Brd4	Bromodomain-containing protein 4	Q3UH70	S1154	IHSPIIR	S3	1.004	0.959
Brd4	Bromodomain-containing protein 4	Q3UH70	S613	TKPPPTYESEEEDKCKPMSYEEK	S19	0.935	0.393

Brd4	Bromodomain-containing protein 4	Q3UH70	Ambiguous	MPDEPEEPVVTVSSPAVPPPTK	S/T	1.057	0.204
Brd4	Bromodomain-containing protein 4	Q3UH70	Ambiguous	MPDEPEEPVVTVSSPAVPPPTK	T/S	1.102	0.587
Brd4	Bromodomain-containing protein 4	Q3UH70	Ambiguous	HPTTPSSTAK	T/S	0.869	0.158
Brd7	Bromodomain-containing protein 7	O88665	S279	EREDSGDAETQAFR	S5	0.929	0.153
Brd7	Bromodomain-containing protein 7	O88665	S482	SLQDLDMSSPEDEGQTR	S8	0.921	0.380
Brd7	Bromodomain-containing protein 7	O88665	Ambiguous	SLQDLDMSSPEDEGQTR	S/T	0.889	0.222
Brd8	Bromodomain-containing protein 8	D3YZC8	S284	GSLLPTSPR	S7	0.998	0.959
Brd9	Bromodomain-containing protein 9	A0A0R4J175	S103	EHCDSEGEADAFDPGK	S5	1.026	0.609
Brd9	Bromodomain-containing protein 9	A0A0R4J175	S103	EHCDSEGEADAFDPGKK	S5	1.014	0.862
Brd9	Bromodomain-containing protein 9	A0A0R4J175	S103	EKEHCDSEGEADAFDPGK	S7	1.004	0.951
Brd9	Bromodomain-containing protein 9	A0A0R4J175	S588	LSVGEQPDVAHDPYEFLQSPEPAAPAKN	S19	0.923	0.696
Brf1	homolog, subunit of RNA polymerase III transcription initiation factor IIIB (S. cere	G3X8S2	S432	ECISSPSGDPK	S5	0.951	0.166
Brf1	homolog, subunit of RNA polymerase III transcription initiation factor IIIB (S. cere	G3X8S2	S552	GGGSPPRDDSQPPER	S4	0.915	0.335
Brf1	homolog, subunit of RNA polymerase III transcription initiation factor IIIB (S. cere	G3X8S2	T365	CEEDTEDEELEAAASHMNK	T5	1.013	0.905
Brf1	homolog, subunit of RNA polymerase III transcription initiation factor IIIB (S. cere	G3X8S2	T365,T/S	DGSGEDATSSPRCEEDTEDEELEAAASHMNK	T17,T/S	1.017	0.878
Brip1	Fanconi anemia group J protein homolog	Q5SXJ3	Ambiguous	DLLPDTELSPGTEEAK	S/T	1.034	0.861
Brk1	Protein BRICK1	Q91VR8	Y63	RIEYIEAR	Y4	1.071	0.615
Brpf1	Peregrin	A0A0N4SUT9	S77	QSPSPSEVSQSPGR	S4	0.929	0.559
Brpf1	Peregrin	A0A0N4SUT9	S84	QSPSPSEVSQSPGREVMSYAQAQR	S11	0.962	0.583
Brwd1	Bromodomain and WD repeat-containing protein 1	Q921C3	S1789	ADSISEEADSEPESSVLCK	S10	1.098	0.449
Brwd1	Bromodomain and WD repeat-containing protein 1	Q921C3	S1810	ILSDSEDCEEK	S3	1.044	0.348
Brwd1	Bromodomain and WD repeat-containing protein 1	Q921C3	S1830	GPEVEGSPVSEALR	S7	1.057	0.525
Brwd1	Bromodomain and WD repeat-containing protein 1	Q921C3	S1830	RGPEVEGSPVSEALR	S8	0.951	0.196
Brwd1	Bromodomain and WD repeat-containing protein 1	Q921C3	S1857	GSETDVDSDGGAVR	S8	0.996	0.970
Brwd1	Bromodomain and WD repeat-containing protein 1	Q921C3	S1927	LTSDAEDLSLESVCTR	S3	1.019	0.894
Brwd1	Bromodomain and WD repeat-containing protein 1	Q921C3	S1784,S1789	ADSISEEADSEPESSVLCK	S5,S10	0.916	0.211
Brwd1	Bromodomain and WD repeat-containing protein 1	Q921C3	Ambiguous	ASDLGSVTDSDVDCTDNTQTQR	S/T	1.124	0.285
Brwd3	Bromodomain and WD repeat-containing protein 3	A2AHJ4	S1576	LLSASEEDESMGGEEK	S5	1.154	0.355
Bsn	Protein bassoon	O88737	S2822	LLDTSFASSER	S8	0.905	0.487
Bsn	Protein bassoon	O88737	S2860	SLSDPKPLSPTAEESAK	S3	0.935	0.500
Bsn	Protein bassoon	O88737	T2595	IADSSVQTDDEEGEGR	T8	1.058	0.172
Bsn	Protein bassoon	O88737	T2595	RIADSSVQTDDEEGEGR	T9	1.023	0.811
Bsn	Protein bassoon	O88737	T2622	SADCSVQTDDEDNADWEQPVR	T8	0.789	0.248
Bsn	Protein bassoon	O88737	Ambiguous	DYLSDSELNQLR	S/Y	1.016	0.868
Btaf1	B-TFIID TATA-box-binding protein-associated factor 1	E9QAE3	Ambiguous	SRDAVETNEKSNDSTDGEPEEK	S/T	0.918	0.609
Btaf1	B-TFIID TATA-box-binding protein-associated factor 1	E9QAE3	Ambiguous	DAVETNEKSNDSTDGEPEEK	S/T	0.968	0.600
Btbd7	BTB/POZ domain-containing protein 7	Q8CFE5	S722	FGDESPLLTMR	S5	1.003	0.960
Btbd9	BTB/POZ domain-containing protein 9	Q8C726	S599	APSASSLPPSPGPNSR	S10	1.033	0.672
Btf3	Transcription factor BTF3	Q64152	S199	APLATGEDDDDEVPDLVENFDEASKNEAN	S24	0.955	0.758
Btg1	Protein BTG1	P62325	S159	TSPSKNYNMMTVSG	S2	0.943	0.218
Btk	Tyrosine-protein kinase BTK	P35991	Y344	HYVVCSTPQSQYYLAEK	Y12	1.071	0.376
Btk	Tyrosine-protein kinase BTK	P35991	Y551	YVLDDEYTSSVGSK	Y7	0.912	0.424
Bub1b	Mitotic checkpoint serine/threonine-protein kinase BUB1 beta	Q9Z1S0	S502	EISPAENILQEQPDSK	S3	1.219	0.306
Bub1b	Mitotic checkpoint serine/threonine-protein kinase BUB1 beta	Q9Z1S0	Ambiguous	SSSSAPSTSSIK	S/T	1.144	0.170
Bud13	BUD13 homolog	Q8R149	S238	HDLDASPPRK	S6	0.985	0.840
Bud13	BUD13 homolog	Q8R149	S238	HDLDASPPR	S6	1.008	0.874
Bud13	BUD13 homolog	Q8R149	S297	AHHESPDLELHK	S5	0.975	0.749
Bud13	BUD13 homolog	Q8R149	S297	RAHHESPDLELHK	S6	1.011	0.928
Bud13	BUD13 homolog	Q8R149	S376	ASDSDLSPPR	S7	1.033	0.392
Bud13	BUD13 homolog	Q8R149	S371,S376	GVYQKASDSDLSPPR	S7,S12	1.009	0.906
Bud13	BUD13 homolog	Q8R149	T131,S135,S289	HDTPDTSPPR	T3,S7	1.013	0.925
Bud13	BUD13 homolog	Q8R149	T131,T/S;T285,T/S	HDTPDTSPPRK	T3,S/T	0.920	0.519
Bud13	BUD13 homolog	Q8R149	T144,S148,S315	HDTPDPSPPRK	T3,S7	0.953	0.551
Bud13	BUD13 homolog	Q8R149	T144,S148,S315,S495,S714	HDTPDPSPPR	T3,S7	1.026	0.820
Bud13	BUD13 homolog	Q8R149	T196,S200,S404	VRHDTPDLSPPR	T5,S9	0.974	0.732
Bud13	BUD13 homolog	Q8R149	T196,S200,S406	HDTPDLSPPRK	T3,S7	1.076	0.454
Bud13	BUD13 homolog	Q8R149	T196,S200,S406	HDTPDLSPPR	T3,S7	0.987	0.839
Bud13	BUD13 homolog	Q8R149	T222,S226	VRHDTPDPSPPR	T5,S9	1.017	0.874
Bud13	BUD13 homolog	Q8R149	Ambiguous	RGHHGSLGTSSPR	S/T	1.160	0.127
Bzw1	Basic leucine zipper and W2 domain-containing protein 1	A0A087WQS2	S443	NAEEESESEAEEGD	S6	0.856	0.044
C2cd2	C2 domain-containing protein 2	E9Q3C1	S649	DPGLSQSHNDLVFLQQPEGR	S7	0.900	0.064
C2cd2l	Phospholipid transfer protein C2CD2L	Q80X80	S59	DSRPASAAEPGGSLR	S13	1.255	0.015
C2cd2l	Phospholipid transfer protein C2CD2L	Q80X80	S613	SDISERPSVPDDVESETGSTGALETR	S8	1.066	0.436
C2cd2l	Phospholipid transfer protein C2CD2L	Q80X80	S662	EAGLSQSHDDLSNTTATPSVR	S7	0.990	0.802
C2cd2l	Phospholipid transfer protein C2CD2L	Q80X80	S662	QKEAGLSQSHDDLSNTTATPSVR	S9	1.068	0.668
C2cd2l	Phospholipid transfer protein C2CD2L	Q80X80	S48,S52	ADGGRDSRPASAAEPGGSLR	S7,S11	1.077	0.677
C2cd2l	Phospholipid transfer protein C2CD2L	Q80X80	S619,T/S	SDISERPSVPDDVESETGSTGALETR	S14,T/S	0.989	0.873
C2cd3	C2 domain-containing protein 3	Q52KB6	S453	SDVGTSELPSEDDGVEPSR	S10	0.990	0.949
C2cd5	C2 domain-containing protein 5	A0A0N4SW93	S866	ATSVDYSSFADR	S3	1.257	0.006
C2cd5	C2 domain-containing protein 5	Q7TPS5	S295	NQTYSFSPSK	S7	0.991	0.889
C2cd5	C2 domain-containing protein 5	Q7TPS5; A0A0N4SW93	S643;S645	ELYEITPPEVSEEMIGSPIPEPR	S17	1.011	0.946
C2cd5	C2 domain-containing protein 5	Q7TPS5; A0A0N4SW93	S659;S661	SQSESSDEVTELDLSHGK	S3	1.032	0.528
C2cd5	C2 domain-containing protein 5	Q7TPS5	S284,T285	EIPFNEDPNPNTHSSGPSTPLK	S18,T19	1.124	0.218
C2cd5	C2 domain-containing protein 5	Q7TPS5; A0A0N4SW93	S659,S/T;S661,S/T	SQSESSDEVTELDLSHGK	S3,S/T	1.012	0.892
C2cd5	C2 domain-containing protein 5	Q7TPS5	Ambiguous	EIPFNEDPNPNTHSSGPSTPLK	S/T	0.874	0.543
C2cd5	C2 domain-containing protein 5	Q7TPS5	Ambiguous	QSSSSDTDLSLTPK	S/T	1.030	0.579
C2cd5	C2 domain-containing protein 5	Q7TPS5; A0A0N4SW93	Ambiguous	LSSPAAFLPACSSPSR	S	1.102	0.586
C3ar1	C3a anaphylatoxin chemotactic receptor	O09047	S474	NNMSTDV	S4	1.006	0.946
C5ar1	C5a anaphylatoxin chemotactic receptor 1	P30993	S328	NALSEDSVGR	S7	1.041	0.362
C5ar1	C5a anaphylatoxin chemotactic receptor 1	P30993	S328	NALSEDSVGRDSK	S7	0.987	0.805
C5ar1	C5a anaphylatoxin chemotactic receptor 1	P30993	S333	NALSEDSVGRDSKTFTPSTTDTSTR	S12	1.008	0.782
C5ar1	C5a anaphylatoxin chemotactic receptor 1	P30993	S339	TFTPSTTDTSTR	S5	1.054	0.137
C5ar1	C5a anaphylatoxin chemotactic receptor 1	P30993	S348	TFTPSTTDTSTRKSQAV	S14	0.968	0.374
C5ar1	C5a anaphylatoxin chemotactic receptor 1	P30993	T343	TFTPSTTDTSTRK	T9	1.009	0.643
C5ar1	C5a anaphylatoxin chemotactic receptor 1	P30993	S325,S328	NALSEDSVGRDSK	S4,S7	0.983	0.714
C5ar1	C5a anaphylatoxin chemotactic receptor 1	P30993	S333,T337	NALSEDSVGRDSKTFTPSTTDTSTR	S12,T16	0.942	0.520
C5ar1	C5a anaphylatoxin chemotactic receptor 1	P30993	S348,S/T	TFTPSTTDTSTRKSQAV	S14,T/S	1.018	0.821
Caap1	Caspase activity and apoptosis inhibitor 1	Q8VDY9	S183	ILEGDNGLDSDMEEEADDGCK	S10	0.811	0.163
Caap1	Caspase activity and apoptosis inhibitor 1	Q8VDY9	S183	ILEGDNGLDSDMEEEADDGCK	S10	0.986	0.833
Caap1	Caspase activity and apoptosis inhibitor 1	Q8VDY9	S68	STDSSSSVSGSLQQETK	S1	1.131	0.146
Cabin1	Calcineurin binding protein 1, isoform CRA_a	G3X8Q1	S1702	ASPEDGQESPQHPK	S9	1.001	0.992
Cabin1	Calcineurin binding protein 1, isoform CRA_a	G3X8Q1	S2008	APLSPEGEEASGVTEGTSILPQEPR	S4	0.954	0.499
Cabin1	Calcineurin binding protein 1, isoform CRA_a	G3X8Q1	Ambiguous	QQPAPLAPSPAAPTTTAAPTTMAAR	S9	1.105	0.318
Cactin	Cactin	Q9CS00	S148	SPGTATLALSQQQSLQER	S1	0.955	0.807
Cactin	Cactin	Q9CS00	S96	SQSLRPPR	S3	0.963	0.819
Cacul1	CDK2-associated and cullin domain-containing protein 1	Q8R0X2	S366	AGDELAYNSPSACASSR	S9	1.056	0.413
Cad	CAD protein	B2RQC6	S1859	ASDPGLPAEEPK	S2	1.069	0.528
Cad	CAD protein	B2RQC6	S1859	IHRASDPGLPAEEPK	S5	1.003	0.857
Calcoco1	Calcium-binding and coiled-coil domain-containing protein 1	Q8CGU1	S208	GLSRSHGELSEERDILSQQQGDHVAR	S5	0.913	0.272
Calcoco1	Calcium-binding and coiled-coil domain-containing protein 1	Q8CGU1	S563	LPSYGLCESGNTSSSPPGPR	S15	0.989	0.869
Cald1	Caldesmon 1	E9QA16	S254	QTENAFSPSR	S7	1.045	0.657
Camk2d	Calcium/calmodulin-dependent protein kinase type II subunit delta	Q6PHZ2	S333	ESTESSNTTIEDEDVK	S5	1.014	0.745
Camk2d	Calcium/calmodulin-dependent protein kinase type II subunit delta	Q6PHZ2	T287	QETVDCLK	T3	1.193	0.132
Camk2d	Calcium/calmodulin-dependent protein kinase type II subunit delta	Q6PHZ2	Ambiguous	KPDGVKESTESSNTTIEDEDVK	S/T	1.091	0.164
Camk2d	Calcium/calmodulin-dependent protein kinase type II subunit delta	Q6PHZ2	Ambiguous	ESTESSNTTIEDEDVKAR	S/T	0.873	0.443
Camk2d	Calcium/calmodulin-dependent protein kinase type II subunit delta	Q6PHZ2	Ambiguous	ESTESSNTTIEDEDVK	S/T	1.011	0.932
Camk2d	Calcium/calmodulin-dependent protein kinase type II subunit delta	Q6PHZ2	Ambiguous	KPDGVKESTESSNTTIEDEDVK	T/S	1.146	0.115
Camkk2	Calcium/calmodulin-dependent protein kinase kinase 2	Q8C078	S495	SFGNPFEGSR	S1	0.929	0.332
Camkk2	Calcium/calmodulin-dependent protein kinase kinase 2	Q8C078	S85	DQPPEADGQELPLEASDPESR	S16	1.002	0.981
Camkk2	Calcium/calmodulin-dependent protein kinase kinase 2	Q8C078	S91	DQPPEADGQELPLEASDPESRSPLSGR	S22	1.015	0.468
Camkk2	Calcium/calmodulin-dependent protein kinase kinase 2	Q8C078	S85,S	DQPPEADGQELPLEASDPESRSPLSGR	S16,S	0.991	0.973
Camkk2	Calcium/calmodulin-dependent protein kinase kinase 2	Q8C078	Ambiguous	DQPPEADGQELPLEASDPESRSPLSGRK	S	0.982	0.802
Camlg	Calcium signal-modulating cyclophilin ligand	P49070	S53,S55	IMGFHRPGSGSEEENQTK	S9,S11	1.031	0.330
Camsap1	Calmodulin-regulated spectrin-associated protein 1	A0A0A0MQE5	S1133	SPSDPGGELPEK	S1	1.161	0.126
Camsap1	Calmodulin-regulated spectrin-associated protein 1	A0A0A0MQE5	S1133	SLPPSTHPRSPSDPGGELPEK	S10	0.937	0.476
Camsap1	Calmodulin-regulated spectrin-associated protein 1	A0A0A0MQE5	S469	NRPVSQPTSFALHHAASCDVDPSSGDSVSLAR	S5	0.917	0.536
Camsap1	Calmodulin-regulated spectrin-associated protein 1	A0A0A0MQE5	S553	TDVSPPSPQMPR	S7	1.040	0.326
Camsap1	Calmodulin-regulated spectrin-associated protein 1	A0A0A0MQE5	S553	TDVSPPSPQMPR	S7	0.999	0.982
Camsap1	Calmodulin-regulated spectrin-associated protein 1	A0A0A0MQE5	S823	LNSCETK	S3	1.135	0.042
Camsap1	Calmodulin-regulated spectrin-associated protein 1	A0A0A0MQE5	T1111	SKTPTPSVETLPQSR	T3	0.964	0.544
Camsap1	Calmodulin-regulated spectrin-associated protein 1	A0A0A0MQE5	S560,S572	TSPQAPGLVASIRSPQR	S2,S14	1.082	0.450
Camsap1	Calmodulin-regulated spectrin-associated protein 1	A0A0A0MQE5	Ambiguous	TSPQAPGLVASIR	T/S	0.903	0.146
Camsap2	Calmodulin-regulated spectrin-associated protein 2	H7BX08	S1137	ADVSVEKLDGESDKEQFDDDQK	S12	1.078	0.709
Camsap2	Calmodulin-regulated spectrin-associated protein 2	H7BX08	S1257	SIHRDHIESPK	S1	1.022	0.929
Camsap2	Calmodulin-regulated spectrin-associated protein 2	H7BX08	S1308	SESVEGFLSPSR	S9	1.154	0.028
Camsap2	Calmodulin-regulated spectrin-associated protein 2	H7BX08	S456	SVSNEGLTLNNSR	S3	1.322	0.000
Camsap2	Calmodulin-regulated spectrin-associated protein 2	H7BX08	S602	GALSPITDTTEVDTGIHVPSEDIPETMDEDSSLR	S4	0.676	0.065
Camsap2	Calmodulin-regulated spectrin-associated protein 2	H7BX08	S637	DYTVSLDSDMDDASK	S5	0.835	0.200
Camsap2	Calmodulin-regulated spectrin-associated protein 2	H7BX08	S664	EALSPCPSTISTK	S4	1.136	0.081
Camsap2	Calmodulin-regulated spectrin-associated protein 2	H7BX08	Ambiguous	LNQSSPDNLTDTK	S/T	0.881	0.001
Camsap3	Calmodulin-regulated spectrin-associated protein 3	Q80VC9	S193	ASPAAPLDGASPAQPSIR	S2	0.795	0.074
Camsap3	Calmodulin-regulated spectrin-associated protein 3	Q80VC9	S334	DLPDGHAVSPR	S9	1.073	0.531
Camsap3	Calmodulin-regulated spectrin-associated protein 3	Q80VC9	S351	NTETVPSQNNSGSSSPVFNFR	S15	1.129	0.110
Camsap3	Calmodulin-regulated spectrin-associated protein 3	Q80VC9	S431	SVSSDSLGPPRPVSTSSR	S3	1.088	0.188
Camsap3	Calmodulin-regulated spectrin-associated protein 3	Q80VC9	S350,S351	NTETVPSQNNSGSSSPVFNFR	S14,S15	0.754	0.181
Camta2	Calmodulin-binding transcription activator 2	Q80Y50	S974	EDFSEFPDAGASPR	S12	0.699	0.150
Cand1	Cullin-associated NEDD8-dissociated protein 1	Q6ZQ38	Ambiguous	DSSSTNLESMDTS	T/S	1.084	0.222
Canx	Calnexin	P35564	S553	QKSDAEEDGVTGSQDEEDSKPK	S3	1.084	0.459

Canx	Calnexin	P35564	S563	SDAEEDGVTGSQDEEDSKPK	S11	1.043	0.661
Canx	Calnexin	P35564	S582	AEEDEILNRSPR	S10	1.057	0.218
Canx	Calnexin	P35564	T561	QKSDAEEDGVTGSQDEEDSKPKAEEDEILNR	T11	1.067	0.348
Canx	Calnexin	P35564	S553,S563	QKSDAEEDGVTGSQDEEDSKPK	S3,S13	1.112	0.416
Canx	Calnexin	P35564	S553,T/S	QKSDAEEDGVTGSQDEEDSKPKAEEDEILNR	S3,T/S	1.021	0.875
Cap1	Adenylyl cyclase-associated protein 1	P40124	Y353	QVAYIYK	Y6	0.960	0.785
Capg	Capping protein (Actin filament), gelsolin-like	Q99LB4	S324	MRYSPNTQVEILPQGR	S4	0.867	0.070
Capg	Capping protein (Actin filament), gelsolin-like	Q99LB4	S324	MRYSPNTQVEILPQGR	S4	1.020	0.720
Capg	Capping protein (Actin filament), gelsolin-like	Q99LB4	S338	YSPNTQVEILPQGRESPIFK	S16	1.067	0.353
Capg	Capping protein (Actin filament), gelsolin-like	Q99LB4	T133	TTSGATPAAIR	T6	0.970	0.372
Capn1	Calpain-1 catalytic subunit	O35350	S232,Y235	APSDLYQIILK	S3,Y6	1.116	0.125
Capn15	Calpain-15	A0A1D5RLS3	S377	RLSVLEEEVPESSPAR	S3	0.929	0.472
Capn15	Calpain-15	A0A1D5RLS3	T935	ATFGTGGR	T5	1.050	0.719
Capzb	F-actin-capping protein subunit beta	P47757	S184	SGSGTMNLGGSLTR	S3	0.886	0.001
Capzb	F-actin-capping protein subunit beta	P47757	S263	ELSQVLTQR	S3	1.031	0.792
Card11	Caspase recruitment domain-containing protein 11	Q8CIS0	S448	DNGSLDQSLPR	S8	0.929	0.589
Card11	Caspase recruitment domain-containing protein 11	Q8CIS0	S598	STVEEDNDSCGFDALDLDDENHER	S9	0.998	0.971
Card11	Caspase recruitment domain-containing protein 11	Q8CIS0	S891	NTLQPEEMLSTSDPRVSPR	S17	0.980	0.428
Card11	Caspase recruitment domain-containing protein 11	Q8CIS0	S891	NTLQPEEMLSTSDPRVSPR	S17	0.997	0.927
Card11	Caspase recruitment domain-containing protein 11	Q8CIS0	S939	SSLDATK	S2	0.961	0.103
Card11	Caspase recruitment domain-containing protein 11	Q8CIS0	Ambiguous	TNGQEADDSSTSEESPEDSK	S/T	1.394	0.109
Card6	Caspase recruitment domain family, member 6	E9PWH2	S809	QASPVQTHPK	S3	1.067	0.592
Card9	Caspase recruitment domain-containing protein 9	A2AIV8	S50	VLNPDDEEQVLSDPNLVIR	S12	1.187	0.465
Carhsp1	Calcium-regulated heat stable protein 1	Q9CR86	S31	DRSPSPLR	S3	0.981	0.866
Carhsp1	Calcium-regulated heat stable protein 1	Q9CR86	S42	GNVVPSPLPTR	S6	1.059	0.183
Carhsp1	Calcium-regulated heat stable protein 1	Q9CR86	S42	GNVVPSPLPTRR	S6	0.935	0.216
Carhsp1	Calcium-regulated heat stable protein 1	Q9CR86	S59	ASQGPVYK	S2	0.942	0.395
Carhsp1	Calcium-regulated heat stable protein 1	Q9CR86	S42,S/T	DRSPSPLRGNVVPSPLPTR	S14,S/T	1.046	0.643
Carhsp1	Calcium-regulated heat stable protein 1	Q9CR86	Ambiguous	TRTFSATVR	S/T	0.588	0.372
Carmil1	F-actin-uncapping protein LRRC16A	Q6EDY6	S1295	TASRPEDTPDSPSGPSSPK	S17	1.069	0.414
Carmil1	F-actin-uncapping protein LRRC16A	Q6EDY6	S1335	SWGPAQEYQEQK	S1	0.812	0.125
Carmil1	F-actin-uncapping protein LRRC16A	Q6EDY6	S972	RSSGLISELPSEEGR	S3	0.964	0.159
Carmil1	F-actin-uncapping protein LRRC16A	Q6EDY6	Ambiguous	SSGLISELPSEEGR	S	0.978	0.907
Cars	Cysteine--tRNA ligase, cytoplasmic	Q9ER72	S102	VQPQWSPPAGTEPCR	S6	0.909	0.590
Cars	Cysteine--tRNA ligase, cytoplasmic	Q9ER72	S390	ALQEGEGDLSISADR	S12	1.118	0.121
Cars	Cysteine--tRNA ligase, cytoplasmic	Q9ER72	Ambiguous	SSLSGEEVDSK	S	1.019	0.756
Casc3	Protein CASC3	Q8K3W3	S145	GTVTGERQSGDGQESTEPVENK	S9	0.992	0.790
Casc3	Protein CASC3	Q8K3W3	S44	GGGSCSGSVGGGGSGSLPSQR	S14	1.003	0.959
Casc3	Protein CASC3	Q8K3W3	S36,S38	GGGSCSGSVGGGGSGSLPSQR	S6,S8	0.875	0.258
Cask	Peripheral plasma membrane protein CASK (Fragment)	A0A067XG53	S545	TQSSSCEDLPSTTQPK	S5	1.029	0.191
Cask	Peripheral plasma membrane protein CASK (Fragment)	B1AUH5	Ambiguous	FTSSPGLSTEGK	S/T	0.750	0.010
Caskin2	Caskin-2	Q8VHK1	S696	SPSQESIGAR	S3	1.146	0.090
Caskin2	Caskin-2	Q8VHK1	S841	TSPSLTPTR	S2	1.033	0.681
Caskin2	Caskin-2	Q8VHK1	Ambiguous	SRGSGHSQEQPVPQPSVGDPSAPQER	S	1.362	0.518
Caskin2	Caskin-2	Q8VHK1	Ambiguous	VGLSPDSPAGDRNSVGSEGSVGSIR	S	1.165	0.277
Casp3	Caspase-3	P70677	S12	SINNFEVK	S1	1.038	0.708
Casp7	Caspase-7	P97864	S199	GTELDDGIQADSGPINDIDANPR	S12	0.909	0.341
Casp8	Caspase-8	A0A087WQT6	S233	MAELCDSPREQDSESR	S7	1.078	0.227
Casp8	Caspase-8	A0A087WQT6	S233	MAELCDSPREQDSESR	S7	1.055	0.511
Casp8	Caspase-8	A0A087WQT6	T403	GVPDEAGFEQQNHTLEVDSSSHK	T14	1.126	0.063
Cass4	Cas scaffolding protein family member 4	Q08EC4	S297	KNSLPEEPTYAFPTSR	S3	0.875	0.384
Cast	Calpastatin	P51125	S165	SNEQMVSEKPSESK	S1	1.031	0.617
Cast	Calpastatin	P51125	S165	ALSRSNEQMVSEKPSESK	S5	0.890	0.107
Cast	Calpastatin	P51125	S444	SLSESELIGELSADFDR	S3	1.036	0.827
Cast	Calpastatin	P51125	S518	GVVPEDAVETLAGSLGTR	S14	0.990	0.942
Cast	Calpastatin	P51125	T467	STYQDKPSTPAEK	T9	1.034	0.710
Cast	Calpastatin	P51125	T479	SNDTSQTPPGETVPR	T7	0.955	0.153
Cast	Calpastatin	P51125	T479	KSNDTSQTPPGETVPR	T8	0.965	0.293
Cast	Calpastatin	P51125	Ambiguous	ALSRSNEQMVSEKPSESK	S	0.982	0.385
Casz1	Zinc finger protein castor homolog 1	B1AS46	S1761	TPLAALGGPGPAPTAASP	S17	0.906	0.680
Cat	Catalase	P24270	Y231	LVNADGEAVYCK	Y10	1.120	0.375
Cbarp	Voltage-dependent calcium channel beta subunit-associated regulatory protein	E9PW07	S297	HASLDGASPYFK	S3	0.842	0.098
Cbfa2t2	Protein CBFA2T2	O70374	S255	VCTISPAPR	S5	1.050	0.463
Cbfb	Core-binding factor subunit beta	Q08024	S173	QQDPSPGSNLGGGDDLK	S5	0.960	0.380
Cbfb	Core-binding factor subunit beta	Q08024	S173	RQQDPSPGSNLGGGDDLK	S6	1.024	0.525
Cbl	E3 ubiquitin-protein ligase CBL	P22682	S450	QGAEGAPSPNYDDDDDERADDSLFMMK	S8	0.953	0.414
Cbl	E3 ubiquitin-protein ligase CBL	P22682	S450	QGAEGAPSPNYDDDDDERADDSLFMMK	S8	0.951	0.514
Cbl	E3 ubiquitin-protein ligase CBL	P22682	S551	DLPPPPPPDRPYSVGAETRPQR	S13	1.010	0.883
Cbl	E3 ubiquitin-protein ligase CBL	P22682	S722	RPLEATQSSR	S8	1.126	0.364
Cbl	E3 ubiquitin-protein ligase CBL	P22682	S861	ASSYQQGGGATANPVATAPSPQLSSEIER	S20	1.052	0.617
Cbl	E3 ubiquitin-protein ligase CBL	P22682	S861	KASSYQQGGGATANPVATAPSPQLSSEIER	S21	0.970	0.686
Cbl	E3 ubiquitin-protein ligase CBL	P22682	S877	LMSQGYSYQDIQK	S7	1.001	0.978
Cbl	E3 ubiquitin-protein ligase CBL	P22682	S907	EFVSISSPAHVAT	S7	0.796	0.025
Cbl	E3 ubiquitin-protein ligase CBL	P22682	Ambiguous	VERPSSPFSMAPQASLPPVPPR	S	0.992	0.854
Cbl	E3 ubiquitin-protein ligase CBL	P22682	Ambiguous	LPPGEQGESEEDTEYMTPTSRPVGVQKPEPK	Y/S/T	1.065	0.572
Cblb	E3 ubiquitin-protein ligase CBL-B	Q3TTA7	S480	QNSPVTSPGSSPLAQR	S7	1.141	0.395
Cblb	E3 ubiquitin-protein ligase CBL-B	Q3TTA7	S529	SPCGSPTGSPK	S9	1.087	0.258
Cblb	E3 ubiquitin-protein ligase CBL-B	Q3TTA7	S788	GGGSDSASDPVPLPPARPPPRDSPK	S23	1.017	0.707
Cblb	E3 ubiquitin-protein ligase CBL-B	Q3TTA7	S886	ASQDYDQLPSSSDGSQAPARPPKPRPR	S2	1.089	0.501
Cblb	E3 ubiquitin-protein ligase CBL-B	Q3TTA7	S977	EFAFPPPVSPR	S9	1.185	0.025
Cblb	E3 ubiquitin-protein ligase CBL-B	Q3TTA7	S483,S484	QNSPVTSPGSSPLAQR	S10,S11	0.996	0.945
Cblb	E3 ubiquitin-protein ligase CBL-B	Q3TTA7	S521,S525	GIVRSPCGSPTGSPK	S5,S9	1.048	0.224
Cblb	E3 ubiquitin-protein ligase CBL-B	Q3TTA7	S476,S/T	QNSPVTSPGSSPLAQR	S3,S/T	1.090	0.247
Cblb	E3 ubiquitin-protein ligase CBL-B	Q3TTA7	S476,S/T	CTDRQNSPVTSPGSSPLAQR	S7,S/T	1.017	0.866
Cbr1	Carbonyl reductase [NADPH] 1	P48758	S56	LQAEGLSPR	S7	0.975	0.811
Cbx1	Chromobox protein homolog 1	P83917	S89	KADSDSEDKGEESKPK	S4	1.039	0.385
Cbx1	Chromobox protein homolog 1	P83917	S89	ADSDSEDKGEESKPK	S3	0.932	0.495
Cbx3	Chromobox protein homolog 3	P23198	S176	LTWHSCPEDEAQ	S5	0.898	0.279
Cbx3	Chromobox protein homolog 3	P23198	S93	RKSLSDSESDDSK	S3	1.039	0.550
Cbx3	Chromobox protein homolog 3	P23198	S95	SLSDSESDDSK	S3	1.023	0.609
Cbx3	Chromobox protein homolog 3	P23198	S95	KSLSDSESDDSK	S4	0.969	0.444
Cbx3	Chromobox protein homolog 3	P23198	S95	SLSDSESDDSKSK	S3	0.943	0.578
Cbx3	Chromobox protein homolog 3	P23198	S93,S	RKSLSDSESDDSK	S3,S	0.987	0.874
Cbx3	Chromobox protein homolog 3	P23198	S95,S97	SLSDSESDDSK	S3,S5	0.975	0.807
Cbx5	Chromobox protein homolog 5	Q61686	S14	TADSSSSEDEEEYVVEK	S7	0.952	0.443
Cbx5	Chromobox protein homolog 5	Q61686	S97	SSFSNSADDIK	S6	1.019	0.841
Cbx8	Chromobox protein homolog 8	Q9QXV1	S229	RQDSDLVQYGVTSPSSAEASSK	S4	1.280	0.026
Cbx8	Chromobox protein homolog 8	Q9QXV1	S284	HSSGTPASVGSLYR	S3	0.985	0.691
Cc2d1a	Coiled-coil and C2 domain-containing protein 1A	Q8K1A6	S435	LANHDEGSDDEEEETPK	S8	1.044	0.246
Cc2d1a	Coiled-coil and C2 domain-containing protein 1A	Q8K1A6	S435	LANHDEGSDDEEEETPKK	S8	0.982	0.613
Cc2d1a	Coiled-coil and C2 domain-containing protein 1A	Q8K1A6	S459	SSPSKAPPSGPAPAGK	S2	0.996	0.951
Cc2d1b	Coiled-coil and C2 domain-containing protein 1B	Q8BRN9	Ambiguous	LASEDAALVDDDEESDTPAQAPLAK	S/T	0.923	0.482
Ccar2	Cell cycle and apoptosis regulator protein 2	Q8VDP4	S298	EAAPDTGAEPSPEDSDPTYSSK	S11	1.052	0.267
Ccar2	Cell cycle and apoptosis regulator protein 2	Q8VDP4	S626	RPSSGGEEEEK	S4	0.957	0.555
Ccar2	Cell cycle and apoptosis regulator protein 2	Q8VDP4	S677,S680	SVASNQSEMEYSSLQDMPK	S4,S7	0.978	0.712
Ccar2	Cell cycle and apoptosis regulator protein 2	Q8VDP4	Ambiguous	VSKDEVQNEGTAAESDSPLKEDGLLPK	S/T	0.833	0.167
Ccdc115	Coiled-coil domain-containing protein 115	Q8VE99	S100	ADAQTPEEVGPSEASLR	S15	1.072	0.559
Ccdc117	Coiled-coil domain-containing protein 117	Q6PB51	Ambiguous	SLEAATSTEEEMEL	T/S	1.158	0.070
Ccdc12	Coiled-coil domain-containing protein 12	Q8R344	S165	GQEDSLASAVDATTGQEACDSD	S21	1.034	0.872
Ccdc12	Coiled-coil domain-containing protein 12	Q8R344	S165	LKGQEDSLASAVDATTGQEACDSD	S23	0.953	0.498
Ccdc125	Coiled-coil domain-containing protein 125	Q5U465	S56	NSSPPPFPR	S3	0.981	0.272
Ccdc125	Coiled-coil domain-containing protein 125	Q5U465	S95	LSSTDSNSELSDEQLR	S3	1.145	0.120
Ccdc125	Coiled-coil domain-containing protein 125	Q5U465	Ambiguous	LSSTDSNSELSDEQLRR	S/T	1.181	0.487
Ccdc22	Coiled-coil domain-containing protein 22	Q9JIG7	S113	LPSDASEDADQPAGDSAIFLR	S6	1.251	0.629
Ccdc25	Coiled-coil domain-containing protein 25	Q78PG9	S204	VENMSSNQDGNDSDEFM	S13	1.089	0.264
Ccdc25	Coiled-coil domain-containing protein 25	Q78PG9	S204	VENMSSNQDGNDSDEFM	S13	0.976	0.585
Ccdc25	Coiled-coil domain-containing protein 25	Q78PG9	S204	VENMSSNQDGNDSDEFM	S13	0.987	0.698
Ccdc57	Coiled-coil domain-containing protein 57	Q6PHN1	S517	ADSPSTEIQR	S3	1.007	0.918
Ccdc6	Coiled-coil domain-containing protein 6	D3YZP9	S237	LDQPVSAPPSPR	S10	1.075	0.017
Ccdc6	Coiled-coil domain-containing protein 6	D3YZP9	S247	DISMEIDSPENMMR	S8	1.089	0.216
Ccdc6	Coiled-coil domain-containing protein 6	D3YZP9	S316	QLSESESSLEMDDER	S3	1.173	0.001
Ccdc6	Coiled-coil domain-containing protein 6	D3YZP9	S316	QLSESESSLEMDDER	S3	1.070	0.040
Ccdc6	Coiled-coil domain-containing protein 6	D3YZP9	S233,S237	LDQPVSAPPSPR	S6,S10	1.037	0.182
Ccdc6	Coiled-coil domain-containing protein 6	D3YZP9	S316,S318	QLSESESSLEMDDER	S3,S5	1.164	0.228
Ccdc82	Coiled-coil domain-containing protein 82	Q6PG04	S286	NEEGDDENSNQQGENLTTSQLK	S9	1.084	0.134
Ccdc84	Coiled-coil domain-containing protein 84	Q4VA36	S176	SVLEPQAESDPEEGSSAPESWK	S9	1.052	0.747
Ccdc85b	Coiled-coil domain-containing protein 85B	Q6PDY0	S190	DLGDGSSSTSGVGSPDQLPLACSPDD	S14	1.006	0.966
Ccdc86	Coiled-coil domain-containing protein 86	Q9JJ89	S194	QPEPHPGSLQPHQDLGLESPAGQTESSPESPQR	S30	1.073	0.366
Ccdc86	Coiled-coil domain-containing protein 86	Q9JJ89	S283	VIASPQAPASK	S4	1.002	0.974
Ccdc88a	Girdin	Q5SNZ0	S1387	FYDPSPPR	S5	0.973	0.664
Ccdc88a	Girdin	Q5SNZ0	S1417	SLTLTPTR	S1	1.090	0.119
Ccdc88a	Girdin	Q5SNZ0	S1562	DTTSFEDISPQGISDDSSTGSR	S4	0.898	0.274
Ccdc88a	Girdin	Q5SNZ0	S1703	SSSQENLLDEVMK	S2	0.847	0.044
Ccdc88a	Girdin	Q5SNZ0	T1803	DSNPYATLPR	T7	1.008	0.940
Ccdc88a	Girdin	Q5SNZ0	Ambiguous	ASSVISTAEGTTR	S/T	0.862	0.009
Ccdc88a	Girdin	Q5SNZ0	Ambiguous	SSSQENLLDEVMK	S	1.005	0.918
Ccdc88a	Girdin	Q5SNZ0	Ambiguous	QLVNNKDTTSFEDISPQGISDDSSTGSR	T/S	0.919	0.290
Ccdc88b	Coiled-coil domain-containing protein 88B	Q4QRL3	S1350	LGADGAGSTESLGGPLETELPEGR	S8	0.995	0.955

Ccdc88b	Coiled-coil domain-containing protein 88B	Q4QRL3	S1374	EADGTGSSSPAPMR	S8	0.999	0.986
Ccdc88b	Coiled-coil domain-containing protein 88B	Q4QRL3	S1384	VQSSLCLGDETLAGGQR	S3	0.925	0.254
Ccdc88b	Coiled-coil domain-containing protein 88B	Q4QRL3	S1415	SSASFSPGDTPR	S6	0.957	0.190
Ccdc88b	Coiled-coil domain-containing protein 88B	Q4QRL3	S1449	GSGVEWDGSIK	S9	0.945	0.448
Ccdc88b	Coiled-coil domain-containing protein 88B	Q4QRL3	S1480	QFLTPSLSQ	S8	0.998	0.968
Ccdc88b	Coiled-coil domain-containing protein 88B	Q4QRL3	S441	SLEPPPGSPGEASLPGAAPSLQDEVR	S8	0.954	0.504
Ccdc88b	Coiled-coil domain-containing protein 88B	Q4QRL3	S656	SAELTLR	S1	1.039	0.409
Ccdc88b	Coiled-coil domain-containing protein 88B	Q4QRL3	S1350,S1353	LGADGAGSTESLGGPLETELPEGR	S8,S11	0.928	0.157
Ccdc88b	Coiled-coil domain-containing protein 88B	Q4QRL3	S441,S446	SLEPPPGSPGEASLPGAAPSLQDEVR	S8,S13	1.025	0.799
Ccdc88b	Coiled-coil domain-containing protein 88B	Q4QRL3	Ambiguous	EADGTGSSSPAPMRR	S/T	0.996	0.969
Ccdc88b	Coiled-coil domain-containing protein 88B	Q4QRL3	Ambiguous	EADGTGSSSPAPMRR	S/T	0.980	0.837
Ccdc88b	Coiled-coil domain-containing protein 88B	Q4QRL3	Ambiguous	EADGTGSSSPAPMR	T/S	0.968	0.112
Ccdc93	Coiled-coil domain-containing protein 93	Q7TQK5	S305	QSELSAEESPEKLGTSQLHQR	S9	0.957	0.432
Ccdc93	Coiled-coil domain-containing protein 93	Q7TQK5	S305	QSELSAEESPEK	S9	0.971	0.479
Ccl9	C-C motif chemokine 9	P51670	S115	LEQNSQPR	S5	1.083	0.250
Ccm2	Cerebral cavernous malformations protein 2 homolog	Q8K2Y9	S164	TAQDPGISPSQSLCAESSR	S8	0.997	0.934
Ccm2	Cerebral cavernous malformations protein 2 homolog	Q8K2Y9	S393	GIITDSFGR	S6	1.018	0.549
Ccm2	Cerebral cavernous malformations protein 2 homolog	Q8K2Y9	S402	ALSTTSTSTINGNR	S3	1.078	0.253
Ccm2	Cerebral cavernous malformations protein 2 homolog	Q8K2Y9	S402,T/S	ALSTTSTSTINGNR	S3,T/S	0.999	0.994
Ccm2	Cerebral cavernous malformations protein 2 homolog	Q8K2Y9	Ambiguous	TTGSPDDRSAPSEGDEWDR	T/S	0.987	0.885
Ccnb2	G2/mitotic-specific cyclin-B2	P30276	S397	DLASPLLGSP	S9	0.998	0.995
Ccnc	Cyclin C, isoform CRA_f	Q8CAS3	S272	SSLSDSPLMAGPEAAR	S6	1.144	0.222
Ccnc	Cyclin C, isoform CRA_f	Q8CAS3	S272	SSLSDSPLMAGPEAAR	S6	0.994	0.839
Ccnd3	G1/S-specific cyclin-D3	P30282	S264	EAAQTAPSPVPK	S8	1.042	0.198
Ccnd3	G1/S-specific cyclin-D3	P30282	Ambiguous	GSSSQGPSQTSTPTDVTAIHL	S/T	0.952	0.601
Ccne2	G1/S-specific cyclin-E2	Q9Z238	S21	QHAQPNQPDSPQETQIIQAK	S10	1.014	0.786
Ccnk	Cyclin-K	Q3U3M5	S329	KPSPQPSPPR	S7	1.133	0.132
Ccnk	Cyclin-K	Q3U3M5	S341	AVVVSPKEENK	S5	1.092	0.256
Ccnk	Cyclin-K	Q3U3M5	S341	AVVVSPK	S5	1.058	0.342
Ccnk	Cyclin-K	Q3U3M5	S325,S329	KPSPQPSPPR	S3,S7	0.995	0.943
Ccnl1	Cyclin-L1	Q52KE7	Ambiguous	QQASKSPYNGVR	S/Y	0.984	0.762
Ccnl2	Cyclin-L2	Q9JJA7	S345	GGKPSPPSGK	S5	0.990	0.862
Ccnt2	Cyclin-T2	Q7TQK0	Ambiguous	HTADGMPPTVLRSPVGLGPEGVSSASSAR	T/S	0.935	0.391
Ccny	Cyclin-Y	Q8BGU5	S25	LESYRPDTDLSR	S3	1.182	0.014
Ccny	Cyclin-Y	Q8BGU5	S326	SASADNLILPR	S3	0.999	0.982
Ccny	Cyclin-Y	Q8BGU5	S326	RSASADNLILPR	S4	1.038	0.463
Ccny	Cyclin-Y	Q8BGU5	S336	WSPAIIS	S2	0.879	0.527
Ccny	Cyclin-Y	Q8BGU5	Ambiguous	YSSCSTIFLDDSTVSQPNLK	S/T/Y	0.772	0.351
Ccny	Cyclin-Y	Q8BGU5	Ambiguous	NAHSRLESYRPDTDLSR	S/Y/T	1.020	0.809
Ccnyl1	Cyclin Y-like 1	D3YUJ3	S352	SLSADNFIGIQR	S3	1.074	0.584
Ccpg1	Cell cycle progression 1, isoform CRA_d	D3Z7R9	S189	TVSISESEEPPLAEPEDEPSKEPSKR	S5	0.820	0.055
Ccpg1	Cell cycle progression 1, isoform CRA_d	D3Z7R9	S187,S189	TVSISESEEPPLAEPEDEPSKEPSKR	S3,S5	0.966	0.728
Ccr1	C-C chemokine receptor type 1	P51675	S352	TSSISPSTGEHELSAGF	S14	1.021	0.612
Ccr1	C-C chemokine receptor type 1	P51675	S341,S352	TSSISPSTGEHELSAGF	S3,S14	1.114	0.233
Ccr2	C-C chemokine receptor type 2	P51683	Ambiguous	VSSTFTPSTGEQEVSVGL	S/T	0.884	0.440
Ccr3	Probable C-C chemokine receptor type 3	P51678	S356	TSSVSPSTGEQEISVVF	S14	0.995	0.984
Ccr5	C-C chemokine receptor type 5	P51682	S327	RCSIFQQDNPDR	S3	0.963	0.329
Ccr5	C-C chemokine receptor type 5	P51682	S338	CSIFQQDNPDRASSVYTR	S13	1.025	0.663
Ccr5	C-C chemokine receptor type 5	P51682	S344	STGEHEVSTGL	S1	1.054	0.053
Ccr5	C-C chemokine receptor type 5	P51682	S344,S351	STGEHEVSTGL	S1,S8	1.029	0.817
Ccs	Copper chaperone for superoxide dismutase	Q9WU84	S267	KDSAQPPAHL	S3	0.957	0.290
Ccsap	Centriole, cilia and spindle-associated protein	Q8QZT2	S222	AHSVDVEK	S3	0.995	0.957
Ccser2	Serine-rich coiled-coil domain-containing protein 2	Q3UHI0	S205	SSSGESLAQSPDNAK	S10	1.056	0.113
Ccser2	Serine-rich coiled-coil domain-containing protein 2	Q3UHI0	S487	HTSGNNLISPDTDYR	S9	1.002	0.978
Ccsmst1	Protein CCSMST1	Q6RUT7	S34	GRSPAQPADREEEDDDPNLPIQFSGSK	S3	0.980	0.816
Cct3	T-complex protein 1 subunit gamma	P80318	S252	KGESQTDIEITREEDFTR	S4	0.977	0.653
Cct4	T-complex protein 1 subunit delta	P80315	S202	VIDPATATSVDLR	S9	0.851	0.435
Cct4	T-complex protein 1 subunit delta	P80315	S236	VANSGITR	S4	1.037	0.691
Cct4	T-complex protein 1 subunit delta	P80315	T538	IDDVVNTR	T7	0.983	0.690
Cct8	T-complex protein 1 subunit theta	P42932	S23	HFSGLEEAVYR	S3	1.024	0.931
Ccz1	Vacuolar fusion protein CCZ1 homolog	Q8C1Y8	S264	HIEPELAGRDSPVR	S11	1.047	0.551
Ccz1	Vacuolar fusion protein CCZ1 homolog	Q8C1Y8	S264	HIEPELAGRDSPVRAEMPGNLQHYGR	S11	1.027	0.576
Cd200r1	Cell surface glycoprotein CD200 receptor 1	Q9ES57	S276	LEATSAIEEDEMQPYASYTEK	S5	1.067	0.477
Cd200r1	Cell surface glycoprotein CD200 receptor 1	Q9ES57	S276	LEATSAIEEDEMQPYASYTEK	S5	1.053	0.711
Cd200r1	Cell surface glycoprotein CD200 receptor 1	Q9ES57	Y297	SNPLYDTVTK	Y5	1.065	0.560
Cd22	B-cell receptor CD22	Q3UP36	Y863	EDVDYVTLKH	Y5	0.990	0.828
Cd244	Natural killer cell receptor 2B4	Q07763	Ambiguous	ASGSPSAVQEDGR	S	0.971	0.795
Cd274	Programmed cell death 1 ligand 1	Q9EP73	Ambiguous	CGVEDTSSKNRNDTQFEET	S/T	0.987	0.888
Cd28	T-cell-specific surface glycoprotein CD28	P31041	T193	LLQSDYMNMTPR	T10	1.036	0.715
Cd2ap	CD2-associated protein	Q9JLQ0	S458	SVDLDAFVAR	S1	0.922	0.192
Cd2ap	CD2-associated protein	Q9JLQ0	S510	FNGGHSPTQSPEK	S6	1.068	0.087
Cd2ap	CD2-associated protein	Q9JLQ0	S548	SSLSTPSSASK	S4	1.205	0.001
Cd2ap	CD2-associated protein	Q9JLQ0	S580	AEADDGKRNSVDELR	S10	1.018	0.432
Cd2bp2	CD2 antigen cytoplasmic tail-binding protein 2	Q9CWK3	S49	HSLDSDEEDDDEEGSSK	S5	1.028	0.577
Cd300a	CMRF35-like molecule 8	Q6SJQ0	S223	ADRHPELSQNLR	S8	1.013	0.923
Cd34	Hematopoietic progenitor cell antigen CD34	Q64314	S343	LGEDPYYTENGGGQGYSSGPGASPETQGK	S23	1.032	0.712
Cd3eap	DNA-directed RNA polymerase I subunit RPA34	Q76KJ5	Ambiguous	VFTSSPQAR	T/S	1.089	0.165
Cd44	CD44 antigen	A2APM2	S744	SQEMVHLVNKEPSETPDQCMTADETR	S1	1.057	0.505
Cd44	CD44 antigen	A2APM2	S744	SQEMVHLVNKEPSETPDQCMTADETR	S1	0.995	0.941
Cd44	CD44 antigen	A2APM2	S773	NLQSVDMK	S4	1.103	0.363
Cd44	CD44 antigen	A2APM2	T728	LVINGGNGTVEDR	T9	1.022	0.729
Cd47	Leukocyte surface antigen CD47	A0A2R8W6P0	Ambiguous	NVEAQSTEEMFVK	S/T	0.993	0.964
Cd5	T-cell surface glycoprotein CD5	P13379	Ambiguous	SSTQPDNSSDSDYDLQVAQR	S/T/Y	1.078	0.520
Cd68	Macrosialin	A0A0R4J1C8	S118	SSTVGPHPGPPPPSPSPR	S14	1.045	0.368
Cd84	SLAM family member 5	A0A0R4J0K5	S269	TVYAVVSRNAQPTESR	S7	0.982	0.816
Cd84	SLAM family member 5	A0A0R4J0K5	S318	DRSLPK	S3	1.031	0.671
Cd84	SLAM family member 5	A0A0R4J0K5	Y265	TVYAVVSR	Y3	0.975	0.371
Cd84	SLAM family member 5	A0A0R4J0K5	Y280	IYDEIPQSK	Y2	0.973	0.612
Cd84	SLAM family member 5	A0A0R4J0K5	Ambiguous	NAQPTESRIYDEIPQSK	S/T/Y	0.913	0.346
Cd93	Complement component C1q receptor	O89103	S619	KPQNAADSYSWVPER	S8	1.029	0.745
Cd93	Complement component C1q receptor	O89103	Ambiguous	AESQAPENQYSPTPGTDC	S/T/Y	1.027	0.415
Cdan1	Codanin-1	Q8CC12	S148	GSGAAEEGASGESPPWAGGR	S13	1.002	0.986
Cdan1	Codanin-1	Q8CC12	S282	QLQQSPTPASPIPESGSPVPSR	S17	0.951	0.586
Cdc14a	Dual specificity protein phosphatase CDC14A	Q6GQT0	Ambiguous	LASSLGNLNAGTEEPETK	S/T	1.072	0.163
Cdc23	CDC23 (Cell division cycle 23, yeast, homolog), isoform CRA_c	A0A0R4J1W7	T596	VSPLNLSSVTP	T10	1.048	0.709
Cdc23	CDC23 (Cell division cycle 23, yeast, homolog), isoform CRA_a	G3X8W7	T564	NQGETPTSDTPVQL	T7	1.079	0.488
Cdc25b	M-phase inducer phosphatase 2	P30306	S351	SVTPLEEQQLEEPK	S1	1.375	0.070
Cdc26	Anaphase-promoting complex subunit CDC26	Q99JP4	S42	QKEDVEGVGTSDGEGAAGLSSDPK	S11	0.999	0.958
Cdc26	Anaphase-promoting complex subunit CDC26	Q99JP4	T41	EDVEGVGTSDGEGAAGLSSDPK	T8	0.843	0.140
Cdc37	Hsp90 co-chaperone Cdc37	Q61081	S378	AGNEKDVSA	S8	0.974	0.423
Cdc42	Cell division control protein 42 homolog	P60766	T161	YVECSALTQK	T8	0.902	0.184
Cdc42bpb	Serine/threonine-protein kinase MRCK beta	Q7TT50	S1661	SMSDPDQDFDKEPDSDSTK	S3	1.286	0.018
Cdc42bpb	Serine/threonine-protein kinase MRCK beta	Q7TT50	S1661	SMSDPDQDFDKEPDSDSTK	S3	1.150	0.252
Cdc42bpb	Serine/threonine-protein kinase MRCK beta	Q7TT50	S972	TSSASDQETQASK	S5	1.092	0.242
Cdc42bpg	Serine/threonine-protein kinase MRCK gamma	Q80UW5	S1481	SSGPQRPHSFSEAFR	S9	0.991	0.958
Cdc42bpg	Serine/threonine-protein kinase MRCK gamma	Q80UW5	S1491	RPVSTGSDGLPGETDPLVK	S4	0.983	0.760
Cdc42bpg	Serine/threonine-protein kinase MRCK gamma	Q80UW5	S1540	SLPPDPESESSP	S1	0.981	0.498
Cdc42ep3	Cdc42 effector protein 3	Q9CQC5	S89	ANSTSDSMFTETPSPVLK	S3	1.390	0.110
Cdc42ep4	Cdc42 effector protein 4	Q9JM96	S116	NAMSLPQLNEK	S4	0.945	0.406
Cdc42ep4	Cdc42 effector protein 4	Q9JM96	S302	DSSSLSSYTSGVLEER	S4	1.385	0.042
Cdc42ep4	Cdc42 effector protein 4	Q9JM96	S64	EADDESLDEQASASK	S6	1.115	0.236
Cdc42ep4	Cdc42 effector protein 4	Q9JM96	S64	AREADDESLDEQASASK	S8	1.022	0.812
Cdca2	Cell division cycle-associated protein 2	Q14B71	S296	SPETPMCGSSSPSAK	S11	1.042	0.615
Cdca2	Cell division cycle-associated protein 2	Q14B71	S758	ESECLAPQGSLQGSPVSTDSK	S14	1.341	0.109
Cdca2	Cell division cycle-associated protein 2	Q14B71	Ambiguous	ISSQDSPDNYLSGTK	S/Y/T	1.023	0.818
Cdca3	Cell division cycle-associated protein 3	Q99M54	S29	VADPRSPSAGIQR	S6	1.059	0.292
Cdca3	Cell division cycle-associated protein 3	Q99M54	S67	QAQDPDPRSPTLGIAR	S9	1.121	0.009
Cdca7l	Cell division cycle-associated 7-like protein	Q922M5	Ambiguous	RSSFGLR	S	1.190	0.010
Cdiptos	CDIP transferase, opposite strand	A0A1D5RM53	S32	LSCSEEFLTR	S4	1.084	0.274
Cdk11b	Cyclin-dependent kinase 11B	P24788	S47	RDSLEEGELR	S3	1.024	0.680
Cdk11b	Cyclin-dependent kinase 11B	P24788	Ambiguous	RGTSPRPPEGGLGYSQLGDDDLK	T/S/Y	1.055	0.382
Cdk11b	Cyclin-dependent kinase 11B	P24788	Ambiguous	GTSPRPPEGGLGYSQLGDDDLK	T/S/Y	1.140	0.435
Cdk12	Cyclin-dependent kinase 12	Q14AX6	S1079	ETTSGTTAEPVKNNSPAPPQPAPVK	S15	1.064	0.203
Cdk12	Cyclin-dependent kinase 12	Q14AX6	S1079	NNSPAPPQPAPVK	S3	0.969	0.427
Cdk12	Cyclin-dependent kinase 12	Q14AX6	T688	AITPPQQPYK	T3	0.956	0.150
Cdk12	Cyclin-dependent kinase 12	Q14AX6	T889	LYNSEESRPYTNK	T11	0.968	0.713
Cdk12	Cyclin-dependent kinase 12	Q14AX6	S300,S302	QRSVSPYSR	S3,S5	1.022	0.783
Cdk13	Cyclin-dependent kinase 13	Q69ZA1	S384	GGDVSPSPYSSSSWR	S5	0.998	0.952
Cdk13	Cyclin-dependent kinase 13	Q69ZA1	S526	TEHAPSPSSGGTVK	S6	0.891	0.394
Cdk13	Cyclin-dependent kinase 13	Q69ZA1; A0A1Y7VLM0	T1147;T473	QTDPSTPQQESSK	T6	1.013	0.817
Cdk13	Cyclin-dependent kinase 13	Q69ZA1; A0A1Y7VLM0	T871;T257	LYSSEESRPYTNK	T11	1.056	0.331
Cdk13	Cyclin-dependent kinase 13	Q69ZA1	S359,S361	RLPRSPSPYSR	S5,S7	0.973	0.659
Cdk13	Cyclin-dependent kinase 13	A0A1Y7VLM0	Ambiguous	SQSNSNVAPGEKQTDPSTPQQESSK	S/T	1.056	0.638
Cdk13	Cyclin-dependent kinase 13	Q69ZA1; A0A1Y7VLM0	Ambiguous	ENGSGHEAPLQLRPPLEPSTPGSGQDDLIQHQDRR	S/T	1.181	0.082
Cdk14	Cyclin-dependent kinase 14	O35495	S134	ADSYEKLEK	S3	1.012	0.835
Cdk14	Cyclin-dependent kinase 14	O35495	S24	TLSESFSR	S3	1.230	0.011
Cdk14	Cyclin-dependent kinase 14	O35495	S24	RTLSESFSR	S4	1.107	0.189
Cdk14	Cyclin-dependent kinase 14	O35495	Ambiguous	HSSPSSPTSPK	S/T	1.076	0.211

Cdk14	Cyclin-dependent kinase 14	O35495	Ambiguous	HSSPSSPTSPK	S/T	0.977	0.793
Cdk15	Cyclin-dependent kinase 15	E3W997	S77	SNSDSFQEENLR	S3	1.024	0.784
Cdk17	Cyclin-dependent kinase 17	Q8K0D0	S122	MGSDGESDQASGTSSDEVQSPTGVCLR	S20	1.025	0.709
Cdk17	Cyclin-dependent kinase 17	Q8K0D0	S122	MGSDGESDQASGTSSDEVQSPTGVCLR	S20	1.033	0.514
Cdk17	Cyclin-dependent kinase 17	Q8K0D0; Q04735	S146;S119	RLSLPADIR	S3	0.901	0.271
Cdk17	Cyclin-dependent kinase 17	Q8K0D0	S165	LQISSPPFDQPMSR	S5	1.059	0.707
Cdk17	Cyclin-dependent kinase 17	Q8K0D0	S105,S122	MGSDGESDQASGTSSDEVQSPTGVCLR	S3,S20	0.885	0.332
Cdk18	Cyclin-dependent kinase 18	Q04899	S66	RFSMEDLNK	S3	0.887	0.346
Cdk18	Cyclin-dependent kinase 18	Q04899	S75	RLSLPMDIR	S3	0.986	0.931
Cdk2	Cyclin-dependent kinase 2	P97377	T14	IGEGTYGVVYK	T5	1.030	0.507
Cdk2	Cyclin-dependent kinase 2	P97377	T160	TYTHEVVTLWYR	T3	1.114	0.528
Cdk20	Cyclin-dependent kinase 20	Q9JHU3	T222	ILGTPSPR	T4	0.955	0.637
Cdk4	Cyclin-dependent kinase 4	P30285	S300	ALQHSYLHKEESDAE	S12	1.126	0.092
Cdk5rap2	CDK5 regulatory subunit-associated protein 2	Q8K389	S248	SQMASPDENVSSGELR	S5	0.942	0.481
Cdkn1a	Cyclin-dependent kinase inhibitor 1	P39689	S78	VYLSPGSR	S4	1.024	0.624
Cdkn1b	Cyclin-dependent kinase inhibitor 1B	P46414	S10	VSNGSPSLER	S5	1.010	0.714
Cdkn1b	Cyclin-dependent kinase inhibitor 1B	P46414	S178	ANRTEENVSDGSPNAGTVEQTPK	S12	0.949	0.256
Cdkn1b	Cyclin-dependent kinase inhibitor 1B	P46414	S178	TEENVSDGSPNAGTVEQTPK	S9	0.975	0.597
Cdkn1c	Cyclin-dependent kinase inhibitor 1C	P49919	S329	ASNDVPPGCPSPNVAPGVGAVEQTPR	S11	1.057	0.823
Cdkn2aip	CDKN2A-interacting protein	Q8BI72	S124	GISSSNEGVEEPSK	S3	1.458	0.000
Cdkn2aip	CDKN2A-interacting protein	Q8BI72	Ambiguous	SAAQQENSSPSRGSSTK	S/T	1.042	0.744
Cdkn2aip	CDKN2A-interacting protein	Q8BI72	Ambiguous	SSSEANISSSVSK	S	1.068	0.251
Cdr2	Cerebellar degeneration-related protein 2	P97817	Ambiguous	SSSETALSSLAGDDIVK	S/T	0.809	0.132
Cdr2	Cerebellar degeneration-related protein 2	P97817	Ambiguous	SSSETALSSLAGDDIVKDHEDTCIR	S/T	0.943	0.447
Cdr2l	Cerebellar degeneration-related protein 2-like	A2A6T1	S407	DLLGGEESPGEGK	S8	1.128	0.198
Cds1	Phosphatidate cytidylyltransferase 1	P98191	T33	EGEAAGGDHETESTSDKETDIDDR	T11	0.936	0.772
Cds2	Phosphatidate cytidylyltransferase 2	Q99L43	S20	EDAPPEDKESESEAK	S10	1.103	0.521
Cds2	Phosphatidate cytidylyltransferase 2	Q99L43	S32	EDAPPEDKESESEAKLDGETASDSESR	S22	1.081	0.513
Cds2	Phosphatidate cytidylyltransferase 2	Q99L43	S32	LDGETASDSESR	S7	1.130	0.279
Cds2	Phosphatidate cytidylyltransferase 2	Q99L43	S46	AETAPLPTSVDDTPEVLNR	S9	1.169	0.157
Cds2	Phosphatidate cytidylyltransferase 2	Q99L43	T30	LDGETASDSESRAETAPLPTSVDDTPEVLNR	T5	0.947	0.577
Cds2	Phosphatidate cytidylyltransferase 2	Q99L43	S20,S32	EDAPPEDKESESEAKLDGETASDSESR	S10,S22	1.094	0.323
Cds2	Phosphatidate cytidylyltransferase 2	Q99L43	Ambiguous	ESESEAKLDGETASDSESR	S/T	1.167	0.070
Cds2	Phosphatidate cytidylyltransferase 2	Q99L43	Ambiguous	VVREDAPPEDKESESEAK	S	1.019	0.752
Cds2	Phosphatidate cytidylyltransferase 2	Q99L43	Ambiguous	GILTSALEDE	T/S	0.684	0.187
Cdt1	DNA replication factor Cdt1	Q8R4E9	Ambiguous	SAEPGSPGTSTPPLPATPPATPPAASPSALK	T/S	1.238	0.306
Cdv3	Protein CDV3	Q4VAA2	S155	EDPGDNWEEGGGGSGAEK	S14	0.932	0.535
Cdv3	Protein CDV3	Q4VAA2	S51	AANAASGAGGSSAAAGSRPGDGGSLGSGAR	S24	0.968	0.439
Cdv3	Protein CDV3	Q4VAA2	Y120	EVDYSGLR	Y4	0.953	0.335
Ceacam1	Carcinoembryonic antigen-related cell adhesion molecule 1	P31809	Y515	ATETVYSEVK	Y6	1.011	0.703
Cebpb	CCAAT/enhancer-binding protein beta	P28033	T188,S/T/Y	AYLGYQATPSGSSGSLSTSSSSSPPGTPSPADAK	T27,S/T/Y	0.851	0.346
Cebpb	CCAAT/enhancer-binding protein beta	P28033	Ambiguous	AYLGYQATPSGSSGSLSTSSSSSPPGTPSPADAK	S/T/Y	0.987	0.837
Cebpz	CCAAT/enhancer-binding protein zeta	A0A0R4J046	Ambiguous	ETEPESSAEAEKPK	S/T	0.998	0.970
CEFIP	Cardiac-enriched FHL2-interacting protein	D3Z1D3	T153,T155	TETQSCDSRPPPSKPPALK	T1,T3	0.839	0.122
Cemip2	Cell surface hyaluronidase	Q5FWI3	S24	SPGYVPGK	S1	1.058	0.342
Cemip2	Cell surface hyaluronidase	Q5FWI3	S71	SQDAPATFAFSPEEQRTPSESR	S19	1.078	0.431
Cenpc	Centromere protein C	P49452	S158	VASVSRSPVDR	S7	1.075	0.513
Cenpc	Centromere protein C	P49452	S699	LVLPSNSPNVR	S7	1.001	0.992
Cenpc	Centromere protein C	P49452	Ambiguous	NYEMPGSSNSEMGDEQDQK	S/Y	1.116	0.454
Cenpf	Centromere protein F	E9Q3P4	S827	SDCSVDGGRSPEHIAILQNR	S10	1.071	0.435
Cenpj	Centromere protein J	Q569L8	S457	ESLSPPGLK	S4	1.015	0.808
Cenps	Centromere protein S	Q9D084	Ambiguous	ESMAEELDGAEELQSES	S	1.006	0.968
Cenpt	Centromere protein T	Q3TJM4	S279	HSLPSPSRPGVEDVER	S5	1.069	0.235
Cenpt	Centromere protein T	Q3TJM4	S313	SGPAASPSPFLEPQPPPAEPR	S6	1.176	0.353
Cenpu	Centromere protein U	Q8C4M7	S106	NLDPSEDEASGNESIK	S5	0.992	0.205
Cenpu	Centromere protein U	Q8C4M7	S164	QAAPAAPSPPERPAEPVTPR	S8	1.009	0.875
Cenpu	Centromere protein U	Q8C4M7	S186	LHSAQLSPVDETPATQSQLK	S7	0.991	0.900
Cenpu	Centromere protein U	Q8C4M7	S106,S111	NLDPSEDEASGNESIK	S5,S10	1.090	0.345
Cenpu	Centromere protein U	Q8C4M7	S164,T174	QAAPAAPSPPERPAEPVTPR	S8,T18	1.095	0.432
Cenpu	Centromere protein U	Q8C4M7	S182,S186	LHSAQLSPVDETPATQSQLK	S3,S7	1.169	0.007
Cep131	Centrosomal protein of 131 kDa	J3QMP9	S222	EGGEGSDLGKPR	S6	0.785	0.063
Cep131	Centrosomal protein of 131 kDa	J3QMP9	S431	QDLAVSGSSR	S8	0.963	0.442
Cep131	Centrosomal protein of 131 kDa	J3QMP9	T473	YAWTDEEEDANSLTADNLEK	T4	1.186	0.383
Cep152	Centrosomal protein of 152 kDa	A2AUM9	S1575	ALMCGSPTETDSIASEK	S6	0.955	0.832
Cep152	Centrosomal protein of 152 kDa	A2AUM9	S1733	LTAPMPSQQDSGFDSPLE	S15	1.031	0.805
Cep170	Centrosomal protein of 170 kDa	Q6A065	S1102	LGEASDSELADADK	S5	1.026	0.672
Cep170	Centrosomal protein of 170 kDa	Q6A065	S1102	ARLGEASDSELADADK	S7	0.961	0.837
Cep170	Centrosomal protein of 170 kDa	Q6A065	S1155	SDSEATISR	S1	1.045	0.642
Cep170	Centrosomal protein of 170 kDa	Q6A065	S1155	LGSLSARSDSEATISR	S8	0.968	0.427
Cep170	Centrosomal protein of 170 kDa	Q6A065	S1188	LVPSDKLSPR	S8	0.981	0.829
Cep170	Centrosomal protein of 170 kDa	Q6A065	S1231	RFPTDYASTSEDEFGSNR	S10	1.053	0.637
Cep170	Centrosomal protein of 170 kDa	Q6A065	S1231	FPTDYASTSEDEFGSNR	S9	0.973	0.795
Cep170	Centrosomal protein of 170 kDa	Q6A065	S1529	SSPVNNHSSPSQTPALCPPETR	S9	1.021	0.845
Cep170	Centrosomal protein of 170 kDa	Q6A065	S662	VVPQRSEIGEK	S6	1.066	0.352
Cep170	Centrosomal protein of 170 kDa	Q6A065	S1150,S1155	LGSLSARSDSEATISR	S3,S8	1.192	0.081
Cep170	Centrosomal protein of 170 kDa	Q6A065	S1229,T/S/Y	RFPTDYASTSEDEFGSNR	S8,T/S/Y	1.014	0.891
Cep170	Centrosomal protein of 170 kDa	Q6A065	Ambiguous	QKSSPVNNHSSPSQTPALCPPETR	S/T	1.195	0.004
Cep170	Centrosomal protein of 170 kDa	Q6A065	Ambiguous	SSPVNNHSSPSQTPALCPPETR	S/T	1.161	0.661
Cep170	Centrosomal protein of 170 kDa	Q6A065	Ambiguous	SHGRTPLTSADEHNIHSK	T/S	1.268	0.175
Cep170b	Centrosomal protein of 170 kDa protein B	Q80U49	S1122	LGDASDTEAVDGER	S5	1.062	0.313
Cep170b	Centrosomal protein of 170 kDa protein B	Q80U49	S1166	AGSFTGPSDSETAPAR	S3	0.992	0.750
Cep170b	Centrosomal protein of 170 kDa protein B	Q80U49	S1349	SPSLGNVPNTPASTISAR	S3	0.803	0.311
Cep170b	Centrosomal protein of 170 kDa protein B	Q80U49	S490	SFGSVGRR	S4	1.020	0.613
Cep170b	Centrosomal protein of 170 kDa protein B	Q80U49	S534	GASPVTPSTTPPPPTDPQLTK	S3	1.055	0.619
Cep170b	Centrosomal protein of 170 kDa protein B	Q80U49	S843	SGRSPEPDPAPPK	S4	0.991	0.803
Cep170b	Centrosomal protein of 170 kDa protein B	Q80U49	S862	QESFTKEPTSGPPAPGK	S3	1.163	0.039
Cep170b	Centrosomal protein of 170 kDa protein B	Q80U49	S981	ESPLSPPTVPDPGGATPGSAR	S5	1.091	0.611
Cep170b	Centrosomal protein of 170 kDa protein B	Q80U49	T1345	EIHDVAGDGDSLGSPGPTR	T18	0.980	0.839
Cep170b	Centrosomal protein of 170 kDa protein B	Q80U49	T529	TPPVLPAPLTPR	T10	1.043	0.572
Cep170b	Centrosomal protein of 170 kDa protein B	Q80U49	S1166,S1171	AGSFTGPSDSETAPAR	S3,S8	0.994	0.971
Cep170b	Centrosomal protein of 170 kDa protein B	Q80U49	S534,T537	GASPVTPSTTPPPPTDPQLTK	S3,T6	0.932	0.467
Cep170b	Centrosomal protein of 170 kDa protein B	Q80U49	Ambiguous	SNSLSTPRPTR	S/T	1.061	0.505
Cep170b	Centrosomal protein of 170 kDa protein B	Q80U49	Ambiguous	SQSFTHTPPADPK	S/T	1.009	0.650
Cep170b	Centrosomal protein of 170 kDa protein B	Q80U49	Ambiguous	LGNTSPVPR	T/S	0.942	0.060
Cep192	Centrosomal protein 192	E9Q4Y4	S1795	GQDQDCFQIQNTFGSEER	S15	0.916	0.570
Cep192	Centrosomal protein 192	E9Q4Y4	Ambiguous	TNGDNGINSPDTLWSPTSERR	S/T	1.116	0.178
Cep250	Centrosome-associated protein CEP250	A3KGJ7	S1308	QLSQSQEEK	S3	1.095	0.526
Cep250	Centrosome-associated protein CEP250	A3KGJ7	S2240	VGQGSQSGEVETEPSPGVEEK	S5	1.253	0.062
Cep250	Centrosome-associated protein CEP250	A3KGJ7	S2240	VGQGSQSGEVETEPSPGVEEKER	S5	0.961	0.551
Cep250	Centrosome-associated protein CEP250	A3KGJ7	S2322	ETMTSSPTQQDGR	S5	0.964	0.610
Cep350	Centrosome-associated protein 350	E9Q309	S1259	TPTSPLSPSSQK	S7	1.047	0.766
Cep350	Centrosome-associated protein 350	E9Q309	S1606	FDESMTEDEIEEK	S4	1.049	0.628
Cep350	Centrosome-associated protein 350	E9Q309	S1641	GHHDDSDEDASPDKTALSSTK	S6	0.992	0.619
Cep350	Centrosome-associated protein 350	E9Q309	S1930	GIASEEGSPMPSYSPR	S14	0.905	0.143
Cep350	Centrosome-associated protein 350	E9Q309	S2221	DSLESAPSLSPVK	S10	1.080	0.494
Cep350	Centrosome-associated protein 350	E9Q309	S2421	ATGSGSDEEISECLGEK	S6	1.063	0.192
Cep41	Centrosomal protein of 41 kDa	Q99NF3	Ambiguous	GGQNLPTSCPASHSSPR	S/T	1.011	0.939
Cep41	Centrosomal protein of 41 kDa	Q99NF3	Ambiguous	LEDNDSATSEADAEIAAK	T/S	0.993	0.951
Cep57	Centrosomal protein of 57 kDa	Q8CEE0	S53	SPNKPTFAYPESNSR	S1	0.971	0.828
Cep63	Centrosomal protein of 63 kDa	F8VPJ7	S362	AVGTQQSVERPLEDCQK	S7	1.030	0.592
Cep78	Centrosomal protein of 78 kDa	Q6IRU7	S658	TGSPLAGGIPGGR	S3	0.953	0.727
Cep83	Centrosomal protein of 83 kDa	Q9D5R3	S689	QLEELGSPGE	S7	1.073	0.520
Cep83	Centrosomal protein of 83 kDa	Q9D5R3	S689	KQLEELGSPGE	S8	0.944	0.278
Cep85	Centrosomal protein of 85 kDa	Q8BMK0	S16	SHATSPGSNVIQK	S5	0.962	0.709
Cep97	Centrosomal protein of 97 kDa	Q9CZ62	S308	QLMSQSQDEELSPLAAVETR	S6	1.039	0.853
Cerk	Ceramide kinase	Q8K4Q7	S498	SSWNCDGEVMHSPAIEVR	S2	1.007	0.888
Cetn2	Centrin-2	Q9R1K9	S20	MSPKPELTEDQKQEIR	S2	0.897	0.057
Cetn2	Centrin-2	Q9R1K9	S20	RMSPKPELTEDQKQEIR	S3	1.101	0.496
Cfap97	Cilia- and flagella-associated protein 97	E9Q1K1	S230	CVSSVTPSSPK	S9	0.993	0.886
Cfl1	Cofilin-1	P18760	S23	SSTPEEVK	S1	0.993	0.880
Cfl1	Cofilin-1	P18760	T25	KSSTPEEVK	T4	1.069	0.093
Cfl1	Cofilin-1	P18760	Y85	YALYDATYETK	Y4	0.886	0.010
Cfl1	Cofilin-1	P18760	Ambiguous	YALYDATYETKESK	T/Y/S	0.910	0.065
Cggbp1	CGG triplet repeat-binding protein 1	Q8BHG9	S164	AYLPDGYENENQLLSSQDC	S16	1.010	0.939
Cgnl1	Cingulin-like protein 1	Q6AW69	S284	QDSAGPILDGAR	S3	1.088	0.200
Cgnl1	Cingulin-like protein 1	Q6AW69	S284	RQDSAGPILDGAR	S4	1.065	0.313
Cgnl1	Cingulin-like protein 1	Q6AW69	S199,S203	SYGSQPNSPTSEDLAK	S4,S8	0.958	0.310
Cgnl1	Cingulin-like protein 1	Q6AW69	Ambiguous	SYGSQPNSPTSEDLAK	S/T/Y	0.877	0.199
Cgnl1	Cingulin-like protein 1	Q6AW69	Ambiguous	RSSSSSTTPTSATSLYK	S/T/Y	0.963	0.524
Cgnl1	Cingulin-like protein 1	Q6AW69	Ambiguous	AAGSAQGSNQAPNSPSEGNSLLDQK	S	1.049	0.277
Cgnl1	Cingulin-like protein 1	Q6AW69	Ambiguous	SSSSSTTPTSATSLYK	T/S/Y	0.893	0.365
Cgnl1	Cingulin-like protein 1	Q6AW69	Ambiguous	SSSSSTTPTSATSLYK	T/S/Y	0.947	0.624
Chaf1a	Chromatin assembly factor 1 subunit A	Q9QWF0	S173	QQEHSAAELCLLETPSDITCHMEEEPGSPGDPKR	S28	1.021	0.796
Chaf1a	Chromatin assembly factor 1 subunit A	Q9QWF0	S51	EKVEEDTSPK	S8	0.997	0.970
Chaf1a	Chromatin assembly factor 1 subunit A	Q9QWF0	S776	LISENSAYEK	S3	1.125	0.173
Chaf1b	Chromatin assembly factor 1 subunit B	Q9D0N7	Ambiguous	TQDPSSPCTTPSPTTQSPAPSAIK	S/T	0.959	0.624
Champ1	Chromosome alignment-maintaining phosphoprotein 1	A0A140T8S5	S405	AVPPVSPELR	S6	1.019	0.665
Champ1	Chromosome alignment-maintaining phosphoprotein 1	A0A140T8S5	S416	RPGPPLSPEIR	S7	1.004	0.927

Champ1	Chromosome alignment-maintaining phosphoprotein 1	A0A140T8S5	S532	AVLPASPEPR	S6	1.063	0.098
Champ1	Chromosome alignment-maintaining phosphoprotein 1	A0A140T8S5	S603	LLDEALSPSSK	S7	0.818	0.253
Champ1	Chromosome alignment-maintaining phosphoprotein 1	A0A140T8S5	S617	DSQENSDAELSSSEYIR	S6	1.010	0.920
Champ1	Chromosome alignment-maintaining phosphoprotein 1	A0A140T8S5	S617	KDSQENSDAELSSSEYIR	S7	0.964	0.543
Champ1	Chromosome alignment-maintaining phosphoprotein 1	A0A140T8S5	S617,S/Y	KDSQENSDAELSSSEYIR	S7,S/Y	1.032	0.540
Champ1	Chromosome alignment-maintaining phosphoprotein 1	A0A140T8S5	S617,S622	DSQENSDAELSSSEYIR	S6,S11	0.888	0.468
Champ1	Chromosome alignment-maintaining phosphoprotein 1	A0A140T8S5	S416,S421,S425	RPGPPLSPEIRSPAGSPELK	S7,S12,S16	0.987	0.926
Chchd3	MICOS complex subunit Mic19	Q9CRB9	S113	ISSEEER	S3	1.056	0.721
Chchd3	MICOS complex subunit Mic19	Q9CRB9	S51	YSSVYGASVSDEDLK	S3	1.067	0.542
Chd1	Chromodomain-helicase-DNA-binding protein 1	P40201	S1678	AASSGPRSPLDQR	S8	1.048	0.263
Chd1	Chromodomain-helicase-DNA-binding protein 1	P40201	S90	SSPSILAVQR	S2	0.919	0.384
Chd2	Chromodomain-helicase-DNA-binding protein 2	E9PZM4	S1365	LKDEHGLEPASPR	S11	1.029	0.532
Chd3	Chromodomain helicase DNA-binding protein 3	B1AR17	S1651	MEAEVDSPSPAPSLGER	S7	1.088	0.515
Chd3	Chromodomain helicase DNA-binding protein 3	B1AR17	S1651	MEAEVDSPSPAPSLGER	S7	0.921	0.100
Chd3	Chromodomain helicase DNA-binding protein 3	B1AR17	S649	KNDMDEPPPLDYGSGEDDGK	S14	0.877	0.204
Chd3	Chromodomain helicase DNA-binding protein 3	B1AR17	S764	ELQGDGPPSSPTNDPTVK	S9	1.055	0.268
Chd3	Chromodomain helicase DNA-binding protein 3	B1AR17	S1653,S1657	MEAEVDSPSPAPSLGER	S9,S13	1.025	0.775
Chd4	Chromodomain-helicase-DNA-binding protein 4	E9QAS5	S1535	MSQPGSPSPK	S6	1.040	0.351
Chd4	Chromodomain-helicase-DNA-binding protein 4	E9QAS5	S1535	MSQPGSPSPK	S6	1.051	0.065
Chd4	Chromodomain-helicase-DNA-binding protein 4	E9QAS5	T1545	TPTPSTPGDTQPNTPAPVPPAEDGIK	T6	1.044	0.421
Chd4	Chromodomain-helicase-DNA-binding protein 4	E9QAS5	S1531,S1535	KMSQPGSPSPK	S3,S7	0.944	0.590
Chd4	Chromodomain-helicase-DNA-binding protein 4	E9QAS5	S1531,S1535	KMSQPGSPSPK	S3,S7	1.042	0.276
Chd4	Chromodomain-helicase-DNA-binding protein 4	E9QAS5	T1549,T1553	TPTPSTPGDTQPNTPAPVPPAEDGIK	T10,T14	1.015	0.849
Chd6	Chromodomain-helicase-DNA-binding protein 6	A3KFM7	S1357	QTASPSDGSDGIFGEK	S4	1.053	0.458
Chd6	Chromodomain-helicase-DNA-binding protein 6	A3KFM7	S2706	EEGLKDSNEDTN	S7	1.038	0.691
Chd7	Chromodomain-helicase-DNA-binding protein 7	A2AJK6	S2548	NIPSPGQLDPDTR	S4	0.970	0.828
Chd8	Chromodomain-helicase-DNA-binding protein 8	Q09XV5	S1997	TASPSPLRPDAPVEK	S3	1.096	0.369
Chd8	Chromodomain-helicase-DNA-binding protein 8	Q09XV5	S2040	SRLTSQDYEVR	S5	1.250	0.037
Chd8	Chromodomain-helicase-DNA-binding protein 8	Q09XV5	S2520	TTGYPSSPATTTSGTALR	S7	1.112	0.240
Chd8	Chromodomain-helicase-DNA-binding protein 8	Q09XV5	S555	NTSSDNSDVEVMPAQSPREDEESSIQK	S7	0.921	0.046
Chd8	Chromodomain-helicase-DNA-binding protein 8	Q09XV5	S555	NTSSDNSDVEVMPAQSPR	S7	1.055	0.736
Chd8	Chromodomain-helicase-DNA-binding protein 8	Q09XV5	Ambiguous	NTSSDNSDVEVMPAQSPREDEESSIQK	S/T	1.033	0.623
Chd9	Chromodomain-helicase-DNA-binding protein 9	Q8BYH8	S2302	LLDSPGAATER	S4	0.983	0.941
Chd9	Chromodomain-helicase-DNA-binding protein 9	Q8BYH8	S549	VMSPENFPSASVEGK	S3	0.858	0.312
Chd9	Chromodomain-helicase-DNA-binding protein 9	Q8BYH8	S653	YAEDAEGKQSEEEVK	S10	1.146	0.159
Chek1	Serine/threonine-protein kinase Chk1	O35280	Ambiguous	ATSGGMSESSSGFSK	T/S	1.030	0.597
Chm	Rab proteins geranylgeranyltransferase component A	A2AD03	S165	SCELPAEQSQCTGPESSPQVNDAEVGEKETQSDAK	S1	1.024	0.687
Chm	Rab proteins geranylgeranyltransferase component A	A2AD03	S165	SCELPAEQSQCTGPESSPQVNDAEVGEK	S1	1.115	0.281
Chm	Rab proteins geranylgeranyltransferase component A	A2AD03	S661	ESTVLGDSEEPSE	S12	1.044	0.295
Chm	Rab proteins geranylgeranyltransferase component A	A2AD03	S657,S661	ESTVLGDSEEPSE	S8,S12	1.034	0.901
Chm	Rab proteins geranylgeranyltransferase component A	A2AD03	Ambiguous	SSTEQSSEILPK	S/T	1.020	0.782
Chmp2a	Charged multivesicular body protein 2a	Q9DB34	Ambiguous	AEATASALADADADLEER	T/S	1.028	0.852
Chmp2b	Charged multivesicular body protein 2b	Q8BJF9	S187	SLPSASTSK	S1	0.961	0.484
Chmp2b	Charged multivesicular body protein 2b	Q8BJF9	S199	ATISDEEIER	S4	0.978	0.632
Chmp3	Charged multivesicular body protein 3	Q9CQ10	S200	VTDALPEPEPAGAMAASEEGEEEEDEEDLEAMQSR	S17	0.881	0.188
Chmp3	Charged multivesicular body protein 3	Q9CQ10	S200	VTDALPEPEPAGAMAASEEGEEEEDEEDLEAMQSR	S17	0.850	0.458
Chmp3	Charged multivesicular body protein 3	Q9CQ10	S200	VTDALPEPEPAGAMAASEEGEEEEDEEDLEAMQSR	S17	0.986	0.753
Chmp5	Charged multivesicular body protein 5	Q9D7S9	S16	APPPSLTDCIGTVDSR	S5	0.923	0.472
Chmp6	Charged multivesicular body protein 6	P0C0A3	T130	ILDETQEAVEYQR	T5	0.828	0.330
Chrac1	Chromatin accessibility complex protein 1	Q9JKP8	S122	EEEEDNEDDGSDLGEALA	S11	0.975	0.758
Chrac1	Chromatin accessibility complex protein 1	Q9JKP8	S122	REEEEDNEDDGSDLGEALA	S12	0.956	0.178
Chrac1	Chromatin accessibility complex protein 1	Q9JKP8	S122	EKREEEEDNEDDGSDLGEALA	S14	1.030	0.295
Chtf18	3, chromosome transmission fidelity factor 18 homolog (S. cerevisiae), isoform C	A0A0R4J0I4	S91	DQPAPSSPMVK	S7	1.094	0.160
Chtf18	3, chromosome transmission fidelity factor 18 homolog (S. cerevisiae), isoform C	A0A0R4J0I4	Ambiguous	RDQPAPSSPMVK	S	1.154	0.046
Chtf8	Chromosome transmission fidelity protein 8 homolog isoform 2	P0CG14	S160	AGGLLGASPDPR	S8	0.989	0.887
Chtop	Chromatin target of PRMT1 protein	Q9CY57	Ambiguous	GHLDAELDAYMAQTDPETND	T/Y	1.119	0.052
Cic	Protein capicua homolog	Q924A2	S1080	TQSLSALPK	S3	1.140	0.025
Cic	Protein capicua homolog	Q924A2	S2311	NSTDLDSAPEDPTSPK	S14	0.959	0.545
Cic	Protein capicua homolog	Q924A2	S2311	KNSTDLDSAPEDPTSPK	S15	0.782	0.021
Cic	Protein capicua homolog	Q924A2	S2311	NSTDLDSAPEDPTSPKR	S14	0.783	0.006
Cic	Protein capicua homolog	Q924A2	S2321	SSCSSEPNTPK	S2	1.087	0.500
Cic	Protein capicua homolog	Q924A2	S454	SSSVASLEK	S3	1.129	0.343
Cic	Protein capicua homolog	Q924A2	S553	EGSTEFDWGDETSR	S3	1.222	0.179
Cic	Protein capicua homolog	Q924A2	S662	HLSASTPK	S3	1.104	0.063
Cic	Protein capicua homolog	Q924A2	S766	AVSPAVPFSR	S3	1.001	0.993
Cic	Protein capicua homolog	Q924A2	T470	ARTPLTAAQQK	T3	1.048	0.284
Cic	Protein capicua homolog	Q924A2	T753	SDSLDSGVDSVSHTPTPSTPAGFR	T14	0.934	0.692
Cic	Protein capicua homolog	Q924A2	S2299,S2311	RKNSTDLDSAPEDPTSPK	S4,S16	1.084	0.426
Cic	Protein capicua homolog	Q924A2	S2299,S2311	KNSTDLDSAPEDPTSPKR	S3,S15	1.099	0.496
Cic	Protein capicua homolog	Q924A2	S2299,S2311	KNSTDLDSAPEDPTSPK	S3,S15	1.085	0.145
Cic	Protein capicua homolog	Q924A2	S766,S	AVSPAVPFSRSR	S3,S	0.908	0.459
Cic	Protein capicua homolog	Q924A2	T2192,S2195	GEPPTPPSPAPATGPSGSSSGSSEGSSGR	T5,S8	0.857	0.079
Cic	Protein capicua homolog	Q924A2	S2304,S2311,S/T	KNSTDLDSAPEDPTSPK	S8,S15,S/T	0.842	0.365
Cic	Protein capicua homolog	Q924A2	Ambiguous	ACPGLAGSASGSKSPPATR	S/T	0.945	0.242
Cic	Protein capicua homolog	Q924A2	Ambiguous	SSPPPPLPAEERPGTK	S/T	1.053	0.268
Cic	Protein capicua homolog	Q924A2	Ambiguous	VTDSESGDSSGEDPEGNKGFGR	S/T	1.082	0.611
Cic	Protein capicua homolog	Q924A2	Ambiguous	GEPPTPPSPAPATGPSGSSSGSSEGSSGR	T/S	1.002	0.945
Cip2a	Protein CIP2A	D3Z7B5	T586	CLTPPPSK	T3	1.071	0.121
Cit	Citron Rho-interacting kinase	D3YU89	S2036	ERSPGRLFEDSSR	S3	0.970	0.725
Ckap4	Cytoskeleton-associated protein 4	Q8BMK4	S211	ERDFTSLENTVEER	S6	1.007	0.893
Ckap4	Cytoskeleton-associated protein 4	Q8BMK4	S367	VGAHGSEEGAVFK	S6	1.062	0.432
Ckap4	Cytoskeleton-associated protein 4	Q8BMK4	T210	DFTSLENTVEER	T3	0.969	0.510
Ckap5	Cytoskeleton-associated protein 5	A2AGT5	Ambiguous	HSTSATDEGEDGEEPGEGGNDVVDLLPR	S/T	0.919	0.699
Clasp1	CLIP-associating protein 1	A0A087WNW8	S1067	NSSNTGVGSPSNTIGR	S9	1.026	0.658
Clasp1	CLIP-associating protein 1	A0A087WNW8	S1133	SQEDLNEPIKR	S1	0.954	0.301
Clasp1	CLIP-associating protein 1	A0A087WNW8	S545	NSDSIVSLPQSDR	S4	1.043	0.517
Clasp1	CLIP-associating protein 1	A0A087WNW8	S559	SSSSSQESLNRPLSAK	S5	0.993	0.864
Clasp1	CLIP-associating protein 1	A0A087WNW8	S594	SVSTTGSLQR	S7	0.871	0.248
Clasp1	CLIP-associating protein 1	A0A087WNW8	S600	SRSDIDVNAAASAK	S3	1.006	0.876
Clasp1	CLIP-associating protein 1	A0A087WNW8	S662	RQSSGSTTNVASTPSDSR	S3	1.116	0.087
Clasp1	CLIP-associating protein 1	A0A087WNW8	S665	QSSGSTTNVASTPSDSR	S5	0.961	0.812
Clasp1	CLIP-associating protein 1	A0A087WNW8	S688	VVSQSQPGSR	S5	1.040	0.355
Clasp1	CLIP-associating protein 1	A0A087WNW8	T716	GPPVTPSSEK	T5	1.020	0.802
Clasp1	CLIP-associating protein 1	A0A087WNW8	S562,S	SSSSSQESLNRPLSAK	S8,S	1.044	0.530
Clasp1	CLIP-associating protein 1	A0A087WNW8	S662,S665	RQSSGSTTNVASTPSDSR	S3,S6	1.247	0.212
Clasp1	CLIP-associating protein 1	A0A087WNW8	S688,S692	VVSQSQPGSR	S5,S9	1.035	0.396
Clasp1	CLIP-associating protein 1	A0A087WNW8	S686,S	VVSQSQPGSRSSSPGK	S3,S	1.023	0.814
Clasp2	CLIP-associating protein 2	F7DCH5	S319	SSGSVASLPQSDR	S4	0.927	0.323
Clasp2	CLIP-associating protein 2	F7DCH5	S333	SSSSSQESLNRPFSSK	S5	1.007	0.781
Clasp2	CLIP-associating protein 2	F7DCH5	S376	SDIDVNAAAGAK	S1	1.097	0.241
Clasp2	CLIP-associating protein 2	F7DCH5	S376	SRSDIDVNAAAGAK	S3	0.992	0.637
Clasp2	CLIP-associating protein 2	F7DCH5	S461	MVSQSQPGSR	S5	1.065	0.664
Clasp2	CLIP-associating protein 2	F7DCH5	S461	MVSQSQPGSR	S5	1.037	0.666
Clasp2	CLIP-associating protein 2	F7DCH5	S513	EASPSRLSVAR	S3	0.921	0.569
Clasp2	CLIP-associating protein 2	F7DCH5	Y1035	DYNPYNYSDSISPFNK	Y5	1.127	0.599
Clasp2	CLIP-associating protein 2	F7DCH5	S336,S	SSSSSQESLNRPFSSK	S8,S	0.905	0.259
Clasp2	CLIP-associating protein 2	F7DCH5	S461,S465	MVSQSQPGSR	S5,S9	1.065	0.324
Clasp2	CLIP-associating protein 2	F7DCH5	Ambiguous	MVSQSQPGSRSGSPGR	S	1.012	0.909
Clasrp	CLK4-associating serine/arginine rich protein	Q8CFC7	S294	RDSPTYDPYKR	S3	0.943	0.483
Clasrp	CLK4-associating serine/arginine rich protein	Q8CFC7	S541	LTRPAASPAVGEK	S7	1.015	0.719
Clcc1	Chloride channel CLIC-like 1, isoform CRA_a	A2AEM2	S498	DVSGQVPSAGK	S8	1.011	0.912
Clcc1	Chloride channel CLIC-like 1, isoform CRA_a	A2AEM2	S518	TDSECSPPGGCPPSK	S6	1.135	0.231
Clcc1	Chloride channel CLIC-like 1, isoform CRA_a	A2AEM2	S63	EVSPDSSEAEELSDCLHR	S6	1.023	0.267
Clcc1	Chloride channel CLIC-like 1, isoform CRA_a	A2AEM2	S63	KEVSPDSSEAEELSDCLHR	S7	0.989	0.800
Clcc1	Chloride channel CLIC-like 1, isoform CRA_a	A2AEM2	Ambiguous	DVSGQVPSAGKSSPTVDK	S/T	1.157	0.108
Clcn5	Chloride channel protein	B1ATV0	Ambiguous	DDVPPLDREIEGNKSYNGGGIGSSNR	S/Y	1.143	0.340
Clcn7	H(+)/Cl(-) exchange transporter 7	O70496	T803	GGLEELSLAQT	T11	1.003	0.974
Clec16a	Protein CLEC16A	Q80U30	S406	NVGEEEDEERGSAEDAQEDAEK	S12	1.081	0.410
Clec4a2	C-type lectin domain family 4, member a2	Q923C7	S25	FKNESNSLHTYSESPAAPR	S14	1.021	0.916
Clec4a2	C-type lectin domain family 4, member a2	Q923C7	Ambiguous	NESNSLHTYSESPAAPR	S/T/Y	1.162	0.079
Clip1	CAP-Gly domain-containing linker protein 1	F8WIA1	S194	TASESISNLSEAGSVK	S5	0.974	0.383
Clip1	CAP-Gly domain-containing linker protein 1	F8WIA1	S194,S203	TASESISNLSEAGSVK	S5,S14	0.960	0.366
Clip1	CAP-Gly domain-containing linker protein 1	F8WIA1	S194,S199,S203	TASESISNLSEAGSVKKGER	S5,S10,S14	0.857	0.239
Clip1	CAP-Gly domain-containing linker protein 1	F8WIA1	Ambiguous	ASGPPSSETQEEFVDDFR	S/T	1.071	0.456
Clip2	CAP-Gly domain-containing linker protein 2	Q9Z0H8	S208	TGNESGSNLSDSGSVK	S10	1.023	0.688
Clip2	CAP-Gly domain-containing linker protein 2	Q9Z0H8	S924	VLLLEANRHSPGPER	S10	0.999	0.993
Clip2	CAP-Gly domain-containing linker protein 2	Q9Z0H8	S208,S212	TGNESGSNLSDSGSVKR	S10,S14	1.066	0.469
Clip2	CAP-Gly domain-containing linker protein 2	Q9Z0H8	S208,S212	TGNESGSNLSDSGSVK	S10,S14	1.061	0.142
Clip2	CAP-Gly domain-containing linker protein 2	Q9Z0H8	S208,S/T	TGNESGSNLSDSGSVKRGDK	S10,S/T	1.064	0.345
Clk1	Dual specificity protein kinase CLK1	P22518	S340	VVDFGSATYDDEHHSTLVSTR	S19	1.016	0.897
Clk3	Dual specificity protein kinase CLK3	O35492	S157	YRSPEPDPYLSYR	S3	0.999	0.973
Clk3	Dual specificity protein kinase CLK3	O35492	S157	RYRSPEPDPYLSYR	S4	0.939	0.196
Clk3	Dual specificity protein kinase CLK3	O35492	S283	SSRSVEDDKEGHLVCR	S4	0.875	0.179
Cln3	Battenin	Q61124	S14	LEDSEREETDSEPQAPR	S4	1.083	0.111
Cln3	Battenin	Q61124	S14	RLEDSEREETDSEPQAPR	S5	1.117	0.005
Clns1a	Chloride channel, nucleotide-sensitive, 1A	Q923F1	S100	LGEESKEPLSDEDEEDNDDVEPISEFR	S10	0.996	0.713
Clns1a	Chloride channel, nucleotide-sensitive, 1A	Q923F1	S100	EPLSDEDEEDNDDVEPISEFR	S4	0.968	0.651
Clock	Circadian locomoter output cycles protein kaput	O08785	S406	ELGIEESLPETAADKSQDSGSDNR	S19	1.055	0.426

Clock	Circadian locomoter output cycles protein kaput	O08785	S845	HRTDSLTDPSKVQPQ	S5	1.132	0.091
Cptm1	Cleft lip and palate transmembrane protein 1 homolog	Q8VBZ3	Ambiguous	AQASTAAGALTPAPSTAVSGEDASTVPK	T/S	1.113	0.667
Clptm1l	Cleft lip and palate transmembrane protein 1-like protein	Q8BXA5	S527	VNEFGESYEEQPK	S7	1.052	0.547
Clpx	ATP-dependent Clp protease ATP-binding subunit clpX-like, mitochondrial	Q9JHS4	Ambiguous	APSKESSEEEYDSGVEEDGWPR	S/Y	1.258	0.122
Clspn	Claspin	Q80YR7	S823	LSEPSLPVEDSQDLYTASPEPK	S18	1.227	0.292
Clta	Clathrin light chain	Q6PFA2	S105	LQSEPESIR	S3	1.095	0.021
Clta	Clathrin light chain	Q6PFA2	S105	LQSEPESIRK	S3	1.046	0.436
Cltc	Clathrin heavy chain	Q5SXR6	S67	RPISADSAIMNPASK	S4	0.990	0.893
Cltc	Clathrin heavy chain	Q5SXR6	T398	GILRTPDTIR	T5	1.062	0.108
Cltc	Clathrin heavy chain	Q5SXR6	T398	GILRTPDTIRR	T5	1.057	0.539
Cluap1	Clusterin-associated protein 1	Q8R3P7	S409	IRKPEPLDESDNDF	S10	0.983	0.769
Cluap1	Clusterin-associated protein 1	Q8R3P7	S409	KPEPLDESDNDF	S8	1.189	0.331
Cluh	Clustered mitochondria protein homolog	A0A0R4J140	S1338	DKAEEQPMAPEPEPEPERAVEDMGSPQTAK	S25	1.194	0.146
Cluh	Clustered mitochondria protein homolog	A0A0R4J140	S1338	DKAEEQPMAPEPEPEPERAVEDMGSPQTAK	S25	1.011	0.883
Cluh	Clustered mitochondria protein homolog	A0A0R4J140	S1338	AVEDMGSPQTAK	S7	1.012	0.825
Cluh	Clustered mitochondria protein homolog	A0A0R4J140	S1348	EGPSSLNLQG	S5	1.170	0.408
Cluh	Clustered mitochondria protein homolog	A0A0R4J140	S696	SEDSIGPEAGCEEEGSSVSGLAK	S4	1.215	0.197
Cmc2	COX assembly mitochondrial protein 2 homolog	Q8K199	S71	RLSDPPEEAGR	S3	1.030	0.502
Cmip	C-Maf-inducing protein	Q9D486	S349	DNSPSLK	S3	1.016	0.819
Cmklr1	Chemokine-like receptor 1	P97468	T370	ASVNEKETSTL	T10	0.977	0.669
Cmtm4	CKLF-like MARVEL transmembrane domain-containing protein 4	Q8CJ61	S194	TESRDVDSRPEIQRLDT	S3	1.164	0.170
Cmtm6	CKLF-like MARVEL transmembrane domain-containing protein 6	Q9CZ69	T178	VEALTEPLNA	T5	0.844	0.405
Cmtm7	CKLF-like MARVEL transmembrane domain-containing protein 7	Q9ESD6	S14	TTCSSGGALGPGQPSEGLLDR	S5	1.021	0.533
Cmtr1	Cap-specific mRNA (nucleoside-2'-O-)-methyltransferase 1	Q9DBC3	S27	HLSSTSDDEPLSSVNHAAK	S3	1.114	0.178
Cmtr1	Cap-specific mRNA (nucleoside-2'-O-)-methyltransferase 1	Q9DBC3	S27,S28	HLSSTSDDEPLSSVNHAAK	S3,S4	1.109	0.160
Cnksr3	Connector enhancer of kinase suppressor of ras 3	Q8BMA3	S383	GSESPSNFSLDQESQR	S4	1.087	0.638
Cnksr3	Connector enhancer of kinase suppressor of ras 3	Q8BMA3	S383	KGSESPSNFSLDQESQR	S5	1.179	0.336
Cnnm3	Metal transporter CNNM3	Q32NY4	S663	AQSLPPSPENAELQAIPGSQTR	S3	0.960	0.739
Cnnm3	Metal transporter CNNM3	Q32NY4	S700	ETAGSTNSRPSIPVEESPGR	S11	0.828	0.130
Cnnm3	Metal transporter CNNM3	Q32NY4	S706	ETAGSTNSRPSIPVEESPGRNPGV	S17	0.995	0.855
Cnnm3	Metal transporter CNNM3	Q32NY4	Ambiguous	SEILDESEDYSDTK	S/Y/T	1.079	0.378
Cnnm4	Metal transporter CNNM4	Q69ZF7	S657	SPALPTPLSR	S9	0.809	0.308
Cnnm4	Metal transporter CNNM4	Q69ZF7	S661	SASLSYPDR	S3	1.118	0.060
Cnot2	CCR4-NOT transcription complex subunit 2	Q8C5L3	S165	TNSMSSSGLGSPNR	S11	0.878	0.123
Cnot2	CCR4-NOT transcription complex subunit 2	Q8C5L3	S165	TNSMSSSGLGSPNR	S11	0.969	0.607
Cnot2	CCR4-NOT transcription complex subunit 2	Q8C5L3	T246	EGSGNPTPLINPLAGR	T7	0.972	0.884
Cnot3	CCR4-NOT transcription complex subunit 3	Q8K0V4	S299	STDSEVSQSPAK	S9	1.025	0.391
Cnpy2	Protein canopy homolog 2	Q9QXT0	S115	NGESSELDLQGIR	S4	1.059	0.463
Cnpy2	Protein canopy homolog 2	Q9QXT0	S63	INPDGSQSVVEVPYAR	S6	1.068	0.602
Cnpy4	Protein canopy homolog 4	Q8BQ47	Ambiguous	ISDGQEEADDEEEEEEEEITKTSGNPK	T/S	1.008	0.878
Cnst	Consortin	Q8CBC4	S34	LPSVSDENENQLAGDGPAGLTTSEGAMGR	S3	0.965	0.797
Cnst	Consortin	Q8CBC4	S34	LPSVSDENENQLAGDGPAGLTTSEGAMGR	S3	1.350	0.392
Coa6	Cytochrome c oxidase assembly factor 6 homolog	Q8BGD8	S79	FEAGGFQSSQSTENS	S15	0.914	0.389
Cobll1	Cordon-bleu protein-like 1	Q3UMF0	S300	EQTASAPATPLVSK	S5	0.947	0.678
Cobll1	Cordon-bleu protein-like 1	Q3UMF0	S300,T304	EQTASAPATPLVSK	S5,T9	1.171	0.210
Cobll1	Cordon-bleu protein-like 1	Q3UMF0	Ambiguous	QSSLTFQSSDPEHVR	S/T	1.077	0.368
Cog1	Conserved oligomeric Golgi complex subunit 1	Q9Z160	S928	SVETQAQVGPPALSR	S1	0.911	0.455
Cog2	Conserved oligomeric Golgi complex subunit 2	Q921L5	S665	RSPATNPVSSSGGGMSDDDKIR	S2	1.072	0.511
Cog2	Conserved oligomeric Golgi complex subunit 2	Q921L5	Ambiguous	DVSSQEASSPLLTGQVLER	S/T	0.776	0.130
Coil	Coilin	E9Q284	S149	QEDSGQNEKASDLETK	S11	0.999	0.976
Coil	Coilin	E9Q284	S149	RQEDSGQNEKASDLETK	S12	1.066	0.192
Coil	Coilin	E9Q284	S174	VTGSPAEEDEEETKK	S4	0.931	0.261
Coil	Coilin	E9Q284	S174	VTGSPAEEDEEETK	S4	1.078	0.270
Coil	Coilin	E9Q284	S174	SKVTGSPAEEDEEETK	S6	0.960	0.616
Coil	Coilin	E9Q284	S225	EGASQNCSFPRASPR	S13	0.952	0.484
Coil	Coilin	E9Q284	T125	LMEDEETDQGYK	T7	0.994	0.945
Coil	Coilin	E9Q284	T125	LMEDEETDQGYK	T7	1.208	0.198
Col17a1	Collagen alpha-1(XVII) chain	Q07563	T565,S575	LMTEQENGNLRGSPGPK	T3,S13	1.026	0.753
Col3a1	Collagen alpha-1(III) chain	P08121	S735	GGPGSPGPK	S5	0.943	0.705
Col4a3bp	Collagen type IV alpha-3-binding protein	Q9EQG9	S377	SSSMSSIDLVSASDDVHR	S3	0.961	0.535
Col4a3bp	Collagen type IV alpha-3-binding protein	Q9EQG9	Ambiguous	SSSMSSIDLVSASDDVHR	S	1.050	0.226
Col4a3bp	Collagen type IV alpha-3-binding protein	Q9EQG9	Ambiguous	SSSMSSIDLVSASDDVHR	S	1.078	0.663
Colgalt1	Procollagen galactosyltransferase 1	Q8K297	S611	NSDVLQSPLDSTARDEL	S11	0.887	0.259
Comt	Catechol O-methyltransferase	O88587	S261	AVYQGPGSSPVK	S9	1.058	0.026
Comt	Catechol O-methyltransferase	O88587	S265	AVYQGPGSSPVKS	S13	1.002	0.974
Copa	Coatomer subunit alpha	F8WHL2	S1202	CPLSGACYSPEFK	S9	1.013	0.904
Copa	Coatomer subunit alpha	F8WHL2	S173	NLSPGAVESDVR	S3	1.041	0.391
Copa	Coatomer subunit alpha	F8WHL2	S173	KNLSPGAVESDVR	S4	0.998	0.954
Copg1	Coatomer subunit gamma-1	Q9QZE5	S642	SSPEPVALTESETEYVIR	S11	0.915	0.474
Coprs	Coordinator of PRMT5 and differentiation stimulator	Q9CQ13	Ambiguous	GPGERSSQEAPSAEAGFATADLSGR	S/T	1.183	0.012
Coprs	Coordinator of PRMT5 and differentiation stimulator	Q9CQ13	Ambiguous	SSQEAPSAEAGFATADLSGR	S/T	0.950	0.701
Cops3	COP9 signalosome complex subunit 3	O88543	S410	SMGSQEDDSGNKPSSYS	S4	0.939	0.284
Coq8b	Atypical kinase COQ8B, mitochondrial	Q6QLB8	S533	QTLPLPAAS	S9	0.856	0.542
Coq9	Ubiquinone biosynthesis protein COQ9, mitochondrial	Q8K1Z0	S81	YTDQSGEEEEDYESEEQLQHR	S5	0.947	0.410
Coro1a	Coronin-1A	O89053	S426	ATPEPSGTPSSDTVSR	S10	1.047	0.120
Coro1a	Coronin-1A	O89053	T418	ATPEPSGTPSSDTVSRLEEDVR	T2	1.079	0.010
Coro1a	Coronin-1A	O89053	T418	RATPEPSGTPSSDTVSRLEEDVR	T3	1.013	0.692
Coro1a	Coronin-1A	O89053	T418	RATPEPSGTPSSDTVSR	T3	1.009	0.730
Coro1a	Coronin-1A	O89053	T418	RRATPEPSGTPSSDTVSR	T4	1.012	0.849
Coro1b	Coronin-1B	Q9WUM3	S421	NVLSDSRPASYSR	S12	1.025	0.539
Coro1b	Coronin-1B	Q9WUM3	S470	EQGERISRLEEQLGR	S7	1.028	0.699
Coro1c	Coronin-1C	Q9WUM4	S187	NGSLICTASK	S3	0.922	0.354
Coro7	Coronin-7	Q9D2V7	S666	SSPLPLQEGPGPEGGR	S2	1.047	0.655
Coro7	Coronin-7	Q9D2V7	S876	RAPSSAQYLEEK	S4	1.019	0.657
Cotl1	Coactosin-like protein	Q9CQI6	S141	AGGANYDAQSE	S10	1.043	0.432
Cotl1	Coactosin-like protein	Q9CQI6	S141	KAGGANYDAQSE	S11	1.029	0.709
Cpeb2	Cytoplasmic polyadenylation element-binding protein 2	E9Q5X2	S608	STPSLTPK	S1	0.925	0.398
Cpeb3	Cytoplasmic polyadenylation element-binding protein 3	A0A0R4J102	S194	SPASPSQAPYAQR	S1	0.958	0.681
Cpeb4	Cytoplasmic polyadenylation element-binding protein 4	Q7TN98	S252	RSPASPHPPPFTHR	S2	0.995	0.935
Cpeb4	Cytoplasmic polyadenylation element-binding protein 4	Q7TN98	S255	SPASPHPPPFTHR	S4	1.015	0.759
Cpeb4	Cytoplasmic polyadenylation element-binding protein 4	Q7TN98	S97	QQLSPSPGQEAGILPETEK	S4	1.019	0.355
Cpeb4	Cytoplasmic polyadenylation element-binding protein 4	Q7TN98	S97,S99	QQLSPSPGQEAGILPETEK	S4,S6	1.029	0.662
Cpne1	Copine-1	Q8C166	T525	APPTPGKGPAQAPQA	T4	0.960	0.778
Cpne3	Copine-3	Q8BT60	S243	SSPVEYECINEK	S2	0.896	0.112
Cpne3	Copine-3	Q8BT60	Ambiguous	EASRSSPVEYECINEK	S/Y	1.001	0.974
Cpox	Oxygen-dependent coproporphyrinogen-III oxidase, mitochondrial	P36552	S101	SPSPGRREEDGDELAR	S3	0.831	0.025
Cpsf3	Cleavage and polyadenylation specificity factor subunit 3	Q9QXK7	S659	AVECEEGSEDDESLR	S8	0.991	0.942
Cpsf3	Cleavage and polyadenylation specificity factor subunit 3	Q9QXK7	T681	LYEALTPVH	T6	0.861	0.556
Cpsf4	Cleavage and polyadenylation specificity factor subunit 4	E0CXT7	S202	SSSLIQLTSQNSSPNQQR	S3	1.050	0.523
Cpsf6	Cleavage and polyadenylation-specificity factor subunit 6	H3BJW3	Ambiguous	EMDTARTPLSEAEFEEIMNR	T/S	0.833	0.432
Cpsf7	Cleavage and polyadenylation specificity factor subunit 7	Q8BTV2	S48	SSSTEPPPPVR	S3	0.984	0.603
Cpsf7	Cleavage and polyadenylation specificity factor subunit 7	Q8BTV2	T203	DSSDSADGRATPSENLVPSSAR	T11	0.929	0.012
Cr1l	Complement component receptor 1-like protein (Fragment)	A0A0A6YXR6	S158	TTSPARNSLTQEVS	S8	1.027	0.711
Cracr2a	EF-hand calcium-binding domain-containing protein 4B	A0A1D5RLZ8	S420	SQSEEEEDVFSAPR	S3	0.835	0.032
Cracr2b	EF-hand calcium-binding domain-containing protein 4A	Q8OZJ8	S104	FVGVESAPGGSPLR	S11	1.033	0.802
Cramp1	Protein cramped-like	Q6PG95	S1284	EVLFDGGGGGNPVGDLSQ	S17	1.054	0.863
Creb1	Cyclic AMP-responsive element-binding protein 1	Q01147	S340	DLYCHKSD	S7	0.977	0.626
Creb1	Cyclic AMP-responsive element-binding protein 1	Q01147	Ambiguous	TAPTSTIAPGVVMASSPALPTQPAEEAAR	S/T	1.054	0.745
Crebbp	CREB-binding protein	F8VPR5	S1077	VEAKEEEENSSNDTASQSTSPSQPR	S20	1.080	0.024
Crebbp	CREB-binding protein	F8VPR5	S1077,T/S	VEAKEEEENSSNDTASQSTSPSQPR	S20,T/S	1.006	0.966
Crebbp	CREB-binding protein	F8VPR5	Ambiguous	WGLGLDDEGSSQGEPQSKSPQESR	S	0.909	0.446
Creld2	Cysteine-rich with EGF-like domain protein 2	Q9CYA0	S346	VTEENPTQPPSREDL	S11	0.985	0.729
Crk	Adapter molecule crk	Q64010	S125	QGSGVILRQEEAEYVR	S3	0.963	0.420
Crk	Adapter molecule crk	Q64010	S41	DSSTSPGDYVLSVSENSR	S3	0.987	0.868
Crkl	Crk-like protein	P47941	S107	YPSPPVGSVSAPNLPTAEENLEYVR	S3	1.373	0.170
Crkl	Crk-like protein	P47941	Ambiguous	DSSTCPGDYVLSVSENSR	S/T/Y	1.122	0.420
Crlf2	Cytokine receptor-like factor 2	A0A0R4J0F5	S330	RVEPEDGTSLCTVPRPPSFEPR	S18	0.940	0.598
Crnkl1	Crooked neck-like protein 1	P63154	S689	EAAEQDPDKDIDESESSSF	S18	0.991	0.891
Crocc	Rootletin	Q8CJ40	S1469	EAPAGGSGDGLSSPSPLEYSPR	S13	0.997	0.962
Crtc2	CREB-regulated transcription coactivator 2	Q3U182	S171	TSSDSALHTSVMNPNPQDTYPGPTPPSVLPSRR	S3	0.968	0.581
Crtc2	CREB-regulated transcription coactivator 2	Q3U182	S448	SQQLPK	S1	1.034	0.760
Crtc2	CREB-regulated transcription coactivator 2	Q3U182	Ambiguous	TSSDSALHTSVMNPNPQDTYPGPTPPSVLPSR	S/T/Y	1.164	0.412
Crtc3	CREB-regulated transcription coactivator 3	Q91X84	S329	SNPSIQATLSK	S1	1.044	0.257
Crtc3	CREB-regulated transcription coactivator 3	Q91X84	S62	LTQYHGGSLPNVSQLR	S8	1.072	0.164
Crtc3	CREB-regulated transcription coactivator 3	Q91X84	S391,S396	QPPVSPLTLSPGPEAHQGFSR	S5,S10	0.913	0.390
Crtc3	CREB-regulated transcription coactivator 3	Q91X84	S391,S396	RQPPVSPLTLSPGPEAHQGFSR	S6,S11	1.006	0.854
Cry2	Cryptochrome-2	Q9R194	S557	ALSSGPASPK	S8	0.943	0.404
Crybg3	Beta/gamma crystallin domain-containing protein 3	A0A338P6N4	S1736	DSEGDTDSISEGMPAK	S8	1.067	0.245
Crybg3	Beta/gamma crystallin domain-containing protein 3	A0A338P6N4	S1917	GYESPTLSK	S4	1.017	0.805
Crybg3	Beta/gamma crystallin domain-containing protein 3	A0A338P6N4	S649	TSDYSASPPTLASR	S7	0.992	0.820
Crybg3	Beta/gamma crystallin domain-containing protein 3	A0A338P6N4	S790	SDSFPLETQTAK	S3	0.969	0.525
Crybg3	Beta/gamma crystallin domain-containing protein 3	A0A338P6N4	S825	ALSTPSVSQAEPR	S3	1.064	0.298
Crybg3	Beta/gamma crystallin domain-containing protein 3	A0A338P6N4	Ambiguous	RDNAYFQSAGTGNTSPSFCHTK	T/S/Y	0.994	0.960
Csde1	Cold shock domain-containing protein E1	Q91W50	S123	SPAAPGQSPTGSVCYER	S8	0.976	0.722
Csf1r	Macrophage colony-stimulating factor 1 receptor	P09581	Ambiguous	AEAMLGPSLSPGQDSEGDSSYK	S/Y	1.104	0.186
Csf2rb	Cytokine receptor common subunit beta	P26955	S565	VPPSGPDTTPAASSESTEQLPNVQVEGPTPNRPR	S16	1.026	0.735
Csf2rb	Cytokine receptor common subunit beta	P26955	Ambiguous	SPPGHPAPPVASSPTVIPGEPR	S/T	1.093	0.415
Csf3r	Granulocyte colony-stimulating factor receptor	P40223	S773	SDSTQPLLGGPTPSPK	S3	1.048	0.441

Csnk1a1	Casein kinase I isoform alpha	E9PWB2	T321	AAQQAASSSGQGQQAQTPTGK	T17	1.002	0.986
Csnk1a1	Casein kinase I isoform alpha	E9Q2U6	Ambiguous	AAQQAASSSGQGQQAQTPTGF	T/S	1.010	0.832
Csnk1d	Casein kinase I isoform delta	Q9DC28	S328	GLPSTASGR	S4	0.982	0.527
Csnk1d	Casein kinase I isoform delta	Q9DC28	S384	GAPVNVSSSDLTGR	S9	1.007	0.655
Csnk1d	Casein kinase I isoform delta	Q9DC28	T352,T/S	GTQEVAPPTPLTPTSHTANTSPRPVSGMER	T17,T/S	1.107	0.071
Csnk1d	Casein kinase I isoform delta	Q9DC28	Ambiguous	GTQEVAPPTPLTPTSHTANTSPRPVSGMER	T/S	1.041	0.081
Csnk1d	Casein kinase I isoform delta	Q9DC28	Ambiguous	GTQEVAPPTPLTPTSHTANTSPRPVSGMER	T/S	0.941	0.293
Csnk1e	Casein kinase I isoform epsilon	Q9JMK2	S363	IQQTGNTSPR	S8	1.052	0.162
Csnk1g1	Casein kinase I isoform gamma-1	A0A0U1RP94	Ambiguous	QTNTSYLTSHLAADR	T/S/Y	0.958	0.831
Csnk1g3	Casein kinase I isoform gamma-3	Q8C4X2	S32	GTGSSSSGVLMVGPNFR	S5	0.962	0.854
Csnk1g3	Casein kinase I isoform gamma-3	Q8C4X2	S367	NQVVSSTNGELNTDDPTAGR	S6	0.916	0.010
Csnk1g3	Casein kinase I isoform gamma-3	Q8C4X2	Ambiguous	QLPTPVGAVQQDPALSSNR	S/T	1.112	0.383
Csrp1	Cysteine and glycine-rich protein 1	P97315	S192	GFGFGQGAGALVHSE	S14	0.978	0.755
Cstf2	Cleavage stimulation factor subunit 2 (Fragment)	F6ZKC7	S145	GQGTLQHSPVGPAGPASIER	S8	0.976	0.317
Cstf2t	Cleavage stimulation factor subunit 2 tau variant	Q8C7E9	S579	QGGGQPSSFSPGQSQVTPQDQEK	S10	0.976	0.577
Cstf3	Cleavage stimulation factor subunit 3	Q99LI7	S691	RPNEDSDEDEEKGAVVPPVHDIYR	S6	0.946	0.406
Cstf3	Cleavage stimulation factor subunit 3	Q99LI7	S691	RPNEDSDEDEEK	S6	0.983	0.838
Ctdp1	RNA polymerase II subunit A C-terminal domain phosphatase	Q7TSG2	S730	DNSPAVFPDR	S3	1.027	0.766
Ctdp1	RNA polymerase II subunit A C-terminal domain phosphatase	Q7TSG2	S730	THRDNSPAVFPDR	S6	1.116	0.437
Ctdp1	RNA polymerase II subunit A C-terminal domain phosphatase	Q7TSG2	S814	AVQPHQQQMFGEELPESQDGEQPGPAR	S17	1.011	0.922
Ctdp1	RNA polymerase II subunit A C-terminal domain phosphatase	Q7TSG2	Ambiguous	EQPVGLPSSGERSTPGMR	S/T	0.928	0.273
Ctdp1	RNA polymerase II subunit A C-terminal domain phosphatase	Q7TSG2	Ambiguous	TTPGVQPTQGDAATQDLDFDLSSDSESSESSSR	S/T	1.022	0.906
Ctdspl2	CTD small phosphatase-like protein 2	Q8BG15	S165	NGTSGSDSPGQAVEAEEIVK	S8	0.942	0.337
Ctif	CBP80/20-dependent translation initiation factor	E9Q1U6	S18	SQEIEELER	S1	1.042	0.544
Ctnna1	Catenin alpha-1	P26231	S641	TPEELDDSDFETEDFDVR	S8	1.052	0.115
Ctnna1	Catenin alpha-1	P26231	S655	TSVQTEDDQLIAGQSAR	S2	0.903	0.243
Ctnna1	Catenin alpha-1	P26231	S655	SRTSVQTEDDQLIAGQSAR	S4	0.983	0.764
Ctnna1	Catenin alpha-1	P26231	Ambiguous	SRTSVQTEDDQLIAGQSAR	S/T	1.031	0.637
Ctnnb1	Catenin beta-1	Q02248	S552	TSMGGTQQQFVEGVR	S2	1.002	0.987
Ctnnb1	Catenin beta-1	Q02248	S552	RTSMGGTQQQFVEGVR	S3	1.149	0.057
Ctnnb1	Catenin beta-1	Q02248	Ambiguous	RTSMGGTQQQFVEGVR	T/S	1.048	0.265
Ctnnbl1	Beta-catenin-like protein 1	Q9CWL8	S545	EYAENIGDGRSPEFR	S11	1.013	0.707
Ctnnd1	Catenin delta-1	E9Q8Z8; E9Q8Z5	S230;S230	HYEDGYPGGSDNYGSLSR	S15	0.990	0.711
Ctnnd1	Catenin delta-1	E9Q8Z8; E9Q8Z5	S252;S252	APSRQDVYGPQPQVR	S3	0.998	0.930
Ctnnd1	Catenin delta-1	E9Q8Z8; E9Q8Z5	S268;S268	VGGSSVDLHR	S4	0.960	0.507
Ctnnd1	Catenin delta-1	E9Q8Z8; E9Q8Z5	S288;S288	SMGYDDLDYGMMSDYGTAR	S1	0.962	0.734
Ctnnd1	Catenin delta-1	E9Q8Z8; E9Q8Z5	S349;S349	GSLASLDSLR	S5	1.007	0.956
Ctnnd1	Catenin delta-1	E9Q8Z8; E9Q8Z5	S864;S858	SQSSHSYDDSTLPLIDR	S6	0.967	0.353
Ctnnd1	Catenin delta-1	E9Q8Z8	S899	SLDNNYSTLNER	S1	1.173	0.169
Ctnnd1	Catenin delta-1	E9Q8Z5	Ambiguous	SDNNYSTLNER	S/T/Y	1.175	0.062
Ctnnd1	Catenin delta-1	E9Q8Z8; E9Q8Z5	Ambiguous	FHPEPYGLEDDQRSMGYDDLDYGMMSDYGTAR	Y/S/T	1.217	0.213
Ctnnd2	Catenin delta-2	O35927	S1149	NSYGAPAEDIK	S2	0.915	0.367
Ctnnd2	Catenin delta-2	O35927	S435	SLSQSQGDPLPPAHTGTFR	S3	0.994	0.928
Ctnnd2	Catenin delta-2	O35927	Ambiguous	TSTAPSSPGVDSVPLQR	S/T	1.196	0.047
Ctnnd2	Catenin delta-2	O35927	Ambiguous	TGSQHGPQNAAAATFQR	S/T	1.042	0.727
Ctnnd2	Catenin delta-2	O35927	Ambiguous	TSTAPSSPGVDSVPLQR	S/T	0.894	0.439
Ctps1	CTP synthase 1	P70698	S562	GCRLSPR	S5	1.065	0.440
Ctr9	RNA polymerase-associated protein CTR9 homolog	Q62018	S970	GEEGSEEEETENGPKPK	S5	0.976	0.818
Ctr9	RNA polymerase-associated protein CTR9 homolog	Q62018	T925	GGEFDEFVNDDTDDDLPVSK	T12	1.020	0.495
Ctr9	RNA polymerase-associated protein CTR9 homolog	Q62018	S941,S943	KGSGSEQEGEEEEGGER	S3,S5	1.022	0.716
Ctr9	RNA polymerase-associated protein CTR9 homolog	Q62018	S941,S943	RKGSGSEQEGEEEEGGER	S4,S6	0.943	0.545
Ctsb	Cathepsin B	P10605	Ambiguous	EQWSNCPTIGQIR	T/S	0.973	0.862
Ctsl	Cathepsin L1	P06797	S198	ENGGLDSEESYPYEAK	S7	1.015	0.894
Cttn	Src substrate cortactin	Q60598	S407,S/Y/T	QTPPASPSPQPIEDRPPSSPIYEDAAPFK	S8,S/Y/T	1.191	0.230
Cttn	Src substrate cortactin	Q60598	Ambiguous	QTPPASPSPQPIEDRPPSSPIYEDAAPFK	S/Y/T	1.080	0.632
Cttn	Src substrate cortactin	Q60598	Ambiguous	QTPPASPSPQPIEDRPPSSPIYEDAAPFKAEPSYR	S/Y/T	1.236	0.360
Cttnbp2nl	CTTNBP2 N-terminal-like protein	Q99LJ0	S481	FQSQADQDQQASGLQSPPSR	S16	1.042	0.278
Cttnbp2nl	CTTNBP2 N-terminal-like protein	Q99LJ0	S488	DLSPTLLDNSAAK	S3	1.002	0.989
Cttnbp2nl	CTTNBP2 N-terminal-like protein	Q99LJ0	S548	GDTSHSPTPGK	S6	1.128	0.199
Cttnbp2nl	CTTNBP2 N-terminal-like protein	Q99LJ0	Ambiguous	HKFQSQADQDQQASGLQSPPSR	S	1.013	0.796
Ctu1	Cytoplasmic tRNA 2-thiolation protein 1	Q99J10	S416	AGATCVSQCDLSPVAE	S12	1.103	0.018
Ctu2	Cytoplasmic tRNA 2-thiolation protein 2	Q3U308	S200	VLGAEAGASPAQGEAR	S9	1.049	0.752
Cul4b	Cullin-4B	A2A432	S104	SATDGNTSTTPPTSAK	S8	1.017	0.854
Cul4b	Cullin-4B	A2A432	S200	MAEESSSSSSSSPTAATSQQQQQQQLK	S13	0.962	0.769
Cul4b	Cullin-4B	A2A432	S82	SVCPGTSGFSSPNPSAASAAAQEVR	S11	1.038	0.831
Cul4b	Cullin-4B	A2A432	T140	KLNSSSSSSNSSNEREDFDSTSSSSTPPQPR	T26	0.998	0.983
Cul4b	Cullin-4B	A2A432	S110,S/T	SATDGNTSTTPPTSAK	S14,T/S	1.084	0.219
Cul4b	Cullin-4B	A2A432	Ambiguous	MAEESSSSSSSSPTAATSQQQQQQQLK	S/T	1.030	0.489
Cul4b	Cullin-4B	A2A432	Ambiguous	LNSSSSSSNSSNEREDFDSTSSSSTPPQPR	T/S	1.007	0.959
Cul9	Cullin-9	S4R1Y1	S2440	VGLQSPSVETR	S5	1.072	0.462
Cul9	Cullin-9	S4R1Y1	S584	ELGSLPSSR	S4	0.984	0.828
Cux1	Homeobox protein cut-like	H3BJQ9	S1213	HSSVSDSQPCEPPSVGIDYSQGASPQPQHQLK	S3	0.843	0.320
Cux1	Homeobox protein cut-like	H3BJQ9	S1267	AYQQKPYPSPK	S9	1.036	0.795
Cux1	Homeobox protein cut-like	H3BJQ9	S1330	AAPSSEGDSCDGVEATDAEEPGGNIVATK	S5	1.004	0.975
Cux1	Homeobox protein cut-like	H3BJQ9	S429	RPGPLPASPPPQLPR	S8	0.944	0.315
Cux1	Homeobox protein cut-like	H3BJQ9	S1051,S1056	TSASCSPAPESPMSSSESVK	S6,S11	0.893	0.112
Cux1	Homeobox protein cut-like	H3BJQ9	S1329,S1330	AAPSSEGDSCDGVEATDAEEPGGNIVATK	S4,S5	0.935	0.455
Cux1	Protein CASP (Fragment)	H3BLJ7	S63,S67	APDVLQVDGSPSSSPR	S10,S14	1.021	0.680
Cwc22	Pre-mRNA-splicing factor CWC22 homolog	Q8C5N3	S106	GGGQSSPQEEPTWK	S6	1.012	0.887
Cwc22	Pre-mRNA-splicing factor CWC22 homolog	Q8C5N3	S60	GYSYDDSMESR	S3	1.009	0.858
Cwc22	Pre-mRNA-splicing factor CWC22 homolog	Q8C5N3	S60	GYSYDDSMESR	S3	0.977	0.669
Cwc22	Pre-mRNA-splicing factor CWC22 homolog	Q8C5N3	S829	RDSFSENEK	S3	1.128	0.067
Cwc22	Pre-mRNA-splicing factor CWC22 homolog	Q8C5N3	S829	ERRDSFSENEK	S5	1.066	0.357
Cwc22	Pre-mRNA-splicing factor CWC22 homolog	Q8C5N3	S829	RDSFSENEKQR	S3	0.948	0.493
Cx3cl1	Fractalkine	O35188	S262	SLGSEEINPVHTDNFQER	S4	1.086	0.248
Cyba	Cytochrome b-245 light chain	Q61462	S168	KKPSEGEEEAASAGGPQVNPMPVTDEVV	S4	0.923	0.091
Cyba	Cytochrome b-245 light chain	Q61462	S168	KKPSEGEEEAASAGGPQVNPMPVTDEVV	S4	0.994	0.883
Cyba	Cytochrome b-245 light chain	Q61462	T147	ERPQVGGTIK	T8	1.086	0.413
Cyba	Cytochrome b-245 light chain	Q61462	T153	QPPTNPPPRPPAEVR	T4	1.048	0.693
Cybc1	Cytochrome b-245 chaperone 1	Q3TYS2	S148	SDVEAIAK	S1	1.045	0.704
Cybc1	Cytochrome b-245 chaperone 1	Q3TYS2	S176	SQSSDSEPDGPGGQS	S4	1.064	0.665
Cyfip1	Cytoplasmic FMR1-interacting protein 1	Q7TMB8	S872	TVLPFSQEFQR	S6	1.011	0.966
Cyfip1	Cytoplasmic FMR1-interacting protein 1	Q7TMB8	T1068	LGTPQQIAIAR	T3	0.933	0.841
Cyfip1	Cytoplasmic FMR1-interacting protein 1	Q7TMB8	Y108	CNEQPNRVEIYEK	Y11	0.996	0.945
Cyld	Ubiquitin carboxyl-terminal hydrolase CYLD	Z4YJJ5	S13	VTSPYWEER	S3	1.102	0.272
Cyth4	Cytohesin-4	Q80YW0	S222	GINSGSDLPEEQLR	S4	1.175	0.103
Cytip	Cytohesin-interacting protein	Q91VY6	S242	LSSESSCK	S3	1.409	0.013
Cytip	Cytohesin-interacting protein	Q91VY6	S279	QTSTDDECFHSK	S3	1.126	0.140
Cytip	Cytohesin-interacting protein	Q91VY6	Ambiguous	QTSTDDECFHSKDGDEILR	T/S	1.205	0.047
D10Wsu102e	Uncharacterized protein C12orf45 homolog	Q9CX66	S143	DSSGDSSESEEDVCVPSEVTIENIK	S7	0.909	0.553
D10Wsu102e	Uncharacterized protein C12orf45 homolog	Q9CX66	S177	IEILDSPASK	S6	0.946	0.563
D10Wsu102e	Uncharacterized protein C12orf45 homolog	Q9CX66	S177	GKIEILDSPASK	S8	1.075	0.464
D17Wsu92e	Uncharacterized protein C6orf106 homolog	B2KF52	S222	NRQSDENNLTDPGGSEFDSISK	S4	1.062	0.618
D5Ertd579e	DNA segment, Chr 5, ERATO Doi 579,-expressed	E9QPJ5	S1295	ALYSPLFPTSECEECYTNAK	S4	0.985	0.836
D5Ertd579e	DNA segment, Chr 5, ERATO Doi 579,-expressed	E9QPJ5	S155	LEGSPSPEEELSPTAK	S4	1.084	0.444
D5Ertd579e	DNA segment, Chr 5, ERATO Doi 579,-expressed	E9QPJ5	S155	KLEGSPSPEEELSPTAK	S5	1.025	0.855
D5Ertd579e	DNA segment, Chr 5, ERATO Doi 579,-expressed	E9QPJ5	S840	SSVAAVETER	S2	1.096	0.140
D6Wsu163e	Protein C12orf4 homolog	Q91YN0	Ambiguous	NPNNSSLSEEIK	S	1.127	0.219
Dab2	Disabled homolog 2	A0A0R4J104	S227	LGVDQMDLFGDMSTPPDLNSPTESK	S20	1.136	0.091
Dab2	Disabled homolog 2	A0A0R4J104	S325	ASLSSSSTPQSK	S4	1.354	0.002
Dab2	Disabled homolog 2	A0A0R4J104	S401	SSPNPFVGSPPK	S9	0.998	0.969
Dab2	Disabled homolog 2	A0A0R4J104	S731	SADNSLENPFSK	S5	1.091	0.395
Dab2	Disabled homolog 2	A0A0R4J104	S753	GFSSSNPSVVSQPASSDPHR	S15	1.071	0.352
Dab2	Disabled homolog 2	A0A0R4J104	S401,S	SSPNPFVGSPPK	S9,S	1.093	0.346
Dab2	Disabled homolog 2	A0A0R4J104	T329,S	ASLSSSSTPQSK	T8,S	1.056	0.210
Dab2	Disabled homolog 2	A0A0R4J104	Ambiguous	DDPFAQPDQSAPSSFDSLTSPDQK	S/T	1.438	0.107
Dab2	Disabled homolog 2	Q9DCE6	Ambiguous	LGVDQMDLFGDMSTPPDLNSPTGDCSEV	T/S	1.088	0.774
Dab2ip	Disabled homolog 2-interacting protein	Q3UHC7	S728	SSSYSEANEPDLQMANGSK	S3	0.948	0.410
Dab2ip	Disabled homolog 2-interacting protein	Q3UHC7	S995	QGPSPVSPNALDR	S7	1.087	0.540
Dag1	Dystroglycan	Q62165	S886	NMTPYRSPPPYVPP	S7	1.099	0.214
Dagla	Sn1-specific diacylglycerol lipase alpha	Q6WQJ1	S808	GSPSLHAVLER	S2	1.019	0.879
Daglb	Sn1-specific diacylglycerol lipase beta	Q91WC9	S570	YSPGYCSSDSPLDSPTK	S2	1.143	0.072
Daglb	Sn1-specific diacylglycerol lipase beta	Q91WC9	S578	YSPGYCSSDSPLDSPTKYPTLYPPGR	S10	1.033	0.738
Daglb	Sn1-specific diacylglycerol lipase beta	Q91WC9	S659	TACVSCPGQGGSSVP	S5	1.014	0.787
Dapk1	Death-associated protein kinase 1	Q80YE7	T336	SDDTLDEEDSFVMK	T4	1.009	0.891
Dapk1	Death-associated protein kinase 1	Q80YE7	Ambiguous	SNMSVARSDDTLDEEDSFVMK	S/T	1.130	0.159
Dapp1	Dual adapter for phosphotyrosine and 3-phosphotyrosine and 3-phosphoinositide	Q9QXT1	Y139	EVEEPCIYESVR	Y8	0.994	0.957
Dars	Aspartate--tRNA ligase, cytoplasmic	Q922B2	S190	TSTSQAIFR	S2	0.921	0.267
Dars	Aspartate--tRNA ligase, cytoplasmic	Q922B2	S249	NNAYLAQSPQLYK	S8	0.936	0.256
Dars	Aspartate--tRNA ligase, cytoplasmic	Q922B2	T370	EAGVEMDDEEDLSTPNEK	T14	0.921	0.224
Daxx	Death domain-associated protein 6	Q3UKR0	S516	RNSEPAEGLR	S3	1.119	0.159
Daxx	Death domain-associated protein 6	Q3UKR0	T524	NSEPAEGLRTPEGQQKR	T10	0.877	0.303
Daxx	Death domain-associated protein 6	Q3UKR0	T524	NSEPAEGLRTPEGQQK	T10	0.957	0.366
Daxx	Death domain-associated protein 6	Q3UKR0	S516,T524	RNSEPAEGLRTPEGQQK	S3,T11	1.133	0.076
Daxx	Death domain-associated protein 6	Q3UKR0	Ambiguous	TSVATQCDPEEIIVLSDSD	S/T	1.262	0.280
Dbn1	Drebrin	Q9QXS6	S142	LSSPVLHR	S3	1.016	0.575
Dbn1	Drebrin	Q9QXS6	S387	SPSDSSTASTPIAEQIER	S3	0.912	0.658
Dbndd2	Dysbindin domain-containing protein 2	Q9CRD4	S113	TTSRTSSLSSDSSNLR	S3	1.048	0.412

Dbndd2	Dysbindin domain-containing protein 2	Q9CRD4	S117	TSSLSSDSSNLR	S3	1.040	0.570
Dbndd2	Dysbindin domain-containing protein 2	Q9CRD4	S142	SPNPSDGGGDTPLAQSDEEDGDDGGAEPGPCS	S16	1.020	0.839
Dbndd2	Dysbindin domain-containing protein 2	Q9CRD4	S113,S/T	TTSRTSSLSSDSSNLR	S3,S/T	1.175	0.098
Dbnl	Drebrin-like protein	Q62418	S277	AMSTTSVTSSQPGK	S3	1.019	0.854
Dbnl	Drebrin-like protein	Q62418	T299	QLTQPETSYGR	T3	0.983	0.517
Dbnl	Drebrin-like protein	Q62418	Ambiguous	AMSTTSVTSSQPGK	T/S	1.129	0.313
Dbp	D site-binding protein	Q60925	S86	ATLPSAPVPGPGLETAGPADAPSGAVSGGGSPR	S31	1.114	0.766
Dbr1	Lariat debranching enzyme	Q923B1	S491	NLPSSMFVSSDDASRSPASGEGK	S16	1.025	0.352
Dbr1	Lariat debranching enzyme	Q923B1	S505	CGETVESGDEK	S7	0.996	0.966
Dbr1	Lariat debranching enzyme	Q923B1	S548	NQAIYAAVDDGDASAE	S14	1.018	0.903
Dbr1	Lariat debranching enzyme	Q923B1	S548	RNQAIYAAVDDGDASAE	S15	1.038	0.551
Dbr1	Lariat debranching enzyme	Q923B1	S489,S491	NLPSSMFVSSDDASRSPASGEGK	S14,S16	1.006	0.930
Dcaf10	DDB1- and CUL4-associated factor 10	A2AKB9	S356	TTSSSDLTTTSSSSGSR	S3	1.117	0.295
Dcaf10	DDB1- and CUL4-associated factor 10	A2AKB9	S57	GADAASPPPVAGSPR	S13	1.057	0.472
Dcaf10	DDB1- and CUL4-associated factor 10	A2AKB9	S67	LPGGPAVSPAER	S8	1.056	0.394
Dcaf10	DDB1- and CUL4-associated factor 10	A2AKB9	S50,S57	GADAASPPPVAGSPR	S6,S13	0.971	0.520
Dcaf11	DDB1- and CUL4-associated factor 11	Q91VU6	S21	NSSSAGSGSLEPSEGLSR	S17	1.058	0.274
Dcaf11	DDB1- and CUL4-associated factor 11	Q91VU6	S21	NSSSAGSGSLEPSEGLSRR	S17	0.959	0.717
Dcaf17	DDB1- and CUL4-associated factor 17	Q3TUL7	S412	NVSLLDDDPEQETFK	S3	1.543	0.021
Dcaf5	DDB1- and CUL4-associated factor 5	Q80T85	S799	AEEPPASPGPK	S7	0.976	0.614
Dcaf6	DDB1- and CUL4-associated factor 6	Q9DC22	S336	DGEQSPNVSLMQR	S5	0.874	0.342
Dcaf6	DDB1- and CUL4-associated factor 6	Q9DC22	S336	ERDGEQSPNVSLMQR	S7	1.193	0.299
Dcbld2	Discoidin, CUB and LCCL domain-containing protein 2	Q91ZV3	S719,S/T/Y	TDSCSSGQAQYDTPK	S3,S/T/Y	0.815	0.010
Dck	Deoxycytidine kinase	P43346	S11	RFCPSPSTSSEGTR	S5	1.002	0.970
Dck	Deoxycytidine kinase	P43346	S11	FCPSPSTSSEGTR	S4	0.988	0.427
Dck	Deoxycytidine kinase	P43346	S13,S16	FCPSPSTSSEGTR	S6,S9	1.050	0.114
Dclk1	Dclk1 protein	Q80VB6	S352	DLYRPLSSDDLDSVGDSV	S7	1.273	0.494
Dclre1b	5' exonuclease Apollo	Q8C7W7	S357	TQGVVFESPEEK	S8	0.924	0.624
Dclre1c	Protein artemis	Q8K4J0	Ambiguous	DEITGECLEHLPSSIETGGSQSPK	S/T	0.979	0.902
Dcp1a	mRNA-decapping enzyme 1A	Q91YD3	S543	ASSPSPLTVGTAESQR	S3	0.981	0.488
Dcp1a	mRNA-decapping enzyme 1A	Q91YD3	S545	KASSPSPLTVGTAESQR	S6	0.922	0.138
Dcp1a	mRNA-decapping enzyme 1A	Q91YD3	S82	SASPYHGFTIVNR	S3	0.981	0.654
Dctn1	Dynactin subunit 1	O08788	T108	QSQIQVFEDGADTTSPETPDSSASK	T18	1.021	0.627
Dctn2	Dynactin subunit 2	Q99KJ8	S392	ENLATVEGNFASIDAR	S12	0.923	0.735
Dctn2	Dynactin subunit 2	Q99KJ8	Y6	YADLPGIAR	Y1	0.878	0.116
Dctn4	Dynactin subunit 4	Q8CBY8	Ambiguous	ATSISTQLPDDPAK	T/S	0.968	0.779
Dctpp1	dCTP pyrophosphatase 1	Q9QY93	Ambiguous	GTISENQAVGAGDPASELRDQAST	S/T	0.973	0.718
Dda1	DET1- and DDB1-associated protein 1	Q9D9Z5	S33	RPSVYLPTR	S3	1.071	0.109
Dda1	DET1- and DDB1-associated protein 1	Q9D9Z5	S95	TDSPDMPEDT	S3	0.984	0.627
Dda1	DET1- and DDB1-associated protein 1	Q9D9Z5	S95	TDSPDMPEDT	S3	1.055	0.650
Ddhd1	Phospholipase DDHD1	F8WIJ5	Ambiguous	VCGPSSSFGEEDEEDSACGFCPR	S	0.930	0.444
Ddi2	Protein DDI1 homolog 2	A2ADY9	S121	TQAQHSSPGEMASSPQGLDNPALLR	S7	1.035	0.523
Ddi2	Protein DDI1 homolog 2	A2ADY9	S121	TQAQHSSPGEMASSPQGLDNPALLR	S7	1.003	0.956
Ddi2	Protein DDI1 homolog 2	A2ADY9	S128,S/T	TQAQHSSPGEMASSPQGLDNPALLR	S14,S/T	1.054	0.629
Ddx1	ATP-dependent RNA helicase DDX1	Q91VR5	S481	DNTRPGANSPEMWSEAIK	S9	0.895	0.044
Ddx1	ATP-dependent RNA helicase DDX1	Q91VR5	S481	DNTRPGANSPEMWSEAIK	S9	1.091	0.498
Ddx10	Probable ATP-dependent RNA helicase DDX10	Q80Y44	S549	APSLPNDEADESPAYVSEK	S12	0.979	0.810
Ddx10	Probable ATP-dependent RNA helicase DDX10	Q80Y44	T587	LASGDGDEEQDEETEDEETEDHLGK	T14	1.015	0.726
Ddx17	DEAD (Asp-Glu-Ala-Asp) box polypeptide 17, isoform CRA_a	Q3U741	S494	TTSSANNPNLMYQDECDRR	S3	2.112	0.000
Ddx17	DEAD (Asp-Glu-Ala-Asp) box polypeptide 17, isoform CRA_a	Q3U741	S494	TTSSANNPNLMYQDECDR	S3	1.259	0.026
Ddx18	ATP-dependent RNA helicase DDX18	Q8K363	T101	LTTLANGEAAPTPPPDSEVK	T12	0.929	0.751
Ddx20	Probable ATP-dependent RNA helicase DDX20	Q9JJY4	S37	AAAPVQAVEPTPASPWTQR	S14	0.760	0.287
Ddx20	Probable ATP-dependent RNA helicase DDX20	Q9JJY4	S472	AAMHTYSSPTVATQSPK	S15	1.030	0.685
Ddx20	Probable ATP-dependent RNA helicase DDX20	Q9JJY4	S496	TPGNQTPSPR	S8	1.068	0.518
Ddx21	Nucleolar RNA helicase 2	Q9JIK5	S118	QADSETKEIITEEPSEEEADMPKPK	S15	0.952	0.693
Ddx21	Nucleolar RNA helicase 2	Q9JIK5	S118	QADSETKEIITEEPSEEEADMPKPK	S15	1.002	0.992
Ddx21	Nucleolar RNA helicase 2	Q9JIK5	S118	EIITEEPSEEEADMPKPK	S8	1.021	0.750
Ddx21	Nucleolar RNA helicase 2	Q9JIK5	S118	EIITEEPSEEEADMPKPK	S8	1.016	0.750
Ddx21	Nucleolar RNA helicase 2	Q9JIK5	S144;S315	EANGDAGEKSPK	S10	1.083	0.481
Ddx21	Nucleolar RNA helicase 2	Q9JIK5	S155	NGLSQPSEEEADIPKPK	S7	1.080	0.512
Ddx21	Nucleolar RNA helicase 2	Q9JIK5	S192	NGLSQPSEEEVDIPKPK	S7	1.035	0.817
Ddx21	Nucleolar RNA helicase 2	Q9JIK5	S218	EASGDAGEKSPR	S10	1.134	0.102
Ddx21	Nucleolar RNA helicase 2	Q9JIK5	S243	SNSSDAPGEESSSETEKEIPVEQK	S11	0.998	0.954
Ddx21	Nucleolar RNA helicase 2	Q9JIK5	S243	SNSSDAPGEESSSETEK	S11	1.112	0.111
Ddx21	Nucleolar RNA helicase 2	Q9JIK5	S243,S245	SNSSDAPGEESSSETEKEIPVEQK	S11,S13	1.025	0.505
Ddx21	Nucleolar RNA helicase 2	Q9JIK5	T114,S118	EIITEEPSEEEADMPKPK	T4,S8	1.017	0.878
Ddx21	Nucleolar RNA helicase 2	Q9JIK5	Ambiguous	KQEEEPQDDTASTSK	S/T	1.113	0.513
Ddx23	DEAD (Asp-Glu-Ala-Asp) box polypeptide 23	D3Z0M9	S106,S108	RSSLSPGR	S3,S5	1.095	0.258
Ddx24	ATP-dependent RNA helicase DDX24	F8WJA0	S113	LFSSEETK	S3	1.224	0.057
Ddx24	ATP-dependent RNA helicase DDX24	F8WJA0	S126	AQAVSEEEEEEGQSSSPK	S5	1.057	0.181
Ddx24	ATP-dependent RNA helicase DDX24	F8WJA0	S169	AAEGAAAQNEYEVKASEPEAQGEVTACSDQK	S16	1.168	0.120
Ddx24	ATP-dependent RNA helicase DDX24	F8WJA0	S126,S138	KAQAVSEEEEEEGQSSSPK	S6,S18	0.901	0.252
Ddx24	ATP-dependent RNA helicase DDX24	F8WJA0	Ambiguous	APPQPGSSTS	T/S	0.985	0.766
Ddx3x	ATP-dependent RNA helicase DDX3X	Q62167	S594	FSGGFGAR	S2	0.943	0.327
Ddx3x	ATP-dependent RNA helicase DDX3X	Q62167	S594	SSRFSGGFGAR	S5	1.010	0.905
Ddx3x	ATP-dependent RNA helicase DDX3X	Q62167	Ambiguous	VGSTSENITQK	S/T	1.007	0.891
Ddx41	DEAD (Asp-Glu-Ala-Asp) box polypeptide 41	A0A1S6GWJ4	Ambiguous	ARADEATAGGSRSEDEDEDDEDYVPYVPLR	T/S/Y	0.818	0.296
Ddx41	DEAD (Asp-Glu-Ala-Asp) box polypeptide 41	A0A1S6GWJ4	Ambiguous	ADEATAGGSRSEDEDEDDEDYVPYVPLR	T/S/Y	0.901	0.561
Ddx42	ATP-dependent RNA helicase DDX42	Q810A7	Ambiguous	YMAENPTAGVVQEEEEDNLEYDSDGNPIAPSK	Y/S/T	0.968	0.476
Ddx50	ATP-dependent RNA helicase DDX50	Q99MJ9	S85	FSDEFSPSHK	S6	1.195	0.060
Ddx51	ATP-dependent RNA helicase DDX51	Q6P9R1	S432	LGQQSPKDTAEVDENSGK	S5	1.024	0.709
Ddx51	ATP-dependent RNA helicase DDX51	Q6P9R1	S77	RVSGSATPNSEAPR	S3	0.963	0.713
Ddx54	ATP-dependent RNA helicase DDX54	Q8K4L0	S74	ALPSFPTSECVSDVEPDTR	S12	0.971	0.834
Ddx54	ATP-dependent RNA helicase DDX54	Q8K4L0	S774	IDDRDSEEEGPSNQR	S6	1.167	0.080
Ddx54	ATP-dependent RNA helicase DDX54	Q8K4L0	S774	QKIDDRDSEEEGPSNQR	S8	1.218	0.044
Ddx55	ATP-dependent RNA helicase DDX55	Q6ZPL9	S544	DEGSDIDDEDMEELLNDTR	S4	0.726	0.100
Ddx55	ATP-dependent RNA helicase DDX55	Q6ZPL9	S594	TVQLTDLGVSDLEEDS	S10	1.145	0.625
Deaf1	Deformed epidermal autoregulatory factor 1 (Drosophila), isoform CRA_d	A0A1B0GRZ5	S177	GPAAPLTPGPQSPPTPLAPGQEK	S12	0.948	0.670
Deaf1	Deformed epidermal autoregulatory factor 1 (Drosophila), isoform CRA_d	A0A1B0GRZ5	T172,S/T	GPAAPLTPGPQSPPTPLAPGQEK	T7,T/S	1.135	0.496
Def6	Differentially-expressed in FDCP 6	A0A0R4IZX1	S597	TLSVNSSEQK	S3	1.209	0.018
Dek	Protein DEK	Q7TNV0	Ambiguous	ESESEDSSDDEPLIK	S	0.856	0.110
Dennd1a	DENN domain-containing protein 1A	Q8K382	S473	TMPSPQAETQDPR	S4	1.014	0.856
Dennd1a	DENN domain-containing protein 1A	Q8K382	S473	TMPSPQAETQDPR	S4	0.997	0.954
Dennd1a	DENN domain-containing protein 1A	Q8K382	S520	RTSVSSPEQPQPYR	S3	1.024	0.492
Dennd1a	DENN domain-containing protein 1A	Q8K382	S523	TSVSSPEQPQPYR	S5	0.945	0.391
Dennd1a	DENN domain-containing protein 1A	Q8K382	S592	SLEDLRAPK	S1	0.936	0.210
Dennd1a	DENN domain-containing protein 1A	Q8K382	S991	QDVSPSPAPALAPASTSVEQLRR	S4	1.064	0.545
Dennd1a	DENN domain-containing protein 1A	Q8K382	S991	QDVSPSPAPALAPASTSVEQLR	S4	0.980	0.246
Dennd1a	DENN domain-containing protein 1A	Q8K382	T24	TGGTLSDPEVQR	T1	1.059	0.248
Dennd1a	DENN domain-containing protein 1A	Q8K382	S520,S522	RTSVSSPEQPQPYR	S3,S5	1.018	0.745
Dennd1a	DENN domain-containing protein 1A	Q8K382	S538,T/S	TLKESDSAEGDETESPEQLVR	S7,T/S	0.957	0.758
Dennd1a	DENN domain-containing protein 1A	Q8K382	Ambiguous	TLKESDSAEGDETESPEQLVR	T/S	1.039	0.565
Dennd1b	DENN domain-containing protein 1B	Q3U1T9	S516	ASKISSEDGEETR	S5	0.999	0.993
Dennd1b	DENN domain-containing protein 1B	Q3U1T9	S517	ISSEDGEETR	S3	1.074	0.006
Dennd1b	DENN domain-containing protein 1B	Q3U1T9	S530	AYFYESDDSVEAQVK	S6	1.113	0.430
Dennd1b	DENN domain-containing protein 1B	Q3U1T9	S516,S517	ASKISSEDGEETR	S5,S6	1.045	0.195
Dennd1c	DENN domain-containing protein 1C	Q8CFK6	S703	HSPPESPQLLVSTEPNSDAVQR	S6	0.953	0.702
Dennd1c	DENN domain-containing protein 1C	Q8CFK6	T696	VPTRHSPPESPQLLVSTEPNSDAVQR	T3	0.939	0.622
Dennd2c	DENN domain-containing protein 2C	E9Q4E4	S443	GETSGNESDAEYLPK	S8	0.876	0.080
Dennd4a	DENN/MADD domain-containing 4A	E9Q8V6	S1225	TPSIDLQR	S3	1.000	1.000
Dennd4a	DENN/MADD domain-containing 4A	E9Q8V6	S911	SDLGYNSLSK	S7	0.818	0.228
Dennd4b	DENN domain-containing protein 4B	A0A0R4J172	S727	AELFESPQEQQGALPVPGPSR	S6	1.614	0.034
Dennd4b	DENN domain-containing protein 4B	A0A0R4J172	S747	SAPSSPAPR	S5	1.045	0.584
Dennd4b	DENN domain-containing protein 4B	A0A0R4J172	S941	SGSSQTEPYLERPSPTRPLQR	S14	1.021	0.531
Dennd4b	DENN domain-containing protein 4B	A0A0R4J172	S956	SLREPSSPMGR	S1	1.039	0.610
Dennd4b	DENN domain-containing protein 4B	A0A0R4J172	S962	SLREPSSPMGR	S7	1.022	0.376
Dennd4b	DENN domain-containing protein 4B	A0A0R4J172	S972	SGSLGSAR	S3	1.041	0.611
Dennd4b	DENN domain-containing protein 4B	A0A0R4J172	T878	WPSGTPGGR	T5	0.948	0.085
Dennd4b	DENN domain-containing protein 4B	A0A0R4J172	S743,S747	SAPSSPAPR	S1,S5	1.043	0.401
Dennd4b	DENN domain-containing protein 4B	A0A0R4J172	S956,S	SLREPSSPMGR	S1,S	0.981	0.876
Dennd4c	DENN domain-containing protein 4C	A6H8H2	S1087	TSPPHVTR	S2	1.040	0.542
Dennd4c	DENN domain-containing protein 4C	A6H8H2	S1096	THSFENVNCHLADSR	S3	1.128	0.033
Dennd4c	DENN domain-containing protein 4C	A6H8H2	S1248	SCSMELHGEGNQEPGSPAVFAHPLER	S3	1.035	0.755
Dennd4c	DENN domain-containing protein 4C	A6H8H2	S1248	SCSMELHGEGNQEPGSPAVFAHPLER	S3	1.002	0.963
Dennd4c	DENN domain-containing protein 4C	A6H8H2	S1274	SSSLPSDRGPPAR	S3	1.137	0.010
Dennd4c	DENN domain-containing protein 4C	A6H8H2	S1293	DSTETEKSSPAVSSSK	S9	0.987	0.512
Dennd4c	DENN domain-containing protein 4C	A6H8H2	S1248,S1261	SCSMELHGEGNQEPGSPAVFAHPLER	S3,S16	1.057	0.695
Dennd4c	DENN domain-containing protein 4C	A6H8H2	Ambiguous	SCSFSSESR	S	1.246	0.001
Dennd4c	DENN domain-containing protein 4C	A6H8H2	Ambiguous	HPPGSSITNSPALMAK	T/S	1.009	0.921
Dennd5a	DENN domain-containing protein 5A	Q6PAL8	S1085	TPPLQQSPSVIR	S7	1.082	0.070
Dennd5a	DENN domain-containing protein 5A	Q6PAL8	S1085	TPPLQQSPSVIRR	S7	0.961	0.716
Dennd5a	DENN domain-containing protein 5A	Q6PAL8	S193	FNSYDISR	S3	1.394	0.006
Dennd6a	Protein DENND6A	Q8BH65	S16	GSLDEAGAEGR	S2	1.081	0.237
Denr	Density-regulated protein	Q9CQJ6	S73	LTVENSPKQETGITEGQGPVGEEEEK	S6	1.016	0.764
Depdc5	GATOR complex protein DEPDC5	P61460	S1518	RNSTSSTNQNMFCEER	S3	0.940	0.527
Depdc5	GATOR complex protein DEPDC5	P61460	S1518	RNSTSSTNQNMFCEER	S3	1.046	0.749
Depdc5	GATOR complex protein DEPDC5	P61460	S503	SASSCDVSSSPSLPSR	S10	0.894	0.331
Depdc5	GATOR complex protein DEPDC5	P61460	Ambiguous	KSASSCDVSSSPSLPSR	S	1.108	0.428

Dffa	DNA fragmentation factor subunit alpha	O54786	S217	AALSEELDAVDTGVGR	S4	1.021	0.935
Dgat1	Diacylglycerol O-acyltransferase 1	Q9Z2A7	S17	TGSRVSVQGGSGPKVEEDEVR	S3	0.969	0.745
Dgat1	Diacylglycerol O-acyltransferase 1	Q9Z2A7	S25	VSVQGGSGPKVEEDEVR	S7	1.041	0.426
Dgcr8	Microprocessor complex subunit DGCR8	Q9EQM6	S377	EQNCDLAPSGEVSPVKPLGR	S13	1.090	0.322
Dgcr8	Microprocessor complex subunit DGCR8	Q9EQM6	S377	DNEEREQNCDLAPSGEVSPVKPLGR	S18	1.054	0.315
Dgcr8	Microprocessor complex subunit DGCR8	Q9EQM6	S95	LLIDPNCSGHSPR	S11	0.942	0.339
Dgcr8	Microprocessor complex subunit DGCR8	Q9EQM6	S271,S275	YGGDSDHPSDGETSVQPMMTK	S5,S9	1.006	0.976
Dgcr8	Microprocessor complex subunit DGCR8	Q9EQM6	S271,S275	YGGDSDHPSDGETSVQPMMTK	S5,S9	0.964	0.409
Dgcr8	Microprocessor complex subunit DGCR8	Q9EQM6	S373,S377	EQNCDLAPSGEVSPVKPLGR	S9,S13	1.035	0.414
Dgkd	Diacylglycerol kinase	E9PUQ8	S733	AGMSGSLPGGSVISR	S6	0.954	0.383
Dgkd	Diacylglycerol kinase	E9PUQ8	S733	AGMSGSLPGGSVISR	S6	1.058	0.490
Dgkg	Diacylglycerol kinase gamma	Q91WG7	S107	EGASSSEPNVSDYNSDNAAK	S15	1.052	0.720
Dgkq	Diacylglycerol kinase theta	Q6P5E8	S17	TWPGSGSPR	S7	1.003	0.842
Dgkz	Diacylglycerol kinase	A2AHK0	Ambiguous	RRSTAPLHSDQQPVPEQLR	S/T	0.824	0.443
Dhx16	DEAH (Asp-Glu-Ala-His) box polypeptide 16	G3X8X0	S163	SPTEEKPASEDEWERTER	S9	1.180	0.097
Dhx16	DEAH (Asp-Glu-Ala-His) box polypeptide 16	G3X8X0	S155,S163	AGGSKSPTEEKPASEDEWERTER	S6,S14	0.913	0.200
Dhx16	DEAH (Asp-Glu-Ala-His) box polypeptide 16	G3X8X0	S163,S/T	AGGSKSPTEEKPASEDEWER	S14,S/T	0.999	0.990
Dhx29	ATP-dependent RNA helicase DHX29	Q6PGC1	S27	SAEAGAVGEAQSK	S1	1.040	0.340
Dhx29	ATP-dependent RNA helicase DHX29	Q6PGC1	S27	ARSAEAGAVGEAQSK	S3	1.052	0.414
Dhx29	ATP-dependent RNA helicase DHX29	Q6PGC1	S69	IYSFNSANDSGGSANLDK	S6	1.854	0.036
Dhx30	ATP-dependent RNA helicase DHX30	Q99PU8	S249	GGSFEMTDDDSAIR	S3	1.020	0.490
Dhx33	ATP-dependent RNA helicase DHX33	Q80VY9	T43	RQTPPLAQPSASPYR	T3	0.988	0.888
Dhx38	DEAH (Asp-Glu-Ala-His) box polypeptide 38	Q80X98	T117	VETPSHPGGVSEEFWER	T3	0.821	0.096
Dhx38	DEAH (Asp-Glu-Ala-His) box polypeptide 38	Q80X98	T266	SSDDTPLPTPSYK	T5	1.079	0.347
Dhx38	DEAH (Asp-Glu-Ala-His) box polypeptide 38	Q80X98	Ambiguous	SKSSDDTPLPTPSYK	S/T/Y	1.026	0.712
Dhx8	ATP-dependent RNA helicase DHX8	A2A4P0	S484	QSMDMSPIK	S6	0.964	0.831
Dhx8	ATP-dependent RNA helicase DHX8	A2A4P0	S484	QSMDMSPIK	S6	0.992	0.911
Dhx9	ATP-dependent RNA helicase A	A0A087WPL5	S136	AEENNSGVESSGYGSPGPTWDR	S15	1.004	0.938
Dhx9	ATP-dependent RNA helicase A	E9QNN1	S137	AEAENNSGVESSGYGSPGPTWDR	S16	1.070	0.221
Dhx9	ATP-dependent RNA helicase A	E9QNN1; A0A087WPL5	S324;S323	LAHFEPSQR	S7	1.027	0.756
Dhx9	ATP-dependent RNA helicase A	E9QNN1	S137,S/Y/T	AEAENNSGVESSGYGSPGPTWDR	S16,S/Y/T	1.420	0.113
Diaph1	Protein diaphanous homolog 1	E9PV41	S22	GRSPDELPATGGDGGK	S3	0.981	0.136
Dicer1	Endoribonuclease Dicer	F8VQ54	S1609	SLSGSCASPVGPR	S8	0.951	0.756
Dido1	Death-inducer obliterator 1	Q8C9B9	S1027	YLSVPPSPSISESR	S7	1.244	0.351
Dido1	Death-inducer obliterator 1	Q8C9B9	S1035	SPPEGDTTLFLSR	S1	1.035	0.859
Dido1	Death-inducer obliterator 1	Q8C9B9	S1336	ESVGSTLSPHQDSK	S8	1.043	0.189
Dido1	Death-inducer obliterator 1	Q8C9B9	S1840	GLSPSYLGGPR	S3	1.010	0.852
Dido1	Death-inducer obliterator 1	Q8C9B9	S657	LGPVSPAPSQPNSQIR	S5	1.031	0.678
Dido1	Death-inducer obliterator 1	Q8C9B9	S802,S806	NTTKPETIPDMEDSPPVSDSEEQQESVR	S14,S18	1.044	0.748
Dido1	Death-inducer obliterator 1	Q8C9B9	Ambiguous	ICTGQVPSSEDEPAPK	S/T	0.875	0.405
Dip2b	Disco-interacting protein 2 homolog B	Q3UH60	S152	STFVQSPADACTPPDTSSASEDEGSLRR	S25	1.026	0.918
Dip2b	Disco-interacting protein 2 homolog B	Q3UH60	S152	RSTFVQSPADACTPPDTSSASEDEGSLRR	S26	1.051	0.732
Dip2b	Disco-interacting protein 2 homolog B	Q3UH60	S256	GSNRSSLMDTADGVPVNSR	S6	0.983	0.768
Dip2b	Disco-interacting protein 2 homolog B	Q3UH60	S256	SSLMDTADGVPVNSR	S2	1.093	0.411
Dip2b	Disco-interacting protein 2 homolog B	Q3UH60	S53	LLSPYSPQTQETDSIGQK	S6	0.954	0.654
Dip2b	Disco-interacting protein 2 homolog B	Q3UH60	S9	GLEPSPAAVAALPPEVR	S5	1.065	0.768
Dip2b	Disco-interacting protein 2 homolog B	Q3UH60	T78	NQTPAPTAAQTSAPSK	T11	0.950	0.842
Dip2b	Disco-interacting protein 2 homolog B	Q3UH60	Ambiguous	GSNRSSLMDTADGVPVNSR	S/T	1.012	0.764
Dip2b	Disco-interacting protein 2 homolog B	Q3UH60	Ambiguous	GTSGSLADVFANTR	T/S	0.681	0.009
Dip2c	Disco-interacting protein 2 homolog C	E9PWR4	T67	RAPVTPSSASR	T5	1.198	0.124
Dip2c	Disco-interacting protein 2 homolog C	E9PWR4	T67	APVTPSSASR	T4	0.971	0.391
Dis3l2	DIS3-like exonuclease 2	Q8CI75	S12	LNLRSPGTPR	S5	1.036	0.435
Disc1	Disrupted in schizophrenia 1 homolog	D3YYC9	S484	RLSQELEEQEVLLR	S3	1.226	0.226
Dkc1	H/ACA ribonucleoprotein complex subunit DKC1	Q9ESX5	S481	TVLESGGETGDGDNDTTK	S5	1.027	0.339
Dkc1	H/ACA ribonucleoprotein complex subunit DKC1	Q9ESX5	S508	VVEEMSE	S6	1.120	0.138
Dkc1	H/ACA ribonucleoprotein complex subunit DKC1	Q9ESX5	S508	VVEEMSE	S6	1.025	0.244
Dkc1	H/ACA ribonucleoprotein complex subunit DKC1	Q9ESX5	S508	VKVVEEMSE	S8	0.969	0.259
Dkc1	H/ACA ribonucleoprotein complex subunit DKC1	Q9ESX5	S508	VKVVEEMSE	S8	0.964	0.398
Dkc1	H/ACA ribonucleoprotein complex subunit DKC1	Q9ESX5	S451,S453	RDSESESDETPTVPQLK	S3,S5	0.975	0.647
Dkc1	H/ACA ribonucleoprotein complex subunit DKC1	Q9ESX5	S481,T485	TVLESGGETGDGDNDTTK	S5,T9	1.070	0.290
Dkc1	H/ACA ribonucleoprotein complex subunit DKC1	Q9ESX5	Ambiguous	TVLESGGETGDGDNDTTKK	S/T	1.092	0.117
Dlg1	Disks large homolog 1	E9Q9H0	S575	EQMMNSSVSSGSGSLR	S14	0.923	0.078
Dlg1	Disks large homolog 1	E9Q9H0	S575	EQMMNSSVSSGSGSLR	S14	1.016	0.864
Dlg1	Disks large homolog 1	E9Q9H0	Ambiguous	EQMMNSSVSSGSGSLR	S	0.916	0.422
Dlg4	Disks large homolog 4	A0A338P6I6	S462	EQLMNSSLGSGTASLR	S14	0.868	0.012
Dlgap4	Disks large-associated protein 4	B1AZP2	S716	DTDSDTQDANDSSCK	S4	0.898	0.414
Dlgap4	Disks large-associated protein 4	B1AZP2	S716	RDTDSDTQDANDSSCK	S5	1.119	0.101
Dlgap4	Disks large-associated protein 4	B1AZP2	S744	SLPDCTSHPNSISIDAGPR	S13	0.938	0.300
Dlgap4	Disks large-associated protein 4	B1AZP2	S973	QNSATESADSIEIYVPEAQTR	S3	1.004	0.970
Dlgap4	Disks large-associated protein 4	B1AZP2	Ambiguous	SEVTSQSGLSNSSDSLDSSTRPPSVTR	S/T	0.832	0.014
Dlgap5	Disks large-associated protein 5	A0A0R4J0G7	S328	SYQVAPLSPR	S8	1.052	0.429
Dmtn	Dematin	Q9WV69	S226	KGAEEEEEEEDDDSEEEIK	S14	0.943	0.613
Dmtn	Dematin	Q9WV69	Ambiguous	STSPPPSPEVWAESR	T/S	1.105	0.573
Dmwd	Dystrophia myotonica WD repeat-containing protein	Q08274	S537	YHSLGNISR	S3	0.999	0.993
Dmwd	Dystrophia myotonica WD repeat-containing protein	Q08274	S653	SVVEGISSQPGSSPSGTVV	S7	1.155	0.508
Dmwd	Dystrophia myotonica WD repeat-containing protein	Q08274	Ambiguous	SNSLPHPAGGGK	S	0.919	0.068
Dmwd	Dystrophia myotonica WD repeat-containing protein	Q08274	Ambiguous	TRTLPGTPGATPPASGSSR	T/S	0.990	0.916
Dmxl1	DmX-like protein 1	F8WGX5	S1920	CCAADWSPSLTNGLESSSEGSSER	S7	1.023	0.804
Dmxl1	DmX-like protein 1	F8WGX5	S2404	VSARESPVSSSSGNQEPPAVK	S6	0.968	0.598
Dmxl1	DmX-like protein 1	F8WGX5	S916	ISEASWLPEEHYSSSPEK	S15	0.894	0.153
Dmxl2	DmX-like protein 2	B0V2P5	S1399	TISVSGSTAK	S3	1.156	0.022
Dmxl2	DmX-like protein 2	B0V2P5	S2395	RQSESIAAPPVASEDMDK	S3	1.064	0.173
Dnajb4	DnaJ homolog subfamily B member 4	Q9D832	S248	DGSNIVYTAK	S3	0.919	0.665
Dnajc2	DnaJ homolog subfamily C member 2	P54103	Ambiguous	NASTSFQELEDK	S/T	0.925	0.039
Dnajc21	DnaJ homolog subfamily C member 21	E9Q8D0	S283	EFGDGSDENEVEDQEPR	S6	1.082	0.465
Dnajc21	DnaJ homolog subfamily C member 21	E9Q8D0	S283	YEKEFGDGSDENEVEDQEPR	S9	0.964	0.612
Dnajc8	DnaJ homolog subfamily C member 8	Q6NZB0	S35	RDSVLTSK	S3	1.005	0.873
Dnm1l	Dynamin-1-like protein	Q8K1M6	S535	ELPSAGSRDK	S7	0.980	0.675
Dnm2	Dynamin-2	F8WIV5	T353	IEGSGDQVDTLELSGGAR	T10	1.184	0.380
Dnmbp	Dynamin-binding protein	A0A0R4J055	S1472	GSPHSEVVGYSVPGQNDQGSDSIK	S2	1.009	0.925
Dnmt1	DNA (cytosine-5)-methyltransferase	J3QNW0	S31	SDSDTLFETSPSSVATR	S10	0.975	0.901
Dnmt1	DNA (cytosine-5)-methyltransferase	J3QNW0; P13864	S598;S717	EADDDEEADDDVSEMPSPK	S17	0.991	0.919
Dnmt1	DNA (cytosine-5)-methyltransferase	J3QNW0; P13864	S598;S717	EADDDEEADDDVSEMPSPK	S17	1.133	0.122
Dnmt1	DNA (cytosine-5)-methyltransferase	J3QNW0; P13864	T189;T308	EAEPEQVAPETPEDRDEDEREEK	T11	1.065	0.563
Dnmt1	DNA (cytosine-5)-methyltransferase 1	P13864	S146	SDSDTLSVETSPSSVATRR	S7	0.902	0.274
Dnmt1	DNA (cytosine-5)-methyltransferase 1	P13864	S140,S150	SKSDSDTLSVETSPSSVATR	S3,S13	1.187	0.041
Dnmt1	DNA (cytosine-5)-methyltransferase 1	P13864	S150,S/T	RSKSDSDTLSVETSPSSVATR	S14,S/T	1.013	0.851
Dnmt1	DNA (cytosine-5)-methyltransferase 1	P13864	Ambiguous	SDSDTLSVETSPSSVATR	S/T	0.889	0.109
Dnmt1	DNA (cytosine-5)-methyltransferase 1	P13864	Ambiguous	SKSDSDTLSVETSPSSVATR	S/T	1.197	0.097
Dnmt3a	DNA (cytosine-5)-methyltransferase 3A	O88508	S102	RSEPQPEEGSPAAGQK	S10	1.109	0.598
Dnmt3a	DNA (cytosine-5)-methyltransferase 3A	O88508	S102	SEPQPEEGSPAAGQK	S9	0.998	0.947
Dnmt3a	DNA (cytosine-5)-methyltransferase 3A	O88508	T120	GGAPAEGEGTETPPEASR	T12	1.022	0.575
Dnttip1	Deoxynucleotidyltransferase terminal-interacting protein 1	Q99LB0	S160	QAEEESHRGSPIPK	S10	1.002	0.988
Dnttip2	Deoxynucleotidyltransferase terminal-interacting protein 2	Q8R2M2	S248	NMPNVSDSETYNSDFDDSSPR	S6	0.948	0.542
Dnttip2	Deoxynucleotidyltransferase terminal-interacting protein 2	Q8R2M2	S255	NMPNVSDSETYNSDFDDSSPR	S13	1.067	0.236
Dnttip2	Deoxynucleotidyltransferase terminal-interacting protein 2	Q8R2M2	S248,S255	NMPNVSDSETYNSDFDDSSPR	S6,S13	1.045	0.625
Dnttip2	Deoxynucleotidyltransferase terminal-interacting protein 2	Q8R2M2	Ambiguous	ESGSDGSTAESQPAEK	S/T	0.898	0.339
Dock1	Dedicator of cytokinesis protein 1	Q8BUR4	S1743	SQVINVIGNER	S1	1.068	0.335
Dock1	Dedicator of cytokinesis protein 1	Q8BUR4	S1858	KQTSVDSGIVQ	S4	1.148	0.156
Dock1	Dedicator of cytokinesis protein 1	Q8BUR4	T1767	FSVSPASPSSQQTPPPVTPR	T13	0.993	0.963
Dock10	Dedicator of cytokinesis protein 10	E9QM99	S12	SLLRPGQAAELR	S1	1.039	0.368
Dock10	Dedicator of cytokinesis protein 10	E9QM99	S1232	DLYPFTVNTSNQGSR	S10	0.887	0.487
Dock10	Dedicator of cytokinesis protein 10	E9QM99	S1292	ASLASLDSNPSTTEK	S5	0.952	0.200
Dock10	Dedicator of cytokinesis protein 10	E9QM99	S877	EKDMSQSPTSSFVR	S7	1.017	0.653
Dock10	Dedicator of cytokinesis protein 10	E9QM99	S877	DMSQSPTSSFVR	S5	1.024	0.459
Dock10	Dedicator of cytokinesis protein 10	E9QM99	T1125	SANIPDPLTPSESIR	T9	1.022	0.915
Dock10	Dedicator of cytokinesis protein 10	E9QM99	T1406	FVQSTQNNGTLK	T10	1.011	0.867
Dock10	Dedicator of cytokinesis protein 10	E9QM99	T1440	SQTLPIIR	T3	1.025	0.647
Dock10	Dedicator of cytokinesis protein 10	E9QM99	T196	GNFNSTVNNTVTVR	T6	1.092	0.178
Dock10	Dedicator of cytokinesis protein 10	E9QM99	S1292,S1295	ASLASLDSNPSTTEK	S5,S8	0.999	0.983
Dock10	Dedicator of cytokinesis protein 10	E9QM99	Ambiguous	DMSQSPTSSFVR	S/T	1.038	0.169
Dock10	Dedicator of cytokinesis protein 10	E9QM99	Ambiguous	EKDMSQSPTSSFVR	S/T	0.999	0.987
Dock10	Dedicator of cytokinesis protein 10	E9QM99	Ambiguous	HATSVDTSFSK	T/S	0.958	0.562
Dock11	Dedicator of cytokinesis protein 11	A2AF47	S1208	DEFPCGFVSPTNR	S9	1.468	0.292
Dock11	Dedicator of cytokinesis protein 11	A2AF47	S1237	REDSRGSLIPEGATGFPDPGSTSENTR	S4	1.153	0.258
Dock11	Dedicator of cytokinesis protein 11	A2AF47	S1240	EDSRGSLIPEGATGFPDPGSTSENTR	S6	0.984	0.716
Dock11	Dedicator of cytokinesis protein 11	A2AF47	S306	AENIMASLER	S7	0.992	0.950
Dock2	Dedicator of cytokinesis protein 2	Q8C3J5	S1683	TPKVEEEPISPGSTLPEVK	S10	1.059	0.597
Dock2	Dedicator of cytokinesis protein 2	Q8C3J5	S1683	VEEEPISPGSTLPEVK	S7	1.143	0.013
Dock2	Dedicator of cytokinesis protein 2	Q8C3J5	S1703	SSVVFADEK	S1	1.070	0.026
Dock2	Dedicator of cytokinesis protein 2	Q8C3J5	S1704	RSSVVFADEK	S3	1.012	0.664
Dock2	Dedicator of cytokinesis protein 2	Q8C3J5	S1729	QEFMSDTNLSEHAAIPAR	S5	1.042	0.608
Dock2	Dedicator of cytokinesis protein 2	Q8C3J5	S1729	QEFMSDTNLSEHAAIPAR	S5	0.945	0.626
Dock2	Dedicator of cytokinesis protein 2	Q8C3J5	S1729	KQEFMSDTNLSEHAAIPAR	S6	1.001	0.952
Dock2	Dedicator of cytokinesis protein 2	Q8C3J5	S1729	KQEFMSDTNLSEHAAIPAR	S6	0.953	0.694
Dock2	Dedicator of cytokinesis protein 2	Q8C3J5	S587	SSSSVGGLSVSSR	S3	0.944	0.291
Dock2	Dedicator of cytokinesis protein 2	Q8C3J5	S583,S/T	GATLSRSSSSVGGLSVSSR	S5,S/T	1.013	0.741
Dock4	Dedicator of cytokinesis protein 4	P59764	S1623	VCRNSAPASMSPDGTR	S5	1.036	0.771

Dock4	Dedicator of cytokinesis protein 4	P59764	S1643	RSPLSYPAVNR	S5	1.083	0.450
Dock4	Dedicator of cytokinesis protein 4	P59764	S1709	ASPLLSDK	S2	0.991	0.880
Dock4	Dedicator of cytokinesis protein 4	P59764	S1726	ENSCLSPR	S6	1.074	0.155
Dock4	Dedicator of cytokinesis protein 4	P59764	S1733	DRPCSAIYPTPVEPSQR	S5	0.896	0.270
Dock4	Dedicator of cytokinesis protein 4	P59764	T1911	ESKTPPPYSVYER	T4	1.010	0.931
Dock4	Dedicator of cytokinesis protein 4	P59764	Ambiguous	NSAPASMSPDGTR	S/T	1.039	0.854
Dock5	Dedicator of cytokinesis protein 5	B2RY04	S1765	SLQLVDSR	S1	0.930	0.096
Dock5	Dedicator of cytokinesis protein 5	B2RY04	S1832	NSAEIAPPLPVRR	S2	1.084	0.203
Dock5	Dedicator of cytokinesis protein 5	B2RY04	Ambiguous	SKPYESSQRNSAEIAPPLPVR	S/Y	1.012	0.906
Dock6	Dedicator of cytokinesis protein 6	A0A1L1SQR4	S178	TGPEDVDDPQHCSGSPEDTPR	S15	1.137	0.265
Dock6	Dedicator of cytokinesis protein 6	A0A1L1SQR4	S870	GSGRPASLYLAR	S7	0.974	0.647
Dock6	Dedicator of cytokinesis protein 6	A0A1L1SQR4	S949	NSSRASSYLEASSSALPAPQSR	S6	0.934	0.476
Dock7	Dedicator of cytokinesis protein 7	A2A9M4	S1420	GQLERSPSGSAFGSQENLR	S6	0.989	0.584
Dock7	Dedicator of cytokinesis protein 7	A2A9M4	S1428	SPSGSAFGSQENLR	S9	1.046	0.132
Dock7	Dedicator of cytokinesis protein 7	A2A9M4	S180	SMSIDDTPR	S1	0.913	0.050
Dock7	Dedicator of cytokinesis protein 7	A2A9M4	S182	SMSIDDTPR	S3	1.016	0.534
Dock7	Dedicator of cytokinesis protein 7	A2A9M4	S30	QISGQYSGSPQLLK	S9	0.872	0.053
Dock7	Dedicator of cytokinesis protein 7	A2A9M4	S439	RNSSLVGR	S3	1.087	0.505
Dock7	Dedicator of cytokinesis protein 7	A2A9M4	S888	SAVRPASLNLNR	S7	1.085	0.161
Dock7	Dedicator of cytokinesis protein 7	A2A9M4	S896	SRSLSNSNPDISGTPTSPDDEVR	S3	1.074	0.168
Dock7	Dedicator of cytokinesis protein 7	A2A9M4	S900	SLSNSNPDISGTPTSPDDEVR	S5	0.873	0.001
Dock7	Dedicator of cytokinesis protein 7	A2A9M4	S945	AAPWGSNPSPSAESTQAMDR	S9	1.080	0.415
Dock7	Dedicator of cytokinesis protein 7	A2A9M4	S1420,S1428	GQLERSPSGSAFGSQENLR	S6,S14	0.968	0.202
Dock7	Dedicator of cytokinesis protein 7	A2A9M4	S1422,S1428	SPSGSAFGSQENLR	S3,S9	0.969	0.830
Dock7	Dedicator of cytokinesis protein 7	A2A9M4	S896,S898	SLSNSNPDISGTPTSPDDEVR	S1,S3	0.948	0.445
Dock7	Dedicator of cytokinesis protein 7	A2A9M4	S896,S900	SRSLSNSNPDISGTPTSPDDEVR	S3,S7	1.039	0.260
Dock7	Dedicator of cytokinesis protein 7	A2A9M4	Ambiguous	AAPWGSNPSPSAESTQAMDR	S/T	1.017	0.950
Dock8	Dedicator of cytokinesis protein 8	Q8C147	S20	INRYSSSEIR	S5	0.933	0.522
Dock8	Dedicator of cytokinesis protein 8	Q8C147	S2097	CETQLSQGS	S6	1.202	0.011
Dock8	Dedicator of cytokinesis protein 8	Q8C147	S2100	KCETQLSQGS	S7	1.319	0.008
Dock8	Dedicator of cytokinesis protein 8	Q8C147	S433	ESTDVEPGVGRNSVGEK	S13	1.099	0.026
Dock8	Dedicator of cytokinesis protein 8	Q8C147	S433	ESTDVEPGVGRNSVGEKR	S13	0.973	0.590
Dock8	Dedicator of cytokinesis protein 8	Q8C147	S905	VMSSSNPDLTGSHCAADEEVK	S5	0.999	0.980
Dock8	Dedicator of cytokinesis protein 8	Q8C147	S905	VMSSSNPDLTGSHCAADEEVK	S5	1.003	0.944
Dock8	Dedicator of cytokinesis protein 8	Q8C147	S937	MSYYCSGNSDAPGSTAAPRPVSK	S2	1.039	0.393
Dock8	Dedicator of cytokinesis protein 8	Q8C147	Ambiguous	GPLTSCDFDLR	S/T	0.883	0.305
Dock8	Dedicator of cytokinesis protein 8	Q8C147	Ambiguous	DSFHRSSFR	S	0.894	0.390
Dock8	Dedicator of cytokinesis protein 8	Q8C147	Ambiguous	QTFESETLECSEPDTQTGPR	T/S	0.895	0.784
Dock8	Dedicator of cytokinesis protein 8	Q8C147	Ambiguous	TLSLEENGVGSNFK	T/S	1.311	0.145
Dok1	Docking protein 1	P97465	S269	VGQAQDILRTDSHDGETEGK	S12	1.517	0.003
Dok1	Docking protein 1	P97465	S269	TDSHDGETEGK	S3	1.250	0.014
Dok1	Docking protein 1	P97465	S355	TKLTDSKEDPIYDEPEGLAPAPPR	S6	0.704	0.152
Dok1	Docking protein 1	P97465	S355	LTDSKEDPIYDEPEGLAPAPPR	S4	1.074	0.577
Dok1	Docking protein 1	P97465	S445	GFSSDTALYSQVQK	S4	0.947	0.225
Dok1	Docking protein 1	P97465	Y361	EDPIYDEPEGLAPAPPR	Y5	0.853	0.059
Dok1	Docking protein 1	P97465	Y376	GLYDLPQEPR	Y3	0.928	0.161
Dok1	Docking protein 1	P97465	Ambiguous	LKEEGYELPYNPATDDYAVPPPR	T/Y	0.777	0.461
Dok2	Docking protein 2	O70469	S124	GSPGLEEK	S2	0.984	0.702
Dok2	Docking protein 2	O70469	S124	KGSPGLEEK	S3	1.004	0.906
Dok2	Docking protein 2	O70469	S133	SGSPCMEENELYSSSTTGLCK	S3	0.988	0.787
Dok2	Docking protein 2	O70469	S133	SGSPCMEENELYSSSTTGLCK	S3	0.994	0.956
Dok2	Docking protein 2	O70469	S221	RCLSGEGNFEFETR	S4	1.024	0.795
Dok2	Docking protein 2	O70469	Ambiguous	EQATADGGPSSLQQDSSVPDWPQATEYDNVILK	S/Y/T	1.211	0.646
Dok3	Docking protein 3	Q9QZK7	S274	ALSLPSLEPPGELR	S3	1.050	0.137
Dop1b	Protein dopey-2	Q3UHQ6	S597	SEDSGIGLSASSPELSEHLR	S12	1.031	0.382
Dop1b	Protein dopey-2	Q3UHQ6	S552,S/Y	VQMPPSYLDLEPSSGNSSPVKGR	S14,S/Y	0.970	0.729
Dop1b	Protein dopey-2	Q3UHQ6	S719,S	SSESLSSVPSSPHRK	S11,S	1.038	0.142
Dop1b	Protein dopey-2	Q3UHQ6	Ambiguous	VQMPPSYLDLEPSSGNSSPVK	S/Y	0.782	0.087
Dop1b	Protein dopey-2	Q3UHQ6	Ambiguous	SSESLSSVPSSPHRK	S	1.045	0.801
Dopey1	Protein dopey-1	H7BWZ9	S1235	NSSSPCISGTAQTLNDSSVPSETK	S3	1.110	0.164
Dot1l	Histone-lysine N-methyltransferase, H3 lysine-79 specific	Q6XZL8	S1003	NSGPASPAHQLTASPR	S6	1.080	0.454
Dot1l	Histone-lysine N-methyltransferase, H3 lysine-79 specific	Q6XZL8	S1037	GELPSDPAFSDPESEAK	S10	0.909	0.518
Dot1l	Histone-lysine N-methyltransferase, H3 lysine-79 specific	Q6XZL8	S1105	ASAGTPSLSTGVSPK	S13	1.087	0.237
Dot1l	Histone-lysine N-methyltransferase, H3 lysine-79 specific	Q6XZL8	S373	AAATEAPADSGAEEEK	S10	1.052	0.465
Dot1l	Histone-lysine N-methyltransferase, H3 lysine-79 specific	Q6XZL8	S814	ESSLPYQSPGLSNSMK	S8	0.968	0.719
Dot1l	Histone-lysine N-methyltransferase, H3 lysine-79 specific	Q6XZL8	S900,S/T	ARSTPSPVPQPR	S6,S/T	0.869	0.482
Dot1l	Histone-lysine N-methyltransferase, H3 lysine-79 specific	Q6XZL8	Ambiguous	SQSTLDLLHSPPPAPPSASPQDAYR	S/T/Y	1.091	0.689
Dot1l	Histone-lysine N-methyltransferase, H3 lysine-79 specific	Q6XZL8	Ambiguous	STPSPVPQPR	T/S	0.996	0.913
Dpf2	Zinc finger protein ubi-d4	D3Z5N6	S142	GAPDPRVDDDSLGEFPVSNSR	S11	0.948	0.650
Dpf2	Zinc finger protein ubi-d4	D3Z5N6	S142	VDDDSLGEFPVSNSR	S5	1.065	0.693
Dpf2	Zinc finger protein ubi-d4	D3Z5N6	Ambiguous	ASIYQNQNSS	S/Y	0.919	0.156
Dph1	2-(3-amino-3-carboxypropyl)histidine synthase subunit 1	Q5NCQ5	S418	GGSPAPACESCNCADQK	S3	0.968	0.648
Dph2	2-(3-amino-3-carboxypropyl)histidine synthase subunit 2	Q9CR25	Ambiguous	RGIAIAYEDEGSS	S/Y	1.002	0.985
Dph2	2-(3-amino-3-carboxypropyl)histidine synthase subunit 2	Q9CR25	Ambiguous	GIAIAYEDEGSS	S/Y	1.001	0.982
Dpm1	Dolichol-phosphate mannosyltransferase subunit 1	O70152	S13	SLAASPRPPQGR	S5	0.995	0.905
Dpp8	Dipeptidyl peptidase 8	Q80YA7	S489	SSGGLPAPSDFK	S2	1.068	0.735
Dpy19l1	Probable C-mannosyltransferase DPY19L1	A6X919	S22	SSPPPLSGASEVDAGELGSER	S1	0.974	0.606
Dpy19l1	Probable C-mannosyltransferase DPY19L1	A6X919	S46	TPPSPGRR	S4	0.980	0.679
Dpy19l1	Probable C-mannosyltransferase DPY19L1	A6X919	Ambiguous	SARSSPPPLSGASEVDAGELGSER	S	1.163	0.284
Dpysl2	Dihydropyrimidinase-related protein 2	O08553	T509	GLYDGPVCEVSVTPK	T13	0.873	0.048
Dpysl2	Dihydropyrimidinase-related protein 2	O08553	T514	TVTPASSAK	T3	0.962	0.203
Dpysl2	Dihydropyrimidinase-related protein 2	O08553	T514,S518	TVTPASSAKTSPAK	T3,S7	0.870	0.156
Dpysl2	Dihydropyrimidinase-related protein 2	O08553	T514,S517,T/S	TVTPASSAKTSPAK	T3,S6,S/T	0.960	0.213
Dpysl3	Dihydropyrimidinase-related protein 3	E9PWE8	S101	EESREPAPESPKPAGVEIR	S10	1.096	0.259
Dpysl3	Dihydropyrimidinase-related protein 3	E9PWE8	S101	EPAPESPKPAGVEIR	S6	1.222	0.037
Dpysl3	Dihydropyrimidinase-related protein 3	E9PWE8	S635	GSPTRPNPPVR	S2	1.080	0.480
Dpysl3	Dihydropyrimidinase-related protein 3	E9PWE8	T622	GMYDGPVFDLTTTPK	T13	0.968	0.769
Dse	Dermatan-sulfate epimerase	Q8BLI4	S797	IFAISQQR	S5	1.114	0.387
Dse	Dermatan-sulfate epimerase	Q8BLI4	T786	FSDKRQTEEAIDR	T7	0.993	0.930
Dsn1	Kinetochore-associated protein DSN1 homolog	Q9CYC5	Ambiguous	LPLQSSPSTQ	S/T	0.959	0.724
Dst	Dystonin	S4R1P5	S157	CSMSSSADFSDEDDFSQK	S10	0.927	0.389
Dst	Dystonin	S4R1P5	Ambiguous	LLDPEDVDVSSPDEK	S	1.121	0.466
Dstn	Destrin	Q9R0P5	S88	YALYDASFETK	S7	1.104	0.170
Dstyk	Dual serine/threonine and tyrosine protein kinase	Q6XUX1	S926	GLDDST	S5	1.092	0.376
Dstyk	Dual serine/threonine and tyrosine protein kinase	Q6XUX1	Ambiguous	CSSEQPNRGLDDST	S/T	0.989	0.847
Dtd1	D-aminoacyl-tRNA deacylase 1	Q9DD18	S197	SASSGAEGDVSSEREP	S4	0.998	0.984
Dtnbp1	Dysbindin	K3W4R3	S340	STPGVSDDSDQCDSTQDI	S6	0.913	0.522
Dtnbp1	Dysbindin	K3W4R3	S340,S343	STPGVSDDSDQCDSTQDI	S6,S9	0.978	0.694
Dtx2	Probable E3 ubiquitin-protein ligase DTX2	Q8R3P2	Ambiguous	YRHSMTNLPAYPAPQAPHR	T/S/Y	0.992	0.872
Dtx4	E3 ubiquitin-protein ligase DTX4	Q6PDK8	S273	QVSNAPAGATVGSPASPQGSNR	S16	1.066	0.499
Dtx4	E3 ubiquitin-protein ligase DTX4	Q6PDK8	S273	QVSNAPAGATVGSPASPQGSNRK	S16	0.979	0.832
Dusp9	Dual specificity protein phosphatase	Q7TNL7	Ambiguous	GSGGPESTVSDPPSFFTTPTSDGVFELDPT	S/T	1.401	0.227
Dvl1	Segment polarity protein dishevelled homolog DVL-1	P51141	Ambiguous	ELAAVPPELTGSR	T/S	1.002	0.987
Dync1h1	Cytoplasmic dynein 1 heavy chain 1	Q9JHU4	S2382	LRSIPLDEGEDEAQR	S3	1.012	0.920
Dync1h1	Cytoplasmic dynein 1 heavy chain 1	Q9JHU4	S4366	TDSTSDGRPAWMR	S3	1.052	0.069
Dync1h1	Cytoplasmic dynein 1 heavy chain 1	Q9JHU4	S4366	TDSTSDGRPAWMR	S3	1.028	0.483
Dync1i2	Cytoplasmic dynein 1 intermediate chain 2	A2BFF9	S51	EAAVSVQEESDLEKK	S10	0.987	0.861
Dync1i2	Cytoplasmic dynein 1 intermediate chain 2	A2BFF9	Ambiguous	SVSTPSEAGSQDSGDGAVGSR	T/S	1.025	0.696
Dync1li1	Cytoplasmic dynein 1 light intermediate chain 1	Q8R1Q8	S207	DFQEYVEPGEDFPASPQR	S15	1.078	0.114
Dync1li1	Cytoplasmic dynein 1 light intermediate chain 1	Q8R1Q8	S207	DFQEYVEPGEDFPASPQRR	S15	1.000	1.000
Dync1li1	Cytoplasmic dynein 1 light intermediate chain 1	Q8R1Q8	S398	QPPTAAGRPVDASPR	S13	0.876	0.262
Dync1li1	Cytoplasmic dynein 1 light intermediate chain 1	Q8R1Q8	S510	KPASVSPTTPTSPTEGEAS	S6	1.026	0.457
Dync1li1	Cytoplasmic dynein 1 light intermediate chain 1	Q8R1Q8	S523,S/T	KPASVSPTTPTSPTEGEAS	S19,S/T	1.077	0.238
Dync1li1	Cytoplasmic dynein 1 light intermediate chain 1	Q8R1Q8	Ambiguous	SVSSNVASVSPIPAGSK	S	1.072	0.764
Dync1li2	Cytoplasmic dynein 1 light intermediate chain 2	Q6PDL0	S194	EFQDYIEPEEGCQGSPQR	S15	1.110	0.003
Dync1li2	Cytoplasmic dynein 1 light intermediate chain 2	Q6PDL0	S194	EFQDYIEPEEGCQGSPQRR	S15	1.012	0.667
Dync1li2	Cytoplasmic dynein 1 light intermediate chain 2	Q6PDL0	S383	TSESPARGPSGSPR	S4	1.003	0.965
Dync1li2	Cytoplasmic dynein 1 light intermediate chain 2	Q6PDL0	S407	GGPASVPSASPGTSVK	S10	0.900	0.135
Dync1li2	Cytoplasmic dynein 1 light intermediate chain 2	Q6PDL0	S446	TGSPGSPSAGGVQSTAK	S6	0.951	0.390
Dyrk1a	Dual specificity tyrosine-phosphorylation-regulated kinase 1A	Q61214	S748	QGADREESPMTGVCVQQSPVASS	S8	1.000	0.994
Dyrk1a	Dual specificity tyrosine-phosphorylation-regulated kinase 1A	Q61214	Y321	IYQYIQSR	Y4	1.036	0.752
Dyrk1a	Dual specificity tyrosine-phosphorylation-regulated kinase 1A	Q61214	S748,S/T	QGADREESPMTGVCVQQSPVASS	S8,S/T	1.073	0.489
Dyrk1a	Dual specificity tyrosine-phosphorylation-regulated kinase 1A	Q61214	S748,S758	QGADREESPMTGVCVQQSPVASS	S8,S18	0.892	0.485
Dyrk1a	Dual specificity tyrosine-phosphorylation-regulated kinase 1A	Q61214	Ambiguous	QGADREESPMTGVCVQQSPVASS	S/T	1.029	0.674
Dyrk2	Dual specificity tyrosine-phosphorylation-regulated kinase 2	Q5U4C9	S30	QLQASPGIGAGAPR	S5	1.122	0.056
Dyrk2	Dual specificity tyrosine-phosphorylation-regulated kinase 2	Q5U4C9	Y380	VYTYIQSR	Y4	0.998	0.951
E2f6	Transcription factor E2F6	O54917	S254	TNDGIGASPSK	S8	1.056	0.033
E2f8	Transcription factor E2F8	Q58FA4	S102	DCLHEHLSGDEFEK	S8	1.041	0.286
E2f8	Transcription factor E2F8	Q58FA4	S416,S	ISSAPSSPVK	S7,S	1.141	0.315
E2f8	Transcription factor E2F8	Q58FA4	Ambiguous	AESSQNSPPVPNK	S	1.100	0.386
E4f1	Transcription factor E4F1	Q8CCE9	S352	ALTPESPDSEELPCSSENSR	S6	1.167	0.279
Eaf1	ELL-associated factor 1	Q9D4C5	S165	DNPSPEPQLDDIKR	S4	0.982	0.716
Eaf1	ELL-associated factor 1	Q9D4C5	S165,T/S	TSPLKDNPSPEPQLDDIK	S9,S/T	1.091	0.583
Eaf1	ELL-associated factor 1	Q9D4C5	Ambiguous	NDLQLSESGSDSDD	S	1.169	0.080
Ecd	Protein ecdysoneless homolog	Q9CS74	S640	LPDNADHNPQVSQ	S12	1.043	0.635
Ecm1	Extracellular matrix protein 1	Q61508	S556	GTDANPAPGSKEE	S10	1.054	0.724
Edc3	Enhancer of mRNA-decapping protein 3	Q8K2D3	S131	SQDVAISPQQQQCSK	S7	0.985	0.690
Edc3	Enhancer of mRNA-decapping protein 3	Q8K2D3	S140	SQDVAISPQQQQCSKSYVDR	S16	1.146	0.310

Edc3	Enhancer of mRNA-decapping protein 3	Q8K2D3	S131,S140	SQDVAISPQQQQCSKSYVDR	S7,S16	1.108	0.141
Edc4	Enhancer of mRNA decapping 4, isoform CRA_b	G5E896	S796	QGPELGSQLGLDGGPGDGDR	S7	0.950	0.606
Edc4	Enhancer of mRNA decapping 4, isoform CRA_b	G5E896	S849	ETCSTLTESPR	S9	0.980	0.764
Edc4	Enhancer of mRNA decapping 4, isoform CRA_b	G5E896	S884	DSQDTSAEQSDHDDEVASLASASGGFGSK	S10	0.998	0.965
Eea1	Early endosome antigen 1	Q8BL66	S52	SLGSADELFK	S4	0.920	0.347
Eea1	Early endosome antigen 1	Q8BL66	S760	QLSTDLELR	S3	1.099	0.380
Eef1b	Elongation factor 1-beta	O70251	S106	DDDDIDLFGSDDEEESEEAK	S10	1.013	0.829
Eef1b	Elongation factor 1-beta	O70251	S128	LAQYESK	S6	1.011	0.587
Eef1b	Elongation factor 1-beta	O70251	Ambiguous	DDDDIDLFGSDDEEESEEAKK	S	1.150	0.245
Eef1d	Elongation factor 1-delta (Fragment)	F6ZFU0	S335	QSSGPGASSGPGGDHSELIVR	S2	0.897	0.203
Eef1d	Elongation factor 1-delta (Fragment)	F6ZFU0	S404	ATAPQTQHVSPMR	S10	0.966	0.520
Eef1d	Elongation factor 1-delta (Fragment)	F6ZFU0	S404	ATAPQTQHVSPMR	S10	1.003	0.892
Eef1d	Elongation factor 1-delta (Fragment)	F6ZFU0	S433	GATPAEDDEDKDIDLFGSDEEEEDK	S18	1.006	0.972
Eef1d	Elongation factor 1-delta (Fragment)	F6ZFU0	S433	GATPAEDDEDKDIDLFGSDEEEEDKEAAR	S18	0.998	0.980
Eef1d	Elongation factor 1-delta (Fragment)	F6ZFU0	T356	ITSLEVENQNLR	T2	1.023	0.869
Eef2	Elongation factor 2	P58252	S584	ETVSEESNVLCLSK	S4	0.866	0.364
Eef2	Elongation factor 2	P58252	T57	AGETRFTDTR	T7	1.065	0.337
Eef2k	Eukaryotic elongation factor 2 kinase	O08796	S394,S/T	LSENSGDENMSDVTFDSLPSSPSSATPHSQK	S20,S/T	1.209	0.561
Eef2k	Eukaryotic elongation factor 2 kinase	O08796	Ambiguous	LSENSGDENMSDVTFDSLPSSPSSATPHSQK	S/T	1.039	0.777
Eepd1	Endonuclease/exonuclease/phosphatase family domain-containing protein 1	Q3TGW2	S173	SVEDLVR	S1	1.079	0.503
Eepd1	Endonuclease/exonuclease/phosphatase family domain-containing protein 1	Q3TGW2	S173	EHGPFRSVEDLVR	S7	0.991	0.845
Eepd1	Endonuclease/exonuclease/phosphatase family domain-containing protein 1	Q3TGW2	S173	REHGPFRSVEDLVR	S8	1.007	0.955
Eepd1	Endonuclease/exonuclease/phosphatase family domain-containing protein 1	Q3TGW2	Ambiguous	DIPGRESSPSNGHAK	S	1.089	0.359
Efcc1	EF-hand and coiled-coil domain-containing protein 1	A0A0A0MQ84	S317	SEGPQLLTPQQASPRPGNR	S13	0.960	0.596
Efhd2	EF-hand domain-containing protein D2	Q8C845	S74	ADLNQGIGEPQSPSRR	S12	0.998	0.958
Efhd2	EF-hand domain-containing protein D2	Q8C845	S74	ADLNQGIGEPQSPSR	S12	1.014	0.719
Efhd2	EF-hand domain-containing protein D2	Q8C845	S74	RADLNQGIGEPQSPSR	S13	1.034	0.266
Efhd2	EF-hand domain-containing protein D2	Q8C845	S74	RADLNQGIGEPQSPSRR	S13	1.021	0.525
Efr3a	Protein EFR3 homolog A	A0A1D5RLL3	S719	LTVPYVPQVTDEDRLSR	S16	1.002	0.983
Efr3a	Protein EFR3 homolog A	A0A1D5RLL3	S719	LTVPYVPQVTDEDRLSRR	S16	0.924	0.514
Efr3b	Protein EFR3 homolog B	Q6ZQ18	S216	SPSPLQAPEK	S3	0.977	0.526
Egln1	Egl nine homolog 1	Q91YE3	S129	SGPGPAEPGSEDPPLSRSPGPER	S16	0.954	0.436
Ehbp1l1	EH domain-binding protein 1-like protein 1	E9QP49	S1382	VASRDTDLSCSSKDGEAEVAQEAIPQEAPTDGPR	S3	0.932	0.578
Ehbp1l1	EH domain-binding protein 1-like protein 1	E9QP49	S1382	VASRDTDLSCSSK	S3	1.016	0.840
Ehbp1l1	EH domain-binding protein 1-like protein 1	E9QP49	S1446	SSVNGEAGPVPPPR	S2	1.015	0.478
Ehbp1l1	EH domain-binding protein 1-like protein 1	E9QP49	S1446	RSSVNGEAGPVPPPR	S3	1.087	0.071
Ehbp1l1	EH domain-binding protein 1-like protein 1	E9QP49	S1462	AHGSFSHVR	S4	1.035	0.497
Ehbp1l1	EH domain-binding protein 1-like protein 1	E9QP49	S284	GQGSEPAAITGGQVGPETPEPPPSPPETR	S24	1.067	0.053
Ehbp1l1	EH domain-binding protein 1-like protein 1	E9QP49	S284	AFRGQGSEPAAITGGQVGPETPEPPPSPPETR	S27	1.033	0.868
Ehbp1l1	EH domain-binding protein 1-like protein 1	E9QP49	T448	LGDTPEAPPR	T4	0.960	0.367
Ehbp1l1	EH domain-binding protein 1-like protein 1	E9QP49	T278,S284	GQGSEPAAITGGQVGPETPEPPPSPPETR	T18,S24	1.075	0.501
Ehd1	EH domain-containing protein 1	Q9WVK4	S355	EHQISSGDFPSLR	S11	1.063	0.304
Ehd4	EH domain-containing protein 4	Q9EQP2	S15	ERSGGMDAVQTVTGGLR	S3	1.028	0.770
Ehmt1	Histone-lysine N-methyltransferase EHMT1	Q5DW34	Ambiguous	YVSQNCVTSPMNIDR	T/S/Y	1.132	0.486
Ehmt2	Histone-lysine N-methyltransferase EHMT2 (Fragment)	A2CG77	S207	ALVIQESESPPSPPPSPDRR	S9	0.826	0.366
Ehmt2	Histone-lysine N-methyltransferase EHMT2 (Fragment)	A2CG77	T119	GDGGTPPIGTAAPALPPLAHDAPGR	T5	1.065	0.689
Ei24	Etoposide induced 2.4 mRNA	A0A0R4J250	S65	ASSLLAQR	S3	1.046	0.551
Ei24	Etoposide induced 2.4 mRNA	A0A0R4J250	S344,S348	FPSPHPSPAK	S3,S7	0.946	0.640
Eif2a	Eukaryotic translation initiation factor 2A	Q8BJW6	T508	SDAAPTPVPQSAPR	T6	0.948	0.733
Eif2ak2	Interferon-induced, double-stranded RNA-activated protein kinase	Q03963	S110	KLSVNYEQCEPNSELPQR	S3	1.072	0.157
Eif2ak2	Interferon-induced, double-stranded RNA-activated protein kinase	Q03963	S223	SGVKVSPDDVQR	S6	0.997	0.863
Eif2ak2	Interferon-induced, double-stranded RNA-activated protein kinase	Q03963	Ambiguous	ELSTSGPPHDRR	S/T	1.061	0.665
Eif2ak3	Eukaryotic translation initiation factor 2-alpha kinase 3	E9QQ30	S711	EQIEVIAPSPER	S9	1.366	0.001
Eif2ak3	Eukaryotic translation initiation factor 2-alpha kinase 3	E9QQ30	S715	EQIEVIAPSPERSR	S13	1.012	0.876
Eif2ak4	eIF-2-alpha kinase GCN2	A2AUM0	T666	HERPAVPGTPPPDCTPQAQDSPATCGK	T9	1.012	0.665
Eif2b4	Translation initiation factor eIF-2B subunit delta	D3Z780	S63	TELSPRPGAAGR	S4	1.052	0.323
Eif2b5	Translation initiation factor eIF-2B subunit epsilon	Q8CHW4	S540	AGSPQLDDIR	S3	0.995	0.839
Eif2s1	Eukaryotic translation initiation factor 2 subunit 1	Q6ZWX6	T279	VVTDTDETELAR	T3	1.016	0.547
Eif2s1	Eukaryotic translation initiation factor 2 subunit 1	Q6ZWX6	Ambiguous	SIAEMGAYVSLLEYNNIEGMILLSELSRR	S/Y	0.834	0.342
Eif2s2	Eukaryotic translation initiation factor 2 subunit 2	Q99L45	S105	IESDAQEPAEPEDDLDIMLGNK	S3	0.990	0.889
Eif2s2	Eukaryotic translation initiation factor 2 subunit 2	Q99L45	S105	IESDAQEPAEPEDDLDIMLGNK	S3	1.045	0.768
Eif3a	Eukaryotic translation initiation factor 3 subunit A	P23116	S1028	GLDDERGSWR	S8	1.051	0.684
Eif3a	Eukaryotic translation initiation factor 3 subunit A	P23116	S1149	EESWGPPR	S3	1.008	0.963
Eif3a	Eukaryotic translation initiation factor 3 subunit A	P23116	S1149	AREESWGPPR	S5	1.007	0.902
Eif3a	Eukaryotic translation initiation factor 3 subunit A	P23116	S492	TLSFGSDLNYATR	S3	0.982	0.924
Eif3b	Eukaryotic translation initiation factor 3 subunit B	Q8JZQ9	S111	GHPSAGAEEEGGSDGSAAEAEPR	S4	0.966	0.742
Eif3b	Eukaryotic translation initiation factor 3 subunit B	Q8JZQ9	S75	AKPAAQSEEETATSPAASPTPQSAER	S14	1.036	0.420
Eif3b	Eukaryotic translation initiation factor 3 subunit B	Q8JZQ9	S90	SPSQEPSAPGKAEAVGEQAR	S3	1.029	0.345
Eif3b	Eukaryotic translation initiation factor 3 subunit B	Q8JZQ9	S90	SPSQEPSAPGK	S3	1.043	0.491
Eif3b	Eukaryotic translation initiation factor 3 subunit B	Q8JZQ9	S120,S123	GHPSAGAEEEGGSDGSAAEAEPR	S13,S16	0.978	0.767
Eif3b	Eukaryotic translation initiation factor 3 subunit B	Q8JZQ9	S75,S/T	AKPAAQSEEETATSPAASPTPQSAER	S14,S/T	1.054	0.344
Eif3b	Eukaryotic translation initiation factor 3 subunit B	Q8JZQ9	S68,S75,S/T	AKPAAQSEEETATSPAASPTPQSAER	S7,S14,S/T	0.977	0.251
Eif3b	Eukaryotic translation initiation factor 3 subunit B	Q8JZQ9	Ambiguous	ALENGEADEPSFSDPEDFVDDVSEEELLGDVLK	S	1.009	0.953
Eif3c	Eukaryotic translation initiation factor 3 subunit C	Q8R1B4	S166	QNPEQSADEDAEK	S6	0.965	0.566
Eif3c	Eukaryotic translation initiation factor 3 subunit C	Q8R1B4	S39	QPLLLSEDEEDTKR	S6	0.967	0.143
Eif3c	Eukaryotic translation initiation factor 3 subunit C	Q8R1B4	S39	QPLLLSEDEEDTK	S6	0.980	0.721
Eif3c	Eukaryotic translation initiation factor 3 subunit C	Q8R1B4	S530	QLTPPEGSSKSEQDQAENEGEDSAVLMER	S11	0.942	0.469
Eif3c	Eukaryotic translation initiation factor 3 subunit C	Q8R1B4	T522	QLTPPEGSSK	T3	1.069	0.538
Eif3g	Eukaryotic translation initiation factor 3 subunit G	Q9Z1D1	Ambiguous	GIPLPTGDTSPEPELLPGDPLPPPK	T/S	0.962	0.851
Eif3h	Eukaryotic translation initiation factor 3 subunit H	Q91WK2	Ambiguous	EGTGSTATSSGSAGGAVGK	T/S	0.891	0.222
Eif3j2	Eukaryotic translation initiation factor 3 subunit J-B	Q66JS6	S132	LQEESDLELAK	S5	0.951	0.409
Eif3l	Eukaryotic translation initiation factor 3 subunit L	Q8QZY1	Ambiguous	VSSDVIDQK	S	1.032	0.916
Eif4b	Eukaryotic translation initiation factor 4B	Q8BGD9	S207	ARPTTDSFDDYPPR	S7	1.080	0.081
Eif4b	Eukaryotic translation initiation factor 4B	Q8BGD9	S283	AFGSGYRR	S4	0.997	0.964
Eif4b	Eukaryotic translation initiation factor 4B	Q8BGD9	S422	TGSESSQTGASATSGR	S3	0.999	0.990
Eif4b	Eukaryotic translation initiation factor 4B	Q8BGD9	S445	ESEKSLENETLNK	S5	1.102	0.131
Eif4b	Eukaryotic translation initiation factor 4B	Q8BGD9	S497	SQSSDTEQPSPTSGGGK	S3	1.093	0.081
Eif4b	Eukaryotic translation initiation factor 4B	Q8BGD9	S597	YAALSVDGEDEDEGDDCTE	S5	0.989	0.748
Eif4b	Eukaryotic translation initiation factor 4B	Q8BGD9	S422,S425	TGSESSQTGASATSGR	S3,S6	1.113	0.560
Eif4b	Eukaryotic translation initiation factor 4B	Q8BGD9	S497,S498	SQSSDTEQPSPTSGGGK	S3,S4	1.086	0.131
Eif4ebp1	Eukaryotic translation initiation factor 4E-binding protein 1	Q60876	Ambiguous	VALGDGVQLPPGDYSTTPGGTLFSTTPGGTR	S/T/Y	1.440	0.168
Eif4ebp2	Eukaryotic translation initiation factor 4E-binding protein 2	P70445	T41,S/T/Y	TVAISDAAQLPQDYCTTPGGTLFSTTPGGTR	T21,S/T/Y	1.576	0.446
Eif4ebp2	Eukaryotic translation initiation factor 4E-binding protein 2	P70445	Ambiguous	TVAISDAAQLPQDYCTTPGGTLFSTTPGGTR	T/S/Y	1.041	0.897
Eif4enif1	Eukaryotic translation initiation factor 4E transporter	Q9EST3	S563	APSPPMSQVFR	S3	1.016	0.725
Eif4enif1	Eukaryotic translation initiation factor 4E transporter	Q9EST3	S563	APSPPMSQVFR	S3	0.987	0.929
Eif4enif1	Eukaryotic translation initiation factor 4E transporter	Q9EST3	S77	SSPVESLK	S2	0.982	0.768
Eif4enif1	Eukaryotic translation initiation factor 4E transporter	Q9EST3	S949	SSSPVGLAK	S3	0.987	0.767
Eif4g1	Eukaryotic translation initiation factor 4 gamma 1	Q6NZJ6	S1189	SFSKEVEER	S3	1.051	0.291
Eif4g1	Eukaryotic translation initiation factor 4 gamma 1	Q6NZJ6	S1211	AASLTEDR	S3	1.015	0.693
Eif4g1	Eukaryotic translation initiation factor 4 gamma 1	Q6NZJ6	S1211	KAASLTEDR	S4	1.027	0.622
Eif4g1	Eukaryotic translation initiation factor 4 gamma 1	Q6NZJ6	S1231	EATLPPVSPPK	S8	1.045	0.365
Eif4g1	Eukaryotic translation initiation factor 4 gamma 1	Q6NZJ6	T1226,S1231	EATLPPVSPPK	T3,S8	0.822	0.168
Eif4g1	Eukaryotic translation initiation factor 4 gamma 1	Q6NZJ6	Ambiguous	TASTPTPPQTGGSLEPQPNGESPQVAVIIRPDDR	T/S	1.032	0.608
Eif4g1	Eukaryotic translation initiation factor 4 gamma 1	Q6NZJ6	Ambiguous	TASTPTPPQTGGSLEPQPNGESPQVAVIIRPDDR	S/T	1.204	0.173
Eif4g2	Eukaryotic translation initiation factor 4 gamma 2	G3XA17	Ambiguous	FGASSGGGGSR	S	0.969	0.597
Eif4g2	Eukaryotic translation initiation factor 4 gamma 2	G3XA17	Ambiguous	TQTPPLGQTPQLGLK	T	0.899	0.681
Eif4g3	Eukaryotic translation initiation factor 4 gamma 3	A0A0R4J112	S1106	ASESAPSGSPSATPLEFDSRR	S9	0.967	0.462
Eif4g3	Eukaryotic translation initiation factor 4 gamma 3	A0A0R4J112	S1106	ASESAPSGSPSATPLEFDSR	S9	1.129	0.099
Eif4g3	Eukaryotic translation initiation factor 4 gamma 3	A0A0R4J112	S1150	GSSKDLLDNQSQEEQR	S3	1.286	0.002
Eif4g3	Eukaryotic translation initiation factor 4 gamma 3	A0A0R4J112	S285	TSSPTSLPPLAR	S3	1.016	0.533
Eif4g3	Eukaryotic translation initiation factor 4 gamma 3	A0A0R4J112	S488	EMQDKAESESDGQAEETADPQSLHSGR	S8	0.930	0.087
Eif4g3	Eukaryotic translation initiation factor 4 gamma 3	A0A0R4J112	S488	EMQDKAESESDGQAEETADPQSLHSGR	S8	1.049	0.734
Eif4g3	Eukaryotic translation initiation factor 4 gamma 3	A0A0R4J112	T277	EQAGQMPETAAGEPTPEPPR	T15	0.990	0.925
Eif4g3	Eukaryotic translation initiation factor 4 gamma 3	A0A0R4J112	Ambiguous	EEDAPPVPSPTSCTAASGPSLTDNSDICK	S/T	0.926	0.475
Eif4g3	Eukaryotic translation initiation factor 4 gamma 3	A0A0R4J112	Ambiguous	EQAGQMPETAAGEPTPEPPRTSSPTSLPPLAR	S/T	0.984	0.614
Eif4g3	Eukaryotic translation initiation factor 4 gamma 3	A0A0R4J112	Ambiguous	GSSKDLLDNQSQEEQRR	S	1.105	0.259
Eif4g3	Eukaryotic translation initiation factor 4 gamma 3	A0A0R4J112	Ambiguous	EQAGQMPETAAGEPTPEPPRTSSPTSLPPLAR	T/S	1.014	0.852
Eif4g3	Eukaryotic translation initiation factor 4 gamma 3	A0A0R4J112	Ambiguous	EQTRTPDEVLEAEAEPK	T	1.217	0.173
Eif4g3	Eukaryotic translation initiation factor 4 gamma 3	A0A0R4J112	Ambiguous	EQAGQMPETAAGEPTPEPPRTSSPTSLPPLAR	S/T	0.726	0.159
Eif4g3	Eukaryotic translation initiation factor 4 gamma 3	A0A0R4J112	Ambiguous	EQAGQMPETAAGEPTPEPPRTSSPTSLPPLAR	T/S	1.219	0.424
Eif4h	Eukaryotic translation initiation factor 4H	Q9WUK2	S21	AYSSFGGGRGSR	S11	0.957	0.453
Eif5	Eukaryotic translation initiation factor 5	P59325	S227	GLTLSDDLER	S5	0.987	0.656
Eif5	Eukaryotic translation initiation factor 5	P59325	T153	NPPENSDIGTGKK	T10	1.316	0.160
Eif5b	Eukaryotic translation initiation factor 5B	Q05D44	S114	TSFDENDSEELEDKDSK	S8	0.978	0.669
Eif5b	Eukaryotic translation initiation factor 5B	Q05D44	S114	TSFDENDSEELEDK	S8	0.974	0.792
Eif5b	Eukaryotic translation initiation factor 5B	Q05D44	S114	KTSFDENDSEELEDKDSK	S9	1.011	0.883
Eif5b	Eukaryotic translation initiation factor 5B	Q05D44	S1164	IEPIPGESPK	S8	1.007	0.957
Eif5b	Eukaryotic translation initiation factor 5B	Q05D44	S137	TARPNSEAPLSGSEDADDSNKLSK	S11	0.971	0.244
Eif5b	Eukaryotic translation initiation factor 5B	Q05D44	S137	TARPNSEAPLSGSEDADDSNK	S11	1.025	0.755
Eif5b	Eukaryotic translation initiation factor 5B	Q05D44	S137	KTARPNSEAPLSGSEDADDSNK	S12	1.070	0.106
Eif5b	Eukaryotic translation initiation factor 5B	Q05D44	S187	VNSSGESGGESDEFLQSR	S7	0.927	0.221
Eif5b	Eukaryotic translation initiation factor 5B	Q05D44	S187	SRVNSSGESGGESDEFLQSR	S9	0.981	0.859
Eif5b	Eukaryotic translation initiation factor 5B	Q05D44	S215	SVPTVDSGNEDDDSSFKIK	S7	0.782	0.176
Eif5b	Eukaryotic translation initiation factor 5B	Q05D44	S215	SVPTVDSGNEDDDSSFK	S7	0.993	0.766
Eif5b	Eukaryotic translation initiation factor 5B	Q05D44	S294	GTPDTGAASEEKGDTAAALEDDNEGDKK	S9	0.946	0.175
Eif5b	Eukaryotic translation initiation factor 5B	Q05D44	S137,S139	TARPNSEAPLSGSEDADDSNK	S11,S13	1.021	0.751
Eif5b	Eukaryotic translation initiation factor 5B	Q05D44	S183,S187	SRVNSSGESGGESDEFLQSR	S5,S9	1.110	0.155
Eif6	Eukaryotic translation initiation factor 6	O55135	T245	DSLIDSLT	T8	0.908	0.269

Elac2	Zinc phosphodiesterase ELAC protein 2	Q80Y81	S195	TSPNRLSPK	S2	0.974	0.747
Elac2	Zinc phosphodiesterase ELAC protein 2	Q80Y81	S797	AALLTQQADSPEDREPQQK	S10	1.015	0.750
Elac2	Zinc phosphodiesterase ELAC protein 2	Q80Y81	S815	AHTDEPHSPQSK	S8	1.031	0.705
Elac2	Zinc phosphodiesterase ELAC protein 2	Q80Y81	S191,S200,T/S	QQPSQSPRTSPNRLSPK	S6,S15,S/T	0.892	0.317
Elf1	ETS-related transcription factor Elf-1	Q60775	S334	VSSSPGIK	S4	1.040	0.433
Elf1	ETS-related transcription factor Elf-1	Q60775	S611	QNELLEPNSF	S9	1.006	0.949
Elf2	ETS-related transcription factor Elf-2	Q9JHC9	S188	TQQSPVSNGSPELGIK	S7	1.047	0.549
Elf2	ETS-related transcription factor Elf-2	Q9JHC9	S363	VVNITSPTHDGSSR	S6	1.029	0.735
Elf2	ETS-related transcription factor Elf-2	Q9JHC9	T523	LSVPAQQASGQTPPR	T12	0.928	0.231
Elf4	ETS-related transcription factor Elf-4	Q9Z2U4	S156	TSDAGDQKEHSEEEKVSR	S11	1.007	0.948
Elf4	ETS-related transcription factor Elf-4	Q9Z2U4	S187	STSPVTDPSMPIR	S3	0.970	0.332
Elf4	ETS-related transcription factor Elf-4	Q9Z2U4	Ambiguous	DLVVIDDEEESPETPEDSSQASTSSTPSTSTIR	S/T	0.975	0.802
Elf4	ETS-related transcription factor Elf-4	Q9Z2U4	Ambiguous	STSPVTDPSMPIR	T/S	1.006	0.889
Elk3	ETS domain-containing protein Elk-3	P41971	S173	TEKLEEPCDDSPPVEEVR	S11	1.036	0.633
Ell	RNA polymerase II elongation factor ELL	O08856	Ambiguous	KLCQPQNATTDSSPPREHGR	S/T	1.333	0.130
Ell2	RNA polymerase II elongation factor ELL2	Q3UKU1	S304	SESPLCSSK	S3	0.995	0.862
Ell2	RNA polymerase II elongation factor ELL2	Q3UKU1	S501	LSNASPNPNEGVK	S5	1.048	0.193
Elmo1	Engulfment and cell motility protein 1	Q8BPU7	S344	IAFDAESEPNNSSGSMEK	S15	0.902	0.063
Elmo1	Engulfment and cell motility protein 1	Q8BPU7	S344	RIAFDAESEPNNSSGSMEK	S16	0.816	0.171
Elmo1	Engulfment and cell motility protein 1	Q8BPU7	S344	IAFDAESEPNNSSGSMEK	S15	0.742	0.248
Elmo1	Engulfment and cell motility protein 1	Q8BPU7	S344	IAFDAESEPNNSSGSMEKR	S15	0.974	0.713
Elmo1	Engulfment and cell motility protein 1	Q8BPU7	S94	IQSSSMDAK	S3	1.064	0.375
Elmo1	Engulfment and cell motility protein 1	Q8BPU7	Y724	EPSNYDFVYDCN	Y9	1.108	0.112
Elmo1	Engulfment and cell motility protein 1	Q8BPU7	Ambiguous	RIAFDAESEPNNSSGSMEKR	S	1.036	0.585
Elmo2	Engulfment and cell motility protein 2	Q8BHL5	S515	SLSYSEILR	S3	1.045	0.507
Elmo2	Engulfment and cell motility protein 2	Q8BHL5	S529	MSQDDFQSPPIVELR	S2	1.002	0.986
Elmo2	Engulfment and cell motility protein 2	Q8BHL5	S529	QSERMSQDDFQSPPIVELR	S6	1.071	0.700
Elmo2	Engulfment and cell motility protein 2	Q8BHL5	S529	QSERMSQDDFQSPPIVELR	S6	1.001	0.986
Elmsan1	ELM2 and Myb/SANT-like domain-containing 1	E9Q2I4	S1083	EAAAAAAHQQALREESGEGEKG	S16	0.983	0.833
Elmsan1	ELM2 and Myb/SANT-like domain-containing 1	E9Q2I4	S34	EQPPPLQSPPQSLR	S8	0.974	0.806
Elmsan1	ELM2 and Myb/SANT-like domain-containing 1	E9Q2I4	S564	STRVPGTDAAAQAEDLNVK	S1	1.892	0.003
Elmsan1	ELM2 and Myb/SANT-like domain-containing 1	E9Q2I4	S653	SFEPPPYTPPPILSPVR	S14	0.923	0.751
Elmsan1	ELM2 and Myb/SANT-like domain-containing 1	E9Q2I4	S988	SHEPNAPGSAGIQTSEKPR	S9	1.307	0.001
Elmsan1	ELM2 and Myb/SANT-like domain-containing 1	E9Q2I4	T696,T707	SNSSEVTPPVLSVMGEATPVSIEPR	T7,T18	0.874	0.662
Elmsan1	ELM2 and Myb/SANT-like domain-containing 1	E9Q2I4	T707,S/T	SNSSEVTPPVLSVMGEATPVSIEPR	T18,S/T	0.968	0.835
Eloa	Elongin-A	Q8CB77	S424	TSSTALGEK	S3	0.964	0.732
Eloa	Elongin-A	Q8CB77	S515	AFSSPQEEEEAGFTGRR	S4	1.117	0.416
Eloa	Elongin-A	Q8CB77	Ambiguous	AFSSPQEEEEAGFTGR	S/T	0.996	0.958
Emb	Embigin	P21995	S312	SDDSNGIENNVPR	S4	1.040	0.517
Emc4	ER membrane protein complex subunit 4	Q9CZX9	S36	GRSDRGSGQGDSLYPVGYLDK	S7	1.081	0.567
Emd	Emerin	O08579	T123	QPGTSLVDADTFHHQVR	T4	0.824	0.224
Eme2	Probable crossover junction endonuclease EME2	Q56A04	S49	EVGTQAPSPAGER	S8	0.939	0.350
Emilin2	EMILIN-2	Q8K482	Ambiguous	TGDSMEASGFTK	T/S	0.981	0.894
Emilin2	EMILIN-2	Q8K482	Ambiguous	TGDSMEASGFTK	T/S	1.049	0.305
Eml3	Echinoderm microtubule-associated protein-like 3	Q8VC03	S177	AASSANLLLR	S3	0.947	0.273
Eml3	Echinoderm microtubule-associated protein-like 3	Q8VC03	S890	TPSLSPASSLDV	S5	1.003	0.981
Eml3	Echinoderm microtubule-associated protein-like 3	Q8VC03	T882	VLGAGSSGPAPATPSR	T13	0.925	0.363
Eml3	Echinoderm microtubule-associated protein-like 3	Q8VC03	Ambiguous	GNKDPLSSPGGPGSR	S	1.099	0.093
Eml4	Echinoderm microtubule-associated protein-like 4	F8WJ93	S144	EDSHSNDQSPQIRASPSPQPSSQPLQINR	S15	0.780	0.150
Eml4	Echinoderm microtubule-associated protein-like 4	F8WJ93	S144	ASPSPQPSSQPLQINR	S2	0.980	0.337
Eml4	Echinoderm microtubule-associated protein-like 4	F8WJ93	S908	TPASSSETARPSNSPPLPPSLPLTGTAEEESR	S14	1.046	0.105
Emsy	BRCA2-interacting transcriptional repressor EMSY	Q8BMB0	S173	TNSSSSSPVVLK	S3	0.989	0.812
Emsy	BRCA2-interacting transcriptional repressor EMSY	Q8BMB0	Ambiguous	ESCSSPSAVGPPLTTR	S/T	1.028	0.816
Emsy	BRCA2-interacting transcriptional repressor EMSY	Q8BMB0	Ambiguous	DGVSCSDEDEKPR	S	1.290	0.002
Engase	Cytosolic endo-beta-N-acetylglucosaminidase	Q8BX80	S16	QRSPAAPEK	S3	1.069	0.098
Engase	Cytosolic endo-beta-N-acetylglucosaminidase	Q8BX80	S56	EVVSFTPDPLPAR	S4	0.927	0.537
Enkd1	Enkurin domain-containing protein 1	Q7TSV9	Ambiguous	DLDSSPPRGPR	S	0.985	0.362
Eno1	Alpha-enolase	P17182	T272	YITPDQLADLYK	T3	1.011	0.936
Eno1	Alpha-enolase	P17182	Y44	AAVPSGASTGIYEALELR	Y12	1.106	0.642
Ensa	Alpha-endosulfine	P60840	S67	YFDSGDYNMAK	S4	0.984	0.571
Ensa	Alpha-endosulfine	P60840	S67	YFDSGDYNMAK	S4	0.928	0.257
Entr1	Endosome-associated-trafficking regulator 1	A2AIW0	S241	ISPASPAGSPNADFAAHEESLGDR	S5	0.950	0.600
Entr1	Endosome-associated-trafficking regulator 1	A2AIW0	S241,S245	ISPASPAGSPNADFAAHEESLGDR	S5,S9	1.014	0.861
Ep300	Histone acetyltransferase p300	B2RWS6	S1030	TDGKEEEEQPSTSATQSSPAPGQSK	S11	1.050	0.280
Ep300	Histone acetyltransferase p300	B2RWS6	Ambiguous	LGLGLDDESNNQQAAATQSPGDSRR	S/T	0.867	0.063
Ep300	Histone acetyltransferase p300	B2RWS6	Ambiguous	GPELKTDGKEEEEQPSTSATQSSPAPGQSK	S/T	0.932	0.703
Ep300	Histone acetyltransferase p300	B2RWS6	Ambiguous	QTPTPPTHLPPQVQPSLPAAPSADQSQQQPR	T/S	0.980	0.640
Ep400	E1A-binding protein p400	Q8CHI8	S735	ALSPITSR	S3	0.949	0.380
Ep400	E1A-binding protein p400	Q8CHI8	S741,S747	SPGVAVSAPPKPQSPAQNAASSQDGSQDK	S1,S7	1.049	0.617
Ep400	E1A-binding protein p400	Q8CHI8	Ambiguous	GFDTSPEHSLDLGISGR	T/S	1.271	0.232
Epb41	Protein 4.1 (Fragment)	A0A1D5RLV1; A2A841	T282;T585	SPRPTSAPAIAQSQVTEGPGAPIK	T5	0.874	0.203
Epb41	Protein 4.1	A2A841	S207	EGEGIEECSGTEVKEDPESR	S9	1.023	0.713
Epb41	Protein 4.1	A2A841	S568	SLDGAAAAESTDR	S1	0.992	0.851
Epb41	Protein 4.1	A2A841	Ambiguous	EGEGIEECSGTEVK	S/T	1.105	0.024
Epb41	Protein 4.1	A2A841	Ambiguous	ASRSLDGAAAAESTDR	S/T	1.074	0.633
Epb41	Protein 4.1	A2A841	Ambiguous	VVVHQETEISEE	T/S	1.087	0.740
Epb41l1	Band 4.1-like protein 1 (Fragment)	A0A2R8VHB6; A2AUK7; A2AUK5	S185;S497;S510	HQASINELKR	S4	1.081	0.061
Epb41l1	Band 4.1-like protein 1 (Fragment)	A0A2R8VHB6; A2AUK7; A2AUK5	S185;S497;S510	HQASINELK	S4	1.018	0.895
Epb41l1	Band 4.1-like protein 1 (Fragment)	A0A2R8VHB6; A2AUK7; A2AUK5	S73;S378;S378	LVSPEPPPK	S3	1.106	0.038
Epb41l1	Band 4.1-like protein 1 (Fragment)	A0A2R8VHB6; A2AUK7; A2AUK5	T162;T474;T475	SEAEEGEVRTPTK	T10	1.104	0.027
Epb41l1	Band 4.1-like protein 1 (Fragment)	A0A2R8VHB6; A2AUK7; A2AUK5	T162;T474;T475	RSEAEEGEVRTPTK	T11	1.186	0.016
Epb41l1	Band 4.1-like protein 1	A2AUK5	S441	SLDGAEFSRPASVSENHDAGPDGDKREDDAESGGR	S12	0.987	0.952
Epb41l1	Band 4.1-like protein 1	A2AUK5	S578	APESDTGDEDQDQERDAVFLK	S4	1.024	0.106
Epb41l1	Band 4.1-like protein 1	A2AUK5	S666	DLKGPSSQEDESGGLEDSPDR	S7	1.006	0.963
Epb41l1	Band 4.1-like protein 1	A2AUK5	S430,S	SLDGAEFSRPASVSENHDAGPDGDKREDDAESGGR	S1,S	1.099	0.248
Epb41l1	Band 4.1-like protein 1	A2AUK5	S437,S441	SLDGAEFSRPASVSENHDAGPDGDKR	S8,S12	1.069	0.501
Epb41l1	Band 4.1-like protein 1	A2AUK7; A2AUK5	S531;S544	LPSSPASPSPK	S7	0.946	0.447
Epb41l1	Band 4.1-like protein 1	A2AUK7; A2AUK5	S531;S544	RLPSSPASPSPK	S8	1.019	0.675
Epb41l1	Band 4.1-like protein 1	A2AUK7; A2AUK5	S719;S868	AVVYRETDPSPEER	S10	0.868	0.258
Epb41l1	Band 4.1-like protein 1	A2AUK7; A2AUK5	S719;S868	ETDPSPEER	S5	0.975	0.753
Epb41l1	Band 4.1-like protein 1	A2AUK7	S436,S440	SLDGEFSRPASVSENHDAGPDGDKR	S7,S11	1.128	0.411
Epb41l1	Band 4.1-like protein 1	A2AUK7; A2AUK5	S527,S531,S544	RLPSSPASPSPK	S4,S8	1.101	0.093
Epb41l1	Band 4.1-like protein 1	A2AUK5	Ambiguous	GPSSQEDESGGLEDSPDRGACSTPEMPQFESVK	S/T	1.189	0.093
Epb41l1	Band 4.1-like protein 1	A2AUK5	Ambiguous	SLDGAEFSRPASVSENHDAGPDGDKR	S	1.099	0.788
Epb41l1	Band 4.1-like protein 1	A2AUK7; A2AUK5	Ambiguous	AQEETPQQPEAAAAVTTPVTPAGHSHPETNSNEK	T/S	1.342	0.088
Epb41l2	Band 4.1-like protein 2	Q80UE5	S116	EPTQADVEDQILGKEESLPEEESR	S17	0.985	0.851
Epb41l2	Band 4.1-like protein 2	Q80UE5	S38	EVENEQTPVSEPEEEKGSQPGPPVER	S10	1.025	0.129
Epb41l2	Band 4.1-like protein 2	Q80UE5	S38	EVENEQTPVSEPEEEK	S10	0.930	0.377
Epb41l2	Band 4.1-like protein 2	Q80UE5	S38	AKEVENEQTPVSEPEEEK	S12	0.985	0.835
Epb41l2	Band 4.1-like protein 2	Q80UE5	S38	AKEVENEQTPVSEPEEEKGSQPGPPVER	S12	0.999	0.964
Epb41l2	Band 4.1-like protein 2	Q80UE5	S492	LVSPEQPPKTK	S3	1.082	0.277
Epb41l2	Band 4.1-like protein 2	Q80UE5	S492	LVSPEQPPK	S3	1.013	0.501
Epb41l2	Band 4.1-like protein 2	Q80UE5	S628	AEVGKDESVITEEMNGKEMSPGHGPGETR	S20	0.919	0.290
Epb41l2	Band 4.1-like protein 2	Q80UE5	S628	AEVGKDESVITEEMNGKEMSPGHGPGETR	S20	0.990	0.864
Epb41l2	Band 4.1-like protein 2	Q80UE5	S628	EMSPGHGPGETR	S3	1.041	0.087
Epb41l2	Band 4.1-like protein 2	Q80UE5	S628	EMSPGHGPGETR	S3	1.051	0.460
Epb41l2	Band 4.1-like protein 2	Q80UE5	S86	SYNLVVAK	S1	1.199	0.076
Epb41l2	Band 4.1-like protein 2	Q80UE5	S86	QRSYNLVVAK	S3	1.015	0.904
Epb41l2	Band 4.1-like protein 2	Q80UE5	T192	EVQTSELK	T4	0.889	0.244
Epb41l2	Band 4.1-like protein 2	Q80UE5	Ambiguous	SSHETLNVVEEK	S/T	1.022	0.925
Epb41l3	Band 4.1-like protein 3 (Fragment)	A0A286YDY4	S251	SLDGEVGTGQYATTK	S1	1.000	1.000
Epb41l3	Band 4.1-like protein 3	Q9WV92	S451	SLDGASVSENHEIYMK	S1	0.996	0.976
Epb41l3	Band 4.1-like protein 3	Q9WV92; A0A286YDY4	S804;S762	VESTSVGSISPGGAK	S10	0.959	0.617
Epb41l3	Band 4.1-like protein 3	Q9WV92	S96	QLEYQQFEDDKLSQR	S13	1.085	0.663
Epb41l3	Band 4.1-like protein 3	Q9WV92; A0A286YDY4	S599,T/S;S557,T/S	RLSTSPVR	S3,T/S	0.975	0.856
Epc1	Enhancer of polycomb homolog 1	Q8C9X6	S534	TPSLPDSDSGELSSR	S3	1.019	0.792
Epg5	Ectopic P granules protein 5 homolog	Q80TA9	S45	EQESPCPASELKGDDLK	S4	0.872	0.228
Epn1	Epsin-1	Q80VP1	Ambiguous	TALPTSGSSTGELELLAGEVPAR	S/T	1.416	0.321
Epn1	Epsin-1	Q80VP1	Ambiguous	SPGAFDMSGVGGSLAESVGSPPPAATPTPTPPTR	S/T	0.737	0.411
Epn2	Epsin-2	J3QNT7	S173	GSSQPNLSTSYSEQEYGK	S3	0.920	0.195
Epn2	Epsin-2	J3QNT7	Ambiguous	GSSQPNLSTSYSEQEYGK	S/T/Y	0.808	0.376
Eprs	Bifunctional glutamate/proline--tRNA ligase	Q8CGC7	S998	SQGSGLSSGGAGEGQGPK	S7	0.889	0.087
Eps15	Epidermal growth factor receptor substrate 15	H3BK65	T622	SSPEIAPSDVTDESEAVTVAGNEKVTPR	T26	1.112	0.671
Eps15	Epidermal growth factor receptor substrate 15	H3BK65	T815	VGTPTRPCPPPPGK	T3	1.190	0.293
Eps15	Epidermal growth factor receptor substrate 15	H3BK65	Ambiguous	SSPEIAPSDVTDESEAVTVAGNEK	S/T	0.950	0.315
Eps15	Epidermal growth factor receptor substrate 15	H3BK65	Ambiguous	TDKDMDGYVSGLEVR	S/Y/T	1.049	0.268
Eps15l1	Epidermal growth factor receptor substrate 15-like 1	Q60902	S255	STPSHGSVSSLNSTGSLSPK	S18	0.980	0.821
Eps15l1	Epidermal growth factor receptor substrate 15-like 1	Q60902	S353	GIDPPQVLSPDMVPPSER	S9	0.913	0.591
Eps15l1	Epidermal growth factor receptor substrate 15-like 1	Q60902	S241,S255	STPSHGSVSSLNSTGSLSPK	S4,S18	1.035	0.861
Eps8	Epidermal growth factor receptor kinase substrate 8	Q08509	S814	ISAAASDSGVESFDEGSSH	S12	0.976	0.443
Eps8	Epidermal growth factor receptor kinase substrate 8	Q08509	S658,S	QNSSSSDSGGSIVR	S3,S	1.164	0.113
Eps8	Epidermal growth factor receptor kinase substrate 8	Q08509	S810,S814	ISAAASDSGVESFDEGSSH	S8,S12	0.868	0.360
Eps8	Epidermal growth factor receptor kinase substrate 8	Q08509	Ambiguous	QNSSSSDSGGSIVR	S	1.167	0.005
Epsti1	Epithelial stromal interaction 1 (Breast)	A0A140T8I6	S39	RQSLEVAAPEGPK	S3	1.318	0.001
Erbin	Erbb2ip protein	B7ZNX6	S1077	QSSVSSTASVNLGDPTR	S3	1.268	0.001
Erbin	Erbb2ip protein	B7ZNX6	S1077	QSSVSSTASVNLGDPTRR	S3	1.135	0.126
Erbin	Erbb2ip protein	B7ZNX6	S1125	AYSIDGPNTSRPQSARPSINEIPER	S3	1.404	0.002
Erbin	Erbb2ip protein	B7ZNX6	S1150	TMSVSDFNYSR	S3	1.296	0.098

Erbin	Erbb2ip protein	B7ZNX6	S1171	VGSEHSLLDPPGK	S3	1.111	0.535
Erbin	Erbb2ip protein	B7ZNX6	S1231	DLHGSQGSLALSVADGR	S5	1.095	0.373
Erbin	Erbb2ip protein	B7ZNX6	S639	DDAKDADSLSDEVTHNSNQNNSNCSSPSR	S8	1.089	0.359
Erbin	Erbb2ip protein	B7ZNX6	S849	ASSSADVGISKSTEDLSPQR	S12	1.000	0.994
Erbin	Erbb2ip protein	B7ZNX6	S854	STEDLSPQR	S6	1.042	0.400
Erbin	Erbb2ip protein	B7ZNX6	S869	SHSITNMETGGLK	S3	1.014	0.840
Erbin	Erbb2ip protein	B7ZNX6	T485	RYPTPYPDELK	T4	1.020	0.870
Erc1	ELKS/Rab6-interacting/CAST family member 1	F8VPM7	S21	VEPSSQSPGRSPR	S11	0.985	0.900
Erc1	ELKS/Rab6-interacting/CAST family member 1	F8VPM7	S37	TNSTGGSSGNSVGGGSGK	S3	1.032	0.664
Erc1	ELKS/Rab6-interacting/CAST family member 1	F8VPM7	Ambiguous	RTNSTGGSSGNSVGGGSGK	S/T	1.070	0.542
Erc1	ELKS/Rab6-interacting/CAST family member 1	F8VPM7	Ambiguous	SVGKVEPSSQSPGRSPR	S	1.081	0.305
Erc1	ELKS/Rab6-interacting/CAST family member 1	F8VPM7	Ambiguous	SVGKVEPSSQSPGR	S	1.023	0.700
Erc1	ELKS/Rab6-interacting/CAST family member 1	F8VPM7	Ambiguous	SVGKVEPSSQSPGRSPR	S	0.960	0.326
Ercc3	General transcription and DNA repair factor IIH helicase subunit XPB	P49135	S34	QYEEEEEDEDDIPGNESQEAVPSAAGK	S17	0.959	0.859
Ercc4	DNA repair endonuclease XPF	Q9QZD4	S522	QTSSSPEGCGVEIKR	S5	1.038	0.378
Ercc4	DNA repair endonuclease XPF	Q9QZD4	S522	QTSSSPEGCGVEIK	S5	1.001	0.951
Ercc4	DNA repair endonuclease XPF	Q9QZD4	S521,S522	QTSSSPEGCGVEIK	S4,S5	1.100	0.433
Ercc4	DNA repair endonuclease XPF	Q9QZD4	Ambiguous	TSSPEVQETK	T/S	1.076	0.193
Ercc4	DNA repair endonuclease XPF	Q9QZD4	Ambiguous	TSEKTSSPEVQETK	T/S	1.058	0.635
Ercc5	DNA repair protein-complementing XP-G cells homolog	E9QM61	S341	VKPEREPEAAPPSPR	S13	1.054	0.203
Ercc5	DNA repair protein-complementing XP-G cells homolog	E9QM61	S384	NSGATADAGSISPR	S12	1.011	0.779
Ercc5	DNA repair protein-complementing XP-G cells homolog	E9QM61	S572	LSSDDETEGGQNPAPK	S3	1.052	0.525
Ercc6	ision repair cross-complementing rodent repair deficiency, complementation grou	F8VPZ5	S158	IIEQLSPQAASNR	S6	0.809	0.379
Ercc6l	DNA excision repair protein ERCC-6-like	Q8BHK9	S1021	ILSDDEDEDEEDAFK	S3	1.090	0.536
Ercc6l	DNA excision repair protein ERCC-6-like	Q8BHK9	S1021	RILSDDEDEDEEDAFK	S4	1.237	0.093
Erf	ETS domain-containing transcription factor ERF	A0A0R4J0I0	S185	GSVSDCSDGTSELEEPLGEDPR	S2	0.933	0.570
Erf	ETS domain-containing transcription factor ERF	A0A0R4J0I0	S185	LGRGSVSDCSDGTSELEEPLGEDPR	S5	1.279	0.029
Erf	ETS domain-containing transcription factor ERF	A0A0R4J0I0	T529	GDVGPGESGGPLTPR	T13	1.240	0.003
Ergic3	Endoplasmic reticulum-Golgi intermediate compartment protein 3	Q9CQE7	Ambiguous	RLDKDGVPVSSEAER	S	1.005	0.940
Ergic3	Endoplasmic reticulum-Golgi intermediate compartment protein 3	Q9CQE7	Ambiguous	LDKDGVPVSSEAER	S	1.031	0.524
Erich1	Glutamate-rich 1	E9PY43	Ambiguous	ASGVSFTYQPESSEEAEAEDDVGGEEEGDPER	S/T/Y	1.206	0.592
Ero1a	ERO1-like protein alpha	Q8R180	S144	LGAVDESLSEETQK	S9	0.962	0.700
Esco1	N-acetyltransferase ESCO1	Q69Z69	S33	ESEDENLEEVESSQNSPTKK	S17	1.074	0.381
Esco2	N-acetyltransferase ESCO2	Q8CIB9	S200	NYPNSPPR	S5	1.014	0.756
Esd	S-formylglutathione hydrolase	H3BKH6	S202	AFSGYLGPDESK	S3	0.929	0.699
Esf1	ESF1 homolog	Q3V1V3	S156	IDSEISPK	S6	1.011	0.898
Espl1	Separin	P60330	Ambiguous	VLVGEGGSSPGPLSPQR	S	0.938	0.698
Esrra	Steroid hormone receptor ERR1	O08580	S19	AEPASPDSPKGSSETETEPPVTLASGPAPAR	S5	1.081	0.684
Esrra	Steroid hormone receptor ERR1	O08580	S19,S/T	AEPASPDSPKGSSETETEPPVTLASGPAPAR	S5,S/T	0.838	0.045
Ess2	Expressed sequence 2 embryonic lethal, isoform CRA_a	Q3UFM6	S296	ELIPQESPR	S7	0.974	0.306
Ess2	Expressed sequence 2 embryonic lethal, isoform CRA_a	Q3UFM6	S399	GLSPAMSPALQR	S7	0.796	0.151
Ess2	Expressed sequence 2 embryonic lethal, isoform CRA_a	Q3UFM6	S399	GLSPAMSPALQR	S7	0.965	0.620
Ess2	Expressed sequence 2 embryonic lethal, isoform CRA_a	Q3UFM6	T441	TPAGGPQTPTSTPAPGSATR	T8	1.034	0.445
Ess2	Expressed sequence 2 embryonic lethal, isoform CRA_a	Q3UFM6	S395,S399	GLSPAMSPALQR	S3,S7	0.934	0.562
Ess2	Expressed sequence 2 embryonic lethal, isoform CRA_a	Q3UFM6	S395,S399	GLSPAMSPALQR	S3,S7	1.014	0.807
Esyt1	Extended synaptotagmin-1	Q3U7R1	S113	ITAETLYMSHR	S9	1.138	0.089
Esyt1	Extended synaptotagmin-1	Q3U7R1	S512	ATYSTNSPVWEEAFR	S7	0.937	0.565
Esyt1	Extended synaptotagmin-1	Q3U7R1	S951	LTHGDSPSEAPVGPLGQVK	S6	1.011	0.868
Esyt2	Extended synaptotagmin-2	Q3TZZ7	S682	EPTPSIASDISLPIATQELR	S8	0.822	0.445
Esyt2	Extended synaptotagmin-2	Q3TZZ7	Y748	NLIAFSEDGSDPYVR	Y13	1.439	0.312
Esyt2	Extended synaptotagmin-2	Q3TZZ7	S682,S685	EPTPSIASDISLPIATQELR	S8,S11	0.932	0.838
Etfdh	Electron transfer flavoprotein-ubiquinone oxidoreductase, mitochondrial	Q921G7	S550	NLSIYDGPEQR	S3	1.002	0.977
Ethe1	Persulfide dioxygenase ETHE1, mitochondrial	Q9DCM0	S14	RLSQQSASGAPVLLR	S3	0.898	0.092
Etl4	Enhancer trap locus 4	E9QAU4	S1956	AAPTTSSSSSPPSPASPTSLNQGAR	S10	0.899	0.208
Etl4	Enhancer trap locus 4	E9QAU4	S1956,S/T	AAPTTSSSSSPPSPASPTSLNQGAR	S16,S/T	0.960	0.851
Etv3	ETS translocation variant 3	Q8R4Z4	S139	SSGVVPQSAPPVPTASSR	S8	1.125	0.166
Etv3	ETS translocation variant 3	Q8R4Z4	S181	FSASSLSASGPESGVTTDR	S13	1.158	0.101
Etv6	Transcription factor ETV6	P97360	T18	ISYTPPESPVASHR	T4	1.059	0.311
Etv6	Transcription factor ETV6	P97360	T234	LQQENNHQETYPLSVSPVENNHCLPSSPWQESTR	T10	1.013	0.947
Etv6	Transcription factor ETV6	P97360	T18,S22	ISYTPPESPVASHR	T4,S8	0.957	0.274
Etv6	Transcription factor ETV6	P97360	Ambiguous	SSTPLHVHTVPR	S/T	0.954	0.486
Evi2b	Protein EVI2B	Q8VD58	S295	LFESSEHINDTSNLK	S4	0.987	0.944
Evi2b	Protein EVI2B	Q8VD58	T250	SPFADGETPEMCMDNIR	T8	1.060	0.320
Evi2b	Protein EVI2B	Q8VD58	T250	SPFADGETPEMCMDNIR	T8	1.088	0.338
Evi2b	Protein EVI2B	Q8VD58	S264,T/S	SPFADGETPEMCMDNIRESEASTKR	S22,T/S	0.838	0.456
Evi5	Ecotropic viral integration site 5 protein	F8VPT6	Ambiguous	SGPMSLNPALADGSESEAEDGMLGPQESDPEAPQK	S	1.197	0.269
Evl	Ena/VASP-like protein	P70429	S130	QVQNGPSPEEMDIQR	S7	1.050	0.089
Evl	Ena/VASP-like protein	P70429	S130	QVQNGPSPEEMDIQR	S7	0.967	0.208
Evl	Ena/VASP-like protein	P70429	S130	QVQNGPSPEEMDIQRR	S7	1.074	0.629
Evl	Ena/VASP-like protein	P70429	S130	QVQNGPSPEEMDIQRR	S7	0.993	0.902
Evl	Ena/VASP-like protein	P70429	S150	QVMEQQHRQESLER	S11	1.062	0.570
Evl	Ena/VASP-like protein	P70429	S240	RVQRPEDASGGSSPSGTSK	S9	0.858	0.037
Evl	Ena/VASP-like protein	P70429	S243	VQRPEDASGGSSPSGTSK	S11	1.010	0.629
Evl	Ena/VASP-like protein	P70429	S257	SDANRASSGGGGGGLMEEMNK	S7	0.936	0.041
Evl	Ena/VASP-like protein	P70429	S257	SDANRASSGGGGGGLMEEMNK	S7	0.949	0.121
Evl	Ena/VASP-like protein	P70429	S257	SDANRASSGGGGGGLMEEMNK	S7	0.920	0.234
Evl	Ena/VASP-like protein	P70429	S257	ASSGGGGGGLMEEMNK	S2	0.971	0.130
Evl	Ena/VASP-like protein	P70429	S302	AASQTDKPADRKEDESQTEDPSTSPSPGTR	S24	1.047	0.286
Evl	Ena/VASP-like protein	P70429	S347	TPSVAKSPEAK	S7	1.093	0.069
Evl	Ena/VASP-like protein	P70429	S352	SPEAKSPLQSQPHSR	S6	1.053	0.270
Evl	Ena/VASP-like protein	P70429	S240,S244	VQRPEDASGGSSPSGTSK	S8,S12	1.012	0.799
Evl	Ena/VASP-like protein	P70429	S294,S/T	KEDESQTEDPSTSPSPGTR	S5,S/T	1.353	0.144
Evl	Ena/VASP-like protein	P70429	S302,S/T	AASQTDKPADRKEDESQTEDPSTSPSPGTR	S24,S/T	1.027	0.841
Evl	Ena/VASP-like protein	P70429	S343,S347	TPSVAKSPEAK	S3,S7	1.086	0.400
Evl	Ena/VASP-like protein	P70429	Ambiguous	KEDESQTEDPSTSPSPGTR	S/T	1.042	0.504
Evl	Ena/VASP-like protein	P70429	Ambiguous	ATSQPPNSSEAGR	S/T	1.018	0.787
Evl	Ena/VASP-like protein	P70429	Ambiguous	ASSGGGGGGLMEEMNK	S	0.983	0.664
Evl	Ena/VASP-like protein	P70429	Ambiguous	EDESQTEDPSTSPSPGTR	T/S	1.172	0.017
Exoc1	Exocyst complex component 1	Q6P1Y9	S305	ALQEGDLVSSR	S10	0.957	0.582
Exoc1	Exocyst complex component 1	Q6P1Y9	S477	LTGSTSSLNK	S4	1.005	0.678
Exoc1	Exocyst complex component 1	Q6P1Y9	S496	SQSSSLLDMGNMSASDLDVADR	S3	0.956	0.193
Exoc1	Exocyst complex component 1	Q6P1Y9	S496	RSQSSSLLDMGNMSASDLDVADR	S4	0.961	0.749
Exoc1	Exocyst complex component 1	Q6P1Y9	S477,S480	LTGSTSSLNK	S4,S7	1.050	0.173
Exoc1	Exocyst complex component 1	Q6P1Y9	S508,S	RSQSSSLLDMGNMSASDLDVADR	S16,S	1.057	0.660
Exoc1	Exocyst complex component 1	Q6P1Y9	S508,S	SQSSSLLDMGNMSASDLDVADR	S15,S	1.124	0.498
Exoc1	Exocyst complex component 1	Q6P1Y9	Ambiguous	SQSSSLLDMGNMSASDLDVADR	S	1.088	0.165
Exoc1	Exocyst complex component 1	Q6P1Y9	Ambiguous	LQQHQNMSASMTEAEDLDGGSLLR	T/S	0.987	0.871
Exoc2	Exocyst complex component 2	Q9D4H1	S435	GSSFQSGRDDTWR	S6	0.988	0.788
Exoc4	Exocyst complex component 4	O35382	S32	TLSTSDDVEDRENEK	S3	1.307	0.000
Exoc4	Exocyst complex component 4	O35382	S469	SGELQGGPDDNLIEGGGTK	S1	1.064	0.147
Exoc4	Exocyst complex component 4	O35382	Ambiguous	ELYSRSGELQGGPDDNLIEGGGTK	S/Y/T	0.917	0.044
Exoc4	Exocyst complex component 4	O35382	Ambiguous	TASEPSAQLSYASTGR	T/S/Y	0.940	0.003
Exoc6	Exocyst complex component	Q3U9D6	S446	DIFEEDNYSPIPIGSEEEYK	S9	0.791	0.092
Exoc7	Exocyst complex component 7	O35250	S250	SSSSSGVPYSPAIPNKR	S10	0.919	0.083
Exoc7	Exocyst complex component 7	O35250	S250	SSSSSGVPYSPAIPNK	S10	0.954	0.365
Exoc7	Exocyst complex component 7	O35250	S250,S/Y	KSSSSSGVPYSPAIPNK	S11,S/Y	1.091	0.376
Exoc7	Exocyst complex component 7	O35250	Ambiguous	SSSSSGVPYSPAIPNK	Y/S	1.111	0.446
Exoc8	Exocyst complex component 8	Q6PGF7	S15	QLESGGFEAR	S4	1.017	0.648
Exoc8	Exocyst complex component 8	Q6PGF7	S15	RQLESGGFEAR	S5	0.975	0.487
Exoc8	Exocyst complex component 8	Q6PGF7	S28	QLSQQSDGDRDLQEHR	S3	1.338	0.014
Exoc8	Exocyst complex component 8	Q6PGF7	T313	GSNPFEDEDDEELATPEAEEEK	T15	0.795	0.298
Exosc1	Exosome complex component CSL4	Q9DAA6	S21	LCNLEEGSPGSGTYTR	S8	0.871	0.526
Exosc5	Exosome complex component RRP46	Q9CRA8	S20	ADANLLTDTGTESSPR	S14	0.982	0.569
Exosc5	Exosome complex component RRP46	Q9CRA8	S20	RADANLLTDTGTESSPR	S15	1.085	0.097
Exosc5	Exosome complex component RRP46	Q9CRA8	S23	ADANLLTDTGTESSPRSPVCSLR	S17	1.021	0.176
Exosc5	Exosome complex component RRP46	Q9CRA8	S23	RADANLLTDTGTESSPRSPVCSLR	S18	0.975	0.769
Exosc5	Exosome complex component RRP46	Q9CRA8	S23,T/S	RADANLLTDTGTESSPRSPVCSLR	S18,T/S	0.984	0.494
Exosc5	Exosome complex component RRP46	Q9CRA8	S23,T/S	ADANLLTDTGTESSPRSPVCSLR	S17,T/S	0.903	0.257
Exosc9	Exosome complex component RRP45	Q9JHI7	S306	METAPIDTSNIEER	S9	1.121	0.043
Extl3	Exostoses (Multiple)-like 3	Q6P1H4	S362	SFEEEMEGDPPADYDDR	S1	0.874	0.010
Extl3	Exostoses (Multiple)-like 3	Q6P1H4	S362	SFEEEMEGDPPADYDDR	S1	0.905	0.515
Extl3	Exostoses (Multiple)-like 3	Q6P1H4	S362	SSLQEARSFEEEMEGDPPADYDDR	S8	1.018	0.566
Eya1	Eyes absent homolog 1	P97767	S313	NNNPSPPPDSDLER	S5	1.077	0.347
Eya1	Eyes absent homolog 1	P97767	S313	RNNNPSPPPDSDLER	S6	1.016	0.955
Eya3	Eyes absent homolog	Q6P4T3	Ambiguous	LPSDSSASPPLSQTTPNKDADDQAR	S/T	1.074	0.720
Eya4	Eyes absent homolog	A0A1W2P721	S361	NNPSPPPDSDLER	S4	0.959	0.464
Eya4	Eyes absent homolog	A0A1W2P721	S361	KNNPSPPPDSDLER	S5	1.063	0.399
Ezh2	Histone-lysine N-methyltransferase EZH2	Q61188	T487	ESSIIAPVPTEDVDTPPR	T15	0.933	0.173
Ezh2	Histone-lysine N-methyltransferase EZH2	Q61188	T487	VKESSIIAPVPTEDVDTPPR	T17	1.041	0.826
F11r	Junctional adhesion molecule A	O88792	S288	VIYSQPSTRSEGEFK	S10	0.863	0.111
Fadd	FAS-associated death domain protein	Q61160	S191	SENMSPVLR	S5	1.072	0.509
Fadd	FAS-associated death domain protein	Q61160	S191	SENMSPVLR	S5	0.980	0.779
Fadd	FAS-associated death domain protein	Q61160	T204	DSTVSSSETP	T9	1.058	0.296
Faf1	FAS-associated factor 1	P54731	S581	TPSGEFLER	S3	1.035	0.692
Fah	Fumarylacetoacetase	P35505	S417	VLPALSPA	S6	1.064	0.583
Fam102a	Protein FAM102A	Q78T81	S190	GGGTSSGGSSSTNSLTGSRPPK	S14	1.071	0.636
Fam102a	Protein FAM102A	Q78T81	S251	SSSLSDLTHR	S2	0.934	0.190
Fam102a	Protein FAM102A	Q78T81	Ambiguous	VSSGVYEPVVIESH	S/Y	0.980	0.735

Fam102b	Protein FAM102B	E9Q4R1	S360	VGSGAYEQVVIK	S3	1.017	0.911
Fam104a	Family with sequence similarity 104, member A	A2A6P4	S82	NGSDDDNHPPPQTK	S3	0.973	0.689
Fam104a	Family with sequence similarity 104, member A	A2A6P4	S62,S64	RSPSPAAMSER	S2,S4	1.040	0.437
Fam104a	Family with sequence similarity 104, member A	A2A6P4	S62,S64	RSPSPAAMSER	S2,S4	1.031	0.541
Fam110a	Protein FAM110A	Q8R184	Ambiguous	ASSDIVSLAGPSAGPCSSEGGCSR	S	0.915	0.305
Fam111a	Protein FAM111A	Q9D2L9	S59	DQRPLSPR	S6	1.074	0.319
Fam111a	Protein FAM111A	Q9D2L9	T68	QDQTPPLNK	T4	1.071	0.285
Fam114a1	Protein Noxp20	Q9D281	T186	IHSANSASPEGAPTDAENGISGNVTPDQDPAR	T25	1.516	0.346
Fam117a	Protein FAM117A	Q7TNF9	S29	GCSPPAPAGSPR	S3	1.061	0.177
Fam117a	Protein FAM117A	Q7TNF9	S29	RGCSPPAPAGSPR	S4	1.160	0.443
Fam117a	Protein FAM117A	Q7TNF9	S412	NCPSSPGSPLPTASPR	S14	0.996	0.952
Fam117a	Protein FAM117A	Q7TNF9	S67	AASVPCSVAPEK	S3	0.962	0.391
Fam117a	Protein FAM117A	Q7TNF9	S402,S412	NCPSSPGSPLPTASPR	S4,S14	0.858	0.277
Fam117b	Protein FAM117B	Q3U3E2	S10	RNGSPTPAGALAGGAVGPPGGPGSR	S4	1.074	0.195
Fam117b	Protein FAM117B	Q3U3E2	S10	NGSPTPAGALAGGAVGPPGGPGSR	S3	1.128	0.145
Fam117b	Protein FAM117B	Q3U3E2	S102	TSPTVATQTGASVTSTR	S2	0.954	0.542
Fam117b	Protein FAM117B	Q3U3E2	S190	SPSAPVCK	S3	0.961	0.353
Fam117b	Protein FAM117B	Q3U3E2	S190	RSPSAPVCK	S4	1.057	0.260
Fam117b	Protein FAM117B	Q3U3E2	S268	SSSWGSTEQLK	S3	0.940	0.235
Fam117b	Protein FAM117B	Q3U3E2	S444	DKENGNNSPLPK	S8	0.935	0.414
Fam117b	Protein FAM117B	Q3U3E2	S479	VFEECSPK	S6	0.773	0.078
Fam117b	Protein FAM117B	Q3U3E2	Ambiguous	SIDTQTPGGADKGSNNSSR	S/T	0.954	0.503
Fam117b	Protein FAM117B	Q3U3E2	Ambiguous	GGGGSAAARTSPTVATQTGASVTSTR	T/S	0.882	0.236
Fam118a	Protein FAM118A	Q91YN1	S311	QRSPDAERVDSTTLLGNACQDCAK	S3	0.992	0.824
FAM120A	Constitutive coactivator of PPAR-gamma-like protein 1	Q6A0A9	S1021	SQGAIQGRPPYAASAEEVAK	S14	1.004	0.966
FAM120A	Constitutive coactivator of PPAR-gamma-like protein 1	Q6A0A9	S415	NLTEQNSYSNIPHEGK	S7	1.089	0.296
FAM120A	Constitutive coactivator of PPAR-gamma-like protein 1	Q6A0A9	T426	NLTEQNSYSNIPHEGKHTPLYER	T18	0.913	0.331
FAM120A	Constitutive coactivator of PPAR-gamma-like protein 1	Q6A0A9	T506,S511	GDPGDQVKVEGSSTASSGSQLAEGK	T14,S19	0.955	0.593
FAM120A	Constitutive coactivator of PPAR-gamma-like protein 1	Q6A0A9	Ambiguous	GDPGDQVKVEGSSTASSGSQLAEGK	S/T	0.849	0.381
Fam122a	Protein FAM122A	Q9DB52	S34	SNSAPLIHGLSDSSPVFQAEAPSAR	S3	0.882	0.489
Fam122a	Protein FAM122A	Q9DB52	S59	RNSTTFPSR	S3	1.092	0.318
Fam122a	Protein FAM122A	Q9DB52	S73	HGLLLPASPVR	S8	1.166	0.444
Fam122a	Protein FAM122A	Q9DB52	S140,S144	RIDFIPVSPAPSPTR	S8,S12	0.978	0.794
Fam122a	Protein FAM122A	Q9DB52	Ambiguous	SNSAPLIHGLSDSSPVFQAEAPSAR	S	0.756	0.072
Fam126a	Hyccin	Q6P9N1	S321	NAVTSMSIR	S7	1.041	0.401
Fam126a	Hyccin	Q6P9N1	S453	SFEQVSGAPVPR	S1	1.078	0.602
Fam126a	Hyccin	Q6P9N1	S467	SPSPAIGCVAGADANR	S3	0.968	0.701
Fam126a	Hyccin	Q6P9N1	S485	FSACSLQEEK	S5	0.913	0.237
Fam126a	Hyccin	Q6P9N1	T306	CIQVEITPTSSR	T7	1.036	0.707
Fam126a	Hyccin	Q6P9N1	S465,S	SPSPAIGCVAGADANRFSACSLQEEK	S1,S	0.894	0.305
Fam126b	Protein FAM126B	Q8C729	S321	TAITTASIRR	S7	0.973	0.486
Fam126b	Protein FAM126B	Q8C729	S398	SNESPRDSVVGK	S4	0.954	0.252
Fam126b	Protein FAM126B	Q8C729	S442	TNSLSLIR	S3	1.040	0.791
Fam126b	Protein FAM126B	Q8C729	S491	YSTISLQEDR	S5	0.990	0.685
Fam126b	Protein FAM126B	Q8C729	S521	SPSFNMQLISQV	S3	1.037	0.932
Fam126b	Protein FAM126B	Q8C729	T306	VEVTPTVPR	T4	1.130	0.011
Fam129a	Protein Niban	Q3UW53	S591	TAMGSNQASPAR	S5	0.852	0.069
Fam129a	Protein Niban	Q3UW53	S595	TAMGSNQASPAR	S9	0.979	0.672
Fam129a	Protein Niban	Q3UW53	S614	VSAILPGAPDNELPSNEVFQEPEEK	S15	0.923	0.809
Fam129a	Protein Niban	Q3UW53	S755	IHPEASHPAAIQQDSCEER	S15	0.967	0.628
Fam129a	Protein Niban	Q3UW53	S787	GSTSQSTSGGLTENTSCPGPIEEPFEAQEPAEK	S2	1.161	0.048
Fam129a	Protein Niban	Q3UW53	S591,S595	TAMGSNQASPAR	S5,S9	1.062	0.415
Fam129a	Protein Niban	Q3UW53	S591,S595	TAMGSNQASPAR	S5,S9	1.053	0.465
Fam129a	Protein Niban	Q3UW53	S591,S595	TAMGSNQASPARR	S5,S9	1.096	0.380
Fam129b	Niban-like protein 1	Q8R1F1	S148	SGSTPILK	S3	0.958	0.083
Fam129b	Niban-like protein 1	Q8R1F1	S628	QVMSVVQDEESGLPFEAGVEPPSPASPDSVTELR	S4	1.392	0.377
Fam129b	Niban-like protein 1	Q8R1F1	S628,S650	QVMSVVQDEESGLPFEAGVEPPSPASPDSVTELR	S4,S26	1.456	0.181
Fam133b	Protein FAM133B	Q9CVI2	S82	LLSGNESSSK	S3	0.998	0.926
Fam13a	Protein FAM13A	Q8BGI4	S322	SSSLGSYDDEQEDLTPVQLTR	S3	1.088	0.381
Fam149b	Family with sequence similarity 149, member B	A0A2C9F2B4	S231	EAIREPSPDGSLILDPR	S7	0.890	0.207
Fam149b1	Protein FAM149B1	Q6NSV7	S299	SHWEGCASDDESNVEITR	S8	1.199	0.018
Fam160a1	Protein FAM160A1	E9PX40	Ambiguous	QVLATGLSGLYSSLPTK	T/S/Y	1.004	0.976
Fam160a2	FTS and Hook-interacting protein	A0A1C7CYU5	S530	QQSLGGSESPGPVPR	S9	1.173	0.162
Fam160a2	FTS and Hook-interacting protein	A0A1C7CYU5	S537	SPGLTASPTSSPSR	S1	1.053	0.285
Fam160a2	FTS and Hook-interacting protein	A0A1C7CYU5	S914	DGTGLGLGGGSPGASTPVLLPR	S11	1.069	0.634
Fam160a2	FTS and Hook-interacting protein	A0A1C7CYU5	Ambiguous	EDITGPGSPSVDSSSVVTVPRPSTPSR	T/S	1.201	0.330
Fam160b1	Protein FAM160B1	Q8CDM8	S187	GALSVISEDGPK	S4	1.156	0.470
Fam168b	Myelin-associated neurite-outgrowth inhibitor	G3UWF0	S75	VSCSPTSGAVPPYSSSPNPYQTAVYPVR	S4	1.148	0.479
Fam171a2	Protein FAM171A2	A2A699	S785	RDSLTSPEDELGAEVGDEAGDKK	S3	0.973	0.696
Fam189b	Protein FAM189B	Q5HZJ5	S494	SHSDPGITTSSDIADFR	S3	0.936	0.534
Fam193a	Protein FAM193A (Fragment)	A0A0J9YTZ5	S128	SCSDDEDVAPLSAK	S3	1.069	0.151
Fam193a	Protein FAM193A	M0QWZ1	S1458	TSSSLDDVFLPK	S4	0.851	0.529
Fam193a	Protein FAM193A	M0QWZ1	T679	TEESNVNTPPPSYPAQQAEQTPNTCECHVCK	T8	1.103	0.417
Fam193a	Protein FAM193A	M0QWZ1	Ambiguous	NFQAATESISNSENIHNGSLEQTEEPETSSHSPSR	S/T	0.924	0.456
Fam193b	Protein FAM193B	Q3U2K0	S776	VGSGAEPGEGSPGSRPGPIQADSPK	S23	0.916	0.218
Fam193b	Protein FAM193B	Q3U2K0	S813	TLEQESEQTPGPARPQSLSQGK	S17	1.191	0.028
Fam206a	Protein Simiate	Q80ZQ9	T192	TNATATTP	T7	0.912	0.402
Fam207a	Protein FAM207A	P58468	S192	LLASPTYR	S4	1.138	0.024
Fam207a	Protein FAM207A	P58468	S38	AVEPALPQSPAGGAGAK	S9	1.150	0.036
Fam208a	Protein TASOR	Q69ZR9	S628	QQFSPLSDYEGQEEELNGSK	S4	0.852	0.028
Fam208a	Protein TASOR	Q69ZR9	S653	NNSRDETTEPEQQK	S3	1.050	0.576
Fam208a	Protein TASOR	Q69ZR9	S836	DAQSPLLEVDAASVK	S4	1.065	0.557
Fam20c	Extracellular serine/threonine protein kinase FAM20C	Q5MJS3	S47	SSGEPGCSCAQPAAEAAGPGWAQAR	S8	0.879	0.391
Fam20c	Extracellular serine/threonine protein kinase FAM20C	Q5MJS3	S563	DCVEKDGLSSVVEDDLATEHR	S10	1.122	0.464
Fam20c	Extracellular serine/threonine protein kinase FAM20C	Q5MJS3	S576	DCVEKDGLSSVVEDDLATEHRASTER	S23	1.045	0.518
Fam20c	Extracellular serine/threonine protein kinase FAM20C	Q5MJS3	S576	DGLSSVVEDDLATEHRASTER	S18	1.032	0.658
Fam214b	Protein FAM214B	Q8BR27	S9	HVQAELSPSSEPEAGPSQPPVR	S7	1.226	0.002
Fam219a	2310028H24Rik protein	A2ANP1	S55	NGSMGSPVNQQPK	S6	1.003	0.972
Fam222b	Protein FAM222B	Q6P539	S296	AGISTTSVCEGQIANPSPISR	S17	0.937	0.708
Fam241a	Uncharacterized protein FAM241A	Q9CZL2	S38	VEEPEHGASPR	S9	1.092	0.483
Fam50b	Protein FAM50B	Q9WTJ8	T7	GTMREAGRAMHLIK	T2	1.119	0.227
Fam53a	Protein FAM53A	E9PV82	S119	SLSEPEELAR	S3	0.954	0.570
Fam53a	Protein FAM53A	E9PV82	S13	LQNQSLDDLTR	S5	0.905	0.024
Fam53a	Protein FAM53A	E9PV82	S265	CRSQPCVLDGR	S3	0.983	0.831
Fam53a	Protein FAM53A	E9PV82	S306	SLCSLDYEDDEDDTQEK	S1	0.973	0.841
Fam53a	Protein FAM53A	E9PV82	S306,S309	NSKSLCSLDYEDDEDDTQEK	S4,S7	0.936	0.356
Fam53a	Protein FAM53A	E9PV82	Ambiguous	NSKSLCSLDYEDDEDDTQEK	S/Y/T	0.927	0.344
Fam53b	Protein FAM53B	Q8BGR5	S119	SLSFSDEMSSCR	S3	1.094	0.005
Fam53b	Protein FAM53B	Q8BGR5	S119	SLSFSDEMSSCR	S3	1.070	0.382
Fam53b	Protein FAM53B	Q8BGR5	S167	SSSFSLPAR	S2	0.972	0.124
Fam53c	Protein FAM53C	Q8BXQ8	S122	SLSVPVDLSR	S3	0.903	0.265
Fam53c	Protein FAM53C	Q8BXQ8	S162	GNAGGGGPQVPQQSPPK	S14	1.310	0.331
Fam53c	Protein FAM53C	Q8BXQ8	S273	SRSQPCDLDAR	S3	0.932	0.246
Fam53c	Protein FAM53C	Q8BXQ8	Ambiguous	SSPASSPELPWRPR	S	1.055	0.747
Fam76a	Protein FAM76A	Q922G2	S306	SGTITSP	S6	0.996	0.932
Fam76b	Protein FAM76B	Q80XP8	S193	VSSLSPEQEQGLWK	S5	1.018	0.491
Fam76b	Protein FAM76B	Q80XP8	S338	SGSVLTSP	S7	0.962	0.453
Fam76b	Protein FAM76B	Q80XP8	T215	SSAAIQNETPK	T9	0.897	0.211
Fam83f	Expressed sequence AW544981	A0A0R4J033	S477	HNEGSNANISGK	S5	0.962	0.581
Fam83f	Expressed sequence AW544981	A0A0R4J033	S477,S482	HNEGSNANISGK	S5,S10	1.067	0.425
Fam83f	Expressed sequence AW544981	A0A0R4J033	Ambiguous	IGLNYSSTVAR	S/T/Y	0.954	0.517
Fam83f	Expressed sequence AW544981	A0A0R4J033	Ambiguous	QALCAAWSPYEEAVSSTR	S/Y/T	1.256	0.300
Fam83h	Protein FAM83H	Q148V8	S871;S1750;S2640	KGSPTPAYPER	S3	1.011	0.783
Fam83h	Protein FAM83H	Q148V8	S871;S1751;S2642	GSPTPAYPER	S2	0.952	0.557
Fam83h	Protein FAM83H	Q148V8	S904	KGSPTQAYPER	S3	0.994	0.927
Fam83h	Protein FAM83H	Q148V8	S926	RGSPTTGLMEQK	S3	1.025	0.647
Fam83h	Protein FAM83H	Q148V8	S948;S1904	RGSPVPPVPER	S3	1.053	0.541
Fam83h	Protein FAM83H	Q148V8	S948;S1905	GSPVPPVPERR	S2	1.052	0.507
Fam83h	Protein FAM83H	Q148V8	S948;S1905	GSPVPPVPER	S2	0.947	0.652
Fam83h	Protein FAM83H	Q148V8	S970	GSLTFAGESSK	S2	0.980	0.827
Fam83h	Protein FAM83H	Q148V8	S970	RGSLTFAGESSK	S3	1.009	0.826
Fam83h	Protein FAM83H	Q148V8	S835,S841	SLSPQGRDSPPPEGLGTHQLPYSEPK	S3,S9	1.021	0.845
Fam83h	Protein FAM83H	Q148V8	Ambiguous	LEYVPSSASREVR	S/Y	1.117	0.127
Fam84b	Family with sequence similarity 84, member B	D3YXJ5	S291	TTPSPLRPPASGSEEENGDSVVH	S4	0.930	0.558
Fam91a1	Protein FAM91A1	Q3UVG3	S332	MLLSWDGGESR	S4	1.133	0.267
Fam98c	Family with sequence similarity 98, member C	E9PYD1	S129	DSSPVPSFGEGTK	S3	0.960	0.217
Fam98c	Family with sequence similarity 98, member C	E9PYD1	S129	DSSPVPSFGEGTKK	S3	0.958	0.584
Fancd2	Fanconi anemia group D2 protein homolog	Q80V62	Ambiguous	ASCSDTLLTEDTSECDMAPSGR	S/T	0.688	0.257
Fancm	Fanconi anemia group M protein homolog	Q8BGE5	S1368	SSDQVFVHQSPR	S10	1.036	0.802
Farp1	FERM, ARHGEF and pleckstrin domain-containing protein 1	F8VPU2	S373	SLVSQPTAPNSEVPK	S1	0.943	0.585
Farp1	FERM, ARHGEF and pleckstrin domain-containing protein 1	F8VPU2	S512	QASPLISPLLNDQACPR	S3	1.000	1.000
Farp1	FERM, ARHGEF and pleckstrin domain-containing protein 1	F8VPU2	Ambiguous	LGAPENSGISTLER	S/T	1.096	0.264
Farp2	FERM, ARHGEF and pleckstrin domain-containing protein 2	D3Z4C0	S491	GQSLNAVDISK	S3	0.955	0.821
Farp2	FERM, ARHGEF and pleckstrin domain-containing protein 2	Q91VS8	S863	TIGDSPPVLLGGPVYTR	S5	0.891	0.153
Farp2	FERM, ARHGEF and pleckstrin domain-containing protein 2	Q91VS8; D3Z4C0	Ambiguous	LGGQTAIGVSTLEPEQSLSPR	S/T	0.978	0.876
Farsa	Phenylalanine--tRNA ligase alpha subunit	Q8C0C7	S193	QEAELSPEMISSGSWR	S6	0.987	0.937
Fbf1	Fas-binding factor 1	A2A870	S206	SPGAAAGQGPSVPLTPGDTPIR	S1	0.871	0.080
Fbf1	Fas-binding factor 1	A2A870	Ambiguous	QSSPPASSPIHPR	S	1.257	0.185

Fbh1	F-box DNA helicase 1	Q8K2I9	S172	QGAEDSLSQCSPVPGEAGR	S11	0.945	0.520
Fbn1	Fibrillin-1	Q61554	S2704	GGPEPPASSEMDDNSLSPEACYECK	S8	0.978	0.861
Fbn1	Fibrillin-1	Q61554	S2704	GGPEPPASSEMDDNSLSPEACYECK	S8	1.079	0.220
Fbxl19	F-box/LRR-repeat protein 19	Q6PB97	S235	GADPGSPGLLPPR	S6	1.030	0.522
Fbxl20	F-box/LRR-repeat protein 20	Q9CZV8	T417	VHAYFAPVTPPPSVGGSR	T9	1.020	0.830
Fbxl20	F-box/LRR-repeat protein 20	Q9CZV8	T417,S421	VHAYFAPVTPPPSVGGSR	T9,S13	1.015	0.805
Fbxl4	F-box/LRR-repeat protein 4	Q8BH70	Ambiguous	TSPLNAEVVQYAK	T/S/Y	1.034	0.910
Fbxo11	F-box only protein 11	Q7TPD1	S100	NNAGERDDVPADMVAEESGPGAQNSPYQLR	S25	1.113	0.200
Fbxo28	F-box only protein 28	Q8BIG4	S344	EVMESAVGTSSGSGQSEESPR	S19	1.059	0.726
Fbxo31	F-box only protein 31	Q3TQF0	S33,T37	RGPAETAAADSEADTDPEEERIEAGPAR	S11,T15	0.917	0.454
Fbxo38	F-box only protein 38	Q8BMI0	S708	DVYPSCSSSSSSTAASTAGNASSPSTASQSPDFAR	S8	1.205	0.370
Fbxo38	F-box only protein 38	Q8BMI0	S746	TVTSSGSSEPSPPEVDVSR	S11	1.097	0.344
Fbxo4	F-box only protein 4	Q8CHQ0	S11	GAGSPPPASDWGR	S4	1.021	0.204
Fbxo42	F-box only protein 42	Q6PDJ6	S488	RGSLPDQK	S3	0.974	0.400
Fbxo46	F-box only protein 46	Q8BG80	S273	EPHSPDGNLPNGGGGRPGCAYPGSPGPGTR	S4	0.792	0.095
Fbxo9	F-box only protein 9	Q8BK06	Ambiguous	ITYTRSPDGDGVGSGYIEENEDASK	T/S/Y	0.930	0.456
Fbxw8	F-box/WD repeat-containing protein 8	Q8BIA4	Ambiguous	SRSPPDRDATEPEPLVDQLIR	S/T	0.937	0.553
Fbxw9	F-box and WD-40 domain protein 9	F8VPX2	S18	SCEEESDPEPDPDPDTQAEAYVAR	S6	1.092	0.291
Fcer1g	High affinity immunoglobulin epsilon receptor subunit gamma	P20491	S72	SQETYETLK	S1	0.955	0.386
Fcer1g	High affinity immunoglobulin epsilon receptor subunit gamma	P20491	T66	ADAVYTGLNTR	T6	0.973	0.712
Fcer1g	High affinity immunoglobulin epsilon receptor subunit gamma	P20491	T78	SQETYETLKHEKPPQ	T7	1.035	0.868
Fcer1g	High affinity immunoglobulin epsilon receptor subunit gamma	P20491	T75,T78	SQETYETLKHEKPPQ	T4,T7	1.012	0.820
Fcer1g	High affinity immunoglobulin epsilon receptor subunit gamma	P20491	Ambiguous	EKADAVYTGLNTRSQETYETLK	S/T/Y	1.038	0.686
Fcer1g	High affinity immunoglobulin epsilon receptor subunit gamma	P20491	Ambiguous	EKADAVYTGLNTR	Y/T	0.937	0.162
Fcgr1	High affinity immunoglobulin gamma Fc receptor I	P26151	S339	YNLEVPLVSEQGK	S9	0.847	0.327
Fcgr1	High affinity immunoglobulin gamma Fc receptor I	P26151	S347	ANSFQQVR	S3	1.051	0.329
Fcgr1	High affinity immunoglobulin gamma Fc receptor I	P26151	S347	KANSFQQVR	S4	1.038	0.503
Fcgr1	High affinity immunoglobulin gamma Fc receptor I	P26151	S365	SDGVYEEVTATASQTTPK	S13	0.979	0.681
Fcgr1	High affinity immunoglobulin gamma Fc receptor I	P26151	T368	SDGVYEEVTATASQTTPKEAPDGPR	T16	0.968	0.604
Fcgr1	High affinity immunoglobulin gamma Fc receptor I	P26151	Y357,T/S	SDGVYEEVTATASQTTPK	Y5,T/S	0.875	0.485
Fcgr1	High affinity immunoglobulin gamma Fc receptor I	P26151	Ambiguous	SSVGDCGPEQPEPLPPSDSTGAQTSQS	S/T	1.042	0.430
Fcgr1	High affinity immunoglobulin gamma Fc receptor I	P26151	Ambiguous	EAPDGPRSSVGDCGPEQPEPLPPSDSTGAQTSQS	S/T	1.021	0.764
Fcgr2b	Fc receptor, IgG, low affinity IIb	A0A0B4J1E6	Ambiguous	HPEALDEETEHDYQNHI	T/Y	0.916	0.599
Fcho1	F-BAR domain only protein 1	Q8K285	S518	APSPGPWGPEGGADSLTPADPTR	S3	1.033	0.720
Fcho1	F-BAR domain only protein 1	Q8K285	Ambiguous	FSSSDSDFDDEEPR	S	0.902	0.403
Fcho2	F-BAR domain only protein 2	Q3UQN2	S507	AESSSSISSSASLSAANTPTVGVSR	S3	0.951	0.349
Fcho2	F-BAR domain only protein 2	Q3UQN2	S681	CSASTTDLR	S4	1.011	0.800
Fcho2	F-BAR domain only protein 2	Q3UQN2	S507,S/T	AESSSSISSSASLSAANTPTVGVSR	S3,S/T	0.931	0.517
Fcho2	F-BAR domain only protein 2	Q3UQN2	Ambiguous	NSSNEELTK	S/T	0.974	0.456
Fchsd2	F-BAR and double SH3 domains protein 2	Q3USJ8	S683	SPSANENSLHAESPGFSQASR	S3	1.102	0.535
Fer	Tyrosine-protein kinase Fer	P70451	S716	QEDGGVYSSSGLK	S8	1.005	0.905
Fer	Tyrosine-protein kinase Fer	P70451	Y402	VQENDGKEPPPVVNYEEDAR	Y15	0.889	0.128
Fer	Tyrosine-protein kinase Fer	P70451	Y402,S/T	VQENDGKEPPPVVNYEEDARSVTSMER	Y15,S/T	1.046	0.620
Fermt3	Fermitin family homolog 3	Q8K1B8	S8	TASGDYIDSSWELR	S3	0.974	0.628
Fermt3	Fermitin family homolog 3	Q8K1B8	Ambiguous	AGGSNGGSGNKPQGPEAPAEGLNPYGLVAPR	S/Y	0.962	0.850
Fes	Tyrosine-protein kinase Fes/Fps	P16879	T421	EGGRTPTLEILK	T5	1.054	0.558
Fes	Tyrosine-protein kinase Fes/Fps	P16879	Y713	EEADGIYAASAGLR	Y7	0.980	0.721
Fez2	Fasciculation and elongation protein zeta-2	D3Z6D5	S130	GMNDGLLFDASDEEELR	S11	1.132	0.318
Fez2	Fasciculation and elongation protein zeta-2	D3Z6D5	S200	RSSMSSYEER	S3	1.049	0.621
Fgd2	FYVE, RhoGEF and PH domain-containing protein 2	Q8BY35	S654	TPEGPDEEDVSD	S11	0.977	0.707
Fgd2	FYVE, RhoGEF and PH domain-containing protein 2	Q8BY35	S654	AASGRTPEGPDEEDVSD	S16	1.021	0.779
Fgd2	FYVE, RhoGEF and PH domain-containing protein 2	Q8BY35	S93	SCQPGVSPGPETQEPEEK	S2	0.951	0.467
Fgd2	FYVE, RhoGEF and PH domain-containing protein 2	Q8BY35	S39,S/T	TPGEAPGSHSLEDQPHSPEHQLSLSPEPWEAPPVK	S17,S/T	1.094	0.487
Fgd3	FYVE, RhoGEF and PH domain containing 3, isoform CRA_b	Q3TNB8	S124	VPQDNPQEEEDSGMGEEPDPK	S12	1.065	0.735
Fgd3	FYVE, RhoGEF and PH domain containing 3, isoform CRA_b	Q3TNB8	S543	SCGETFNSITK	S8	0.906	0.155
Fgd3	FYVE, RhoGEF and PH domain containing 3, isoform CRA_b	Q3TNB8	S589	ECFLEEPLVPPSPSSETPTELK	S12	0.957	0.836
Fgd3	FYVE, RhoGEF and PH domain containing 3, isoform CRA_b	Q3TNB8	T732	ALGTAVHGDTAGDRPGASQPQAPAGTDTP	T28	0.849	0.497
Fgd6	FYVE, RhoGEF and PH domain-containing protein 6	Q69ZL1	S1167	ITFCPSRSLDEDSER	S8	1.064	0.436
Fgd6	FYVE, RhoGEF and PH domain-containing protein 6	Q69ZL1	S1257	LDHQLSPR	S6	1.146	0.185
Fgd6	FYVE, RhoGEF and PH domain-containing protein 6	Q69ZL1	S156	GSSWDSSSEK	S3	0.914	0.225
Fgd6	FYVE, RhoGEF and PH domain-containing protein 6	Q69ZL1	S494	AASEELVEK	S3	0.958	0.200
Fgd6	FYVE, RhoGEF and PH domain-containing protein 6	Q69ZL1	S583	SLSAVDADR	S1	0.997	0.944
Fgd6	FYVE, RhoGEF and PH domain-containing protein 6	Q69ZL1	S583	AKSLSAVDADR	S3	0.983	0.502
Fgd6	FYVE, RhoGEF and PH domain-containing protein 6	Q69ZL1	S670	AHSAENCSLESQK	S3	1.053	0.229
Fgd6	FYVE, RhoGEF and PH domain-containing protein 6	Q69ZL1	Y717	HTSCTGDFGPEYENVR	Y12	1.005	0.888
Fhdc1	FH2 domain-containing protein 1	Q3ULZ2	Ambiguous	SSSENDVQMLAK	S	0.963	0.491
Fhl3	Four and a half LIM domains protein 3	Q9R059	Ambiguous	RPITGGSGGGEGAGLGGGK	T/S	0.982	0.712
Fhod1	FH1/FH2 domain-containing protein 1	Q6P9Q4	S370	SLEGGGCPVR	S1	0.953	0.747
Fhod1	FH1/FH2 domain-containing protein 1	Q6P9Q4	S490	ELWDSPEPASAPR	S5	1.177	0.343
Fhod1	FH1/FH2 domain-containing protein 1	Q6P9Q4	S502	TPQSPVSR	S4	1.043	0.414
Fhod1	FH1/FH2 domain-containing protein 1	Q6P9Q4	S514	TQRSLEPEPK	S4	1.297	0.010
Fhod1	FH1/FH2 domain-containing protein 1	Q6P9Q4	T499,S502	ELWDSPEPASAPRTPQSPVSR	T14,S17	1.393	0.008
Fig4	Polyphosphoinositide phosphatase	Q91WF7	T777	STPVKMTDTGDSAK	T2	1.052	0.627
Fignl1	Fidgetin-like protein 1	Q8BPY9	S374	AGSAEPAHLTDDCLK	S3	0.927	0.265
Filip1l	Filamin A-interacting protein 1-like	E0CYH7	S1047	SNSNSSSVITTEDNK	S3	1.178	0.021
Filip1l	Filamin A-interacting protein 1-like	E0CYH7	T992	AQTPESCGSVTPER	T11	1.027	0.900
Fip1l1	Pre-mRNA 3'-end-processing factor FIP1	Q9D824	S280	AESPDLR	S3	1.044	0.157
Fip1l1	Pre-mRNA 3'-end-processing factor FIP1	Q9D824	S280	YGRAESPDLR	S6	0.953	0.335
Fip1l1	Pre-mRNA 3'-end-processing factor FIP1	Q9D824	S479	DHSPTPSVFNSDEER	S3	1.027	0.376
Fip1l1	Pre-mRNA 3'-end-processing factor FIP1	Q9D824	S479	ERDHSPTPSVFNSDEER	S5	1.030	0.241
Fip1l1	Pre-mRNA 3'-end-processing factor FIP1	Q9D824	S479	ERERDHSPTPSVFNSDEER	S7	1.045	0.664
Fip1l1	Pre-mRNA 3'-end-processing factor FIP1	Q9D824	S566	EAGSEPVPEQESTEAAPAE	S4	0.974	0.856
Fip1l1	Pre-mRNA 3'-end-processing factor FIP1	Q9D824	S566	SKEGKEAGSEPVPEQESTEAAPAE	S9	1.019	0.815
Fip1l1	Pre-mRNA 3'-end-processing factor FIP1	Q9D824	S479,S487	DHSPTPSVFNSDEER	S3,S11	0.956	0.486
Fip1l1	Pre-mRNA 3'-end-processing factor FIP1	Q9D824	S479,S487	DHSPTPSVFNSDEERYR	S3,S11	1.000	0.997
Fip1l1	Pre-mRNA 3'-end-processing factor FIP1	Q9D824	S479,S487	ERDHSPTPSVFNSDEER	S5,S13	1.000	1.000
Fkbp15	FK506-binding protein 15	Q6P9Q6	S1087	EVATDGLLQGNSR	S12	0.972	0.839
Fkbp15	FK506-binding protein 15	Q6P9Q6	S1091	RLSLTPDPEK	S3	0.942	0.480
Fkbp15	FK506-binding protein 15	Q6P9Q6	S1091	RLSLTPDPEKGEPPALDPESQGGEAQPPECK	S3	1.057	0.600
Fkbp15	FK506-binding protein 15	Q6P9Q6	S1127	QAEDVSSSGPR	S8	0.955	0.406
Fkbp15	FK506-binding protein 15	Q6P9Q6	S1159	HNQDSQHCSLSGDEEDELFK	S11	0.975	0.870
Fkbp15	FK506-binding protein 15	Q6P9Q6	S1190	VPRPTAQPEEEDEDEVSMK	S17	0.967	0.573
Fkbp15	FK506-binding protein 15	Q6P9Q6	S344	SNSLSEQLTVNSNPDTVK	S3	0.962	0.637
Fkbp15	FK506-binding protein 15	Q6P9Q6	S617	NNSLQTATENTQAR	S3	0.872	0.668
Fkbp15	FK506-binding protein 15	Q6P9Q6	S1091,T1093	RLSLTPDPEKGEPPALDPESQGGEAQPPECK	S3,T5	0.892	0.188
Fkbp15	FK506-binding protein 15	Q6P9Q6	Ambiguous	MGQPMLPILPPQLDSNDSETEDATVLR	T/S	1.142	0.603
Flcn	Folliculin	Q8QZS3	S62	AHSPAEGASSESSSPGPK	S3	0.957	0.403
Flcn	Folliculin	Q8QZS3	S62,S68	AHSPAEGASSESSSPGPK	S3,S9	0.954	0.560
Fli1	Friend leukemia integration 1 transcription factor	P26323	S39	ADMTASGSPDYGQPHK	S8	0.994	0.836
Fli1	Friend leukemia integration 1 transcription factor	P26323	S39	ADMTASGSPDYGQPHK	S8	1.034	0.341
Fli1	Friend leukemia integration 1 transcription factor	P26323	S79	EYDHMNGSRESPVDCSVSK	S11	1.001	0.990
Flii	Protein flightless-1 homolog	Q9JJ28	S436	KDSAQDVQAK	S3	1.077	0.042
Flna	Filamin-A	Q8BTM8	S1084	AFGPGLQGGNAGSPAR	S13	1.025	0.567
Flna	Filamin-A	Q8BTM8	S1459	CSGPGLSPGMVR	S7	0.981	0.623
Flna	Filamin-A	Q8BTM8	S1459	CSGPGLSPGMVR	S7	1.013	0.744
Flna	Filamin-A	Q8BTM8	S16	CGQSAAVASPGGSIDSR	S9	1.081	0.322
Flna	Filamin-A	Q8BTM8	S1630	YGGDEIPFSPYR	S9	1.028	0.593
Flna	Filamin-A	Q8BTM8	S310	SAGQGEVLVYVEDPAGHQEEAK	S1	1.194	0.311
Flna	Filamin-A	Q8BTM8	S512	GAGSGELK	S4	1.005	0.949
Flna	Filamin-A	Q8BTM8	T1639	AVPTGDASK	T4	1.005	0.971
Flnb	Filamin-B	Q80X90	S1505	YADEEIPRSPFK	S9	0.894	0.509
Flrt2	Leucine-rich repeat transmembrane protein FLRT2	Q8BLU0	S650	YCNSSVPDLEHCHT	S4	1.020	0.188
Flvcr1	Feline leukemia virus subgroup C receptor-related protein 1	B2RXV4	S553	VMVSIQSESSL	S4	0.994	0.983
Fmn1	Formin-1	Q05860	S797	DVCISTDDDCSPK	S11	1.047	0.572
Fmnl1	Formin-like protein 1	A0A1W2P6X3	S1027	EAAADTSGREEPPTPKSPPK	S17	1.030	0.672
Fmnl1	Formin-like protein 1	A0A1W2P6X3	S1027	EEPPTPKSPPK	S8	1.063	0.513
Fmnl1	Formin-like protein 1	A0A1W2P6X3	S1027	EEPPTPKSPPKAR	S8	0.996	0.938
Fmnl1	Formin-like protein 1	A0A1W2P6X3	T515	GPGDVVSIEILPGAAATPSGDDAQAPR	T17	1.143	0.493
Fmnl2	Formin-like protein 2	A2APV2	S1015	LLEQEALMEQQDAKSPSHK	S15	0.927	0.089
Fmnl2	Formin-like protein 2	A2APV2	S1075	VRISSSTPVVEDTQS	S4	1.063	0.142
Fmnl2	Formin-like protein 2	A2APV2	S1076	ISSSTPVVEDTQS	S3	1.034	0.542
Fmnl2	Formin-like protein 2	A2APV2	S171	SIEDLHR	S1	1.012	0.831
Fmnl2	Formin-like protein 2	A2APV2	S183	GSNLPSPVGNSVSR	S6	0.955	0.142
Fmr1	Synaptic functional regulator FMR1	E9QAS8	T462	GYVTDDGQGMGR	T4	0.990	0.865
Fnbp1	Formin-binding protein 1	Q80TY0	S296	TVSDNSLSSSK	S3	0.976	0.559
Fnbp1	Formin-binding protein 1	Q80TY0	S296	RTVSDNSLSSSK	S4	0.890	0.235
Fnbp1	Formin-binding protein 1	Q80TY0	S496	RQSGLYDGQTHQTVTNCAQDR	S3	0.919	0.375
Fnbp1	Formin-binding protein 1	Q80TY0	S296,S299	RTVSDNSLSSSK	S4,S7	1.029	0.528
Fnbp1	Formin-binding protein 1	Q80TY0	S296,S299	TVSDNSLSSSK	S3,S6	1.010	0.781
Fnbp1l	Formin-binding protein 1-like	E9PUI5	S295	TISDGTISAAK	S3	1.001	0.985
Fnbp1l	Formin-binding protein 1-like	E9PUI5	S501	HSSDINHLVTQGRESPEGSYTDDANQEVR	S15	1.162	0.022
Fnbp1l	Formin-binding protein 1-like	E9PUI5	S525	GPPQQHGHHSEFDDEFEDDDPLPAIGHCK	S10	0.961	0.606
Fnbp4	Formin-binding protein 4	A0A0R4IZZ6	S166	ATGGLCLLGAYADSDDDESDVSEK	S14	0.928	0.526
Fnbp4	Formin-binding protein 4	A0A0R4IZZ6	S486	ALEEGDGSVSGSSPR	S13	1.053	0.091
Fnbp4	Formin-binding protein 4	A0A0R4IZZ6	S533	TGRDSPENGETAIGAEDSEK	S5	1.043	0.518
Fnbp4	Formin-binding protein 4	A0A0R4IZZ6	S553	IDEISDKETEVEESSEK	S5	1.023	0.856
Fnbp4	Formin-binding protein 4	A0A0R4IZZ6	S481,S	ALEEGDGSVSGSSPR	S8,S	1.096	0.695
Fndc3a	Fibronectin type-III domain-containing protein 3A	Q8BX90	S213	CPSPISEHNGLIK	S3	1.123	0.472

Fndc3a	Fibronectin type-III domain-containing protein 3A	Q8BX90	S244	GRSCTQVDPEMEEKDEETK	S3	1.429	0.027
Fndc3a	Fibronectin type-III domain-containing protein 3A	Q8BX90	S244	GRSCTQVDPEMEEKDEETK	S3	1.435	0.031
Fndc3a	Fibronectin type-III domain-containing protein 3A	Q8BX90	Ambiguous	MSSPPPSPQK	S	1.069	0.475
Fndc3a	Fibronectin type-III domain-containing protein 3A	Q8BX90	Ambiguous	MSSPPPSPQK	S	1.031	0.606
Fndc3b	Fibronectin type III domain-containing protein 3B	A0A0R4J0H8	S208	LNSPPSTIYK	S3	1.012	0.793
Fndc3b	Fibronectin type III domain-containing protein 3B	A0A0R4J0H8	S263	SSDSDLQEYELEVK	S4	0.923	0.510
Fndc3b	Fibronectin type III domain-containing protein 3B	A0A0R4J0H8	Ambiguous	SSPKSSDSDLQEYELEVK	S/Y	1.117	0.046
Fnip1	Folliculin-interacting protein 1	Q68FD7	S292	SQTTSLENGVFPR	S1	1.013	0.742
Fnip1	Folliculin-interacting protein 1	Q68FD7	S945	NESSDSALGDSESEDTGPDIR	S11	1.000	1.000
Fnip1	Folliculin-interacting protein 1	Q68FD7	S945	NAVGTEWDIPRNESSDSALGDSESEDTGPDIRR	S22	0.895	0.076
Fnip1	Folliculin-interacting protein 1	Q68FD7	S945	NAVGTEWDIPRNESSDSALGDSESEDTGPDIR	S22	0.972	0.679
Fnip1	Folliculin-interacting protein 1	Q68FD7	S945	NESSDSALGDSESEDTGPDIRR	S11	0.939	0.162
Fnip2	Folliculin-interacting protein 2	D3YUC5	S313	RSTDETFSLAEETCSSNPAMVR	S2	1.090	0.692
Fnip2	Folliculin-interacting protein 2	D3YUC5	T302	SQTTSLENGIFPR	T4	0.876	0.484
Fnip2	Folliculin-interacting protein 2	D3YUC5	Ambiguous	ADFEKDITPQDHSSGGEGVSEDR	S/T	0.927	0.313
Fopnl	LisH domain-containing protein FOPNL	Q9CZS3	S173	TTTEELPAAAQAVSR	S14	0.937	0.376
Fos	Proto-oncogene c-Fos	P01101	Ambiguous	GSSSNEPSSDSLSSPTLLAL	S/T	1.012	0.908
Fosb	Protein fosB	P13346	Ambiguous	TSGSEQPSDPLNSPSLLAL	S/T	1.034	0.526
Fosl1	Fos-related antigen 1	P48755	Ambiguous	SSSSSGDPSSDPLGSPTLLAL	T/S	0.803	0.540
Fosl2	Fos-related antigen 2	P47930	S200	SPPTSGLQSLR	S1	1.021	0.635
Fosl2	Fos-related antigen 2	P47930	S200	RSPPTSGLQSLR	S2	0.967	0.624
Fosl2	Fos-related antigen 2	P47930	S320	SSSSGDQSSDSLNSPTLLAL	S14	0.934	0.581
Fosl2	Fos-related antigen 2	P47930	Ambiguous	RSSSSGDQSSDSLNSPTLLAL	S/T	0.817	0.300
Fosl2	Fos-related antigen 2	P47930	Ambiguous	GSSGSPAHAESYSSGGGGQK	S/Y	1.109	0.455
Fosl2	Fos-related antigen 2	P47930	Ambiguous	QEPPEEDSPSSSAGMDK	S	1.057	0.586
Fosl2	Fos-related antigen 2	P47930	Ambiguous	RSSSSGDQSSDSLNSPTLLAL	S/T	1.229	0.210
Foxd2	Forkhead box protein D2	O35392	S96	GAAAAAGSPGPGVQAAR	S8	1.022	0.864
Foxj2	Forkhead box protein J2	Q9ES18	Ambiguous	HPPDDDLSQDSPEQEASKSPR	S	0.975	0.540
Foxj2	Forkhead box protein J2	Q9ES18	Ambiguous	RHPPDDDLSQDSPEQEASKSPR	S	0.988	0.939
Foxj3	Forkhead box protein J3	Q8BUR3	S223	VTLYNADQDGSDSPR	S13	1.026	0.729
Foxk1	Forkhead box protein K1	P42128	S406	SAPASPTHPGLMSPR	S5	1.052	0.159
Foxk1	Forkhead box protein K1	P42128	S431	EGSPIPHDPDLGSK	S3	1.039	0.496
Foxk1	Forkhead box protein K1	P42128	S87	QSPGPALAR	S2	1.087	0.019
Foxk1	Forkhead box protein K1	P42128	T422	SSGLQTPECLSR	T6	0.971	0.496
Foxk1	Forkhead box protein K1	P42128	S402,S406	SAPASPTHPGLMSPR	S1,S5	0.962	0.520
Foxk1	Forkhead box protein K1	P42128	S402,S406	SAPASPTHPGLMSPR	S1,S5	0.998	0.964
Foxk1	Forkhead box protein K1	P42128	S431,T/S	SSGLQTPECLSREGSPIPHDPDLGSK	S15,T/S	1.108	0.356
Foxk1	Forkhead box protein K1	P42128	S402,S406,S414	SAPASPTHPGLMSPR	S1,S5,S13	0.991	0.773
Foxk1	Forkhead box protein K1	P42128	S402,S406,S414	SAPASPTHPGLMSPR	S1,S5,S13	1.131	0.058
Foxk1	Forkhead box protein K1	P42128	T422,S427,S431	SSGLQTPECLSREGSPIPHDPDLGSK	T6,S11,S15	1.078	0.402
Foxk2	Forkhead box protein K2	Q3UCQ1	S389	EGSPAPLEPEPGASQPK	S3	1.068	0.146
Foxn2	Forkhead box N2	E9Q7L6	S271	SYGSAFSAPGAVR	S1	0.818	0.055
Foxn2	Forkhead box N2	E9Q7L6	S289	LQESPSPTAGIDPK	S6	1.088	0.253
Foxn3	Forkhead box protein N3	Q499D0	S319	STSPTSDSISSSSSSADDHYEFATK	S3	1.107	0.808
Foxn3	Forkhead box protein N3	Q499D0	S85,T/S/Y	SVSPVQDLDDDTPPSPAHSDMPYDAR	S3,T/S/Y	1.220	0.021
Foxo1	Forkhead box protein O1	Q9R1E0	S467	ELLTSDSPPHNDIMSPVDPGVAQPNSR	S7	1.100	0.463
Foxo3	Forkhead box protein O3	Q9WVH4	S252	AVSMDNSNK	S3	1.075	0.246
Foxo3	Forkhead box protein O3	Q9WVH4	S252	AVSMDNSNK	S3	1.094	0.319
Foxo3	Forkhead box protein O3	Q9WVH4	S252	AVSMDNSNKYTK	S3	0.977	0.658
Foxo3	Forkhead box protein O3	Q9WVH4	S283	AALQAAPESADDSPSQLSK	S13	1.018	0.655
Foxo3	Forkhead box protein O3	Q9WVH4	S310	SSDELDAWTDFRSR	S13	0.932	0.360
Fra10ac1	Protein FRA10AC1 homolog	Q8BP78	S243	TTPECDESPR	S8	1.118	0.275
Fra10ac1	Protein FRA10AC1 homolog	Q8BP78	S250	SRSPPSEEASK	S3	0.997	0.965
Fra10ac1	Protein FRA10AC1 homolog	Q8BP78	S283	NAEEEDSASDSELWK	S7	1.143	0.439
Fra10ac1	Protein FRA10AC1 homolog	Q8BP78	S285	NAEEEDSASDSELWKGPLPETDEK	S9	0.921	0.501
Fra10ac1	Protein FRA10AC1 homolog	Q8BP78	S248,S250	SRSPPSEEASK	S1,S3	0.956	0.770
Frat1	Proto-oncogene FRAT1	P70339	S274	TGDDLLVPGS	S10	1.111	0.411
Frmd4a	FERM domain-containing protein 4A	H3BIZ7	S694	SVDISPTR	S5	0.970	0.549
Frmd4a	FERM domain-containing protein 4A	H3BIZ7	S727	LLGSENDTGSPDFYTPR	S10	0.789	0.270
Frmd4a	FERM domain-containing protein 4A	H3BIZ7	S800	AAGALGSASSGSMPNLAAR	S12	0.962	0.342
Frmd4a	FERM domain-containing protein 4A	H3BIZ7	S800	AAGALGSASSGSMPNLAAR	S12	1.229	0.204
Frmd4a	FERM domain-containing protein 4A	H3BIZ7	Ambiguous	SSSLESQGK	S	0.929	0.271
Frmd4a	FERM domain-containing protein 4A	H3BIZ7	Ambiguous	TSNSYTAGGLFK	T/S/Y	1.131	0.134
Frmd4b	FERM domain-containing protein 4B	Q920B0	S372	SLDDIAMDLTETGTQR	S1	0.970	0.901
Frmd4b	FERM domain-containing protein 4B	Q920B0	S663	SMPTTPVLTR	S1	1.090	0.391
Frmd4b	FERM domain-containing protein 4B	Q920B0	S663,T	SMPTTPVLTR	S1,T	0.807	0.342
Frmd4b	FERM domain-containing protein 4B	Q920B0	S663,T	RPQGGRSMPTTPVLTR	S7,T	0.949	0.159
Frmd4b	FERM domain-containing protein 4B	Q920B0	S663,T667	SMPTTPVLTR	S1,T5	0.906	0.363
Frmd4b	FERM domain-containing protein 4B	Q920B0	T666,T667	RPQGGRSMPTTPVLTR	T10,T11	1.068	0.665
Frmd6	FERM domain-containing protein 6	Q8C0V9	S511	EIGSSTSSSSETVVR	S9	0.971	0.448
Frmd6	FERM domain-containing protein 6	Q8C0V9	S544	HSLSLDDIR	S4	0.996	0.952
Frmd6	FERM domain-containing protein 6	Q8C0V9	S544	TSTDRHSLSLDDIR	S9	0.939	0.113
Frmd6	FERM domain-containing protein 6	Q8C0V9	S522,S525	LRGQSTDSLPQTICR	S5,S8	1.035	0.527
Frmd8	FERM domain-containing protein 8	Q3UFK8	S447	QLSSSQGSYTVVQPTDDSLEQS	S3	1.133	0.291
Frmd8	FERM domain-containing protein 8	Q3UFK8	S21,S23	SSVSSVGAR	S2,S4	0.996	0.960
Frmd8	FERM domain-containing protein 8	Q3UFK8	Ambiguous	SSVSSVGAR	S	0.970	0.191
Frs2	Fibroblast growth factor receptor substrate 2	Q8C180	S327	LTSTSTSDTQNINNSAQR	S3	0.981	0.870
Frs2	Fibroblast growth factor receptor substrate 2	Q8C180	S503	HNSTDLPM	S3	1.063	0.399
Frs2	Fibroblast growth factor receptor substrate 2	Q8C180	Ambiguous	TPTTPLPQTPTR	T	1.023	0.731
Fry	Protein furry homolog	E9Q8I9	S1936	NSDLFTVLSR	S9	0.961	0.662
Fry	Protein furry homolog	E9Q8I9	S1940	SSSPDLSSSSK	S3	1.053	0.271
Fry	Protein furry homolog	E9Q8I9	S1955	STGQLNVNPGTPGSGGGGGGSGNTTTAER	S1	1.028	0.812
Fry	Protein furry homolog	E9Q8I9	S2815	ATFAGGSRDGVITCQPGDSEEK	S7	0.901	0.040
Fry	Protein furry homolog	E9Q8I9	Ambiguous	ASTPEIMATTPDGTVSYDNK	S/T/Y	1.111	0.554
Fry	Protein furry homolog	E9Q8I9	Ambiguous	ASTPEIMATTPDGTVSYDNK	S/T/Y	0.886	0.476
Fryl	FRY-like transcription coactivator	F8VQ05	S890	CSPPETLASTPDSGYSIDSK	S2	0.915	0.478
Fryl	FRY-like transcription coactivator	F8VQ05	T1960	SNTLDITDGR	T3	0.971	0.635
Fryl	FRY-like transcription coactivator	F8VQ05	T1960	RSNTLDITDGR	T4	1.038	0.608
Fryl	FRY-like transcription coactivator	F8VQ05	Ambiguous	TRSLSSLR	S/T	0.988	0.863
Fryl	FRY-like transcription coactivator	F8VQ05	Ambiguous	IPSVTSGTTSSSNTMVAPTDGNPDSK	S/T	0.953	0.716
Fryl	FRY-like transcription coactivator	F8VQ05	Ambiguous	DGDPPMPTTGVILPSGNTLR	T/S	1.035	0.708
Fsd1l	FSD1-like protein	Q8BYN5	Ambiguous	SENGMTGSTSSLNNVTQ	S/T	1.064	0.652
Fubp1	Far upstream element-binding protein 1	A0A0G2JGW9	S136	IQIAPDSGGLPER	S7	0.898	0.430
Fubp1	Far upstream element-binding protein 1	A0A0G2JGW9	S411	IELQRSPPPNADPNMK	S6	0.987	0.884
Fubp1	Far upstream element-binding protein 1	A0A0G2JGW9	S411	IELQRSPPPNADPNMK	S6	0.998	0.954
Fubp1	Far upstream element-binding protein 1	A0A0G2JGW9	S629	QQAAYYAQTSPQGMPQHPPAPQGQ	S10	1.010	0.473
Fubp1	Far upstream element-binding protein 1	A0A0G2JGW9	Ambiguous	QQAAYYAQTSPQGMPQHPPAPQGQ	T/S/Y	1.040	0.285
Fubp3	Far upstream element (FUSE)-binding protein 3	A2AJ72	S566	QQAAFYGQTLGQAQAHSQEQ	S17	1.096	0.495
Fubp3	Far upstream element (FUSE)-binding protein 3	Q3TIX6	Ambiguous	QQAAFYGQTLGQAQAHSQVCSQSPAP	S/Y/T	1.033	0.646
Fundc1	FUN14 domain-containing protein 1	Q9DB70	S13	NPPPQDYESDDESYEVLDLTEYAR	S9	0.893	0.241
Fxr1	Fragile X mental retardation syndrome-related protein 1	A0A0G2JEP0	S553	RTDEDAVLMDGLTESDTASVNENGLGKR	S15	0.886	0.175
Fxr1	Fragile X mental retardation syndrome-related protein 1	Q61584; E9QAS8; A0A0G2JEP0; Q6P5B5	S11;S11;S11;S21	GSNGAFYK	S2	0.936	0.485
Fxr1	Fragile X mental retardation syndrome-related protein 1	Q61584; A0A0G2JEP0	S438;S438	RGPNYTSGYGTNSELSNPSETESER	S19	0.986	0.891
Fxr1	Fragile X mental retardation syndrome-related protein 1	Q61584; A0A0G2JEP0	S449;S449	KDELSDWSLAGEDDRETR	S5	0.996	0.965
Fxr1	Fragile X mental retardation syndrome-related protein 1	Q61584; A0A0G2JEP0	T398;T398	EKGYATDESTVSSVQGSR	T6	1.096	0.231
Fxr1	Fragile X mental retardation syndrome-related protein 1	Q61584; A0A0G2JEP0	S435,S438;S438	RGPNYTSGYGTNSELSNPSETESER	S16,S19	0.993	0.896
Fxr1	Fragile X mental retardation syndrome-related protein 1	Q61584; A0A0G2JEP0	S449,S452,S452	KDELSDWSLAGEDDRETR	S5,S8	0.934	0.181
Fxr1	Fragile X mental retardation syndrome-related protein 1	Q61584	Ambiguous	AINGPTSASGDEIPK	S/T	1.021	0.857
Fxr1	Fragile X mental retardation syndrome-related protein 1	A0A0G2JEP0	Ambiguous	TDEDAVLMDGLTESDTASVNENGLGK	T/S	1.328	0.212
Fxr2	Fragile X mental retardation syndrome-related protein 2	Q6P5B5	S455	TGGPAYGPSSDPSTASETESEKREESNR	S16	0.931	0.532
Fxr2	Fragile X mental retardation syndrome-related protein 2	Q6P5B5	S455	RTGGPAYGPSSDPSTASETESEKR	S17	1.078	0.427
Fxr2	Fragile X mental retardation syndrome-related protein 2	Q6P5B5	S455	TGGPAYGPSSDPSTASETESEK	S16	0.947	0.494
Fxr2	Fragile X mental retardation syndrome-related protein 2	Q6P5B5	S568	TVMDGGLESDGPNMTENGLEDESRPQR	S9	0.907	0.243
Fxr2	Fragile X mental retardation syndrome-related protein 2	Q6P5B5	S568	TVMDGGLESDGPNMTENGLEDESRPQR	S9	1.060	0.365
Fxr2	Fragile X mental retardation syndrome-related protein 2	Q6P5B5	S568	TVMDGGLESDGPNMTENGLEDESRPQRR	S9	0.970	0.561
Fxr2	Fragile X mental retardation syndrome-related protein 2	Q6P5B5	S568	TVMDGGLESDGPNMTENGLEDESRPQRR	S9	0.976	0.882
Fxr2	Fragile X mental retardation syndrome-related protein 2	Q6P5B5	S603	GNRTDGSISGDRQPVTVADYISR	S7	1.009	0.908
Fxr2	Fragile X mental retardation syndrome-related protein 2	Q6P5B5	S605	TDGSISGDRQPVTVADYISR	S6	1.045	0.320
Fxr2	Fragile X mental retardation syndrome-related protein 2	Q6P5B5	S603,S605	TDGSISGDRQPVTVADYISR	S4,S6	0.990	0.846
Fxr2	Fragile X mental retardation syndrome-related protein 2	Q6P5B5	S603,S605	GNRTDGSISGDRQPVTVADYISR	S7,S9	1.015	0.677
Fxr2	Fragile X mental retardation syndrome-related protein 2	Q6P5B5	Ambiguous	TGGPAYGPSSDPSTASETESEKR	S/T/Y	1.101	0.232
Fxr2	Fragile X mental retardation syndrome-related protein 2	Q6P5B5	Ambiguous	GGSGGGSDKAGYTTDESSSSSLHTTR	T/Y/S	1.218	0.525
Fxyd5	FXYD domain-containing ion transport regulator 5	P97808	Ambiguous	DNVPDPDQTSPGVQTTPLIWTR	S/T	1.063	0.834
Fyb1	FYN-binding protein 1	O35601	S201	HTFGQKPSLSTEDSQEENTSK	S8	1.461	0.010
Fyb1	FYN-binding protein 1	O35601	S222	GSPVQLGAK	S2	1.047	0.487
Fyb1	FYN-binding protein 1	O35601	S28	VAGQSSPSGIQSR	S6	1.006	0.849
Fyb1	FYN-binding protein 1	O35601	S46	NLFDNQGNASPPAGPSSMPK	S10	1.071	0.479
Fyb1	FYN-binding protein 1	O35601	S546	GSYGYIK	S2	1.028	0.674
Fyb1	FYN-binding protein 1	O35601	S546	TARGSYGYIK	S5	0.919	0.325
Fyb1	FYN-binding protein 1	O35601	S561	TTAVEIDYDSLKR	S10	0.934	0.257
Fyb1	FYN-binding protein 1	O35601	Y559	TTAVEIDYDSLK	Y8	0.978	0.614
Fyb1	FYN-binding protein 1	O35601	Y559,S561	TTAVEIDYDSLKR	Y8,S10	0.992	0.778
Fyb1	FYN-binding protein 1	O35601	Ambiguous	QLDVGEEVYDDVDASDFPPPPAEMSQGMSVGR	S/Y	1.058	0.735
Fyb1	FYN-binding protein 1	O35601	Ambiguous	VSESDNNEGSSLPSQHK	S	1.061	0.742
Fyb1	FYN-binding protein 1	O35601	Ambiguous	SYLVDNDGEIYDDIADGCIYDND	Y/S	0.968	0.922
Fyco1	FYVE and coiled-coil domain-containing protein 1	Q8VDC1	S329	EYSPSALQLENMAK	S3	0.916	0.038
Fyn	Tyrosine-protein kinase Fyn	P39688	S21	DGSLNQSSGYR	S3	1.002	0.988
Fyn	Tyrosine-protein kinase Fyn	P39688	S21	LTEERDGSLNQSSGYR	S8	0.980	0.837

Fzr1	Fizzy-related protein homolog	Q9R1K5	S70	INENEKSPSQNR	S7	0.995	0.950
G3bp1	Ras GTPase-activating protein-binding protein 1	P97855	S149	YQDEVFGGFVTEPQEESEEEVEEPEER	S17	1.043	0.438
G3bp1	Ras GTPase-activating protein-binding protein 1	P97855	S231	STSPAPADVAPAQEDLR	S3	1.000	0.988
G3bp2	Ras GTPase-activating protein-binding protein 2	P97379	Ambiguous	SATPPPAEPASLPQEPPK	S/T	1.012	0.734
G6pdx	Glucose-6-phosphate 1-dehydrogenase X	Q00612	Y503	VGFQYEGTYK	Y5	0.937	0.213
G6pdx	Glucose-6-phosphate 1-dehydrogenase X	Q00612	Ambiguous	VQPNEAVYTK	Y/T	1.029	0.344
Gab1	GRB2-associated-binding protein 1	A0A1B0GS41	S253	LTSVSGESSLYNLPR	S5	1.000	1.000
Gab1	GRB2-associated-binding protein 1	A0A1B0GS41	S266	SYSHDVLPK	S3	1.050	0.707
Gab1	GRB2-associated-binding protein 1	A0A1B0GS41	S420	SSSLEGFHSQYK	S3	1.060	0.132
Gab1	GRB2-associated-binding protein 1	A0A1B0GS41	S548	TDSQTIGDFATR	S3	1.069	0.292
Gab1	GRB2-associated-binding protein 1	A0A1B0GS41	S714	EAWTDGRQSTESETPTK	S9	1.026	0.789
Gab1	GRB2-associated-binding protein 1	A0A1B0GS41	Y690	VDYVVVDQQK	Y3	1.023	0.697
Gab1	GRB2-associated-binding protein 1	A0A1B0GS41	S501,T504	SSPKTPPRRPVPVADCEPPPVDR	S2,T5	1.043	0.359
Gab1	GRB2-associated-binding protein 1	A0A1B0GS41	Ambiguous	SSGSGSSMADERVDYVVVDQQK	S/Y	0.997	0.975
Gab2	GRB2-associated-binding protein 2	Q3ZB57	S143	SSPAEFSSSQHLLR	S2	1.009	0.887
Gab2	GRB2-associated-binding protein 2	Q3ZB57	S211	SASFSQGTR	S3	0.998	0.939
Gab2	GRB2-associated-binding protein 2	Q3ZB57	S261	HNTEFKDSTYDLPR	S8	0.983	0.836
Gab2	GRB2-associated-binding protein 2	Q3ZB57	S282	SSLTGSETDNEDVYTFK	S6	0.958	0.596
Gab2	GRB2-associated-binding protein 2	Q3ZB57	S401	LHRASSCETYEYPAR	S5	0.876	0.005
Gab2	GRB2-associated-binding protein 2	Q3ZB57	S402	ASSCETYEYPAR	S3	1.131	0.114
Gab2	GRB2-associated-binding protein 2	Q3ZB57	S413	GSGESASWSAEPPGK	S2	1.065	0.202
Gab2	GRB2-associated-binding protein 2	Q3ZB57	T388	RNTLPAMDNSR	T3	1.003	0.972
Gab2	GRB2-associated-binding protein 2	Q3ZB57	T388	RNTLPAMDNSR	T3	1.001	0.988
Gab2	GRB2-associated-binding protein 2	Q3ZB57	Ambiguous	GPSRGSEIQPPPVNR	S	1.014	0.879
Gab3	GRB2-associated-binding protein 3	Q8BSM5	S399	FSPIYPTASPSAEDSYVPMSPK	S20	0.950	0.796
Gabpb2	GA-binding protein subunit beta-2	P81069	S218	ATSAHLEEMEEGNSLDSSTQQVVGSGGQR	S14	0.797	0.045
Galk1	Galactokinase	Q9R0N0	S230	HSLGSSEYPVR	S2	1.015	0.900
Galnt2	Polypeptide N-acetylgalactosaminyltransferase 2	Q6PB93	S93	SGQDPYAR	S1	1.009	0.842
Galnt7	N-acetylgalactosaminyltransferase 7	Q80VA0	S103	HAGGDSQRDVMQR	S6	1.066	0.018
Ganab	Neutral alpha-glucosidase AB	Q8BHN3	Ambiguous	MLDYLQGSGETPQTDIR	S/T/Y	1.231	0.307
Gapdh	Glyceraldehyde-3-phosphate dehydrogenase	A0A0A0MQF6; S4R1W1	S234;S208	GAAQNIIPASTGAAK	S10	0.993	0.868
Gapdh	Glyceraldehyde-3-phosphate dehydrogenase	A0A0A0MQF6; S4R1W1	Y342;Y316	LISWYDNEYGYSNR	Y9	0.852	0.526
Gapvd1	GTPase-activating protein and VPS9 domain-containing protein 1	Q6PAR5	S466	SSSLDMTPYSTPQMSPATTPANK	S15	0.818	0.188
Gapvd1	GTPase-activating protein and VPS9 domain-containing protein 1	Q6PAR5	S746	LQELESCSGLGSTSDDTDVR	S12	1.033	0.718
Gapvd1	GTPase-activating protein and VPS9 domain-containing protein 1	Q6PAR5	S902	SSDIVSSVR	S1	1.011	0.608
Gapvd1	GTPase-activating protein and VPS9 domain-containing protein 1	Q6PAR5	S903	SRSSDIVSSVR	S4	1.037	0.312
Gapvd1	GTPase-activating protein and VPS9 domain-containing protein 1	Q6PAR5	S914	RPMSDPSWNR	S4	1.057	0.630
Gapvd1	GTPase-activating protein and VPS9 domain-containing protein 1	Q6PAR5	Ambiguous	EEALQNISADDLPDSASQAAHPQDSAFSYR	S/Y	0.714	0.096
Gapvd1	GTPase-activating protein and VPS9 domain-containing protein 1	Q6PAR5	Ambiguous	RTSPSEGAMANDESAEVMGDGESAHDSPR	T/S	1.052	0.768
Gars	Glycine--tRNA ligase	Q9CZD3	S690	AEVSELPNVVR	S4	1.117	0.313
Gas2	Growth arrest-specific protein 2	P11862	S186	EIEQEETLSAPSPSPSPSSK	S14	1.096	0.240
Gas2l1	GAS2-like protein 1	Q8JZP9	S306	GSPTPSPRPGSPVPGSER	S11	1.041	0.398
Gas2l1	GAS2-like protein 1	Q8JZP9	S306	GSPTPSPRPGSPVPGSERR	S11	1.065	0.644
Gas2l1	GAS2-like protein 1	Q8JZP9	S316	SSRPEVTPISLR	S2	1.021	0.853
Gas2l1	GAS2-like protein 1	Q8JZP9	S352	RYSGDSDSSASSAQSGPMGAR	S3	1.159	0.102
Gas2l1	GAS2-like protein 1	Q8JZP9	S352	RYSGDSDSSASSAQSGPMGAR	S3	0.989	0.941
Gas2l1	GAS2-like protein 1	Q8JZP9	S436	SQSREEQAVLMVR	S1	0.852	0.012
Gas2l1	GAS2-like protein 1	Q8JZP9	S436	TRSQSREEQAVLMVR	S3	0.829	0.056
Gas2l1	GAS2-like protein 1	Q8JZP9	S482	ALSPAAPRPSR	S3	1.039	0.364
Gas2l1	GAS2-like protein 1	Q8JZP9	S493	GPSPGPELAATPASIFR	S3	1.139	0.230
Gas2l1	GAS2-like protein 1	Q8JZP9	S292,S297	TGTFSPQRGSPTPSPRPGSPVPGSER	S5,S10	0.982	0.819
Gas2l1	GAS2-like protein 1	Q8JZP9	S297,S306	GSPTPSPRPGSPVPGSER	S2,S11	1.019	0.723
Gas2l1	GAS2-like protein 1	Q8JZP9	S352,S355	RYSGDSDSSASSAQSGPMGAR	S3,S6	1.021	0.866
Gas2l1	GAS2-like protein 1	Q8JZP9	S292,S297,T/S	TGTFSPQRGSPTPSPRPGSPVPGSERR	S5,S10,T/S	0.955	0.507
Gas2l1	GAS2-like protein 1	Q8JZP9	S292,S297,T/S	TGTFSPQRGSPTPSPRPGSPVPGSER	S5,S10,T/S	0.938	0.461
Gas7	Growth arrest-specific protein 7	Q3U432	S57	YYVNTTTNETTWERPSSSPGISASPGPHR	S24	1.147	0.219
Gas7	Growth arrest-specific protein 7	Q3U432	S57	RYYVNTTTNETTWERPSSSPGISASPGPHR	S25	1.005	0.963
Gas7	Growth arrest-specific protein 7	Q3U432	S92	STGDSQNLGSSSPGR	S1	1.016	0.501
Gas7	Growth arrest-specific protein 7	Q3U432	S92	KSTGDSQNLGSSSPGR	S2	0.941	0.507
Gas7	Growth arrest-specific protein 7	Q3U432	S102,S103	STGDSQNLGSSSPGRK	S11,S12	1.004	0.943
Gas7	Growth arrest-specific protein 7	Q3U432	Ambiguous	STGDSQNLGSSSPGRK	S/T	1.044	0.382
Gatad2a	Transcriptional repressor p66 alpha	E9QMN5	S510	LAFRSGEAR	S5	1.002	0.976
Gatad2a	Transcriptional repressor p66 alpha	E9QMN5	S69	TTEAMGTGSGEGLLGDGPVDMR	S9	0.885	0.292
Gatad2a	Transcriptional repressor p66 alpha	E9QMN5	S96	RPPSPDVIVLSDSEQPSSPR	S4	1.003	0.976
Gatad2a	Transcriptional repressor p66 alpha	E9QMN5	T185	EATAQKPTASSGSTVTTPPPLVR	T17	1.024	0.696
Gatad2a	Transcriptional repressor p66 alpha	E9QMN5	T20	TLEPDLTEDDVENK	T7	0.930	0.352
Gatad2a	Transcriptional repressor p66 alpha	E9QMN5	S96,S	RPPSPDVIVLSDSEQPSSPR	S4,S	0.984	0.623
Gatad2a	Transcriptional repressor p66 alpha	E9QMN5	Ambiguous	GTAVASAQANSTPTSVASVVASAESPASR	S/T	0.752	0.127
Gatad2b	Transcriptional repressor p66-beta	Q8VHR5	S136	LTPSPDIIVLSDNEASSPR	S17	0.856	0.468
Gatad2b	Transcriptional repressor p66-beta	Q8VHR5	S335	VSSPLPSPSAMSDAANSQAAAK	S3	0.978	0.628
Gatad2b	Transcriptional repressor p66-beta	Q8VHR5	T121	GRLTPSPDIIVLSDNEASSPR	T4	1.180	0.549
Gatad2b	Transcriptional repressor p66-beta	Q8VHR5	S123,S130	LTPSPDIIVLSDNEASSPR	S4,S11	1.010	0.912
Gatad2b	Transcriptional repressor p66-beta	Q8VHR5	S335,S339	VSSPLPSPSAMSDAANSQAAAK	S3,S7	1.085	0.294
Gatad2b	Transcriptional repressor p66-beta	Q8VHR5	S339,S	VSSPLPSPSAMSDAANSQAAAK	S7,S	0.986	0.934
Gatad2b	Transcriptional repressor p66-beta	Q8VHR5	T121,S130	GRLTPSPDIIVLSDNEASSPR	T4,S13	1.052	0.643
Gatad2b	Transcriptional repressor p66-beta	Q8VHR5	T121,S135,S136	GRLTPSPDIIVLSDNEASSPR	T4,S18,S19	1.065	0.704
Gatad2b	Transcriptional repressor p66-beta	Q8VHR5	Ambiguous	VSSPLPSPSAMSDAANSQAAAK	S	1.051	0.772
Gatad2b	Transcriptional repressor p66-beta	Q8VHR5	Ambiguous	TTTPNMNPAISYQPQSSSSVPCQR	T/S/Y	1.006	0.956
Gba2	Non-lysosomal glucosylceramidase	Q69ZF3	S893	RPSVTQGTGLSTQPECGPK	S3	0.974	0.856
Gba2	Non-lysosomal glucosylceramidase	Q69ZF3	S917	SLANLNSE	S7	0.919	0.584
Gbf1	Golgi-specific brefeldin A-resistance factor 1	Q6DFZ1	S1298	ADAPDAGAQSDSELPSYHQNDVSLDR	S10	1.020	0.875
Gbf1	Golgi-specific brefeldin A-resistance factor 1	Q6DFZ1	S1318	GYTSDSEVYTDHGRPGK	S4	1.021	0.787
Gbf1	Golgi-specific brefeldin A-resistance factor 1	Q6DFZ1	S1782	APSSSSPGSPMASSPSK	S5	1.056	0.250
Gcfc2	GC-rich sequence DNA-binding factor 2	Q8BKT3	S169	SNEEDPESDPDDHEKR	S8	0.936	0.448
Gcfc2	GC-rich sequence DNA-binding factor 2	Q8BKT3	S418,S419	ELSGSCDHQEGMSSDDELSPAEMTNFHK	S13,S14	1.065	0.591
Gcfc2	GC-rich sequence DNA-binding factor 2	Q8BKT3	S85,T86	TAELSTDEEEGTHTLTGSK	S5,T6	0.970	0.410
Gch1	GTP cyclohydrolase 1	Q05915	S24	ELPRPGASPPAEK	S8	1.038	0.367
Gcsh	Glycine cleavage system H protein, mitochondrial	Q91WK5	S157	MTLSDPSEMDELMSEEAYEK	S14	0.837	0.074
Gcsh	Glycine cleavage system H protein, mitochondrial	Q91WK5	S157	MTLSDPSEMDELMSEEAYEK	S14	1.122	0.329
Gda	Guanine deaminase	Q9R111	S453	QVVPFSSSV	S8	1.068	0.632
Gdap2	Ganglioside-induced differentiation-associated protein 2	Q9DBL2	S495	ENGPYFASYPPSPDL	S12	0.979	0.944
Gemin4	Gem (Nuclear organelle) associated protein 4	Q6P6L6	S84	VLQPSPVSPSDTDTR	S8	1.627	0.010
Gemin5	Gem-associated protein 5	Q8BX17	S1414	APSQPPSPTEER	S7	0.928	0.652
Gemin5	Gem-associated protein 5	Q8BX17	S48	VGPGAGASPGAPPFR	S8	0.944	0.122
Gemin5	Gem-associated protein 5	Q8BX17	S852	SMLPLSTSLDHR	S8	1.057	0.657
Gemin5	Gem-associated protein 5	Q8BX17	Ambiguous	DEPSRDEPSQEAERAPSQPPSPTEER	S/T	0.964	0.829
Gfpt1	Glutamine--fructose-6-phosphate aminotransferase [isomerizing] 1	P47856	S259	VDSTTCLFPVEEK	S3	1.035	0.256
Gga2	ADP-ribosylation factor-binding protein GGA2	Q6P5E6	S235	RVSAVEEVR	S3	0.982	0.767
Ggnbp2	Gametogenetin-binding protein 2	Q5SV77	S360	LEQLCEEFSEEERVR	S9	0.891	0.334
Ggnbp2	Gametogenetin-binding protein 2	Q5SV77	S360	LEQLCEEFSEEER	S9	1.004	0.911
Ggta1	Glycoprotein galactosyltransferase alpha 1, 3, isoform CRA_e	Q9DBU1	S20	SKSETSLPSSR	S3	0.969	0.165
Gigyf1	GRB10-interacting GYF protein 1	Q99MR1	S137	SIEEGDGAFGR	S1	1.222	0.011
Gigyf1	GRB10-interacting GYF protein 1	Q99MR1	S157	SQSWDDRGER	S3	1.054	0.649
Gigyf1	GRB10-interacting GYF protein 1	Q99MR1	S343	GLEEEEEEEEEPSEGVDEERPEAGGK	S13	1.079	0.296
Gigyf1	GRB10-interacting GYF protein 1	Q99MR1	S413	ISSPPGPPGDLEDEEGLK	S3	1.044	0.188
Gigyf1	GRB10-interacting GYF protein 1	Q99MR1	S21,S28	ALSSGGSVASPPPSPAMPK	S7,S14	1.020	0.925
Gigyf1	GRB10-interacting GYF protein 1	Q99MR1	Ambiguous	ALSSGGSVASPPPSPAMPK	S	1.060	0.715
Gigyf2	GRB10-interacting GYF protein 2 (Fragment)	G3UZS9; Q6Y7W8	S39;S237	WRPHSPDGPR	S5	0.995	0.961
Gigyf2	GRB10-interacting GYF protein 2 (Fragment)	G3UZS9; Q6Y7W8	S4;S202	SESENWR	S3	1.734	0.000
Gigyf2	GRB10-interacting GYF protein 2 (Fragment)	G3UZS9	Ambiguous	AGAASEEVPQTSLSSARPGTPSDHQPQEATQFER	T/S	0.968	0.529
Gigyf2	GRB10-interacting GYF protein 2	Q6Y7W8	Ambiguous	AGAEASEEVPQTSLSSARPGTPSDHQPQEATQFER	T/S	1.020	0.872
Gins2	DNA replication complex GINS protein PSF2	Q9D600	S182	TNLQPSESTQSQDF	S11	1.043	0.403
Gipc1	PDZ domain-containing protein GIPC1	Q9Z0G0	Ambiguous	SRGPATVEDLPSAFEEK	S/T	1.454	0.006
Gipc1	PDZ domain-containing protein GIPC1	Q9Z0G0	Ambiguous	SAGGHPGSGPQLGTGRGTLR	T/S	1.172	0.092
Git1	ARF GTPase-activating protein GIT1	Q68FF6	S371	SLSSPTDNLELSAR	S4	0.991	0.908
Git1	ARF GTPase-activating protein GIT1	Q68FF6	S394	SQSELDDQHDYDSVASDEDTDQEPLPSAGATR	S13	0.985	0.775
Git1	ARF GTPase-activating protein GIT1	Q68FF6	S601	HGSGADSDYENTQSGDPLLGLEGK	S3	1.034	0.142
Git1	ARF GTPase-activating protein GIT1	Q68FF6	S601	HGSGADSDYENTQSGDPLLGLEGKR	S3	1.034	0.847
Git1	ARF GTPase-activating protein GIT1	Q68FF6	Ambiguous	GVSASSVPFTPSSPLLSCSQEGSR	T/S	1.120	0.471
Git2	ARF GTPase-activating protein GIT2	E9PVA6	S397	TVSTQHSTESQDNDQPDYDSVASDEDTDVETR	S23	0.958	0.564
Git2	ARF GTPase-activating protein GIT2	E9PVA6	S510	GSRPMSMYETGSGQKPYLPMGEASHPEESR	S2	1.047	0.406
Git2	ARF GTPase-activating protein GIT2	E9PVA6	S514	GSRPMSMYETGSGQKPYLPMGEASHPEESR	S6	0.934	0.554
Git2	ARF GTPase-activating protein GIT2	E9PVA6	S586	LEKQNSTPESDYDNTACDPEPDDTGSTR	S6	1.202	0.435
Git2	ARF GTPase-activating protein GIT2	E9PVA6	S586	QNSTPESDYDNTACDPEPDDTGSTRK	S3	1.339	0.026
Git2	ARF GTPase-activating protein GIT2	E9PVA6	S586	QNSTPESDYDNTACDPEPDDTGSTR	S3	1.137	0.304
Git2	ARF GTPase-activating protein GIT2	E9PVA6	S668	AAQENKHDSYIPCSER	S9	0.904	0.034
Glcci1	Glucocorticoid-induced transcript 1 protein	E9QKK4	S26	STAGSPPAAAGSGTGPAGSCAPAAGAGR	S5	0.946	0.219
Glcci1	Glucocorticoid-induced transcript 1 protein	E9QKK4	S294	VPCNVEGISPELEK	S9	0.931	0.711
Glcci1	Glucocorticoid-induced transcript 1 protein	E9QKK4	S97	GPSPSSPTPPPAAAPAEQAPR	S3	0.973	0.695
Glcci1	Glucocorticoid-induced transcript 1 protein	E9QKK4	S97,S/T	GPSPSSPTPPPAAAPAEQAPR	S3,S/T	0.958	0.309
Glcci1	Glucocorticoid-induced transcript 1 protein	E9QKK4	Ambiguous	RSTAGSPPAAAGSGTGPAGSCAPAAGAGR	S/T	1.074	0.524
Glcci1	Glucocorticoid-induced transcript 1 protein	E9QKK4	Ambiguous	DSGSSSPLPK	S	0.935	0.293
Glcci1	Glucocorticoid-induced transcript 1 protein	E9QKK4	Ambiguous	SASWGSADQLK	S	1.086	0.375
Glcci1	Glucocorticoid-induced transcript 1 protein	E9QKK4	Ambiguous	SIDTQTPSVQER	T/S	0.988	0.788
Glce	D-glucuronyl C5-epimerase	Q9EPS3	S73	QQSEEAFPQEQQK	S3	1.016	0.760
Gle1	Nucleoporin GLE1	Q8R322	S352	RQESQVQQGPAPPTQTSAPSPSPVGAQNEDLQVK	S4	1.410	0.000
Gle1	Nucleoporin GLE1	Q8R322	Ambiguous	QESQVQQGPAPPTQTSAPSPSPVGAQNEDLQVK	T/S	0.949	0.462
Glg1	Golgi apparatus protein 1	Q61543	S412	GRQVSSECQGEMLDYR	S5	1.000	1.000

Glg1	Golgi apparatus protein 1	Q61543	S412	QVSSECQGEMLDYRR	S3	1.039	0.722
Glg1	Golgi apparatus protein 1	Q61543	S412	GRQVSSECQGEMLDYR	S5	1.109	0.308
Glg1	Golgi apparatus protein 1	Q61543	S412	QVSSECQGEMLDYR	S3	1.049	0.370
Glg1	Golgi apparatus protein 1	Q61543	S991	IIIQESALDYR	S6	0.970	0.715
Glod4	Glyoxalase domain-containing protein 4	Q9CPV4	S133	SPSQSDPVLK	S3	1.010	0.777
Glrx5	Glutaredoxin-related protein 5, mitochondrial	Q80Y14	S151	SALVDEKDQDSK	S11	0.964	0.714
Glud1	Glutamate dehydrogenase 1, mitochondrial	P26443	S128	DDGSWEVIEGYR	S4	0.994	0.982
Glud1	Glutamate dehydrogenase 1, mitochondrial	P26443	S128	RDDGSWEVIEGYR	S5	1.033	0.793
Glud1	Glutamate dehydrogenase 1, mitochondrial	P26443	T484	HGGTIPVVPTAEFQDR	T4	1.121	0.004
Gm10073	Predicted pseudogene 10073	E9Q3T0	S101	KEESEESEDDMGFGLFD	S4	0.951	0.353
Gm10073	Predicted pseudogene 10073	E9Q3T0	S104	KEESEESEDDMGFGLFD	S7	1.008	0.877
Gm10073	Predicted pseudogene 10073	E9Q3T0	S101,S104	KEESEESEDDMGFGLFD	S4,S7	0.970	0.552
Gm10073	Predicted pseudogene 10073	E9Q3T0	S101,S104	KEESEESEDDMGFGLFD	S4,S7	0.969	0.702
Gm10093	Histone deacetylase	D3YYI8	S393	MLPHAPGVQMQAIPEDAIPEESGDEDEEDPDKR	S22	0.996	0.982
Gm10093	Histone deacetylase	D3YYI8	S423	IACEEEFSDSDEEGEGGRK	S10	1.010	0.849
Gm10093	Histone deacetylase	D3YYI8	S421,S423	IACEEEFSDSDEEGEGGRK	S8,S10	0.997	0.935
Gm10093	Histone deacetylase	D3YYI8	Ambiguous	IACEEEFSDSDEEGEGGR	S	1.291	0.371
Gm14399	Predicted gene 14399	A2ARW8	S70	SCSAEQPSEFIQCGK	S3	1.077	0.470
Gm20390	Nucleoside diphosphate kinase	E9PZF0	S235	NIIHGSDSVESAEK	S6	1.037	0.291
Gm20425	Predicted gene 20425	E9Q035	S964	GDTGSADIQDLEK	S5	1.133	0.263
Gm20425	Predicted gene 20425	E9Q035	S964	GGRGDTGSADIQDLEK	S8	1.018	0.684
Gm20425	Predicted gene 20425	E9Q035	T921	SAAPSTLDSSSTAPAQLGK	T6	0.988	0.914
Gm20431	Predicted gene 20431	E9PY39	S370	LPQPPEGQCYSN	S11	1.051	0.274
Gm20431	Predicted gene 20431	E9PY39	S370	ENMKLPQPPEGQCYSN	S15	0.914	0.237
Gm20431	Predicted gene 20431	E9PY39	Ambiguous	VNMSGVSSSNGVVDPR	S	1.026	0.498
Gm20521	Predicted gene 20521	D3Z5F7	S177	QMNMSPPPGNAGPVIMSLEEK	S5	0.968	0.707
Gm20521	Predicted gene 20521	D3Z5F7	S177	QMNMSPPPGNAGPVIMSLEEK	S5	0.984	0.810
Gm21992	Predicted gene 21992	F7BGR7	S202	LHVGNISPTCTNQELR	S7	1.136	0.440
Gm27029	Predicted gene, 27029	A2A4P4	S219	RGAQADHFTESPLSPGSQVQVR	S14	1.025	0.825
Gm27029	Predicted gene, 27029	A2A4P4	S219	GAQADHFTESPLSPGSQVQVR	S13	0.956	0.616
Gm340	Predicted gene 340	F6RGI3	S788	EGGASESAEPLDEEDEDSVVEEQPK	S18	1.001	0.989
Gm340	Predicted gene 340	F6RGI3	Ambiguous	YSAENDPSEPNSDSPEENVDK	S/Y	1.000	1.000
Gm340	Predicted gene 340	F6RGI3	Ambiguous	YSAENDPSEPNSDSPEENVDKK	S/Y	0.947	0.722
Gm340	Predicted gene 340	F6RGI3	Ambiguous	EDSEDLLCRPQSSPK	S	1.043	0.606
Gm45837	Phosphodiesterase	F7D3W5	Ambiguous	RPEAVQNTSVDASEDQKDEK	T/S	0.957	0.674
Gm49324	Predicted gene, 49324 (Fragment)	A0A1L1SQF0	S275	GDVSPTPIYLR	S4	0.945	0.582
Gm49324	Predicted gene, 49324 (Fragment)	A0A1L1SQF0	Ambiguous	DVEAEATSPRPSEWTSVR	T/S	0.968	0.504
Gm49391	Predicted gene, 49391	A0A286YD17	S52	AYSLDEER	S3	1.253	0.159
Gm6377	Predicted gene 6377	Q8BHV4	S204	QPLSNSMLNNYIER	S6	1.113	0.545
Gm7694	Predicted gene 7694	J3QNH8	S221	SSSPGPIER	S3	1.036	0.414
Gm7694	Predicted gene 7694	J3QNH8	S256	TSSLDNEGLHPDLLSFE	S3	1.009	0.900
Gm9044	Predicted gene 9040	A0A1W2P6Q8	T424	TPSNGAEGLTPR	T10	1.006	0.916
Gm9044	Predicted gene 9040	A0A1W2P6Q8	T385,T396	GGLNTPLHESDFSGVTPQR	T5,T16	1.053	0.753
Gm9044	Predicted gene 9040	A0A1W2P6Q8	Ambiguous	GGLNTPLHESDFSGVTPQR	S/T	0.985	0.844
Gm9044	Predicted gene 9040	A0A1W2P6Q8	Ambiguous	SGTTPKPVTNATPGR	T/S	0.991	0.917
Gm9774	MCG119397	A0A0A6YVU8	S213	SQSAAVTPSSSTSSAR	S3	1.224	0.380
Gmip	GEM-interacting protein	Q6PGG2	S247	SQGSPEDPPSQASPGSNK	S13	0.999	0.980
Gmip	GEM-interacting protein	Q6PGG2	S436	SLDSPTSSPGAGAR	S4	0.994	0.894
Gmip	GEM-interacting protein	Q6PGG2	S479	ASSTGTESSDDFEERDPDLGDGIENGVGSPFRK	S29	0.998	0.955
Gmip	GEM-interacting protein	Q6PGG2	S75	DLSPEGPAPLTEEELDLR	S3	1.063	0.355
Gmip	GEM-interacting protein	Q6PGG2	S924	GLGPAAACPEGSPLR	S12	1.261	0.017
Gmip	GEM-interacting protein	Q6PGG2	T659	TLHADPGDDPGTPNPSPEIIR	T12	1.175	0.452
Gmip	GEM-interacting protein	Q6PGG2	T820	CPEVTPPELATLQR	T5	0.848	0.229
Gmip	GEM-interacting protein	Q6PGG2	S238,S247	SQGSPEDPPSQASPGSNK	S4,S13	0.992	0.902
Gmip	GEM-interacting protein	Q6PGG2	S436,S440	SLDSPTSSPGAGAR	S4,S8	0.997	0.901
Gmip	GEM-interacting protein	Q6PGG2	S439,S440	SLDSPTSSPGAGARR	S7,S8	0.799	0.140
Gmip	GEM-interacting protein	Q6PGG2	Ambiguous	ASSTGTESSDDFEERDPDLGDGIENGVGSPFR	S/T	0.805	0.153
Gmnn	Geminin	O88513	S19	QEGAQENVKNSPVPR	S11	1.173	0.051
Gmpr2	GMP reductase 2	Q99L27	S184	VGIGPGSVCTTR	S7	1.145	0.173
Gmpr2	GMP reductase 2	Q99L27	S28	SRSEVELTR	S3	0.974	0.675
Gmpr2	GMP reductase 2	Q99L27	S347	VTQQVNPIFSNSQ	S12	1.041	0.600
Gmps	GMP synthase [glutamine-hydrolyzing]	Q3THK7	S332	TLNMTTSPEEK	S7	0.969	0.284
Gmps	GMP synthase [glutamine-hydrolyzing]	Q3THK7	S332	TLNMTTSPEEKR	S7	1.073	0.386
Gmps	GMP synthase [glutamine-hydrolyzing]	Q3THK7	S332	TLNMTTSPEEK	S7	0.912	0.366
Gnai2	Guanine nucleotide-binding protein G(i) subunit alpha-2	P08752	S166	IAQSDYIPTQQDVLR	S4	0.997	0.987
Gnas	Guanine nucleotide-binding protein G(s) subunit alpha isoforms XLas	Q6R0H7	S1091	ISTASGDGR	S5	1.058	0.262
Gng12	Guanine nucleotide-binding protein subunit gamma	A0A0N4SW28	S10	TASTNSIAQAR	S6	1.040	0.212
Gng12	Guanine nucleotide-binding protein subunit gamma	A0A0N4SW28	S7,S10	TASTNSIAQAR	S3,S6	1.060	0.308
Gng2	Guanine nucleotide-binding protein G(I)/G(S)/G(O) subunit gamma-2	P63213	T52	EDPLLTPVPASENPFR	T6	1.104	0.500
Gnl1	Guanine nucleotide-binding protein-like 1	P36916	S36,S	GLQDGLRSSSNSR	S12,S	1.019	0.774
Gnl1	Guanine nucleotide-binding protein-like 1	P36916	T48,T/S	EEQTDTSDGESVTHHIR	T4,T/S	1.039	0.788
Gnl1	Guanine nucleotide-binding protein-like 1	P36916	Ambiguous	REEQTDTSDGESVTHHIR	S/T	1.381	0.281
Gnl1	Guanine nucleotide-binding protein-like 1	P36916	Ambiguous	EEQTDTSDGESVTHHIR	T/S	1.110	0.660
Gnl2	Nucleolar GTP-binding protein 2	Q99LH1	S647	TSAEDSDAAPTKK	S6	1.106	0.122
Gnl2	Nucleolar GTP-binding protein 2	Q99LH1	Ambiguous	SDSITEKEPEGDCSQDRNSEMQQILAR	S/T	1.133	0.255
Gnl2	Nucleolar GTP-binding protein 2	Q99LH1	Ambiguous	SDSITEKEPEGDCSQDR	T/S	1.068	0.696
Gnl3	Guanine nucleotide-binding protein-like 3	Q8CI11	S101	KLEVSPGDEQSNVETR	S11	1.007	0.840
Gnl3	Guanine nucleotide-binding protein-like 3	Q8CI11	S375	GGSPNVESAAK	S3	0.946	0.397
Gnl3	Guanine nucleotide-binding protein-like 3	Q8CI11	S505	ELSPEQSTAGKPSDGSSALDR	S3	1.009	0.889
Gnl3	Guanine nucleotide-binding protein-like 3	Q8CI11	S95	LEVSPGDEQSNVETR	S4	0.954	0.566
Gnl3	Guanine nucleotide-binding protein-like 3	Q8CI11	Ambiguous	NAEISDVAPVEETRELSPEQSTAGKPSDGSSALDR	T/S	1.056	0.468
Golga1	Golgin subfamily A member 1	Q9CW79	S30	SVSKESVASMGADSGDDFASDGSSSR	S3	1.158	0.055
Golga1	Golgin subfamily A member 1	Q9CW79	S36	ESVASMGADSGDDFASDGSSSR	S5	1.000	1.000
Golga1	Golgin subfamily A member 1	Q9CW79	S30,S36	SVSKESVASMGADSGDDFASDGSSSR	S3,S9	0.987	0.823
Golga2	Golgin subfamily A member 2 (Fragment)	Z4YJU8	S26	NSPGVPAGAK	S2	1.197	0.028
Golga2	Golgin subfamily A member 2 (Fragment)	Z4YJU8	S44	NGHSPERPTASDCQSPENSHDAGNCSNLMEETK	S4	0.885	0.171
Golga2	Golgin subfamily A member 2 (Fragment)	Z4YJU8	S44	NGHSPERPTASDCQSPENSHDAGNCSNLMEETK	S4	0.926	0.484
Golga2	Golgin subfamily A member 2 (Fragment)	Z4YJU8	S748	NVDDSASEESNQALHVAMEK	S7	1.037	0.629
Golga2	Golgin subfamily A member 2 (Fragment)	Z4YJU8	S748	NVDDSASEESNQALHVAMEK	S7	0.797	0.031
Golga2	Golgin subfamily A member 2 (Fragment)	Z4YJU8	Ambiguous	TFSSTESLR	S/T	1.005	0.920
Golga3	Golgin subfamily A member 3	E9QP99	S1479	LHNQNSVPRDGLGQ	S6	1.124	0.266
Golga3	Golgin subfamily A member 3	E9QP99	S270	TEDPSDSGGLGSTADAVGSSLK	S20	1.177	0.181
Golga3	Golgin subfamily A member 3	E9QP99	S461	QDSLSSEVDTLK	S3	1.014	0.919
Golga3	Golgin subfamily A member 3	E9QP99	S735	GQSLDDLHTR	S3	0.956	0.780
Golga3	Golgin subfamily A member 3	E9QP99	Ambiguous	SSTSVVSEVSPSSETDNR	S1	0.995	0.966
Golga4	Golgin subfamily A member 4	Q91VW5	S41	TSSFTDQLDDVTPNR	S3	1.046	0.615
Golga4	Golgin subfamily A member 4	Q91VW5	S41	TSSFTDQLDDVTPNRENASTQATK	S3	1.123	0.003
Golga4	Golgin subfamily A member 4	Q91VW5	Ambiguous	SPDGVSKDESSPSQSGDTQTFAQK	S/T	0.826	0.063
Golga4	Golgin subfamily A member 4	Q91VW5	Ambiguous	SRTSSFTDQLDDVTPNR	T/S	1.069	0.411
Golga5	Golgin subfamily A member 5	Q9QYE6	S150	APVSPSSPSGVSSVNTSVTTTK	S4	1.383	0.023
Golgb1	Golgi autoantigen, golgin subfamily b, macrogolgin 1	E9PVZ8	S1760	QASLEATEK	S3	1.072	0.354
Golgb1	Golgi autoantigen, golgin subfamily b, macrogolgin 1	E9PVZ8	S1792	DALSDSAK	S4	1.173	0.078
Golgb1	Golgi autoantigen, golgin subfamily b, macrogolgin 1	E9PVZ8	S2989	QASPETSASLDGSQK	S3	1.083	0.223
Golgb1	Golgi autoantigen, golgin subfamily b, macrogolgin 1	E9PVZ8	S548	SDSSPEENGQAVLENTFSQK	S3	1.131	0.008
Golgb1	Golgi autoantigen, golgin subfamily b, macrogolgin 1	E9PVZ8	T477	NETASQTSLPDVNNEGDQAVMEETVASLQK	T24	0.845	0.342
Golim4	Golgi integral membrane protein 4	D3YVW2	S147	QGEDFSRTFNDHK	S6	1.029	0.676
Golim4	Golgi integral membrane protein 4	D3YVW2	S568	EENLPEESEEQKQSEAK	S8	1.026	0.782
Golim4	Golgi integral membrane protein 4	D3YVW2	Y661	GRQEHYEEEEDEEDGAAVAEK	Y6	1.139	0.216
Golim4	Golgi integral membrane protein 4	D3YVW2	Ambiguous	EMEHNVEETYGEHPDDKNNDGEEQGVHNR	T/Y	1.004	0.988
Golm1	Golgi membrane protein 1	Q91XA2	S187	NEAPGSRDLAETNNQHQQALKPQPK	S6	1.034	0.400
Golm1	Golgi membrane protein 1	Q91XA2	S213	LQEEVPSEEQMPQEK	S7	1.021	0.476
Golm1	Golgi membrane protein 1	Q91XA2	S213	LQEEVPSEEQMPQEKGDVPR	S7	0.957	0.773
Golm1	Golgi membrane protein 1	Q91XA2	S213	LQEEVPSEEQMPQEK	S7	0.957	0.286
Golm1	Golgi membrane protein 1	Q91XA2	S213	LQEEVPSEEQMPQEKGDVPR	S7	0.996	0.979
Golm1	Golgi membrane protein 1	Q91XA2	S375	GIINVPVGSER	S9	1.017	0.657
Golph3	Golgi phosphoprotein 3	Q9CRA5	S9	SSGLVQR	S2	0.916	0.255
Golph3l	Golgi phosphoprotein 3-like	H3BJ07	S170	SDSPTGDVLLDETLK	S3	1.138	0.350
Golph3l	Golgi phosphoprotein 3-like	H3BJ07	S90	KIESEEDTNQERSPDNEDPGDSK	S13	0.985	0.684
Gon4l	GON-4-like protein	Q9DB00	S1412	EDMQAAKSPSVPQDAAAEGR	S8	1.093	0.109
Gopc	Golgi-associated PDZ and coiled-coil motif-containing protein	K3W4Q9	Ambiguous	LYLDELEGSGNSGASCK	S/Y	0.767	0.077
Gorab	RAB6-interacting golgin	A0A087WQS3	S149	EISLGSPPTR	S6	0.852	0.084
Gorab	RAB6-interacting golgin	A0A087WQS3; Q8BRM2	S82;S82	SPSPVAPSPLTPTSSSGDGK	S3	1.038	0.810
Gorasp1	Golgi reassembly-stacking protein 1	Q91X51	S434	AETEGRASQAQATPDPEPGL	S8	0.977	0.535
Gorasp1	Golgi reassembly-stacking protein 1	Q91X51	T439	ASQAQATPDPEPGL	T7	0.963	0.439
Gorasp2	Golgi reassembly-stacking protein 2	Q99JX3	S411	ADASSLTVDVTSPASK	S5	1.230	0.094
Gorasp2	Golgi reassembly-stacking protein 2	Q99JX3	S432	VPTTVEDRVSDCTPAVEKPVSDADASEPS	S10	0.998	0.979
Gorasp2	Golgi reassembly-stacking protein 2	Q99JX3	S451	VSDCTPAVEKPVSDADASEPS	S21	0.966	0.430
Gp49a	Mast cell surface glycoprotein Gp49A	Q61450	S279	NTQSEDNAELNSWNPQNEDPPR	S4	1.064	0.121
Gpalpp1	GPALPP motifs-containing protein 1	Q69ZC8	S146	GREDPGQVSSFFNSEEAESGEDEDIVGPMPAK	S19	0.965	0.776
Gpalpp1	GPALPP motifs-containing protein 1	Q69ZC8	Ambiguous	EDPGQVSSFFNSEEAESGEDEDIVGPMPAK	S	1.025	0.776
Gpatch2	G patch domain-containing protein 2	Q7TQC7	S283	QGDDEQSDWFYEK	S7	1.091	0.148
Gpatch2	G patch domain-containing protein 2	Q7TQC7	S518	SSPTSPAPTSGNPA	S5	1.027	0.772
Gpatch2l	G patch domain-containing protein 2-like	Q6PE65	Ambiguous	EVAPLTNFSDSDDTVVAK	T/S	1.072	0.559
Gpatch3	G patch domain-containing protein 3	Q8BIY1	S61	GPPQASPEAAR	S6	1.235	0.113
Gpatch4	G patch domain-containing protein 4	Q3TFK5	S130	DLGNCSDVDNHEPTPPK	S6	0.999	0.983
Gpatch8	G patch domain-containing protein 8	A2A6A1	S1039	SQSPHYFQSGR	S3	0.980	0.659
Gpatch8	G patch domain-containing protein 8	A2A6A1	S1082	GSESDCSPEDKNSVTAR	S4	0.957	0.430
Gpatch8	G patch domain-containing protein 8	A2A6A1	S1179	KCEESGLERGEEQEHSEPEEGSPR	S16	1.067	0.304

Gpatch8	G patch domain-containing protein 8	A2A6A1	S451	TAASPGAER	S4	1.062	0.505
Gpatch8	G patch domain-containing protein 8	A2A6A1	S503	ETSESPMCESNPK	S5	1.005	0.958
Gpatch8	G patch domain-containing protein 8	A2A6A1	S610	EPNKSQEEEQDVVLSSEGR	S5	1.044	0.245
Gpatch8	G patch domain-containing protein 8	A2A6A1	S648	VDEPASGAACSSLNKQEPGGSHMSETEDTGR	S24	0.989	0.811
Gpatch8	G patch domain-containing protein 8	A2A6A1	S648	VDEPASGAACSSLNKQEPGGSHMSETEDTGR	S24	1.001	0.983
Gpatch8	G patch domain-containing protein 8	A2A6A1	S648	QEPGGSHMSETEDTGR	S9	0.910	0.570
Gpatch8	G patch domain-containing protein 8	A2A6A1	S735	SEPPGSGSPAPPR	S8	1.005	0.890
Gpatch8	G patch domain-containing protein 8	A2A6A1	S735	SEPPGSGSPAPPRR	S8	1.096	0.532
Gpatch8	G patch domain-containing protein 8	A2A6A1	S753	SLPAEEGNSGK	S1	1.014	0.669
Gpatch8	G patch domain-containing protein 8	A2A6A1	S753	SLPAEEGNSGKK	S1	1.010	0.869
Gpatch8	G patch domain-containing protein 8	A2A6A1	S1179,S1185	CEESGLERGEEQEHSEPEEGSPR	S15,S21	1.023	0.821
Gpatch8	G patch domain-containing protein 8	A2A6A1	Ambiguous	QEPGGSHMSETEDTGR	S/T	1.112	0.627
Gpbp1	Vasculin	Q6NXH3	S381	SSTFPQTDVLSSSLEAEHR	S13	1.062	0.397
Gpbp1	Vasculin	Q6NXH3	S49	HNSSDGFDSGIGRPNGGNFGR	S3	1.018	0.808
Gpbp1l1	Vasculin-like protein 1	Q6NZP2	S76	HDSVDSGVSK	S3	1.233	0.003
Gpbp1l1	Vasculin-like protein 1	Q6NZP2	T298	ESPSSTTPPIEISSSR	T7	1.024	0.860
Gpbp1l1	Vasculin-like protein 1	Q6NZP2	T353	LEGSHTPEPK	T6	1.019	0.769
Gphn	Gephyrin	A0JNY3	S194	EVHDELEDLPSPPPPLSPPPTTSPHK	S17	1.077	0.674
Gphn	Gephyrin	A0JNY3	S306	DTASLSTTPSESPR	S12	1.059	0.473
Gphn	Gephyrin	A0JNY3	S341	ASHSAVDITK	S4	0.923	0.011
Gphn	Gephyrin	A0JNY3	S188,S194	EVHDELEDLPSPPPPLSPPPTTSPHK	S11,S17	0.770	0.276
Gpi	Glucose-6-phosphate isomerase	P06745	T215	TFTTQETITNAETAK	T4	1.137	0.469
Gpkow	G-patch domain and KOW motifs-containing protein	Q56A08	S348	KHSPDRQDGPVPK	S3	0.999	0.992
Gpn1	GPN-loop GTPase 1	Q8VCE2	S314	GNASPVLDPSDLILTR	S4	1.071	0.745
Gpn1	GPN-loop GTPase 1	Q8VCE2	Ambiguous	GTLDEEDEEADSDTDDIDHR	S/T	1.006	0.945
Gpnmb	Transmembrane glycoprotein NMB	Q99P91	S362	SMELSDISNENCR	S1	1.127	0.109
Gpnmb	Transmembrane glycoprotein NMB	Q99P91	S362	SMELSDISNENCR	S1	1.179	0.322
Gpr107	Protein GPR107	Q8BUV8	S537	KVSNGAVEPQGSWEGTA	S3	0.958	0.840
Gpr107	Protein GPR107	Q8BUV8	Ambiguous	ILSQSQEPAVSSNPK	S	1.114	0.055
Gpr155	G protein-coupled receptor 155	A2AWR3	S739	EAAADRESPVSEEIK	S8	1.084	0.376
Gpr157	G-protein coupled receptor 157	Q8C206	S318	IGESQESR	S4	1.134	0.390
Gpr176	G-protein coupled receptor 176	Q80WT4	S396	YLGSADFQAK	S4	1.017	0.896
Gpr176	G-protein coupled receptor 176	Q80WT4	Ambiguous	NVVSTGSGVAEPSLEPSMR	T/S	0.887	0.402
Gpr84	G-protein-coupled receptor 84	A0A0R4J100	S221	ALDQYGLHQASIR	S11	1.024	0.847
Gpr84	G-protein-coupled receptor 84	A0A0R4J100	S224	SHQVAGTQEAMPGHFQELDSGVASR	S1	1.014	0.870
Gpr84	G-protein-coupled receptor 84	A0A0R4J100	S383	HAYGSILK	S5	1.017	0.843
Gpr84	G-protein-coupled receptor 84	A0A0R4J100	Ambiguous	GPSEGISSEPVSAATTQTLEGDSSEAGGQGIR	S/T	1.011	0.919
Gprc5a	G protein-coupled receptor, family C, group 5, member A	G5E8C3	S344	AQAPASPYNDYEGR	S6	1.186	0.321
Gprc5b	G protein-coupled receptor family C group 5 member B variant 2	D2DFA9	S355	SAVGFSNGSLEQR	S9	0.996	0.954
Gprc5c	G protein-coupled receptor, family C, group 5, member C	I7HPW4	S343	AFSMDEPASAK	S3	0.915	0.681
Gprc5c	G protein-coupled receptor, family C, group 5, member C	I7HPW4	S402	ATANSQVMGSANSTLR	S10	1.210	0.011
Gprc5c	G protein-coupled receptor, family C, group 5, member C	I7HPW4	S434	ISQDQSPTNK	S6	0.984	0.859
Gprc5c	G protein-coupled receptor, family C, group 5, member C	I7HPW4	Y386	GPSEGAYDVILPR	Y7	0.866	0.283
Gps1	COP9 signalosome complex subunit 1	G3UXW9	S503	NQIHVKSPPREGSQGELTPANSQSR	S7	0.985	0.908
Gps1	COP9 signalosome complex subunit 1	G3UXW9	S509	EGSQGELTPANSQSR	S3	1.026	0.639
Gps1	COP9 signalosome complex subunit 1	G3UXW9	S509	SPPREGSQGELTPANSQSR	S7	1.133	0.349
Gpsm1	G-protein-signaling modulator 1	Q6IR34	S469	EGSHSPLDSADVR	S5	0.966	0.670
Gpsm1	G-protein-signaling modulator 1	Q6IR34	S653	VDLAGSPEQEASGLPDPQQQCPPGAS	S6	0.833	0.098
Gpsm2	G-protein-signaling modulator 2	Q8VDU0	Ambiguous	GNSPSVLER	S	1.084	0.385
Gpsm3	G-protein-signaling modulator 3	Q3U1Z5	S38	SAPPSPPPPGTR	S5	1.021	0.728
Gpsm3	G-protein-signaling modulator 3	Q3U1Z5	T82	RLEEQRATFHTPEAPPNLAPAPPR	T8	1.034	0.703
Gpsm3	G-protein-signaling modulator 3	Q3U1Z5	T85	ATFHTPEAPPNLAPAPPR	T5	1.018	0.806
Gpsm3	G-protein-signaling modulator 3	Q3U1Z5	S34,S38	SAPPSPPPPGTR	S1,S5	1.011	0.606
Gpsm3	G-protein-signaling modulator 3	Q3U1Z5	Ambiguous	SGSLLSLQTELLLDLVAEAQSR	S/T	1.050	0.747
Gramd1a	GRAM domain-containing protein 1A	Q8VEF1	S12	SSPSSSPSLR	S2	1.030	0.365
Gramd1b	GRAM domain-containing protein 1B	D3YWR0	S301	SSIETKPDASPQLPK	S10	1.100	0.144
Gramd1b	GRAM domain-containing protein 1B	D3YWR0	S43	STPACSPILR	S1	1.029	0.553
Gramd1b	GRAM domain-containing protein 1B	D3YWR0	S57	SRSPTPQNQDGDTMVEK	S3	1.027	0.485
Gramd1b	GRAM domain-containing protein 1B	D3YWR0	S57	SRSPTPQNQDGDTMVEK	S3	1.061	0.301
Gramd1b	GRAM domain-containing protein 1B	D3YWR0	S577	TESTYLAEIHRQSPK	S13	1.152	0.422
Gramd1b	GRAM domain-containing protein 1B	D3YWR0	S608	VPHLEEVMSPVTTPTDEDVGHR	S9	0.946	0.098
Gramd1b	GRAM domain-containing protein 1B	D3YWR0	S40,S43	VRDESTASNSNRSTPACSPILR	S10,S13	1.006	0.893
Gramd1b	GRAM domain-containing protein 1B	D3YWR0	S43,S48	STPACSPILR	S1,S6	0.971	0.795
Gramd1b	GRAM domain-containing protein 1B	D3YWR0	Ambiguous	VRDESTASNSNRSTPACSPILR	S/T	1.044	0.644
Gramd2b	GRAM domain-containing protein 2B	Q6PEM6	S71	SPTAQSPTSSVEAESPDQKR	S15	1.091	0.334
Gramd2b	GRAM domain-containing protein 2B	Q6PEM6	Ambiguous	SVCGHLENTSVGNSPNPSSAENSFR	S/T	0.972	0.936
Gramd2b	GRAM domain-containing protein 2B	Q6PEM6	Ambiguous	SVCGHLENTSVGNSPNPSSAENSFR	T/S	0.799	0.449
Grb2	Growth factor receptor-bound protein 2	Q60631	S18	ATADDELSFK	S8	1.109	0.073
Grb2	Growth factor receptor-bound protein 2	Q60631	Ambiguous	ESESAPGDFSLSVK	S	1.034	0.369
Grem1	Gremlin-1	O70326	Ambiguous	GTAMPGEEVLESSQEALHVTER	S/T	0.945	0.807
Grem2	Gremlin-2	O88273	S55	EVLASSQEALVVTER	S5	0.996	0.964
Gripap1	GRIP1-associated protein 1	A2AEW8	S826	ECVGSPDPDLEPGEAN	S5	1.070	0.087
Gripap1	GRIP1-associated protein 1	A2AEW8	S826	LSKECVGSPDPDLEPGEAN	S8	1.019	0.872
Gripap1	GRIP1-associated protein 1	A2AEW8	S662,S665	TQTGDSSSVSSFSYR	S8,S11	0.894	0.074
Gripap1	GRIP1-associated protein 1	A2AEW8	Ambiguous	TQTGDSSSVSSFSYR	S/Y/T	0.974	0.699
Grk5	G protein-coupled receptor kinase 5	Q8VEB1	Ambiguous	INSNHINSNSTGSS	S/T	1.147	0.251
Gsdmdc1	Gasdermin-D	Q9D8T2	S245	TFEPSSGDRK	S6	0.905	0.039
Gse1	Genetic suppressor element 1 (Fragment)	M0QWG1	S234	SSTPEGNDVAGSLGDEGLERPPR	S12	1.008	0.919
Gse1	Genetic suppressor element 1	Q3U3C9	S826,S828	ERSPSPPAVQCK	S3,S5	0.951	0.535
Gse1	Genetic suppressor element 1	Q3U3C9	Ambiguous	GSSLSSESSPVSSPATNHSSPASTPK	S/T	0.718	0.244
Gsk3a	Glycogen synthase kinase-3 alpha	Q2NL51	S21	TSSFAEPGGGGGGGGGGPGGSASGPGGTGGGK	S3	1.037	0.161
Gsk3a	Glycogen synthase kinase-3 alpha	Q2NL51; E9QAQ5	S278;S215	GEPNVSYICSR	S6	1.017	0.479
Gsk3a	Glycogen synthase kinase-3 alpha	Q2NL51	S21,S41	TSSFAEPGGGGGGGGGGPGGSASGPGGTGGGK	S3,S23	1.016	0.749
Gsk3a	Glycogen synthase kinase-3 alpha	Q2NL51; E9QAQ5	S282,S/Y;S219,S/Y	GEPNVSYICSR	S10,Y/S	0.959	0.397
Gsk3b	Glycogen synthase kinase-3 beta	E9QAQ5	S402	IQAAASPPANATAASDTNAGDR	S6	0.967	0.559
Gsn	Gelsolin	P13020	S560	DGGQTAPASIR	S9	1.058	0.423
Gspt1	Eukaryotic peptide chain release factor GTP-binding subunit ERF3A	Q8R050	S199	SAVAPPGAPK	S1	1.011	0.939
Gstm1	Glutathione S-transferase Mu 1	A2AE89	T34	RYTMGDAPDFDR	T3	1.146	0.013
Gtf2e2	General transcription factor IIE subunit 2	Q9D902	S31	AVPSESPSSSSSK	S6	1.078	0.157
Gtf2f1	General transcription factor IIF subunit 1	Q3THK3	S431	MDTGPQSLSGKSTPSSGDVQVTEDAVR	S7	0.806	0.161
Gtf2f1	General transcription factor IIF subunit 1	Q3THK3	S433	MDTGPQSLSGK	S9	0.933	0.395
Gtf2f1	General transcription factor IIF subunit 1	Q3THK3	S433	MDTGPQSLSGK	S9	1.000	1.000
Gtf2f1	General transcription factor IIF subunit 1	Q3THK3	T389	GTSRPGTPSAEAASTSSTLR	T7	1.183	0.019
Gtf2f1	General transcription factor IIF subunit 1	Q3THK3	S431,S/T	MDTGPQSLSGKSTPSSGDVQVTEDAVRR	S7,S/T	0.886	0.323
Gtf2f1	General transcription factor IIF subunit 1	Q3THK3	S433,S436	MDTGPQSLSGKSTPSSGDVQVTEDAVR	S9,S12	0.916	0.223
Gtf2f1	General transcription factor IIF subunit 1	Q3THK3	T384,T389	GTSRPGTPSAEAASTSSTLR	T2,T7	0.858	0.079
Gtf2f2	General transcription factor IIF subunit 2	Q8R0A0	S248	HYQTEEKSD	S8	0.982	0.796
Gtf2i	General transcription factor II-I	G3UYD0	S210	SMLSPSGSCGPIK	S4	1.045	0.476
Gtf2ird2	General transcription factor II-I repeat domain-containing protein 2	Q99NI3	S290	MEGNASPSNLVNSAAGVEDLR	S6	0.874	0.269
Gtf3c1	General transcription factor 3C polypeptide 1	Q8K284	S1063	ICPDPGSDPEGSLR	S7	1.030	0.465
Gtf3c1	General transcription factor 3C polypeptide 1	Q8K284	S1063	ICPDPGSDPEGSLRK	S7	1.050	0.660
Gtf3c1	General transcription factor 3C polypeptide 1	Q8K284	S1890	VAASPRPGAEEQAEAQAPAQLAAPEDADAGGPR	S4	1.025	0.590
Gtf3c2	General transcription factor 3C polypeptide 2	D3Z7I0	S163	GLDQPESPHPK	S7	0.964	0.301
Gtf3c2	General transcription factor 3C polypeptide 2	D3Z7I0	Ambiguous	ADLIPYQDSPEDQDYSSTSSETPNPPK	T/S/Y	1.191	0.385
Gtf3c3	General transcription factor IIIC, polypeptide 3	Q3TMP1	S43	SSPKENPDDSEVPSSSGIDSAK	S2	1.023	0.876
Gtf3c4	General transcription factor 3C polypeptide 4	Q8BMQ2	S17	ADEPSPPAEEKDEGGGK	S5	1.056	0.246
Gtf3c4	General transcription factor 3C polypeptide 4	Q8BMQ2	S17	ADEPSPPAEEKDEGGGKEAAADAAPGPSASFR	S5	0.952	0.451
Gtf3c4	General transcription factor 3C polypeptide 4	Q8BMQ2	S607	ILLVDSPGMGDGEDEQQEEGTSK	S6	1.084	0.152
Gtpbp1	GTP-binding protein 1	O08582	S25	SPVDSPVPASMFAPEPSSPGAAR	S18	1.008	0.912
Gtpbp1	GTP-binding protein 1	O08582	S12,S	SPVDSPVPASMFAPEPSSPGAAR	S5,S	0.995	0.966
Gtpbp1	GTP-binding protein 1	O08582	S25,S	SRSPVDSPVPASMFAPEPSSPGAAR	S20,S	0.978	0.573
Gtpbp1	GTP-binding protein 1	O08582	S6,S25	SRSPVDSPVPASMFAPEPSSPGAAR	S1,S20	1.019	0.801
Gtpbp1	GTP-binding protein 1	O08582	Ambiguous	SRSPVDSPVPASMFAPEPSSPGAAR	S	1.022	0.381
Gtpbp1	GTP-binding protein 1	O08582	Ambiguous	SPVDSPVPASMFAPEPSSPGAAR	S	0.789	0.517
Gtse1	G2 and S phase-expressed protein 1	Q8R080	T420	LDPDTETPQLNK	T7	0.945	0.517
Gvin1	Interferon-induced very large GTPase 1	L7N451	S177	TLAHSPNLSDGDLVR	S9	0.889	0.362
Gys1	Glycogen [starch] synthase, muscle	Q9Z1E4	S645	YPRPASVPPSPSLSR	S10	1.049	0.675
Gys1	Glycogen [starch] synthase, muscle	Q9Z1E4	S728	SNSVDTGPSSSLSTPTEPLSPTSSLGEERN	S20	1.053	0.498
Gys1	Glycogen [starch] synthase, muscle	Q9Z1E4	S728	SNSVDTGPSSSLSTPTEPLSPTSSLGEER	S20	1.004	0.947
Gys1	Glycogen [starch] synthase, muscle	Q9Z1E4	S641,S645	YPRPASVPPSPSLSR	S6,S10	0.981	0.759
Gys1	Glycogen [starch] synthase, muscle	Q9Z1E4	Ambiguous	RSNSVDTGPSSSLSTPTEPLSPTSSLGEERN	S/T	1.449	0.001
Gzf1	GDNF-inducible zinc finger protein 1	Q4VBD9	Ambiguous	SAEEAAENDKCPQDQSPDNERMETEPAAK	S/T	1.048	0.801
H1f0	Histone H1.0	P10922	S92	GVGASGSFR	S7	1.189	0.006
H2afx	Histone H2AX	P27661	S137	KASQASQEY	S6	1.061	0.056
H2afx	Histone H2AX	P27661	S137,S140	KASQASQEY	S3,S6	0.885	0.092
H2afy	Core histone macro-H2A.1	Q9QZQ8	S173	AASADSTTEGTPTDGFTVLSTK	S6	1.042	0.542
H2afy	Core histone macro-H2A.1	Q9QZQ8	Ambiguous	AASADSTTEGTPTDGFTVLSTK	S/T	1.519	0.441
H2-D1	H-2 class I histocompatibility antigen, D-B alpha chain	P01899	S350	GGDYALAPGSQSSEMSLR	S10	1.014	0.724
H2-D1	H-2 class I histocompatibility antigen, D-B alpha chain	P01899	S356	GGDYALAPGSQSSEMSLR	S16	1.018	0.632
H2-D1	H-2 class I histocompatibility antigen, D-B alpha chain	P01899	S352,S356	GGDYALAPGSQSSEMSLR	S12,S16	1.089	0.457
H2-D1	H-2 class I histocompatibility antigen, D-B alpha chain	P01899	S356,S/Y	GGDYALAPGSQSSEMSLR	S16,S/Y	1.023	0.359
H2-D1	H-2 class I histocompatibility antigen, D-B alpha chain	P01899	Ambiguous	GGDYALAPGSQSSEMSLRDCKA	S/Y	1.092	0.394
H2-K1	H-2 class I histocompatibility antigen, K-B alpha chain	P01901	S354	GGDYALAPGSQTSDLSLPDCK	S16	1.034	0.112
H2-K1	H-2 class I histocompatibility antigen, K-B alpha chain	P01901	T350,S354	GGDYALAPGSQTSDLSLPDCK	T12,S16	1.019	0.669
H3f3c	Histone H3.3C	P02301	S29	SAPSTGGVK	S1	1.301	0.001
H3f3c	Histone H3.3C	P02301; P68433; A0A1W2P768	S58;S58;S103	STELLIR	S1	1.233	0.017
Hacd3	Very-long-chain (3R)-3-hydroxyacyl-CoA dehydratase 3	Q8K2C9	S114	WLDESDAEMELR	S5	1.060	0.367
Hacd3	Very-long-chain (3R)-3-hydroxyacyl-CoA dehydratase 3	Q8K2C9	S114	WLDESDAEMELR	S5	1.070	0.466

Hacd3	Very-long-chain (3R)-3-hydroxyacyl-CoA dehydratase 3	Q8K2C9	S138	EGSPETLTNLK	S3	0.990	0.915
Hadh	Hydroxyacyl-coenzyme A dehydrogenase, mitochondrial	Q61425	Ambiguous	SMSSSSSASAAAK	S	1.100	0.163
Hars2	Probable histidine--tRNA ligase, mitochondrial	Q99KK9	S66	DLSPQQMVVR	S3	1.048	0.361
Hars2	Probable histidine--tRNA ligase, mitochondrial	Q99KK9	S66	DLSPQQMVVR	S3	0.994	0.935
Haus6	HAUS augmin-like complex, subunit 6	Q6NV99	S500	TIADSEEEDSPLSDTAK	S5	0.981	0.831
Haus6	HAUS augmin-like complex, subunit 6	Q6NV99	S550	VVLSDSPQLSER	S6	0.922	0.660
Haus6	HAUS augmin-like complex, subunit 6	Q6NV99	S500,S505	TIADSEEEDSPLSDTAK	S5,S10	1.126	0.326
Hbs1l	HBS1-like protein	L7N209	S349	TNSLPFSQR	S3	0.983	0.782
Hbs1l	HBS1-like protein	L7N209; Q69ZS7	S63;S63	ESSNSLLNHQLSEIDQAR	S5	1.109	0.386
Hbs1l	HBS1-like protein	Q69ZS7	T229	SALPPPSLQTSEELGSTPTPVR	T17	0.930	0.544
Hcfc1	Host cell factor 1	B1AUX2	T1141	TTNTPTVVR	T4	0.957	0.143
Hcfc1	Host cell factor 1	B1AUX2	Ambiguous	VASSPVMVSNPATR	S/T	1.031	0.604
Hcfc1	Host cell factor 1	B1AUX2	Ambiguous	YDIPATAATATSPTPNPVPSVPANPPK	T/S/Y	0.882	0.046
Hck	Tyrosine-protein kinase	F6UND7	S209	TLDSGGFYISPR	S10	1.015	0.841
Hck	Tyrosine-protein kinase	F6UND7	S57	GPVYVPDPTSSSK	S11	0.957	0.271
Hck	Tyrosine-protein kinase	F6UND7	Y125	EGYIPSNYVAR	Y3	1.127	0.295
Hck	Tyrosine-protein kinase	F6UND7; P39688; P05480	Ambiguous	LIEDNEYTAR	T/Y	1.052	0.062
Hck	Tyrosine-protein kinase	F6UND7	Ambiguous	LIEDNEYTAREGAK	Y/T	0.957	0.774
Hcls1	Hematopoietic lineage cell-specific protein	P49710	S282	EVQQPSMPVEEPAAPAQLPK	S6	0.852	0.250
Hcls1	Hematopoietic lineage cell-specific protein	P49710	S333	EYPVPSLPTRQSPLQNHLEDNEEPPALPPR	S12	1.049	0.371
Hcls1	Hematopoietic lineage cell-specific protein	P49710	S333	QSPLQNHLEDNEEPPALPPR	S2	1.009	0.908
Hcls1	Hematopoietic lineage cell-specific protein	P49710	S333	REYPVPSLPTRQSPLQNHLEDNEEPPALPPR	S13	0.904	0.024
Hcls1	Hematopoietic lineage cell-specific protein	P49710	Y103	SAVGHEYVADVEK	Y7	0.964	0.696
Hcls1	Hematopoietic lineage cell-specific protein	P49710	Y198	GFGGQYGIQK	Y6	0.927	0.680
Hcls1	Hematopoietic lineage cell-specific protein	P49710	Y222	SAVGFNEMEAPTTAYK	Y15	0.884	0.070
Hcls1	Hematopoietic lineage cell-specific protein	P49710	Y323	EYPVPSLPTR	Y2	0.984	0.815
Hcls1	Hematopoietic lineage cell-specific protein	P49710	Y323	REYPVPSLPTR	Y3	0.956	0.514
Hcls1	Hematopoietic lineage cell-specific protein	P49710	Ambiguous	TTPIEAASSGAR	S/T	0.997	0.950
Hdac2	Histone deacetylase	A0A0R4J008	S394	MLPHAPGVQMQAIPEDAVHEDSGDEDGEDPDKR	S22	1.018	0.766
Hdac2	Histone deacetylase	A0A0R4J008	S422	IACDEEFSDSEDEGEGGRR	S8	1.046	0.233
Hdac2	Histone deacetylase	A0A0R4J008	S422,S424	IACDEEFSDSEDEGEGGRR	S8,S10	1.030	0.685
Hdac3	Histone deacetylase	Q3UM33	S424	GPEENYSRPEAPNEFYDGDHDNDKESDVEI	S26	0.893	0.358
Hdac4	Histone deacetylase 4	Q6NZM9	S562	QEPIESEEEEAEATR	S6	0.987	0.695
Hdac4	Histone deacetylase 4	Q6NZM9	S629	AQSSPASATFPMSVQEPPTKPR	S3	1.048	0.334
Hdac5	Histone deacetylase	B7ZDF5; Q6NZM9	S269;S245	TASEPNLK	S3	0.977	0.607
Hdac5	Histone deacetylase	B7ZDF5; Q6NZM9	S289;S265	RSSPLLR	S	0.914	0.083
Hdac5	Histone deacetylase	B7ZDF5	S620	DEDGESGPDEGPDLEESSAGYKK	S6	1.157	0.192
Hdac5	Histone deacetylase	B7ZDF5	S620	DEDGESGPDEGPDLEESSAGYK	S6	1.413	0.392
Hdac5	Histone deacetylase	B7ZDF5	Ambiguous	TQSSPAAPGSMK	S/T	0.950	0.208
Hdac6	Histone deacetylase 6	Q9Z2V5	S43	CAVPHSSPNLAEVK	S7	0.990	0.930
Hdac7	Histone deacetylase 7	Q8C2B3	S178	TVSEPNLK	S3	0.990	0.805
Hdac7	Histone deacetylase 7	Q8C2B3	S204	KESAPPSLR	S3	1.016	0.755
Hdgf	Hepatoma-derived growth factor	P51859	S165	AGDVLEDSPKRPK	S8	0.987	0.760
Hdgf	Hepatoma-derived growth factor	P51859	S165	AGDVLEDSPK	S8	1.004	0.975
Hdgf	Hepatoma-derived growth factor	P51859	S165	RAGDVLEDSPK	S9	1.028	0.817
Hdgf	Hepatoma-derived growth factor	P51859	S202	NSTPSEPDSGQGPPAEEEEGEEEAAK	S5	1.014	0.915
Hdgf	Hepatoma-derived growth factor	P51859	T200	NSTPSEPDSGQGPPAEEEEGEEEAAKEEAEAQGVR	T3	0.970	0.823
Hdgf	Hepatoma-derived growth factor	P51859	S132,S133	GSAEGSSDEEGKLVIDEPAK	S6,S7	0.993	0.882
Hdgf	Hepatoma-derived growth factor	P51859	Ambiguous	GSAEGSSDEEGKLVIDEPAK	S	1.024	0.808
Hdgf	Hepatoma-derived growth factor	P51859	Ambiguous	SCAAEPEVEPEAHEGDGDKKGSAEGSSDEEGK	S	1.065	0.271
Hdgfl2	Hepatoma-derived growth factor-related protein 2	Q3UMU9	S367	GGSSGEELEDEEPVK	S4	1.067	0.260
Hdgfl2	Hepatoma-derived growth factor-related protein 2	Q3UMU9	S454	SEGLSLER	S5	0.972	0.456
Hdgfl2	Hepatoma-derived growth factor-related protein 2	Q3UMU9	S620	AQEDGQDSEDGPR	S8	0.909	0.712
Hdgfl2	Hepatoma-derived growth factor-related protein 2	Q3UMU9	S635	GGSSEELHDSPRDNSDPAKPGNER	S10	0.868	0.081
Hdgfl2	Hepatoma-derived growth factor-related protein 2	Q3UMU9	S661	LASESANDDNEDS	S5	1.052	0.077
Hdgfl2	Hepatoma-derived growth factor-related protein 2	Q3UMU9	S366,S367	GGSSGEELEDEEPVK	S3,S4	1.047	0.136
Hdgfl2	Hepatoma-derived growth factor-related protein 2	Q3UMU9	S366,S367	GGSSGEELEDEEPVKKR	S3,S4	0.947	0.265
Hdgfl2	Hepatoma-derived growth factor-related protein 2	Q3UMU9	S366,S367	AERGGSSGEELEDEEPVK	S6,S7	1.027	0.211
Hdgfl2	Hepatoma-derived growth factor-related protein 2	Q3UMU9	S366,S367	AERGGSSGEELEDEEPVKK	S6,S7	0.998	0.957
Hdgfl2	Hepatoma-derived growth factor-related protein 2	Q3UMU9	Ambiguous	AQEDGQDSEDGPRGGSSEELHDSPR	S	0.959	0.774
Hdgfl2	Hepatoma-derived growth factor-related protein 2	Q3UMU9	Ambiguous	AERGGSSGEELEDEEPVK	S	0.972	0.622
Hdgfl2	Hepatoma-derived growth factor-related protein 2	Q3UMU9	Ambiguous	TRLASESANDDNEDS	T/S	1.005	0.971
Hdlbp	Vigilin	Q8VDJ3	S31	VATLNSEEENDPPTYK	S6	1.060	0.247
Heatr3	HEAT repeat-containing protein 3	Q8BQM4	S144	ECLSGLDSNEMSPQEK	S12	1.057	0.571
Heatr3	HEAT repeat-containing protein 3	Q8BQM4	S144	ECLSGLDSNEMSPQEK	S12	0.985	0.752
Heatr5a	HEAT repeat-containing protein 5A	Q5PRF0	S1647	SAEVDDGASEK	S1	1.079	0.104
Heatr5a	HEAT repeat-containing protein 5A	Q5PRF0	S1647	SAEVDDGASEKETLPEFGEGK	S1	1.036	0.512
Heatr5a	HEAT repeat-containing protein 5A	Q5PRF0	S1704	LAGSPGGK	S4	0.966	0.565
Heatr6	HEAT repeat-containing protein 6	Q6P1G0	S23	ELSPEQDDGFR	S3	1.168	0.278
Heatr6	HEAT repeat-containing protein 6	Q6P1G0	S23	ELSPEQDDGFRR	S3	1.006	0.817
Heatr6	HEAT repeat-containing protein 6	Q6P1G0	S23	EAPRELSPEQDDGFRR	S7	0.884	0.237
Heatr6	HEAT repeat-containing protein 6	Q6P1G0	S646	TFPAGSSLEEASLSSPK	S15	1.025	0.715
Heatr6	HEAT repeat-containing protein 6	Q6P1G0	S675	EDSCSGSDAGSALGSTYEPSPMR	S7	0.856	0.150
Heatr6	HEAT repeat-containing protein 6	Q6P1G0	Ambiguous	TFPAGSSLEEASLSSPK	S/T	0.954	0.801
Hebp1	Heme-binding protein 1	A0A140T8J4	S111	IPNQFQGSPPAPSDESVK	S8	0.982	0.825
Hectd1	E3 ubiquitin-protein ligase HECTD1	Q69ZR2	S1549	ETSSLESFVR	S4	0.983	0.607
Hectd1	E3 ubiquitin-protein ligase HECTD1	Q69ZR2	S1576	SSSDNNTNTLGR	S3	1.078	0.398
Hectd1	E3 ubiquitin-protein ligase HECTD1	Q69ZR2	S1575,S1576	TNATNNMNLSRSSSDNNTNTLGR	S13,S14	0.940	0.450
Hectd1	E3 ubiquitin-protein ligase HECTD1	Q69ZR2	Ambiguous	RLDSSGER	S	1.047	0.605
Hectd1	E3 ubiquitin-protein ligase HECTD1	Q69ZR2	Ambiguous	VSALAGPSSDDENEEESKPEKEDEPQEDAK	S	0.881	0.103
Hectd2	Hectd2 protein	B2RPZ5	S9	DLSPGAPPAVAAAAPEER	S3	1.007	0.949
Hectd4	HECT domain E3 ubiquitin protein ligase 4	E9Q2E4	S1498	SISGTPAETPACK	S3	1.807	0.052
Hectd4	HECT domain E3 ubiquitin protein ligase 4	E9Q2E4	T2503	TEGTPPPPGQPAK	T4	1.019	0.626
Hectd4	HECT domain E3 ubiquitin protein ligase 4	E9Q2E4	Ambiguous	SMSAPSDLEMIGNEDLEFTR	S/T	0.968	0.896
Helb	DNA helicase B	Q6NVF4	T992	WLSASVNDDVDTDEESAQLR	T12	1.099	0.737
Helb	DNA helicase B	Q6NVF4	S942,S/T	LSGSCAPSTGFASQPSSPR	S13,S/T	0.988	0.896
Helb	DNA helicase B	Q6NVF4	Ambiguous	LSGSCAPSTGFASQPSSPR	S/T	0.964	0.286
Helq	Helicase POLQ-like	Q2VPA6	T40	ASPTPAELQPAEDTEDEAAAGSR	T14	1.127	0.469
Helq	Helicase POLQ-like	Q2VPA6	S28,T40	GNLENLRASPTPAELQPAEDTEDEAAAGSR	S9,T21	1.085	0.304
Helz	Probable helicase with zinc finger domain	A0A0R4J0Y3	S1761	TVSASSLPSLEEYEPR	S3	0.969	0.747
Helz	Probable helicase with zinc finger domain	A0A0R4J0Y3	S1786	RISSSSAQPCVEEASAPQDSLAQGK	S3	1.093	0.051
Helz	Probable helicase with zinc finger domain	A0A0R4J0Y3	T1164	AYTPPPPLGPHPNLGK	T3	1.080	0.553
Helz	Probable helicase with zinc finger domain	A0A0R4J0Y3	S1761,S/T/Y	TVSASSLPSLEEYEPR	S3,S/T/Y	0.861	0.414
Helz	Probable helicase with zinc finger domain	A0A0R4J0Y3	Ambiguous	RISSSSAQPCVEEASAPQDSLAQGK	S	0.987	0.877
Herc1	HECT and RLD domain-containing E3 ubiquitin protein ligase family member 1	E9PZP8	S1428	RVSTDLPEGQDVYTAACNSVIHR	S3	0.963	0.682
Herc1	HECT and RLD domain-containing E3 ubiquitin protein ligase family member 1	E9PZP8	S1491	SESLTAESR	S3	1.389	0.000
Herc1	HECT and RLD domain-containing E3 ubiquitin protein ligase family member 1	E9PZP8	S4855	NVDNAEGSDTDY	S8	1.007	0.854
Herc1	HECT and RLD domain-containing E3 ubiquitin protein ligase family member 1	E9PZP8	T2699	AQTPPISSLPASPSDEVGRR	T3	0.974	0.501
Herc1	HECT and RLD domain-containing E3 ubiquitin protein ligase family member 1	E9PZP8	T2699	AQTPPISSLPASPSDEVGR	T3	0.984	0.841
Herc1	HECT and RLD domain-containing E3 ubiquitin protein ligase family member 1	E9PZP8	T2699	RAQTPPISSLPASPSDEVGR	T4	1.081	0.282
Herc1	HECT and RLD domain-containing E3 ubiquitin protein ligase family member 1	E9PZP8	T2699	RAQTPPISSLPASPSDEVGRR	T4	1.047	0.526
Herc1	HECT and RLD domain-containing E3 ubiquitin protein ligase family member 1	E9PZP8	S1510,S1521	SRSESDLSQPESDEEGYALSGR	S1,S12	0.928	0.639
Herc1	HECT and RLD domain-containing E3 ubiquitin protein ligase family member 1	E9PZP8	S2708,S/T	AQTPPISSLPASPSDEVGRR	S12,S/T	0.948	0.511
Herc1	HECT and RLD domain-containing E3 ubiquitin protein ligase family member 1	E9PZP8	Ambiguous	AGLSTSPSAIASTSER	S/T	1.129	0.150
Herc1	HECT and RLD domain-containing E3 ubiquitin protein ligase family member 1	E9PZP8	Ambiguous	SRSESDLSQPESDEEGYALSGR	S/Y	1.032	0.372
Hexim2	Protein HEXIM2	Q3TVI4	S75	TQSPGGCSVEAVLAR	S3	0.980	0.781
Hgsnat	Heparan-alpha-glucosaminide N-acetyltransferase	Q3UDW8	S234	LINSELGSPSR	S4	1.116	0.164
Hgsnat	Heparan-alpha-glucosaminide N-acetyltransferase	Q3UDW8	S238	LINSELGSPSRADPLSADYQPETR	S8	1.241	0.407
Hibadh	3-hydroxyisobutyrate dehydrogenase, mitochondrial	Q99L13	S86	EAGEQVASSPAEVAEK	S8	0.888	0.278
Hid1	Protein HID1	Q8R1F6	S593	TGSQEGTSMEGSRPAAPAEPGTLK	S3	1.123	0.141
Hid1	Protein HID1	Q8R1F6	S655	SLEPESQQSSAENSPSDGESSQTWR	S16	1.021	0.925
Hint2	Histidine triad nucleotide-binding protein 2, mitochondrial	Q9D0S9	S52	AAPGGASPTIFSR	S7	1.037	0.473
Hipk1	Homeodomain-interacting protein kinase 1	O88904	S1200	GSTIYTGYPLSPTK	S11	1.069	0.512
Hipk1	Homeodomain-interacting protein kinase 1	O88904; Q9QZR5	T351;T360	AVCSTYLQSR	T5	0.950	0.074
Hipk2	Homeodomain-interacting protein kinase 2	Q9QZR5	S827	NVSTCEVTSSQAISSPQR	S15	0.974	0.754
Hipk3	Homeodomain-interacting protein kinase 3	A2AQH3	Ambiguous	TVCSTYLQSR	T/Y/S	0.940	0.020
Hirip3	HIRA-interacting protein 3	Q8BLH7	S134	AVESTDEDHQTDLDAK	S4	0.961	0.272
Hirip3	HIRA-interacting protein 3	Q8BLH7	S153	MGLEESSEGEAEGSVR	S7	0.963	0.620
Hirip3	HIRA-interacting protein 3	Q8BLH7	S366	DDSNSTQEQAAAQGTTK	S5	1.015	0.817
Hirip3	HIRA-interacting protein 3	Q8BLH7	S389	SGSLGSSNGDSDTEREVSDSQAGQNTK	S11	1.036	0.286
Hirip3	HIRA-interacting protein 3	Q8BLH7	S564	NAWNPSGEGTSPGETYR	S11	1.015	0.836
Hirip3	HIRA-interacting protein 3	Q8BLH7	S564	NAWNPSGEGTSPGETYRR	S11	0.810	0.385
Hirip3	HIRA-interacting protein 3	Q8BLH7	S575	TLDSEEEQPR	S4	0.980	0.531
Hirip3	HIRA-interacting protein 3	Q8BLH7	S96	FNSESESSSSPSSPDGSGPSTK	S3	1.141	0.105
Hirip3	HIRA-interacting protein 3	Q8BLH7	S134,T141	AVESTDEDHQTDLDAK	S4,T11	0.978	0.683
Hirip3	HIRA-interacting protein 3	Q8BLH7	S231,S234	VEGSTGANCQEESEESGEESPAK	S13,S16	1.048	0.830
Hirip3	HIRA-interacting protein 3	Q8BLH7	Ambiguous	DIISSDGDSS	S	0.918	0.198
Hist1h1b	Histone H1.5	P43276; P43277	S102;S103	GTGASGSFK	S5	1.181	0.084
Hist1h2bq	Histone H2B	Q8CBB6	S39	ESYSVYVYK	S4	1.117	0.201
Hist1h2bq	Histone H2B	Q8CBB6	S57	QVHPDTGISSK	S10	0.920	0.381
Hist1h3a	Histone H3.1	P68433; A0A1W2P768	S29;S74	SAPATGGVK	S1	1.266	0.108
Hist1h4a	Histone H4	P62806	S48	RISGLIYEETR	S3	1.065	0.248
Hivep1	Zinc finger protein 40	F8VPM9	S682	SESADQAVSPPTPFAR	S9	0.911	0.575
Hivep1	Zinc finger protein 40	F8VPM9	S774	RGSIDSPK	S3	1.041	0.744
Hivep2	Transcription factor HIVEP2	Q3UHF7	S1062	SFDYGNLSHAPVAGTSPSTLSPSRER	S21	1.014	0.809
Hivep2	Transcription factor HIVEP2	Q3UHF7	S2423	ELSLSTEEGNEPSPEKNQLH	S13	1.081	0.409
Hivep2	Transcription factor HIVEP2	Q3UHF7	Ambiguous	QAPQVLQSSGLPSSPSSPR	S	0.901	0.658
Hivep2	Transcription factor HIVEP2	Q3UHF7	Ambiguous	QSTSEDSLNSTEREQEENIQTCTK	T/S	1.101	0.454

Hivep3	Transcription factor HIVEP3	A2A884	S1032	QASLNRPPEAELEAVPK	S3	0.973	0.854
Hivep3	Transcription factor HIVEP3	A2A884	S1922	DMSSAPSPK	S7	0.968	0.585
Hivep3	Transcription factor HIVEP3	A2A884	S1991	DLGPHLCGSPR	S9	0.934	0.669
Hivep3	Transcription factor HIVEP3	A2A884	S398	SESAEQQVSPPNTNAK	S9	0.973	0.555
Hivep3	Transcription factor HIVEP3	A2A884	S489	RSSVESPK	S3	1.098	0.554
Hivep3	Transcription factor HIVEP3	A2A884	S2199,S2209	SRWSPTESPSASVSPVAK	S4,S14	1.041	0.819
Hivep3	Transcription factor HIVEP3	A2A884	Ambiguous	SSSMDCLAETSTYSPPR	T/S/Y	0.978	0.805
Hjurp	Holliday junction recognition protein	E9QKG8	S463	QDVETCRPSSPFGR	S10	0.984	0.774
Hjurp	Holliday junction recognition protein	E9QKG8	S602	LNPDSPQQSSQKR	S5	0.974	0.730
Hk1	Hexokinase-1	P17710	S471	TTVGVDGSLYK	S8	1.233	0.309
Hk3	Hexokinase-3	Q3TRM8	S189	CSGVEGQDVVQLLR	S2	0.996	0.969
Hk3	Hexokinase-3	Q3TRM8	S65	GQDSPAPSVR	S4	1.081	0.604
Hlcs	Biotin--protein ligase	A0A2I3BPG4	S271	SASAENIPDLPCDHSGVEGAAGELCPERK	S3	0.904	0.287
Hltf	Helicase-like transcription factor	Q6PCN7	S21	YSQSVQYGSHENIPR	S9	0.968	0.527
Hmg20a	High mobility group protein 20A	Q9DC33	S104	DSNAPKSPLTGYVR	S7	1.001	0.985
Hmga1	High mobility group protein HMG-I/HMG-Y	A0A338P6E8	S103	LEKEEEEGISQESSEEEQ	S10	1.048	0.476
Hmga1	High mobility group protein HMG-I/HMG-Y	A0A338P6E8; Q3TE85	S106;S101	EEEEGISQESSEEEQ	S10	0.939	0.504
Hmga1	High mobility group protein HMG-I/HMG-Y	A0A338P6E8; Q3TE85	S9;S9	SSQPLASK	S2	1.037	0.588
Hmga1	High mobility group protein HMG-I/HMG-Y	A0A338P6E8; Q3TE85	T57;T53	EPSEVPTPK	T7	0.971	0.647
Hmga1	High mobility group protein HMG-I/HMG-Y	A0A338P6E8	S106,S107	KLEKEEEEGISQESSEEEQ	S14,S15	1.006	0.849
Hmga1	High mobility group protein HMG-I/HMG-Y	A0A338P6E8	Ambiguous	KLEKEEEEGISQESSEEEQ	S	1.032	0.608
Hmga1	High mobility group protein HMG-I/HMG-Y	Q3TE85	Ambiguous	KLKEEEEGISQESSEEEQ	S	0.998	0.982
Hmga2	High mobility group protein HMGI-C	P52927	S100	KPAQETEETSSQESAEED	S10	1.063	0.460
Hmga2	High mobility group protein HMGI-C	P52927	S44	QQQEPTCEPSPK	S10	1.016	0.836
Hmga2	High mobility group protein HMGI-C	P52927	S44	KQQQEPTCEPSPK	S11	1.046	0.579
Hmga2	High mobility group protein HMGI-C	P52927	T99,S101	KPAQETEETSSQESAEED	T9,S11	1.084	0.188
Hmgn1	Non-histone chromosomal protein HMG-14	P18608	S7	KVSADGAAK	S3	1.181	0.063
Hmgn1	Non-histone chromosomal protein HMG-14	P18608	S84	QADVADQQTTELPAENGETENQSPASEEEK	S23	0.984	0.848
Hmgn1	Non-histone chromosomal protein HMG-14	P18608	S84	GKQADVADQQTTELPAENGETENQSPASEEEK	S25	0.997	0.978
Hmgn1	Non-histone chromosomal protein HMG-14	P18608	S87	QADVADQQTTELPAENGETENQSPASEEEKEAK	S26	0.995	0.913
Hmgn1	Non-histone chromosomal protein HMG-14	P18608	S95	QADVADQQTTELPAENGETENQSPASEEEKEAKSD	S34	0.933	0.644
Hmgn1	Non-histone chromosomal protein HMG-14	P18608	S84,S87	QADVADQQTTELPAENGETENQSPASEEEKEAK	S23,S26	0.975	0.653
Hmgn1	Non-histone chromosomal protein HMG-14	P18608	S87,S95	QADVADQQTTELPAENGETENQSPASEEEKEAKSD	S26,S34	1.016	0.869
Hmgn3	High mobility group nucleosome-binding domain-containing protein 3	Q9DCB1	S93	VEEAQRTESIEKEGE	S9	1.059	0.505
Hmgxb4	HMG box domain-containing 4	Q80Y32	S105	SSPQSPDTAMDLLK	S5	0.917	0.583
Hmox1	Heme oxygenase 1	P14901	T252	QRPASLVQDTAPAETPR	T15	0.990	0.764
Hnrnpa1	Heterogeneous nuclear ribonucleoprotein A1	Q5EBP8	S199	SGSGNFGGGR	S3	1.051	0.454
Hnrnpa1	Heterogeneous nuclear ribonucleoprotein A1	Q5EBP8	S257	GGGGYGGSGDGYNGFGNDGGYGGGSPGYSGGSR	S25	1.053	0.326
Hnrnpa1	Heterogeneous nuclear ribonucleoprotein A1	Q5EBP8	S339	SSGPYGGGGQYFAKPR	S2	1.028	0.563
Hnrnpa1	Heterogeneous nuclear ribonucleoprotein A1	Q5EBP8	S6	SESPKEPEQLRK	S3	0.991	0.928
Hnrnpa1	Heterogeneous nuclear ribonucleoprotein A1	Q5EBP8	S6	SESPKEPEQLR	S3	1.004	0.952
Hnrnpa2b1	Heterogeneous nuclear ribonucleoproteins A2/B1	O88569	S225	GGGGNFGPGPGSNFR	S12	0.951	0.717
Hnrnpa2b1	Heterogeneous nuclear ribonucleoproteins A2/B1	O88569	S259	GFGDGYNGYGGGPGGGNFGGSPGYGGGR	S21	1.033	0.797
Hnrnpa2b1	Heterogeneous nuclear ribonucleoproteins A2/B1	O88569	S344	NMGGPYGGGNYGPGGSGGSGGYGGR	S19	1.089	0.298
Hnrnpa3	Heterogeneous nuclear ribonucleoprotein A3	Q8BG05	S371	SSGSPYGGGYGSGGGSGGYGSR	S16	1.004	0.898
Hnrnpa3	Heterogeneous nuclear ribonucleoprotein A3	Q8BG05	S359,S371	SSGSPYGGGYGSGGGSGGYGSR	S4,S16	1.019	0.872
Hnrnpab	Heterogeneous nuclear ribonucleoprotein A/B	Q99020	S247	EVYQQQQYGSGGR	S10	1.039	0.735
Hnrnpab	Heterogeneous nuclear ribonucleoprotein A/B	Q99020	S260	GSGGGQGSTNYGK	S2	1.027	0.642
Hnrnpc	Heterogeneous nuclear ribonucleoproteins C1/C2	Q9Z204	S232	QADLSFSSPVEMK	S8	1.024	0.624
Hnrnpc	Heterogeneous nuclear ribonucleoproteins C1/C2	Q9Z204	S241	NEKSEEEQSSASVK	S4	0.970	0.378
Hnrnpc	Heterogeneous nuclear ribonucleoproteins C1/C2	Q9Z204	S249	SEEEQSSASVK	S9	1.110	0.224
Hnrnpc	Heterogeneous nuclear ribonucleoproteins C1/C2	Q9Z204	S268	MESEAGADDSAEEGDLLDDDDNEDRGDDQLELK	S10	1.095	0.549
Hnrnpc	Heterogeneous nuclear ribonucleoproteins C1/C2	Q9Z204	S268	MESEAGADDSAEEGDLLDDDDNEDR	S10	1.076	0.487
Hnrnpc	Heterogeneous nuclear ribonucleoproteins C1/C2	Q9Z204	S313	DDEKEPEEGEDDRDSANGEDDS	S22	1.070	0.389
Hnrnpc	Heterogeneous nuclear ribonucleoproteins C1/C2	Q9Z204	Ambiguous	SEEEQSSASVKKDETNVK	S/T	0.973	0.689
Hnrnpd	Heterogeneous nuclear ribonucleoprotein D0	Q60668	S190	IFVGGLSPDTPEEK	S7	0.891	0.363
Hnrnpd	Heterogeneous nuclear ribonucleoprotein D0	Q60668	S80,S83	IDASKNEEDEGHSNSSPR	S13,S16	1.010	0.663
Hnrnpd	Heterogeneous nuclear ribonucleoprotein D0	Q60668	Ambiguous	IDASKNEEDEGHSNSSPR	S	1.037	0.721
Hnrnpf	Heterogeneous nuclear ribonucleoprotein F	Q9Z2X1	S104	HSGPNSADSANDGFVR	S6	0.990	0.391
Hnrnpf	Heterogeneous nuclear ribonucleoprotein F	Q9Z2X1	S193	SSQEEVRSYSDPPLK	S8	0.876	0.386
Hnrnpf	Heterogeneous nuclear ribonucleoprotein F	Q9Z2X1	S63	QSGEAFVELESEDDVK	S11	0.859	0.476
Hnrnph1	Heterogeneous nuclear ribonucleoprotein H	Q8C2Q7; P70333	S104;S104	HTGPNSPDTANDGFVR	S6	1.005	0.931
Hnrnph1	Heterogeneous nuclear ribonucleoprotein H	Q8C2Q7	S23	GLPWSCSADEVQR	S7	1.376	0.025
Hnrnph1	Heterogeneous nuclear ribonucleoprotein H	Q8C2Q7; P70333	S272;S272	DLNYCFSGMSDHR	S10	1.131	0.212
Hnrnph1	Heterogeneous nuclear ribonucleoprotein H	Q8C2Q7; P70333	S63;S63	EGRPSGEAFVELESEDEVK	S14	1.022	0.831
Hnrnph2	Heterogeneous nuclear ribonucleoprotein H2	P70333	S23	GLPWSCSAEEVMR	S7	0.870	0.543
Hnrnpk	Heterogeneous nuclear ribonucleoprotein K	B2M1R6	S260	DYDDMSPR	S6	0.987	0.626
Hnrnpk	Heterogeneous nuclear ribonucleoprotein K	B2M1R6	S260	DYDDMSPR	S6	1.025	0.814
Hnrnpk	Heterogeneous nuclear ribonucleoprotein K	B2M1R6	S260	RDYDDMSPR	S7	0.894	0.157
Hnrnpk	Heterogeneous nuclear ribonucleoprotein K	B2M1R6	S260	RDYDDMSPR	S7	0.996	0.840
Hnrnpk	Heterogeneous nuclear ribonucleoprotein K	B2M1R6	S406	IDEPLEGSEDR	S8	1.103	0.248
Hnrnpk	Heterogeneous nuclear ribonucleoprotein K	B2M1R6	S77	TDYNASVSVPDSSGPER	S8	0.947	0.613
Hnrnpl	Heterogeneous nuclear ribonucleoprotein L (Fragment)	G5E924	S174	ISRPGDSDDSR	S7	1.063	0.472
Hnrnpl	Heterogeneous nuclear ribonucleoprotein L (Fragment)	G5E924	S90	TPASPVVHIR	S4	0.961	0.452
Hnrnpl	Heterogeneous nuclear ribonucleoprotein L (Fragment)	G5E924	T513	FSTPEQAAK	T3	1.020	0.862
Hnrnpll	Heterogeneous nuclear ribonucleoprotein L-like	Q921F4	S37	TEEGEIVYSAEESENR	S9	1.147	0.158
Hnrnpm	Heterogeneous nuclear ribonucleoprotein M	Q9D0E1	S396	QGGGGAGGSVPGIER	S9	0.976	0.519
Hnrnpm	Heterogeneous nuclear ribonucleoprotein M	Q9D0E1	S539	MVPTGMGASLER	S9	0.944	0.491
Hnrnpm	Heterogeneous nuclear ribonucleoprotein M	Q9D0E1	S700	GCGVVKFESPEVAER	S9	0.885	0.119
Hnrnpul1	Heterogeneous nuclear ribonucleoprotein U-like protein 1	Q8VDM6	S513	NYILDQTNVYGSAQR	S12	1.128	0.377
Hnrnpul1	Heterogeneous nuclear ribonucleoprotein U-like protein 1	Q8VDM6	S721	APQQQPPPQQPPPPQPPPQQPPPPPSYSPAR	S28	1.017	0.751
Hnrnpul2	Heterogeneous nuclear ribonucleoprotein U-like protein 2	Q00PI9	S159	SGDETPGSEAPGDK	S1	1.050	0.469
Hnrnpul2	Heterogeneous nuclear ribonucleoprotein U-like protein 2	Q00PI9	S166	SGDETPGSEAPGDKAVEEQGDDQDSEK	S8	0.983	0.744
Hnrnpul2	Heterogeneous nuclear ribonucleoprotein U-like protein 2	Q00PI9	S183	AVEEQGDDQDSEK	S11	0.928	0.457
Hnrnpul2	Heterogeneous nuclear ribonucleoprotein U-like protein 2	Q00PI9	S183	AVEEQGDDQDSEKSKPAGSDGER	S11	1.006	0.950
Hnrnpul2	Heterogeneous nuclear ribonucleoprotein U-like protein 2	Q00PI9	S191	AVEEQGDDQDSEKSKPAGSDGERR	S19	0.966	0.488
Hnrnpul2	Heterogeneous nuclear ribonucleoprotein U-like protein 2	Q00PI9	S183,S186	AVEEQGDDQDSEKSKPAGSDGER	S11,S14	0.980	0.877
Hnrnpul2	Heterogeneous nuclear ribonucleoprotein U-like protein 2	Q00PI9	S183,S186	AVEEQGDDQDSEKSKPAGSDGERR	S11,S14	0.868	0.296
Hook2	Protein Hook homolog 2	Q7TMK6	Ambiguous	DTPDSLSPENYGNFDTQSR	S/Y/T	0.954	0.916
Hook3	Protein Hook homolog 3	Q8BUK6	S238	LNQSDSIEDPNSPAGRR	S12	0.999	0.993
Hook3	Protein Hook homolog 3	Q8BUK6	S707	SYPGHVQPATAR	S1	1.020	0.722
Hp1bp3	Heterochromatin protein 1-binding protein 3 (Fragment)	A2AM62	T34	LAEGEEEKPEEQENETPPATSSEAEQPK	T16	1.215	0.112
Hp1bp3	Heterochromatin protein 1-binding protein 3	Q3TEA8	S513	ARPSPSVIK	S4	1.180	0.074
Hpcal1	Hippocalcin-like protein 1	P62748	Ambiguous	LLQCDPSSASQF	S	0.832	0.476
Hps1	Hermansky-Pudlak syndrome 1 protein homolog	Q3U309	S278	SSQNIPVQQAR	S2	0.959	0.757
Hps4	Hermansky-Pudlak syndrome 4 protein homolog	Q99KG7	S478	SPDSPGPSPSADR	S4	1.013	0.690
Hps4	Hermansky-Pudlak syndrome 4 protein homolog	Q99KG7	S478,S482	SPDSPGPSPSADR	S4,S8	1.036	0.462
Hps5	Hermansky-Pudlak syndrome 5 protein homolog	P59438	S662	DFPGISNTSSPTVK	S10	1.280	0.106
Hps5	Hermansky-Pudlak syndrome 5 protein homolog	P59438	S693	RVSLVTEEAGGQITCDPVSNLSEPSADR	S3	1.008	0.947
Hsd17b4	Peroxisomal multifunctional enzyme type 2	P51660	S308	VDSEGISPNR	S7	1.043	0.500
Hsd17b8	Estradiol 17-beta-dehydrogenase 8	P50171	S58	LLGSPGSEDGAPR	S7	0.989	0.806
Hsf1	Heat shock factor protein 1	A0A075F5C6	T365	ESEPTPAASNTAPMDTTGAQAPALPTPSTPEK	T29	1.157	0.190
Hsf1	Heat shock factor protein 1	A0A075F5C6	S303,S307	VKQEPPSPPHSPR	S7,S11	1.077	0.375
Hsp90aa1	Heat shock protein HSP 90-alpha	P07901	S231	DKEVSDDEAEEKEEK	S5	0.925	0.597
Hsp90aa1	Heat shock protein HSP 90-alpha	P07901	S231	DKEVSDDEAEEK	S5	0.955	0.888
Hsp90aa1	Heat shock protein HSP 90-alpha	P07901	S263	ESDDKPEIEDVGSDEEEEK	S13	1.120	0.393
Hsp90aa1	Heat shock protein HSP 90-alpha	P07901	S263	ESDDKPEIEDVGSDEEEEKK	S13	1.040	0.734
Hsp90aa1	Heat shock protein HSP 90-alpha	P07901	S263	ESDDKPEIEDVGSDEEEEKKDGDK	S13	1.006	0.959
Hsp90aa1	Heat shock protein HSP 90-alpha	P07901	S263	EEKESDDKPEIEDVGSDEEEEK	S16	0.954	0.592
Hsp90aa1	Heat shock protein HSP 90-alpha	P07901	S454	LGIHEDSQNR	S7	0.932	0.616
Hsp90ab1	Heat shock protein HSP 90-beta	P11499	S226	EKEISDDEAEEEKGEK	S5	0.960	0.615
Hsp90ab1	Heat shock protein HSP 90-beta	P11499	S226	EKEISDDEAEEEK	S5	1.003	0.979
Hsp90ab1	Heat shock protein HSP 90-beta	P11499	S226	EREKEISDDEAEEEK	S7	1.062	0.463
Hsp90ab1	Heat shock protein HSP 90-beta	P11499	S255	IEDVGSDEEDDSGK	S6	1.043	0.540
Hsp90ab1	Heat shock protein HSP 90-beta	P11499	S255	IEDVGSDEEDDSGKDKK	S6	0.987	0.882
Hsp90ab1	Heat shock protein HSP 90-beta	P11499	S255	IEDVGSDEEDDSGKDK	S6	1.055	0.611
Hsp90ab1	Heat shock protein HSP 90-beta	P11499	Y484	SIYYITGESK	Y3	1.049	0.534
Hsp90b1	Endoplasmin	P08113	S447	GVVDSDDLPLNVSR	S13	1.048	0.746
Hsp90b1	Endoplasmin	P08113	Ambiguous	DISTNYYASQK	S/T/Y	1.162	0.214
Hspa14	Heat shock 70 kDa protein 14	Q99M31	S61	HVSSTVVK	S3	0.984	0.864
Hspa14	Heat shock 70 kDa protein 14	Q99M31	Ambiguous	ESTSGDDSVMIECSAK	T/S	0.959	0.614
Hspa4	Heat shock 70 kDa protein 4	Q3U2G2	S31	AGGIETIANEYSDR	S12	0.987	0.901
Hspa4	Heat shock 70 kDa protein 4	Q3U2G2	S76	AFSDPFVEAEK	S3	1.040	0.632
Hspa4	Heat shock 70 kDa protein 4	Q3U2G2	T540	MQVDQEEPHTEEQQQQPQTPAENK	T19	1.433	0.279
Hspa4	Heat shock 70 kDa protein 4	Q3U2G2	Y626	NAVEEYVYEMR	Y6	1.196	0.034
Hspa4	Heat shock 70 kDa protein 4	Q3U2G2	Y725	NKEDQYEHLDAADVTK	Y6	1.052	0.590
Hspa4	Heat shock 70 kDa protein 4	Q3U2G2	Ambiguous	HAEQNGPVDGQGDNPGSQAAEHGADTAVPSDGDK	T/S	1.087	0.315
Hspa5	Endoplasmic reticulum chaperone BiP	P20029	T649	LYGSGGPPPTGEEDTSEKDEL	T15	1.038	0.616
Hspa8	Heat shock cognate 71 kDa protein	P63017; P17879; P17156	S40;S40;S41	TTPSYVAFTDTER	S4	1.108	0.558
Hspa8	Heat shock cognate 71 kDa protein	P63017; P17156	T226;T229	STAGDTHLGGEDFDNR	T6	1.157	0.062
Hspa9	Stress-70 protein, mitochondrial	P38647	Ambiguous	TTPSVVAFTADGER	T/S	1.016	0.956
Hspb1	Heat shock protein beta-1	P14602	S86	QLSSGVSEIR	S3	1.034	0.718
Hspbp1	Hsp70-binding protein 1	Q99P31	S349	LLQTCFSSPTDDSMDR	S8	1.039	0.467
Hspbp1	Hsp70-binding protein 1	Q99P31	Ambiguous	LLQTCFSSPTDDSMDR	S/T	0.902	0.472
Hsph1	Heat shock protein 105 kDa	Q61699	S852	NNLGAEAPHQNGECHPNEKGSVNMDLD	S21	0.897	0.225
Htatsf1	HIV Tat-specific factor 1 homolog	Q8BGC0	S441	AEEGGESEGDASEK	S7	0.991	0.743
Htatsf1	HIV Tat-specific factor 1 homolog	Q8BGC0	S441	AEEGGESEGDASEKDAK	S7	0.997	0.924
Htatsf1	HIV Tat-specific factor 1 homolog	Q8BGC0	S528	ESEDNDLGKESEGEDSLK	S11	1.071	0.683

Htatsf1	HIV Tat-specific factor 1 homolog	Q8BGC0	S613	VLDEEGSER	S7	0.996	0.961
Htatsf1	HIV Tat-specific factor 1 homolog	Q8BGC0	S621	EFEEDSDEKEEEGDDDEEEVVYER	S6	0.868	0.291
Htatsf1	HIV Tat-specific factor 1 homolog	Q8BGC0	S679	LFDDSDEKEDEEDTDGK	S5	0.936	0.553
Htatsf1	HIV Tat-specific factor 1 homolog	Q8BGC0	S705	DDDASDKVFEDNSNEKLFDEEEGPNEK	S13	1.103	0.400
Htatsf1	HIV Tat-specific factor 1 homolog	Q8BGC0	S705	VFEDNSNEK	S6	0.834	0.039
Htatsf1	HIV Tat-specific factor 1 homolog	Q8BGC0	S705	VFEDNSNEKLFDEEEGPNEK	S6	0.937	0.653
Htatsf1	HIV Tat-specific factor 1 homolog	Q8BGC0	S724	LFDDSDER	S5	1.025	0.500
Htatsf1	HIV Tat-specific factor 1 homolog	Q8BGC0	S724	LFDDSDERGTVGNVK	S5	1.003	0.965
Htatsf1	HIV Tat-specific factor 1 homolog	Q8BGC0	S441,S446	AEEGGESEGDASEKDAK	S7,S12	1.045	0.613
Htt	Huntingtin	G3X9H5	S1180	EKEPGEQASTPMSPK	S13	1.182	0.009
Htt	Huntingtin	G3X9H5	S1854	SLNPQKSGEEEDSGSAAQLGMCNR	S7	1.029	0.635
Htt	Huntingtin	G3X9H5	S2094	SDSALLEGAELVNR	S3	0.891	0.555
Htt	Huntingtin	G3X9H5	S2917	ASPGRASDPSPATPDSESVIVAMER	S7	0.771	0.044
Htt	Huntingtin	G3X9H5	S621	QPSDSSIDK	S3	1.170	0.012
Htt	Huntingtin	G3X9H5	Ambiguous	ASPGRASDPSPATPDSESVIVAMER	S/T	1.010	0.913
Huwe1	E3 ubiquitin-protein ligase HUWE1	A2AFQ0	S1395	AIAMSLGQDIPMDQRAESPEEVACR	S18	1.005	0.952
Huwe1	E3 ubiquitin-protein ligase HUWE1	A2AFQ0	S1395	AESPEEVACR	S3	1.129	0.188
Huwe1	E3 ubiquitin-protein ligase HUWE1	A2AFQ0	S1395	AESPEEVACRKEEEER	S3	0.972	0.580
Huwe1	E3 ubiquitin-protein ligase HUWE1	A2AFQ0	S1907	GSGTASDDEFENLR	S6	0.988	0.764
Huwe1	E3 ubiquitin-protein ligase HUWE1	A2AFQ0	S2363	DGGSGNSTIIVSR	S4	1.002	0.892
Huwe1	E3 ubiquitin-protein ligase HUWE1	A2AFQ0	S2378	SGEDESQEDVLMDEAPSNLSQASTLQANR	S6	1.309	0.353
Huwe1	E3 ubiquitin-protein ligase HUWE1	A2AFQ0	S2547	LTQGIGRSQR	S8	1.083	0.414
Huwe1	E3 ubiquitin-protein ligase HUWE1	A2AFQ0	S3664	AQCETLSPDGLPEEQPQTTK	S7	1.077	0.327
Huwe1	E3 ubiquitin-protein ligase HUWE1	A2AFQ0	S3819	REESPMDVDQPSPSAQDTQSIVISDGTPQGEK	S12	1.299	0.222
Huwe1	E3 ubiquitin-protein ligase HUWE1	A2AFQ0	T2739	TNDSTEQNVSDGTPMPDSYPTTPSSTDAPTSESK	T13	1.040	0.520
Huwe1	E3 ubiquitin-protein ligase HUWE1	A2AFQ0	S2363,S/T	DGGSGNSTIIVSR	S4,S/T	1.045	0.487
Huwe1	E3 ubiquitin-protein ligase HUWE1	A2AFQ0	Ambiguous	ESNQPPEDSSPPASSESSSTR	S/T	0.942	0.758
Huwe1	E3 ubiquitin-protein ligase HUWE1	A2AFQ0	Ambiguous	REESPMDVDQPSPSAQDTQSIVISDGTPQGEKEK	S/T	0.963	0.787
Huwe1	E3 ubiquitin-protein ligase HUWE1	A2AFQ0	Ambiguous	HADHSSLTLGSGSSTTR	S/T	1.001	0.989
Huwe1	E3 ubiquitin-protein ligase HUWE1	A2AFQ0	Ambiguous	TNDSTEQNVSDGTPMPDSYPTTPSSTDAPTSESK	T/S/Y	0.609	0.074
Ice1	Little elongation complex subunit 1	E9Q286	S1616	SSQTLSPLVPNSGPSSR	S6	1.121	0.283
Ice1	Little elongation complex subunit 1	E9Q286	S1820	LDNKSPEPDTR	S5	0.952	0.364
Ice1	Little elongation complex subunit 1	E9Q286	S676	ENDPENSLSTLSPK	S12	1.072	0.544
Idh1	Isocitrate dehydrogenase [NADP] cytoplasmic	O88844	T77	CATITPDEK	T5	1.034	0.673
Ifi203	Interferon-activable protein 203	E9QAN9	S110	GQDELSSSESLFNNK	S6	0.931	0.644
Ifi203	Interferon-activable protein 203	E9QAN9	T177	SPPTPSSSSSNK	T4	1.131	0.184
Ifi204	Interferon-activable protein 204	P0DOV2	S190	CQSPILHSSSSASSNIPSAK	S3	1.125	0.789
Ifi204	Interferon-activable protein 204	P0DOV2	T106	NGQEAGPATPTSTTSHMLASER	T9	1.074	0.087
Ifi204	Interferon-activable protein 204	P0DOV2	T106	NGQEAGPATPTSTTSHMLASER	T9	1.020	0.662
Ifi204	Interferon-activable protein 204	P0DOV2	T106,T/S	NGQEAGPATPTSTTSHMLASER	T9,T/S	0.978	0.693
Ifi207	Interferon-activated gene 207	E9Q3L4	T106	NGQEAGPATPTSTTSHMLTSER	T9	1.100	0.021
Ifi207	Interferon-activated gene 207	E9Q3L4	T106	NGQEAGPATPTSTTSHMLTSER	T9	1.000	0.996
Ifih1	Interferon-induced helicase C domain-containing protein 1	Q8R5F7	S289	DSGTMGSDSDESVIQTK	S7	0.996	0.905
Ifih1	Interferon-induced helicase C domain-containing protein 1	Q8R5F7	S289	DSGTMGSDSDESVIQTK	S7	1.006	0.950
Ifih1	Interferon-induced helicase C domain-containing protein 1	Q8R5F7	S289,S291	DSGTMGSDSDESVIQTK	S7,S9	0.953	0.119
Ifit2	Ifit2 protein	Q6GTM0	Ambiguous	GVDSANQVPSASLDEAGAEY	S/Y	1.150	0.249
Ifnar1	Interferon alpha/beta receptor 1	P33896	S579	YLQSPALR	S4	1.043	0.520
Ifnar2	Interferon alpha/beta receptor 2	O35664	Ambiguous	VLHDEDASETLSLEEDTILLDEGPQR	S/T	1.063	0.701
Ifngr1	Interferon gamma receptor 1	P15261	S402	NGSDSGLVGSGSSISDLESLPNNNSETK	S3	1.011	0.851
Ifngr1	Interferon gamma receptor 1	P15261	T373	ALEAGGSTSAMTPDSPPTPTQR	T18	1.008	0.765
Ifngr1	Interferon gamma receptor 1	P15261	T373	ALEAGGSTSAMTPDSPPTPTQR	T18	1.079	0.312
Ifngr1	Interferon gamma receptor 1	P15261	S370,T373	ALEAGGSTSAMTPDSPPTPTQRR	S15,T18	0.990	0.818
Ifngr1	Interferon gamma receptor 1	P15261	S370,T373	ALEAGGSTSAMTPDSPPTPTQR	S15,T18	1.014	0.239
Ifngr1	Interferon gamma receptor 1	P15261	S370,T373	ALEAGGSTSAMTPDSPPTPTQRR	S15,T18	1.105	0.412
Ifngr1	Interferon gamma receptor 1	P15261	S402,S/T	NGSDSGLVGSGSSISDLESLPNNNSETK	S3,S/T	1.071	0.706
Ifngr1	Interferon gamma receptor 1	P15261	T367,T373	ALEAGGSTSAMTPDSPPTPTQR	T12,T18	1.025	0.192
Igf1r	Insulin-like growth factor 1 receptor	Q60751	T1372	ALPLPQSSTC	T9	1.025	0.859
Igf1r	Insulin-like growth factor 1 receptor	Q60751; P15208	Y1167;Y1179	DIYETDYYR	Y7	1.028	0.791
Igf2bp1	Insulin-like growth factor 2 mRNA-binding protein 1	O88477	S181	QGSPVAAGAPAK	S3	0.786	0.004
Igf2bp2	Insulin-like growth factor 2 mRNA-binding protein 2	Q5SF07	S164	ISYIPDEEVSSPSPPHR	S13	1.030	0.239
Igf2bp2	Insulin-like growth factor 2 mRNA-binding protein 2	Q5SF07	S162,S164	ISYIPDEEVSSPSPPHR	S11,S13	1.021	0.700
Igf2bp3	Insulin-like growth factor 2 mRNA-binding protein 3	Q9CPN8	S165	VAYIPDETAAQQNPSPQLR	S15	0.945	0.699
Igf2bp3	Insulin-like growth factor 2 mRNA-binding protein 3	Q9CPN8	S184	QASPGSVSK	S3	1.047	0.631
Igf2bp3	Insulin-like growth factor 2 mRNA-binding protein 3	Q9CPN8	S184,S	GSSRQASPGSVSK	S7,S	0.936	0.520
Igf2r	Cation-independent mannose-6-phosphate receptor	Q07113	S2337	RSSGVSYK	S3	0.959	0.394
Igf2r	Cation-independent mannose-6-phosphate receptor	Q07113	S2401	AEALSSLHGDDQDSEDEVLTVPEVK	S14	0.984	0.858
Ighmbp2	DNA-binding protein SMUBP-2	A0A0B4J1E3	S800	RSPASSGSVAPQPSSPPSPAQAEPEPR	S18	1.077	0.569
Ighmbp2	DNA-binding protein SMUBP-2	A0A0B4J1E3	Ambiguous	SPASSGSVAPQPSSPPSPAQAEPEPR	S	1.145	0.368
Ikbkb	Inhibitor of nuclear factor kappa-B kinase subunit beta	O88351	S672	GPVSGSPDSMNVSR	S6	0.985	0.778
Ikbkb	Inhibitor of nuclear factor kappa-B kinase subunit beta	O88351	S672	VRGPVSGSPDSMNVSR	S8	1.157	0.069
Ikbke	Inhibitor of nuclear factor kappa-B kinase subunit epsilon	Q9R0T8	S665	ASEQGAEVSPQPMAPHPGPDPK	S9	0.999	0.943
Ikbke	Inhibitor of nuclear factor kappa-B kinase subunit epsilon	Q9R0T8	S713	LHRVPSAPDV	S6	1.194	0.233
Ikbkg	Inhibitor of kappaB kinase gamma	Q7TSS3	S398	SPPEEPPDFCCPK	S1	0.983	0.771
Ikbkg	Inhibitor of kappaB kinase gamma	Q7TSS3	S398	RSPPEEPPDFCCPK	S2	1.033	0.333
Ikzf1	DNA-binding protein Ikaros	G5E8H3	S441	AASENSQDAFR	S6	0.926	0.582
Ikzf1	DNA-binding protein Ikaros	G5E8H3	Ambiguous	VETQSDEENGR	T/S	1.018	0.664
Ikzf1	DNA-binding protein Ikaros	G5E8H3	Ambiguous	GMASNVKVETQSDEENGR	T/S	0.947	0.501
Il10rb	Interleukin 10 receptor 2	Q8VHM7	S336	ELESKDEAPSPPHDDPK	S10	1.012	0.883
Il16	Pro-interleukin-16	O54824	S917	QTEVESMSTTFPNSSEVRDPGLPESPPPGQRPSTK	S25	0.937	0.195
Il16	Pro-interleukin-16	O54824	S917	QTEVESMSTTFPNSSEVRDPGLPESPPPGQRPSTK	S25	0.943	0.556
Il16	Pro-interleukin-16	O54824	S917	DPGLPESPPPGQRPSTK	S7	1.031	0.655
Il16	Pro-interleukin-16	O54824	S930	ALSPDPLLR	S3	1.047	0.311
Il16	Pro-interleukin-16	O54824	S959	ARSFPLTR	S3	0.905	0.181
Il16	Pro-interleukin-16	O54824	Ambiguous	ISSFENFGSSQLPDR	S	1.104	0.371
Il1rap	Interleukin-1 receptor accessory protein	Q61730	S566	QGLSYSSLK	S7	0.981	0.662
Il6st	Interleukin-6 receptor subunit beta	Q00560	S837	SNQVLSGNEEDFVR	S6	0.738	0.574
Il7r	Interleukin-7 receptor subunit alpha	P16872	S356	SPETSVSPPETVR	S7	0.965	0.601
Il7r	Interleukin-7 receptor subunit alpha	P16872	S356	SPETSVSPPETVRR	S7	1.043	0.734
Il7r	Interleukin-7 receptor subunit alpha	P16872	S375	NLSTCNAPPLLSSR	S3	0.993	0.948
Il7r	Interleukin-7 receptor subunit alpha	P16872	Ambiguous	NLSTCNAPPLLSSRSPDYR	S/T/Y	0.905	0.304
Il7r	Interleukin-7 receptor subunit alpha	P16872	Ambiguous	AAVHSANRSPETSVSPPETVR	S/T	1.051	0.729
Il7r	Interleukin-7 receptor subunit alpha	P16872	Ambiguous	NLSTCNAPPLLSSRSPDYR	S/T/Y	1.095	0.291
Ilf3	Interleukin enhancer-binding factor 3	Q9Z1X4	T67	GNSELSEAENMDTPPDDESK	T13	1.274	0.044
Ilf3	Interleukin enhancer-binding factor 3	Q9Z1X4	T67	GNSELSEAENMDTPPDDESKEGAGEQK	T13	1.034	0.111
Ilf3	Interleukin enhancer-binding factor 3	Q9Z1X4	Ambiguous	GYGHGQGSYSSYSNSYNSPGGGGGSDYSYDSK	S/Y	0.826	0.033
Ilf3	Interleukin enhancer-binding factor 3	Q9Z1X4	Ambiguous	QGGYSSQSNYSSPGSSQSYSGPASSYQSSQGGYSR	S/Y	1.039	0.563
Immt	MICOS complex subunit Mic60	Q8CAQ8; A0A0U1RP81	S34;S34	YSTSSSSGLTAGK	S2	1.022	0.792
Immt	MICOS complex subunit MIC60	A0A0U1RP81	Ambiguous	STSETTALAK	T/S	1.114	0.134
Impa1	Inositol monophosphatase 1	O55023	Ambiguous	SSPADLVTVTDQK	S/T	0.980	0.717
Impact	Protein IMPACT	O55091	S137	TEEVEVESEEDPILEHPPENPVK	S8	0.990	0.862
Impact	Protein IMPACT	O55091	S137	KTEEVEVESEEDPILEHPPENPVK	S9	0.882	0.011
Impdh2	Inosine-5'-monophosphate dehydrogenase 2	P24547	S416	GMGSLDAMDK	S4	1.009	0.889
Incenp	Inner centromere protein	Q9WU62	S284	SLISQDSQVPLASK	S1	1.133	0.239
Incenp	Inner centromere protein	Q9WU62	S72	RVSNIQDENRDPVR	S3	1.110	0.307
Inf2	Inverted formin-2	E9QLA5	S1170	TEADSTSEGPEDEAQR	S5	1.012	0.763
Inf2	Inverted formin-2	E9QLA5	S1249	RPSRNQEEFVPDSDDIK	S3	0.961	0.141
Inf2	Inverted formin-2	E9QLA5	T1201	TGPGEDEDGEDTAPESALDTSLDR	T12	1.064	0.531
Inf2	Inverted formin-2	E9QLA5	T1228	SFSEDAVTDSSGSGTLPR	T15	0.873	0.138
Inf2	Inverted formin-2	E9QLA5	Ambiguous	TEADSTSEGPEDEAQRGQSTHLPR	T/S	0.966	0.870
Ing4	Inhibitor of growth protein 4	Q8C0D7	S123	QIESSDYDSSSSK	S9	0.966	0.593
Ing5	Inhibitor of growth protein 5	Q9D8Y8	S118	MDGSDFESTGAR	S4	1.027	0.542
Ing5	Inhibitor of growth protein 5	Q9D8Y8	S118	MDGSDFESTGAR	S4	0.987	0.894
Ing5	Inhibitor of growth protein 5	Q9D8Y8	S118	DRMDGSDFESTGAR	S6	0.982	0.871
Ing5	Inhibitor of growth protein 5	Q9D8Y8	S118	DRMDGSDFESTGAR	S6	1.005	0.947
Ing5	Inhibitor of growth protein 5	Q9D8Y8	S148	RTSEEDTPK	S3	0.934	0.389
Ino80b	INO80 complex subunit B	Q99PT3	S370	LGGPEGPGSPLLAT	S9	1.013	0.902
Ino80c	INO80 complex subunit C	F8WIA0	S26	RPASPSHNSSGGGYGASK	S4	1.216	0.082
Ino80d	INO80 complex subunit D	E9Q9Q0	S132	MPNGLDSMSLSPPGAR	S11	1.127	0.200
Ino80e	INO80 complex subunit E (Fragment)	A0A0U1RP99	T186	LSDTPAPK	T4	1.071	0.111
Ino80e	INO80 complex subunit E (Fragment)	A0A0U1RPF6	Ambiguous	SVEEELELGEAEGRESS	S	0.938	0.005
Inpp4a	Type I inositol 3,4-bisphosphate 4-phosphatase	D3Z230	S487	ASPTSTEEEQVMLR	S2	0.922	0.703
Inpp5d	Phosphatidylinositol 3,4,5-trisphosphate 5-phosphatase 1	Q9ES52	S1090	IRSFTCSSSAEGR	S3	0.955	0.645
Inpp5d	Phosphatidylinositol 3,4,5-trisphosphate 5-phosphatase 1	Q9ES52	S310	SESLGIPQK	S3	0.945	0.583
Inpp5d	Phosphatidylinositol 3,4,5-trisphosphate 5-phosphatase 1	Q9ES52	S775	SQEGENEEGSEGELVVR	S10	1.074	0.095
Inpp5d	Phosphatidylinositol 3,4,5-trisphosphate 5-phosphatase 1	Q9ES52	S935	STLSPDQQLTAWSYDQLPK	S4	0.800	0.353
Inpp5d	Phosphatidylinositol 3,4,5-trisphosphate 5-phosphatase 1	Q9ES52	S972	DSSLGPGRGEGPPTPPSQPPLSPK	S22	0.977	0.716
Inpp5d	Phosphatidylinositol 3,4,5-trisphosphate 5-phosphatase 1	Q9ES52	T1092	SFTCSSSAEGR	T3	1.061	0.097
Inpp5d	Phosphatidylinositol 3,4,5-trisphosphate 5-phosphatase 1	Q9ES52	T888	NLTSHDPMR	T3	1.292	0.035
Inpp5d	Phosphatidylinositol 3,4,5-trisphosphate 5-phosphatase 1	Q9ES52	T964	GEGPPTPPSQPPLSPK	T6	1.011	0.759
Inpp5d	Phosphatidylinositol 3,4,5-trisphosphate 5-phosphatase 1	Q9ES52	Y376	EYVFADSK	Y2	0.853	0.399
Inpp5d	Phosphatidylinositol 3,4,5-trisphosphate 5-phosphatase 1	Q9ES52	T964,S972	DSSLGPGRGEGPPTPPSQPPLSPK	T14,S22	1.029	0.479
Inpp5d	Phosphatidylinositol 3,4,5-trisphosphate 5-phosphatase 1	Q9ES52	T964,S972	GEGPPTPPSQPPLSPK	T6,S14	1.103	0.238
Inpp5d	Phosphatidylinositol 3,4,5-trisphosphate 5-phosphatase 1	Q9ES52	T964,S972,S	DSSLGPGRGEGPPTPPSQPPLSPK	T14,S22,S	1.250	0.146
Inpp5d	Phosphatidylinositol 3,4,5-trisphosphate 5-phosphatase 1	Q9ES52	Ambiguous	ASASSQAPVPVK	S	0.859	0.066
Inpp5e	72 kDa inositol polyphosphate 5-phosphatase	Q9JII1	S103	FRGSQEDLTVQNGASPCR	S4	1.028	0.711
Inpp5e	72 kDa inositol polyphosphate 5-phosphatase	Q9JII1	S89	TLSLDDK	S3	1.061	0.258
Inpp5f	Phosphatidylinositide phosphatase SAC2	Q8CDA1	S1103	VQKSPAEPEAVNEIQQNELK	S4	1.100	0.188
Inpp5f	Phosphatidylinositide phosphatase SAC2	Q8CDA1	S123	IIPSPDDSK	S4	1.089	0.283

Inpp5f	Phosphatidylinositide phosphatase SAC2	Q8CDA1	S830	SDSSLETMENPGVMGNK	S4	1.035	0.159
Inpp5f	Phosphatidylinositide phosphatase SAC2	Q8CDA1	S830	SDSSLETMENPGVMGNK	S4	0.944	0.411
Inpp5f	Phosphatidylinositide phosphatase SAC2	Q8CDA1	S910	SQSASSIDVSTHAPSEAAAGPGSELGK	S5	0.961	0.808
Inpp5f	Phosphatidylinositide phosphatase SAC2	Q8CDA1	S943	SPSADSIHTR	S3	1.116	0.454
Inpp5f	Phosphatidylinositide phosphatase SAC2	Q8CDA1	S943	KSPSADSIHTR	S4	1.085	0.502
Inpp5f	Phosphatidylinositide phosphatase SAC2	Q8CDA1	S908,S/T	SQSASSIDVSTHAPSEAAAGPGSELGK	S3,S/T	1.160	0.361
Inpp5j	Phosphatidylinositol 4,5-bisphosphate 5-phosphatase A	P59644	S69	AAVSPPSERPR	S4	1.048	0.680
Inpp5j	Phosphatidylinositol 4,5-bisphosphate 5-phosphatase A	P59644	Ambiguous	AAGPATSPPR	T/S	1.019	0.505
Inppl1	Phosphatidylinositol 3,4,5-trisphosphate 5-phosphatase 2	Q6P549	S1166	GPSDYGRPLSFPPPR	S10	1.007	0.927
Inppl1	Phosphatidylinositol 3,4,5-trisphosphate 5-phosphatase 2	Q6P549	S132	EPDPPDDRDASDVEDEKPPLPPR	S11	1.146	0.004
Inppl1	Phosphatidylinositol 3,4,5-trisphosphate 5-phosphatase 2	Q6P549	S132	DASDVEDEKPPLPPR	S3	0.948	0.579
Inppl1	Phosphatidylinositol 3,4,5-trisphosphate 5-phosphatase 2	Q6P549	S241	VFDQQSSPMVTR	S7	0.994	0.881
Inppl1	Phosphatidylinositol 3,4,5-trisphosphate 5-phosphatase 2	Q6P549	S241	VFDQQSSPMVTR	S7	0.873	0.037
Inppl1	Phosphatidylinositol 3,4,5-trisphosphate 5-phosphatase 2	Q6P549	S301	ALQDMSSTAPPAPLQPSIR	S17	0.939	0.278
Inppl1	Phosphatidylinositol 3,4,5-trisphosphate 5-phosphatase 2	Q6P549	S301	ALQDMSSTAPPAPLQPSIR	S17	0.817	0.327
Inppl1	Phosphatidylinositol 3,4,5-trisphosphate 5-phosphatase 2	Q6P549	S353	QRDSQEDWTTFTHDR	S4	1.024	0.453
Inppl1	Phosphatidylinositol 3,4,5-trisphosphate 5-phosphatase 2	Q6P549; Q9ES52	T538;T520	TGIANTLGNK	T6	1.082	0.553
Inppl1	Phosphatidylinositol 3,4,5-trisphosphate 5-phosphatase 2	Q6P549	Y1136	TLSEVDYAPGPGR	Y7	0.976	0.740
Inppl1	Phosphatidylinositol 3,4,5-trisphosphate 5-phosphatase 2	Q6P549	Y190	GSYGLDLEAVR	Y3	0.936	0.656
Insr	Insulin receptor	P15208	S1340	EGGSSLSIK	S7	1.065	0.451
Ints1	Integrator complex subunit 1	A0A0G2JH17	Ambiguous	DSTEAPKPESSPEPPPGQGR	S/T	0.942	0.264
Ints10	Integrator complex subunit 10	A0A0R4J0V1	S231	STQSENQHQGAQDTSDLMSPSKR	S19	0.934	0.760
Ints10	Integrator complex subunit 10	A0A0R4J0V1	S382	TMSSDDEECSAK	S4	1.041	0.522
Ints10	Integrator complex subunit 10	A0A0R4J0V1	Ambiguous	EKTMSSDDEECSAK	S/T	1.048	0.518
Ints10	Integrator complex subunit 10	A0A0R4J0V1	Ambiguous	EKTMSSDDEECSAK	S/T	1.006	0.900
Ints10	Integrator complex subunit 10	A0A0R4J0V1	Ambiguous	TMSSDDEECSAK	S/T	1.002	0.957
Ints12	Integrator complex subunit 12	Q9D168	S377	SVSCDNVSK	S3	1.038	0.326
Ints12	Integrator complex subunit 12	Q9D168	Ambiguous	SSPITVQTSK	S/T	0.982	0.488
Ipo11	Importin-11	Q8K2V6	S343	NFEDSSPETLEAHK	S6	0.990	0.783
Ipo5	Importin-5	Q8BKC5	S827	QDEDYDEQVEESLQDEDDNDVYILTK	S12	1.180	0.389
Ipo5	Importin-5	Q8BKC5	S827	RQDEDYDEQVEESLQDEDDNDVYILTK	S13	0.858	0.565
Iqce	IQ domain-containing protein E	Q6PCQ0	S374	SPSNISVQK	S3	0.973	0.762
Iqce	IQ domain-containing protein E	Q6PCQ0	S387	GDQSPEDLPK	S4	0.961	0.557
Iqce	IQ domain-containing protein E	Q6PCQ0	S661	ETVSLTPSGSASPPSLR	S12	0.980	0.757
Iqce	IQ domain-containing protein E	Q6PCQ0	S668	ASPGVIR	S2	1.109	0.208
Iqgap1	Ras GTPase-activating-like protein IQGAP1	Q9JKF1	S1511	LQQTYSALNSK	S6	0.905	0.160
Iqgap1	Ras GTPase-activating-like protein IQGAP1	Q9JKF1	Ambiguous	QLSSSVTGLTNIEEENCQR	S/T	1.139	0.595
Iqgap2	Ras GTPase-activating-like protein IQGAP2	Q3UQ44	S16	YGSIVDDER	S3	1.391	0.019
Iqgap2	Ras GTPase-activating-like protein IQGAP2	Q3UQ44	S16	YGSIVDDERLSAEEMDER	S3	1.348	0.082
Iqgap2	Ras GTPase-activating-like protein IQGAP2	Q3UQ44	T1349	AVVDSRTPEEGKQSQAVIEDAR	T7	1.073	0.086
Iqgap2	Ras GTPase-activating-like protein IQGAP2	Q3UQ44	Ambiguous	AVVDSRTPEEGK	S/T	1.066	0.452
Iqsec1	IQ motif and SEC7 domain-containing protein 1	E9PUA3	S165	MQFSFEGPEK	S4	1.162	0.174
Iqsec1	IQ motif and SEC7 domain-containing protein 1	E9PUA3	S237	SLAESIDDALNCR	S5	0.978	0.833
Iqsec1	IQ motif and SEC7 domain-containing protein 1	E9PUA3	S246	SLHSEEVPASDTAR	S1	1.147	0.380
Iqsec1	IQ motif and SEC7 domain-containing protein 1	E9PUA3	S348	STPSLERPEPR	S4	1.117	0.075
Iqsec1	IQ motif and SEC7 domain-containing protein 1	E9PUA3	S401	SLGGQQGSPK	S8	0.960	0.576
Iqsec1	IQ motif and SEC7 domain-containing protein 1	E9PUA3	S450	QSKSESDYSDGDNDSINSTSNSNDTINCSSESSSR	S9	1.038	0.859
Iqsec1	IQ motif and SEC7 domain-containing protein 1	E9PUA3	S496	NSWDSPAFSNDVIR	S2	1.000	1.000
Iqsec1	IQ motif and SEC7 domain-containing protein 1	E9PUA3	S496	ETRNSWDSPAFSNDVIR	S5	1.003	0.935
Iqsec1	IQ motif and SEC7 domain-containing protein 1	E9PUA3	S447,S450	QSKSESDYSDGDNDSINSTSNSNDTINCSSESSSR	S6,S9	0.992	0.974
Iqsec1	IQ motif and SEC7 domain-containing protein 1	E9PUA3	S924,S927	SSAGSLESNVEGSIISSPHMR	S2,S5	0.878	0.458
Iqsec1	IQ motif and SEC7 domain-containing protein 1	E9PUA3	Ambiguous	SLSESYELSSDLQDK	S/Y	1.223	0.276
Iqsec1	IQ motif and SEC7 domain-containing protein 1	E9PUA3	Ambiguous	RSSAGSLESNVEGSIISSPHMR	S	1.059	0.763
Iqsec2	IQ motif and SEC7 domain-containing protein 2	D3Z5I6	S1124	SSLEDTYGAGDGLKR	S2	0.999	0.978
Iqsec2	IQ motif and SEC7 domain-containing protein 2	D3Z5I6	S1143	GALSSSLR	S6	1.047	0.329
Iqsec2	IQ motif and SEC7 domain-containing protein 2	D3Z5I6	S627	QLVYEADGCSPHGTLK	S10	0.970	0.851
Iqsec2	IQ motif and SEC7 domain-containing protein 2	D3Z5I6	S741	ETRHSWDSPAFNNDVVQR	S5	0.984	0.542
Iqsec2	IQ motif and SEC7 domain-containing protein 2	D3Z5I6	Ambiguous	SSLEDTYGAGDGLK	S/T/Y	0.968	0.670
Iqsec2	IQ motif and SEC7 domain-containing protein 2	D3Z5I6	Ambiguous	GALSSSLRDLSDAGVCY	S/Y	1.168	0.514
Iqsec2	IQ motif and SEC7 domain-containing protein 2	D3Z5I6	Ambiguous	TVSVEGDAPGSDLSTAVDSPGSQPPYR	T/S/Y	1.227	0.298
Iqsec3	IQ motif and SEC7 domain-containing protein 3	Q3TES0	S1083,S/T	GAPAPSPPTSPPPLPPDPQPSPLR	S21,S/T	1.241	0.192
Irak1	Interleukin-1 receptor-associated kinase 1	B1AUW6	S186	QSSPESPVSGLQR	S6	0.924	0.148
Irak1	Interleukin-1 receptor-associated kinase 1	Q62406	S185	SSPESPVSGLQR	S5	1.087	0.323
Irak2	Interleukin-1 receptor-associated kinase-like 2	Q8CFA1	S168	TDAPDSPQSK	S6	1.082	0.049
Irak4	Interleukin-1 receptor-associated kinase 4	Q8R4K2	Ambiguous	LEHSCEPPDSSSPDNR	S	0.978	0.855
Irf2bp1	Interferon regulatory factor 2-binding protein 1	Q8R3Y8	S371	APSRNLAPTPR	S3	1.061	0.444
Irf2bp1	Interferon regulatory factor 2-binding protein 1	Q8R3Y8	S371	SFREPAPAEALPQQYPEPAPAALCGPPPRAPSR	S32	1.025	0.897
Irf2bp1	Interferon regulatory factor 2-binding protein 1	Q8R3Y8	S384	KASPEPEGETAGK	S3	0.974	0.520
Irf2bp1	Interferon regulatory factor 2-binding protein 1	Q8R3Y8	S436	NVAEALGHSPKDPGGGGGSVR	S9	1.021	0.715
Irf2bp1	Interferon regulatory factor 2-binding protein 1	Q8R3Y8	S453	AGGASPAASSTTQPPAQHR	S5	0.979	0.707
Irf2bp1	Interferon regulatory factor 2-binding protein 1	Q8R3Y8	S66	SPGPPALK	S1	1.064	0.378
Irf2bp2	Interferon regulatory factor 2-binding protein 2	E9Q1P8	S169	LEEPPELNRQSPNPR	S11	0.969	0.175
Irf2bp2	Interferon regulatory factor 2-binding protein 2	E9Q1P8	S169	LEEPPELNRQSPNPR	S11	1.019	0.902
Irf2bp2	Interferon regulatory factor 2-binding protein 2	E9Q1P8	S226	RPASVSSAAAEHEAREPSK	S4	0.720	0.169
Irf2bp2	Interferon regulatory factor 2-binding protein 2	E9Q1P8	S226	RPASVSSAAAEHEAR	S4	0.949	0.653
Irf2bp2	Interferon regulatory factor 2-binding protein 2	E9Q1P8	S250	SPADSLSSAAGASELSAEGAGK	S1	1.078	0.289
Irf2bp2	Interferon regulatory factor 2-binding protein 2	E9Q1P8	S250	AQPAHRSPADSLSSAAGASELSAEGAGK	S7	0.984	0.650
Irf2bp2	Interferon regulatory factor 2-binding protein 2	E9Q1P8	S343	KPSPEPEGEVGPPK	S3	0.995	0.891
Irf2bp2	Interferon regulatory factor 2-binding protein 2	E9Q1P8	S343	RKPSPEPEGEVGPPK	S4	0.981	0.699
Irf2bp2	Interferon regulatory factor 2-binding protein 2	E9Q1P8	S439	NSSSPPSPSSMNQR	S3	0.994	0.954
Irf2bp2	Interferon regulatory factor 2-binding protein 2	E9Q1P8	S440	NSSSPPSPSSMNQR	S4	1.042	0.553
Irf2bp2	Interferon regulatory factor 2-binding protein 2	E9Q1P8	S438,S440	RNSSSPPSPSSMNQR	S3,S5	0.895	0.207
Irf2bp2	Interferon regulatory factor 2-binding protein 2	E9Q1P8	S439,S443	NSSSPPSPSSMNQR	S3,S7	1.024	0.678
Irf2bpl	Interferon regulatory factor 2-binding protein-like	Q8K3X4	S313	RPGSVSSTDQER	S/T	1.054	0.411
Irf2bpl	Interferon regulatory factor 2-binding protein-like	Q8K3X4	S498	APSAPPGTGALPPAAPTGR	S3	1.011	0.662
Irf2bpl	Interferon regulatory factor 2-binding protein-like	Q8K3X4	S526	ASPEPPDSAESALK	S2	1.119	0.034
Irf2bpl	Interferon regulatory factor 2-binding protein-like	Q8K3X4	S526	KASPEPPDSAESALK	S3	0.947	0.249
Irf2bpl	Interferon regulatory factor 2-binding protein-like	Q8K3X4	S526	RKASPEPPDSAESALK	S4	1.067	0.488
Irf2bpl	Interferon regulatory factor 2-binding protein-like	Q8K3X4	S638	NSSSPVSPASVPGQR	S4	0.968	0.614
Irf2bpl	Interferon regulatory factor 2-binding protein-like	Q8K3X4	S638	RNSSSPVSPASVPGQR	S5	0.981	0.481
Irf2bpl	Interferon regulatory factor 2-binding protein-like	Q8K3X4	S69	SPGPPPPVGVK	S1	0.974	0.564
Irf2bpl	Interferon regulatory factor 2-binding protein-like	Q8K3X4	S69	AHGCFQDGRSPGPPPPVGVK	S10	1.064	0.687
Irf2bpl	Interferon regulatory factor 2-binding protein-like	Q8K3X4	S636,S638	RNSSSPVSPASVPGQR	S3,S5	1.024	0.664
Irf2bpl	Interferon regulatory factor 2-binding protein-like	Q8K3X4	Ambiguous	NSSSPVSPASVPGQR	S	1.090	0.285
Irf3	Interferon regulatory factor 3	P70671	S135	SSLPHSQENLPK	S6	1.015	0.492
Irf3	Interferon regulatory factor 3	P70671	S131,S135	SSLPHSQENLPK	S2,S6	1.021	0.875
Irf3	Interferon regulatory factor 3	P70671	Ambiguous	EGGASSLK	S	1.035	0.615
Irf8	Interferon regulatory factor 8	P23611	S164	SPSPPEACR	S3	1.004	0.928
Irf9	Interferon regulatory factor 9	E9PZJ2	S205	SISCVSPEREENMENGR	S6	0.956	0.753
Irf9	Interferon regulatory factor 9	E9PZJ2	S205	SISCVSPEREENMENGR	S6	0.948	0.121
Irgm1	Immunity-related GTPase family M protein 1	Q60766	S202	DLSTSVLSEVR	S8	1.109	0.347
Irs2	Insulin receptor substrate 2	P81122	S1089	VASPTSGLK	S3	0.933	0.109
Irs2	Insulin receptor substrate 2	P81122	S1089	VASPTSGLKR	S3	0.985	0.701
Irs2	Insulin receptor substrate 2	P81122	S1165	HNSASVENVSLR	S5	0.994	0.944
Irs2	Insulin receptor substrate 2	P81122	S388	TASEGDGGAAGGAGTAGGRPMSVAGSPLSPGPVR	S29	0.930	0.643
Irs2	Insulin receptor substrate 2	P81122	S388	TASEGDGGAAGGAGTAGGRPMSVAGSPLSPGPVR	S29	0.934	0.280
Irs2	Insulin receptor substrate 2	P81122	S573	TYSLTTPAR	S3	1.017	0.552
Irs2	Insulin receptor substrate 2	P81122	S590	QVPQPSSASLDEYTLMR	S9	1.064	0.427
Irs2	Insulin receptor substrate 2	P81122	S604	ATFSGSSGR	S6	0.995	0.885
Irs2	Insulin receptor substrate 2	P81122	S675	SDDYMPMSPTSVSAPK	S8	1.126	0.051
Irs2	Insulin receptor substrate 2	P81122	S728	ASSPAESSPEDSGYMR	S8	1.211	0.303
Irs2	Insulin receptor substrate 2	P81122	S796	GIPGHCYSSLPR	S9	0.995	0.972
Irs2	Insulin receptor substrate 2	P81122	T347	SRTDSLAATPPAAK	T9	1.058	0.537
Irs2	Insulin receptor substrate 2	P81122	T517	SNTPESIAETPPAR	T3	1.012	0.544
Irs2	Insulin receptor substrate 2	P81122	S362,S/T	TASEGDGGAAGGAGTAGGRPMSVAGSPLSPGPVR	S3,S/T	0.952	0.219
Irs2	Insulin receptor substrate 2	P81122	S362,S388	TASEGDGGAAGGAGTAGGRPMSVAGSPLSPGPVR	S3,S29	1.197	0.271
Irs2	Insulin receptor substrate 2	P81122	Ambiguous	SDDYMPMSPTSVSAPK	S/T/Y	1.127	0.400
Irs2	Insulin receptor substrate 2	P81122	Ambiguous	QVPQPSSASLDEYTLMR	T/S/Y	1.261	0.174
Iscu	Iron-sulfur cluster assembly enzyme ISCU, mitochondrial	D3Z7W0	S15	AASALLLR	S3	1.003	0.923
Iscu	Iron-sulfur cluster assembly enzyme ISCU, mitochondrial	D3Z7W0	S15	RAASALLLR	S4	1.097	0.442
Iscu	Iron-sulfur cluster assembly enzyme ISCU, mitochondrial	D3Z7W0	S30	ELSAPAR	S3	1.026	0.873
Itch	E3 ubiquitin-protein ligase Itchy	Q8C863	S176	DDTRVSTNGSEDPEVAASGENKR	S6	0.908	0.154
Itfg2	KICSTOR complex protein ITFG2	Q91WI7	T217	DTGSPPASEEATGDSR	T2	0.831	0.549
Itga4	Integrin alpha-4	Q00651	S1028	RDSWSYVNSK	S3	0.995	0.914
Itga6	Integrin alpha-6 (Fragment)	F6VSK8	S658	AEIHTQPSDKER	S8	1.102	0.143
Itgal	Integrin alpha-L	P24063	T1136	MEADGGVPNGSPPEDTDPLAVPGEETK	T16	1.127	0.072
Itgav	Integrin alpha-V	P43406	S1042	EQLQPHENGEGNSET	S13	1.012	0.876
Itgav	Integrin alpha-V	P43406	Ambiguous	VRPPQEEQEREQLQPHENGEGNSET	T/S	0.936	0.411
Itgax	Integrin alpha-X	Q9QXH4	S1159	EMLEEANGQFVSDGTPTPQVAQ	S12	1.156	0.012
Itgax	Integrin alpha-X	Q9QXH4	Ambiguous	EMLEEANGQFVSDGTPTPQVAQ	T/S	0.880	0.624
Itgb1	Integrin beta-1	P09055	T777	WDTGENPIYK	T3	0.984	0.856
Itgb1bp1	Integrin beta-1-binding protein 1	O35671	S41	SSTVASLDTDSTK	S6	1.016	0.714
Itgb1bp1	Integrin beta-1-binding protein 1	O35671	S37,S41	SSTVASLDTDSTK	S2,S6	1.036	0.612
Itgb3bp	Centromere protein R	Q9CQ82	Ambiguous	SNLSSPVRQESTVK	S/T	1.060	0.287
Itgb5	Integrin beta	Q6PE70	S770	YEMASNPLYR	S5	1.051	0.798
Itgb5	Integrin beta	Q6PE70	S770	YEMASNPLYR	S5	1.021	0.817
Itgb5	Integrin beta	Q6PE70	S770	ARYEMASNPLYR	S7	0.957	0.522
Itgb5	Integrin beta	Q6PE70	S770	ARYEMASNPLYR	S7	1.037	0.749

Itm2b	Integral membrane protein 2B	O89051	S104	YIKDDVILNEPSADAPAAR	S12	1.076	0.373
Itm2b	Integral membrane protein 2B	O89051	S23	SSEEALIVPPDAVAVDCK	S2	0.859	0.407
Itpkb	Kinase	B2RXC2	S125	ILSPPGPEEAQR	S3	1.130	0.043
Itpkb	Kinase	B2RXC2	S179	ARSPSPCPFR	S3	0.987	0.870
Itpkb	Kinase	B2RXC2	S200	VLAPCSPSEER	S6	1.009	0.760
Itpkb	Kinase	B2RXC2	S200	VLAPCSPSEERR	S6	0.963	0.074
Itpkb	Kinase	B2RXC2	S209	SWGEQCTETPDTNSGRR	S1	1.063	0.182
Itpkb	Kinase	B2RXC2	S209	SWGEQCTETPDTNSGR	S1	1.039	0.445
Itpkb	Kinase	B2RXC2	S209	TKSWGEQCTETPDTNSGR	S3	1.159	0.092
Itpkb	Kinase	B2RXC2	S247	EGVAPLLGPASPTR	S11	0.985	0.699
Itpkb	Kinase	B2RXC2	S255	LGTQSPSTSVR	S5	0.984	0.649
Itpkb	Kinase	B2RXC2	S274	CGSPTPMETDKR	S3	0.934	0.584
Itpkb	Kinase	B2RXC2	S274	CGSPTPMETDKR	S3	1.048	0.530
Itpkb	Kinase	B2RXC2	S439	EGSPALGLLGGSQAAQPGAR	S3	1.231	0.373
Itpkb	Kinase	B2RXC2	S48	AALSPGSVFSPGR	S10	1.091	0.224
Itpkb	Kinase	B2RXC2	S179,S181	ARSPSPCPFR	S3,S5	1.043	0.285
Itpkb	Kinase	B2RXC2	S42,S48	AALSPGSVFSPGR	S4,S10	1.441	0.004
Itpkb	Kinase	B2RXC2	Ambiguous	TKSWGEQCTETPDTNSGRR	T/S	1.079	0.591
Itpkc	Inositol-trisphosphate 3-kinase C	Q7TS72	T330	LIITPETPEPEAQPVGPQSR	T7	0.908	0.713
Itpr1	Inositol 1,4,5-trisphosphate receptor type 1	P11881	S1588	RDSVLAASR	S3	0.974	0.785
Itpr2	Inositol 1,4,5-trisphosphate receptor type 2	Q9Z329	S1160	GGEEANEESNLLSPVQDGAK	S13	1.122	0.489
Itprid2	Protein ITPRID2	Q922B9	S281	EVADSPPPLTR	S5	0.951	0.117
Itprid2	Protein ITPRID2	Q922B9	S738	SQSLPTTLLSPVR	S3	1.218	0.055
Itprid2	Protein ITPRID2	Q922B9	S90	TPLGASLDEQSSGTPK	S6	0.834	0.344
Itprid2	Protein ITPRID2	Q922B9	S738,S745	SQSLPTTLLSPVR	S3,S10	1.102	0.493
Itprid2	Protein ITPRID2	Q922B9	T85,S90	DCRTPLGASLDEQSSGTPK	T4,S9	0.951	0.510
Itpripl2	Inositol 1,4,5-trisphosphate receptor-interacting protein-like 2	Q3UV16	S216	APPSPSGASTGQWHR	S4	0.948	0.368
Itsn1	Intersectin-1	E9Q3I5	S203	AQSFDVASAPPAAEWAVPQSSR	S3	1.027	0.825
Itsn1	Intersectin-1	E9Q3I5	S315	SGSGMSVISSSSVDQR	S3	1.052	0.400
Itsn1	Intersectin-1	E9Q3I5	S335	LPEEPSSEDEQQPEK	S7	1.005	0.823
Itsn1	Intersectin-1	E9Q3I5	S361	ENFERGSVELEK	S7	0.936	0.008
Itsn1	Intersectin-1	E9Q3I5	S558	QVQQNSLHR	S6	1.000	1.000
Itsn1	Intersectin-1	E9Q3I5	S623	QRSLEAAR	S3	0.907	0.179
Itsn1	Intersectin-1	E9Q3I5	S984	STSIDTGPTESPASLK	S11	0.971	0.496
Itsn1	Intersectin-1	E9Q3I5	S984	STSIDTGPTESPASLKR	S11	1.025	0.596
Itsn1	Intersectin-1	E9Q3I5	T895	SAFTPATATGSSPSPVLGQGEK	T7	0.993	0.912
Itsn1	Intersectin-1	E9Q3I5	S318,S	SGSGMSVISSSSVDQR	S6,S	0.786	0.454
Itsn1	Intersectin-1	E9Q3I5	S334,S335	LPEEPSSEDEQQPEK	S6,S7	0.897	0.115
Itsn1	Intersectin-1	E9Q3I5	S984,S/T	STSIDTGPTESPASLK	S11,S/T	0.907	0.440
Itsn1	Intersectin-1	E9Q3I5	Ambiguous	LPEEPSSEDEQQPEKK	S	1.040	0.193
Itsn1	Intersectin-1	E9Q3I5	Ambiguous	SGSGMSVISSSSVDQRLPEEPSSEDEQQPEK	S	0.773	0.280
Itsn1	Intersectin-1	E9Q3I5	Ambiguous	SGSGMSVISSSSVDQR	S	0.987	0.511
Itsn2	Intersectin-2	B2RR82	S870	TVSPGSVSPIHGQGQAVENLK	S8	1.007	0.898
Itsn2	Intersectin-2	B2RR82	Y949	GEPEALYAAVTK	Y7	0.900	0.007
Itsn2	Intersectin-2	B2RR82	S865,S870	TVSPGSVSPIHGQGQAVENLK	S3,S8	0.995	0.942
Ivns1abp	Influenza virus NS1A-binding protein homolog	Q920Q8	S326	NSPQSSPTSTPK	S6	1.027	0.613
Iws1	Protein IWS1 homolog	Q8C1D8	S183	ISDSESEELPKPR	S2	1.067	0.078
Iws1	Protein IWS1 homolog	Q8C1D8	S198;S418	VSDSESEDPPRPQASDSESEELPKPR	S4	1.020	0.331
Iws1	Protein IWS1 homolog	Q8C1D8	S261	VSDSESEDPQKGPASDSEAEDASR	S15	0.983	0.807
Iws1	Protein IWS1 homolog	Q8C1D8	S321	IDSDDDEEKEGDEEKVAK	S3	0.873	0.200
Iws1	Protein IWS1 homolog	Q8C1D8	S321	IDSDDDEEKEGDEEK	S3	0.979	0.862
Iws1	Protein IWS1 homolog	Q8C1D8	S321	QKIDSDDDEEKEGDEEK	S5	0.987	0.902
Iws1	Protein IWS1 homolog	Q8C1D8	S345	AAVLSDSEDDAGNASAK	S7	0.977	0.407
Iws1	Protein IWS1 homolog	Q8C1D8	S345	AAVLSDSEDDAGNASAKK	S7	1.018	0.845
Iws1	Protein IWS1 homolog	Q8C1D8	S366	VVCDADDSDSDVVSDK	S8	0.975	0.413
Iws1	Protein IWS1 homolog	Q8C1D8	S384	ETTVASDSEEEAGKEESSVK	S6	0.959	0.612
Iws1	Protein IWS1 homolog	Q8C1D8	S666	RLSSTGGQTPR	S/T	1.036	0.596
Iws1	Protein IWS1 homolog	Q8C1D8	T672	LSSTGGQTPR	T8	1.017	0.772
Iws1	Protein IWS1 homolog	Q8C1D8	S183,S185	ISDSESEELPKPR	S2,S4	1.024	0.578
Iws1	Protein IWS1 homolog	Q8C1D8	S343,S345	AAVLSDSEDDAGNASAK	S5,S7	1.016	0.597
Iws1	Protein IWS1 homolog	Q8C1D8	S343,S345	KAAVLSDSEDDAGNASAK	S6,S8	1.001	0.988
Iws1	Protein IWS1 homolog	Q8C1D8	S366,S368	VVCDADDSDSDVVSDK	S8,S10	1.021	0.688
Iws1	Protein IWS1 homolog	Q8C1D8	S384,S386	ETTVASDSEEEAGKEESSVK	S6,S8	0.991	0.852
Iws1	Protein IWS1 homolog	Q8C1D8	S666,S667	RLSSTGGQTPR	S3,S4	1.231	0.008
Jade1	Protein Jade-1	Q6ZPI0	S802	LRADSENDGYAPDGEMSDSESEASEKK	S17	0.970	0.781
Jade3	Protein Jade-3	A0A140T8R5	Ambiguous	ASYQETDGYCPDLEPSDSEAEGEGSK	S/Y/T	0.847	0.427
Jag1	Protein jagged-1	Q9QXX0	S1210	DLESAQSLNR	S7	1.051	0.756
Jak2	Tyrosine-protein kinase	G5E852	S523	TNGISDVQISPTLQR	S10	0.864	0.344
Jmjd1c	Probable JmjC domain-containing histone demethylation protein 2C	G3UZM1	S1052	VVPSSSSPK	S7	1.000	0.993
Jmjd1c	Probable JmjC domain-containing histone demethylation protein 2C	G3UZM1	S1177	SDCRSPTHLTVSSTNALR	S5	0.910	0.707
Jmjd1c	Probable JmjC domain-containing histone demethylation protein 2C	G3UZM1	S1220	ESSYSSLSPPTLTPVMPVNAGGK	S8	0.951	0.634
Jmjd1c	Probable JmjC domain-containing histone demethylation protein 2C	G3UZM1	S1681	LQNSNSAGVPR	S4	0.968	0.617
Jmjd1c	Probable JmjC domain-containing histone demethylation protein 2C	G3UZM1	S1970	TSVSLPESQQQNSPQK	S13	1.032	0.538
Jmjd1c	Probable JmjC domain-containing histone demethylation protein 2C	G3UZM1	S316	KGSDSSIPDEEK	S3	1.077	0.045
Jmjd1c	Probable JmjC domain-containing histone demethylation protein 2C	G3UZM1	S652	ITHSPDSVK	S4	0.936	0.440
Jmjd1c	Probable JmjC domain-containing histone demethylation protein 2C	G3UZM1	S316,S319	KGSDSSIPDEEK	S3,S6	0.951	0.554
Jmjd1c	Probable JmjC domain-containing histone demethylation protein 2C	G3UZM1	Ambiguous	SSEQATVADHNSNDSVLQECNVENQR	S/T	0.923	0.530
Jmjd1c	Probable JmjC domain-containing histone demethylation protein 2C	G3UZM1	Ambiguous	EIKEDGDQDASDSPNGSTSPPASQSNEQGSTLR	S/T	1.058	0.246
Jmjd1c	Probable JmjC domain-containing histone demethylation protein 2C	G3UZM1	Ambiguous	TDNASDASESSDAESSSK	S/T	0.925	0.384
Jmjd1c	Probable JmjC domain-containing histone demethylation protein 2C	G3UZM1	Ambiguous	LRTDNASDASESSDAESSSK	S/T	0.949	0.510
Jmjd1c	Probable JmjC domain-containing histone demethylation protein 2C	G3UZM1	Ambiguous	SSSSPEVVKPK	S	1.004	0.975
Jmy	Junction-mediating and -regulatory protein	A0A0R4J0V4	S130	GPESPLRSPAR	S8	1.240	0.151
Jmy	Junction-mediating and -regulatory protein	A0A0R4J0V4	S704	STASPVPCEEQCHSLPTVLQGQEK	S4	0.861	0.052
Jmy	Junction-mediating and -regulatory protein	A0A0R4J0V4	S969	EASPESEDEEEALPCTDWEN	S6	0.888	0.285
Jpt1	Jupiter microtubule associated homolog 1	P97825	S87	SNSSEASSGDFLDLK	S3	1.241	0.004
Jpt1	Jupiter microtubule associated homolog 1	P97825	S87,S	SNSSEASSGDFLDLK	S3,S	1.480	0.057
Jsrp1	Junctional sarcoplasmic reticulum protein 1	M0QW57	S228	GGSISEASGEESVPLGDR	S8	0.759	0.440
Jun	Transcription factor AP-1	P05627	S63	NSDLLTSPDVGLLK	S7	1.114	0.347
Jun	Transcription factor AP-1	P05627	S73	LASPELER	S3	1.120	0.037
Jun	Transcription factor AP-1	P05627	T234,T/S	EEPQTVPEMPGETPPLSPIDMESQER	T5,T/S	1.220	0.030
Junb	Transcription factor jun-B	P09450	T252	DATPPVSPINMEDQER	T3	0.958	0.689
Junb	Transcription factor jun-B	P09450	T252	SRDATPPVSPINMEDQER	T5	0.939	0.280
Junb	Transcription factor jun-B	P09450	T252,S256	DATPPVSPINMEDQER	T3,S7	1.171	0.355
Junb	Transcription factor jun-B	P09450	T252,S256	SRDATPPVSPINMEDQER	T5,S9	1.068	0.334
Junb	Transcription factor jun-B	P09450	T252,S256	SRDATPPVSPINMEDQER	T5,S9	0.993	0.916
Junb	Transcription factor jun-B	P09450	S248,T252,S256	SRDATPPVSPINMEDQER	S1,T5,S9	1.141	0.167
Kank2	KN motif and ankyrin repeat domain-containing protein 2	Q8BX02	S246	SRSELCLDLPEAPDDPAALETR	S1	1.490	0.006
Kank2	KN motif and ankyrin repeat domain-containing protein 2	Q8BX02	S358	AQSLEPYGTGLK	S3	1.424	0.056
Kank2	KN motif and ankyrin repeat domain-containing protein 2	Q8BX02	S570	VPAPEVASGPDPEEEIR	S8	1.046	0.694
Kank2	KN motif and ankyrin repeat domain-containing protein 2	Q8BX02	S174,S177	SSGLSTPVAPSAGHLAHVR	S2,S5	1.035	0.679
Kank3	KN motif and ankyrin repeat domain-containing protein 3	Q9Z1P7	Ambiguous	RSEGALQVLDPGSRTPDGEPR	S/T	1.003	0.956
Kansl1	KAT8 regulatory NSL complex subunit 1	A2A5Y4	S1076	EAEVAPTSPPVVPLK	S8	1.005	0.924
Kansl1	KAT8 regulatory NSL complex subunit 1	A2A5Y4	S249	LSPSTDSSSNLTNVK	S2	1.043	0.754
Kansl1	KAT8 regulatory NSL complex subunit 1	A2A5Y4	S822	QLSTSSDTSTPTSSGSQVAASTSQPVRR	S3	0.989	0.923
Kansl1	KAT8 regulatory NSL complex subunit 1	A2A5Y4	S985	SPISPELHSAPLTPVAR	S1	0.811	0.286
Kansl1	KAT8 regulatory NSL complex subunit 1	A2A5Y4	T1016	CSTPELGLDEQSVQPWER	T3	1.102	0.472
Kansl1	KAT8 regulatory NSL complex subunit 1	A2A5Y4	S822,T/S	QLSTSSDTSTPTSSGSQVAASTSQPVRR	S3,T/S	1.095	0.252
Kansl1	KAT8 regulatory NSL complex subunit 1	A2A5Y4	S822,S/T	QLSTSSDTSTPTSSGSQVAASTSQPVR	S3,S/T	1.423	0.225
Kansl1	KAT8 regulatory NSL complex subunit 1	A2A5Y4	S988,T997	SPISPELHSAPLTPVAR	S4,T13	0.940	0.713
Kansl1	KAT8 regulatory NSL complex subunit 1	A2A5Y4	Ambiguous	QLSTSSDTSTPTSSGSQVAASTSQPVR	S/T	1.019	0.862
Kansl2	KAT8 regulatory NSL complex subunit 2	Q8BQR4	T131	TELGSQTPESSR	T7	1.021	0.743
Kansl3	KAT8 regulatory NSL complex subunit 3	A2RSY1	S875	ITTLSPMGSGATPSEEPNSQMLPSSSQR	S5	0.870	0.502
Kansl3	KAT8 regulatory NSL complex subunit 3	A2RSY1	Ambiguous	LPTSPSGSEDLSSVSSSPTSSPK	S/T	1.087	0.165
Kansl3	KAT8 regulatory NSL complex subunit 3	A2RSY1	Ambiguous	LPTSPSGSEDLSSVSSSPTSSPK	T/S	1.032	0.861
Kars	Lysine--tRNA ligase	Q8R2P8	S623	ETAATTETPESTEASPSV	S17	0.996	0.940
Kars	Lysine--tRNA ligase	Q8R2P8	S623	KETAATTETPESTEASPSV	S18	1.129	0.619
Kars	Lysine--tRNA ligase	Q8R2P8	Ambiguous	ETAATTETPESTEASPSV	S/T	0.954	0.437
Kat14	Cysteine-rich protein 2-binding protein	Q8CID0	S284,T/S	EAAGLLDRSTSSTPVK	S9,T/S	0.937	0.375
Kat5	Histone acetyltransferase KAT5	Q8CHK4	S199	KSNCLGTDEDSQDSSDGIPSAPR	S11	0.981	0.936
Kat5	Histone acetyltransferase KAT5	Q8CHK4	S86,S90	NGLPGSRPGSPER	S6,S10	1.021	0.751
Kat5	Histone acetyltransferase KAT5	Q8CHK4	Ambiguous	SNCLGTDEDSQDSSDGIPSAPR	S/T	0.939	0.737
Kat6a	Histone acetyltransferase	G3X940; Q8BRB7	S419;S446	FFTPSPDGR	S5	0.902	0.339
Kat6a	Histone acetyltransferase	G3X940	T1228	KEEMHTEPDEEAEEEEDTTSSDIR	T6	1.054	0.354
Kat6b	Histone acetyltransferase KAT6B	Q8BRB7	S1302	ELAGDTGTAPESDAEPPEEQTQK	S12	1.006	0.937
Kat7	Histone acetyltransferase KAT7	Q5SVQ0	S104	SSGSETEQVVDFSDR	S4	1.072	0.102
Kat7	Histone acetyltransferase KAT7	Q5SVQ0	S113	SSGSETEQVVDFSDRETK	S13	0.974	0.521
Kat7	Histone acetyltransferase KAT7	Q5SVQ0	S12	NAGSSSDGTEDSDFSTDLEHTDSSESDGTSR	S4	1.093	0.661
Kat7	Histone acetyltransferase KAT7	Q5SVQ0	S59	LSQSSQDSSPVR	S9	0.971	0.404
Kat7	Histone acetyltransferase KAT7	Q5SVQ0	T90	SQQQPTPVTPK	T9	0.978	0.648
Kat7	Histone acetyltransferase KAT7	Q5SVQ0	S52,S	SSARLSQSSQDSSPVR	S6,S	1.209	0.082
Kbtbd11	Kelch repeat and BTB domain-containing protein 11	Q8BNW9	S70	ASAAEGSEASPPSLR	S10	0.993	0.532
Kcnab2	Voltage-gated potassium channel subunit beta-2	E0CXZ9	S14	MYPESTTGSPARLSLR	S14	0.957	0.373
Kcnab2	Voltage-gated potassium channel subunit beta-2	E0CXZ9	S14	MYPESTTGSPARLSLR	S14	0.940	0.379
Kcnab2	Voltage-gated potassium channel subunit beta-2	E0CXZ9	S20	QTGSPGMIYSTR	S4	0.967	0.537
Kcnab2	Voltage-gated potassium channel subunit beta-2	E0CXZ9	S9	MYPESTTGSPAR	S9	0.988	0.670
Kcnab2	Voltage-gated potassium channel subunit beta-2	E0CXZ9	Ambiguous	QTGSPGMIYSTR	T/S/Y	1.053	0.483
Kcnab2	Voltage-gated potassium channel subunit beta-2	E0CXZ9	Ambiguous	MYPESTTGSPAR	T/S/Y	0.970	0.845
Kcnj2	Inward rectifier potassium channel 2	P35561	S10	TNRYSIVSSEEDGMK	S5	1.067	0.125

Kcnj2	Inward rectifier potassium channel 2	P35561	S10	TNRYSIVSSEEDGMK	S5	1.056	0.385
Kcnj2	Inward rectifier potassium channel 2	P35561	S13	YSIVSSEEDGMK	S5	0.985	0.853
Kcnj2	Inward rectifier potassium channel 2	P35561	S10,S13	TNRYSIVSSEEDGMK	S5,S8	1.113	0.047
Kcnk13	Potassium channel subfamily K member 13	Q8R1P5	S326	CNISIETDGVMESDTDGR	S4	0.904	0.569
Kcnn4	Intermediate conductance calcium-activated potassium channel protein 4	O89109	S176	SGVLLNASYR	S8	1.090	0.184
Kcnt1	Potassium channel subfamily T member 1	A2AHB8	Ambiguous	SDPLAHVASSSQSR	S	0.908	0.365
Kctd1	BTB/POZ domain-containing protein KCTD1	E9Q6T9	S468	LGSPAPQGCYTDSLNGSAR	S3	1.111	0.264
Kctd12	BTB/POZ domain-containing protein KCTD12	A0A0R4J2B2	S189	SPSGGAAGPLLTPSQSLDGSR	S3	1.049	0.452
Kctd12	BTB/POZ domain-containing protein KCTD12	A0A0R4J2B2	S209	SGYITIGYR	S1	0.973	0.285
Kctd12	BTB/POZ domain-containing protein KCTD12	A0A0R4J2B2	S219	GSYTLGR	S2	0.987	0.895
Kctd12	BTB/POZ domain-containing protein KCTD12	A0A0R4J2B2	S258	EVFGDTLNESRDPDRPPER	S10	0.993	0.918
Kctd12	BTB/POZ domain-containing protein KCTD12	A0A0R4J2B2	Y119	EAEYFELPELVR	Y4	0.796	0.367
Kctd12	BTB/POZ domain-containing protein KCTD12	A0A0R4J2B2	T198,S202	SPSGGAAGPLLTPSQSLDGSRR	T12,S16	1.213	0.040
Kctd12	BTB/POZ domain-containing protein KCTD12	A0A0R4J2B2	T198,S202	SPSGGAAGPLLTPSQSLDGSR	T12,S16	1.139	0.151
Kctd12	BTB/POZ domain-containing protein KCTD12	A0A0R4J2B2	Ambiguous	SPSGGAAGPLLTPSQSLDGSRR	S/T	1.121	0.237
Kctd18	Potassium channel tetramerisation domain containing 18, isoform CRA_a	E0CZ26	Ambiguous	AAQCSVATGASGHAPASPDAQSSENQATHPPPAK	S/T	0.940	0.527
Kctd2	BTB/POZ domain-containing protein KCTD2	A2A9V9	S33	GPPSPRPAGPTPR	S4	1.004	0.905
Kctd3	BTB/POZ domain-containing protein KCTD3	Q8BFX3	S808	SDSSGQEYSL	S3	0.994	0.935
Kctd3	BTB/POZ domain-containing protein KCTD3	Q8BFX3	Ambiguous	LLDQCDLSTSR	S/T	1.174	0.059
Kdm1a	Lysine-specific histone demethylase 1A	Q6ZQ88	S132	EMDESLANLSEDEYYSEEER	S10	1.090	0.539
Kdm1a	Lysine-specific histone demethylase 1A	Q6ZQ88	S167	LPPPPPQAPPEEENESEPEEPSGVEGAAFQSR	S16	0.899	0.212
Kdm1a	Lysine-specific histone demethylase 1A	Q6ZQ88	S167	KLPPPPPQAPPEEENESEPEEPSGVEGAAFQSR	S17	0.879	0.199
Kdm1a	Lysine-specific histone demethylase 1A	Q6ZQ88	S850	QATPGVPAQQSPSM	S11	0.904	0.021
Kdm1a	Lysine-specific histone demethylase 1A	Q6ZQ88	S850	QATPGVPAQQSPSM	S11	1.017	0.538
Kdm1a	Lysine-specific histone demethylase 1A	Q6ZQ88	S132,S138	EMDESLANLSEDEYYSEEER	S10,S16	1.120	0.483
Kdm1b	Lysine-specific histone demethylase 1B	Q8CIG3	S17	SNLELSPDNLPLR	S6	0.941	0.460
Kdm1b	Lysine-specific histone demethylase 1B	Q8CIG3	T247	ATVTAATTTTGSASPGEMEPSK	T8	0.953	0.625
Kdm2a	F-box and leucine-rich repeat protein 11	F6YRW4	S28	RYEDDGISDDEIEGK	S8	0.951	0.518
Kdm2a	F-box and leucine-rich repeat protein 11	F6YRW4	S28	RYEDDGISDDEIEGKR	S8	1.005	0.935
Kdm2a	F-box and leucine-rich repeat protein 11	F6YRW4	S422	TLSGDSSSDSTR	S3	0.995	0.875
Kdm2a	F-box and leucine-rich repeat protein 11	F6YRW4	S692	KIEESDEEAVQAK	S5	1.013	0.835
Kdm2a	F-box and leucine-rich repeat protein 11	F6YRW4	T713,S/T	SCEEPLTPPPHSPTSMLQLIHDPVSPR	T7,S/T	0.973	0.859
Kdm2b	Lysine-specific demethylase 2B	Q6P1G2	S447	TLSNESEESVK	S3	1.056	0.309
Kdm2b	Lysine-specific demethylase 2B	Q6P1G2	S887	SSSPTAGPSTEGAEGPEEK	S3	1.134	0.020
Kdm2b	Lysine-specific demethylase 2B	Q6P1G2	S447,S450	TLSNESEESVK	S3,S6	1.014	0.872
Kdm3a	Lysine-specific demethylase 3A	Q6PCM1	S325	QQNTPQAANSPPNIGAK	S10	0.939	0.524
Kdm3b	Jumonji domain containing 1B	B9EKS2	S290	SASDSGCDPATK	S1	1.250	0.015
Kdm4c	Lysine-specific demethylase 4C	Q8VCD7	S395	RPKPEEDEEFAEFSGEEGANPAMGPR	S14	1.072	0.352
Kdm5a	Lysine-specific demethylase 5A	Q3UXZ9	S1666	QGPDSPGQAPPPPFLMSYK	S5	0.838	0.525
Kdm5a	Lysine-specific demethylase 5A	Q3UXZ9	S204	VEAEVLSTDIQPSPERGTR	S13	1.043	0.517
Kdm5a	Lysine-specific demethylase 5A	Q3UXZ9	S204	VEAEVLSTDIQPSPER	S13	1.031	0.885
Kdm6a	Lysine-specific demethylase 6A	O70546	S829	TDNSVASSPSSAISTATPSPK	S19	1.164	0.039
Kdm6a	Lysine-specific demethylase 6A	O70546	T827,S829	TDNSVASSPSSAISTATPSPK	T17,S19	0.881	0.135
Khsrp	Far upstream element-binding protein 2	Q3U0V1	S182	VQISPDSGGLPER	S4	0.990	0.845
Khsrp	Far upstream element-binding protein 2	Q3U0V1	S194	SVSLTGAPESVQK	S3	0.863	0.167
Khsrp	Far upstream element-binding protein 2	Q3U0V1	S481	GSPQQIDHAK	S2	0.957	0.435
Kiaa0513	Uncharacterized protein KIAA0513	Q8R0A7	S72	SSSNESFSSNQSADSAPDEETLALR	S1	1.033	0.661
Kiaa1109	Transmembrane protein KIAA1109	A2AAE1	S1290	RDDGQASVPTEISGTSPVSPNTQDK	S19	1.058	0.692
Kiaa1109	Transmembrane protein KIAA1109	A2AAE1	S1355	SDENVLDSPKQR	S8	1.089	0.051
Kiaa1109	Transmembrane protein KIAA1109	A2AAE1	S1355	SDENVLDSPK	S8	1.033	0.776
Kiaa1109	Transmembrane protein KIAA1109	A2AAE1	S1681	TTFSENLSPK	S8	1.060	0.711
Kiaa1109	Transmembrane protein KIAA1109	A2AAE1	S1971	TSPSSDINGNSVSDEQDEGVESDDLKK	S13	0.881	0.229
Kiaa1109	Transmembrane protein KIAA1109	A2AAE1	S2603	YTAGSASPTPTFK	S7	1.012	0.847
Kiaa1109	Transmembrane protein KIAA1109	A2AAE1	S2737	SSETFGPAGVRSPTEPTCR	S12	1.093	0.059
Kiaa1109	Transmembrane protein KIAA1109	A2AAE1	S2755	VVFENEQDNNSLTK	S11	0.833	0.052
Kiaa1109	Transmembrane protein KIAA1109	A2AAE1	S628	MVPETGIPAECQSGQK	S13	1.092	0.248
Kiaa1109	Transmembrane protein KIAA1109	A2AAE1	S1805,S1808	MQPQSSGSLRSNAGAEK	S8,S11	0.980	0.368
Kiaa1522	Uncharacterized protein KIAA1522	A2A7S8	S125	SYMTESSTAEDALSVR	S14	0.992	0.894
Kiaa1522	Uncharacterized protein KIAA1522	A2A7S8	S189	EAPGTPQPPGSRDAVR	S11	0.839	0.134
Kiaa1522	Uncharacterized protein KIAA1522	A2A7S8	S213	VSLQALEAETEAGTDAEAVIQR	S2	1.365	0.182
Kiaa1522	Uncharacterized protein KIAA1522	A2A7S8	S325	CSLLSASPASVR	S7	0.992	0.891
Kiaa1522	Uncharacterized protein KIAA1522	A2A7S8	S340	FSSASSPRPR	S6	0.997	0.974
Kiaa1522	Uncharacterized protein KIAA1522	A2A7S8	S402	GSPSGGSTAETSDTASIR	S2	1.026	0.477
Kiaa1522	Uncharacterized protein KIAA1522	A2A7S8	S445	TYSLHQR	S3	0.954	0.660
Kiaa1522	Uncharacterized protein KIAA1522	A2A7S8	S543	GLAVAPASPGK	S8	1.028	0.705
Kiaa1522	Uncharacterized protein KIAA1522	A2A7S8	S610	VPAPFSPPPSK	S6	0.791	0.082
Kiaa1522	Uncharacterized protein KIAA1522	A2A7S8	S842	ASPVTAPSSGLHAAVR	S2	1.046	0.152
Kiaa1522	Uncharacterized protein KIAA1522	A2A7S8	T183	EAPGTPQPPGSR	T5	0.992	0.801
Kiaa1522	Uncharacterized protein KIAA1522	A2A7S8	Ambiguous	SPGASVSSSLTSLCSSSSDPTPLDR	S/T	1.127	0.434
Kiaa1522	Uncharacterized protein KIAA1522	A2A7S8	Ambiguous	TLSPSSGYSSQSGTPTLPPK	S/T/Y	1.034	0.619
Kiaa1551	Uncharacterized protein KIAA1551	Q5DTW7	S1142	SNTSSVQSVSPEKK	S10	0.974	0.791
Kiaa1551	Uncharacterized protein KIAA1551	Q5DTW7	S1215	TVPSPESSDPK	S4	1.191	0.193
Kiaa1551	Uncharacterized protein KIAA1551	Q5DTW7	S1514	SLGSSPVK	S1	1.174	0.505
Kidins220	Kinase D-interacting substrate 220	E9Q9B7	S1549	YQKLPSDEDESGTEESDNTPLLK	S11	0.958	0.588
Kidins220	Kinase D-interacting substrate 220	E9Q9B7	S1578	VAKSPEHSVEPIR	S4	1.106	0.195
Kidins220	Kinase D-interacting substrate 220	E9Q9B7	S1704	TPSTVTLNNNTAPTNR	S3	1.042	0.352
Kidins220	Kinase D-interacting substrate 220	E9Q9B7	S885	VSQNSLGEMTK	S5	1.065	0.569
Kidins220	Kinase D-interacting substrate 220	E9Q9B7	S918	QMSFDLTK	S3	1.019	0.916
Kidins220	Kinase D-interacting substrate 220	E9Q9B7	Ambiguous	SSPHSTYYIGQSSSGGSIHSNLEQER	S/T/Y	0.874	0.569
Kidins220	Kinase D-interacting substrate 220	E9Q9B7	Ambiguous	LPSDEDESGTEESDNTPLLK	S/T	0.986	0.944
Kidins220	Kinase D-interacting substrate 220	E9Q9B7	Ambiguous	AYNLNRTPSTVTLNNNTAPTNR	T/S/Y	0.836	0.050
Kif11	Kinesin-like protein KIF11	Q6P9P6	Ambiguous	LDIPTGMTPER	T	1.081	0.613
Kif13a	Kinesin-like protein KIF13A	F8VQ75	S1447	AFSPQPPK	S3	0.857	0.127
Kif13a	Kinesin-like protein KIF13A	F8VQ75	S1600	EVECAEPPGPSLAPDVRPASNQELTEVGR	S20	0.924	0.705
Kif13a	Kinesin-like protein KIF13A	F8VQ75	S1633	GTPSPQSIPEESSR	S4	1.071	0.460
Kif13a	Kinesin-like protein KIF13A	F8VQ75	S1650	TASCSELDVGPSK	S3	1.069	0.098
Kif13a	Kinesin-like protein KIF13A	F8VQ75	T1717	ALTNETDHK	T3	0.987	0.789
Kif13b	Kinesin family member 13B	A0A286YCV9	S1380	RSISSPSMNR	S4	1.042	0.567
Kif13b	Kinesin family member 13B	A0A286YCV9	S1380	RSISSPSMNR	S4	1.023	0.882
Kif13b	Kinesin family member 13B	A0A286YCV9	S1380	SISSPSMNR	S3	0.980	0.470
Kif13b	Kinesin family member 13B	A0A286YCV9	S1380	SISSPSMNR	S3	1.006	0.875
Kif13b	Kinesin family member 13B	A0A286YCV9	S1395	QELSPSHSLGSNK	S4	0.984	0.915
Kif13b	Kinesin family member 13B	A0A286YCV9	S1409	WESQQDVSQTLVSR	S3	1.072	0.539
Kif13b	Kinesin family member 13B	A0A286YCV9	S1409	GRWESQQDVSQTLVSR	S5	0.983	0.762
Kif13b	Kinesin family member 13B	A0A286YCV9	S1512	TEEAQQGSPGPSGALESMVK	S8	1.047	0.651
Kif13b	Kinesin family member 13B	A0A286YCV9	S1512	TEEAQQGSPGPSGALESMVK	S8	1.065	0.570
Kif13b	Kinesin family member 13B	A0A286YCV9	S1682	SFTGMLGGASSGAEEDPLASEDPSNAR	S11	1.112	0.120
Kif13b	Kinesin family member 13B	A0A286YCV9	S1682	SFTGMLGGASSGAEEDPLASEDPSNAR	S11	1.167	0.555
Kif13b	Kinesin family member 13B	A0A286YCV9	S1798	SSGLQPQGAPEVR	S1	1.052	0.350
Kif13b	Kinesin family member 13B	A0A286YCV9	S1799	RSSGLQPQGAPEVR	S3	1.000	1.000
Kif13b	Kinesin family member 13B	A0A286YCV9	S661	LSFHSPSAQQR	S5	1.049	0.023
Kif13b	Kinesin family member 13B	A0A286YCV9	T902	YDFWDQQEPVTVAPEVDTSSSPTSK	T11	1.173	0.629
Kif13b	Kinesin family member 13B	A0A286YCV9	Ambiguous	APLLSEPASAVPTSPFR	S/T	0.923	0.226
Kif13b	Kinesin family member 13B	A0A286YCV9	Ambiguous	RRSSGLQPQGAPEVR	S	1.079	0.472
Kif13b	Kinesin family member 13B	A0A286YCV9	Ambiguous	LEVTSDSEDASEVPEWLR	T/S	1.039	0.404
Kif16b	Kinesin superfamily protein 16B	E7FLY0	S673	SFHIENK	S1	0.988	0.861
Kif16b	Kinesin superfamily protein 16B	E7FLY0	T1322	GVFDYSSHGTG	T10	1.074	0.383
Kif16b	Kinesin superfamily protein 16B	E7FLY0	Ambiguous	CSTLDLEIQEQR	S/T	0.963	0.647
Kif1a	Kinesin-like protein KIF1A	A0A087WQE8	S1629,S1633	TPQPCSRPASPEPELLPELDSK	S6,S10	0.817	0.460
Kif1b	Kinesin family member 1B, isoform CRA_a	A2AH75	S1050	SNSLNNGQPK	S3	1.083	0.269
Kif1b	Kinesin family member 1B, isoform CRA_a	A2AH75	T1129	QTDKPSHCNQFVTPPR	T13	1.088	0.207
Kif1b	Kinesin-like protein KIF1B	Q60575	S1057	SGLSLEELR	S4	0.950	0.439
Kif1b	Kinesin-like protein KIF1B	Q60575	S1075	IVEGQGQSSEVISPPEEVNR	S13	1.074	0.350
Kif1b	Kinesin-like protein KIF1B	Q60575	S1454	MADTGSPGMQR	S6	1.031	0.605
Kif1b	Kinesin-like protein KIF1B	Q60575	S1454	MADTGSPGMQR	S6	0.988	0.642
Kif1b	Kinesin-like protein KIF1B	Q60575	S1454	MADTGSPGMQR	S6	0.999	0.983
Kif1c	Kinesin-like protein KIF1C	O35071	S1031	RNSLDGGSR	S3	0.959	0.750
Kif1c	Kinesin-like protein KIF1C	O35071	S1089	SAPDLKESGAAV	S1	0.956	0.672
Kif1c	Kinesin-like protein KIF1C	O35071	S1089	QRSAPDLK	S3	1.071	0.193
Kif1c	Kinesin-like protein KIF1C	O35071	S1089	QRSAPDLKESGAAV	S3	0.883	0.442
Kif1c	Kinesin-like protein KIF1C	O35071	S494	EDGGTVGVFSPK	S10	0.922	0.290
Kif1c	Kinesin-like protein KIF1C	O35071	S676	LYADSDSGEDSDKR	S7	0.998	0.934
Kif1c	Kinesin-like protein KIF1C	O35071	S820	DVWDTVGEEEGCGGGGGGSEEGAR	S19	0.972	0.889
Kif1c	Kinesin-like protein KIF1C	O35071	S989	SNPQHRESWPGMGSGEAPAPQPPEEVTVPPAPPNR	S8	0.934	0.555
Kif1c	Kinesin-like protein KIF1C	O35071	T1080	YPPYTTPPR	T6	0.938	0.526
Kif21b	Kinesin-like protein KIF21B	Q9QXL1	S1150	AVSAECLGPPLDSSTK	S3	0.991	0.844
Kif21b	Kinesin-like protein KIF21B	Q9QXL1	S1217	GATDTSPLTR	S6	0.977	0.578
Kif21b	Kinesin-like protein KIF21B	Q9QXL1	S1242,S1243	STDMGFTPPSSPPTRPR	S10,S11	0.994	0.812
Kif21b	Kinesin-like protein KIF21B	Q9QXL1	T1239,S1243	STDMGFTPPSSPPTRPR	T7,S11	1.019	0.555
Kif22	Kinesin-like protein KIF22	Q3V300	S407	GPEEESTGSPESTAAPASASQK	S9	1.270	0.108
Kif23	Kinesin-like protein KIF23	E9Q5G3	S860	YMLTHQELASDGEIQTK	S10	1.043	0.642
Kif2a	Kinesin-like protein	E0CZ72	S139	ARPSQLPEQSSSAQQNGSVSDISPVQAAK	S23	1.242	0.121
Kif3b	Kinesin-like protein KIF3B	Q61771	S681	DYEGPAISPK	S8	1.012	0.865
Kif4	Chromosome-associated kinesin KIF4	P33174	S1224	ALASNTSFFSGCSPIQEESH	S13	1.123	0.352
Kif4	Chromosome-associated kinesin KIF4	P33174	S802	TFSYDEIHGQDSGAEDSIAK	S3	1.761	0.105
Kif5b	Kinesin-1 heavy chain	Q61768	S819	SAEVDSDDTGGSAAQK	S6	1.069	0.198
Kif7	Kinesin-like protein KIF7	B7ZNG0	S662	AGLSPGSPPDR	S7	1.085	0.100
Kif7	Kinesin-like protein KIF7	B7ZNG0	S898	SGSNGSVVSLEQQQK	S1	0.951	0.395

Kifc1	Kinesin-like protein KIFC1	Q9QWT9	S492	RASPGSEELTVTNAR	S3	1.089	0.484
Klc1	Kinesin light chain 1	Q8CD76	S460	VDSPTVTTTLK	S3	1.039	0.671
Klc1	Kinesin light chain 1	Q8CD76	S521	SRESLNMDVVK	S1	1.030	0.783
Klc1	Kinesin light chain 1	Q8CD76	S596	NPGASPAEPLCVENDSSSLEDASTN	S5	1.040	0.343
Klc1	Kinesin light chain 1	Q8CD76	S521,S524	SRESLNMDVVK	S1,S4	1.056	0.477
Klc1	Kinesin light chain 1	Q8CD76	S521,S524	SRESLNMDVVK	S1,S4	1.067	0.344
Klc2	Kinesin light chain 2	Q91YS4	S445	VDSPTVNTTLR	S3	0.940	0.431
Klc2	Kinesin light chain 2	Q91YS4	S508	RGSRDVAGPQSESDLEESGPAAEWSGDGSGSLR	S3	1.056	0.452
Klc2	Kinesin light chain 2	Q91YS4	S606	TLSSSSMDLSR	S4	0.998	0.963
Klc2	Kinesin light chain 2	Q91YS4	S606	TLSSSSMDLSRR	S4	1.009	0.878
Klc2	Kinesin light chain 2	Q91YS4	Ambiguous	TLSSSSMDLSRR	S/T	0.927	0.075
Klc2	Kinesin light chain 2	Q91YS4	Ambiguous	TLSSSSMDLSR	S/T	0.931	0.170
Klc3	Kinesin light chain 3	Q91W40	Ambiguous	TLSISTQDLSPR	S/T	1.060	0.199
Klc4	Kinesin light chain 4	Q9DBS5	S565	RSSELLVR	S2	1.013	0.746
Klc4	Kinesin light chain 4	Q9DBS5	S590	AASLNYLNQPNAAPLQVSR	S3	1.000	1.000
Klc4	Kinesin light chain 4	Q9DBS5	S590	RAASLNYLNQPNAAPLQVSR	S4	0.987	0.873
Klc4	Kinesin light chain 4	Q9DBS5	S611	GLSASTVDLSSSS	S5	1.017	0.633
Klc4	Kinesin light chain 4	Q9DBS5	S611,S/T	GLSASTVDLSSSS	S5,S/T	1.103	0.564
Klf10	Krueppel-like factor 10	O89091	S248	SQPAPSSPVQK	S7	0.949	0.705
Klf13	Krueppel-like factor 13	Q9JJZ6	S163	ADLESPQRK	S5	1.136	0.322
Klf13	Krueppel-like factor 13	Q9JJZ6	S163	ADLESPQR	S5	1.003	0.962
Klf13	Krueppel-like factor 13	Q9JJZ6	S163	SRADLESPQRK	S7	1.075	0.523
Klf13	Krueppel-like factor 13	Q9JJZ6	S271	TGSLSDYSR	S5	1.131	0.079
Klf13	Krueppel-like factor 13	Q9JJZ6	S288	SDASSPTISPASSP	S13	1.032	0.383
Klf13	Krueppel-like factor 13	Q9JJZ6	S280,T282	SDASSPTISPASSP	S5,T7	0.985	0.843
Klf16	Krueppel-like factor 16	P58334	Ambiguous	GGPVVATAASTAGGTSPVSSSSAASSPSSGR	T/S	0.870	0.068
Klf3	Krueppel-like factor 3	Q60980	S249	RPLPVESPDTQR	S7	1.034	0.501
Klf3	Krueppel-like factor 3	Q60980	S71	GSPPAAGGSPSSLK	S2	0.985	0.668
Klf3	Krueppel-like factor 3	Q60980	S71,S78	GSPPAAGGSPSSLK	S2,S9	1.129	0.225
Klf3	Krueppel-like factor 3	Q60980	S91,S	RASPGLSMPSSSPPIK	S3,S	1.093	0.084
Klhdc4	Kelch domain containing 4, isoform CRA_a	G3X961	S418	ADDSASEASSTGQEPCPR	S4	1.010	0.910
Klhl17	Kelch-like protein 17	Q6TDP3	Ambiguous	TQSPEHSSPGPGPEAPPPPQPPAPEAER	T/S	1.009	0.931
Klhl24	Kelch-like protein 24	Q8BRG6	S19	EDLGVRDSPATK	S8	1.003	0.958
Klhl9	Kelch-like protein 9	Q6ZPT1	Ambiguous	ACTLTVFPPEENPGSPSRESPLSAPSDHS	S/T	0.932	0.434
Klhl9	Kelch-like protein 9	Q6ZPT1	Ambiguous	ACTLTVFPPEENPGSPSR	S/T	1.108	0.387
Kmt2a	Histone-lysine N-methyltransferase 2A	P55200	S1839	GPGEPDSPTPLHPPTPPILSTDR	S7	1.042	0.539
Kmt2a	Histone-lysine N-methyltransferase 2A	P55200	S1860	SREDSPELNPPPGIDDNR	S5	1.022	0.749
Kmt2a	Histone-lysine N-methyltransferase 2A	P55200	S2542	ESGPGSPAHIESVCPAEPVSASR	S6	1.000	1.000
Kmt2a	Histone-lysine N-methyltransferase 2A	P55200	T504	SNTPEVHTPLPISQSPENESNDRR	T3	1.046	0.467
Kmt2a	Histone-lysine N-methyltransferase 2A	P55200	Ambiguous	SSVSSVPSLGTATDPEASAK	S/T	1.407	0.272
Kmt2a	Histone-lysine N-methyltransferase 2A	P55200	Ambiguous	NSGTPGLQVPVSPTVPVQNQK	S/T	1.164	0.295
Kmt2a	Histone-lysine N-methyltransferase 2A	P55200	Ambiguous	GPGEPDSPTPLHPPTPPILSTDR	S/T	0.829	0.563
Kmt2b	Histone-lysine N-methyltransferase	F8WJ40	S2355	GVLDLNNPGEQPEEESPGRPQDR	S16	0.948	0.712
Kmt2b	Histone-lysine N-methyltransferase	F8WJ40	S504	SGPEGTLSPTPNPSTTTGSPLEDSPTVVPK	S8	0.950	0.875
Kmt2b	Histone-lysine N-methyltransferase	F8WJ40	S639	ELPPPPPAPPPAPSPPPAPATPSR	S14	1.014	0.836
Kmt2b	Histone-lysine N-methyltransferase	F8WJ40	S866	ASGPESPLQGPR	S6	1.033	0.421
Kmt2b	Histone-lysine N-methyltransferase	F8WJ40	S941	EKVESAGPGGDSEPTGSTGALAHTPR	S12	0.966	0.727
Kmt2b	Histone-lysine N-methyltransferase	F8WJ40	T2088	GQGTPPSGPGVGR	T4	0.956	0.443
Kmt2b	Histone-lysine N-methyltransferase	F8WJ40	Ambiguous	IEQLDGVDDGTDSEAEAVQQPR	T/S	1.208	0.295
Kmt2c	Histone-lysine N-methyltransferase 2C	F8WI37	S28	SAEQQQPPPAPPEEPGAPAPSPAAADKRPR	S21	1.195	0.197
Kmt2c	Histone-lysine N-methyltransferase 2C	F8WI37	S594	SNPLESPDTVGLITSESSDNK	S6	1.161	0.062
Kmt2c	Histone-lysine N-methyltransferase 2C	F8WI37	Ambiguous	TVDVPASLPTPPHNNHEELR	S/T	0.933	0.578
Kmt2d	Histone-lysine N-methyltransferase 2D	A0A0A0MQ73	S1136	QAGQGSPCEEQEEPGAPMAPMPPTLIK	S6	0.747	0.013
Kmt2d	Histone-lysine N-methyltransferase 2D	A0A0A0MQ73	S1627	LEGPASPDVELGKEETEESK	S6	1.058	0.437
Kmt2d	Histone-lysine N-methyltransferase 2D	A0A0A0MQ73	S2231	ASEPLLSPPPFGESR	S7	1.059	0.331
Kmt2d	Histone-lysine N-methyltransferase 2D	A0A0A0MQ73	S2299	ASQVEPQSPGLGLR	S8	1.171	0.004
Kmt2d	Histone-lysine N-methyltransferase 2D	A0A0A0MQ73	S2318	AQEPPPAQALAPSPPSHPDVFR	S13	1.024	0.557
Kmt2d	Histone-lysine N-methyltransferase 2D	A0A0A0MQ73	S3618	QQQQQQQQQQQQQQQHSAVLAVSPSQNPR	S23	1.039	0.573
Kmt2d	Histone-lysine N-methyltransferase 2D	A0A0A0MQ73	S727	LCPQPEEPYLSPQPEEPR	S11	1.026	0.744
Kmt2d	Histone-lysine N-methyltransferase 2D	A0A0A0MQ73	S2208,S2217	CPSLDNLAVPESPGVAGGK	S3,S12	0.997	0.961
Kmt5a	Histone-lysine N-methyltransferase	D3YXI6	T152	SEAAEPPKTPPPSCDSTNVAVAK	T9	0.939	0.212
Kmt5b	Histone-lysine N-methyltransferase KMT5B	Q3U8K7	S427	VPAASNSTSPK	S9	1.100	0.497
Kmt5b	Histone-lysine N-methyltransferase KMT5B	Q3U8K7	S630	TEDCSPEHSFPGK	S5	1.037	0.540
Kmt5c	Histone-lysine N-methyltransferase KMT5C	Q6Q783	S346	QASLPPVLR	S3	1.033	0.534
Kpna2	Importin subunit alpha-1	P52293	S62	NVSSFPDDATSPLQENR	S11	0.992	0.702
Kpna3	Importin subunit alpha-4	O35344	S60	NVPQEESLEDSDVDADFK	S11	1.046	0.326
Kpna3	Importin subunit alpha-4	O35344	S60	RNVPQEESLEDSDVDADFK	S12	0.925	0.436
Kpna4	Importin subunit alpha-3	A0A0B4J1E7	S162	NVPQEDICEDSDIDGDYR	S11	1.004	0.947
Kpna4	Importin subunit alpha-3	A0A0B4J1E7	S162	RNVPQEDICEDSDIDGDYR	S12	1.074	0.370
Kri1	Protein KRI1 homolog	F6WIU1	S142	GGKYVDEDNSDGETVDHR	S10	1.026	0.768
Kri1	Protein KRI1 homolog	F6WIU1	S142	YVDEDNSDGETVDHR	S7	1.011	0.692
Ksr1	Kinase suppressor of Ras 1	Q61097	S320	FELPHGSPQLVR	S7	1.059	0.811
Ksr1	Kinase suppressor of Ras 1	Q61097	S392	RTESVPSDINNPVDR	S4	1.069	0.021
Ksr1	Kinase suppressor of Ras 1	Q61097	S392	TESVPSDINNPVDR	S3	0.946	0.211
Ksr1	Kinase suppressor of Ras 1	Q61097	T411	AAEPHFGTLPK	T8	0.907	0.457
Ksr2	Kinase suppressor of Ras 2	Q3UVC0	S348	IHSGVGSCENIPAQQR	S7	0.918	0.127
Ksr2	Kinase suppressor of Ras 2	Q3UVC0	S484	TESVPCDINNPVR	S3	0.981	0.419
Ktn1	Kinectin	F8VQC7	S75	EIQNGTLRESDSEHVPR	S10	1.070	0.011
L3mbtl3	Lethal(3)malignant brain tumor-like protein 3	Q8BLB7	Ambiguous	LSGDTSPPTTPSFPR	T/S	0.874	0.046
Lamtor1	Ragulator complex protein LAMTOR1	Q9CQ22	S42	ALNGAEPNYHSLPSAR	S11	1.297	0.003
Lamtor1	Ragulator complex protein LAMTOR1	Q9CQ22	T28	LLLDPSSTPTK	T8	1.375	0.096
Large1	LARGE xylosyl- and glucuronyltransferase 1	Q9Z1M7	S756	YLTAENNS	S8	0.909	0.529
Larp1	La-related protein 1	Q6ZQ58	S1032	CPSQSSSRPATGISQPPTTPTGQATR	S3	1.083	0.061
Larp1	La-related protein 1	Q6ZQ58	S302	VEPAWHDQDETSSVKSDGAGGAR	S16	1.099	0.500
Larp1	La-related protein 1	Q6ZQ58	S498	ETESAPGSPR	S8	1.100	0.459
Larp1	La-related protein 1	Q6ZQ58	S604	NTFTAWSEEDSDYEIDDRDVNK	S7	0.888	0.430
Larp1	La-related protein 1	Q6ZQ58	S751	SLPTTVPESPNYR	S9	1.061	0.603
Larp1	La-related protein 1	Q6ZQ58	S81	ESPRPPAAAEAPAGSDGEDGGRR	S15	1.078	0.242
Larp1	La-related protein 1	Q6ZQ58	S81	ESPRPPAAAEAPAGSDGEDGGR	S15	1.019	0.780
Larp1	La-related protein 1	Q6ZQ58	S824	TASISSSPSEGTPAVGSYGCTPQSLPK	S3	1.143	0.249
Larp1	La-related protein 1	Q6ZQ58	T1040	RCPSQSSSRPATGISQPPTTPTGQATR	T12	0.989	0.947
Larp1	La-related protein 1	Q6ZQ58	T503	AVTPVPTK	T3	1.055	0.386
Larp1	La-related protein 1	Q6ZQ58	T503	AVTPVPTKTEEVSNLK	T3	1.059	0.444
Larp1	La-related protein 1	Q6ZQ58	T626	ILIVTQTPPYMR	T7	0.971	0.883
Larp1	La-related protein 1	Q6ZQ58	S1032,T/S	CPSQSSSRPATGISQPPTTPTGQATR	S3,T/S	1.210	0.029
Larp1	La-related protein 1	Q6ZQ58	S1032,T/S	RCPSQSSSRPATGISQPPTTPTGQATR	S4,T/S	1.040	0.875
Larp1	La-related protein 1	Q6ZQ58	S494,S498	QHYQKETESAPGSPR	S9,S13	0.998	0.978
Larp1	La-related protein 1	Q6ZQ58	S494,S498	ETESAPGSPR	S4,S8	1.149	0.237
Larp1	La-related protein 1	Q6ZQ58	S604,S608	NTFTAWSEEDSDYEIDDRDVNK	S7,S11	0.841	0.462
Larp1	La-related protein 1	Q6ZQ58	S743,T/S/Y	SLPTTVPESPNYR	S1,T/S/Y	1.161	0.015
Larp1	La-related protein 1	Q6ZQ58	S824,S826	TASISSSPSEGTPAVGSYGCTPQSLPK	S3,S5	1.019	0.715
Larp1	La-related protein 1	Q6ZQ58	T762,T765	TPRTPRTPQLK	T4,T7	1.014	0.819
Larp1	La-related protein 1	Q6ZQ58	S824,S/T/Y	TASISSSPSEGTPAVGSYGCTPQSLPK	S3,S/T/Y	1.040	0.661
Larp1	La-related protein 1	Q6ZQ58	Ambiguous	HSSNPPLESHVGWVMDSR	S	1.025	0.817
Larp1	La-related protein 1	Q6ZQ58	Ambiguous	VEPAWHDQDETSSVK	T/S	0.961	0.149
Larp1b	La ribonucleoprotein domain family, member 1B (Fragment)	A0A0A6YXJ7	S42	LDGPTENISEDEAQSSSQR	S9	1.017	0.655
Larp4	La-related protein 4	G3X9Q6	S396	SSSGSEHSTEGSVSLGDGPLSR	S21	1.354	0.165
Larp4	La-related protein 4	G3X9Q6	S69	EYEVMYSPSCETTR	S7	1.078	0.246
Larp4	La-related protein 4	G3X9Q6	S717	EQYVPPRSPK	S8	1.052	0.566
Larp4b	La-related protein 4B	Q6A0A2	S490	VEPSSLESSPGLGR	S9	1.184	0.017
Larp4b	La-related protein 4B	Q6A0A2	S570	ERNLSTDASTNTVPVVGPR	S5	1.080	0.201
Larp4b	La-related protein 4B	Q6A0A2	S570	NLSTDASTNTVPVVGPR	S3	1.048	0.415
Larp4b	La-related protein 4B	Q6A0A2	S603	SPSPVHLPEDPKVAEK	S3	0.904	0.237
Larp4b	La-related protein 4B	Q6A0A2	S721	RASPPAAGK	S3	1.044	0.261
Larp4b	La-related protein 4B	Q6A0A2	S721	RASPPAAGKR	S3	0.955	0.579
Larp4b	La-related protein 4B	Q6A0A2	T735	EQNTPPKSPQ	T4	1.043	0.095
Larp4b	La-related protein 4B	Q6A0A2	T735,S739	EQNTPPKSPQ	T4,S8	1.055	0.256
Larp4b	La-related protein 4B	Q6A0A2	T735,S739	LSKEQNTPPKSPQ	T7,S11	0.930	0.370
Larp4b	La-related protein 4B	Q6A0A2	Ambiguous	ETQSVDRLPSTPTTTACK	S/T	0.949	0.724
Larp4b	La-related protein 4B	Q6A0A2	Ambiguous	SPSPVHLPEDPK	S	0.997	0.880
Larp7	La-related protein 7	Q05CL8	S253	TASEGSEAETPEAPK	S3	1.002	0.945
Larp7	La-related protein 7	Q05CL8	S253	TASEGSEAETPEAPKQPAK	S3	0.975	0.336
Larp7	La-related protein 7	Q05CL8	S334	DLEFCSTEEEKETDR	S6	1.064	0.345
Larp7	La-related protein 7	Q05CL8	S239,S240	ESAVDSSSSGVCK	S8,S9	1.135	0.365
Larp7	La-related protein 7	Q05CL8	S253,S256	TASEGSEAETPEAPK	S3,S6	1.092	0.104
Larp7	La-related protein 7	Q05CL8	S253,S256	TASEGSEAETPEAPKQPAKK	S3,S6	1.033	0.703
Larp7	La-related protein 7	Q05CL8	S253,S256	TASEGSEAETPEAPKQPAK	S3,S6	1.037	0.418
Larp7	La-related protein 7	Q05CL8	Ambiguous	DKVEASSLPEAR	S	0.998	0.983
Las1l	Ribosomal biogenesis protein LAS1L	A2BE28	S20	AWDGQSFKENQPESPSAR	S14	0.954	0.450
Lasp1	LIM and SH3 domain protein 1	Q61792	T104	GFSVVADTPELQR	T8	0.957	0.194
Lasp1	LIM and SH3 domain protein 1	Q61792	T68	QSFTMVADTPENLR	T9	0.977	0.876
Lat	Linker for activation of T-cells family member 1	O54957	S199	EYVNVSPEQQPVTR	S6	0.975	0.748
Lat	Linker for activation of T-cells family member 1	O54957	S41	ELPVSYDSTSTESLYPR	S10	1.032	0.623
Lat	Linker for activation of T-cells family member 1	O54957	Ambiguous	CRELPVSYDSTSTESLYPR	T/S/Y	0.850	0.341
Lat2	Linker for activation of T-cells family member 2	Q9JHL0	S60	NRQENAQSSAAAQTYSLAR	S16	0.968	0.733
Lat2	Linker for activation of T-cells family member 2	Q9JHL0	S95	MLFSHLEGPESPR	S11	1.031	0.545
Lat2	Linker for activation of T-cells family member 2	Q9JHL0	Ambiguous	QENAQSSAAAQTYSLAR	T/S/Y	0.884	0.236
Lats1	Serine/threonine-protein kinase LATS1	Q8BYR2	S278	RYSGNMEYVISR	S3	0.972	0.725

Lats1	Serine/threonine-protein kinase LATS1	Q8BYR2	S463	SNSFNNPLGSR	S3	0.983	0.737
Lats1	Serine/threonine-protein kinase LATS1	Q8BYR2	S612	QITTSPITVR	S5	0.987	0.653
Lats1	Serine/threonine-protein kinase LATS1	Q8BYR2	T17	TFPASNYPGSSR	T1	1.012	0.763
Lats1	Serine/threonine-protein kinase LATS1	Q8BYR2	T246	SVTPPPPPR	T3	0.975	0.775
Lats1	Serine/threonine-protein kinase LATS1	Q8BYR2	T246,T255	SVTPPPPPRGQTPPPR	T3,T12	0.979	0.833
Lats1	Serine/threonine-protein kinase LATS1	Q8BYR2	Ambiguous	GTTPPPPSWEPSSQTK	T/S	0.938	0.558
Lats2	Serine/threonine-protein kinase LATS2	Q7TSJ6	S534	QIQTSPVPVR	S5	1.112	0.066
Layn	Layilin	Q8C351	S286	QENSPNLDVYNVIR	S4	1.135	0.150
Lbr	Lamin-B receptor	Q3U9G9	S103	SVSASHEGDVK	S3	1.066	0.200
Lbr	Lamin-B receptor	Q3U9G9	S67,S	SGSISSSPSRR	S3,S	0.953	0.652
Lca5	Lebercilin	G5E886	S543	GEGGSPGPIRSPGQIR	S11	0.910	0.383
Lcmt1	Leucine carboxyl methyltransferase 1	A0A0U1RNF2	S247	SCDLAGVETCK	S1	0.892	0.163
Lcmt1	Leucine carboxyl methyltransferase 1	A0A0U1RNF2	S247	RRSCDLAGVETCK	S3	0.974	0.645
Lcp1	Plastin-2	Q61233	S257	EGESLEDLMK	S4	0.979	0.650
Lcp1	Plastin-2	Q61233	S257	EGESLEDLMK	S4	1.011	0.814
Lcp1	Plastin-2	Q61233	S323	GDEEGIPAVVIDMSGLR	S14	0.933	0.794
Lcp1	Plastin-2	Q61233	S406	NWMNSLGVNPR	S5	1.052	0.203
Lcp1	Plastin-2	Q61233	S5	GSVSDEEMMELR	S2	1.099	0.004
Lcp1	Plastin-2	Q61233	S5	GSVSDEEMMELR	S2	1.223	0.041
Lcp1	Plastin-2	Q61233	S5	GSVSDEEMMELR	S2	1.160	0.008
Lcp1	Plastin-2	Q61233	T353	QFVTATDVVR	T6	0.960	0.199
Lcp1	Plastin-2	Q61233	Y48	AACLPLPGYR	Y9	0.997	0.962
Lcp2	Lymphocyte cytosolic protein 2	Q60787	S210	NHSPLSPPHPNHEEPSR	S6	0.972	0.877
Lcp2	Lymphocyte cytosolic protein 2	Q60787	T341	ENDEDDVHQRPLPQPSLPSMSSNTFPSR	T24	0.944	0.357
Lcp2	Lymphocyte cytosolic protein 2	Q60787	T341	ENDEDDVHQRPLPQPSLPSMSSNTFPSR	T24	0.863	0.097
Ldha	L-lactate dehydrogenase	A0A1B0GSX0	Y268	QVVDSAYEVIK	Y7	0.960	0.826
Ldlrap1	Low density lipoprotein receptor adapter protein 1	Q8C142	S198	RDSTPSLK	S3	1.108	0.016
Lemd2	LEM domain-containing protein 2	Q6DVA0	S130	GLSYPPHAGPGPLR	S3	1.067	0.514
Lemd2	LEM domain-containing protein 2	Q6DVA0	Ambiguous	WTKPSSFSDSER	S/T	0.945	0.120
Lemd3	Inner nuclear membrane protein Man1	D3YU56	S140	VLLGFSSDESDVEASPR	S10	0.992	0.928
Lemd3	Inner nuclear membrane protein Man1	D3YU56	S260	EHYSDSEEEEEEGEEDGDVAPAR	S6	0.952	0.802
Lemd3	Inner nuclear membrane protein Man1	D3YU56	S258,S260	EHYSDSEEEEEEGEEDGDVAPAR	S4,S6	1.272	0.047
Leng8	Leukocyte receptor cluster (LRC) member 8, isoform CRA_a	D3YWS8	S352	EPVAESPK	S6	1.031	0.773
Leo1	RNA polymerase-associated protein LEO1	Q5XJE5	S609	IYSSDSDEGSEEDKAQR	S4	0.999	0.995
Leo1	RNA polymerase-associated protein LEO1	Q5XJE5	S631	KLNSDEEGESSGK	S4	0.975	0.729
Leo1	RNA polymerase-associated protein LEO1	Q5XJE5	S631	KLNSDEEGESSGKR	S4	0.989	0.796
Leo1	RNA polymerase-associated protein LEO1	Q5XJE5	S659	YVISDEEEEEDD	S4	0.983	0.742
Leo1	RNA polymerase-associated protein LEO1	Q5XJE5	S659	KYVISDEEEEEDD	S5	0.986	0.882
Lig1	DNA ligase	Q3U4X8	S67	ERNQVVPESDSPVKR	S11	1.155	0.093
Lig1	DNA ligase	Q3U4X8	S67	NQVVPESDSPVKR	S9	0.971	0.467
Lig1	DNA ligase	Q3U4X8	S67	NQVVPESDSPVK	S9	0.968	0.580
Lig1	DNA ligase	Q3U4X8	T93	VAQVLSCEGEDEDEAPGTPK	T18	1.060	0.361
Lig1	DNA ligase	Q3U4X8	T93	KVAQVLSCEGEDEDEAPGTPK	T19	0.896	0.016
Lig1	DNA ligase	Q3U4X8	S81,T93	VAQVLSCEGEDEDEAPGTPK	S6,T18	1.049	0.431
Lig1	DNA ligase	Q3U4X8	Ambiguous	KQSQIQNQQSSDLDSDVEDY	S/Y	1.068	0.503
Lig1	DNA ligase	Q3U4X8	Ambiguous	QSQIQNQQSSDLDSDVEDY	S/Y	1.014	0.887
Lig3	DNA ligase	K4DI59	S211	LTTTGQVTSPVK	S9	0.948	0.734
Lig3	DNA ligase	K4DI59	Ambiguous	ADFAVVAGDEASPTTGGSSGENEGTAGSAGPCK	S/T	1.294	0.174
Lilrb3	Leukocyte immunoglobulin-like receptor subfamily B member 3	P97484	S684	DLQLSSGAEEPITR	S5	1.104	0.041
Lilrb3	Leukocyte immunoglobulin-like receptor subfamily B member 3	P97484	S757	AGHVSPSVMSR	S5	0.924	0.269
Lilrb3	Leukocyte immunoglobulin-like receptor subfamily B member 3	P97484	S757	AGHVSPSVMSR	S5	0.949	0.661
Lima1	LIM domain and actin-binding protein 1	Q9ERG0	S360	SEAQQPMHPKPLSPDAR	S13	1.070	0.124
Lima1	LIM domain and actin-binding protein 1	Q9ERG0	S360	SEAQQPMHPKPLSPDAR	S13	1.004	0.930
Lima1	LIM domain and actin-binding protein 1	Q9ERG0	S372	TSSLPESSPSK	S8	1.079	0.054
Lima1	LIM domain and actin-binding protein 1	Q9ERG0	S488	SDNEETLGRPAQPPNAGESPHSPGVEDAPIAK	S22	1.049	0.378
Lima1	LIM domain and actin-binding protein 1	Q9ERG0	S607	ASSPSLR	S3	0.994	0.853
Lima1	LIM domain and actin-binding protein 1	Q9ERG0	S485,S488	SDNEETLGRPAQPPNAGESPHSPGVEDAPIAK	S19,S22	1.035	0.769
Lima1	LIM domain and actin-binding protein 1	Q9ERG0	Ambiguous	QSSPASYTNELK	S/Y/T	1.060	0.501
Limd1	LIM domain-containing protein 1	Q9QXD8	S308	RDSGLGYEASGR	S3	0.930	0.265
Limd1	LIM domain-containing protein 1	Q9QXD8	S659	GPSPAPLHQHHF	S3	1.017	0.851
Limd1	LIM domain-containing protein 1	Q9QXD8	Ambiguous	ESSPSWSTDSSLEPVLPGSPTPSR	S/T	0.970	0.379
Limk1	LIM domain kinase 1	P53668	S210	VQGVDPGCMSPDVK	S10	1.166	0.295
Limk1	LIM domain kinase 1	P53668	S310	SCSIDTSPGTSSLASPASQR	S15	1.070	0.042
Limk1	LIM domain kinase 1	P53668	S298,S310	SCSIDTSPGTSSLASPASQRK	S3,S15	1.117	0.380
Limk1	LIM domain kinase 1	P53668	S310,T/S	SCSIDTSPGTSSLASPASQR	S15,T/S	0.956	0.765
Limk1	LIM domain kinase 1	P53668	Ambiguous	DLGRSESLR	S	0.986	0.912
Lin37	Protein lin-37 homolog	Q9D8N6	S135	ERERSPGSPLPPLPEDGEGSEVINSK	S5	0.968	0.663
Lin37	Protein lin-37 homolog	Q9D8N6	S138	SPGSPLPPLPEDGEGSEVINSK	S4	0.933	0.305
Lin37	Protein lin-37 homolog	Q9D8N6	S182	SRIPSPLQPETEGTPDDEPSEPEPSPSTLIYR	S5	0.843	0.105
Lin37	Protein lin-37 homolog	Q9D8N6	Ambiguous	ERSPGSPLPPLPEDGEGSEVINSK	S	0.978	0.523
Lin52	Protein lin-52 homolog	Q8CD94	S53	SPITSSPPK	S6	0.992	0.786
Lin54	Protein lin-54 homolog	Q571G4	T280	VLSQSTPGTPSK	T9	0.912	0.415
Lin9	Protein lin-9 homolog	A0A0A6YVZ7	S81	LFSDEDDR	S3	1.300	0.086
Llgl1	Lethal(2) giant larvae protein homolog 1	A0A0R4J0S4	S964	LQESPKLSQANGTR	S4	0.972	0.743
Llgl1	Lethal(2) giant larvae protein homolog 1	A0A0R4J0S4	S986	DIILAPESCEGSPSSAHSK	S12	0.883	0.264
Llgl1	Lethal(2) giant larvae protein homolog 1	A0A0R4J0S4	T703	LQEANAQLAEQTCPHDLEMTPVQR	T20	0.936	0.452
Llgl1	Lethal(2) giant larvae protein homolog 1	A0A0R4J0S4	S982,S986	DIILAPESCEGSPSSAHSK	S8,S12	1.151	0.641
Lman1	Protein ERGIC-53	Q9D0F3	S333	GHPDLQGQPADDIFESIGDR	S16	0.913	0.679
Lmbrd1	Probable lysosomal cobalamin transporter	Q8K0B2	Ambiguous	SVIEGVDEDSDLSDDEPSAYSA	S/Y	1.097	0.714
Lmna	Prelamin-A/C	P48678	S12	SGAQASSTPLSPTR	S1	1.009	0.576
Lmna	Prelamin-A/C	P48678	S212	NIYSEELR	S4	0.971	0.359
Lmna	Prelamin-A/C	P48678	S277	LDNARQSAER	S7	1.014	0.792
Lmna	Prelamin-A/C	P48678	S390	LRLSPSPTSQR	S4	1.027	0.476
Lmna	Prelamin-A/C	P48678	S392	LSPSPTSQR	S4	1.008	0.911
Lmna	Prelamin-A/C	P48678	S458	NKSNEDQSMGNWQIR	S3	0.969	0.535
Lmna	Prelamin-A/C	P48678	S463	SNEDQSMGNWQIR	S6	1.025	0.404
Lmna	Prelamin-A/C	P48678	S51	SLETENAGLR	S1	1.088	0.525
Lmna	Prelamin-A/C	P48678	S51	VRSLETENAGLR	S3	1.013	0.835
Lmna	Prelamin-A/C	P48678	S533	TALINSTGEEVAMR	S6	0.859	0.280
Lmna	Prelamin-A/C	P48678	S533	TALINSTGEEVAMR	S6	1.020	0.283
Lmna	Prelamin-A/C	P48678	S573	GSHCSGSGDPAEYNLR	S5	1.011	0.712
Lmna	Prelamin-A/C	P48678	S629	SVGGSGGGSFGDNLVTR	S1	0.952	0.295
Lmna	Prelamin-A/C	P48678	S66	ITESEEVVSR	S4	1.050	0.394
Lmna	Prelamin-A/C	P48678	S390,S392	LRLSPSPTSQR	S4,S6	1.058	0.008
Lmna	Prelamin-A/C	P48678	S407,S/T	ASSHSSQSQGGGSVTK	S6,S/T	1.081	0.398
Lmna	Prelamin-A/C	P48678	T19,S22	SGAQASSTPLSPTR	T8,S11	1.064	0.395
Lmna	Prelamin-A/C	P48678	Ambiguous	ASSHSSQSQGGGSVTK	S/T	1.182	0.522
Lmna	Prelamin-A/C	P48678	Ambiguous	SYLLGNSSPR	S/Y	1.062	0.245
Lmna	Prelamin-A/C	P48678	Ambiguous	ASSHSSQSQGGGSVTKK	S/T	1.112	0.512
Lmnb1	Lamin-B1	P14733	S17	AGSRASAPATPLSPTR	S6	0.982	0.671
Lmnb1	Lamin-B1	P14733	S201	CQSLTEDLEFR	S3	1.023	0.761
Lmnb1	Lamin-B1	P14733	S279	LENARLSSEMNTSTVNSAR	S7	1.126	0.057
Lmnb1	Lamin-B1	P14733; A0A0R4J0Q5	S376;S386	LALDMEISAYR	S8	0.922	0.170
Lmnb1	Lamin-B1	P14733; A0A0R4J0Q5	S394;S404	LSPSPSSR	S4	0.950	0.375
Lmnb1	Lamin-B1	P14733	T21	ASAPATPLSPTR	T6	1.118	0.030
Lmnb1	Lamin-B1	P14733	S14,S17	AGSRASAPATPLSPTR	S3,S6	0.994	0.909
Lmnb1	Lamin-B1	P14733	T21,S24	ASAPATPLSPTR	T6,S9	0.972	0.882
Lmnb1	Lamin-B1	P14733	Ambiguous	ASAPATPLSPTRLSR	S/T	1.122	0.268
Lmnb2	Lamin-B2	A0A0R4J0Q5	S418	ATSSSSSSSGVGMSVGQGR	S5	1.020	0.602
Lmnb2	Lamin-B2	A0A0R4J0Q5	S446	RLETEDTSGSPSR	S10	0.971	0.671
Lmnb2	Lamin-B2	A0A0R4J0Q5	S446	LETEDTSGSPSR	S9	1.040	0.328
Lmnb2	Lamin-B2	A0A0R4J0Q5	Ambiguous	ATSSSSSSSGVGMSVGQGR	S/T	0.964	0.853
Lmo2	Rhombotin-2	A2BHP3	T65	GAPPPPGTPPPSPMSSAIER	T8	1.129	0.102
Lmo2	Rhombotin-2	A2BHP3	T65	GAPPPPGTPPPSPMSSAIER	T8	1.135	0.276
Lmo2	Rhombotin-2	A2BHP3	T65,S69	GAPPPPGTPPPSPMSSAIER	T8,S12	0.921	0.520
Lmo2	Rhombotin-2	A2BHP3	T65,S69	GAPPPPGTPPPSPMSSAIER	T8,S12	0.978	0.853
Lmod1	Leiomodin-1	F8VPR1	S550	NSLSPATQR	S4	0.977	0.744
Lmtk2	Serine/threonine-protein kinase LMTK2	Q3TYD6	S1418	YFSPPPPAR	S3	1.044	0.211
Lnpep	Leucyl-cystinyl aminopeptidase	Q8C129	S51	EPCLHPLEPDEVEYEPRGSR	S19	1.036	0.680
Lnpep	Leucyl-cystinyl aminopeptidase	Q8C129	Y70	GLGEHEMDEDEEDYESSAK	Y14	1.001	0.976
Lnpep	Leucyl-cystinyl aminopeptidase	Q8C129	Y70	GLGEHEMDEDEEDYESSAK	Y14	0.905	0.026
Lnpk	Endoplasmic reticulum junction formation protein lunapark	Q7TQ95	S177	NLSPAPASSSQGPPPQGPVSPGPAK	S3	1.040	0.212
Lnpk	Endoplasmic reticulum junction formation protein lunapark	Q7TQ95	S411	ADSVPNLEPSEESLVTK	S3	1.272	0.001
Lnpk	Endoplasmic reticulum junction formation protein lunapark	Q7TQ95	T392	TSGPEEPAENQEETENEETSTNEAK	T14	1.012	0.841
Lpin1	Phosphatidate phosphatase LPIN1	E9QKQ5	S483	SANQSPQSVGGSGIDSGVESTSDSLR	S16	0.930	0.676
Lpin1	Phosphatidate phosphatase LPIN1	E9QKQ5	S468,S472	SANQSPQSVGGSGIDSGVESTSDSLR	S1,S5	1.192	0.314
Lpin1	Phosphatidate phosphatase LPIN1	E9QKQ5	Ambiguous	EPLPPFENQDMHSASA	S	1.110	0.309
Lpin2	Phosphatidate phosphatase LPIN2	E9PWN0	S225	KEEQAASPVAEDVGDVGVSSDDEK	S20	1.049	0.562
Lpin2	Phosphatidate phosphatase LPIN2	E9PWN0	S281	SDSELEVKPSESLLR	S1	1.017	0.790
Lpin2	Phosphatidate phosphatase LPIN2	E9PWN0	T329	TATITPSENTHFR	T5	1.050	0.587
Lpin2	Phosphatidate phosphatase LPIN2	E9PWN0	Ambiguous	KEEQAASPVAEDVGDVGVSSDDEKR	S	1.082	0.237
Lpp	Lipoma-preferred partner homolog	Q8BFW7	Y302	YYEPYYAAGPSYGGR	Y6	0.931	0.515
Lpxn	Leupaxin	Q99N69	S188	EELGSSPFFER	S6	1.057	0.360
Lpxn	Leupaxin	Q99N69	S48	TLSSQGNTSPLK	S3	0.977	0.646
Lpxn	Leupaxin	Q99N69	T327	RGTLCHDCGQPITGR	T3	1.050	0.601
Lpxn	Leupaxin	Q99N69	Y213	CAYCAAPITDK	Y3	0.958	0.429
Lpxn	Leupaxin	Q99N69	Y62	VQLVYATNIQEPNVYSEVQEPK	Y5	0.815	0.083
Lpxn	Leupaxin	Q99N69	S48,S54	TLSSQGNTSPLK	S3,S9	0.861	0.247

Lpxn	Leupaxin	Q99N69	Ambiguous	CTFQDSEEYSNPVSCHLDQQSTEESK	Y/S/T	1.153	0.662
Lrba	Lipopolysaccharide-responsive and beige-like anchor protein	Q9ESE1	S1000	ASSIDSASNTELQTHDMSSDEK	S3	1.274	0.184
Lrba	Lipopolysaccharide-responsive and beige-like anchor protein	Q9ESE1	S1219	ATNLAGETESVSDCADNVSEAPATSEQK	S10	0.964	0.556
Lrba	Lipopolysaccharide-responsive and beige-like anchor protein	Q9ESE1	S982	DSPISPHFTR	S5	1.027	0.646
Lrch1	Leucine-rich repeat and calponin homology domain-containing protein 1	P62046	S317	DSDSGVGSDNGDKR	S8	1.165	0.011
Lrch1	Leucine-rich repeat and calponin homology domain-containing protein 1	P62046	S539	SGSWCPEEVQGSLQAESSPR	S3	0.871	0.283
Lrch3	Leucine-rich repeat and calponin homology domain-containing protein 3	E9QAU9	S324	WSGNEPTDEFSDLPLR	S2	1.149	0.215
Lrch3	Leucine-rich repeat and calponin homology domain-containing protein 3	E9QAU9	S625	GRASPLLLSSAPATDPTDAITR	S4	1.523	0.388
Lrch4	Leucine-rich repeat and calponin homology domain-containing protein 4	Q921G6	S313	RWSGNESTDDFSELSFR	S3	0.797	0.020
Lrch4	Leucine-rich repeat and calponin homology domain-containing protein 4	Q921G6	S517,S518,S	SSSQSGSSPSSPESVLRPRPFPQEK	S7,S8,S	1.476	0.033
Lrmp	Lymphoid-restricted membrane protein	G5E880	S362	VTIASLPR	S5	1.026	0.712
Lrmp	Lymphoid-restricted membrane protein	G5E880	S443	DAPEPQGEEAVEGTRKPSLSER	S18	1.122	0.141
Lrp1	Low density lipoprotein receptor-related protein 1	A0A0R4J0I9	S4524	HSLASTDEKR	S5	1.008	0.872
Lrp1	Low density lipoprotein receptor-related protein 1	A0A0R4J0I9	S4524	HSLASTDEK	S5	0.812	0.236
Lrp12	Low-density lipoprotein receptor-related protein 12	Q8BUJ9	S850	TETSDDEALLLC	S4	1.052	0.752
Lrp12	Low-density lipoprotein receptor-related protein 12	Q8BUJ9	T655	SLFSVESDDTDTENERR	T10	1.007	0.959
Lrp6	Low-density lipoprotein receptor-related protein	A0A0R4J0A9	S1490	GTYFPAILNPPPSPATER	S13	1.039	0.831
Lrp6	Low-density lipoprotein receptor-related protein	A0A0R4J0A9	S1590	SQYLSAEENYESCPPSPYTER	S16	1.105	0.531
Lrp6	Low-density lipoprotein receptor-related protein	A0A0R4J0A9	T1572	GYTSDVNYDSEPVPPPPTPR	T18	1.030	0.805
Lrpap1	Alpha-2-macroglobulin receptor-associated protein	P55302	S138	DAQMVHSNALNEDTQDELGDPR	S7	1.042	0.450
Lrpap1	Alpha-2-macroglobulin receptor-associated protein	P55302	S138	DAQMVHSNALNEDTQDELGDPR	S7	0.998	0.941
Lrpap1	Alpha-2-macroglobulin receptor-associated protein	P55302	S53	RESGEEFRMEK	S3	0.891	0.362
Lrpap1	Alpha-2-macroglobulin receptor-associated protein	P55302	S53	RESGEEFR	S3	1.062	0.621
Lrrc14	Leucine-rich repeat-containing protein 14	Q8VC16	Ambiguous	TCIAQQQGGTAEPGLSPVPVEIR	T/S	0.902	0.719
Lrrc25	Leucine-rich repeat-containing protein 25	Q8K1T1	S211	AQMSPHDIPKPVTDFLPR	S4	1.207	0.217
Lrrc25	Leucine-rich repeat-containing protein 25	Q8K1T1	S238	APDSPPSR	S4	1.074	0.005
Lrrc25	Leucine-rich repeat-containing protein 25	Q8K1T1	S267	NSPSGDSDCYMNYR	S2	1.046	0.216
Lrrc25	Leucine-rich repeat-containing protein 25	Q8K1T1	S267	NSPSGDSDCYMNYR	S2	0.974	0.665
Lrrc25	Leucine-rich repeat-containing protein 25	Q8K1T1	S294	SVDQDSQPVYCNLESLGR	S15	1.009	0.857
Lrrc25	Leucine-rich repeat-containing protein 25	Q8K1T1	S267,S269	NSPSGDSDCYMNYR	S2,S4	0.978	0.707
Lrrc25	Leucine-rich repeat-containing protein 25	Q8K1T1	S267,S272	NSPSGDSDCYMNYR	S2,S7	0.973	0.371
Lrrc39	Leucine-rich repeat-containing protein 39	Q8BGI7	Ambiguous	RTEDQVNCLTQMPSSIHSDGESN	T/S	1.068	0.163
Lrrc41	Leucine-rich repeat-containing protein 41	Q8K1C9	S276	APSRDEGSLLLGSR	S3	1.175	0.007
Lrrc41	Leucine-rich repeat-containing protein 41	Q8K1C9	S326	STQESLAIGGTDSK	S1	1.923	0.002
Lrrc75a	Leucine-rich repeat-containing protein 75A	Q7TSF4	S322	EGTDQQDPIGSPVTPAR	S11	1.029	0.663
Lrrc8a	Volume-regulated anion channel subunit LRRC8A	Q80WG5	Ambiguous	SSTVSEDVEATVPMLQR	S/T	1.104	0.144
Lrrc8b	Volume-regulated anion channel subunit LRRC8B	Q5DU41	S191	TLLSTSGGSADIDASK	S4	0.948	0.363
Lrrc8c	Volume-regulated anion channel subunit LRRC8C	Q8R502	T198	SGTIQSGPEGNLVR	T3	1.047	0.016
Lrrc8d	Volume-regulated anion channel subunit LRRC8D	Q8BGR2	T234	ITGAQTLPK	T6	1.112	0.458
Lrrfip1	Leucine-rich repeat flightless-interacting protein 1	A0A087WPK3; Q3UZ39	S16;S16	EIDCLSPEAQR	S6	1.005	0.907
Lrrfip1	Leucine-rich repeat flightless-interacting protein 1	A0A087WPK3	S232	ASPVVEERPDKDFAEK	S2	0.980	0.840
Lrrfip1	Leucine-rich repeat flightless-interacting protein 1	A0A087WPK3; Q3UZ39	S271;S88	GSGDTSISMDTEASIR	S2	0.838	0.479
Lrrfip1	Leucine-rich repeat flightless-interacting protein 1	A0A087WPK3; Q3UZ39	S271;S88	RGSGDTSISMDTEASIR	S3	0.966	0.551
Lrrfip1	Leucine-rich repeat flightless-interacting protein 1	A0A087WPK3; Q3UZ39	S271;S88	RGSGDTSISMDTEASIR	S3	1.012	0.722
Lrrfip1	Leucine-rich repeat flightless-interacting protein 1	Q3UZ39	S284	GVSHPGEK	S3	1.395	0.031
Lrrfip1	Leucine-rich repeat flightless-interacting protein 1	Q3UZ39	S302	AENQRPAEDSALSPGPLAGAK	S13	1.025	0.696
Lrrfip1	Leucine-rich repeat flightless-interacting protein 1	Q3UZ39	S317	CEQQVQSQDQENTSDLK	S7	1.129	0.294
Lrrfip1	Leucine-rich repeat flightless-interacting protein 1	Q3UZ39	S348	ASNSPEQSSCLEGLDSEVPGPTEDLK	S4	1.040	0.540
Lrrfip1	Leucine-rich repeat flightless-interacting protein 1	Q3UZ39	S501	DEKPIQAEAQASPGAPINQSGHQDTTGPGSTDAQR	S12	1.053	0.725
Lrrfip1	Leucine-rich repeat flightless-interacting protein 1	Q3UZ39	S547	SEQQAEALDSPQKK	S10	0.974	0.205
Lrrfip1	Leucine-rich repeat flightless-interacting protein 1	Q3UZ39	S547	SEQQAEALDSPQK	S10	1.028	0.576
Lrrfip1	Leucine-rich repeat flightless-interacting protein 1	Q3UZ39	S605	QASGSPEQK	S5	0.896	0.298
Lrrfip1	Leucine-rich repeat flightless-interacting protein 1	Q3UZ39	S614	AGSREPVEDPQSGSSGK	S3	1.029	0.268
Lrrfip1	Leucine-rich repeat flightless-interacting protein 1	Q3UZ39	S649	QNKVEEDGPTEGPTDILDQNSPQCEDR	S21	1.005	0.906
Lrrfip1	Leucine-rich repeat flightless-interacting protein 1	Q3UZ39	Ambiguous	SDSRASNSPEQSSCLEGLDSEVPGPTEDLK	S/T	0.954	0.602
Lrrfip1	Leucine-rich repeat flightless-interacting protein 1	Q3UZ39	Ambiguous	VEEDGPTEGPTDILDQNSPQCEDR	S/T	1.472	0.005
Lrrfip2	Leucine-rich repeat flightless-interacting protein 2	A0A1L1SSC0	S269	NSASATTPLSGNSSR	S10	0.984	0.733
Lrrfip2	Leucine-rich repeat flightless-interacting protein 2	A0A1L1SSC0	S277	GSGDTSSLIDPDTSLSELR	S2	1.062	0.610
Lrrfip2	Leucine-rich repeat flightless-interacting protein 2	A0A1L1SSC0	S277	RGSGDTSSLIDPDTSLSELR	S3	0.930	0.173
Lrrfip2	Leucine-rich repeat flightless-interacting protein 2	A0A1L1SSC0	S263,T/S	NSASATTPLSGNSSRRGSGDTSSLIDPDTSLSELR	S4,T/S	0.949	0.364
Lrrfip2	Leucine-rich repeat flightless-interacting protein 2	A0A1L1SSC0	S269,S/T	NSASATTPLSGNSSR	S10,S/T	0.976	0.587
Lrrfip2	Leucine-rich repeat flightless-interacting protein 2	A0A1L1SSC0	Ambiguous	NSASATTPLSGNSSR	S/T	0.976	0.825
Lrrfip2	Leucine-rich repeat flightless-interacting protein 2	A0A1L1SSC0	Ambiguous	QYAENYTRPSSR	S/Y/T	0.952	0.387
Lrrfip2	Leucine-rich repeat flightless-interacting protein 2	A0A1L1SSC0	Ambiguous	SDKQYAENYTRPSSR	T/S/Y	1.197	0.235
Lrrfip2	Leucine-rich repeat flightless-interacting protein 2	A0A1L1SSC0	Ambiguous	NSASATTPLSGNSSR	S/T	0.932	0.085
Lrrk1	Leucine-rich repeat serine/threonine-protein kinase 1	Q3UHC2	S1068	VTIYSFTGSQR	S9	0.879	0.634
Lrrk2	Leucine-rich repeat serine/threonine-protein kinase 2	Q5S006	S910	SNSISVGEVYR	S3	1.069	0.649
Lsm11	U7 snRNA-associated Sm-like protein LSm11	Q8BUV6	S281	SVPSSLQASAR	S3	1.054	0.243
Lsm11	U7 snRNA-associated Sm-like protein LSm11	Q8BUV6	S15,S21	AGSPASPPSPR	S3,S9	1.061	0.370
Lsm12	Protein LSM12 homolog	Q9D0R8	Ambiguous	SQAQPQKEAALSS	S	0.978	0.711
Lsm14a	Protein LSM14 homolog A	Q8K2F8	S183	SSPQLDPLR	S2	0.951	0.463
Lsm14a	Protein LSM14 homolog A	Q8K2F8	S183	SSPQLDPLRK	S2	0.985	0.858
Lsm14a	Protein LSM14 homolog A	Q8K2F8	S216	SPVPARPLPPTSQK	S1	1.044	0.796
Lsm14a	Protein LSM14 homolog A	Q8K2F8	S216	RSPVPARPLPPTSQK	S2	0.992	0.926
Lsp1	Lymphocyte-specific protein 1	P19973	S152	VHLEESNLSYREPDPEDAVGGSGEAEEHLIR	S22	1.223	0.199
Lsp1	Lymphocyte-specific protein 1	P19973	S195	LADRTESLNR	S7	1.010	0.850
Lsp1	Lymphocyte-specific protein 1	P19973	S243	QPSIELPSMAVASTK	S3	1.048	0.314
Lsp1	Lymphocyte-specific protein 1	P19973	S273	TPSCQDIVAGDMSK	S3	1.098	0.100
Lsp1	Lymphocyte-specific protein 1	P19973	S328	VLVDEGSAP	S7	1.123	0.487
Lsp1	Lymphocyte-specific protein 1	P19973	T235	LQQYTQATESSGRTPK	T14	0.870	0.017
Lsr	Lipolysis-stimulated lipoprotein receptor	Q99KG5	S436	ARSVDALDDINRPGSTESGR	S3	1.109	0.387
Ltn1	E3 ubiquitin-protein ligase listerin	Q6A009	Ambiguous	SMSSEGENSEGSDGGAQSPLSNTSSDLVSPLR	S/T	0.973	0.804
Luc7l	Putative RNA-binding protein Luc7-like 1	Q9CYI4	S336	SLREESWEYGR	S6	0.943	0.335
Luc7l3	Luc7-like protein 3	Q5SUF2	S431	NEVNGTSEDIKSEGDTQSN	S18	1.033	0.559
Luc7l3	Luc7-like protein 3	Q5SUF2	S431	ESDTKNEVNGTSEDIKSEGDTQSN	S23	1.006	0.896
Luc7l3	Luc7-like protein 3	Q5SUF2	S425,S431	NEVNGTSEDIKSEGDTQSN	S12,S18	1.015	0.815
Luc7l3	Luc7-like protein 3	Q5SUF2	S431,S/T	ESDTKNEVNGTSEDIKSEGDTQSN	S23,S/T	0.999	0.984
Luzp1	Leucine zipper protein 1	Q8R4U7	S982	VGNSGDAPELSPR	S11	1.044	0.205
Luzp1	Leucine zipper protein 1	Q8R4U7	S982	RVGNSGDAPELSPR	S12	1.070	0.157
Luzp1	Leucine zipper protein 1	Q8R4U7	S988	RTQSSLTASEVLTR	S4	0.983	0.798
Ly9	T-lymphocyte surface antigen Ly-9	Q01965	S644	KTAQTPQQDAESPETPTYENFT	S12	1.101	0.327
Ly9	T-lymphocyte surface antigen Ly-9	Q01965	T499	QAEVPAEIPETPTGHGQFSVLSQR	T11	1.106	0.087
Ly9	T-lymphocyte surface antigen Ly-9	Q01965	T637	TAQTPQQDAESPETPTYENFT	T4	0.996	0.963
Ly9	T-lymphocyte surface antigen Ly-9	Q01965	T499,S510	QAEVPAEIPETPTGHGQFSVLSQR	T11,S22	1.117	0.285
Ly9	T-lymphocyte surface antigen Ly-9	Q01965	Ambiguous	HQPTPTSDTSSESSATTEEDDEKTR	T/S	0.994	0.968
Ly9	T-lymphocyte surface antigen Ly-9	Q01965	Ambiguous	EDSNTIYCSVQKPK	Y/S/T	1.047	0.413
Ly9	T-lymphocyte surface antigen Ly-9	Q01965	Ambiguous	HQPTPTSDTSSESSATTEEDDEKTR	S/T	1.019	0.857
Lyn	Tyrosine-protein kinase Lyn	P25911	S187	IRSLDNGGYYISPR	S3	1.060	0.170
Lyn	Tyrosine-protein kinase Lyn	P25911	S19	DNLNDDEVDSKTQPVR	S10	1.060	0.436
Lyn	Tyrosine-protein kinase Lyn	P25911	Y194	SLDNGGYYISPR	Y8	1.003	0.971
Lyn	Tyrosine-protein kinase Lyn	P25911	Y397	VIEDNEYTAR	Y7	1.067	0.051
Lyn	Tyrosine-protein kinase Lyn	P25911	Y473	MENCPDELYDIMK	Y9	1.000	1.000
Lyn	Tyrosine-protein kinase Lyn	P25911	Ambiguous	VIEDNEYTAREGAK	Y/T	0.863	0.390
Lyplal1	Lysophospholipase-like protein 1	E9QLB2	S238	LPGETDGQSE	S9	0.930	0.387
Lysmd1	LysM and putative peptidoglycan-binding domain-containing protein 1	Q9D0E3	S23	ARSYGSLVQSSCSPVR	S3	1.031	0.694
Lysmd1	LysM and putative peptidoglycan-binding domain-containing protein 1	Q9D0E3	S33	SYGSLVQSSCSPVRER	S11	0.960	0.428
Lysmd1	LysM and putative peptidoglycan-binding domain-containing protein 1	Q9D0E3	Ambiguous	KGESGVPEEDTGLYPSSPR	S/T/Y	1.053	0.663
Lysmd2	LysM and putative peptidoglycan-binding domain-containing protein 2	G3X915	Ambiguous	STSEPEEAELSLSLAR	S/T	0.770	0.322
Lyst	Lysosomal-trafficking regulator	P97412	S216	EQVLSDTMSVENSR	S5	0.958	0.584
Lyst	Lysosomal-trafficking regulator	P97412	S216	EQVLSDTMSVENSR	S5	0.785	0.102
Lyst	Lysosomal-trafficking regulator	P97412	S2203	DVGTEPRSDDDSPGDESYPR	S8	0.999	0.995
Lyst	Lysosomal-trafficking regulator	P97412	S866	QQASSDSPCSLR	S7	1.063	0.142
Lyst	Lysosomal-trafficking regulator	P97412	Ambiguous	EEAFISSCESAK	S	1.062	0.228
Lyst	Lysosomal-trafficking regulator	P97412	Ambiguous	VDHKDVGTEPRSDDDSPGDESYPR	T/S/Y	0.931	0.348
M6pr	Cation-dependent mannose-6-phosphate receptor	P24668	S268	GVGDDQLGEESEERDDHLLPM	S11	1.032	0.350
M6pr	Cation-dependent mannose-6-phosphate receptor	P24668	S268	GVGDDQLGEESEERDDHLLPM	S11	0.933	0.421
Macf1	Microtubule-actin cross-linking factor 1	E9PVY8	S2077	SVVGIECSPAESPER	S8	1.051	0.757
Macf1	Microtubule-actin cross-linking factor 1	E9PVY8	S280	LLDAEDVDVPSPDEK	S11	1.168	0.144
Macf1	Microtubule-actin cross-linking factor 1	E9PVY8	S2968	EAAVLSEEELDQEVTIGDEPDSFVK	S6	0.966	0.626
Macf1	Microtubule-actin cross-linking factor 1	E9PVY8	S3085	LNSQESDEEPQISDVPHISK	S6	0.891	0.052
Macf1	Microtubule-actin cross-linking factor 1	E9PVY8	S3887	QGSFSEDVISHK	S3	0.910	0.084
Macf1	Microtubule-actin cross-linking factor 1	E9PVY8	S3887	RQGSFSEDVISHK	S4	1.041	0.505
Macf1	Microtubule-actin cross-linking factor 1	E9PVY8	S4456	AMDATLSPTK	S7	1.039	0.547
Macf1	Microtubule-actin cross-linking factor 1	E9PVY8	S4481	AFLAELEQNSPK	S10	0.887	0.410
Macf1	Microtubule-actin cross-linking factor 1	E9PVY8	S5552	EAEEELAASGGQSPTGEQIPQFQQR	S13	1.003	0.983
Macf1	Microtubule-actin cross-linking factor 1	E9PVY8	S7297	RGSDASDFDLLETQSACSDTSESSAAGGQGSSR	S3	1.134	0.299
Macf1	Microtubule-actin cross-linking factor 1	E9PVY8	Ambiguous	GSDASDFDLLETQSACSDTSESSAAGGQGSSR	S/T	0.982	0.858
Maco1	Macoilin	Q7TQE6	S332	NASGVVNSSPR	S9	1.047	0.383
Madd	MAP kinase-activating death domain protein	A2AGQ4	S819	LASDSDAESDSR	S5	1.014	0.587
Madd	MAP kinase-activating death domain protein	A2AGQ4	Ambiguous	ATLSDSEIETNSATSAIFGK	T/S	1.128	0.459
Maf	Transcription factor Maf	P54843	Ambiguous	ENGSSSDNPSSPEFFM	S	0.675	0.026
Maf1	Repressor of RNA polymerase III transcription MAF1 homolog	Q9D0U6	S75	SQGGEDESPLSDK	S1	1.033	0.485
Maf1	Repressor of RNA polymerase III transcription MAF1 homolog	Q9D0U6	S75	LSKSQGGEDESPLSDK	S4	0.947	0.563
Mafb	Transcription factor MafB	P54841	S315	EAGSTSDSPSSPEFFL	S8	1.045	0.722
Mafk	Transcription factor MafK	Q61827	S25	EAGENAPVLSDDELVSMSVR	S10	1.075	0.719
Maged1	Melanoma-associated antigen D1	Q9QYH6	Ambiguous	AGPGTTYNFPQSPSANEMTNNQPK	S/T/Y	1.029	0.808
Maged2	Maged2 protein	Q9ER67	Ambiguous	APEASEAAATQASPTTQLSETQVLATENK	S/T	0.913	0.686
Magi3	rane associated guanylate kinase, WW and PDZ domain containing 3, isoform C	G5E8T6	S833	IFYGEKQPEDESHQAFSQNGSPR	S21	1.037	0.253

Malt1	Mucosa-associated lymphoid tissue lymphoma translocation protein 1 homolog	Q2TBA3	Ambiguous	QPPTASSGPSTSPPAGATLNR	S/T	1.201	0.569
Maml1	Mastermind-like protein 1	Q6T264	S310	TEFSPAAFEQEQLGSPQVR	S4	0.960	0.625
Maml1	Mastermind-like protein 1	Q6T264	S45	YEAVSPERLELER	S5	1.031	0.395
Maml1	Mastermind-like protein 1	Q6T264	S310,S321	TEFSPAAFEQEQLGSPQVR	S4,S15	1.314	0.033
Man1c1	alpha-1,2-Mannosidase	Q6NXK9	S159	TDESKESQSLVR	S4	1.003	0.951
Man1c1	alpha-1,2-Mannosidase	Q6NXK9	S566	TEAGFSGIQDVYSSNPNHDNR	S14	0.749	0.166
Man2a1	Alpha-mannosidase 2	P27046	S82	DSVINLSESVEDGPR	S9	1.014	0.781
Man2c1	Alpha-mannosidase 2C1	Q91W89	S479	RLSNTDGLPR	S3	0.939	0.041
Mansc1	MANSC domain-containing protein 1	Q9CR33	Ambiguous	ADETSMQLPEEK	S/T	1.101	0.134
Maoa	Amine oxidase [flavin-containing] A	Q64133	S388	VLGSQEALSPVHYEEK	S9	1.102	0.559
Map1a	Microtubule-associated protein 1A	A2ARP8	S1219	CLSPDDSTVK	S3	1.066	0.324
Map1a	Microtubule-associated protein 1A	A2ARP8	S1844	ALGLEESPEEEGK	S7	1.128	0.512
Map1a	Microtubule-associated protein 1A	A2ARP8	T742	ELSSEPRTPPAQK	T8	1.039	0.418
Map1a	Microtubule-associated protein 1A	A2ARP8	Ambiguous	ETSPTRGEPVPAWEGK	T/S	1.014	0.879
Map1b	Microtubule-associated protein 1B	P14873	S1260	SPSLSPSPPSPIEK	S10	1.108	0.455
Map1b	Microtubule-associated protein 1B	P14873	S1293	SVSPGVTQAVVEEHCASPEEK	S3	0.962	0.771
Map1b	Microtubule-associated protein 1B	P14873	S1789	ESSPLYSPGFSDSTSAAK	S3	1.021	0.835
Map1b	Microtubule-associated protein 1B	P14873	S1813	ETAAAHQASSSPPIDAATAEPYGFR	S9	0.905	0.125
Map1b	Microtubule-associated protein 1B	P14873	Ambiguous	ASLSPMDEPVPDSESPVEK	S	0.959	0.882
Map1s	Microtubule-associated protein 1S	Q8C052	S462	ANSQDSLASR	S3	1.026	0.657
Map1s	Microtubule-associated protein 1S	Q8C052	S462	RPVVTTHDLEAPSRANSQDSLASR	S17	0.915	0.331
Map1s	Microtubule-associated protein 1S	Q8C052	S684	KPPPPASPGSSDSSAR	S7	1.070	0.102
Map1s	Microtubule-associated protein 1S	Q8C052	S670,T/S	STSPHDVDLCLVSPCEFSHR	S13,T/S	1.039	0.711
Map1s	Microtubule-associated protein 1S	Q8C052	Ambiguous	STSPHDVDLCLVSPCEFSHR	S/T	1.009	0.853
Map1s	Microtubule-associated protein 1S	Q8C052	Ambiguous	RSTSPHDVDLCLVSPCEFSHR	S/T	0.941	0.228
Map2	Microtubule-associated protein 2	P20357	T1609	SGTSTPTTPGSTAITPGTPPSYSSR	T18	0.970	0.767
Map2k4	Dual specificity mitogen-activated protein kinase kinase 4	P47809	Ambiguous	ILDQMPATPSSPMYVD	S/T/Y	1.052	0.717
Map2k4	Dual specificity mitogen-activated protein kinase kinase 4	P47809	Ambiguous	ILDQMPATPSSPMYVD	S/Y/T	0.999	0.988
Map2k6	Dual specificity mitogen-activated protein kinase kinase 6	P70236	Ambiguous	EAFEQPQTSSTPPRDLDSK	S/T	1.073	0.224
Map3k1	Mitogen-activated protein kinase kinase kinase 1	F8VQ72	S142	SPAGAEPPSAAAPSGR	S1	1.081	0.108
Map3k1	Mitogen-activated protein kinase kinase kinase 1	F8VQ72	S142	DSGARSPAGAEPPSAAAPSGR	S6	1.004	0.932
Map3k1	Mitogen-activated protein kinase kinase kinase 1	F8VQ72	S261	SVKPESPGVR	S6	0.981	0.670
Map3k1	Mitogen-activated protein kinase kinase kinase 1	F8VQ72	S270	RVSPVPFQSGR	S3	0.964	0.763
Map3k1	Mitogen-activated protein kinase kinase kinase 1	F8VQ72	S287	RAPSPDGFSPYSPEETSR	S4	0.925	0.173
Map3k1	Mitogen-activated protein kinase kinase kinase 1	F8VQ72	S287	RAPSPDGFSPYSPEETSRR	S4	0.968	0.630
Map3k1	Mitogen-activated protein kinase kinase kinase 1	F8VQ72	S518	AVQQPSSPQQPVAGSQR	S7	1.074	0.314
Map3k1	Mitogen-activated protein kinase kinase kinase 1	F8VQ72	S913	GLSATRLSASSEDISDR	S8	1.163	0.159
Map3k1	Mitogen-activated protein kinase kinase kinase 1	F8VQ72	S915	LSASSEDISDR	S4	0.977	0.354
Map3k1	Mitogen-activated protein kinase kinase kinase 1	F8VQ72	S999	IPSASPQTQR	S5	0.996	0.911
Map3k1	Mitogen-activated protein kinase kinase kinase 1	F8VQ72	T1095	CDDSFGGGGNSGNAVIPSEETVFTPVEDK	T24	1.588	0.065
Map3k1	Mitogen-activated protein kinase kinase kinase 1	F8VQ72	Ambiguous	SHDFYSHELSSPVESPTSLR	S/Y/T	1.045	0.813
Map3k11	Mitogen-activated protein kinase kinase kinase 11	Q80XI6	S508	ITVQASPGLDR	S6	0.931	0.051
Map3k11	Mitogen-activated protein kinase kinase kinase 11	Q80XI6	S508	ITVQASPGLDRR	S6	1.039	0.504
Map3k11	Mitogen-activated protein kinase kinase kinase 11	Q80XI6	S525	NVFEVGAGDSPTFPR	S10	1.027	0.839
Map3k11	Mitogen-activated protein kinase kinase kinase 11	Q80XI6	S549	AIQLEPTESGQTWGRQSPR	S17	1.075	0.467
Map3k11	Mitogen-activated protein kinase kinase kinase 11	Q80XI6	S743	TVSPPPGISR	S3	1.055	0.292
Map3k15	Mitogen-activated protein kinase kinase kinase 15	A2AQW0	S73	SESSQGAAAGGGPEAGALK	S3	0.980	0.500
Map3k2	Mitogen-activated protein kinase kinase kinase 2	G5E8L8	S153	RLSVVGPPNR	S3	1.005	0.953
Map3k2	Mitogen-activated protein kinase kinase kinase 2	G5E8L8	S239	AQSYPDNHQEFTDYDNPIFEK	S3	1.002	0.986
Map3k2	Mitogen-activated protein kinase kinase kinase 2	G5E8L8	S331	RGSDIDNPTLTVTDISPPSR	S3	1.071	0.230
Map3k2	Mitogen-activated protein kinase kinase kinase 2	G5E8L8	S347	GSDIDNPTLTVTDISPPSR	S18	1.015	0.823
Map3k2	Mitogen-activated protein kinase kinase kinase 2	G5E8L8	S349	GSDIDNPTLTVTDISPPSRSPR	S20	1.068	0.500
Map3k2	Mitogen-activated protein kinase kinase kinase 2	G5E8L8	S331,S344	RRGSDIDNPTLTVTDISPPSR	S4,S17	0.917	0.635
Map3k2	Mitogen-activated protein kinase kinase kinase 2	G5E8L8	S331,S344	RGSDIDNPTLTVTDISPPSR	S3,S16	0.858	0.528
Map3k2	Mitogen-activated protein kinase kinase kinase 2	G5E8L8	S344,S/T	RGSDIDNPTLTVTDISPPSRSPR	S16,S/T	0.832	0.358
Map3k2	Mitogen-activated protein kinase kinase kinase 2	G5E8L8	S344,S349	GSDIDNPTLTVTDISPPSRSPR	S15,S20	0.889	0.157
Map3k2	Mitogen-activated protein kinase kinase kinase 2	G5E8L8	Ambiguous	RGSDIDNPTLTVTDISPPSRSPR	S/T	0.930	0.452
Map3k2	Mitogen-activated protein kinase kinase kinase 2	G5E8L8	Ambiguous	DRSSPPPGYIPDELHQIAR	S/Y	1.045	0.354
Map3k20	Mitogen-activated protein kinase kinase kinase 20	Q9ESL4	S634	YQQITPSINPSR	S11	1.023	0.710
Map3k20	Mitogen-activated protein kinase kinase kinase 20	Q9ESL4	S668	DSGFSSLNDSSSERGR	S12	0.974	0.854
Map3k20	Mitogen-activated protein kinase kinase kinase 20	Q9ESL4	S668	DSGFSSLNDSSSER	S12	1.051	0.396
Map3k20	Mitogen-activated protein kinase kinase kinase 20	Q9ESL4	S687	GSVSLNSSPK	S4	1.000	0.994
Map3k20	Mitogen-activated protein kinase kinase kinase 20	Q9ESL4	Ambiguous	SSSPTQYGLSR	S/T/Y	0.899	0.050
Map3k20	Mitogen-activated protein kinase kinase kinase 20	Q9ESL4	Ambiguous	SDSSADCQWLDTLR	S/T	1.642	0.093
Map3k3	Mitogen-activated protein kinase kinase kinase 3	Q61084	S147	IKPSQSAGDINTIYQAPEPR	S6	1.007	0.868
Map3k3	Mitogen-activated protein kinase kinase kinase 3	Q61084	S176	HLSVSSQNPGRSSPPPGYVPER	S13	0.999	0.989
Map3k3	Mitogen-activated protein kinase kinase kinase 3	Q61084	S176	SSPPPGYVPER	S2	0.993	0.816
Map3k3	Mitogen-activated protein kinase kinase kinase 3	Q61084	S237	SADSPSFR	S4	0.986	0.801
Map3k3	Mitogen-activated protein kinase kinase kinase 3	Q61084	S237	SADSPSFRK	S4	0.977	0.806
Map3k3	Mitogen-activated protein kinase kinase kinase 3	Q61084	S250	ARSFPDNR	S3	1.032	0.614
Map3k3	Mitogen-activated protein kinase kinase kinase 3	Q61084	S316	SLSTNGENMGVAVQYLDPR	S3	1.008	0.947
Map3k3	Mitogen-activated protein kinase kinase kinase 3	Q61084	S337	SADSENALTVQER	S1	1.033	0.389
Map3k3	Mitogen-activated protein kinase kinase kinase 3	Q61084	S337	LRSADSENALTVQER	S3	1.150	0.038
Map3k3	Mitogen-activated protein kinase kinase kinase 3	Q61084	S166,S168	HLSVSSQNPGRSSPPPGYVPER	S3,S5	0.994	0.912
Map3k3	Mitogen-activated protein kinase kinase kinase 3	Q61084	S337,S340	LRSADSENALTVQER	S3,S6	1.099	0.236
Map3k4	Mitogen-activated protein kinase kinase kinase 4	O08648	S492	LESEEDSIGWGTADCGPEASR	S3	1.440	0.107
Map3k4	Mitogen-activated protein kinase kinase kinase 4	O08648	S449,T/S	ELESGTEESDEEPTPSPR	S4,T/S	1.104	0.564
Map3k4	Mitogen-activated protein kinase kinase kinase 4	O08648	Ambiguous	HSSPTEERDEPAYPR	S/T/Y	1.046	0.344
Map3k4	Mitogen-activated protein kinase kinase kinase 4	O08648	Ambiguous	ELESGTEESDEEPTPSPR	T/S	1.010	0.903
Map3k5	Mitogen-activated protein kinase kinase kinase 5	O35099	Ambiguous	LSALSTGSNEYLR	S/T/Y	1.000	1.000
Map3k6	Mitogen-activated protein kinase kinase kinase 6	A0A0A0MQ82	S965	CLSYGDTSQLR	S3	1.024	0.590
Map3k7	Mitogen-activated protein kinase kinase kinase 7	Q923A8	S389	MSADMSEIEAR	S2	0.953	0.473
Map3k7	Mitogen-activated protein kinase kinase kinase 7	Q923A8	S389	MSADMSEIEAR	S2	1.068	0.694
Map3k7	Mitogen-activated protein kinase kinase kinase 7	Q923A8	S389	MSADMSEIEAR	S2	1.031	0.556
Map3k7	Mitogen-activated protein kinase kinase kinase 7	Q923A8	S389	RMSADMSEIEAR	S3	0.920	0.127
Map3k7	Mitogen-activated protein kinase kinase kinase 7	Q923A8	S389	RMSADMSEIEAR	S3	1.043	0.735
Map3k7	Mitogen-activated protein kinase kinase kinase 7	Q923A8	S389	RMSADMSEIEAR	S3	1.011	0.883
Map3k7	Mitogen-activated protein kinase kinase kinase 7	Q923A8	S439	SIQDLTVTGTEPGQVSSR	S1	1.117	0.089
Map3k7	Mitogen-activated protein kinase kinase kinase 7	Q923A8	S439	RRSIQDLTVTGTEPGQVSSR	S3	0.951	0.553
Map3k7	Mitogen-activated protein kinase kinase kinase 7	Q923A8	S439,S/T	SIQDLTVTGTEPGQVSSR	S1,S/T	1.041	0.258
Map3k7	Mitogen-activated protein kinase kinase kinase 7	Q923A8	Ambiguous	QQSESGRLSLGASR	S	1.195	0.021
Map3k8	Mitogen-activated protein kinase kinase kinase 8	Q07174	S141	NIGSGFVPR	S4	1.022	0.729
Map3k9	Mitogen-activated protein kinase kinase kinase	A0A1Y7VKX4	S545,S/T	SSPPASPTIIPR	S6,S/T	1.075	0.460
Map4	Microtubule-associated protein 4	P27546	S254	ALETMAEQTTDVVHSPSTDTTPGPDTEAALAK	S15	1.033	0.788
Map4	Microtubule-associated protein 4	P27546	S345	DVAPPMEEEIVPGNDTTSPKETETTLPIK	S18	0.773	0.018
Map4	Microtubule-associated protein 4	P27546	S345	DVAPPMEEEIVPGNDTTSPK	S18	0.811	0.043
Map4	Microtubule-associated protein 4	P27546	S345	DVAPPMEEEIVPGNDTTSPK	S18	1.048	0.543
Map4	Microtubule-associated protein 4	P27546	S475	DMSPLPESEVTLGK	S3	1.060	0.352
Map4	Microtubule-associated protein 4	P27546	S475	DMSPLPESEVTLGK	S3	1.051	0.605
Map4	Microtubule-associated protein 4	P27546	S506	VAEFNNVTPLSEEEVTSVK	S11	0.974	0.770
Map4	Microtubule-associated protein 4	P27546	S517	DMSPSAETEAPLAK	S3	1.041	0.476
Map4	Microtubule-associated protein 4	P27546	S517	DMSPSAETEAPLAK	S3	1.082	0.157
Map4	Microtubule-associated protein 4	P27546	S598	GQSTVPPCTASPEPVK	S11	1.072	0.120
Map4	Microtubule-associated protein 4	P27546	S630	ETPGSQPSEPCSGVSR	S5	0.945	0.425
Map4	Microtubule-associated protein 4	P27546; E9PZ43	S785;S632	ATSPSTLVSTGPSSR	S3	1.083	0.041
Map4	Microtubule-associated protein 4	P27546; E9PZ43	S828;S675	SASADLSR	S3	0.941	0.295
Map4	Microtubule-associated protein 4	P27546; E9PZ43	S914;S761	VGSTENIK	S3	1.005	0.978
Map4	Microtubule-associated protein 4	P27546; E9PZ43	S914;S761	SKVGSTENIK	S5	1.045	0.643
Map4	Microtubule-associated protein 4	P27546; E9PZ43	Ambiguous	NTTPTGAAPPAGMTSTR	T/S	0.925	0.139
Map4	Microtubule-associated protein 4	P27546; E9PZ43	Ambiguous	RNTTPTGAAPPAGMTSTR	T/S	0.884	0.160
Map4	Microtubule-associated protein 4	P27546; E9PZ43	Ambiguous	RNTTPTGAAPPAGMTSTR	T/S	1.038	0.581
Map4	Microtubule-associated protein 4	P27546; E9PZ43	Ambiguous	NTTPTGAAPPAGMTSTR	T/S	1.034	0.585
Map4	Microtubule-associated protein 4	P27546	Ambiguous	DVAPPMEEEIVPGNDTTSPKETETTLPIK	T/S	1.022	0.868
Map4k1	Mitogen-activated protein kinase kinase kinase kinase 1	P70218	S419	SPSDDGSGGIRDDGQLSPGVLVR	S17	1.085	0.016
Map4k1	Mitogen-activated protein kinase kinase kinase kinase 1	P70218	Ambiguous	LYPPAHFGSTSPR	S/T/Y	1.228	0.309
Map4k2	Mitogen-activated protein kinase kinase kinase kinase 2	Q61161	S328	TPSEIQFHQVK	S3	1.000	1.000
Map4k4	Mitogen-activated protein kinase kinase kinase kinase 4	A0A0A6YW53	S611	SHSFSDPSPK	S3	1.059	0.423
Map4k4	Mitogen-activated protein kinase kinase kinase kinase 4	A0A0A6YW53; A0A0A6YWM8; F8VPL5	S676;S599;S630	TTSRSPVLSR	S5	0.952	0.250
Map4k4	Mitogen-activated protein kinase kinase kinase kinase 4	A0A0A6YW53; A0A0A6YWM8; F8VPL5	S684;S607;S638	RDSPLQGGGQQNSQAGQR	S3	0.776	0.036
Map4k4	Mitogen-activated protein kinase kinase kinase kinase 4	A0A0A6YW53; A0A0A6YWM8	S701;S624	NSTSSIEPR	S2	1.103	0.307
Map4k4	Mitogen-activated protein kinase kinase kinase kinase 4	A0A0A6YW53; A0A0A6YWM8	S701;S624	DSPLQGGGQQNSQAGQRNSTSSIEPR	S19	1.060	0.620
Map4k4	Mitogen-activated protein kinase kinase kinase kinase 4	A0A0A6YW53; A0A0A6YWM8; F8VPL5	S850;S770;S800	AASSPNLSNGETESVK	S4	0.982	0.610
Map4k4	Mitogen-activated protein kinase kinase kinase kinase 4	A0A0A6YW53; F8VPL5	S891;S841	QTQSASSTLQK	S4	1.028	0.448
Map4k4	Mitogen-activated protein kinase kinase kinase kinase 4	A0A0A6YW53; A0A0A6YWM8; F8VPL5	S904;S888;S854	SSSSFTPFIDPR	S4	0.952	0.567
Map4k4	Mitogen-activated protein kinase kinase kinase kinase 4	A0A0A6YW53	S616,S	SHSFSDPSPK	S8,S	1.033	0.336
Map4k4	Mitogen-activated protein kinase kinase kinase kinase 4	A0A0A6YW53; A0A0A6YWM8	S704,S/T;S627,S/T	DSPLQGGGQQNSQAGQRNSTSSIEPR	S22,S/T	0.918	0.355
Map4k4	Mitogen-activated protein kinase kinase kinase kinase 4	A0A0A6YWM8	T879	LTANETQSASSTLQK	T12	1.043	0.671
Map4k4	Mitogen-activated protein kinase kinase kinase kinase 4	F8VPL5	S655	DSPLQGGGQQNSQAGQRNSTSIEPR	S19	1.056	0.363
Map4k4	Mitogen-activated protein kinase kinase kinase kinase 4	A0A0A6YW53; A0A0A6YWM8	Ambiguous	RDSPLQGGGQQNSQAGQRNSTSSIEPR	T/S	0.949	0.723
Map4k5	Mitogen-activated protein kinase kinase kinase kinase	E9PX30	S433	QSSPSCVPVAETSSSIGNGDGISK	S2	1.193	0.280
Map4k5	Mitogen-activated protein kinase kinase kinase kinase	E9PX30	T400	VNTYPEDSLPDEEK	T3	1.012	0.923
Map7d1	MAP7 domain-containing protein 1	A2AJI0	S118	SSQPSPTTVPASDSPPAKQDVK	S5	0.978	0.724
Map7d1	MAP7 domain-containing protein 1	A2AJI0	S127	SSQPSPTTVPASDSPPAK	S14	1.072	0.189
Map7d1	MAP7 domain-containing protein 1	A2AJI0	S275	SSATLWNSPSR	S1	1.086	0.408
Map7d1	MAP7 domain-containing protein 1	A2AJI0	S315	SRSAVTLPR	S3	0.991	0.876
Map7d1	MAP7 domain-containing protein 1	A2AJI0	S368	SASASPLTPCSAPR	S3	0.911	0.430
Map7d1	MAP7 domain-containing protein 1	A2AJI0	S401	KPGAGGSPALAR	S7	0.951	0.328
Map7d1	MAP7 domain-containing protein 1	A2AJI0	S454	LSTGSELSPK	S2	1.053	0.499

Map7d1	MAP7 domain-containing protein 1	A2AJI0	S519	ESPSPSGPEDK	S4	0.909	0.351
Map7d1	MAP7 domain-containing protein 1	A2AJI0	S544	AAEEKEPAAPASPAPSPVPSPTPAQPQK	S12	1.265	0.069
Map7d1	MAP7 domain-containing protein 1	A2AJI0	S115,S118	RSSQPSPTTVPASDSPPAK	S3,S6	1.069	0.187
Map7d1	MAP7 domain-containing protein 1	A2AJI0	S118,S/T	RSSQPSPTTVPASDSPPAKQDVK	S6,S/T	0.934	0.428
Map7d1	MAP7 domain-containing protein 1	A2AJI0	S118,S127	SSQPSPTTVPASDSPPAK	S5,S14	1.046	0.282
Map7d1	MAP7 domain-containing protein 1	A2AJI0	S118,S127	SSQPSPTTVPASDSPPAKQDVK	S5,S14	0.957	0.393
Map7d1	MAP7 domain-containing protein 1	A2AJI0	S460,S/T	LSTGSELSPK	S8,S/T	1.053	0.570
Map7d1	MAP7 domain-containing protein 1	A2AJI0	S544,S548	AAEEKEPAAPASPAPSPVPSPTPAQPQK	S12,S16	1.139	0.045
Map7d1	MAP7 domain-containing protein 1	A2AJI0	S95,T99	SRGPTPTATGPR	S1,T5	0.953	0.552
Map7d1	MAP7 domain-containing protein 1	A2AJI0	S115,S118,S127	SSQPSPTTVPASDSPPAKQDVK	S2,S5,S14	0.964	0.478
Map7d1	MAP7 domain-containing protein 1	A2AJI0	S118,S127,S/T	RSSQPSPTTVPASDSPPAKQDVK	S6,S15,S/T	1.007	0.909
Map7d1	MAP7 domain-containing protein 1	A2AJI0	S544,S548,S/T	AAEEKEPAAPASPAPSPVPSPTPAQPQK	S12,S16,S/T	1.104	0.215
Map7d1	MAP7 domain-containing protein 1	A2AJI0	Ambiguous	RSSQPSPTTVPASDSPPAK	S/T	1.090	0.072
Map7d1	MAP7 domain-containing protein 1	A2AJI0	Ambiguous	QLPLEPGNPTGQISPQPAPPQEECPSSEAK	T/S	1.135	0.183
Mapk1	Mitogen-activated protein kinase 1	P63085	T183	VADPDHDHTGFLTEYVATR	T13	1.848	0.000
Mapk1	Mitogen-activated protein kinase 1	P63085	T183,Y185	VADPDHDHTGFLTEYVATR	T13,Y15	1.119	0.456
Mapk14	Mitogen-activated protein kinase 14	P47811	Y182	HTDDEMTGYVATR	Y9	1.104	0.022
Mapk14	Mitogen-activated protein kinase 14	P47811	Y182	HTDDEMTGYVATR	Y9	1.096	0.046
Mapk14	Mitogen-activated protein kinase 14	P47811	T180,Y182	HTDDEMTGYVATR	T7,Y9	1.126	0.469
Mapk1ip1	MAPK-interacting and spindle-stabilizing protein	Q9D7G9	Ambiguous	MNEIPGGSPSDSSNPESTLESTGQK	S/T	0.832	0.162
Mapk3	Mitogen-activated protein kinase	D3Z3G6	Y205	IADPEHDHTGFLTEYVATR	Y15	1.353	0.001
Mapk3	Mitogen-activated protein kinase	D3Z3G6	T203,Y205	IADPEHDHTGFLTEYVATR	T13,Y15	1.544	0.026
Mapk6	Mitogen-activated protein kinase 6	Q61532	S189	GHLSEGLVTK	S4	0.910	0.508
Mapk6	Mitogen-activated protein kinase 6	Q61532	S386	ALSDVTDEEEVQVDPR	S3	1.132	0.562
Mapk6	Mitogen-activated protein kinase 6	Q61532	Ambiguous	SSPQIPHK	S	0.889	0.319
Mapk8	Mitogen-activated protein kinase	A6P3E4	S377	GQPSPLAQVQQ	S4	1.033	0.199
Mapk8ip3	C-Jun-amino-terminal kinase-interacting protein 3	K3W4S4	S373,S374	TGSSPTQGIVNK	S3,S4	1.005	0.935
Mapk8ip3	C-Jun-amino-terminal kinase-interacting protein 3	K3W4S4	Ambiguous	TGSSPTQGIVNK	S/T	0.946	0.236
Mapk9	Mitogen-activated protein kinase 9	Q9WTU6	Y185	TACTNFMMTPYVVTR	Y11	1.108	0.382
Mapkapk2	MAP kinase-activated protein kinase 2	P49138	T320	VPQTPLHTSR	T4	1.287	0.008
Mapkbp1	Mitogen-activated protein kinase-binding protein 1	A0A2R8VK23	S1258	SISVGENPGLATEPQAPVPIR	S3	1.000	1.000
Mapkbp1	Mitogen-activated protein kinase-binding protein 1	A0A2R8VK23	S888	GPSQDSLAVSPAGPGK	S3	1.024	0.521
Mapre3	Microtubule-associated protein RP/EB family member 3	D3Z6G3	Ambiguous	QGQDVAPPPNPVPQRTSPTGPK	T/S	0.986	0.590
March1	E3 ubiquitin-protein ligase MARCH1	D3YVY0	S71	SQSRLSVCPSTQDICR	S6	1.064	0.352
March6	E3 ubiquitin-protein ligase MARCH6	Q6ZQ89	T901	QGPSTPPPVSSQE	T5	1.230	0.246
March7	E3 ubiquitin-protein ligase MARCH7	Q9WV66	S312	SLSSENYISPR	S3	0.873	0.009
March7	E3 ubiquitin-protein ligase MARCH7	Q9WV66	Ambiguous	TLQAHMEDLETSEDEF	T/S	1.022	0.892
Marcks	Myristoylated alanine-rich C-kinase substrate	P26645	S138	AEDGAAPSPSSETPKK	S8	0.985	0.651
Marcks	Myristoylated alanine-rich C-kinase substrate	P26645	S27	GEATAERPGEAAVASSPSK	S16	1.099	0.142
Marcks	Myristoylated alanine-rich C-kinase substrate	P26645	S46	VNGDASPAAAEPGAK	S6	1.101	0.312
Marcks	Myristoylated alanine-rich C-kinase substrate	P26645	T143	AEDGAAPSPSSETPK	T13	0.988	0.824
Marcks	Myristoylated alanine-rich C-kinase substrate	P26645	S138,S/T	AEDGAAPSPSSETPKK	S8,S/T	0.944	0.599
Marcks	Myristoylated alanine-rich C-kinase substrate	P26645	S138,T143	AEDGAAPSPSSETPKK	S8,T13	0.961	0.586
Marcks	Myristoylated alanine-rich C-kinase substrate	P26645	S138,T143	AEDGAAPSPSSETPK	S8,T13	1.025	0.714
Marcks	Myristoylated alanine-rich C-kinase substrate	P26645	Ambiguous	EAAEAEPAEPSSPAAEAEGASASSTSSPK	S/T	1.267	0.060
Marcks	Myristoylated alanine-rich C-kinase substrate	P26645	Ambiguous	AEDGAAPSPSSETPKK	S/T	0.929	0.435
Marcks	Myristoylated alanine-rich C-kinase substrate	P26645	Ambiguous	GEATAERPGEAAVASSPSKANGQENGHVK	S/T	0.997	0.957
Marcksl1	MARCKS-related protein	P28667	S135	EGGGDSSASSPTEEEQEQGEMSACSDEGTAQEGK	S25	1.117	0.109
Marcksl1	MARCKS-related protein	P28667	S22	GDVTAEEAAGASPAKANGQENGHVR	S12	1.410	0.111
Marcksl1	MARCKS-related protein	P28667	S22	GDVTAEEAAGASPAK	S12	1.041	0.435
Marcksl1	MARCKS-related protein	P28667	S36	SNGDLTPK	S1	1.075	0.060
Marcksl1	MARCKS-related protein	P28667	S48	GEGESPPVNGTDEAAGATGDAIEPAPPSQEAEAK	S5	1.037	0.687
Marcksl1	MARCKS-related protein	P28667	T148	AAATPESQEPQAK	T4	1.031	0.676
Marcksl1	MARCKS-related protein	P28667	T85	GEVAPKETPK	T8	0.906	0.213
Marcksl1	MARCKS-related protein	P28667	S116,S/T	KEGGGDSSASSPTEEEQEQGEMSACSDEGTAQEGK	S7,S/T	1.042	0.710
Marcksl1	MARCKS-related protein	P28667	Ambiguous	EGDTEEEAGPQAAEPSTPSGPESGPTPASAEQNE	T/S	0.784	0.070
Marcksl1	MARCKS-related protein	P28667	Ambiguous	EGGGDSSASSPTEEEQEQGEMSACSDEGTAQEGK	S/T	1.224	0.472
Mark2	Non-specific serine/threonine protein kinase	A0A0R4J0B1	S40	GRNSATSADEQPHIGNYR	S4	1.067	0.277
Mark2	Non-specific serine/threonine protein kinase	A0A0R4J0B1	S408	SSDQAVPAIPTSNSYSK	S1	0.990	0.893
Mark2	Non-specific serine/threonine protein kinase	A0A0R4J0B1	S43	NSATSADEQPHIGNYR	S5	0.999	0.985
Mark2	Non-specific serine/threonine protein kinase	A0A0R4J0B1	S453	VPASPLPGLDR	S4	1.029	0.445
Mark2	Non-specific serine/threonine protein kinase	A0A0R4J0B1	S468	TTPAPSTNSVLSTSTNR	S6	1.007	0.914
Mark2	Non-specific serine/threonine protein kinase	A0A0R4J0B1	S483	SRNSPLLDR	S4	1.031	0.287
Mark2	Non-specific serine/threonine protein kinase	A0A0R4J0B1	S514	DSTAPQRVPVASPSAHNISSSSGAPDR	S14	0.970	0.812
Mark2	Non-specific serine/threonine protein kinase	A0A0R4J0B1	S514	VPVASPSAHNISSSSGAPDR	S7	1.013	0.606
Mark2	Non-specific serine/threonine protein kinase	A0A0R4J0B1	S562	DQQNLPYGVTPASPSGHSQGR	S13	0.981	0.680
Mark2	Non-specific serine/threonine protein kinase	A0A0R4J0B1	T539	STFHAGQLR	T2	0.988	0.877
Mark2	Non-specific serine/threonine protein kinase	A0A0R4J0B1	Ambiguous	RSSDQAVPAIPTSNSYSK	S/T/Y	1.134	0.012
Mark3	Non-specific serine/threonine protein kinase	A0A1Y7VNZ6	S376	SAELDASDSSSSSNLSLAK	S9	0.961	0.297
Mark3	Non-specific serine/threonine protein kinase	A0A1Y7VNZ6	S419	RYSDHAGPAIPSVVAYPK	S3	0.989	0.772
Mark3	Non-specific serine/threonine protein kinase	A0A1Y7VNZ6	S469	GIAPASPMLGNAGNPNKADIPER	S6	1.025	0.700
Mark3	Non-specific serine/threonine protein kinase	A0A1Y7VNZ6	S593	TPVASTHSISSATTPDR	S5	1.033	0.793
Mark3	Non-specific serine/threonine protein kinase	A0A1Y7VNZ6	S636	TATYNGPPASPSLSHEATPLSQTR	S10	0.963	0.558
Mark3	Non-specific serine/threonine protein kinase	A0A1Y7VNZ6	S383,S	SAELDASDSSSSSNLSLAK	S16,S	0.775	0.294
Mark3	Non-specific serine/threonine protein kinase	A0A1Y7VNZ6	S593,S596	TPVASTHSISSATTPDR	S5,S8	0.961	0.687
Mark3	Non-specific serine/threonine protein kinase	A0A1Y7VNZ6	S598,S/T	TPVASTHSISSATTPDRIR	S10,S/T	0.938	0.285
Mark4	MAP/microtubule affinity-regulating kinase 4	Q8CIP4	S438	RSPTSTGDTELKEER	S2	1.114	0.359
Mark4	MAP/microtubule affinity-regulating kinase 4	Q8CIP4	S545	VPPASPSSHSLAPPSGER	S7	0.999	0.968
Mast2	Microtubule-associated serine/threonine-protein kinase 2	E9Q1Q1	S1037	LLSGDSIEK	S3	0.865	0.017
Mast2	Microtubule-associated serine/threonine-protein kinase 2	E9Q1Q1	S1037	LLSGDSIEKR	S3	1.056	0.540
Mast2	Microtubule-associated serine/threonine-protein kinase 2	E9Q1Q1	S1385	LHLSPPLGR	S4	0.970	0.844
Mast2	Microtubule-associated serine/threonine-protein kinase 2	E9Q1Q1	S1403	SAEPPRSPLLK	S7	0.978	0.745
Mast2	Microtubule-associated serine/threonine-protein kinase 2	E9Q1Q1	S93	QLSQDDCK	S3	1.078	0.085
Mast2	Microtubule-associated serine/threonine-protein kinase 2	E9Q1Q1	S93	QLSQDDCKLR	S3	0.958	0.577
Mast3	Microtubule-associated serine/threonine-protein kinase 3	E9Q8S5	S168	SSESVVDEDGGRSPR	S13	1.119	0.044
Mast3	Microtubule-associated serine/threonine-protein kinase 3	E9Q8S5	S702	HLGSEDDETNDEESSTEIPQFSSCSHR	S4	0.929	0.729
Mast3	Microtubule-associated serine/threonine-protein kinase 3	E9Q8S5	S732	VYSSSEFLAVQPTPTFAER	S4	0.887	0.384
Matr3	Matrin-3	Q8K310	S164	SATREPPYRVPR	S1	0.999	0.986
Matr3	Matrin-3	Q8K310	S164	SATREPPYR	S1	1.028	0.808
Matr3	Matrin-3	Q8K310	S188	RDSFDDRGPSLNPVLDYDHGSR	S3	1.017	0.860
Matr3	Matrin-3	Q8K310	S195	DSFDDRGPSLNPVLDYDHGSR	S9	1.015	0.914
Matr3	Matrin-3	Q8K310	S598	SYSPDGKESPSDKK	S3	1.037	0.866
Matr3	Matrin-3	Q8K310	S619	TDAQKTESPAEGKEQEEK	S8	0.940	0.423
Matr3	Matrin-3	Q8K310	S598,S604	SYSPDGKESPSDKK	S3,S9	1.006	0.957
Matr3	Matrin-3	Q8K310	Ambiguous	SYSPDGKESPSDK	S/Y	1.081	0.733
Mavs	Mitochondrial antiviral-signaling protein	Q8VCF0	S152	ETPSCPKPVQDTQPPESPVENSEQLLQTNSGAVAR	S17	1.205	0.112
Mavs	Mitochondrial antiviral-signaling protein	Q8VCF0	S186	MSGGSLIPSPNQQALSPQPSR	S16	1.064	0.691
Max	Protein max	P28574	Ambiguous	GGTISAFDGGSDSSSESEPEEPQSR	S/T	0.783	0.116
Mbd1	Methyl-CpG-binding domain protein 1	A0A286YDI1	S564	AADPDLSPVKQEPPGPEEDGEEK	S7	1.161	0.278
Mbd2	Methyl-CpG-binding domain protein 2	Q9Z2E1	S184	SDVYYFSPSGK	S7	0.830	0.124
Mbd2	Methyl-CpG-binding domain protein 2	Q9Z2E1	S410	AADTEEVDIDMDSGDEA	S13	1.027	0.378
Mbd2	Methyl-CpG-binding domain protein 2	Q9Z2E1	S410	AADTEEVDIDMDSGDEA	S13	1.038	0.615
Mbd3	Methyl-CpG-binding domain protein 3	Q9Z2D8	S85	VRYDSSNQVK	S5	1.087	0.196
Mbd3	Methyl-CpG-binding domain protein 3	Q9Z2D8	S85	YDSSNQVK	S3	1.101	0.164
Mbd5	Methyl-CpG-binding domain protein 5	B1AYB5	S246	TDPLGSPDVFTR	S6	0.876	0.424
Mbip	MAP3K12-binding inhibitory protein 1	Q99LQ1	S22	SLEQCSSPLLTR	S7	1.046	0.390
Mbp	Myelin basic protein	P04370	S112	DRPSESDELQTIQEDPTAASGGLDVMASQK	S4	1.167	0.138
Mcat	Malonyl-CoA-acyl carrier protein transacylase, mitochondrial	Q8R3F5	S41	DSSVAEEGAQEAVAR	S3	0.969	0.527
Mcf2l	Guanine nucleotide exchange factor DBS	E9PXE2	Ambiguous	TSSTGEEEESLAILR	T/S	0.941	0.687
Mcl1	Induced myeloid leukemia cell differentiation protein Mcl-1 homolog	P97287	T144	SSGADGSLPSTPPPPEEEEDDLYR	T11	1.186	0.001
Mcl1	Induced myeloid leukemia cell differentiation protein Mcl-1 homolog	P97287	S140,S143	SSGADGSLPSTPPPPEEEEDDLYR	S7,S10	0.966	0.737
Mcm10	Protein MCM10 homolog	Q0VBD2	Ambiguous	SSTPSPQAVPGNK	S/T	0.973	0.815
Mcm2	DNA replication licensing factor MCM2	P97310	S139	GLLYDSSEEDEERPARK	S6	1.028	0.898
Mcm2	DNA replication licensing factor MCM2	P97310	S139	GLLYDSSEEDEERPAR	S6	0.979	0.317
Mcm2	DNA replication licensing factor MCM2	P97310	S21	RISDPLTSSPGR	S3	1.075	0.293
Mcm2	DNA replication licensing factor MCM2	P97310	S27	ISDPLTSSPGR	S8	0.966	0.354
Mcm2	DNA replication licensing factor MCM2	P97310	S40	ADALTSSPGR	S6	1.124	0.011
Mcm2	DNA replication licensing factor MCM2	P97310	S139,S140	GLLYDSSEEDEERPAR	S6,S7	1.010	0.799
Mcm2	DNA replication licensing factor MCM2	P97310	S21,T/S	RRISDPLTSSPGR	S4,T/S	1.155	0.411
Mcm2	DNA replication licensing factor MCM2	P97310	S21,T25	RISDPLTSSPGR	S3,T7	1.049	0.192
Mcm2	DNA replication licensing factor MCM2	P97310	S26,S27	ISDPLTSSPGR	S7,S8	1.157	0.111
Mcm2	DNA replication licensing factor MCM2	P97310	S32,S/T	ISDPLTSSPGRSSR	S13,S/T	1.003	0.941
Mcm2	DNA replication licensing factor MCM2	P97310	Ambiguous	RADALTSSPGR	S/T	1.181	0.018
Mcm3	DNA replication licensing factor MCM3	P25206	S672	ASEDESDLEDEEEKSQEDTEQK	S6	0.960	0.213
Mcm3	DNA replication licensing factor MCM3	P25206	S672	ASEDESDLEDEEEK	S6	1.040	0.470
Mcm3	DNA replication licensing factor MCM3	P25206	S672	ASEDESDLEDEEEKSQEDTEQKR	S6	0.981	0.785
Mcm3	DNA replication licensing factor MCM3	P25206	S672	KASEDESDLEDEEEKSQEDTEQK	S7	0.981	0.783
Mcm3	DNA replication licensing factor MCM3	P25206	S732	TDDSQETQDSQKVELSEPR	S4	1.061	0.364
Mcm3	DNA replication licensing factor MCM3	P25206	S738	TDDSQEKTDDSQETQDSQKVELSEPR	S17	1.029	0.533
Mcm3	DNA replication licensing factor MCM3	P25206	T719	DGESYDPYDFSEAETQMPQVHTPKTDDSQEK	T22	0.994	0.952
Mcm3	DNA replication licensing factor MCM3	P25206	T719	DGESYDPYDFSEAETQMPQVHTPK	T22	1.037	0.769
Mcm3	DNA replication licensing factor MCM3	P25206	T719	DGESYDPYDFSEAETQMPQVHTPK	T22	0.998	0.960
Mcm3	DNA replication licensing factor MCM3	P25206	T719	DGESYDPYDFSEAETQMPQVHTPKTDDSQEK	T22	0.961	0.632
Mcm3	DNA replication licensing factor MCM3	P25206	S668,S672	KASEDESDLEDEEEKSQEDTEQK	S3,S7	0.947	0.675
Mcm3	DNA replication licensing factor MCM3	P25206	S672,S681	ASEDESDLEDEEEKSQEDTEQKR	S6,S15	0.968	0.646
Mcm3	DNA replication licensing factor MCM3	P25206	T729,T735	TDDSQEKTDDSQETQDSQKVELSEPR	T8,T14	0.879	0.026
Mcm4	DNA replication licensing factor MCM4	P49717	S119	QRPDLGSAR	S7	1.098	0.478
Mcm4	DNA replication licensing factor MCM4	P49717	T19	GRVTPTQSLR	T4	0.892	0.164

Mcm5	DNA helicase	Q52KC3	S315	SFAGSVSPQEEEEFRR	S7	1.023	0.779
Mcm6	DNA replication licensing factor MCM6	P97311	S699	FNGSSEDASQETVSKPSLR	S4	1.028	0.757
Mcm7	DNA replication licensing factor MCM7	Q61881	S314	SDDDVSGAGELSSEELK	S6	1.115	0.399
Mcm9	DNA helicase MCM9	Q2KHI9	S1079	CHSPPATTAPVLGQQR	S3	0.980	0.809
Mcm9	DNA helicase MCM9	Q2KHI9	S1079	DRCHSPPATTAPVLGQQR	S5	0.984	0.925
Mcm9	DNA helicase MCM9	Q2KHI9	S709	SGADSPPGPGLNRPTSCNNSAENR	S5	1.012	0.927
Mcm9	DNA helicase MCM9	Q2KHI9	S885	SAQVEEPEPEGMETPTVK	S1	1.201	0.337
Mcmbp	Mini-chromosome maintenance complex-binding protein	H9KV04	S167	SYEDDEDMDLQPSK	S1	1.030	0.611
Mcmbp	Mini-chromosome maintenance complex-binding protein	H9KV04	S298	VHSPPASLVPR	S3	1.132	0.038
Mcmbp	Mini-chromosome maintenance complex-binding protein	H9KV04	T160	VSPSTSYTPSR	T8	1.001	0.984
Mcoln1	Mucolipin-1	Q99J21	S557	RGSGSACSLFCCCGR	S3	0.963	0.571
Mcoln1	Mucolipin-1	Q99J21	T34	LLTPNPGYGTQVGTSPAPTTPTEEEDLR	T20	0.800	0.169
Mcoln1	Mucolipin-1	Q99J21	T34	LLTPNPGYGTQVGTSPAPTTPTEEEDLRR	T20	1.008	0.929
Mcoln2	Mucolipin-2	Q8K595	S558	RRSNDHLILID	S3	0.972	0.657
Mcph1	Microcephalin	Q7TT79	S260	SANEMTTVTCPSSPVLR	S13	1.047	0.405
Mcph1	Microcephalin	Q7TT79	S260	SANEMTTVTCPSSPVLR	S13	1.034	0.805
Mcph1	Microcephalin	Q7TT79	S273	ASSFYGSASPNHLR	S9	0.991	0.854
Mcph1	Microcephalin	Q7TT79	S288	APDSPSKESINCQK	S4	0.909	0.413
Mcph1	Microcephalin	Q7TT79	S358	AADLGSSPK	S7	1.020	0.864
Mcrip1	Mapk-regulated corepressor-interacting protein 1	Q3UGS4	S21	SPTNSSEIFTPAHEENVR	S1	1.014	0.552
Mcrip1	Mapk-regulated corepressor-interacting protein 1	Q3UGS4	Ambiguous	NSSPRSPTNSSEIFTPAHEENVR	S/T	1.028	0.689
Mcrip2	MAPK regulated corepressor interacting protein 2	Q9CQB2	S82	RPLTTSPSLEGTQETYTVAHEENVR	S8	1.025	0.609
Mcrs1	Microspherule protein 1	Q99L90	Ambiguous	APSTPVPPSPAPTPGLTK	S/T	0.893	0.396
Mdc1	Mediator of DNA damage checkpoint protein 1	E9QK89	S1444	SYSALSEPEPQSSASQSSGASEADSPR	S25	1.111	0.081
Mdc1	Mediator of DNA damage checkpoint protein 1	E9QK89	S157	IRIESQNSR	S5	1.179	0.096
Mdc1	Mediator of DNA damage checkpoint protein 1	E9QK89	S168	VLLAADSEEEGDFPSGR	S7	1.067	0.130
Mdc1	Mediator of DNA damage checkpoint protein 1	E9QK89	S596	SQSGSPAAPVEQVVIHTDTSGDPTLPQR	S5	0.903	0.477
Mdc1	Mediator of DNA damage checkpoint protein 1	E9QK89	T783	EMADRMTPEREPLER	T7	1.342	0.036
Mdc1	Mediator of DNA damage checkpoint protein 1	E9QK89	Ambiguous	SQPQVLVEQSQSASGRDSDTDVEEESSGR	S/T	1.110	0.162
Mdc1	Mediator of DNA damage checkpoint protein 1	E9QK89	Ambiguous	SQPQVLVEQSQSASGRDSDTDVEEESSGR	S/T	0.793	0.396
Mdfic	MyoD family inhibitor domain-containing protein	A0A087WRH9	S110	LGSPAHEACNEDNTEK	S3	1.090	0.365
Mdfic	MyoD family inhibitor domain-containing protein	A0A087WRH9	S211	ISAPVSQK	S6	1.093	0.325
Mdm1	Mdm1 protein	B2RPU6	S566	ASSQDGLETLK	S3	0.992	0.885
Mdm2	E3 ubiquitin-protein ligase Mdm2	P23804	S163	SISETEENTDELPGER	S3	1.026	0.632
Mdn1	Midasin	A2ANY6	S4827	HQDEKEDSEPEDVEQGQEK	S8	1.005	0.949
Mecp2	Methyl-CpG-binding protein 2	Q9Z2D6	S421	GGSLESDGCPKEPAK	S3	0.997	0.972
Mecp2	Methyl-CpG-binding protein 2	Q9Z2D6	S80	AETSESSGSAPAVPEASASPK	S19	0.996	0.946
Med1	Mediator of RNA polymerase II transcription subunit 1	Q925J9	S1435	NYGSPLISGSTPK	S4	1.003	0.927
Med1	Mediator of RNA polymerase II transcription subunit 1	Q925J9	S1465	SPAYTPQNVDSESESGSSIAER	S11	1.063	0.591
Med1	Mediator of RNA polymerase II transcription subunit 1	Q925J9	S1481	SYQNSPSSEDGIRPLPEYSTEK	S5	1.030	0.744
Med1	Mediator of RNA polymerase II transcription subunit 1	Q925J9	S664	DNPAQDFSTLYGSSPLER	S14	0.991	0.971
Med1	Mediator of RNA polymerase II transcription subunit 1	Q925J9	T1442	NYGSPLISGSTPKHER	T11	0.944	0.218
Med1	Mediator of RNA polymerase II transcription subunit 1	Q925J9	T805	LPSTSDDCPPIGTPVR	T13	0.944	0.707
Med1	Mediator of RNA polymerase II transcription subunit 1	Q925J9	S1435,T1442	NYGSPLISGSTPK	S4,T11	1.088	0.548
Med1	Mediator of RNA polymerase II transcription subunit 1	Q925J9	S1435,T1442	NYGSPLISGSTPKHER	S4,T11	1.004	0.927
Med1	Mediator of RNA polymerase II transcription subunit 1	Q925J9	S1481,S/Y/T	SYQNSPSSEDGIRPLPEYSTEK	S5,S/Y/T	0.934	0.263
Med1	Mediator of RNA polymerase II transcription subunit 1	Q925J9	T1051,T1057	SQTPPGVATPPIPK	T3,T9	0.986	0.879
Med1	Mediator of RNA polymerase II transcription subunit 1	Q925J9	T1459,S/Y	GSPSHSKSPAYTPQNVDSESESGSSIAER	T12,S/Y	1.156	0.199
Med1	Mediator of RNA polymerase II transcription subunit 1	Q925J9	Ambiguous	SQTPPGVATPPIPK	S/T	1.034	0.390
Med12	Mediator of RNA polymerase II transcription subunit 12	A2AGH6	S699	GSPSPEKPDVEK	S2	1.111	0.078
Med12	Mediator of RNA polymerase II transcription subunit 12	A2AGH6	Ambiguous	GSGFTVPGGTEELPEEEGGGSSGR	S/T	0.709	0.427
Med12	Mediator of RNA polymerase II transcription subunit 12	A2AGH6	Ambiguous	GSGFTVPGGTEELPEEEGGGSSGRR	S/T	1.187	0.132
Med13	Mediator of RNA polymerase II transcription subunit 13	Q5SWW4	S481	VSVSDEIGMDTDSASQR	S2	0.840	0.033
Med13	Mediator of RNA polymerase II transcription subunit 13	Q5SWW4	T326,T/S	DPAMSSVTLTPPTSPEEVQTVDPQSAQK	T10,T/S	0.999	0.991
Med13	Mediator of RNA polymerase II transcription subunit 13	Q5SWW4	Ambiguous	YVWQGPTSAPILFPVTEEDPILSSFSR	T/S/Y	0.692	0.094
Med13l	Mediator of RNA polymerase II transcription subunit 13	A0A0J9YUA8	S918	MEVEDGLGSPKPEEIK	S9	0.749	0.009
Med13l	Mediator of RNA polymerase II transcription subunit 13	A0A0J9YUA8	T393	SQMSTPTREEEAAHSPAAWDFVDPTQR	T5	1.141	0.149
Med13l	Mediator of RNA polymerase II transcription subunit 13	A0A0J9YUA8	T758	DVTTPGHSTPVPDGK	T9	0.942	0.447
Med14	Mediator of RNA polymerase II transcription subunit 14	A2ABV5	S1117	AGNWPGSPQVSGPSPATR	S7	0.915	0.439
Med14	Mediator of RNA polymerase II transcription subunit 14	A2ABV5	S1117,S1124	AGNWPGSPQVSGPSPATR	S7,S14	1.129	0.095
Med19	Mediator of RNA polymerase II transcription subunit 19	Q8C1S0	S194	TQDPVPPETPSDSDHK	S11	1.154	0.350
Med19	Mediator of RNA polymerase II transcription subunit 19	Q8C1S0	S194	TQDPVPPETPSDSDHKK	S11	0.952	0.700
Med23	Mediator of RNA polymerase II transcription subunit 23	E9QNV2	Ambiguous	MEPAVPPQALNSGSPAPQSNQVPASLPVTQ	S/T	0.821	0.007
Med24	Mediator of RNA polymerase II transcription subunit 24	A6PW47	S879	LLSSSDDDANILSSPTDR	S3	1.307	0.010
Med24	Mediator of RNA polymerase II transcription subunit 24	A6PW47	S879,S890	LLSSSDDDANILSSPTDR	S3,S14	1.134	0.296
Med26	Mediator of RNA polymerase II transcription subunit 26	Q7TN02	S361	AGFSPDSSKADSDATSSGGSDSK	S4	1.014	0.601
Med26	Mediator of RNA polymerase II transcription subunit 26	Q7TN02	S435	ADSPVPTEQLPR	S3	1.005	0.962
Med26	Mediator of RNA polymerase II transcription subunit 26	Q7TN02	S435	EPVRADSPVPTEQLPR	S7	1.046	0.207
Med26	Mediator of RNA polymerase II transcription subunit 26	Q7TN02	S361,S/T	AGFSPDSSKADSDATSSGGSDSK	S4,S/T	0.943	0.482
Mef2a	Myocyte-specific enhancer factor 2A	Q60929	S406	SEPISPPR	S5	1.068	0.237
Mef2a	Myocyte-specific enhancer factor 2A	Q60929	S406	SEPISPPRDR	S5	0.971	0.514
Mef2a	Myocyte-specific enhancer factor 2A	Q60929	S444	QEMGRSPVDSLSSSSSSYDGSDR	S6	1.146	0.413
Mef2a	Myocyte-specific enhancer factor 2A	Q60929	S459	SPVDSLSSSSSSYDGSDREDPR	S16	1.033	0.628
Mef2a	Myocyte-specific enhancer factor 2A	Q60929	S98	GLNGCESPDADDYFEHSPLSEDR	S7	1.090	0.551
Mef2a	Myocyte-specific enhancer factor 2A	Q60929	T413	MTPSGFQQQQQPQQQPPPQPPQPQPR	T2	0.703	0.037
Mef2a	Myocyte-specific enhancer factor 2A	Q60929	T413	DRMTPSGFQQQQQPQQQPPPQPPQPQPR	T4	1.055	0.676
Mef2a	Myocyte-specific enhancer factor 2A	Q60929	S98,S108	GLNGCESPDADDYFEHSPLSEDR	S7,S17	1.205	0.177
Mef2c	Myocyte-specific enhancer factor 2C	A0A0H2UH28	S118	GSESPDPDSSYALTPR	S4	0.805	0.019
Mef2c	Myocyte-specific enhancer factor 2C	A0A0H2UH28	S118,T128	GSESPDPDSSYALTPR	S4,T14	0.987	0.875
Mef2c	Myocyte-specific enhancer factor 2C	A0A0H2UH28	S118,T128	ENKGSESPDPDSSYALTPR	S7,T17	1.039	0.729
Mef2c	Myocyte-specific enhancer factor 2C	Q8CFN5; A0A0H2UH28	S222;S240	NSPGLLVSPGNLNK	S2	0.892	0.503
Mef2c	Myocyte-specific enhancer factor 2C	Q8CFN5; A0A0H2UH28	S240;S258	SPPPMNLGMNNR	S1	1.201	0.004
Mef2c	Myocyte-specific enhancer factor 2C	Q8CFN5; A0A0H2UH28	S396;S406	SEPVSPPRDR	S5	1.027	0.477
Mef2c	Myocyte-specific enhancer factor 2C	Q8CFN5	S98	GLNGCDSPDPDADDSVGHSPESEDKYR	S7	1.201	0.596
Mef2c	Myocyte-specific enhancer factor 2C	Q8CFN5	S98,S110	GLNGCDSPDPDADDSVGHSPESEDKYR	S7,S19	1.162	0.585
Mef2d	Myocyte-specific enhancer factor 2D	Q63943	S180	LLSPQQPALQR	S3	1.015	0.780
Mef2d	Myocyte-specific enhancer factor 2D	Q63943	S98	GFNGCDSPEPDGEDSLEQSPLLEDK	S7	1.082	0.205
Mefv	Mefv protein	Q32MT0	S241	SLEITTYSR	S1	0.942	0.478
Melk	Maternal embryonic leucine zipper kinase	Q61846	S490	TPGNSAGADTLTTGVISPERR	S17	1.261	0.231
Melk	Maternal embryonic leucine zipper kinase	Q61846	S497	SMDVDLNQAHMEDTPK	S1	1.043	0.309
Melk	Maternal embryonic leucine zipper kinase	Q61846	S497	CRSMDVDLNQAHMEDTPK	S3	0.980	0.711
Melk	Maternal embryonic leucine zipper kinase	Q61846	S521	GTNVFGSLER	S7	1.043	0.337
Men1	Menin	A0A0R4J1I3	S550	GGQEVGNAAQAPAPAASPPPEGPVLTFQSEK	S17	0.980	0.789
Mepce	7SK snRNA methylphosphate capping enzyme	Q8K3A9	S126	RNSCNVGGGSGGSFK	S3	1.400	0.002
Mepce	7SK snRNA methylphosphate capping enzyme	Q8K3A9	T188	ALNAETPK	T6	1.006	0.907
Mepce	7SK snRNA methylphosphate capping enzyme	Q8K3A9	T485	LQAQTSEGDPGTEGEEGTITVR	T12	1.037	0.094
Mertk	Tyrosine-protein kinase Mer	Q60805	S538	FGGAFSEEDSQLVVNYR	S10	0.994	0.977
Mettl16	U6 small nuclear RNA (adenine-(43)-N(6))-methyltransferase	Q5SW15	T503	LLCQEETPEATEDERDEER	T11	1.000	0.993
Mettl16	U6 small nuclear RNA (adenine-(43)-N(6))-methyltransferase	Q5SW15	T503	LLCQEETPEATEDERDEERGGMEAMESCK	T11	0.986	0.911
Mettl16	U6 small nuclear RNA (adenine-(43)-N(6))-methyltransferase	Q5SW15	T499,T503	LLCQEETPEATEDERDEER	T7,T11	0.982	0.777
Mettl18	Histidine protein methyltransferase 1 homolog	Q9CZ09	S67	CSSLGNAASSEDTGSPPPIADR	S15	0.987	0.890
Mettl2	Methyltransferase-like protein 2	Q8BMK1	S140	CSCASPGCETQVPPLEEPVTQK	S5	0.968	0.646
Mettl22	Methyltransferase-like protein 22	Q8R1C6	S118	AASDPNPAEPAR	S3	1.038	0.330
Mettl3	N6-adenosine-methyltransferase subunit METTL3	A0A0R4J041	S43	NPEAALSPTFR	S7	1.232	0.039
Mettl3	N6-adenosine-methyltransferase subunit METTL3	A0A0R4J041	Ambiguous	SDSPVPTAPTSSGPKPSTTSVAPELATDPELEK	S/T	0.905	0.592
Mex3d	RNA-binding protein MEX3D	D3YTR3	Ambiguous	GDNALGAASTPEPFYVGSR	S/T/Y	0.923	0.313
Mfap1b	Microfibrillar-associated protein 1B	C0HKD9	S94	LQNRISEDVEER	S6	1.037	0.478
Mfap1b	Microfibrillar-associated protein 1B	C0HKD9	S94	RLQNRISEDVEER	S7	1.059	0.264
Mfap1b	Microfibrillar-associated protein 1B	C0HKD9	Ambiguous	AKEQEAEPEEQEEDSSSDPR	S	1.091	0.529
Mfap1b	Microfibrillar-associated protein 1B	C0HKD9	Ambiguous	EQEAEPEEQEEDSSSDPR	S	0.909	0.546
Mfap1b	Microfibrillar-associated protein 1B	C0HKD9	Ambiguous	IVEPEVVGESDSEVEGDAWR	S	1.104	0.801
Mfap3l	Microfibrillar-associated protein 3-like	Q9D3X9	Ambiguous	SESPTADSDASSLHEQPQQIAIK	S/T	0.890	0.118
Mff	Mitochondrial fission factor	Q6PCP5	S131	SMSENAVR	S3	1.087	0.015
Mff	Mitochondrial fission factor	Q6PCP5	S146	NDSIVTPSPPQAR	S3	0.989	0.928
Mfsd5	Molybdate-anion transporter	Q921Y4	S440	VPSPTGEPYAPEL	S3	1.070	0.260
Mfsd5	Molybdate-anion transporter	Q921Y4	S440	HDAELRVPSPTGEPYAPEL	S9	1.123	0.337
Mfsd6	Major facilitator superfamily domain-containing protein 6	D3Z183	T11	VAILTDDEEEQK	T5	1.111	0.579
Mga	MAX gene-associated protein	E9QLG3	S2519	LGSDEFCVSPR	S9	0.988	0.873
Mgrn1	E3 ubiquitin-protein ligase MGRN1	Q9D074	Ambiguous	ESSSPESFGTEEGDEPSLK	S/T	1.050	0.158
Mia2	Melanoma inhibitory activity protein 2	A0A1W2P711	S1125	EHSPYGPSPLGRPPSETR	S15	1.037	0.388
Mia2	Melanoma inhibitory activity protein 2	A0A1W2P711	S1155	GSRGPENLLDHQMNTER	S2	1.124	0.172
Mia2	Melanoma inhibitory activity protein 2	A0A1W2P711	S1155	GSRGPENLLDHQMNTER	S2	0.916	0.212
Mia2	Melanoma inhibitory activity protein 2	A0A1W2P711	S1179	GPENLLDHQMNTERGESSYDRLSDAPR	S23	1.059	0.657
Mia2	Melanoma inhibitory activity protein 2	A0A1W2P711	S1179	GESSYDRLSDAPR	S9	1.085	0.089
Mia2	Melanoma inhibitory activity protein 2	A0A1W2P711	S1229	LSGPAELR	S2	1.034	0.656
Mia2	Melanoma inhibitory activity protein 2	A0A1W2P711	S1229	FYPNSGRLSGPAELR	S9	0.997	0.949
Mia2	Melanoma inhibitory activity protein 2	A0A1W2P711	S1229	QDRFYPNSGRLSGPAELR	S12	0.990	0.956
Mia2	Melanoma inhibitory activity protein 2	A0A1W2P711	S1272	DNLGNSNVPDSPIPAECEAAGR	S11	1.229	0.134
Mia2	Melanoma inhibitory activity protein 2	A0A1W2P711	S1118,S1125	EHSPYGPSPLGRPPSETR	S8,S15	1.037	0.490
Mia2	Melanoma inhibitory activity protein 2	A0A1W2P711	S1189,S1191	APSDRSLSPPWEQDRR	S6,S8	0.960	0.630
Mia2	Melanoma inhibitory activity protein 2	A0A1W2P711	Ambiguous	SLSPPWEQDRR	S	1.000	1.000
Mia3	Transport and Golgi organization protein 1 homolog	Q8BI84	S1458	LECELESEDPDKGGNESDDLANGETGGDR	S17	1.306	0.050
Mia3	Transport and Golgi organization protein 1 homolog	Q8BI84	S1733	SEFGSLDR	S5	1.073	0.124
Mia3	Transport and Golgi organization protein 1 homolog	Q8BI84	S1770	SSSPAKAVDEGK	S3	0.987	0.787
Mia3	Transport and Golgi organization protein 1 homolog	Q8BI84	S1915	EEAKPASPSSVQDR	S7	1.009	0.908
Mia3	Transport and Golgi organization protein 1 homolog	Q8BI84	S1915	DSLPSGPREEAKPASPSSVQDR	S15	0.967	0.592
Mia3	Transport and Golgi organization protein 1 homolog	Q8BI84	S659	TGFPEEESREDGTDAEQAR	S8	1.063	0.238
Mia3	Transport and Golgi organization protein 1 homolog	Q8BI84	S820	DETAAQTPGSGEAVLSK	S10	1.023	0.822

Mia3	Transport and Golgi organization protein 1 homolog	Q8BI84	T1929	SQASKPTP	T7	1.059	0.445
Mia3	Transport and Golgi organization protein 1 homolog	Q8BI84	Ambiguous	KPQPAAGYTDSEDEGDDLFVEEPK	S/T/Y	0.833	0.373
Mia3	Transport and Golgi organization protein 1 homolog	Q8BI84	Ambiguous	TKQPANMILETGQESETTSEEAGDVGK	S/T	1.133	0.159
Mia3	Transport and Golgi organization protein 1 homolog	Q8BI84	Ambiguous	HSASDPGPAPVVNSSSR	S	0.984	0.797
Mia3	Transport and Golgi organization protein 1 homolog	Q8BI84	Ambiguous	QPANMILETGQESETTSEEAGDVGK	T/S	1.023	0.760
Mia3	Transport and Golgi organization protein 1 homolog	Q8BI84	Ambiguous	HSASDPGPAPVVNSSSRSSSPAKAVDEGK	S	0.913	0.199
Mia3	Transport and Golgi organization protein 1 homolog	Q8BI84	Ambiguous	HSASDPGPAPVVNSSSRSSSPAK	S	1.000	0.996
Mia3	Transport and Golgi organization protein 1 homolog	Q8BI84	Ambiguous	HSASDPGPAPVVNSSSRSSSPAK	S	1.114	0.338
Mib2	E3 ubiquitin-protein ligase MIB2	Q8R516	S251	RVSADGQPFQR	S3	1.017	0.695
Mical1	[F-actin]-monooxygenase MICAL1	Q8VDP3	S770	EADNNGSLESQELPTPGDSNMQPDPSSPPVTR	S27	1.116	0.511
Mical1	[F-actin]-monooxygenase MICAL1	Q8VDP3	S777	VSPVPSPSQPAR	S2	1.047	0.230
Mical1	[F-actin]-monooxygenase MICAL1	Q8VDP3	S777	VSPVPSPSQPARR	S2	0.971	0.256
Mical1	[F-actin]-monooxygenase MICAL1	Q8VDP3	S777,S781	VSPVPSPSQPAR	S2,S6	1.017	0.762
Mical2	[F-actin]-monooxygenase MICAL2	Q8BML1	S515	RESDIRPNK	S3	0.897	0.229
Mical3	[F-actin]-monooxygenase MICAL3	Q8CJ19	S1154	GTSQVSSPSQPPEK	S7	1.096	0.338
Mical3	[F-actin]-monooxygenase MICAL3	Q8CJ19	S1783	RQLSFSEDSDLSSDDILER	S4	1.023	0.900
Micall1	MICAL-like protein 1	Q8BGT6	S128	DPAPPSPTSTSPAVQPGEEAQGDDLSPDSLSEQGK	S6	0.970	0.927
Micall1	MICAL-like protein 1	Q8BGT6	S319	SSLQQDGTVEQSVSSGLVNGR	S1	0.931	0.494
Micall1	MICAL-like protein 1	Q8BGT6	S496	APSASPLAIHASR	S5	1.137	0.334
Micall1	MICAL-like protein 1	Q8BGT6	S647	KPSPSASPTVR	S3	1.113	0.050
Micall1	MICAL-like protein 1	Q8BGT6	S494,S496	APSASPLAIHASR	S3,S5	0.963	0.515
Micall1	MICAL-like protein 1	Q8BGT6	S647,S/T	KPSPSASPTVR	S3,S/T	1.026	0.520
Micall1	MICAL-like protein 1	Q8BGT6	T311,T314	KASESSALTPPTPRPR	T9,T12	1.009	0.913
Micall1	MICAL-like protein 1	Q8BGT6	Ambiguous	QCSSTLVPGSYSSGPEEGTFVCAER	S/T/Y	0.894	0.753
Micall1	MICAL-like protein 1	Q8BGT6	Ambiguous	ENPFNRKPSPSASPTVR	S/T	1.042	0.368
Micall2	MICAL-like protein 2	Q3TN34	S766,S768	RKPCPAGSGPSPAALSPSPSHR	S16,S18	1.002	0.983
Micall2	MICAL-like protein 2	Q3TN34	Ambiguous	RPSSDSTEELSGK	S/T	0.997	0.864
Micall2	MICAL-like protein 2	Q3TN34	Ambiguous	SAVLDSGLAPPGEAVTSPVR	T/S	0.983	0.788
Mid1ip1	Mid1-interacting protein 1	Q9CQ20	S74	TTPAPSPGSANESFFAPSR	S9	0.830	0.339
Mief1	Mitochondrial dynamics protein MID51	Q8BGV8	S59	AISAPTSPTR	S7	1.031	0.563
Mier1	Mesoderm induction early response protein 1	Q5UAK0	S368	LLDESESAASSR	S7	0.994	0.919
Mier1	Mesoderm induction early response protein 1	Q5UAK0	S376	APSPPPTASNSSNSQSEKEDGAVSSR	S3	1.085	0.150
Mier1	Mesoderm induction early response protein 1	Q5UAK0	S376,T380	APSPPPTASNSSNSQSEKEDGAVSSR	S3,T7	1.011	0.871
Mier2	Mesoderm induction early response protein 2	Q3U3N0	Ambiguous	FLAGDNGPGSSASSDTEEDALPANK	S/T	0.872	0.407
Mier3	Mesoderm induction early response protein 3	Q3UHF3	S156	SNTTCDGDKESEIEDVETDSGNSPEDLRR	S11	0.993	0.826
Mif4gd	MIF4G domain-containing protein	Q3UBZ5	S221	YYYSEVSD	S7	1.047	0.783
Miga2	Mitoguardin 2	Q8BK03	T206	GDGGSTPTPGDSLQNPDTASEALSEPESQRR	T6	1.012	0.903
Miga2	Mitoguardin 2	Q8BK03	T206,S228	GDGGSTPTPGDSLQNPDTASEALSEPESQRR	T6,S28	0.954	0.687
Miga2	Mitoguardin 2	Q8BK03	Ambiguous	TLMLPLTEGSLR	T/S	0.686	0.137
Miga2	Mitoguardin 2	Q8BK03	Ambiguous	GDGGSTPTPGDSLQNPDTASEALSEPESQR	T/S	0.970	0.834
Milr1	Allergin-1	Q3TB92	S190	GSGDAPAQDELYVNACK	S2	1.045	0.313
Milr1	Allergin-1	Q3TB92	S242	EMAPMEEEGGTDGKADYIYSELTH	S20	1.022	0.896
Milr1	Allergin-1	Q3TB92	T218	TQTEQPQEIHYATPVFK	T13	0.764	0.133
Milr1	Allergin-1	Q3TB92	Y241	ADYIYSELTH	Y5	1.018	0.848
Milr1	Allergin-1	Q3TB92	Ambiguous	TQREDQSKGSGDAPAQDELYVNACK	S/T/Y	0.985	0.764
Milr1	Allergin-1	Q3TB92	Ambiguous	EDQSKGSGDAPAQDELYVNACK	S/Y	1.012	0.838
Mindy1	Ubiquitin carboxyl-terminal hydrolase MINDY-1	Q76LS9	S103	VETAEVCSRPQELPQSPR	S16	1.269	0.008
Mindy1	Ubiquitin carboxyl-terminal hydrolase MINDY-1	Q76LS9	S440	TRAPSPQGR	S5	1.080	0.267
Mindy2	Ubiquitin carboxyl-terminal hydrolase MINDY-2	Q6PDI6	S111	VTASPELAEAAAGRGSGPVGDTGTCR	S16	1.016	0.836
Mindy2	Ubiquitin carboxyl-terminal hydrolase MINDY-2	Q6PDI6	S99	VTASPELAEAAAGR	S4	0.996	0.951
Mindy2	Ubiquitin carboxyl-terminal hydrolase MINDY-2	Q6PDI6	Ambiguous	SSLGDPTSPSQLGCGAGSDLK	S/T	1.020	0.907
Mindy4	Probable ubiquitin carboxyl-terminal hydrolase MINDY-4	Q3UQI9	Ambiguous	LRSVSEDSPLGYSHTEGNSR	S/Y/T	1.072	0.539
Mink1	Misshapen-like kinase 1	G3X9G2	S537	QQNSPLAK	S4	0.960	0.606
Mink1	Misshapen-like kinase 1	G3X9G2	S603	SQSLQDQPTR	S3	1.026	0.500
Mink1	Misshapen-like kinase 1	G3X9G2	S644	QNSDPTSEGPGPSPNPPSWVRPDNEAPPKVPQR	S3	1.126	0.139
Mink1	Misshapen-like kinase 1	G3X9G2	S644	QNSDPTSEGPGPSPNPPSWVRPDNEAPPK	S3	1.007	0.934
Mink1	Misshapen-like kinase 1	G3X9G2	S704	SNSAWQIYLQR	S3	1.045	0.764
Mink1	Misshapen-like kinase 1	G3X9G2	S766	SDSVLPASHGHLPQAGSLER	S17	1.015	0.895
Mink1	Misshapen-like kinase 1	G3X9G2	S783	LDSSPVLSPGNK	S4	0.933	0.135
Mink1	Misshapen-like kinase 1	G3X9G2	S939	GQSPPTKDGGSDYQSR	S3	1.027	0.795
Mink1	Misshapen-like kinase 1	G3X9G2	S783,S787	LDSSPVLSPGNK	S4,S8	1.090	0.128
Mink1	Misshapen-like kinase 1	G3X9G2	Ambiguous	GTPKPPGPPAQPPGPPNASSNPDLRR	S/T	1.036	0.573
Mink1	Misshapen-like kinase 1	G3X9G2	Ambiguous	GTPKPPGPPAQPPGPPNASSNPDLR	S/T	1.007	0.879
Mios	GATOR complex protein MIOS	Q8VE19	S766	GFSQYGVSGSPTK	S10	0.966	0.477
Misp	Mitotic interactor and substrate of PLK1	Q9D279	S364	SLSSDCILSPDAR	S3	1.049	0.540
Mki67	Proliferation marker protein Ki-67	E9PVX6	S125	RASRDSFCADPDGEGQDTK	S3	1.699	0.001
Mki67	Proliferation marker protein Ki-67	E9PVX6	S128	ASRDSFCADPDGEGQDTK	S5	1.288	0.019
Mki67	Proliferation marker protein Ki-67	E9PVX6	S1587	SPQLGPVR	S1	1.210	0.094
Mki67	Proliferation marker protein Ki-67	E9PVX6	S213	SSPTQSLSNSNEKESPFEK	S2	1.152	0.092
Mki67	Proliferation marker protein Ki-67	E9PVX6	S213	SSPTQSLSNSNEK	S2	1.056	0.308
Mki67	Proliferation marker protein Ki-67	E9PVX6	S2255	LDFAENSSGSK	S8	0.840	0.315
Mki67	Proliferation marker protein Ki-67	E9PVX6	S2333	EGHSPLSK	S4	0.900	0.281
Mki67	Proliferation marker protein Ki-67	E9PVX6	S287	SSGSTPVTAASSPK	S12	1.087	0.594
Mki67	Proliferation marker protein Ki-67	E9PVX6	S2934	SNPLLSPK	S6	1.095	0.768
Mki67	Proliferation marker protein Ki-67	E9PVX6	S2980	HVSPEPVK	S3	1.178	0.050
Mki67	Proliferation marker protein Ki-67	E9PVX6	S2980	RHVSPEPVK	S4	1.098	0.283
Mki67	Proliferation marker protein Ki-67	E9PVX6	S517	ERPQSPGKQESPGITPPR	S5	1.069	0.499
Mki67	Proliferation marker protein Ki-67	E9PVX6	S561	SGNLPAK	S1	1.095	0.236
Mki67	Proliferation marker protein Ki-67	E9PVX6	S588	SGASEANLIVAK	S1	1.053	0.028
Mki67	Proliferation marker protein Ki-67	E9PVX6	S767	YSASPTLR	S4	1.104	0.223
Mki67	Proliferation marker protein Ki-67	E9PVX6	T1159;T3144;T5367	APGTPAPVQEENDSTAFMETPK	T4	1.167	0.087
Mki67	Proliferation marker protein Ki-67	E9PVX6	T1159;T3144;T5367	APGTPAPVQEENDSTAFMETPK	T4	1.683	0.122
Mki67	Proliferation marker protein Ki-67	E9PVX6	Ambiguous	LPSSPPLEPTDTSVTSR	S/T	0.786	0.392
Mknk1	MAP kinase-interacting serine/threonine-protein kinase 1	O08605	T344	GLPTPQVLQR	T4	0.835	0.030
Mknk1	MAP kinase-interacting serine/threonine-protein kinase 1	O08605	T423	DANPCLTPAGL	T7	1.107	0.197
Mknk2	MAP kinase-interacting serine/threonine-protein kinase 2	Q8CDB0	S446	ASLSATPVVLVGDRA	S2	1.082	0.634
Mknk2	MAP kinase-interacting serine/threonine-protein kinase 2	Q8CDB0	S446	QRASLSATPVVLVGDRA	S4	0.935	0.107
Mknk2	MAP kinase-interacting serine/threonine-protein kinase 2	Q8CDB0	T6	TAELQGFHR	T1	0.932	0.271
Mkrn2	Probable E3 ubiquitin-protein ligase makorin-2	Q9ERV1	Ambiguous	VNNPGGCSDPQTSPEMKPHSYLDAIR	S/T/Y	0.990	0.888
Mlf2	Myeloid leukemia factor 2	Q99KX1	S237	LAIQGPEDSPSR	S9	0.989	0.870
Mlh1	DNA mismatch repair protein Mlh1	Q9JK91	Ambiguous	TEGSPERATREDEEMLALPAPAEAAAESENLER	T/S	0.930	0.618
Mllt1	Btk-PH-domain binding protein	Q9ERL0	S261	LESLSPK	S5	0.889	0.417
Mllt1	Btk-PH-domain binding protein	Q9ERL0	S463	SPEPCSKPEK	S1	1.313	0.039
Mllt1	Btk-PH-domain binding protein	Q9ERL0	S463	RSPEPCSKPEK	S2	1.006	0.928
Mllt1	Btk-PH-domain binding protein	Q9ERL0	S347,S349	TLQTEDSNSEDEASFR	S7,S9	1.209	0.346
Mllt10	Protein AF-10	O54826	S436	SFDNSPGELGSSSLPTAGYK	S5	0.927	0.505
Mllt6	Myeloid/lymphoid or mixed-lineage leukemia	B1AR10	S441	APSPGDYK	S3	1.046	0.580
Mlx	MAX-like protein X, isoform CRA_b	A2BFB0	S48	GAPQLSPESPLLSGAR	S9	1.000	1.000
Mlxip	MLX interacting protein, isoform CRA_a	G5E8D8	Ambiguous	DCPNSGQASPCPSEQSPSPQSPQNNCSGK	S	0.933	0.460
Mlxip	MLX interacting protein, isoform CRA_a	G5E8D8	Ambiguous	SVLLKPQVPEDDDDSDTDEPSPPPPSGVATSAR	S/T	0.960	0.782
Mlxip	MLX interacting protein, isoform CRA_a	G5E8D8	Ambiguous	DCPNSGQASPCPSEQSPSPQSPQNNCSGK	S	1.043	0.461
Mmtag2	Multiple myeloma tumor-associated protein 2 homolog	Q99LX5	S232	HSDFSPCSK	S5	0.994	0.838
Mnat1	CDK-activating kinase assembly factor MAT1	P51949	S279	AASPQDLAGGYTSSLACHR	S3	1.040	0.421
Mndal	Myeloid cell nuclear differentiation antigen-like protein	D0QMC3	S170	TEEDCLQNSPKPPPTSPSSSSNK	S9	1.054	0.339
Mndal	Myeloid cell nuclear differentiation antigen-like protein	D0QMC3	S170,S177	TEEDCLQNSPKPPPTSPSSSSNK	S9,S16	1.072	0.475
Mob1b	MOB kinase activator 1B	Q3UDM0	S38	HAEATLGSGNLR	S8	0.988	0.858
Mocs2	Molybdopterin synthase catalytic subunit	A0A0R4J206	Ambiguous	LPSSRQSVEDNASEPSGK	S	1.187	0.217
Mocs3	Adenylyltransferase and sulfurtransferase MOCS3	A2BDX3	S150	RLNSAVECVAYPR	S4	1.273	0.205
Mon1a	Vacuolar fusion protein MON1 homolog A	A0A0R4J0D5	S31	AESPTPGLTQGTEPGAGQEGAMFVHTR	S3	1.093	0.042
Mon1a	Vacuolar fusion protein MON1 homolog A	A0A0R4J0D5	S31	AESPTPGLTQGTEPGAGQEGAMFVHTR	S3	1.096	0.368
Mon1a	Vacuolar fusion protein MON1 homolog A	A0A0R4J0D5	S56	SYEDLTELEDR	S1	1.077	0.306
Mon1a	Vacuolar fusion protein MON1 homolog A	A0A0R4J0D5	S72	EASGDSPKECVGSPPPLATDMR	S6	1.042	0.250
Mon1a	Vacuolar fusion protein MON1 homolog A	A0A0R4J0D5	S79	ECVGSPPPLATDMR	S5	1.082	0.370
Mon1a	Vacuolar fusion protein MON1 homolog A	A0A0R4J0D5	Ambiguous	GAATEPSQEGSTEGEEEDATEAWR	S/T	1.042	0.321
Mon2	Protein MON2 homolog	Q80TL7	S1213	TDSIGEKLGR	S3	0.975	0.916
Mon2	Protein MON2 homolog	Q80TL7	S534	DEQAASDPMDQETVSR	S6	1.054	0.401
Mon2	Protein MON2 homolog	Q80TL7	S534	RDEQAASDPMDQETVSR	S7	1.054	0.455
Mon2	Protein MON2 homolog	Q80TL7	S534	RDEQAASDPMDQETVSR	S7	1.071	0.663
Morc2a	MORC family CW-type zinc finger protein 2A	Q69ZX6	S741	SLAVSDEEEAEEEAEK	S5	0.908	0.106
Morc2a	MORC family CW-type zinc finger protein 2A	Q69ZX6	S741	SLAVSDEEEAEEEAEKR	S5	0.950	0.117
Morc2a	MORC family CW-type zinc finger protein 2A	Q69ZX6	S741	SLAVSDEEEAEEEAEKRR	S5	0.909	0.420
Morc2a	MORC family CW-type zinc finger protein 2A	Q69ZX6	S741	RSLAVSDEEEAEEEAEK	S6	1.042	0.386
Morc2a	MORC family CW-type zinc finger protein 2A	Q69ZX6	S775	KEANELSDSAGEDHPAELR	S7	1.035	0.630
Morc2a	MORC family CW-type zinc finger protein 2A	Q69ZX6	S777	EANELSDSAGEDHPAELR	S8	1.048	0.593
Morc2a	MORC family CW-type zinc finger protein 2A	Q69ZX6	S854	LMKPPSPEHQSPDTQQEGGEEEEAMVAR	S6	1.129	0.774
Morc2a	MORC family CW-type zinc finger protein 2A	Q69ZX6	Ambiguous	EEKKEANELSDSAGEDHPAELR	S	0.964	0.814
Morc3	MORC family CW-type zinc finger protein 3	F7BJB9	S563	RLSNPPVENSSYK	S3	1.341	0.006
Morc3	MORC family CW-type zinc finger protein 3	F7BJB9	Ambiguous	STGQQAAADGSTASNAEEPVSYVDGESLK	S/T/Y	0.908	0.554
Mospd2	Motile sperm domain-containing protein 2	B1AU74	S283	ETLETISNEEPPALSEK	S7	1.162	0.445
Mov10	Putative helicase MOV-10	D3YVL0	S1043	LSPSTSGPR	S2	0.975	0.642
Mphosph8	M-phase phosphoprotein 8	Q3TYA6	S51	GTVAVGDSEEDGEDVFEVER	S8	1.012	0.915
Mphosph8	M-phase phosphoprotein 8	Q3TYA6	Ambiguous	GYTSEDDTWEPEVHLEDCK	T/S/Y	0.883	0.474
Mphosph8	M-phase phosphoprotein 8	Q3TYA6	Ambiguous	CKEETSPEDLR	T/S	1.019	0.748
Mpp1	55 kDa erythrocyte membrane protein	P70290	S52	NRPETSLNQSNVTTEDMYTNGSPAPGSPAHAK	S22	0.997	0.961
Mpp1	55 kDa erythrocyte membrane protein	P70290	S57	NRPETSLNQSNVTTEDMYTNGSPAPGSPAHAK	S27	0.968	0.729
Mpp1	55 kDa erythrocyte membrane protein	P70290	S52,S57	NRPETSLNQSNVTTEDMYTNGSPAPGSPAHAK	S22,S27	0.927	0.214
Mprip	Myosin phosphatase Rho-interacting protein (Fragment)	F6RND9	S160	AEEQLPPLLSPPSPSTPHSR	S13	1.015	0.873
Mprip	Myosin phosphatase Rho-interacting protein (Fragment)	F6RND9	S350	DFASEAPTAPLSDACPLSPHR	S18	1.197	0.237

Mprip	Myosin phosphatase Rho-interacting protein (Fragment)	F6RND9	S350	DFASEAPTAPLSDACPLSPHRR	S18	1.053	0.429
Mprip	Myosin phosphatase Rho-interacting protein (Fragment)	F6RND9	S87	DQPDGTSLSPAQSPSQSQPPAACTPR	S7	1.008	0.964
Mprip	Myosin phosphatase Rho-interacting protein (Fragment)	F6RND9	T86	AKDQPDGTSLSPAQSPSQSQPPAACTPR	T8	1.102	0.602
Mprip	Myosin phosphatase Rho-interacting protein (Fragment)	F6RND9	S173,S184	YGCPGSPSQELSHPLHSPGLPAPSR	S6,S17	1.026	0.800
Mprip	Myosin phosphatase Rho-interacting protein (Fragment)	F6RND9	S93,S/T	AKDQPDGTSLSPAQSPSQSQPPAACTPR	S15,S/T	1.396	0.069
Mrc1	Macrophage mannose receptor 1	Q61830	S1045	SSLSYEDADCVVVIGGNSR	S4	0.944	0.612
Mrc1	Macrophage mannose receptor 1	Q61830	Ambiguous	RSSLSYEDADCVVVIGGNSR	S/Y	0.953	0.438
Mre11	Double-strand break repair protein MRE11	Q61216	S2	MSPTDPLDDEDTFK	S2	0.932	0.267
Mre11	Double-strand break repair protein MRE11	Q61216	S2	MSPTDPLDDEDTFK	S2	0.811	0.321
Mre11	Double-strand break repair protein MRE11	Q61216	S686	GVDFESDEDDDDDPFMSSSCPR	S6	0.974	0.831
Mre11	Double-strand break repair protein MRE11	Q61216	Ambiguous	NYSETIEVDDSDEDDIFPTNSR	T/S/Y	1.056	0.856
Mrgbp	MRG/MORF4L-binding protein	Q9DAT2	S191,S195	VLTANSNPSSPSAAK	S6,S10	1.062	0.391
Mrm3	rRNA methyltransferase 3, mitochondrial	Q5ND52	S42	VLSPSGQVEER	S3	0.972	0.656
Mrnip	MRN complex-interacting protein	Q9D1F5	Ambiguous	TQLSTSAERPSSPAQPR	S/T	1.219	0.135
Mrpl1	39S ribosomal protein L1, mitochondrial	Q99N96	S85	SYTYMESDPEDDVYLK	S7	1.279	0.161
Mrpl12	39S ribosomal protein L12, mitochondrial	Q9DB15	S46	SEALAGAPLDNAPK	S1	0.926	0.504
Mrpl52	39S ribosomal protein L52, mitochondrial	Q9D0Y8	S116	GTLLRSPLPNQ	S6	0.965	0.726
Mrpl55	39S ribosomal protein L55, mitochondrial	Q9CZ83	S112	REEEPEVVDSFDTERYK	S10	0.904	0.550
Mrpl55	39S ribosomal protein L55, mitochondrial	Q9CZ83	S84	MLAMPLDLDALSPEER	S12	1.245	0.263
Mrps2	28S ribosomal protein S2, mitochondrial	Q924T2	S290	GSEGSGTSPVPDKSHSP	S16	0.988	0.940
Mrps26	28S ribosomal protein S26, mitochondrial	Q80ZS3	S183	IEEALDSPK	S7	0.930	0.531
Mrps36	28S ribosomal protein S36, mitochondrial	Q9CQX8	Ambiguous	GSTSPDLLMHQGPPDTAEIIK	S/T	1.089	0.298
Mrtfa	Myocardin-related transcription factor A	Q8K4J6	S351	SLSTSSSPSSGTPGPSGLAR	S3	1.215	0.035
Mrtfa	Myocardin-related transcription factor A	Q8K4J6	S423	AYQDQVSPAPGAPK	S7	0.959	0.830
Mrtfa	Myocardin-related transcription factor A	Q8K4J6	S548	AASCCLSPGAR	S7	1.053	0.350
Mrtfa	Myocardin-related transcription factor A	Q8K4J6	S606	AQQPAPASSPVKR	S9	1.083	0.176
Mrtfa	Myocardin-related transcription factor A	Q8K4J6	T488,S492	FGSTGSTPPVSPTPSER	T7,S11	1.012	0.225
Mrtfa	Myocardin-related transcription factor A	Q8K4J6	Ambiguous	AQQPAPASSPVK	S	0.972	0.726
Mrtfa	Myocardin-related transcription factor A	Q8K4J6	Ambiguous	FGSTGSTPPVSPTPSER	T/S	0.863	0.260
Ms4a6d	MS4a6D protein	Q2TVW7	S232	SSVSSESLCNPTYENILTS	S4	1.096	0.432
Ms4a6d	MS4a6D protein	Q2TVW7	S232,S235	SSVSSESLCNPTYENILTS	S4,S7	1.015	0.964
Ms4a6d	MS4a6D protein	Q2TVW7	Ambiguous	NKSSVSSESLCNPTYENILTS	S/T/Y	0.833	0.562
Msantd2	Myb/SANT-like DNA-binding domain-containing protein 2	Q6NZR2	S27	MEVLSPASPGDLSDGNPSLSDPSTPR	S8	1.088	0.580
Msh3	DNA mismatch repair protein Msh3	A0A087WQ16	Y804	QGNYCRPTLQEEK	Y4	0.755	0.088
Msh6	DNA mismatch repair protein Msh6	P54276	S137	VHVQFFDDSPTR	S9	0.925	0.460
Msh6	DNA mismatch repair protein Msh6	P54276	S63	SAAVSASSPEAK	S8	0.962	0.316
Msh6	DNA mismatch repair protein Msh6	P54276	Ambiguous	QEGSSDDASSGVGDSDSEDLGTFGK	S/T	0.884	0.366
Msl1	Male-specific lethal 1 homolog	Q6PDM1	S127	QAGIGGEPVAAGAGCSPRPK	S16	1.022	0.694
Msl1	Male-specific lethal 1 homolog	Q6PDM1	S207	SPLGGGGGSGASSQAACLK	S1	1.126	0.472
Msl1	Male-specific lethal 1 homolog	Q6PDM1	Ambiguous	EPGPPLASSQGGSPSPSPAGCGGGK	S	1.053	0.661
Msl3	Male-specific lethal 3 homolog	Q9WVG9	S315	SQEELSPSPPLLNPSTPQSTESQPPTGEPATPK	S8	1.023	0.866
Msl3	Male-specific lethal 3 homolog	Q9WVG9	S371	LSESSSSPQPK	S7	0.992	0.847
Msl3	Male-specific lethal 3 homolog	Q9WVG9	S404	SSSPIPLTPSK	S3	1.077	0.256
Msn	Moesin	P26041	S407	EALLQASR	S7	1.031	0.795
Msn	Moesin	P26041	S429	ISQLEMAR	S2	1.007	0.915
Msn	Moesin	P26041	S468	TAMSTPHVAEPAENEHDEQDENGAEASAELR	S4	1.027	0.475
Msn	Moesin	P26041	S491	TAMSTPHVAEPAENEHDEQDENGAEASAELR	S27	1.191	0.155
Msn	Moesin	P26041	S527	ALTSELANAR	S4	1.172	0.145
Msn	Moesin	P26041	S576	IDEFESM	S6	0.899	0.475
Msn	Moesin	P26041	S576	IDEFESM	S6	1.018	0.560
Msn	Moesin	P26041	S576	QRIDEFESM	S8	1.096	0.216
Msr1	Macrophage scavenger receptor types I and II	P30204	S23	LCPHEQEDADCSSESVKFDAR	S13	0.973	0.501
Msr1	Macrophage scavenger receptor types I and II	P30204	S23	LCPHEQEDADCSSESVK	S13	1.019	0.730
Msr1	Macrophage scavenger receptor types I and II	P30204	S32	SMTASLPHSTK	S1	0.994	0.961
Msr1	Macrophage scavenger receptor types I and II	P30204	S22,S	LCPHEQEDADCSSESVKFDAR	S12,S	0.989	0.892
Msr1	Macrophage scavenger receptor types I and II	P30204	S36,S40	SMTASLPHSTK	S5,S9	0.971	0.768
Msr1	Macrophage scavenger receptor types I and II	P30204	S32,T34,S40	SMTASLPHSTK	S1,T3,S9	1.087	0.639
Msr1	Macrophage scavenger receptor types I and II	P30204	S32,S40,T/S	SMTASLPHSTK	S1,S9,S/T	0.964	0.134
Msr1	Macrophage scavenger receptor types I and II	P30204	Ambiguous	SMTASLPHSTK	S/T	1.022	0.857
Msto1	Protein misato homolog 1	E9PUB7	S41	MAEDEESPGELCPDVLYR	S7	1.184	0.460
Msto1	Protein misato homolog 1	E9PUB7	S555	LAQCYQEGDSLSN	S12	1.000	1.000
Mta1	Metastasis-associated protein MTA1	Q8K4B0	S386	ATVVNGTGTPGQSPGAGR	S13	0.988	0.933
Mta1	Metastasis-associated protein MTA1	Q8K4B0	S449	NNMSPHGIPAR	S4	1.046	0.734
Mta1	Metastasis-associated protein MTA1	Q8K4B0	S449	NNMSPHGIPAR	S4	0.971	0.812
Mta1	Metastasis-associated protein MTA1	Q8K4B0	S639	GGSLPPVK	S3	0.878	0.298
Mta2	Metastasis-associated protein MTA2	Q9R190	S435	GHLSRPEAQSLSPYTTSANR	S12	1.053	0.334
Mta2	Metastasis-associated protein MTA2	Q9R190	S54	DISSLNSLADSNAR	S5	1.017	0.574
Mta3	Metastasis-associated protein MTA3	Q924K8	S425	MPTQSDEEKSPSPTAEDPR	S10	1.086	0.556
Mta3	Metastasis-associated protein MTA3	Q924K8	S427	MPTQSDEEKSPSPTAEDPR	S12	1.012	0.860
Mta3	Metastasis-associated protein MTA3	Q924K8	S229	HAELSGSPLK	S7	0.962	0.485
Mta3	Metastasis-associated protein MTA3	Q924K8	S425,S427	MPTQSDEEKSPSPTAEDPR	S10,S12	1.004	0.946
Mtch1	Mitochondrial carrier homolog 1	Q791T5	S382	VSSGSCFALE	S3	0.926	0.273
Mtch1	Mitochondrial carrier homolog 1	Q791T5	Ambiguous	RVSSGSCFALE	S	0.917	0.530
Mtdh	Protein LYRIC	Q80WJ7	S297	LSSQLSEEK	S6	1.414	0.007
Mtdh	Protein LYRIC	Q80WJ7	S423	SQEPISNDQKVSDDDKEK	S12	1.010	0.948
Mtdh	Protein LYRIC	Q80WJ7	S454	QGEDNSHTQDTEDLEK	S6	1.089	0.591
Mtdh	Protein LYRIC	Q80WJ7	S491	TMSTSDPAEVLIK	S3	1.149	0.316
Mtdh	Protein LYRIC	Q80WJ7	S565	SETNWESPK	S7	1.056	0.644
Mtdh	Protein LYRIC	Q80WJ7	T96	EEAAPPTPAPDDLAQLK	T7	1.084	0.369
Mtf1	Metal regulatory transcription factor 1	Q07243	S655	GCSSPPPPEPNPQPPDGPSLQLPP	S4	1.042	0.726
Mtf1	Metal regulatory transcription factor 1	Q07243	Ambiguous	KGCSSPPPPEPNPQPPDGPSLQLPP	S	1.015	0.949
Mtfr1l	Mitochondrial fission regulator 1-like	Q9CWE0	S100	NASVPNLR	S3	0.935	0.298
Mtfr1l	Mitochondrial fission regulator 1-like	Q9CWE0	S220	TTCSSSEEDDCISLSK	S4	1.005	0.966
Mtfr1l	Mitochondrial fission regulator 1-like	Q9CWE0	S235	ASSFADMMGILK	S3	0.958	0.315
Mthfsl	5-formyltetrahydrofolate cyclo-ligase	L7N466	Ambiguous	VDEVLYEDSPAS	S/Y	1.053	0.856
Mtm1	Myotubularin	Q9Z2C5	S13	YNSHSLENESIK	S5	0.981	0.863
Mtm1	Myotubularin	Q9Z2C5	S28	KVSQDGVSQDVSETVPR	S8	1.134	0.128
Mtmr1	Myotubularin-related protein 1	I7HJQ9	Ambiguous	GSSPTHSATPVHTSV	S/T	0.999	0.992
Mtmr10	Myotubularin-related protein 10	Q7TPM9	S763	IWLSTETLANED	S4	0.989	0.954
Mtmr10	Myotubularin-related protein 10	Q7TPM9	Y704	SGPLEACYAELDQSR	Y8	1.012	0.902
Mtmr12	Myotubularin-related protein 12	Q80TA6	S601	LINSSDDLQDSSR	S4	0.993	0.940
Mtmr12	Myotubularin-related protein 12	Q80TA6	S699	QEAVHAEAPGLLRNSVR	S15	0.984	0.437
Mtmr2	Myotubularin-related protein 2	Q9Z2D1	S58	SASAISSDSISTSADNFSPDLR	S18	1.034	0.536
Mtmr2	Myotubularin-related protein 2	Q9Z2D1	S631	ASSPAQCVTPVQTVV	S3	1.051	0.236
Mtmr2	Myotubularin-related protein 2	Q9Z2D1	S9,S	SSSCESLGAQLPAAR	S6,S	1.001	0.988
Mtmr2	Myotubularin-related protein 2	Q9Z2D1	Ambiguous	SSSCESLGAQLPAAR	S	1.013	0.802
Mtmr3	Myotubularin-related protein 3	Q8K296	S613	SFDNLTTTCENMVPLASR	S1	0.868	0.428
Mtmr3	Myotubularin-related protein 3	Q8K296	S613	TRSFDNLTTTCENMVPLASR	S3	0.897	0.254
Mtmr3	Myotubularin-related protein 3	Q8K296	S633	SSDPSLNEK	S2	1.017	0.635
Mtmr3	Myotubularin-related protein 3	Q8K296	S633	RSSDPSLNEK	S3	1.020	0.595
Mtmr3	Myotubularin-related protein 3	Q8K296	Ambiguous	LESQYLTSSLR	S/T/Y	1.469	0.321
Mtmr3	Myotubularin-related protein 3	Q8K296	Ambiguous	FNGDFGDEVTSIPDSESNLDQNCVSR	S/T	1.228	0.405
Mtmr4	Myotubularin-related protein 4	Q91XS1	S610	TRSMDDLLSACDTSSPLTR	S3	1.058	0.504
Mtmr4	Myotubularin-related protein 4	Q91XS1	S610	TRSMDDLLSACDTSSPLTR	S3	1.010	0.933
Mtmr4	Myotubularin-related protein 4	Q91XS1	Ambiguous	TSSDPNLNNHSQEVR	S/T	0.970	0.805
Mtmr6	Myotubularin-related protein 6	L8AZD2	S622	AIEGSSPADNR	S6	1.118	0.106
Mtmr6	Myotubularin-related protein 6	L8AZD2	S645	SEPTVVSLEYGVAR	S7	1.402	0.299
Mtpn	Myotrophin	P62774	T31	GEDVNRTLEGGR	T7	1.070	0.262
Mtss1	Metastasis suppressor 1, isoform CRA_e	G3X9H7	T262	GSDYSWSYQTPPSSPSTTMSR	T10	0.964	0.845
Mtss1	Metastasis suppressor 1, isoform CRA_e	G3X9H7	T262,S266	GSDYSWSYQTPPSSPSTTMSR	T10,S14	0.970	0.693
Mtss1	Metastasis suppressor 1, isoform CRA_e	G3X9H7	T262,S266	GSDYSWSYQTPPSSPSTTMSR	T10,S14	1.009	0.860
Mtus1	Microtubule-associated tumor suppressor 1 homolog	A0A0R4J1L9	S1143	QLSTEQAALQESLEK	S3	1.305	0.028
Mtus1	Microtubule-associated tumor suppressor 1 homolog	A0A0R4J1L9	S1208	NSGSFSSPSISPR	S11	1.086	0.296
Mtus1	Microtubule-associated tumor suppressor 1 homolog	A0A0R4J1L9	S1204,S1208	NSGSFSSPSISPR	S7,S11	0.859	0.214
Mtus1	Microtubule-associated tumor suppressor 1 homolog	A0A0R4J1L9	Ambiguous	DTPSPQVTGGSSPSPGPSK	S/T	1.003	0.957
Mvb12b	Multivesicular body subunit 12B	Q6KAU4	S199,T/S	NHDSSQPTTPSQSSASSTPAPNLPR	S5,T/S	0.812	0.124
Myadm	Myeloid-associated differentiation marker	O35682	S16	TTITTTTSSSTTVGSAR	S9	0.974	0.646
Mybbp1a	Myb-binding protein 1A	Q7TPV4	S1164	DIPSDTQSPVSTKR	S8	1.105	0.009
Mybbp1a	Myb-binding protein 1A	Q7TPV4	S1164	DIPSDTQSPVSTK	S8	1.142	0.111
Mybbp1a	Myb-binding protein 1A	Q7TPV4	S1253	SPAPSNPTLSPSTPAK	S10	1.141	0.168
Mybbp1a	Myb-binding protein 1A	Q7TPV4	S1280	LSQVNGATPVSPIEPESK	S11	1.077	0.371
Mybbp1a	Myb-binding protein 1A	Q7TPV4	S562	NVTSVTSLTTQQR	S7	1.347	0.050
Mybbp1a	Myb-binding protein 1A	Q7TPV4	S6	SPTKAEPATPAEAAQSDR	S1	0.882	0.311
Mybbp1a	Myb-binding protein 1A	Q7TPV4	T14	AEPATPAEAAQSDR	T5	1.164	0.384
Mybbp1a	Myb-binding protein 1A	Q7TPV4	S1160,S1164	DIPSDTQSPVSTK	S4,S8	1.060	0.054
Mybbp1a	Myb-binding protein 1A	Q7TPV4	Ambiguous	SEGTTPEKNAASQQDAVTEGAMPAATGK	T/S	1.103	0.689
Mybbp1a	Myb-binding protein 1A	Q7TPV4	Ambiguous	SPAPSNPTLSPSTPAKTPK	T/S	1.129	0.125
Myc	Myc proto-oncogene protein	B2RSN1	Ambiguous	SCTSSDSTAFSPSSDSLLSSESSPR	S/T	0.936	0.581
Mycbp2	E3 ubiquitin-protein ligase MYCBP2	Q7TPH6	S1621	QVSTENDSTLVHR	S3	1.078	0.430
Mycbp2	E3 ubiquitin-protein ligase MYCBP2	Q7TPH6	S1643	LSQSEENISGMTSFR	S2	0.972	0.835
Mycbp2	E3 ubiquitin-protein ligase MYCBP2	Q7TPH6	S2884	TSSGFRAESPGPGSR	S9	0.967	0.859
Mycbp2	E3 ubiquitin-protein ligase MYCBP2	Q7TPH6	S2943	SKSDSYTLDPDTLR	S3	1.150	0.344
Mycbp2	E3 ubiquitin-protein ligase MYCBP2	Q7TPH6	S3550	SYSVVASEYDK	S3	1.069	0.442
Mycbp2	E3 ubiquitin-protein ligase MYCBP2	Q7TPH6	S3550	SYSVVASEYDKQHSILPAR	S3	1.088	0.511
Mycbp2	E3 ubiquitin-protein ligase MYCBP2	Q7TPH6	S3577	VNSGDTVGSSLLR	S3	1.010	0.902
Mycbp2	E3 ubiquitin-protein ligase MYCBP2	Q7TPH6	S4002	ALLSSPEGEEK	S4	1.013	0.911
Mycbp2	E3 ubiquitin-protein ligase MYCBP2	Q7TPH6	T3992	LISGDAEPTPEQEEK	T9	1.104	0.444
Mycbp2	E3 ubiquitin-protein ligase MYCBP2	Q7TPH6	S2884,S2889	TSSGFRAESPGPGSR	S9,S14	1.044	0.676

Mycbp2	E3 ubiquitin-protein ligase MYCBP2	Q7TPH6	Ambiguous	STSVPAPYISVTPDASPNVFEEPESNMK	T/S/Y	1.536	0.218
Myh9	Myosin-9	Q8VDD5	S1714	QAQQERDELADEIANSSGK	S17	1.031	0.202
Myh9	Myosin-9	Q8VDD5	S1943	GTGDCSDEEVDGK	S6	1.051	0.459
Myh9	Myosin-9	Q8VDD5	S1943	GTGDCSDEEVDGKADGADAK	S6	1.036	0.514
Myh9	Myosin-9	Q8VDD5	S1943	KGTGDCSDEEVDGK	S7	1.024	0.833
Myh9	Myosin-9	Q8VDD5	S1943	KGTGDCSDEEVDGKADGADAK	S7	0.988	0.844
Myh9	Myosin-9	Q8VDD5	Y754	ALELDSNLYR	Y9	1.091	0.217
Myh9	Myosin-9	Q8VDD5	T1939,S1943	KGTGDCSDEEVDGKADGADAK	T3,S7	1.078	0.313
Myh9	Myosin-9	Q8VDD5	Ambiguous	ELDDTRASREEILAQAK	T/S	1.031	0.612
Mylip	E3 ubiquitin-protein ligase MYLIP	Q8BM54	S345	SDQSPPSSPLK	S4	1.071	0.534
Mylip	E3 ubiquitin-protein ligase MYLIP	Q8BM54	S348,S349	SDQSPPSSPLK	S7,S8	0.999	0.890
Myo18a	Unconventional myosin-XVIIIa	E9QAX2	S1327	NELRLSSDRLETR	S6	0.957	0.471
Myo18a	Unconventional myosin-XVIIIa	E9QAX2	S140	RFSFSQR	S3	0.963	0.786
Myo18a	Unconventional myosin-XVIIIa	E9QAX2	S1684	LSTLSDQVNQR	S5	1.017	0.847
Myo18a	Unconventional myosin-XVIIIa	E9QAX2; F6ZGN3	S2027;S57	APSDDGSLK	S3	0.906	0.362
Myo18a	Unconventional myosin-XVIIIa	E9QAX2	S2067	FSHSYLSDSDTEAKLTETSA	S4	0.954	0.828
Myo18a	Unconventional myosin-XVIIIa	E9QAX2	S2070	FSHSYLSDSDTEAK	S7	1.009	0.788
Myo18a	Unconventional myosin-XVIIIa	E9QAX2	S420	SEELSLPEGK	S5	1.091	0.320
Myo18a	Unconventional myosin-XVIIIa	E9QAX2	S74	VASGSDLHLTDIDSDSNR	S5	0.998	0.952
Myo18a	Unconventional myosin-XVIIIa	E9QAX2	S2070,S2072	FSHSYLSDSDTEAK	S7,S9	1.023	0.652
Myo18a	Unconventional myosin-XVIIIa (Fragment)	F6ZGN3	S181	SSTVSAPVSR	S5	1.017	0.875
Myo18a	Unconventional myosin-XVIIIa	E9QAX2	Ambiguous	STSFRQGPEESGLGEGTK	S/T	0.988	0.916
Myo1c	Unconventional myosin-Ic	Q9WTI7	S408	DAESPSWR	S4	0.957	0.544
Myo1f	Myosin IF	Q8CG29	S1001	ARPPSEHSTEFLNVPDQGVAGMQR	S5	1.003	0.959
Myo1f	Myosin IF	Q8CG29	S1001	ARPPSEHSTEFLNVPDQGVAGMQR	S5	0.999	0.987
Myo1f	Myosin IF	Q8CG29	S671	AVNMEPDQYQMGSTK	S13	0.987	0.853
Myo1f	Myosin IF	Q8CG29	S888	EGWGGGSTR	S7	1.139	0.080
Myo1f	Myosin IF	Q8CG29	S966	SLPLEITSGR	S1	1.119	0.139
Myo1f	Myosin IF	Q8CG29	S977	SSQRPPRGPPSSTLGASR	S2	0.945	0.179
Myo1f	Myosin IF	Q8CG29	S986	GPPSSTLGASR	S4	1.074	0.216
Myo1f	Myosin IF	Q8CG29	S1001,S1004	ARPPSEHSTEFLNVPDQGVAGMQR	S5,S8	1.115	0.218
Myo1g	Unconventional myosin-Ig	Q5SUA5	S99	SRDTCIVISGESGAGK	S1	1.041	0.261
Myo5a	Unconventional myosin-Va	D3Z4J3	S1292	NTMTDSTILLEDVQK	S6	1.131	0.159
Myo5a	Unconventional myosin-Va	D3Z4J3	S600	AISPTSATSSGR	S3	1.037	0.197
Myo5a	Unconventional myosin-Va	D3Z4J3	Ambiguous	RTDSTHSSNESEYTFSSEFAETEDIAPR	S/T/Y	0.957	0.072
Myo5a	Unconventional myosin-Va	D3Z4J3	Ambiguous	TSSIADEGTYTLDSILR	S/T/Y	1.446	0.322
Myo5a	Unconventional myosin-Va	D3Z4J3	Ambiguous	RTDSTHSSNESEYTFSSEFAETEDIAPR	S/T/Y	1.037	0.860
Myo9a	Unconventional myosin-IXa	D3Z3A8	S1243	RQSGTDLQEDVIVR	S3	0.978	0.597
Myo9a	Unconventional myosin-IXa	D3Z3A8	S1259	SLEDLHQK	S1	0.971	0.683
Myo9a	Unconventional myosin-IXa	D3Z3A8	S1300	SLGGMSPSEER	S6	1.017	0.672
Myo9a	Unconventional myosin-IXa	D3Z3A8	S1300	SLGGMSPSEER	S6	1.124	0.120
Myo9a	Unconventional myosin-IXa	D3Z3A8	S1583	GEAGVLGSPSAVTK	S8	1.074	0.681
Myo9a	Unconventional myosin-IXa	D3Z3A8	S2385	GNYPSPSSPVIVR	S8	1.051	0.270
Myo9b	Unconventional myosin-IXb	A0A1D5RLD1	S718	AAAGISSPATR	S7	0.971	0.452
Myo9b	Unconventional myosin-IXb	E9PZW8; A0A1D5RLD1	S1043;S1044	SFSQMVSEK	S1	0.974	0.448
Myo9b	Unconventional myosin-IXb	E9PZW8; A0A1D5RLD1	S1106;S1107	SPDGMSPK	S6	1.052	0.498
Myo9b	Unconventional myosin-IXb	E9PZW8; A0A1D5RLD1	S1106;S1107	SPDGMSPKKETPSPEMETAAQK	S6	0.866	0.373
Myo9b	Unconventional myosin-IXb	E9PZW8; A0A1D5RLD1	S1113;S1114	KETPSPEMETAAQK	S5	1.081	0.020
Myo9b	Unconventional myosin-IXb	E9PZW8; A0A1D5RLD1	S1113;S1114	KETPSPEMETAAQK	S5	1.024	0.529
Myo9b	Unconventional myosin-IXb	E9PZW8; A0A1D5RLD1	S1113;S1114	ETPSPEMETAAQK	S4	0.997	0.950
Myo9b	Unconventional myosin-IXb	E9PZW8; A0A1D5RLD1	S1113;S1114	ETPSPEMETAAQK	S4	0.959	0.399
Myo9b	Unconventional myosin-IXb	E9PZW8; A0A1D5RLD1	S1175;S1176	ASLEIGESFPEGTK	S2	0.987	0.857
Myo9b	Unconventional myosin-IXb	E9PZW8; A0A1D5RLD1	S1175;S1176	RASLEIGESFPEGTK	S3	1.044	0.828
Myo9b	Unconventional myosin-IXb	E9PZW8; A0A1D5RLD1	S1218;S1219	QAQVVGDPPGSPSPVQRPTTLALDSR	S11	1.023	0.589
Myo9b	Unconventional myosin-IXb	E9PZW8; A0A1D5RLD1	S1275;S1265	AQDKPESPSGSTQIQR	S7	1.090	0.153
Myo9b	Unconventional myosin-IXb	E9PZW8; A0A1D5RLD1	S1384;S1372	SGDPSAGPDAGLSPGSQGDSK	S13	1.123	0.019
Myo9b	Unconventional myosin-IXb	E9PZW8; A0A1D5RLD1	S1384;S1372	KSGDPSAGPDAGLSPGSQGDSK	S14	1.145	0.056
Myo9b	Unconventional myosin-IXb	E9PZW8; A0A1D5RLD1	S1963;S1935	GSDEENLDSETSASTESLLEER	S2	1.000	1.000
Myo9b	Unconventional myosin-IXb	E9PZW8	S2021	GRPTSFVTVR	S5	0.873	0.080
Myo9b	Unconventional myosin-IXb	E9PZW8	S2112	RYSDPPTYCLPPSSGQANG	S3	1.043	0.082
Myo9b	Unconventional myosin-IXb	E9PZW8	S2112	RRYSDPPTYCLPPSSGQANG	S4	1.030	0.571
Myo9b	Unconventional myosin-IXb	E9PZW8	T1328;T1318	ATGAALTPTEER	T7	1.360	0.000
Myo9b	Unconventional myosin-IXb	E9PZW8; A0A1D5RLD1	T1328;T1318	ATGAALTPTEERR	T7	1.261	0.010
Myo9b	Unconventional myosin-IXb	E9PZW8; A0A1D5RLD1	T1910;T1882	SPRTPVVQDLELGALPEEAAGGDEDREK	T4	1.432	0.005
Myo9b	Unconventional myosin-IXb	E9PZW8; A0A1D5RLD1	T1111,S1113,S1114	KETPSPEMETAAQK	T3,S5	1.001	0.987
Myo9b	Unconventional myosin-IXb	E9PZW8; A0A1D5RLD1	Ambiguous	LPELDPRGSDEENLDSETSASTESLLEER	T/S	1.028	0.943
Myof	Myoferlin	A0A286YDF5	S174	GPGPKGPSGTVSEAQLAR	S12	0.995	0.964
Myof	Myoferlin	A0A286YDF5	S174	GPSGTVSEAQLAR	S7	1.045	0.173
Myof	Myoferlin	A0A286YDF5	Ambiguous	DLTQTASSTAR	S/T	1.073	0.156
N4bp1	NEDD4-binding protein 1	Q6A037	T241	ARTPVSELTK	T3	1.023	0.726
N4bp2	NEDD4-binding protein 2	F8VQG7	S546	DSEAISSAKEDSVSSASPK	S17	1.052	0.669
N4bp2	NEDD4-binding protein 2	F8VQG7	S546	EDSVSSASPK	S8	1.083	0.542
Naa10	N-alpha-acetyltransferase 10	Q9QY36	Ambiguous	DLSEVSETTESTDVKDSSEASDSAS	S/T	0.916	0.562
Naa15	N-alpha-acetyltransferase 15, NatA auxiliary subunit	G3X8Y3	S854	ITVNGDSSAETEELANEI	S7	1.025	0.637
Nab1	NGFI-A-binding protein 1	Q61122	S327	CGERDELSPK	S8	0.960	0.621
Nab1	NGFI-A-binding protein 1	Q61122	S401	QSSGEQSPDGGLPSDSSDGQGERPLNLR	S3	1.009	0.942
Nab2	NGFI-A-binding protein 2	Q61127	S6	APSPTAEQPPGR	S3	1.101	0.086
Nab2	NGFI-A-binding protein 2	Q61127	S6	APSPTAEQPPGRGDNTR	S3	1.021	0.843
Nabp1	SOSS complex subunit B2	Q8BGW5	S170	SNGPGPISPQLPGTPSSQTVR	S8	0.942	0.480
Naca	Nascent polypeptide-associated complex subunit alpha, muscle-specific form	P70670	Ambiguous	VQGEAVSNIQENTQTPTVQEESEEEEVDETGVEVK	T/S	0.976	0.621
Naca	Nascent polypeptide-associated complex subunit alpha, muscle-specific form	P70670	Ambiguous	VQGEAVSNIQENTQTPTVQEESEEEEVDETGVEVK	T/S	0.969	0.750
Nadk	NAD kinase	P58058	S48	SLSASPALGSTK	S3	1.029	0.728
Nadk	NAD kinase	P58058	S64	SLHGPCPVTTFGPK	S1	1.199	0.247
Nadk	NAD kinase	P58058	S46,S50	SLSASPALGSTK	S1,S5	1.126	0.404
Nadk2	NAD kinase 2, mitochondrial	Q8C5H8	Ambiguous	VTNEYNESLLYSPEEPK	Y/S/T	1.291	0.240
Naf1	H/ACA ribonucleoprotein complex non-core subunit NAF1	E9QJT2	S416	NDQEPPPEVLDFSDDEKEK	S13	1.058	0.435
Nagk	N-acetyl-D-glucosamine kinase	Q9D997	S94	SLGLSLSGGEQEDAVR	S7	1.084	0.381
Nap1l4	Nap1l4 protein	B7ZNL2	S49	LDNVSHTPSSYIETLPK	S5	1.037	0.603
Nap1l4	Nap1l4 protein	B7ZNL2	Ambiguous	EFITGDVEPTDAESAWHSENEEEDKLAGDMK	S/T	0.906	0.374
Napg	Gamma-soluble NSF attachment protein	Q9CWZ7	S308	SPATPQAKPDGAAGMAAEEEEDEYSGGLC	S25	1.052	0.484
Napg	Gamma-soluble NSF attachment protein	Q9CWZ7	T287	KSPATPQAKPDGAAGMAAEEEEDEYSGGLC	T5	0.883	0.645
Nasp	Nuclear autoantigenic sperm protein	B1AU75	S764	EVSENMEAEAENQAESQTAEGTVESAATIK	S25	0.890	0.470
Nat10	RNA cytidine acetyltransferase	Q8K224	Ambiguous	TLSDDLDEAAK	T/S	1.217	0.194
Nav1	Neuron navigator 1	Q8CH77	S1251	SASSYSDIEEIATPDSSAPSSPK	S21	0.834	0.089
Nav1	Neuron navigator 1	Q8CH77	S202	APEAAVSDDGKSDDELLSSK	S12	0.999	0.969
Nav1	Neuron navigator 1	Q8CH77	S299	GSQVTHSSLEMPCYDSDDANPR	S16	1.214	0.081
Nav1	Neuron navigator 1	Q8CH77	S299	GSQVTHSSLEMPCYDSDDANPR	S16	1.029	0.735
Nav1	Neuron navigator 1	Q8CH77	S311	SVSSLSNR	S6	0.963	0.613
Nav1	Neuron navigator 1	Q8CH77	S394	SGYMSDSDLMGK	S5	1.064	0.336
Nav1	Neuron navigator 1	Q8CH77	S394	SGYMSDSDLMGK	S5	0.959	0.579
Nav1	Neuron navigator 1	Q8CH77	S455	SSTIVLR	S2	0.842	0.259
Nav1	Neuron navigator 1	Q8CH77	S455	RSSTIVLR	S3	0.910	0.500
Nav1	Neuron navigator 1	Q8CH77	S510	ERPESCDDASK	S5	1.098	0.492
Nav1	Neuron navigator 1	Q8CH77	S762	SIGSPESTPK	S4	0.998	0.906
Nav1	Neuron navigator 1	Q8CH77	S998	GQLTNIVSPTAATTPR	S8	0.998	0.978
Nav1	Neuron navigator 1	Q8CH77	S1251,S/Y/T	SASSYSDIEEIATPDSSAPSSPK	S21,S/T/Y	0.881	0.284
Nav1	Neuron navigator 1	Q8CH77	S197,S202	APEAAVSDDGKSDDELLSSK	S7,S12	1.052	0.548
Nav1	Neuron navigator 1	Q8CH77	S759,S762	SIGSPESTPK	S1,S4	0.886	0.216
Nav1	Neuron navigator 1	Q8CH77	Ambiguous	VAPGPSSGCTPGQVPGSSALSSPR	S/T	1.073	0.792
Nav1	Neuron navigator 1	Q8CH77	Ambiguous	SVQPEVELSGGSGSGGDEGADESR	S	1.129	0.538
Nav2	Neuron navigator 2	E9Q842	S1262	GISSDSESVASCNSVK	S4	1.156	0.011
Nav2	Neuron navigator 2	E9Q842	S1301	YADVASPTLR	S6	1.020	0.600
Nav2	Neuron navigator 2	E9Q842	S1568	THSLSNADGQYDPYTDSR	S3	0.999	0.990
Nav2	Neuron navigator 2	E9Q842	S1921	APSQVSISASPR	S10	1.058	0.301
Nav2	Neuron navigator 2	E9Q842	S290	SQSFNNYDK	S3	1.033	0.645
Nav2	Neuron navigator 2	E9Q842	S461	ALSTVGPASSSPK	S11	1.098	0.274
Nav2	Neuron navigator 2	E9Q842	Ambiguous	KTSMDGAPNQDDGYLSLSSR	S/T/Y	0.929	0.150
Nbas	Neuroblastoma-amplified sequence	E9Q411	S472	AGEEDEGEVDSDSDPETSAK	S11	0.982	0.766
Nbeal2	Neurobeachin-like protein 2	E9Q9L6	S1304	LYVLEAATDSSPPR	S11	1.033	0.847
Nbeal2	Neurobeachin-like protein 2	E9Q9L6	S2735	RISQVSSGETEYNPGEAR	S3	1.193	0.065
Nbeal2	Neurobeachin-like protein 2	E9Q9L6	T1862	DNLGEAPMTPTEETSLPLAVTK	T9	1.051	0.606
Nbr1	Next to BRCA1 gene 1 protein	K3W4P1	S657	SLTLDAAPDHNPPCR	S1	0.979	0.825
Ncam1	Neural cell adhesion molecule 1	P13595	S1005	NPPEAATAPASPK	S11	0.984	0.906
Ncapd2	Condensin complex subunit 1	A0A0R4J0H7	S1300	GMDGIEEFETGQGGSQR	S15	0.950	0.660
Ncapg	Non-SMC condensin I complex, subunit G	E9PWG6	S967	RQIAGPDSESDHEPPSEMK	S10	1.009	0.880
Ncapg	Non-SMC condensin I complex, subunit G	E9PWG6	S967	QIAGPDSESDHEPPSEMK	S9	0.971	0.354
Ncapg	Non-SMC condensin I complex, subunit G	E9PWG6	Ambiguous	QIAGPDSESDHEPPSEMK	S	0.914	0.240
Ncaph	Condensin complex subunit 2	Q8C156	S191	DTPPQGEESHSGDGSTLETER	S11	1.005	0.956
Ncaph	Condensin complex subunit 2	Q8C156	S25	GQQDVLSSPLER	S8	1.205	0.027
Ncaph	Condensin complex subunit 2	Q8C156	Ambiguous	KEADTEANHTESGQEGAPEEVADEK	T/S	1.053	0.601
Ncbp2	Nuclear cap-binding protein subunit 2	Q9CQ49	S13	SDSYVELSEYR	S3	1.262	0.016
Ncbp2	Nuclear cap-binding protein subunit 2	Q9CQ49	S13	ALRSDSYVELSEYR	S6	1.185	0.374
Ncbp3	Nuclear cap-binding protein subunit 3	Q8BZR9	S25	AEAPAGPALGLPSPEVESGLER	S13	1.025	0.790
Ncbp3	Nuclear cap-binding protein subunit 3	Q8BZR9	S439	ADSISTSNIK	S3	0.981	0.823
Ncbp3	Nuclear cap-binding protein subunit 3	Q8BZR9	S495	RPYSPEKAFSSNQVVR	S4	0.982	0.860
Ncf1	Neutrophil cytosol factor 1	F8WH69	S321	RLSQDTYR	S3	0.998	0.970
Ncf2	Neutrophil cytosol factor 2	O70145	S332	LQLSPGHK	S4	1.022	0.272
Ncf2	Neutrophil cytosol factor 2	O70145	S385	LALSPEHTK	S4	0.937	0.247

Ncf2	Neutrophil cytosol factor 2	O70145	Ambiguous	IHPQSQPQEDTSPESDIPPPPNSSPPGR	S/T	0.989	0.624
Ncf2	Neutrophil cytosol factor 2	O70145	Ambiguous	IHPQSQPQEDTSPESDIPPPPNSSPPGR	S/T	1.038	0.428
Ncf4	Neutrophil cytosol factor 4	P97369	S161	GVSPQGAIMDR	S3	1.056	0.182
Ncf4	Neutrophil cytosol factor 4	P97369	Y243	CYFYEDTGK	Y2	1.038	0.876
Nck1	Cytoplasmic protein NCK1	Q99M51	S85	RKPSVPDTASPADDSFVDPGER	S4	0.967	0.634
Nck1	Cytoplasmic protein NCK1	Q99M51	T89	KPSVPDTASPADDSFVDPGER	T7	1.051	0.696
Nck2	Cytoplasmic protein NCK2	O55033	S90	DASPTPSTDAEYPANGSGADR	S3	1.100	0.034
Nck2	Cytoplasmic protein NCK2	O55033	S90,T92	DASPTPSTDAEYPANGSGADR	S3,T5	0.989	0.848
Nckap1	Nck-associated protein 1	A2AS98	S343	EAAVSHAGSMHRERR	S9	0.873	0.315
Nckap5l	Nck-associated protein 5-like	Q6GQX2	S473	VPSPGGPQLSPQLPR	S10	1.030	0.915
Nckap5l	Nck-associated protein 5-like	Q6GQX2	S494	NSGSDGSPSPLLAR	S9	1.014	0.778
Nckap5l	Nck-associated protein 5-like	Q6GQX2	S601	SPGAPSPGLPESCPYSGPQEK	S6	1.045	0.563
Nckap5l	Nck-associated protein 5-like	Q6GQX2	S760	VDLEPISPR	S7	1.089	0.342
Nckipsd	NCK-interacting protein with SH3 domain	Q9ESJ4	S147	QHSLPSSEHLGTDGALYQVPPQPR	S3	1.015	0.698
Nckipsd	NCK-interacting protein with SH3 domain	Q9ESJ4	T173	AAPTTPPPPVK	T4	1.013	0.807
Nckipsd	NCK-interacting protein with SH3 domain	Q9ESJ4	Ambiguous	RAAPTTPPPPVK	T	1.346	0.007
Ncl	Nucleolin	P09405	S460	SVSLYYTGEK	S1	0.991	0.847
Ncl	Nucleolin	P09405	S616	GFGFVDFNSEEDAK	S9	0.929	0.566
Ncl	Nucleolin	P09405	S145,S157	KEDSDEDEDEEDEDDSDEDEDDEEEDEFEPPIVK	S4,S16	0.984	0.795
Ncl	Nucleolin	P09405	Ambiguous	KEDSDEDEDEEDEDDSDEDEDDEEEDEFEPPIVK	S	0.976	0.825
Ncoa2	Nuclear receptor coactivator 2	Q61026	S29	ECPDQLGPSPK	S9	1.056	0.534
Ncoa2	Nuclear receptor coactivator 2	Q61026	S487	MNSPSQSSPGMNPGQASSVLSPR	S21	0.881	0.419
Ncoa2	Nuclear receptor coactivator 2	Q61026	S493	MSPGVAGSPR	S2	1.160	0.022
Ncoa2	Nuclear receptor coactivator 2	Q61026	S565	MGNLQNSPVNMNPPPLSK	S7	0.734	0.083
Ncoa2	Nuclear receptor coactivator 2	Q61026	S736	ELSQESSSTAPGSEVTVKQEPASPK	S23	0.988	0.951
Ncoa2	Nuclear receptor coactivator 2	Q61026	S469,S	MNSPSQSSPGMNPGQASSVLSPR	S3,S	0.976	0.684
Ncoa2	Nuclear receptor coactivator 2	Q61026	Ambiguous	LLQDSSSPVDLAK	S	1.029	0.811
Ncoa3	Nuclear receptor coactivator	Q05BA5	S214	THDILEDVNASPETR	S11	0.967	0.367
Ncoa3	Nuclear receptor coactivator	Q05BA5	S543	LDNSPNMNISQPSK	S4	0.996	0.920
Ncoa3	Nuclear receptor coactivator	Q05BA5	S543	LDNSPNMNISQPSK	S4	0.993	0.917
Ncoa3	Nuclear receptor coactivator	Q05BA5	S686	LLQNGNSPAEVAK	S7	0.966	0.766
Ncoa3	Nuclear receptor coactivator	Q05BA5	S719	QEQLSPK	S5	0.948	0.399
Ncoa3	Nuclear receptor coactivator	Q05BA5	S846	AVSLDSPVSVGSGPPVK	S3	1.062	0.526
Ncoa3	Nuclear receptor coactivator	Q05BA5	S924	MEPLASSPLGR	S7	1.009	0.847
Ncoa3	Nuclear receptor coactivator	Q05BA5	Ambiguous	GSSPPSAMMSSR	S	1.054	0.546
Ncoa3	Nuclear receptor coactivator	Q05BA5	Ambiguous	GSSPPSAMMSSR	S	1.031	0.633
Ncoa4	Nuclear receptor coactivator 4	Q5U4H9	S583	AQEAFLNSPLQEER	S8	0.912	0.191
Ncoa5	Nuclear receptor coactivator 5	B7ZC24	S381	SSADSLPGELR	S5	0.938	0.005
Ncoa5	Nuclear receptor coactivator 5	Q91W39; B7ZC24	S116;S116	DIRDSRDPLYR	S5	0.935	0.477
Ncoa5	Nuclear receptor coactivator 5	Q91W39	S378	SSADSLPGPISR	S2	1.037	0.605
Ncoa5	Nuclear receptor coactivator 5	Q91W39; B7ZC24	S29,S34,S34	DRSPIRGSPR	S3,S8	1.030	0.561
Ncoa5	Nuclear receptor coactivator 5	B7ZC24	Ambiguous	LLRSSADSLPGELR	S	0.945	0.564
Ncoa5	Nuclear receptor coactivator 5	Q91W39	Ambiguous	LLRSSADSLPGPISR	S	1.020	0.887
Ncoa7	Nuclear receptor coactivator 7	Q6DFV7	S211	VLSSTSEEDEPGVVK	S3	1.096	0.306
Ncoa7	Nuclear receptor coactivator 7	Q6DFV7	S92	YYSVDDNQNK	S3	1.446	0.001
Ncoa7	Nuclear receptor coactivator 7	Q6DFV7	S92	RYYSVDDNQNK	S4	1.216	0.105
Ncoa7	Nuclear receptor coactivator 7	Q6DFV7	S211,T213	VLSSTSEEDEPGVVK	S3,T5	1.143	0.052
Ncor1	Nuclear receptor corepressor 1 (Fragment)	Q3UV08; Q5RIM6; Q60974	S370;S780;S779	GNTEPVAELEATTDPAPCASPSSAVPTTKPAER	S20	1.041	0.488
Ncor1	Nuclear receptor corepressor 1 (Fragment)	Q3UV08; Q5RIM6; Q60974	S941;S1293;S1292	GSPHSDLK	S2	1.067	0.566
Ncor1	Nuclear receptor corepressor 1 (Fragment)	Q3UV08	S639,S644	SASPCRLSPR	S3,S8	1.031	0.660
Ncor1	Nuclear receptor co-repressor 1, isoform CRA_a	Q5RIM6; Q60974	S1482;S1481	AQLSPGLYDDSSAR	S4	1.073	0.059
Ncor1	Nuclear receptor co-repressor 1, isoform CRA_a	Q5RIM6; Q60974	S1482;S1481	AQLSPGLYDDSSARR	S4	0.870	0.268
Ncor1	Nuclear receptor co-repressor 1, isoform CRA_a	Q5RIM6; Q60974	S2040;S2039	SQQESPSPQQQPPLPPSSQSEGMGQVPR	S5	0.942	0.482
Ncor1	Nuclear receptor co-repressor 1, isoform CRA_a	Q5RIM6; Q60974	S2040;S2039	SQQESPSPQQQPPLPPSSQSEGMGQVPR	S5	0.854	0.302
Ncor1	Nuclear receptor co-repressor 1, isoform CRA_a	Q5RIM6; Q60974	S2135;S2134	VSPENLVDK	S2	1.024	0.518
Ncor1	Nuclear receptor co-repressor 1, isoform CRA_a	Q5RIM6; Q60974	S2166;S2165	SHIPSEPYEPISPPQGPAVHEK	S12	0.997	0.969
Ncor1	Nuclear receptor co-repressor 1, isoform CRA_a	Q5RIM6	Ambiguous	TGYEQFHSGPSPVDHDSLESK	S/T/Y	1.028	0.748
Ncor1	Nuclear receptor co-repressor 1, isoform CRA_a	Q5RIM6	Ambiguous	HEAPSSPLAGQPCGDDQNASPSK	S	1.040	0.420
Ncor1	Nuclear receptor corepressor 1	Q60974	Ambiguous	HEAPSSPLSGQPCGDDQNASPSK	S	1.031	0.600
Ncor1	Nuclear receptor co-repressor 1, isoform CRA_a	Q5RIM6	Ambiguous	DSAFGSKHEAPSSPLAGQPCGDDQNASPSK	S	0.981	0.961
Ncor1	Nuclear receptor co-repressor 1, isoform CRA_a	Q5RIM6; Q60974	Ambiguous	EPAPLLSAQYETLSDSDD	T/S/Y	0.910	0.288
Ncor2	Nuclear receptor corepressor 2 (Fragment)	D3Z2J5; E9PY55	S164;S750	VGECSGPAAVNNSSDTESVPSPR	S21	0.866	0.013
Ncor2	Nuclear receptor corepressor 2 (Fragment)	D3Z2J5; E9PY55	S335;S921	GSSSGATQDSDSSATCSADEVDEPEGGDKGR	S17	0.922	0.652
Ncor2	Nuclear receptor corepressor 2 (Fragment)	D3Z2J5; E9PY55	S437;S1023	SRSPVPPAEK	S3	1.014	0.658
Ncor2	Nuclear receptor corepressor 2 (Fragment)	D3Z2J5	S592	QEQLSPR	S5	1.014	0.695
Ncor2	Nuclear receptor corepressor 2 (Fragment)	D3Z2J5; E9PY55	S668;S1219	IVGEDSPSRLDR	S6	0.959	0.630
Ncor2	Nuclear receptor corepressor 2 (Fragment)	D3Z2J5; E9PY55	S757;S1308	AIPEQHSPHLK	S7	0.905	0.281
Ncor2	Nuclear receptor corepressor 2 (Fragment)	D3Z2J5; E9PY55	T798;T1349	EGTPPPPPPPR	T3	0.998	0.987
Ncor2	Nuclear receptor corepressor 2 (Fragment)	D3Z2J5; E9PY55	T798;T1349	REGTPPPPPPPR	T4	0.903	0.087
Ncor2	Nuclear receptor corepressor 2	E9PY55	S2177	TSVLGSSEDAIEPVSPPEGMTEPGHAR	S15	0.790	0.090
Ncor2	Nuclear receptor corepressor 2	E9PY55	S2177	TSVLGSSEDAIEPVSPPEGMTEPGHAR	S15	0.950	0.254
Ncor2	Nuclear receptor corepressor 2	E9PY55	S54	DYTSHLSPGSIIQPQR	S7	1.038	0.796
Ncor2	Nuclear receptor corepressor 2	E9PY55	S67	RPSLLSEFQPGSER	S3	1.318	0.029
Ncor2	Nuclear receptor corepressor 2 (Fragment)	D3Z2J5; E9PY55	Ambiguous	QLGAISQGMSVQLR	S	1.094	0.492
Ncor2	Nuclear receptor corepressor 2	E9PY55	Ambiguous	SDHALTSPGGGGK	T/S	0.857	0.149
Ndel1	Nuclear distribution protein nudE-like 1	Q9ERR1	S198	SAPSSPTLDCEK	S5	0.954	0.460
Ndel1	Nuclear distribution protein nudE-like 1	Q9ERR1	S231	GTENSFPSPK	S8	1.009	0.858
Ndel1	Nuclear distribution protein nudE-like 1	Q9ERR1	S194,S197	SAPSSPTLDCEK	S1,S4	1.060	0.207
Ndrg1	Protein NDRG1	Q62433	S330	TASGSSVTSLEGTR	S3	1.071	0.272
Ndrg1	Protein NDRG1	Q62433	S342	TASGSSVTSLEGTRSR	S15	0.992	0.846
Ndrg1	Protein NDRG1	Q62433	T328	SRTASGSSVTSLEGTR	T3	1.059	0.297
Ndrg1	Protein NDRG1	Q62433	S330,S332	TASGSSVTSLEGTR	S3,S5	1.064	0.117
Ndrg1	Protein NDRG1	Q62433	T328,S330	SRTASGSSVTSLEGTR	T3,S5	0.977	0.668
Ndrg1	Protein NDRG1	Q62433	T328,S342	TASGSSVTSLEGTRSR	T1,S15	1.099	0.329
Ndrg2	Protein NDRG2	Q9QYG0	Ambiguous	TASLTSAASIDGSR	T/S	0.979	0.826
Ndrg3	Ndrg3 protein	Q8VCV2	S344	THSTSSSIGSGESPFSR	S3	1.081	0.147
Ndrg3	Ndrg3 protein	Q8VCV2	S374	SVTSNQSDGTQESCESPDVLDR	S16	0.917	0.129
Ndrg3	Ndrg3 protein	Q8VCV2	S344,S346	THSTSSSIGSGESPFSR	S3,S5	0.872	0.193
Ndrg3	Ndrg3 protein	Q8VCV2	Ambiguous	SVTSNQSDGTQESCESPDVLDRHQTMEVSC	S/T	0.907	0.691
Ndst2	Bifunctional heparan sulfate N-deacetylase/N-sulfotransferase 2	P52850	S881	EELQHSSVG	S7	1.038	0.534
Ndufa2	NADH dehydrogenase [ubiquinone] 1 alpha subcomplex subunit 2	Q9CQ75	S78	TVSLNNLSADEVTR	S3	0.943	0.622
Ndufaf2	NADH dehydrogenase [ubiquinone] 1 alpha subcomplex assembly factor 2	Q59J78	S122	ETSEELLPSPTATQVK	S9	1.148	0.246
Necap2	Adaptin ear-binding coat-associated protein 2	Q9D1J1	S252	STGSPSSQSQPGTGWVQF	S4	1.063	0.799
Nectin2	Nectin-2	P32507	T450	YHELPTLEER	T6	0.918	0.276
Nectin4	Nectin-4	Q8R007	S460	EIETQTELLSPGSGR	S10	1.116	0.452
Nedd4	E3 ubiquitin-protein ligase NEDD4	P46935	T287	RPSPDDDLTDEDNDDMQLQAQR	T9	1.055	0.550
Nedd4l	E3 ubiquitin-protein ligase NEDD4-like	E9PXB7	S304	TSPQELSEEVSRR	S2	0.900	0.206
Nedd4l	E3 ubiquitin-protein ligase NEDD4-like	E9PXB7	S304	TSPQELSEEVSR	S2	1.033	0.554
Nedd4l	E3 ubiquitin-protein ligase NEDD4-like	E9PXB7	S341	SCSVTDTVAEQAHLPPPSTPTRR	S1	1.156	0.002
Nedd4l	E3 ubiquitin-protein ligase NEDD4-like	E9PXB7	S476	DTLSNPQSPQPSPYNSPKPQHK	S4	0.960	0.548
Nedd4l	E3 ubiquitin-protein ligase NEDD4-like	E9PXB7	Ambiguous	SSTVTGGEESTPSVAYVHTTPGLPSGWEER	S/T/Y	0.905	0.325
Nedd4l	E3 ubiquitin-protein ligase NEDD4-like	E9PXB7	Ambiguous	SCSVTDTVAEQAHLPPPSTPTR	S/T	0.931	0.278
Nedd9	Enhancer of filamentation 1	A0A0R4J212	Y91	LYQVPNSQAASR	Y2	0.817	0.211
Nek1	Nek1 protein	B2RXX0	S1069	TCSLPDLSK	S3	0.981	0.691
Nek1	Nek1 protein	B2RXX0	S1143	EQPGDEYSEEEESVLK	S8	0.908	0.466
Nek1	Nek1 protein	B2RXX0	S990	DPAVDDSPQSGCDVEK	S7	1.049	0.684
Nek1	Nek1 protein	B2RXX0	Ambiguous	EINPSATVDSTETKSPK	T/S	1.118	0.467
Nek9	Serine/threonine-protein kinase Nek9	Q8K1R7	S331	SSTVTEAPIAVVTSR	S1	1.018	0.866
Nek9	Serine/threonine-protein kinase Nek9	Q8K1R7	Ambiguous	HCDSINSDFGSESGGGGDSGPGPSAVPGPR	S	0.891	0.527
Nelfa	Negative elongation factor A	Q8BG30	S233	SPTTPSVFSPSGNR	S9	1.053	0.578
Nelfa	Negative elongation factor A	Q8BG30	S363	EASRPPEEPSAPSPTLPTQFK	S13	0.918	0.349
Nelfe	Negative elongation factor E	P19426	S115	SMSADEDLQEPSR	S3	1.235	0.000
Nelfe	Negative elongation factor E	P19426	S115	SMSADEDLQEPSR	S3	1.296	0.004
Nelfe	Negative elongation factor E	P19426	S131	SLYESFVSSSDR	S1	1.368	0.260
Nelfe	Negative elongation factor E	P19426	S51	SLSEQPVVDTATATEQAK	S3	1.799	0.001
Nelfe	Negative elongation factor E	P19426	Ambiguous	RSLSEQPVVDTATATEQAK	S/T	1.275	0.011
Nemf	Nuclear export mediator factor Nemf	Q8CCP0	S417	NPYLLSEEEDGDGDASIENSDAEAPK	S6	1.104	0.271
Nemf	Nuclear export mediator factor Nemf	Q8CCP0	S737	DELSSEDGEAK	S4	1.141	0.236
Nemf	Nuclear export mediator factor Nemf	Q8CCP0	S417,S/Y	NPYLLSEEEDGDGDASIENSDAEAPK	S6,S/Y	1.143	0.304
Nes	Nestin	Q6P5H2	S1216	SLGEVEWELPGSGSQQR	S1	1.152	0.284
Nes	Nestin	Q6P5H2	S1216	EQHQESLRSLGEVEWELPGSGSQQR	S9	1.270	0.050
Nes	Nestin	Q6P5H2	S1380	EEAIHPSLGEESVK	S7	1.252	0.023
Nes	Nestin	Q6P5H2	S1565	SPASPKWEQAGEQR	S4	0.944	0.537
Nes	Nestin	Q6P5H2	S169	RPPAPAHASPIRAPEVEELAR	S9	0.900	0.038
Nes	Nestin	Q6P5H2	S235	LEVQQLQADRDSLQER	S12	0.991	0.840
Nes	Nestin	Q6P5H2	S575	ENCNSSIEENSGTVKSPEKEK	S16	1.043	0.643
Nes	Nestin	Q6P5H2	S688	SPEEDQQAFRPLEK	S1	1.016	0.886
Nes	Nestin	Q6P5H2	S688	FPRSPEEDQQAFRPLEK	S4	1.083	0.318
Nes	Nestin	Q6P5H2	S728;S1495	ERQESLKSPEEEDQQAFR	S5	1.125	0.001
Nes	Nestin	Q6P5H2	S731;S1500	QESLKSPEEEDQQAFR	S6	1.016	0.687
Nes	Nestin	Q6P5H2	S819	LVEKESQESLKSPEEEDQR	S12	1.042	0.515
Nes	Nestin	Q6P5H2	S819	ESQESLKSPEEEDQR	S8	1.138	0.032
Nes	Nestin	Q6P5H2	S862	NQRPLRSLEVEEEEQR	S7	1.021	0.844
Nes	Nestin	Q6P5H2	S894	VSQVSLESLEKENVQSPR	S16	1.151	0.147
Nes	Nestin	Q6P5H2	S963	ESQEFLRSPEAEEEEEQVMVR	S8	1.222	0.121
Nes	Nestin	Q6P5H2	S963	ESQEFLRSPEAEEEEEQVMVR	S8	1.022	0.808
Nes	Nestin	Q6P5H2	S977	SLEGENHDPLSSVVK	S1	1.026	0.822
Nes	Nestin	Q6P5H2	T158	AHLNAQAACTPR	T10	0.987	0.948

Nes	Nestin	Q6P5H2	Ambiguous	TQNHETPGKENCNSSIEENSGTVK	S/T	1.132	0.575
Nes	Nestin	Q6P5H2	Ambiguous	FTLSGVDGDSWSSGED	S/T	1.066	0.872
Nes	Nestin	Q6P5H2	Ambiguous	VSQVSLESLEK	S	1.089	0.592
Neurl4	Neuralized-like protein 4	Q5NCX5	S504	ALSPEGALRR	S3	0.964	0.316
Neurl4	Neuralized-like protein 4	Q5NCX5	S504	ALSPEGALR	S3	0.939	0.262
Nf1	Neurofibromin	Q04690	S2498	ETQPWSSPK	S7	1.037	0.773
Nf1	Neurofibromin	Q04690	S2517	GSEGYLAATYPAVGQTSPR	S17	1.028	0.665
Nf1	Neurofibromin	Q04690	S2525	SMSLDMGQPSQANTK	S3	0.978	0.871
Nf1	Neurofibromin	Q04690	S2819	HGSASQVQK	S3	0.873	0.379
Nf1	Neurofibromin	Q04690	S866	SSSGLATYSPPMGAVSER	S9	1.028	0.142
Nf1	Neurofibromin	Q04690	S866	SSSGLATYSPPMGAVSER	S9	1.004	0.950
Nf1	Neurofibromin	Q04690	T2567	RQEMESGITTPPK	T10	1.117	0.009
Nfam1	NFAT activation molecule 1	Q8R4V1	S217	STVGTTKPPAESVYTSLQR	S16	1.070	0.192
Nfam1	NFAT activation molecule 1	Q8R4V1	S241	EETGSPVFSQSPATK	S11	1.135	0.009
Nfam1	NFAT activation molecule 1	Q8R4V1	S235,S241	EETGSPVFSQSPATK	S5,S11	1.102	0.349
Nfam1	NFAT activation molecule 1	Q8R4V1	Ambiguous	STVGTTKPPAESVYTSLQRR	T/S/Y	0.990	0.823
Nfatc1	Nuclear factor of activated T-cells c1 isoform IA-IXL	B5B2N2	S766	NTLMPTPPNASPK	S11	1.020	0.794
Nfatc2	Nuclear factor of activated T-cells, cytoplasmic 2	Q60591	S136	DAGLSPEQPALALAGVAASPR	S5	0.873	0.435
Nfatc2	Nuclear factor of activated T-cells, cytoplasmic 2	Q60591	S328	TSPDPTPVSTAPSK	S2	1.018	0.588
Nfatc2	Nuclear factor of activated T-cells, cytoplasmic 2	Q60591	S739	SGLSSPDAR	S5	1.090	0.039
Nfatc2	Nuclear factor of activated T-cells, cytoplasmic 2	Q60591	S748	YQQQSPAAALYQR	S5	0.944	0.643
Nfatc2	Nuclear factor of activated T-cells, cytoplasmic 2	Q60591	S860	LSPGAYPTVIQQQTAPSQR	S2	1.144	0.046
Nfatc2	Nuclear factor of activated T-cells, cytoplasmic 2	Q60591	Y348	HIYPTVEFLGPCEQEER	Y3	0.931	0.741
Nfatc2	Nuclear factor of activated T-cells, cytoplasmic 2	Q60591	S136,S150	DAGLSPEQPALALAGVAASPR	S5,S19	1.246	0.166
Nfatc2	Nuclear factor of activated T-cells, cytoplasmic 2	Q60591	S136,S150	GRDAGLSPEQPALALAGVAASPR	S7,S21	1.007	0.943
Nfatc2	Nuclear factor of activated T-cells, cytoplasmic 2	Q60591	S219,S223	TSPIMSPR	S2,S6	1.031	0.419
Nfatc2	Nuclear factor of activated T-cells, cytoplasmic 2	Q60591	S238,S245	TSLAEDSCLGRHSPVPRPASR	S13,S20	0.975	0.328
Nfatc2	Nuclear factor of activated T-cells, cytoplasmic 2	Q60591	S257,S270	RHSCAEALVAPLPAASPQR	S3,S16	1.018	0.871
Nfatc3	Nuclear factor of activated T-cells c3 isoform IB-deltaXa	Q3UZ64	S1066	DTPLPGPASPDLMTSHSAQ	S9	1.000	1.000
Nfe2l2	Nuclear factor erythroid 2-related factor 2	Q60795	S400	AQPAHSPGDTVQPLSPAQGHSAPMR	S15	0.958	0.683
Nfe2l2	Nuclear factor erythroid 2-related factor 2	Q60795	S425	EVPVSPGHQK	S5	1.038	0.540
Nfe2l2	Nuclear factor erythroid 2-related factor 2	Q60795	S391,S406	AQPAHSPGDTVQPLSPAQGHSAPMR	S6,S21	0.915	0.440
Nfia	Nuclear factor 1 A-type	Q02780	S310	SVEDEMDSPGEEPFYTGQGR	S8	0.873	0.245
Nfia	Nuclear factor 1 A-type	Q02780	S310	SVEDEMDSPGEEPFYTGQGR	S8	1.014	0.735
Nfic	Nuclear factor 1	A0A1Y7VK55	S327	SGSMEEDVDTSPGGDYYTSPNSPTSSSR	S22	0.853	0.070
Nfic	Nuclear factor 1	A0A1Y7VK55	S327	SGSMEEDVDTSPGGDYYTSPNSPTSSSR	S22	1.022	0.749
Nfic	Nuclear factor 1	A0A1Y7VK55	S361	SPFNSPSPQDSPR	S7	0.987	0.762
Nfic	Nuclear factor 1	A0A1Y7VK55	S361	TEMDKSPFNSPSPQDSPR	S12	0.982	0.464
Nfic	Nuclear factor 1	A0A1Y7VK55	S306,S327	HKSGSMEEDVDTSPGGDYYTSPNSPTSSSR	S3,S24	0.989	0.946
Nfic	Nuclear factor 1	A0A1Y7VK55	S355,S/T	TEMDKSPFNSPSPQDSPR	S6,S/T	1.013	0.890
Nfic	Nuclear factor 1	A0A1Y7VK55	S355,S361	TEMDKSPFNSPSPQDSPR	S6,S12	1.149	0.513
Nfic	Nuclear factor 1	A0A1Y7VK55	Ambiguous	SGSMEEDVDTSPGGDYYTSPNSPTSSSR	S/T/Y	0.933	0.666
Nfil3	Nuclear factor interleukin-3-regulated protein	O08750	S286	TSEADEGVVGKSSDGEDEQQVPK	S13	0.966	0.539
Nfil3	Nuclear factor interleukin-3-regulated protein	O08750	S301	GPIHSPVELQR	S5	1.032	0.557
Nfix	Nuclear factor 1	E9PUH7	S280	SIDDSEMESPVDDVFYPGTGR	S9	0.878	0.281
Nfix	Nuclear factor 1	E9PUH7	Ambiguous	SITSPPSTSSTK	T/S	1.092	0.090
Nfkb2	Nuclear factor NF-kappa-B p100 subunit	Q9WTK5	S715	AGADIHAENEEPLCPLPSPSTSGSDSDSEGPER	S26	0.667	0.326
Nfkb2	Nuclear factor NF-kappa-B p100 subunit	Q9WTK5	T425	DTDAGEGAEEPRTPPEAPQGEPQALDTLQR	T13	1.255	0.147
Nfkb2	Nuclear factor NF-kappa-B p100 subunit	Q9WTK5	T425	RDTDAGEGAEEPRTPPEAPQGEPQALDTLQR	T14	0.909	0.490
Nfkbib	NF-kappa-B inhibitor beta	Q60778	S346	QPPSPASKPLPDDPNPA	S4	1.003	0.881
Nfrkb	Nuclear factor related to kappa-B-binding protein	Q6PIJ4	S1288	AVSTVVVTTAPSPK	S12	1.051	0.615
Nfrkb	Nuclear factor related to kappa-B-binding protein	Q6PIJ4; A0A1L1SSC9	S228;S240	SPSPAVPLR	S3	1.065	0.091
Nfrkb	Nuclear factor related to kappa-B-binding protein	Q6PIJ4	S493	DQAFCKENEDSSDAMTPVPR	S11	0.957	0.542
Nfrkb	Nuclear factor-related to kappa-B-binding protein (Fragment)	A0A1L1SSC9	Ambiguous	EVKEECGDTALSSDEEATLDLQEK	T/S	0.997	0.986
Nfx1	Transcriptional repressor NF-X1	B1AY10	S147	EHSPSESEKEVVIADPR	S3	1.019	0.847
Nfx1	Transcriptional repressor NF-X1	B1AY10	S147	SESGTNPREHSPSESEKEVVIADPR	S11	0.823	0.178
Nfx1	Transcriptional repressor NF-X1	B1AY10	S320	RQDPQVLSPFPR	S8	0.851	0.133
Nfx1	Transcriptional repressor NF-X1	B1AY10	S51	NYSSSPPCHLPR	S5	1.067	0.468
Nfx1	Transcriptional repressor NF-X1	B1AY10	S49,S/Y	NYSSSPPCHLPR	S3,S/Y	1.080	0.029
Nfya	Nfya protein	Q9DBV7	S326	EKDSPHMQDPNQADEEAMTQIIR	S4	0.975	0.557
Nfya	Nfya protein	Q9DBV7	S326	EKDSPHMQDPNQADEEAMTQIIR	S4	0.957	0.175
Nhej1	Non-homologous end-joining factor 1	Q3KNJ2	Ambiguous	GTDNQPEEPVSLSSTLSEPEYEPVAASGPMHR	S/T/Y	0.830	0.609
Nhlrc2	NHL repeat-containing protein 2	Q8BZW8	S575	SDSAVVDGSFPR	S3	1.013	0.939
Nhsl2	NHS-like protein 2	E9Q0V6	S1145	NTADSPTGEAAAAPGPSSSACLDAGRNDDFK	S5	1.011	0.879
Nhsl2	NHS-like protein 2	E9Q0V6	S481	GTSRLETGPGGTNR	S3	0.987	0.924
Nhsl2	NHS-like protein 2	E9Q0V6	S499	SLSVPTDSGVTSVDYDEEQK	S3	0.974	0.736
Nhsl2	NHS-like protein 2	E9Q0V6	S527	ILPYASTSSEGSNSTDNIAALSTEQEAR	S6	0.872	0.651
Nhsl2	NHS-like protein 2	E9Q0V6	T685	ATTPSQLSIEVEAR	T3	1.137	0.567
Nid2	Nidogen-2	O88322	Ambiguous	ITQTAEGLDPENYLSIK	Y/S/T	1.234	0.075
Nifk	MKI67 FHA domain-interacting nucleolar phosphoprotein	Q91VE6	S304	TPTSASPGGK	S6	1.060	0.277
Nifk	MKI67 FHA domain-interacting nucleolar phosphoprotein	Q91VE6	T257	SSVDSQGPTPVCTPTFLER	T9	0.990	0.960
Nifk	MKI67 FHA domain-interacting nucleolar phosphoprotein	Q91VE6	T257,T261	SSVDSQGPTPVCTPTFLER	T9,T13	1.035	0.835
Nifk	MKI67 FHA domain-interacting nucleolar phosphoprotein	Q91VE6	Ambiguous	EDTQKTPTSASPGGK	S/T	1.267	0.040
Nin	Ninein	Q61043	Ambiguous	DLASTGTSSVQR	S/T	1.002	0.980
Nipbl	Nipped-B-like protein	Q6KCD5	S280	SPQPVCSPAGSDGTPK	S7	1.050	0.290
Nipbl	Nipped-B-like protein	Q6KCD5	S318	DVPPDILLDSPER	S10	0.959	0.498
Nipbl	Nipped-B-like protein	Q6KCD5	S318	DVPPDILLDSPERK	S10	0.989	0.765
Nipbl	Nipped-B-like protein	Q6KCD5	S350	AAMYDIISSPTK	S9	1.072	0.582
Nipbl	Nipped-B-like protein	Q6KCD5	S350	AAMYDIISSPTKDSTK	S9	1.031	0.630
Nisch	Nischarin	Q80TM9	S545	ACSDSLESIPAGQVASDDLR	S5	0.967	0.749
Nisch	Nischarin	Q80TM9	S669	YFEMGPPDAEEEEGSGQGEEDEEDEDEEAEEER	S15	0.608	0.293
Nisch	Nischarin	Q80TM9	S669	YFEMGPPDAEEEEGSGQGEEDEEDEDEEAEEER	S15	0.987	0.859
Nisch	Nischarin	Q80TM9	S545,S548	ACSDSLESIPAGQVASDDLR	S5,S8	0.885	0.285
Nkapd1	NKAP domain-containing 1	E9PUQ3	S184	EVTDITADSSSEFSEETR	S9	1.204	0.080
Nktr	NK-tumor recognition protein	P30415	S1060	TCDEGSSPSKPK	S7	1.052	0.218
Nktr	NK-tumor recognition protein	P30415	S1139	GEVVSPLANHR	S5	0.970	0.812
Nktr	NK-tumor recognition protein	P30415	S1148	LDSPEVNIIPEQDECMAHPR	S3	0.928	0.632
Nktr	NK-tumor recognition protein	P30415	S1148	LDSPEVNIIPEQDECMAHPR	S3	1.011	0.732
Nktr	NK-tumor recognition protein	P30415	S1195	QDSSTSVTSPVETSGKK	S9	0.977	0.688
Nktr	NK-tumor recognition protein	P30415	S255	TVSPEGYSER	S3	1.034	0.447
Nktr	NK-tumor recognition protein	P30415	S563,S565	RTSISPK	S3,S5	0.996	0.954
Nktr	NK-tumor recognition protein	P30415	Ambiguous	IVMPDLEPSRSPTHR	S/T	0.887	0.361
Nktr	NK-tumor recognition protein	P30415	Ambiguous	IVMPDLEPSRSPTHR	S/T	1.005	0.952
Nlk	Serine/threonine-protein kinase NLK	O54949	T298	HMTQEVVTQYYR	T3	0.967	0.731
Nln	Neurolysin, mitochondrial	Q91YP2	S32	EAASPLQAMSSYTAAGR	S4	1.030	0.716
Nlrc4	NLR family CARD domain-containing protein 4	Q3UP24	S533	QESIQSLR	S3	1.256	0.022
Nmd3	60S ribosomal export protein NMD3	Q99L48	S468	DATIPVESDTDDEGAPR	S8	0.983	0.807
Nme4	Nucleoside diphosphate kinase, mitochondrial	Q9WV84	S152	NVIHASDSVDGAQR	S6	1.038	0.656
Nme7	Non-metastatic cells 7, protein expressed in, isoform CRA_c	Q3UMG6	S14	SSSLVSPYMAPK	S6	0.878	0.026
Nmt1	Glycylpeptide N-tetradecanoyltransferase 1	O70310	S47	SGLSPANDTGAK	S4	1.009	0.782
Nmt1	Glycylpeptide N-tetradecanoyltransferase 1	O70310	S69	EKGSDMESTQDQPVK	S4	0.898	0.006
Nmt1	Glycylpeptide N-tetradecanoyltransferase 1	O70310	S69	EKGSDMESTQDQPVK	S4	0.979	0.657
Nmt1	Glycylpeptide N-tetradecanoyltransferase 1	O70310	Ambiguous	LPAPSLPLMMEGNGNGHEHCSDCENEEDNSHNR	S	0.949	0.721
Nmt2	Glycylpeptide N-tetradecanoyltransferase 2	O70311	S38	GSPGGDLGAK	S2	1.012	0.780
Nmt2	Glycylpeptide N-tetradecanoyltransferase 2	O70311	S68	SDSASDSQEIK	S3	1.031	0.556
Nol4l	Nucleolar protein 4-like	Q3UH85	S374	NTPESPPYSSGSYDSIK	S5	0.973	0.708
Nol4l	Nucleolar protein 4-like	Q3UH85	S643	TMPTAQLSPTEISAVR	S8	0.668	0.092
Nol4l	Nucleolar protein 4-like	Q3UH85	T620	GGASTPTPPTPTPSSNSTSR	T5	0.922	0.242
Nolc1	Nucleolar and coiled-body phosphoprotein 1 (Fragment)	A0A286YDV7	S554	ANGTPASLNGK	S7	1.111	0.370
Nolc1	Nucleolar and coiled-body phosphoprotein 1 (Fragment)	A0A286YDV7	S563	AAKESEEEEEEEETEEKK	S5	0.969	0.594
Nolc1	Nucleolar and coiled-body phosphoprotein 1 (Fragment)	A0A286YDV7	S563	AAKESEEEEEEEETEEK	S5	1.006	0.913
Nolc1	Nucleolar and coiled-body phosphoprotein 1 (Fragment)	A0A286YDV7	S646	VADNSFDAK	S5	1.054	0.313
Nolc1	Nucleolar and coiled-body phosphoprotein 1 (Fragment)	A0A286YDV7	T212	AEAKPGTPAK	T7	1.082	0.042
Nolc1	Nucleolar and coiled-body phosphoprotein 1 (Fragment)	A0A286YDV7	T594	QNETADEATTPQAK	T4	1.163	0.232
Nolc1	Nucleolar and coiled-body phosphoprotein 1 (Fragment)	A0A286YDV7	T594	KQNETADEATTPQAK	T5	0.999	0.986
Nomo1	Nodal modulator 1	Q6GQT9	Ambiguous	ALGQAASDSSGPEDMKR	S	1.141	0.240
Nomo1	Nodal modulator 1	Q6GQT9	Ambiguous	ALGQAASDSSGPEDMK	S	0.989	0.863
Nop14	Nucleolar protein 14	Q8R3N1	S96	FGEYNSNISPEEK	S9	0.985	0.818
Nop2	Probable 28S rRNA (cytosine-C(5))-methyltransferase	E9QN31	T169	AQDATAGVLWNEEDTDEDEDDDGVSPESHPR	T15	1.131	0.035
Nop2	Probable 28S rRNA (cytosine-C(5))-methyltransferase	E9QN31	T707	VEDSRTPPVPTPSEIR	T6	1.007	0.935
Nop53	Ribosome biogenesis protein NOP53	Q8BK35	S305	EMCEGLLEESDGEDEHEAGR	S10	1.006	0.860
Nop53	Ribosome biogenesis protein NOP53	Q8BK35	S305	EMCEGLLEESDGEDEHEAGR	S10	1.007	0.971
Nop53	Ribosome biogenesis protein NOP53	Q8BK35	S329	AGQPEAGDGTTEISPTGAAGPEK	S14	1.081	0.222
Nop53	Ribosome biogenesis protein NOP53	Q8BK35	S329	AGQPEAGDGTTEISPTGAAGPEKR	S14	0.972	0.729
Nop56	Nucleolar protein 56	Q9D6Z1	S462	LAALALASSENSSTPEECEEVNEK	S9	0.964	0.330
Nop56	Nucleolar protein 56	Q9D6Z1	S513	EELASDLEEMATSSAKR	S5	0.950	0.540
Nop56	Nucleolar protein 56	Q9D6Z1	S513	EELASDLEEMATSSAK	S5	1.033	0.665
Nop56	Nucleolar protein 56	Q9D6Z1	S513	EELASDLEEMATSSAK	S5	1.134	0.443
Nop56	Nucleolar protein 56	Q9D6Z1	S536	EEVASEPEEAASPTTPK	S5	1.099	0.056
Nop56	Nucleolar protein 56	Q9D6Z1	S536	SSPKEEVASEPEEAASPTTPK	S9	1.031	0.426
Nop56	Nucleolar protein 56	Q9D6Z1	S536	KSSPKEEVASEPEEAASPTTPK	S10	1.042	0.340
Nop56	Nucleolar protein 56	Q9D6Z1	S536,S543	KSSPKEEVASEPEEAASPTTPK	S10,S17	1.057	0.412
Nop56	Nucleolar protein 56	Q9D6Z1	S536,S543	EEVASEPEEAASPTTPK	S5,S12	0.996	0.869
Nop56	Nucleolar protein 56	Q9D6Z1	S536,S543	SSPKEEVASEPEEAASPTTPK	S9,S16	1.018	0.211
Nop56	Nucleolar protein 56	Q9D6Z1	S536,S543	EEVASEPEEAASPTTPKK	S5,S12	0.955	0.510
Nop58	Nucleolar protein 58	Q6DFW4	S509	EEPLSEEEPCTSTAVPSPEK	S5	0.916	0.299
Nop58	Nucleolar protein 58	Q6DFW4	S509	EEPLSEEEPCTSTAVPSPEKK	S5	1.007	0.860
Nop58	Nucleolar protein 58	Q6DFW4	S509	HIKEEPLSEEEPCTSTAVPSPEKK	S8	1.038	0.463

Nop58	Nucleolar protein 58	Q6DFW4	S509	HIKEEPLSEEEPCTSTAVPSPEK	S8	1.103	0.408
Nop58	Nucleolar protein 58	Q6DFW4	S509,S521	EEPLSEEEPCTSTAVPSPEKK	S5,S17	1.168	0.426
Nop58	Nucleolar protein 58	Q6DFW4	S509,S521	EEPLSEEEPCTSTAVPSPEK	S5,S17	0.929	0.648
Nop9	Nucleolar protein 9	Q8BMC4	S631	ALSSILED	S3	1.785	0.001
Nos1ap	Carboxyl-terminal PDZ ligand of neuronal nitric oxide synthase protein	Q9D3A8	S248	DGGSHIDSTVSPHPQEPMLTASPR	S8	0.993	0.958
Nostrin	Nostrin	Q6WKZ7	S114	SLDNEVEK	S1	1.118	0.250
Nostrin	Nostrin	Q6WKZ7	Ambiguous	APAGGQNNPSSSPSGSTVSQASK	S/T	1.064	0.349
Nostrin	Nostrin	Q6WKZ7	Ambiguous	APAGGQNNPSSSPSGSTVSQASK	S/T	1.258	0.114
Notch2	Neurogenic locus notch homolog protein 2	G5E8J0	S1780	AEDEALLSEDDPIDR	S8	1.055	0.713
Npat	Protein NPAT	Q8BMA5	T1264	SADTDLPVPRTPGSGAGEK	T11	1.061	0.101
Npm1	Nucleophosmin	Q61937	S125	CGSGPVHISGQHLVAVEEDAESEDEDEEDVK	S22	1.464	0.487
Npm1	Nucleophosmin	Q61937	S241	GPSSVEDIK	S4	1.007	0.876
Nr2c2ap	Nuclear receptor 2C2-associated protein	Q3TV70	S22	VSSVLNRNSR	S9	1.096	0.204
Nr3c1	Glucocorticoid receptor	E9PYV1	T168	SSTPAAGCATPTEKEFPQTHSDPSSEQQNR	T10	1.127	0.194
Nr3c1	Glucocorticoid receptor	E9PYV1	T168	SSTPAAGCATPTEK	T10	1.057	0.276
Nr3c1	Glucocorticoid receptor	E9PYV1	Ambiguous	VSASSPSVAAASQADSK	S	1.014	0.883
Nras	GTPase NRas	P08556	T122	CDLPTRTVDTK	T5	1.120	0.277
Nrbf2	Nuclear receptor-binding factor 2	Q8VCQ3	S112	DGAPHLQAPPRPSEDAEGQSPLLSQPYIPSTER	S20	0.845	0.173
Nrbp1	Nuclear receptor-binding protein	D3YUV1	T441	TPTPEPAEVETR	T3	1.020	0.650
Nrbp1	Nuclear receptor-binding protein	D3YUV1	Ambiguous	NSTLNTATVTVSS	S/T	1.052	0.798
Nrd1	Nardilysin, N-arginine dibasic convertase, NRD convertase 1	A2A9Q2	S60	STCSCPDLQPNGQDLGESGR	S4	1.127	0.586
Nrd1	Nardilysin, N-arginine dibasic convertase, NRD convertase 1	A2A9Q2	S85	LGADESEEEGR	S6	1.016	0.407
Nrd1	Nardilysin, N-arginine dibasic convertase, NRD convertase 1	A2A9Q2	Ambiguous	LGADESEEEGRSFSNVGDPEIIK	S	1.012	0.909
Nrde2	Protein NRDE2 homolog	Q80XC6	S54	QTEEVTALASEGSPPPR	S13	1.011	0.854
Nsd1	Histone-lysine N-methyltransferase, H3 lysine-36 and H4 lysine-20-specific	E9QAE4	S2232	EYVPPTATSPPSPGTQPK	S12	0.876	0.175
Nsd1	Histone-lysine N-methyltransferase, H3 lysine-36 and H4 lysine-20-specific	E9QAE4	S2472	AASPQEVTPQADEK	S3	1.046	0.463
Nsd1	Histone-lysine N-methyltransferase, H3 lysine-36 and H4 lysine-20-specific	E9QAE4	S2572	TPVGAEQTPGPPSPAPGLVK	S13	0.884	0.174
Nsd1	Histone-lysine N-methyltransferase, H3 lysine-36 and H4 lysine-20-specific	E9QAE4	Ambiguous	DTPSSEGELLIHR	S/T	0.890	0.209
Nsd2	Histone-lysine N-methyltransferase NSD2	D3Z3E4	S408	SSSAENQEGDPGTDKSTPPK	S3	0.901	0.442
Nsd2	Histone-lysine N-methyltransferase NSD2	D3Z3E4	S408	SSSAENQEGDPGTDK	S3	1.080	0.235
Nsd2	Histone-lysine N-methyltransferase NSD2	D3Z3E4	S618	SSSPSASLTEHEVK	S3	1.073	0.313
Nsd2	Histone-lysine N-methyltransferase NSD2	D3Z3E4	S408,T418	SSSAENQEGDPGTDKSTPPKMAEAEPK	S3,T13	0.960	0.630
Nsd2	Histone-lysine N-methyltransferase NSD2	D3Z3E4	T115,T	GIGTPPNTTPIK	T9,T	0.942	0.620
Nsd2	Histone-lysine N-methyltransferase NSD2	D3Z3E4	Ambiguous	RSSSAENQEGDPGTDK	S/T	0.990	0.915
Nsd3	Histone-lysine N-methyltransferase NSD3	D3Z357	S1440	CKWESQDSGEEVKE	S8	1.024	0.711
Nsd3	Histone-lysine N-methyltransferase NSD3	D3Z357	S434	SVLNSQPEQTNAGEVASSQSSTDLR	S1	1.333	0.162
Nsd3	Histone-lysine N-methyltransferase NSD3	D3Z357	S681	QVDSPSATADADASDAQSVDSSLSR	S4	0.987	0.831
Nsd3	Histone-lysine N-methyltransferase NSD3	D3Z357	Ambiguous	SEKPAQSASSPEATSGSAGPVEK	S/T	0.972	0.714
Nsd3	Histone-lysine N-methyltransferase NSD3	D3Z357	Ambiguous	SEKPAQSASSPEATSGSAGPVEKK	S/T	1.093	0.404
Nsd3	Histone-lysine N-methyltransferase NSD3	D3Z357	Ambiguous	LIISSPSQR	S	0.984	0.830
Nsd3	Histone-lysine N-methyltransferase NSD3	D3Z357	Ambiguous	HTSLEEEEPPPVK	T/S	1.118	0.389
Nsd3	Histone-lysine N-methyltransferase NSD3	D3Z357	Ambiguous	RHTSLEEEEPPPVK	T/S	1.081	0.693
Nsf	Vesicle-fusing ATPase	P46460	S739	EEGASPLDFD	S5	1.130	0.401
Nsfl1c	NSFL1 cofactor p47	Q9CZ44	S140	SPGETSKPRPFAGGGYR	S1	1.058	0.518
Nsfl1c	NSFL1 cofactor p47	Q9CZ44	S165	LGAAPEEESAYVAGER	S9	1.059	0.410
Nsfl1c	NSFL1 cofactor p47	Q9CZ44	S165	LGAAPEEESAYVAGERR	S9	0.905	0.089
Nsfl1c	NSFL1 cofactor p47	Q9CZ44	S176	RHSGQDVHVVLK	S3	0.999	0.989
Nsfl1c	NSFL1 cofactor p47	Q9CZ44	S99	FYAGGSER	S6	0.899	0.036
Nsfl1c	NSFL1 cofactor p47	Q9CZ44	Ambiguous	LGSTAPQVLNTSSPAQQAENEAK	S/T	1.003	0.956
Nsmce4a	MCG1618, isoform CRA_c	G3XA30	Ambiguous	TFEISEPVIPLSQSQQR	S/T	0.966	0.796
Nsrp1	Nuclear speckle splicing regulatory protein 1	Q5NCR9	S254	GAQEAEENPDADREFDDESSEDGEKR	S19	0.925	0.212
Nsrp1	Nuclear speckle splicing regulatory protein 1	Q5NCR9	S254,S255	GAQEAEENPDADREFDDESSEDGEKR	S19,S20	1.001	0.989
Nsun2	tRNA (cytosine(34)-C(5))-methyltransferase	Q1HFZ0	S23	FQQPPQPEGEEDASDGGRK	S14	0.864	0.244
Nsun2	tRNA (cytosine(34)-C(5))-methyltransferase	Q1HFZ0	S23	FQQPPQPEGEEDASDGGRKR	S14	0.926	0.622
Nsun2	tRNA (cytosine(34)-C(5))-methyltransferase	Q1HFZ0	S23	FQQPPQPEGEEDASDGGR	S14	1.012	0.698
Nsun2	tRNA (cytosine(34)-C(5))-methyltransferase	Q1HFZ0	S23	RFQQPPQPEGEEDASDGGR	S15	1.043	0.410
Nsun2	tRNA (cytosine(34)-C(5))-methyltransferase	Q1HFZ0	S592	NNSGEEFDCAFR	S3	0.815	0.220
Nsun2	tRNA (cytosine(34)-C(5))-methyltransferase	Q1HFZ0	S723	EGVILTNENAASPEQPGDEDAK	S12	1.007	0.844
Nsun2	tRNA (cytosine(34)-C(5))-methyltransferase	Q1HFZ0	S723	KEGVILTNENAASPEQPGDEDAK	S13	1.057	0.335
Nsun2	tRNA (cytosine(34)-C(5))-methyltransferase	Q1HFZ0	S723	KKEGVILTNENAASPEQPGDEDAK	S14	0.946	0.643
Nsun2	tRNA (cytosine(34)-C(5))-methyltransferase	Q1HFZ0	S744	QTAQDPCVPDSVPGCDAAAAEPSR	S11	0.867	0.227
Nucb1	Nucleobindin-1	Q02819	S368	LSQETEALGR	S2	0.993	0.806
Nucb1	Nucleobindin-1	Q02819	S368	AQRLSQETEALGR	S5	1.027	0.070
Nucb1	Nucleobindin-1	Q02819	S440	ADTDDAPVPAPAGDQKDVPASEK	S21	0.952	0.539
Nucb1	Nucleobindin-1	Q02819	S456	VPEQPPELPQLDSQHL	S13	1.024	0.869
Nucb1	Nucleobindin-1	Q02819	S456	KVPEQPPELPQLDSQHL	S14	1.015	0.817
Nucb1	Nucleobindin-1	Q02819	T147	QFEHLDPQNQHTFEAR	T12	0.959	0.350
Nucb1	Nucleobindin-1	Q02819	Ambiguous	AAPPQEDSQATETPDTGLYYHR	T/S/Y	0.939	0.553
Nucks1	Nuclear ubiquitous casein and cyclin-dependent kinase substrate 1	Q80XU3	S113	EMLLEDVGSEEEPEEDDEAPFQEK	S9	1.097	0.250
Nucks1	Nuclear ubiquitous casein and cyclin-dependent kinase substrate 1	Q80XU3	S181	ATVTPSPVK	S6	1.058	0.341
Nucks1	Nuclear ubiquitous casein and cyclin-dependent kinase substrate 1	Q80XU3	S19	VVDYSQFQESDDADEDYGR	S10	0.982	0.682
Nucks1	Nuclear ubiquitous casein and cyclin-dependent kinase substrate 1	Q80XU3	S19	VVDYSQFQESDDADEDYGRDSGPPAKK	S10	0.975	0.892
Nucks1	Nuclear ubiquitous casein and cyclin-dependent kinase substrate 1	Q80XU3	S19	VVDYSQFQESDDADEDYGRDSGPPAK	S10	0.896	0.177
Nucks1	Nuclear ubiquitous casein and cyclin-dependent kinase substrate 1	Q80XU3	S19	KVVDYSQFQESDDADEDYGR	S11	1.021	0.834
Nucks1	Nuclear ubiquitous casein and cyclin-dependent kinase substrate 1	Q80XU3	S19	KVVDYSQFQESDDADEDYGRDSGPPAK	S11	0.979	0.861
Nucks1	Nuclear ubiquitous casein and cyclin-dependent kinase substrate 1	Q80XU3	S214	TPSPKEEDEEAESPPEKK	S13	0.992	0.912
Nucks1	Nuclear ubiquitous casein and cyclin-dependent kinase substrate 1	Q80XU3	S214	EEDEEAESPPEKK	S8	1.010	0.923
Nucks1	Nuclear ubiquitous casein and cyclin-dependent kinase substrate 1	Q80XU3	S54	SGKNSQEDSEDSEEK	S5	1.063	0.472
Nucks1	Nuclear ubiquitous casein and cyclin-dependent kinase substrate 1	Q80XU3	S58	NSQEDSEDSEEK	S6	0.837	0.272
Nucks1	Nuclear ubiquitous casein and cyclin-dependent kinase substrate 1	Q80XU3	S58	NSQEDSEDSEEKDVK	S6	1.005	0.931
Nucks1	Nuclear ubiquitous casein and cyclin-dependent kinase substrate 1	Q80XU3	S58	SGKNSQEDSEDSEEKDVK	S9	0.891	0.187
Nucks1	Nuclear ubiquitous casein and cyclin-dependent kinase substrate 1	Q80XU3	S79	DDSHSAEDSEDEKDDHK	S9	0.989	0.797
Nucks1	Nuclear ubiquitous casein and cyclin-dependent kinase substrate 1	Q80XU3	S79	DDSHSAEDSEDEKDDHKNVR	S9	0.922	0.105
Nucks1	Nuclear ubiquitous casein and cyclin-dependent kinase substrate 1	Q80XU3	S181,T	ATVTPSPVKGK	S6,T	1.458	0.009
Nucks1	Nuclear ubiquitous casein and cyclin-dependent kinase substrate 1	Q80XU3	S54,S58	SGKNSQEDSEDSEEK	S5,S9	0.934	0.245
Nucks1	Nuclear ubiquitous casein and cyclin-dependent kinase substrate 1	Q80XU3	S54,S58	SGKNSQEDSEDSEEKDVK	S5,S9	0.943	0.417
Nucks1	Nuclear ubiquitous casein and cyclin-dependent kinase substrate 1	Q80XU3	S58,S61	NSQEDSEDSEEKDVK	S6,S9	1.012	0.874
Nucks1	Nuclear ubiquitous casein and cyclin-dependent kinase substrate 1	Q80XU3	S75,S79	DDSHSAEDSEDEKDDHKNVR	S5,S9	0.928	0.301
Nucks1	Nuclear ubiquitous casein and cyclin-dependent kinase substrate 1	Q80XU3	S75,S79	DDSHSAEDSEDEKDDHK	S5,S9	1.011	0.880
Nucks1	Nuclear ubiquitous casein and cyclin-dependent kinase substrate 1	Q80XU3	S75,S79	KDDSHSAEDSEDEKDDHK	S6,S10	1.014	0.823
Nucks1	Nuclear ubiquitous casein and cyclin-dependent kinase substrate 1	Q80XU3	S54,S58,S61	SGKNSQEDSEDSEEKDVK	S5,S9,S12	0.918	0.302
Nucks1	Nuclear ubiquitous casein and cyclin-dependent kinase substrate 1	Q80XU3	Ambiguous	KDDSHSAEDSEDEKDDHK	S	0.988	0.933
Nudc	Nuclear migration protein nudC	O35685	S139	NGSLDSPGKQDAEDEEDEEDEKDK	S6	0.977	0.854
Nudc	Nuclear migration protein nudC	O35685	T108	AETPGPQIKELTDEEAER	T12	0.965	0.440
Nudc	Nuclear migration protein nudC	O35685	T99	AETPGPQIK	T3	1.109	0.097
Nudc	Nuclear migration protein nudC	O35685	Ambiguous	LVTSDPEINTK	T/S	0.938	0.579
Nudt5	ADP-sugar pyrophosphatase	Q9JKX6	Ambiguous	ESTESSPGKHLVTSEELISEGK	S/T	0.900	0.410
Nufip2	Nuclear fragile X mental retardation-interacting protein 2	Q5F2E7	S113	SLSSDEATNPISR	S3	0.924	0.102
Nufip2	Nuclear fragile X mental retardation-interacting protein 2	Q5F2E7	S213	GADNDGSGSESGYTTPK	S7	0.991	0.820
Nufip2	Nuclear fragile X mental retardation-interacting protein 2	Q5F2E7	S649	NDSWGSFDLR	S3	1.005	0.939
Nufip2	Nuclear fragile X mental retardation-interacting protein 2	Q5F2E7	S689	IITYNEAMDSPDQ	S10	0.992	0.970
Nufip2	Nuclear fragile X mental retardation-interacting protein 2	Q5F2E7	S213,S215	GADNDGSGSESGYTTPK	S7,S9	0.956	0.292
Nufip2	Nuclear fragile X mental retardation-interacting protein 2	Q5F2E7	Ambiguous	GADNDGSGSESGYTTPKK	S/Y/T	0.961	0.765
Numa1	Nuclear mitotic apparatus protein 1	E9Q7G0	S167	VPYTHASTLSEELSPPSHQTK	S14	0.829	0.026
Numa1	Nuclear mitotic apparatus protein 1	E9Q7G0	S1739	TQPDGTSVPGEPASPISQR	S14	1.077	0.046
Numa1	Nuclear mitotic apparatus protein 1	E9Q7G0	S1951	RASMQPAQIAEGVGITTR	S3	1.167	0.432
Numa1	Nuclear mitotic apparatus protein 1	E9Q7G0	S270	QAASSQEPSELEELRGK	S5	0.893	0.138
Numa1	Nuclear mitotic apparatus protein 1	E9Q7G0	S270	QAASSQEPSELEELR	S5	0.970	0.533
Numa1	Nuclear mitotic apparatus protein 1	E9Q7G0	S398	LQESPAEK	S4	1.031	0.662
Numa1	Nuclear mitotic apparatus protein 1	E9Q7G0	T1982	VSSETHQGPGTPESK	T11	1.098	0.062
Numa1	Nuclear mitotic apparatus protein 1	E9Q7G0	T2085	IATTTTGTATVATTPR	T14	0.977	0.610
Numa1	Nuclear mitotic apparatus protein 1	E9Q7G0	S1732,S1739	TQPDGTSVPGEPASPISQR	S7,S14	0.929	0.345
Numa1	Nuclear mitotic apparatus protein 1	E9Q7G0	T1976,T1982	VSSETHQGPGTPESK	T5,T11	1.082	0.528
Numb	Protein numb homolog	Q9QZS3	S194	EGSFRVTTATEQAER	S3	1.033	0.737
Numb	Protein numb homolog	Q9QZS3	T633	TNPSPTNPFSSDLQK	T1	1.055	0.780
Nup107	Nuclear pore complex protein Nup107	Q8BH74	S11	SGFGGMSSPVIR	S8	0.978	0.726
Nup107	Nuclear pore complex protein Nup107	Q8BH74	S87	HPDISYILGTEGRSPR	S14	0.980	0.722
Nup107	Nuclear pore complex protein Nup107	Q8BH74	Ambiguous	SGFGGMSSPVIR	S	1.013	0.851
Nup107	Nuclear pore complex protein Nup107	Q8BH74	Ambiguous	VLIQANQEDNFGTATPR	T	1.171	0.067
Nup133	Nuclear pore complex protein Nup133	Q8R0G9	S500	GSESMVFETTTK	S4	0.987	0.836
Nup133	Nuclear pore complex protein Nup133	Q8R0G9	Ambiguous	RGPLVGIGPTSTPR	S/T	1.019	0.827
Nup153	Nucleoporin 153	E9Q3G8	S210	NTSLPPLWSPEAER	S9	1.052	0.490
Nup153	Nucleoporin 153	E9Q3G8	S258	TSQLGDSPFYPGK	S7	1.045	0.644
Nup153	Nucleoporin 153	E9Q3G8	T594	SAPCEDPFTPAK	T9	1.040	0.609
Nup153	Nucleoporin 153	E9Q3G8	S204,S210	NTSLPPLWSPEAER	S3,S9	0.944	0.828
Nup153	Nucleoporin 153	E9Q3G8	Ambiguous	EIKDSTSQHDDDNISTTSGFSSR	S/T	0.940	0.154
Nup153	Nucleoporin 153	E9Q3G8	Ambiguous	DSTSQHDDDNISTTSGFSSR	T/S	1.243	0.050
Nup155	Nuclear pore complex protein Nup155	Q99P88	S992	AAPQSPSVPK	S5	1.036	0.533
Nup188	Nucleoporin NUP188 homolog	Q6ZQH8	S1719	GAPSSPAAGVLPSPQGK	S5	1.052	0.361
Nup188	Nucleoporin NUP188 homolog	Q6ZQH8	S1727,S	GAPSSPAAGVLPSPQGK	S13,S	0.986	0.796
Nup210	Nuclear pore membrane glycoprotein 210	Q9QY81	T1844	ELASPLALTPR	T9	1.111	0.245
Nup210	Nuclear pore membrane glycoprotein 210	Q9QY81	S1839,T1844	ELASPLALTPR	S4,T9	1.073	0.440
Nup214	Nuclear pore complex protein Nup214	Q80U93	S1139	SNPSPPSAAMGSAVQHSAAK	S4	0.974	0.602
Nup214	Nuclear pore complex protein Nup214	Q80U93	S1139	SNPSPPSAAMGSAVQHSAAK	S4	0.992	0.946
Nup214	Nuclear pore complex protein Nup214	Q80U93	S492	SSASVTGEPPLYPTGSDSSR	S4	1.301	0.009
Nup214	Nuclear pore complex protein Nup214	Q80U93	S660	STQTAPSSAPSTGQKSPR	S16	1.051	0.557
Nup35	Nucleoporin NUP35	Q8R4R6	T106	SIYDDISSPGLGSTPLTSR	T14	1.013	0.927
Nup35	Nucleoporin NUP35	Q8R4R6	T272	TLGTPTQSGSTPR	T4	1.028	0.666

Nup35	Nucleoporin NUP35	Q8R4R6	T307	ASTSDYQVISDRQTPK	T14	1.078	0.182
Nup50	Nuclear pore complex protein Nup50	Q9JIH2	Ambiguous	NVGFESDSGGAFK	S	1.193	0.306
Nup54	Nuclear pore complex protein Nup54	Q8BTS4	S292	TELSPAQIK	S4	0.958	0.689
Nup85	Nuclear pore complex protein Nup85	Q8R480	S90	IEEELSGK	S6	0.933	0.690
Nup88	Nuclear pore complex protein Nup88	Q8CEC0	T526	ILAETPDSFEK	T5	0.912	0.403
Nup88	Nuclear pore complex protein Nup88	Q8CEC0	Ambiguous	NQSPAEAEKPAASTSPSCPSLPPHLPTR	S/T	1.002	0.983
Nup93	Nuclear pore complex protein Nup93	Q8BJ71	S180	SSLDSIEMAYAR	S5	1.077	0.531
Nup93	Nuclear pore complex protein Nup93	Q8BJ71	S180	SSLDSIEMAYAR	S5	1.090	0.634
Nup93	Nuclear pore complex protein Nup93	Q8BJ71	S52	TSQETADVK	S2	1.077	0.270
Nup93	Nuclear pore complex protein Nup93	Q8BJ71	T51	TLTRTSQETADVK	T5	0.997	0.939
Nup93	Nuclear pore complex protein Nup93	Q8BJ71	Ambiguous	LNQVCFDDDGTSSPQDR	T/S	1.438	0.300
Nup98	Nuclear pore complex protein Nup98-Nup96	A0A1B0GSX7	S1059	FASGTFLSPSASVQECR	S8	0.972	0.926
Nup98	Nuclear pore complex protein Nup98-Nup96	A0A1B0GSX7	S1815	ELTVGSQ	S6	1.021	0.122
Nup98	Nuclear pore complex protein Nup98-Nup96	A0A1B0GSX7	S623	NLNNSNLFSPVNHDSEDLASPSEYPENGER	S20	0.864	0.245
Nup98	Nuclear pore complex protein Nup98-Nup96	A0A1B0GSX7	S888	YGLQDSDEEEEEHPPK	S6	1.089	0.316
Nup98	Nuclear pore complex protein Nup98-Nup96	A0A1B0GSX7	Ambiguous	NNSSSNVEDTIVALNMR	S/T	0.761	0.101
Nup98	Nuclear pore complex protein Nup98-Nup96	A0A1B0GSX7	Ambiguous	ALTTPTHYK	T/Y	1.053	0.554
Nusap1	Nucleolar and spindle-associated protein 1	Q9ERH4	Ambiguous	DTAEVPSLPEQRPEDGNAASSGEGEVNDIK	S/T	1.106	0.380
Nvl	Nuclear valosin-containing protein-like	Q9DBY8	S190	ESLPLDLSDDQSNSK	S8	1.021	0.508
Ofd1	Oral-facial-digital syndrome 1 gene homolog (Human)	A2AEG2	S749	SLDNEMYLEGLGR	S1	1.175	0.273
Ofd1	Oral-facial-digital syndrome 1 gene homolog (Human)	A2AEG2	Ambiguous	ATQNPSTSPQPAK	T/S	0.943	0.588
Ogfr	Opioid growth factor receptor	Q99PG2	S403	IALNLEGCALSPTSQEPR	S11	0.945	0.738
Ogfr	Opioid growth factor receptor	Q99PG2	S545	GQVGPEDPKSQVGPEDPK	S10	1.018	0.663
Ogfr	Opioid growth factor receptor	Q99PG2	S563	SQVEPEDPKSQVEPEDPK	S10	0.980	0.831
Ogfr	Opioid growth factor receptor	Q99PG2	S608	SLGEDPDSDTTGTSMSESEELAR	S8	1.087	0.243
Ogfr	Opioid growth factor receptor	Q99PG2	S616	SLGEDPDSDTTGTSMSESEELAR	S16	0.557	0.034
Ogfrl1	Opioid growth factor receptor-like protein 1	Q8VE52	S65	ASPVPEDHAEAAGAEQGGESTEGNAKPK	S2	1.109	0.054
Ophn1	Oligophrenin-1	Q99J31	S652	SGETDPGRKSPSRPVSDCQSEPCLETDVGR	S10	0.984	0.785
Ophn1	Oligophrenin-1	Q99J31	S652	SPSRPVSDCQSEPCLETDVGR	S1	1.141	0.095
Optn	Optineurin	Q8K3K8	S187	LNSGGSSEDSFVEIR	S10	1.138	0.156
Optn	Optineurin	Q8K3K8	S218	TDPISLSNCTEDAR	S5	1.104	0.466
Optn	Optineurin	Q8K3K8	S530	TSDSDQQTYLFQR	S2	1.050	0.451
Optn	Optineurin	Q8K3K8	T210	NCPTPTRTDPISLSNCTEDAR	T4	1.102	0.057
Optn	Optineurin	Q8K3K8	Ambiguous	HGARTSDSDQQTYLFQR	T/S/Y	1.030	0.581
Orai1	Calcium release-activated calcium channel protein 1	Q8BWG9	S296	GDHSLTPGTHYA	S4	1.146	0.348
Orai3	Protein orai-3	Q6P8G8	S20	GGEGDTGEQAPLNPEVDSPAGSATYR	S18	1.036	0.569
Os9	Protein OS-9	Q8K2C7	S514	LQENQSPELVQK	S6	0.960	0.760
Osbp	Oxysterol-binding protein 1	Q3B7Z2	S191	MLAESDDSGDEESVSQTDKTELQSTLR	S8	0.965	0.790
Osbp	Oxysterol-binding protein 1	Q3B7Z2	S380	TGSNISGASSDVSLDEQYK	S6	0.943	0.352
Osbp	Oxysterol-binding protein 1	Q3B7Z2	S800	QDWGSCPDIF	S5	0.785	0.443
Osbp	Oxysterol-binding protein 1	Q3B7Z2	S188,S191	MLAESDDSGDEESVSQTDK	S5,S8	0.958	0.514
Osbp	Oxysterol-binding protein 1	Q3B7Z2	S188,S191	MLAESDDSGDEESVSQTDKTELQSTLR	S5,S8	1.176	0.298
Osbp	Oxysterol-binding protein 1	Q3B7Z2	S380,T/S/Y	TGSNISGASSDVSLDEQYK	S6,T/S/Y	0.998	0.965
Osbp	Oxysterol-binding protein 1	Q3B7Z2	S380,T/S/Y	RTGSNISGASSDVSLDEQYK	S7,T/S/Y	0.995	0.937
Osbp	Oxysterol-binding protein 1	Q3B7Z2	Ambiguous	RTGSNISGASSDVSLDEQYK	S/T/Y	0.937	0.418
Osbpl11	Oxysterol-binding protein	G5E8A0	S192	SFSLASSGNSPISQR	S10	1.021	0.566
Osbpl11	Oxysterol-binding protein	G5E8A0	S183,S192	SRSFSLASSGNSPISQR	S3,S12	0.989	0.894
Osbpl11	Oxysterol-binding protein	G5E8A0	S185,S192	SFSLASSGNSPISQR	S3,S10	1.076	0.147
Osbpl3	Oxysterol-binding protein	D3YTT6	S191	QNSFPPGSNLSFSCGGDTR	S3	1.177	0.071
Osbpl3	Oxysterol-binding protein	D3YTT6	S303	LHSSNPNLSTLDFGEEK	S4	0.961	0.499
Osbpl3	Oxysterol-binding protein	D3YTT6	S319	SYSDGSEASSEFSK	S3	0.960	0.125
Osbpl3	Oxysterol-binding protein	D3YTT6	S33	QDSWEVVEGLR	S3	1.418	0.022
Osbpl3	Oxysterol-binding protein	D3YTT6	S33	QGSRQDSWEVVEGLR	S7	1.125	0.161
Osbpl3	Oxysterol-binding protein	D3YTT6	S504	QTLGPVLESSGEAR	S10	0.983	0.719
Osbpl3	Oxysterol-binding protein	D3YTT6	T45,Y46,T47	GEMTYTQEPPVQK	T4,Y5,T6	1.162	0.017
Osbpl3	Oxysterol-binding protein	D3YTT6	Ambiguous	ALVHQLSNESR	S	1.188	0.180
Osbpl3	Oxysterol-binding protein	D3YTT6	Ambiguous	TYSAPAINAIQGGAFESPK	T/S/Y	0.884	0.387
Osbpl3	Oxysterol-binding protein	D3YTT6	Ambiguous	SYSDGSEASSEFSK	S/Y	0.934	0.344
Osbpl5	Oxysterol-binding protein	G5E833	S770	ETMSGQTTFLGSPDSR	S12	1.221	0.079
Osbpl7	Oxysterol-binding protein	A2A716	S393	LHGSVPNLSR	S4	1.027	0.482
Osbpl8	Oxysterol-binding protein-related protein 8	B9EJ86	S105	LYNGSEKDSSTSSK	S5	0.978	0.749
Osbpl8	Oxysterol-binding protein-related protein 8	B9EJ86	S314	ANNLHSGDNFQLNDSEIER	S15	1.047	0.188
Osbpl8	Oxysterol-binding protein-related protein 8	B9EJ86	S65,S68	DLPQPSLSPASLHSQGFER	S8,S11	1.187	0.110
Osbpl8	Oxysterol-binding protein-related protein 8	B9EJ86	T54,S65	DANPTPTRDLPQPSLSPASLHSQGFER	T5,S16	0.975	0.877
Osbpl8	Oxysterol-binding protein-related protein 8	B9EJ86	S/Y/T	GYSSPEPDIQDSSGSEAQSVKPSTR	S/Y/T	1.109	0.702
Osbpl8	Oxysterol-binding protein-related protein 8	B9EJ86	Ambiguous	GYSSPEPDIQDSSGSEAQSVKPSTR	S/Y/T	1.170	0.085
Osbpl8	Oxysterol-binding protein-related protein 8	B9EJ86	Ambiguous	DLPQPSLSPASLHSQGFER	S	1.098	0.369
Osbpl8	Oxysterol-binding protein-related protein 8	B9EJ86	Ambiguous	GYSSPEPDIQDSSGSEAQSVKPSTR	S/Y/T	1.055	0.167
Ostf1	Osteoclast-stimulating factor 1	Q62422	S203	TLSNAEDYLDDEDSD	S3	0.908	0.021
Ostm1	Osteopetrosis-associated transmembrane protein 1	Q8BGT0	S329	SSTSFANIQENAT	S4	1.048	0.453
Otol1	Otolin-1	Q4ZJM7	T194	GTIGPAGTK	T2	1.013	0.879
Otud4	OTU domain-containing protein 4	B2RRE7	S1000	TAADVVSPGANSVDR	S7	1.078	0.166
Otud4	OTU domain-containing protein 4	B2RRE7	S442	ESYYFGLSPEER	S8	0.969	0.859
Otud5	OTU domain-containing protein 5	A2AES4	S177	QAPGVGAVGGASPEREEVGAGYNSEDEYEAAAAR	S24	1.003	0.971
Otud5	OTU domain-containing protein 5	A2AES4	S508	ATSPLVSLYPALECR	S3	1.269	0.165
Otud5	OTU domain-containing protein 5	A2AES4	S64	ASPPPQGPLPGPPGALHR	S2	1.045	0.178
Otud5	OTU domain-containing protein 5	A2AES4	S165,S177	QAPGVGAVGGASPEREEVGAGYNSEDEYEAAAAR	S12,S24	1.057	0.568
Otud6b	Deubiquitinase OTUD6B	A0A0A0MQF5	S210	EQDCALTVASLRR	S10	0.959	0.474
Otud7b	OTU domain-containing protein 7B	B2RUR8	S449	WIPLSSDSQAPLAQPESPTASAGDEPR	S17	0.711	0.192
Otud7b	OTU domain-containing protein 7B	B2RUR8	S467	STPESGESDSKESVGSSSLGNEGSR	S8	0.992	0.921
Oxr1	Oxidation resistance protein 1	E9Q0A7	S195	VVSSTSEEEEAFTEK	S4	1.097	0.056
Oxr1	Oxidation resistance protein 1	E9Q0A7	S481	RDSETEVEELR	S3	1.051	0.621
Oxr1	Oxidation resistance protein 1	E9Q0A7	S481	DQQGQELKRDSETEVEELR	S11	0.985	0.736
Oxr1	Oxidation resistance protein 1	E9Q0A7	S59	EPAPGDPGCGPEELRPPSPASPEGPDTGQKK	S18	0.974	0.500
Oxr1	Oxidation resistance protein 1	E9Q0A7	S59	EPAPGDPGCGPEELRPPSPASPEGPDTGQK	S18	1.080	0.507
Oxr1	Oxidation resistance protein 1	E9Q0A7	T196,S/T	VVSSTSEEEEAFTEK	T5,S/T	1.057	0.028
Oxsr1	Serine/threonine-protein kinase OSR1	Q6P9R2	S339	TEDGGWEWSDDEFDEESEEGR	S9	0.900	0.553
Oxsr1	Serine/threonine-protein kinase OSR1	Q6P9R2	S359	AAISQLRSPR	S8	1.206	0.000
P2rx4	P2X purinoceptor	Q9Z257	Ambiguous	YVEDYEQGLSGETDQ	S/T/Y	1.054	0.418
P2rx4	P2X purinoceptor	Q9Z257	Ambiguous	YKYVEDYEQGLSGETDQ	T/S/Y	0.817	0.351
P2ry10b	Purinergic receptor P2Y, G-protein-coupled 10B	Q8BY68	S337	ENLQTISLECLDVPTQCDEMVI	S7	1.387	0.042
P2ry14	P2Y purinoceptor 14	A0A0G2JEW9	S343	ENAIHESTDTL	S7	1.017	0.734
P2ry2	P2Y purinoceptor 2	A0A0R4J289	S329	DAKPPTEPTPSPQAR	S11	1.091	0.148
P2ry2	P2Y purinoceptor 2	A0A0R4J289	S352	DLSVSSDDSR	S5	1.146	0.094
P2ry2	P2Y purinoceptor 2	A0A0R4J289	T327,S329	DAKPPTEPTPSPQAR	T9,S11	0.869	0.102
P33monox	Putative monooxygenase p33MONOX	Q9DBN4	Ambiguous	SDSGFAIQAYK	S/Y	0.967	0.834
P4hb	Protein disulfide-isomerase	P09103	S90	VDATEESDLAQQYGVR	S7	0.944	0.822
Pa2g4	Proliferation-associated protein 2G4	P50580	S361	ALLQSSASR	S6	0.993	0.880
Pa2g4	Proliferation-associated protein 2G4	P50580	S375	KASKTVENATSGETLEENGAGD	S3	1.017	0.808
Pa2g4	Proliferation-associated protein 2G4	P50580	S383	TVENATSGETLEENGAGD	S7	0.927	0.266
Pabpc1	Polyadenylate-binding protein 1	P29341	S629	AVNSATGVPTV	S4	0.840	0.414
Pabpc4	Polyadenylate-binding protein	Q6PHQ9	S660	VGTVAAATS	S9	0.984	0.593
Pacs1	Phosphofurin acidic cluster sorting protein 1	Q8K212	S28	GSGVAQSPQQQPQQQPPQPQQPTPPK	S7	0.936	0.281
Pacs1	Phosphofurin acidic cluster sorting protein 1	Q8K212	S493	ADLQGSASPSKVEGTHTPR	S8	0.960	0.184
Pacs1	Phosphofurin acidic cluster sorting protein 1	Q8K212	S532	TNSSDSERSPDLGHSTQIPR	S9	1.036	0.756
Pacs1	Phosphofurin acidic cluster sorting protein 1	Q8K212	S493,T502	ADLQGSASPSKVEGTHTPR	S8,T17	0.953	0.310
Pacs1	Phosphofurin acidic cluster sorting protein 1	Q8K212	S532,S/T	TNSSDSERSPDLGHSTQIPR	S9,S/T	0.976	0.886
Pacs1	Phosphofurin acidic cluster sorting protein 1	Q8K212	Ambiguous	DTTSPMELAALEK	T/S	1.214	0.138
Pacs2	Phosphofurin acidic cluster sorting protein 2	A0A1Y7VLZ7	S349	SHREPPSPADVPEK	S7	1.133	0.030
Pacs2	Phosphofurin acidic cluster sorting protein 2	A0A1Y7VLZ7	S391	EPSGQPEDSPEAETSTLDVFTEK	S9	1.119	0.249
Pacs2	Phosphofurin acidic cluster sorting protein 2	A0A1Y7VLZ7	S417	TESLVIPSTR	S3	1.001	0.967
Pacs2	Phosphofurin acidic cluster sorting protein 2	A0A1Y7VLZ7	S454	ANSLDNER	S3	1.047	0.450
Pacs2	Phosphofurin acidic cluster sorting protein 2	A0A1Y7VLZ7	S454	ANSLDNERCPDTR	S3	1.026	0.750
Pacsin3	Protein kinase C and casein kinase II substrate protein 3	Q99JB8	S354	DGTAPPPQSPSSPGSGQDEDWSDEESPR	S22	1.042	0.587
Pacsin3	Protein kinase C and casein kinase II substrate protein 3	Q99JB8	S354	DGTAPPPQSPSSPGSGQDEDWSDEESPRK	S22	1.081	0.564
Pag1	Phosphoprotein associated with glycosphingolipid-enriched microdomains 1	Q3U1F9	S349	SSSSCNDLYATVK	S2	0.968	0.456
Pag1	Phosphoprotein associated with glycosphingolipid-enriched microdomains 1	Q3U1F9	S416	ESDYESIGDLQQCR	S6	1.223	0.116
Pag1	Phosphoprotein associated with glycosphingolipid-enriched microdomains 1	Q3U1F9	Y165	AADTELGPVEGPYEVLK	Y14	1.055	0.453
Pag1	Phosphoprotein associated with glycosphingolipid-enriched microdomains 1	Q3U1F9	Y183	DSSSQENMVEDCLYETVK	Y14	1.074	0.696
Pag1	Phosphoprotein associated with glycosphingolipid-enriched microdomains 1	Q3U1F9	Y224	ADFAEYASVDR	Y6	1.059	0.122
Pag1	Phosphoprotein associated with glycosphingolipid-enriched microdomains 1	Q3U1F9	S350,S351	SSSSCNDLYATVK	S3,S4	0.958	0.674
Pag1	Phosphoprotein associated with glycosphingolipid-enriched microdomains 1	Q3U1F9	Ambiguous	HSANAESILGTCSDLDEESPPPVPVK	S/T	0.936	0.275
Pagr1a	PAXIP1-associated glutamate-rich protein 1A	Q99L02	S236	DLFSLDSEGPSPTSPPLR	S14	1.027	0.613
Paics	Multifunctional protein ADE2	Q9DCL9	Y22	EVYELLDTPGR	Y3	0.928	0.567
Paip1	Polyadenylate-binding protein-interacting protein 1	F6Y616	S47	SQHQSPPPPQPK	S5	0.929	0.592
Pak1	Non-specific serine/threonine protein kinase	G5E884	S155	SAEDYNSSNTLNVK	S7	1.066	0.512
Pak1	Non-specific serine/threonine protein kinase	G5E884	S203	SVIEPLPVTPTR	S1	1.200	0.177
Pak1	Non-specific serine/threonine protein kinase	G5E884	S219	DVATSPISPTENNTTPPDALTR	S5	1.025	0.705
Pak1	Non-specific serine/threonine protein kinase	G5E884	Ambiguous	TVSETPAVPPVSEDEDDDDATPPPVIAPRPEHTK	S/T	1.016	0.750
Pak1ip1	p21-activated protein kinase-interacting protein 1	Q9DCE5	S361	GNSPVTAK	S3	1.057	0.399
Pak2	Serine/threonine-protein kinase PAK 2	Q8CIN4	S132	FYDSNTVK	S4	0.978	0.400
Pak2	Serine/threonine-protein kinase PAK 2	Q8CIN4	S141	YLSFTPPEK	S3	1.035	0.549
Pak2	Serine/threonine-protein kinase PAK 2	Q8CIN4	S152	DGFPSGTPALNTK	S5	1.042	0.709
Pak2	Serine/threonine-protein kinase PAK 2	Q8CIN4	S197	SVIDPIPAPVGDSNVDSGAK	S1	0.949	0.431
Pak2	Serine/threonine-protein kinase PAK 2	Q8CIN4	T169	GSETSAVVTEEDDDDEDAAPPVIAPRPDHTK	T9	0.984	0.803
Pak2	Serine/threonine-protein kinase PAK 2	Q8CIN4	S197,S213	SVIDPIPAPVGDSNVDSGAK	S1,S17	1.085	0.464
Pak2	Serine/threonine-protein kinase PAK 2	Q8CIN4	Ambiguous	SIYTRSVIDPIPAPVGDSNVDSGAK	T/S/Y	0.943	0.532
Pak4	Serine/threonine-protein kinase PAK 4	Q8BTW9	S104	RESPPPPAR	S3	0.962	0.507

Pak4	Serine/threonine-protein kinase PAK 4	Q8BTW9	S257	GAPSPGVLGPHASEPQLAPPAR	S4	1.158	0.034
Pak4	Serine/threonine-protein kinase PAK 4	Q8BTW9	S293	ALAAPAVPPAPGPPGPRSPQREPQR	S18	1.078	0.171
Pak4	Serine/threonine-protein kinase PAK 4	Q8BTW9	T207	LAAGRPFNTYPR	T9	0.977	0.869
Pak4	Serine/threonine-protein kinase PAK 4	Q8BTW9	S257,S266	GAPSPGVLGPHASEPQLAPPAR	S4,S13	0.872	0.484
Pakap	Paralemmin A kinase anchor protein (Fragment)	F7AA26	S396	ANGHSTDQPQDLLGNSLQAPASPSSSTSSHCSSR	S22	1.080	0.756
Pakap	Paralemmin A kinase anchor protein (Fragment)	F7AA26	S984	TLSMIEEEIR	S3	1.121	0.139
Pakap	Paralemmin A kinase anchor protein (Fragment)	F7AA26	S984	TLSMIEEEIR	S3	1.107	0.158
Palb2	Partner and localizer of BRCA2	Q3U0P1	S559	LIESPDSK	S4	1.071	0.635
Palld	Palladin	Q9ET54	S901	IASDEEIQGTK	S3	1.186	0.051
Palm	Paralemmin-1	Q9Z0P4	S116	ENSAAPSPGRPQSASPAKEEQK	S7	0.993	0.948
Palm	Paralemmin-1	Q9Z0P4	S345	EPAPLNGSAAELPATK	S8	1.145	0.069
Palm	Paralemmin-1	Q9Z0P4	T145	SETLVNAQQTPLGTPK	T14	0.969	0.612
Palm	Paralemmin-1	Q9Z0P4	T141,T145	SETLVNAQQTPLGTPKENR	T10,T14	0.972	0.425
Palm	Paralemmin-1	Q9Z0P4	T141,T145	SETLVNAQQTPLGTPK	T10,T14	0.987	0.884
Palm3	Paralemmin-3	A2TJV2	S139	EGPRPLSQSAMEAAPTAPTDVDKR	S7	1.122	0.098
Pan2	PAN2-PAN3 deadenylation complex catalytic subunit Pan2	Q8BGF7	S1188	VPEPESQTSPK	S9	0.982	0.704
Pan3	PAN2-PAN3 deadenylation complex subunit PAN3	H3BKF3	S354	ITPHTSPAPR	S6	1.035	0.122
Pank1	Pantothenate kinase 1	Q8K4K6	S11	RAPRPGSTEVPAAAR	S7	1.129	0.108
Pank1	Pantothenate kinase 1	Q8K4K6	S30	AASPVRGAQLAEDVGTPTGGGEER	S3	0.905	0.204
Pank2	Pantothenate kinase 2	Q3U4S0	S42	RASSAAPSGSGEAESVR	S3	0.938	0.586
Pank2	Pantothenate kinase 2	Q3U4S0	S43	ASSAAPSGSGEAESVRR	S3	0.937	0.153
Pank2	Pantothenate kinase 2	Q3U4S0	S43	ASSAAPSGSGEAESVR	S3	1.053	0.189
Pank2	Pantothenate kinase 2	Q3U4S0	S62	ERPGSLGGSTSAGRPR	S5	0.982	0.699
Pank4	Pantothenate kinase 4	Q80YV4	Ambiguous	SGTFDLLEMDR	S/T	1.330	0.180
Panx1	Pannexin-1	Q9JIP4	S405	DLDLSSEAAANNGEK	S5	0.994	0.928
Papola	Poly(A) polymerase alpha	Q61183	S545	TSPLNSSGSSQGR	S2	0.994	0.866
Papolg	Poly(A) polymerase gamma	Q6PCL9	Ambiguous	DTDSGTPFNSPVSANKPSNPDSPTGEIER	S/T	1.305	0.036
Pard3	Par-3 (Partitioning defective 3) homolog (C. elegans)	A5D6P2	Ambiguous	RSSDPALTGLSTSVSDNNFSSEEPSR	S/T	0.940	0.364
Pard6a	Partitioning defective 6 homolog alpha	A0A1D5RLU4; Q9Z101	S328;S345	GDVSGFSL	S7	0.887	0.477
Pard6a	Partitioning defective 6 homolog alpha	A0A1D5RLU4	S92	EGDSSGLAFASNSLQR	S13	1.050	0.685
Pard6a	Partitioning defective 6 homolog alpha	Q9Z101	S109	AEGDSSGLAFASNSLQR	S14	1.000	1.000
Pard6a	Partitioning defective 6 homolog alpha	A0A1D5RLU4; Q9Z101	Ambiguous	LTGPSSVGPGPTDPDSDDDSSDLVIENR	T/S	0.903	0.419
Parg	Poly(ADP-ribose) glycohydrolase	O88622	S311	NSCQDSEADEETSPVFDEQDDRSSQTANK	S6	0.874	0.618
Parg	Poly(ADP-ribose) glycohydrolase	O88622	S311	NSCQDSEADEETSPVFDEQDDR	S6	0.965	0.759
Parn	Poly(A)-specific ribonuclease PARN	A0A0R4J0P6	S573	EAALEDRESEEVSDSELEQTDSCTDPLPEGR	S13	1.196	0.240
Parp1	Poly [ADP-ribose] polymerase	Q921K2	S782	GGSDDSSKDPIDVNYEK	S3	1.018	0.851
Parp10	Poly [ADP-ribose] polymerase	Q8CIE4	T959	NILPGTP	T6	0.979	0.820
Parp10	Poly [ADP-ribose] polymerase	Q8CIE4	Ambiguous	AGPVGAPGQALPVDSGSIR	S	0.939	0.770
Parp14	Poly [ADP-ribose] polymerase 14	Q2EMV9	S1407	EGSPAPPKQSPAK	S10	1.050	0.515
Parp14	Poly [ADP-ribose] polymerase 14	Q2EMV9	Ambiguous	QGDLDITHSPSSGSEKTEDVPK	T/S	0.974	0.862
Parp2	Poly [ADP-ribose] polymerase 2	O88554	S32	ATEDDSPPGKK	S6	0.912	0.289
Parp3	Poly [ADP-ribose] polymerase	Q3ULW8	S167	ALSPQVYSGPVR	S3	0.817	0.547
Parp4	Poly [ADP-ribose] polymerase	E9PYK3	S1266	KCSLDISEDFEDR	S3	1.099	0.460
Parp4	Poly [ADP-ribose] polymerase	E9PYK3	S96	NYDPLSPAPAAPPAER	S6	0.917	0.432
Parp8	Poly [ADP-ribose] polymerase	F8WIK2	S330	RSPSYPPPGCGK	S4	1.026	0.658
Parp8	Poly [ADP-ribose] polymerase	F8WIK2	S472	SYSSAPK	S3	0.817	0.082
Parp9	Poly [ADP-ribose] polymerase 9	Q8CAS9	S42	GHSEGDYPPLR	S3	0.953	0.565
Parvb	Beta-parvin	Q9ES46	S52	SAINSPMAPALVDIHPEDTQLEENEER	S5	0.984	0.907
Pask	PAS domain-containing serine/threonine-protein kinase	Q8CEE6	S852	QTSDILVDR	S3	1.254	0.014
Patl1	Protein PAT1 homolog 1	Q3TC46	S179	STSPIIGSPPVR	S3	1.000	1.000
Patl1	Protein PAT1 homolog 1	Q3TC46	T194	AVPIGTPPK	T6	1.014	0.771
Patl1	Protein PAT1 homolog 1	Q3TC46	S179,S184	RSTSPIIGSPPVR	S4,S9	0.957	0.064
Patl1	Protein PAT1 homolog 1	Q3TC46	S184,S/T	STSPIIGSPPVR	S8,S/T	0.992	0.916
Patl1	Protein PAT1 homolog 1	Q3TC46	Ambiguous	RSTSPIIGSPPVR	T/S	0.935	0.260
Paxbp1	PAX3- and PAX7-binding protein 1	P58501	S264	LVREDENDASDDEDDDEK	S10	0.953	0.509
Paxbp1	PAX3- and PAX7-binding protein 1	P58501	S264	LVREDENDASDDEDDDEKR	S10	1.038	0.509
Paxbp1	PAX3- and PAX7-binding protein 1	P58501	S182,S193	DTNPEDGVVISEHGEDEMDMESEKEEEKPK	S11,S22	1.066	0.607
Paxbp1	PAX3- and PAX7-binding protein 1	P58501	S182,S193	DTNPEDGVVISEHGEDEMDMESEKEEEKPK	S11,S22	1.005	0.980
Paxip1	PAX-interacting protein 1	Q6NZQ4	S223,S230	SSVASSAVASPAEQPCSPKPR	S10,S17	1.064	0.505
Paxx	Protein PAXX	Q8K0Y7	S150	LAAAEETITSPK	S10	1.276	0.146
Pbdc1	Protein PBDC1	Q9D0B6	S184	GADSGGEKEEGANR	S4	1.047	0.475
Pbrm1	Protein polybromo-1	F8VQD1	S10	ATSPSSSVSGDFDDGHHSVPTPGPSR	S3	0.983	0.746
Pbrm1	Protein polybromo-1	F8VQD1	S1002	LWEDSAGEK	S5	1.029	0.929
Pbrm1	Protein polybromo-1	F8VQD1	S505	RDDIEDGDSMISSATSDTGSAK	S16	1.023	0.786
Pbrm1	Protein polybromo-1	F8VQD1	S505	RDDIEDGDSMISSATSDTGSAK	S16	1.032	0.766
Pbrm1	Protein polybromo-1	F8VQD1	S949	EAEKSEDSSGTTGLSGLHR	S8	0.939	0.669
Pbrm1	Protein polybromo-1	F8VQD1	S963	TYSQDCSFK	S3	1.183	0.207
Pbrm1	Protein polybromo-1	F8VQD1	S10,S12	ATSPSSSVSGDFDDGHHSVPTPGPSR	S3,S5	0.914	0.040
Pbrm1	Protein polybromo-1	F8VQD1	Ambiguous	GEADDEDDDEDGQDNQGTLADGSSPGYLK	S/T/Y	0.800	0.201
Pbrm1	Protein polybromo-1	F8VQD1	Ambiguous	AAQQQQPSASPR	S	0.930	0.224
Pbrm1	Protein polybromo-1	F8VQD1	Ambiguous	RATSPSSSVSGDFDDGHHSVPTPGPSR	T/S	1.456	0.013
Pbrm1	Protein polybromo-1	F8VQD1	Ambiguous	RATSPSSSVSGDFDDGHHSVPTPGPSR	T/S	1.006	0.920
Pbx2	Pre-B-cell leukemia transcription factor 2	O35984	S151	GGGSAAAAAAAAASGGGVSPDNSIEHSDYR	S19	0.973	0.732
Pbx2	Pre-B-cell leukemia transcription factor 2	O35984	S330	TAVSVAQGGHSR	S11	1.106	0.469
Pbx2	Pre-B-cell leukemia transcription factor 2	O35984	S395	HSMGPGSYGDNIGGGQIYSPR	S19	0.818	0.111
Pbxip1	Pre-B-cell leukemia transcription factor-interacting protein 1	Q3TVI8	Ambiguous	GREPSSSQPVVPVDVEDQAK	S	1.165	0.067
Pcbp1	Poly(rC)-binding protein 1	P60335	S190	VMTIPYQPMPASSPVICAGGQDR	S13	1.158	0.256
Pcbp1	Poly(rC)-binding protein 1	P60335	S190	VMTIPYQPMPASSPVICAGGQDR	S13	0.984	0.886
Pcbp1	Poly(rC)-binding protein 1	P60335	Ambiguous	IANPVEGSSGR	S	1.002	0.990
Pcbp2	Poly(rC)-binding protein 2	Q61990	S362	LSSETGGMGSS	S11	1.047	0.394
Pcbp2	Poly(rC)-binding protein 2	Q61990	Ambiguous	LSSETGGMGSS	S/T	1.021	0.555
Pcca	Propionyl-CoA carboxylase alpha chain, mitochondrial	Q91ZA3	S248	FSSQEAASSFGDDR	S9	1.056	0.375
Pcdh7	Protocadherin 7	A0A0A6YY83	S1243	TGEYKPSPVNTLTR	S7	0.860	0.021
Pcdh7	Protocadherin 7	A0A0A6YY83	S192	YELLQEPGGGGGSGEGR	S13	0.783	0.256
Pcdh7	Protocadherin 7	A0A0A6YY83	S213	LGPADSAPYPGGGGNSASGGGSGGSK	S16	1.051	0.495
Pcdh7	Protocadherin 7	A0A0A6YY83	S213	RLGPADSAPYPGGGGNSASGGGSGGSK	S17	0.922	0.477
Pcdh7	Protocadherin 7	A0A0A6YY83	S974	LSDSPSMGR	S4	0.967	0.512
Pcdh7	Protocadherin 7	A0A0A6YY83	S974	LSDSPSMGR	S4	0.982	0.724
Pcdh7	Protocadherin 7	A0A0A6YY83	S989	SVNGGPGSPDLAR	S8	0.958	0.234
Pcf11	Cleavage and polyadenylation factor subunit homolog (S. cerevisiae)	G3X9Z4	S1518	NELQEPCESPK	S9	1.019	0.904
Pcf11	Cleavage and polyadenylation factor subunit homolog (S. cerevisiae)	G3X9Z4	S794	FDGSPGQMGGGGPMR	S4	1.066	0.709
Pcf11	Cleavage and polyadenylation factor subunit homolog (S. cerevisiae)	G3X9Z4	T785	IDGPPTPGSLR	T6	0.972	0.683
Pcf11	Cleavage and polyadenylation factor subunit homolog (S. cerevisiae)	G3X9Z4	S490,S495	SRSPIIHSPK	S3,S8	1.006	0.895
Pcf11	Cleavage and polyadenylation factor subunit homolog (S. cerevisiae)	G3X9Z4	S510,S512	SMSPNLAPK	S1,S3	1.093	0.177
Pcf11	Cleavage and polyadenylation factor subunit homolog (S. cerevisiae)	G3X9Z4	S510,S512	QRSMSPNLAPK	S3,S5	1.029	0.303
Pcgf6	Polycomb group RING finger protein 6	Q99NA9	S118	LESGRADSEDEEER	S8	1.013	0.806
Pcif1	Phosphorylated CTD-interacting factor 1	P59114	S116	QLSEEQPSGNGVK	S3	1.017	0.796
Pcif1	Phosphorylated CTD-interacting factor 1	P59114	S19,S30	EGASLLSHSPGTSSQSQPCSPKPVR	S9,S20	1.079	0.139
Pcm1	Pericentriolar material 1 protein	Q9R0L6	S1228	TGFTESVEK	S6	0.948	0.537
Pcm1	Pericentriolar material 1 protein	Q9R0L6	S1729	ILEGDHGSPAGEIDDEDKDKDETETVK	S8	0.932	0.334
Pcm1	Pericentriolar material 1 protein	Q9R0L6	S1769	SDVSDQEEDEESERCPVSINLSK	S4	0.955	0.849
Pcm1	Pericentriolar material 1 protein	Q9R0L6	S1978	LTVYSEEELR	S5	0.983	0.729
Pcm1	Pericentriolar material 1 protein	Q9R0L6	S372	QAESLSLTR	S6	0.966	0.571
Pcm1	Pericentriolar material 1 protein	Q9R0L6	S384	NPSVSEHLPDEK	S3	1.104	0.179
Pcm1	Pericentriolar material 1 protein	Q9R0L6	S65	VTNAISPESSPGVGR	S6	1.021	0.339
Pcm1	Pericentriolar material 1 protein	Q9R0L6	S65	VTNAISPESSPGVGRR	S6	0.884	0.446
Pcm1	Pericentriolar material 1 protein	Q9R0L6	S957	SNRPVSADGNYRPLAK	S6	1.000	0.997
Pcm1	Pericentriolar material 1 protein	Q9R0L6	S1766,S1769	NVRSDVSDQEEDEESER	S4,S7	1.031	0.581
Pcm1	Pericentriolar material 1 protein	Q9R0L6	S380,S384	EISQSRNPSVSEHLPDEK	S5,S9	1.115	0.473
Pcm1	Pericentriolar material 1 protein	Q9R0L6	S65,S/T	VTNAISPESSPGVGR	S6,S/T	0.989	0.827
Pcm1	Pericentriolar material 1 protein	Q9R0L6	S65,S69	VTNAISPESSPGVGRR	S6,S10	1.025	0.524
Pcm1	Pericentriolar material 1 protein	Q9R0L6	Ambiguous	SAQETPESSLAGSPDTESPVLVNDYEAESGNISQK	S/T/Y	1.073	0.488
Pcm1	Pericentriolar material 1 protein	Q9R0L6	Ambiguous	RSSLVDEAPEDEEFEQK	S	0.851	0.307
Pcm1	Pericentriolar material 1 protein	Q9R0L6	Ambiguous	YMTQMSVPEQAELEK	T/S/Y	1.529	0.000
Pcm1	Pericentriolar material 1 protein	Q9R0L6	Ambiguous	YLPAITSTPTVNENDSSTSK	T/S/Y	1.099	0.222
Pcmtd2	Protein-L-isoaspartate O-methyltransferase domain-containing protein 2	Q8BHD8	S308	ISNPSDDTSCEDAEEDRR	S9	1.078	0.290
Pcnp	PEST proteolytic signal-containing nuclear protein	F8WI88	S130	TLSVAAAFNEDEDSEPEEMPPEAK	S14	0.986	0.899
Pcnp	PEST proteolytic signal-containing nuclear protein	F8WI88	S158	DTPTSAGPNSFNK	S10	1.024	0.677
Pcnp	PEST proteolytic signal-containing nuclear protein	F8WI88	T150	NIGRDTPTSAGPNSFNK	T6	0.929	0.356
Pcnt	Pericentrin	F8VPV0	S1437	RNSEIDELK	S3	0.883	0.077
Pcnt	Pericentrin	F8VPV0	S432	LQEAREDSLESTEISSSCVLPEETSGR	S8	1.051	0.719
Pcnx3	Pecanex-like protein 3	Q8VI59	S1955	GLGGPDGEPASGSPK	S13	1.070	0.402
Pcnx3	Pecanex-like protein 3	Q8VI59	S287	GSGDPMPQK	S2	0.985	0.538
Pcnx3	Pecanex-like protein 3	Q8VI59	S287	GSGDPMPQK	S2	0.985	0.771
Pcnx3	Pecanex-like protein 3	Q8VI59	S505	VLSMDGAGGDVLR	S3	0.977	0.847
Pcnx3	Pecanex-like protein 3	Q8VI59	S96	RNSTMGEQEEEAAQGESSLPR	S3	0.887	0.679
Pcnx3	Pecanex-like protein 3	Q8VI59	T1028	SLETARVEPPDPLPEK	T4	1.084	0.618
Pcnx3	Pecanex-like protein 3	Q8VI59	Ambiguous	RNSTMGEQEEEAAQGESSLPR	T/S	1.030	0.842
Pcp4l1	Purkinje cell protein 4-like protein 1	Q6W8Q3	S16	TPPAANQASDPEEK	S9	1.015	0.779
Pcyt1a	Choline-phosphate cytidylyltransferase A	P49586	S315	MLQAISPK	S6	1.033	0.764
Pcyt1a	Choline-phosphate cytidylyltransferase A	P49586	S343	TSPSSSPASLSR	S2	1.066	0.038
Pcyt1a	Choline-phosphate cytidylyltransferase A	P49586	S362	AVTCDISEDEED	S7	0.995	0.856
Pcyt1a	Choline-phosphate cytidylyltransferase A	P49586	S315,S319	MLQAISPKQSPSSSPTHER	S6,S10	0.997	0.968
Pcyt1a	Choline-phosphate cytidylyltransferase A	P49586	S343,S346	TSPSSSPASLSR	S2,S5	1.057	0.077
Pcyt1a	Choline-phosphate cytidylyltransferase A	P49586	T358,S362	AVTCDISEDEED	T3,S7	0.993	0.929
Pcyt1a	Choline-phosphate cytidylyltransferase A	P49586	S315,S323,S/T	MLQAISPKQSPSSSPTHER	S6,S14,S/T	0.987	0.503
Pcyt1a	Choline-phosphate cytidylyltransferase A	P49586	Ambiguous	TEGISTSDIITR	S/T	1.014	0.820
Pdap1	28 kDa heat- and acid-stable phosphoprotein	Q3UHX2	S19	QYTSPEEIDAQLQAEK	S4	0.913	0.384

Pdap1	28 kDa heat- and acid-stable phosphoprotein	Q3UHX2	S48	ANEEDEQEEGGDGASGDPKK	S15	0.861	0.449
Pdap1	28 kDa heat- and acid-stable phosphoprotein	Q3UHX2	S63	SLDSDESEDEDDDYQQK	S7	0.982	0.419
Pdap1	28 kDa heat- and acid-stable phosphoprotein	Q3UHX2	S63	SLDSDESEDEDDDYQQKR	S7	1.003	0.934
Pdap1	28 kDa heat- and acid-stable phosphoprotein	Q3UHX2	S60,S63	SLDSDESEDEDDDYQQK	S4,S7	0.962	0.465
Pdap1	28 kDa heat- and acid-stable phosphoprotein	Q3UHX2	S60,S63	SLDSDESEDEDDDYQQKR	S4,S7	0.988	0.885
Pdap1	28 kDa heat- and acid-stable phosphoprotein	Q3UHX2	S60,S63	KSLDSDESEDEDDDYQQKR	S5,S8	0.884	0.057
Pdap1	28 kDa heat- and acid-stable phosphoprotein	Q3UHX2	S60,S63	KSLDSDESEDEDDDYQQK	S5,S8	0.953	0.293
Pdcd11	Protein RRP5 homolog	Q6NS46	S1468	QESESEQELVNK	S3	1.227	0.031
Pdcd11	Protein RRP5 homolog	Q6NS46	S1468	ERQESESEQELVNK	S5	1.088	0.306
Pdcd11	Protein RRP5 homolog	Q6NS46	S1490	KSGAAEEDDSGVEVYYR	S10	1.126	0.613
Pdcd4	Programmed cell death protein 4	Q61823	S457	FVSEGDGGR	S3	1.352	0.002
Pdcd4	Programmed cell death protein 4	Q61823	S94	SGVAVPTSPK	S8	0.982	0.806
Pdcd5	Programmed cell death protein 5	D3Z7Q5	S198	VMDSDEDDADY	S4	1.017	0.407
Pdcd5	Programmed cell death protein 5	D3Z7Q5	S198	VMDSDEDDADY	S4	1.015	0.508
Pdcd5	Programmed cell death protein 5	D3Z7Q5	S198	KVMDSDEDDADY	S5	0.984	0.780
Pdcd5	Programmed cell death protein 5	D3Z7Q5	S198	KVMDSDEDDADY	S5	1.000	0.998
Pdcl	Phosducin-like protein	Q9DBX2	S296	NSATCHSEDSDLEID	S10	1.006	0.909
Pdcl	Phosducin-like protein	Q9DBX2	S293,S296	NSATCHSEDSDLEID	S7,S10	0.940	0.340
Pde12	2',5'-phosphodiesterase 12	Q3TIU4	S101	AHSSGGAACEATGPEPVATCEPVVK	S3	1.147	0.281
Pde3b	cGMP-inhibited 3',5'-cyclic phosphodiesterase B	Q61409	Ambiguous	SSCVSLGESAAGYYGSGK	S/Y	1.073	0.772
Pde4a	cAMP-specific 3',5'-cyclic phosphodiesterase 4A	O89084	S147	SDSDYDMSPK	S3	1.042	0.669
Pde4a	cAMP-specific 3',5'-cyclic phosphodiesterase 4A	O89084	S147	SDSDYDMSPK	S3	0.906	0.274
Pde4d	Phosphodiesterase	E9PVU7	S192	SDSDYDLSPK	S3	0.997	0.941
Pde4dip	Myomegalin	G3X9L9	S862	STLGDSDTVAGLEK	S1	0.799	0.188
Pde4dip	Myomegalin	G3X9L9	Ambiguous	EEASIPRSTLGDSDTVAGLEK	S/T	0.992	0.900
Pde7a	High affinity cAMP-specific 3',5'-cyclic phosphodiesterase 7A	P70453	S58	RGSHPYIDFR	S3	0.970	0.802
Pde7b	Phosphodiesterase	E9Q0W7	S478	GSGQDLAGPAPETLEQTEGATP	S2	1.039	0.726
Pde7b	Phosphodiesterase	E9Q0W7	S478	RGSGQDLAGPAPETLEQTEGATP	S3	0.852	0.113
Pde7b	Phosphodiesterase	E9Q0W7	S478	RRGSGQDLAGPAPETLEQTEGATP	S4	0.922	0.098
Pde7b	Phosphodiesterase	E9Q0W7	S97	RGSYPFIDFR	S3	1.062	0.619
Pde8a	ligh affinity cAMP-specific and IBMX-insensitive 3',5'-cyclic phosphodiesterase 8.	O88502	S18	TFSHSDGEDEDVDVDVPGPAPR	S5	1.039	0.292
Pdha1	yruvate dehydrogenase E1 component subunit alpha, somatic form, mitochondri	P35486	S232	YGMGTSVER	S6	1.007	0.873
Pdha1	yruvate dehydrogenase E1 component subunit alpha, somatic form, mitochondri	P35486	S293	YHGHSMSDPGVSYR	S5	0.984	0.752
Pdha1	yruvate dehydrogenase E1 component subunit alpha, somatic form, mitochondri	P35486	S293	YHGHSMSDPGVSYR	S5	1.010	0.728
Pdha1	yruvate dehydrogenase E1 component subunit alpha, somatic form, mitochondri	P35486	Y242	AAASTDYYK	Y7	1.034	0.683
Pdha1	yruvate dehydrogenase E1 component subunit alpha, somatic form, mitochondri	P35486	S293,S295	YHGHSMSDPGVSYR	S5,S7	1.019	0.402
Pdha1	yruvate dehydrogenase E1 component subunit alpha, somatic form, mitochondri	P35486	S293,S300	YHGHSMSDPGVSYR	S5,S12	1.017	0.362
Pdlim2	PDZ and LIM domain protein 2	Q8R1G6	S87	SQTASPGQTNGEGSLEVLATR	S5	1.068	0.290
Pdlim2	PDZ and LIM domain protein 2	Q8R1G6	S199,S	VLLHSPGRPSSPR	S5,S	1.005	0.911
Pdlim4	PDZ and LIM domain protein 4	P70271	S111	IHIDPESQDCSPATSRR	S11	0.893	0.241
Pdlim4	PDZ and LIM domain protein 4	P70271	S111	IHIDPESQDCSPATSR	S11	0.986	0.675
Pdlim4	PDZ and LIM domain protein 4	P70271	S119	SSVSGISLEDNR	S2	1.088	0.064
Pdlim4	PDZ and LIM domain protein 4	P70271	S119	RSSVSGISLEDNR	S3	1.042	0.257
Pdlim4	PDZ and LIM domain protein 4	P70271	S107,S111	IHIDPESQDCSPATSR	S7,S11	0.959	0.198
Pdlim5	PDZ and LIM domain protein 5	Q8CI51	S228	RGSQGDIK	S3	1.068	0.122
Pdlim5	PDZ and LIM domain protein 5	Q8CI51	S359	SPSWQRPNQAAPSTGR	S1	1.076	0.176
Pdlim5	PDZ and LIM domain protein 5	Q8CI51	S382	SSGTGASVGPPQPSDQDTLVQR	S2	1.073	0.220
Pdlim7	PDZ and LIM domain protein 7	Q3TJD7	T96	ALTPPADPPR	T3	1.002	0.979
Pdpk1	3-phosphoinositide-dependent protein kinase 1	Q9Z2A0	S244	ANSFVGTAQYVSPELLTEK	S3	1.075	0.507
Pdpk1	3-phosphoinositide-dependent protein kinase 1	Q9Z2A0	S38	SQTEPGSSPGIPSGVSR	S8	1.042	0.374
Pds5a	Sister chromatid cohesion protein PDS5 homolog A	E9QPI5	S1174	SAGTETGSNINANSELSPSAGSR	S17	1.003	0.887
Pds5a	Sister chromatid cohesion protein PDS5 homolog A	E9QPI5	S1189	SREQSSEASETGVSENEENPVR	S9	0.998	0.956
Pds5a	Sister chromatid cohesion protein PDS5 homolog A	E9QPI5	S1194	EQSSEASETGVSENEENPVR	S12	1.104	0.382
Pds5a	Sister chromatid cohesion protein PDS5 homolog A	E9QPI5	S1300	AAGSQESLEAGNAK	S4	1.069	0.400
Pds5b	Sister chromatid cohesion protein PDS5 homolog B	Q4VA53	S1166	METVSNASSSSNPSSPGR	S15	1.143	0.153
Pds5b	Sister chromatid cohesion protein PDS5 homolog B	Q4VA53	S1166	METVSNASSSSNPSSPGRIK	S15	1.080	0.615
Pds5b	Sister chromatid cohesion protein PDS5 homolog B	Q4VA53	S1166	METVSNASSSSNPSSPGR	S15	0.969	0.698
Pds5b	Sister chromatid cohesion protein PDS5 homolog B	Q4VA53	S1182	GRLDSSEMDHSENEDYTMSSPLPGK	S11	1.043	0.772
Pds5b	Sister chromatid cohesion protein PDS5 homolog B	Q4VA53	S1255	TASDSDEQQWPEEKR	S3	1.021	0.485
Pds5b	Sister chromatid cohesion protein PDS5 homolog B	Q4VA53	S1255	TASDSDEQQWPEEK	S3	0.977	0.522
Pds5b	Sister chromatid cohesion protein PDS5 homolog B	Q4VA53	S1255	GRTASDSDEQQWPEEK	S5	1.084	0.154
Pds5b	Sister chromatid cohesion protein PDS5 homolog B	Q4VA53	S1281	EELLENEDEQNSPPKK	S12	1.122	0.231
Pds5b	Sister chromatid cohesion protein PDS5 homolog B	Q4VA53	S1281	HKEELLENEDEQNSPPK	S14	1.140	0.043
Pds5b	Sister chromatid cohesion protein PDS5 homolog B	Q4VA53	S1356	AESPETSAVESTQSTPQK	S3	1.023	0.648
Pds5b	Sister chromatid cohesion protein PDS5 homolog B	Q4VA53	S1356	AQQRAESPETSAVESTQSTPQK	S7	0.921	0.550
Pds5b	Sister chromatid cohesion protein PDS5 homolog B	Q4VA53	S1381	APSPSQPPK	S3	1.095	0.043
Pds5b	Sister chromatid cohesion protein PDS5 homolog B	Q4VA53	S1381	APSPSQPPKK	S3	1.091	0.253
Pds5b	Sister chromatid cohesion protein PDS5 homolog B	Q4VA53	T1253	GRTASDSDEQQWPEEKR	T3	1.010	0.837
Pds5b	Sister chromatid cohesion protein PDS5 homolog B	Q4VA53	S1166,S/T	METVSNASSSSNPSSPGR	S15,S/T	0.949	0.294
Pds5b	Sister chromatid cohesion protein PDS5 homolog B	Q4VA53	S1182,S/Y/T	GRLDSSEMDHSENEDYTMSSPLPGK	S11,S/Y/T	1.035	0.595
Pds5b	Sister chromatid cohesion protein PDS5 homolog B	Q4VA53	S1356,T1368	AESPETSAVESTQSTPQKGR	S3,T15	1.094	0.295
Pds5b	Sister chromatid cohesion protein PDS5 homolog B	Q4VA53	S1356,T1368	AESPETSAVESTQSTPQK	S3,T15	1.188	0.019
Pds5b	Sister chromatid cohesion protein PDS5 homolog B	Q4VA53	Ambiguous	LDSSEMDHSENEDYTMSSPLPGK	T/S/Y	1.229	0.179
Pdxdc1	MCG129810, isoform CRA_c	A0A0R4J034	S718	GSDAVSETSSVSHIEDLEK	S6	0.960	0.718
Pdxdc1	MCG129810, isoform CRA_c	A0A0R4J034	T687	VQGTGVTPPPTPLGTR	T7	0.975	0.229
Pdxdc1	MCG129810, isoform CRA_c	A0A0R4J034	T687,T691	VQGTGVTPPPTPLGTR	T7,T11	1.061	0.158
Pdxdc1	MCG129810, isoform CRA_c	A0A0R4J034	Ambiguous	VEQLSSGLEHDNLEAHSPEQPPR	S	1.021	0.771
Pdzd8	PDZ domain-containing protein 8	B9EJ80	S515	VQTELKEETQPLSHSPK	S15	1.110	0.118
Pdzd8	PDZ domain-containing protein 8	B9EJ80	S960	LSEPGTDLVEPSPK	S12	1.012	0.913
Pdzd8	PDZ domain-containing protein 8	B9EJ80	S973,T/S	HTPNTSDNEGSDTEVCGSNSPSKR	S11,T/S	1.135	0.321
Pdzd8	PDZ domain-containing protein 8	B9EJ80	Ambiguous	HTPNTSDNEGSDTEVCGSNSPSKR	T/S	0.691	0.032
Pea15	Astrocytic phosphoprotein PEA-15	Q62048	S116	QPSEEEIIK	S3	1.143	0.009
Pea15	Astrocytic phosphoprotein PEA-15	Q62048	S116	DIIRQPSEEEIIK	S7	1.178	0.025
Pea15	Astrocytic phosphoprotein PEA-15	Q62048	S116	YKDIIRQPSEEEIIK	S9	1.174	0.291
Peak1	Inactive tyrosine-protein kinase PEAK1	Q69Z38	S1050	VTHEVAGELSPR	S10	1.024	0.553
Peak1	Inactive tyrosine-protein kinase PEAK1	Q69Z38	S1050	VTHEVAGELSPRDPR	S10	1.039	0.564
Peak1	Inactive tyrosine-protein kinase PEAK1	Q69Z38	S1239	DRFSNSMESLSSR	S6	0.949	0.248
Peak1	Inactive tyrosine-protein kinase PEAK1	Q69Z38	S1239	DRFSNSMESLSSR	S6	1.005	0.921
Peak1	Inactive tyrosine-protein kinase PEAK1	Q69Z38	S282	ANTLSPVR	S5	0.967	0.375
Peak1	Inactive tyrosine-protein kinase PEAK1	Q69Z38	S362	SETASSLSQK	S8	1.047	0.533
Peak1	Inactive tyrosine-protein kinase PEAK1	Q69Z38	S371	VCNGGISPGNPGNSK	S7	1.060	0.189
Peak1	Inactive tyrosine-protein kinase PEAK1	Q69Z38	S390	DIAETESNFESPPGNNEEKDESLTSK	S11	0.904	0.323
Peak1	Inactive tyrosine-protein kinase PEAK1	Q69Z38	S565	SAPTSPTATNISSK	S1	1.017	0.705
Peak1	Inactive tyrosine-protein kinase PEAK1	Q69Z38	S691	GFSNSTER	S5	0.983	0.607
Peak1	Inactive tyrosine-protein kinase PEAK1	Q69Z38	S727	SYSSTHSSPAK	S8	1.187	0.485
Peak1	Inactive tyrosine-protein kinase PEAK1	Q69Z38	S791	ACSVEELYAVPPDADTTK	S3	0.999	0.985
Peak1	Inactive tyrosine-protein kinase PEAK1	Q69Z38	S836	SLFTSQSSGEGEAHQTTESPTAK	S19	1.067	0.760
Peak1	Inactive tyrosine-protein kinase PEAK1	Q69Z38	T1141	TDQEGLNASQPTPPPLPK	T12	1.070	0.394
Peak1	Inactive tyrosine-protein kinase PEAK1	Q69Z38	T1154	ANTEPISK	T3	0.976	0.647
Peak1	Inactive tyrosine-protein kinase PEAK1	Q69Z38	T536	YQEVWTSSTSPR	T9	1.030	0.702
Peak1	Inactive tyrosine-protein kinase PEAK1	Q69Z38	S565,T/S	SAPTSPTATNISSK	S1,T/S	1.037	0.103
Peak1	Inactive tyrosine-protein kinase PEAK1	Q69Z38	S565,T568	NCHKSAPTSPTATNISSK	S5,T8	1.066	0.309
Pebp1	Phosphatidylethanolamine-binding protein 1	P70296	Y181	LYEQLSGK	Y2	1.082	0.317
Pelp1	Proline-, glutamic acid- and leucine-rich protein 1	Q9DBD5	T751	AGSGEDPVLAPSGTPPPSIPPDETFGGR	T14	1.052	0.112
Pepd	Xaa-Pro dipeptidase	Q11136	S162	EASFEGISK	S3	0.927	0.529
Per1	Period circadian protein homolog 1	O35973	Ambiguous	TEGGAAAGGPGSSAGPLPPSEETAEPEAR	S/T	0.869	0.036
Pex1	Peroxisome biogenesis factor 1	Q5BL07	S1182	TPSQEGCQDLTQEQR	S3	0.952	0.434
Pex1	Peroxisome biogenesis factor 1	Q5BL07	S1212	SQSGEDESLNQPGPIK	S3	0.978	0.529
Pex1	Peroxisome biogenesis factor 1	Q5BL07	S1212,S1217	SQSGEDESLNQPGPIK	S3,S8	0.985	0.897
Pex1	Peroxisome biogenesis factor 1	Q5BL07	Ambiguous	YRSQSGEDESLNQPGPIK	S/Y	1.024	0.913
Pex14	Peroxisomal membrane protein PEX14	Q9R0A0	S232	QFPPSPSAPK	S5	0.976	0.638
Pex14	Peroxisomal membrane protein PEX14	Q9R0A0	Ambiguous	DSHSPEGSTATYHLLGPQEEGEGVLDVK	S/T/Y	0.913	0.327
Pex19	Peroxisomal biogenesis factor 19	Q8VCI5	S66	DALFASQEK	S6	1.014	0.935
Pex19	Peroxisomal biogenesis factor 19	Q8VCI5	T236	ICEQFEAETPTDSEATQR	T9	0.981	0.859
Pex19	Peroxisomal biogenesis factor 19	Q8VCI5	Ambiguous	VGSDASSQQEFTSCLK	S/T	0.848	0.191
Pfdn2	Prefoldin subunit 2	O70591	S148	ASSAGVLVS	S3	0.992	0.908
Pfdn4	Prefoldin subunit 4	Q3UWL8	S125	FGSNINLEADES	S3	0.943	0.670
Pfkfb2	6-phosphofructo-2-kinase/fructose-2,6-bisphosphatase 2	A0A087WRM7	S468	RNSFTPLSSSNTIR	S3	1.041	0.421
Pfkfb3	6-phosphofructo-2-kinase/fructose-2, 6-biphosphatase 3 splice variant 2	A7UAK5	S490	RNSVTPLASPEPTK	S3	1.042	0.458
Pfkfb3	6-phosphofructo-2-kinase/fructose-2, 6-biphosphatase 3 splice variant 2	A7UAK5	S490,S496	RNSVTPLASPEPTK	S3,S9	1.069	0.464
Pfkl	ATP-dependent 6-phosphofructokinase, liver type	P12382	S775	TLSIDKGF	S3	1.127	0.563
Pfkp	ATP-dependent 6-phosphofructokinase	Q8C605	S772	YEASYDMSDSGKLESLQHHEEL	S10	1.073	0.373
Pgam1	Phosphoglycerate mutase 1	Q9DBJ1	S118	SYDVPPPPMEPDHPFYSNISK	S1	1.053	0.631
Pgam1	Phosphoglycerate mutase 1	Q9DBJ1	S118	SYDVPPPPMEPDHPFYSNISK	S1	1.012	0.915
Pgam1	Phosphoglycerate mutase 1	Q9DBJ1	S14	HGESAWNLENR	S4	0.989	0.815
Pgam1	Phosphoglycerate mutase 1	Q9DBJ1	S31	FSGWYDADLSPAGHEEAK	S10	1.053	0.197
Pgam1	Phosphoglycerate mutase 1	Q9DBJ1	Y92	HYGGLTGLNK	Y2	1.038	0.459
Pgd	6-phosphogluconate dehydrogenase, decarboxylating	Q9DCD0	S257	IRDSAGQK	S4	0.971	0.685
Pgm2l1	Glucose 1,6-bisphosphate synthase	Q8CAA7	S175	AVAGVMITASHNR	S10	0.982	0.393
Pgm2l1	Glucose 1,6-bisphosphate synthase	Q8CAA7	S175	AVAGVMITASHNR	S10	0.982	0.815
Pgm3	Phosphoacetylglucosamine mutase	Q9CYR6	S64	STIGVMVTASHNPEEDNGVK	S10	0.960	0.092
Pgm3	Phosphoacetylglucosamine mutase	Q9CYR6	Ambiguous	STIGVMVTASHNPEEDNGVK	T/S	0.947	0.701
Pgrmc1	Membrane-associated progesterone receptor component 1	O55022	S181	LLKEGEEPTVYSDDEEPKDETAR	S12	0.786	0.442
Pgrmc1	Membrane-associated progesterone receptor component 1	O55022	S181	EGEEPTVYSDDEEPK	S9	1.245	0.141
Pgrmc1	Membrane-associated progesterone receptor component 1	O55022	S181	EGEEPTVYSDDEEPKDETAR	S9	1.048	0.732
Pgrmc1	Membrane-associated progesterone receptor component 1	O55022	S181	EGEEPTVYSDDEEPKDETARK	S9	1.075	0.540
Pgrmc2	Membrane-associated progesterone receptor component 2	Q80UU9	S19	LSTLGSGGESGGDGSPGGAGATAAR	S10	1.010	0.930
Pgrmc2	Membrane-associated progesterone receptor component 2	Q80UU9	S84	GLCSGPGAGEESPAATLPR	S12	0.925	0.096

Pgrmc2	Membrane-associated progesterone receptor component 2	Q80UU9	S98	DFSLEQLR	S3	0.871	0.037
Pgrmc2	Membrane-associated progesterone receptor component 2	Q80UU9	T205	LLKPGEEPSEYTDEEDTKDHSKQD	T12	1.028	0.717
Phactr2	Phosphatase and actin regulator	F7D4H5	S419	DELGPQLLTPGQMGDSSESFSAPEDEAPR	S21	1.000	1.000
Phactr2	Phosphatase and actin regulator	F7D4H5	S48	WLPGDPSPR	S7	0.859	0.092
Phactr2	Phosphatase and actin regulator	F7D4H5	S514	RLSQRPTTEELEQR	S3	1.072	0.042
Phactr2	Phosphatase and actin regulator	F7D4H5	S55	SQSDLSSASSR	S3	1.062	0.062
Phactr2	Phosphatase and actin regulator	F7D4H5	T95	ASIANSDGPPAGSQTPPFK	T15	1.052	0.533
Phactr2	Phosphatase and actin regulator	F7D4H5	S86,S/T	ASIANSDGPPAGSQTPPFK	S6,S/T	1.039	0.856
Phactr4	Phosphatase and actin regulator 4	Q501J7	S118	SSSPVLVEEEPER	S3	0.970	0.287
Phactr4	Phosphatase and actin regulator 4	Q501J7	S148	LGSTGSQPNSEAEPGPEHAPK	S6	0.993	0.605
Phactr4	Phosphatase and actin regulator 4	Q501J7	S174	RPLSSSCEAK	S4	1.053	0.443
Phactr4	Phosphatase and actin regulator 4	Q501J7	S582	RLSQRPTAEELEQR	S3	1.071	0.120
Phactr4	Phosphatase and actin regulator 4	Q501J7	S145,S148	RLGSTGSQPNSEAEPGPEHAPK	S4,S7	1.076	0.299
Phb	Prohibitin	P67778	S151	QVSDDLTER	S3	1.006	0.861
Phc2	Polyhomeotic-like protein 2	Q9QWH1	S743	CSDNSSYEEPLSPISASSSTSR	S12	1.123	0.283
Phc3	Polyhomeotic-like protein 3	Q8CHP6	S261	ISQRDPSPESK	S7	0.967	0.448
Phc3	Polyhomeotic-like protein 3	Q8CHP6	T607	MDRTPPPPTLSPAAVTVGR	T4	1.125	0.023
Phc3	Polyhomeotic-like protein 3	Q8CHP6	T607,S614	MDRTPPPPTLSPAAVTVGR	T4,S11	1.088	0.213
Phc3	Polyhomeotic-like protein 3	Q8CHP6	T607,S614	MDRTPPPPTLSPAAVTVGR	T4,S11	1.019	0.788
Pheta2	Sesquipedalian-2	A0A0R4J1Z2	S32	SWGGPGSPPTPSGTGR	S7	0.921	0.460
Phf10	PHD finger protein 10	K4DI61	S21	CDSDPASPGAQSPK	S7	0.930	0.347
Phf10	PHD finger protein 10	K4DI61	S326	GTSDSSSGNVSEGDSPPDSQEDTFHGR	S11	0.948	0.721
Phf10	PHD finger protein 10	K4DI61	S326	NKGTSDSSSGNVSEGDSPPDSQEDTFHGR	S13	1.171	0.446
Phf11	PHD finger protein 11	A6H5X4	S169	RLSSGPPAQPK	S3	0.878	0.119
Phf11	PHD finger protein 11	A6H5X4	S17	GDSPIIEK	S3	0.897	0.228
Phf11	PHD finger protein 11	A6H5X4	S170	LSSGPPAQPK	S3	1.039	0.718
Phf11	PHD finger protein 11	A6H5X4	S326	SQESPEVQAGSGDSL	S4	0.958	0.514
Phf11a	PHD finger protein 11A	Q8BVM9	S282	CQESPEVQAGSGDSL	S4	1.238	0.209
Phf12	PHD finger protein 12	Q5SPL2	S131	TTSPSSDTDLLDRPASK	S3	1.037	0.416
Phf12	PHD finger protein 12	Q5SPL2	T670	VLTPPQAAGDSILATGANQR	T3	0.749	0.079
Phf12	PHD finger protein 12	Q5SPL2	T130,S134	TTSPSSDTDLLDRPASK	T2,S6	0.945	0.142
Phf12	PHD finger protein 12	Q5SPL2	Ambiguous	RTTSPSSDTDLLDRPASK	T/S	1.068	0.365
Phf14	PHD finger protein 14	G5E8S0	S287	NSADDEELTNDSLTLSQSK	S16	1.021	0.735
Phf14	PHD finger protein 14	G5E8S0	S295	SNEDSLILEK	S5	0.888	0.497
Phf14	PHD finger protein 14	G5E8S0	S828	SFVPEEEKHEER	S1	1.228	0.014
Phf2	Lysine-specific demethylase PHF2	Q9WTU0	S876	DSDYVYPSLESDEDNPVFK	S11	1.084	0.101
Phf2	Lysine-specific demethylase PHF2	Q9WTU0	S893	KGSDDAPYSPTAR	S3	1.016	0.645
Phf2	Lysine-specific demethylase PHF2	Q9WTU0	S458,S474	TVRPEVNAAASSDEVCDGDREKEEPPSPVETTPPR	S11,S27	1.068	0.309
Phf2	Lysine-specific demethylase PHF2	Q9WTU0	Ambiguous	TVRPEVNAAASSDEVCDGDREKEEPPSPVETTPPR	S/T	1.170	0.029
Phf2	Lysine-specific demethylase PHF2	Q9WTU0	Ambiguous	RPSASSPNNTAAK	S/T	0.978	0.864
Phf20	PHD finger protein 20	Q8BLG0	S522	VSASSPTAK	S5	1.050	0.615
Phf20	PHD finger protein 20	Q8BLG0	S867	GSALDAAVSPLCENGDDSLSPR	S9	1.000	1.000
Phf20	PHD finger protein 20	Q8BLG0	S900	GDIDPKPSSPK	S9	1.071	0.539
Phf20l1	PHD finger protein 20-like protein 1	Q8CCJ9	S584	QHDYSDYEDSSLDFLER	S5	1.136	0.637
Phf3	PHD finger protein 3	B2RQG2	S1112	MAPPIDDLSPK	S9	1.104	0.383
Phf3	PHD finger protein 3	B2RQG2	S1112	MAPPIDDLSPK	S9	1.013	0.963
Phf3	PHD finger protein 3	B2RQG2	S1592	TSSDSPCWPGTGK	S5	0.924	0.413
Phf3	PHD finger protein 3	B2RQG2	Ambiguous	HACNNQGEVNVPSPELDCPLLSETSASVEEK	S/T	0.876	0.591
Phf6	PHD finger protein 6	Q9D4J7	S155	TAHNSEADLEESFNEHELEPSSPK	S22	0.939	0.152
Phf6	PHD finger protein 6	Q9D4J7	S154,S155	TAHNSEADLEESFNEHELEPSSPK	S21,S22	0.930	0.419
Phf8	Histone lysine demethylase PHF8	Q80TJ7	S768	SSGSSSSGLGTVSSSPASQR	S15	1.073	0.288
Phf8	Histone lysine demethylase PHF8	Q80TJ7	S820	DAEYIYPSLESDDDDPALK	S11	1.014	0.852
Phf8	Histone lysine demethylase PHF8	Q80TJ7	S843	NSDDAPWSPK	S8	0.975	0.790
Phf8	Histone lysine demethylase PHF8	Q80TJ7	S984	RPSVGSQSSQAGQGK	S3	1.465	0.002
Phf8	Histone lysine demethylase PHF8	Q80TJ7	S790,S	NESEEEENASLDEQDSLGACFK	S3,S	0.716	0.127
Phip	PH-interacting protein	F8VQ93	S1783	RTAFYNEDDSEEEQR	S10	1.013	0.821
Phip	PH-interacting protein	F8VQ93	S1783	TAFYNEDDSEEEQR	S9	1.020	0.727
Phip	PH-interacting protein	F8VQ93	S911	ANEEKDGPTSPK	S10	0.841	0.383
Phip	PH-interacting protein	F8VQ93	Ambiguous	SEVSTSPFSIPTR	T/S	1.418	0.010
Phka2	Phosphorylase b kinase regulatory subunit alpha, liver isoform	Q8BWJ3	S1040,S/T	SSTPSSPTGTSSTDSGGQHLGWGEQQGQWLR	S2,S/T	0.860	0.262
Phka2	Phosphorylase b kinase regulatory subunit alpha, liver isoform	Q8BWJ3	Y49,S50,Y61	DNIYSILAVWGLGMAYR	Y4,S5,Y16	0.984	0.878
Phkb	Phosphorylase b kinase regulatory subunit beta	Q7TSH2	S19	SGSVYEPLK	S3	1.123	0.172
Phkb	Phosphorylase b kinase regulatory subunit beta	Q7TSH2	Ambiguous	QSSTADAPEAQHEPGITITEWK	S/T	1.229	0.385
Phldb1	Pleckstrin homology-like domain family B member 1	D3Z0X5	S382	SQPASIPGSPK	S9	0.958	0.479
Phldb1	Pleckstrin homology-like domain family B member 1	D3Z0X5	S387	FQSPVPAPR	S3	0.910	0.013
Phldb1	Pleckstrin homology-like domain family B member 1	D3Z0X5	S405	IGTLQDRPPSPFREPPGTER	S10	0.967	0.419
Phldb1	Pleckstrin homology-like domain family B member 1	D3Z0X5	S431	TFSDGLAATR	S3	0.999	0.967
Phldb1	Pleckstrin homology-like domain family B member 1	D3Z0X5	S445	TLQPPESPR	S7	1.010	0.552
Phldb1	Pleckstrin homology-like domain family B member 1	D3Z0X5	S463	ELPPLSPSLSR	S6	1.019	0.720
Phldb1	Pleckstrin homology-like domain family B member 1	D3Z0X5	S472	ALSPLPAR	S3	0.783	0.124
Phldb1	Pleckstrin homology-like domain family B member 1	D3Z0X5	S472	RALSPLPAR	S4	1.019	0.786
Phldb1	Pleckstrin homology-like domain family B member 1	D3Z0X5	S522	SPSPTLGESLAPR	S3	1.165	0.327
Phldb1	Pleckstrin homology-like domain family B member 1	D3Z0X5	T518	TRSPSPTLGESLAPR	T1	1.024	0.583
Phldb1	Pleckstrin homology-like domain family B member 1	D3Z0X5	S382,S	SQPASIPGSPK	S9,S	0.971	0.732
Phldb1	Pleckstrin homology-like domain family B member 1	D3Z0X5	S520,S522	TRSPSPTLGESLAPR	S3,S5	1.082	0.232
Phldb1	Pleckstrin homology-like domain family B member 1	D3Z0X5	Ambiguous	VLTSSPSR	S/T	0.953	0.418
Phldb2	Pleckstrin homology-like domain family B member 2	Q8K1N2	S333	TCLSEGNPYVSSALSVPASPR	S19	1.166	0.160
Phldb2	Pleckstrin homology-like domain family B member 2	Q8K1N2	S976	TASESNVYLNSFHYPDR	S3	0.942	0.462
Phldb2	Pleckstrin homology-like domain family B member 2	Q8K1N2	S199,S203	SGAASMPSSPK	S5,S9	0.942	0.547
Phlpp2	MCG16947	J3QM82	S1242	QNSVNSGILLPANR	S3	0.935	0.427
Phlpp2	MCG16947	J3QM82	Ambiguous	GREPESSPPLPK	S	1.030	0.056
Phrf1	PHD and RING finger domain-containing protein 1	A6H619	S1201	EVSPAPATQGESR	S3	1.021	0.132
Phrf1	PHD and RING finger domain-containing protein 1	A6H619	S622	TLSDENRPSR	S3	0.917	0.394
Phrf1	PHD and RING finger domain-containing protein 1	A6H619	S867	TISINSPK	S6	0.897	0.129
Phrf1	PHD and RING finger domain-containing protein 1	A6H619	S919	GSAEGTSDLEQEGLGEIEPTEIR	S7	1.064	0.726
Phrf1	PHD and RING finger domain-containing protein 1	A6H619	S982	TVTCVTVEEPGVLPSPDAPQITTHR	S15	0.985	0.933
Pi4k2a	Phosphatidylinositol 4-kinase type 2-alpha	Q2TBE6	S462	SASESYTQSFQSR	S3	1.091	0.133
Pi4k2a	Phosphatidylinositol 4-kinase type 2-alpha	Q2TBE6	S47	VAAAAGSGPSPPCSPGHDRER	S10	1.032	0.422
Pi4k2a	Phosphatidylinositol 4-kinase type 2-alpha	Q2TBE6	S47	VAAAAGSGPSPPCSPGHDR	S10	1.014	0.767
Pi4k2a	Phosphatidylinositol 4-kinase type 2-alpha	Q2TBE6	S47,S51	VAAAAGSGPSPPCSPGHDRER	S10,S14	1.093	0.011
Pi4k2a	Phosphatidylinositol 4-kinase type 2-alpha	Q2TBE6	S47,S51	VAAAAGSGPSPPCSPGHDR	S10,S14	1.166	0.514
Pi4ka	Phosphatidylinositol 4-kinase alpha	E9Q3L2	S1427	YLTASQLVPPDNQDTR	S5	1.146	0.498
Pi4ka	Phosphatidylinositol 4-kinase alpha	E9Q3L2	S1439	SNLDITVGSR	S1	1.039	0.404
Pi4ka	Phosphatidylinositol 4-kinase alpha	E9Q3L2	S263	TSSVSSISQVSPER	S6	1.029	0.344
Pi4ka	Phosphatidylinositol 4-kinase alpha	E9Q3L2	S268,T/S	TSSVSSISQVSPER	S11,T/S	0.890	0.109
Pi4kb	Phosphatidylinositol 4-kinase beta	E9Q8A3	S440	SVENLPECGITHEQR	S1	1.026	0.334
Pi4kb	Phosphatidylinositol 4-kinase beta	E9Q8A3	Ambiguous	STRSVENLPECGITHEQR	S/T	1.051	0.506
Pid1	PTB-containing, cubilin and LRP1-interacting protein	Q3UBG2	S204	SSSSEEASQELESDDG	S3	1.037	0.383
Pid1	PTB-containing, cubilin and LRP1-interacting protein	Q3UBG2	S204,S214	SSSSEEASQELESDDG	S3,S13	1.040	0.792
Pid1	PTB-containing, cubilin and LRP1-interacting protein	Q3UBG2	Ambiguous	IHRSSSSEEASQELESDDG	S	1.110	0.569
Piezo1	Piezo-type mechanosensitive ion channel component 1	E2JF22	S1385	DPQDPSQEPGPDSPGGSSPPR	S13	1.018	0.799
Piezo1	Piezo-type mechanosensitive ion channel component 1	E2JF22	S1385	GQLQSKDPQDPSQEPGPDSPGGSSPPR	S19	1.124	0.035
Piezo1	Piezo-type mechanosensitive ion channel component 1	E2JF22	S1385	GQLQSKDPQDPSQEPGPDSPGGSSPPRR	S19	1.035	0.500
Piezo1	Piezo-type mechanosensitive ion channel component 1	E2JF22	S1470	AEQLASGGDLNPDVEPVDVPEDEMAGR	S6	0.793	0.216
Piezo1	Piezo-type mechanosensitive ion channel component 1	E2JF22	S1600	DGPSTASSGLGAEEPLSSMTDDTSSPLSTGYNTR	S25	1.581	0.002
Piezo1	Piezo-type mechanosensitive ion channel component 1	E2JF22	S1631	SGSEEIVTDAGDLQAGTSLHGSQELLANAR	S22	1.057	0.860
Piezo1	Piezo-type mechanosensitive ion channel component 1	E2JF22	S1646	TASELLLDRR	S3	0.924	0.319
Piezo1	Piezo-type mechanosensitive ion channel component 1	E2JF22	S1646	TASELLLDR	S3	1.043	0.481
Piezo1	Piezo-type mechanosensitive ion channel component 1	E2JF22	S1864	DHIQGKGSIR	S8	1.074	0.564
Piezo1	Piezo-type mechanosensitive ion channel component 1	E2JF22	S2415	EQVGTGASGEQAGTK	S8	1.021	0.788
Piezo1	Piezo-type mechanosensitive ion channel component 1	E2JF22	S399	DATQGEMPMTTEPDLDNCTVHVLTSQSPVR	S27	1.049	0.567
Piezo1	Piezo-type mechanosensitive ion channel component 1	E2JF22	S399	DATQGEMPMTTEPDLDNCTVHVLTSQSPVR	S27	1.024	0.955
Piezo1	Piezo-type mechanosensitive ion channel component 1	E2JF22	T1917	GTAVMETEHEEGEGKETTER	T17	1.028	0.505
Pif1	ATP-dependent DNA helicase PIF1	Q80SX8	S40	VAVEELSPGGQPR	S7	1.095	0.146
Pik3ap1	Phosphoinositide 3-kinase adapter protein 1	Q9EQ32	S150	KAISEDSGCDSVTDTEPEDERELPFSK	S11	1.051	0.760
Pik3ap1	Phosphoinositide 3-kinase adapter protein 1	Q9EQ32	S150	AISEDSGCDSVTDTEPEDER	S10	0.881	0.411
Pik3ap1	Phosphoinositide 3-kinase adapter protein 1	Q9EQ32	S591	DRPPSSIYDPFAGMK	S5	1.025	0.594
Pik3ap1	Phosphoinositide 3-kinase adapter protein 1	Q9EQ32	S642	SESFRFQQENLK	S3	0.977	0.823
Pik3ap1	Phosphoinositide 3-kinase adapter protein 1	Q9EQ32	S656	LRESITR	S4	0.969	0.450
Pik3ap1	Phosphoinositide 3-kinase adapter protein 1	Q9EQ32	S734	SLLSVSSGMEGDNEDNEIPEITR	S1	0.916	0.450
Pik3ap1	Phosphoinositide 3-kinase adapter protein 1	Q9EQ32	T152	AISEDSGCDSVTDTEPEDERELPFSK	T12	0.929	0.402
Pik3ap1	Phosphoinositide 3-kinase adapter protein 1	Q9EQ32	T774	SRGPGPTQVDGAPVVTGTPVGTLERPPR	T18	1.018	0.730
Pik3ap1	Phosphoinositide 3-kinase adapter protein 1	Q9EQ32	T778	GPGPTQVDGAPVVTGTPVGTLERPPR	T20	0.880	0.159
Pik3ap1	Phosphoinositide 3-kinase adapter protein 1	Q9EQ32	T154,S/T	AISEDSGCDSVTDTEPEDERELPFSK	T14,S/T	0.857	0.597
Pik3ap1	Phosphoinositide 3-kinase adapter protein 1	Q9EQ32	T778,T/S	SRGPGPTQVDGAPVVTGTPVGTLERPPR	T22,T/S	0.800	0.353
Pik3ap1	Phosphoinositide 3-kinase adapter protein 1	Q9EQ32	T778,T	GPGPTQVDGAPVVTGTPVGTLERPPR	T20,T	1.067	0.386
Pik3ap1	Phosphoinositide 3-kinase adapter protein 1	Q9EQ32	Ambiguous	LGNFYVSSESIR	S/Y	1.033	0.788
Pik3c3	Phosphatidylinositol 3-kinase catalytic subunit type 3	Q6PF93	S165	TSSTLSEDQMSR	S3	1.017	0.615
Pik3c3	Phosphatidylinositol 3-kinase catalytic subunit type 3	Q6PF93	Ambiguous	TPGRTSSTLSEDQMSR	T/S	1.097	0.531
Pik3cd	Phosphatidylinositol 4,5-bisphosphate 3-kinase catalytic subunit	Q3T9Y0	Y524	GSGELYEHEK	Y6	1.090	0.415
Pik3r4	Phosphoinositide 3-kinase regulatory subunit 4	Q8VD65	S853	QDSNVNEEWK	S3	1.055	0.438
Pik3r5	Phosphoinositide 3-kinase regulatory subunit 5	Q5SW28	S425	STSQLVLR	S3	1.054	0.246
Pik3r5	Phosphoinositide 3-kinase regulatory subunit 5	Q5SW28	S451	AGSLCSPLDCPAQLPSR	S3	0.922	0.512
Pik3r5	Phosphoinositide 3-kinase regulatory subunit 5	Q5SW28	S500	RPFLSGDEDPK	S5	1.071	0.249
Pikfyve	1-phosphatidylinositol 3-phosphate 5-kinase	D3Z5N5; Q9Z1T6	S1503;S1548	NISPGLHNGEKEDR	S3	0.976	0.588
Pikfyve	1-phosphatidylinositol 3-phosphate 5-kinase	D3Z5N5; Q9Z1T6	S310;S299	LVSVQEDAGKSPAR	S11	0.967	0.004
Pikfyve	1-phosphatidylinositol 3-phosphate 5-kinase	D3Z5N5; Q9Z1T6	S318;S307	SASITNLSLDR	S3	1.004	0.918
Pikfyve	1-phosphatidylinositol 3-phosphate 5-kinase	D3Z5N5; Q9Z1T6	S340;S329	SGSPMVPSYETSVSPQANR	S14	1.042	0.594

Pikfyve	1-phosphatidylinositol 3-phosphate 5-kinase	D3Z5N5	S486	FDDSDTEQIAEEGDDNLAK	S4	0.992	0.832
Pikfyve	1-phosphatidylinositol 3-phosphate 5-kinase	D3Z5N5	S486	DIKFDDSDTEQIAEEGDDNLAK	S7	0.909	0.318
Pikfyve	1-phosphatidylinositol 3-phosphate 5-kinase	D3Z5N5; Q9Z1T6	S76;S76	GEGGQGEQQSPSSSWASPQIPSR	S17	0.925	0.501
Pikfyve	1-phosphatidylinositol 3-phosphate 5-kinase	D3Z5N5; Q9Z1T6	S857;S902	GEEGASQEQVSGSSLPQDPECPR	S6	1.142	0.314
Pikfyve	1-phosphatidylinositol 3-phosphate 5-kinase	D3Z5N5; Q9Z1T6	S88;S88	TQSVRSPVPYK	S6	0.956	0.721
Pikfyve	1-phosphatidylinositol 3-phosphate 5-kinase	D3Z5N5; Q9Z1T6	S329,S340,S329	SGSPMVPSYETSVSPQANR	S3,S14	1.116	0.394
Pikfyve	1-phosphatidylinositol 3-phosphate 5-kinase	D3Z5N5; Q9Z1T6	S340,S/Y/T;S329,S/Y/T	SGSPMVPSYETSVSPQANR	S14,S/Y/T	0.908	0.625
Pikfyve	1-phosphatidylinositol 3-phosphate 5-kinase	Q9Z1T6	S105	RSSVLDSR	S3	0.921	0.318
Pikfyve	1-phosphatidylinositol 3-phosphate 5-kinase	Q9Z1T6	S475	FDDSDTEQIAEEGDDNLANSASPSKR	S4	0.894	0.200
Pikfyve	1-phosphatidylinositol 3-phosphate 5-kinase	Q9Z1T6	S475	FDDSDTEQIAEEGDDNLANSASPSK	S4	0.975	0.870
Pikfyve	1-phosphatidylinositol 3-phosphate 5-kinase	Q9Z1T6	S475	DIKFDDSDTEQIAEEGDDNLANSASPSK	S7	1.063	0.436
Pikfyve	1-phosphatidylinositol 3-phosphate 5-kinase	D3Z5N5; Q9Z1T6	Ambiguous	SGSPMVPSYETSVSPQANR	S/T/Y	0.994	0.810
Pikfyve	1-phosphatidylinositol 3-phosphate 5-kinase	D3Z5N5; Q9Z1T6	Ambiguous	SSSPIRLPEISGGQTNR	S/T	1.046	0.533
Pikfyve	1-phosphatidylinositol 3-phosphate 5-kinase	D3Z5N5	Ambiguous	RSSVLENTLPHPQESTDSR	S/T	1.011	0.941
Pimreg	Protein PIMREG	Q8BFY7	S128	DRGSPPPSLSQK	S4	1.149	0.009
Pimreg	Protein PIMREG	Q8BFY7	S72	RLESPEPEQQGLQAAAR	S4	1.117	0.166
Pinx1	PIN2/TERF1-interacting telomerase inhibitor 1	Q9CZX5	T228,S233	EAADTDVENSPQHK	T5,S10	1.083	0.034
Pinx1	PIN2/TERF1-interacting telomerase inhibitor 1	Q9CZX5	T228,S233	NKEAADTDVENSPQHK	T7,S12	1.038	0.554
Pip4p2	Type 2 phosphatidylinositol 4,5-bisphosphate 4-phosphatase	Q9CZX7	S18,T22	SPLLSASHSGNVTPTAPPYLQESSPR	S9,T13	0.917	0.614
Pip4p2	Type 2 phosphatidylinositol 4,5-bisphosphate 4-phosphatase	Q9CZX7	Ambiguous	SPLLSASHSGNVTPTAPPYLQESSPR	S/T/Y	0.999	0.994
Pip5k1a	Phosphatidylinositol 4-phosphate 5-kinase type-1 alpha	P70182	S453	SGPSFSRR	S4	0.854	0.074
Pip5k1a	Phosphatidylinositol 4-phosphate 5-kinase type-1 alpha	P70182	S458	SGPSGNSCTSQLMASGEHR	S1	0.990	0.892
Pip5k1a	Phosphatidylinositol 4-phosphate 5-kinase type-1 alpha	P70182	S458	SGPSGNSCTSQLMASGEHR	S1	1.027	0.681
Pip5k1a	Phosphatidylinositol 4-phosphate 5-kinase type-1 alpha	P70182	S458,S472	SGPSGNSCTSQLMASGEHR	S1,S15	1.253	0.023
Pip5k1c	Phosphatidylinositol 4-phosphate 5-kinase type-1 gamma	F8WHW6	S554	TQSSGQDGRPQEEPHAEDLQK	S3	1.033	0.320
Pip5k1c	Phosphatidylinositol 4-phosphate 5-kinase type-1 gamma	F8WHW6	S554	RTQSSGQDGRPQEEPHAEDLQK	S4	1.084	0.423
Pip5k1c	Phosphatidylinositol 4-phosphate 5-kinase type-1 gamma	F8WHW6	Ambiguous	RRTQSSGQDGRPQEEPHAEDLQK	T/S	0.991	0.937
Pitpna	Phosphatidylinositol transfer protein alpha isoform	J3QPW1	Y142	HVEAIYIDIADR	Y6	0.780	0.041
Pitpnm1	Membrane-associated phosphatidylinositol transfer protein 1	O35954	S1236	SISLKLDSEE	S3	1.125	0.253
Pitpnm1	Membrane-associated phosphatidylinositol transfer protein 1	O35954	S300	SSTEARPGTSTAGTPDGPEAPPGPDASPDASFGK	S27	1.043	0.356
Pitpnm1	Membrane-associated phosphatidylinositol transfer protein 1	O35954	S316	SSYSSQHGGGVSPQSLSEWR	S2	1.084	0.612
Pitpnm1	Membrane-associated phosphatidylinositol transfer protein 1	O35954	S621	ASPEPSALPAQR	S2	1.057	0.189
Pitpnm1	Membrane-associated phosphatidylinositol transfer protein 1	O35954	S895	ERPQLTECEEPSIYSPAFPR	S15	1.007	0.970
Pitpnm1	Membrane-associated phosphatidylinositol transfer protein 1	O35954	Ambiguous	SSTEARPGTSTAGTPDGPEAPPGPDASPDASFGK	T/S	0.912	0.550
Pja1	E3 ubiquitin-protein ligase Praja-1	O55176	S32	SRSPFASTR	S3	1.081	0.676
Pja1	E3 ubiquitin-protein ligase Praja-1	O55176	T231	VFFDTDDDDDVPHSTSR	T5	1.004	0.948
Pja2	E3 ubiquitin-protein ligase Praja-2	Q80U04	Ambiguous	EALEVEECSSDDPIIK	S	1.105	0.563
Pkd2	Polycystin-2	O35245	S810	SLDDSEEEDDEDSGHSSR	S5	1.085	0.224
Pkm	Pyruvate kinase PKM	P52480	S127	GSGTAEVELK	S2	1.064	0.266
Pkm	Pyruvate kinase PKM	P52480	S37	LDIDSAPITAR	S5	1.000	1.000
Pkm	Pyruvate kinase PKM	P52480	S55	NTGIICTIGPASR	S12	0.932	0.587
Pkn1	Serine/threonine-protein kinase N1	P70268	S562	LNLGADSDSSSQK	S7	1.046	0.203
Pkn1	Serine/threonine-protein kinase N1	P70268	S920	TDVSNFDEEFTGEAPTLSPPR	S18	1.013	0.788
Pkn1	Serine/threonine-protein kinase N1	P70268	S540,S/T	LIPSAVATGTFSPNASPGAEIR	S16,T/S	1.038	0.742
Pkn2	Serine/threonine-protein kinase N2	Q8BWW9	S582	ASSLGETDESSELR	S3	1.019	0.556
Pkn2	Serine/threonine-protein kinase N2	Q8BWW9	S619	SKSEYELSIPDSGR	S1	1.087	0.624
Pkn3	Serine/threonine-protein kinase N3	Q8K045	T516	LYLQEPAPGTPCTK	T10	0.971	0.883
Pknox1	Homeobox protein PKNOX1	O70477	Ambiguous	TEQDPNCSDPDAEGVSPPPIESQTPMDADK	S/T	0.969	0.789
Pknox1	Homeobox protein PKNOX1	O70477	Ambiguous	TEQDPNCSDPDAEGVSPPPIESQTPMDADK	S/T	1.177	0.311
Pkp2	Plakophilin 2	Q9CQ73	S132	AAAQYSSQK	S7	0.983	0.795
Pkp2	Plakophilin 2	Q9CQ73	S151	LEISPDSSPER	S4	0.922	0.521
Pkp2	Plakophilin 2	Q9CQ73	S151	RLEISPDSSPER	S5	1.027	0.614
Pkp2	Plakophilin 2	Q9CQ73	S188	YARSEILGLR	S4	0.859	0.459
Pkp2	Plakophilin 2	Q9CQ73	S82	TSSVPEYVYK	S3	1.007	0.935
Pkp2	Plakophilin 2	Q9CQ73	S151,S	RLEISPDSSPER	S5,S	0.982	0.737
Pkp4	Plakophilin-4	Q68FH0	S1185	AEQYPGSPDSWV	S7	1.158	0.217
Pkp4	Plakophilin-4	Q68FH0	S220	AQSPSYVTSTGVSPSR	S3	0.985	0.875
Pkp4	Plakophilin-4	Q68FH0	S313	VGSPLTLTDAQTR	S3	0.870	0.487
Pkp4	Plakophilin-4	Q68FH0	S405	SAVSPDLHITPIYEGR	S4	1.136	0.321
Pkp4	Plakophilin-4	Q68FH0	S75	LGAESPSIASTSSTEK	S5	0.891	0.270
Pkp4	Plakophilin-4	Q68FH0	S326,S336	VASPSQGQVGSSSPKR	S3,S13	1.003	0.958
Pkp4	Plakophilin-4	Q68FH0	Ambiguous	AASPYSQRPASPTAVR	S/Y/T	0.987	0.808
Pkp4	Plakophilin-4	Q68FH0	Ambiguous	VASPSQGQVGSSSPKR	S	1.161	0.249
Pla2g4a	Cytosolic phospholipase A2	P47713	S437	HIVSNDSSDSDDEAQGPK	S10	1.002	0.914
Pla2g4a	Cytosolic phospholipase A2	P47713	S726	CSVSLSNVEAR	S2	1.079	0.053
Pla2g4a	Cytosolic phospholipase A2	P47713	S726	QNPSRCSVSLSNVEAR	S7	1.044	0.507
Pla2g4a	Cytosolic phospholipase A2	P47713	S434,S437	HIVSNDSSDSDDEAQGPK	S7,S10	0.955	0.069
Pla2g4a	Cytosolic phospholipase A2	P47713	Ambiguous	HIVSNDSSDSDDEAQGPKGTENEEAEK	S/T	0.963	0.681
Pla2g4a	Cytosolic phospholipase A2	P47713	Ambiguous	RQNPSRCSVSLSNVEAR	S	1.054	0.623
Plaa	Phospholipase A-2-activating protein	P27612	S318	ELSQATIDSK	S3	0.898	0.252
Plcb2	1-phosphatidylinositol 4,5-bisphosphate phosphodiesterase beta-2	A3KGF7	S472	NQFSGPASPSK	S8	0.964	0.739
Plcb3	1-phosphatidylinositol 4,5-bisphosphate phosphodiesterase beta-3	P51432	S1105	NNSISEAK	S3	1.023	0.630
Plcg2	1-phosphatidylinositol 4,5-bisphosphate phosphodiesterase gamma-2	Q8CIH5	T677	EGTNSYAITFR	T3	0.901	0.105
Plcl2	Inactive phospholipase C-like protein 2	Q8K394	S1114	SLEAIPEK	S1	0.892	0.232
Plcl2	Inactive phospholipase C-like protein 2	Q8K394	S1114	SLEAIPEKASDENGD	S1	1.000	0.993
Plcl2	Inactive phospholipase C-like protein 2	Q8K394	S1114	RSLEAIPEKASDENGD	S2	1.031	0.628
Plcl2	Inactive phospholipase C-like protein 2	Q8K394	S1114	RSLEAIPEK	S2	1.099	0.107
Plcl2	Inactive phospholipase C-like protein 2	Q8K394	S18	GAAGGALPTSPSPALGAK	S12	1.020	0.448
Plcl2	Inactive phospholipase C-like protein 2	Q8K394	S65	YDSGGVSNGDCSLGVSGDEAR	S16	0.866	0.013
Plcl2	Inactive phospholipase C-like protein 2	Q8K394	S72	TSPGRGPLGVALAR	S2	0.969	0.644
Plcl2	Inactive phospholipase C-like protein 2	Q8K394	S87	TPSPAAGPVPR	S3	0.980	0.658
Plcl2	Inactive phospholipase C-like protein 2	Q8K394	T585	LSSNCSGVEGDVTDEDEGAEMSQR	T13	1.060	0.566
Plcl2	Inactive phospholipase C-like protein 2	Q8K394	T585	LSSNCSGVEGDVTDEDEGAEMSQR	T13	1.027	0.696
Plcl2	Inactive phospholipase C-like protein 2	Q8K394	T585	KLSSNCSGVEGDVTDEDEGAEMSQR	T14	1.063	0.182
Plcl2	Inactive phospholipase C-like protein 2	Q8K394	T585	KLSSNCSGVEGDVTDEDEGAEMSQR	T14	1.010	0.853
Plcl2	Inactive phospholipase C-like protein 2	Q8K394	Ambiguous	ARYDSGGVSNGDCSLGVSGDEAR	S/Y	0.978	0.758
Pld1	Phospholipase	D6RH77	S167	SSENAIQEEQFFGR	S2	1.135	0.626
Pld2	Phospholipase	Q6NV49	S146	EAAAEDIPSLPR	S9	1.077	0.083
Plec	Plectin (Fragment)	A0A0R4J221	S48	SGGGSVGNGSVLDPAER	S5	0.986	0.775
Plec	Plectin (Fragment)	A0A0R4J221; Q9QXS1	S626;S728	NDESQLSPATR	S7	1.070	0.002
Plec	Plectin (Fragment)	A0A0R4J221; Q9QXS1	S626;S728	ARNDESQLSPATR	S9	0.854	0.260
Plec	Plectin	Q9QXS1	S1443	AQLEPVASPAK	S8	1.056	0.451
Plec	Plectin	Q9QXS1	S1729	RASFAEK	S3	0.922	0.272
Plec	Plectin	Q9QXS1	S2369	QRSQVEEELFSVR	S3	1.117	0.446
Plec	Plectin	Q9QXS1	S4393	SSSVGSSSSYPISSAGPR	S3	1.079	0.072
Plec	Plectin	Q9QXS1	S4573	LRDVSAYSK	S5	0.990	0.878
Plec	Plectin	Q9QXS1	S4633	GYYSPYSVSGSGSTAGSR	S17	0.996	0.914
Plec	Plectin	Q9QXS1	S4633	GYYSPYSVSGSGSTAGSRTGSR	S17	0.980	0.665
Plec	Plectin	Q9QXS1	S4682	YASGPSASLGGPESAVA	S8	1.286	0.059
Plec	Plectin	Q9QXS1	T4037	QITVEELVR	T3	1.100	0.596
Plec	Plectin	Q9QXS1	Y3797	LTAEDLYEAR	Y7	1.016	0.920
Plec	Plectin	Q9QXS1	S4396,S/Y	SSSVGSSSSYPISSAGPR	S6,S/Y	1.011	0.809
Plec	Plectin	Q9QXS1	S4625,S4629	GYYSPYSVSGSGSTAGSR	S9,S13	0.926	0.150
Plec	Plectin	Q9QXS1	S4629,S/T/Y	GYYSPYSVSGSGSTAGSRTGSR	S13,T/S/Y	0.911	0.284
Plec	Plectin	Q9QXS1	S4620,S4633,T/S/Y	GYYSPYSVSGSGSTAGSRTGSR	S4,S17,T/S/Y	1.054	0.765
Plec	Plectin	Q9QXS1	Ambiguous	SRSSSVGSSSSYPISSAGPR	S/Y	0.874	0.121
Plec	Plectin	Q9QXS1	Ambiguous	QQNLASYDYVR	S/Y	1.061	0.293
Plec	Plectin (Fragment)	A0A0R4J221	Ambiguous	RSGGGSVGNGSVLDPAER	S	1.143	0.449
Plec	Plectin	Q9QXS1	Ambiguous	DAPDGPSVEAEPEYTFEGLR	Y/T/S	0.997	0.986
Plec	Plectin	Q9QXS1	Ambiguous	GYYSPYSVSGSGSTAGSRTGSRTGSR	S/T/Y	1.092	0.244
Plec	Plectin	Q9QXS1	Ambiguous	SRSSSVGSSSSYPISSAGPR	S/Y	1.030	0.707
Plec	Plectin	Q9QXS1	Ambiguous	GYYSPYSVSGSGSTAGSRTGSRTGSR	S/T/Y	1.160	0.124
Plekha1	Pleckstrin homology domain-containing family A member 1	Q8BUL6	S308	RLSNPCIQR	S3	0.928	0.557
Plekha1	Pleckstrin homology domain-containing family A member 1	Q8BUL6	S375	SRLSLQESQLPK	S4	0.965	0.700
Plekha2	Pleckstrin homology domain-containing family A member 2	Q9ERS5	S184	SQSYIPTSGCRPSTGPPLIK	S3	1.050	0.203
Plekha2	Pleckstrin homology domain-containing family A member 2	Q9ERS5	S401	HRSEPQHPK	S3	0.997	0.797
Plekha2	Pleckstrin homology domain-containing family A member 2	Q9ERS5	T133	NLTAPPTLEK	T3	0.983	0.614
Plekha3	Pleckstrin homology domain-containing family A member 3	Q9ERS4	S289	TQSEEPLLPFS	S3	1.403	0.339
Plekha5	Pleckstrin homology domain-containing, family A member 5	E9Q6H8	S1243	SLSPSPDSSTAADPPTPPQLR	S3	1.044	0.648
Plekha5	Pleckstrin homology domain-containing, family A member 5	E9Q6H8	S1243,T1256	SLSPSPDSSTAADPPTPPQLR	S3,T16	1.091	0.426
Plekhf2	Pleckstrin homology domain-containing family F member 2	Q91WB4	S226	SDSYSQSLK	S3	1.113	0.081
Plekhf2	Pleckstrin homology domain-containing family F member 2	Q91WB4	S239	SPLNDASDDDDDDSSD	S7	1.023	0.658
Plekhg1	rin homology domain-containing, family G (with RhoGef domain) member 1 (Fra	F6S200	S1423	GPSPNQQNIVQSLR	S3	1.125	0.340
Plekhg1	rin homology domain-containing, family G (with RhoGef domain) member 1 (Fra	F6S200	S669	RASSAGESNACPPEVR	S3	1.076	0.426
Plekhg1	rin homology domain-containing, family G (with RhoGef domain) member 1 (Fra	F6S200	Ambiguous	ASSAGESNACPPEVR	S	1.113	0.092
Plekhg2	Pleckstrin homology domain-containing family G member 2	D3Z5N8	S1302	APSPPPQPQPPAPPAR	S3	1.020	0.713
Plekhg2	Pleckstrin homology domain-containing family G member 2	D3Z5N8	S442	HIPEPPTSPLDSPRPR	S8	1.021	0.758
Plekhg2	Pleckstrin homology domain-containing family G member 2	D3Z5N8	S49	GSGSSTSLSTVGSEGDPSPACSASRPEPLPEPPIR	S2	0.856	0.275
Plekhg3	Pleckstrin homology domain-containing family G member 3	Q4VAC9	S1339	FQALNSVG	S6	0.809	0.201
Plekhg3	Pleckstrin homology domain-containing family G member 3	Q4VAC9	S550	QQSFSGSLEGLAGHDGSEK	S7	0.973	0.747
Plekhg3	Pleckstrin homology domain-containing family G member 3	Q4VAC9	S736	SSLAIEDGK	S1	0.961	0.334
Plekhg3	Pleckstrin homology domain-containing family G member 3	Q4VAC9	S737	RSSLAIEDGK	S3	1.056	0.190
Plekhg3	Pleckstrin homology domain-containing family G member 3	Q4VAC9	S758	SSSVLSLEGSDK	S2	0.866	0.122
Plekhg3	Pleckstrin homology domain-containing family G member 3	Q4VAC9	S76	GPLSPFNGR	S4	1.060	0.102
Plekhg3	Pleckstrin homology domain-containing family G member 3	Q4VAC9	S762,S	SSSVLSLEGSDK	S6,S	1.148	0.352
Plekhg3	Pleckstrin homology domain-containing family G member 3	Q4VAC9	Ambiguous	SSAAVETSPR	T/S	0.963	0.657
Plekhm1	Pleckstrin homology domain-containing family M member 1	Q7TSI1	S466	SAAGLCTSPVQDTPESR	S8	1.305	0.010
Plekhm1	Pleckstrin homology domain-containing family M member 1	Q7TSI1	S491	SCSLGALDK	S3	1.093	0.308

Plekhm1	Pleckstrin homology domain-containing family M member 1	Q7TSI1	S436,S/T	LVVSSPTSPK	S8,S/T	1.021	0.823
Plekhm1	Pleckstrin homology domain-containing family M member 1	Q7TSI1	Ambiguous	QLGTAQAGPAQSTSDQQPSSPVGGAAGQGSGPWK	T/S	1.151	0.480
Plekhm2	Pleckstrin homology domain-containing family M member 2	Z4YJW6	S573	DQPSPCLSSAEDSGVEEGQGSPSEMTHPSEFR	S4	0.914	0.333
Plekhm2	Pleckstrin homology domain-containing family M member 2	Z4YJW6	S573	DQPSPCLSSAEDSGVEEGQGSPSEMTHPSEFR	S4	0.951	0.519
Plekhm2	Pleckstrin homology domain-containing family M member 2	Z4YJW6	T256	STASDLTSSK	T7	1.041	0.505
Plekhm2	Pleckstrin homology domain-containing family M member 2	Z4YJW6	S250,S253	STASDLTSSK	S1,S4	1.236	0.021
Plekhm3	Pleckstrin homology domain-containing family M member 3	Q8BM47	S152	SRSDVTHVDWR	S3	0.985	0.778
Plekhn1	Probable pleckstrin homology domain-containing family N member 1	E9QP36	S17	LRASFSR	S4	0.914	0.511
Plekho1	Pleckstrin homology domain-containing family O member 1	Q9JIY0	S220	SVAQLAGSRR	S8	1.057	0.148
Plekho1	Pleckstrin homology domain-containing family O member 1	Q9JIY0	S220	SVAQLAGSR	S8	1.060	0.298
Plekho1	Pleckstrin homology domain-containing family O member 1	Q9JIY0	S226	ADSDRIQPSSQR	S3	0.879	0.222
Plekho1	Pleckstrin homology domain-containing family O member 1	Q9JIY0	S270	CASLEEILSQR	S3	1.008	0.834
Plekho1	Pleckstrin homology domain-containing family O member 1	Q9JIY0	T253	GAPYTPQALK	T5	1.026	0.551
Plekho2	Pleckstrin homology domain-containing family O member 2	Q8K124	S211	ALMPPVKPSPGPETSAVEDSKETPAGER	S9	0.979	0.838
Plekho2	Pleckstrin homology domain-containing family O member 2	Q8K124	S268	SLPNSTLSEK	S8	1.018	0.734
Plekho2	Pleckstrin homology domain-containing family O member 2	Q8K124	S292	VSWENPSPEKPSAPESAQLSSSETPEATPR	S20	1.036	0.426
Plekho2	Pleckstrin homology domain-containing family O member 2	Q8K124	S395	SSSLGDLLR	S3	0.999	0.980
Plekho2	Pleckstrin homology domain-containing family O member 2	Q8K124	S403	SSSLGDLLRESPQHPR	S11	0.934	0.273
Plekho2	Pleckstrin homology domain-containing family O member 2	Q8K124	T233	ALTPDSASSGANPESQEDAETPAK	T3	1.043	0.629
Plekho2	Pleckstrin homology domain-containing family O member 2	Q8K124	T233	ALTPDSASSGANPESQEDAETPAKEDSDVK	T3	1.011	0.639
Plekho2	Pleckstrin homology domain-containing family O member 2	Q8K124	T476	DLGELNQETPGLVQK	T9	1.033	0.769
Plekho2	Pleckstrin homology domain-containing family O member 2	Q8K124	S279,S/T	VSWENPSPEKPSAPESAQLSSSETPEATPR	S7,S/T	0.944	0.616
Plekho2	Pleckstrin homology domain-containing family O member 2	Q8K124	S394,S403	SSSLGDLLRESPQHPR	S2,S11	0.879	0.442
Plekho2	Pleckstrin homology domain-containing family O member 2	Q8K124	T233,S236	ALTPDSASSGANPESQEDAETPAKEDSDVK	T3,S6	1.036	0.344
Plin3	Perilipin-3	Q9DBG5	S245	LGSLSER	S3	0.991	0.923
Plk2	Serine/threonine-protein kinase PLK2	P53351	Ambiguous	GTLGSCSSSSECLEDSTMGSVADTVAR	S/T	1.137	0.042
Plp2	Proteolipid protein 2	Q9R1Q7	T149	HTAAPTDPTDGP	T9	1.197	0.112
Pls3	Plastin-3	B1AX58	T540,S542	ANDDIIVNWVNRTLSEAGK	T13,S15	1.014	0.878
Plxna1	Plexin-A1	P70206	S1633	TASSPDSLR	S7	1.050	0.416
Plxna1	Plexin-A1	P70206	T1613	QTSAYNISNSSTFTK	T12	0.963	0.518
Plxna2	Plexin-A2	P70207	S1630	YTGSPDSLR	S4	0.961	0.085
Plxnb2	Plexin-B2	B2RXS4	S1248	SQLEGLEESVRDR	S9	1.028	0.734
Plxnb2	Plexin-B2	B2RXS4	S1574	VGVSQQPEDSQQDLPGER	S10	1.020	0.754
Pmf1	Polyamine-modulated factor 1	Q9CPV5	Ambiguous	GPEGSSPEAVPGDATIPR	S/T	1.036	0.845
Pml	Protein PML	Q60953	S17	VPAPPGSPCR	S7	1.060	0.444
Pml	Protein PML	Q60953	S404	INAAVASPEAASNQPEAASTHPVTTSTPEDLEQPK	S7	1.083	0.799
Pml	Protein PML	Q60953	S557	ILLPNNNHVTSDTGETEER	S11	1.013	0.839
Pml	Protein PML	Q60953	S609	ALDESLAEPHLEDR	S5	0.945	0.580
Pml	Protein PML	Q60953	S404,S/T	INAAVASPEAASNQPEAASTHPVTTSTPEDLEQPK	S7,S/T	1.431	0.447
Pml	Protein PML	Q60953	Ambiguous	ATSPPHLDGTSNPESTVPEK	S/T	0.972	0.559
Pml	Protein PML	Q60953	Ambiguous	ATSPPHLDGTSNPESTVPEKK	T/S	0.959	0.340
Pml	Protein PML	Q60953	Ambiguous	ATSPPHLDGTSNPESTVPEK	T/S	0.992	0.905
Pml	Protein PML	Q60953	Ambiguous	ATSPPHLDGTSNPESTVPEKK	T/S	0.971	0.520
Pnkd	MCG114807, isoform CRA_c	A0A0R4J1J1	Ambiguous	ASPQQAQFPEVGSGAQT	T/S	1.114	0.001
Pnkp	Bifunctional polynucleotide phosphatase/kinase	G5E8N7	S114	WEELSTSGSQPDAPPDTPGDPEEGEDTEPQK	S9	0.979	0.461
Pnn	Pinin	Q3TUQ5	S100	QESDPEDDDVKKPALQSSVVATSK	S3	0.962	0.478
Pnn	Pinin	Q3TUQ5	S100	QESDPEDDDVK	S3	0.940	0.673
Pnn	Pinin	Q3TUQ5	S100	ESRQESDPEDDDVK	S6	0.988	0.873
Pnn	Pinin	Q3TUQ5	S347	EAGIVHSDAEKEQEEEEQKQEMEVK	S7	1.061	0.442
Pnn	Pinin	Q3TUQ5	S347	EAGIVHSDAEKEQEEEEQKQEMEVK	S7	0.934	0.536
Pnn	Pinin	Q3TUQ5	S66	GFSDSGGGPPAK	S3	1.630	0.003
Pnn	Pinin	Q3TUQ5	S66	RGFSDSGGGPPAK	S4	1.631	0.003
Pnpla2	Patatin-like phospholipase domain-containing protein 2	Q8BJ56	Ambiguous	APASPTAADPATPQDPPGLPPC	T/S	1.050	0.347
Pnpla6	Neuropathy target esterase	Q3TRM4	S1319	DEGGSPEGASPSTASEVEEEK	S5	1.176	0.115
Pnpla7	Patatin-like phospholipase domain-containing protein 7	A2AJ88	S341	QMSYGPEEQLER	S3	1.228	0.036
Pnpla7	Patatin-like phospholipase domain-containing protein 7	A2AJ88	S341	QMSYGPEEQLER	S3	1.080	0.631
Pnpla7	Patatin-like phospholipase domain-containing protein 7	A2AJ88	S341	RQMSYGPEEQLER	S4	1.077	0.137
Pnpla7	Patatin-like phospholipase domain-containing protein 7	A2AJ88	S379	SCSVPLPSNHGEVDELR	S3	1.092	0.563
Pnpo	Pyridoxine-5'-phosphate oxidase	Q91XF0	S164	SSQIGAVVSR	S1	0.989	0.862
Pnpo	Pyridoxine-5'-phosphate oxidase	Q91XF0	Ambiguous	GLATGDSPLGPMTHHGEEDWVYER	T/S/Y	1.014	0.811
Pola2	DNA polymerase alpha subunit B	P33611	S126	RVSSTPETPLTK	S4	1.048	0.389
Pola2	DNA polymerase alpha subunit B	P33611	S126	VSSTPETPLTK	S3	1.019	0.766
Pold3	DNA polymerase delta subunit 3	Q9EQ28	S306	VDLSDEEAKETEHLK	S4	0.949	0.469
Pold3	DNA polymerase delta subunit 3	Q9EQ28	S306	VDLSDEEAK	S4	0.998	0.987
Pold3	DNA polymerase delta subunit 3	Q9EQ28	S306	RVDLSDEEAK	S5	0.938	0.490
Polk	DNA polymerase kappa	Q9QUG2	S748	QDSVQPCTDEVVTGR	S3	0.950	0.476
Poll	DNA polymerase lambda	Q9QXE2	S167	TTLSLSPPHTR	S6	1.199	0.174
Poll	DNA polymerase lambda	Q9QXE2	S175	AVSPPPTAEKPSR	S3	1.005	0.938
Polm	DNA-directed DNA/RNA polymerase mu	Q5NCI3	S12	AGSPHSAVASSTPPSVVR	S3	0.971	0.529
Polr1a	DNA-directed RNA polymerase I subunit RPA1	O35134	S1436	EKQEEEVDYESEEEGEEEEEEEVQEEGNIK	S11	1.088	0.267
Polr2a	DNA-directed RNA polymerase II subunit RPB1	P08775	S1815	YTPQSPTYTPSSPSYSPSSPSYSPTSPK	S5	1.046	0.461
Polr2a	DNA-directed RNA polymerase II subunit RPB1	P08775	S1833;S3692;S5558;S7473;S9402	YSPTSPK	S2	0.999	0.988
Polr2a	DNA-directed RNA polymerase II subunit RPB1	P08775	S1857	YTPTSPSYSPSSPEYTPASPK	S19	0.988	0.823
Polr2a	DNA-directed RNA polymerase II subunit RPB1	P08775	S1878	YSPTSPTYSPTTPK	S5	1.005	0.925
Polr2a	DNA-directed RNA polymerase II subunit RPB1	P08775	S1906	YSPTSPTYSPTSPVYTPTSPK	S19	1.014	0.759
Polr2a	DNA-directed RNA polymerase II subunit RPB1	P08775	S1920;S3842	YSPTSPTYSPTSPK	S12	1.003	0.947
Polr2a	DNA-directed RNA polymerase II subunit RPB1	P08775	S1966	GSTYSPTSPGYSPTSPTYSLTSPAISPDDSDEEN	S30	1.143	0.624
Polr2a	DNA-directed RNA polymerase II subunit RPB1	P08775	S1857,S/Y/T	YTPTSPSYSPSSPEYTPASPK	S19,S/Y/T	0.982	0.789
Polr2a	DNA-directed RNA polymerase II subunit RPB1	P08775	S1875,T/S/Y	YSPTSPKYSPTSPTYSPTTPK	S9,T/S/Y	0.968	0.813
Polr2a	DNA-directed RNA polymerase II subunit RPB1	P08775	S1878,T1880	YSPTSPTYSPTTPK	S5,T7	1.032	0.238
Polr2a	DNA-directed RNA polymerase II subunit RPB1	P08775	T1894,S/T/Y	YSPTSPTYSPTSPVYTPTSPK	T7,S/T/Y	1.035	0.786
Polr2a	DNA-directed RNA polymerase II subunit RPB1	P08775	T1912,S1920,S3842	YSPTSPTYSPTSPK	T4,S12	1.006	0.957
Polr2a	DNA-directed RNA polymerase II subunit RPB1	P08775	S1843,S1857,T/S/Y	YTPTSPSYSPSSPEYTPASPK	S5,S19,T/S/Y	0.925	0.195
Polr2a	DNA-directed RNA polymerase II subunit RPB1	P08775	T1891,T1894,S1906	YSPTSPTYSPTSPVYTPTSPK	T4,T7,S19	1.047	0.607
Polr2a	DNA-directed RNA polymerase II subunit RPB1	P08775	Ambiguous	YSPTSPTYSPTSPKYSPTSPTYSPTSPK	S/Y/T	0.906	0.698
Polr2a	DNA-directed RNA polymerase II subunit RPB1	P08775	Ambiguous	YSPTSPKYSPTSPK	T/S/Y	0.967	0.516
Polr2m	DNA-directed RNA polymerase II subunit GRINL1A	Q6P6I6	Ambiguous	GNEETLEAGYTVNSSPAAHIR	S/T/Y	1.138	0.358
Polr3c	DNA-directed RNA polymerase III subunit RPC3	Q9D483	S204	SSDEDATGEPK	S1	0.999	0.977
Polr3e	DNA-directed RNA polymerase III subunit RPC5	Q9CZT4	S161	EAANEAGDSSQDEAEEDVK	S9	1.011	0.757
Pom121	Nuclear envelope pore membrane protein POM 121	Q8K3Z9	S159	RPPARSPPPASAVQR	S6	0.903	0.673
Pom121	Nuclear envelope pore membrane protein POM 121	Q8K3Z9	S244	MVCSPVTVR	S4	0.874	0.331
Pom121	Nuclear envelope pore membrane protein POM 121	Q8K3Z9	S409	SGPTSSPFSSPASSR	S6	1.062	0.403
Pom121	Nuclear envelope pore membrane protein POM 121	Q8K3Z9	S441	TREEEPCQQSSSSPPLVTDKESPGEK	S13	1.043	0.619
Pom121	Nuclear envelope pore membrane protein POM 121	Q8K3Z9	S409,S413	SGPTSSPFSSPASSR	S6,S10	0.942	0.191
Pom121	Nuclear envelope pore membrane protein POM 121	Q8K3Z9	Ambiguous	TSSVSSLASACTGGIPSSSR	S/T	0.837	0.019
Pop1	Processing of 1, ribonuclease P/MRP family, (S. cerevisiae)	Q8K205	S62	MSPHLLPESDVNEQSSSK	S2	1.031	0.695
Pop1	Processing of 1, ribonuclease P/MRP family, (S. cerevisiae)	Q8K205	S62	MSPHLLPESDVNEQSSSK	S2	1.068	0.558
Pop1	Processing of 1, ribonuclease P/MRP family, (S. cerevisiae)	Q8K205	S784	EICQLSNEAGIAENQPR	S6	1.106	0.373
Pop5	Ribonuclease P/MRP protein subunit POP5	Q9DB28	S159	SCLLDREPVEELSDSAGEEVAEAME	S15	1.143	0.368
Pou2f2	POU domain protein	H3BKB7	S26	QSLDSPSEHTDTER	S5	1.134	0.036
Ppa1	Inorganic pyrophosphatase	Q9D819	S272;S275;T278	AIVDALPPPCESACSLPTDVDK	S12;S15;T18	0.878	0.282
Ppan	Suppressor of SWI4 1 homolog	Q91YU8	S362	ADGDSDAEDPGAPPEAVGAGQPEDEEDDAEYFR	S5	0.912	0.548
Ppan	Suppressor of SWI4 1 homolog	Q91YU8	S362	ARADGDSDAEDPGAPPEAVGAGQPEDEEDDAEYFR	S7	0.975	0.863
Pparg	Peroxisome proliferator-activated receptor gamma	P37238	S112	VEPASPPYYSEK	S5	0.992	0.937
Ppfia1	Ppfia1 protein	B2RXQ2	S150	QAQSPAGVSSEVEVLK	S4	0.938	0.407
Ppfia1	Ppfia1 protein	B2RXQ2	S239	SSDGSLSHEDLAK	S2	0.916	0.613
Ppfia1	Ppfia1 protein	B2RXQ2	S692	VGSGSLDNLGR	S5	0.924	0.041
Ppfia1	Ppfia1 protein	B2RXQ2	S717	SMSSIPPYPASSLAGSSPPGSGR	S17	1.041	0.145
Ppfia1	Ppfia1 protein	B2RXQ2	S797	GAPHTVSHEDIR	S7	0.994	0.939
Ppfia1	Ppfia1 protein	B2RXQ2	S239,S242	SSDGSLSHEDLAK	S2,S5	0.971	0.417
Ppfia1	Ppfia1 protein	B2RXQ2	S242,S	RSSDGSLSHEDLAK	S6,S	1.032	0.138
Ppfia1	Ppfia1 protein	B2RXQ2	S721,S/Y	SMSSIPPYPASSLAGSSPPGSGR	S21,S/Y	0.725	0.148
Ppfia1	Ppfia1 protein	B2RXQ2	S775,S/T	CETSPPSSPRPLR	S4,S/T	1.132	0.194
Ppfia1	Ppfia1 protein	B2RXQ2	Ambiguous	CETSPPSSPRPLR	S/T	1.049	0.342
Ppfia1	Ppfia1 protein	B2RXQ2	Ambiguous	VTSSMSSPSMQPK	S/T	0.986	0.717
Ppfia1	Ppfia1 protein	B2RXQ2	Ambiguous	SMSSIPPYPASSLAGSSPPGSGR	S/Y	1.041	0.626
Ppfia1	Ppfia1 protein	B2RXQ2	Ambiguous	RSSDGSLSHEDLAK	S	1.022	0.699
Ppfibp1	Liprin-beta-1 (Fragment)	F6YZ95; Q8C8U0	S137;S538	GSQGTSPFPMSPPSPDSR	S14	0.989	0.702
Ppfibp1	Liprin-beta-1 (Fragment)	F6YZ95; Q8C8U0	S137;S538	SKGSQGTSPFPMSPPSPDSR	S16	0.804	0.431
Ppfibp1	Liprin-beta-1 (Fragment)	F6YZ95; Q8C8U0	S137;S538	GSQGTSPFPMSPPSPDSR	S14	1.019	0.893
Ppfibp1	Liprin-beta-1 (Fragment)	F6YZ95; Q8C8U0	S159;S560	SQSTTFNPDDMSEPEFK	S3	1.017	0.140
Ppfibp1	Liprin-beta-1 (Fragment)	F6YZ95; Q8C8U0	S159;S560	SQSTTFNPDDMSEPEFK	S3	1.105	0.076
Ppfibp1	Liprin-beta-1 (Fragment)	F6YZ95; Q8C8U0	S159;S560	SQSTTFNPDDMSEPEFKR	S3	1.011	0.851
Ppfibp1	Liprin-beta-1 (Fragment)	F6YZ95; Q8C8U0	S159;S560	RSQSTTFNPDDMSEPEFKR	S4	1.132	0.019
Ppfibp1	Liprin-beta-1 (Fragment)	F6YZ95; Q8C8U0	S23;S435	SSSLGNLK	S3	1.079	0.197
Ppfibp1	Liprin-beta-1 (Fragment)	F6YZ95	S88	TASAPNLDR	S3	1.037	0.536
Ppfibp1	Liprin-beta-1 (Fragment)	F6YZ95	S99	SASAPTLAETEKETAEHLNLAGTSR	S3	1.055	0.132
Ppfibp1	Liprin-beta-1 (Fragment)	F6YZ95	S99	SASAPTLAETEK	S3	1.047	0.589
Ppfibp1	Liprin-beta-1	Q8C8U0	S500	TASAPNLAETEKETAEHLNLAGTSR	S3	1.014	0.720
Ppfibp1	Liprin-beta-1	Q8C8U0	S500	TASAPNLAETEK	S3	1.037	0.728
Ppfibp1	Liprin-beta-1	Q8C8U0	S595	DLGQSNSDLDMPFAK	S5	0.859	0.007
Ppfibp1	Liprin-beta-1	Q8C8U0	S753	RPSDENSITPSEVQQWTNHR	S3	1.092	0.289
Ppfibp1	Liprin-beta-1	Q8C8U0	S959	SPSASITDEDSNV	S3	0.987	0.852
Ppfibp1	Liprin-beta-1	Q8C8U0	S957,S959	DFAARSPSASITDEDSNV	S6,S8	1.127	0.343
Ppfibp1	Liprin-beta-1 (Fragment)	F6YZ95; Q8C8U0	Ambiguous	RSQSTTFNPDDMSEPEFK	S/T	1.162	0.194
Ppfibp1	Liprin-beta-1 (Fragment)	F6YZ95; Q8C8U0	Ambiguous	SQSTTFNPDDMSEPEFKR	S/T	1.007	0.814
Ppfibp1	Liprin-beta-1 (Fragment)	F6YZ95; Q8C8U0	Ambiguous	RSQSTTFNPDDMSEPEFK	T/S	1.038	0.502
Ppfibp2	Liprin-beta-2	G3X957	S328	TLSINEDEIEGSFR	S3	1.155	0.186

Ppfibp2	Liprin-beta-2	G3X957	S357	SPEEVPKQEISPR	S11	1.016	0.752
Ppfibp2	Liprin-beta-2	G3X957	S405	ESGDKCVDGNQLSPVGEPK	S13	1.259	0.026
Ppfibp2	Liprin-beta-2	G3X957	S518	TQSGNFNTDAPGMAEFR	S3	1.083	0.471
Ppfibp2	Liprin-beta-2	G3X957	S518	TQSGNFNTDAPGMAEFR	S3	0.990	0.831
Ppfibp2	Liprin-beta-2	G3X957	S518	RTQSGNFNTDAPGMAEFR	S4	0.989	0.810
Ppfibp2	Liprin-beta-2	G3X957	T539	GGLRATAGPR	T6	1.139	0.225
Ppfibp2	Liprin-beta-2	G3X957	Ambiguous	CSSPTPGPPPLPQK	S/T	1.055	0.373
Pphln1	Periphilin 1, isoform CRA_a	G3X959	S147	DAPFFRESPVGR	S8	0.952	0.648
Pphln1	Periphilin 1, isoform CRA_a	G3X959	S219	DASPSSSSAVASSK	S3	1.066	0.086
Pphln1	Periphilin 1, isoform CRA_a	G3X959	S219,T/S	TSRDASPSSSSAVASSK	S6,T/S	0.998	0.978
Ppig	Peptidyl-prolyl cis-trans isomerase G	A2AR02	S685	ADREQSPVSK	S6	0.995	0.852
Ppig	Peptidyl-prolyl cis-trans isomerase G	A2AR02	S685	KADREQSPVSK	S7	1.037	0.676
Ppig	Peptidyl-prolyl cis-trans isomerase G	A2AR02	T356	SETPPHWR	T3	0.919	0.141
Ppig	Peptidyl-prolyl cis-trans isomerase G	A2AR02	T356	RSETPPHWR	T4	1.081	0.155
Ppil1	Peptidyl-prolyl cis-trans isomerase-like 1	Q9D0W5	Ambiguous	VGMVETNSQDRPVDDVK	T/S	1.003	0.920
Ppil2	RING-type E3 ubiquitin-protein ligase PPIL2	Q9D787	S493	SAEEEPSTSTATPTAK	S1	1.039	0.322
Ppip5k1	Inositol hexakisphosphate and diphosphoinositol-pentakisphosphate kinase 1	A2ARP1	S1147	TLHSPPLQLR	S4	1.011	0.896
Ppip5k1	Inositol hexakisphosphate and diphosphoinositol-pentakisphosphate kinase 1	A2ARP1	T958	ALQTSPQPVEGTGLPR	T4	0.955	0.443
Ppip5k1	Inositol hexakisphosphate and diphosphoinositol-pentakisphosphate kinase 1	A2ARP1	Ambiguous	ASDEPDRALQTSPQPVEGTGLPR	T/S	0.998	0.980
Ppip5k2	Inositol hexakisphosphate and diphosphoinositol-pentakisphosphate kinase	E9Q9J4	S1005	SGEQITSSPVSPK	S1	1.008	0.757
Ppip5k2	Inositol hexakisphosphate and diphosphoinositol-pentakisphosphate kinase	E9Q9J4	T927	SLKTDDDEPHTSK	T4	0.977	0.653
Ppip5k2	Inositol hexakisphosphate and diphosphoinositol-pentakisphosphate kinase	E9Q9J4	S1005,S1015	SGEQITSSPVSPK	S1,S11	0.666	0.056
Ppm1a	Protein phosphatase 1A	P49443	S375	LNPYKNDDTDSASTDDMW	S11	1.152	0.189
Ppm1g	Protein phosphatase 1G	Q61074	S524	LEEALSTEGAEDTGNSDK	S6	1.032	0.755
Ppm1g	Protein phosphatase 1G	Q61074	S524	LEEALSTEGAEDTGNSDKK	S6	0.991	0.932
Ppm1g	Protein phosphatase 1G	Q61074	Ambiguous	KLEEALSTEGAEDTGNSDKK	S/T	0.991	0.870
Ppm1h	Protein phosphatase 1H	Q3UYC0	S123	SSLPNGEGLQLK	S2	1.339	0.036
Ppm1h	Protein phosphatase 1H	Q3UYC0	S123	RSSLPNGEGLQLK	S3	1.035	0.354
Ppm1h	Protein phosphatase 1H	Q3UYC0	S210	AASLRGGVGAPGSPSTPPTR	S3	0.730	0.304
Ppm1h	Protein phosphatase 1H	Q3UYC0	S220	GGVGAPGSPSTPPTR	S8	1.025	0.406
Ppm1h	Protein phosphatase 1H	Q3UYC0	T111	KAGTITSTPNR	T6	0.950	0.337
Ppm1h	Protein phosphatase 1H	Q3UYC0	T113	AGTITSTPNR	T7	1.013	0.514
Ppm1h	Protein phosphatase 1H	Q3UYC0	S210,S220	AASLRGGVGAPGSPSTPPTR	S3,S13	0.865	0.530
Ppm1h	Protein phosphatase 1H	Q3UYC0	T109,T113	AGTITSTPNR	T3,T7	1.082	0.439
Ppme1	Protein phosphatase methylesterase 1	Q8BVQ5	S243	QCEGITSPEGSK	S7	1.137	0.021
Ppp1ca	Serine/threonine-protein phosphatase PP1-alpha catalytic subunit	P62137	T320	YGQFSGLNPGGRPITPPR	T15	0.993	0.918
Ppp1cb	Serine/threonine-protein phosphatase PP1-beta catalytic subunit	P62141	T316	YQYGGLNSGRPVTPPR	T13	1.088	0.274
Ppp1cb	Serine/threonine-protein phosphatase PP1-beta catalytic subunit	P62141	T316	YQYGGLNSGRPVTPPRTANPPK	T13	0.933	0.521
Ppp1r11	E3 ubiquitin-protein ligase PPP1R11	A5A4Y9	S82	AFGESSTESDEDEEEGCSHK	S9	0.955	0.209
Ppp1r11	E3 ubiquitin-protein ligase PPP1R11	A5A4Y9	S82,S/T	AFGESSTESDEDEEEGCSHK	S9,S/T	1.157	0.440
Ppp1r11	E3 ubiquitin-protein ligase PPP1R11	A5A4Y9	T111,T/S	RPTTPAPTPTTPPQPPDPSKPPPGPMQH	T8,T/S	0.796	0.161
Ppp1r11	E3 ubiquitin-protein ligase PPP1R11	A5A4Y9	Ambiguous	RPTTPAPTPTTPPQPPDPSKPPPGPMQH	T/S	1.054	0.359
Ppp1r12a	Protein phosphatase 1 regulatory subunit 12A	Q9DBR7	S507	LASTSDIEEKENR	S3	1.354	0.001
Ppp1r12a	Protein phosphatase 1 regulatory subunit 12A	Q9DBR7	S507	RLASTSDIEEKENR	S4	1.290	0.001
Ppp1r12a	Protein phosphatase 1 regulatory subunit 12A	Q9DBR7	S507	LASTSDIEEK	S3	1.469	0.000
Ppp1r12a	Protein phosphatase 1 regulatory subunit 12A	Q9DBR7	S507	RLASTSDIEEK	S4	1.488	0.001
Ppp1r12a	Protein phosphatase 1 regulatory subunit 12A	Q9DBR7	S666	SYLTPVRDEESESQR	S1	0.935	0.273
Ppp1r12a	Protein phosphatase 1 regulatory subunit 12A	Q9DBR7	S902	YDSSSTSSSDRYDSLLGR	S14	1.038	0.641
Ppp1r12a	Protein phosphatase 1 regulatory subunit 12A	Q9DBR7	S909	SASYSYLEDR	S3	1.120	0.229
Ppp1r12a	Protein phosphatase 1 regulatory subunit 12A	Q9DBR7	S994	RISEMEEELK	S3	1.096	0.047
Ppp1r12a	Protein phosphatase 1 regulatory subunit 12A	Q9DBR7	T498	LAYVTPTIPR	T5	0.947	0.424
Ppp1r12a	Protein phosphatase 1 regulatory subunit 12A	Q9DBR7	S870,S/T	RSTGVSFWTQDSDENEQERQSDTEDGSSK	S21,S/T	1.137	0.180
Ppp1r12a	Protein phosphatase 1 regulatory subunit 12A	Q9DBR7	T852,S855	STGVSFWTQDSDENEQERQSDTEDGSSKR	T2,S5	1.138	0.017
Ppp1r12a	Protein phosphatase 1 regulatory subunit 12A	Q9DBR7	Ambiguous	TGSYGALAEISASK	S/T/Y	0.974	0.769
Ppp1r12a	Protein phosphatase 1 regulatory subunit 12A	Q9DBR7	Ambiguous	STGVSFWTQDSDENEQERQSDTEDGSSK	S/T	1.435	0.033
Ppp1r12b	MCG130490	A6H644	S833	LESGGTNPTSSDSYSDR	S3	0.947	0.271
Ppp1r12b	MCG130490	A6H644; Q9DBR7	Ambiguous	RSTQGVTLTDLQEAEK	S/T	0.921	0.430
Ppp1r12b	MCG130490	A6H644; Q9DBR7	Ambiguous	STQGVTLTDLQEAEK	S/T	1.008	0.937
Ppp1r12c	Protein phosphatase 1 regulatory subunit 12C	Q3UMT1	S403	ALNGVSSPVSSNPK	S7	1.057	0.381
Ppp1r12c	Protein phosphatase 1 regulatory subunit 12C	Q3UMT1	S454	SASSSLLEK	S3	0.973	0.625
Ppp1r12c	Protein phosphatase 1 regulatory subunit 12C	Q3UMT1	S647	RGPAEGEEAGPAERSPECSTVDGGSQVR	S15	1.050	0.900
Ppp1r12c	Protein phosphatase 1 regulatory subunit 12C	Q3UMT1	S647	GPAEGEEAGPAERSPECSTVDGGSQVR	S14	0.939	0.743
Ppp1r12c	Protein phosphatase 1 regulatory subunit 12C	Q3UMT1	Ambiguous	RSTQGVTLTDLK	S/T	0.911	0.041
Ppp1r12c	Protein phosphatase 1 regulatory subunit 12C	Q3UMT1	Ambiguous	TGSTGALGPSERR	S/T	0.892	0.545
Ppp1r12c	Protein phosphatase 1 regulatory subunit 12C	Q3UMT1	Ambiguous	TGSTGALGPSER	T/S	0.979	0.860
Ppp1r13b	Apoptosis-stimulating of p53 protein 1	Q62415	S331	VNGTSSPQSPLSTSGR	S5	1.045	0.682
Ppp1r13b	Apoptosis-stimulating of p53 protein 1	Q62415	S708	RSSITEPEGPGGPNIQK	S3	1.048	0.496
Ppp1r13b	Apoptosis-stimulating of p53 protein 1	Q62415	Ambiguous	SSITEPEGPGGPNIQK	S/T	0.986	0.934
Ppp1r13l	RelA-associated inhibitor	Q5I1X5	S158	APSPRPGIGPVR	S3	1.186	0.246
Ppp1r13l	RelA-associated inhibitor	Q5I1X5	T340	SLGSAGPSGTLPR	T10	0.881	0.244
Ppp1r13l	RelA-associated inhibitor	Q5I1X5	T72	YSTSPVPEHFGSR	T3	0.950	0.609
Ppp1r13l	RelA-associated inhibitor	Q5I1X5	Ambiguous	SESAPSLHPYSPLSPK	S/Y	1.043	0.210
Ppp1r13l	RelA-associated inhibitor	Q5I1X5	Ambiguous	IPMPPSSPQPR	S	0.977	0.821
Ppp1r13l	RelA-associated inhibitor	Q5I1X5	Ambiguous	AGPPAPAPPAPIPPPAPPQSSPPEQPQSMEMR	S	0.839	0.397
Ppp1r18	Phostensin	Q8BQ30	S134	SSPGNLRDQSPK	S10	0.875	0.395
Ppp1r18	Phostensin	Q8BQ30	S146	EERLSPR	S5	1.048	0.557
Ppp1r18	Phostensin	Q8BQ30	S194	LSPGETPEESLR	S2	1.061	0.380
Ppp1r18	Phostensin	Q8BQ30	S194	WRLSPGETPEESLR	S4	1.050	0.346
Ppp1r18	Phostensin	Q8BQ30	S208	LAGSGDDSPK	S4	0.996	0.966
Ppp1r18	Phostensin	Q8BQ30	S212	LAGSGDDSPKR	S8	1.015	0.752
Ppp1r18	Phostensin	Q8BQ30	S381	AQQNLCSGPSPLPPEHSGTEGSR	S10	1.070	0.163
Ppp1r18	Phostensin	Q8BQ30	S464	CAPPASPAPPVNPATADAAGSGSGK	S6	0.975	0.635
Ppp1r2	Protein phosphatase inhibitor 2	Q9DCL8	S122	TREQESSGEEDNDLSPEER	S6	1.043	0.533
Ppp1r2	Protein phosphatase inhibitor 2	Q9DCL8	S122,S123	TREQESSGEEDNDLSPEEREK	S6,S7	0.847	0.133
Ppp1r2	Protein phosphatase inhibitor 2	Q9DCL8	S122,S123	TREQESSGEEDNDLSPEER	S6,S7	0.977	0.868
Ppp1r2	Protein phosphatase inhibitor 2	Q9DCL8	Ambiguous	IDEPNTPYHNMIGDDEDAYSDSEGNEVMTPDILAK	S/T/Y	1.082	0.664
Ppp1r35	Protein phosphatase 1 regulatory subunit 35	Q9D8C8	S53	APEPEPGLDLSLSPSPLPESPK	S20	1.728	0.374
Ppp1r35	Protein phosphatase 1 regulatory subunit 35	Q9D8C8	S85	FQLAPPSPVR	S7	1.005	0.983
Ppp1r3d	Protein phosphatase 1 regulatory subunit	A2AJW4	S25	SLSCLSDMDR	S3	1.244	0.004
Ppp1r7	Protein phosphatase 1 regulatory subunit 7	Q3UM45	S12	GAGQQQSQEMMEVDR	S7	0.842	0.395
Ppp1r7	Protein phosphatase 1 regulatory subunit 7	Q3UM45	S24	VESEESGDEEGKK	S3	1.019	0.664
Ppp1r7	Protein phosphatase 1 regulatory subunit 7	Q3UM45	S24	RVESEESGDEEGKK	S4	0.949	0.546
Ppp1r7	Protein phosphatase 1 regulatory subunit 7	Q3UM45	S27	VESEESGDEEGK	S6	1.083	0.426
Ppp1r7	Protein phosphatase 1 regulatory subunit 7	Q3UM45	S323	SLETVYLER	S1	1.078	0.066
Ppp1r7	Protein phosphatase 1 regulatory subunit 7	Q3UM45	S24,S27	VESEESGDEEGKK	S3,S6	1.013	0.908
Ppp1r7	Protein phosphatase 1 regulatory subunit 7	Q3UM45	S24,S27	RVESEESGDEEGK	S4,S7	0.977	0.393
Ppp1r7	Protein phosphatase 1 regulatory subunit 7	Q3UM45	S24,S27	RVESEESGDEEGKK	S4,S7	0.991	0.932
Ppp1r9a	Protein phosphatase 1, regulatory subunit 9A	H3BJD6	S192	GSSDSLDSLSPR	S10	1.076	0.474
Ppp1r9b	Neurabin-2	Q6R891	S100	ASSLNENVDHSALLK	S3	1.465	0.034
Ppp1r9b	Neurabin-2	Q6R891	S94	GVRLSLPR	S5	1.108	0.392
Ppp1r9b	Neurabin-2	Q6R891	T361	AEAVPEEEAAASVATLER	T15	1.041	0.686
Ppp2r3c	Serine/threonine-protein phosphatase 2A regulatory subunit B'' subunit gamma	Q9JK24	S13	LASPNTDPK	S3	1.002	0.991
Ppp2r5a	Serine/threonine-protein phosphatase 2A 56 kDa regulatory subunit alpha isoforn	Q6PD03	S41	SQGSSQFR	S4	0.953	0.363
Ppp2r5c	erine/threonine-protein phosphatase 2A 56 kDa regulatory subunit gamma isofor	Q60996	S497	RKSELPQDPHTEK	S3	0.999	0.991
Ppp2r5d	Serine/threonine-protein phosphatase 2A 56 kDa regulatory subunit	Q91V89	S44	DGGGENTDEAQPQPQSQSPSSNK	S18	1.032	0.602
Ppp2r5d	Serine/threonine-protein phosphatase 2A 56 kDa regulatory subunit	Q91V89	S590	AEEFLTASQEAL	S8	1.146	0.491
Ppp2r5e	erine/threonine-protein phosphatase 2A 56 kDa regulatory subunit epsilon isofor	Q61151	Ambiguous	SQSSSQFR	S	0.984	0.834
Ppp4r1	Serine/threonine-protein phosphatase 4 regulatory subunit 1	E9QPR5	T950	LSEDAMSTASSTY	T12	0.955	0.450
Ppp4r1	Serine/threonine-protein phosphatase 4 regulatory subunit 1	E9QPR5	S945,T/S/Y	LSEDAMSTASSTY	S7,T/S/Y	0.927	0.396
Ppp4r1	Serine/threonine-protein phosphatase 4 regulatory subunit 1	E9QPR5	Ambiguous	LSEDAMSTASSTY	Y/S/T	0.967	0.745
Ppp4r2	Serine/threonine-protein phosphatase 4 regulatory subunit 2	A0A0R4J0U2	S159	MNGVMFPGNSPNYTDR	S10	0.883	0.147
Ppp4r2	Serine/threonine-protein phosphatase 4 regulatory subunit 2	A0A0R4J0U2	S159	MNGVMFPGNSPNYTDR	S10	1.156	0.297
Ppp4r2	Serine/threonine-protein phosphatase 4 regulatory subunit 2	A0A0R4J0U2	S159	MNGVMFPGNSPNYTDR	S10	0.978	0.709
Ppp4r2	Serine/threonine-protein phosphatase 4 regulatory subunit 2	A0A0R4J0U2	S226	GHSDSSASESEVSLLSPVK	S16	0.961	0.840
Ppp4r2	Serine/threonine-protein phosphatase 4 regulatory subunit 2	A0A0R4J0U2	S388	SGSDCLETQESGGPPSSK	S16	1.032	0.666
Ppp6r1	Serine/threonine-protein phosphatase 6 regulatory subunit 1	Q7TSI3	S668	SGGSTDSEEEDEEDEEEDEGAEQAACGR	S7	1.042	0.861
Ppp6r1	Serine/threonine-protein phosphatase 6 regulatory subunit 1	Q7TSI3	S739	EADMSSIQIPSSPPAHGSPQLR	S12	1.003	0.973
Ppp6r1	Serine/threonine-protein phosphatase 6 regulatory subunit 1	Q7TSI3	S745	EADMSSIQIPSSPPAHGSPQLR	S18	1.074	0.085
Ppp6r1	Serine/threonine-protein phosphatase 6 regulatory subunit 1	Q7TSI3	S739,S745	EADMSSIQIPSSPPAHGSPQLR	S12,S18	0.929	0.030
Ppp6r1	Serine/threonine-protein phosphatase 6 regulatory subunit 1	Q7TSI3	Ambiguous	IQQFDDEEEEEEEGQGSAESDGEYGAWQGSQPVR	S/Y	0.927	0.671
Ppp6r2	SAPS domain family, member 2, isoform CRA_a	G3X9K4	S289	RVGTEGLVDSFSQGLER	S10	1.006	0.927
Ppp6r2	SAPS domain family, member 2, isoform CRA_a	G3X9K4	S670	NVPGLAAPSSPTQK	S10	1.069	0.241
Ppp6r2	SAPS domain family, member 2, isoform CRA_a	G3X9K4	S746	CSSPVDMDHSNAEGGQSPGPEK	S3	0.932	0.294
Ppp6r2	SAPS domain family, member 2, isoform CRA_a	G3X9K4	Ambiguous	CSSPVDMDHSNAEGGQSPGPEK	S	0.854	0.147
Ppp6r3	SAPS domain family, member 3, isoform CRA_c	G5E8R4	S617	ERIQQFDDGGSDEEDIWEEK	S11	0.835	0.154
Ppp6r3	SAPS domain family, member 3, isoform CRA_c	G5E8R4	S617	IQQFDDGGSDEEDIWEEK	S9	0.977	0.746
Ppp6r3	SAPS domain family, member 3, isoform CRA_c	G5E8R4	T631	HIAFTPESQR	T5	0.913	0.435
Ppp6r3	SAPS domain family, member 3, isoform CRA_c	G5E8R4	T849,S/T	CTAPLTPSSSPEQR	T6,S/T	1.094	0.477
Ppp6r3	SAPS domain family, member 3, isoform CRA_c	G5E8R4	Ambiguous	CTAPLTPSSSPEQR	S/T	0.922	0.252
Pprc1	Peroxisome proliferator-activated receptor gamma coactivator-related protein 1	Q6NZN1	S541	SAGQDSPAEEDALDLCPK	S6	0.879	0.572
Pqlc1	PQ-loop repeat-containing protein 1	Q80XM9	S110	SFAATDSKDEELR	S1	1.053	0.583
Prag1	Inactive tyrosine-protein kinase PRAG1	Q571I4	S1056	DTAPAVSPQPPAQEQDCVVVITR	S7	0.937	0.639
Prag1	Inactive tyrosine-protein kinase PRAG1	Q571I4	S40	ANSLPAGTR	S3	1.039	0.465
Prag1	Inactive tyrosine-protein kinase PRAG1	Q571I4	S532	SSPGGSPVSPAPPLTDHSDGNTGGSSVGPQLLSR	S2	1.274	0.259
Prag1	Inactive tyrosine-protein kinase PRAG1	Q571I4	S687	GRLEAFSPPPPPPK	S7	1.038	0.722
Prag1	Inactive tyrosine-protein kinase PRAG1	Q571I4	S720	VSQSSAESLSPSFR	S10	1.112	0.139
Prag1	Inactive tyrosine-protein kinase PRAG1	Q571I4	S885	GGCGSPSLQCR	S5	1.015	0.886
Prag1	Inactive tyrosine-protein kinase PRAG1	Q571I4	Ambiguous	ESLPSEDDSDQRCSPSGDSEGGEYCSILDCCPESK	S/Y	1.042	0.825

Pram1	PML-RARA-regulated adapter molecule 1	Q6BCL1	S226	SQPQSESIEVSQTSPSK	S1	1.013	0.844
Pram1	PML-RARA-regulated adapter molecule 1	Q6BCL1	S239	KSQPQSESIEVSQTSPSK	S15	1.138	0.265
Pram1	PML-RARA-regulated adapter molecule 1	Q6BCL1	S252	ELDSHSPQPDISTFPK	S6	0.967	0.701
Pram1	PML-RARA-regulated adapter molecule 1	Q6BCL1	S332	ASSEPEVCK	S3	1.039	0.466
Pram1	PML-RARA-regulated adapter molecule 1	Q6BCL1	S368	TSSDPEFNSLPR	S3	0.973	0.499
Pram1	PML-RARA-regulated adapter molecule 1	Q6BCL1	S374,T/S	TSSDPEFNSLPR	S9,T/S	0.983	0.925
Pram1	PML-RARA-regulated adapter molecule 1	Q6BCL1	Ambiguous	ASSEPEVCKVPK	S	0.942	0.189
Prdm2	PR domain-containing 2, with ZNF domain	A2A7B5	S1536	STGPAQASLPSSSFR	S8	0.988	0.658
Prdm2	PR domain-containing 2, with ZNF domain	A2A7B5	S330	SVPTETSEGSPGATPPHAPR	S10	1.061	0.523
Prdm2	PR domain-containing 2, with ZNF domain	A2A7B5	S777	SDSPAWSLSGRDER	S3	1.024	0.706
Prdm2	PR domain-containing 2, with ZNF domain	A2A7B5	S792	ETGSPPCFDEYK	S4	1.070	0.137
Prdm2	PR domain-containing 2, with ZNF domain	A2A7B5	S735,S/T	TSSPPSSPQHSPALR	S3,S/T	0.993	0.672
Prdm2	PR domain-containing 2, with ZNF domain	A2A7B5	S738,S739,S743	TSSPPSSPQHSPALR	S6,S7,S11	0.998	0.946
Prdm2	PR domain-containing 2, with ZNF domain	A2A7B5	Ambiguous	DESSPPQLGQDCLILNSEK	S	1.055	0.759
Prdm2	PR domain-containing 2, with ZNF domain	A2A7B5	Ambiguous	TASPPVLPK	T/S	0.897	0.301
Prdx1	Peroxiredoxin-1	P35700	S32	DISLSEYK	S5	0.949	0.539
Prex1	Phosphatidylinositol 3,4,5-trisphosphate-dependent Rac exchanger 1 protein	Q69ZK0	S1170	DSYSECNSNRDSVLSYTSVR	S12	0.934	0.524
Prex1	Phosphatidylinositol 3,4,5-trisphosphate-dependent Rac exchanger 1 protein	Q69ZK0	S1191	SNSSYLGSDEMGSGDELPCDMR	S13	0.848	0.169
Prex1	Phosphatidylinositol 3,4,5-trisphosphate-dependent Rac exchanger 1 protein	Q69ZK0	S1191	SNSSYLGSDEMGSGDELPCDMR	S13	0.974	0.786
Prex1	Phosphatidylinositol 3,4,5-trisphosphate-dependent Rac exchanger 1 protein	Q69ZK0	S1191	SNSSYLGSDEMGSGDELPCDMR	S13	1.565	0.298
Prex1	Phosphatidylinositol 3,4,5-trisphosphate-dependent Rac exchanger 1 protein	Q69ZK0	S1186,S1191	SNSSYLGSDEMGSGDELPCDMR	S8,S13	1.109	0.468
Prickle3	Prickle planar cell polarity protein 3	Q80VL3	S491	TLSAPPAQR	S3	0.922	0.034
Prickle3	Prickle planar cell polarity protein 3	Q80VL3	T471,S475	QSTPRVSFRDPLVSEGGPR	T3,S7	0.837	0.041
Prickle3	Prickle planar cell polarity protein 3	Q80VL3	Ambiguous	SAPEPPTESPGHPAPHPDDNAFGR	T/S	1.143	0.132
Prkaa1	5'-AMP-activated protein kinase catalytic subunit alpha-1	Q5EG47	S486	SIDDEITEAKSGTATPQR	S11	1.020	0.785
Prkaa1	5'-AMP-activated protein kinase catalytic subunit alpha-1	Q5EG47	S496	SGSISNYR	S3	1.030	0.339
Prkaa1	5'-AMP-activated protein kinase catalytic subunit alpha-1	Q5EG47	T488,T490	SIDDEITEAKSGTATPQR	T13,T15	0.987	0.798
Prkab1	5'-AMP-activated protein kinase subunit beta-1	Q9R078	S182	CSDVSELSSSPPGPYHQEPYMSKPEER	S10	0.923	0.476
Prkab1	5'-AMP-activated protein kinase subunit beta-1	Q9R078	Ambiguous	CSDVSELSSSPPGPYHQEPYMSKPEER	S/Y	0.991	0.944
Prkab2	5'-AMP-activated protein kinase subunit beta-2	Q6PAM0	S182	DLSSSPPGPYGQEMYVFR	S4	0.943	0.414
Prkaca	cAMP-dependent protein kinase catalytic subunit alpha	P05132; P68181	T52;T52	TLGTGSFGR	T4	0.986	0.854
Prkacb	cAMP-dependent protein kinase catalytic subunit beta	P68181	S322	FRGSGDTSNFDDYEEEEIR	S4	1.058	0.453
Prkacb	cAMP-dependent protein kinase catalytic subunit beta	P68181	S339	GSGDTSNFDDYEEEEIRVSITEK	S19	0.990	0.721
Prkag2	5'-AMP-activated protein kinase subunit gamma-2	Q91WG5	S113	TVFPFSYQESPPR	S10	0.892	0.257
Prkag2	5'-AMP-activated protein kinase subunit gamma-2	Q91WG5	S113	TVFPFSYQESPPRSPR	S10	0.971	0.731
Prkag2	5'-AMP-activated protein kinase subunit gamma-2	Q91WG5	S143	ESSPNSNPSTSPGGIR	S11	1.037	0.438
Prkag2	5'-AMP-activated protein kinase subunit gamma-2	Q91WG5	S15	EVSSPGGSSGK	S3	1.103	0.032
Prkag2	5'-AMP-activated protein kinase subunit gamma-2	Q91WG5	S161	TSSVSSSPSTPTQVTK	S6	0.947	0.518
Prkag2	5'-AMP-activated protein kinase subunit gamma-2	Q91WG5	S117,S/Y/T	TVFPFSYQESPPRSPR	S14,S/Y/T	0.983	0.931
Prkag2	5'-AMP-activated protein kinase subunit gamma-2	Q91WG5	S87,S90	GPQPRPSSPVSAPVRPK	S8,S11	1.113	0.216
Prkag2	5'-AMP-activated protein kinase subunit gamma-2	Q91WG5	Ambiguous	IYASSSPPDTGQR	S/Y/T	1.062	0.495
Prkag2	5'-AMP-activated protein kinase subunit gamma-2	Q91WG5	Ambiguous	SSSKESSPNSNPSTSPGGIR	S/T	1.030	0.474
Prkar1a	cAMP-dependent protein kinase type I-alpha regulatory subunit	Q9DBC7	S83	EDEISPPPPNPVVK	S5	1.049	0.287
Prkar1a	cAMP-dependent protein kinase type I-alpha regulatory subunit	Q9DBC7	S83	TDSREDEISPPPPNPVVK	S9	0.991	0.594
Prkar1a	cAMP-dependent protein kinase type I-alpha regulatory subunit	Q9DBC7	S77,S83	TDSREDEISPPPPNPVVK	S3,S9	1.068	0.187
Prkar1a	cAMP-dependent protein kinase type I-alpha regulatory subunit	Q9DBC7	S77,S83	TGIRTDSREDEISPPPPNPVVK	S7,S13	1.033	0.801
Prkar2a	Protein kinase, cAMP dependent regulatory, type II alpha	Q8K1M3	S393	MFGSNLDLMDPGQ	S4	0.925	0.516
Prkar2a	Protein kinase, cAMP dependent regulatory, type II alpha	Q8K1M3	S97	RVSVCAETFNPDEEEEDNDPR	S3	0.996	0.944
Prkar2b	cAMP-dependent protein kinase type II-beta regulatory subunit	P31324	S112	RASVCAEAYNPDEEEDDAESR	S3	1.026	0.850
Prkar2b	cAMP-dependent protein kinase type II-beta regulatory subunit	P31324	S83	GVNFAEEPMRSDSENGEEEEAAEAGAFNAPVINR	S11	0.903	0.640
Prkcb	Protein kinase C beta type	P68404	T500	TFCGTPDYIAPEIIAYQPYGK	T1	1.044	0.758
Prkcb	Protein kinase C beta type	P68404	T642	QPVELTPTDK	T6	1.001	0.992
Prkcd	Protein kinase C delta type	P28867	Y64	TTFDAHIYEGR	Y8	0.851	0.118
Prkce	Protein kinase C epsilon type	P16054	S729	GFSYFGEDLMP	S3	1.113	0.578
Prkce	Protein kinase C epsilon type	P16054	Ambiguous	LAAGAESPQPASGNSPSEDDRSK	S	0.886	0.545
Prkch	Protein kinase C eta type	P23298	S675	NFSYVSPELQL	S3	1.157	0.593
Prkd2	Serine/threonine-protein kinase D2	Q8BZ03	S214	LGSSESLPCTAEELSR	S6	0.870	0.024
Prkd2	Serine/threonine-protein kinase D2	Q8BZ03	S225	STTDLLPR	S1	0.846	0.418
Prkd2	Serine/threonine-protein kinase D2	Q8BZ03	S375	AQSSLGYIPLMR	S3	0.984	0.794
Prkd2	Serine/threonine-protein kinase D2	Q8BZ03	S197,T199	RLSSTSLASGHSVR	S3,T5	1.165	0.096
Prkd3	Serine/threonine-protein kinase	Q5FWX6	S364	GLDDSEEPSPPEDK	S9	1.031	0.692
Prkd3	Serine/threonine-protein kinase	Q5FWX6	S391	TISPSTSNNIPLMR	S3	1.144	0.141
Prkd3	Serine/threonine-protein kinase	Q5FWX6	S735	SVVGTPAYLAPEVLR	S1	0.935	0.489
Prkra	Interferon-inducible double-stranded RNA-dependent protein kinase activator A	Q9WTX2	S18	EDSGTFSLGK	S3	1.277	0.006
Prkra	Interferon-inducible double-stranded RNA-dependent protein kinase activator A	Q9WTX2	Ambiguous	AEAPPLQREDSGTFSLGK	S/T	1.234	0.376
Prmt3	Protein arginine N-methyltransferase 3	Q922H1	Ambiguous	GAELGPEPLELSDSGDDAGWEDEDADTEPAHGR	S/T	0.880	0.583
Proser1	Proline and serine-rich protein 1	Q5PRE5	S596	SEPTSPPPSAFK	S5	1.082	0.047
Prpf3	U4/U6 small nuclear ribonucleoprotein Prp3	Q922U1	S619	WDEQTSNTKGDDDEESDEEAVK	S16	1.059	0.122
Prpf3	U4/U6 small nuclear ribonucleoprotein Prp3	Q922U1	S619	GDDDEESDEEAVKK	S7	1.121	0.166
Prpf3	U4/U6 small nuclear ribonucleoprotein Prp3	Q922U1	T469	LGLTPPPEPK	T4	1.209	0.222
Prpf3	U4/U6 small nuclear ribonucleoprotein Prp3	Q922U1	Ambiguous	IKWDEQTSNTKGDDDEESDEEAVK	T/S	1.080	0.040
Prpf31	U4/U6 small nuclear ribonucleoprotein Prp31	Q8CCF0	S439	QSVVYGGKSTIR	S9	0.996	0.939
Prpf40a	Pre-mRNA-processing factor 40 homolog A	Q9R1C7	S884	SDSPESDTEREK	S6	1.073	0.620
Prpf40a	Pre-mRNA-processing factor 40 homolog A	Q9R1C7	S934	DSGNWDTSGSELSEGELEKR	S13	1.096	0.465
Prpf40a	Pre-mRNA-processing factor 40 homolog A	Q9R1C7	Ambiguous	HKSDSPESDTEREK	S/T	0.986	0.794
Prpf4b	Serine/threonine-protein kinase PRP4 homolog	Q61136	S33	SVNEENGEVSEDQSQNK	S10	1.061	0.370
Prpf4b	Serine/threonine-protein kinase PRP4 homolog	Q61136	S94	EVIEASDKEGLSPAKR	S12	1.046	0.645
Prpf4b	Serine/threonine-protein kinase PRP4 homolog	Q61136	S21,S24	EQAEMDDADNSEKSVNEENGEVSEDQSQNK	S11,S14	0.955	0.551
Prpf4b	Serine/threonine-protein kinase PRP4 homolog	Q61136	S366,S368	SRSPLLNDR	S1,S3	0.975	0.673
Prpf4b	Serine/threonine-protein kinase PRP4 homolog	Q61136	S431,S437	DASPINRWSPTR	S3,S9	1.005	0.938
Prpf4b	Serine/threonine-protein kinase PRP4 homolog	Q61136	S431,S437	SKDASPINRWSPTR	S5,S11	1.066	0.080
Prpf4b	Serine/threonine-protein kinase PRP4 homolog	Q61136	S88,S94	EVIEASDKEGLSPAK	S6,S12	0.876	0.010
Prpf4b	Serine/threonine-protein kinase PRP4 homolog	Q61136	S88,S94	EVIEASDKEGLSPAKR	S6,S12	0.935	0.205
Prpf4b	Serine/threonine-protein kinase PRP4 homolog	Q61136	Ambiguous	EVIEASDKEGLSPAK	S	1.055	0.468
Prpf8	Pre-mRNA-processing-splicing factor 8	Q99PV0	S1358	SGMSHEEDQLIPNLYR	S4	1.094	0.232
Prr12	Proline-rich protein 12	E9PYL2	S330	ASLACSPLGGGEPSPGAGEPSK	S6	1.039	0.849
Prr12	Proline-rich protein 12	E9PYL2	S383	ATGPETAGGGAAGGGGGYRPIIQSPGYK	S24	1.001	0.996
Prr12	Proline-rich protein 12	E9PYL2	Ambiguous	GHGLEPTAPSPR	T/S	1.015	0.820
Prr14	Proline-rich protein 14	Q7TPN9	S135	AASGPEESPSKK	S3	1.315	0.020
Prr14	Proline-rich protein 14	Q7TPN9	S135	AASGPEESPSK	S3	1.115	0.073
Prr14l	Proline-rich 14-like	E9Q7C4	S63	ASPLGLQR	S2	1.025	0.565
Prrc2a	Protein PRRC2A	Q7TSC1	S1002	ETPPGGNLSPAPR	S9	1.019	0.575
Prrc2a	Protein PRRC2A	Q7TSC1	S1087	TASETRSEGSEYEEIPK	S7	0.969	0.543
Prrc2a	Protein PRRC2A	Q7TSC1	S1108	GSETGSETHESDLAPSDK	S6	0.959	0.463
Prrc2a	Protein PRRC2A	Q7TSC1	S1145	EGVLGQVPLAPPQPGAPPSPAPAR	S19	1.025	0.887
Prrc2a	Protein PRRC2A	Q7TSC1	S1217	LISGPLSPMSR	S7	1.137	0.312
Prrc2a	Protein PRRC2A	Q7TSC1	S1687	RPGGPSPLNAVPGESASGSEPSEPPR	S6	0.918	0.140
Prrc2a	Protein PRRC2A	Q7TSC1	S2114	IDLYQQASPPDALR	S8	1.077	0.748
Prrc2a	Protein PRRC2A	Q7TSC1	S761	SDSGGSSSEPFER	S3	1.022	0.701
Prrc2a	Protein PRRC2A	Q7TSC1	S761	ERSDSGGSSSEPFER	S5	1.065	0.484
Prrc2a	Protein PRRC2A	Q7TSC1	T609	EGPEPPEEVPPPTTPPAPK	T14	1.066	0.133
Prrc2a	Protein PRRC2A	Q7TSC1	T782	ERGTPPVDPK	T4	0.966	0.609
Prrc2a	Protein PRRC2A	Q7TSC1	S1083,T/S/Y	TASETRSEGSEYEEIPKR	S3,T/S/Y	1.076	0.168
Prrc2a	Protein PRRC2A	Q7TSC1	S1087,S1090	TASETRSEGSEYEEIPK	S7,S10	0.962	0.267
Prrc2a	Protein PRRC2A	Q7TSC1	S1104,T/S	QRGSETGSETHESDLAPSDK	S4,S/T	1.073	0.462
Prrc2a	Protein PRRC2A	Q7TSC1	S761,S764	ERSDSGGSSSEPFER	S5,S8	1.102	0.295
Prrc2a	Protein PRRC2A	Q7TSC1	Ambiguous	TASETRSEGSEYEEIPKR	T/S/Y	1.074	0.202
Prrc2a	Protein PRRC2A	Q7TSC1	Ambiguous	GTSPGSELPPPK	T/S	1.020	0.389
Prrc2a	Protein PRRC2A	Q7TSC1	Ambiguous	GRTASETRSEGSEYEEIPK	T/S/Y	0.969	0.200
Prrc2b	Protein PRRC2B	F8WHT3	S1151	IASETHSEGSEYEELPK	S7	0.860	0.425
Prrc2b	Protein PRRC2B	F8WHT3; Q7TPM1	S1493;S806	SPDEALPGGLGSHSPYALER	S14	1.013	0.875
Prrc2b	Protein PRRC2B	F8WHT3; Q7TPM1	S387;S387	LKFSDDEDEEDVVK	S4	0.918	0.395
Prrc2b	Protein PRRC2B	F8WHT3; Q7TPM1	S387;S387	LKFSDDEDEEDVVKDGR	S4	1.036	0.597
Prrc2b	Protein PRRC2B	F8WHT3; Q7TPM1	S415;S415	ALSLSSADSTDAKR	S3	1.181	0.029
Prrc2b	Protein PRRC2B	F8WHT3; Q7TPM1	S415;S415	ALSLSSADSTDAK	S3	1.056	0.449
Prrc2b	Protein PRRC2B	F8WHT3; Q7TPM1	S479;S479	QHSAEDKEDKPPPR	S3	1.149	0.006
Prrc2b	Protein PRRC2B	F8WHT3; Q7TPM1	S555;S555	EVPRSPGIEK	S5	1.006	0.878
Prrc2b	Protein PRRC2B	F8WHT3; Q7TPM1	S621;S621	ESGSPAQEFSK	S4	1.096	0.121
Prrc2b	Protein PRRC2B	Q7TPM1	S1469	QNVPTGGSAPSPQAYR	S11	1.014	0.824
Prrc2c	Protein PRRC2C	Q3TLH4	S1215	QREESETRSESSDFEVVPK	S5	1.026	0.582
Prrc2c	Protein PRRC2C	Q3TLH4	S1517	SFSSQRPGVDR	S4	1.004	0.951
Prrc2c	Protein PRRC2C	Q3TLH4	S189	SAGSPSSDQDEKQLGQDESTAITSEQNDILK	S4	0.988	0.889
Prrc2c	Protein PRRC2C	Q3TLH4	S189	SAGSPSSDQDEK	S4	1.104	0.377
Prrc2c	Protein PRRC2C	Q3TLH4	S853	SLEDVKPR	S1	0.984	0.851
Prrc2c	Protein PRRC2C	Q3TLH4	T2633	STTPTSSPFR	T2	0.965	0.350
Prrc2c	Protein PRRC2C	Q3TLH4	T2645	ATSTSPNSQSSK	T4	1.088	0.457
Prrc2c	Protein PRRC2C	Q3TLH4	Ambiguous	SESSDFEVVPK	S	0.960	0.716
Prrc2c	Protein PRRC2C	Q3TLH4	Ambiguous	ESSSEDQTAKTPESTENRK	T/S	1.183	0.441
Prrc2c	Protein PRRC2C	Q3TLH4	Ambiguous	QDSNRSEKETTQVVQEAEPESGAQPRPGYFK	S/T/Y	1.126	0.061
Prrc2c	Protein PRRC2C	Q3TLH4	Ambiguous	QREESETRSESSDFEVVPK	S/T	0.961	0.420
Prune2	Protein prune homolog 2	Q52KR3	S2850	SSGQESESIPEYTAEEER	S8	0.909	0.316
Prune2	Protein prune homolog 2	Q52KR3	S2850	SSGQESESIPEYTAEEEREDNR	S8	1.054	0.466
Psat1	Phosphoserine aminotransferase	Q99K85	S344	ASLYNAVTTEDVEK	S2	1.028	0.800
Psd3	PH and SEC7 domain-containing protein 3	A0A1D5RMH7	S1252	SHSSPSLNPDASPVTAK	S3	1.058	0.636
Psd3	PH and SEC7 domain-containing protein 3	A0A1D5RMH7	S619	APSERPGTSAGTLSPMPLGESGEDDVFLR	S14	1.199	0.041
Psd3	PH and SEC7 domain-containing protein 3	A0A1D5RMH7	T212	RGTVNQEVLPK	T3	0.989	0.714
Psd3	PH and SEC7 domain-containing protein 3	A0A1D5RMH7	S1252,S1261	SHSSPSLNPDASPVTAK	S3,S12	1.085	0.640
Psd4	PH and SEC7 domain-containing protein 4	Q8BLR5	S375	EDPSRSQETLISQNR	S6	1.032	0.676

Psd4	PH and SEC7 domain-containing protein 4	Q8BLR5	S458	DRSELSSLKEEETEEVPSLR	S3	1.026	0.620
Psd4	PH and SEC7 domain-containing protein 4	Q8BLR5	S706	DQPSPSAGK	S4	1.135	0.148
Psd4	PH and SEC7 domain-containing protein 4	Q8BLR5	S427,S	GSGPQPSPVSSQDSSPR	S7,S	1.043	0.111
Psd4	PH and SEC7 domain-containing protein 4	Q8BLR5	S968,S971	SHSSPSLHQEEAPTTAK	S3,S6	0.927	0.608
Psd4	PH and SEC7 domain-containing protein 4	Q8BLR5	Ambiguous	SHSSPSLHQEEAPTTAK	S/T	1.002	0.984
Psd4	PH and SEC7 domain-containing protein 4	Q8BLR5	Ambiguous	GSGPQPSPVSSQDSSPR	S	1.149	0.125
Psd4	PH and SEC7 domain-containing protein 4	Q8BLR5	Ambiguous	QENELDLGSDTADHGGCSTPPFPVPSYK	T/S/Y	0.926	0.662
Psen1	Presenilin-1	P49769	S337	ETQDSGSGNDDGGFSEEWEAQR	S15	1.071	0.618
Psen1	Presenilin-1	P49769	S367	AAVQELSGSILTSEDPEER	S9	0.926	0.177
Psen1	Presenilin-1	P49769	S367,S371	AAVQELSGSILTSEDPEER	S9,S13	1.054	0.755
Psen1	Presenilin-1	P49769	Ambiguous	LDNPEPISNGRPQSNSR	S	0.992	0.815
Psen2	Presenilin	Q3U4P5	S25	TSLMSAESPTSR	S8	1.002	0.963
Psen2	Presenilin	Q3U4P5	S30	SCQEGRPGPEDGESTAQWR	S1	1.086	0.076
Psen2	Presenilin	Q3U4P5	Ambiguous	TSLMSAESPTSR	T/S	1.020	0.559
Psip1	PC4 and SFRS1-interacting protein	Q99JF8	S106	QSNASSDVEVEEKETNVSKEDTDQEEK	S6	1.018	0.744
Psip1	PC4 and SFRS1-interacting protein	Q99JF8	S106	QSNASSDVEVEEKETNVSK	S6	1.004	0.958
Psip1	PC4 and SFRS1-interacting protein	Q99JF8	S106	QSNASSDVEVEEK	S6	0.980	0.740
Psip1	PC4 and SFRS1-interacting protein	Q99JF8	S129	EDTDQEEKASNEDVTK	S10	1.013	0.855
Psip1	PC4 and SFRS1-interacting protein	Q99JF8	S129	ETNVSKEDTDQEEKASNEDVTK	S16	0.974	0.642
Psip1	PC4 and SFRS1-interacting protein	Q99JF8	S205	QPCPSDGDMVIDEDKSK	S5	1.086	0.068
Psip1	PC4 and SFRS1-interacting protein	Q99JF8	S205	QPCPSDGDMVIDEDKSK	S5	1.073	0.435
Psip1	PC4 and SFRS1-interacting protein	Q99JF8	T122	ETNVSKEDTDQEEK	T9	1.040	0.763
Psip1	PC4 and SFRS1-interacting protein	Q99JF8	S106,T122	QSNASSDVEVEEKETNVSKEDTDQEEK	S6,T22	1.020	0.589
Psip1	PC4 and SFRS1-interacting protein	Q99JF8	T122,S129	ETNVSKEDTDQEEKASNEDVTK	T9,S16	1.030	0.769
Psip1	PC4 and SFRS1-interacting protein	Q99JF8	T271,S/T	NLAKPGVTSTSDSEDEDDQEGEK	T10,S/T	1.024	0.698
Psip1	PC4 and SFRS1-interacting protein	Q99JF8	Ambiguous	AVDITTPK	T	0.956	0.602
Psma1	Proteasome subunit alpha type-1	Q9R1P4	S247	AQPSQAAEEPAEKADEPMEH	S4	1.082	0.523
Psma1	Proteasome subunit alpha type-1	Q9R1P4	S247	AQPSQAAEEPAEK	S4	1.178	0.075
Psma3	Proteasome subunit alpha type-3	O70435	S250	YAKESLKEEDESDDDNM	S12	0.767	0.295
Psma3	Proteasome subunit alpha type-3	O70435	S250	ESLKEEDESDDDNM	S9	0.998	0.944
Psma3	Proteasome subunit alpha type-3	O70435	S250	ESLKEEDESDDDNM	S9	0.995	0.825
Psma5	Proteasome subunit alpha type-5	Q9Z2U1	S56	ITSPLMEPSSIEK	S3	1.071	0.475
Psma5	Proteasome subunit alpha type-5	Q9Z2U1	S56	ITSPLMEPSSIEK	S3	0.997	0.951
Psma6	Proteasome subunit alpha type-6	Q9QUM9	Ambiguous	QTESTSFLEK	S/T	1.055	0.729
Psmb1	Proteasome subunit beta type-1	O09061	T194	NMQNVEHVPLTLDR	T11	1.010	0.935
Psmb10	Proteasome subunit beta type-10	O35955	S229	ALSTPTEPVQR	S3	1.100	0.105
Psmb10	Proteasome subunit beta type-10	O35955	T248	FAPGTTPVLTR	T6	0.786	0.154
Psmb2	Proteasome subunit beta type-2	Q9R1P3	S76	NGYELSPTAAANFTR	S6	1.121	0.454
Psmb6	Proteasome subunit beta type-6	Q60692	T22	AGSAPAFGPEALTPDWENR	T13	1.214	0.577
Psmc1	26S proteasome regulatory subunit 4	P62192	T53	LPLVTPHTQCR	T5	1.088	0.010
Psmc3	26S proteasome regulatory subunit 6A	B7ZCF1	S379	KMNVSPDVNYEELAR	S5	1.045	0.613
Psmd1	26S proteasome non-ATPase regulatory subunit 1	Q3TXS7	T273	TVGTPIASVPGSTNTGTVPGSEKDSDPMETEEK	T4	0.997	0.981
Psmd1	26S proteasome non-ATPase regulatory subunit 1	Q3TXS7	T311,S315	TASAVAGKTPDASPEPK	T9,S13	1.026	0.750
Psmd10	26S proteasome non-ATPase regulatory subunit 10	Q9Z2X2	S230	LAESEEASM	S8	1.029	0.647
Psmd11	26S proteasome non-ATPase regulatory subunit 11	Q8BG32	S14	AQSLLSTDR	S3	1.644	0.001
Psmd2	26S proteasome non-ATPase regulatory subunit 2	Q8VDM4	S363	FGGSGSQVDSAR	S6	1.016	0.414
Psmd2	26S proteasome non-ATPase regulatory subunit 2	Q8VDM4	T20	TPVQSQQPSATTPSGADEK	T12	1.037	0.129
Psmd2	26S proteasome non-ATPase regulatory subunit 2	Q8VDM4	T20	DKTPVQSQQPSATTPSGADEK	T14	1.035	0.499
Psmd3	26S proteasome non-ATPase regulatory subunit 3	P14685	S45	EEAAAGSGSTGEGDGK	S9	1.005	0.949
Psmd4	26S proteasome non-ATPase regulatory subunit 4 (Fragment)	F7AI87	T65	AAAASAAEAGIATPGTEGERDSDDALLK	T13	1.037	0.441
Psmd4	26S proteasome non-ATPase regulatory subunit 4	O35226	T250	AAAASAAEAGIATPGTEDSDDALLK	T13	1.096	0.083
Psmd4	26S proteasome non-ATPase regulatory subunit 4	O35226	T250	RAAAASAAEAGIATPGTEDSDDALLK	T14	1.106	0.108
Psmd4	26S proteasome non-ATPase regulatory subunit 4	O35226	T250,T/S	RAAAASAAEAGIATPGTEDSDDALLK	T14,T/S	0.773	0.044
Psmd4	26S proteasome non-ATPase regulatory subunit 4 (Fragment)	F7AI87	Ambiguous	AAAASAAEAGIATPGTEGERDSDDALLK	T/S	1.005	0.965
Psmd9	26S proteasome non-ATPase regulatory subunit 9	Q9CR00	S128	LASNSPVLPQAFAR	S5	0.954	0.480
Psme3	Proteasome activator complex subunit 3	P61290	S247	SSNAETLY	S1	0.963	0.775
Psmf1	Proteasome inhibitor PI31 subunit	Q8BHL8	S153	ANSPPREFPPATAR	S3	0.996	0.913
Pspc1	Paraspeckle component 1	Q8R326	T487	TGSETPQAPMSGVGPVSGGPGGFGR	T5	0.881	0.167
Pstpip1	Proline-serine-threonine phosphatase-interacting protein 1	A0A0R4J0P5	T334	TTPSAPAASTETLTPTPER	T14	1.018	0.783
Pstpip1	Proline-serine-threonine phosphatase-interacting protein 1	A0A0R4J0P5	Ambiguous	EVTPLIGSPSVQPSCGVIK	S/T	1.126	0.656
Ptafr	Platelet-activating factor receptor	Q62035	Ambiguous	ATSDTCTEVIVPANQTPIVSLKN	S/T	0.771	0.293
Ptbp1	MCG13402, isoform CRA_c	Q922I7	Ambiguous	ELKTDSSPNQAR	S/T	0.904	0.400
Ptcd3	Pentatricopeptide repeat domain-containing protein 3, mitochondrial	Q14C51	Ambiguous	EALGNLTELNSSDGESSSDSDSDDK	S/T	1.063	0.886
Ptdss2	Phosphatidylserine synthase 2	Q9Z1X2	S12	VAGGSGSESPLLK	S5	0.965	0.663
Pten	ylinositol 3,4,5-trisphosphate 3-phosphatase and dual-specificity protein phospha	O08586	S385	YSDTTDSDPENEPFDEDQHSQITKV	S7	1.021	0.866
Ptger2	Prostaglandin E2 receptor EP2 subtype	Q62053	S240	CGLSGSSLR	S6	1.045	0.337
Ptger2	Prostaglandin E2 receptor EP2 subtype	Q62053	Ambiguous	CGLSGSSLRGPGSR	S	0.974	0.702
Ptges3	Prostaglandin E synthase 3	Q9R0Q7	S113	DWEDDSDEDMSNFDR	S6	1.047	0.476
Ptges3	Prostaglandin E synthase 3	Q9R0Q7	S113	DWEDDSDEDMSNFDR	S6	1.069	0.524
Ptges3l	Putative protein PTGES3L	Q9D9A7	S131	DWEGDDEVELAQVEHYAEDDSDS	S23	1.064	0.436
Ptges3l	Putative protein PTGES3L	A2A4P5	Ambiguous	RPPPAMDDLDDDSDS	S	0.918	0.044
Ptk2	Focal adhesion kinase 1	P34152	S948	LQPQEISPPPTANLDR	S7	1.010	0.847
Ptk2	Focal adhesion kinase 1	P34152	Ambiguous	YMEDSTYYK	S/T/Y	0.972	0.400
Ptk2b	Protein-tyrosine kinase 2-beta	Q9QVP9	Y579	YIEDEDYYK	Y7	0.911	0.033
Ptk2b	Protein-tyrosine kinase 2-beta	Q9QVP9	Y580	YIEDEDYYKASVTR	Y8	0.973	0.601
Ptk2b	Protein-tyrosine kinase 2-beta	Q9QVP9	Y849	EAGYTEFTGPPQKPPR	Y4	1.116	0.178
Ptpdc1	Protein tyrosine phosphatase domain-containing protein 1	Q6NZK8	S394	QLSYSDSDLKR	S5	0.946	0.099
Ptpn1	Tyrosine-protein phosphatase non-receptor type 1	P35821	S392	MVGGGLQSAQASVPTEEELSSTEEEHK	S20	1.040	0.648
Ptpn1	Tyrosine-protein phosphatase non-receptor type 1	P35821	S50	DVSPFDHSR	S3	1.044	0.387
Ptpn1	Tyrosine-protein phosphatase non-receptor type 1	P35821	S50	YRDVSPFDHSR	S5	0.890	0.196
Ptpn1	Tyrosine-protein phosphatase non-receptor type 1	P35821	Ambiguous	AQSSAMHSVSSMSPDTEVR	S/T	1.171	0.321
Ptpn11	Tyrosine-protein phosphatase non-receptor type 11	P35235	Y584	VYENVGLMQQQR	Y2	0.952	0.388
Ptpn11	Tyrosine-protein phosphatase non-receptor type 11	P35235	Y584	VYENVGLMQQQR	Y2	1.000	0.990
Ptpn11	Tyrosine-protein phosphatase non-receptor type 11	P35235	Y584	EDSARVYENVGLMQQQR	Y7	0.896	0.268
Ptpn11	Tyrosine-protein phosphatase non-receptor type 11	P35235	Ambiguous	IQNTGDYYDLYGGEK	Y/T	0.895	0.350
Ptpn12	Tyrosine-protein phosphatase non-receptor type 12	P35831	S331	QDSPPPKPPR	S3	1.060	0.466
Ptpn12	Tyrosine-protein phosphatase non-receptor type 12	P35831	S588	TVSSTPNSTAEEEAHDLTEHHNSSPLLK	S24	1.112	0.176
Ptpn12	Tyrosine-protein phosphatase non-receptor type 12	P35831	S673	DADVSEESPPPLPER	S8	0.988	0.868
Ptpn12	Tyrosine-protein phosphatase non-receptor type 12	P35831	S748	SPAEVTDIGFGNR	S1	0.992	0.965
Ptpn12	Tyrosine-protein phosphatase non-receptor type 12	P35831	T519	AAESSEESQSNSHTPPRPDCLPLDK	T14	1.062	0.801
Ptpn12	Tyrosine-protein phosphatase non-receptor type 12	P35831	T630	TSISTASATVSPASSAESACTR	T9	1.010	0.853
Ptpn12	Tyrosine-protein phosphatase non-receptor type 12	P35831	S670,S673	QEVAGTPHSGAEKDADVSEESPPPLPER	S18,S21	1.049	0.530
Ptpn12	Tyrosine-protein phosphatase non-receptor type 12	P35831	Ambiguous	QEVAGTPHSGAEKDADVSEESPPPLPER	S/T	1.013	0.787
Ptpn12	Tyrosine-protein phosphatase non-receptor type 12	P35831	Ambiguous	HDAGGIHTEASADSPPAFSDK	S/T	0.961	0.357
Ptpn12	Tyrosine-protein phosphatase non-receptor type 12	P35831	Ambiguous	APLSFTNPLHSDDSDSDGGSSDGAVTR	S/T	1.067	0.816
Ptpn14	Tyrosine-protein phosphatase non-receptor type 14	Q62130	S314	ICTEQSNSPPPIR	S8	1.103	0.130
Ptpn18	Tyrosine-protein phosphatase	Q3V441	S33	APTSTDTPIYSQVAPR	S11	0.974	0.631
Ptpn21	Tyrosine-protein phosphatase non-receptor type	G5E8J4	S590	HLYISSSNPDLITR	S7	1.013	0.798
Ptpn21	Tyrosine-protein phosphatase non-receptor type	G5E8J4	S658	TVSASAADVAPR	S3	1.172	0.034
Ptpn21	Tyrosine-protein phosphatase non-receptor type	G5E8J4	S731	EQAISAVSEPR	S8	1.066	0.235
Ptpn21	Tyrosine-protein phosphatase non-receptor type	G5E8J4	S799	RDGLLTPSMSESDLTTSGR	S10	0.906	0.277
Ptpn21	Tyrosine-protein phosphatase non-receptor type	G5E8J4	S799	DGLLTPSMSESDLTTSGR	S9	1.437	0.046
Ptpn21	Tyrosine-protein phosphatase non-receptor type	G5E8J4	S799	DGLLTPSMSESDLTTSGR	S9	0.933	0.387
Ptpn21	Tyrosine-protein phosphatase non-receptor type	G5E8J4	Ambiguous	TQVYRPPPPYPYPRPANSTPDLSR	S/T/Y	0.965	0.143
Ptpn22	Tyrosine-protein phosphatase non-receptor type 22	P29352	S355	QGAEAESTGSSLGLR	S11	1.022	0.846
Ptpn22	Tyrosine-protein phosphatase non-receptor type 22	P29352	S405	ASPVVGEPLQK	S2	1.062	0.589
Ptpn22	Tyrosine-protein phosphatase non-receptor type 22	P29352	S452	STPFELIQQR	S1	0.922	0.432
Ptpn22	Tyrosine-protein phosphatase non-receptor type 22	P29352	S497	VAHVSSEELNYSLPGACDASCVPR	S5	1.069	0.466
Ptpn22	Tyrosine-protein phosphatase non-receptor type 22	P29352	S687	HQDGSPPPPLPER	S5	1.038	0.394
Ptpn23	Tyrosine-protein phosphatase non-receptor type 23	Q6PB44	T744	REEGEAVEAGDTPEELR	T12	0.971	0.535
Ptpn3	Tyrosine-protein phosphatase non-receptor type 3	A2ALK8	Ambiguous	SSSSVSPSSNAPGSCSPDGVDQR	S	1.023	0.890
Ptpn6	Tyrosine-protein phosphatase non-receptor type 6	P29351	S10	DLSGPDAETLLK	S3	1.060	0.499
Ptpn6	Tyrosine-protein phosphatase non-receptor type 6	P29351	S534	GQESEYGNITYPPAVR	S4	0.969	0.435
Ptpn6	Tyrosine-protein phosphatase non-receptor type 6	P29351	S57	IQNSGDFYDLYGGEK	S4	1.085	0.683
Ptpn7	Tyrosine-protein phosphatase non-receptor type 7	Q8BUM3	S143	AQSQEDSDYINANYIR	S3	1.062	0.533
Ptpra	Receptor-type tyrosine-protein phosphatase alpha	P18052	S202	TEDVEPQSVPLLARSPSTNR	S15	1.066	0.053
Ptpra	Receptor-type tyrosine-protein phosphatase alpha	P18052	T188	LSNGRTEDVEPQSVPLLAR	T6	0.893	0.363
Ptpra	Receptor-type tyrosine-protein phosphatase alpha	P18052	S176,S180	QAGSHSNSFR	S4,S8	1.061	0.523
Ptpra	Receptor-type tyrosine-protein phosphatase alpha	P18052	S202,S204	TEDVEPQSVPLLARSPSTNR	S15,S17	1.079	0.290
Ptprc	Receptor-type tyrosine-protein phosphatase C	P06800	S1229	TNSQDKIEFHNEVDGGK	S3	1.048	0.172
Ptprc	Receptor-type tyrosine-protein phosphatase C	P06800	S1229	KTNSQDKIEFHNEVDGGK	S4	1.013	0.864
Ptprc	Receptor-type tyrosine-protein phosphatase C	P06800	S1286	GVGTPEPTNSAEEPEHAANGSASPAPTQSS	S23	1.051	0.223
Ptprc	Receptor-type tyrosine-protein phosphatase C	P06800	S599	SSNLDEQQELVERDDEK	S2	1.044	0.487
Ptprc	Receptor-type tyrosine-protein phosphatase C	P06800	S964	NSNVVPYDFNR	S2	1.001	0.994
Ptprc	Receptor-type tyrosine-protein phosphatase C	P06800	S964	NRNSNVVPYDFNR	S4	1.019	0.520
Ptprc	Receptor-type tyrosine-protein phosphatase C	P06800	S964	KNRNSNVVPYDFNR	S5	1.023	0.643
Ptprc	Receptor-type tyrosine-protein phosphatase C	P06800	Y672	YVDILPYDYNR	Y1	1.151	0.580
Ptprc	Receptor-type tyrosine-protein phosphatase C	P06800	T1267,S1284	GVGTPEPTNSAEEPEHAANGSASPAPTQSS	T4,S21	1.046	0.444
Ptpro	Receptor-type tyrosine-protein phosphatase O	E9Q612	S875	ECGAGTFVNFASLER	S12	1.216	0.090
Ptrh2	Peptidyl-tRNA hydrolase 2, mitochondrial	Q8R2Y8	S59	DTETGTEASILGESGEYK	S9	1.195	0.379
Pts	6-pyruvoyl tetrahydrobiopterin synthase	Q9R1Z7	S27	LHSPSLSDEENLR	S3	0.957	0.216
Pttg1	Securin	Q9CQJ7	S100	TQSSVPAPDDAYPEIEK	S4	1.640	0.003
Pum1	Pumilio homolog 1	Q80U78	S124	SMDELNHDFQALALEGR	S1	0.993	0.921
Pum1	Pumilio homolog 1	Q80U78	S710	RDSLTGSSDLYK	S3	1.061	0.253
Pum1	Pumilio homolog 1	Q80U78	S710	RDSLTGSSDLYKR	S3	1.011	0.890
Pum1	Pumilio homolog 1	Q80U78	T112	HRWPTGDNIHAEHQVR	T5	0.905	0.615
Pum2	Pumilio homolog 2	Q80U58	S136	ASPFEEDQNR	S2	0.957	0.610
Pum2	Pumilio homolog 2	Q80U58	S136	GKASPFEEDQNR	S4	0.988	0.717

Pum2	Pumilio homolog 2	Q80U58	S181	QASPTEVVER	S3	1.008	0.762
Pum2	Pumilio homolog 2	Q80U58	S587	RESLSTSSDLYK	S3	1.229	0.061
Pum2	Pumilio homolog 2	Q80U58	S82	SGQSFHGNSEVNAILSPR	S16	1.028	0.282
Pum2	Pumilio homolog 2	Q80U58	S177,S181	TPGSRQASPTEVVER	S4,S8	0.962	0.301
Pum2	Pumilio homolog 2	Q80U58	S181,T/S	DFNRTPGSRQASPTEVVER	S12,S/T	1.045	0.590
Pura	Transcriptional activator protein Pur-alpha	P42669	S255	NSITVPYK	S2	0.858	0.075
Purb	Transcriptional activator protein Pur-beta	O35295	S316	GGGSGGGDESEGEEVDED	S10	1.051	0.468
Purb	Transcriptional activator protein Pur-beta	O35295	S316	RGGGSGGGDESEGEEVDED	S11	1.124	0.350
Pus1	tRNA pseudouridine synthase	H7BX59	T440	VPSSLEGSEGDGDTD	T14	1.077	0.084
Pus1	tRNA pseudouridine synthase	H7BX59	S434,T440	VPSSLEGSEGDGDTD	S8,T14	1.040	0.520
Pus7	Pseudouridylate synthase 7 homolog	Q91VU7	S40	VSEGCLTSSQDGVENDGLHR	S9	0.995	0.944
Pus7l	Pseudouridylate synthase 7 homolog-like protein	Q8CE46	Ambiguous	NSEGAADLPGCSDGDRSHQSDSEKENSVNSVTSK	S/T	0.962	0.789
Pvr	Poliovirus receptor	Q8K094	S393	ENVQYSSVNGDCR	S7	0.925	0.514
Pwp1	Periodic tryptophan protein 1 homolog	Q99LL5	S49	LQEEGGSEEEEAGNPSEDGMQSGPTQAPPR	S7	0.883	0.428
Pwp1	Periodic tryptophan protein 1 homolog	Q99LL5	S49	LQEEGGSEEEEAGNPSEDGMQSGPTQAPPR	S7	1.004	0.899
Pwp1	Periodic tryptophan protein 1 homolog	Q99LL5	S49	GKLQEEGGSEEEEAGNPSEDGMQSGPTQAPPR	S9	1.027	0.558
Pwp1	Periodic tryptophan protein 1 homolog	Q99LL5	S49	GKLQEEGGSEEEEAGNPSEDGMQSGPTQAPPR	S9	1.174	0.033
Pwp1	Periodic tryptophan protein 1 homolog	Q99LL5	S493	GLSVSGPCGSRSPQQTPMES	S12	1.021	0.642
Pwp1	Periodic tryptophan protein 1 homolog	Q99LL5	S49,S58	GKLQEEGGSEEEEAGNPSEDGMQSGPTQAPPR	S9,S18	1.060	0.248
Pwp1	Periodic tryptophan protein 1 homolog	Q99LL5	S49,S58	GKLQEEGGSEEEEAGNPSEDGMQSGPTQAPPR	S9,S18	1.153	0.153
Pwwp2b	PWWP domain-containing 2B	E9Q9M8	S190	STVSPQEASPSPLNTPKPR	S4	0.948	0.343
Pwwp2b	PWWP domain-containing 2B	E9Q9M8	S210	LGSGPDSEHR	S3	1.128	0.449
Pwwp2b	PWWP domain-containing 2B	E9Q9M8	S210	LGSGPDSEHRKPEEPEDSAVIATAAPR	S3	1.037	0.618
Pwwp2b	PWWP domain-containing 2B	E9Q9M8	S450	SSGSEVTSPDTGDLSSGDSASVPSSSADTR	S8	1.040	0.724
Pwwp2b	PWWP domain-containing 2B	E9Q9M8	S197,T201	STVSPQEASPSPLNTPKPR	S11,T15	0.934	0.320
Pxn	Paxillin	F8VQ28	S106	NSSASNTQDGVGSLCSR	S13	1.006	0.961
Pxn	Paxillin	F8VQ28	S126	SAEPSPTVMSSSLGSNLSELDR	S1	0.892	0.512
Pxn	Paxillin	F8VQ28	S137	SAEPSPTVMSSSLGSNLSELDR	S12	1.099	0.260
Pxn	Paxillin	F8VQ28	S258	ISASSATR	S2	0.960	0.054
Pxn	Paxillin	F8VQ28	S303	QSSPEGQDEGGFMAQGK	S3	0.973	0.396
Pxn	Paxillin	F8VQ28	S303	QSSPEGQDEGGFMAQGK	S3	0.997	0.951
Pxn	Paxillin	F8VQ28	S83	YAHQQPPSPLPVYSSSAK	S8	1.103	0.036
Pxn	Paxillin	F8VQ28	Y118	AGEEEHVYSFPNK	Y8	0.895	0.036
Pycard	Apoptosis-associated speck-like protein containing a CARD	Q9EPB4	S193	EIHPYLVMDLEQS	S13	1.038	0.540
Pycard	Apoptosis-associated speck-like protein containing a CARD	Q9EPB4	S193	EIHPYLVMDLEQS	S13	0.956	0.742
Pygl	Glycogen phosphorylase, liver form	Q9ET01; Q9QZS3	S15;S295	RQLSLR	S4	0.984	0.757
Pygo2	Pygopus 2	Q80V76	T301	GGGTPDANSLAPPGK	T4	0.933	0.312
Pyhin1	Pyrin and HIN domain-containing protein 1	Q8BV49	S128	STPDTQVAQLSLPTASR	S1	1.169	0.321
Pyhin1	Pyrin and HIN domain-containing protein 1	Q8BV49	S152	NQAIQISPTIASSSGQTSSR	S7	0.973	0.510
Qser1	Glutamine and serine-rich 1	A0A338P6K9	S95	GQTLQSSIPSPDPK	S10	0.977	0.702
Qser1	Glutamine and serine-rich 1	A0A338P6K9	Ambiguous	VPAAASSPSCSGSSPSLK	S	0.750	0.442
R3hcc1l	Coiled-coil domain-containing protein R3HCC1L	Q8BJM3	S339	ECESDSSADELCVK	S6	1.025	0.758
R3hdm1	R3H domain-containing 1	E9Q9Q2	S361	YSEPRPWSSTDSDSSLR	S8	0.938	0.123
R3hdm1	R3H domain-containing 1	E9Q9Q2; Q80TM6	Ambiguous	SKSIEEREEEYQR	S/Y	1.315	0.051
R3hdm1	R3H domain-containing 1	E9Q9Q2	Ambiguous	AASTDLGAGEAVVGK	T/S	0.976	0.822
R3hdm2	R3H domain-containing protein 2	Q80TM6	S923	SASTDLGTADVVLGR	S3	0.897	0.260
Rab10	Ras-related protein Rab-10	P61027; P62821; P63011; Q91V41; Q91ZR1; P61028; A2CG35; P55258; P35279; P35276; Q6PHN9	T65;T67;T78;T67;T64;T64;T145;T64;T69;T78;T64	LQIWDTAGQER	T6	0.967	0.918
Rab11fip1	Rab11 family-interacting protein 1	E9Q8L9	S204	DSASDTASAIVPSVTPSVDSDDESFSK	S20	1.030	0.698
Rab11fip1	Rab11 family-interacting protein 1	E9Q8L9	S282	TSSTDQQPNQSNFSLPK	S3	0.921	0.493
Rab11fip1	Rab11 family-interacting protein 1	E9Q8L9	S346	ESSPSNSPSPQGFR	S9	1.103	0.086
Rab11fip1	Rab11 family-interacting protein 1	E9Q8L9	S358	HLFSSTENLAAR	S5	1.051	0.316
Rab11fip1	Rab11 family-interacting protein 1	E9Q8L9	S541	LEVSPEAQPK	S4	0.925	0.159
Rab11fip1	Rab11 family-interacting protein 1	E9Q8L9	S780	AGSPESPHTEGTGQEPVTAEAQPNISSEGK	S3	1.046	0.626
Rab11fip1	Rab11 family-interacting protein 1	E9Q8L9	S340,S342	ESSPSNSPSPQGFR	S3,S5	1.056	0.358
Rab11fip1	Rab11 family-interacting protein 1	E9Q8L9	S346,S	ESSPSNSPSPQGFRR	S9,S	0.992	0.927
Rab11fip5	Rab11 family-interacting protein 5	A0A0N4SW73	S1218	TSLSTALSSGLER	S8	0.902	0.388
Rab11fip5	Rab11 family-interacting protein 5	A0A0N4SW73	S278	SPSHSSWLSTEGGR	S1	1.032	0.023
Rab11fip5	Rab11 family-interacting protein 5	A0A0N4SW73	S307	TYSDEASQLR	S3	1.031	0.774
Rab11fip5	Rab11 family-interacting protein 5	A0A0N4SW73	S307	RTYSDEASQLR	S4	0.980	0.521
Rab11fip5	Rab11 family-interacting protein 5	A0A0N4SW73	S382	SSEEGSRSSDDSWGR	S8	1.109	0.073
Rab11fip5	Rab11 family-interacting protein 5	A0A0N4SW73	S395	GSHGTSSSEAVPGQEELSK	S6	1.027	0.740
Rab11fip5	Rab11 family-interacting protein 5	A0A0N4SW73	S474	RGSVGEK	S3	0.953	0.462
Rab11fip5	Rab11 family-interacting protein 5	A0A0N4SW73	S626	ASLAPLASPGK	S8	1.096	0.109
Rab11fip5	Rab11 family-interacting protein 5	A0A0N4SW73	S693	RGSNIWLEPK	S3	0.992	0.941
Rab11fip5	Rab11 family-interacting protein 5	A0A0N4SW73	S725	VSVDSELDQPSTSMSDPGPFGSSGSGPSSR	S25	1.347	0.088
Rab11fip5	Rab11 family-interacting protein 5	A0A0N4SW73	T1011	VLGTSETEEGFETKSQELAK	T13	0.897	0.408
Rab11fip5	Rab11 family-interacting protein 5	A0A0N4SW73	T886	LFTPTNSQVEEEDKEEEENATGR	T3	1.139	0.351
Rab11fip5	Rab11 family-interacting protein 5	A0A0N4SW73	S280,S283	SPSHSSWLSTEGGR	S3,S6	1.157	0.072
Rab11fip5	Rab11 family-interacting protein 5	A0A0N4SW73	T886,T888	LFTPTNSQVEEEDKEEEENATGR	T3,T5	1.033	0.784
Rab12	Ras-related protein Rab-12	A2CG35	S153	FNSITSAYYR	S3	0.979	0.858
Rab12	Ras-related protein Rab-12	A2CG35	S68	RPAGGSLGAVSPALSGGQAR	S11	1.043	0.162
Rab12	Ras-related protein Rab-12	A2CG35	S63,S68	RPAGGSLGAVSPALSGGQAR	S6,S11	1.116	0.131
Rab14	Ras-related protein Rab-14	Q91V41	T135	DVTYEEAK	T3	0.939	0.750
Rab34	Ras-related protein Rab-34	A0A140LIX7	S13	VVVGSPRPR	S5	1.096	0.404
Rab3a	Ras-related protein Rab-3A	P63011	Ambiguous	MSESLDTADPAVTGAK	S/T	1.021	0.808
Rab3gap1	Rab3 GTPase-activating protein catalytic subunit	A0A1D5RLG3	S540	TSLSDSTTSAYPGDAGK	S6	0.804	0.166
Rab3gap1	Rab3 GTPase-activating protein catalytic subunit	A0A1D5RLG3	T663	LGTSAEGAHLR	T3	1.113	0.201
Rab3gap2	Rab3 GTPase-activating protein non-catalytic subunit	E9QKE4	S449	GGFSPFGNTQGPSR	S4	1.064	0.093
Rab3gap2	Rab3 GTPase-activating protein non-catalytic subunit	E9QKE4	S572	SPRPDSFETEIK	S6	1.020	0.658
Rab3gap2	Rab3 GTPase-activating protein non-catalytic subunit	E9QKE4	S951	QDLSPELLK	S4	1.042	0.687
Rab3il1	Guanine nucleotide exchange factor for Rab-3A	Q8VDV3	S169	TLVITSTPASPNR	S10	1.061	0.226
Rab3il1	Guanine nucleotide exchange factor for Rab-3A	Q8VDV3	S180	ELHPQLLSPTK	S8	1.065	0.063
Rab40c	Ras-related protein Rab-40C	Q8VHQ4	S268	SIRPPQSPPQNCSR	S7	0.951	0.390
Rab44	Ras-related protein Rab-44	Q8CB87	S520	DQLAFEAEPPPQGLSSSPQSPAGSR	S20	1.476	0.198
Rab7b	Ras-related protein Rab-7b	Q8VEA8	S186	YQGTAENHLIDSIK	S12	1.032	0.878
Rab8a	Ras-related protein Rab-8A	P55258	S181	LEGNSPQGSSHGVK	S5	1.117	0.093
Rab8a	Ras-related protein Rab-8A	P55258	S181,S	KLEGNSPQGSSHGVK	S6,S	1.123	0.388
Rab8a	Ras-related protein Rab-8A	P55258	S181,S185	LEGNSPQGSSHGVK	S5,S9	1.067	0.130
Rab8b	Ras-related protein Rab-8B	P61028	S182	MNDSNSSGAGGPVK	S6	1.051	0.366
Rab8b	Ras-related protein Rab-8B	P61028	S182	KMNDSNSSGAGGPVK	S7	1.064	0.440
Rab8b	Ras-related protein Rab-8B	P61028	T164	SSTNVEEAFFTLAR	T11	0.646	0.104
Rab8b	Ras-related protein Rab-8B	P61028; P55258	T72;T72	FRTITTAYYR	T3	1.051	0.673
Rab8b	Ras-related protein Rab-8B	P61028; P55258	Y5;Y5	TYDYLFK	Y2	0.972	0.629
Rab8b	Ras-related protein Rab-8B	P61028	S180,S183	MNDSNSSGAGGPVK	S4,S7	1.064	0.309
Rabep1	Rab GTPase-binding effector protein 1	O35551	S407	RAQSTDSLGTSSSLQSK	S4	0.957	0.511
Rabep1	Rab GTPase-binding effector protein 1	O35551	S410	AQSTDSLGTSSSLQSK	S6	0.956	0.507
Rabep1	Rab GTPase-binding effector protein 1	O35551	S407,S410	RAQSTDSLGTSSSLQSK	S4,S7	1.039	0.626
Rabep1	Rab GTPase-binding effector protein 1	O35551	S407,S410	AQSTDSLGTSSSLQSK	S3,S6	1.058	0.306
Rabep2	Rab GTPase-binding effector protein 2	Q91WG2	S176,S180	QPASLHGSTELLPLSR	S4,S8	0.833	0.188
Rabgap1	Rab GTPase-activating protein 1	A2AWA9	S42	QGDETPSTNNGSDDEKTGLK	S12	1.004	0.923
Rabgap1	Rab GTPase-activating protein 1	A2AWA9	S42	QGDETPSTNNGSDDEK	S12	1.042	0.319
Rabgap1	Rab GTPase-activating protein 1	A2AWA9	T991	GVSDEDTDEEKETLK	T7	1.085	0.137
Rabgap1	Rab GTPase-activating protein 1	A2AWA9	S42,T/S	QGDETPSTNNGSDDEKTGLK	S12,T/S	1.035	0.528
Rabgap1l	Rab GTPase-activating protein 1-like	A6H6A9	S49	IASNGDEQLEK	S3	0.987	0.875
Rabgef1	Rab5 GDP/GTP exchange factor	Q9JM13	S124	APSPSINR	S3	0.971	0.422
Rabgef1	Rab5 GDP/GTP exchange factor	Q9JM13	S132	QTSIETDR	S3	1.231	0.003
Rabgef1	Rab5 GDP/GTP exchange factor	Q9JM13	S132	QTSIETDRVTK	S3	1.121	0.083
Rabl2	Rab-like protein 2A	E9Q9D5	S221	LEQKEEDTSGQEQSDTTKSPSPS	S21	1.086	0.338
Rabl6	Rab-like protein 6	Q5U3K5	S377	LFGTSPAAEVTPSPPEPAPALEAPAR	S5	1.096	0.350
Rabl6	Rab-like protein 6	Q5U3K5	S482	NISLSSEEEAEGLAGHPR	S5	0.989	0.797
Rabl6	Rab-like protein 6	Q5U3K5	S502	VAPQQCSEPETK	S7	0.978	0.650
Rabl6	Rab-like protein 6	Q5U3K5	T526	GTPPWSDGLTTDDSERPQEGK	T2	1.024	0.656
Rabl6	Rab-like protein 6	Q5U3K5	T526	APTRGTPPWSDGLTTDDSERPQEGK	T6	1.083	0.185
Rabl6	Rab-like protein 6	Q5U3K5	S482,S483	NISLSSEEEAEGLAGHPR	S5,S6	0.998	0.978
Rack1	Receptor of activated protein C kinase 1	P68040	S276	QEVISTSSK	S5	1.027	0.831
Rad17	Cell cycle checkpoint protein RAD17	Q6NXW6	S70	GRLSLEQTHGLETSR	S4	1.048	0.516
Rad21	Double-strand-break repair protein rad21 homolog	Q61550	S549	EKEKEEEEEEDEDASGGDQDQEER	S16	1.021	0.924
Rad23a	UV excision repair protein RAD23 homolog A	P54726	S123	EDKSPSEESTTTTSPESISGSVPSSGSSGR	S4	1.016	0.747
Rad50	DNA repair protein RAD50	P70388	S237	EAQLASSQEIVR	S7	1.094	0.250
Rad51ap1	RAD51-associated protein 1	A0A0R4J1G4	Ambiguous	SGSPSLPQAVGLPSEATR	S/T	1.003	0.988
Rad54l2	Helicase ARIP4	E9QKL0	S74	CTSTTSSQSEPSEQLR	S3	1.159	0.012
Raf1	RAF proto-oncogene serine/threonine-protein kinase	Q99N57	S244	YSTPHAFTFNTSSPSSEGSLSQR	S13	0.826	0.398
Raf1	RAF proto-oncogene serine/threonine-protein kinase	Q99N57	S43	RASDDGKLTDSSK	S3	1.234	0.048
Raf1	RAF proto-oncogene serine/threonine-protein kinase	Q99N57; P04627	S621;S580	SASEPSLHR	S3	0.988	0.845
Raf1	RAF proto-oncogene serine/threonine-protein kinase	Q99N57	S301,S/T	SHSESASPSALSSSPNNLSPTGWSQPK	S19,S/T	0.909	0.707
Raf1	RAF proto-oncogene serine/threonine-protein kinase	Q99N57	Ambiguous	AAHTEDINACTLTTSPR	T/S	1.029	0.368
Rai1	Retinoic acid-induced protein 1	Q61818	S1090	AASSPSNPAALPVASDSSPMGSK	S3	1.016	0.783
Rai1	Retinoic acid-induced protein 1	Q61818	S1332	VAGAPPGTPRSPALPER	S11	1.135	0.264
Rai1	Retinoic acid-induced protein 1	Q61818	S1093,S	AASSPSNPAALPVASDSSPMGSK	S6,S	1.250	0.194
Rai14	Ankycorbin	Q9EP71; A0A2I3BPY7	S317;S288	AEISSIQENK	S4	1.083	0.373
Rai14	Ankycorbin	Q9EP71; A0A2I3BPY7	S414;S385	SSPPVEHPAGTSTTDNDVIIR	S2	1.020	0.850
Rai14	Ankycorbin	Q9EP71; A0A2I3BPY7	S293,T297,T268	SITSTPLSGK	S1,T5	1.129	0.118
Rai14	Ankycorbin	Q9EP71; A0A2I3BPY7	Ambiguous	ASDIPSSDAKSSPPVEHPAGTSTTDNDVIIR	S/T	0.961	0.849
Rai14	Ankycorbin	A0A2I3BPY7	Ambiguous	QLSDVSSPR	S	1.126	0.015
Rala	Ras-related protein Ral-A	P63321	S11	GQNSLALHK	S4	1.030	0.784
Ralbp1	RalA-binding protein 1	Q62172	S29	TPSSEEISPTK	S3	1.017	0.785

Ralbp1	RalA-binding protein 1	Q62172	S29	TPSSEEISPTKFPGLYR	S3	0.957	0.323
Ralbp1	RalA-binding protein 1	Q62172	S62	TGEPSPPHDVLHEPPDTVSDDDKDHGK	S19	1.014	0.774
Ralbp1	RalA-binding protein 1	Q62172	S48,S62	TGEPSPPHDVLHEPPDTVSDDDKDHGKK	S5,S19	0.989	0.814
Ralbp1	RalA-binding protein 1	Q62172	S48,T/S	TGEPSPPHDVLHEPPDTVSDDDKDHGK	S5,T/S	0.921	0.574
Ralbp1	RalA-binding protein 1	Q62172	S92,S93	TEGYAAFQEDSSGDEAESPSK	S11,S12	1.065	0.263
Ralbp1	RalA-binding protein 1	Q62172	S92,S93	RTEGYAAFQEDSSGDEAESPSK	S12,S13	0.941	0.466
Ralgapa1	Ral GTPase-activating protein subunit alpha-1	A0A2I3BRX9	S1459	SQTPSPSTLSR	S5	1.054	0.063
Ralgapa1	Ral GTPase-activating protein subunit alpha-1	A0A2I3BRX9	S739	SATTTGSPGTEK	S7	1.025	0.509
Ralgapa1	Ral GTPase-activating protein subunit alpha-1	A0A2I3BRX9	S772	HFSQSEDTGNEVFGALHEEQPLPR	S3	1.004	0.958
Ralgapa1	Ral GTPase-activating protein subunit alpha-1	A0A2I3BRX9	S796	SSSTSDILEPFTVER	S3	0.973	0.194
Ralgapa1	Ral GTPase-activating protein subunit alpha-1	A0A2I3BRX9	S1315,S1316	RGSSPGSLEIPK	S3,S4	0.989	0.920
Ralgapa1	Ral GTPase-activating protein subunit alpha-1	A0A2I3BRX9	S1455,T1457	SQTPSPSTLSR	S1,T3	0.990	0.866
Ralgapa1	Ral GTPase-activating protein subunit alpha-1	A0A2I3BRX9	Ambiguous	GSSPGSLEIPK	S	0.930	0.344
Ralgapa1	Ral GTPase-activating protein subunit alpha-1	A0A2I3BRX9	Ambiguous	RGSSPGSLEIPK	S	0.889	0.238
Ralgapa2	Ral GTPase-activating protein subunit alpha-2	A0A2I3BPN8	S373	RLSNSSLCSIEEEHR	S3	0.948	0.086
Ralgapa2	Ral GTPase-activating protein subunit alpha-2	A0A2I3BPN8	S379	LSNSSLCSIEEEHR	S8	0.986	0.820
Ralgapa2	Ral GTPase-activating protein subunit alpha-2	A0A2I3BPN8	S486	TYSFTSAMSR	S3	0.907	0.502
Ralgapa2	Ral GTPase-activating protein subunit alpha-2	A0A2I3BPN8	S812	SSSTSDITER	S3	1.055	0.199
Ralgapa2	Ral GTPase-activating protein subunit alpha-2	A0A2I3BPN8	S905	SSSPAELELK	S3	1.056	0.424
Ralgapa2	Ral GTPase-activating protein subunit alpha-2	A0A2I3BPN8	T715	SATTSGAPGVEK	T3	0.992	0.906
Ralgapa2	Ral GTPase-activating protein subunit alpha-2	A0A2I3BPN8	S373,S375	RLSNSSLCSIEEEHR	S3,S5	1.020	0.806
Ralgapa2	Ral GTPase-activating protein subunit alpha-2	A0A2I3BPN8	S905,S	RSSSPAELELK	S4,S	1.043	0.646
Ralgapa2	Ral GTPase-activating protein subunit alpha-2	A0A2I3BPN8	Ambiguous	TYSFTSAMSR	S/T/Y	1.008	0.885
Ralgapa2	Ral GTPase-activating protein subunit alpha-2	A0A2I3BPN8	Ambiguous	SSSPAELELKDDLQQAHGR	S	1.088	0.183
Ralgapb	Ral GTPase-activating protein subunit beta	A2ACC6	S359	SDSAPPTPVNR	S3	1.072	0.175
Ralgapb	Ral GTPase-activating protein subunit beta	A2ACC6	S479	DNSMTAIATQASMEFR	S12	1.140	0.512
Ralgapb	Ral GTPase-activating protein subunit beta	A2ACC6	S716	SHSRTNSGISSASGGSTEPTTPDSERPAQALLR	S3	0.748	0.273
Ralgapb	Ral GTPase-activating protein subunit beta	A2ACC6	T379	LSMPQSAAVNTTPPHNR	T12	1.120	0.305
Ralgapb	Ral GTPase-activating protein subunit beta	A2ACC6	T379	LSMPQSAAVNTTPPHNR	T12	0.950	0.514
Ralgapb	Ral GTPase-activating protein subunit beta	A2ACC6	T734	TNSGISSASGGSTEPTTPDSERPAQALLR	T17	1.029	0.607
Ralgapb	Ral GTPase-activating protein subunit beta	A2ACC6	S716,S720	SHSRTNSGISSASGGSTEPTTPDSERPAQALLR	S3,S7	1.083	0.070
Ralgapb	Ral GTPase-activating protein subunit beta	A2ACC6	S720,T734	TNSGISSASGGSTEPTTPDSERPAQALLR	S3,T17	1.018	0.865
Ralgapb	Ral GTPase-activating protein subunit beta	A2ACC6	Ambiguous	LSMPQSAAVNTTPPHNRR	T/S	1.182	0.108
Ralgps1	Ras-specific guanine nucleotide-releasing factor RalGPS1	A2AR50	S291	IEPGSSSPR	S6	1.036	0.251
Ralgps1	Ras-specific guanine nucleotide-releasing factor RalGPS1	A2AR50	S298	LVSSKEDLAGPSAGSSSAR	S4	1.067	0.664
Ralgps1	Ras-specific guanine nucleotide-releasing factor RalGPS1	A2AR50	T360	SATFPSEK	T3	0.970	0.644
Ralgps1	Ras-specific guanine nucleotide-releasing factor RalGPS1	A2AR50	T319,T331	RPTCPDTSVAGSLPTPPVPR	T3,T15	1.207	0.155
Ralgps1	Ras-specific guanine nucleotide-releasing factor RalGPS1	A2AR50	T319,T331	RRPTCPDTSVAGSLPTPPVPR	T4,T16	1.007	0.955
Ralgps2	Ras-specific guanine nucleotide-releasing factor RalGPS2	Q9ERD6	S308	EDLAGPDIGASPQGGR	S11	0.883	0.133
Ralgps2	Ras-specific guanine nucleotide-releasing factor RalGPS2	Q9ERD6	S308	EDLAGPDIGASPQGGRK	S11	1.015	0.813
Ralgps2	Ras-specific guanine nucleotide-releasing factor RalGPS2	Q9ERD6	S429	LYHSLGPVTR	S4	0.987	0.744
Ralgps2	Ras-specific guanine nucleotide-releasing factor RalGPS2	Q9ERD6	T290	IEPGASTPR	T7	1.031	0.589
Ralgps2	Ras-specific guanine nucleotide-releasing factor RalGPS2	Q9ERD6	T368	SATFPNAGPR	T3	1.018	0.546
Ralgps2	Ras-specific guanine nucleotide-releasing factor RalGPS2	Q9ERD6	S296,S308	SAASREDLAGPDIGASPQGGR	S4,S16	1.051	0.560
Ralgps2	Ras-specific guanine nucleotide-releasing factor RalGPS2	Q9ERD6	Ambiguous	SAASREDLAGPDIGASPQGGR	S	0.964	0.432
Raly	RNA-binding protein Raly	Q64012	S135	LSPVPVPR	S2	0.937	0.258
Raly	RNA-binding protein Raly	Q64012	S135	GRLSPVPVPR	S4	0.978	0.550
Raly	RNA-binding protein Raly	Q64012	S177	STAVTTGSAK	S8	0.989	0.892
Raly	RNA-binding protein Raly	Q64012	T274	LPAPQEDTASEAGTPQGEVQTR	T14	1.026	0.295
Raly	RNA-binding protein Raly	Q64012	S270,T274	LPAPQEDTASEAGTPQGEVQTR	S10,T14	0.996	0.869
Raly	RNA-binding protein Raly	Q64012	S294,T304	DDGDEEGLLTHSEEELEHSQDTDAEDGALQ	S12,T22	1.061	0.349
Raly	RNA-binding protein Raly	Q64012	T268,S270,T274	LPAPQEDTASEAGTPQGEVQTR	T8,S10,T14	0.975	0.675
Raly	RNA-binding protein Raly	Q64012	Ambiguous	DDGDEEGLLTHSEEELEHSQDTDAEDGALQ	S/T	0.945	0.011
Ramac	RNA guanine-N7 methyltransferase activating subunit	Q9CQY2	S36	RPPESPPIVEEWNSR	S5	1.070	0.122
Ranbp1	Ran-specific GTPase-activating protein	P34022	T13	DSHEDHDTSTENADESNHDPQFEPIVSLPEQEIK	T8	0.880	0.790
Ranbp10	Ran-binding protein 10	A0A0R4J0G4	S393,S397	SQDSYPGSPSLSPR	S8,S12	1.029	0.552
Ranbp10	Ran-binding protein 10	A0A0R4J0G4	Ambiguous	SQPHSSTSNQETSDSEMEMEAEHYPNGVLESVSTR	S/T/Y	1.125	0.744
Ranbp10	Ran-binding protein 10	A0A0R4J0G4	Ambiguous	YPAPSSSSSSSSSSSSSSPSSVNYSESNSTDSTK	S/Y/T	1.165	0.458
Ranbp10	Ran-binding protein 10	A0A0R4J0G4	Ambiguous	SQDSYPGSPSLSPR	S/Y	0.973	0.570
Ranbp2	E3 SUMO-protein ligase RanBP2	Q9ERU9	S1154	SHETDGGSAHGDEEDDGPHFEPVVPLPDK	S8	1.100	0.523
Ranbp2	E3 SUMO-protein ligase RanBP2	Q9ERU9	S1605	QNQPTSCVSAPASSETSRSPK	S19	1.116	0.172
Ranbp2	E3 SUMO-protein ligase RanBP2	Q9ERU9	S2035	VTDEENASSGADAPSASDTTAK	S8	0.972	0.619
Ranbp2	E3 SUMO-protein ligase RanBP2	Q9ERU9	S21	YIASVQGSAPSPR	S11	0.998	0.945
Ranbp2	E3 SUMO-protein ligase RanBP2	Q9ERU9	S2505	NRPGYVSEEEEDDEYEMAVK	S7	0.941	0.244
Ranbp2	E3 SUMO-protein ligase RanBP2	Q9ERU9	S2505	NRPGYVSEEEEDDEYEMAVK	S7	0.943	0.281
Ranbp2	E3 SUMO-protein ligase RanBP2	Q9ERU9	S954	FESPATGILSPR	S10	1.051	0.393
Ranbp2	E3 SUMO-protein ligase RanBP2	Q9ERU9	T2130	LNQSGASVGTDEESDVTQEEER	T10	0.874	0.184
Ranbp2	E3 SUMO-protein ligase RanBP2	Q9ERU9	S2087,S2088	EDALDDSVSSSSVHASPLASSPVRK	S20,S21	1.146	0.209
Ranbp2	E3 SUMO-protein ligase RanBP2	Q9ERU9	S2088,S	EDALDDSVSSSSVHASPLASSPVRK	S21,S	1.113	0.632
Ranbp2	E3 SUMO-protein ligase RanBP2	Q9ERU9	Ambiguous	HSTPSPTK	S/T	1.067	0.777
Ranbp2	E3 SUMO-protein ligase RanBP2	Q9ERU9	Ambiguous	HSSSSPVSGTMDKPVDLSTR	S/T	0.923	0.012
Ranbp2	E3 SUMO-protein ligase RanBP2	Q9ERU9	Ambiguous	HSSSSPVSGTMDKPVDLSTR	S/T	1.047	0.631
Ranbp2	E3 SUMO-protein ligase RanBP2	Q9ERU9	Ambiguous	TDLTQGDEVIDTTSEAGETSSTSETTPK	T/S	1.170	0.034
Ranbp3	Ran-binding protein 3	Q9CT10	S146	SPSESAEETHTLEEKVPQK	S1	1.030	0.636
Ranbp3	Ran-binding protein 3	Q9CT10	S148	SPSESAEETHTLEEK	S3	1.020	0.312
Ranbp3	Ran-binding protein 3	Q9CT10	S257	VLSPPKLNEANSDTSR	S3	0.971	0.628
Ranbp3	Ran-binding protein 3	Q9CT10	S279	ETTHAQSGSESSSQEAAPK	S9	0.874	0.237
Ranbp3	Ran-binding protein 3	Q9CT10	S40	SAGSSSPEAGEDSDHEDGNYCPPVKR	S13	0.977	0.524
Ranbp3	Ran-binding protein 3	Q9CT10	S463	ATEEEDSDEDAVLAPSGVTGAGTGDEGDGQAPGST	S7	0.895	0.123
Ranbp3	Ran-binding protein 3	Q9CT10	S58	ERTSSLTHSEEK	S5	1.054	0.676
Ranbp3	Ran-binding protein 3	Q9CT10	S463,S/T	ATEEEDSDEDAVLAPSGVTGAGTGDEGDGQAPGST	S7,S/T	1.028	0.872
Ranbp3	Ran-binding protein 3	Q9CT10	Ambiguous	TSSLTHSEEK	S/T	1.075	0.161
Ranbp3	Ran-binding protein 3	Q9CT10	Ambiguous	SAGSSSPEAGEDSDHEDGNYCPPVK	S/Y	1.072	0.828
Ranbp3	Ran-binding protein 3	Q9CT10	Ambiguous	SAGSSSPEAGEDSDHEDGNYCPPVKR	S/Y	0.982	0.943
Rap1a	Ras-related protein Rap-1A	P62835	S39	YDPTIEDSYRK	S8	1.006	0.967
Rap1gap2	Rap1 GTPase-activating protein 2	A0A171EBK8	S650	SETSSNPSSPEICPNK	S5	1.108	0.133
Rap1gap2	Rap1 GTPase-activating protein 2	A0A171EBK8	S650,S/T	SETSSNPSSPEICPNK	S5,S/T	1.056	0.761
Rap1gds1	RAP1, GTP-GDP dissociation stimulator 1	E9Q912	S597	SVAQQASLTEQR	S7	0.975	0.739
Rapgef1	Rap guanine nucleotide exchange factor (GEF) 1	Q3UHC1	S297	VVDNSPPALPPK	S5	1.046	0.385
Rapgef1	Rap guanine nucleotide exchange factor (GEF) 1	Q3UHC1	S329	ATSGSSLPVGINR	S6	1.009	0.849
Rapgef1	Rap guanine nucleotide exchange factor (GEF) 1	Q3UHC1	S364	SCGGESPRLSPCSSTGK	S10	1.056	0.181
Rapgef1	Rap guanine nucleotide exchange factor (GEF) 1	Q3UHC1	S375	LSRSDEQLSSLDR	S4	1.059	0.519
Rapgef1	Rap guanine nucleotide exchange factor (GEF) 1	Q3UHC1	S375	LSRSDEQLSSLDRDSGQCSR	S4	1.068	0.070
Rapgef1	Rap guanine nucleotide exchange factor (GEF) 1	Q3UHC1	S386	SDEQLSSLDRDSGQCSR	S12	1.082	0.334
Rapgef1	Rap guanine nucleotide exchange factor (GEF) 1	Q3UHC1	S360,S364	SCGGESPRLSPCSSTGK	S6,S10	1.012	0.500
Rapgef1	Rap guanine nucleotide exchange factor (GEF) 1	Q3UHC1	S355,S360,S/T	SCGGESPRLSPCSSTGK	S1,S6,S/T	0.913	0.575
Rapgef2	Rap guanine nucleotide exchange factor 2	A0A0A6YWG7	S1267,S/T	QAEDTISNASSQLSSPPTSPQSSPR	S19,S/T	0.940	0.790
Rapgef2	Rap guanine nucleotide exchange factor 2	A0A0A6YWG7	Ambiguous	SETSPVAPR	T/S	0.962	0.623
Rapgef6	Rap guanine nucleotide exchange factor (GEF) 6	Q5NCJ1	S1165	SSEMSPVPLR	S5	1.044	0.282
Rapgef6	Rap guanine nucleotide exchange factor (GEF) 6	Q5NCJ1	S1165	SSEMSPVPLR	S5	1.032	0.636
Rapgef6	Rap guanine nucleotide exchange factor (GEF) 6	Q5NCJ1	S1598	LADVADADSEADENEQVSAV	S9	0.995	0.955
Rapgef6	Rap guanine nucleotide exchange factor (GEF) 6	Q5NCJ1	Ambiguous	SNRHSIQDVPGDMEQAPQEK	S	1.024	0.791
Raph1	Ras association (RalGDS/AF-6) and pleckstrin homology domains 1	F2Z3U3	S1169	LTQADISEQPAMTTVVPQVPTSPK	S22	1.000	1.000
Raph1	Ras association (RalGDS/AF-6) and pleckstrin homology domains 1	F2Z3U3	S192	TASAGTVSDAEAR	S3	1.014	0.774
Raph1	Ras association (RalGDS/AF-6) and pleckstrin homology domains 1	F2Z3U3	S192	RTASAGTVSDAEAR	S4	1.039	0.751
Raph1	Ras association (RalGDS/AF-6) and pleckstrin homology domains 1	F2Z3U3	S611	SYTSLMPPLSPQTK	S10	1.027	0.756
Raph1	Ras association (RalGDS/AF-6) and pleckstrin homology domains 1	F2Z3U3	S899	SLPASGTPQSPPAVK	S10	1.123	0.007
Rars	Arginine--tRNA ligase, cytoplasmic	Q9D0I9	Ambiguous	SDGGYTYDTSDLAAIK	T/Y/S	0.971	0.893
Rasa1	RAS p21 protein activator 1	E9PYG6	Y451	EIYNTIR	Y3	0.980	0.485
Rasa3	Ras GTPase-activating protein 3	Q60790	S26	NLPSYPGPNK	S4	0.978	0.802
Rasa3	Ras GTPase-activating protein 3	Q60790	S809	YGSQEHPIGDK	S3	1.009	0.866
Rasa3	Ras GTPase-activating protein 3	Q60790	S809	TRYGSQEHPIGDK	S5	1.022	0.434
Rasa3	Ras GTPase-activating protein 3	Q60790	S833	QQSEISTHSI	S9	1.028	0.895
Rasa3	Ras GTPase-activating protein 3	Q60790	S827,S833	QQSEISTHSI	S3,S9	1.136	0.024
Rasa4	Ras GTPase-activating protein 4	Q6PFQ7	S756	GATEAGVSPTGCDGAPEDSLAQLLR	S8	0.910	0.610
Rasal2	RAS protein activator-like 2	E9PW37	S1093	QQTQQVQSPVDSATMSPVER	S8	1.402	0.172
Rasal2	RAS protein activator-like 2	E9PW37	S133	TVSVPSEGQFPEYPQEGTTK	S3	1.064	0.437
Rasal2	RAS protein activator-like 2	E9PW37	S158	LEVPAERSPR	S8	0.974	0.780
Rasal2	RAS protein activator-like 2	E9PW37	T131	RTVSVPSEGQFPEYPQEGTTK	T2	0.766	0.028
Rasal2	RAS protein activator-like 2	E9PW37	T767	SLTNPTPIQQQLR	T6	0.883	0.343
Rasal3	RAS protein activator like-3	Q8C2K5	S239	AKSELGAYTPR	S3	1.137	0.007
Rasal3	RAS protein activator like-3	Q8C2K5	S252	DGPPSALGSR	S5	1.061	0.642
Rasal3	RAS protein activator like-3	Q8C2K5	S74	ALSHQEPMVNSQPAPR	S3	1.171	0.094
Rasal3	RAS protein activator like-3	Q8C2K5	S816	SFQGAGSWASR	S1	0.632	0.373
Rasal3	RAS protein activator like-3	Q8C2K5	S813,S816	SQSLRSFQGAGSWASR	S3,S6	0.910	0.508
Rasal3	RAS protein activator like-3	Q8C2K5	Ambiguous	DGPPSALGSRESLATLSELDLGAER	S/T	1.446	0.052
Rassf2	Ras association domain-containing protein 2	Q8BMS9	S145	TRSDVGVR	S3	1.011	0.860
Raver1	Ribonucleoprotein PTB-binding 1	Q9CW46	S576	LLSPIASNR	S3	0.940	0.504
Raver1	Ribonucleoprotein PTB-binding 1	Q9CW46	S626	HKMSPPPSSFNEPR	S4	0.986	0.719
Raver1	Ribonucleoprotein PTB-binding 1	Q9CW46	S626	HKMSPPPSSFNEPR	S4	0.954	0.550
Rb1	Retinoblastoma-associated protein	P13405	S243	TAAIPINGSPR	S9	1.012	0.777
Rb1	Retinoblastoma-associated protein	P13405	S31	AAAAEPPPPPPPPREDDPAQDSGPEELPLAR	S23	0.985	0.806
Rb1	Retinoblastoma-associated protein	P13405	S31	RAAAAEPPPPPPPPREDDPAQDSGPEELPLAR	S24	0.937	0.460
Rb1	Retinoblastoma-associated protein	P13405	S31	EDDPAQDSGPEELPLAR	S8	1.160	0.395
Rb1	Retinoblastoma-associated protein	P13405	T350	TLQTDPIDSFETERTPR	T15	1.057	0.625
Rb1	Retinoblastoma-associated protein	P13405	T367	KNNPDEEANVVTPHTPVR	T15	1.056	0.338
Rb1	Retinoblastoma-associated protein	P13405	T814	ISEGLPTPTK	T7	1.045	0.732
Rb1cc1	RB1-inducible coiled-coil protein 1	Q9ESK9	S222	ECLGRPDSLNEHEGSEK	S8	1.113	0.029

Rb1cc1	RB1-inducible coiled-coil protein 1	Q9ESK9	S237	STELVLSPDMPR	S1	0.942	0.046
Rb1cc1	RB1-inducible coiled-coil protein 1	Q9ESK9	S237	RSTELVLSPDMPR	S2	0.993	0.870
Rb1cc1	RB1-inducible coiled-coil protein 1	Q9ESK9	S237	RSTELVLSPDMPR	S2	1.073	0.589
Rb1cc1	RB1-inducible coiled-coil protein 1	Q9ESK9	S281	ELRESCQSTVQQEEASVDAK	S5	1.115	0.256
Rb1cc1	RB1-inducible coiled-coil protein 1	Q9ESK9	S652	VSTSQASPQSAASPR	S13	1.006	0.893
Rb1cc1	RB1-inducible coiled-coil protein 1	Q9ESK9	T238	STELVLSPDMPR	T2	1.093	0.132
Rb1cc1	RB1-inducible coiled-coil protein 1	Q9ESK9	S646,S652	VSTSQASPQSAASPR	S7,S13	0.892	0.367
Rb1cc1	RB1-inducible coiled-coil protein 1	Q9ESK9	Ambiguous	EDSSSLVAELQEK	S	0.763	0.431
Rbbp4	Histone-binding protein RBBP4	Q60972	Ambiguous	TPSSDVLVFDYTK	S/T/Y	1.103	0.626
Rbbp6	E3 ubiquitin-protein ligase RBBP6	P97868	S1053	VDGDREKSPR	S8	0.969	0.535
Rbbp6	E3 ubiquitin-protein ligase RBBP6	P97868	S1278	VAGSEGSSSTLVDYTSTSSTGGSPVR	S23	0.996	0.978
Rbbp6	E3 ubiquitin-protein ligase RBBP6	P97868	S1683	ESLTVATASQPGADR	S9	0.988	0.915
Rbbp6	E3 ubiquitin-protein ligase RBBP6	P97868	T985	DDATPVRDEPMDAESITFK	T4	1.012	0.824
Rbbp6	E3 ubiquitin-protein ligase RBBP6	P97868	Ambiguous	VAGSEGSSSTLVDYTSTSSTGGSPVRK	S/T/Y	1.103	0.457
Rbbp6	E3 ubiquitin-protein ligase RBBP6	P97868	Ambiguous	GYAVGAQPRPSANREDFSPER	S/Y	1.027	0.743
Rbbp8	DNA endonuclease RBBP8	Q80YR6	S233	DSAPAPVNSEEHEILVADTCDQNHSPLSK	S25	0.976	0.757
Rbbp8	DNA endonuclease RBBP8	Q80YR6	S885	QPYNAVFSPK	S8	1.073	0.678
Rbck1	RanBP-type and C3HC4-type zinc finger-containing protein 1	Q9WUB0	S329	NSQEAEVACPFIDSTYSCPGK	S17	1.053	0.496
Rbl1	Retinoblastoma-like protein 1	Q64701	S1036	VIAISGDADSPAKR	S10	0.968	0.723
Rbl1	Retinoblastoma-like protein 1	Q64701	S640	QDMQPLSPISVHER	S7	1.086	0.523
Rbl1	Retinoblastoma-like protein 1	Q64701	S640	QDMQPLSPISVHER	S7	0.987	0.859
Rbl1	Retinoblastoma-like protein 1	Q64701	S650	YSSPAAGSAK	S3	1.092	0.177
Rbl1	Retinoblastoma-like protein 1	Q64701	T369	SFAPSTPLTGR	T6	1.098	0.069
Rbl1	Retinoblastoma-like protein 1	Q64701	T385	EAVTTPVASATQSVSR	T5	1.160	0.041
Rbl2	Retinoblastoma-like protein 2	Q64700	S1108	GILLDDGSESPAKR	S10	0.997	0.908
Rbl2	Retinoblastoma-like protein 2	Q64700	S37	AQPAGSPSHQIQQR	S6	1.125	0.272
Rbl2	Retinoblastoma-like protein 2	Q64700	S410	YVQENSPCVTPVSTAAHSLSR	S6	1.059	0.838
Rbl2	Retinoblastoma-like protein 2	Q64700	S636	TDEIYIAGSPLTPR	S9	1.046	0.664
Rbl2	Retinoblastoma-like protein 2	Q64700	S659	SITSPTTLYDR	S4	0.956	0.640
Rbl2	Retinoblastoma-like protein 2	Q64700	S684	LFENDSPSEGSTSGR	S6	0.893	0.054
Rbl2	Retinoblastoma-like protein 2	Q64700	S976	SNSTLPVPQPSSAPPTPTR	S12	0.910	0.309
Rbl2	Retinoblastoma-like protein 2	Q64700	T1027	YSQANAQTDTPPLSPYPFVR	T10	0.836	0.618
Rbl2	Retinoblastoma-like protein 2	Q64700	S410,T414	YVQENSPCVTPVSTAAHSLSR	S6,T10	1.137	0.516
Rbl2	Retinoblastoma-like protein 2	Q64700	S976,T980	SNSTLPVPQPSSAPPTPTR	S12,T16	1.012	0.921
Rbl2	Retinoblastoma-like protein 2	Q64700	S967,T980,S/T	SNSTLPVPQPSSAPPTPTR	S3,T16,S/T	1.085	0.553
Rbl2	Retinoblastoma-like protein 2	Q64700	Ambiguous	YSSPTVSTTR	S/T/Y	1.004	0.915
Rbl2	Retinoblastoma-like protein 2	Q64700	Ambiguous	GILLDDGSESPAK	S	0.786	0.243
Rbm10	RNA-binding protein 10	Q99KG3	S723	LASDDRPSPPR	S8	1.026	0.410
Rbm12b2	RNA-binding protein 12B-B	Q66JV4	S555	QSDRCSPEDFR	S6	0.865	0.282
Rbm12b2	RNA-binding protein 12B-B	Q66JV4	S681	RVSVEDLR	S3	0.996	0.926
Rbm14	RNA-binding protein 14	Q8C2Q3	S220	SPPRASYVAPLTAQPATYR	S1	0.869	0.326
Rbm14	RNA-binding protein 14	Q8C2Q3	S256	AQPSASLGVGYR	S6	1.198	0.013
Rbm14	RNA-binding protein 14	Q8C2Q3	S280	AQPSVSLGAPYR	S6	1.383	0.058
Rbm14	RNA-binding protein 14	Q8C2Q3	S520	TQSSASLAASYAAQQHPQAAASYR	S3	1.127	0.083
Rbm14	RNA-binding protein 14	Q8C2Q3	S618	RLSESQLSFR	S3	0.942	0.240
Rbm14	RNA-binding protein 14	Q8C2Q3	T206	QPTPPFFGR	T3	0.959	0.402
Rbm15	RNA-binding protein 15	Q0VBL3	S127	LHSYSSPSTK	S6	0.954	0.414
Rbm15	RNA-binding protein 15	Q0VBL3	S20	RASPLCETSAGWR	S3	1.099	0.400
Rbm15	RNA-binding protein 15	Q0VBL3	S293	SLSPGGAALGYR	S3	0.957	0.267
Rbm15	RNA-binding protein 15	Q0VBL3	S364	VGAGAGAAPFREVDEISPEDDQR	S17	1.224	0.150
Rbm15	RNA-binding protein 15	Q0VBL3	S621	DGWSLDRDR	S4	0.937	0.058
Rbm15	RNA-binding protein 15	Q0VBL3	S655	HLDRSPESERPR	S5	0.955	0.105
Rbm15	RNA-binding protein 15	Q0VBL3	S782	QDGGTAPVAASSPK	S12	1.035	0.478
Rbm15	RNA-binding protein 15	Q0VBL3	T567	DRTPPLLYR	T3	1.050	0.593
Rbm15	RNA-binding protein 15	Q0VBL3	S256,S258	SRSPLDKDAYAPSSSVVGTSVGSHR	S1,S3	0.911	0.104
Rbm15	RNA-binding protein 15	Q0VBL3	Ambiguous	AATSAVTAYEPLDSLDR	S/T/Y	0.812	0.242
Rbm15b	Putative RNA-binding protein 15B	Q6PHZ5	S107	ASGDPGAGGASPR	S11	0.974	0.793
Rbm15b	Putative RNA-binding protein 15B	Q6PHZ5	S111	ASPLPPPPPPGAEPAGPGSTAAPEYK	S2	0.997	0.988
Rbm15b	Putative RNA-binding protein 15B	Q6PHZ5	S263	SLSPVAAPPLREPR	S3	0.928	0.062
Rbm15b	Putative RNA-binding protein 15B	Q6PHZ5	T529	DRTPPHLLYSDRDR	T3	0.908	0.167
Rbm17	Splicing factor 45	Q8JZX4	S155	RPDPDSDEDEDYER	S6	1.027	0.639
Rbm17	Splicing factor 45	Q8JZX4	S155	RPDPDSDEDEDYERER	S6	1.036	0.789
Rbm25	RNA-binding protein 25	B2RY56	S578	QEPESEEEEEKQEK	S5	1.006	0.925
Rbm25	RNA-binding protein 25	B2RY56	S578	QEPESEEEEEKQEKEEK	S5	1.005	0.954
Rbm25	RNA-binding protein 25	B2RY56	S578	QEPESEEEEEK	S5	1.004	0.965
Rbm25	RNA-binding protein 25	B2RY56	S672	LGASNSPGQPNSVKR	S6	0.926	0.210
Rbm25	RNA-binding protein 25	B2RY56	S672	LGASNSPGQPNSVK	S6	0.985	0.792
Rbm26	RNA-binding protein 26	Q6NZN0	S127	LNHSPPQSSSR	S4	1.025	0.591
Rbm26	RNA-binding protein 26	Q6NZN0	S523	TNSPGFQK	S3	1.001	0.973
Rbm26	RNA-binding protein 26	Q6NZN0	S621	EGTTQQLQTTSPK	S11	1.104	0.306
Rbm3	RNA-binding protein 3	O89086	S135	DYSGSQGGYDR	S5	1.032	0.615
Rbm3	RNA-binding protein 3	O89086	S143	DYSGSQGGYDRYSGGNYR	S13	0.955	0.758
Rbm3	RNA-binding protein 3	O89086	S143	DYSGSQGGYDRYSGGNYRDNYDN	S13	0.949	0.287
Rbm3	RNA-binding protein 3	Q8BG13; O89086	S117;S117	YDSRPGGYGYGYGR	S3	1.178	0.117
Rbm3	RNA-binding protein 3	Q8BG13	S144	SQGGYDRYSGGNYRDNYDN	S9	0.952	0.770
Rbm33	RNA-binding protein 33	Q9CXK9	S1032	ANSGGGGDGPHVSSK	S3	1.343	0.030
Rbm33	RNA-binding protein 33	Q9CXK9	S243	DIKEESDEEDDDDEESGRLR	S6	0.929	0.461
Rbm33	RNA-binding protein 33	Q9CXK9	S243	DIKEESDEEDDDDEESGR	S6	1.033	0.743
Rbm33	RNA-binding protein 33	Q9CXK9	S792	SIVNTSPPCR	S6	1.008	0.776
Rbm33	RNA-binding protein 33	Q9CXK9	S816	AKPLSPEAQPK	S5	0.999	0.994
Rbm39	RNA-binding protein 39	Q8VH51	S136	SPVREPIDNLTPEER	S1	0.855	0.160
Rbm39	RNA-binding protein 39	Q8VH51	S136	DKSPVREPIDNLTPEER	S3	0.979	0.478
Rbm39	RNA-binding protein 39	Q8VH51	S97	YRSPYSGPK	S3	1.017	0.898
Rbm39	RNA-binding protein 39	Q8VH51	Ambiguous	TDASSASSFLDSDELER	S/T	1.061	0.753
Rbm45	RNA-binding protein 45	Q8BHN5	S199	VSGSPEQDDYSSGR	S4	0.991	0.945
Rbm45	RNA-binding protein 45	Q8BHN5	Ambiguous	VSGSPEQDDYSSGRQEALGQEPR	S/Y	0.997	0.963
Rbm5	RNA-binding protein 5	Q91YE7	S59	YDDYRDYDSPERER	S9	1.050	0.025
Rbm5	RNA-binding protein 5	Q91YE7	S624	GLVAAYSGDSDNEEELVER	S10	1.006	0.934
Rbm6	RNA-binding motif protein 6	S4R1W5	S1020	LQSFDSPER	S6	0.958	0.235
Rbm6	RNA-binding motif protein 6	S4R1W5	S1020	EKLQSFDSPER	S8	1.006	0.907
Rbm6	RNA-binding motif protein 6	S4R1W5	S299	VLDYIQPSPQER	S8	1.103	0.586
Rbm6	RNA-binding motif protein 6	S4R1W5	S362	EGETQSGTFEHESQSDFQNSQSPVQEQDKPK	S22	0.996	0.987
Rbm6	RNA-binding motif protein 6	S4R1W5	S888	KEESPPPPK	S4	0.999	0.990
Rbm7	RNA-binding protein 7	Q9CQT2	S125	TVGNVSPTAQMVQR	S6	1.035	0.576
Rbm7	RNA-binding protein 7	Q9CQT2	S136	SFSSPEDYQR	S3	1.374	0.011
Rbm7	RNA-binding protein 7	Q9CQT2	S203	QNSHPYLADR	S3	1.367	0.021
Rbm8a	RNA-binding protein 8A	Q9CWZ3	S42	GFGSEEGSR	S4	0.981	0.178
Rbm8a	RNA-binding protein 8A	Q9CWZ3	S56	MREDYDSVEQDGDEPGPQR	S7	0.971	0.439
Rbms2	RNA-binding motif, single-stranded-interacting protein 2	Q8VC70	S33	LYVAQQMAPPSPR	S11	0.927	0.236
Rbms2	RNA-binding motif, single-stranded-interacting protein 2	Q8VC70	S41	NSTPNSSGGGGGSGGNDQLSK	S6	1.021	0.832
Rbmxl1	RNA binding motif protein, X-linked-like-1	Q91VM5	S205	DVYLSPR	S5	1.065	0.615
Rbmxl1	RNA binding motif protein, X-linked-like-1	Q91VM5	S205	DVYLSPRDDGYSTK	S5	1.045	0.780
Rbmxl1	RNA binding motif protein, X-linked-like-1	Q91VM5	S205	RDVYLSPR	S6	1.003	0.922
Rbmxl1	RNA binding motif protein, X-linked-like-1	Q91VM5	S216	DDGYSTKDSYSSR	S9	1.033	0.562
Rbmxl1	RNA binding motif protein, X-linked-like-1	Q91VM5	S323	DGYGGSRDSYSSSR	S9	1.069	0.066
Rbmxl1	RNA binding motif protein, X-linked-like-1	Q91VM5	S329	DSYSSSRSDLYSSGR	S8	0.961	0.312
Rbmxl1	RNA binding motif protein, X-linked-like-1	Q91VM5	S349	GLPPSMER	S5	1.021	0.679
Rbmxl1	RNA binding motif protein, X-linked-like-1	Q91VM5	S205,S212	DVYLSPRDDGYSTKDSYSSR	S5,S12	0.978	0.833
Rbmxl1	RNA binding motif protein, X-linked-like-1	Q91VM5	Ambiguous	RSTPSGPVR	S/T	1.019	0.575
Rbmxl1	RNA binding motif protein, X-linked-like-1	Q91VM5	Ambiguous	YDDYSSSRDGYGGSR	S/Y	0.998	0.964
Rc3h1	Roquin-1	Q4VGL6	S1110	TSSLNLSEDSEGGGDNNDSQR	S10	0.969	0.621
Rc3h1	Roquin-1	Q4VGL6	S861	AVETSDDDLIPFGDRPTVSR	S5	1.028	0.706
Rc3h1	Roquin-1	Q4VGL6	Ambiguous	RAVETSDDDLIPFGDRPTVSR	S/T	0.998	0.970
Rcc1	Rcc1 protein	Q6PFB2	S11	RRSPPEDAIPK	S3	0.979	0.574
Rcc2	Protein RCC2	Q8BK67	S48,S49	CSSSSGGGSSGDEDGPELDGAPGGGK	S9,S10	1.076	0.809
Rcc2	Protein RCC2	Q8BK67	Ambiguous	CSSSSGGGSSGDEDGPELDGAPGGGKR	S	0.967	0.776
Rcc2	Protein RCC2	Q8BK67	Ambiguous	CSSSSGGGSSGDEDGPELDGAPGGGK	S	1.015	0.898
Rcor1	REST corepressor 1	Q8CFE3	S254	EREESEDELEETNGSNPVDIEIDPNKESK	S5	1.033	0.731
Rcor3	REST corepressor 3	A0A0A6YXM5	S212	HNQGDSDDDVEEAHPMDGNDSDYDPK	S6	1.109	0.047
Rcor3	REST corepressor 3	A0A0A6YXM5	S212	HNQGDSDDDVEEAHPMDGNDSDYDPKK	S6	1.149	0.105
Rcsd1	CapZ-interacting protein	Q3UZA1	S177	SQSDCGDFR	S1	1.154	0.001
Rcsd1	CapZ-interacting protein	Q3UZA1	S216	SKDPGSPQLNQEAMADGVEGTPWSAEKPR	S6	1.119	0.235
Rcsd1	CapZ-interacting protein	Q3UZA1	S246	NTCNSTEKPEELVR	S5	0.875	0.023
Rcsd1	CapZ-interacting protein	Q3UZA1	S333	VADEDRLGQKSPDANMPEEEGVVR	S11	0.982	0.724
Rcsd1	CapZ-interacting protein	Q3UZA1	S333	VADEDRLGQKSPDANMPEEEGVVR	S11	0.931	0.400
Rcsd1	CapZ-interacting protein	Q3UZA1	S333	LGQKSPDANMPEEEGVVR	S5	1.053	0.119
Rcsd1	CapZ-interacting protein	Q3UZA1	S333	LGQKSPDANMPEEEGVVR	S5	0.933	0.294
Rcsd1	CapZ-interacting protein	Q3UZA1	S380	QKEEAPLEPSCSPGADHAAGEITSEIQNEK	S12	0.938	0.452
Rcsd1	CapZ-interacting protein	Q3UZA1	S401	AVSMDDIPIEDTRM	S3	0.829	0.362
Rcsd1	CapZ-interacting protein	Q3UZA1	S401	AVSMDDIPIEDTR	S3	0.999	0.992
Rcsd1	CapZ-interacting protein	Q3UZA1	S83	SSPLIEK	S2	1.090	0.247
Rcsd1	CapZ-interacting protein	Q3UZA1	T256	NTCNSTEKPEELVRTPEEANAGEK	T15	0.997	0.968
Rcsd1	CapZ-interacting protein	Q3UZA1	S116,S120	AIVSPFHSPPSTPSSPGIR	S4,S8	0.994	0.918
Rcsd1	CapZ-interacting protein	Q3UZA1	T243,S/T	RNTCNSTEKPEELVR	T3,S/T	1.135	0.051
Rcsd1	CapZ-interacting protein	Q3UZA1	S116,S120,S123	AIVSPFHSPPSTPSSPGIR	S4,S8,S11	1.173	0.063
Rcsd1	CapZ-interacting protein	Q3UZA1	Ambiguous	VGQNPDTASQGHPEVQAPSQTGSPEAENGCGSPR	T/S	0.944	0.717
Rcsd1	CapZ-interacting protein	Q3UZA1	Ambiguous	VGQNPDTASQGHPEVQAPSQTGSPEAENGCGSPR	S/T	0.741	0.347
Rdx	Radixin	P26043; P26041; P26040	Y270;Y270;Y270	APDFVFYAPR	Y7	0.715	0.373

Recql5	ATP-dependent DNA helicase Q5	Q8VID5	S773	TESPPLPASVPR	S3	0.971	0.520
Recql5	ATP-dependent DNA helicase Q5	Q8VID5	T527	IEEFTPPDEDCPLR	T7	1.064	0.540
Reep3	Receptor expression-enhancing protein	A0A1W2P8A8	T200,S209	DGSEQTDEEAEGPFSDDEMVTHK	T6,S15	1.155	0.092
Reep4	Receptor expression-enhancing protein	A0A2I3BQJ3	T198	SIPDTPVPTYQPLYLEDQVPR	T5	1.657	0.087
Rela	Transcription factor p65	Q04207	S238	GSFSQADVHR	S2	1.108	0.127
Rela	Transcription factor p65	Q04207	S45	SAGSIPGER	S4	1.018	0.855
Relb	Transcription factor RelB	Q04863	S552	EAAFGGGLLSPGPEAT	S10	1.297	0.272
Relch	RAB11-binding protein RELCH	E9QM90	Ambiguous	QSGTPPGMGAPGIPGASIVGGAGGR	S/T	0.975	0.868
Relch	RAB11-binding protein RELCH	E9QM90	Ambiguous	EPSTTSGGGQLNR	S/T	1.070	0.376
Rell1	RELT-like protein 1	Q8K2J7	S225	RQGEVTVLSVGR	S9	1.046	0.235
Relt	Tumor necrosis factor receptor superfamily member 19L	Q8BX43	S389	TITEAGPSEGDLPDSPQPGLPPEQR	S15	0.887	0.298
Rem1	GTP-binding protein REM 1	O35929	S18	RASTPLPLSSR	S3	1.016	0.899
Renbp	N-acylglucosamine 2-epimerase	P82343	S420	LEPAPLDSSPAVSTHEGSK	S9	1.007	0.945
Renbp	N-acylglucosamine 2-epimerase	P82343	Ambiguous	LEPAPLDSSPAVSTHEGSK	S/T	1.032	0.837
Reps1	RalBP1-associated Eps domain-containing protein 1	O54916	S118	LAASYSSDSENQGSYSGVIPPPPGR	S7	0.900	0.321
Reps1	RalBP1-associated Eps domain-containing protein 1	O54916	S272	RQSSSYEDPWKITDEQR	S3	0.918	0.323
Reps1	RalBP1-associated Eps domain-containing protein 1	O54916	S272	RQSSSYEDPWK	S3	1.013	0.856
Reps1	RalBP1-associated Eps domain-containing protein 1	O54916	S273	QSSSYEDPWKITDEQR	S3	1.390	0.046
Reps1	RalBP1-associated Eps domain-containing protein 1	O54916	S708	RLKSEDELRPDVDEHTQK	S4	1.105	0.306
Reps1	RalBP1-associated Eps domain-containing protein 1	O54916	Ambiguous	SHSGTSPDNTAPPPPPPRPQPSHSR	S/T	1.165	0.340
Reps2	RalBP1-associated Eps domain-containing protein 2	B9EI38	S240	RQASLIR	S4	0.994	0.886
Reps2	RalBP1-associated Eps domain-containing protein 2	B9EI38	S246	SFSVEREPQENNSNYPDEPWR	S3	1.048	0.586
Reps2	RalBP1-associated Eps domain-containing protein 2	B9EI38	S532	APSQAAESSPTK	S3	1.015	0.659
Rer1	Protein RER1	Q9CQU3	S95	VDPSLMEDSDDGPSLPTK	S9	1.051	0.487
Rer1	Protein RER1	Q9CQU3	S95	VDPSLMEDSDDGPSLPTK	S9	1.125	0.357
Rere	Arginine-glutamic acid dipeptide repeats protein	Q80TZ9	S1258	TLSEYARPHVMSPTNR	S12	0.949	0.232
Rere	Arginine-glutamic acid dipeptide repeats protein	Q80TZ9	S1258	TLSEYARPHVMSPTNR	S12	0.991	0.813
Rere	Arginine-glutamic acid dipeptide repeats protein	Q80TZ9	S600	QPTSPDGRASPINEDIR	S10	0.972	0.423
Rere	Arginine-glutamic acid dipeptide repeats protein	Q80TZ9	S600	ASPINEDIR	S2	1.052	0.481
Rere	Arginine-glutamic acid dipeptide repeats protein	Q80TZ9	S613	SSGRNSPSAASTSSNDSK	S6	1.107	0.057
Rere	Arginine-glutamic acid dipeptide repeats protein	Q80TZ9	S656	VASDTEDTDR	S3	1.074	0.485
Rere	Arginine-glutamic acid dipeptide repeats protein	Q80TZ9	S656	VASDTEDTDRITSK	S3	1.012	0.818
Rere	Arginine-glutamic acid dipeptide repeats protein	Q80TZ9	S656	EKVASDTEDTDR	S5	0.934	0.353
Rere	Arginine-glutamic acid dipeptide repeats protein	Q80TZ9	S692	SVNDEGSSDPK	S1	0.895	0.081
Rere	Arginine-glutamic acid dipeptide repeats protein	Q80TZ9	S53,S56	NYAESDHSEDEDNDNNSATTEESNKK	S5,S8	0.928	0.700
Rere	Arginine-glutamic acid dipeptide repeats protein	Q80TZ9	S600,T/S	QPTSPDGRASPINEDIR	S10,S/T	1.062	0.214
Rere	Arginine-glutamic acid dipeptide repeats protein	Q80TZ9	S675,S679	TQEISRPNSPSEGEGSSDSR	S5,S9	0.975	0.681
Rere	Arginine-glutamic acid dipeptide repeats protein	Q80TZ9	Ambiguous	KQPTSPDGRASPINEDIR	S/T	1.034	0.753
Rest	RE1-silencing transcription factor	Q8VIG1	S760	ELPSPVEPAQIEVAQTAPTQVQEEPPPVSEPPR	S4	1.326	0.229
Rest	RE1-silencing transcription factor	Q8VIG1	S848	SAQDPPAPPSPSPK	S10	0.969	0.672
Retreg2	Reticulophagy regulator 2	Q6NS82	Ambiguous	ATTPQLTDVSEDLDQQSLPSEPEEALNR	S/T	0.798	0.575
Retreg3	Reticulophagy regulator 3	Q9CQV4	S26	AMSGSWER	S3	1.116	0.149
Retreg3	Reticulophagy regulator 3	Q9CQV4	S26	AMSGSWERDQQVEAAQR	S3	1.042	0.605
Retreg3	Reticulophagy regulator 3	Q9CQV4	S26	RAMSGSWER	S4	1.018	0.827
Retreg3	Reticulophagy regulator 3	Q9CQV4	S320	GQTPLTEGSEDLDGHSDPEESFAR	S16	1.005	0.855
Retreg3	Reticulophagy regulator 3	Q9CQV4	S313,S320	GQTPLTEGSEDLDGHSDPEESFAR	S9,S16	1.070	0.349
Rfc1	Replication factor C subunit 1	G3UWX1	S258	KDSEEGEESFSSVQDDLSK	S3	1.009	0.960
Rfc1	Replication factor C subunit 1	G3UWX1	S379	ESVSPEDSEK	S4	1.030	0.658
Rfc1	Replication factor C subunit 1	G3UWX1	S68	IINDSDSESEETVQVK	S5	0.928	0.417
Rfc1	Replication factor C subunit 1	G3UWX1	S68,S70	IINDSDSESEETVQVK	S5,S7	1.081	0.094
Rffl	E3 ubiquitin-protein ligase rififylin	Q3UCG9	Ambiguous	VPTEDETQSVDSEDSFVPGR	S/T	1.037	0.891
Rffl	E3 ubiquitin-protein ligase rififylin	Q3UCG9	Ambiguous	TQAYSNPGYSSFPSPTGSEPSCK	S/Y/T	1.005	0.974
Rftn1	Raftlin	Q6A0D4	S183	LKPVANSVEDAR	S7	1.011	0.924
Rftn1	Raftlin	Q6A0D4	S199	CTLGDRSSLENDTPKAAETDAATAGVNR	S8	0.785	0.489
Rftn1	Raftlin	Q6A0D4	S199	CTLGDRSSLENDTPK	S8	0.999	0.990
Rftn1	Raftlin	Q6A0D4	T549	AEAELAAGPTPTEAN	T10	0.845	0.186
Rftn1	Raftlin	Q6A0D4	Ambiguous	FVSVVPQYQPSVSSAGSR	S/Y	1.194	0.311
Rftn1	Raftlin	Q6A0D4	Ambiguous	FVSVVPQYQPSVSSAGSRR	S/Y	0.937	0.595
Rftn1	Raftlin	Q6A0D4	Ambiguous	FVSVVPQYQPSVSSAGSRR	S/Y	0.846	0.459
Rfx1	MHC class II regulatory factor RFX1	P48377	S113	ASETVSEASPSSTASQTGVPTQVVQQVQGTQQR	S9	1.097	0.734
Rfx7	Regulatory factor X, 7	F8VPJ6	S322	LQSPLPGESSAK	S3	0.976	0.733
Rfx7	Regulatory factor X, 7	F8VPJ6	S379	QLVTSPSPMNSPDGK	S5	1.004	0.932
Rfx7	Regulatory factor X, 7	F8VPJ6	Ambiguous	LAQTTPVDSALGSSR	T/S	1.255	0.392
Rfx7	Regulatory factor X, 7	F8VPJ6	Ambiguous	QLVTSPSPMNSPDGK	T/S	1.016	0.856
Rgcc	Regulator of cell cycle RGCC	Q9DBX1	S75	SSASVSDSSGFSDSESADSVYR	S12	0.799	0.224
Rgl2	Ral guanine nucleotide dissociation stimulator-like 2	Q61193	S518	VSCEVEPPGTSDSPAAR	S13	1.006	0.934
Rgl2	Ral guanine nucleotide dissociation stimulator-like 2	Q61193	S619	SASCGSPLSGNTGEGTSR	S3	1.054	0.319
Rgl2	Ral guanine nucleotide dissociation stimulator-like 2	Q61193	S619	RSASCGSPLSGNTGEGTSR	S4	0.930	0.089
Rgl2	Ral guanine nucleotide dissociation stimulator-like 2	Q61193	S762	RPSAATPGSHSGPSASGTPPSEGGGGSFPR	S27	0.933	0.501
Rgl3	Ral guanine nucleotide dissociation stimulator-like 3	Q3UYI5	S568	NREPPPPGSPPASPGPQSPSTK	S9	0.996	0.885
Rgl3	Ral guanine nucleotide dissociation stimulator-like 3	Q3UYI5	S506,S	VIEPPAASCPSSPR	S8,S	1.053	0.538
Rgl3	Ral guanine nucleotide dissociation stimulator-like 3	Q3UYI5	S568,S572	NREPPPPGSPPASPGPQSPSTK	S9,S13	1.026	0.658
Rgl3	Ral guanine nucleotide dissociation stimulator-like 3	Q3UYI5	Ambiguous	VIEPPAASCPSSPR	S	1.076	0.255
Rgl3	Ral guanine nucleotide dissociation stimulator-like 3	Q3UYI5	Ambiguous	EGTGHTLSASPT	T/S	1.003	0.865
Rgs10	Regulator of G-protein signaling 10	Q9CQE5	S16	RPPSDIHDGDGSSSSGHQSLK	S4	0.986	0.878
Rgs12	Regulator of G-protein-signaling 12	E9Q652	S850	TLPDSQQVPSSPASK	S11	0.924	0.455
Rgs12	Regulator of G-protein-signaling 12	E9Q652	S934	RESQGSVSSAGSLDLSEACR	S3	1.176	0.172
Rgs12	Regulator of G-protein-signaling 12	E9Q652	S876,S879	SKSGRSLNEDVGEEDSEK	S3,S6	0.961	0.607
Rgs12	Regulator of G-protein-signaling 12	E9Q652	Ambiguous	DCISNNSLSSNASLPSVQSCR	S	1.096	0.610
Rgs12	Regulator of G-protein-signaling 12	E9Q652	Ambiguous	SLSESAALR	S	1.142	0.057
Rgs14	Regulator of G-protein signaling 14	P97492	S20	MVLAVSDGELTSTAGSQAQGEGR	S6	0.765	0.109
Rgs14	Regulator of G-protein signaling 14	P97492	S289	SLGSGESESESRPGK	S1	1.040	0.190
Rgs14	Regulator of G-protein signaling 14	P97492	Ambiguous	EPGSSHLGSPDTAR	S/T	0.794	0.050
Rgs18	Regulator of G-protein signaling 18	Q99PG4	S218	SFTYNDFQDVK	S1	1.012	0.840
Rgs18	Regulator of G-protein signaling 18	Q99PG4	S218	SRSFTYNDFQDVK	S3	1.093	0.764
Rgs19	Regulator of G-protein-signaling 19	B7ZCT1	S107	LQPLPSCEVCTPPSPK	S14	1.047	0.639
Rgs19	Regulator of G-protein-signaling 19	B7ZCT1	Ambiguous	SLLLQGAPQSSEA	S	1.069	0.793
Rgs3	Regulator of G-protein signaling 3	Q9DC04	S526	DSSGQEAAPGPESPSSEDIATCPKPPQSPETSTSK	S28	0.877	0.193
Rgs3	Regulator of G-protein signaling 3	Q9DC04	S712	THSEGSLLQESR	S3	1.040	0.413
Rhbdd1	Rhomboid-related protein 4	Q8BHC7	S269	NYDVYTAGLSEEEQLER	S10	1.370	0.210
Rhbdf2	Inactive rhomboid protein 2	Q80WQ6	S197	SGYSHLPR	S1	1.053	0.693
Rhbdf2	Inactive rhomboid protein 2	Q80WQ6	S359	SISSTVQR	S3	1.040	0.357
Rhbdf2	Inactive rhomboid protein 2	Q80WQ6	S60	SVSLQEPR	S3	1.382	0.003
Rhbdf2	Inactive rhomboid protein 2	Q80WQ6	S83	QASLSQSIR	S3	1.461	0.000
Rhbdf2	Inactive rhomboid protein 2	Q80WQ6	S83	RQASLSQSIR	S4	1.220	0.068
Rheb	GTP-binding protein Rheb	Q921J2	S130	VISYEEGK	S3	0.934	0.308
Rhobtb1	Rho-related BTB domain-containing protein 1	Q9DAK3	S325	TQSLGSAEEGKEGPQR	S3	1.019	0.870
Rhov	Rho-related GTP-binding protein RhoV	Q8VDU1	S25	SAPPELGIK	S1	1.040	0.165
Ric1	RAB6A-GEF complex partner protein 1	E9QPA1	S1016	SISLSQSAENVPPGK	S3	1.139	0.213
Ric1	RAB6A-GEF complex partner protein 1	E9QPA1	Ambiguous	TLSMPTGPSGK	T/S	1.130	0.101
Ric1	RAB6A-GEF complex partner protein 1	E9QPA1	Ambiguous	AIGSGESETPPSTPTSQEPSSSGGFEFFR	T/S	0.944	0.840
Ric8a	Synembryn-A	Q3TIR3	S435	GLMAGGRPEGQYSEDEDTDTEEYR	S13	0.993	0.799
Ric8a	Synembryn-A	Q3TIR3	S501	VIQPMGMSPR	S8	1.071	0.016
Ric8a	Synembryn-A	Q3TIR3	S501	VIQPMGMSPR	S8	0.965	0.507
Ric8a	Synembryn-A	Q3TIR3	S435,T440	GLMAGGRPEGQYSEDEDTDTEEYR	S13,T18	1.080	0.493
Ric8a	Synembryn-A	Q3TIR3	S435,T440	GLMAGGRPEGQYSEDEDTDTEEYR	S13,T18	1.034	0.579
Ric8a	Synembryn-A	Q3TIR3	S435,T440	GLMAGGRPEGQYSEDEDTDTEEYREAK	S13,T18	0.928	0.237
Ric8a	Synembryn-A	Q3TIR3	T440,Y/S/T	GLMAGGRPEGQYSEDEDTDTEEYREAK	T18,Y/S/T	0.805	0.124
Ric8a	Synembryn-A	Q3TIR3	Ambiguous	GLMAGGRPEGQYSEDEDTDTEEYREAK	S/Y/T	0.934	0.636
Rictor	Rapamycin-insensitive companion of mTOR	Q6QI06	S1234	SQSFNTDTTTSGISSMSSSPSR	S19	1.022	0.899
Rictor	Rapamycin-insensitive companion of mTOR	Q6QI06	S1576	FSGISGCSDGASQEEGSASSTK	S8	1.211	0.009
Rictor	Rapamycin-insensitive companion of mTOR	Q6QI06	S21	NDSGEENVPLDLTR	S3	1.150	0.043
Rictor	Rapamycin-insensitive companion of mTOR	Q6QI06	S21	GRNDSGEENVPLDLTR	S5	0.960	0.309
Rictor	Rapamycin-insensitive companion of mTOR	Q6QI06	S21	GRNDSGEENVPLDLTREPSDNLR	S5	0.994	0.919
Rictor	Rapamycin-insensitive companion of mTOR	Q6QI06	S1173,T/S	HLEDAGSTPSIGENDLK	S7,T/S	0.935	0.079
Rictor	Rapamycin-insensitive companion of mTOR	Q6QI06	S1218,S1234	SQSFNTDTTTSGISSMSSSPSR	S3,S19	0.919	0.637
Rif1	Telomere-associated protein RIF1	Q6PR54	S1351	DGDPKPAVENASLEDLTTEEK	S12	0.816	0.105
Rif1	Telomere-associated protein RIF1	Q6PR54	S1387	TEQIVNEDSQAAALAPNPK	S9	1.050	0.780
Rif1	Telomere-associated protein RIF1	Q6PR54	S1457	LTDESPIQENLTEK	S5	1.169	0.115
Rif1	Telomere-associated protein RIF1	Q6PR54	S1565	SSDSVDIEEQEEK	S4	1.545	0.001
Rif1	Telomere-associated protein RIF1	Q6PR54	S1683	VSDSSLSPEK	S7	0.974	0.756
Rif1	Telomere-associated protein RIF1	Q6PR54	S2144	SQEDEISPVNK	S7	1.112	0.299
Rif1	Telomere-associated protein RIF1	Q6PR54	Ambiguous	VIAGSSPEGVETMELNVR	S/T	1.015	0.869
Rilpl1	RILP-like protein 1	Q9JJC6	S280	LQGEHSQNGEEEEAEIQPQPDGEESISDAEK	S27	0.995	0.904
Rilpl1	RILP-like protein 1	Q9JJC6	S349	TSPQPESGIKR	S2	1.087	0.464
Rims3	Regulating synaptic membrane exocytosis protein 3	Q80U57	S22	SSSISGEICGSQQAGGAGTTTAK	S3	0.880	0.364
Rims3	Regulating synaptic membrane exocytosis protein 3	Q80U57	S294	RLSQSSLESATSPSCS	S3	0.893	0.439
Rims3	Regulating synaptic membrane exocytosis protein 3	Q80U57	S294,S296	RLSQSSLESATSPSCS	S3,S5	0.921	0.363
Rims3	Regulating synaptic membrane exocytosis protein 3	Q80U57	Ambiguous	QGSRESTDGSTNSNSSEGTFIFPTR	T/S	0.996	0.981
Rin1	Ras and Rab interactor 1	A0A2C9F2A2	Ambiguous	SMSSAFCSLLAPER	S	1.289	0.511
Rin2	Ras and Rab interactor 2	Q9D684	S79	DSGYDSLSNR	S6	0.971	0.545
Rin3	Ras and Rab interactor 3	P59729	S501	QATGASWEGLSPVR	S11	1.103	0.086
Ring1	E3 ubiquitin-protein ligase RING1	O35730	S38	TPQEAIMDGTEIAVSPR	S15	0.993	0.941
Riok1	Serine/threonine-protein kinase RIO1	Q922Q2	Ambiguous	MASVVPGQFDDADSSDSENK	S	1.073	0.672
Riok3	Serine/threonine-protein kinase RIO3	Q9DBU3	S512	DDGSPPVLSAD	S4	0.856	0.157
Riox1	Ribosomal oxygenase 1	Q9JJF3	S107	SASATLPSR	S3	1.433	0.002
Riox1	Ribosomal oxygenase 1	Q9JJF3	S68	SEDSDSKVAVASVR	S4	0.964	0.407
Ripk1	Receptor-interacting serine/threonine-protein kinase 1	Q60855	S313	EYPDQSPVLQR	S6	1.017	0.719

Ripk1	Receptor-interacting serine/threonine-protein kinase 1	Q60855	S313	KEYPDQSPVLQR	S7	0.980	0.617
Ripk1	Receptor-interacting serine/threonine-protein kinase 1	Q60855	S321	MFSLQHDCVPLPPSR	S3	1.135	0.044
Ripk1	Receptor-interacting serine/threonine-protein kinase 1	Q60855	S415	RVSHDPFAQQR	S3	1.066	0.469
Ripk2	Receptor-interacting serine/threonine-protein kinase 2	P58801	S362	SLSAPQDK	S1	0.975	0.610
Ripk2	Receptor-interacting serine/threonine-protein kinase 2	P58801	S362	NTGSPGPSRSLSAPQDK	S10	0.957	0.572
Ripk2	Receptor-interacting serine/threonine-protein kinase 2	P58801	S356,S362	NTGSPGPSRSLSAPQDK	S4,S10	0.996	0.809
Ripk3	Receptor-interacting serine/threonine-protein kinase 3	Q9QZL0	S326	NLSAREPSQR	S8	0.891	0.122
Ripk3	Receptor-interacting serine/threonine-protein kinase 3	Q9QZL0	S369	QAQDTSVGPATPAR	S6	0.916	0.452
Ripor1	Rho family-interacting cell polarization regulator 1	Q68FE6	S347	FSTYSQSPPDTPSLR	S7	0.995	0.807
Ripor1	Rho family-interacting cell polarization regulator 1	Q68FE6	Ambiguous	RFSTYSQSPPDTPSLR	S/T/Y	0.971	0.770
Rlf	MCG122876	A2A7F4	S1037	EHQGYSPEPSICASK	S6	1.083	0.148
Rlf	MCG122876	A2A7F4	S631	GIYPKSPTGSLEQNPEQAR	S6	0.956	0.354
Rlim	E3 ubiquitin-protein ligase RLIM	Q9WTV7	S163	RLSVENMESSSQR	S3	1.031	0.675
Rlim	E3 ubiquitin-protein ligase RLIM	Q9WTV7	S163	RLSVENMESSSQR	S3	1.021	0.725
Rlim	E3 ubiquitin-protein ligase RLIM	Q9WTV7	S194	AERNSAEAVTEVPTTR	S5	1.091	0.559
Rlim	E3 ubiquitin-protein ligase RLIM	Q9WTV7	S229	SRSPLQPTSEIPR	S3	1.034	0.618
Rmdn3	Regulator of microtubule dynamics protein 3	Q3UJU9	S212	KDSLDLDVEAASSPAAAALEEDDSSGR	S3	0.673	0.270
Rmdn3	Regulator of microtubule dynamics protein 3	Q3UJU9	S46	SHSLPNSLDYAQASER	S3	0.994	0.751
Rmdn3	Regulator of microtubule dynamics protein 3	Q3UJU9	S46,S50	SHSLPNSLDYAQASER	S3,S7	0.938	0.091
Rnaseh1	Ribonuclease H1	E9QLN8	Ambiguous	SSSSPDGSKGQESAHEQK	S	0.888	0.654
Rnaseh2a	Ribonuclease H2 subunit A	Q9CWY8	S258	EAEDVIWEDSEAEEDPERPGK	S10	1.160	0.249
Rnasel	2-5A-dependent ribonuclease	Q05921	S735	LSVPEAVGPGGIQS	S14	1.011	0.936
Rnf10	RING finger protein 10	D3Z1N2	S41	SASAGPAGESKPK	S3	1.020	0.766
Rnf11	RING finger protein 11	Q9QYK7	S14	SPTSDDISLLHESQSDR	S8	0.990	0.871
Rnf111	E3 ubiquitin-protein ligase Arkadia	Q99ML9	T22	SGTPDAPTTQESLK	T3	1.117	0.422
Rnf113a2	Ring finger protein 113A2	Q14B01	S253	YGVYEDENYEVESDDEEIPFK	S13	1.066	0.508
Rnf113a2	Ring finger protein 113A2	Q14B01	S329	KAEGGASSTPEDADGI	S8	0.990	0.920
Rnf113a2	Ring finger protein 113A2	Q14B01	Ambiguous	GAYCDLSSEEEEK	S/Y	1.076	0.298
Rnf121	RING finger protein 121	Q8R1Z9	S28	ELDEVDMSDLSPEEQWR	S11	1.005	0.968
Rnf13	E3 ubiquitin-protein ligase RNF13	O54965	Ambiguous	VVPSQGDSDSDTDSSQEENQVSEHTPLLPPSASAR	S/T	0.862	0.099
Rnf130	E3 ubiquitin-protein ligase RNF130	Q8VEM1	S363	TSGISPLPQDGELTPR	S5	0.838	0.280
Rnf139	E3 ubiquitin-protein ligase RNF139	A0A0R4J0K8	T667	NGGIQHTGAAAAAAEFNDDTD	T20	0.947	0.786
Rnf168	E3 ubiquitin-protein ligase RNF168	E9PYW4	S113	RLSEPGELR	S3	1.097	0.526
Rnf168	E3 ubiquitin-protein ligase RNF168	E9PYW4	Ambiguous	ETDRSDTKSPVLQDTEIEK	T/S	0.954	0.286
Rnf169	E3 ubiquitin-protein ligase RNF169	E9Q7F2	S12	ASSAAAAAALSR	S3	0.954	0.110
Rnf169	E3 ubiquitin-protein ligase RNF169	E9Q7F2	S234	TSLEQCPARLSDSENEEPSR	S11	1.102	0.159
Rnf169	E3 ubiquitin-protein ligase RNF169	E9Q7F2	Ambiguous	AGAPGPASSPALLVLR	S	0.974	0.930
Rnf19b	E3 ubiquitin-protein ligase RNF19B	A2A7Q9	S553	DTASLGAISDSASTR	S4	0.991	0.935
Rnf19b	E3 ubiquitin-protein ligase RNF19B	A2A7Q9	Ambiguous	STSLHAAAPDPK	T/S	1.020	0.848
Rnf20	E3 ubiquitin-protein ligase BRE1A	Q5DTM8	S138	ALVVPEPEPDSDSNQER	S13	1.001	0.981
Rnf20	E3 ubiquitin-protein ligase BRE1A	Q5DTM8	S138	KALVVPEPEPDSDSNQER	S14	1.028	0.660
Rnf213	E3 ubiquitin-protein ligase RNF213	E9Q555	S180	TDAQSNLAPSDLAEVK	S5	1.008	0.951
Rnf213	E3 ubiquitin-protein ligase RNF213	E9Q555	S4511	TQTGHVLGSPQSSGVAEVSDR	S9	0.976	0.769
Rnf213	E3 ubiquitin-protein ligase RNF213	E9Q555	T46	NDNTLVVSSTPEGK	T10	0.883	0.038
Rnf214	RING finger protein 214	Q8BFU3	S36	SDESLPDGLSPK	S10	1.071	0.361
Rnf219	RIKEN cDNA 2610206B13, isoform CRA_b	G3X8V5	S566	SVEVEGPSKSPQGR	S10	0.964	0.409
Rnf25	E3 ubiquitin-protein ligase RNF25	Q9QZR0	S452	EPLGLESEEGS	S7	0.936	0.298
Rnf34	E3 ubiquitin-protein ligase RNF34	Q99KR6	S163	GLGSGDDLDSSSLNSSR	S12	1.105	0.495
Rnf38	E3 ubiquitin-protein ligase RNF38	Q8BI21	S170	AFHPPNVSPR	S8	1.079	0.443
Rnf40	E3 ubiquitin-protein ligase BRE1B	Q3U319	T122	ELSSGTEVPGCQEGLTR	T6	0.798	0.382
Rnh1	Ribonuclease inhibitor	A0A1B0GSG5	S397	ALSQPDTVLR	S3	1.024	0.844
Rnh1	Ribonuclease inhibitor	A0A1B0GSG5	S487	ALEEERPSLR	S8	0.990	0.834
Rnmt	mRNA cap guanine-N7 methyltransferase	Q9D0L8	S100	GDVSEDEPSLGR	S4	0.999	0.971
Rnmt	mRNA cap guanine-N7 methyltransferase	Q9D0L8	S15	ASVASDPESPPGGNEPAAASGQR	S9	1.013	0.578
Rnmt	mRNA cap guanine-N7 methyltransferase	Q9D0L8	S64	EFGEDLVEQNSSYVQDSPSK	S17	0.988	0.886
Rnmt	mRNA cap guanine-N7 methyltransferase	Q9D0L8	T34	LPENTPPCQQVDQPK	T5	0.986	0.801
Rnmt	mRNA cap guanine-N7 methyltransferase	Q9D0L8	S11,S15	ASVASDPESPPGGNEPAAASGQR	S5,S9	1.029	0.382
Rnpc3	RNA-binding region-containing protein 3	Q3UZ01	S21	GGPGSASLSPPRGDR	S9	0.994	0.736
Rnpc3	RNA-binding region-containing protein 3	Q3UZ01	Ambiguous	DLETQPNNEEENSDSPDTGLDSNTGFGK	S/T	1.076	0.264
Rnpep	Aminopeptidase B	Q8VCT3	Y409	VKIEPGVDPDDTYNETPYEK	Y13	0.997	0.979
Rock1	Rho-associated protein kinase 1	P70335	S1328	NPPSGFVRASPR	S10	0.947	0.408
Rock1	Rho-associated protein kinase 1	P70335	S1341	STANQSFR	S6	1.025	0.592
Rock1	Rho-associated protein kinase 1	P70335	Ambiguous	YLPSANASENRSSSNVDK	S/Y	1.015	0.714
Rock2	Rho-associated protein kinase 2 (Fragment)	A0A1Y7VMN0	S1380	VYYDISSAK	S7	0.945	0.672
Rock2	Rho-associated protein kinase 2 (Fragment)	A0A1Y7VMN0	S1431	IQQNQSIR	S6	0.962	0.522
Rock2	Rho-associated protein kinase 2 (Fragment)	A0A1Y7VMN0	S423	ENLLLSDSPPCRENDAIQTR	S6	1.003	0.984
Rpap1	RNA polymerase II-associated protein 1	Q80TE0	T1132	ASDTPSGPPAADTVGVAMR	T4	0.926	0.432
Rpap3	RNA polymerase II-associated protein 3	Q9D706	S429	AVDNPPRGSPK	S9	0.894	0.502
Rpgr	X-linked retinitis pigmentosa GTPase regulator	A0A067XG46	S952	SSSSEILGGNDPTSK	S3	0.901	0.059
Rpgrip1l	Protein fantom (Fragment)	A0A1B0GQY2	T112,S113	IFCTTSLVGIFELMDK	T5,S6	1.172	0.053
Rpl13	60S ribosomal protein L13	P47963	S106	NKSTESLQANVQR	S3	1.047	0.237
Rpl13	60S ribosomal protein L13	P47963	S140	KGDSSAEELK	S5	0.998	0.982
Rpl15	60S ribosomal protein L15	Q9CZM2	S34	QLSALHR	S3	1.090	0.210
Rpl15	60S ribosomal protein L15	Q9CZM2	S97	SLQSVAEER	S1	1.140	0.030
Rpl22l1	60S ribosomal protein L22-like 1	Q9D7S7	Ambiguous	YFQISQDEDGSESED	S/Y	1.038	0.226
Rpl23	60S ribosomal protein L23	P62830	S138	IASNAGSIA	S7	1.036	0.756
Rpl29	60S ribosomal protein L29	P47915	S138	AQASAPAQAPK	S4	1.034	0.476
Rpl3	60S ribosomal protein L3	P27659	S304	NNASTDYDLSDK	S4	1.073	0.182
Rpl30	60S ribosomal protein L30	P62889	S10	SLESINSR	S1	0.927	0.136
Rpl34	60S ribosomal protein L34	Q9D1R9	S12	RLSYNTASNK	S3	1.157	0.414
Rpl37	60S ribosomal protein L37	Q9D823	Ambiguous	AAVAASSSS	S	0.954	0.269
Rplp0	60S acidic ribosomal protein P0	P14869	S304	AEAKEESEESDEDMGFGLFD	S7	0.942	0.290
Rplp0	60S acidic ribosomal protein P0	P14869	S304,S307	AEAKEESEESDEDMGFGLFD	S7,S10	1.125	0.657
Rplp2	60S acidic ribosomal protein P2	P99027	S102	KEESEESDDDMGFGLFD	S4	0.988	0.859
Rplp2	60S acidic ribosomal protein P2	P99027	S102,S105	KEESEESDDDMGFGLFD	S4,S7	1.033	0.651
Rplp2	60S acidic ribosomal protein P2	P99027	S102,S105	KEESEESDDDMGFGLFD	S4,S7	0.947	0.688
Rplp2	60S acidic ribosomal protein P2	P99027	Ambiguous	LASVPAGGAVAVSAAPGSAAPAAGSAPAAAEEK	S	0.836	0.357
Rpp21	Ribonuclease P protein subunit p21	Q8R040	S149	ENIQTQALNTSE	S11	1.042	0.608
Rpp30	Ribonuclease P protein subunit p30	O88796	S251	KPRPSEADDESLPVCK	S5	1.019	0.692
Rprd1b	Regulation of nuclear pre-mRNA domain-containing protein 1B	Q9CSU0	S134	LSMEDSKSPPPK	S8	1.022	0.847
Rprd2	Regulation of nuclear pre-mRNA domain-containing protein 2	Q6NXI6	S392	DVEDMELSDVEDDGSK	S8	1.016	0.793
Rprd2	Regulation of nuclear pre-mRNA domain-containing protein 2	Q6NXI6	S392	DVEDMELSDVEDDGSK	S8	1.051	0.583
Rprd2	Regulation of nuclear pre-mRNA domain-containing protein 2	Q6NXI6	S392	SATPEPVTDNRDVEDMELSDVEDDGSK	S19	0.898	0.507
Rprd2	Regulation of nuclear pre-mRNA domain-containing protein 2	Q6NXI6	S612	SFNYSPSSSTSEVSSTSASK	S5	0.981	0.434
Rprd2	Regulation of nuclear pre-mRNA domain-containing protein 2	Q6NXI6	T742	GPTSTSVDSIDGTPVRDER	T13	1.010	0.728
Rprd2	Regulation of nuclear pre-mRNA domain-containing protein 2	Q6NXI6	S738,T742	GPTSTSVDSIDGTPVRDER	S9,T13	0.931	0.212
Rprd2	Regulation of nuclear pre-mRNA domain-containing protein 2	Q6NXI6	Ambiguous	VSSSCLDLPDSTEEK	S/T	1.090	0.509
Rps10	40S ribosomal protein S10	P63325	S130	SAVPPGADK	S1	1.042	0.518
Rps10	40S ribosomal protein S10	P63325	S146	AEAGAGSATEFQFR	S7	1.018	0.619
Rps10	40S ribosomal protein S10	P63325	S146	KAEAGAGSATEFQFR	S8	1.002	0.975
Rps11	40S ribosomal protein S11	P62281	S67	CPFTGNVSIR	S8	1.038	0.697
Rps13	40S ribosomal protein S13	P62301	Ambiguous	YESSTASALVA	T/S/Y	0.881	0.409
Rps14	40S ribosomal protein S14	P62264	S139	IGRIEDVTPIPSDSTR	S14	0.961	0.532
Rps14	40S ribosomal protein S14	P62264	S139	IEDVTPIPSDSTR	S11	1.037	0.510
Rps14	40S ribosomal protein S14	P62264	Ambiguous	IEDVTPIPSDSTRR	S/T	0.999	0.988
Rps14	40S ribosomal protein S14	P62264	Ambiguous	IGRIEDVTPIPSDSTRR	T/S	0.813	0.044
Rps2	40S ribosomal protein S2	P25444	T293	TQAPAVATT	T9	1.043	0.499
Rps27	40S ribosomal protein S27	Q6ZWU9	S11	DLLHPSPEEEKRK	S6	1.085	0.188
Rps27	40S ribosomal protein S27	Q6ZWU9	S11	DLLHPSPEEEKR	S6	0.911	0.221
Rps27	40S ribosomal protein S27	Q6ZWU9	S11	DLLHPSPEEEK	S6	1.071	0.849
Rps27a	Ubiquitin-40S ribosomal protein S27a	P62983	S57	TLSDYNIQK	S3	1.026	0.715
Rps3	40S ribosomal protein S3	P62908	S104	GLCAIAQAESLR	S10	0.765	0.128
Rps3	40S ribosomal protein S3	P62908	T221	DEILPTTPISEQK	T7	1.004	0.852
Rps3a	40S ribosomal protein S3a	P97351	S237	LMELHGEGGSSGK	S10	0.996	0.961
Rps3a	40S ribosomal protein S3a	P97351	S263	ADGYEPPVQESV	S11	1.022	0.514
Rps3a	40S ribosomal protein S3a	P97351	Ambiguous	LMELHGEGGSSGKAAGDETGAK	S/T	0.947	0.507
Rps6	40S ribosomal protein S6	P62754	S235,S240	RLSSLRASTSK	S/T	1.013	0.891
Rps6	40S ribosomal protein S6	P62754	S236,S240	LSSLRASTSK	S3,S7	1.214	0.108
Rps6ka1	Ribosomal protein S6 kinase alpha-1 (Fragment)	F6Q8A4; P18654	S227;S227	AYSFCGTVEYMAPEVVNR	S3	0.743	0.240
Rps6ka1	Ribosomal protein S6 kinase alpha-1 (Fragment)	F6Q8A4; P18654	S227;S227	AYSFCGTVEYMAPEVVNR	S3	1.123	0.659
Rps6ka1	Ribosomal protein S6 kinase alpha-1 (Fragment)	F6Q8A4	S369	DSPGIPPSAGAHQLFR	S2	0.952	0.534
Rps6ka1	Ribosomal protein S6 kinase alpha-1 (Fragment)	F6Q8A4	S369	TPRDSPGIPPSAGAHQLFR	S5	0.941	0.649
Rps6ka3	Ribosomal protein S6 kinase alpha-3	P18654	S369	DSPGIPPSANAHQLFR	S2	0.996	0.871
Rps6ka3	Ribosomal protein S6 kinase alpha-3	P18654	S415	NSIQFTDGYEVK	S2	1.180	0.696
Rps6ka3	Ribosomal protein S6 kinase alpha-3	P18654	S715	NQSPVLEPVGR	S3	1.037	0.356
Rps6ka4	Ribosomal protein S6 kinase alpha-4	Q9Z2B9	S343	LEPVYSPAGSPPPGDPR	S6	1.070	0.556
Rps6ka4	Ribosomal protein S6 kinase alpha-4	Q9Z2B9	S771	ANGPLSPS	S6	1.212	0.148
Rps6ka4	Ribosomal protein S6 kinase alpha-4	Q9Z2B9	T687	SSPPLRTPDVLESSGPAVR	T7	0.992	0.896
Rps6ka4	Ribosomal protein S6 kinase alpha-4	Q9Z2B9	S343,S347	LEPVYSPAGSPPPGDPR	S6,S10	1.219	0.062
Rps6ka4	Ribosomal protein S6 kinase alpha-4	Q9Z2B9	S682,T687	SSPPLRTPDVLESSGPAVR	S2,T7	1.219	0.186
Rps6kc1	Ribosomal protein S6 kinase delta-1	E9QMX4	S301	AESICSLR	S3	1.116	0.050
Rps6kc1	Ribosomal protein S6 kinase delta-1	E9QMX4	S426	SSQESLDIK	S5	1.004	0.872
Rps6kc1	Ribosomal protein S6 kinase delta-1	E9QMX4	S448	VCLQQPSASPQGGSSFESR	S9	0.992	0.876
Rps6kc1	Ribosomal protein S6 kinase delta-1	E9QMX4	S493	TSLTPSSQDDSNQEDDGQPSSPK	S21	0.945	0.548
Rps6kc1	Ribosomal protein S6 kinase delta-1	E9QMX4	S527	MDLVSLSEESVMQPEGDK	S5	1.004	0.984
Rps6kc1	Ribosomal protein S6 kinase delta-1	E9QMX4	S634	ALFTLEDGDSPSQSLDPGESKR	S10	0.944	0.261
Rps6kc1	Ribosomal protein S6 kinase delta-1	E9QMX4	S779	SSPDQFLSSSLR	S2	1.087	0.666
Rps6kc1	Ribosomal protein S6 kinase delta-1	E9QMX4	S435,S	SSQESLDIKEGRPSMPPR	S5,S14	0.981	0.732

Rps6kc1	Ribosomal protein S6 kinase delta-1	E9QMX4	S492,S493	TSLTPSSQDDSNQEDDGQPSSPK	S20,S21	0.717	0.004
Rps6kc1	Ribosomal protein S6 kinase delta-1	E9QMX4	Ambiguous	GVDLLLEGVQGESSPTR	S/T	1.104	0.463
Rps6kc1	Ribosomal protein S6 kinase delta-1	E9QMX4	Ambiguous	VFSGGEDLEAVSSPPTSESLSR	S/T	0.898	0.701
Rps6kc1	Ribosomal protein S6 kinase delta-1	E9QMX4	Ambiguous	ALFTLEDGDSPSQSLDPGESK	S/T	0.791	0.167
Rps6kc1	Ribosomal protein S6 kinase delta-1	E9QMX4	Ambiguous	GVDLLLEGVQGESSPTRR	S/T	0.993	0.962
Rps6kc1	Ribosomal protein S6 kinase delta-1	E9QMX4	Ambiguous	ESEAQDSVSRGSDDSVPVISFK	S	1.135	0.020
Rptor	Regulatory-associated protein of mTOR	A2ACM0	S721	SVSSYGNIR	S3	0.982	0.817
Rptor	Regulatory-associated protein of mTOR	A2ACM0	S877	GMHMHQVGGSPPASSTSSCSLTNDVAK	S10	0.948	0.373
Rptor	Regulatory-associated protein of mTOR	A2ACM0	S855,S863	ILDTSSLTQSAPASPTNK	S6,S14	1.041	0.446
Rragc	Ras-related GTP-binding protein C	Q99K70	S381	SCSHQTSAPSLK	S7	1.001	0.997
Rragc	Ras-related GTP-binding protein C	Q99K70	S94	MSPNETLFLESTNK	S11	0.894	0.570
Rragc	Ras-related GTP-binding protein C	Q99K70	S94	MSPNETLFLESTNK	S11	1.044	0.722
Rragd	Ras-related GTP-binding protein D	B1AWT3	Ambiguous	MSPSETLFLESTNR	T/S	0.911	0.608
Rras	Ras-related protein R-Ras	P10833	S201	KYQEQELPPSPPSAPR	S10	1.024	0.580
Rras	Ras-related protein R-Ras	P10833	S201	YQEQELPPSPPSAPR	S9	1.088	0.597
Rras2	Ras-related protein R-Ras2	P62071	S186	KFQEQECPPSPEPTR	S10	1.060	0.556
Rras2	Ras-related protein R-Ras2	P62071	S186	KFQEQECPPSPEPTRK	S10	1.029	0.649
Rras2	Ras-related protein R-Ras2	P62071	S186	FQEQECPPSPEPTR	S9	1.235	0.013
Rras2	Ras-related protein R-Ras2	P62071	S186	FQEQECPPSPEPTRK	S9	1.135	0.098
Rrbp1	Ribosome-binding protein 1	Q99PL5	S1472	SHVEDGDVAGSPAVPPAEQDPMK	S11	1.063	0.643
Rrbp1	Ribosome-binding protein 1	Q99PL5	S1472	SHVEDGDVAGSPAVPPAEQDPMK	S11	1.046	0.448
Rrbp1	Ribosome-binding protein 1	Q99PL5	S1604	AEDGSSSKEGTSV	S12	0.981	0.831
Rrbp1	Ribosome-binding protein 1	Q99PL5	S766	SEGSPNQGK	S4	1.014	0.870
Rrbp1	Ribosome-binding protein 1	Q99PL5	Ambiguous	LASSPKDR	S	1.106	0.453
Rrbp1	Ribosome-binding protein 1	Q99PL5	Ambiguous	ETSYEEALANQR	T/S/Y	1.110	0.235
Rreb1	Ras-responsive element-binding protein 1	Q3UH06	S1097	VADPGPSITSSNTVATDSPGSSIPK	S/T	1.254	0.253
Rreb1	Ras-responsive element-binding protein 1	Q3UH06	S1138	ESSEPPPAASSPEEALPTEQGPAATSSSR	S11	0.989	0.910
Rreb1	Ras-responsive element-binding protein 1	Q3UH06	S1309	EQPPAEGASEISPASQDLAIK	S12	1.015	0.808
Rreb1	Ras-responsive element-binding protein 1	Q3UH06	S1452	AVEGPSPSPEPEEKPAESLAIDPTPGTR	S8	1.027	0.685
Rreb1	Ras-responsive element-binding protein 1	Q3UH06	S175	KLSHDAESEDPGPAK	S3	1.301	0.018
Rreb1	Ras-responsive element-binding protein 1	Q3UH06	T1119	AAATPTDTTSSK	T4	1.100	0.571
Rreb1	Ras-responsive element-binding protein 1	Q3UH06	S1450,S1452	AVEGPSPSPEPEEKPAESLAIDPTPGTR	S6,S8	1.137	0.288
Rrp1	Ribosomal RNA processing protein 1 homolog A	P56183	S434	EAGSEAESSSADPGPGR	S4	1.015	0.904
Rrp1	Ribosomal RNA processing protein 1 homolog A	P56183	S434	EAGSEAESSSADPGPGRK	S4	1.237	0.038
Rrp12	RRP12-like protein	Q6P5B0	S1081	GDSIEEILADSEDEDEEEER	S11	1.206	0.003
Rrp12	RRP12-like protein	Q6P5B0	S1081	GDSIEEILADSEDEDEEEERGR	S11	0.965	0.622
Rrp12	RRP12-like protein	Q6P5B0	T88	SQAPETTMDQDPEVAFTEK	T17	0.990	0.967
Rrp15	RRP15-like protein	Q9CYX7	S242	SEESPGWK	S4	1.039	0.036
Rrp15	RRP15-like protein	Q9CYX7	S265	DWDKESEGEEPAGGR	S6	1.074	0.420
Rrp1b	Ribosomal RNA processing protein 1 homolog B	Q91YK2	S405	VPDSESEPDTAQR	S4	1.018	0.785
Rrp1b	Ribosomal RNA processing protein 1 homolog B	Q91YK2	S528	SGTPTSPVEGK	S6	1.117	0.073
Rrp1b	Ribosomal RNA processing protein 1 homolog B	Q91YK2	S705	TATSSPASTPLSPMR	S12	1.024	0.879
Rrp36	Ribosomal RNA processing protein 36 homolog	Q3UFY0	S66	TRSPQQPVCVADK	S3	0.999	0.991
Rrp8	Ribosomal RNA-processing protein 8	E9PVA2	S151	QAPSISDSEGKEIR	S6	1.004	0.956
Rrp8	Ribosomal RNA-processing protein 8	E9PVA2	S151	QAPSISDSEGK	S6	1.046	0.222
Rrp8	Ribosomal RNA-processing protein 8	E9PVA2	S223	TSTATIDPSKPSPESMSPNSSHTLSR	S17	0.966	0.834
Rrp9	U3 small nucleolar RNA-interacting protein 2	Q91WM3	S470	LPVSPVAGS	S4	1.024	0.791
Rrs1	Ribosome biogenesis regulatory protein homolog	Q9CYH6	S56	QAGPSPEAELR	S5	1.002	0.944
Rsbn1	Lysine-specific demethylase 9	Q80T69	S199	GSDGDPGACVPGDLK	S2	1.019	0.866
Rsbn1	Lysine-specific demethylase 9	Q80T69	T793	LDSDQQHSLQAHPSTPV	T15	1.110	0.443
Rsf1	Remodeling and spacing factor 1	E9PWW9	S1286	STDDYSEADEDDEEEGKPSR	S6	0.803	0.187
Rsf1	Remodeling and spacing factor 1	E9PWW9	S1286	STDDYSEADEDDEEEGKPSRK	S6	0.758	0.001
Rsf1	Remodeling and spacing factor 1	E9PWW9	S1347	IESDEEEDFENK	S3	1.032	0.681
Rsf1	Remodeling and spacing factor 1	E9PWW9	S668	GSSESQSLENASPEILKEDSESSREEVAK	S12	0.990	0.813
Rsf1	Remodeling and spacing factor 1	E9PWW9	S668	GSSESQSLENASPEILKEDSESSR	S12	0.970	0.869
Rsf1	Remodeling and spacing factor 1	E9PWW9	S668	GSSESQSLENASPEILK	S12	1.007	0.958
Rsf1	Remodeling and spacing factor 1	E9PWW9	T1308	LHRIETDEESCDNAHGDADQPAR	T6	1.316	0.019
Rsf1	Remodeling and spacing factor 1	E9PWW9	T1308,S1312	LHRIETDEESCDNAHGDADQPAR	T6,S10	1.036	0.796
Rsl1d1	Ribosomal L1 domain-containing protein 1	Q8BVY0	T429	KPEASSFSTPR	T9	1.112	0.246
Rsrc2	Arginine/serine-rich coiled-coil protein 2	S4R2L4	S103	LNSSENGEDR	S3	1.010	0.881
Rsrc2	Arginine/serine-rich coiled-coil protein 2	S4R2L4	S32	KEQSDISISPR	S9	0.948	0.298
Rsrc2	Arginine/serine-rich coiled-coil protein 2	S4R2L4	Ambiguous	EQSDISISPR	S	1.077	0.057
Rsrc2	Arginine/serine-rich coiled-coil protein 2	S4R2L4	Ambiguous	DGLALEKTSPDREK	T/S	0.941	0.123
Rsrp1	Arginine/serine-rich protein 1	Q3UC65	S17	YVNDMWPGSPQEK	S9	0.944	0.423
Rtf1	RNA polymerase-associated protein RTF1 homolog	A2AQ19	Ambiguous	TSEVYSDDEEEEDDDKSSEK	S/Y/T	1.016	0.879
Rtkn	Rhotekin	Q8C6B2	S520	TFSLDAAPADHSLGPSR	S3	0.955	0.210
Rtn4	Reticulon-4	Q99P72	S105	GPLPAAPPTAPERQPSWER	S16	1.178	0.013
Rtn4	Reticulon-4	Q99P72	S145	LPEDDEPPARPPAPAGASPLAEPAAPPSTPAAPK	S18	1.203	0.407
Rtn4	Reticulon-4	Q99P72	S408	CFEDSLEQK	S5	1.011	0.922
Rtn4	Reticulon-4	Q99P72	S792	DAASNEIPTLTK	S4	1.119	0.324
Rtn4	Reticulon-4	Q99P72	S857	DDSPKEYTDLEVSNK	S3	1.006	0.963
Rtn4	Reticulon-4	Q99P72	S953	EAEEKLPSDTEKEDR	S8	1.183	0.106
Rtn4	Reticulon-4	Q99P72	S690,T/S/Y	ETKLSTEPSPEFSNYSEIAK	S9,T/S/Y	1.127	0.645
Rubcn	Run domain Beclin-1-interacting and cysteine-rich domain-containing protein	D3Z7B1	S328	SSFSEGQTAPVASGTK	S1	0.992	0.856
Rubcn	Run domain Beclin-1-interacting and cysteine-rich domain-containing protein	D3Z7B1	S329	RSSFSEGQTAPVASGTK	S3	1.001	0.956
Rubcn	Run domain Beclin-1-interacting and cysteine-rich domain-containing protein	D3Z7B1	S351	SHSDTNIASR	S3	1.026	0.808
Rubcn	Run domain Beclin-1-interacting and cysteine-rich domain-containing protein	D3Z7B1	S369	GAAEGGQYLCSGEGMFR	S11	0.975	0.800
Rubcn	Run domain Beclin-1-interacting and cysteine-rich domain-containing protein	D3Z7B1	S437	CNMMSQCLEEEEVEEEDSDREIQELK	S18	0.994	0.863
Rubcn	Run domain Beclin-1-interacting and cysteine-rich domain-containing protein	D3Z7B1	S437	CNMMSQCLEEEEVEEEDSDREIQELK	S18	0.976	0.877
Rubcn	Run domain Beclin-1-interacting and cysteine-rich domain-containing protein	D3Z7B1	Ambiguous	DSTQLSESGSAEDADDLEIQDADIR	S/T	1.285	0.320
Rubcnl	Protein RUBCNL-like	F8WGX2	S173	TVATSPSPK	S7	0.959	0.639
Rubcnl	Protein RUBCNL-like	F8WGX2	S183	SESPHLTASTDDGDAR	S3	0.891	0.591
Rufy1	RUN and FYVE domain-containing protein 1	Q8BIJ7	S52	SQLPSPGELR	S5	1.010	0.564
Rufy3	Protein RUFY3	A0A0G2JFT8	S27	RPSEPLEPR	S3	1.079	0.131
Runx1	Runt-related transcription factor	Q3UM65	S226	VSPHHPAPTPNPR	S2	1.045	0.853
Rusc1	RUN and SH3 domain-containing protein 1	Q8BG26	T817	RGTPPETPPEALVSSPGSVVQADR	T3	1.084	0.208
Rusc1	RUN and SH3 domain-containing protein 1	Q8BG26	T821	GTPPETPPEALVSSPGSVVQADR	T6	0.972	0.876
Rusc2	Iporin	Q3V1Z0	S542	LSEGPAALAGPASPPRR	S13	1.035	0.530
Rusc2	Iporin	Q3V1Z0	S542	LSEGPAALAGPASPPR	S13	0.998	0.978
Rusc2	Iporin	Q3V1Z0	S565	AAGSGSPPLR	S6	1.025	0.545
Rusc2	Iporin	Q3V1Z0	S580	ASVGDSSQEFSPIQEAQQDR	S11	1.059	0.422
Rusc2	Iporin	Q3V1Z0	S785	NAGPEPETSRPSPLGSYSPVR	S18	1.142	0.049
Rusc2	Iporin	Q3V1Z0	S779,S/T/Y	NAGPEPETSRPSPLGSYSPVR	S12,S/T/Y	1.331	0.140
Rxrb	Retinoic acid receptor RXR-beta	Q3TWJ1	Ambiguous	SDQGVEGPGATGGGGSSPNDPVTNICQAADK	T/S	0.938	0.372
Rxylt1	Ribitol-5-phosphate xylosyltransferase 1	Q8VDX6	S64	EQSALESEEWNPWEGDEK	S7	1.202	0.221
Rybp	RING1 and YY1-binding protein	Q8CCI5	S227	GDMSAVNDESF	S10	1.014	0.881
Rybp	RING1 and YY1-binding protein	Q8CCI5	T215	SSTPKGDMSAVNDESF	T3	1.062	0.203
Rybp	RING1 and YY1-binding protein	Q8CCI5	Ambiguous	SSSTSSSTVTSSAGSEQQNQSSSGSESTDK	S/T	1.308	0.189
Rybp	RING1 and YY1-binding protein	Q8CCI5	Ambiguous	SSTPKGDMSAVNDESF	T/S	0.984	0.771
Ryr2	Ryanodine receptor 2	E9Q401	S3961,S3962	LSQDSSQIELLK	S5,S6	1.172	0.148
Ryr2	Ryanodine receptor 2	E9Q401	Ambiguous	IAEDPSRDGPSPTSGSSK	S/T	0.950	0.659
S1pr2	Sphingosine 1-phosphate receptor 2	P52592	S329,S	LLPLRSSSSLER	S9,S	1.155	0.074
S1pr2	Sphingosine 1-phosphate receptor 2	P52592	Ambiguous	GMHMPTSPTFLEGNTVV	T/S	0.781	0.166
Sacs	Sacsin	E9QNY8	Ambiguous	DSAPTTPTSPTEFLTPGLR	S/T	1.137	0.196
Safb	Scaffold attachment factor B1	S4R1M2; Q80YR5	S235;S229	SEPVKEEGSELEQPFAQATSSVGPDR	S9	1.020	0.705
Safb	Scaffold attachment factor B1	S4R1M2; Q80YR5	S366;S387	APTAALSPEPQDSKEDVK	S7	0.915	0.125
Safb	Scaffold attachment factor B1	S4R1M2; Q80YR5	S366;S387	APTAALSPEPQDSK	S7	1.031	0.528
Safb	Scaffold attachment factor B1	S4R1M2; Q80YR5	S465;S488	VVTNARSPGAR	S7	0.963	0.502
Safb	Scaffold attachment factor B1	S4R1M2	S623	SVVSFDK	S1	1.007	0.882
Safb	Scaffold attachment factor B1	S4R1M2; Q80YR5	Ambiguous	SEPVKEEGSELEQPFAQATSSVGPDRK	T/S	0.910	0.369
Safb2	Scaffold attachment factor B2	Q80YR5	S95	MEEEGSEDNGLEEDSR	S6	0.896	0.272
Safb2	Scaffold attachment factor B2	Q80YR5	S95	MEEEGSEDNGLEEDSR	S6	1.095	0.567
Safb2	Scaffold attachment factor B2	Q80YR5	S95	GSKMEEEGSEDNGLEEDSR	S9	0.971	0.520
Safb2	Scaffold attachment factor B2	Q80YR5	S95	GSKMEEEGSEDNGLEEDSR	S9	0.964	0.655
Samd1	Atherin	D3YXK1	T107	RGATPPAPPRVPR	T4	0.963	0.642
Samd1	Atherin	D3YXK1	T146	AAAATAPPSPGPAQPGPR	T5	1.032	0.672
Samd4b	Protein Smaug homolog 2	G5E8A7	S271	AAFTTPDHAPLSPQSSVASSGSEQTEEQGSSR	S12	1.035	0.613
Samd4b	Protein Smaug homolog 2	G5E8A7	T599	HALTSPSLGGQGR	T4	0.825	0.106
Samd4b	Protein Smaug homolog 2	G5E8A7	S271,S/T	AAFTTPDHAPLSPQSSVASSGSEQTEEQGSSR	S12,S/T	0.763	0.047
Samd8	Sphingomyelin synthase-related protein 1	Q9DA37	Ambiguous	TPDSNGDLDTGSELGPGSPAPTAEEVEK	S/T	1.482	0.107
Samhd1	Deoxynucleoside triphosphate triphosphohydrolase SAMHD1 (Fragment)	F6TVP2	T290	EQIMGPPITPVK	T9	1.008	0.769
Samhd1	Deoxynucleoside triphosphate triphosphohydrolase SAMHD1 (Fragment)	F6TVP2	T32	TPPSTPPATANLSADDDFQNTDLR	T1	1.045	0.688
Samhd1	Deoxynucleoside triphosphate triphosphohydrolase SAMHD1 (Fragment)	F6TVP2	T32	CDGSPRTPPSTPPATANLSADDDFQNTDLR	T7	1.001	0.988
Samhd1	Deoxynucleoside triphosphate triphosphohydrolase SAMHD1 (Fragment)	F6TVP2	S29,T32	CDGSPRTPPSTPPATANLSADDDFQNTDLR	S4,T7	0.986	0.806
Samhd1	Deoxynucleoside triphosphate triphosphohydrolase SAMHD1 (Fragment)	F6TVP2	T32,S/T	TPPSTPPATANLSADDDFQNTDLR	T1,S/T	0.747	0.522
Samsn1	SAM domain-containing protein SAMSN-1	P57725	S125	ASDSMDSLYSGQSSSSGITSCSDGTSNR	S4	1.115	0.066
Samsn1	SAM domain-containing protein SAMSN-1	P57725	S125	ASDSMDSLYSGQSSSSGITSCSDGTSNR	S4	1.035	0.403
Samsn1	SAM domain-containing protein SAMSN-1	P57725	S23	SSSFGNFDR	S3	1.055	0.139
Samsn1	SAM domain-containing protein SAMSN-1	P57725	S23	RSSSFGNFDR	S4	1.031	0.537
Samsn1	SAM domain-containing protein SAMSN-1	P57725	S371	IAITESSD	S7	0.967	0.595
Samsn1	SAM domain-containing protein SAMSN-1	P57725	S97	ALSEEKEEESGEEALPYR	S10	1.104	0.747
Samsn1	SAM domain-containing protein SAMSN-1	P57725	Ambiguous	SSSFGNFDRFR	S	0.934	0.465
Samsn1	SAM domain-containing protein SAMSN-1	P57725	Ambiguous	ALSEEKEEESGEEALPYRNSDPMIGTHTEK	Y/S/T	0.997	0.941
Sap130	Histone deacetylase complex subunit SAP130	J3QNK5	Ambiguous	VVPQQITHTSPR	T/S	1.087	0.098
Sap30bp	SAP30-binding protein	Q02614	S113	NMSPDEIKIPPEPPGR	S3	0.998	0.970
Sar1a	GTP-binding protein SAR1a	P36536	T139	IDRTDAISEEK	T4	1.107	0.145
Saraf	Store-operated calcium entry-associated regulatory factor	A0A0R4J0D1	S332	AYSPLGGGAGSYCASSNADSR	S3	0.953	0.552
Sars	Serine--tRNA ligase, cytoplasmic	Q8C483	T534	EVPLENQLQSMEVTEA	T14	1.487	0.158

Sart1	U4/U6.U5 tri-snRNP-associated protein 1	Q9Z315	T430	ADDLLPLGDQTQDGDFGSR	T11	0.824	0.424
Sart1	U4/U6.U5 tri-snRNP-associated protein 1	Q9Z315	Ambiguous	SANGGSESDGEENIGWSTVNLDEEK	S/T	1.123	0.698
Sash1	SAM and SH3 domain-containing protein 1	F8VQK5	S1014	ASPASPVSPSDCPSPR	S8	1.044	0.361
Sash1	SAM and SH3 domain-containing protein 1	F8VQK5	S1014	KASPASPVSPSDCPSPR	S9	0.988	0.807
Sash1	SAM and SH3 domain-containing protein 1	F8VQK5	S130	SNSEDGCVGK	S3	0.976	0.569
Sash1	SAM and SH3 domain-containing protein 1	F8VQK5	S241	EQSDDETEDSVK	S3	1.041	0.319
Sash1	SAM and SH3 domain-containing protein 1	F8VQK5	S380	SLTEGEMK	S1	0.985	0.653
Sash1	SAM and SH3 domain-containing protein 1	F8VQK5	S380	SLTEGEMK	S1	1.002	0.948
Sash1	SAM and SH3 domain-containing protein 1	F8VQK5	S392	GLGSLSHGR	S4	0.975	0.881
Sash1	SAM and SH3 domain-containing protein 1	F8VQK5	S400	TCSFGGFDLTNR	S3	1.006	0.893
Sash1	SAM and SH3 domain-containing protein 1	F8VQK5	S435	EVIKSPPAPR	S5	1.038	0.309
Sash1	SAM and SH3 domain-containing protein 1	F8VQK5	S500	AGGSVESLR	S4	1.025	0.272
Sash1	SAM and SH3 domain-containing protein 1	F8VQK5	S500	LKAGGSVESLR	S6	0.979	0.682
Sash1	SAM and SH3 domain-containing protein 1	F8VQK5	S534	ESVKSEDGDDEEPPYR	S5	1.045	0.622
Sash1	SAM and SH3 domain-containing protein 1	F8VQK5	S534	ESVKSEDGDDEEPPYRGPFCGR	S5	1.014	0.839
Sash1	SAM and SH3 domain-containing protein 1	F8VQK5	S714	LLVDNQGLSGRSPR	S12	0.936	0.281
Sash1	SAM and SH3 domain-containing protein 1	F8VQK5	S718	SPRDSGCYESSENLENAK	S5	1.017	0.881
Sash1	SAM and SH3 domain-containing protein 1	F8VQK5	S723	DSGCYESSENLENAK	S7	0.980	0.666
Sash1	SAM and SH3 domain-containing protein 1	F8VQK5	S795	SCDVPSVTDLSK	S6	1.004	0.964
Sash1	SAM and SH3 domain-containing protein 1	F8VQK5	S805	SLPVSICR	S1	0.934	0.207
Sash1	SAM and SH3 domain-containing protein 1	F8VQK5	S831	SHSLDDLQGDADVGK	S3	0.993	0.880
Sash1	SAM and SH3 domain-containing protein 1	F8VQK5	S892	RFSDPPK	S3	1.015	0.878
Sash1	SAM and SH3 domain-containing protein 1	F8VQK5	S937	TSLEGLR	S2	0.891	0.123
Sash1	SAM and SH3 domain-containing protein 1	F8VQK5	S1011,S1014	ASPASPVSPSDCPSPR	S5,S8	1.007	0.418
Sash1	SAM and SH3 domain-containing protein 1	F8VQK5	S1011,S1014	KASPASPVSPSDCPSPR	S6,S9	0.907	0.361
Sash1	SAM and SH3 domain-containing protein 1	F8VQK5	S112,S/T	SQIEESLGFCSAVSTPEVER	S11,S/T	1.017	0.888
Sash1	SAM and SH3 domain-containing protein 1	F8VQK5	S718,S723	SPRDSGCYESSENLENAK	S5,S10	0.937	0.387
Sash1	SAM and SH3 domain-containing protein 1	F8VQK5	Ambiguous	SSLSGQSSMSGQTVSTTDSSTSNR	S/T	1.212	0.078
Sash1	SAM and SH3 domain-containing protein 1	F8VQK5	Ambiguous	QTSKGEDVGYVASEITMSDEER	T/S/Y	1.162	0.001
Sash1	SAM and SH3 domain-containing protein 1	F8VQK5	Ambiguous	QTSKGEDVGYVASEITMSDEER	T/S/Y	1.222	0.004
Sash1	SAM and SH3 domain-containing protein 1	F8VQK5	Ambiguous	KPSTEGGEEHVFENSPVQDER	T/S	1.136	0.107
Sash3	SAM and SH3 domain-containing protein 3	Q8K352	S120	ALSEEMGDTLEEGSASPTSPDCSLDSPGPEK	S26	1.069	0.091
Sash3	SAM and SH3 domain-containing protein 3	Q8K352	T61	EFNLDDNIPEDDSGVLTPEDSGK	T17	0.728	0.173
Sash3	SAM and SH3 domain-containing protein 3	Q8K352	Ambiguous	QTSTGSELCSPGPGSGSFLEESPAPQYTGPFCGR	T/S/Y	0.915	0.659
Sash3	SAM and SH3 domain-containing protein 3	Q8K352	Ambiguous	MALAFTEQEEREPPSLSR	S/T	0.966	0.788
Sash3	SAM and SH3 domain-containing protein 3	Q8K352	Ambiguous	ALSEEMGDTLEEGSASPTSPDCSLDSPGPEK	S/T	1.286	0.058
Sash3	SAM and SH3 domain-containing protein 3	Q8K352	Ambiguous	ALSEEMGDTLEEGSASPTSPDCSLDSPGPEK	S/T	1.338	0.026
Satb2	DNA-binding protein SATB2	Q8VI24	Ambiguous	ESSPPREEAPPPPPPTEDSCAK	S/T	1.176	0.227
Sav1	Protein salvador homolog 1	Q8VEB2	S95	ESHRLSAPSYLVR	S6	0.959	0.648
Sbf1	Myotubularin-related protein 5	Q6ZPE2	Ambiguous	ALYLEPSDGVSPTQETGEAQSQDDER	S/T/Y	0.942	0.754
Sbf1	Myotubularin-related protein 5	Q6ZPE2	Ambiguous	STSTLYSQFQTAESENR	T/S/Y	1.071	0.290
Sbf1	Myotubularin-related protein 5	Q6ZPE2	Ambiguous	AQNTPSPGQAQADSSSLEQEK	T/S	0.954	0.479
Sbf2	SET-binding factor 2	E9PXF8	S1212	SQNSPQAVSTSSLESSSSIEQEK	S4	0.998	0.984
Sbf2	SET-binding factor 2	E9PXF8	S1710	HSAGSPGIASTNVPSYQK	S5	1.131	0.455
Sbf2	SET-binding factor 2	E9PXF8	T1123	LGLGTISGNSSR	T5	0.944	0.437
Sbf2	SET-binding factor 2	E9PXF8	Ambiguous	ASEKSTMEQLVEK	S/T	1.020	0.859
Scaf1	Splicing factor, arginine/serine-rich 19	Q5U4C3	S510	QRSGDPAPPDSPSWEAK	S3	0.919	0.161
Scaf1	Splicing factor, arginine/serine-rich 19	Q5U4C3	S518	SGDPAPPDSPSWEAK	S9	0.984	0.223
Scaf1	Splicing factor, arginine/serine-rich 19	Q5U4C3	S682	APSPAPAVSPK	S9	0.920	0.266
Scaf1	Splicing factor, arginine/serine-rich 19	Q5U4C3	S691	REVLYDSEGLSADER	S7	0.989	0.781
Scaf1	Splicing factor, arginine/serine-rich 19	Q5U4C3	S821	SPFLKPDER	S1	0.984	0.830
Scaf1	Splicing factor, arginine/serine-rich 19	Q5U4C3	S821	DRESRSPFLKPDER	S6	0.922	0.244
Scaf1	Splicing factor, arginine/serine-rich 19	Q5U4C3	S912	GTEETSWSGEER	S8	0.990	0.604
Scaf1	Splicing factor, arginine/serine-rich 19	Q5U4C3	S922	APSTPPPK	T4	1.021	0.572
Scaf1	Splicing factor, arginine/serine-rich 19	Q5U4C3	T328	EPPPPQTLGAPGTPPQADSTR	T13	1.014	0.825
Scaf1	Splicing factor, arginine/serine-rich 19	Q5U4C3	T328	REPPPPQTLGAPGTPPQADSTR	T14	1.186	0.136
Scaf1	Splicing factor, arginine/serine-rich 19	Q5U4C3	T936	VAPPPPALTPDSQTVDSSCK	T9	0.891	0.385
Scaf1	Splicing factor, arginine/serine-rich 19	Q5U4C3	S491,S493	QRSASPGPPPARK	S3,S5	1.138	0.470
Scaf1	Splicing factor, arginine/serine-rich 19	Q5U4C3	S491,S493	QRSASPGPPPAR	S3,S5	0.995	0.968
Scaf1	Splicing factor, arginine/serine-rich 19	Q5U4C3	S510,S518	SGDPAPPDSPSWEAKK	S1,S9	1.031	0.557
Scaf1	Splicing factor, arginine/serine-rich 19	Q5U4C3	S510,S518	QRSGDPAPPDSPSWEAK	S3,S11	1.063	0.396
Scaf1	Splicing factor, arginine/serine-rich 19	Q5U4C3	S510,S518	ERQRSGDPAPPDSPSWEAK	S5,S13	1.032	0.799
Scaf1	Splicing factor, arginine/serine-rich 19	Q5U4C3	S510,S518	SGDPAPPDSPSWEAK	S1,S9	0.924	0.507
Scaf1	Splicing factor, arginine/serine-rich 19	Q5U4C3	S577,S579	SASPPPAASSSSSSR	S1,S3	1.092	0.435
Scaf1	Splicing factor, arginine/serine-rich 19	Q5U4C3	S676,S682	APSPAPAVSPK	S3,S9	1.001	0.985
Scaf1	Splicing factor, arginine/serine-rich 19	Q5U4C3	S691,S695	REVLYDSEGLSADERGGK	S7,S11	0.848	0.101
Scaf1	Splicing factor, arginine/serine-rich 19	Q5U4C3	Ambiguous	DSTPGSGPLPK	S/T	0.997	0.976
Scaf1	Splicing factor, arginine/serine-rich 19	Q5U4C3	Ambiguous	SGGSSTASGGPGSLK	S/T	0.811	0.084
Scaf1	Splicing factor, arginine/serine-rich 19	Q5U4C3	Ambiguous	FDIYDPFHPTDEAYSPPPAPEQK	Y/S/T	0.853	0.220
Scaf11	SR-related CTD-associated factor 11	E9PZM7	S357	SPVSNYSECDAPGNSNSSVSISPPAESEK	S22	0.976	0.643
Scaf11	SR-related CTD-associated factor 11	E9PZM7	S402	SSSDSVEEETVDSDTPPVLEK	S5	1.085	0.398
Scaf11	SR-related CTD-associated factor 11	E9PZM7	S498	EFCADHDISPDTSLR	S9	0.993	0.790
Scaf11	SR-related CTD-associated factor 11	E9PZM7	S609	TGESLTVESTDLNDSAVNIEAFVESPK	S25	0.945	0.768
Scaf11	SR-related CTD-associated factor 11	E9PZM7	S857	RETVVESQSSQSPSPK	S12	0.904	0.231
Scaf11	SR-related CTD-associated factor 11	E9PZM7	S857	ETVVESQSSQSPSPKR	S11	1.034	0.440
Scaf11	SR-related CTD-associated factor 11	E9PZM7	S859	ETVVESQSSQSPSPK	S13	1.027	0.432
Scaf11	SR-related CTD-associated factor 11	E9PZM7	T1456	ALEEPGSTEKT	T11	1.046	0.258
Scaf11	SR-related CTD-associated factor 11	E9PZM7	S402,T/S	SSSDSVEEETVDSDTPPVLEK	S5,T/S	0.984	0.904
Scaf11	SR-related CTD-associated factor 11	E9PZM7	S857,S859	ETVVESQSSQSPSPKR	S11,S13	1.057	0.306
Scaf11	SR-related CTD-associated factor 11	E9PZM7	S857,S859	ETVVESQSSQSPSPKRESAR	S11,S13	0.939	0.492
Scaf11	SR-related CTD-associated factor 11	E9PZM7	S857,S859	ETVVESQSSQSPSPK	S11,S13	0.900	0.164
Scaf11	SR-related CTD-associated factor 11	E9PZM7	Ambiguous	AEESLTVESTDLNDER	S/T	0.993	0.922
Scaf8	Protein SCAF8	Q6DID3	S617	ASEPVKEPVQTAQSPAPVEK	S14	1.123	0.048
Scamp1	Secretory carrier-associated membrane protein 1	Q8K021	S50	TPPPGSVK	S6	0.972	0.840
Scamp3	Secretory carrier-associated membrane protein	Q3UXS0	S79	KLSPTEPR	S3	0.959	0.468
Scap	Sterol regulatory element-binding protein cleavage-activating protein	Q6GQT6	S850	ASPEEPGDSPPLRR	S9	1.138	0.089
Scap	Sterol regulatory element-binding protein cleavage-activating protein	Q6GQT6	S850	ASPEEPGDSPPLR	S9	1.026	0.273
Scap	Sterol regulatory element-binding protein cleavage-activating protein	Q6GQT6	S934	MPALRPPSPGPPLPQASQEEGTAPEK	S8	0.947	0.326
Scarf1	Scavenger receptor class F member 1	Q5ND28	S612	SSGDLSSPIR	S7	1.014	0.712
Scarf1	Scavenger receptor class F member 1	Q5ND28	Y808	QAEEKEQEEPLYENVVPMSVPPQH	Y12	1.001	0.992
Scarf1	Scavenger receptor class F member 1	Q5ND28	Y808	QAEEKEQEEPLYENVVPMSVPPQH	Y12	0.907	0.232
Scarf1	Scavenger receptor class F member 1	Q5ND28	Ambiguous	GLGDGIESSGTVQEPDAGGSSLEQDSQK	S/T	0.904	0.078
Scfd1	Sec1 family domain-containing protein 1	Q8BRF7	S300	VNLEESTGVENSPAGARPK	S12	1.022	0.531
Scrib	Protein scribble homolog (Fragment)	D3YVA3	S60	GGPCSPPSPDELPANVK	S8	1.022	0.668
Scrib	Protein scribble homolog	Q80U72	S1292	TTEAPCSPGSQQPPSPDELPANVK	S15	0.947	0.243
Scrib	Protein scribble homolog	Q80U72	S1361	RVSLVGADDLR	S3	0.982	0.547
Scrib	Protein scribble homolog	Q80U72	S1457	MQSPELPAPER	S3	1.023	0.659
Scrib	Protein scribble homolog	Q80U72	S1457	MQSPELPAPER	S3	1.015	0.749
Scrib	Protein scribble homolog	Q80U72	S506	RNEAFVCKPDPSPPSPSEEEK	S15	1.018	0.826
Scrib	Protein scribble homolog	Q80U72	S506	RNEAFVCKPDPSPPSPSEEEKR	S15	1.076	0.382
Scrib	Protein scribble homolog	Q80U72	S506	NEAFVCKPDPSPPSPSEEEKR	S14	1.024	0.702
Scrib	Protein scribble homolog	Q80U72	S821	MVEPENAVTITPLRPEDDYSPR	S20	1.169	0.010
Scrib	Protein scribble homolog	Q80U72	T475	RATPHPSELK	T3	0.980	0.903
Scrib	Protein scribble homolog	Q80U72	S1284,S1292	TTEAPCSPGSQQPPSPDELPANVK	S7,S15	1.189	0.012
Scyl2	SCY1-like protein 2	Q8CFE4	S677	RGSLTLEEK	S3	1.057	0.591
Scyl2	SCY1-like protein 2	Q8CFE4	S677	GSLTLEEK	S2	0.943	0.242
Scyl3	Protein-associating with the carboxyl-terminal domain of ezrin	Q9DBQ7	S435	TSDLSPEGSPMHVVCSQQSR	S5	0.950	0.510
Scyl3	Protein-associating with the carboxyl-terminal domain of ezrin	Q9DBQ7	S435	TSDLSPEGSPMHVVCSQQSR	S5	1.012	0.934
Sdccag8	Serologically defined colon cancer antigen 8 homolog	Q80UF4	S4	SPGNSTLEDSLGQYQR	S1	0.894	0.273
Sdccag8	Serologically defined colon cancer antigen 8 homolog	Q80UF4	Ambiguous	QQTNKENETSPPR	T/S	1.062	0.286
Sde2	Replication stress response regulator SDE2	Q8K1J5	S269	EDGIDAVEVAADRPGSPR	S16	0.977	0.470
Sdf4	45 kDa calcium-binding protein	Q61112	S157	AVDPDGDGHVSWDEYK	S11	0.957	0.727
Sdha	Succinate dehydrogenase [ubiquinone] flavoprotein subunit, mitochondrial	Q8K2B3	S530	VGSVLQEGCEK	S3	0.936	0.520
Sec13	Protein SEC13 homolog	Q9D1M0	S313	GQGSVSASITEGQQNEQ	S8	0.962	0.738
Sec16a	Protein transport protein sec16	A2AIX1	S1028	MYSPSPSDGPASQQPLPNHPR	S3	0.898	0.121
Sec16a	Protein transport protein sec16	A2AIX1	S1028	MYSPSPSDGPASQQPLPNHPR	S3	0.988	0.718
Sec16a	Protein transport protein sec16	A2AIX1	S1245	ASHYSDQLAPR	S5	0.988	0.747
Sec16a	Protein transport protein sec16	A2AIX1	S1342	FTGSFDDDAEIHR	S4	1.121	0.535
Sec16a	Protein transport protein sec16	A2AIX1	S1995	GPLELSPAYGPPGSALGFPESSR	S6	1.299	0.414
Sec16a	Protein transport protein sec16	A2AIX1	S2053	SPDPEMVPRGSPVR	S11	0.942	0.651
Sec16a	Protein transport protein sec16	A2AIX1	S2053	GSPVRHSPPELSQEEFGESFADPGSSR	S2	0.904	0.238
Sec16a	Protein transport protein sec16	A2AIX1	S2101	APSLTSDSEGK	S3	1.097	0.010
Sec16a	Protein transport protein sec16	A2AIX1	S2315	CSSLSSLSQEVSR	S3	0.984	0.726
Sec16a	Protein transport protein sec16	A2AIX1	S422	GPSSEAAQEGDAQPYFSQSVGVR	S4	0.590	0.031
Sec16a	Protein transport protein sec16	A2AIX1	S467	CASGPQCENVENLEFVQNQEVLPR	S3	1.370	0.031
Sec16a	Protein transport protein sec16	A2AIX1	S1028,S1030	MYSPSPSDGPASQQPLPNHPR	S3,S5	0.967	0.790
Sec16a	Protein transport protein sec16	A2AIX1	S1242,S1245,S/Y	YSEPERPSSRASHYSDQLAPR	S12,S15,S/Y	0.977	0.802
Sec16a	Protein transport protein sec16	A2AIX1	Ambiguous	YSEPERPSSR	S/Y	0.914	0.745
Sec22b	Vesicle-trafficking protein SEC22b	O08547	S137	NLGSINTELQDVQR	S4	0.962	0.737
Sec22b	Vesicle-trafficking protein SEC22b	O08547	S137	RNLGSINTELQDVQR	S5	1.014	0.929
Sec31a	Protein transport protein Sec31A	Q3UPL0	S526	VDGPDVALKDSDQVAQSDGEESPAAEEQLLGER	S17	0.811	0.121
Sec31a	Protein transport protein Sec31A	Q3UPL0	S531	DSDQVAQSDGEESPAAEEQLLGER	S13	0.974	0.562
Sec31a	Protein transport protein Sec31A	Q3UPL0	S801	AQGKPVSGQESSQSPYER	S14	1.136	0.011
Sec31a	Protein transport protein Sec31A	Q3UPL0	S526,S531	DSDQVAQSDGEESPAAEEQLLGER	S8,S13	0.973	0.772
Sec61a1	Protein transport protein Sec61 subunit alpha isoform 1	P61620	Ambiguous	ETSMVHELNR	T/S	0.928	0.219
Sec62	Translocation protein SEC62	Q8BU14	T158	EETPGTPK	T6	1.036	0.750
Sec62	Translocation protein SEC62	Q8BU14	T158	KEETPGTPK	T7	1.054	0.540
Sec62	Translocation protein SEC62	Q8BU14	T375	EELEQQTDGDCDEEDDDKDGEVPK	T7	1.013	0.902

Secisbp2l	SECIS binding protein 2-like	A2AQE2	Ambiguous	SMVETSDGLEPSEMEK	T/S	1.015	0.633
Selenon	Selenoprotein N	D3Z2R5	S13	RPHSPDPGAQPAPPR	S4	1.160	0.017
Selenoo	Selenoprotein O	Q9DBC0	S544	LEQLSPSDLQR	S5	0.899	0.362
Selplg	P-selectin glycoprotein ligand 1	Q3TA56	S402	TEPSGDRDGDDLTLHSFLP	S4	1.029	0.651
Sema4b	Semaphorin-4B	Q62179	Ambiguous	GYQALSDSSPGPR	S/Y	0.974	0.682
Sema4c	Semaphorin-4C	Q64151	S761	CQPGGGPPSPPPGIPGQPLPSPTR	S9	1.126	0.617
Sema4c	Semaphorin-4C	Q64151	S761,S773	CQPGGGPPSPPPGIPGQPLPSPTR	S9,S21	1.107	0.306
Sema4d	Semaphorin-4D	O09126	S787	SDFSDLEQSVK	S9	1.017	0.782
Sema4d	Semaphorin-4D	O09126	S832	VPTDREDSQRIDELSAR	S8	1.078	0.350
Sema4d	Semaphorin-4D	O09126	S856	FADSDADGD	S4	1.032	0.445
Sema6b	Semaphorin-6B	O54951	T792,S796	ASHPGDFPLTPHASPDR	T10,S14	0.886	0.386
Sema6b	Semaphorin-6B	O54951	T792,S796	ASHPGDFPLTPHASPDRR	T10,S14	0.957	0.670
Senp1	Sentrin-specific protease 1	M0QWX4	S438	SHQLTDSEDEFPEITEEMEK	S7	0.977	0.824
Senp3	Sentrin-specific protease 3	Q9EP97	S163	NHLSPQEGGATPQVPSPCCR	S4	0.994	0.761
Senp3	Sentrin-specific protease 3	Q9EP97	S206	GSPPVPSGPPMEEDGLR	S2	0.908	0.167
Senp3	Sentrin-specific protease 3	Q9EP97	S206	GSPPVPSGPPMEEDGLR	S2	1.038	0.838
Senp3	Sentrin-specific protease 3	Q9EP97	S163,S175	NHLSPQEGGATPQVPSPCCR	S4,S16	1.094	0.586
Senp6	Sentrin-specific protease 6	F6Z9A1	S1138	DSLAETSLGDGAEQYASASGGSE	S22	0.922	0.586
Senp6	Sentrin-specific protease 6	F6Z9A1	Ambiguous	SSPAEPELNTIVIPR	S/T	0.896	0.665
Senp7	Sentrin-specific protease 7	Q8BUH8; Q9CQN9	S12;S12	RASSEIVTEGK	S3	1.181	0.007
Senp7	Sentrin-specific protease 7	Q8BUH8; Q9CQN9	S12;S12	RRASSEIVTEGK	S4	0.978	0.746
Senp7	Sentrin-specific protease 7	Q8BUH8; Q9CQN9	S47;S47	SEDVLAQSPLSK	S8	1.123	0.259
Senp7	Sentrin-specific protease 7	Q8BUH8	T248	QGDDGGVTPEMAAPHPK	T8	0.978	0.863
Senp7	Sentrin-specific protease 7	Q9CQN9	S241	STTATGQTETDQPSPGPACTNK	S14	0.954	0.555
Sephs1	Selenide, water dikinase 1	Q8BH69	Ambiguous	IIEVAPQVATQNVNPTPGATS	T/S	0.964	0.855
Sephs2	Selenide, water dikinase 2	P97364	Ambiguous	GASAAAAAAPDNSNAASEPSS	S	1.119	0.470
Sept2	Septin-2 (Fragment)	D3Z3C0	S218	IYHLPDAESDED	S9	1.145	0.489
Sept2	Septin-2	P42208	S218	IYHLPDAESDEDEDFK	S9	0.910	0.366
Sept2	Septin-2	P42208	S218	IYHLPDAESDEDEDFKEQTR	S9	0.972	0.647
Sept5	Sept5 protein	B7ZNM7	S339	MESPIPILPLPTPDAETEK	S3	1.399	0.265
Sept7	Septin-7	E9Q1G8	T228	IYEFPETDDEEENK	T7	0.993	0.982
Sept7	Septin-7	E9Q1G8	T426	ILEQQNSSRTLEK	T10	0.956	0.102
Sept8	Septin-8	B1AQZ0	S141	SLFDYHDTR	S1	0.996	0.946
Sept8	Septin-8	B1AQZ0	S444	SDIAAAQQSGMSLSNSK	S14	0.972	0.889
Sept9	Septin-9	Q80UG5	S228	LEPRPSVAEVPYR	S6	0.895	0.310
Sept9	Septin-9	Q80UG5	S30	SFEVEEIEPPNSTPPR	S1	0.913	0.554
Sept9	Septin-9	Q80UG5	S325	SVQPTSEERIPK	S1	0.963	0.541
Serbp1	Plasminogen activator inhibitor 1 RNA-binding protein	Q9CY58	S329	SKSEEAHAEDSVMDHHFR	S3	1.076	0.277
Serbp1	Plasminogen activator inhibitor 1 RNA-binding protein	Q9CY58	S329	SKSEEAHAEDSVMDHHFR	S3	1.046	0.543
Serbp1	Plasminogen activator inhibitor 1 RNA-binding protein	Q9CY58	S393	SSASAPDVDDPEAFPALA	S4	1.224	0.581
Serbp1	Plasminogen activator inhibitor 1 RNA-binding protein	Q9CY58	S63	SAAQAAAQTNSNAAGK	S11	0.879	0.032
Serbp1	Plasminogen activator inhibitor 1 RNA-binding protein	Q9CY58	Ambiguous	TDKSSASAPDVDDPEAFPALA	S/T	1.029	0.769
Serinc1	Serine incorporator 1	Q9QZI8	S364	SDGSLDDGDGIHR	S4	1.117	0.098
Serinc1	Serine incorporator 1	Q9QZI8	T347	LTLTSDESTLIEDGNGR	T4	1.168	0.160
Sesn1	Sestrin-1	E9PXR3	Ambiguous	ESMFVFSSDDDEVTPAR	S/T	0.933	0.701
Set	Protein SET	Q9EQU5	S160	EFHLNESGDPSSK	S7	0.929	0.281
Set	Protein SET	Q9EQU5	S30	SASPGLPK	S3	0.975	0.744
Setd1a	Histone-lysine N-methyltransferase SETD1A	E9PYH6	S483	SGSPAPETTNESVPFAQHSSLDSR	S3	0.951	0.078
Setd1a	Histone-lysine N-methyltransferase SETD1A	E9PYH6	S521	FSFLASDTEEEEENSSAGPGAR	S6	1.086	0.785
Setd1a	Histone-lysine N-methyltransferase SETD1A	E9PYH6	Ambiguous	RPRPSTPAEEDEDDPEREK	S/T	1.067	0.448
Setd1a	Histone-lysine N-methyltransferase SETD1A	E9PYH6	Ambiguous	EVPEPLPAPDEKPETDGLVDSPVMPLSEK	S/T	1.977	0.010
Setd1a	Histone-lysine N-methyltransferase SETD1A	E9PYH6	Ambiguous	SSSDTAAYPAGTTVGGTPGNGTPCSQDTNFSSSR	T/S/Y	1.000	1.000
Setd1b	Histone-lysine N-methyltransferase SETD1B	Q8CFT2	S977	LRPSTSVDEEDEESERER	S6	1.054	0.452
Setd1b	Histone-lysine N-methyltransferase SETD1B	Q8CFT2	S1682,S	HEDLVAPSASPEPSPPQPLFRPR	S14,S	0.942	0.201
Setd2	Histone-lysine N-methyltransferase SETD2	E9Q5F9	S104	QSDSPNSPAPPLQVDSNPK	S7	0.939	0.377
Setd2	Histone-lysine N-methyltransferase SETD2	E9Q5F9	S132	MDAGDTFPATEESSPPK	S14	0.940	0.583
Setd2	Histone-lysine N-methyltransferase SETD2	E9Q5F9	S1819	TAVPQLSEGDGYSSENTSR	S14	1.098	0.172
Setd2	Histone-lysine N-methyltransferase SETD2	E9Q5F9	S322	SSQFEGTFLGSESDEDSVR	S11	0.880	0.381
Setd2	Histone-lysine N-methyltransferase SETD2	E9Q5F9	S532	CCSPPNELGFR	S3	0.976	0.444
Setd2	Histone-lysine N-methyltransferase SETD2	E9Q5F9	T1827	AHTPLNTPDPSAKPSTEMDTDTPK	T3	1.151	0.042
Setd2	Histone-lysine N-methyltransferase SETD2	E9Q5F9	S1812,S1818	TAVPQLSEGDGYSSENTSR	S7,S13	1.171	0.211
Setd2	Histone-lysine N-methyltransferase SETD2	E9Q5F9	S2053,S2055	RGSLSPPSSAYER	S3,S5	0.968	0.258
Setd2	Histone-lysine N-methyltransferase SETD2	E9Q5F9	S624,S/T	TGSPTPSNQLNDSPTFK	S13,S/T	1.018	0.702
Setd2	Histone-lysine N-methyltransferase SETD2	E9Q5F9	Ambiguous	MDAGDTFPATEESSPPKSR	S/T	0.975	0.589
Setd2	Histone-lysine N-methyltransferase SETD2	E9Q5F9	Ambiguous	CEENTSPTLDAVESK	T/S	0.961	0.183
Setd2	Histone-lysine N-methyltransferase SETD2	E9Q5F9	Ambiguous	NSTLPMEETSPCSSR	T/S	1.023	0.813
Setd2	Histone-lysine N-methyltransferase SETD2	E9Q5F9	Ambiguous	TGSPTPSNQLNDSPTFK	T/S	0.986	0.831
Setd3	Histone-lysine N-methyltransferase setd3	Q91WC0	S571	SENESLSPEESENVTGEESSGSMAK	S7	0.933	0.345
Setd3	Histone-lysine N-methyltransferase setd3	Q91WC0	S571	SENESLSPEESENVTGEESSGSMAK	S7	1.056	0.284
Setd5	SET domain-containing protein 5	Q5XJV7	S1232	GATVYSPSR	S6	1.000	1.000
Setd5	SET domain-containing protein 5	Q5XJV7	S72	SDLNGLPSPVEER	S8	0.986	0.824
Setd5	SET domain-containing protein 5	Q5XJV7	Ambiguous	TAAPESEVSSPVSNVAIPSTPQSTGVNTR	S/T	0.840	0.192
Setd6	N-lysine methyltransferase SETD6	Q9CWY3	S22	VSGGSPLVAPCPSPR	S13	0.958	0.542
Setd6	N-lysine methyltransferase SETD6	Q9CWY3	S14,S22	VSGGSPLVAPCPSPR	S5,S13	1.152	0.359
Setx	Probable helicase senataxin	A2AKX3	S1376	IVQGSDVLEADSDEPDDHR	S12	1.007	0.864
Sf1	Splicing factor 1	Q64213	S82	SPSPEPIYNSEGK	S3	1.024	0.552
Sf1	Splicing factor 1	Q64213	S80,S82	TGDLGIPPNPEDRSPSPEPIYNSEGKR	S14,S16	0.966	0.626
Sf1	Splicing factor 1	Q64213	S80,S82	TGDLGIPPNPEDRSPSPEPIYNSEGK	S14,S16	0.998	0.955
Sf1	Splicing factor 1	Q64213	Ambiguous	TGDLGIPPNPEDRSPSPEPIYNSEGK	S/Y/T	0.991	0.906
Sf3a1	Splicing factor 3A subunit 1	Q8K4Z5	S329	FGESEEVEMEVESDEEDQEK	S13	0.974	0.823
Sf3a1	Splicing factor 3A subunit 1	Q8K4Z5	S329	FGESEEVEMEVESDEEDQEK	S13	0.972	0.874
Sf3b1	Splicing factor 3B subunit 1	G5E866	S194	VVNGAAASQPPSKR	S12	0.973	0.647
Sf3b1	Splicing factor 3B subunit 1	G5E866	T326	TDRGGDSIGETPTPGASK	T11	0.970	0.495
Sf3b1	Splicing factor 3B subunit 1	G5E866	T326	GGDSIGETPTPGASK	T8	1.040	0.072
Sf3b1	Splicing factor 3B subunit 1	G5E866	T426	VLPPPAGYVPIRTPAR	T13	0.997	0.966
Sf3b1	Splicing factor 3B subunit 1	G5E866	T207,T211	WDQTADQTPGATPK	T8,T12	1.044	0.329
Sf3b1	Splicing factor 3B subunit 1	G5E866	T207,T211	RWDQTADQTPGATPK	T9,T13	0.875	0.069
Sf3b1	Splicing factor 3B subunit 1	G5E866	T244,T248	AKGSETPGATPGSK	T6,T10	0.973	0.746
Sf3b1	Splicing factor 3B subunit 1	G5E866	T244,T248	GSETPGATPGSK	T4,T8	1.050	0.308
Sf3b1	Splicing factor 3B subunit 1	G5E866	T326,T328	TDRGGDSIGETPTPGASKR	T11,T13	1.033	0.817
Sf3b1	Splicing factor 3B subunit 1	G5E866	T326,S332	GGDSIGETPTPGASK	T8,S14	0.897	0.338
Sf3b1	Splicing factor 3B subunit 1	G5E866	Ambiguous	GSETPGATPGSK	S/T	1.039	0.329
Sf3b2	Splicing factor 3b, subunit 2	Q3UJB0	S292	SSLGQSASETEEDTVSISK	S8	0.851	0.194
Sf3b2	Splicing factor 3b, subunit 2	Q3UJB0	S844	EQQAQVEKEDFSDMVEHAAK	S12	0.996	0.941
Sf3b2	Splicing factor 3b, subunit 2	Q3UJB0	S290,S292	SSLGQSASETEEDTVSISK	S6,S8	0.962	0.690
Sf3b2	Splicing factor 3b, subunit 2	Q3UJB0	Ambiguous	SSLGQSASETEEDTVSISKK	T/S	1.015	0.916
Sfmbt1	Scm-like with four MBT domains protein 1	Q9JMD1	S764	DAQTSSVSDDENKPPSPK	S8	0.943	0.090
Sfmbt1	Scm-like with four MBT domains protein 1	Q9JMD1	S762,S772	DAQTSSVSDDENKPPSPK	S6,S16	0.995	0.964
Sfmbt1	Scm-like with four MBT domains protein 1	Q9JMD1	Ambiguous	SSSSSPTQSETPTPLPPDTQTNKR	S/T	1.168	0.118
Sfpq	Splicing factor, proline- and glutamine-rich	Q8VIJ6	T679	GMGPGTPAGYGR	T6	1.011	0.637
Sfr1	Swi5-dependent recombination DNA repair protein 1 homolog	Q8BP27	S115	ENPPSPHSNSSGKQPLSGTPK	S5	0.976	0.797
Sfr1	Swi5-dependent recombination DNA repair protein 1 homolog	Q8BP27	S115	ENPPSPHSNSSGK	S5	0.970	0.861
Sfr1	Swi5-dependent recombination DNA repair protein 1 homolog	Q8BP27	S163	VENDENNETLSEPGESSKEENCSK	S11	0.968	0.182
Sfr1	Swi5-dependent recombination DNA repair protein 1 homolog	Q8BP27	S163	MKVENDENNETLSEPGESSKEENCSK	S13	1.098	0.064
Sfr1	Swi5-dependent recombination DNA repair protein 1 homolog	Q8BP27	S163	MKVENDENNETLSEPGESSKEENCSK	S13	0.955	0.475
Sfr1	Swi5-dependent recombination DNA repair protein 1 homolog	Q8BP27	S67;S145;S239	ENPPSPPTSPAAPQPR	S5	0.975	0.382
Sfr1	Swi5-dependent recombination DNA repair protein 1 homolog	Q8BP27	T129	QPLSGTPK	T6	1.013	0.910
Sfr1	Swi5-dependent recombination DNA repair protein 1 homolog	Q8BP27	S115,T129	ENPPSPHSNSSGKQPLSGTPK	S5,T19	1.026	0.779
Sfr1	Swi5-dependent recombination DNA repair protein 1 homolog	Q8BP27	S67,S71,S149,S243	ENPPSPPTSPAAPQPR	S5,S9	0.991	0.767
Sfswap	Splicing factor, suppressor of white-apricot homolog	Q3USH5	S283	SGPTSDNEEEDDEEDGSYLHPSLFASK	S5	1.067	0.014
Sfswap	Splicing factor, suppressor of white-apricot homolog	Q3USH5	Ambiguous	MSGSPGVSR	S	1.056	0.438
Sgo1	Shugoshin 1	Q9CXH7	S120	GRPSDVVSSIDNTTR	S4	0.870	0.143
Sgpp1	Sphingosine-1-phosphate phosphatase 1	Q9JI99	S101	NSLTGEEGELVK	S2	1.004	0.960
Sgpp1	Sphingosine-1-phosphate phosphatase 1	Q9JI99	S101	RNSLTGEEGELVK	S3	1.062	0.145
Sgta	Small glutamine-rich tetratricopeptide repeat-containing protein alpha	Q8BJU0	T82	APDRTPPSEEDSAEAER	T5	1.030	0.515
Sgtb	Small glutamine-rich tetratricopeptide repeat-containing protein beta	Q8VD33	S297	SFSSSADEHS	S3	1.050	0.757
Sh2b2	SH2B adapter protein 2	Q9JID9	S107	SSEDVSAHAATK	S2	1.033	0.700
Sh2b2	SH2B adapter protein 2	Q9JID9	S145	SSPEPDGGATPK	S1	0.917	0.219
Sh2b2	SH2B adapter protein 2	Q9JID9	S146	RSSPEPDGGATPK	S3	0.972	0.781
Sh2b2	SH2B adapter protein 2	Q9JID9	Ambiguous	SNSTEHLLDAASGATEEPTEATLGR	S/T	0.836	0.486
Sh2b2	SH2B adapter protein 2	Q9JID9	Ambiguous	TATYGHSRSSEDVSAHAATK	T/S/Y	1.222	0.305
Sh2b2	SH2B adapter protein 2	Q9JID9	Ambiguous	AVENQYSFY	Y/S	0.853	0.563
Sh2b3	SH2B adapter protein 3	O09039	S102	SSEDLGPRPACALQHLR	S1	1.031	0.508
Sh2b3	SH2B adapter protein 3	O09039	S129	SAGELPGATSDTNDIDTTAASRPGPAR	S1	1.028	0.428
Sh2b3	SH2B adapter protein 3	O09039	S129	RRSAGELPGATSDTNDIDTTAASRPGPAR	S3	1.167	0.278
Sh2b3	SH2B adapter protein 3	O09039	S13,S17	TSSAPASPASPR	S3,S7	1.050	0.075
Sh2d3c	SH2 domain-containing protein 3C	Q9QZS8	S341	TASPASPSGSK	S6	1.140	0.138
Sh2d3c	SH2 domain-containing protein 3C	Q9QZS8	S435	VHAPSATPSTSAQPASPVAR	S16	1.025	0.831
Sh3bgrl3	SH3 domain-binding glutamic acid-rich-like protein 3	Q91VW3	S14	VYSTSVTGSREIK	S9	0.926	0.233
Sh3bp1	SH3 domain-binding protein 1	P55194	S262	DNHSQADHSPLTTAAPFSR	S9	1.035	0.350
Sh3bp1	SH3 domain-binding protein 1	P55194	S535	TEADLPKPTSPK	S10	0.996	0.939
Sh3bp1	SH3 domain-binding protein 1	P55194	S535	ERTEADLPKPTSPK	S12	1.029	0.622
Sh3bp1	SH3 domain-binding protein 1	P55194	S632	LPASPVISNMPAQVDQGVATEDR	S4	1.623	0.074
Sh3bp1	SH3 domain-binding protein 1	P55194	S632	RLPASPVISNMPAQVDQGVATEDR	S5	0.913	0.105
Sh3bp1	SH3 domain-binding protein 1	P55194	S677	GLISETE	S4	0.892	0.203
Sh3bp1	SH3 domain-binding protein 1	P55194	T592	SSPPAPSLPPGSVSPGTPQALPR	T17	0.981	0.577
Sh3bp1	SH3 domain-binding protein 1	P55194	S257,S262	DNHSQADHSPLTTAAPFSR	S4,S9	1.074	0.511
Sh3bp1	SH3 domain-binding protein 1	P55194	S577,S589	SSPPAPSLPPGSVSPGTPQALPR	S2,S14	1.032	0.655

Sh3bp2	SH3 domain-binding protein 2	E9QJU7	S470	SPPDGQSFR	S1	1.023	0.484
Sh3bp4	SH3 domain-binding protein 4	Q921I6	S131	NSTLSDSGMIDNLPDSPEEVAK	S16	1.080	0.521
Sh3bp5	SH3 domain-binding protein 5	Q9Z131	S10	SRSDEPAELPPPAR	S3	0.959	0.201
Sh3bp5	SH3 domain-binding protein 5	Q9Z131	S421	SQSSTSLESQALETR	S3	0.885	0.074
Sh3bp5	SH3 domain-binding protein 5	Q9Z131	S378,S379	SECSGASSPECEVERGDREAGAENK	S7,S8	1.041	0.619
Sh3bp5	SH3 domain-binding protein 5	Q9Z131	S421,T423	SQSSTSLESQALETR	S3,T5	1.030	0.638
Sh3bp5	SH3 domain-binding protein 5	Q9Z131	S421,T/S	SRSQSSTSLESQALETR	S5,S/T	0.889	0.365
Sh3bp5l	SH3 domain-binding protein 5-like	Q99LH9	S30	SEVVEDEGPRSPVAEEPGGSGSNSSETK	S11	1.089	0.321
Sh3bp5l	SH3 domain-binding protein 5-like	Q99LH9	S342	TVASDLQK	S4	0.984	0.812
Sh3bp5l	SH3 domain-binding protein 5-like	Q99LH9	S361	GLSDHASLDGQELGAQSR	S7	1.007	0.909
Sh3bp5l	SH3 domain-binding protein 5-like	Q99LH9	S377	GSDIGVR	S2	1.019	0.855
Sh3bp5l	SH3 domain-binding protein 5-like	Q99LH9	S377	RGSDIGVR	S3	1.051	0.359
Sh3bp5l	SH3 domain-binding protein 5-like	Q99LH9	T13	AAGGRETPQGELR	T7	0.989	0.734
Sh3bp5l	SH3 domain-binding protein 5-like	Q99LH9	S342,S349	TVASDLQKCDSVEHLR	S4,S11	1.035	0.711
Sh3bp5l	SH3 domain-binding protein 5-like	Q99LH9	Ambiguous	SPVAEEPGGSGSNSSETKLSPR	S/T	1.040	0.590
Sh3gl1	Endophilin-A2	Q62419	S288	ITASSSFR	S5	0.889	0.050
Sh3kbp1	SH3 domain-containing kinase-binding protein 1 (Fragment)	B0R0Y8	S80	RPPSQSLTSVSDNK	S4	0.996	0.937
Sh3kbp1	SH3 domain-containing kinase-binding protein 1	Q8R550	S108	CQVAFSYLPQNDDELELK	S6	0.976	0.934
Sh3kbp1	SH3 domain-containing kinase-binding protein 1	Q8R550	S156	ELSGESDELGISQDEQLSK	S3	1.027	0.805
Sh3kbp1	SH3 domain-containing kinase-binding protein 1	Q8R550	S227	ETTGSESDGGDSSSTK	S7	1.135	0.003
Sh3kbp1	SH3 domain-containing kinase-binding protein 1	Q8R550	S227	ETTGSESDGGDSSSTKSEGANGTMATAAIQPK	S7	1.050	0.674
Sh3kbp1	SH3 domain-containing kinase-binding protein 1	Q8R550	S227	ETTGSESDGGDSSSTKSEGANGTMATAAIQPK	S7	0.868	0.091
Sh3kbp1	SH3 domain-containing kinase-binding protein 1	Q8R550	S274	SIEVENDFLPVEK	S1	1.044	0.399
Sh3kbp1	SH3 domain-containing kinase-binding protein 1	Q8R550	S454	TNSLNRPGALPPR	S3	1.004	0.968
Sh3kbp1	SH3 domain-containing kinase-binding protein 1	Q8R550	S227,S237	ETTGSESDGGDSSSTKSEGANGTMATAAIQPK	S7,S17	1.110	0.456
Sh3pxd2a	SH3 and PX domain-containing protein 2A	O89032	S1008	NSSFSTAR	S3	1.003	0.954
Sh3pxd2a	SH3 and PX domain-containing protein 2A	O89032	S1034	AASQGSESPLLPTQR	S8	0.962	0.642
Sh3pxd2a	SH3 and PX domain-containing protein 2A	O89032	S546	EAEENPVGACESQGSPLK	S15	1.081	0.177
Sh3pxd2a	SH3 and PX domain-containing protein 2A	O89032	S546	EAEENPVGACESQGSPLKVK	S15	1.000	0.994
Sh3pxd2a	SH3 and PX domain-containing protein 2A	O89032	S592	RISPASSLQR	S3	1.023	0.607
Sh3pxd2a	SH3 and PX domain-containing protein 2A	O89032	S643	GSSGDSDSPGSSSLSLAVK	S8	1.002	0.989
Sh3pxd2a	SH3 and PX domain-containing protein 2A	O89032	S721	SASDAGIRDTPK	S3	1.044	0.377
Sh3pxd2a	SH3 and PX domain-containing protein 2A	O89032	S916	SSQNEGKSDSLEK	S10	1.043	0.523
Sh3pxd2a	SH3 and PX domain-containing protein 2A	O89032	S993	NESLTATDSLR	S3	0.962	0.481
Sh3pxd2a	SH3 and PX domain-containing protein 2A	O89032	S993	RNESLTATDSLR	S4	1.080	0.012
Sh3pxd2a	SH3 and PX domain-containing protein 2A	O89032	S1029,S1034	AASQGSESPLLPTQR	S3,S8	1.007	0.941
Sh3pxd2a	SH3 and PX domain-containing protein 2A	O89032	Ambiguous	AQISSPNLR	S	1.000	0.994
Sh3pxd2a	SH3 and PX domain-containing protein 2A	O89032	Ambiguous	YEEPEYDVPAFGFDSEPEMNEEPSGDR	Y/S	0.831	0.582
Sh3pxd2b	SH3 and PX domain-containing protein 2B	A2AAY5	S717	QDGLSPK	S5	1.075	0.500
Sh3pxd2b	SH3 and PX domain-containing protein 2B	A2AAY5	S717	SGRQDGLSPK	S8	0.997	0.976
Sh3pxd2b	SH3 and PX domain-containing protein 2B	A2AAY5	Y661	TEPAQSEDHVDIYNLR	Y13	1.005	0.974
Sh3pxd2b	SH3 and PX domain-containing protein 2B	A2AAY5	Ambiguous	TSSSPLSCRPLPEVR	S/T	1.007	0.949
Sh3pxd2b	SH3 and PX domain-containing protein 2B	A2AAY5	Ambiguous	LGPSSPAHSGALDLDGVSR	S	0.913	0.011
Sh3tc1	SH3 domain and tetratricopeptide repeats 1	G3X9F6	S105	SPSLQQALR	S3	1.067	0.065
Sh3tc1	SH3 domain and tetratricopeptide repeats 1	G3X9F6	S31	GSCSPEDQIQSVSMGLPTGQER	S4	1.137	0.413
Sh3tc1	SH3 domain and tetratricopeptide repeats 1	G3X9F6	S481	RASSLDLEEPR	S3	1.030	0.258
Sh3tc1	SH3 domain and tetratricopeptide repeats 1	G3X9F6	S482	ASSLDLEEPR	S3	1.023	0.616
Sh3tc1	SH3 domain and tetratricopeptide repeats 1	G3X9F6	S52	TGSAEAEATVR	S3	1.063	0.355
Sh3tc1	SH3 domain and tetratricopeptide repeats 1	G3X9F6	S481,S482	RASSLDLEEPR	S3,S4	1.066	0.053
Shb	SH2 domain-containing adapter protein B	Q6PD21	S382	HGSPEFCGILGER	S3	1.245	0.239
Shc1	SHC-transforming protein 1	P98083	S449	QAGGGAGPPNPSLNGSAPR	S16	1.104	0.346
Shkbp1	SH3KBP1-binding protein 1	Q6P7W2	S185	APPSPSGQPEEPGMVR	S4	1.071	0.295
Shkbp1	SH3KBP1-binding protein 1	Q6P7W2	S621	GSFPSPSPR	S7	0.975	0.654
Shkbp1	SH3KBP1-binding protein 1	Q6P7W2	S646	GSPSPPQAEAR	S4	0.927	0.315
Shkbp1	SH3KBP1-binding protein 1	Q6P7W2	S644,S646	GSPSPPQAEAR	S2,S4	0.951	0.351
Shprh	E3 ubiquitin-protein ligase SHPRH	Q7TPQ3	S626	DCAESPNPAAEELAQSNTSSPCETSDYR	S5	1.077	0.681
Shtn1	Shootin-1	Q8K2Q9	S506	SMPVLGSVSSVTK	S1	1.261	0.251
Shtn1	Shootin-1	Q8K2Q9	T537	TLEAEFNNPCPLTPEPGEGPR	T13	1.110	0.252
Shtn1	Shootin-1	Q8K2Q9	Y24	EQAIGEYEDLRAENQK	Y7	0.955	0.224
Shtn1	Shootin-1	Q8K2Q9	S464,S467	SLKSLGPENSETELER	S1,S4	1.009	0.931
Shtn1	Shootin-1	Q8K2Q9	Ambiguous	LTAEADSSSPTGILATSESK	S/T	0.922	0.440
Sidt2	SID1 transmembrane family member 2 (Fragment)	E0CXC7	S342	ACPESGLGSPLHR	S9	0.982	0.834
Sidt2	SID1 transmembrane family member 2 (Fragment)	E0CXC7	S433	SFDAVGPRPR	S1	1.034	0.499
Sigirr	Single Ig IL-1-related receptor	Q9JLZ8	T367	GPVFGEPPTPLQETR	T9	0.890	0.393
Siglec1	Sialic acid binding Ig-like lectin 1, sialoadhesin, isoform CRA_b	G3X8X6	S1683	SSTKLNEDENSAEMATK	S11	1.036	0.639
Siglec1	Sialic acid binding Ig-like lectin 1, sialoadhesin, isoform CRA_b	G3X8X6	S1683	LNEDENSAEMATK	S7	1.023	0.694
Siglec1	Sialic acid binding Ig-like lectin 1, sialoadhesin, isoform CRA_b	G3X8X6	S1683	LNEDENSAEMATK	S7	1.077	0.410
Siglec1	Sialic acid binding Ig-like lectin 1, sialoadhesin, isoform CRA_b	G3X8X6	S1683	LNEDENSAEMATKK	S7	0.981	0.783
Siglec1	Sialic acid binding Ig-like lectin 1, sialoadhesin, isoform CRA_b	G3X8X6	S1683	LNEDENSAEMATKK	S7	1.022	0.657
Siglec1	Sialic acid binding Ig-like lectin 1, sialoadhesin, isoform CRA_b	G3X8X6	Ambiguous	LDLDTSGVGDGR	T/S	0.812	0.530
Siglec1	Sialic acid binding Ig-like lectin 1, sialoadhesin, isoform CRA_b	G3X8X6	Ambiguous	LDLDTSGVGDGRR	T/S	0.898	0.118
Sik3	Serine/threonine-protein kinase SIK3	E9PU87	S592	GPSPLVTMTPAVPAVTPVDEESSDGEPDQEAVQR	S23	0.956	0.641
Sik3	Serine/threonine-protein kinase SIK3	E9PU87	S914	QLSADSAEAHSLNMNR	S3	0.936	0.506
Sik3	Serine/threonine-protein kinase SIK3	E9PU87	S914	QLSADSAEAHSLNMNR	S3	0.971	0.672
Sik3	Serine/threonine-protein kinase SIK3	E9PU87	S572,T576	GPSPLVTMTPAVPAVTPVDEESSDGEPDQEAVQR	S3,T7	1.935	0.324
Sik3	Serine/threonine-protein kinase SIK3	E9PU87	Ambiguous	GPSPLVTMTPAVPAVTPVDEESSDGEPDQEAVQR	S/T	1.487	0.459
Sik3	Serine/threonine-protein kinase SIK3	E9PU87	Ambiguous	HTLAMTSPTAEIPPDLQR	T/S	1.062	0.654
Simc1	SUMO-interacting motifs-containing 1	E9Q6E9	Ambiguous	AVTENELTPPQDETQTSPR	T/S	0.893	0.073
Sin3a	Paired amphipathic helix protein Sin3a	Q60520	S1113	YMSSDTTSPELR	S8	1.036	0.806
Sin3a	Paired amphipathic helix protein Sin3a	Q60520	S1113	YMSSDTTSPELR	S8	1.032	0.201
Sin3a	Paired amphipathic helix protein Sin3a	Q60520	S833	GDLSDVEEEEEEEMDVDEATGAPK	S4	1.056	0.110
Sin3a	Paired amphipathic helix protein Sin3a	Q60520	S833	GDLSDVEEEEEEEMDVDEATGAPKK	S4	0.969	0.822
Sin3a	Paired amphipathic helix protein Sin3a	Q60520	S833	GDLSDVEEEEEEEMDVDEATGAPK	S4	1.008	0.930
Sin3a	Paired amphipathic helix protein Sin3a	Q60520	S941	DKSDSPAIQLR	S5	0.986	0.827
Sin3b	Paired amphipathic helix protein Sin3b	Q62141	S698	RPTDEKPPADASPEPPK	S12	0.950	0.588
Sipa1	Signal-induced proliferation-associated protein 1	E9Q0Y4	S65	SGSDAGEVRPPTPASPR	S15	0.960	0.310
Sipa1	Signal-induced proliferation-associated protein 1	E9Q0Y4	S53,S65	SGSDAGEVRPPTPASPR	S3,S15	1.023	0.524
Sipa1	Signal-induced proliferation-associated protein 1	E9Q0Y4	Ambiguous	DSSSPPGPGDLTEER	S/T	0.996	0.949
Sipa1	Signal-induced proliferation-associated protein 1	E9Q0Y4	Ambiguous	ETPPSQDQSGSPSSHEDTSDSGPELR	S/T	1.000	1.000
Sipa1l1	Signal-induced proliferation-associated 1-like protein 1	Q8C0T5	S1412	HSASPVVFSSAR	S4	0.949	0.400
Sipa1l1	Signal-induced proliferation-associated 1-like protein 1	Q8C0T5	S1528	LIDLESPTPESQK	S6	1.111	0.074
Sipa1l1	Signal-induced proliferation-associated 1-like protein 1	Q8C0T5	S1564	TLSDESIYSSQR	S3	0.982	0.723
Sipa1l1	Signal-induced proliferation-associated 1-like protein 1	Q8C0T5	S162	FLMPEAYPSSPR	S10	1.344	0.284
Sipa1l1	Signal-induced proliferation-associated 1-like protein 1	Q8C0T5	S1626	SLHGEFSASDSSLTDIQETR	S9	0.962	0.791
Sipa1l1	Signal-induced proliferation-associated 1-like protein 1	Q8C0T5	S208	EYGSTSSIDK	S4	1.021	0.601
Sipa1l1	Signal-induced proliferation-associated 1-like protein 1	Q8C0T5	S288	SKSETGDSSIFR	S3	1.127	0.080
Sipa1l1	Signal-induced proliferation-associated 1-like protein 1	Q8C0T5	T1090	GAHSPQVPSQLQSPMTSR	T16	0.943	0.643
Sipa1l1	Signal-induced proliferation-associated 1-like protein 1	Q8C0T5	Ambiguous	FLMPEAYPSSPR	S/Y	0.947	0.359
Sipa1l1	Signal-induced proliferation-associated 1-like protein 1	Q8C0T5	Ambiguous	SISSDGRPLER	S	1.367	0.003
Sipa1l2	Signal-induced proliferation-associated 1-like protein 2	Q80TE4	S1030	VVIIQPHEDGSPR	S11	0.920	0.511
Sipa1l2	Signal-induced proliferation-associated 1-like protein 2	Q80TE4	S1082	ASPVPGTPDR	S2	0.999	0.991
Sipa1l2	Signal-induced proliferation-associated 1-like protein 2	Q80TE4	S1406	SEGSPPPEEPEVTECPR	S4	0.939	0.661
Sipa1l2	Signal-induced proliferation-associated 1-like protein 2	Q80TE4	S1406	KSEGSPPPEEPEVTECPR	S5	1.039	0.435
Sipa1l2	Signal-induced proliferation-associated 1-like protein 2	Q80TE4	S1461	LMLPDSPLVEEGR	S6	0.986	0.710
Sipa1l2	Signal-induced proliferation-associated 1-like protein 2	Q80TE4	S1478	FSFYGNVSPR	S8	0.939	0.487
Sipa1l2	Signal-induced proliferation-associated 1-like protein 2	Q80TE4	S1488	TLSDESVCSNRR	S3	0.950	0.361
Sipa1l2	Signal-induced proliferation-associated 1-like protein 2	Q80TE4	S1488	TLSDESVCSNR	S3	1.002	0.919
Sipa1l2	Signal-induced proliferation-associated 1-like protein 2	Q80TE4	S163	SNSDITISDIDTEDVLDQHAVNPNTGAALHR	S3	0.970	0.855
Sipa1l2	Signal-induced proliferation-associated 1-like protein 2	Q80TE4	S195	EYGSTSSIDR	S4	0.949	0.383
Sipa1l3	Signal-induced proliferation-associated 1-like protein 3	G3X9J0	S1154	QPSGSFSTPGSATYAR	S5	1.030	0.615
Sipa1l3	Signal-induced proliferation-associated 1-like protein 3	G3X9J0	S1358	EVSPAPVVAGQNK	S3	1.031	0.371
Sipa1l3	Signal-induced proliferation-associated 1-like protein 3	G3X9J0	S1376	LYSSGSCTPPGLVGGSR	S3	1.162	0.274
Sipa1l3	Signal-induced proliferation-associated 1-like protein 3	G3X9J0	S1538	TLSDESLCSGR	S3	1.002	0.957
Sipa1l3	Signal-induced proliferation-associated 1-like protein 3	G3X9J0	S1538	TLSDESLCSGRR	S3	0.976	0.725
Sipa1l3	Signal-induced proliferation-associated 1-like protein 3	G3X9J0	S1614	SAISASELSLADGR	S6	0.791	0.282
Sipa1l3	Signal-induced proliferation-associated 1-like protein 3	G3X9J0	S94	EQSNPSPSQDTDGVK	S6	1.034	0.307
Sipa1l3	Signal-induced proliferation-associated 1-like protein 3	G3X9J0	Ambiguous	SSSEITLSECDVEEPGDPR	S/T	0.942	0.262
Sirpa	Tyrosine-protein phosphatase non-receptor type substrate 1	E0CYM8	Ambiguous	APEPNNHTEYASIETGK	Y/T/S	0.936	0.341
Sirt1	NAD-dependent protein deacetylase sirtuin-1	Q923E4	S737	QELTDVNYPSDKS	S13	0.930	0.027
Sirt2	NAD-dependent protein deacetylase sirtuin-2	Q8VDQ8	Ambiguous	SPSTGLYANLEK	S/T/Y	1.093	0.315
Sirt2	NAD-dependent protein deacetylase sirtuin-2	Q8VDQ8	Ambiguous	VQEAQDSDSDTEGGATGGEAEMDFLR	S/T	0.507	0.144
Sirt2	NAD-dependent protein deacetylase sirtuin-2	Q8VDQ8	Ambiguous	VQEAQDSDSDTEGGATGGEAEMDFLR	S/T	0.938	0.480
Ska3	Spindle and kinetochore-associated protein 3	Q8C263	S34	ALDGEDSDFEDSPGR	S7	0.961	0.622
Skap2	Src kinase-associated phosphoprotein 2	Q3UND0	Y260	SQPIDDEIYEELPEEEEDTASVK	Y9	1.099	0.263
Skap2	Src kinase-associated phosphoprotein 2	Q3UND0	Y260,S272	SQPIDDEIYEELPEEEEDTASVK	Y9,S21	0.918	0.629
Skap2	Src kinase-associated phosphoprotein 2	Q3UND0	Ambiguous	SQPIDDEIYEELPEEEEDTASVKMDEQGKGSR	S/Y/T	0.779	0.319
Skap2	Src kinase-associated phosphoprotein 2	Q3UND0	Ambiguous	SQPIDDEIYEELPEEEEDTASVKMDEQGK	Y/T/S	0.971	0.900
Skil	Ski-like protein	Q60665	S507	DSEDSSPLLVR	S6	0.791	0.013
Skint6	Selection and upkeep of intraepithelial T-cells protein 6	A7XUZ6	Ambiguous	SLLLGSIQSILHSFSVQK	S	1.158	0.624
Skiv2l	Superkiller viralicidic activity 2-like (S. cerevisiae)	Q6NZR5	S221	LLEPLDLSGGDEDEGEAAGGPR	S8	1.025	0.866
Skiv2l	Superkiller viralicidic activity 2-like (S. cerevisiae)	Q6NZR5	S240	GDNASPSPSGTPLVR	S5	1.000	0.999
Skiv2l	Superkiller viralicidic activity 2-like (S. cerevisiae)	Q6NZR5	Ambiguous	ASSLEDLVLK	S	0.940	0.490
Slain2	SLAIN motif-containing protein 2	Q8CI08	S248	SSSDRNPPLSPQSSIDSELSASELDEDSIGSNYK	S10	0.966	0.834
Slain2	SLAIN motif-containing protein 2	Q8CI08	S423	TSSTQVDSVK	S3	1.052	0.375
Slain2	SLAIN motif-containing protein 2	Q8CI08	S468	SPAAPSPLALR	S6	0.919	0.510
Slain2	SLAIN motif-containing protein 2	Q8CI08	S48	SGAVQGAGLLGPGSPAR	S14	0.937	0.342
Slain2	SLAIN motif-containing protein 2	Q8CI08	S63	VGVSTPSSGAASPR	S12	0.986	0.627
Slain2	SLAIN motif-containing protein 2	Q8CI08	S354,S358	NSPRPSPKQSPR	S6,S10	1.007	0.953

Slain2	SLAIN motif-containing protein 2	Q8CI08	Ambiguous	RGTFSDQELDAQSLDDEDDSLQHAVHPALNR	T/S	0.996	0.974
Slbp	Histone RNA hairpin-binding protein	P97440	S182	SWDQQIK	S1	1.038	0.273
Slc12a2	Solute carrier family 12 member 2	E9QM38	T211	TFGHNTMDAVPR	T6	0.827	0.072
Slc12a4	Solute carrier family 12 member 4	Q9JIS8	S37	AEREDSDGQGNHRENSPFLCPLDASR	S6	0.969	0.686
Slc12a4	Solute carrier family 12 member 4	Q9JIS8	S47	ENSPFLCPLDASR	S3	0.798	0.095
Slc12a4	Solute carrier family 12 member 4	Q9JIS8	S967	LESLYSDEEEESVAGADK	S6	0.975	0.610
Slc12a5	Solute carrier family 12 member 5	Q91V14	S963	EIQSITDESRGSIR	S12	0.962	0.197
Slc12a5	Solute carrier family 12 member 5	Q91V14	Ambiguous	EIQSITDESRGSIRR	S/T	1.108	0.547
Slc12a6	Solute carrier family 12 member 6	Q924N4	S1032	LTSIGSDEDEETETYQEK	S6	0.997	0.924
Slc12a6	Solute carrier family 12 member 6	Q924N4	S148	VSSLLNR	S3	1.089	0.379
Slc12a6	Solute carrier family 12 member 6	Q924N4	S32	IDDIPGLSDTSPDLSSR	S11	1.074	0.246
Slc12a6	Solute carrier family 12 member 6	Q924N4	S96	TSNPQDVTEDPSQNSITGEHSQLLDDGHK	S15	1.029	0.797
Slc12a6	Solute carrier family 12 member 6	Q924N4	S1029,S1032	LTSIGSDEDEETETYQEK	S3,S6	1.435	0.019
Slc12a6	Solute carrier family 12 member 6	Q924N4	S50,S/T	FSSRESVPETSR	S6,S/T	1.112	0.051
Slc12a6	Solute carrier family 12 member 6	Q924N4	Ambiguous	TSNPQDVTEDPSQNSITGEHSQLLDDGHKK	S/T	1.047	0.402
Slc12a6	Solute carrier family 12 member 6	Q924N4	Ambiguous	FSSRESVPETSR	S/T	1.100	0.096
Slc12a7	Solute carrier family 12 member 7	Q9WVL3	S30	ADGAGDEAAERTEEPESPESVDQTSPTPGDGNPR	S17	0.937	0.557
Slc12a7	Solute carrier family 12 member 7	Q9WVL3	S50	ENSPFINNVEVER	S3	1.149	0.179
Slc12a7	Solute carrier family 12 member 7	Q9WVL3	S62	ENSPFINNVEVERESYFEGK	S15	0.904	0.015
Slc12a9	Solute carrier family 12 member 9	Q99MR3	S659	EGGSPALSTLFPPPR	S4	0.743	0.115
Slc15a3	Solute carrier family 15 member 3	Q8BPX9	S283	SSSRDSESAHLLPDQR	S6	0.892	0.219
Slc15a3	Solute carrier family 15 member 3	Q8BPX9	S285	DSESAHLLPDQR	S4	1.044	0.388
Slc15a4	Solute carrier family 15 member 4	Q91W98	S281	SGEGLGVFQQSSK	S1	0.967	0.564
Slc15a4	Solute carrier family 15 member 4	Q91W98	S299	HSLFDSCK	S6	1.099	0.028
Slc16a1	Monocarboxylate transporter 1	P53986	S213	SKESLQEAGK	S4	1.069	0.480
Slc16a1	Monocarboxylate transporter 1	P53986	S477	AAQSPQQHSSGDPTEEESPV	S4	1.024	0.526
Slc16a1	Monocarboxylate transporter 1	P53986	S461,T462	EGKEDEASTDVDEKPK	S8,T9	1.033	0.689
Slc16a1	Monocarboxylate transporter 1	P53986	Ambiguous	EGKEDEASTDVDEKPK	T/S	1.037	0.562
Slc16a10	Monocarboxylate transporter 10	Q3U9N9	S266	ESGGSRSSFFSR	S8	1.036	0.555
Slc16a3	Monocarboxylate transporter 4	P57787	S430	RPEVTEPEEVASEEK	S12	1.009	0.873
Slc16a3	Monocarboxylate transporter 4	P57787	S430	KRPEVTEPEEVASEEK	S13	0.960	0.519
Slc16a3	Monocarboxylate transporter 4	P57787	S469	NGEVVHTPETSV	S11	0.917	0.043
Slc16a3	Monocarboxylate transporter 4	P57787	T465	AEPEKNGEVVHTPETSV	T12	1.009	0.870
Slc16a3	Monocarboxylate transporter 4	P57787	T465,T468	NGEVVHTPETSV	T7,T10	1.059	0.640
Slc16a3	Monocarboxylate transporter 4	P57787	T468,S469	AEPEKNGEVVHTPETSV	T15,S16	1.005	0.944
Slc16a7	Monocarboxylate transporter 2	O70451	Ambiguous	ASNAHNPPSDRDKESNI	S	0.933	0.379
Slc17a5	Sialin	Q8BN82	S19	GPAGNDDEESSDSTPLLPGAR	S13	0.938	0.548
Slc19a1	Folate transporter 1	P41438	Ambiguous	SPLETSVQAISLQDGDLR	T/S	0.909	0.692
Slc1a5	Amino acid transporter	Q9ESU7	S508	MPSSEPELIQVK	S4	1.011	0.963
Slc20a1	Sodium-dependent phosphate transporter 1	Q61609	S269,S271	SSPSESPLMEK	S2,S4	1.128	0.134
Slc20a1	Sodium-dependent phosphate transporter 1	Q61609	S271,S	EVKSSPSESPLMEK	S7,S	1.338	0.060
Slc22a4	Solute carrier family 22 member 4	Q9Z306	S539	STVSVDREESPK	S4	1.040	0.615
Slc22a5	Solute carrier family 22 member 5	Q9Z0E8	S548	DGEESPTVLK	S5	1.036	0.574
Slc23a2	Solute carrier family 23 member 2	Q9EPR4	S81	SSLAETLDSTGSLDPQR	S12	0.998	0.901
Slc23a2	Solute carrier family 23 member 2	Q9EPR4	T647	SSDKDSQATV	S6	1.040	0.581
Slc23a2	Solute carrier family 23 member 2	Q9EPR4	S78,S81	SSLAETLDSTGSLDPQR	S9,S12	1.015	0.854
Slc27a1	Long-chain fatty acid transport protein 1	Q60714	S468	FDGYVSDSATNK	S6	0.859	0.273
Slc28a2	Sodium/nucleoside cotransporter 2	O88627	S9	SVSQATVENCMENPGLELMEGGNLEQR	S1	1.020	0.910
Slc29a3	Equilibrative nucleoside transporter 3	Q99P65	S21	APSNHQEADQEALLGK	S3	1.082	0.013
Slc29a3	Equilibrative nucleoside transporter 3	Q99P65	S271	VFSGEDNPSQDAPSASSVAPASR	S3	1.126	0.052
Slc2a1	Solute carrier family 2, facilitated glucose transporter member 1	P17809	T478	QGGASQSDKTPEELFHPLGADSQV	T10	0.969	0.685
Slc2a9	Solute carrier family 2 (facilitated glucose transporter), member 9	Q3T9X0	Ambiguous	ADSAMTEEKANSQTEPDSSSTLDSYGQNK	T/S/Y	1.091	0.756
Slc30a1	Zinc transporter 1	Q60738	S502	NVPNKQPESSL	S10	1.008	0.865
Slc33a1	Acetyl-coenzyme A transporter 1	Q99J27	S42	RDSVGGEGDREVLLGDAGPGDLPK	S3	1.121	0.185
Slc35a5	Probable UDP-sugar transporter protein SLC35A5	Q921R7	S407	HLSGSLWER	S3	1.133	0.128
Slc35a5	Probable UDP-sugar transporter protein SLC35A5	Q921R7	S432	LKSDDSDDDTL	S6	0.998	0.980
Slc35a5	Probable UDP-sugar transporter protein SLC35A5	Q921R7	S429,S432	LKSDDSDDDTL	S3,S6	1.016	0.886
Slc35c2	Solute carrier family 35 member C2	Q8VCX2	S335	SLGSSADLELLLR	S4	1.022	0.805
Slc35f6	Solute carrier family 35 member F6	Q8VE96	T352	LPTQEGEQER	T3	1.067	0.492
Slc35f6	Solute carrier family 35 member F6	Q8VE96	T366	LLGDSRTPINEAS	T7	1.081	0.100
Slc37a2	Glucose-6-phosphate exchanger SLC37A2	Q9WU81	S274	ESNVDIAASSSK	S9	1.087	0.152
Slc37a2	Glucose-6-phosphate exchanger SLC37A2	Q9WU81	Ambiguous	HHDDPEKEQDNPEDPVNSPYSSRESNVDIAASSSK	S/Y	1.081	0.675
Slc37a2	Glucose-6-phosphate exchanger SLC37A2	Q9WU81	Ambiguous	HHDDPEKEQDNPEDPVNSPYSSR	S/Y	1.114	0.180
Slc37a2	Glucose-6-phosphate exchanger SLC37A2	Q9WU81	Ambiguous	EQDNPEDPVNSPYSSRESNVDIAASSSK	Y/S	1.327	0.036
Slc37a2	Glucose-6-phosphate exchanger SLC37A2	Q9WU81	Ambiguous	HHDDPEKEQDNPEDPVNSPYSSRESNVDIAASSSK	S/Y	1.005	0.950
Slc37a2	Glucose-6-phosphate exchanger SLC37A2	Q9WU81	Ambiguous	HHDDPEKEQDNPEDPVNSPYSSRESNVDIAASSSK	S/Y	0.947	0.693
Slc38a1	Sodium-coupled neutral amino acid transporter 1	Q8K2P7	S52	SLTNSHLEK	S1	1.176	0.022
Slc38a10	Putative sodium-coupled neutral amino acid transporter 10	J3QNE8	S1029	DLASHPEQELAPK	S4	1.040	0.576
Slc38a10	Putative sodium-coupled neutral amino acid transporter 10	J3QNE8	S441	LSVQDPVVVVAEDSQEK	S14	0.963	0.842
Slc38a10	Putative sodium-coupled neutral amino acid transporter 10	J3QNE8	S545	GQGAPPLPESEKEKQEPER	S10	1.003	0.958
Slc38a10	Putative sodium-coupled neutral amino acid transporter 10	J3QNE8	S608	DPQPAAQARDSVELK	S11	1.133	0.191
Slc38a10	Putative sodium-coupled neutral amino acid transporter 10	J3QNE8	S866	AAPPEVPKSPEK	S9	0.957	0.670
Slc38a10	Putative sodium-coupled neutral amino acid transporter 10	J3QNE8	S887	QDVFGEGSEER	S8	1.097	0.162
Slc38a10	Putative sodium-coupled neutral amino acid transporter 10	J3QNE8	S887	QDVFGEGSEERK	S8	0.964	0.751
Slc38a10	Putative sodium-coupled neutral amino acid transporter 10	J3QNE8	S991	QRPDPNSGPK	S7	0.986	0.814
Slc38a10	Putative sodium-coupled neutral amino acid transporter 10	J3QNE8	Ambiguous	DLADLPAGGSETEPQGAPIDLREDPK	T/S	0.952	0.858
Slc38a10	Putative sodium-coupled neutral amino acid transporter 10	J3QNE8	Ambiguous	DLADLPAGGSETEPQGAPIDLR	T/S	0.614	0.202
Slc38a2	Sodium-coupled neutral amino acid transporter 2	Q8CFE6	Ambiguous	FNISPDEDSSSYSSNSDFNYSYPTK	S/Y/T	1.098	0.706
Slc39a6	Zinc transporter ZIP6	Q8C145	S191	ESASSSEVTSAVYNAVSEGTR	S5	1.200	0.051
Slc39a6	Zinc transporter ZIP6	Q8C145	S488	YDSQLSSNEEKVDPGERPESYLR	S6	1.232	0.162
Slc39a6	Zinc transporter ZIP6	Q8C145	S488	YDSQLSSNEEK	S6	1.137	0.107
Slc40a1	Solute carrier family 40 member 1	Q9JHI9	Ambiguous	EVTDENQPNTSVV	S/T	0.885	0.052
Slc44a1	Choline transporter-like protein 1	A2AMH5	S654	ELKPMASGASSA	S10	0.976	0.738
Slc44a2	Choline transporter-like protein 2	A0A1L1SVG6	T12	DAVYGTPQKYDPTFK	T6	0.896	0.163
Slc45a4	Solute carrier family 45 member 4	Q0P5V9	S408	GSPPINSLSR	S2	1.028	0.707
Slc45a4	Solute carrier family 45 member 4	Q0P5V9	S442	QASSTFSYYGK	S3	1.219	0.008
Slc45a4	Solute carrier family 45 member 4	Q0P5V9	S472	SMSDLYDLQQR	S1	1.040	0.767
Slc46a1	Proton-coupled folate transporter	Q6PEM8	S458	VNPHPEFQQFPQSP	S13	0.956	0.469
Slc49a3	Solute carrier family 49 member A3	Q8CE47	Ambiguous	RLEAESGGSSSPTMCAR	S/T	0.918	0.289
Slc49a3	Solute carrier family 49 member A3	Q8CE47	Ambiguous	LEAESGGSSSPTMCAR	S/T	0.989	0.905
Slc4a1ap	Solute carrier family 4 (anion exchanger), member 1, adaptor protein	E9PX68	S258	MLGEDSDEEEEANTTEGK	S6	1.008	0.688
Slc4a1ap	Solute carrier family 4 (anion exchanger), member 1, adaptor protein	E9PX68	S258	MLGEDSDEEEEANTTEGK	S6	1.097	0.254
Slc4a1ap	Solute carrier family 4 (anion exchanger), member 1, adaptor protein	E9PX68	S258	KMLGEDSDEEEEANTTEGK	S7	0.818	0.171
Slc4a1ap	Solute carrier family 4 (anion exchanger), member 1, adaptor protein	E9PX68	S258	KMLGEDSDEEEEANTTEGK	S7	0.980	0.635
Slc4a2	Anion exchange protein	A0A0R4J101	S112	RRPGASPTGETPTIEEGEEDEEEASEAEGFR	S6	1.246	0.239
Slc4a2	Anion exchange protein	A0A0R4J101	S172	TSPSPPTQTPHQEAAPR	S4	1.105	0.143
Slc4a2	Anion exchange protein	A0A0R4J101	Ambiguous	IGSMTGVEQALLPR	S/T	0.837	0.360
Slc4a2	Anion exchange protein	A0A0R4J101	Ambiguous	KPERTSPSPPTQTPHQEAAPR	T/S	1.024	0.902
Slc4a7	Anion exchange protein	F8VQC9	S1130	YMDAETSL	S7	0.982	0.644
Slc4a7	Anion exchange protein	F8VQC9	S238	SFADIGK	S1	0.946	0.545
Slc4a7	Anion exchange protein	F8VQC9	S263	NGILASPQSAPGNLDNSK	S9	0.946	0.617
Slc4a7	Anion exchange protein	F8VQC9	S284	GNGSGGSRENSTVDFSK	S7	1.078	0.133
Slc4a7	Anion exchange protein	F8VQC9	S284,S288	GNGSGGSRENSTVDFSK	S7,S11	1.061	0.557
Slc7a11	Cystine/glutamate transporter	Q9WTR6	S26	LPSMGDQEPPGQEK	S3	0.997	0.977
Slc7a4	Cationic amino acid transporter 4	Q8BLQ7	S427	ASPPSSPCLASPGPTAK	S11	1.100	0.081
Slc7a6os	Probable RNA polymerase II nuclear localization protein SLC7A6OS	Q7TPE5	S165	TSDPDVILCNSVELIR	S11	0.885	0.188
Slc7a6os	Probable RNA polymerase II nuclear localization protein SLC7A6OS	Q7TPE5	S305	EFDYDSPHGLDSD	S12	0.914	0.219
Slc7a7	Y+L amino acid transporter 1	Q9Z1K8	S19	YEVAAQHEADDGSALGDGASPVAEQVK	S13	1.021	0.896
Slc7a8	Large neutral amino acids transporter small subunit 2	Q9QXW9	S18	NHPGSDTSPEAEASSGGGGVALK	S5	1.053	0.429
Slc7a8	Large neutral amino acids transporter small subunit 2	Q9QXW9	S18	NHPGSDTSPEAEASSGGGGVALKK	S5	0.962	0.512
Slc8a1	Sodium/calcium exchanger 1	G3X9J1	S389	AVSMHEVNMEMAENDPVSK	S3	1.272	0.265
Slc8a1	Sodium/calcium exchanger 1	G3X9J1	S389	AVSMHEVNMEMAENDPVSK	S3	0.990	0.789
Slc8a1	Sodium/calcium exchanger 1	G3X9J1	S389	KAVSMHEVNMEMAENDPVSK	S4	1.007	0.923
Slc9a1	Sodium/hydrogen exchanger 1	Q61165	S697	LDSPTLSR	S3	1.040	0.484
Slc9a1	Sodium/hydrogen exchanger 1	Q61165	S707	IGSDPLAYEPK	S3	1.220	0.085
Slc9a1	Sodium/hydrogen exchanger 1	Q61165	S790	EPSSPGTDDVFTPGSSDSPSSQR	S18	0.924	0.758
Slc9a1	Sodium/hydrogen exchanger 1	Q61165	S790	SKEPSSPGTDDVFTPGSSDSPSSQR	S20	1.028	0.618
Slc9a1	Sodium/hydrogen exchanger 1	Q61165	S801	CLSDPGPHPEPGEGEPFIPK	S3	1.314	0.008
Slc9a1	Sodium/hydrogen exchanger 1	Q61165	T755	GPRVTPEEEEEDEDGIIMIR	T5	1.094	0.188
Slc9a1	Sodium/hydrogen exchanger 1	Q61165	T755	GPRVTPEEEEEDEDGIIMIR	T5	0.975	0.753
Slc9a1	Sodium/hydrogen exchanger 1	Q61165	S776,S790	SKEPSSPGTDDVFTPGSSDSPSSQR	S6,S20	1.067	0.489
Slc9a3r1	Na(+)/H(+) exchange regulatory cofactor NHE-RF1	P70441	S275	EALVEPASESPRPALAR	S10	0.975	0.329
Slc9a3r1	Na(+)/H(+) exchange regulatory cofactor NHE-RF1	P70441	S283	SASSDTSEELNSQDSPK	S1	1.087	0.003
Slc9a3r1	Na(+)/H(+) exchange regulatory cofactor NHE-RF1	P70441	S285	SASSDTSEELNSQDSPKR	S3	0.995	0.927
Slc9a3r1	Na(+)/H(+) exchange regulatory cofactor NHE-RF1	P70441	S285,T288	SASSDTSEELNSQDSPK	S3,T6	1.038	0.383
Slc9a3r1	Na(+)/H(+) exchange regulatory cofactor NHE-RF1	P70441	S285,S297	SASSDTSEELNSQDSPKR	S3,S15	0.993	0.882
Slc9a6	Sodium/hydrogen exchanger	A1L3P4	S693	LVLPMDDSEPALNSLDDTR	S14	1.008	0.953
Slc9a6	Sodium/hydrogen exchanger	A1L3P4	S700	LVLPMDDSEPALNSLDDTRHSPA	S21	1.051	0.880
Slc9a6	Sodium/hydrogen exchanger	A1L3P4	S700	LVLPMDDSEPALNSLDDTRHSPA	S21	0.933	0.248
Slc9a7	Sodium/hydrogen exchanger 7	Q8BLV3	S546	VGVEELSEEDQNENR	S7	1.042	0.176
Slc9a7	Sodium/hydrogen exchanger 7	Q8BLV3	S694	TKSSSEEVLER	S3	1.461	0.004
Slc9a8	Sodium/hydrogen exchanger 8	Q8R4D1	S568	QGPSGSEDDEQELF	S6	0.929	0.442
Slc9a8	Sodium/hydrogen exchanger 8	Q8R4D1	S566,S568	QGPSGSEDDEQELF	S4,S6	1.069	0.676
Slirp	SRA stem-loop-interacting RNA-binding protein, mitochondrial	Q9D8T7	S105	ALHGAQTSDEER	S8	1.156	0.097
Slitrk4	SLIT and NTRK-like protein 4	Q810B8	S800	KSLIGGNHSK	S9	0.952	0.267
Slk	STE20-like serine/threonine-protein kinase	O54988	S340	RASSDLSIASSEEDK	S3	1.029	0.653
Slk	STE20-like serine/threonine-protein kinase	O54988	S444	LENLPDTQDQQTVDVNSVSEENENNR	S17	0.901	0.748
Slk	STE20-like serine/threonine-protein kinase	O54988	S666	ALGSEGEAAATEVDLER	S4	0.847	0.257

Slk	STE20-like serine/threonine-protein kinase	O54988	S666	ALGSEGEAAATEVDLERK	S4	0.955	0.470
Slk	STE20-like serine/threonine-protein kinase	O54988	S777	DSGSVSLQETR	S4	1.064	0.350
Slk	STE20-like serine/threonine-protein kinase	O54988	S777	AKDSGSVSLQETR	S6	0.970	0.556
Slk	STE20-like serine/threonine-protein kinase	O54988	Ambiguous	VITSDRSSEVGTDEALDDTQK	S/T	0.931	0.582
Sltm	SAFB-like transcription modulator	Q8CH25	S1011	IVQMSGNSLPR	S8	0.981	0.846
Sltm	SAFB-like transcription modulator	Q8CH25	S289	DVQDAIAQSPEK	S9	1.072	0.326
Sltm	SAFB-like transcription modulator	Q8CH25	S289	DVQDAIAQSPEKEAK	S9	1.039	0.542
Sltm	SAFB-like transcription modulator	Q8CH25	S999	AGAGMITQHSSTASPVNR	S14	1.039	0.295
Slu7	Pre-mRNA-splicing factor SLU7	Q8BHJ9	S215	LVEQANSPK	S7	1.002	0.973
Slx4	Structure-specific endonuclease subunit SLX4	Q6P1D7	S880	SLSPTTPTK	S3	1.015	0.823
Slx4	Structure-specific endonuclease subunit SLX4	Q6P1D7	Ambiguous	TALGDDVPRSPPEETVGENEYK	T/S/Y	1.064	0.646
Smad6	Mothers against decapentaplegic homolog 6	O35182	S275	LCGPESPPPPYSR	S6	0.802	0.393
Smap	Small acidic protein	Q9R0P4	S17	SASPDDDLGSSNWEAADLGNEERK	S3	0.987	0.754
Smap	Small acidic protein	Q9R0P4	S17	SASPDDDLGSSNWEAADLGNEER	S3	1.041	0.561
Smap	Small acidic protein	Q9R0P4	S17	RSASPDDDLGSSNWEAADLGNEER	S4	1.000	1.000
Smap	Small acidic protein	Q9R0P4	S87	INEELESQYQQSMDSK	S12	0.973	0.706
Smap	Small acidic protein	Q9R0P4	Ambiguous	RSASPDDDLGSSNWEAADLGNEERK	S	0.947	0.144
Smap2	Stromal membrane-associated protein 2	Q7TN29	S219	DLDLLASVPSPSSVSR	S10	1.118	0.362
Smarca2	Probable global transcription activator SNF2L2	H3BLH0	S602	KAEENAEGGEPALGPDGEPIDESSQMSDLPVK	S27	0.913	0.640
Smarca4	Transcription activator BRG1	A0A0R4J170	S1349	EVDYSDSLTEK	S7	1.110	0.278
Smarca4	Transcription activator BRG1	A0A0R4J170	S613	AENAEGQTPAIGPDGEPLDETSQMSDLPVK	S25	1.035	0.379
Smarca4	Transcription activator BRG1	A0A0R4J170	S695	IPDPDSDDVSEVDAR	S6	1.047	0.451
Smarca4	Transcription activator BRG1	A0A0R4J170	S699	KIPDPDSDDVSEVDAR	S11	1.064	0.040
Smarca4	Transcription activator BRG1	A0A0R4J170	Ambiguous	AENAEGQTPAIGPDGEPLDETSQMSDLPVK	S/T	0.905	0.483
Smarca5	-related matrix-associated actin-dependent regulator of chromatin subfamily A m	Q91ZW3	S65	GGPEGGAAPAAPCAAGSGPADTEMEEVFDHGSPGK	S32	1.084	0.608
Smarca5	-related matrix-associated actin-dependent regulator of chromatin subfamily A m	Q91ZW3	S65	GGPEGGAAPAAPCAAGSGPADTEMEEVFDHGSPGK	S32	1.000	1.000
Smarcad1	l matrix-associated actin-dependent regulator of chromatin subfamily A containin	Q04692	S124	DTVIIVSEPSEDEESHDLPSVTR	S7	1.045	0.567
Smarcad1	l matrix-associated actin-dependent regulator of chromatin subfamily A containin	Q04692	S235	GDRGEESNESAEASSNWEK	S7	0.889	0.319
Smarcad1	l matrix-associated actin-dependent regulator of chromatin subfamily A containin	Q04692	S235,S238	GDRGEESNESAEASSNWEK	S7,S10	0.954	0.631
Smarcad1	l matrix-associated actin-dependent regulator of chromatin subfamily A containin	Q04692	Ambiguous	AIQEYIDLSSDTEDVSPNCSSTVQEK	S/T/Y	0.695	0.134
Smarcc1	SWI/SNF complex subunit SMARCC1	P97496	S327	KPSPSPPPPTATESR	S3	1.013	0.686
Smarcc1	SWI/SNF complex subunit SMARCC1	P97496	S327	RKPSPSPPPPTATESR	S4	0.983	0.795
Smarcc1	SWI/SNF complex subunit SMARCC1	P97496	S327,S329	KPSPSPPPPTATESR	S3,S5	1.116	0.206
Smarcc1	SWI/SNF complex subunit SMARCC1	P97496	S327,S329	RKPSPSPPPPTATESR	S4,S6	1.044	0.661
Smarcc2	SWI/SNF complex subunit SMARCC2	Q6PDG5	S283	TLTDEVNSPDSDRR	S8	0.963	0.363
Smarcc2	SWI/SNF complex subunit SMARCC2	Q6PDG5	S283	TLTDEVNSPDSDRRDK	S8	0.901	0.373
Smarcc2	SWI/SNF complex subunit SMARCC2	Q6PDG5	S283	TLTDEVNSPDSDR	S8	1.015	0.752
Smarcc2	SWI/SNF complex subunit SMARCC2	Q6PDG5	S304	SPSPSPTPEAK	S3	0.941	0.180
Smarcc2	SWI/SNF complex subunit SMARCC2	Q6PDG5	S347	GHREEEQEDLTKDMDEPSPVPNVEEVTLPK	S18	1.037	0.462
Smarcc2	SWI/SNF complex subunit SMARCC2	Q6PDG5	S347	DMDEPSPVPNVEEVTLPK	S6	1.171	0.221
Smarcc2	SWI/SNF complex subunit SMARCC2	Q6PDG5	S347	DMDEPSPVPNVEEVTLPK	S6	1.060	0.384
Smarcc2	SWI/SNF complex subunit SMARCC2	Q6PDG5	S387	GGTMTDLDEQDDESMETTGKDEDENSTGNK	S14	1.075	0.449
Smarcc2	SWI/SNF complex subunit SMARCC2	Q6PDG5	S387	GGTMTDLDEQDDESMETTGKDEDENSTGNK	S14	0.924	0.607
Smarcc2	SWI/SNF complex subunit SMARCC2	Q6PDG5	S283,S286	TLTDEVNSPDSDRR	S8,S11	1.025	0.426
Smarcc2	SWI/SNF complex subunit SMARCC2	Q6PDG5	S283,S286	TLTDEVNSPDSDRRDK	S8,S11	1.013	0.820
Smarcc2	SWI/SNF complex subunit SMARCC2	Q6PDG5	S302,S304	SPSPSPTPEAK	S1,S3	0.997	0.933
Smarcc2	SWI/SNF complex subunit SMARCC2	Q6PDG5	S302,S304	RSPSPSPTPEAK	S2,S4	1.046	0.286
Smarcc2	SWI/SNF complex subunit SMARCC2	Q6PDG5	S302,S304	KRSPSPSPTPEAK	S3,S5	1.005	0.919
Smarcc2	SWI/SNF complex subunit SMARCC2	Q6PDG5	Ambiguous	GGTMTDLDEQDDESMETTGKDEDENSTGNKGEQTK	S/T	1.023	0.780
Smc1a	Structural maintenance of chromosomes protein 1A	Q9CU62	S957	GTMDDISQEEGSSQGEESVSGSQR	S13	0.979	0.770
Smc1a	Structural maintenance of chromosomes protein 1A	Q9CU62	S966	GTMDDISQEEGSSQGEESVSGSQR	S22	0.955	0.454
Smc1a	Structural maintenance of chromosomes protein 1A	Q9CU62	Ambiguous	MEEESQSQGRDLTLEENQVK	S/T	1.100	0.343
Smc3	Structural maintenance of chromosomes protein 3	Q9CW03	S1067	KGDVEGSQSQDEGEGSGESER	S9	1.004	0.880
Smc3	Structural maintenance of chromosomes protein 3	Q9CW03	S1065,S1067	KGDVEGSQSQDEGEGSGESER	S7,S9	1.006	0.901
Smcr8	Guanine nucleotide exchange protein SMCR8	Q3UMB5	S497	SDSQASLTVPLSPHVVR	S12	1.014	0.851
Smcr8	Guanine nucleotide exchange protein SMCR8	Q3UMB5	S488,S491	SDSQASLTVPLSPHVVR	S3,S6	0.938	0.012
Smcr8	Guanine nucleotide exchange protein SMCR8	Q3UMB5	Ambiguous	GSISSGESIEVLGTEK	S/T	1.082	0.398
Smg1	Serine/threonine-protein kinase SMG1	Q8BKX6	S32	TDSASADPDTLK	S3	1.112	0.165
Smg1	Serine/threonine-protein kinase SMG1	Q8BKX6	S3553	TQPDVMSQNAK	S7	0.989	0.699
Smg1	Serine/threonine-protein kinase SMG1	Q8BKX6	S3553	TQPDVMSQNAK	S7	1.004	0.940
Smg1	Serine/threonine-protein kinase SMG1	Q8BKX6	T3570	NLATSADTPPSTIPGTGK	T8	0.831	0.181
Smg1	Serine/threonine-protein kinase SMG1	Q8BKX6	T3547,S3553	SNTGQKTQPDVMSQNAK	T7,S13	0.898	0.320
Smg6	Telomerase-binding protein EST1A	P61406	S331	NWSGCGEGEQK	S3	1.107	0.076
Smg8	Protein SMG8	Q8VE18	S668	NEPSPAPPDSDAEK	S4	1.044	0.433
Smg8	Protein SMG8	Q8VE18	S742	QASTVEYLPGMLHSNCPK	S3	1.187	0.137
Smg8	Protein SMG8	Q8VE18	S98	AVSVGEAGGAGDPGAAAGDSLR	S3	0.923	0.329
Smg9	Protein SMG9	Q9DB90	S510	KSSALAEYSR	S3	0.974	0.777
Smg9	Protein SMG9	Q9DB90	S53	DGSEDPSTNVMQK	S3	1.182	0.074
Smg9	Protein SMG9	Q9DB90	S53	RDGSEDPSTNVMQK	S4	0.946	0.264
Smg9	Protein SMG9	Q9DB90	Ambiguous	EEGKGPVAGTGASTPEGTAPPPPTAPAPPKGEK	T/S	1.185	0.266
Smim13	Small integral membrane protein 13	E9Q942	Ambiguous	ELVGDTGSQEGDNEQPSGSETEEDPSASPQK	S/T	1.054	0.317
Smim13	Small integral membrane protein 13	E9Q942	Ambiguous	ELVGDTGSQEGDNEQPSGSETEEDPSASPQK	T/S	1.078	0.116
Smim24	Small integral membrane protein 24	Q0VG18	Ambiguous	GGQSNEALELEEKESSDEER	S	1.092	0.445
Smn1	Survival motor neuron protein	P97801	S25	GTGQSDDSDIWDDTALIK	S5	1.013	0.961
Smn1	Survival motor neuron protein	P97801	Ambiguous	RGTGQSDDSDIWDDTALIK	S/T	0.980	0.862
Smpd3	Sphingomyelin phosphodiesterase 3	Q9JJY3	S292	DGDSGSLGSPSASR	S9	0.979	0.594
Smurf1	E3 ubiquitin-protein ligase	E9PYU8	S200	FVESPSQDQR	S4	1.133	0.049
Snap23	Synaptosomal-associated protein	Q9D3L3	S121	ATWGDGGDNSPSNVVSK	S10	1.035	0.519
Snap23	Synaptosomal-associated protein	Q9D3L3	S121	ATWGDGGDNSPSNVVSKQPSR	S10	0.946	0.846
Snap23	Synaptosomal-associated protein	Q9D3L3	S146	ITNGQPQQTTGAASGGYIK	S14	1.075	0.540
Snap23	Synaptosomal-associated protein	Q9D3L3	S20	AHQVTDESLESTR	S8	0.771	0.110
Snap23	Synaptosomal-associated protein	Q9D3L3	S20	AHQVTDESLESTRR	S8	1.030	0.580
Snap23	Synaptosomal-associated protein	Q9D3L3	S121,T/S	ATWGDGGDNSPSNVVSK	S10,T/S	1.098	0.378
Snap23	Synaptosomal-associated protein	Q9D3L3	Ambiguous	QPSRITNGQPQQTTGAASGGYIK	S/T/Y	1.068	0.480
Snap23	Synaptosomal-associated protein	Q9D3L3	Ambiguous	AHQVTDESLESTRR	S/T	1.043	0.729
Snap29	Synaptosomal-associated protein 29	Q9ERB0	S65	SLSLMYESEK	S3	1.030	0.867
Snap29	Synaptosomal-associated protein 29	Q9ERB0	S77	IGVASSEELVR	S5	0.947	0.179
Snap29	Synaptosomal-associated protein 29	Q9ERB0	Ambiguous	SLSLMYESEK	S/Y	1.246	0.238
Snapc1	snRNA-activating protein complex subunit 1	Q8K0S9	S311	LDSSDSDSGSGQVQGR	S6	0.962	0.606
Snapc4	snRNA-activating protein complex subunit 4	A2AIV6	S1301	APSPGEVSAPSPLDASDGLDDLNVLR	S3	1.756	0.235
Snapin	SNARE-associated protein Snapin	Q9Z266	S126	AMLDSGVYPPGSPSK	S5	1.046	0.088
Snapin	SNARE-associated protein Snapin	Q9Z266	S133	AMLDSGVYPPGSPSK	S12	1.003	0.953
Sncaip	Synphilin-1	G5E848	S120	GGETDQGPQPEELSPEDGVGGLPGK	S14	1.100	0.531
Sncaip	Synphilin-1	G5E848	S675	SLSESDTDSNNSEDPK	S3	1.253	0.002
Snd1	Staphylococcal nuclease domain-containing protein 1	Q78PY7	S150	ANNPEQNRLSECEEQAK	S10	1.069	0.283
Snd1	Staphylococcal nuclease domain-containing protein 1	Q78PY7	S909	YGDFRADDADEFGYSR	S15	0.945	0.465
Snd1	Staphylococcal nuclease domain-containing protein 1	Q78PY7	T235	RETDGSETPEPFAAEAK	T3	1.045	0.268
Snd1	Staphylococcal nuclease domain-containing protein 1	Q78PY7	Y109	EYGMIYLGK	Y2	1.034	0.735
Snd1	Staphylococcal nuclease domain-containing protein 1	Q78PY7	Y908	ADDADEFGYSR	Y9	1.063	0.381
Snip1	Smad nuclear-interacting protein 1	Q8BIZ6	S18	SGDALTTVVVK	S1	1.001	0.991
Snip1	Smad nuclear-interacting protein 1	Q8BIZ6	S18	HRSGDALTTVVVK	S3	1.030	0.717
Snip1	Smad nuclear-interacting protein 1	Q8BIZ6	S33	LSPEPVAHR	S2	1.057	0.653
Snip1	Smad nuclear-interacting protein 1	Q8BIZ6	S33	QERLSPEPVAHR	S5	0.997	0.976
Snip1	Smad nuclear-interacting protein 1	Q8BIZ6	S50	RPDAPAASLSPPAAEPGHSGHR	S10	1.028	0.665
Snip1	Smad nuclear-interacting protein 1	Q8BIZ6	S91	SQSPHYPMVK	S3	1.011	0.837
Snip1	Smad nuclear-interacting protein 1	Q8BIZ6	S91	SQSPHYPMVK	S3	1.027	0.929
Snip1	Smad nuclear-interacting protein 1	Q8BIZ6	S48,S50	RPDAPAASLSPPAAEPGHSGHR	S8,S10	0.996	0.975
Snip1	Smad nuclear-interacting protein 1	Q8BIZ6	S89,S91	SQSPHYPMVK	S1,S3	1.002	0.948
Snip1	Smad nuclear-interacting protein 1	Q8BIZ6	S89,S91	SQSPHYPMVK	S1,S3	1.024	0.706
Snip1	Smad nuclear-interacting protein 1	Q8BIZ6	Ambiguous	RSQSPHYPMVK	S/Y	1.069	0.558
Snn	Stannin	P61807	S49	ISQSEDEESIVGDGETK	S9	0.951	0.673
Snrk	SNF-related serine/threonine-protein kinase	Q8VDU5	S569	RDSSEGPPGSEGDGGQSKPSSGGVDK	S3	1.005	0.956
Snrnp200	U5 small nuclear ribonucleoprotein 200 kDa helicase	Q6P4T2	S225	EEASDDDMEGDEAVVR	S4	1.030	0.322
Snrnp200	U5 small nuclear ribonucleoprotein 200 kDa helicase	Q6P4T2	S225	EEASDDDMEGDEAVVR	S4	1.054	0.457
Snrnp70	U1 small nuclear ribonucleoprotein 70 kDa	Q62376	S226	DDTSRYDERPGPSPLPHR	S13	1.019	0.888
Snrnp70	U1 small nuclear ribonucleoprotein 70 kDa	Q62376	S226	YDERPGPSPLPHR	S8	1.043	0.563
Snrnp70	U1 small nuclear ribonucleoprotein 70 kDa	Q62376	S408	GGGGSGQDNGLEGLGSDGR	S5	0.951	0.602
Snrpa1	U2 small nuclear ribonucleoprotein A'	P57784	S239	SGPSDEGEEEIEDDTVTNGS	S4	0.953	0.451
Snrpa1	U2 small nuclear ribonucleoprotein A'	P57784	Ambiguous	RSGPSDEGEEEIEDDTVTNGS	S/T	1.013	0.865
Snrpd2	Small nuclear ribonucleoprotein Sm D2	P62317	T12	SEMTPEELQKR	T4	1.083	0.363
Snrpe	Small nuclear ribonucleoprotein E	P62305	S91	GDNITLLQSVSN	S11	0.830	0.294
Snta1	Alpha-1-syntrophin	Q61234	S183	NSAGGTSVGWDSPPASPLQR	S12	1.010	0.730
Snta1	Alpha-1-syntrophin	Q61234	S195	QPSSPGPQPR	S4	0.958	0.459
Snta1	Alpha-1-syntrophin	Q61234	S183,S187	NSAGGTSVGWDSPPASPLQR	S12,S16	0.874	0.029
Sntb1	Beta-1-syntrophin	Q99L88	S86	GSGTGHPVPGVAQAPDSPAGVR	S17	0.989	0.939
Sntb2	Beta-2-syntrophin	B7ZNU9	S75	GLGPPSPPAPPR	S6	1.068	0.082
Sntb2	Beta-2-syntrophin	B7ZNU9	S90	GPAGEASASPPVR	S9	1.050	0.243
Sntb2	Beta-2-syntrophin	B7ZNU9	S90	GPAGEASASPPVRR	S9	0.991	0.895
Sntb2	Beta-2-syntrophin	B7ZNU9	S90	GLGPPSPPAPPRGPAGEASASPPVR	S21	1.094	0.281
Sntb2	Beta-2-syntrophin	B7ZNU9	S75,S90	GLGPPSPPAPPRGPAGEASASPPVRR	S6,S21	0.999	0.979
Sntb2	Beta-2-syntrophin	B7ZNU9	S75,S90	GLGPPSPPAPPRGPAGEASASPPVR	S6,S21	1.027	0.585
Snu13	NHP2-like protein 1	Q9D0T1	S122	QQIQSIQQSIER	S9	0.940	0.475
Snw1	SNW domain-containing protein 1	A0A0B4J1E2	S224	GPPSPPAPVMHSPSR	S4	1.004	0.918
Snw1	SNW domain-containing protein 1	A0A0B4J1E2	S224,S232	GPPSPPAPVMHSPSRK	S4,S12	1.017	0.537
Snw1	SNW domain-containing protein 1	A0A0B4J1E2	S224,S232	GPPSPPAPVMHSPSRK	S4,S12	0.988	0.752
Snw1	SNW domain-containing protein 1	A0A0B4J1E2	S224,S232	GPPSPPAPVMHSPSRK	S4,S12	1.015	0.659
Snw1	SNW domain-containing protein 1	A0A0B4J1E2	S224,S232	GPPSPPAPVMHSPSR	S4,S12	0.973	0.740
Snx11	Sorting nexin-11	Q91WL6	S192	RSSPSPPLSEEK	S3	1.004	0.970

Snx11	Sorting nexin-11	Q91WL6	S194,S	RSSPSPPLSEEK	S5,S	0.976	0.799
Snx15	Sorting nexin-15	Q91WE1	T148	ILPPPLIPTPPPDEAR	T9	0.935	0.808
Snx16	Sorting nexin-16	Q8C080	S51	GQLEDSAVGSLK	S10	1.007	0.957
Snx16	Sorting nexin-16	Q8C080	S70	QTNVQDQMDSASSMCGSPLIR	S17	1.068	0.136
Snx16	Sorting nexin-16	Q8C080	S70	QTNVQDQMDSASSMCGSPLIR	S17	0.947	0.162
Snx16	Sorting nexin-16	Q8C080	S32,S/T	SSSFGSVSTSSTSSK	S6,S/T	1.114	0.170
Snx16	Sorting nexin-16	Q8C080	S70,S/T	QTNVQDQMDSASSMCGSPLIR	S17,S/T	1.088	0.160
Snx16	Sorting nexin-16	Q8C080	S70,S/T	QTNVQDQMDSASSMCGSPLIR	S17,S/T	0.947	0.639
Snx16	Sorting nexin-16	Q8C080	Ambiguous	SSSFGSVSTSSTSSK	S/T	1.012	0.810
Snx17	Sorting nexin-17	Q8BVL3	S336	VTSSVPLPSGGTSSPSR	S14	0.991	0.812
Snx17	Sorting nexin-17	Q8BVL3	S409	RSDSQQAVK	S4	1.172	0.078
Snx17	Sorting nexin-17	Q8BVL3	S415	RSDSQQAVKSPPLLESPDASR	S10	1.085	0.256
Snx17	Sorting nexin-17	Q8BVL3	S415	SDSQQAVKSPPLLESPDASR	S9	1.000	0.986
Snx17	Sorting nexin-17	Q8BVL3	S421	SPPLLESPDASR	S7	0.984	0.728
Snx17	Sorting nexin-17	Q8BVL3	S415,S421	RSDSQQAVKSPPLLESPDASR	S10,S16	1.161	0.006
Snx17	Sorting nexin-17	Q8BVL3	S415,S421	SDSQQAVKSPPLLESPDASR	S9,S15	1.047	0.248
Snx17	Sorting nexin-17	Q8BVL3	S409,S415,S421	RSDSQQAVKSPPLLESPDASR	S4,S10,S16	1.085	0.215
Snx18	Sorting nexin	Q8C788	S20	SENPGEISLR	S8	1.116	0.043
Snx19	Sorting nexin-19	Q6P4T1	S704	SEPQSPTEELSEAENESKPQTEGK	S5	1.123	0.097
Snx19	Sorting nexin-19	Q6P4T1	S704,T706	SEPQSPTEELSEAENESKPQTEGK	S5,T7	1.181	0.124
Snx2	Sorting nexin-2	Q9CWK8	S119	SISAPVIFDR	S3	0.941	0.618
Snx2	Sorting nexin-2	Q9CWK8	S226	EDSSSTEFVEK	S3	1.117	0.362
Snx2	Sorting nexin-2	Q9CWK8	S264	QFLESSELPR	S5	0.913	0.139
Snx2	Sorting nexin-2	Q9CWK8; Q9WV80	S365;S368	ALSQLAEVEEK	S3	0.985	0.933
Snx24	Sorting nexin-24	Q9CRB0	S113	AESCGSFDETESEESSK	S3	0.938	0.098
Snx25	Sorting nexin-25	E9Q1K0	S420	ILGGPAYDQQEDGASDEGEGPQSQK	S15	1.012	0.966
Snx27	Sorting nexin-27	Q3UHD6	S49	SESGYGFNVR	S3	1.107	0.130
Snx29	Sorting nexin-29	Q9D3S3	S268	VTNIVSFDDDEEEQGTGDTLK	S6	1.037	0.849
Snx29	Sorting nexin-29	Q9D3S3	S291	KMPGTAESSEENSDR	S8	1.054	0.633
Snx29	Sorting nexin-29	Q9D3S3	S330	IDSASLNGELGYQK	S5	1.015	0.549
Snx29	Sorting nexin-29	Q9D3S3	S344	SIDDDVDENEEDAYR	S1	0.920	0.579
Snx29	Sorting nexin-29	Q9D3S3	S344	LDVKSIDDDVDENEEDAYR	S5	0.987	0.854
Snx29	Sorting nexin-29	Q9D3S3	S642	GPVPGDLSQTSEDQSLSDFEISNR	S8	0.992	0.923
Snx29	Sorting nexin-29	Q9D3S3	S815	NVEPQSGDL	S6	0.976	0.263
Snx29	Sorting nexin-29	Q9D3S3	S642,T/S	GPVPGDLSQTSEDQSLSDFEISNR	S8,S/T	1.304	0.539
Snx30	Sorting nexin-30	Q8CE50	S16	ALPSTGPQSLR	S9	1.083	0.202
Snx30	Sorting nexin-30	Q8CE50	S40	DMPHPLAGSSSEEAVGGDSTPSPDLLMAR	S22	0.995	0.961
Snx30	Sorting nexin-30	Q8CE50	S40	DMPHPLAGSSSEEAVGGDSTPSPDLLMAR	S22	0.999	0.978
Snx30	Sorting nexin-30	Q8CE50	Ambiguous	DMPHPLAGSSSEEAVGGDSTPSPDLLMAR	S/T	1.519	0.103
Snx33	Sorting nexin-33	Q4VAA7	S169	QDSLASAK	S3	1.145	0.010
Snx33	Sorting nexin-33	Q4VAA7	S169	APLERQDSLASAK	S8	1.064	0.469
Snx6	Sorting nexin-6	Q6P8X1	S316	SLVDYENANK	S1	1.293	0.002
Snx6	Sorting nexin-6	Q6P8X1	S316	SRSLVDYENANK	S3	1.385	0.107
Snx7	Sorting nexin-7	F8WI30	S8	LASGSSELAVGESPR	S3	1.133	0.020
Snx7	Sorting nexin-7	F8WI30	S8,S18	LASGSSELAVGESPR	S3,S13	1.077	0.436
Snx9	Sorting nexin-9	Q91VH2	S175	SSSPYFKDSEPAEAGGIQR	S3	0.902	0.383
Soat1	Sterol O-acyltransferase 1	Q61263	S10	LSKSGENPEQDEAQK	S4	1.083	0.075
Socs6	Suppressor of cytokine signaling 6	Q9JLY0	S104	GGTASTDEDTFSSASAPGGLK	S15	1.023	0.821
Socs6	Suppressor of cytokine signaling 6	Q9JLY0	S159	ALVHAASPGPVNGVR	S7	1.114	0.255
Socs6	Suppressor of cytokine signaling 6	Q9JLY0	S189	DLQPEPRPESR	S10	1.021	0.843
Socs6	Suppressor of cytokine signaling 6	Q9JLY0	S309	GGHDDAPPLSPLLPPMQNNPIQR	S10	0.909	0.659
Sod1	Superoxide dismutase [Cu-Zn]	P08228	S99	DGVANVSIEDR	S7	1.036	0.496
Sod1	Superoxide dismutase [Cu-Zn]	P08228	Ambiguous	VISLSGEHSIIGR	S	0.779	0.201
Soga1	Protein SOGA1	A2ACV6	S1251	VYYSPPVAR	S4	1.005	0.906
Soga1	Protein SOGA1	A2ACV6	S173	TGQPPQPAQSGQQPPRPPASPDEPSVAASSVGSSR	S20	1.089	0.082
Son	Protein SON	H9KV00	S1723	ESAQAVAVALSPK	S11	0.956	0.751
Son	Protein SON	H9KV00	S1794	SAASPVVISIPER	S4	0.927	0.022
Son	Protein SON	H9KV00	S94	CVSVQTDPTDEVPTKK	S3	1.287	0.007
Son	Protein SON	H9KV00	T954	LGHDPYRLTPDPYR	T9	1.037	0.646
Sorbs1	Sorbin and SH3 domain-containing protein 1	A0A286YCI8	S58	GTPSSSPVSPQESPK	S9	0.976	0.789
Sorbs1	Sorbin and SH3 domain-containing protein 1	A0A286YCI8	T184	RAGEQDPVPTPAELTSPGR	T15	1.053	0.469
Sorbs1	Sorbin and SH3 domain-containing protein 1	A0A286YCI8	T286	SATLPLPAR	T3	1.066	0.627
Sorbs1	Sorbin and SH3 domain-containing protein 1	A0A286YCI8	Ambiguous	STQDLSDVSTDEVGIPLR	S/T	1.106	0.600
Sorbs1	Sorbin and SH3 domain-containing protein 1	A0A286YCI8	Ambiguous	AGEQDPVPTPAELTSPGR	T/S	0.834	0.192
Sord	Sorbitol dehydrogenase	Q64442	S169	RGSVSLGNK	S3	1.004	0.942
Sort1	Sortilin	Q6PHU5	S819	SGYHDDSDEDLLE	S7	1.005	0.856
Sos1	Son of sevenless homolog 1	Q62245	S1082	IPESETESTASAPNSPR	S15	0.996	0.953
Sos1	Son of sevenless homolog 1	Q62245	S1196	TSISDPPESPPLLPPREPVR	S9	0.947	0.092
Sos1	Son of sevenless homolog 1	Q62245	S1078,S1082	IPESETESTASAPNSPR	S11,S15	1.002	0.944
Sos1	Son of sevenless homolog 1	Q62245	Ambiguous	SASVSSISLSK	S	1.169	0.465
Sos2	Son of sevenless homolog 2	Q02384	S1316	ELSHPPLYR	S3	1.222	0.025
Sos2	Son of sevenless homolog 2	Q02384	T1262	DVSTCPNSPSTPPTTPSPR	T11	0.913	0.372
Sos2	Son of sevenless homolog 2	Q02384	Ambiguous	QNSSPLLPK	S	1.122	0.400
Sowahc	Ankyrin repeat domain-containing protein SOWAHC	Q8C0J6	S391	NLVGALDEDDGDSPAAR	S13	1.000	1.000
Sowahc	Ankyrin repeat domain-containing protein SOWAHC	Q8C0J6	S82	FCTGDSPPLEAK	S6	1.017	0.383
Sowahc	Ankyrin repeat domain-containing protein SOWAHC	Q8C0J6	S82	RFCTGDSPPLEAK	S7	0.922	0.076
Sowahc	Ankyrin repeat domain-containing protein SOWAHC	Q8C0J6	T189	LPPQGEAEGGSSPSGPNTPR	T18	1.030	0.589
Sowahc	Ankyrin repeat domain-containing protein SOWAHC	Q8C0J6	S185,T189	LPPQGEAEGGSSPSGPNTPR	S14,T18	0.963	0.622
Sowahc	Ankyrin repeat domain-containing protein SOWAHC	Q8C0J6	Ambiguous	DLVLGSSPQLK	S	1.173	0.242
Sowahc	Ankyrin repeat domain-containing protein SOWAHC	Q8C0J6	Ambiguous	DSPPQEVEAVSWASGPGSSENLK	S	0.920	0.737
Sp100	Nuclear autoantigen Sp-100	Q8C405	S190	NSNLMQANQTENHQLAESPGHLDSCELQVQLNNR	S18	1.105	0.378
Sp100	Nuclear autoantigen Sp-100	Q8C405	S314	GGDTSDTESSIIIR	S5	1.002	0.974
Sp100	Nuclear autoantigen Sp-100	Q8C405	S314	DRGGDTSDTESSIIIR	S7	1.003	0.959
Sp100	Nuclear autoantigen Sp-100	Q8C405	S314	DRGGDTSDTESSIIIRR	S7	0.942	0.703
Sp100	Nuclear autoantigen Sp-100	Q8C405	T209	DATPESCSLLPQNEER	T3	1.011	0.810
Sp100	Nuclear autoantigen Sp-100	Q8C405	S314,S/T	DRGGDTSDTESSIIIR	S7,S/T	1.001	0.983
Sp100	Nuclear autoantigen Sp-100	Q8C405	T313,S319	GGDTSDTESSIIIR	T4,S10	0.960	0.663
Sp100	Nuclear autoantigen Sp-100	Q8C405	Ambiguous	DRGGDTSDTESSIIIRR	T/S	0.973	0.838
Sp110	Sp110 nuclear body protein	Q8BVK9	S214	EMPHSPSGPESVVK	S5	0.954	0.375
Sp110	Sp110 nuclear body protein	Q8BVK9	S226	EMPHSPSGPESVVKDDSPAANDLEMAR	S17	1.052	0.106
Sp110	Sp110 nuclear body protein	Q8BVK9	S277	QDEMMGVASPGHGVQEK	S9	0.980	0.787
Sp110	Sp110 nuclear body protein	Q8BVK9	S322	AQTSNSQEISK	S6	1.075	0.348
Sp110	Sp110 nuclear body protein	Q8BVK9	S214,S	EMPHSPSGPESVVKDDSPAANDLEMAR	S5,S	0.885	0.055
Sp110	Sp110 nuclear body protein	Q8BVK9	S214,S216	EMPHSPSGPESVVKDDSPAANDLEMAR	S5,S7	0.854	0.224
Sp110	Sp110 nuclear body protein	Q8BVK9	Ambiguous	EMPHSPSGPESVVKDDSPAANDLEMAR	S	0.963	0.490
Sp2	Transcription factor Sp2	Q8C5J0	T56	IGPPAVEAAVTPPAPPQPTPR	T11	0.995	0.971
Sp4	Transcription factor Sp4	A0A1Y7VJR5	Ambiguous	ENNVSQPASSSSSSSSNNGSSSPTK	S/T	0.866	0.511
Sp7	Transcription factor Sp7	Q8VI67	S419	AHGGSPEQSNLLEI	S5	1.124	0.524
Sp7	Transcription factor Sp7	Q8VI67	Ambiguous	SSTSPAPPEK	S/T	1.019	0.463
Spag7	Sperm-associated antigen 7	Q7TNE3	S114	EFAPSDEELDSYR	S5	1.031	0.435
Spag7	Sperm-associated antigen 7	Q7TNE3	S158	QEEEAAQQGPAVVSPASDYKDK	S14	0.962	0.574
Spag7	Sperm-associated antigen 7	Q7TNE3	S219	LRQSGEELPTTS	S4	1.236	0.003
Spag9	C-Jun-amino-terminal kinase-interacting protein 4	Q58A65	S1188	TSGTPGNRPGSVIR	S11	1.014	0.780
Spag9	C-Jun-amino-terminal kinase-interacting protein 4	Q58A65; F6SH61	S705;S541	DGGSVVGASVFYK	S4	1.141	0.283
Spag9	C-Jun-amino-terminal kinase-interacting protein 4	Q58A65; F6SH61	S815;S651	ETDYPAGEELSESGQVDK	S13	0.999	0.975
Spag9	C-Jun-amino-terminal kinase-interacting protein 4	Q58A65; F6SH61	T226;T62	GGETPGSEQWK	T4	1.323	0.000
Spag9	C-Jun-amino-terminal kinase-interacting protein 4	Q58A65; F6SH61	T586;T422	YNAPTSHVTPSVK	T9	0.957	0.614
Spag9	C-Jun-amino-terminal kinase-interacting protein 4	Q58A65; F6SH61	T595;T431	SSTLSQLPGDK	T3	0.986	0.615
Spag9	C-Jun-amino-terminal kinase-interacting protein 4	Q58A65; F6SH61	S730,S;S566,S	SASQSSLDKLDQELK	S3,S	1.023	0.596
Spag9	C-Jun-amino-terminal kinase-interacting protein 4	Q58A65; F6SH61	T292,T/S;T128,T/S	ATTPASTANSDVSAIPPDTPSKEDNEGFVK	T19,T/S	1.446	0.128
Spag9	C-Jun-amino-terminal kinase-interacting protein 4	Q58A65; F6SH61	Ambiguous	GSSTPTKGIENK	S/T	0.976	0.701
Spag9	C-Jun-amino-terminal kinase-interacting protein 4	Q58A65; F6SH61	Ambiguous	ATTPASTANSDVSAIPPDTPSKEDNEGFVK	T/S	1.303	0.139
Spast	Spastin	Q9QYY8	S99	SSGTAPAPASPSPPEPGPGGEAESVR	S10	0.985	0.830
Spast	Spastin	Q9QYY8	T301	TNKPSTPTTAVR	T6	0.865	0.009
Spata2	Spermatogenesis-associated protein 2	Q8K004	S247	SVDAYDSYWESR	S1	0.967	0.766
Spata2l	Spermatogenesis-associated protein 2-like protein	Q8BNN1	S329	SGDLAPPESPSSPGQASPR	S12	0.986	0.817
Spata2l	Spermatogenesis-associated protein 2-like protein	Q8BNN1	Ambiguous	ELSRSGDLAPPESPSSPGQASPR	S	0.835	0.003
Spata5	ATPase family protein 2 homolog	Q3UMC0	S18	EGAEGASPSLSAAPSR	S7	1.063	0.635
Spata5	ATPase family protein 2 homolog	Q3UMC0	T271	AGEVLLDVTQSPR	T9	0.896	0.791
Spata6	Spermatogenesis-associated protein 6	Q3U6K5	S354	HCASPVLNR	S4	0.886	0.494
Spata6	Spermatogenesis-associated protein 6	Q3U6K5	S424	DSAYDSDPEYSSFQRPR	S6	0.988	0.794
Spata6	Spermatogenesis-associated protein 6	Q3U6K5	S437	GSFHLDDGECWSNR	S2	0.973	0.653
Spdl1	Protein Spindly	Q923A2	S518	QPASSCVQPASLSPHK	S13	0.973	0.325
Specc1	Cytospin-B	Q5SXY1	S111	ELSVTISR	S3	1.115	0.417
Specc1	Cytospin-B	Q5SXY1	S119	ERSVPRGPSSSK	S3	0.926	0.037
Specc1	Cytospin-B	Q5SXY1	S133	LGSSPTSSCNPTPTK	S4	0.994	0.793
Specc1	Cytospin-B	Q5SXY1	S355	GSPTGSSPNNASELSLASLTEK	S2	1.144	0.488
Specc1	Cytospin-B	Q5SXY1	S37	SSTSLAFESR	S1	0.944	0.426
Specc1	Cytospin-B	Q5SXY1	S37	SSKSSTSLAFESR	S4	0.907	0.380
Specc1	Cytospin-B	Q5SXY1	S654	ASESDAEIK	S4	1.105	0.078
Specc1	Cytospin-B	Q5SXY1	S654	ASESDAEIKDMK	S4	1.106	0.342
Specc1	Cytospin-B	Q5SXY1	S76	TSTSGAISELTESR	S2	0.910	0.048
Specc1	Cytospin-B	Q5SXY1	S847	SPLSGIPVR	S1	1.125	0.315
Specc1	Cytospin-B	Q5SXY1	T77	KTSTSGAISELTESR	T4	0.924	0.397
Specc1	Cytospin-B	Q5SXY1	Ambiguous	GVYVNRTSPAPSDSATTVK	S/T/Y	1.094	0.279
Specc1	Cytospin-B	Q5SXY1	Ambiguous	ASSEDTLNKPGSASSGVAR	S/T	0.993	0.848
Specc1	Cytospin-B	Q5SXY1	Ambiguous	TSGSSSSDVTK	S/T	0.972	0.629
Specc1l	Cytospin-A	A0A0R4J0J8	S849	SSTSSEPTPTVK	S1	0.997	0.959

Specc1l	Cytospin-A	A0A0R4J0J8	S886	TPLSPSPMK	S4	1.020	0.856
Specc1l	Cytospin-A	A0A0R4J0J8	Ambiguous	RSSTSSEPTPTVK	S/T	1.065	0.093
Speg	Striated muscle-specific serine/threonine-protein kinase	E9QQ25	S1177	MPSIPEEPEHGDLER	S3	1.045	0.152
Speg	Striated muscle-specific serine/threonine-protein kinase	E9QQ25	S2004	TPSKDQEAPSPEALPSPGQESPDGPSPR	S21	0.983	0.870
Speg	Striated muscle-specific serine/threonine-protein kinase	E9QQ25	S2042	AASVELPQRR	S3	1.089	0.413
Speg	Striated muscle-specific serine/threonine-protein kinase	E9QQ25	S2288	VASPPPGVSEK	S3	1.085	0.038
Speg	Striated muscle-specific serine/threonine-protein kinase	E9QQ25	S2396	SRSVQDLR	S3	1.015	0.810
Speg	Striated muscle-specific serine/threonine-protein kinase	E9QQ25	S2451	SGDGESSEGGSSARASPVLAVR	S16	1.081	0.446
Speg	Striated muscle-specific serine/threonine-protein kinase	E9QQ25	S2451	ASPVLAVR	S2	1.010	0.900
Speg	Striated muscle-specific serine/threonine-protein kinase	E9QQ25	S2499	ATSEGESLR	S3	0.963	0.416
Speg	Striated muscle-specific serine/threonine-protein kinase	E9QQ25	S2777	GTPDSPAQPAAAPR	S5	1.016	0.443
Speg	Striated muscle-specific serine/threonine-protein kinase	E9QQ25	S2943	EVVSSPTPESTTLR	S4	1.043	0.588
Speg	Striated muscle-specific serine/threonine-protein kinase	E9QQ25	S3261	SYPGSP	S5	1.112	0.237
Speg	Striated muscle-specific serine/threonine-protein kinase	E9QQ25	T2847	AVGPPPATPPRK	T8	0.954	0.090
Speg	Striated muscle-specific serine/threonine-protein kinase	E9QQ25	T2847	AVGPPPATPPR	T8	1.089	0.321
Speg	Striated muscle-specific serine/threonine-protein kinase	E9QQ25	S1993,S1999	TPSKDQEAPSPEALPSPGQESPDGPSPR	S10,S16	0.996	0.946
Spen	Msx2-interacting protein	A2ADB0	S1582	QASEGANSTSDSVQEPVVLFHSR	S3	1.016	0.817
Spen	Msx2-interacting protein	A2ADB0	S2129	AAGQAADKEAGPAAASPQESESPQK	S16	1.188	0.269
Spen	Msx2-interacting protein	A2ADB0	S750	SQSPVHLR	S3	1.021	0.138
Spen	Msx2-interacting protein	A2ADB0	T2375	ASEAEQTQSESPAAEEATAATPEAPQEEK	T21	0.909	0.293
Spen	Msx2-interacting protein	A2ADB0	S748,S750	RPIERSQSPVHLR	S6,S8	1.060	0.637
Spen	Msx2-interacting protein	A2ADB0	Ambiguous	ASEAEQTQSESPAAEEATAATPEAPQEEK	T/S	1.169	0.086
Spg21	Maspardin	Q9CQC8	S304	LGLSQEEP	S4	1.049	0.348
Spg21	Maspardin	Q9CQC8	S304	GRLGLSQEEP	S6	0.986	0.647
Sphk1	Sphingosine kinase 1	Q8CI15	S225	RPASTLVQK	S4	0.971	0.519
Sphk2	Sphingosine kinase 2	Q9JIA7	S379	SELVLAPAPAPAATHSPLHR	S16	1.039	0.654
Sphk2	Sphingosine kinase 2	Q9JIA7	S364,S379	AKSELVLAPAPAPAATHSPLHR	S3,S18	0.998	0.985
Spi1	Transcription factor PU.1	P17433	S148	QSPPLEVSDGEADGLEPGPGLLHGETGSK	S8	1.150	0.264
Spice1	Spindle and centriole-associated protein 1	Q8C804	T236	IATQSQRTPPGSPSSELSAEDQK	T8	0.847	0.336
Spice1	Spindle and centriole-associated protein 1	Q8C804	T236,S/T	IATQSQRTPPGSPSSELSAEDQK	T8,S/T	0.996	0.937
Spindoc	Spindlin interactor and repressor of chromatin-binding protein	Q05AH6	S313	AAEGLPQPQNPSSASPPGLR	S15	1.005	0.951
Spire1	Protein spire homolog 1	D3YTL8	S229	LRPVSPEEIR	S5	1.022	0.733
Spire1	Protein spire homolog 1	D3YTL8	S229	LRPVSPEEIRR	S5	1.006	0.952
Spire1	Protein spire homolog 1	D3YTL8	S245	LDVTTPESPK	S8	1.119	0.352
Spire1	Protein spire homolog 1	D3YTL8	S293	APTLAELDSSDSEEEK	S10	0.959	0.734
Spire1	Protein spire homolog 1	D3YTL8	S622	KTQSFYMSSAGPSEYCPSER	S4	1.021	0.798
Spire1	Protein spire homolog 1	D3YTL8	T338	FLPISSTPQPERR	T7	0.862	0.127
Spire1	Protein spire homolog 1	D3YTL8	T338	FLPISSTPQPER	T7	1.123	0.257
Spn	Leukosialin	P15702	S371	GEEEPLVGSEDEAVETPTSDGPQAK	S9	1.006	0.944
Spn	Leukosialin	P15702	S394	DEAAPQSL	S7	1.278	0.011
Spp1	Osteopontin	F8WIP8	S148	GDSLAYGLR	S3	0.991	0.881
Spp1	Osteopontin	F8WIP8	S163	SFQVSDEQYPDATDEDLTSHMK	S5	0.983	0.855
Spp1	Osteopontin	F8WIP8	S232	LEHSKESQESADQSDVIDSQASSK	S7	1.032	0.795
Spp1	Osteopontin	F8WIP8	S244	ESQESADQSDVIDSQASSK	S14	0.941	0.499
Spp1	Osteopontin	F8WIP8	S251	ASLEHQSHK	S2	1.025	0.736
Spp1	Osteopontin	F8WIP8	S272	LVLDPKSKEDDR	S7	0.911	0.576
Spp1	Osteopontin	F8WIP8	S62	QNLLAPQNAVSSEEK	S11	1.041	0.694
Spp1	Osteopontin	F8WIP8	S229,S232	LEHSKESQESADQSDVIDSQASSK	S4,S7	1.075	0.365
Spp1	Osteopontin	F8WIP8	S284,S	ISHELESSSSEVN	S2,S	0.927	0.169
Spp1	Osteopontin	F8WIP8	S284,S289	FRISHELESSSSEVN	S4,S9	1.037	0.433
Spp1	Osteopontin	F8WIP8	Ambiguous	ISHELESSSSEVN	S	1.059	0.020
Spp1	Osteopontin	F8WIP8	Ambiguous	SFQVSDEQYPDATDEDLTSHMK	S/T/Y	0.931	0.641
Sppl2a	Signal peptide peptidase-like 2A	Q9JJF9	Ambiguous	GSSYQVMDHLDYSTNEENPVTTDEQIVQQ	S/T/Y	1.158	0.714
Sppl2a	Signal peptide peptidase-like 2A	Q9JJF9	Ambiguous	GSSYQVMDHLDYSTNEENPVTTDEQIVQQ	T/S/Y	0.796	0.272
Sppl2b	Signal peptide peptidase-like 2B	Q3TD49	S521	AVGSSLSEQPPSEELAK	S5	1.187	0.348
Spred1	Sprouty-related, EVH1 domain-containing protein 1	Q924S8	S239	HVSFQDEDEIVR	S3	1.091	0.152
Spred2	Sprouty-related, EVH1 domain-containing protein 2	Q5SRF8	S194	TISSPTSCEHR	S3	0.935	0.583
Spryd3	SPRY domain-containing 3	E9Q9B3	S339	DYILDSEGDSDDSCDTVILSPTAR	S6	1.204	0.413
Sptan1	Spectrin alpha chain, non-erythrocytic 1	A3KGU7	S1217	SLQQLAEER	S1	1.138	0.345
Sptbn1	Spectrin beta chain, non-erythrocytic 1	Q62261	S2102	RPPSPDPNTK	S4	1.007	0.862
Sptbn1	Spectrin beta chain, non-erythrocytic 1	Q62261	S2137	GDQVSQNGLPAEQGSPR	S15	1.054	0.352
Sptbn1	Spectrin beta chain, non-erythrocytic 1	Q62261	S2137	VSEEAESQQWDTSKGDQVSQNGLPAEQGSPR	S29	0.975	0.759
Sptbn1	Spectrin beta chain, non-erythrocytic 1	Q62261	S2168	ESSPVPSPTLDRK	S7	1.046	0.551
Sptbn1	Spectrin beta chain, non-erythrocytic 1	Q62261	T2186	SALPAQSAATLPAR	T10	1.087	0.218
Sptbn1	Spectrin beta chain, non-erythrocytic 1	Q62261	S2164,S2168	ESSPVPSPTLDR	S3,S7	0.977	0.614
Sptbn1	Spectrin beta chain, non-erythrocytic 1	Q62261	S2164,S2168	ESSPVPSPTLDRK	S3,S7	1.045	0.126
Sptbn1	Spectrin beta chain, non-erythrocytic 1	Q62261	S2160,S2164,S2168	TSSKESSPVPSPTLDR	S3,S7,S11	0.994	0.932
Sptbn1	Spectrin beta chain, non-erythrocytic 1	Q62261	S2168,S/T	TSSKESSPVPSPTLDRK	S11,S/T	1.056	0.681
Sqstm1	Sequestosome-1	Q64337	S152	CSVCPDYDLCSVCEGK	S11	0.980	0.883
Sqstm1	Sequestosome-1	Q64337	S24	RFSFCFSPEPEAEAQAAAGPGPCER	S3	0.911	0.673
Sqstm1	Sequestosome-1	Q64337	S308	SNTQPSSCSSEVSKPDGAGEGPAQSLTEQMK	S25	1.493	0.003
Sqstm1	Sequestosome-1	Q64337	T269	SRLTPTTPESSSTGTEDK	T4	1.019	0.560
Sqstm1	Sequestosome-1	Q64337	T272	LTPTTPESSSTGTEDK	T5	1.097	0.294
Sqstm1	Sequestosome-1	Q64337	S357,S/T	EVDPSTGELQSLQMPESEGPSSLDPSQEGPTGLK	S11,S/T	1.150	0.033
Sqstm1	Sequestosome-1	Q64337	T269,T271	SRLTPTTPESSSTGTEDK	T4,T6	1.044	0.350
Sqstm1	Sequestosome-1	Q64337	Ambiguous	EVDPSTGELQSLQMPESEGPSSLDPSQEGPTGLK	S/T	1.145	0.002
Sqstm1	Sequestosome-1	Q64337	Ambiguous	EVDPSTGELQSLQMPESEGPSSLDPSQEGPTGLK	S/T	1.236	0.239
Sra1	Steroid receptor RNA activator 1	Q80VJ2	S60	VAAPQDGSPR	S8	0.957	0.630
Sra1	Steroid receptor RNA activator 1	Q80VJ2	S60	VAAPQDGSPRAPETSGPPPVDHPPPSSK	S8	1.064	0.148
Srbd1	S1 RNA-binding domain-containing protein 1	Q497V5	S174	EESEDSFTFGQSPVKR	S12	1.145	0.344
Src	Neuronal proto-oncogene tyrosine-protein kinase Src	P05480	S17	SLEPSENVHGAGGAFPASQTPSKPASADGHR	S1	1.238	0.181
Srcap	Snf2-related CREBBP activator protein	A0A087WQ44	S1859	SGPPSPLPTATSFSGPRPR	S5	0.977	0.883
Srcap	Snf2-related CREBBP activator protein	A0A087WQ44	S263	AGSSPCLGSSSAASSPPPPVSR	S4	0.943	0.217
Srcap	Snf2-related CREBBP activator protein	A0A087WQ44	S579	DDERSEVDGGSGPPTPGPTTTLGPK	S11	0.955	0.300
Srcap	Snf2-related CREBBP activator protein	A0A087WQ44	S579	SEVDGGSGPPTPGPTTTLGPK	S7	1.106	0.175
Srcap	Snf2-related CREBBP activator protein	A0A087WQ44	T2999	DLPIPGTISSPGGGNLESR	T7	1.231	0.324
Srcap	Snf2-related CREBBP activator protein	A0A087WQ44	S1855,S1859	SGPPSPLPTATSFSGPRPR	S1,S5	0.916	0.515
Srcap	Snf2-related CREBBP activator protein	A0A087WQ44	S1855,S1859	DEPETLTLRSGPPSPLPTATSFSGPRPR	S10,S14	0.830	0.199
Srcap	Snf2-related CREBBP activator protein	A0A087WQ44	S273,S274	AGSSPCLGSSSAASSPPPPVSR	S14,S15	0.849	0.338
Srcap	Snf2-related CREBBP activator protein	A0A087WQ44	S579,T583	DDERSEVDGGSGPPTPGPTTTLGPK	S11,T15	0.889	0.181
Srcap	Snf2-related CREBBP activator protein	A0A087WQ44	S579,T583	SEVDGGSGPPTPGPTTTLGPK	S7,T11	0.942	0.343
Srebf1	Sterol regulatory element-binding protein 1	Q9WTN3	S448	ASSSGGSDSEPDSPAFEDSQVK	S9	0.923	0.463
Srek1	Splicing regulatory glutamine/lysine-rich protein 1	Q8BZX4	S465	AADEKGSPRTEDEGK	S7	1.032	0.704
Srek1	Splicing regulatory glutamine/lysine-rich protein 1	Q8BZX4	S486	TEDEGKVQHNGNCQPNEESPCSK	S19	0.894	0.536
Srek1	Splicing regulatory glutamine/lysine-rich protein 1	Q8BZX4	S486	TEDEGKVQHNGNCQPNEESPCSKADAV	S19	1.036	0.677
Srek1	Splicing regulatory glutamine/lysine-rich protein 1	Q8BZX4	S486	VQHNGNCQPNEESPCSKADAV	S13	1.106	0.632
Srek1ip1	Protein SREK1IP1	Q4V9W2	S144	SSPYHSELTK	S2	1.015	0.896
Srek1ip1	Protein SREK1IP1	Q4V9W2	S97	SYSSATEEDSAK	S4	0.908	0.003
Srek1ip1	Protein SREK1IP1	Q4V9W2	S94,S96	SYSSATEEDSAK	S1,S3	0.989	0.441
Srf	Serum response factor	Q9JM73	S220	ALIQTCLNSPDSPPR	S12	1.175	0.213
Srf	Serum response factor	Q9JM73	S220	ALIQTCLNSPDSPPRSDPTTDQR	S12	1.049	0.257
Srgap2	SLIT-ROBO Rho GTPase-activating protein 2	A0A087WNM1	S841	GIPRSPAHELPPSQEALR	S5	1.049	0.234
Srgap2	SLIT-ROBO Rho GTPase-activating protein 2	Q91Z67	S1061	TNATSPGVNSSASPQATDK	S13	1.041	0.478
Srgap2	SLIT-ROBO Rho GTPase-activating protein 2	Q91Z67; A0A087WNM1	S796;S796	SSPKSEIEVMSEPPEEK	S2	0.996	0.574
Srgap2	SLIT-ROBO Rho GTPase-activating protein 2	Q91Z67; A0A087WNM1	S805;S805	SEIEVMSEPPEEK	S7	1.033	0.516
Srgap2	SLIT-ROBO Rho GTPase-activating protein 2	Q91Z67; A0A087WNM1	Ambiguous	SSPKSEIEVMSEPPEEK	S	1.047	0.383
Srgap2	SLIT-ROBO Rho GTPase-activating protein 2	Q91Z67	Ambiguous	TNATSPGVNSSASPQATDKSCTV	T/S	1.020	0.837
Srp14	Signal recognition particle 14 kDa protein	P16254	S45	KSSVEGLEPAENK	S3	0.986	0.649
Srp68	Signal recognition particle subunit SRP68	Q8BMA6	S26	QIPGGGSGGGGSGSGGGGGGSGGGR	S21	0.912	0.404
Srp72	Signal recognition particle subunit SRP72	F8VQC1	S524	VDVEALENSPGATYIR	S9	0.911	0.374
Srp72	Signal recognition particle subunit SRP72	F8VQC1	S610	GTQGATAGASSELDASK	S10	0.914	0.203
Srp72	Signal recognition particle subunit SRP72	F8VQC1	T624,S/T	AVSSPPTSPRPGSAATISSSASNIVPPR	T7,S/T	1.001	0.974
Srp72	Signal recognition particle subunit SRP72	F8VQC1	S621,T/S	AVSSPPTSPRPGSAATISSSASNIVPPR	S4,T/S	0.961	0.547
Srpk1	SRSF protein kinase 1	O70551	S450	ADTPSGDEQEPNGALDSK	S5	0.980	0.671
Srpk1	SRSF protein kinase 1	O70551	S51	GSAPHSESDIPEQEEEILGSDDDEQEDPNDYCK	S20	1.034	0.743
Srpk1	SRSF protein kinase 1	O70551	Ambiguous	RPNKQEESESPVDRPLTENPPNK	S/T	1.078	0.661
Srpk1	SRSF protein kinase 1	O70551	Ambiguous	QEESESPVDRPLTENPPNK	S/T	1.020	0.766
Srpk1	SRSF protein kinase 1	O70551	Ambiguous	QDITQLEESIRADTPSGDEQEPNGALDSK	T/S	0.904	0.494
Srpk2	SRSF protein kinase 2	A0A0R4J124	S488	TVSASSTGDLPK	S3	1.061	0.209
Srpk2	SRSF protein kinase 2	A0A0R4J124	T332	AADLEDTTEEETAK	T7	0.984	0.910
Srpk2	SRSF protein kinase 2	A0A0R4J124	S488,S/T	TVSASSTGDLPK	S3,S/T	1.047	0.278
Srpk2	SRSF protein kinase 2	A0A0R4J124	Ambiguous	SSSSERPEPQQK	S	1.040	0.141
Srrm1	Serine/arginine repetitive matrix protein 1 (Fragment)	A2A983	S190	EKSPELPEPSV	S3	0.899	0.370
Srrm1	Serine/arginine repetitive matrix protein 1	E9PUK6	S220	SPELPEPSVR	S1	0.873	0.299
Srrm1	Serine/arginine repetitive matrix protein 1	E9PUK6	S220	EKSPELPEPSVR	S3	0.985	0.763
Srrm1	Serine/arginine repetitive matrix protein 1	E9PUK6	S220	KEKSPELPEPSVR	S4	0.971	0.377
Srrm1	Serine/arginine repetitive matrix protein 1	E9PUK6	S260	APKPEPVPEPKEPSPEK	S14	0.969	0.646
Srrm1	Serine/arginine repetitive matrix protein 1	E9PUK6	S391	LSPSASPPR	S6	1.054	0.595
Srrm1	Serine/arginine repetitive matrix protein 1	E9PUK6	S391	RLSPSASPPR	S7	1.120	0.113
Srrm1	Serine/arginine repetitive matrix protein 1	E9PUK6	S448	RESPSPAPKPR	S3	1.025	0.321
Srrm1	Serine/arginine repetitive matrix protein 1	E9PUK6	S461	KVELSESEEDKGSK	S5	1.041	0.112
Srrm1	Serine/arginine repetitive matrix protein 1	E9PUK6	S616	RYSPPIQR	S3	1.003	0.949
Srrm1	Serine/arginine repetitive matrix protein 1	E9PUK6	S624	RYSPSPPPK	S3	1.037	0.429
Srrm1	Serine/arginine repetitive matrix protein 1	E9PUK6	S635	TASPPPPPKR	S3	0.981	0.556
Srrm1	Serine/arginine repetitive matrix protein 1	E9PUK6	S635	TASPPPPPK	S3	0.995	0.854
Srrm1	Serine/arginine repetitive matrix protein 1	E9PUK6	S635	RTASPPPPPKR	S4	0.811	0.364
Srrm1	Serine/arginine repetitive matrix protein 1	E9PUK6	S645	RASPSPPPK	S3	1.015	0.905
Srrm1	Serine/arginine repetitive matrix protein 1	E9PUK6	S657	VSHSPPPK	S4	0.973	0.755

Srrm1	Serine/arginine repetitive matrix protein 1	E9PUK6	S664	QRSPTVTK	S3	1.034	0.611
Srrm1	Serine/arginine repetitive matrix protein 1	E9PUK6	S694	EARSPQPNK	S4	1.032	0.303
Srrm1	Serine/arginine repetitive matrix protein 1	E9PUK6	S714	APQTSSPPPVRR	S6	1.014	0.661
Srrm1	Serine/arginine repetitive matrix protein 1	E9PUK6	S714	APQTSSPPPVR	S6	1.016	0.702
Srrm1	Serine/arginine repetitive matrix protein 1	E9PUK6	S731	QSPSPSTRPIR	S2	1.030	0.675
Srrm1	Serine/arginine repetitive matrix protein 1	E9PUK6	S756	AASPSPQSVR	S3	1.022	0.532
Srrm1	Serine/arginine repetitive matrix protein 1	E9PUK6	S756	KAASPSPQSVR	S4	1.004	0.931
Srrm1	Serine/arginine repetitive matrix protein 1	E9PUK6	S774	SVSGSPEPAAK	S5	0.982	0.644
Srrm1	Serine/arginine repetitive matrix protein 1	E9PUK6	S815	SPTPSLSPAR	S7	1.227	0.044
Srrm1	Serine/arginine repetitive matrix protein 1	E9PUK6	S892	KETESEAEDDNLDDLER	S5	0.988	0.802
Srrm1	Serine/arginine repetitive matrix protein 1	E9PUK6	S920	KAQVSPQS	S5	1.011	0.877
Srrm1	Serine/arginine repetitive matrix protein 1	E9PUK6	T633	RTASPPPPPK	T2	1.050	0.556
Srrm1	Serine/arginine repetitive matrix protein 1	E9PUK6	T862	AVTIATPATAAPAAVSAATTTSAQEEPAAAPEPR	T9	0.849	0.034
Srrm1	Serine/arginine repetitive matrix protein 1	E9PUK6	S387,S391	RLSPSASPPR	S3,S7	0.987	0.702
Srrm1	Serine/arginine repetitive matrix protein 1	E9PUK6	S401,T404	HRPSSPATPPPK	S5,T8	0.982	0.417
Srrm1	Serine/arginine repetitive matrix protein 1	E9PUK6	S448,S450	RESPSPAPKPR	S3,S5	1.051	0.509
Srrm1	Serine/arginine repetitive matrix protein 1	E9PUK6	S461,S463	KVELSESEEDKGSK	S5,S7	0.975	0.687
Srrm1	Serine/arginine repetitive matrix protein 1	E9PUK6	S561,S563	SPSPPPAR	S1,S3	1.083	0.415
Srrm1	Serine/arginine repetitive matrix protein 1	E9PUK6	S561,S563	RSPSPPPAR	S2,S4	1.045	0.607
Srrm1	Serine/arginine repetitive matrix protein 1	E9PUK6	S561,S563	RRSPSPPPAR	S3,S5	1.003	0.948
Srrm1	Serine/arginine repetitive matrix protein 1	E9PUK6	S572,S574	SPSPAPPPPPPPPPPR	S1,S3	1.018	0.680
Srrm1	Serine/arginine repetitive matrix protein 1	E9PUK6	S572,S574	RSPSPAPPPPPPPPPPR	S2,S4	0.982	0.425
Srrm1	Serine/arginine repetitive matrix protein 1	E9PUK6	S572,S574	RSPSPAPPPPPPPPPPRR	S2,S4	1.012	0.784
Srrm1	Serine/arginine repetitive matrix protein 1	E9PUK6	S572,S574	RRSPSPAPPPPPPPPPPR	S3,S5	0.986	0.560
Srrm1	Serine/arginine repetitive matrix protein 1	E9PUK6	S591,T593	SPTPPPR	S1,T3	0.935	0.612
Srrm1	Serine/arginine repetitive matrix protein 1	E9PUK6	S591,T593	RSPTPPPR	S2,T4	1.013	0.870
Srrm1	Serine/arginine repetitive matrix protein 1	E9PUK6	S591,T593	RRSPTPPPR	S3,T5	0.891	0.033
Srrm1	Serine/arginine repetitive matrix protein 1	E9PUK6	S624,S626	YSPSPPPK	S2,S4	1.126	0.425
Srrm1	Serine/arginine repetitive matrix protein 1	E9PUK6	S624,S626	RYSPSPPPKR	S3,S5	0.963	0.480
Srrm1	Serine/arginine repetitive matrix protein 1	E9PUK6	S624,S626	RYSPSPPPK	S3,S5	1.031	0.438
Srrm1	Serine/arginine repetitive matrix protein 1	E9PUK6	S645,S647	RASPSPPPK	S3,S5	1.014	0.664
Srrm1	Serine/arginine repetitive matrix protein 1	E9PUK6	S645,S647	RASPSPPPKR	S3,S5	1.006	0.823
Srrm1	Serine/arginine repetitive matrix protein 1	E9PUK6	S655,S657	RVSHSPPPK	S3,S5	1.055	0.223
Srrm1	Serine/arginine repetitive matrix protein 1	E9PUK6	S672,S674	RSPSLSSK	S2,S4	0.971	0.790
Srrm1	Serine/arginine repetitive matrix protein 1	E9PUK6	S672,S674	RRSPSLSSK	S3,S5	1.033	0.308
Srrm1	Serine/arginine repetitive matrix protein 1	E9PUK6	S702,S704	RHSPSPRPR	S3,S5	1.001	0.985
Srrm1	Serine/arginine repetitive matrix protein 1	E9PUK6	S714,T/S	APQTSSPPPVR	S6,T/S	1.066	0.265
Srrm1	Serine/arginine repetitive matrix protein 1	E9PUK6	S731,S733	QSPSPSTRPIR	S2,S4	1.045	0.326
Srrm1	Serine/arginine repetitive matrix protein 1	E9PUK6	S743,T745	RVSRTPEPK	S3,T5	0.968	0.575
Srrm1	Serine/arginine repetitive matrix protein 1	E9PUK6	S756,S758	AASPSPQSVR	S3,S5	1.004	0.870
Srrm1	Serine/arginine repetitive matrix protein 1	E9PUK6	S756,S758	KAASPSPQSVR	S4,S6	0.995	0.879
Srrm1	Serine/arginine repetitive matrix protein 1	E9PUK6	S770,S774	VSSSRSVSGSPEPAAK	S6,S10	0.908	0.215
Srrm1	Serine/arginine repetitive matrix protein 1	E9PUK6	S772,S774	SVSGSPEPAAK	S3,S5	1.048	0.141
Srrm1	Serine/arginine repetitive matrix protein 1	E9PUK6	T600,S602	RTPSPPPR	T2,S4	0.895	0.235
Srrm1	Serine/arginine repetitive matrix protein 1	E9PUK6	T600,S602	RRTPSPPPR	T3,S5	1.020	0.539
Srrm1	Serine/arginine repetitive matrix protein 1	E9PUK6	T633,S635	TASPPPPPK	T1,S3	0.986	0.577
Srrm1	Serine/arginine repetitive matrix protein 1	E9PUK6	T633,S635	TASPPPPPKR	T1,S3	0.992	0.824
Srrm1	Serine/arginine repetitive matrix protein 1	E9PUK6	T633,S635	RRTASPPPPPK	T3,S5	0.952	0.608
Srrm1	Serine/arginine repetitive matrix protein 1	E9PUK6	T890,S892	KETESEAEDDNLDDLER	T3,S5	1.005	0.925
Srrm1	Serine/arginine repetitive matrix protein 1	E9PUK6	S387,S389,S391	RLSPSASPPR	S3,S5,S7	1.222	0.136
Srrm1	Serine/arginine repetitive matrix protein 1	E9PUK6	S723,S725,S731	GASASPQGRQSPSPSTRPIR	S3,S5,S11	0.918	0.299
Srrm1	Serine/arginine repetitive matrix protein 1	E9PUK6	S766,S770,S772	RVSSSRSVSGSPEPAAK	S3,S7,S9	1.053	0.302
Srrm1	Serine/arginine repetitive matrix protein 1	E9PUK6	Ambiguous	SPSPAPPPPPPPPPPR	S	0.960	0.718
Srrm1	Serine/arginine repetitive matrix protein 1	E9PUK6	Ambiguous	RRSPSPAPPPPPPPPPPR	S	1.164	0.405
Srrm1	Serine/arginine repetitive matrix protein 1	E9PUK6	Ambiguous	RVSSSRSVSGSPEPAAK	S	0.919	0.708
Srrm2	Serine/arginine repetitive matrix protein 2	Q8BTI8	S1068	SSSPVTELTAR	S3	1.027	0.212
Srrm2	Serine/arginine repetitive matrix protein 2	Q8BTI8	S1094	SGMSPEQSK	S1	1.031	0.328
Srrm2	Serine/arginine repetitive matrix protein 2	Q8BTI8	S1097	SGMSPEQSK	S4	1.016	0.565
Srrm2	Serine/arginine repetitive matrix protein 2	Q8BTI8	S1179	SGAGSPPGKR	S5	1.027	0.575
Srrm2	Serine/arginine repetitive matrix protein 2	Q8BTI8	S1179	SGAGSPPGK	S5	1.034	0.800
Srrm2	Serine/arginine repetitive matrix protein 2	Q8BTI8	S1216	SEQPLSQVLPSLSPEHK	S13	0.936	0.663
Srrm2	Serine/arginine repetitive matrix protein 2	Q8BTI8	S1269	AAETPAVASCWSGPQVSPEHK	S17	1.040	0.688
Srrm2	Serine/arginine repetitive matrix protein 2	Q8BTI8	S1278	ELSHSPPR	S5	0.977	0.853
Srrm2	Serine/arginine repetitive matrix protein 2	Q8BTI8	S1305	NSGPVSEVNTGFSPEVK	S13	1.044	0.547
Srrm2	Serine/arginine repetitive matrix protein 2	Q8BTI8	S1338	SSSELSPEVVEK	S1	0.922	0.067
Srrm2	Serine/arginine repetitive matrix protein 2	Q8BTI8	S1360	VSSPVLETVQQR	S3	0.995	0.759
Srrm2	Serine/arginine repetitive matrix protein 2	Q8BTI8	S1380	SSSASPELK	S5	1.057	0.113
Srrm2	Serine/arginine repetitive matrix protein 2	Q8BTI8	S1439	GRSECDSSPEPK	S8	0.943	0.316
Srrm2	Serine/arginine repetitive matrix protein 2	Q8BTI8	S1475	SGSESSVEQK	S3	1.001	0.978
Srrm2	Serine/arginine repetitive matrix protein 2	Q8BTI8	S1508	SGSSQELDGKPSASPQER	S14	0.971	0.133
Srrm2	Serine/arginine repetitive matrix protein 2	Q8BTI8	S1650	SSRSSPELTR	S5	0.958	0.520
Srrm2	Serine/arginine repetitive matrix protein 2	Q8BTI8	S1996	SRSPLAIR	S1	1.006	0.908
Srrm2	Serine/arginine repetitive matrix protein 2	Q8BTI8	S2084	SPGMLEPLGSAR	S1	1.276	0.261
Srrm2	Serine/arginine repetitive matrix protein 2	Q8BTI8	S2084	SPGMLEPLGSAR	S1	1.081	0.281
Srrm2	Serine/arginine repetitive matrix protein 2	Q8BTI8	S2084	CRSPGMLEPLGSAR	S3	0.945	0.510
Srrm2	Serine/arginine repetitive matrix protein 2	Q8BTI8	S2084	CRSPGMLEPLGSAR	S3	0.996	0.944
Srrm2	Serine/arginine repetitive matrix protein 2	Q8BTI8	S2224	TPAAAAAMNLASPR	S12	1.161	0.198
Srrm2	Serine/arginine repetitive matrix protein 2	Q8BTI8	S2224	TPAAAAAMNLASPR	S12	1.088	0.213
Srrm2	Serine/arginine repetitive matrix protein 2	Q8BTI8	S2335	MAPALSGANLTSPR	S12	0.985	0.797
Srrm2	Serine/arginine repetitive matrix protein 2	Q8BTI8	S2351	TSPLMLDR	S2	0.978	0.622
Srrm2	Serine/arginine repetitive matrix protein 2	Q8BTI8	S2381	ERAPSPASR	S5	1.001	0.976
Srrm2	Serine/arginine repetitive matrix protein 2	Q8BTI8	S2393	MVQASSQSLLPPAQDRPRSPVPSAFSDQSR	S8	1.119	0.457
Srrm2	Serine/arginine repetitive matrix protein 2	Q8BTI8	S2404	SPVPSAFSDQSR	S1	1.035	0.664
Srrm2	Serine/arginine repetitive matrix protein 2	Q8BTI8	S2535	VPSPTPVPK	S3	0.934	0.198
Srrm2	Serine/arginine repetitive matrix protein 2	Q8BTI8	S2535	RVPSPTPVPK	S4	1.006	0.764
Srrm2	Serine/arginine repetitive matrix protein 2	Q8BTI8	S2648	SLSYSPVER	S5	1.070	0.043
Srrm2	Serine/arginine repetitive matrix protein 2	Q8BTI8	S2656	QPSPQPSPR	S3	1.070	0.105
Srrm2	Serine/arginine repetitive matrix protein 2	Q8BTI8	S2656	RQPSPQPSPR	S4	1.006	0.812
Srrm2	Serine/arginine repetitive matrix protein 2	Q8BTI8	S333	QSSSPYEDKDKK	S4	0.955	0.253
Srrm2	Serine/arginine repetitive matrix protein 2	Q8BTI8	S333	QSSSPYEDKDK	S4	0.998	0.971
Srrm2	Serine/arginine repetitive matrix protein 2	Q8BTI8	S454	EISSSPTSK	S5	1.017	0.671
Srrm2	Serine/arginine repetitive matrix protein 2	Q8BTI8	S775	SLSGSSPCPK	S3	0.932	0.045
Srrm2	Serine/arginine repetitive matrix protein 2	Q8BTI8	S850	QSHSESSPDGEVK	S6	1.018	0.827
Srrm2	Serine/arginine repetitive matrix protein 2	Q8BTI8	T1370	VSSPVLETVQQRTPSRER	T13	0.854	0.198
Srrm2	Serine/arginine repetitive matrix protein 2	Q8BTI8	T1370	VSSPVLETVQQRTPSR	T13	1.037	0.519
Srrm2	Serine/arginine repetitive matrix protein 2	Q8BTI8	T1390	DGLPRTPSRR	T6	1.014	0.747
Srrm2	Serine/arginine repetitive matrix protein 2	Q8BTI8	T1428	DTPQTPSR	T5	0.984	0.887
Srrm2	Serine/arginine repetitive matrix protein 2	Q8BTI8	T1448	ALPQTPR	T5	0.839	0.552
Srrm2	Serine/arginine repetitive matrix protein 2	Q8BTI8	T2362	SRTPPSAPSQSR	T3	1.028	0.557
Srrm2	Serine/arginine repetitive matrix protein 2	Q8BTI8	T2553	EGRPQEPTPAKR	T8	1.178	0.198
Srrm2	Serine/arginine repetitive matrix protein 2	Q8BTI8	T823	SGTPPRPGSVTNMQADECTATPQR	T3	0.977	0.736
Srrm2	Serine/arginine repetitive matrix protein 2	Q8BTI8	T973	SMLQTPPDQNLSGSK	T5	0.968	0.334
Srrm2	Serine/arginine repetitive matrix protein 2	Q8BTI8	S1058,S1064	SQTSPKGSLSR	S4,S10	0.911	0.486
Srrm2	Serine/arginine repetitive matrix protein 2	Q8BTI8	S1068,S/T	GSLSRSSSPVTELTAR	S8,S/T	0.981	0.651
Srrm2	Serine/arginine repetitive matrix protein 2	Q8BTI8	S1077,S/T	SSSPVTELTARSPVK	S12,S/T	1.095	0.159
Srrm2	Serine/arginine repetitive matrix protein 2	Q8BTI8	S1335,S1338	SSRRSSSELSPEVVEK	S2,S5	0.933	0.235
Srrm2	Serine/arginine repetitive matrix protein 2	Q8BTI8	S1338,S1339	RSSSELSPEVVEK	S2,S3	1.039	0.267
Srrm2	Serine/arginine repetitive matrix protein 2	Q8BTI8	S1338,S1343	SSSELSPEVVEK	S1,S6	1.023	0.509
Srrm2	Serine/arginine repetitive matrix protein 2	Q8BTI8	S1360,T1370	VSSPVLETVQQRTPSRER	S3,T13	1.041	0.778
Srrm2	Serine/arginine repetitive matrix protein 2	Q8BTI8	S1360,T1365	VSSPVLETVQQRTPSR	S3,T8	1.009	0.815
Srrm2	Serine/arginine repetitive matrix protein 2	Q8BTI8	S1378,S1380	SSSASPELK	S3,S5	1.012	0.677
Srrm2	Serine/arginine repetitive matrix protein 2	Q8BTI8	S1378,S1380	SSSASPELKDGLPR	S3,S5	0.958	0.548
Srrm2	Serine/arginine repetitive matrix protein 2	Q8BTI8	S1380,S	ERSSSASPELK	S7,S	0.995	0.956
Srrm2	Serine/arginine repetitive matrix protein 2	Q8BTI8	S1438,S1439	SECDSSPEPK	S5,S6	0.963	0.629
Srrm2	Serine/arginine repetitive matrix protein 2	Q8BTI8	S1438,S1439	GRSECDSSPEPK	S7,S8	0.903	0.359
Srrm2	Serine/arginine repetitive matrix protein 2	Q8BTI8	S1497,S	SRSGSSQELDGKPSASPQER	S5,S	1.129	0.225
Srrm2	Serine/arginine repetitive matrix protein 2	Q8BTI8	S1508,S	SGSSQELDGKPSASPQER	S14,S	1.111	0.205
Srrm2	Serine/arginine repetitive matrix protein 2	Q8BTI8	S1537,S1538	SHSGSSPEVDSK	S5,S6	0.938	0.489
Srrm2	Serine/arginine repetitive matrix protein 2	Q8BTI8	S1556,S1557	SGSSPEMKDKPR	S3,S4	0.985	0.868
Srrm2	Serine/arginine repetitive matrix protein 2	Q8BTI8	S1576,S1577	AQSGTDSSPEHKIPAPR	S7,S8	0.968	0.665
Srrm2	Serine/arginine repetitive matrix protein 2	Q8BTI8	S1628,S1630	RGSRSSIEPK	S3,S5	0.974	0.467
Srrm2	Serine/arginine repetitive matrix protein 2	Q8BTI8	S1647,S/T	SSRSSPELTR	S2,S/T	1.033	0.668
Srrm2	Serine/arginine repetitive matrix protein 2	Q8BTI8	S1718,S1720	GRSPSPKPR	S3,S5	1.006	0.855
Srrm2	Serine/arginine repetitive matrix protein 2	Q8BTI8	S1878,T1880,T3779,T5690	SRTPPVTR	S1,T3	0.988	0.923
Srrm2	Serine/arginine repetitive matrix protein 2	Q8BTI8	S1972,T1974	SRTPPAIR	S1,T3	0.962	0.771
Srrm2	Serine/arginine repetitive matrix protein 2	Q8BTI8	S231,T233	HRSPTPK	S3,T5	0.989	0.879
Srrm2	Serine/arginine repetitive matrix protein 2	Q8BTI8	S2646,S2648	SLSYSPVER	S3,S5	0.995	0.829
Srrm2	Serine/arginine repetitive matrix protein 2	Q8BTI8	S2656,S2660	QPSPQPSPR	S3,S7	1.105	0.121
Srrm2	Serine/arginine repetitive matrix protein 2	Q8BTI8	S2656,S2660	RQPSPQPSPR	S4,S8	1.004	0.942
Srrm2	Serine/arginine repetitive matrix protein 2	Q8BTI8	S2679,S2681	GQRGDSHSPGHK	S6,S8	1.098	0.346
Srrm2	Serine/arginine repetitive matrix protein 2	Q8BTI8	S349,S351	SAVRPSPSPER	S6,S8	0.993	0.851
Srrm2	Serine/arginine repetitive matrix protein 2	Q8BTI8	S433,S434	HASSSPESLKPTPAPGSR	S3,S4	0.978	0.648
Srrm2	Serine/arginine repetitive matrix protein 2	Q8BTI8	S452,S454	REISSSPTSK	S4,S6	0.976	0.436
Srrm2	Serine/arginine repetitive matrix protein 2	Q8BTI8	S531,S533	GRSRSPQRPGWSR	S3,S5	1.001	0.966
Srrm2	Serine/arginine repetitive matrix protein 2	Q8BTI8	S773,S775	SLSGSSPCPK	S1,S3	1.035	0.133
Srrm2	Serine/arginine repetitive matrix protein 2	Q8BTI8	S773,S775	RSLSGSSPCPK	S2,S4	1.033	0.404
Srrm2	Serine/arginine repetitive matrix protein 2	Q8BTI8	S850,S851	QSHSESSPDGEVK	S6,S7	0.991	0.912
Srrm2	Serine/arginine repetitive matrix protein 2	Q8BTI8	S926,S928	SISPCPK	S1,S3	1.052	0.135

Srrm2	Serine/arginine repetitive matrix protein 2	Q8BTI8	S926,S928	SRSISPCPK	S3,S5	1.007	0.815
Srrm2	Serine/arginine repetitive matrix protein 2	Q8BTI8	S962,T963	HSGSTSPYLK	S4,T5	0.989	0.700
Srrm2	Serine/arginine repetitive matrix protein 2	Q8BTI8	T2021,S2023	SLTRSPPAIR	T3,S5	1.070	0.358
Srrm2	Serine/arginine repetitive matrix protein 2	Q8BTI8	T2056,S	NHSGSRTPPVALSSSR	T7,S	0.997	0.960
Srrm2	Serine/arginine repetitive matrix protein 2	Q8BTI8	T823,S829	SGTPPRPGSVTNMQADECTATPQR	T3,S9	0.977	0.727
Srrm2	Serine/arginine repetitive matrix protein 2	Q8BTI8	T823,T841	SGTPPRPGSVTNMQADECTATPQR	T3,T21	1.102	0.175
Srrm2	Serine/arginine repetitive matrix protein 2	Q8BTI8	T973,S984	SMLQTPPDQNLSGSKSPCPQK	T5,S16	1.011	0.836
Srrm2	Serine/arginine repetitive matrix protein 2	Q8BTI8	S1055,T1057,S1058	SQTSPKGSLSRSSSPVTELTAR	S1,T3,S4	0.988	0.921
Srrm2	Serine/arginine repetitive matrix protein 2	Q8BTI8	S1343,S	SSRRSSSELSPEVVEK	S10,S	1.068	0.384
Srrm2	Serine/arginine repetitive matrix protein 2	Q8BTI8	S2019,T2021,S2023	SLTRSPPAIR	S1,T3,S5	0.970	0.374
Srrm2	Serine/arginine repetitive matrix protein 2	Q8BTI8	T1370,S1372,S/T	VSSPVLETVQQRTPSRER	T13,S15,S/T	0.983	0.880
Srrm2	Serine/arginine repetitive matrix protein 2	Q8BTI8	T823,T831,T841	SGTPPRPGSVTNMQADECTATPQR	T3,T11,T21	0.906	0.580
Srrm2	Serine/arginine repetitive matrix protein 2	Q8BTI8	S884,S886,S887,S888	SSTPPRQSPSRSSSPQPK	S10,S12,S13,S14	1.037	0.689
Srrm2	Serine/arginine repetitive matrix protein 2	Q8BTI8	Ambiguous	TPAGLAPTNLSSSR	S/T	0.872	0.029
Srrm2	Serine/arginine repetitive matrix protein 2	Q8BTI8	Ambiguous	NHSGSRTPPVALSSSR	S/T	0.979	0.830
Srrm2	Serine/arginine repetitive matrix protein 2	Q8BTI8	Ambiguous	AQSGTDSSPEHKIPAPR	S/T	0.990	0.842
Srrm2	Serine/arginine repetitive matrix protein 2	Q8BTI8	Ambiguous	QSSSPYEDK	S/Y	1.101	0.255
Srrm2	Serine/arginine repetitive matrix protein 2	Q8BTI8	Ambiguous	RSSSELSPEVVEK	S	1.048	0.053
Srrm2	Serine/arginine repetitive matrix protein 2	Q8BTI8	Ambiguous	SECDSSPEPK	S	0.968	0.542
Srrm2	Serine/arginine repetitive matrix protein 2	Q8BTI8	Ambiguous	SHSGSSPEVDSK	S	0.860	0.037
Srrm2	Serine/arginine repetitive matrix protein 2	Q8BTI8	Ambiguous	EMPGSNIESSPEVEERPAVLSALDQSQSQPSK	S	0.955	0.549
Srrm2	Serine/arginine repetitive matrix protein 2	Q8BTI8	Ambiguous	HSLSGSSPGMK	S	0.964	0.841
Srrm2	Serine/arginine repetitive matrix protein 2	Q8BTI8	Ambiguous	HSGSTSPYLK	T/S/Y	0.966	0.222
Srrm2	Serine/arginine repetitive matrix protein 2	Q8BTI8	Ambiguous	SGTPPRPGSVTNMQADECTATPQR	T/S	0.921	0.396
Srrm2	Serine/arginine repetitive matrix protein 2	Q8BTI8	Ambiguous	SSTPPRQSPSR	S/T	0.872	0.101
Srrm2	Serine/arginine repetitive matrix protein 2	Q8BTI8	Ambiguous	EISSSPTSK	S/T	1.015	0.731
Srrm2	Serine/arginine repetitive matrix protein 2	Q8BTI8	Ambiguous	SRTSPVTR	S1,S4	0.917	0.599
Srrm2	Serine/arginine repetitive matrix protein 2	Q8BTI8	Ambiguous	GHTQTWPDTSSPEVMQTQVESPLLQSK	T/S	0.983	0.818
Srrt	Serrate RNA effector molecule homolog	Q99MR6	S492	LRECELSPGVNR	S7	0.956	0.718
Srrt	Serrate RNA effector molecule homolog	Q99MR6	S74	HELSPPQKR	S4	0.947	0.743
Srrt	Serrate RNA effector molecule homolog	Q99MR6	S67,S74	ERFSPPRHELSPPQK	S4,S11	0.997	0.940
Srsf1	Serine/arginine-rich-splicing factor 1	H7BX95	S199	VDGPRSPSYGR	S6	1.021	0.832
Srsf1	Serine/arginine-rich-splicing factor 1	H7BX95	S199	VKVDGPRSPSYGR	S8	0.992	0.763
Srsf1	Serine/arginine-rich-splicing factor 1	H7BX95	S199,S201	VDGPRSPSYGR	S6,S8	1.016	0.810
Srsf1	Serine/arginine-rich-splicing factor 1	H7BX95	S199,S201	VKVDGPRSPSYGR	S8,S10	1.052	0.324
Srsf1	Serine/arginine-rich-splicing factor 1	H7BX95	S234,S238	GSPRYSPR	S2,S6	1.032	0.835
Srsf10	Serine/arginine-rich-splicing factor 10	Q3TFP0	S133	SFDYNYR	S1	1.090	0.414
Srsf10	Serine/arginine-rich-splicing factor 10	Q3TFP0	S133	RSRSFDYNYR	S3	0.983	0.771
Srsf10	Serine/arginine-rich-splicing factor 10	Q3TFP0	S23	NVADDTRSEDLR	S8	1.210	0.089
Srsf10	Serine/arginine-rich-splicing factor 10	Q3TFP0	Ambiguous	SRSHSDNDRPNCSWNTQYSSAYYTSR	S/T/Y	1.037	0.763
Srsf10	Serine/arginine-rich-splicing factor 10	Q3TFP0	Ambiguous	NVYSSSRYDDYDR	S/Y	1.018	0.839
Srsf11	Serine/arginine-rich-splicing factor 11 (Fragment)	F6RDI8	S451	DYDEEEQGYDSEKEKK	S11	1.021	0.832
Srsf11	Serine/arginine-rich-splicing factor 11 (Fragment)	F6RDI8	S451	DYDEEEQGYDSEKEK	S11	1.024	0.823
Srsf11	Serine/arginine-rich-splicing factor 11 (Fragment)	F6RDI8	S466	RPTEAVSPK	S7	0.953	0.530
Srsf11	Serine/arginine-rich-splicing factor 11 (Fragment)	F6RDI8	S499	VNGDDHHEEDMDMSD	S14	0.981	0.726
Srsf11	Serine/arginine-rich-splicing factor 11 (Fragment)	F6RDI8	S499	VNGDDHHEEDMDMSD	S14	1.074	0.507
Srsf11	Serine/arginine-rich-splicing factor 11 (Fragment)	F6RDI8	S499	ESKVNGDDHHEEDMDMSD	S17	0.961	0.615
Srsf11	Serine/arginine-rich-splicing factor 11 (Fragment)	F6RDI8	S499	ESKVNGDDHHEEDMDMSD	S17	0.914	0.425
Srsf11	Serine/arginine-rich-splicing factor 11 (Fragment)	F6RDI8	Ambiguous	SASSLHVCDSR	S	1.119	0.019
Srsf2	Serine/arginine-rich splicing factor 2	Q62093	S191	SRSPPPVSK	S3	1.078	0.331
Srsf2	Serine/arginine-rich splicing factor 2	Q62093	S208	SKSPPKSPEEEGAVSS	S3	0.970	0.749
Srsf2	Serine/arginine-rich splicing factor 2	Q62093	S26	TSPDTLR	S2	0.999	0.988
Srsf2	Serine/arginine-rich splicing factor 2	Q62093	T25	VDNLTYRTSPDTLR	T8	1.099	0.681
Srsf2	Serine/arginine-rich splicing factor 2	Q62093	S189,S191	SRSPPPVSK	S1,S3	1.048	0.488
Srsf2	Serine/arginine-rich splicing factor 2	Q62093	S208,S212	SKSPPKSPEEEGAVSS	S3,S7	1.201	0.322
Srsf2	Serine/arginine-rich splicing factor 2	Q62093	S187,S189,S191	SRSRSPPPVSK	S1,S3,S5	1.236	0.268
Srsf2	Serine/arginine-rich splicing factor 2	Q62093	Ambiguous	TSPDTLRR	S/T	1.002	0.965
Srsf2	Serine/arginine-rich splicing factor 2	Q62093	Ambiguous	VDNLTYRTSPDTLRR	T/S/Y	0.968	0.578
Srsf5	MCG7614, isoform CRA_c	Q9D8S5	S231	SGSRSPVPEK	S5	0.984	0.890
Srsf6	Serine/arginine-rich splicing factor 6	Q3TWW8; Q9D8S5	S119;S117	LIVENLSSR	S8	1.024	0.772
Srsf6	Serine/arginine-rich splicing factor 6	Q3TWW8	S303	SHSPLPAPPSK	S3	1.003	0.927
Srsf6	Serine/arginine-rich splicing factor 6	Q3TWW8	S299,S303	SQSRSHSPLPAPPSK	S3,S7	0.968	0.522
Srsf6	Serine/arginine-rich splicing factor 6	Q3TWW8	S314,S316	SMSPPPK	S1,S3	1.028	0.472
Srsf6	Serine/arginine-rich splicing factor 6	Q3TWW8	S314,S316	SMSPPPK	S1,S3	1.022	0.480
Srsf6	Serine/arginine-rich splicing factor 6	Q3TWW8	S314,S316	ARSMSPPPK	S3,S5	1.016	0.751
Srsf6	Serine/arginine-rich splicing factor 6	Q3TWW8	S314,S316	ARSMSPPPK	S3,S5	0.987	0.804
Srsf6	Serine/arginine-rich splicing factor 6	Q3TWW8	S299,S301,S303	SRSQSRSHSPLPAPPSK	S5,S7,S9	0.945	0.171
Srsf7	Serine/arginine-rich splicing factor 7	Q8BL97	S210	SGSIIGSR	S1	1.058	0.058
Srsf7	Serine/arginine-rich splicing factor 7	Q8BL97	S210,S212	SGSIIGSR	S1,S3	0.973	0.578
Srsf7	Serine/arginine-rich splicing factor 7	Q8BL97	S210,S212	SRSGSIIGSR	S3,S5	0.985	0.886
Srsf9	Serine/arginine-rich splicing factor 9	Q9D0B0	S212	GSPHYFSPFRPY	S2	0.976	0.872
Srsf9	Serine/arginine-rich splicing factor 9	Q9D0B0	S212,S217	GSPHYFSPFRPY	S2,S7	0.988	0.923
Ssbp3	Single-stranded DNA-binding protein 3	Q9D032	T360	NSPNNISGISNPPGTPR	T15	1.093	0.064
Ssbp3	Single-stranded DNA-binding protein 3	Q9D032	S347,T360	NSPNNISGISNPPGTPR	S2,T15	0.965	0.749
Ssbp4	Single stranded DNA binding protein 4, isoform CRA_b	Q3U4B1	T333	NSPGAVGGLSNAPGTPR	T15	1.009	0.928
Ssbp4	Single stranded DNA binding protein 4, isoform CRA_b	Q3U4B1	S320,T333	NSPGAVGGLSNAPGTPR	S2,T15	1.002	0.960
Ssh1	Protein phosphatase Slingshot homolog 1	Q76I79	S28	SPTPSAASSSASNSELEAGSDEER	S20	0.931	0.305
Ssh1	Protein phosphatase Slingshot homolog 1	Q76I79	S597	SDSLPQVEELEKDGSPR	S15	0.992	0.832
Ssh1	Protein phosphatase Slingshot homolog 1	Q76I79	S585,S597	SDSLPQVEELEKDGSPR	S3,S15	1.051	0.458
Ssh2	Protein phosphatase Slingshot homolog 2	A0A0R4J2A0	S493	EMTTSADQIAEVK	S5	1.015	0.779
Ssh2	Protein phosphatase Slingshot homolog 2	A0A0R4J2A0	S493	EMTTSADQIAEVK	S5	0.945	0.151
Ssh2	Protein phosphatase Slingshot homolog 2	A0A0R4J2A0	T1428	TTNPFYNTM	T8	0.986	0.749
Ssh2	Protein phosphatase Slingshot homolog 2	A0A0R4J2A0	T1428	TTNPFYNTM	T8	0.974	0.762
Ssh3	Protein phosphatase Slingshot homolog 3	Q8K330	S38	RQSFAVLR	S3	1.083	0.070
Ssh3	Protein phosphatase Slingshot homolog 3	Q8K330	S639	QASVDDSREEDKA	S3	1.009	0.697
Ssh3	Protein phosphatase Slingshot homolog 3	Q8K330	Ambiguous	AFQEQGQGQEQSEPGMSSTPR	T/S	0.970	0.725
Ssr3	Translocon-associated protein subunit gamma	Q9DCF9	S11	QQSEEDLLLQDFSR	S3	0.899	0.223
Ssrp1	FACT complex subunit SSRP1	Q08943	S444	EGINPGYDDYADSDEDQHDAYLER	S13	1.007	0.853
Ssrp1	FACT complex subunit SSRP1	Q08943	S667	EFVSSDESSSGENK	S4	1.160	0.174
Ssrp1	FACT complex subunit SSRP1	Q08943	Ambiguous	SKEFVSSDESSSGENK	S	0.924	0.271
Ssx2ip	Afadin- and alpha-actinin-binding protein	Q8VC66	S313	ERAEDGTGTVAISDIEDDSGELSR	S13	0.944	0.748
Ssx2ip	Afadin- and alpha-actinin-binding protein	Q8VC66	Ambiguous	SLPASPSTSDFR	S/T	1.017	0.913
St5	Suppression of tumorigenicity 5 protein	Q924W7	S48	SPIYPLSDSETSACR	S7	1.067	0.766
Stac2	SH3 and cysteine-rich domain-containing protein 2	Q8R1B0	S221	SSFSSTSESPTR	S2	1.062	0.499
Stac2	SH3 and cysteine-rich domain-containing protein 2	Q8R1B0	S268	SSEEGPGDSVFTAPAESEGSGPEEK	S20	1.719	0.007
Stac2	SH3 and cysteine-rich domain-containing protein 2	Q8R1B0	S78	CPTEVLLTPPTPLPPPSPPPASTDR	S17	0.985	0.938
Stam	Signal transducing adapter molecule 1	P70297	S156	ASPALVAK	S2	1.070	0.393
Stambpl1	AMSH-like protease	Q76N33	S242	SDGSNFANYSPPVNR	S10	0.976	0.380
Stard10	START domain-containing protein 10	E9PVP0	S357	AGGAGGEGSDDDTSLT	S9	0.960	0.521
Stard3nl	STARD3 N-terminal-like protein	A0A1Y7VJA7	S193	LLLVQDASER	S8	1.048	0.247
Stard3nl	STARD3 N-terminal-like protein	A0A1Y7VJA7	S27	DIHSINPAQLMAR	S4	0.864	0.280
Stard8	StAR-related lipid transfer protein 8	Q8K031	S234	SLSIESLCPDEGR	S3	0.979	0.670
Stard8	StAR-related lipid transfer protein 8	Q8K031	S742	SLVGRPGPR	S1	1.134	0.125
Stard9	StAR-related lipid transfer protein 9	Q80TF6	S1696	EAWPSPPPR	S6	1.143	0.471
Stard9	StAR-related lipid transfer protein 9	Q80TF6	S3664	LQSPPLPSPHLR	S3	1.008	0.941
Stard9	StAR-related lipid transfer protein 9	Q80TF6	S3876	TTTGDQSKLPSPPPR	S11	1.031	0.449
Stard9	StAR-related lipid transfer protein 9	Q80TF6	S3664,S3669	LQSPPLPSPHLR	S3,S8	1.232	0.186
Stat1	Signal transducer and activator of transcription	A0A087WSP5	S733	LQTTDNLLPMSPEEFDEMSR	S11	1.185	0.164
Stat5b	Signal transducer and activator of transcription 5B	P42232	S128	EANNGSSPAGSLADAMSQK	S7	1.013	0.836
Stau2	Double-stranded RNA-binding protein Staufen homolog 2	Q8CJ67	Ambiguous	GSSPTSPCSSVQPSK	S/T	1.195	0.028
Steap3	Metalloreductase STEAP3	E9QN92	S55	RLVDSDGSLAEVPK	S5	0.944	0.329
Steap3	Metalloreductase STEAP3	E9QN92	S58	LVDSDGSLAEVPK	S7	0.998	0.988
Steap3	Metalloreductase STEAP3	E9QN92	S58	LVDSDGSLAEVPKEAPK	S7	0.994	0.896
Steap3	Metalloreductase STEAP3	E9QN92	S55,S58	RLVDSDGSLAEVPK	S5,S8	1.057	0.639
Stim1	Stromal interaction molecule 1	P70302	S257	AEQSLHDLQER	S4	0.988	0.587
Stim1	Stromal interaction molecule 1	P70302	S40	NTGASSGATSEESTEAEFCR	S10	1.084	0.345
Stim1	Stromal interaction molecule 1	P70302	S512	LTEPQLGLGSQR	S10	0.948	0.521
Stim1	Stromal interaction molecule 1	P70302	S519	DLTHSDSESSLHMSDR	S5	0.905	0.108
Stim1	Stromal interaction molecule 1	P70302	S519	DLTHSDSESSLHMSDR	S5	1.031	0.511
Stim1	Stromal interaction molecule 1	P70302	S575	LPDSPALAK	S4	0.920	0.291
Stim1	Stromal interaction molecule 1	P70302	S660	AMAEEDNGSIGEETDSSPGR	S9	1.017	0.722
Stim1	Stromal interaction molecule 1	P70302	Ambiguous	AMAEEDNGSIGEETDSSPGRK	S/T	1.036	0.522
Stim1	Stromal interaction molecule 1	P70302	Ambiguous	AMAEEDNGSIGEETDSSPGRK	S/T	1.024	0.603
Stim2	Stromal interaction molecule 2	I1E4X8	S531	SIVPSSPQSQR	S6	1.019	0.671
Stim2	Stromal interaction molecule 2	I1E4X8	S531	RSIVPSSPQSQR	S7	1.065	0.353
Stim2	Stromal interaction molecule 2	I1E4X8	S607	QWEVPDTASECDSLNSSSGR	S13	1.142	0.402
Stim2	Stromal interaction molecule 2	I1E4X8	S648	DELSLEDSSRGESPVTADVSR	S13	1.128	0.423
Stim2	Stromal interaction molecule 2	I1E4X8	S658	GESPVTADVSRGSPECVGLTETK	S13	1.090	0.054
Stim2	Stromal interaction molecule 2	I1E4X8	S658	GSPECVGLTETK	S2	1.012	0.928
Stim2	Stromal interaction molecule 2	I1E4X8	S669	SMIFSPASR	S1	0.981	0.874
Stim2	Stromal interaction molecule 2	I1E4X8	S526,S	SIVPSSPQSQR	S1,S	1.096	0.378
Stim2	Stromal interaction molecule 2	I1E4X8	Ambiguous	SCSMHQLSSGIPVPHPR	S	0.905	0.357
Stip1	Stress-induced-phosphoprotein 1	Q60864	S481	HDSPEDVK	S3	0.978	0.811
Stk10	Serine/threonine-protein kinase 10	O55098	S437	QIPDQDENPSPAASK	S10	0.986	0.779
Stk10	Serine/threonine-protein kinase 10	O55098	S515	ETGSLSLK	S6	1.001	0.981
Stk10	Serine/threonine-protein kinase 10	O55098	S908	ALDESHNQSLK	S9	0.948	0.463
Stk10	Serine/threonine-protein kinase 10	O55098	S966	ASNFFPYSSGDAS	S13	1.058	0.747
Stk10	Serine/threonine-protein kinase 10	O55098	T950	LSEEAEPRPTTPSK	T11	1.102	0.034

Stk11	Serine/threonine-protein kinase STK11	Q9WTK7	S31	IDSTEVIYQPR	S3	1.040	0.673
Stk11ip	Serine/threonine-protein kinase 11-interacting protein	Q3TAA7	S388	RASISEPSDTDPELR	S3	0.964	0.434
Stk11ip	Serine/threonine-protein kinase 11-interacting protein	Q3TAA7	S388,S390	RASISEPSDTDPELR	S3,S5	0.946	0.370
Stk11ip	Serine/threonine-protein kinase 11-interacting protein	Q3TAA7	Ambiguous	SHLETMGSSPLSTTK	S/T	0.938	0.426
Stk24	Serine/threonine-protein kinase 24	Q99KH8	T341	NLENGTLQLSDLER	T6	1.162	0.332
Stk3	Serine/threonine-protein kinase 3	Q9JI10	S316	HEEQQRELEEEEENSDEDELDSHTMVK	S15	0.988	0.884
Stk3	Serine/threonine-protein kinase 3	Q9JI10	S316	ELEEEEENSDEDELDSHTMVK	S9	1.068	0.194
Stk3	Serine/threonine-protein kinase 3	Q9JI10	S316	ELEEEEENSDEDELDSHTMVK	S9	0.985	0.549
Stk3	Serine/threonine-protein kinase 3	Q9JI10	S316	RHEEQQRELEEEEENSDEDELDSHTMVK	S16	0.949	0.379
Stk39	STE20/SPS1-related proline-alanine-rich protein kinase	Q9Z1W9	S397	TEDGDWEWSDDEMDEKSEEGK	S9	0.960	0.781
Stk4	Serine/threonine-protein kinase 4	Q9JI11	S320	EVDQDDEENSEEDEMDSGTMVR	S10	1.137	0.239
Stk4	Serine/threonine-protein kinase 4	Q9JI11	S320	EVDQDDEENSEEDEMDSGTMVR	S10	0.947	0.264
Stk4	Serine/threonine-protein kinase 4	Q9JI11	S320	EVDQDDEENSEEDEMDSGTMVR	S10	1.021	0.453
Stk4	Serine/threonine-protein kinase 4	Q9JI11	S410	ENQINSFGK	S6	0.994	0.870
Stmn1	Stathmin	P54227	S16	RASGQAFELILSPR	S3	1.032	0.761
Stmn1	Stathmin	P54227	S25	ASGQAFELILSPR	S11	1.575	0.000
Stmn1	Stathmin	P54227	S38	ESVPDFPLSPPK	S9	1.056	0.695
Stmn1	Stathmin	P54227	S46	DLSLEEIQK	S3	1.014	0.738
Stmn1	Stathmin	P54227	S16,S25	RASGQAFELILSPR	S3,S12	1.099	0.270
Stom	Erythrocyte band 7 integral membrane protein	P54116	S244	EASMVITESPAALQLR	S9	1.156	0.234
Stoml2	Stomatin-like protein 2, mitochondrial	Q99JB2	S343	DVQATDTSIEELGR	S8	0.982	0.792
Strip1	Striatin-interacting protein 1	Q8C079	S335	AASPPASASDLIEQQQK	S3	1.030	0.225
Strip1	Striatin-interacting protein 1	Q8C079	S335	AASPPASASDLIEQQQKR	S3	0.916	0.591
Strip1	Striatin-interacting protein 1	Q8C079	S335,S339	AASPPASASDLIEQQQK	S3,S7	0.979	0.812
Strn	Striatin	O55106	S245	FLESAAADVSDEDEDEDTDGRAK	S10	0.887	0.470
Strn	Striatin	O55106	S245	FLESAAADVSDEDEDEDTDGR	S10	0.980	0.643
Strn	Striatin	O55106	S259	SVIDTSTIVR	S1	1.132	0.013
Strn4	Striatin-4	P58404	S206	SLELNGAGEPVEGAPR	S1	0.836	0.251
Strn4	Striatin-4	P58404	S223	ASPGPGGLSGGESLLVK	S2	1.136	0.416
Stt3b	Dolichyl-diphosphooligosaccharide--protein glycosyltransferase subunit STT3B	A0A0R4J0D3	S496	ENPPVEDSSDEDDKR	S9	1.055	0.545
Stt3b	Dolichyl-diphosphooligosaccharide--protein glycosyltransferase subunit STT3B	A0A0R4J0D3	S495,S496	ENPPVEDSSDEDDKRNPGNLYDK	S8,S9	1.009	0.870
Stt3b	Dolichyl-diphosphooligosaccharide--protein glycosyltransferase subunit STT3B	A0A0R4J0D3	Ambiguous	ENPPVEDSSDEDDKRNPGNLYDK	S/Y	1.039	0.335
Stt3b	Dolichyl-diphosphooligosaccharide--protein glycosyltransferase subunit STT3B	A0A0R4J0D3	Ambiguous	ENPPVEDSSDEDDK	S	1.089	0.818
Stub1	STIP1 homology and U box-containing protein 1	Q9WUD1	S20	LGTGGGSPDKSPSAQELK	S8	1.010	0.845
Stub1	STIP1 homology and U box-containing protein 1	Q9WUD1	S20,S24	LGTGGGSPDKSPSAQELK	S8,S12	0.988	0.921
Stx12	Syntaxin-12	Q9ER00	S142	AGSRLSAEDR	S6	1.068	0.154
Stx12	Syntaxin-12	Q9ER00	S139,S142	AGSRLSAEDR	S3,S6	1.084	0.315
Stx12	Syntaxin-12	Q9ER00	Ambiguous	ELGSLPLPLSASEQR	S	0.909	0.728
Stx17	Syntaxin-17	Q9D0I4	S288	LTSSCPDLPSQSDK	S4	0.968	0.626
Stx17	Syntaxin-17	Q9D0I4	Ambiguous	QVNDEELLQPSLTR	S/T	1.318	0.124
Stx4	Syntaxin-4	P70452	S117	AIEPQKEEADENYNSVNTR	S15	0.974	0.853
Stx4	Syntaxin-4	P70452	S15	QGDNISDDEDEVR	S6	1.033	0.343
Stx4	Syntaxin-4	P70452	S208	QALNEISARHSEIQQLER	S11	1.022	0.821
Stx4	Syntaxin-4	P70452	S208	HSEIQQLER	S2	1.034	0.594
Stx4	Syntaxin-4	P70452	S36	LGSPDDEFFQK	S3	0.698	0.177
Stx5a	Syntaxin 5A	I1E4X1	Ambiguous	TSQQLQLIDEQDSYIQSR	S/T/Y	1.105	0.694
Stx6	Syntaxin-6	Q9JKK1	Ambiguous	QALLGDSSSQSWNAGVADR	S	1.146	0.338
Stx7	Syntaxin-7	O70439	S129	VSGGFPEDSSKEK	S2	1.147	0.088
Stx7	Syntaxin-7	O70439	S129	VSGGFPEDSSK	S2	1.136	0.340
Stx7	Syntaxin-7	O70439	S129	ASSRVSGGFPEDSSK	S6	1.130	0.086
Stx7	Syntaxin-7	O70439	S129	ASSRVSGGFPEDSSKEK	S6	0.966	0.692
Stx7	Syntaxin-7	O70439	S45	TLNQLGTPQDSPELR	S11	1.084	0.060
Stx7	Syntaxin-7	O70439	S81	EFGSLPTTPSEQR	S10	1.088	0.390
Stx7	Syntaxin-7	O70439	S129,S	ASSRVSGGFPEDSSKEK	S6,S	1.034	0.570
Stx7	Syntaxin-7	O70439	S129,S	ASSRVSGGFPEDSSK	S6,S	1.087	0.073
Stx7	Syntaxin-7	O70439	S75,T79	EFGSLPTTPSEQR	S4,T8	0.983	0.857
Stxbp1	Syntaxin-binding protein 1	O08599	Ambiguous	LNKTDEEISS	S/T	1.104	0.214
Stxbp2	Syntaxin-binding protein 2 (Fragment)	F8WGM5	T216	ADTPSLGEGPEK	T3	0.988	0.866
Stxbp3	Syntaxin-binding protein 3	Q60770	S512	CPAVWNGSGAVSAR	S12	0.906	0.508
Stxbp4	Syntaxin-binding protein 4	Q9WV89	Ambiguous	SSSPLDRDPAFR	S	0.953	0.173
Stxbp5	Syntaxin-binding protein 5	Q8K400	S693	QPSGAGLCDITEGTVVPEDR	S3	1.050	0.670
Stxbp5	Syntaxin-binding protein 5	Q8K400	S693	SRQPSGAGLCDITEGTVVPEDR	S5	1.025	0.646
Stxbp5	Syntaxin-binding protein 5	Q8K400	S783	SSSVTSIDK	S3	0.985	0.807
Stxbp5	Syntaxin-binding protein 5	Q8K400	S783,S786	SSSVTSIDKESR	S3,S6	1.023	0.603
Stxbp5	Syntaxin-binding protein 5	Q8K400	Ambiguous	SSSVTSIDKESR	S/T	0.977	0.362
Sub1	Activated RNA polymerase II transcriptional coactivator p15	P11031	S118	EQISDIDDAVR	S4	1.065	0.275
Sub1	Activated RNA polymerase II transcriptional coactivator p15	P11031	Ambiguous	QSSSSRDDNMFQIGK	S	1.210	0.077
Suds3	Sin3 histone deacetylase corepressor complex component SDS3	A0A0R4J243	S234	RPASPSSPEHLPATPAESPAQR	S4	0.912	0.154
Suds3	Sin3 histone deacetylase corepressor complex component SDS3	A0A0R4J243	S234,S/T	RPASPSSPEHLPATPAESPAQR	S4,S/T	1.079	0.253
Sugp1	SURP and G-patch domain-containing protein 1	Q8CH02	S409	DIDASPSPLSVQDLK	S7	0.828	0.379
Sugp1	SURP and G-patch domain-containing protein 1	Q8CH02	S483	ALQQHQHGYDSDEEVDSELGTWEHQLR	S11	0.992	0.957
Sulf2	Extracellular sulfatase Sulf-2	Q8CFG0	S503	YDGQSSEACSCDSGGGDYK	S5	1.327	0.109
Sumo2	Small ubiquitin-related modifier 2	P61957	S28	VAGQDGSVVQFK	S7	1.058	0.597
Sun1	SUN domain-containing protein 1	Q9D666	S305	VDGESLCDDCK	S5	1.020	0.748
Sun2	SUN domain-containing protein 2	Q8BJS4	S120	GTGGSESSKANGLTAESK	S5	0.916	0.183
Sun2	SUN domain-containing protein 2	Q8BJS4	S39	YSQDDNDGGSSSSGASSVAGSQGTVFKDSPLR	S29	1.192	0.441
Sun2	SUN domain-containing protein 2	Q8BJS4	S83	ESYIGSPR	S6	1.176	0.015
Sun2	SUN domain-containing protein 2	Q8BJS4	S120,S/T	GTGGSESSKANGLTAESK	S5,S/T	1.027	0.872
Sun2	SUN domain-containing protein 2	Q8BJS4	Ambiguous	YSQDDNDGGSSSSGASSVAGSQGTVFK	S/T/Y	1.000	1.000
Sun2	SUN domain-containing protein 2	Q8BJS4	Ambiguous	ASEDFFGSSSGYSSEDDLAGYTDSDQHSSGSR	Y/S/T	1.021	0.823
Supt3	Suppressor of Ty 3 (Fragment)	B0QZW4	S271	SSPDSPENTPPPTPTAPSSGSQHSGR	S5	1.621	0.310
Supt3	Suppressor of Ty 3 (Fragment)	B0QZW4	S271,T275	SSPDSPENTPPPTPTAPSSGSQHSGR	S5,T9	1.061	0.775
Supt5h	Transcription elongation factor SPT5	O55201	S664	DVTNLTVGGFTPMSPR	S14	0.891	0.251
Supt5h	Transcription elongation factor SPT5	O55201	S664	DVTNLTVGGFTPMSPR	S14	0.984	0.976
Supt5h	Transcription elongation factor SPT5	O55201	T769	TPMYGSQTPMYGSGSR	T8	0.998	0.991
Supt5h	Transcription elongation factor SPT5	O55201	T785	TPMYGSQTPLQDGSR	T8	0.980	0.796
Supt5h	Transcription elongation factor SPT5	O55201	Ambiguous	TPHYGSQTPLHDGSR	S/T/Y	0.890	0.532
Supt6h	Transcription elongation factor SPT6	Q62383	S125	KMSDDDEDDEEEYGKEEHEK	S3	0.936	0.594
Supt6h	Transcription elongation factor SPT6	Q62383	T1523	DHYQDPVPGITPSSSNR	T11	0.965	0.742
Supt7l	STAGA complex 65 subunit gamma	Q9CZV5	S106	AEESEPLPSCPGSPPLPDDLQPLDCK	S13	0.943	0.466
Surf6	Surfeit locus protein 6	P70279	T223	GNLTPLTGR	T4	0.963	0.604
Suz12	Polycomb protein Suz12	Q80U70	S548	ASMSEFLESEDGEVEQQR	S9	1.078	0.675
Suz12	Polycomb protein Suz12	Q80U70	S548	ASMSEFLESEDGEVEQQR	S9	0.993	0.924
Svil	Supervillin	A0A1B0GS91	S137	ATDPASPHIGR	S6	0.996	0.866
Svil	Supervillin	A0A1B0GS91	S307	SISFPEVPR	S3	1.401	0.045
Svil	Supervillin	A0A1B0GS91	S321	QIPSSPLQQPASPNHPGDSPLPTEAR	S5	0.996	0.977
Svil	Supervillin	A0A1B0GS91	S719	YPSGSEIPVVEDEEKVDER	S3	1.105	0.056
Svil	Supervillin	A0A1B0GS91	S848	DQANEGRESAEPGEPDSSTLSLAEK	S9	1.067	0.425
Svil	Supervillin	A0A1B0GS91	S944	KLSVDNNTSATDYKSPPAENSDSPVR	S3	1.039	0.722
Svil	Supervillin	A0A1B0GS91	T898	YQTQPVTLGEVEQVQSGK	T3	1.020	0.871
Svil	Supervillin	A0A1B0GS91	S307,S314	SISFPEVPRSPK	S3,S10	1.423	0.009
Svil	Supervillin	A0A1B0GS91	S320,S321	QIPSSPLQQPASPNHPGDSPLPTEAR	S4,S5	0.962	0.342
Svil	Supervillin	A0A1B0GS91	S321,S328,S335	QIPSSPLQQPASPNHPGDSPLPTEAR	S5,S12,S19	0.865	0.290
Svil	Supervillin	A0A1B0GS91	Ambiguous	ESAEPGEPDSSTLSLAEK	S/T	0.789	0.048
Syk	Tyrosine-protein kinase SYK	P48025	Y346	EALPMDTEVYESPYADPEEIRPK	Y14	0.944	0.274
Syk	Tyrosine-protein kinase SYK	P48025	Y624	NYYYDVVN	Y3	0.921	0.538
Syk	Tyrosine-protein kinase SYK	P48025	Y346,T/Y/S	EALPMDTEVYESPYADPEEIRPK	Y14,T/Y/S	1.039	0.615
Syk	Tyrosine-protein kinase SYK	P48025	Ambiguous	EALPMDTEVYESPYADPEEIRPK	S/Y/T	0.928	0.014
Sympk	Symplekin	F8WJD4	S1260	DERSPQNLSHAVEEALK	S4	1.061	0.314
Sympk	Symplekin	F8WJD4	S1276	TSSPETREPESK	S3	1.020	0.867
Syn1	Synapsin-1	O88935	S427	DASPGRGSHSQSSSPGALTLGR	S3	1.015	0.697
Syn1	Synapsin-1	O88935	S438	GSHSQSSSPGALTLGR	S8	0.977	0.619
Syn1	Synapsin-1	O88935	S551	QSRPVAGGPGAPPAARPPASPSPQR	S20	0.930	0.606
Syn1	Synapsin-1	O88935	S568	QASISGPAPTK	S3	1.151	0.042
Syn1	Synapsin-1	O88935	S434,S/T	DASPGRGSHSQSSSPGALTLGR	S10,S/T	0.985	0.919
Syn1	Synapsin-1	O88935	S551,S553	QSRPVAGGPGAPPAARPPASPSPQR	S20,S22	1.007	0.769
Syn1	Synapsin-1	O88935	Ambiguous	QTSQQPAGPPAQQRPPPQGGPPQPGPGPQR	T/S	1.317	0.061
Syne1	Nesprin-1	A0A1L1STC6	S8285	TLPSEDEEGEEDKEFYLR	S4	1.018	0.855
Syne1	Nesprin-1	A0A1L1STC6	S8333	NTSGDPSSLESQMR	S8	0.961	0.811
Syne1	Nesprin-1	A0A1L1STC6	T8363	SKTPTGPDLDTSYK	T3	1.056	0.424
Syne2	Nesprin-2	Q6ZWQ0	S6348	LTSHTPGLDDEKEASENETDIEDPR	S15	0.964	0.667
Synj1	Synaptojanin-1	D3Z656	S1089	TSPCQSPTVPEYSAPSLPIRPSR	S2	1.006	0.975
Synj1	Synaptojanin-1	D3Z656	S1124	TPGPPSSQGSPVDTQPAAQK	S10	1.055	0.082
Synj1	Synaptojanin-1	D3Z656	S1200	EIEAPKSPGTAR	S7	1.017	0.663
Synj1	Synaptojanin-1	D3Z656	S1215	NQPSPQAGLAGPGPAGYGAARPTIPAR	S4	0.937	0.230
Synj1	Synaptojanin-1	D3Z656	S1339	SSQSLPSDSSPQLQVK	S10	1.000	0.995
Synj1	Synaptojanin-1	D3Z656	S1383	AQPSVQISPVLTPDPK	S8	0.941	0.694
Synj1	Synaptojanin-1	D3Z656	S1093,T/S/Y	TSPCQSPTVPEYSAPSLPIRPSR	S6,T/S/Y	0.991	0.900
Synj1	Synaptojanin-1	D3Z656	S1120,S1121	APSRTPGPPSSQGSPVDTQPAAQK	S10,S11	1.099	0.322
Synj1	Synaptojanin-1	D3Z656	S1120,S/T	TPGPPSSQGSPVDTQPAAQK	S6,S/T	0.987	0.624
Synj1	Synaptojanin-1	D3Z656	S1331,S1333	SSQSLPSDSSPQLQVK	S2,S4	0.991	0.890
Synj1	Synaptojanin-1	D3Z656	S1333,S	SRSSQSLPSDSSPQLQVK	S6,S	1.033	0.489
Synj1	Synaptojanin-1	D3Z656	Ambiguous	APSRTPGPPSSQGSPVDTQPAAQK	S/T	1.018	0.739
Synj1	Synaptojanin-1	D3Z656	Ambiguous	SRSSQSLPSDSSPQLQVK	S	1.013	0.862
Synpo	Synaptopodin	E9Q3E2	S134	STSFTENDLK	S3	0.996	0.968
Synrg	Synergin gamma	V9GX40	S1102	SSPSPALEQPFR	S4	0.941	0.144
Synrg	Synergin gamma	V9GX40	S1102	SSPSPALEQPFRDR	S4	0.940	0.185
Synrg	Synergin gamma	V9GX40	S676	TADSVSPLEPPTK	S6	0.967	0.687

Synrg	Synergin gamma	V9GX40	S997	DMMPQTTEQKEFESGDFQDFTR	S14	1.136	0.201
Synrg	Synergin gamma	V9GX40	S997	EFESGDFQDFTR	S4	1.106	0.320
Synrg	Synergin gamma	V9GX40	Ambiguous	SQETSCPSPASSVASHETPKEGADDFGEFQSEK	S/T	1.003	0.993
Synrg	Synergin gamma	V9GX40	Ambiguous	SGSIDDSFTDFQEMPASSK	S/T	0.957	0.723
Synrg	Synergin gamma	V9GX40	Ambiguous	SQETSCPSPASSVASHETPK	S/T	1.007	0.958
Syvn1	E3 ubiquitin-protein ligase synoviolin	A0A0R4J1R1	S608	LESPVAH	S3	0.972	0.578
Szrd1	SUZ domain-containing protein 1	Q6NXN1	S107	ILGSASPEEEQEKPILDRPTR	S6	1.122	0.250
Szt2	KICSTOR complex protein SZT2	A2A9C3	S1650	STSESSASFPRSPGQPSSLR	S12	1.008	0.791
Szt2	KICSTOR complex protein SZT2	A2A9C3	S2135	SQEPISSEDSVAPRSPLDMASSR	S15	1.206	0.171
Szt2	KICSTOR complex protein SZT2	A2A9C3	S2638	FESGGDGSPGR	S8	0.969	0.614
Tab2	TGF-beta-activated kinase 1 and MAP3K7-binding protein 2	Q99K90	S372	NQPTVYIAASPPNTDEMISR	S10	0.993	0.924
Tab2	TGF-beta-activated kinase 1 and MAP3K7-binding protein 2	Q99K90	S450	VLGGNSATSPR	S9	1.147	0.074
Tab3	TGF-beta-activated kinase 1 and MAP3K7-binding protein 3	Q571K4	S389	SPSPISNQPSPR	S3	0.993	0.869
Tab3	TGF-beta-activated kinase 1 and MAP3K7-binding protein 3	Q571K4	Ambiguous	SSSSGSDDYAYTQALLLHQR	S/Y/T	1.428	0.008
Tab3	TGF-beta-activated kinase 1 and MAP3K7-binding protein 3	Q571K4	Ambiguous	NQHSLYTATTPPSSSPSR	T/S/Y	1.059	0.395
Tacc1	Transforming acidic coiled-coil-containing protein 1	F8VQ95	S45	GSGAGEDEAGGPEGDPEEEEDSQAETK	S22	1.076	0.284
Tacc2	Transforming acidic coiled-coil-containing protein 2	E9Q8T1	S2122	VQNSPPVGR	S4	1.018	0.788
Tacc2	Transforming acidic coiled-coil-containing protein 2	E9Q8T1	S2213,T/S	LDNTPASPPRSPTEPSDTPIAK	S7,T/S	0.973	0.662
Taf1	Transcription initiation factor TFIID subunit	D3YZK4	S1834	GLEDSNISYGSYEEPDPK	S11	0.982	0.841
Taf1	Transcription initiation factor TFIID subunit	D3YZK4	Ambiguous	SNTQDTSFSSIGGYEVSEEEEDEEEQR	S/Y/T	0.959	0.878
Taf12	Transcription initiation factor TFIID subunit 12	E9QNT5	S103	LSPENNQVLTK	S2	0.921	0.719
Taf12	Transcription initiation factor TFIID subunit 12	E9QNT5	S103	IAGTPGTGGRLSPENNQVLTK	S12	0.826	0.005
Taf1d	TATA box-binding protein-associated factor RNA polymerase I subunit D	Q9D4V4	S39	TQCAPSPIQK	S6	1.102	0.602
Taf3	Transcription initiation factor TFIID subunit 3	Q5HZG4	S183	RPLDSPEVEEMPSMK	S5	1.046	0.351
Taf3	Transcription initiation factor TFIID subunit 3	Q5HZG4	S183	RPLDSPEVEEMPSMK	S5	1.011	0.829
Taf3	Transcription initiation factor TFIID subunit 3	Q5HZG4	S428	RISGSECATPK	S3	0.977	0.567
Taf6	Transcription initiation factor TFIID subunit 6	Q62311	S532	AAAPPQPSPPPTK	S8	0.976	0.775
Taf6	Transcription initiation factor TFIID subunit 6	Q62311	S653	QEAGDSPPPAPGTPK	S6	1.040	0.320
Taf6	Transcription initiation factor TFIID subunit 6	Q62311	S673	ANGSQPTGPGSPQPAL	S11	1.047	0.209
Taf6	Transcription initiation factor TFIID subunit 6	Q62311	S653,T660	QEAGDSPPPAPGTPK	S6,T13	1.082	0.309
Taf7	Transcription initiation factor TFIID subunit 7	Q9R1C0	S171	RLLSTDAEAVSTR	S4	1.022	0.802
Taf7	Transcription initiation factor TFIID subunit 7	Q9R1C0	Ambiguous	ETENQGLDISSPGMSGHR	S/T	1.164	0.026
Taf7	Transcription initiation factor TFIID subunit 7	Q9R1C0	Ambiguous	LLSTDAEAVSTR	S/T	0.824	0.198
Tagap	T-cell activation Rho GTPase-activating protein	B2RWW0	Ambiguous	SSPGESLGSSPVPSPSCPK	S	0.830	0.080
Tagln2	Transgelin-2	Q9WVA4	S163	NFSDNQLQEGK	S3	0.905	0.063
Taldo1	Transaldolase	A0A1B0GR11	S250	SYEPQEDPGVK	S1	1.169	0.048
Taldo1	Transaldolase	A0A1B0GR11	S282	TIVMGASFR	S7	0.968	0.283
Talpid3	Protein TALPID3	E9PV87	S26	EIVSPNQGNK	S4	0.931	0.217
Talpid3	Protein TALPID3	E9PV87	Ambiguous	AAATSVPGDLSGTETNLLAR	T/S	0.831	0.229
Tanc1	Protein TANC1	Q0VGY8	S1436	QQGPPPAPANDSDNEEDAPASSLK	S12	0.994	0.755
Tanc1	Protein TANC1	Q0VGY8	S267	ADNCSPVAEEETTGSAESVLPK	S5	0.794	0.046
Tanc1	Protein TANC1	Q0VGY8	S267	RADNCSPVAEEETTGSAESVLPK	S6	1.072	0.338
Tanc1	Protein TANC1	Q0VGY8	S445,S	QIASSSPSLSPK	S10,S	1.209	0.100
Tanc1	Protein TANC1	Q0VGY8	Ambiguous	SESGTAYPLPSK	S/T/Y	1.058	0.610
Tanc2	Protein TANC2	A2A690	S1534	EYPSPPPSPLR	S4	0.993	0.898
Tanc2	Protein TANC2	A2A690	S1549	ASPPAESMSVYR	S2	0.983	0.731
Tanc2	Protein TANC2	A2A690	S169	DCSYGAVTSPTSTLESR	S9	1.005	0.926
Tanc2	Protein TANC2	A2A690	S1831	SCDELSPVSPTQGGYPSEPTR	S9	1.293	0.359
Tanc2	Protein TANC2	A2A690	T434	ELPLTQAPSAHSSITSGSCPGTPEMR	T22	1.098	0.633
Tanc2	Protein TANC2	A2A690	S1534,S1538	EYPSPPPSPLR	S4,S8	0.913	0.129
Tanc2	Protein TANC2	A2A690	S1534,S1538	EYPSPPPSPLRR	S4,S8	0.988	0.902
Tanc2	Protein TANC2	A2A690	S400,S404	QIASDSPHASPK	S6,S10	0.969	0.573
Tango6	Transport and Golgi organization protein 6 homolog	Q8C3S2	S561	EAISDDEDEALYQK	S4	1.015	0.693
Tango6	Transport and Golgi organization protein 6 homolog	Q8C3S2	S787	QTSPDISTEGAQKPPR	S3	1.015	0.896
Tank	TRAF family member-associated NF-kappa-B activator	P70347	S107	DNSYGYVPLLEDSDRR	S3	1.066	0.475
Tank	TRAF family member-associated NF-kappa-B activator	P70347	Ambiguous	GLGRDEEDTSFESLSK	T/S	1.895	0.001
Taok1	Serine/threonine-protein kinase TAO1	Q5F2E8	S421	ASDPQSPPQVSR	S6	1.042	0.183
Taok1	Serine/threonine-protein kinase TAO1	Q5F2E8	S965	NSPQALR	S2	1.104	0.569
Taok1	Serine/threonine-protein kinase TAO1	Q5F2E8	Ambiguous	GSSMGVRNSPQALR	S	1.034	0.741
Taok2	Serine/threonine-protein kinase TAO2 (Fragment)	A0A1L1SSV8	S370	AASGGSSGENVGPPAAVPGPLSR	S3	1.098	0.127
Taok2	Serine/threonine-protein kinase TAO2 (Fragment)	A0A1L1SSV8	Ambiguous	RAASGGSSGENVGPPAAVPGPLSR	S	0.931	0.430
Taok3	Serine/threonine-protein kinase TAO3	Q8BYC6	S324	NGPLNESQEEEEDGEQGSNLNR	S7	1.028	0.292
Taok3	Serine/threonine-protein kinase TAO3	Q8BYC6	S359	EVDSLGSIHSIPSTSVSTGSR	S20	0.954	0.517
Taok3	Serine/threonine-protein kinase TAO3	Q8BYC6	S505	EVETHANNSSIELEK	S10	1.042	0.520
Taok3	Serine/threonine-protein kinase TAO3	Q8BYC6	S346,S349	EVDSLGSIHSIPSTSVSTGSR	S7,S10	1.104	0.469
Tapbp	Tapasin	Q3TCU5	S455	ATAASLTIPR	S5	0.995	0.974
Tatdn2	TatD DNase domain-containing 2	B7ZNL9	S371	FSQEEPVPLK	S2	1.186	0.284
Tatdn2	TatD DNase domain-containing 2	B7ZNL9	Ambiguous	FSRSTEEEEVK	S/T	1.170	0.156
Tax1bp1	Tax1-binding protein 1 homolog	Q3UKC1	S632	KLEGQSPQQVSR	S6	1.018	0.727
Tax1bp1	Tax1-binding protein 1 homolog	Q3UKC1	S693	VPSWEDNVVCSQPAR	S3	0.969	0.845
Tbc1d1	TBC1 domain family member 1	Q60949	S231	SFSQPGLR	S3	0.950	0.079
Tbc1d1	TBC1 domain family member 1	Q60949	S621	YHSVSTETPHER	S3	1.118	0.392
Tbc1d1	TBC1 domain family member 1	Q60949	T590	ANTLSHFPVECPAPPEPAQSSPGVSQR	T3	0.975	0.671
Tbc1d1	TBC1 domain family member 1	Q60949	T590	RANTLSHFPVECPAPPEPAQSSPGVSQR	T4	1.217	0.097
Tbc1d1	TBC1 domain family member 1	Q60949	T739	ANHLGDTDGTPVK	T10	1.012	0.706
Tbc1d1	TBC1 domain family member 1	Q60949	T590,S608	ANTLSHFPVECPAPPEPAQSSPGVSQR	T3,S21	0.978	0.852
Tbc1d1	TBC1 domain family member 1	Q60949	T590,S	RANTLSHFPVECPAPPEPAQSSPGVSQR	T4,S	1.189	0.134
Tbc1d1	TBC1 domain family member 1	Q60949	Ambiguous	QSARPSTPEPDCTQLEPTGD	S/T	1.004	0.894
Tbc1d1	TBC1 domain family member 1	Q60949	Ambiguous	RQSARPSTPEPDCTQLEPTGD	T/S	1.043	0.396
Tbc1d10a	TBC1 domain family member 10A	Q5SPX8	S441	AILDAEPGPRPALQPSPSIR	S16	1.071	0.258
Tbc1d10a	TBC1 domain family member 10A	Q5SPX8	S52	ENGPREPAAGGSLSGTR	S12	1.028	0.618
Tbc1d10a	TBC1 domain family member 10A	Q5SPX8	S52	EPAAGGSLSGTR	S7	1.081	0.105
Tbc1d10b	TBC1 domain family member 10B	Q8BHL3	S129	TEEVRASPVPGPGTPTR	S7	1.010	0.833
Tbc1d10b	TBC1 domain family member 10B	Q8BHL3	S22	HGAPAAPSPPPRGSR	S8	0.838	0.313
Tbc1d10b	TBC1 domain family member 10B	Q8BHL3	S22	HGAPAAPSPPPR	S8	1.008	0.877
Tbc1d10b	TBC1 domain family member 10B	Q8BHL3	S22	RHGAPAAPSPPPR	S9	1.002	0.928
Tbc1d10b	TBC1 domain family member 10B	Q8BHL3	S664	AVGGAPSPPPPVR	S7	1.015	0.434
Tbc1d10b	TBC1 domain family member 10B	Q8BHL3	S664	AVGGAPSPPPPVRR	S7	1.020	0.514
Tbc1d10b	TBC1 domain family member 10B	Q8BHL3	T136	ASPVPGPGTPTRTPSR	T9	0.936	0.432
Tbc1d10b	TBC1 domain family member 10B	Q8BHL3	T136	ASPVPGPGTPTR	T9	1.040	0.366
Tbc1d12	TBC1 domain family member 12	E9QQ68	S207	RQSAGDLLPSAGPSAPLPAAEQGPGGTTAR	S3	1.086	0.453
Tbc1d12	TBC1 domain family member 12	E9QQ68	Ambiguous	EGDKNTSPALKS	T/S	1.049	0.277
Tbc1d13	TBC1 domain family member 13	Q8R3D1	S184	SGVTNMSSPHK	S8	0.955	0.405
Tbc1d14	TBC1 domain family member 14	G3UVU5	S112	KQSESEIVPER	S3	1.095	0.256
Tbc1d14	TBC1 domain family member 14	G3UVU5	S709	EMEKGSPSLRH	S6	0.989	0.930
Tbc1d14	TBC1 domain family member 14	G3UVU5	Ambiguous	SLSVPDYGPSLK	S/Y	1.008	0.985
Tbc1d15	TBC1 domain family member 15	Q9CXF4	S662	DDSPTQTLASPNACR	S10	1.067	0.117
Tbc1d15	TBC1 domain family member 15	Q9CXF4	T669	DDSPTQTLASPNACRLTPA	T17	1.058	0.406
Tbc1d16	TBC1 domain family, member 16	A2ABG4	S382	LPSSEIHPEESLYR	S3	1.098	0.395
Tbc1d16	TBC1 domain family, member 16	A2ABG4	T98,S/Y	YITPESSPVR	T3,S/Y	0.983	0.901
Tbc1d17	TBC1 domain family member 17	Q8BYH7	S20	GGVYLHTSAR	S8	1.030	0.528
Tbc1d2	TBC1 domain family member 2A	B1AVH7	S914	APPEGCVSEDEGEGDS	S8	0.988	0.699
Tbc1d22a	TBC1 domain family member 22A	Q8R5A6	S144	SVSESHTPCPSESTGDTVPLQR	S3	0.974	0.636
Tbc1d22b	TBC1 domain family, member 22B	Q80VE5	S116	SQSTTSDVPANYK	S3	1.255	0.002
Tbc1d23	TBC1 domain family member 23	A0A2I3BRD1	Ambiguous	GSISSVDGESCNGSNDR	S	0.918	0.277
Tbc1d25	TBC1 domain family member 25	A1A5B6	S560	QASLDGLQQLR	S3	1.006	0.892
Tbc1d2b	TBC1 domain family member 2B	Q3U0J8	S155	TSPTPGDFPK	S2	1.024	0.157
Tbc1d2b	TBC1 domain family member 2B	Q3U0J8	S959	DTSPDKGELVSDEEEDT	S11	0.980	0.561
Tbc1d2b	TBC1 domain family member 2B	Q3U0J8	S959	ERDTSPDKGELVSDEEEDT	S13	1.021	0.664
Tbc1d2b	TBC1 domain family member 2B	Q3U0J8	S951,S/T	DTSPDKGELVSDEEEDT	S3,S/T	0.907	0.422
Tbc1d2b	TBC1 domain family member 2B	Q3U0J8	Ambiguous	WDSRTSPTPGDFPK	S/T	1.168	0.317
Tbc1d4	TBC1 domain family member 4	Q8BYJ6	S258	GGDPGDEMGVLEVESPVSPDDSLPEKADGTVNSPR	S15	0.927	0.492
Tbc1d4	TBC1 domain family member 4	Q8BYJ6	S348	HASAPSHVQPSDSEK	S3	1.104	0.373
Tbc1d4	TBC1 domain family member 4	Q8BYJ6	S595	LGSMDSFER	S3	1.180	0.081
Tbc1d4	TBC1 domain family member 4	Q8BYJ6	S761	TSSTCSNESLNAGGTPVTPR	S6	1.057	0.299
Tbc1d4	TBC1 domain family member 4	Q8BYJ6	S761,S764	TSSTCSNESLNAGGTPVTPR	S6,S9	1.086	0.589
Tbc1d5	TBC1 domain family member 5	A0A286YDB3	S546	SESMPVQLNK	S3	0.922	0.090
Tbc1d5	TBC1 domain family member 5	A0A286YDB3	S546	SESMPVQLNK	S3	0.886	0.036
Tbc1d5	TBC1 domain family member 5	A0A286YDB3	S587	TISSSPSIESLPGGR	S7	0.987	0.746
Tbc1d5	TBC1 domain family member 5	A0A286YDB3	S600	EFTGSPPPSATK	S5	1.092	0.012
Tbc1d5	TBC1 domain family member 5	A0A286YDB3	S600	EFTGSPPPSATKK	S5	1.028	0.544
Tbc1d5	TBC1 domain family member 5	A0A286YDB3	S587,S590	TISSSPSIESLPGGR	S7,S10	1.023	0.526
Tbc1d5	TBC1 domain family member 5	A0A286YDB3	S600,S/T	EFTGSPPPSATK	S5,S/T	1.497	0.017
Tbc1d8	TBC1 domain family member 8	Q9Z1A9	S1040	GSSSGSCSQECEEPQASAPPEQDSVFAEAGK	S8	1.267	0.189
Tbc1d9b	TBC1 domain family member 9B	Q5SVR0	S1090	DGAHSGDPNSATEEDEPPTPK	S10	1.118	0.082
Tbc1d9b	TBC1 domain family member 9B	Q5SVR0	S1254	QFSTSSDHEPPGVLG	S6	1.057	0.596
Tbc1d9b	TBC1 domain family member 9B	Q5SVR0	S1251,S1253	QFSTSSDHEPPGVLG	S3,S5	1.198	0.113
Tbcb	Tubulin-folding cofactor B	Q9D1E6	S110	YEISPEAYER	S4	1.020	0.764
Tbcb	Tubulin-folding cofactor B	Q9D1E6	S110	YEISPEAYERR	S4	1.005	0.928
Tbcb	Tubulin-folding cofactor B	Q9D1E6	S150	AQQEAEAAQRLSEEK	S12	0.972	0.762
Tbcc	Tubulin-specific Chaperone C	A0A0R4J0M1	S162	DAAGTAQVDAAPVASAAPSPPVTK	S19	1.125	0.506
Tbkbp1	TANK-binding kinase 1-binding protein 1	A2A9T0	S415	SPVPPSCPAPQPRPPPPPGER	S1	0.994	0.893
Tbkbp1	TANK-binding kinase 1-binding protein 1	A2A9T0	S500	AYGGELYGRPLSPR	S12	1.009	0.857
Tbrg1	Transforming growth factor beta regulator 1	Q3UB74	S400	SPSQCSPIQSSD	S6	0.973	0.702
Tcea1	Transcription elongation factor A protein 1	E9PYD5	S111	KEPAISSQNSPEAR	S10	1.039	0.558
Tcea1	Transcription elongation factor A protein 1	E9PYD5	S111	KKEPAISSQNSPEAR	S11	1.106	0.020
Tcea1	Transcription elongation factor A protein 1	E9PYD5	S111	EPAISSQNSPEAR	S9	1.125	0.202
Tcea1	Transcription elongation factor A protein 1	E9PYD5	S111,S	KKEPAISSQNSPEAR	S11,S	0.941	0.023

Tceal5	Transcription elongation factor A protein-like 5	Q8CCT4	S120	GTDDSPKNSQEDLQDR	S5	0.995	0.947
Tcf12	Transcription factor 12 (Fragment)	Q3UXQ3	S216	AGGQAPSSPSYENSLHSLQSR	S8	0.993	0.916
Tcf12	Transcription factor 12 (Fragment)	Q3UXQ3; Q61286	S389;S583	TSSTNEDEDLNPEQK	S3	1.259	0.020
Tcf12	Transcription factor 12	Q61286	S67	GGTTSWGTSGQPSPSYDSSR	S13	0.948	0.438
Tcf12	Transcription factor 12	Q61286	Ambiguous	AGGQAPSSPSYENSLHSLK	S/Y	1.072	0.639
Tcf12	Transcription factor 12 (Fragment)	Q3UXQ3; Q61286	Ambiguous	GRTSSTNEDEDLNPEQK	T/S	1.098	0.224
Tcf20	Transcription factor 20	Q9EPQ8	S567	QLSGQSTSSDTTYK	S3	0.746	0.036
Tcf20	Transcription factor 20	Q9EPQ8	S588	AGSSPTQGAQNEAPR	S4	1.012	0.495
Tcf20	Transcription factor 20	Q9EPQ8	S603	LSTSPATRDEAASPGAK	S4	1.006	0.917
Tcf20	Transcription factor 20	Q9EPQ8	S612	DEAASPGAKDTSLSSEGNTK	S5	1.055	0.180
Tcf20	Transcription factor 20	Q9EPQ8	S994	GNASPGAAAHDSISDYGPQDSR	S4	1.119	0.272
Tcf20	Transcription factor 20	Q9EPQ8	T1699	SLTPPPSSTESK	T3	0.954	0.073
Tcf20	Transcription factor 20	Q9EPQ8	S612,T/S	LSTSPATRDEAASPGAK	S13,S/T	0.952	0.374
Tcf20	Transcription factor 20	Q9EPQ8	T1790,T1792	SASNGSKTDTEEEEEQQQQK	T8,T10	0.993	0.908
Tcf20	Transcription factor 20	Q9EPQ8	Ambiguous	SSSQYHDFAEK	S/Y	1.198	0.072
Tcf20	Transcription factor 20	Q9EPQ8	Ambiguous	GSQEDDPAASQRPPSNSGVK	S	1.178	0.130
Tcf3	Transcription factor E2-alpha	E9PVV1	S529	TSSTDEVLSLEEK	S3	1.265	0.027
Tcf3	Transcription factor E2-alpha	E9PVV1	Ambiguous	TRTSSTDEVLSLEEK	S/T	1.227	0.001
Tcf4	Transcription factor 4	E9Q8G4	S114	DLGSHDNLSPPFVNSR	S9	0.968	0.805
Tcf4	Transcription factor 4	E9Q8G4	S568	SRSSNNDDEDLTPEQK	S1	1.399	0.022
Tcf7l2	Transcription factor 7-like 2	E9QQ89	Ambiguous	SSLVNESETNQNSSSDSEAERRPPPR	S/T	0.863	0.472
Tcirg1	V-type proton ATPase subunit a	Q9JHF5	S43	DLNESVSAFQR	S5	1.061	0.154
Tcirg1	V-type proton ATPase subunit a	Q9JHF5	Ambiguous	RPAGQQDEDTDKLLASPDASTLENSWSPDEEK	S/T	1.097	0.631
Tcirg1	V-type proton ATPase subunit a	Q9JHF5	Ambiguous	LLASPDASTLENSWSPDEEK	T/S	1.037	0.407
Tcof1	Treacle protein	H3BL37	S1164	STSSSPAPTQTLPNSITQR	S5	1.001	0.963
Tcof1	Treacle protein	H3BL37	S1227	KLSGDLEAGAPK	S3	0.953	0.260
Tcof1	Treacle protein	H3BL37	S1252	ASAVSPEKAPMTSK	S5	1.020	0.675
Tcof1	Treacle protein	H3BL37	S1303	LESGEQSDPKSK	S7	1.090	0.269
Tcof1	Treacle protein	H3BL37	S1339	DSASPIQK	S4	1.000	0.999
Tcof1	Treacle protein	H3BL37	S1339	DSASPIQKK	S4	0.888	0.060
Tcof1	Treacle protein	H3BL37	S1351	SAEPAV	S1	1.040	0.816
Tcof1	Treacle protein	H3BL37	S794	AASAPAKESPK	S9	0.986	0.891
Tcof1	Treacle protein	H3BL37	S88	VSDPVSSSESSDQEKEEEAATER	S11	1.066	0.303
Tcof1	Treacle protein	H3BL37	S889	NSSPAVPAPTPEGVQAVNTTK	S3	1.058	0.072
Tcof1	Treacle protein	H3BL37	T944	TSVTTPAALSR	T4	1.011	0.938
Tcof1	Treacle protein	H3BL37	S1160,S/T	STSSSPAPTQTLPNSITQR	S1,S/T	0.876	0.017
Tcof1	Treacle protein	H3BL37	S889,T896	NSSPAVPAPTPEGVQAVNTTK	S3,T10	0.963	0.600
Tcof1	Treacle protein	H3BL37	Ambiguous	EASSGSTPQKPK	S/T	1.016	0.884
Tcof1	Treacle protein	H3BL37	Ambiguous	SAEPLANTVLASETEEEGNAQALGPTAK	T/S	0.942	0.420
Tcof1	Treacle protein	H3BL37	Ambiguous	SAEPLANTVLASETEEEGNAQALGPTAK	T/S	1.059	0.631
Tcp1	T-complex protein 1 subunit alpha	P11983	S544	DDKHGSYENAVHSGALDD	S6	1.166	0.375
Tcp1	T-complex protein 1 subunit alpha	P11983	S551	HGSYENAVHSGALDD	S10	1.050	0.615
Tcp11l2	T-complex protein 11-like protein 2	Q8K1H7	Ambiguous	FAEGVASLSDYECSR	S/Y	1.044	0.828
Tdp1	Tyrosyl-DNA phosphodiesterase 1	B8JJC1	S132	VVDRSPPASLRPQR	S5	1.022	0.630
Tdp1	Tyrosyl-DNA phosphodiesterase 1	B8JJC1	Ambiguous	HVSSPDVTTAQK	S/T	0.953	0.513
Tdrd7	Tudor domain-containing protein 7	A0A0A0MQD1	S893	NGGTPAPGSPAESLR	S9	0.987	0.884
Tec	Tyrosine-protein kinase Tec	P24604	Y518	YVLDDQYTSSSGAK	Y7	0.921	0.073
Tec	Tyrosine-protein kinase Tec	P24604	Ambiguous	YNLFESSIR	S/Y	0.830	0.012
Tecpr1	Tectonin beta-propeller repeat-containing protein 1	Q80VP0	S418	GSGTESAPSDTDASLEVER	S14	1.009	0.679
Tecpr1	Tectonin beta-propeller repeat-containing protein 1	Q80VP0	S438	EALDNSTNLK	S6	1.119	0.071
Tecpr1	Tectonin beta-propeller repeat-containing protein 1	Q80VP0	S413,S418	GSGTESAPSDTDASLEVER	S9,S14	0.859	0.314
Telo2	Telomere length regulation protein TEL2 homolog	Q9DC40	S457	GPSPAPVDTESPVEMPEK	S3	0.991	0.818
Telo2	Telomere length regulation protein TEL2 homolog	Q9DC40	S457	GPSPAPVDTESPVEMPEK	S3	1.135	0.193
Telo2	Telomere length regulation protein TEL2 homolog	Q9DC40	S457,T/S	GPSPAPVDTESPVEMPEK	S3,T/S	1.218	0.052
Telo2	Telomere length regulation protein TEL2 homolog	Q9DC40	Ambiguous	LLSSSSPQP	S	1.102	0.089
Ten1	CST complex subunit TEN1	Q9D7K2	Ambiguous	QQPIGDASTLQTPTPAPQSIPSDSLSLEPENR	T/S	1.131	0.586
Tent4a	Non-canonical poly(A) RNA polymerase PAPD7	A0A0G2JDV2	S552	ILPSPDLDNR	S4	0.892	0.548
Tepsin	AP-4 complex accessory subunit Tepsin	Q3U3N6	S400	QQLQELGAGSPGPVTNK	S10	1.058	0.302
Tepsin	AP-4 complex accessory subunit Tepsin	Q3U3N6	T305	AEATPPNDCQQELNLVR	T4	1.127	0.309
Terf2	Telomeric repeat-binding factor 2	O35144	S412	LLLEEDSQSTEPSPGLNSSHK	S7	1.102	0.625
Terf2ip	Telomeric repeat-binding factor 2-interacting protein 1	Q91VL8	S200	YLLGNAPVSPSSQK	S9	0.876	0.546
Tes	Testin	P47226	S173	CHELSPK	S5	0.984	0.782
Tesk1	Dual specificity testis-specific protein kinase 1	O70146	S439	CRSLPSSPELPR	S3	0.903	0.003
Tesk1	Dual specificity testis-specific protein kinase 1	O70146	S439,S	SLPSSPELPR	S1,S	1.020	0.754
Tesk1	Dual specificity testis-specific protein kinase 1	O70146	S439,S443	CRSLPSSPELPR	S3,S7	0.958	0.330
Tesk1	Dual specificity testis-specific protein kinase 1	O70146	Ambiguous	SLPSSPELPR	S	1.009	0.816
Tet2	Methylcytosine dioxygenase TET2	A0A0G2JF55	Ambiguous	DSISPTTVTPPSQSLLAPR	S/T	1.257	0.170
Tex14	Inactive serine/threonine-protein kinase TEX14	Q7M6U3	S1449	IIVLDQSDLSD	S10	0.809	0.064
Tex2	Testis-expressed protein 2	Q6ZPJ0	S733	SGLLPAHSR	S8	1.012	0.753
Tex2	Testis-expressed protein 2	Q6ZPJ0	S803	SPVQSAESSPTASK	S9	1.110	0.471
Tex2	Testis-expressed protein 2	Q6ZPJ0	S265,S/T	NTGGDSKTAPSSPLTSPSDTR	S12,S/T	1.099	0.146
Tex2	Testis-expressed protein 2	Q6ZPJ0	S265,T/S	TAPSSPLTSPSDTR	S5,T/S	1.024	0.653
Tex2	Testis-expressed protein 2	Q6ZPJ0	Ambiguous	TSSSSPLSSPSK	S/T	1.026	0.797
Tex2	Testis-expressed protein 2	Q6ZPJ0	Ambiguous	SLSTDTSR	T/S	1.117	0.265
Tex2	Testis-expressed protein 2	Q6ZPJ0	Ambiguous	NTGGDSKTAPSSPLTSPSDTR	T/S	1.340	0.047
Tex264	Testis-expressed gene 264	E9Q137	S243	ETSATTLSPGAGNR	S8	1.093	0.045
Tex264	Testis-expressed gene 264	E9Q137	T303	LLGPPRELSTPERGEE	T10	0.924	0.497
Tex264	Testis-expressed gene 264	E9Q137	Ambiguous	ELSTPERGEE	T/S	1.052	0.293
Tfe3	Transcription factor E3	A2AEW1	S333	EISETEAK	S3	1.059	0.209
Tfe3	Transcription factor E3	A2AEW1	S565	SSFSMEEES	S2	0.763	0.136
Tfe3	Transcription factor E3	A2AEW1	S565	SSFSMEEES	S2	1.007	0.848
Tfe3	Transcription factor E3	A2AEW1	S545,S553	AASDPLLSSVSPAVSK	S3,S11	1.047	0.501
Tfe3	Transcription factor E3	A2AEW1	Ambiguous	AASDPLLSSVSPAVSK	S	1.058	0.582
Tfeb	Transcription factor EB	Q3UKG7	S155	EYLSETYGNK	S4	0.982	0.790
Tfeb	Transcription factor EB	Q3UKG7	S525	SSFSMEEGDVL	S2	1.129	0.472
Tfeb	Transcription factor EB	Q3UKG7	S525	SSFSMEEGDVL	S2	1.048	0.374
Tfeb	Transcription factor EB	Q3UKG7	S167,S172	FAAHVSPAQGSPKPAPAASPGVR	S6,S11	1.050	0.561
Tfeb	Transcription factor EB	Q3UKG7	S167,S172,S180	FAAHVSPAQGSPKPAPAASPGVR	S6,S11,S19	1.119	0.040
Tfg	TFG protein	Q9Z1A1	S198	NVMSAFGLTDDQVSGPPSAPTEDR	S18	1.119	0.552
Tfip11	Tuftelin-interacting protein 11	Q9ERA6	S211	TTQSLQDFPVADSEEEAEEEFQK	S13	0.994	0.878
Tfip11	Tuftelin-interacting protein 11	Q9ERA6	S569	LEPLYSPVR	S6	1.078	0.578
Tfip11	Tuftelin-interacting protein 11	Q9ERA6	S60	EEATYGVWAERDSDEERPSFGGK	S13	1.010	0.869
Tfip11	Tuftelin-interacting protein 11	Q9ERA6	S96,S99	GAAEEADSEDSDAEEKPVKQEDFPK	S8,S11	0.995	0.893
Tfpt	TCF3 fusion partner homolog	Q3U1J1	S180	TTATLDPTSPAPGEGPSGR	S9	0.977	0.582
Tfpt	TCF3 fusion partner homolog	Q3U1J1	S180	TTATLDPTSPAPGEGPSGRK	S9	0.834	0.415
Tfpt	TCF3 fusion partner homolog	Q3U1J1	S255	LLPYPTLASPPFD	S9	1.264	0.099
Tfpt	TCF3 fusion partner homolog	Q3U1J1	T252	EPDKLLPYPTLASPPFD	T10	1.106	0.621
Tfrc	Transferrin receptor protein 1	Q62351	S55	LAADEEENADNNMKASVR	S16	1.159	0.204
Tgfb1i1	Transforming growth factor beta-1-induced transcript 1 protein	Q62219	S194	EGCPSPPGQTSK	S5	0.957	0.442
Tgm1	Protein-glutamine gamma-glutamyltransferase K	A0A0R4J293	S94	GSGVNAAGDGTIR	S2	0.983	0.772
Tgoln1	Trans-Golgi network integral membrane protein 1	Q62313	S230	GDKSSEPTEDVETK	S4	1.134	0.159
Tgoln1	Trans-Golgi network integral membrane protein 1	Q62313	S267	VPGPSSSENQEGTLTDSMK	S6	1.147	0.001
Tgoln1	Trans-Golgi network integral membrane protein 1	Q62313	S28	DADSGDSQNPPNQPSK	S4	1.051	0.440
Tgoln1	Trans-Golgi network integral membrane protein 1	Q62313	S28,S31	DADSGDSQNPPNQPSK	S4,S7	0.900	0.245
Tgoln1	Trans-Golgi network integral membrane protein 1	Q62313	Ambiguous	VPGPSSSENQEGTLTDSMK	S/T	0.940	0.837
Tgs1	Trimethylguanosine synthase	Q923W1	S152	NDYEEDDLVVSDDPSSVEHCENNR	S11	1.007	0.880
Tgs1	Trimethylguanosine synthase	Q923W1	S175	AGSEVENLPVENTLAPK	S3	0.919	0.451
Thoc1	THO complex subunit 1	Q8R3N6	S560	TGEDEDEEDNDALLKENESPDVRR	S19	0.960	0.575
Thoc1	THO complex subunit 1	Q8R3N6	S560	ELPPPSEEIKTGEDEDEEDNDALLKENESPDVR	S29	1.094	0.445
Thoc2	THO complex subunit 2	B1AZI6	S1401	SDISEPDREQK	S4	1.051	0.323
Thoc2	THO complex subunit 2	B1AZI6	Ambiguous	IDSHPSPSHSSTVK	S/T	0.958	0.402
Thop1	Thimet oligopeptidase	A0A0R4IZY0	S529	TGSEAPQDLLEK	S3	1.097	0.708
Thrap3	Thyroid hormone receptor-associated protein 3	Q569Z6	S207	DSRPSQAAGDNQGDEAKEQTFSGGTSQDIK	S22	0.988	0.856
Thrap3	Thyroid hormone receptor-associated protein 3	Q569Z6	S243	ASVSDLSPR	S7	0.990	0.783
Thrap3	Thyroid hormone receptor-associated protein 3	Q569Z6	S379	GGFSDADVK	S4	0.969	0.440
Thrap3	Thyroid hormone receptor-associated protein 3	Q569Z6	S379	EKGGFSDADVK	S6	1.025	0.544
Thrap3	Thyroid hormone receptor-associated protein 3	Q569Z6	S557	SSFSITR	S2	1.077	0.345
Thrap3	Thyroid hormone receptor-associated protein 3	Q569Z6	S572	MDSFDEDLARPSGLLAQER	S3	0.994	0.962
Thrap3	Thyroid hormone receptor-associated protein 3	Q569Z6	S679	IDISPSTFR	S4	0.983	0.644
Thrap3	Thyroid hormone receptor-associated protein 3	Q569Z6	S679	RIDISPSTFR	S5	1.016	0.703
Thrap3	Thyroid hormone receptor-associated protein 3	Q569Z6	S924	WAHDKFSGEEGEIEDDESGTENR	S7	1.035	0.821
Thrap3	Thyroid hormone receptor-associated protein 3	Q569Z6	S935	FSGEEGEIEDDESGTENREEK	S13	1.159	0.153
Thrap3	Thyroid hormone receptor-associated protein 3	Q569Z6	S935	FSGEEGEIEDDESGTENR	S13	1.112	0.417
Thrap3	Thyroid hormone receptor-associated protein 3	Q569Z6	S945	FSGEEGEIEDDESGTENREEKDSLQPSAE	S23	0.983	0.717
Thrap3	Thyroid hormone receptor-associated protein 3	Q569Z6	T870	SREEEWDPEYTPK	T11	1.000	0.991
Thrap3	Thyroid hormone receptor-associated protein 3	Q569Z6; Q8K019	T870;T839	EEEWDPEYTPK	T9	0.759	0.146
Thrap3	Thyroid hormone receptor-associated protein 3	Q569Z6	S238,S243	ASVSDLSPR	S2,S7	1.148	0.047
Thrap3	Thyroid hormone receptor-associated protein 3	Q569Z6	S530,S	AVQEKSSSPPPR	S6,S	1.014	0.747
Thrap3	Thyroid hormone receptor-associated protein 3	Q569Z6	Ambiguous	SSSKDSRPSQAAGDNQGDEAK	S	1.034	0.566
Thrap3	Thyroid hormone receptor-associated protein 3	Q569Z6	Ambiguous	EQTFSGGTSQDIK	T/S	1.011	0.866
Thumpd1	THUMP domain-containing protein 1	Q99J36	S86,S88	FIDKDQQPSGSEGEDDDAEAALKK	S9,S11	1.061	0.517
Thumpd2	THUMP domain-containing protein 2	Q9CZB3	S172	QEVAKDHGESQEDKLLQGSPEQGEAVTR	S19	0.944	0.371
Thumpd2	THUMP domain-containing protein 2	Q9CZB3	S172	LLQGSPEQGEAVTR	S5	1.022	0.663
Tial1	Nucleolysin TIAR (Fragment)	A0A0U1RPE1	S103	NFQQVSPPQ	S6	0.900	0.555
Tiam1	T-cell lymphoma invasion and metastasis 1	G3UWG2	S231	ANSLGDLYAQK	S3	0.967	0.284
Tiam1	T-cell lymphoma invasion and metastasis 1	G3UWG2	S358	SNATNSSYSPPTGR	S9	1.079	0.541
Ticam2	TIR domain-containing adapter molecule 2	Q8BJQ4	S30	DSVDADQDGHESDSKNSEEACLR	S12	0.899	0.404
Ticam2	TIR domain-containing adapter molecule 2	Q8BJQ4	S48	GFVEQSSGSEPPTGEQDQPEAK	S7	1.028	0.629

Ticrr	Treslin	Q8BQ33	S1027	TNSGSFYSVSQPK	S3	1.058	0.588
Ticrr	Treslin	Q8BQ33	S599	LHPDGSPDTAVEK	S6	1.052	0.304
Ticrr	Treslin	Q8BQ33	Ambiguous	SVSAITESEQLEELR	S/T	1.187	0.524
Ticrr	Treslin	Q8BQ33	Ambiguous	TVSPAKEETSPPLTK	T/S	1.062	0.420
Tifa	TRAF-interacting protein with FHA domain-containing protein A	Q793I8	S177	SSPSEMDENEL	S4	1.047	0.565
Tifa	TRAF-interacting protein with FHA domain-containing protein A	Q793I8	S177	SSPSEMDENEL	S4	1.008	0.947
Timeless	Protein timeless homolog	Q9R1X4	S1165	QLLDSDEEEDDEGRR	S5	0.918	0.021
Timm8a1	Mitochondrial import inner membrane translocase subunit Tim8 A	Q9WVA2	S94	SKPVFSESLSD	S8	0.951	0.346
Timm8b	Mitochondrial import inner membrane translocase subunit Tim8 B	P62077	Ambiguous	LDSRTENCLSSCVDR	S/T	0.952	0.148
Tinf2	TERF1-interacting nuclear factor 2	A0A2I3BRL3	S333	ALEETPPDSPAAEQENSVNCVDPLR	S9	1.016	0.948
Tinf2	TERF1-interacting nuclear factor 2	A0A2I3BRL3	T329,S333	ALEETPPDSPAAEQENSVNCVDPLR	T5,S9	0.861	0.440
Tinf2	TERF1-interacting nuclear factor 2	A0A2I3BRL3	Ambiguous	HSSPPLTVK	S/T	0.986	0.797
Tiprl	TIP41-like protein	Q8BH58	S270	IDPNPVDSQSTPSE	S13	1.015	0.660
Tiprl	TIP41-like protein	Q8BH58	S270	LVFPERIDPNPVDSQSTPSE	S19	0.935	0.754
Tjap1	Protein incorporated later into tight junctions	I7H459	S315	LSPYPTPSPPHPLYPGR	S8	1.145	0.298
Tjap1	Protein incorporated later into tight junctions	I7H459	S537	KDSLTQAQEQGTVLS	S3	1.098	0.254
Tjp1	Tight junction protein ZO-1	P39447	S617	SREDLSAQPVQTK	S1	1.093	0.284
Tjp2	Tight junction protein ZO-2	Q9Z0U1	S107	VQVAPLQGSPPLSHDDR	S9	1.062	0.450
Tjp2	Tight junction protein ZO-2	Q9Z0U1	S1136	GSYGSDPEEEEYRQQLAAHSKR	S5	0.919	0.461
Tjp2	Tight junction protein ZO-2	Q9Z0U1	S1136	GSYGSDPEEEEYR	S5	1.010	0.856
Tjp2	Tight junction protein ZO-2	Q9Z0U1	S1136	GSYGSDPEEEEYRQQLAAHSK	S5	1.058	0.678
Tjp2	Tight junction protein ZO-2	Q9Z0U1	S186	GLDQEDYGRSR	S10	1.099	0.120
Tjp2	Tight junction protein ZO-2	Q9Z0U1	S209	GLDRDFVSRDHSR	S12	1.272	0.403
Tjp2	Tight junction protein ZO-2	Q9Z0U1	S213	SIDRDYDRDYER	S1	1.031	0.571
Tjp2	Tight junction protein ZO-2	Q9Z0U1	S239	SYHEAYEPDYGGGYSPSYDR	S15	1.148	0.288
Tjp2	Tight junction protein ZO-2	Q9Z0U1	S239	SYHEAYEPDYGGGYSPSYDRR	S15	1.033	0.289
Tjp2	Tight junction protein ZO-2	Q9Z0U1	S895	MSYLTAMGADYLSCDSR	S13	1.000	1.000
Tjp2	Tight junction protein ZO-2	Q9Z0U1	S968	DASPPPAFKPEPPK	S3	1.044	0.306
Tjp3	Tight junction protein ZO-3	Q9QXY1	S111	ASPASGHQLSDQEEADHGR	S10	1.373	0.019
Tjp3	Tight junction protein ZO-3	Q9QXY1	S195	RNSEEFGVK	S3	1.123	0.126
Tjp3	Tight junction protein ZO-3	Q9QXY1	S343	AIAEPESPGESR	S7	0.988	0.915
Tjp3	Tight junction protein ZO-3	Q9QXY1	S584	SREDLSALTR	S1	1.038	0.669
Tjp3	Tight junction protein ZO-3	Q9QXY1	Ambiguous	GQRSPEDSQTDSPVETPQPR	S/T	0.957	0.618
Tjp3	Tight junction protein ZO-3	Q9QXY1	Ambiguous	VPSRQSLEDR	S	0.989	0.803
Tkt	Transketolase	P40142	S295	ILATPPQEDAPSVDIANIR	S12	1.176	0.316
Tkt	Transketolase	P40142	S590	LAVSQVPR	S4	0.946	0.588
Tkt	Transketolase	P40142	T305	MPTPPSYK	T3	0.930	0.123
Tldc1	TLD domain-containing protein 1	Q8K0P3	S438	SLLEISGR	S6	1.082	0.311
Tle1	Transducin-like enhancer protein 1	Q5SQA3	S294	DASGSPASTASSGSSSSLK	S5	0.984	0.768
Tle3	Transducin-like enhancer protein 3	Q08122	S203	ERESSTNNSVSPSESLR	S11	0.998	0.965
Tle3	Transducin-like enhancer protein 3	Q08122	S203	ESSTNNSVSPSESLR	S9	1.050	0.176
Tle3	Transducin-like enhancer protein 3	Q08122	S267	VSPAHSPPENGLDK	S6	1.028	0.842
Tle3	Transducin-like enhancer protein 3	Q08122	S286	DAPTSPASVASSSSTPSSK	S5	0.971	0.454
Tle3	Transducin-like enhancer protein 3	Q08122	T328	NDAPTPGTSTTPGLR	T5	1.027	0.793
Tle3	Transducin-like enhancer protein 3	Q08122	S263,S267	VSPAHSPPENGLDK	S2,S6	1.110	0.188
Tle3	Transducin-like enhancer protein 3	Q08122	S286,T296	DAPTSPASVASSSSTPSSK	S5,T15	0.944	0.618
Tle3	Transducin-like enhancer protein 3	Q08122	T328,S/T	NDAPTPGTSTTPGLR	T5,S/T	1.065	0.399
Tle4	Transducin-like enhancer protein 4	Q62441	S208	SSSVSPSASFR	S5	0.972	0.343
Tle4	Transducin-like enhancer protein 4	Q62441	S292	DAPISPASVASSSSTPSSK	S5	1.015	0.832
Tle4	Transducin-like enhancer protein 4	Q62441	S206,S208	SSSVSPSASFR	S3,S5	1.011	0.816
Tle4	Transducin-like enhancer protein 4	Q62441	S292,S295	DAPISPASVASSSSTPSSK	S5,S8	1.033	0.695
Tlk1	Serine/threonine-protein kinase tousled-like 1	Q8C0V0	T21	LSTSPTPGSAAAAR	T3	0.947	0.421
Tlk1	Serine/threonine-protein kinase tousled-like 1	Q8C0V0	Ambiguous	ISDYFEYQGGNGSSPVR	S/Y	0.973	0.812
Tlk1	Serine/threonine-protein kinase tousled-like 1	Q8C0V0	Ambiguous	FTGVATGSTGSTGSCSVGAK	S/T	0.929	0.578
Tlk2	Serine/threonine-protein kinase tousled-like 2	B1ASU9	S111	SSPQHSLSNPLPR	S2	0.949	0.466
Tlk2	Serine/threonine-protein kinase tousled-like 2	B1ASU9	S749	SVSTSSPAGAAIASTSGASNNSSSN	S24	0.955	0.781
Tlk2	Serine/threonine-protein kinase tousled-like 2	B1ASU9	Ambiguous	ISDYFEFAGGSGPGTSPGR	S/T/Y	0.964	0.725
Tln1	Talin-1	P26039	S1225	LLSDSLPPSTGTFQEAQSR	S3	0.972	0.730
Tln1	Talin-1	P26039	S1225	RLLSDSLPPSTGTFQEAQSR	S4	1.015	0.863
Tln1	Talin-1	P26039	S1328	ALSTDPASPNLK	S8	0.945	0.093
Tln1	Talin-1	P26039	S2040	VLVQNAAGSQEK	S9	0.949	0.393
Tln1	Talin-1	P26039	S2162	QELAVFCSPEPPAK	S8	1.477	0.209
Tln1	Talin-1	P26039	S405	SKDHFGLEGDEESTMLEDSVSPK	S1	0.984	0.849
Tln1	Talin-1	P26039	S425	DHFGLEGDEESTMLEDSVSPK	S19	1.027	0.765
Tln1	Talin-1	P26039	S458	SGASGPENFQVGSMPPAQQQITSGQMHR	S4	0.935	0.452
Tln1	Talin-1	P26039	Y1116	LLGEIAQGNENYAGIAAR	Y12	0.914	0.451
Tln1	Talin-1	P26039	Y1945	AGALQCSPSDVYTK	Y12	0.814	0.149
Tln1	Talin-1	P26039	Y26	TMQFEPSTMVYDACR	Y11	0.945	0.318
Tln1	Talin-1	P26039	Y70	ALDYYMLR	Y4	0.915	0.051
Tln1	Talin-1	P26039	Y70	ALDYYMLR	Y4	0.981	0.879
Tln1	Talin-1	P26039	Ambiguous	IGITNHDEYSLVR	Y/S/T	1.086	0.304
Tln2	Talin-2	A0A1L1SQ51	T1845	LDEGTPPEPK	T5	1.091	0.480
Tm7sf2	Delta(14)-sterol reductase	Q71KT5	S/Y	ALVAPASALAPGGNSGNSMYDFFLGR	S/Y	0.993	0.952
Tmbim1	Protein lifeguard 3	Q3U717	S79	AGSDSFRPGEWDDR	S3	0.981	0.134
Tmc6	Transmembrane channel-like protein	B1ATB3	S809	TQDTTEPPAWHEDGGDQKEPCNPRSP	S25	1.243	0.018
Tmc8	Transmembrane channel-like protein	B0QZP7	S663	LLPELSPEPGSPHSR	S11	1.161	0.129
Tmc8	Transmembrane channel-like protein	B0QZP7	S683	SFCPGFPCPGSPGPR	S11	0.699	0.155
Tmc8	Transmembrane channel-like protein	B0QZP7	S701	LSSSSLGAPSASVPASR	S5	0.883	0.164
Tmc8	Transmembrane channel-like protein	B0QZP7	S6,S9	QWSVQSGPAPR	S3,S6	0.967	0.442
Tmc8	Transmembrane channel-like protein	B0QZP7	S683,T688	SFCPGFPCPGSPGPRTPR	S11,T16	0.981	0.914
Tmcc3	Transmembrane and coiled-coil domain protein 3	Q8R310	S235	NSLEEFRPEASPR	S11	0.988	0.782
Tmco3	Transmembrane and coiled-coil domain-containing protein 3	Q8BH01	S85	DTSLSGEEK	S5	0.994	0.917
Tmco3	Transmembrane and coiled-coil domain-containing protein 3	Q8BH01	S85	AVEKDTSLSGEEK	S9	1.006	0.936
Tmed8	Protein TMED8	Q3UHI4	S14	QAAEGPAFWSPAAR	S10	0.996	0.977
Tmem104	Transmembrane protein 104	Q3TB48	S94	RMETHKEEDDEDSSTASDSDLLSQDNYER	S17	1.060	0.694
Tmem104	Transmembrane protein 104	Q3TB48	Ambiguous	METHKEEDDEDSSTASDSDLLSQDNYER	S/T/Y	0.994	0.937
Tmem104	Transmembrane protein 104	Q3TB48	Ambiguous	METHKEEDDEDSSTASDSDLLSQDNYER	S/T/Y	1.017	0.887
Tmem104	Transmembrane protein 104	Q3TB48	Ambiguous	METHKEEDDEDSSTASDSDLLSQDNYER	S/T/Y	0.960	0.605
Tmem106a	Transmembrane protein 106A	Q8VC04	S54	AASSSFVTCPTCQGNGEIPQEQEK	S5	1.008	0.962
Tmem106b	Transmembrane protein 106B	Q80X71	Ambiguous	NGLVSSEVHNEDGR	S	0.957	0.731
Tmem115	Transmembrane protein 115	Q9WUH1	T329	TDSPLPLEEASTPPGK	T12	1.063	0.271
Tmem120a	Transmembrane protein 120A	Q8C1E7	S50	LQANCTNSITR	S8	0.945	0.560
Tmem123	Porimin	Q91Z22	S187	YRSIDEHDAII	S3	1.053	0.392
Tmem131	Transmembrane protein 131	O70472	T1598,T/S	QTSPTPASPSLPTAPCPFTSR	T5,T/S	1.150	0.409
Tmem131	Transmembrane protein 131	O70472	Ambiguous	QTSPTPASPSLPTAPCPFTSR	S/T	1.000	1.000
Tmem131	Transmembrane protein 131	O70472	Ambiguous	RSSDPWSNSHFPHEN	S	1.022	0.563
Tmem131	Transmembrane protein 131	O70472	Ambiguous	TSPQAAASQSTSK	T/S	1.191	0.360
Tmem132c	Transmembrane protein 132C	E9QP73	T5,T8	MDGVTAATMRSEGAAPR	T5,T8	0.876	0.283
Tmem134	Transmembrane protein 134	Q8R0J4	S70	YQNLENDEDGAQASPEPDGGVSTR	S14	0.713	0.054
Tmem150b	Modulator of macroautophagy TMEM150B	Q8R218	Ambiguous	LDSSLPQAPSGSPNIQMAQVL	S	0.827	0.611
Tmem154	Transmembrane protein 154	Q8C4Q9	S113	QEPSSQGSQSALQTHELGGETLK	S8	1.143	0.084
Tmem154	Transmembrane protein 154	Q8C4Q9	S177	EEKEPNPSPSDNES	S10	0.989	0.885
Tmem160	Transmembrane protein 160	Q9D938	S48	AGASPPPVSELDR	S4	1.174	0.212
Tmem179b	Transmembrane protein 179B	Q9CY24	Ambiguous	GDQEWSSETDALVGHHQSHS	S/T	1.084	0.439
Tmem184b	Transmembrane protein 184B	A0A2I3BQI5	Ambiguous	TLLLSSDDEF	S/T	0.967	0.804
Tmem185a	Transmembrane protein 185A	A2AF53	S333	VVITQSPGK	S6	1.021	0.746
Tmem186	Transmembrane protein 186	Q9CR76	S48	EKSPGTETETFHTIYR	S3	1.072	0.566
Tmem19	Transmembrane protein 19	Q91W52	S293	TGLVVSSPTQETK	S6	0.994	0.937
Tmem192	Transmembrane protein 192	Q9CXT7	S221	IYAYPSNTASETGFR	S10	0.761	0.173
Tmem201	Transmembrane protein 201	A2A8U2	S441	RVSPSSLPGR	S3	1.083	0.010
Tmem206	Transmembrane protein 206	Q9D771	Ambiguous	DKELVQVQGPGVVPGVDNESASSSIR	S	0.729	0.200
Tmem209	Transmembrane protein 209	Q8BRG8	Ambiguous	LGSPDSTSPSTSPTFWNYSR	S/T/Y	0.951	0.770
Tmem221	Transmembrane protein 221	Q8K071	S190	ELSPPSFEDEPARPSEDSK	S6	1.005	0.903
Tmem230	Transmembrane protein 230	Q8CIB6	S24	LASTDDGYIDLQFK	S3	1.299	0.043
Tmem245	Transmembrane protein 245	D3YWD3	S12	GGPAEAPSPR	S8	1.113	0.108
Tmem245	Transmembrane protein 245	D3YWD3	S329	SSPSSPSPTLGR	S7	1.021	0.514
Tmem245	Transmembrane protein 245	D3YWD3	S878	DISEDLKSSVD	S9	1.055	0.609
Tmem245	Transmembrane protein 245	D3YWD3	S12,S16	GGPAEAPSPRGSPRPESR	S8,S12	1.006	0.920
Tmem245	Transmembrane protein 245	D3YWD3	S320,S/T	GEPPPALSASSSSSSRSSPSSPSPTLGR	S14,S/T	1.058	0.679
Tmem245	Transmembrane protein 245	D3YWD3	S323,S326	SSPSSPSPTLGR	S1,S4	0.995	0.856
Tmem259	Membralin	Q8CIV2	Ambiguous	RPTAPSTPDSSRPDPGVPLEDAPAPAGS	S/T	1.097	0.127
Tmem26	Transmembrane protein 26	Q3UP23	T365	TSPVTSEESYPTTP	T13	1.064	0.031
Tmem30a	Cell cycle control protein 50A	Q8VEK0	T363	NSSNTADITI	T9	0.961	0.743
Tmem40	Transmembrane protein 40	E9QN39	S170	RGSGSAEGEVEASQLR	S3	1.116	0.390
Tmem43	Transmembrane protein 43	Q9DBS1	Ambiguous	VTSEPQPGFLER	S/T	0.956	0.805
Tmem51	Transmembrane protein 51	Q99LG1	S114	IQQQAGTVPHSQEEDSQEEEEDVSSR	S16	1.079	0.193
Tmem51	Transmembrane protein 51	Q99LG1	S129	YYVPSYEEVMNTGYPETR	S5	1.183	0.135
Tmem51	Transmembrane protein 51	Q99LG1	S122,S/T	IQQQAGTVPHSQEEDSQEEEEDVSSR	S24,S/T	1.068	0.741
Tmem63a	CSC1-like protein 1	Q91YT8	S767	TALSPQQQQTYGAIR	S4	0.926	0.382
Tmem63b	CSC1-like protein 2	Q3TWI9	Ambiguous	LTSVSSSVDFDQR	T/S	0.750	0.429
Tmf1	TATA element modulatory factor	B9EKI3	S315	LNESCCSSDAFER	S7	1.028	0.748
Tmf1	TATA element modulatory factor	B9EKI3	S329	IDSFSVQSLDSR	S8	0.955	0.682
Tmf1	TATA element modulatory factor	B9EKI3	S340	SVSEINSDDELPGK	S7	1.059	0.049
Tmf1	TATA element modulatory factor	B9EKI3	S334,S340	SVSEINSDDELPGK	S1,S7	0.984	0.699
Tmod3	Tropomodulin-3	Q9JHJ0	S300	QQLGTSVELEMAK	S6	0.978	0.767
Tmpo	Lamina-associated polypeptide 2, isoforms beta/delta/epsilon/gamma	Q61029; Q61033	S155;S155	LREQGTESR	S8	1.013	0.831
Tmpo	Lamina-associated polypeptide 2, isoforms beta/delta/epsilon/gamma	Q61029	S183	QNGSNDSDRYSDNDEDSKIELK	S11	0.998	0.966
Tmpo	Lamina-associated polypeptide 2, isoforms beta/delta/epsilon/gamma	Q61029	S288	ASSNESLVANR	S3	1.061	0.222

Tmpo	Lamina-associated polypeptide 2, isoforms beta/delta/epsilon/gamma	Q61029; Q61033	S82;S82	GPPDFSSDEEREPTPVLGSGASVGR	S22	1.039	0.429
Tmpo	Lamina-associated polypeptide 2, isoforms beta/delta/epsilon/gamma	Q61029; Q61033	T159;T159	SSTPLPTVSSSAENTR	T3	1.048	0.235
Tmpo	Lamina-associated polypeptide 2, isoforms beta/delta/epsilon/gamma	Q61029	T354	EMFPYEASTPTGISASCR	T9	0.845	0.333
Tmpo	Lamina-associated polypeptide 2, isoforms beta/delta/epsilon/gamma	Q61029; Q61033	S155,S158,S158	LREQGTESRSSTPLPTVSSSAENTR	S8,S11	0.993	0.909
Tmpo	Lamina-associated polypeptide 2, isoforms beta/delta/epsilon/gamma	Q61029; Q61033	S158,T159,T159	EQGTESRSSTPLPTVSSSAENTR	S9,T10	1.039	0.340
Tmpo	Lamina-associated polypeptide 2, isoforms beta/delta/epsilon/gamma	Q61029	S179,S183	QNGSNDSDRYSDNDEDSKIELK	S7,S11	1.014	0.861
Tmpo	Lamina-associated polypeptide 2, isoforms beta/delta/epsilon/gamma	Q61029; Q61033	S66,S67,S67	GPPDFSSDEEREPTPVLGSGASVGR	S6,S7	1.067	0.006
Tmpo	Lamina-associated polypeptide 2, isoforms beta/delta/epsilon/gamma	Q61029; Q61033	T159,S167,S167	SSTPLPTVSSSAENTR	T3,S11	1.006	0.848
Tmpo	Lamina-associated polypeptide 2, isoforms beta/delta/epsilon/gamma	Q61029; Q61033	S167,T/S;S167,T/S	LREQGTESRSSTPLPTVSSSAENTR	S20,T/S	0.978	0.565
Tmpo	Lamina-associated polypeptide 2, isoforms alpha/zeta	Q61033	S308	LVSAAASPSLIR	S7	1.112	0.523
Tmpo	Lamina-associated polypeptide 2, isoforms alpha/zeta	Q61033	S368	SQVISPPLAQAIR	S5	1.179	0.685
Tmpo	Lamina-associated polypeptide 2, isoforms alpha/zeta	Q61033	S411	RLSQSSYQDSESLSPPR	S3	1.062	0.069
Tmpo	Lamina-associated polypeptide 2, isoforms alpha/zeta	Q61033	S422	LSQSSYQDSESLSPPR	S13	1.048	0.168
Tmpo	Lamina-associated polypeptide 2, isoforms beta/delta/epsilon/gamma	Q61029; Q61033	Ambiguous	EQGTESRSSTPLPTVSSSAENTR	S/T	0.988	0.591
Tmpo	Lamina-associated polypeptide 2, isoforms beta/delta/epsilon/gamma	Q61029; Q61033	Ambiguous	LREQGTESRSSTPLPTVSSSAENTR	S/T	0.966	0.606
Tmppe	Transmembrane protein with metallophosphoesterase domain	D3Z286	S315	ISAPGDPDSAGRDR	S9	1.072	0.044
Tmppe	Transmembrane protein with metallophosphoesterase domain	D3Z286	S315	ISAPGDPDSAGR	S9	1.046	0.345
Tmsb4x	Thymosin beta-4	P20065	S50	ETIEQEKQAGES	S12	1.005	0.904
Tmub1	Transmembrane and ubiquitin-like domain-containing protein 1	E9QN77	S86	EEAPGAESPSLR	S8	1.136	0.245
Tmx1	Thioredoxin-related transmembrane protein 1	Q8VBT0	S245	VEEEQEADEEDVSEEEAEDREGASK	S13	0.980	0.353
Tmx1	Thioredoxin-related transmembrane protein 1	Q8VBT0	S245	VEEEQEADEEDVSEEEAEDR	S13	1.029	0.722
Tmx1	Thioredoxin-related transmembrane protein 1	Q8VBT0	S245	KVEEEQEADEEDVSEEEAEDR	S14	1.088	0.236
Tmx1	Thioredoxin-related transmembrane protein 1	Q8VBT0	S245	KVEEEQEADEEDVSEEEAEDREGASK	S14	1.053	0.339
Tmx1	Thioredoxin-related transmembrane protein 1	Q8VBT0	Ambiguous	CVGLPSATDTS	T/S	1.016	0.624
Tmx4	Thioredoxin-related transmembrane protein 4	Q8C0L0	S304	EGSVSPKEDGAHPADTQDVVEDALR	S5	1.065	0.269
Tnfaip1	B/POZ domain-containing adapter for CUL3-mediated RhoA degradation proteir	O70479	S278	SQASPSEDEDTFELR	S4	0.957	0.347
Tnfaip3	Tumor necrosis factor alpha-induced protein 3	Q60769	S535	TTAEPSSSLTSSIPASCHQR	S8	0.961	0.699
Tnfaip3	Tumor necrosis factor alpha-induced protein 3	Q60769	S571	TGNVSPSGCLSQAAR	S5	1.040	0.519
Tnfrsf21	Tumor necrosis factor receptor superfamily member 21	Q9EPU5	S315	LLPSSMEATGEK	S4	0.922	0.155
Tnik	Tnik protein	B2RQ80	S707	ASNPDLR	S2	1.045	0.474
Tnik	Tnik protein	B2RQ80	Ambiguous	TTSISPALAR	S/T	1.032	0.597
Tnip1	TNFAIP3-interacting protein 1	E9QM75	S279	EGLCGQPSSPKPEGAGK	S9	1.048	0.496
Tnip1	TNFAIP3-interacting protein 1	E9QM75	S416	YLQDQLSPLTR	S7	1.133	0.321
Tnip2	TNFAIP3-interacting protein 2	Q99JG7	S187	QCAQGDAGEKSPAELEQTSSDASGQSVIK	S11	1.026	0.800
Tnip2	TNFAIP3-interacting protein 2	Q99JG7	S414	CFSDEQGEAFLR	S3	1.161	0.060
Tnip3	TNFAIP3-interacting protein 3	A0A1D5RMN0	S98	DLTNSLER	S5	1.181	0.042
Tnk2	Activated CDC42 kinase 1	G3X9X7	S772	VELSPAPSGEETSR	S4	0.990	0.872
Tnk2	Activated CDC42 kinase 1	G3X9X7	S808,S/T	TPSPLVPPGSSPLPHR	S3,S/T	1.404	0.131
Tnks1bp1	182 kDa tankyrase-1-binding protein	P58871	S1022	DVGHLEEGASGGLLSPSTPHSR	S15	1.011	0.864
Tnks1bp1	182 kDa tankyrase-1-binding protein	P58871	S1063	MQAESQSPTNVDLEDKER	S7	0.993	0.765
Tnks1bp1	182 kDa tankyrase-1-binding protein	P58871	S1136	SGGGHFVPPGETK	S1	0.973	0.282
Tnks1bp1	182 kDa tankyrase-1-binding protein	P58871	S1290	NMAPGAGCSPGEPR	S9	1.122	0.196
Tnks1bp1	182 kDa tankyrase-1-binding protein	P58871	S1375	SSGSLSPGLETEDPLEAR	S6	1.021	0.798
Tnks1bp1	182 kDa tankyrase-1-binding protein	P58871	S1611	ASRVPSSDEEVVEEPQSR	S6	1.091	0.331
Tnks1bp1	182 kDa tankyrase-1-binding protein	P58871	S1657	NRSAEEGEVTESK	S3	1.059	0.258
Tnks1bp1	182 kDa tankyrase-1-binding protein	P58871	S602	ENYEDQEPLVGHESPITLAAR	S14	1.040	0.764
Tnks1bp1	182 kDa tankyrase-1-binding protein	P58871	S763	SPVGDTGLGK	S1	1.129	0.301
Tnks1bp1	182 kDa tankyrase-1-binding protein	P58871	S796	HSLGQEVIGIGGSQDESEVPVR	S2	0.872	0.108
Tnks1bp1	182 kDa tankyrase-1-binding protein	P58871	S866	DSLGSFSTR	S2	0.992	0.835
Tnks1bp1	182 kDa tankyrase-1-binding protein	P58871	S866	RDSLGSFSTR	S3	1.076	0.235
Tnks1bp1	182 kDa tankyrase-1-binding protein	P58871	S887	ASVSTNQDTDENDQELGMK	S2	1.173	0.196
Tnks1bp1	182 kDa tankyrase-1-binding protein	P58871	S887	RASVSTNQDTDENDQELGMK	S3	1.075	0.433
Tnks1bp1	182 kDa tankyrase-1-binding protein	P58871	S976	SLSSGFSPEEAQQQDEEFEK	S3	1.333	0.009
Tnks1bp1	182 kDa tankyrase-1-binding protein	P58871	T533	GEGVSQVGPGTPPAPESPR	T11	1.000	1.000
Tnks1bp1	182 kDa tankyrase-1-binding protein	P58871	S1373,S1375	SSGSLSPGLETEDPLEAR	S4,S6	1.186	0.055
Tnks1bp1	182 kDa tankyrase-1-binding protein	P58871	S1611,S1612	VPSSDEEVVEEPQSR	S3,S4	1.054	0.406
Tnks1bp1	182 kDa tankyrase-1-binding protein	P58871	S1611,S1612	ASRVPSSDEEVVEEPQSR	S6,S7	1.004	0.930
Tnks1bp1	182 kDa tankyrase-1-binding protein	P58871	T533,S539	GEGVSQVGPGTPPAPESPR	T11,S17	1.203	0.177
Tnks1bp1	182 kDa tankyrase-1-binding protein	P58871	Ambiguous	SQEGTAEIPAECQEEHSKTPEER	S/T	0.935	0.346
Tnks1bp1	182 kDa tankyrase-1-binding protein	P58871	Ambiguous	GYSSQDAEEQDREFEKR	S/Y	1.369	0.022
Tnks1bp1	182 kDa tankyrase-1-binding protein	P58871	Ambiguous	GYSSQDAEEQDREFEK	S/Y	1.133	0.268
Tnks1bp1	182 kDa tankyrase-1-binding protein	P58871	Ambiguous	LANISVPASESPR	S	0.966	0.459
Tnks1bp1	182 kDa tankyrase-1-binding protein	P58871	Ambiguous	VPSSDEEVVEEPQSR	S	1.010	0.940
Tnrc18	Trinucleotide repeat-containing gene 18 protein	Q80WC3	S348	EREPGRPGVLQGPPGSPR	S16	1.097	0.300
Tnrc18	Trinucleotide repeat-containing gene 18 protein	Q80WC3	S917,S921,S925	AVSPPPSPRASPVTSLK	S3,S7,S11	1.044	0.278
Tnrc6a	Trinucleotide repeat-containing gene 6A protein	Q3UHK8	S724	QNTAWDTETSPRGER	S10	1.018	0.681
Tnrc6a	Trinucleotide repeat-containing gene 6A protein	Q3UHK8	Ambiguous	GMQQPPAQPLSSSQPNLR	S	0.784	0.062
Tnrc6b	Trinucleotide repeat-containing gene 6B protein	Q8BKI2	S1793	MGSPAPLLPGDLLGGGSDSI	S3	1.131	0.274
Tnrc6b	Trinucleotide repeat-containing gene 6B protein	Q8BKI2	S1793	MGSPAPLLPGDLLGGGSDSI	S3	1.018	0.942
Tnrc6b	Trinucleotide repeat-containing gene 6B protein	Q8BKI2	S913	DDEPSGWEEPSPQSISR	S11	0.875	0.166
Tnrc6c	Trinucleotide repeat-containing gene 6C protein	B1ATC3	S675	QNTAWEFEESPR	S10	1.086	0.627
Tnrc6c	Trinucleotide repeat-containing gene 6C protein	B1ATC3	S675	QNTAWEFEESPRSER	S10	1.046	0.515
Tnrc6c	Trinucleotide repeat-containing gene 6C protein	B1ATC3	S924	DSSEATGWEEPSPPSIR	S12	0.986	0.931
Tnrc6c	Trinucleotide repeat-containing gene 6C protein	B1ATC3	T986	AETPPSHQAGTQLNR	T3	1.096	0.435
Tns1	Tensin 1	E9Q0S6	S1062	AASDGQYENQSPEATSPR	S11	0.934	0.474
Tns1	Tensin 1	E9Q0S6	S1346	TVGTNTPPSPGFGRR	S9	0.844	0.035
Tns1	Tensin 1	E9Q0S6	S1346	TVGTNTPPSPGFGR	S9	1.082	0.470
Tns1	Tensin 1	E9Q0S6	S1363	AVNPTMAAPGSPSLSHR	S11	1.025	0.148
Tns1	Tensin 1	E9Q0S6	S1468	HLGGSGSVVPGSPSLDR	S12	0.912	0.171
Tns1	Tensin 1	E9Q0S6	S1535	QGSPTPALPEKR	S3	0.950	0.225
Tns1	Tensin 1	E9Q0S6	S1535	QGSPTPALPEK	S3	0.972	0.650
Tns1	Tensin 1	E9Q0S6	S792	SQSFPDVEPQLPQAPTR	S3	1.247	0.055
Tns1	Tensin 1	E9Q0S6	S836	QQERSPLQSLAR	S5	0.948	0.468
Tns1	Tensin 1	E9Q0S6	T1015	ETTSDPSRTPEEEPLNLEGLVAHR	T9	1.176	0.058
Tns1	Tensin 1	E9Q0S6	Ambiguous	VSSPPPTITQQGK	S/T	0.930	0.495
Tns3	Tensin-3	Q5SSZ5	S648	GISNGPNPPDTQQLCPGK	S3	1.335	0.009
Tns3	Tensin-3	Q5SSZ5	S745	CSGRLDSVDGPGR	S7	1.177	0.026
Tns3	Tensin-3	Q5SSZ5	S752	LDSVDGPGRSPGR	S10	1.071	0.095
Tns3	Tensin-3	Q5SSZ5	S752	LDSVDGPGRSPGRQGDDPIGGR	S10	0.978	0.808
Tns3	Tensin-3	Q5SSZ5	S844	ESSMCLTPSFPVSPETPYVK	S13	1.232	0.084
Tns3	Tensin-3	Q5SSZ5	S946	QWVESSPK	S6	0.954	0.568
Tns3	Tensin-3	Q5SSZ5	Y601	QQQMVAAHQYSFASDGEAR	Y10	0.909	0.142
Tns3	Tensin-3	Q5SSZ5	T838,S844	ESSMCLTPSFPVSPETPYVK	T7,S13	1.630	0.262
Tns3	Tensin-3	Q5SSZ5	Ambiguous	YPPFSPPEPQLSSPASLHK	S/Y	0.997	0.970
Toe1	Target of EGR1 protein 1	Q9D2E2	S349	SLQSQPGTQTLAEAEDGPPTK	S1	1.479	0.001
Toe1	Target of EGR1 protein 1	Q9D2E2	Ambiguous	RTSAETADVATSELLVNQASTNPVPGDGLHR	T/S	1.118	0.602
Tom1	Target of Myb protein 1	Q3UDC3	S172	TVFNSETPSR	S5	1.111	0.022
Tom1	Target of Myb protein 1	Q3UDC3	S180	TVFNSETPSRQNSVSSNTSQR	S13	1.178	0.166
Tom1	Target of Myb protein 1	Q3UDC3	S429	QQSTGAIPATQAR	S3	1.150	0.218
Tom1	Target of Myb protein 1	Q3UDC3	S486,S496	AADRLPNLASPSAEGPPRPSPGTAPR	S10,S20	1.173	0.143
Tom1	Target of Myb protein 1	Q3UDC3	Ambiguous	AADRLPNLASPSAEGPPRPSPGTAPR	T/S	0.972	0.441
Tom1l2	TOM1-like protein 2	Q5SRX1	S479	AAETVPDLPSPPTEAPAPASNTSTR	S10	1.137	0.063
Tomm20	Mitochondrial import receptor subunit TOM20 homolog	Q9DCC8	S135	IVSAQSLAEDDVE	S3	1.003	0.964
Tomm34	Mitochondrial import receptor subunit TOM34	Q9CYG7	S186	VPSAGDVER	S3	1.021	0.718
Tomm34	Mitochondrial import receptor subunit TOM34	Q9CYG7	S186	SRVPSAGDVER	S5	1.025	0.571
Tomm34	Mitochondrial import receptor subunit TOM34	Q9CYG7	S8	VSDSVEQLR	S4	1.076	0.119
Tomm34	Mitochondrial import receptor subunit TOM34	Q9CYG7	S186,T/S	EATATKSRVPSAGDVER	S11,T/S	0.808	0.009
Tomm70	Mitochondrial import receptor subunit TOM70	Q9CZW5	S94	ASPALGSGHHDGSGDSLEMSSLDR	S2	1.099	0.693
Tomm70	Mitochondrial import receptor subunit TOM70	Q9CZW5	S94	ASPALGSGHHDGSGDSLEMSSLDR	S2	1.040	0.734
Tomm70	Mitochondrial import receptor subunit TOM70	Q9CZW5	S94,S	ASPALGSGHHDGSGDSLEMSSLDR	S2,S	0.902	0.203
Top1	DNA topoisomerase 1	Q04750	S396	VPSPPPGHK	S3	0.994	0.942
Top1	DNA topoisomerase 1	Q04750	Ambiguous	EKENGFSSPPR	S	1.102	0.086
Top1	DNA topoisomerase 1	Q04750	Ambiguous	ENGFSSPPR	S	1.038	0.580
Top2a	DNA topoisomerase 2-alpha	Q01320	S1211	AQMCADVLPSPR	S10	0.936	0.146
Top2a	DNA topoisomerase 2-alpha	Q01320	S1521	YLEESDDDDDLF	S5	0.782	0.264
Top2a	DNA topoisomerase 2-alpha	Q01320	S28	RLSIER	S3	1.060	0.550
Top2a	DNA topoisomerase 2-alpha	Q01320	T1245	IKSENVEGTPAEDGAEPGSLR	T9	1.160	0.031
Top2a	DNA topoisomerase 2-alpha	Q01320	Ambiguous	KPSSSDSSDSDFER	S	1.002	0.967
Top2b	DNA topoisomerase 2-beta	Q64511	S1363	YTFDFSEEEDDDAAAADDSNDLEELK	S6	1.006	0.967
Top2b	DNA topoisomerase 2-beta	Q64511	S1537	KASGSENEGDYNPGR	S3	1.106	0.100
Top2b	DNA topoisomerase 2-beta	Q64511	S1539	ASGSENEGDYNPGR	S4	1.025	0.653
Top2b	DNA topoisomerase 2-beta	Q64511	S1600	YFAESDEEEDVDFAMFN	S5	0.897	0.522
Top2b	DNA topoisomerase 2-beta	Q64511	S1537,S1539	KASGSENEGDYNPGR	S3,S5	1.040	0.238
Topors	E3 ubiquitin-protein ligase Topors	Q80Z37	S1016	EESTFASDLESQSSNVSIQAEPSRPVPSPR	S28	1.086	0.285
Topors	E3 ubiquitin-protein ligase Topors	Q80Z37	S1025	TSLSSVSPGRDCDVS	S7	1.024	0.587
Topors	E3 ubiquitin-protein ligase Topors	Q80Z37	S196	SASLYSPSSTVSR	S6	0.838	0.003
Topors	E3 ubiquitin-protein ligase Topors	Q80Z37	S99	LQQTVPADASPDSK	S10	1.036	0.408
Topors	E3 ubiquitin-protein ligase Topors	Q80Z37	T567	STSLPAPR	T2	0.877	0.255
Topors	E3 ubiquitin-protein ligase Topors	Q80Z37	S1022,T/S	TSLSSVSPGRDCDVS	S4,T/S	0.996	0.978
Tor1aip1	Torsin-1A-interacting protein 1	Q921T2	S140	LEQHSQQPQLSPATSGR	S11	1.038	0.263
Tor1aip1	Torsin-1A-interacting protein 1	Q921T2	S151	GLRDSPSSSEDREEDEPSSRPVTSQTASK	S5	1.047	0.133
Tor1aip1	Torsin-1A-interacting protein 1	Q921T2	S153	DSPSSSEDREEDEPSSRPVTSQTASK	S4	1.067	0.510
Tor1aip1	Torsin-1A-interacting protein 1	Q921T2	S60	EVRFSEEPAEVYGDFEPR	S5	1.157	0.080
Tor1aip1	Torsin-1A-interacting protein 1	Q921T2	S94	FRPASAGEEVR	S5	1.044	0.295
Tor1aip1	Torsin-1A-interacting protein 1	Q921T2	S151,S/T	GLRDSPSSSEDREEDEPSSRPVTSQTASK	S5,S/T	1.067	0.230
Tor1aip1	Torsin-1A-interacting protein 1	Q921T2	Ambiguous	LEQHSQQPQLSPATSGR	T/S	0.962	0.322
Tox2	TOX high mobility group box family member 2	E9Q0B9	S506	GSPPPTTSPK	S2	1.368	0.007
Tox4	TOX high mobility group box family member 4	Q8BU11	S178	LSTTPSPTNSLHEDGVDDFRR	S6	1.094	0.428

Tox4	TOX high mobility group box family member 4	Q8BU11	S178,S182	LSTTPSPTNSLHEDGVDDFRR	S6,S10	1.008	0.830
Tox4	TOX high mobility group box family member 4	Q8BU11	S178,T/S	LSTTPSPTNSLHEDGVDDFR	S6,T/S	0.812	0.109
Tp53bp1	TP53-binding protein 1	P70399	S1096	QSEQPVKPVGPVMDDAAPEDSASPVSQQR	S23	0.969	0.519
Tp53bp1	TP53-binding protein 1	P70399	S1096	QSEQPVKPVGPVMDDAAPEDSASPVSQQR	S23	1.006	0.969
Tp53bp1	TP53-binding protein 1	P70399	S1104	QSEQPVKPVGPVMDDAAPEDSASPVSQQRASQEQR	S31	1.128	0.539
Tp53bp1	TP53-binding protein 1	P70399	S1459	SDSPEIPFQAATGSSDGLDSSSSGNSFVGLR	S3	1.118	0.565
Tp53bp1	TP53-binding protein 1	P70399	S1459	RSDSPEIPFQAATGSSDGLDSSSSGNSFVGLR	S4	0.959	0.689
Tp53bp1	TP53-binding protein 1	P70399	S1631	SNISSPVTPTAASSSSTTPTR	S4	0.969	0.654
Tp53bp1	TP53-binding protein 1	P70399	S268	SEDRPSSPQVSVAAVETK	S7	0.974	0.363
Tp53bp1	TP53-binding protein 1	P70399	S382	STPFIVPSSPTEQGGR	S9	1.048	0.468
Tp53bp1	TP53-binding protein 1	P70399	S533	MESLGSPR	S6	1.005	0.914
Tp53bp1	TP53-binding protein 1	P70399	S552	TEEDRENTQIDDTEPLSPVSNSK	S17	1.092	0.229
Tp53bp1	TP53-binding protein 1	P70399	S763	ADVSCEPLEEVEK	S4	0.916	0.652
Tp53bp1	TP53-binding protein 1	P70399	S776	CSDSQSWEGVAPEEEPCAENR	S4	1.173	0.521
Tp53bp1	TP53-binding protein 1	P70399	S822	AETTEKDAVTEDSPQPPLPSVR	S13	1.083	0.015
Tp53bp1	TP53-binding protein 1	P70399	S822	DAVTEDSPQPPLPSVR	S7	0.951	0.607
Tp53bp1	TP53-binding protein 1	P70399	S552,S/T	MESLGSPRTEEDRENTQIDDTEPLSPVSNSK	S25,S/T	0.937	0.433
Tp53bp1	TP53-binding protein 1	P70399	Ambiguous	SEALSSVLDQEEAMDTK	S/T	1.253	0.034
Tp53bp1	TP53-binding protein 1	P70399	Ambiguous	ETVVSGPLGVEDISPSMSPDDK	S/T	1.091	0.146
Tp53bp1	TP53-binding protein 1	P70399	Ambiguous	ELLEEGPQVQPSSEPEVSSTQEDLFDQSSK	S/T	1.078	0.432
Tpd52	Tumor protein D52	F8WHQ1	S143	TSETLSQAGQK	S6	1.021	0.594
Tpd52l2	Tumor protein D54	A2AUD5	S189	NSATFKSFEDR	S7	0.949	0.288
Tpd52l2	Tumor protein D54	A2AUD5	S215	ENGSDNLPPSPGSGDQTLPDHAPF	S10	1.030	0.284
Tpd52l2	Tumor protein D54	A2AUD5	S215	VVGGRENGSDNLPPSPGSGDQTLPDHAPF	S15	0.986	0.909
Tpi1	Triosephosphate isomerase	P17751	S262	IIYGGSVTGATCK	S6	1.072	0.416
Tpm1	Tropomyosin 1, alpha, isoform CRA_j	G5E8R1	S16	SLQEQADAAEER	S1	1.089	0.344
Tpm1	Tropomyosin 1, alpha, isoform CRA_j	G5E8R1	S51	ETAEADVASLNRR	S9	0.985	0.672
Tpm3	Tropomyosin alpha-3 chain	E9Q7Q3	S51	EQAEAEVASLNR	S9	0.961	0.383
Tpm3	Tropomyosin alpha-3 chain	E9Q7Q3	S51	EQAEAEVASLNRR	S9	0.990	0.883
Tpr	Nucleoprotein TPR	F6ZDS4	S1259	DAVQAPLNVSLNEEGK	S10	1.203	0.175
Tpr	Nucleoprotein TPR	F6ZDS4	S1908	TREEEEDSTMEAGDQVSEDTVEMPLPK	S8	1.077	0.308
Tpr	Nucleoprotein TPR	F6ZDS4	S2141	QTPQAPQSPR	S8	1.028	0.697
Tpr	Nucleoprotein TPR	F6ZDS4	S2223	TDGFAEAIHSPQVAGVPR	S10	1.406	0.001
Tpr	Nucleoprotein TPR	F6ZDS4	T2205	TVPSTPTLVVPHR	T5	1.083	0.023
Tpr	Nucleoprotein TPR	F6ZDS4	T724	SSTSQTVSTPAPEPVIDSTEAIEAK	T6	1.010	0.917
Tpr	Nucleoprotein TPR	F6ZDS4	Ambiguous	RSSTSQTVSTPAPEPVIDSTEAIEAK	S/T	1.066	0.606
Tpr	Nucleoprotein TPR	F6ZDS4	Ambiguous	AADSQNSGEGNTSAAESSFSQEVAR	S/T	1.128	0.031
Tprg1l	Tumor protein p63-regulated gene 1-like protein	Q9DBS2	T13	DTVDSAGTSPTAVLAAGEDAGAGRPGAGTPLR	T8	0.936	0.620
Tprn	Taperin	A2AI08	S184	SLAPASPVR	S6	1.209	0.038
Tpx2	Targeting protein for Xklp2	A2APB8	S737	SSELPLTVPVSPK	S11	0.920	0.771
Tpx2	Targeting protein for Xklp2	A2APB8	T369	DPQTPILQTK	T4	1.041	0.730
Tra2a	Transformer-2 protein homolog alpha	E9QP00	T200	AHTPTPGIYMGRPTHSGGGGGGGGGGGGGGGGR	T3	1.016	0.894
Tra2a	Transformer-2 protein homolog alpha	E9QP00; P62996	S260,S262,S266	RRSPSPYYSR	S3,S5	1.039	0.754
Tra2a	Transformer-2 protein homolog alpha	E9QP00; P62996	S260,S262,S266	RSPSPYYSR	S2,S4	0.999	0.970
Tra2b	Transformer-2 protein homolog beta	P62996	T201	RPHTPTPGIYMGRPTYGSSR	T4	1.036	0.670
Tra2b	Transformer-2 protein homolog beta	P62996	T201	RPHTPTPGIYMGRPTYGSSR	T4	1.016	0.748
Traf1	TNF receptor-associated factor 1	P39428	Ambiguous	ADNLHPVSPGSPLTQEK	S/T	1.073	0.425
Traf3ip1	TRAF3-interacting protein 1	A0A087WQD8	S438	RQESTETLVVDR	S4	0.967	0.674
Traf7	E3 ubiquitin-protein ligase TRAF7	F8WJF7	S60	RTPSSSSTLAYSPR	S4	0.917	0.040
Traf7	E3 ubiquitin-protein ligase TRAF7	F8WJF7	S60	RTPSSSSTLAYSPRDEEDGMPPINTPR	S4	1.009	0.876
Traf7	E3 ubiquitin-protein ligase TRAF7	F8WJF7	S60,S62	RTPSSSSTLAYSPRDEEDGMPPINTPR	S4,S6	1.119	0.550
Traf7	E3 ubiquitin-protein ligase TRAF7	F8WJF7	S60,S63	RTPSSSSTLAYSPR	S4,S7	0.935	0.288
Trafd1	TRAF-type zinc finger domain-containing protein 1	Q3UDK1	S478	SDCQRSPPGVLK	S6	0.989	0.474
Trafd1	TRAF-type zinc finger domain-containing protein 1	Q3UDK1	T277	AGPTSLGDIK	T4	1.473	0.011
Trak2	Trafficking protein, kinesin-binding 2	Q6P9N8	S455	TLLSPGGSTEVPGNSQPTNPPGSPEDSDLATALHR	S4	0.804	0.536
Trak2	Trafficking protein, kinesin-binding 2	Q6P9N8	S729	RDSTITFSSTR	S3	1.033	0.663
Tram1	Translocating chain-associated membrane protein 1	Q91V04	S365	GTENGVNGTVTSNGADSPR	S17	1.028	0.633
Tram1	Translocating chain-associated membrane protein 1	Q91V04	S365	KGTENGVNGTVTSNGADSPR	S18	0.997	0.978
Trappc1	Trafficking protein particle complex subunit 1	Q5NCF2	S132	SRLDSYVR	S5	0.973	0.896
Trappc10	Trafficking protein particle complex subunit 10	Q3TLI0	S685	SPSDNSLNTTGIICR	S3	1.088	0.186
Trappc10	Trafficking protein particle complex subunit 10	Q3TLI0	S708	RQESGSSLEPPSGLALEDGAHVLR	S4	1.065	0.198
Trappc12	Trafficking protein particle complex subunit 12	Q8K2L8	S10	DGEQSPSEASPSAQAGPENIPEPMSR	S5	0.948	0.549
Trappc12	Trafficking protein particle complex subunit 12	Q8K2L8	S10	DGEQSPSEASPSAQAGPENIPEPMSR	S5	1.066	0.555
Trappc12	Trafficking protein particle complex subunit 12	Q8K2L8	T130	LPSSTDKEVVDTLSNGSETDGDDTPR	T19	0.802	0.265
Trappc12	Trafficking protein particle complex subunit 12	Q8K2L8	T143	DISDMTPDSR	T6	0.909	0.226
Trappc12	Trafficking protein particle complex subunit 12	Q8K2L8	S309,S/T/Y	LSSLTAPVGEKSPDSTSPSYSTR	S12,S/T/Y	1.094	0.452
Trappc12	Trafficking protein particle complex subunit 12	Q8K2L8	Ambiguous	SPSFSSTSETSAK	S/T	0.947	0.435
Trappc12	Trafficking protein particle complex subunit 12	Q8K2L8	Ambiguous	SPDSTSPSYSTR	T/S/Y	1.066	0.493
Trappc12	Trafficking protein particle complex subunit 12	Q8K2L8	Ambiguous	GATDHRLPSSTDKEVVDTLSNGSETDGDDTPR	T/S	1.035	0.808
Trappc4	Trafficking protein particle complex subunit 4	Q9ES56	S219	AGTFGPGS	S8	1.073	0.705
Trappc8	Trafficking protein particle complex 8	E9PWG2	S1090	SNSLEDEEGR	S3	1.034	0.565
Trappc8	Trafficking protein particle complex 8	E9PWG2	S259	NSIQNQESYEDGPCTMTSSK	S8	1.208	0.167
Trappc9	Trafficking protein particle complex subunit 9	Q3U0M1	S834	VESRPTNPSEGSK	S3	1.113	0.017
Trem2	Triggering receptor expressed on myeloid cells 2	Q99NH8	T227	GLDCGQDAGHQLQILTGPGGT	T21	1.468	0.050
Trerf1	Transcriptional-regulating factor 1	Q8BXJ2	T773	SSSIDGSNVTVTPGPGEQTVDVEPR	T12	0.930	0.550
Trerf1	Transcriptional-regulating factor 1	Q8BXJ2	S764,T773	SSSIDGSNVTVTPGPGEQTVDVEPR	S3,T12	1.221	0.051
Trex1	Three-prime repair exonuclease 1	Q91XB0	S167	ALEQASSPSGNGSR	S7	1.021	0.636
Trex1	Three-prime repair exonuclease 1	Q91XB0	S178	SYSLGSIYTR	S3	1.078	0.606
Trex1	Three-prime repair exonuclease 1	Q91XB0	S78	ACSPGASEITGLSK	S3	0.989	0.808
Trex1	Three-prime repair exonuclease 1	Q91XB0	Ambiguous	ALEQASSPSGNGSRK	S	1.117	0.096
Trim25	E3 ubiquitin/ISG15 ligase TRIM25	Q61510	S424	ATSPDAAPK	S3	1.000	0.997
Trim28	Transcription intermediary factor 1-beta	Q62318	S473	SGEGEVSGLLR	S1	1.179	0.064
Trim28	Transcription intermediary factor 1-beta	Q62318	S473	SRSGEGEVSGLLR	S3	1.128	0.037
Trim28	Transcription intermediary factor 1-beta	Q62318	Ambiguous	RPAASSAAAASAAASSPAGGGGEAQELLEHCGVCR	S	1.058	0.158
Trim28	Transcription intermediary factor 1-beta	Q62318	Ambiguous	LSPPYSSPQEFAQDVGR	S/Y	0.842	0.140
Trim28	Transcription intermediary factor 1-beta	Q62318	Ambiguous	QGSGSSQPMEVQEGYGFGSDDPYSSAEPHVSGMK	S/Y	0.818	0.492
Trim3	Tripartite motif-containing protein 3	Q9R1R2	S359	TGSAELCAEITGPDGVR	S3	0.842	0.340
Trim3	Tripartite motif-containing protein 3	Q9R1R2	S454	RPSSMYSTGGK	S3	0.968	0.418
Trim3	Tripartite motif-containing protein 3	Q9R1R2	S7	EDSPGPEVQPMDK	S3	0.996	0.955
Trim3	Tripartite motif-containing protein 3	Q9R1R2	S7	REDSPGPEVQPMDK	S4	1.006	0.890
Trim3	Tripartite motif-containing protein 3	Q9R1R2	S7	REDSPGPEVQPMDK	S4	0.994	0.909
Trim32	E3 ubiquitin-protein ligase TRIM32	Q8CH72	S337	EMDMSPEEVAPSPR	S12	0.995	0.857
Trim32	E3 ubiquitin-protein ligase TRIM32	Q8CH72	S337,S341	EMDMSPEEVAPSPRASPAK	S12,S16	1.079	0.277
Trim35	Tripartite motif-containing 35	A0A0R4J031	S23	AATPVVVTAAAPAMEPGPSVSPGPSR	S21	0.629	0.012
Trim35	Tripartite motif-containing 35	A0A0R4J031	S23	AATPVVVTAAAPAMEPGPSVSPGPSR	S21	1.204	0.226
Trim36	E3 ubiquitin-protein ligase Trim36	Q80WG7	S105	RNSLTPRPTTFPCPGCEHDVDLGER	S3	0.949	0.056
Trim37	E3 ubiquitin-protein ligase TRIM37	Q6PCX9	S454	DLSPPDNHLSPQNDDSPETR	S3	1.037	0.614
Trim37	E3 ubiquitin-protein ligase TRIM37	Q6PCX9	S477	AGSCSDMLLEGGPTCASVR	S3	0.922	0.410
Trim47	E3 ubiquitin-protein ligase TRIM47	Q8C0E3	S393	GLGSNEDGLQK	S4	0.931	0.066
Trim47	E3 ubiquitin-protein ligase TRIM47	Q8C0E3	S464	VLCPINYPESPTR	S10	0.781	0.091
Trim47	E3 ubiquitin-protein ligase TRIM47	Q8C0E3	S591	SGALASPTDPFQSR	S6	1.022	0.604
Trim47	E3 ubiquitin-protein ligase TRIM47	Q8C0E3	S591	RSGALASPTDPFQSR	S7	0.957	0.641
Trim47	E3 ubiquitin-protein ligase TRIM47	Q8C0E3	T100	GATPEPSAPSAPPPAPEPSAPCAPECEQWPAGEEPVR	T3	1.745	0.001
Trim47	E3 ubiquitin-protein ligase TRIM47	Q8C0E3	Ambiguous	LGSEDVESQDPDSTSLLESEAPR	S/T	0.983	0.940
Trim56	E3 ubiquitin-protein ligase TRIM56	A0A0R4J0Q6	T413	QGGAQPLTPK	T8	1.045	0.391
Trio	Triple functional domain protein	Q0KL02	S1809	KSADAGSQKDSDDSAATPQDETIEER	S2	1.023	0.463
Trio	Triple functional domain protein	Q0KL02	S2481	EAFPPSSPLQK	S7	1.147	0.004
Trio	Triple functional domain protein	Q0KL02	Ambiguous	DGEDLDSQGDGSSQPDTISIASR	S/T	1.415	0.239
Trio	Triple functional domain protein	Q0KL02	Ambiguous	SADAGSQKDSDDSAATPQDETIEER	S/T	1.054	0.588
Triobp	TRIO and F-actin-binding protein	Q99KW3	S1604	KADGPRPSLDYVELSPLAPSSPQR	S21	0.963	0.686
Trip10	Cdc42-interacting protein 4	Q8CJ53	S296	VPSDSSLGTPDGRPELR	S3	0.918	0.276
Trip10	Cdc42-interacting protein 4	Q8CJ53	S482	VLSNRGDSLSR	S3	1.035	0.460
Trip10	Cdc42-interacting protein 4	Q8CJ53	S296,S/T	VPSDSSLGTPDGRPELR	S3,S/T	1.077	0.628
Trip11	Thyroid hormone receptor interactor 11	E9Q512	S635	SELTQSQTQGSSR	S11	1.089	0.437
Trip11	Thyroid hormone receptor interactor 11	E9Q512	S983	QDSQTENSDIFQETK	S3	0.968	0.747
Trip11	Thyroid hormone receptor interactor 11	E9Q512	S983	AQLHEERQDSQTENSDIFQETK	S10	1.076	0.256
Trip12	E3 ubiquitin-protein ligase TRIP12	G5E870	S1024	DDSLDLSPQGR	S7	1.074	0.281
Trip12	E3 ubiquitin-protein ligase TRIP12	G5E870	S1069	SPTTTQSPK	S7	1.082	0.296
Trip12	E3 ubiquitin-protein ligase TRIP12	G5E870	S1146	YFSSENMDGSNPALNVLQR	S10	0.944	0.521
Trip12	E3 ubiquitin-protein ligase TRIP12	G5E870	S1409	QFSVQAEDERESTDDESNPLGR	S12	1.049	0.192
Trip12	E3 ubiquitin-protein ligase TRIP12	G5E870	S1610	LLDTNPEINQSDSQDSR	S13	0.962	0.646
Trip12	E3 ubiquitin-protein ligase TRIP12	G5E870	S312	SESPPAELPSLRR	S3	0.995	0.945
Trip12	E3 ubiquitin-protein ligase TRIP12	G5E870	S312	RSESPPAELPSLRR	S4	0.976	0.728
Trip12	E3 ubiquitin-protein ligase TRIP12	G5E870	S312	RSESPPAELPSLR	S4	1.046	0.290
Trip12	E3 ubiquitin-protein ligase TRIP12	G5E870	S312	SESPPAELPSLR	S3	1.087	0.162
Trip12	E3 ubiquitin-protein ligase TRIP12	G5E870	S398	TDEAPQGAAASSSVAGAVGMTTSGESESDDSEMGR	S26	1.297	0.034
Trip12	E3 ubiquitin-protein ligase TRIP12	G5E870	S77	SASPDYNR	S3	1.130	0.215
Trip12	E3 ubiquitin-protein ligase TRIP12	G5E870	S85	TNSPSSAK	S3	1.197	0.056
Trip12	E3 ubiquitin-protein ligase TRIP12	G5E870	S1409,T1410	QFSVQAEDERESTDDESNPLGR	S12,T13	1.061	0.639
Trip12	E3 ubiquitin-protein ligase TRIP12	G5E870	S77,S85	SASPDYNRTNSPSSAK	S3,S11	1.049	0.215
Trmt1	tRNA (guanine(26)-N(2))-dimethyltransferase	A0A0R4IZW7	S121	IAVDLSDQEEETAGK	S6	0.949	0.664
Trmt10a	tRNA methyltransferase 10 homolog A	A0A0R4J205	S24	LGTSDGEEERQEPR	S4	0.986	0.691
Trmt10a	tRNA methyltransferase 10 homolog A	A0A0R4J205	S315	DNPVSPQKDEQGQQSSPVLQ	S5	1.054	0.411
Trmt10a	tRNA methyltransferase 10 homolog A	A0A0R4J205	S315,S	DNPVSPQKDEQGQQSSPVLQ	S5,S	1.003	0.955
Trmt10c	tRNA methyltransferase 10 homolog C	Q3UFY8	S85	SSIQEDGVSEVSDKDEDSLASTR	S18	0.975	0.687
Trmt5	tRNA (guanine(37)-N1)-methyltransferase	Q9D0C4	T487	QKTGDPFSGEPQIASDS	T3	1.071	0.312
Trmt5	tRNA (guanine(37)-N1)-methyltransferase	Q9D0C4	Ambiguous	TGDPFSGEPQIASDS	S/T	0.851	0.515
Trp53bp2	Transformation-related protein 53-binding protein 2	E9QJU8	S334	ENLPVSPDGNLPQQAVSAPSR	S6	1.184	0.149

Trp53bp2	Transformation-related protein 53-binding protein 2	E9QJU8	S485	NQSSEDILR	S3	1.028	0.525
Trp53bp2	Transformation-related protein 53-binding protein 2	E9QJU8	Ambiguous	RSSITEPEGPNGPNIQK	S/T	0.982	0.295
Trp53bp2	Transformation-related protein 53-binding protein 2	E9QJU8	Ambiguous	SSITEPEGPNGPNIQK	S/T	1.009	0.818
Trpc4	Short transient receptor potential channel 4	Q9QUQ5	Y271,S276,S282	ELEIILNYRDDNSLIEEQSGNDLARLK	Y8,S13,S19	1.250	0.067
Trpm7	Transient receptor potential cation channel subfamily M member 7	Q923J1	S1403	FSVSTPSQPSCK	S4	1.028	0.167
Trpm7	Transient receptor potential cation channel subfamily M member 7	Q923J1	S1502	RASTEDSPEVDSK	S3	1.038	0.846
Trpm7	Transient receptor potential cation channel subfamily M member 7	Q923J1	T1466	ELLSNDTPENTLK	T7	0.835	0.074
Trpm7	Transient receptor potential cation channel subfamily M member 7	Q923J1	T555	NTSSSTPQLR	T6	1.021	0.817
Trpm7	Transient receptor potential cation channel subfamily M member 7	Q923J1	S1491,T/S	TSTSLHSVQAESCSR	S7,T/S	0.861	0.046
Trps1	Zinc finger transcription factor Trps1	V9GX74	S982	GSGEEQVNGSPLER	S10	1.071	0.615
Trps1	Zinc finger transcription factor Trps1	V9GX74	S1085,S1089	HPNYSPPGSPIEK	S5,S9	0.997	0.950
Trps1	Zinc finger transcription factor Trps1	V9GX74	Ambiguous	ATEETGPVQSGQANCQGLSPVSVASK	S/T	1.071	0.334
Trps1	Zinc finger transcription factor Trps1	V9GX74	Ambiguous	SPQESTGDPGNSSSVSDGKGSSERGSPIEK	S/T	0.927	0.503
Trps1	Zinc finger transcription factor Trps1	V9GX74	Ambiguous	SPQESTGDPGNSSSVSDGKGSSERGSPIEK	S/T	0.866	0.043
Trpv2	Transient receptor potential cation channel subfamily V member 2	Q9WTR1	S15	LETSDGDEEGSAEVNKGK	S4	1.046	0.390
Trpv2	Transient receptor potential cation channel subfamily V member 2	Q9WTR1	S15	LETSDGDEEGSAEVNK	S4	1.037	0.466
Trpv2	Transient receptor potential cation channel subfamily V member 2	Q9WTR1	S37	GKNEPPPMESPFQGEDR	S10	1.132	0.163
Trpv2	Transient receptor potential cation channel subfamily V member 2	Q9WTR1	S37	NEPPPMESPFQGEDR	S8	1.055	0.263
Trpv2	Transient receptor potential cation channel subfamily V member 2	Q9WTR1	S37	NEPPPMESPFQGEDR	S8	0.926	0.450
Trpv2	Transient receptor potential cation channel subfamily V member 2	Q9WTR1	S743	NSASEEDHLPLQVLQSH	S4	1.088	0.479
Trrap	Transformation/transcription domain-associated protein	A0A1D5RLL4	S2050	RGLSVDSAQEVK	S4	1.360	0.011
Tsc1	Hamartin	Q9EP53	S295	SADVTTSPYVDTQNSYGGSTSTPSSSSR	S7	0.999	0.992
Tsc1	Hamartin	Q9EP53	S502	GGFDSPFYR	S5	1.023	0.856
Tsc1	Hamartin	Q9EP53	T550	QAFTPIDPPSGSADVSPAGDRDR	T4	1.007	0.930
Tsc1	Hamartin	Q9EP53	Ambiguous	VTGGSSSSSSELSTPEKPPSQR	S/T	1.017	0.898
Tsc2	Tuberin	Q61037	S1132	SMSGGHGLR	S3	1.028	0.777
Tsc2	Tuberin	Q61037	S1421	SQSGILDGEAATWSATGEESR	S3	1.000	1.000
Tsc2	Tuberin	Q61037	S671	ASGPLSPPTGPPSPVPMGPAVR	S13	0.966	0.589
Tsc2	Tuberin	Q61037	S939	STSLNERPK	T/S	0.874	0.458
Tsc2	Tuberin	Q61037	T1465	GYTISDSAPSR	T3	0.960	0.627
Tsc2	Tuberin	Q61037	T1465	GYTISDSAPSRR	T3	0.939	0.337
Tsc2	Tuberin	Q61037	Y1744	SNPTDIYPSK	Y7	0.919	0.340
Tsc2	Tuberin	Q61037	S664,S671	ASGPLSPPTGPPSPVPMGPAVR	S6,S13	0.800	0.156
Tsc2	Tuberin	Q61037	Ambiguous	ITVPPEGPLPSSSPR	S/T	0.972	0.788
Tsc22d3	TSC22 domain family protein 3	Q9Z2S7	T125	LSPEEPAPEAPETPETPEAPGGSAV	T13	1.050	0.598
Tsc22d4	TSC22 domain family protein 4	Q9EQN3	S386	LGPSAPNGPSI	S10	1.038	0.777
Tsc22d4	TSC22 domain family protein 4	Q9EQN3	S62	NGSPPPGAPASR	S3	0.991	0.931
Tsc22d4	TSC22 domain family protein 4	Q9EQN3	T119	GASGGTGGRSLDSR	T6	0.989	0.878
Tsc22d4	TSC22 domain family protein 4	Q9EQN3	T223	VEVESGGSAAATPPLSR	T12	0.968	0.240
Tsc22d4	TSC22 domain family protein 4	Q9EQN3	T183,S189	VETPPLSASPPQQRPPGPGTGDSAQTLPSLR	T3,S9	1.046	0.421
Tsg101	Tumor susceptibility gene 101 protein	Q61187	T221	DGTISEDTIR	T3	0.940	0.569
Tsnax	Translin-associated protein X	Q9QZE7	S288	TDMIDQEESIS	S9	0.949	0.415
Tsnax	Translin-associated protein X	Q9QZE7	T165	ESKTPPAEGQEK	T4	1.067	0.294
Tsnax	Translin-associated protein X	Q9QZE7	Ambiguous	ITSAPDMEEILTESESK	T/S	1.130	0.580
Tspyl1	Testis-specific Y-encoded-like protein 1	O88852	S51	AGIVGSPAPR	S6	1.129	0.101
Tspyl1	Testis-specific Y-encoded-like protein 1	O88852	S74	QGESPPSVR	S4	1.036	0.488
Tspyl1	Testis-specific Y-encoded-like protein 1	O88852	Ambiguous	GHCDADTVSGTPDRRPLLGEEK	S/T	1.102	0.214
Tsr1	Pre-rRNA-processing protein TSR1 homolog	Q5SWD9	S794	SDISSTVSEVDME	S4	1.084	0.364
Tsr2	Pre-rRNA-processing protein TSR2 homolog	H7BWY8	S143	ETDVAEDDVDSVEEMEVK	S11	0.986	0.786
Tsr2	Pre-rRNA-processing protein TSR2 homolog	H7BWY8	S143	ETDVAEDDVDSVEEMEVK	S11	0.912	0.518
Tssc4	Protein TSSC4	Q9JHE7	S316	DGNPGGPGSERGPSV	S14	1.032	0.511
Tssc4	Protein TSSC4	Q9JHE7	T124	RPVTPPSQTPAR	T4	1.005	0.956
Ttc1	Tetratricopeptide repeat protein 1	Q91Z38	Ambiguous	ASDDSSSELDEEYLIELEK	S/Y	0.939	0.474
Ttc14	MCG123425, isoform CRA_b	G3X9T5	S666	YSTSPASSDYSWK	S4	1.111	0.202
Ttc28	Tetratricopeptide repeat protein 28	A0A0A0MQN9	S2299	DTPVSPADPPLFR	S5	1.236	0.111
Ttc28	Tetratricopeptide repeat protein 28	A0A0A0MQN9	S2327	NTSPACSAPSPALSYSSAGSAR	S3	1.083	0.246
Ttc7a	Tetratricopeptide repeat protein 7A	Q8BGB2	S183	LPNSVASHIR	S4	1.022	0.859
Ttc7a	Tetratricopeptide repeat protein 7A	Q8BGB2	S648	DGSFEGLTVK	S3	0.983	0.822
Ttc7a	Tetratricopeptide repeat protein 7A	Q8BGB2	S679	ASSIAASR	S3	1.021	0.650
Ttc7b	Tetratricopeptide repeat protein 7B	A0A1Y7VL44	S625,S630	SCYNLTNPSDSGRGSSLLDR	S11,S16	0.939	0.366
Ttf1	Transcription termination factor 1	Q62187	S137	ESQTPAQENSESEQPR	S10	1.064	0.788
Ttf1	Transcription termination factor 1	Q62187	S327	SQELEPIPDSLDDSETISER	S14	0.924	0.479
Ttf1	Transcription termination factor 1	Q62187	S437	LEPTHEEESNSESASNSAAR	S9	1.162	0.080
Ttf1	Transcription termination factor 1	Q62187	S845	DIFHCDDDSDEGSPEEPSASDVQ	S9	1.049	0.362
Ttf1	Transcription termination factor 1	Q62187	T374	SVALATSSDSASVTDSK	T6	1.093	0.528
Ttf2	Transcription termination factor 2	Q5NC05	S119	ELSVTSKPQQPSESQLHSPSQPR	S18	0.977	0.868
Tti2	TELO2-interacting protein 2	Q8BGV4	S504	KVQQGSADSPGDDTEGD	S9	0.908	0.255
Ttyh3	Protein tweety homolog 3	Q6P5F7	S496	CENTPLIGRESPPPSYTSSMR	S11	1.000	1.000
Ttyh3	Protein tweety homolog 3	Q6P5F7	S496	CENTPLIGRESPPPSYTSSMR	S11	1.030	0.598
Tuba1b	Tubulin alpha-1B chain	P05213	S439	DYEEVGVDSVEGEGEEEGEEY	S9	0.576	0.008
Tuba1b	Tubulin alpha-1B chain	P05213	Ambiguous	EDMAALEKDYEEVGVDSVEGEGEEEGEEY	S/Y	0.989	0.870
Tuba1b	Tubulin alpha-1B chain	P05213; P68373	Ambiguous	TIGGGDDSFNTFFSETGAGK	T/S	0.820	0.114
Tuba1c	Tubulin alpha-1C chain	P68373	S439	EDMAALEKDYEEVGADSAEGDDEGEEY	S17	1.056	0.445
Tuba1c	Tubulin alpha-1C chain	P68373	S439	DYEEVGADSAEGDDEGEEY	S9	0.937	0.656
Tuba1c	Tubulin alpha-1C chain	P68373	Ambiguous	EDMAALEKDYEEVGADSAEGDDEGEEY	S/Y	1.092	0.684
Tubb5	Tubulin beta-5 chain	P99024	T55	ISVYYNEATGGK	T9	1.077	0.412
Tubgcp6	Gamma-tubulin complex component 6	G5E8P0	S1218	IGENVSDTDLDLQQR	S6	1.006	0.965
Tulp4	Tubby-related protein 4	Q9JIL5	S1347	LDSRAEEGSVQAITEGK	S3	1.296	0.002
Tulp4	Tubby-related protein 4	Q9JIL5	S1444	TASELEEFK	S3	1.376	0.000
Tulp4	Tubby-related protein 4	Q9JIL5	S565	AAQEISRSPR	S8	1.089	0.268
Tut4	Terminal uridylyltransferase 4	A2A8R7	S131	SPNLPVK	S1	0.945	0.331
Tut7	Terminal uridylyltransferase 7	Q5BLK4	S172	DLSSLEAMSEMEAGSPENK	S15	0.863	0.178
Tut7	Terminal uridylyltransferase 7	Q5BLK4	S193	TEDSEQDGDLDGPVIDESVLSTK	S4	0.918	0.433
Tut7	Terminal uridylyltransferase 7	Q5BLK4	S193	TRTEDSEQDGDLDGPVIDESVLSTK	S6	1.034	0.704
Tut7	Terminal uridylyltransferase 7	Q5BLK4	S705	NTTEEVGSPAKEK	S8	0.978	0.784
Twf1	Twinfilin-1	Q91YR1	S142	YLLSQSSPAPLTAAEEELR	S6	1.077	0.448
Twf1	Twinfilin-1	Q91YR1	T349	GPAEAEATTD	T9	1.015	0.811
Twf1	Twinfilin-1	Q91YR1	Ambiguous	LIRGPAEAEATTD	T	0.832	0.051
Twistnb	DNA-directed RNA polymerase I subunit RPA43	Q78WZ7	S306	HQEDQDPIFQASDSSGYQSDHNK	S19	1.026	0.773
Twistnb	DNA-directed RNA polymerase I subunit RPA43	Q78WZ7	S318	KHSEEANFESPK	S3	1.072	0.131
Twistnb	DNA-directed RNA polymerase I subunit RPA43	Q78WZ7	S325	HSEEANFESPK	S9	0.947	0.666
Twistnb	DNA-directed RNA polymerase I subunit RPA43	Q78WZ7	S60	HIALSPR	S5	1.078	0.719
Txlna	Alpha-taxilin	Q6PAM1	Ambiguous	RPEATTASKEQGVESPGAQPASSPR	S/T	0.986	0.873
Txlna	Alpha-taxilin	Q6PAM1	Ambiguous	EQGVESPGAQPASSPR	S	1.067	0.104
Txndc11	Thioredoxin domain-containing protein 11	Q8K2W3	S15	GGGGGNNSEDAEDEGGGPK	S8	1.023	0.834
Txndc16	Thioredoxin domain-containing protein 16	Q7TN22	S777	EAEVQESAELQPGDR	S7	0.812	0.343
Txnl1	Thioredoxin-like protein 1	Q8CDN6	S113	QHLENDPGSNEDADIPK	S9	1.033	0.051
Txnrd3	Thioredoxin reductase 3	Q99MD6	S16	AQTSPGLGK	S4	0.992	0.822
Txnrd3	Thioredoxin reductase 3	Q99MD6	S50	LASPGTSRPSSEAR	S3	1.200	0.129
Tyk2	Tyrosine-protein kinase	E9QJS1	Ambiguous	HQLPEPSSPELATLTR	S/T	0.900	0.484
Tyrobp	TYRO protein tyrosine kinase-binding protein (Fragment)	A0A140LHP7	T115	QHIAETESPYQELQGQRPEVYSDLNTQR	T6	0.984	0.788
Tyrobp	TYRO protein tyrosine kinase-binding protein (Fragment)	A0A140LHP7	Ambiguous	KQHIAETESPYQELQGQRPEVYSDLNTQR	T/S/Y	0.953	0.695
U2af1	Splicing factor U2AF 35 kDa subunit	Q9D883	Ambiguous	NPQNSSQSADGLR	S	1.005	0.796
U2af2	Splicing factor U2AF 65 kDa subunit	P26369	S79	GAKEEHGGLIRSPR	S12	0.977	0.623
U2af2	Splicing factor U2AF 65 kDa subunit	P26369	S79	EEHGGLIRSPR	S9	1.017	0.740
U2surp	U2 snRNP-associated SURP motif-containing protein	Q6NV83	S238	FEPPQSDSDSGQRR	S8	1.058	0.152
U2surp	U2 snRNP-associated SURP motif-containing protein	Q6NV83	T919	EKDECTPTRK	T6	0.931	0.473
Uap1l1	UDP-N-acetylhexosamine pyrophosphorylase-like protein 1	Q3TW96	S490	QLQSPFILDEDQAR	S4	1.023	0.816
Uba1	Ubiquitin-like modifier-activating enzyme 1	Q02053	S46	NGSEADIDESLYSR	S3	1.009	0.935
Uba1	Ubiquitin-like modifier-activating enzyme 1	Q02053	S810	IHVSDQELQSANASVDDSR	S4	1.130	0.290
Ubac1	Ubiquitin-associated domain-containing protein 1	Q8VDI7	S98	RVPSPLPK	S4	0.955	0.268
Ubac2	Ubiquitin-associated domain-containing protein 2	Q8R1K1	S301	SEQRASPPLEVSEEQVAR	S6	1.074	0.161
Ubap1	Ubiquitin-associated protein 1	Q8BH48	S146	VLSPPHTK	S3	1.077	0.025
Ubap2	Ubiquitin-associated protein 2	Q91VX2	S634	IAYQSSASPPDSAPGSVANGHGGGR	S8	0.932	0.774
Ubap2l	Ubiquitin-associated protein 2-like	Q80X50	S436	SANDSTVHSPFTKR	S9	0.936	0.392
Ubap2l	Ubiquitin-associated protein 2-like	Q80X50	S436	SANDSTVHSPFTK	S9	1.021	0.896
Ubap2l	Ubiquitin-associated protein 2-like	Q80X50	S487	STSAPQMSPGSSDNQSSSPQPAQQK	S8	1.020	0.463
Ubap2l	Ubiquitin-associated protein 2-like	Q80X50	S487	STSAPQMSPGSSDNQSSSPQPAQQK	S8	0.992	0.737
Ubap2l	Ubiquitin-associated protein 2-like	Q80X50	S624	RYPSSISSSPQK	S4	1.097	0.022
Ubap2l	Ubiquitin-associated protein 2-like	Q80X50	S628	RYPSSISSSPQKDLTQAK	S8	0.981	0.774
Ubap2l	Ubiquitin-associated protein 2-like	Q80X50	S629	YPSSISSSPQK	S8	1.105	0.098
Ubap2l	Ubiquitin-associated protein 2-like	Q80X50	Y878	DGSLASNPYSGDLTK	Y9	0.831	0.341
Ubap2l	Ubiquitin-associated protein 2-like	Q80X50	S487,S497	STSAPQMSPGSSDNQSSSPQPAQQK	S8,S18	1.083	0.016
Ubap2l	Ubiquitin-associated protein 2-like	Q80X50	S496,S497	STSAPQMSPGSSDNQSSSPQPAQQK	S17,S18	0.975	0.617
Ubap2l	Ubiquitin-associated protein 2-like	Q80X50	S627,S/Y/T	RYPSSISSSPQKDLTQAK	S7,S/Y/T	0.937	0.371
Ubash3b	Ubiquitin-associated and SH3 domain-containing protein B	Q8BGG7	S366	VNSQPGPQK	S3	1.383	0.006
Ube2e1	Ubiquitin-conjugating enzyme E2 E1	P52482	Ambiguous	ASTSSSSSSSSNQQTEKEGSTPK	T/S	1.004	0.979
Ube2j1	Ubiquitin-conjugating enzyme E2 J1	Q9JJZ4	S266	RPSTSPDVLQGQPPR	S3	1.092	0.056
Ube3a	Ubiquitin-protein ligase E3A	O08759	S8	SPGESQSEDIEASR	S1	1.082	0.365
Ube3a	Ubiquitin-protein ligase E3A	O08759	Ambiguous	MGDSSQGDNNVQK	S	1.032	0.599
Ube4b	Ubiquitin conjugation factor E4 B	Q9ES00	S101	SQSSEGVSSLSSSPSNSLETQSQSLSR	S26	0.993	0.894
Ube4b	Ubiquitin conjugation factor E4 B	Q9ES00	S105	SQSMDIDGVSCEK	S3	1.263	0.003
Ube4b	Ubiquitin conjugation factor E4 B	Q9ES00	S105	SQSMDIDGVSCEK	S3	1.304	0.004
Ube4b	Ubiquitin conjugation factor E4 B	Q9ES00	S124	SMSQVDVDSGIENMEVDENDRR	S9	0.912	0.203
Ube4b	Ubiquitin conjugation factor E4 B	Q9ES00	S31	LAGGQTSQPTTPLTSPQR	S15	1.142	0.057
Ube4b	Ubiquitin conjugation factor E4 B	Q9ES00	T234	DENPFASLTATSQPIATAARSPDR	T17	1.422	0.153
Ube4b	Ubiquitin conjugation factor E4 B	Q9ES00	S86,S/T	SQSSEGVSSLSSSPSNSLETQSQSLSR	S11,S/T	1.016	0.951
Ube4b	Ubiquitin conjugation factor E4 B	Q9ES00	S31,T/S	LAGGQTSQPTTPLTSPQR	S15,T/S	1.029	0.812

Ubl7	Ubiquitin-like protein 7	Q91W67	S230	DMPGGFLFDGLSDDEDDFHPSTR	S12	1.017	0.951
Ubn1	Ubinuclein-1	Q4G0F8	S175	QASESEDDFIK	S5	1.041	0.657
Ubn1	Ubinuclein-1	Q4G0F8	S493	ICSDEEEDEEKGGRR	S3	0.963	0.791
Ubn1	Ubinuclein-1	Q4G0F8	S493	ICSDEEEDEEKGGR	S3	0.998	0.993
Ubn1	Ubinuclein-1	Q4G0F8	S173,S175	QASESEDDFIKEK	S3,S5	1.122	0.114
Ubn2	Ubinuclein-2	Q80WC1	T238	QASDTEEDDFTDNQK	T5	1.086	0.346
Ubp1	Upstream-binding protein 1	Q811S7	S54	QEDSSLSLEDEAK	S7	1.009	0.954
Ubqln2	Ubiquilin-2	Q9QZM0	S25	GPAAAPGAASPPAEPK	S10	0.992	0.867
Ubr2	E3 ubiquitin-protein ligase UBR2	Q6WKZ8	Ambiguous	ECSSSSPVAEAEGTIMEESSR	S/T	0.998	0.987
Ubr2	E3 ubiquitin-protein ligase UBR2	Q6WKZ8	Ambiguous	IRECSSSSPVAEAEGTIMEESSR	S/T	0.892	0.179
Ubr4	E3 ubiquitin-protein ligase UBR4	A2AN08	S178	TLSDVEDQKELASPVSPELR	S13	0.997	0.969
Ubr4	E3 ubiquitin-protein ligase UBR4	A2AN08	S181	ELASPVSPELR	S7	1.097	0.237
Ubr4	E3 ubiquitin-protein ligase UBR4	A2AN08	S2882	TSPADHGGSVGSESGGSAVDSVAGEHSVSGR	S2	1.047	0.411
Ubr4	E3 ubiquitin-protein ligase UBR4	A2AN08	S362	TGSTSSKEDDYESDAATIVQK	S3	1.120	0.240
Ubr4	E3 ubiquitin-protein ligase UBR4	A2AN08	T2721	SNTPMGDKDDDDDDDADEKMQSSGIPDGGHIR	T3	0.892	0.015
Ubr4	E3 ubiquitin-protein ligase UBR4	A2AN08	T2721	SNTPMGDKDDDDDDDADEKMQSSGIPDGGHIR	T3	0.898	0.083
Ubr4	E3 ubiquitin-protein ligase UBR4	A2AN08	T2721	SNTPMGDKDDDDDDDADEKMQSSGIPDGGHIR	T3	0.850	0.239
Ubr4	E3 ubiquitin-protein ligase UBR4	A2AN08	T2721	SNTPMGDKDDDDDDDADEK	T3	1.074	0.597
Ubr4	E3 ubiquitin-protein ligase UBR4	A2AN08	T2721	SNTPMGDKDDDDDDDADEK	T3	1.006	0.942
Ubr4	E3 ubiquitin-protein ligase UBR4	A2AN08	T2712,S	HVTLPSSPR	T3,S	0.996	0.960
Ubr4	E3 ubiquitin-protein ligase UBR4	A2AN08	Ambiguous	VNEAAPEKPQEDSGTAGGISSTSASVNR	S/T	0.972	0.848
Ubr4	E3 ubiquitin-protein ligase UBR4	A2AN08	Ambiguous	HVTLPSSPR	S/T	0.993	0.819
Ubr4	E3 ubiquitin-protein ligase UBR4	A2AN08	Ambiguous	MQSSGIPDGGHIR	S	1.174	0.415
Ubr4	E3 ubiquitin-protein ligase UBR4	A2AN08	Ambiguous	AAPPPPPPPPPLESSPR	S	1.010	0.764
Ubr5	E3 ubiquitin-protein ligase UBR5	A0A2I3BQS6	S1549	RISQSQPVR	S3	1.047	0.487
Ubr5	E3 ubiquitin-protein ligase UBR5	A0A2I3BQS6	S2241	DLSLEVDRDR	S3	0.931	0.352
Ubtf	Nucleolar transcription factor 1	P25976	S389	QTTSPASK	S4	1.033	0.764
Ubtf	Nucleolar transcription factor 1	P25976	S433	QLQEERPELSESELTR	S10	0.917	0.067
Ubxn1	UBX domain-containing protein 1	Q922Y1	S188	SSPPATDPGPVPSSPSQEPPTKR	S2	1.037	0.259
Ubxn1	UBX domain-containing protein 1	Q922Y1	S188,S200	YGGSVGSRSSPPATDPGPVPSSPSQEPPTKR	S10,S22	0.981	0.893
Ubxn1	UBX domain-containing protein 1	Q922Y1	T192,S200	SSPPATDPGPVPSSPSQEPPTKR	T6,S14	1.019	0.332
Ubxn1	UBX domain-containing protein 1	Q922Y1	Ambiguous	YGGSVGSRSSPPATDPGPVPSSPSQEPPTK	S/T/Y	0.956	0.646
Ubxn1	UBX domain-containing protein 1	Q922Y1	Ambiguous	SSPPATDPGPVPSSPSQEPPTK	S/T	1.032	0.108
Ubxn1	UBX domain-containing protein 1	Q922Y1	Ambiguous	YGGSVGSRSSPPATDPGPVPSSPSQEPPTKR	S/Y/T	0.913	0.591
Ubxn7	UBX domain-containing protein 7	G5E8R8	S280	SESLIDASEDSQLEAAIR	S3	1.138	0.516
Uchl3	Ubiquitin carboxyl-terminal hydrolase (Fragment)	A0A2I3BQ39	S101	FLEESVSMSPEER	S9	0.859	0.144
Uckl1	Uridine-cytidine kinase-like 1	Q91YL3	S539	YFGTDAVPDGSDDDEAATVG	S11	1.069	0.026
Uckl1	Uridine-cytidine kinase-like 1	Q91YL3	S56	LLPPVGTGRSPR	S10	0.981	0.829
Uckl1	Uridine-cytidine kinase-like 1	Q91YL3	Ambiguous	TTSQCKSEPPLLR	T/S	1.092	0.141
Uevld	Ubiquitin-conjugating enzyme E2 variant 3	Q3U1V6	S365	DGVLSPSSQAQLSSR	S5	0.966	0.661
Ufd1	Ubiquitin recognition factor in ER-associated degradation protein 1	P70362	S299	FIAFSGEGQSLR	S10	0.698	0.059
Ufl1	E3 UFM1-protein ligase 1	Q8CCJ3	S458	KDEDSDDESQSSHGGK	S5	0.906	0.606
Ufl1	E3 UFM1-protein ligase 1	Q8CCJ3	Ambiguous	TGQGEDPSSDELDKEQHDVTNATRK	S/T	0.911	0.370
Ufl1	E3 UFM1-protein ligase 1	Q8CCJ3	Ambiguous	TGQGEDPSSDELDKEQHDVTNATR	S/T	0.995	0.846
Ugdh	UDP-glucose 6-dehydrogenase	O70475	T474	RIPYTPGEIPK	T5	1.220	0.016
Uhrf1	E3 ubiquitin-protein ligase UHRF1	Q8VDF2	S161	ALSEDEPCSSSAVK	S3	1.027	0.409
Uhrf1	E3 ubiquitin-protein ligase UHRF1	Q8VDF2	S656	STGPTLSSPR	S8	1.079	0.011
Uhrf1	E3 ubiquitin-protein ligase UHRF1	Q8VDF2	S91	ERDSELSDSDSGYGVGHSESDK	S7	0.956	0.268
Uhrf1bp1	UHRF1 (ICBP90)-binding protein 1	B2KF50	S1098	TVSQQSFDGVSLDSGGPDDR	S6	1.101	0.129
Uhrf1bp1	UHRF1 (ICBP90)-binding protein 1	B2KF50	S914	SDASSDQGAVSPEK	S5	1.021	0.592
Uhrf1bp1	UHRF1 (ICBP90)-binding protein 1	B2KF50	S929	DGSGENLAASQER	S3	1.030	0.852
Uhrf1bp1	UHRF1 (ICBP90)-binding protein 1	B2KF50	S936	DGSGENLAASQERLPSDGELPDPGPCAQQPVGK	S10	1.048	0.492
Uhrf1bp1	UHRF1 (ICBP90)-binding protein 1	B2KF50	S936,S942	DGSGENLAASQERLPSDGELPDPGPCAQQPVGK	S10,S16	0.964	0.468
Uhrf1bp1	UHRF1 (ICBP90)-binding protein 1	B2KF50	Ambiguous	QSASFGSPVHSEALAPESVSHQR	S	1.168	0.221
Uhrf1bp1l	UHRF1-binding protein 1-like	A2RSJ4	S1008	DVASTDSDSVLNYR	S9	0.948	0.374
Uhrf1bp1l	UHRF1-binding protein 1-like	A2RSJ4	S1020	DGSTRLSLDDDGNHNPPSNPVTGK	S7	1.081	0.387
Uhrf1bp1l	UHRF1-binding protein 1-like	A2RSJ4	S1092	CPPSPAPLSVSYK	S4	0.972	0.598
Uhrf1bp1l	UHRF1-binding protein 1-like	A2RSJ4	S1092	ERCPPSPAPLSVSYK	S6	0.879	0.133
Uhrf1bp1l	UHRF1-binding protein 1-like	A2RSJ4	S952	SASDTNLQK	S3	0.909	0.112
Uhrf1bp1l	UHRF1-binding protein 1-like	A2RSJ4	S988	SGSGEMTSEGSHTK	S3	1.019	0.705
Uhrf1bp1l	UHRF1-binding protein 1-like	A2RSJ4	S414,S418	NLGSPPKSPTHASPQHTQTEK	S4,S8	0.977	0.820
Uhrf1bp1l	UHRF1-binding protein 1-like	A2RSJ4	Ambiguous	EYYSTESEPLTNGGQRPSSDTFLR	S/T/Y	1.033	0.413
Uhrf2	E3 ubiquitin-protein ligase UHRF2	Q7TMI3	S668	RPASDDECPGDSK	S4	0.979	0.822
Uhrf2	E3 ubiquitin-protein ligase UHRF2	Q7TMI3	S684	ASDSTDAVEAFQLTPQQQR	S4	1.134	0.016
Uhrf2	E3 ubiquitin-protein ligase UHRF2	Q7TMI3	Ambiguous	QGSEATKRPASDDECPGDSK	T/S	1.039	0.549
Uimc1	BRCA1-A complex subunit RAP80	A0A0R4J073	S665	MPSPEVEEASCSR	S3	1.132	0.201
Uimc1	BRCA1-A complex subunit RAP80	A0A0R4J073	S665	MPSPEVEEASCSR	S3	1.041	0.483
Uimc1	BRCA1-A complex subunit RAP80	A0A0R4J073	Ambiguous	GQSSQGLFVEETSEEGLK	S/T	1.046	0.521
Ulk1	Serine/threonine-protein kinase	Q6PB82	S225	APFQASSPQDLR	S7	1.031	0.791
Ulk1	Serine/threonine-protein kinase	Q6PB82	S403	TPSPSPTCSSSPSPSGRPGPFSSNR	S3	0.918	0.266
Ulk1	Serine/threonine-protein kinase	Q6PB82	S450	IEQNLQSPTQQQTAR	S7	0.988	0.754
Ulk1	Serine/threonine-protein kinase	Q6PB82	S527	VPSPQGADVR	S3	1.039	0.616
Ulk1	Serine/threonine-protein kinase	Q6PB82	S763	GGGASSPAPVVFTVGSPPSGATPPQSTR	S16	1.013	0.837
Ulk1	Serine/threonine-protein kinase	Q6PB82	T468	SGGTSPLGFGR	T4	0.944	0.056
Ulk1	Serine/threonine-protein kinase	Q6PB82	S403,S405	TPSPSPTCSSSPSPSGRPGPFSSNR	S3,S5	0.949	0.376
Ulk1	Serine/threonine-protein kinase	Q6PB82	S753,S763	GGGASSPAPVVFTVGSPPSGATPPQSTR	S6,S16	0.929	0.558
Ulk1	Serine/threonine-protein kinase	Q6PB82	S766,T769,S/T	GGGASSPAPVVFTVGSPPSGATPPQSTR	S19,T22,S/T	1.607	0.001
Ulk2	Serine/threonine-protein kinase ULK2	Q9QY01	S453	VGSCSPVPGDTVQTGGR	S5	1.095	0.397
Ulk2	Serine/threonine-protein kinase ULK2	Q9QY01	S772	VCVGSPPGPGLGSSPPGAEGAPSLR	S5	0.882	0.563
Ulk2	Serine/threonine-protein kinase ULK2	Q9QY01	S772,S781	VCVGSPPGPGLGSSPPGAEGAPSLR	S5,S14	1.180	0.295
Ulk2	Serine/threonine-protein kinase ULK2	Q9QY01	Ambiguous	SCPVPVPVYSGPVPGSSCSSSPSCR	S/Y	0.782	0.208
Ulk3	Serine/threonine-protein kinase ULK3	Q3U3Q1	S464	ESHWEAESLDKEGLSESVR	S17	0.909	0.148
Unc119	Protein unc-119 homolog A	B1AQD9	S12	KGGGGTGSGAEPVPGASNR	S8	1.164	0.300
Unc119	Protein unc-119 homolog A	B1AQD9	S37	SAEPTREPGAEAESGSESEPEPGPGPR	S14	0.982	0.793
Unc119	Protein unc-119 homolog A	B1AQD9	S37,S39	SAEPTREPGAEAESGSESEPEPGPGPR	S14,S16	0.988	0.808
Unc13d	Protein unc-13 homolog D	B2RUP2	S149	VGVAEGSPVSR	S7	1.037	0.078
Unc13d	Protein unc-13 homolog D	B2RUP2	S149	VGVAEGSPVSRR	S7	1.024	0.753
Unk	RING finger protein unkempt homolog	Q8BL48	S378,S	NSGLGSPSHLCSSPPGPSR	S6,S	0.935	0.078
Unk	RING finger protein unkempt homolog	Q8BL48	Ambiguous	NSGLGSPSHLCSSPPGPSR	S	0.982	0.849
Upf1	Regulator of nonsense transcripts 1	Q9EPU0	S1102	SQIDVALSQDSTYQGER	S8	0.919	0.022
Upf1	Regulator of nonsense transcripts 1	Q9EPU0	S1122	AYQHGGVTGLSQY	S11	0.991	0.853
Upf1	Regulator of nonsense transcripts 1	Q9EPU0	Ambiguous	EAIIPGSVYDR	S/Y	0.908	0.420
Upf3b	UPF3 regulator of nonsense transcripts homolog B (yeast)	Q3ULL6	T169	FLESYATDNEK	T7	1.150	0.154
Uprt	Uracil phosphoribosyltransferase homolog	B1AVZ0	S25	QVNSTSSPSPEHLLAEDR	S9	0.979	0.542
Uprt	Uracil phosphoribosyltransferase homolog	B1AVZ0	Ambiguous	QVNSTSSPSPEHLLAEDRVLDHAEENNAAMAK	S/T	0.837	0.191
Uri1	Unconventional prefoldin RPB5 interactor (Fragment)	A0A0U1RNX4	Ambiguous	STSSEEAVATEAGGSSLDELQENHPK	T/S	1.149	0.124
Usf2	Upstream stimulatory factor 2	Q64705	S222	THPYSPK	S5	0.950	0.668
Uso1	General vesicular transport factor p115	Q9Z1Z0	S940	DLGHPVEEEDESGDQEDDDDEIDDGDKDQDI	S12	0.951	0.386
Uso1	General vesicular transport factor p115	Q9Z1Z0	S940	LKDLGHPVEEEDESGDQEDDDDEIDDGDKDQDI	S14	0.978	0.677
Usp1	Ubiquitin carboxyl-terminal hydrolase 1	Q8BJQ2	S67	GSEIDQVVPAAQSSPVSCEKR	S14	0.996	0.950
Usp1	Ubiquitin carboxyl-terminal hydrolase 1	Q8BJQ2	S67	GSEIDQVVPAAQSSPVSCEK	S14	1.151	0.357
Usp10	Ubiquitin carboxyl-terminal hydrolase 10	P52479	S359	CSPPVPSPLASEK	S2	1.020	0.785
Usp10	Ubiquitin carboxyl-terminal hydrolase 10	P52479	S541	LLSPTHEK	S3	1.079	0.377
Usp10	Ubiquitin carboxyl-terminal hydrolase 10	P52479	S570	SDLIEDEELEDTGKGSEDEWEQVGPK	S16	1.046	0.687
Usp10	Ubiquitin carboxyl-terminal hydrolase 10	P52479	S359,S364	CSPPVPSPLASEK	S2,S7	1.050	0.784
Usp11	Ubiquitin carboxyl-terminal hydrolase 11	Q99K46	S906	RQSQTSSSDTPASPVSSSTPNSDIMDIN	S13	0.864	0.502
Usp14	Ubiquitin carboxyl-terminal hydrolase 14	Q9JMA1	S143	ASGEMASAQYITAALR	S2	0.940	0.662
Usp14	Ubiquitin carboxyl-terminal hydrolase 14	Q9JMA1	T235	LEAIEDDSGRETDSSSAPAVTPSK	T21	1.130	0.241
Usp14	Ubiquitin carboxyl-terminal hydrolase 14	Q9JMA1	Ambiguous	CTESEEEEVTK	S/T	0.805	0.167
Usp15	Ubiquitin carboxyl-terminal hydrolase 15	Q8R5H1	S229	SPGASNFSTLPK	S1	1.007	0.937
Usp15	Ubiquitin carboxyl-terminal hydrolase 15	Q8R5H1	Ambiguous	GASAATGIPLESDEDSNDNDNDLENENCMHTN	S/T	0.956	0.681
Usp15	Ubiquitin carboxyl-terminal hydrolase 15	Q8R5H1	Ambiguous	GASAATGIPLESDEDSNDNDNDLENENCMHTN	S/T	1.000	0.994
Usp16	Ubiquitin carboxyl-terminal hydrolase 16	Q99LG0	S531	TPDVQESPEDLGVK	S7	0.952	0.332
Usp19	Ubiquitin carboxyl-terminal hydrolase 19	Q3UJD6	S220	RGPEGEGSMDGPGPQGDAPSFLSDSATQVEAEEK	S8	1.245	0.393
Usp20	Ubiquitin carboxyl-terminal hydrolase 20	Q8C6M1	S132	AVPIAVADEGESESEDDDLKPR	S12	0.928	0.600
Usp20	Ubiquitin carboxyl-terminal hydrolase 20	Q8C6M1	S342	TNSEQVDEDADVDTAMASLDEQSR	S3	0.949	0.735
Usp20	Ubiquitin carboxyl-terminal hydrolase 20	Q8C6M1	S369	EAQPPSPR	S6	0.999	0.990
Usp20	Ubiquitin carboxyl-terminal hydrolase 20	Q8C6M1	Ambiguous	STSPCQTPEPDNEAHIR	S/T	0.899	0.396
Usp24	Ubiquitin carboxyl-terminal hydrolase 24	E9PV45	S1283	QMSLCGTPEK	S3	1.382	0.000
Usp24	Ubiquitin carboxyl-terminal hydrolase 24	E9PV45	S1852	GEVLEGSNAYYCEK	S7	1.027	0.760
Usp24	Ubiquitin carboxyl-terminal hydrolase 24	E9PV45	S2045	VSDQNSPVLPK	S6	0.960	0.226
Usp24	Ubiquitin carboxyl-terminal hydrolase 24	E9PV45	S2075	QEAEDLSLSAPSSPEISPQSSPRPHRPNNDR	S17	1.152	0.146
Usp24	Ubiquitin carboxyl-terminal hydrolase 24	E9PV45	S2602	HLQQGSESPMMIGELR	S8	1.124	0.057
Usp24	Ubiquitin carboxyl-terminal hydrolase 24	E9PV45	Ambiguous	QEAEDLSLSAPSSPEISPQSSPRPHRPNNDR	S	1.302	0.071
Usp28	Ubiquitin carboxyl-terminal hydrolase 28	Q5I043	S1027	EPPTIRPNSPYDLCNR	S9	0.985	0.820
Usp31	Ubiquitin-specific peptidase 31	E9Q6Y8	S1315	SSQLDSGAPLSPSSR	S11	1.033	0.579
Usp31	Ubiquitin-specific peptidase 31	E9Q6Y8	S49	AGGGGGAGGPGPAAPSSPSSAR	S17	0.966	0.309
Usp31	Ubiquitin-specific peptidase 31	E9Q6Y8	Ambiguous	SQESMSSPSPQK	S	1.099	0.394
Usp32	Ubiquitin-specific peptidase 32	F8VPZ3	Ambiguous	MADTSSMDEDFESDYKK	S/T/Y	1.036	0.469
Usp33	Ubiquitin carboxyl-terminal hydrolase 33	Q8R5K2	Ambiguous	AGSCGEAYAPQGWIAFFMEYVK	Y/S	0.932	0.611
Usp34	Ubiquitin carboxyl-terminal hydrolase 34 (Fragment)	F6WJB7	S3415	RVSSDEEHTVDSCIGDIK	S3	1.075	0.395
Usp34	Ubiquitin carboxyl-terminal hydrolase 34 (Fragment)	F6WJB7	S3443	EVLTPTSTSDNETR	S9	1.170	0.170
Usp34	Ubiquitin carboxyl-terminal hydrolase 34 (Fragment)	F6WJB7	S3463	DSSIIDPGTEQDLPSPENSSVK	S15	1.003	0.945
Usp34	Ubiquitin carboxyl-terminal hydrolase 34 (Fragment)	F6WJB7	S3463,S3468	DSSIIDPGTEQDLPSPENSSVKEYR	S15,S20	0.835	0.159
Usp34	Ubiquitin carboxyl-terminal hydrolase 34 (Fragment)	F6WJB7	Ambiguous	HLTLSQTDMAALTGGK	T/S	0.791	0.044
Usp35	Ubiquitin-specific peptidase 35	M0QWN7	S929	EDPAAEPGSPR	S9	1.016	0.856
Usp36	Ubiquitin carboxyl-terminal hydrolase 36	B1AQJ2	S918	SEGLSQEATPSQDLIQHSCSPVDHSEPEAR	S20	1.054	0.620
Usp37	Ubiquitin carboxyl-terminal hydrolase 37	A0A0R4J2D0	S650	SSVTSCLDSDSEDELKR	S9	0.921	0.059

Usp38	Ubiquitin carboxyl-terminal hydrolase 38	Q8BW70	S575	VAVPTESPGTGDSEK	S7	0.978	0.874
Usp39	U4/U6.U5 tri-snRNP-associated protein 2	Q3TIX9	S46	EKEPEAASSRGSPVR	S12	1.067	0.534
Usp39	U4/U6.U5 tri-snRNP-associated protein 2	Q3TIX9	S81	EADEDSEPEREVR	S6	1.036	0.308
Usp39	U4/U6.U5 tri-snRNP-associated protein 2	Q3TIX9	S81	EREADEDSEPEREVR	S8	0.975	0.763
Usp40	Ubiquitin carboxyl-terminal hydrolase 40	Q8BWR4	Ambiguous	AQSSDLFSNAGVPAR	S	0.914	0.424
Usp42	Ubiquitin carboxyl-terminal hydrolase 42	B2RQC2	S936	AQEPSPAKEK	S5	1.087	0.294
Usp47	Ubiquitin carboxyl-terminal hydrolase 47	A0A1L1SV73	S911	STETSDFENIESPLNER	S12	1.131	0.010
Usp47	Ubiquitin carboxyl-terminal hydrolase 47	A0A1L1SV73	S934	ELEQHIQTSDPENFQSEER	S9	1.004	0.942
Usp47	Ubiquitin carboxyl-terminal hydrolase 47	A0A1L1SV73	Ambiguous	ETWDTAEEDSGTDSEYDESGK	S/T/Y	1.192	0.347
Usp5	Ubiquitin carboxyl-terminal hydrolase 5	P56399	S783	SAAESISESVPVGPK	S5	1.033	0.660
Usp5	Ubiquitin carboxyl-terminal hydrolase 5	P56399	T623	GTGLQPGEEELPDIAPPLVTPDEPK	T20	1.455	0.026
Usp53	Inactive ubiquitin carboxyl-terminal hydrolase 53	P15975	S490	SGSPPASDGFR	S3	1.136	0.105
Usp54	Inactive ubiquitin carboxyl-terminal hydrolase 54	Q8BL06	Ambiguous	NSSSPVSLDAAPPDSVNVYR	S/Y	1.029	0.867
Usp54	Inactive ubiquitin carboxyl-terminal hydrolase 54	Q8BL06	Ambiguous	SSSPSDFMPPLSQGPGR	S	0.881	0.367
Usp6nl	USP6 N-terminal-like protein	Q80XC3	S631	AAYPPSYSNPPVYHGNSPK	S17	0.972	0.174
Usp6nl	USP6 N-terminal-like protein	Q80XC3	S669	RPYGSSLSVDTSPEK	S12	1.231	0.139
Usp6nl	USP6 N-terminal-like protein	Q80XC3	S704	GYGSSGSPK	S7	1.052	0.525
Usp6nl	USP6 N-terminal-like protein	Q80XC3	S669,S/T/Y	RPYGSSLSVDTSPEK	S12,S/T/Y	1.036	0.737
Usp7	Ubiquitin carboxyl-terminal hydrolase 7	F8VPX1	S19	AGEQQLSEPEDMEMEAGDTDDPPR	S7	0.966	0.668
Usp7	Ubiquitin carboxyl-terminal hydrolase 7	F8VPX1	S19	AGEQQLSEPEDMEMEAGDTDDPPR	S7	0.985	0.745
Usp7	Ubiquitin carboxyl-terminal hydrolase 7	F8VPX1	S19	AGEQQLSEPEDMEMEAGDTDDPPR	S7	1.025	0.559
Usp8	Ubiquitin carboxyl-terminal hydrolase 8	A2AI52	S403	AEASPIIQPAPATK	S4	1.070	0.299
Usp8	Ubiquitin carboxyl-terminal hydrolase 8	A2AI52	S546	TEDRELSADGAQEATGTQR	S7	1.060	0.758
Usp8	Ubiquitin carboxyl-terminal hydrolase 8	A2AI52	S691	RSYSSPDITQALQEEEKR	S4	1.014	0.868
Usp8	Ubiquitin carboxyl-terminal hydrolase 8	A2AI52	S691	SYSSPDITQALQEEEK	S3	0.994	0.874
Usp8	Ubiquitin carboxyl-terminal hydrolase 8	A2AI52	S691	SYSSPDITQALQEEEKR	S3	0.949	0.265
Usp8	Ubiquitin carboxyl-terminal hydrolase 8	A2AI52	Ambiguous	RSYSSPDITQALQEEEK	S/Y/T	0.922	0.253
Usp9x	Probable ubiquitin carboxyl-terminal hydrolase FAF-X	P70398	S1600	NGILAIEGTGSDVDDDMSGDEK	S18	0.925	0.467
Usp9x	Probable ubiquitin carboxyl-terminal hydrolase FAF-X	P70398	S1600	NGILAIEGTGSDVDDDMSGDEKQDNESNVDPR	S18	1.047	0.610
Usp9x	Probable ubiquitin carboxyl-terminal hydrolase FAF-X	P70398	S2547	AQENYEGGEEVSPPQTK	S12	1.172	0.335
Usp9x	Probable ubiquitin carboxyl-terminal hydrolase FAF-X	P70398	S374	HGSSEDEEWLTAER	S3	1.058	0.484
Usp9x	Probable ubiquitin carboxyl-terminal hydrolase FAF-X	P70398	Ambiguous	NGILAIEGTGSDVDDDMSGDEKQDNESNVDPR	T/S	1.243	0.364
Utp11	Probable U3 small nucleolar RNA-associated protein 11	Q9CZJ1	S241	ETVNSPAIYR	S5	1.076	0.171
Utp15	U3 small nucleolar RNA-associated protein 15 homolog	Q8C7V3	S528	TESPTQPSDTDKNS	S14	1.068	0.469
Utp15	U3 small nucleolar RNA-associated protein 15 homolog	Q8C7V3	Ambiguous	NDSDPVPEHVPAELPEEKTESPTQPSDTDKNS	T/S	0.900	0.317
Utp18	U3 small nucleolar RNA-associated protein 18 homolog	Q5SSI6	S114,S115	GQLHGSSDESEVENEAK	S6,S7	0.986	0.700
Utp18	U3 small nucleolar RNA-associated protein 18 homolog	Q5SSI6	S206,T/S	RTSSDDESEEDEDDLLQR	S8,T/S	1.151	0.209
Utp20	Small subunit processome component 20 homolog	E9QK83	S1748	EALGAPEAAASEGTVAK	S11	1.086	0.482
Utp3	Something about silencing protein 10	Q9JI13	T356	QAAAVALTDEPDFDGAALK	T8	1.023	0.756
Utrn	Utrophin	E9Q6R7	Ambiguous	SQPPTSPEGR	T/S	1.081	0.477
Uvssa	UV-stimulated scaffold protein A	Q9D479	S291	DSSRDEDEPSDPDDFLR	S10	0.872	0.010
Uxs1	UDP-glucuronic acid decarboxylase 1	Q91XL3	S42	SIQENGELK	S1	1.152	0.113
Vac14	Protein VAC14 homolog	Q80WQ2	S517	GLEGSPSTPTMNSYFYK	S5	0.935	0.720
Vamp1	Vesicle-associated membrane protein 1	D3YTU0	S63	DQKLSELDDR	S5	1.125	0.083
Vamp4	Vesicle-associated membrane protein 4	O70480	S17	HLNDDDVTGSVK	S10	0.988	0.845
Vamp4	Vesicle-associated membrane protein 4	O70480	S30	NLLEDDSDEEEDFFLR	S7	1.057	0.380
Vamp4	Vesicle-associated membrane protein 4	O70480	S30	RNLLEDDSDEEEDFFLR	S8	1.442	0.046
Vamp4	Vesicle-associated membrane protein 4	O70480	S88	GERLDELQDKSESLSDNATAFSNR	S11	0.925	0.081
Vamp8	Vesicle-associated membrane protein 8	A0A0R4J0R1	S18	NLQSEVEGVK	S4	1.207	0.289
Vamp8	Vesicle-associated membrane protein 8	A0A0R4J0R1	T54	NKTEDLEATSEHFK	T9	1.068	0.476
Vapb	Vesicle-associated membrane protein, associated protein B and C	Q8BH80	T158	SLTSPLDDTEVK	T3	1.100	0.206
Vars	Valine--tRNA ligase (Fragment)	G3UY93	S526	VQGSDSDEEVVVATTR	S6	0.983	0.781
Vasp	Vasodilator-stimulated phosphoprotein	P70460	S317	MKSSSSVTTSEAHPSTPCSSDDSDLER	S3	1.114	0.512
Vasp	Vasodilator-stimulated phosphoprotein	P70460	Ambiguous	ATQVGEKPPKDESASEESEAR	S/T	0.969	0.723
Vasp	Vasodilator-stimulated phosphoprotein	P70460	Ambiguous	SSSSVTTSEAHPSTPCSSDDSDLER	S/T	0.963	0.806
Vav2	Guanine nucleotide exchange factor VAV2	Q60992	Ambiguous	MSSPADVDAPGAGPGPK	S	1.039	0.756
Vav3	Guanine nucleotide exchange factor VAV3	Q9R0C8	S786	TGNSLLSPK	S7	0.934	0.685
Vcam1	Vascular cell adhesion protein 1	P29533	Ambiguous	QEFSSEEMTK	S/T	0.974	0.821
Vcl	Vinculin	Q64727	S272	ALASIDSK	S4	1.002	0.977
Vcl	Vinculin	Q64727	S290	DPNASPGDAGEQAIR	S5	1.003	0.938
Vcl	Vinculin	Q64727	S346	GQGASPVAMQK	S5	1.037	0.018
Vcl	Vinculin	Q64727	Y692	NPGNQAAYEHFETMK	Y8	1.069	0.036
Vcl	Vinculin	Q64727	Y822	SFLDSGYR	Y7	0.997	0.958
Vcp	Transitional endoplasmic reticulum ATPase	Q01853	S775	FPSGNQGGAGPSQGSGGGTGGSVYTEDNDDDLYG	S3	1.037	0.780
Vcpip1	Deubiquitinating protein VCIP135	A0A0R4J0M9	S1196	AQKENSMEEPEEMDSQDAETTNTTEPMDHS	S6	1.307	0.017
Vcpip1	Deubiquitinating protein VCIP135	A0A0R4J0M9	S746	TVSPSTIR	S3	0.970	0.458
Vcpip1	Deubiquitinating protein VCIP135	A0A0R4J0M9	S767	APYSPTTSKEK	S4	0.909	0.233
Vcpip1	Deubiquitinating protein VCIP135	A0A0R4J0M9	S767	APYSPTTSK	S4	1.119	0.472
Vcpip1	Deubiquitinating protein VCIP135	A0A0R4J0M9	S767	DGPSSAPATPTKAPYSPTTSK	S16	0.959	0.151
Vcpip1	Deubiquitinating protein VCIP135	A0A0R4J0M9	S767,S/T/Y	DGPSSAPATPTKAPYSPTTSK	S16,S/T/Y	1.138	0.026
Vcpip1	Deubiquitinating protein VCIP135	A0A0R4J0M9	S993,S	SRESSPSHGLLK	S1,S	0.969	0.432
Vcpip1	Deubiquitinating protein VCIP135	A0A0R4J0M9	T760,S767,S/T/Y	DGPSSAPATPTKAPYSPTTSK	T9,S16,S/T/Y	0.972	0.747
Vdac1	Voltage-dependent anion-selective channel protein 1	Q60932	S70	VNGSLETK	S4	1.126	0.271
Vdac2	Voltage-dependent anion-selective channel protein 2	Q60930	T69	VSGTLETK	T4	1.001	0.993
Vill	Villin-like protein	G5E8C6	S766	GSQDSPENELGLDLR	S5	0.951	0.867
Vim	Vimentin	P20152	S201	EEAESTLQSFR	S5	0.917	0.283
Vim	Vimentin	P20152	S214	QDVDNASLAR	S7	1.071	0.123
Vim	Vimentin	P20152	S214	QDVDNASLARLDLER	S7	0.792	0.250
Vim	Vimentin	P20152	S299	FADLSEAANR	S5	1.151	0.008
Vim	Vimentin	P20152	S325	QVQSLTCEVDALK	S4	1.000	1.000
Vim	Vimentin	P20152	S39	TYSLGSALRPSTSR	S3	1.081	0.201
Vim	Vimentin	P20152	S430	ETNLESLPLVDTHSK	S6	1.556	0.000
Vim	Vimentin	P20152	S459	DGQVINETSQHHDDLE	S9	0.999	0.971
Vim	Vimentin	P20152	S66	SLYSSSPGGAYVTRSSAVR	S16	0.801	0.320
Vim	Vimentin	P20152	S7	SVSSSSYR	S3	1.143	0.147
Vim	Vimentin	P20152	S73	SSVPGVR	S2	0.938	0.417
Vim	Vimentin	P20152	T361	EMEENFALEAANYQDTIGR	T16	1.169	0.434
Vim	Vimentin	P20152	Y276	QQYESVAAK	Y3	0.966	0.210
Vim	Vimentin	P20152	Y61	SLYSSSPGGAYVTR	Y11	1.001	0.986
Vim	Vimentin	P20152	S51,S54	SLYSSSPGGAYVTR	S1,S4	0.982	0.923
Vim	Vimentin	P20152	Ambiguous	SYVTTSTR	S/T/Y	0.890	0.096
Vim	Vimentin	P20152	Ambiguous	MFGGSGTSSRPSSNR	S/T	1.012	0.727
Vim	Vimentin	P20152	Ambiguous	MFGGSGTSSRPSSNR	S/T	0.991	0.880
Vim	Vimentin	P20152	Ambiguous	LRSSVPGVR	S	0.983	0.620
Virma	Protein virilizer homolog	E9PZY8	S1628	SFLSEPSSPGR	S8	1.022	0.453
Virma	Protein virilizer homolog	E9PZY8	S173	HADGEKEDQFNGSPPRPQPR	S13	1.014	0.832
Virma	Protein virilizer homolog	E9PZY8	S584	TSAVPNHSEPDQDTDAVLER	S8	1.095	0.429
Vps11	Vacuolar protein sorting-associated protein 11 homolog	Q91W86	S924	LTPSLEAGLQR	S4	0.963	0.686
Vps13b	Vacuolar protein sorting-associated protein 13B	Q80TY5	S1772	YSGAQDSGIGSDSVK	S11	1.031	0.556
Vps13b	Vacuolar protein sorting-associated protein 13B	Q80TY5	S2258	SEMPVPESAPQMPSPVEK	S14	0.980	0.320
Vps13b	Vacuolar protein sorting-associated protein 13B	Q80TY5	Ambiguous	IVQIEQYSGASQHR	S/Y	1.132	0.371
Vps13c	Vacuolar protein sorting-associated protein 13C	Q8BX70	S1956	LSFHNDSFR	S2	0.996	0.967
Vps13c	Vacuolar protein sorting-associated protein 13C	Q8BX70	S736	TANASLEEIIDK	S5	0.583	0.091
Vps13c	Vacuolar protein sorting-associated protein 13C	Q8BX70	T1397	EVSTPQDVHTTQGVPAAR	T4	1.042	0.314
Vps13d	Vacuolar protein sorting 13D	B1ART2	S2435	NASSESAVVPK	S3	1.025	0.386
Vps13d	Vacuolar protein sorting 13D	B1ART2	S2454	RSSLPVSNER	S2	1.094	0.390
Vps13d	Vacuolar protein sorting 13D	B1ART2	T1762,S1766	SYPQTPPPSPSVDEPNMLVGK	T5,S9	0.853	0.061
Vps13d	Vacuolar protein sorting 13D	B1ART2	Ambiguous	EYLSQSCPSVSSVEYPDMPR	S/Y	1.157	0.489
Vps26a	Vacuolar protein sorting-associated protein 26A	P40336	S315	FESPDSQASAEQPEM	S3	1.045	0.412
Vps26a	Vacuolar protein sorting-associated protein 26A	P40336	S315	TNFHQRFESPDSQASAEQPEM	S9	0.929	0.548
Vps26a	Vacuolar protein sorting-associated protein 26A	P40336	S315	TNFHQRFESPDSQASAEQPEM	S9	0.997	0.973
Vps26a	Vacuolar protein sorting-associated protein 26A	P40336	S321	FESPDSQASAEQPEM	S9	1.083	0.044
Vps26b	Vacuolar protein sorting-associated protein 26B	Q8C0E2	S319	FEGTTSLGEVR	S6	1.040	0.343
Vps26b	Vacuolar protein sorting-associated protein 26B	Q8C0E2	S334	TPGQLSDNNSRQ	S10	0.961	0.180
Vps26b	Vacuolar protein sorting-associated protein 26B	Q8C0E2	Ambiguous	SMSHQAAIASQR	S	0.884	0.311
Vps35	Vacuolar protein sorting-associated protein 35	Q9EQH3	S783	RESPESEGPIYEGLIL	S3	0.945	0.506
Vps35	Vacuolar protein sorting-associated protein 35	Q9EQH3	S783	SRRESPESEGPIYEGLIL	S5	0.852	0.327
Vps35	Vacuolar protein sorting-associated protein 35	Q9EQH3	Y507	SDDPDQQYLILNTAR	Y8	0.993	0.941
Vps37c	Vacuolar protein sorting-associated protein 37C	Q8R105	S29	LALESPEVQDLQLER	S5	0.955	0.822
Vps4a	Vacuolar protein sorting-associated protein 4A	Q8VEJ9	S97	GSDSDSEGDNPEKK	S4	1.091	0.320
Vps4a	Vacuolar protein sorting-associated protein 4A	Q8VEJ9	S97	ENQSEGKGSDSDSEGDNPEKK	S11	0.951	0.760
Vps4a	Vacuolar protein sorting-associated protein 4A	Q8VEJ9	S99,S	ENQSEGKGSDSDSEGDNPEKK	S13,S	1.059	0.611
Vps4a	Vacuolar protein sorting-associated protein 4A	Q8VEJ9	Ambiguous	ENQSEGKGSDSDSEGDNPEKK	S	0.940	0.362
Vps4b	Vacuolar protein sorting-associated protein 4B	P46467	S102	EEQSGPVDEKGNDSDGEAESDDPEKK	S14	1.056	0.613
Vps4b	Vacuolar protein sorting-associated protein 4B	P46467	S102	EEQSGPVDEKGNDSDGEAESDDPEKK	S14	1.019	0.808
Vps4b	Vacuolar protein sorting-associated protein 4B	P46467	S102	GNDSDGEAESDDPEKK	S4	0.974	0.741
Vps4b	Vacuolar protein sorting-associated protein 4B	P46467	S102	GNDSDGEAESDDPEK	S4	1.014	0.874
Vps50	Syndetin	Q8CI71	S494	SPSVSPSK	S1	1.071	0.581
Vps50	Syndetin	Q8CI71	S494	FLEQSRSPSVSPSK	S7	1.006	0.812
Vps50	Syndetin	Q8CI71	S559	SAYQDYDSDSDVPEELKR	S8	1.041	0.178
Vps50	Syndetin	Q8CI71	S559,S561	SAYQDYDSDSDVPEELKR	S8,S10	0.959	0.514
Vps50	Syndetin	Q8CI71	Ambiguous	SAYQDYDSDSDVPEELK	S/Y	1.054	0.776
Vps51	Vacuolar protein sorting-associated protein 51 homolog	Q3UVL4	S652	TFSVYSSSR	S6	1.001	0.985
Vps54	Vacuolar protein sorting-associated protein 54	Q5SPW0	Ambiguous	NTSPHSEPCSSDSVSEPECTTDSSSSK	T/S	1.207	0.336
Vps9d1	VPS9 domain-containing protein 1	Q3U280	Ambiguous	SSQSLYCMLSPSEPSAAPR	S/Y	0.879	0.446
Vps9d1	VPS9 domain-containing protein 1	Q3U280	Ambiguous	SSQSLYCMLSPSEPSAAPR	S/Y	1.232	0.573
Vrk2	Serine/threonine-protein kinase VRK2	Q8BN21	S344	TPAQQKVDSPK	S9	0.941	0.600
Vrk2	Serine/threonine-protein kinase VRK2	Q8BN21	T449	YRHTPTGNLGVTDLESSPR	T4	1.014	0.751

Vrk2	Serine/threonine-protein kinase VRK2	Q8BN21	T451	HTPTGNLGVTDLESSPR	T4	0.949	0.650
Vrk3	Inactive serine/threonine-protein kinase VRK3	Q8K3G5	S59	DLNSSFETSPK	S9	0.872	0.026
Vsig8	V-set and immunoglobulin domain-containing protein 8	Q6P3A4	S386	TASASCEAGPSPVYIK	S11	0.998	0.988
Vsir	RIKEN cDNA 4632428N05, isoform CRA_b	A0A171EBK7	S232	MDSSNTQGIENPGFETTPPFQGMPEAK	S3	1.085	0.407
Vsir	RIKEN cDNA 4632428N05, isoform CRA_b	A0A171EBK7	S232	MDSSNTQGIENPGFETTPPFQGMPEAK	S3	1.133	0.123
Vsir	RIKEN cDNA 4632428N05, isoform CRA_b	A0A171EBK7	S232,T/S	MDSSNTQGIENPGFETTPPFQGMPEAK	S3,T/S	1.148	0.053
Vsir	V-type immunoglobulin domain-containing suppressor of T-cell activation	Q9D659	S232	MDSNTQGIENPGFETTPPFQGMPEAK	S3	1.124	0.289
Vsir	V-type immunoglobulin domain-containing suppressor of T-cell activation	Q9D659; A0A171EBK7	S269;S270	TRPPLSYVAQRQPSESGR	S14	1.108	0.379
Vsir	V-type immunoglobulin domain-containing suppressor of T-cell activation	Q9D659	S232,T	MDSNTQGIENPGFETTPPFQGMPEAK	S3,T	1.006	0.929
Vsir	V-type immunoglobulin domain-containing suppressor of T-cell activation	Q9D659	Ambiguous	MDSNTQGIENPGFETTPPFQGMPEAK	S/T	1.125	0.103
Vsir	RIKEN cDNA 4632428N05, isoform CRA_b	A0A171EBK7	Ambiguous	MDSSNTQGIENPGFETTPPFQGMPEAK	T/S	1.032	0.821
Vti1a	Vesicle transport through interaction with t-SNAREs homolog 1A	O89116	S96	IAYSDEVRNELLGDAGNSSENQR	S4	0.983	0.881
Wac	WW domain-containing adapter protein with coiled-coil	Q924H7	S511	QGPVSHSATQQPVTADKQQSHDPVSPR	S25	1.041	0.766
Wac	WW domain-containing adapter protein with coiled-coil	Q924H7	S53	DAADPSPPNK	S6	1.017	0.182
Wac	WW domain-containing adapter protein with coiled-coil	Q924H7	S64	SNSPENKYSDSTGHNK	S3	0.858	0.088
Wapl	Wings apart-like protein homolog	Q65Z40	S226	RTESPSESCPVK	S4	1.017	0.766
Wapl	Wings apart-like protein homolog	Q65Z40	S226	TESPSESCPVK	S3	0.981	0.629
Wapl	Wings apart-like protein homolog	Q65Z40	S77	VEEEDTGDPFGFDSDDESLPVSSK	S14	1.027	0.373
Was	Wiskott-Aldrich syndrome protein homolog	P70315	S501	VIHSSDEGEDQTGEDEEDDEWDD	S4	1.028	0.773
Was	Wiskott-Aldrich syndrome protein homolog	P70315	Y214	YRGLPAPGPGPTDK	Y1	0.985	0.855
Wasf2	Wiskott-Aldrich syndrome protein family member 2	Q8BH43	S20	QTLPSDTSELECR	S5	1.110	0.237
Wasf2	Wiskott-Aldrich syndrome protein family member 2	Q8BH43	S473	DVVGNDVATILSR	S12	0.885	0.666
Washc1	WASH complex subunit 1	Q8VDD8	S376	ATLLESIR	S6	1.133	0.453
Washc2	WASH complex subunit 2	Q6PGL7	S159	AGNSDSEEDDANERVDLILEPK	S6	1.031	0.583
Washc2	WASH complex subunit 2	Q6PGL7	S284	RPTSFADELAAR	S4	1.072	0.443
Washc2	WASH complex subunit 2	Q6PGL7	S388	ARPAQAPVSEELPPSPKPGK	S15	0.974	0.581
Washc2	WASH complex subunit 2	Q6PGL7	S5	TSPDSERPPASEPVWERPWSVEEIR	S2	1.057	0.667
Washc2	WASH complex subunit 2	Q6PGL7	S533	GLFSDEEDSEDLFSSQSSSKPK	S4	1.000	1.000
Washc2	WASH complex subunit 2	Q6PGL7	S747	VSPEVGSADVASIAQK	S2	1.060	0.758
Washc2	WASH complex subunit 2	Q6PGL7	S747	VDNARVSPEVGSADVASIAQK	S7	1.043	0.648
Washc2	WASH complex subunit 2	Q6PGL7	S873	SSVPSGGSLFGDDEDDDLFSSAK	S8	1.100	0.790
Washc2	WASH complex subunit 2	Q6PGL7	S157,S159	AGNSDSEEDDANERVDLILEPK	S4,S6	1.047	0.608
Wbp11	WW domain-binding protein 11	Q923D5	S237	DEDMLYSPELAQR	S7	1.062	0.247
Wbp11	WW domain-binding protein 11	Q923D5	S237	DEDMLYSPELAQR	S7	0.968	0.571
Wbp11	WW domain-binding protein 11	Q923D5	S237	RRDEDMLYSPELAQR	S9	0.905	0.149
Wbp11	WW domain-binding protein 11	Q923D5	S600	SEDDSAVPVAK	S1	1.208	0.202
Wbp11	WW domain-binding protein 11	Q923D5	S604	GATAVPQRRSEDDSAVPVAK	S14	1.287	0.031
Wbp1l	WW domain binding protein 1-like	Q8BGW2	S177	SSTRPPSVADPQSPEVPTDR	S7	1.085	0.197
Wbp1l	WW domain binding protein 1-like	Q8BGW2	T245	GGVSPDSEDKTPGR	T11	1.025	0.873
Wbp1l	WW domain binding protein 1-like	Q8BGW2	Ambiguous	SSTRPPSVADPQSPEVPTDREATK	T/S	0.927	0.261
Wdfy3	WD repeat and FYVE domain-containing protein 3	G3UYW1	S1942	SQSEYCNVGTK	S3	1.044	0.202
Wdfy3	WD repeat and FYVE domain-containing protein 3	G3UYW1	Ambiguous	ATATWCTDSGSDDSR	S/T	1.130	0.139
Wdfy3	WD repeat and FYVE domain-containing protein 3	G3UYW1	Ambiguous	AFAADTGMNRSQSEYCNVGTK	T/S/Y	1.062	0.211
Wdfy4	WD repeat and FYVE domain-containing 4	E9Q2M9	S3122	QAVMEEPSTEPLSPR	S13	1.112	0.047
Wdfy4	WD repeat and FYVE domain-containing 4	E9Q2M9	S3122	QAVMEEPSTEPLSPR	S13	0.896	0.081
Wdhd1	WD repeat and HMG-box DNA-binding protein 1	A0A2K6EDP7	S821	AAELAETQSEEEKEEDFREK	S9	0.997	0.957
Wdhd1	WD repeat and HMG-box DNA-binding protein 1	A0A2K6EDP7	T819,S821	AAELAETQSEEEKEEDFREK	T7,S9	1.003	0.980
Wdhd1	WD repeat and HMG-box DNA-binding protein 1	A0A2K6EDP7	Ambiguous	AAELAETQSEEEKEEDFR	T/S	0.906	0.496
Wdr1	WD repeat-containing protein 1	O88342	S399	DYSGQGVVK	S3	1.062	0.322
Wdr13	WD repeat-containing protein 13	Q91V09	S70	YGPLSEPGSAR	S5	0.964	0.519
Wdr13	WD repeat-containing protein 13	Q91V09	S79	AYSNSIVR	S3	1.030	0.647
Wdr13	WD repeat-containing protein 13	Q91V09	S113,S116	SVSRGSYQLQAQMNR	S3,S6	1.018	0.518
Wdr13	WD repeat-containing protein 13	Q91V09	S70,S74	YGPLSEPGSAR	S5,S9	1.016	0.772
Wdr20	MCG14935, isoform CRA_a	Q3UWE6	S357	RNSTDSRPVSVTYR	S3	0.954	0.635
Wdr20	MCG14935, isoform CRA_a	Q3UWE6	S357	NSTDSRPVSVTYR	S2	1.033	0.671
Wdr20	MCG14935, isoform CRA_a	Q3UWE6	S465	FATLSLHDR	S5	0.842	0.090
Wdr20	MCG14935, isoform CRA_a	Q3UWE6	Ambiguous	SNSLPHSAVSNAASK	S	0.992	0.945
Wdr26	WD repeat-containing protein 26	Q8C6G8	S101	RLSQSDEDVIR	S3	0.985	0.643
Wdr26	WD repeat-containing protein 26	Q8C6G8	S101	KRLSQSDEDVIR	S4	0.998	0.938
Wdr26	WD repeat-containing protein 26	Q8C6G8	S103	LSQSDEDVIR	S4	0.978	0.719
Wdr33	pre-mRNA 3' end processing protein WDR33	Q8K4P0	S1213	SSSLQGMDMASLPPR	S3	0.920	0.330
Wdr34	WD repeat domain 34	A2BE91	S15	VGSAGAAALAAGGAGGAER	S3	0.908	0.731
Wdr4	tRNA (guanine-N(7)-)-methyltransferase non-catalytic subunit WDR4	E9Q156	S435	QRSPFPGSPEQTK	S3	0.995	0.864
Wdr4	tRNA (guanine-N(7)-)-methyltransferase non-catalytic subunit WDR4	E9Q156	S440	SPFPGSPEQTK	S6	0.953	0.471
Wdr4	tRNA (guanine-N(7)-)-methyltransferase non-catalytic subunit WDR4	E9Q156	S435,S440	QRSPFPGSPEQTK	S3,S8	0.975	0.460
Wdr41	WD repeat-containing protein 41	Q3UDP0	S459	LEENGDLYPESP	S11	1.112	0.154
Wdr43	WD repeat-containing protein 43	Q6ZQL4	S432	KLGSTEATIEER	S4	0.969	0.616
Wdr44	WD repeat-containing protein 44	Q6NVE8	S405	EYVSNDATQSDDEEK	S10	1.030	0.364
Wdr44	WD repeat-containing protein 44	Q6NVE8	S50	EAENTANQAGNESPVQELR	S13	1.055	0.270
Wdr44	WD repeat-containing protein 44	Q6NVE8	S563	YNTEGRVSPSPSQESLSSSK	S8	1.018	0.877
Wdr44	WD repeat-containing protein 44	Q6NVE8	T221	HLTPEPDIVASTK	T3	1.044	0.609
Wdr44	WD repeat-containing protein 44	Q6NVE8	T403	EYVSNDATQSDDEEKLQSQQTDTDGGR	T8	1.030	0.511
Wdr44	WD repeat-containing protein 44	Q6NVE8	T403	TKEYVSNDATQSDDEEKLQSQQTDTDGGR	T10	1.182	0.397
Wdr44	WD repeat-containing protein 44	Q6NVE8	T403,S405	TKEYVSNDATQSDDEEKLQSQQTDTDGGR	T10,S12	0.977	0.808
Wdr44	WD repeat-containing protein 44	Q6NVE8	T403,S405	EYVSNDATQSDDEEKLQSQQTDTDGGR	T8,S10	1.119	0.041
Wdr44	WD repeat-containing protein 44	Q6NVE8	Ambiguous	TVDETDNLTEVSSGEQLDASGLEAETLNK	S/T	1.037	0.876
Wdr47	WD repeat-containing protein 47	Q8CGF6	Ambiguous	LSPYPSSPMR	S/Y	1.012	0.852
Wdr48	WD repeat-containing protein 48	Q8BH57	S335	ASGDYDNDCTNPITPLCTQPDQVIK	S2	0.856	0.550
Wdr59	WD repeat-containing protein 59	D3Z1F9	S564	AVSPTEPTPR	S3	1.008	0.915
Wdr62	WD repeat-containing protein 62	E9QK36	T786	WSGPPSQETYASTPSEIR	T13	1.023	0.856
Wdr62	WD repeat-containing protein 62	E9QK36	T799	SLSPGEQTEDEMEEECEPEELLK	T8	0.822	0.171
Wdr62	WD repeat-containing protein 62	E9QK36	T799	SLSPGEQTEDEMEEECEPEELLK	T8	1.234	0.455
Wdr62	WD repeat-containing protein 62	E9QK36	Ambiguous	NQSSPPPAPPLCLR	S	0.941	0.551
Wdr64	WD repeat-containing protein 64	Q9D565	Ambiguous	SSQDSICSSTQCDSSK	S/T	0.913	0.411
Wdr7	WD repeat-containing protein 7	Q920I9	S1153	SSSQIPEGFGLTSGGSNYSLAR	S3	1.004	0.944
Wdr7	WD repeat-containing protein 7	Q920I9	S1455	TYQVPPVQPASPGSHNALK	S11	0.976	0.667
Wdr7	WD repeat-containing protein 7	Q920I9	S935	ARDSPPPSSNIVQGQIK	S4	0.998	0.973
Wdr70	MCG115964	G3X934	S641	TMFAQVESDDEESKNEPEWK	S8	0.990	0.871
Wdr81	WD repeat-containing protein 81 (Fragment)	K4DI77	S1132	GGDDGGAPADKNSVK	S13	1.050	0.272
Wdr81	WD repeat-containing protein 81 (Fragment)	K4DI77	S308	LDLSAYEMPSEDENQEGSEEK	S10	1.011	0.913
Wdr81	WD repeat-containing protein 81 (Fragment)	K4DI77	S593	LAGSPALAPEPPLIPR	S4	1.131	0.332
Wdr81	WD repeat-containing protein 81 (Fragment)	K4DI77	S700	SKAAGLDPGEGEEGK	S1	1.003	0.973
Wdr81	WD repeat-containing protein 81 (Fragment)	K4DI77	T1265	QQFTVSSDDTPPLNAGNIYQK	T10	1.045	0.823
Wdr90	WD repeat-containing protein 90	H7BX49	S20	QGSRPGSPGSEVPAASVTGPR	S7	0.988	0.939
Wdr90	WD repeat-containing protein 90	H7BX49	S16,S/T	QGSRPGSPGSEVPAASVTGPR	S3,S/T	1.094	0.626
Wdr90	WD repeat-containing protein 90	H7BX49	Ambiguous	SCSPPEAVFLGR	S	0.850	0.048
Wdr91	WD repeat-containing protein 91	Q7TMQ7	S346	ELLSTSSSQSQCAER	S4	1.148	0.110
Wdtc1	WD and tetratricopeptide repeats protein 1	Q80ZK9	S511	KDSISEDEMVLR	S3	0.893	0.251
Wee1	Wee1-like protein kinase	P47810	S150	CGGPGDASPQGCGAPR	S8	0.855	0.320
Wfs1	Wolframin	P56695	S158	GITSENEAEVK	S4	1.079	0.363
Whamm	WASP homolog-associated protein with actin, membranes and microtubules	Q571B6	S779	KVSADSEEDNDEPSPTEWDR	S6	1.076	0.467
Whamm	WASP homolog-associated protein with actin, membranes and microtubules	Q571B6	S779	VSADSEEDNDEPSPTEWDR	S5	0.970	0.712
Whamm	WASP homolog-associated protein with actin, membranes and microtubules	Q571B6	S779,S787	KVSADSEEDNDEPSPTEWDR	S6,S14	1.161	0.347
Wipf1	WAS/WASL-interacting protein family member 1	Q8K1I7	S227	GAAFGAGSAR	S8	0.948	0.002
Wipf1	WAS/WASL-interacting protein family member 1	Q8K1I7	S330	NLSLTSSAPPLPSPGR	S3	1.024	0.801
Wipf1	WAS/WASL-interacting protein family member 1	Q8K1I7	T388	ALPATPQLPSR	T5	0.988	0.699
Wipf1	WAS/WASL-interacting protein family member 1	Q8K1I7	Ambiguous	NGFQDSSCEDEWESR	S	1.072	0.450
Wipf2	WAS/WASL-interacting protein family member 2	Q6PEV3	S267	QPPGVPNGPSSPTNESAPELPQR	S11	0.934	0.287
Wipi2	WD repeat domain phosphoinositide-interacting protein 2	Q80W47	S395	GAYVPSSPTR	S7	1.026	0.333
Wiz	Protein Wiz	O88286	S1550	AADSGERPLATSPPGTVK	S12	1.012	0.691
Wiz	Protein Wiz	O88286	S1045,S1050	NPEDKSPQLSLSPRPTSPK	S12,S17	1.011	0.870
Wiz	Protein Wiz	O88286	S1045,S1050	SPQLSLSPRPTSPK	S7,S12	1.080	0.463
Wiz	Protein Wiz	O88286	Ambiguous	VLSTGGPGSSLEAR	S/T	1.275	0.488
Wnk1	Serine/threonine-protein kinase WNK1	P83741	S1256	FIVSPVPESR	S4	1.080	0.375
Wnk1	Serine/threonine-protein kinase WNK1	P83741	S165	DRPVSQPSLVGSK	S5	1.034	0.706
Wnk1	Serine/threonine-protein kinase WNK1	P83741	S172	DRPVSQPSLVGSKEEPPPSR	S12	1.076	0.017
Wnk1	Serine/threonine-protein kinase WNK1	P83741	S1973	EGPVTSPPFR	S6	1.054	0.096
Wnk1	Serine/threonine-protein kinase WNK1	P83741	S2027	GTEDGSGSPHSPPHLCSK	S11	0.981	0.875
Wnk1	Serine/threonine-protein kinase WNK1	P83741	S2367	SISNPPGSNLR	S3	0.953	0.149
Wnk1	Serine/threonine-protein kinase WNK1	P83741	S181,S183	EEPPPSRSGSGSGGASAK	S8,S10	1.009	0.902
Wnk1	Serine/threonine-protein kinase WNK1	P83741	S2024,S2027	GTEDGSGSPHSPPHLCSK	S8,S11	1.012	0.825
Wnk1	Serine/threonine-protein kinase WNK1	P83741	Ambiguous	NGSSSDSSVGEK	S	0.968	0.730
Wtap	MCG16685, isoform CRA_d	E0CYH0	S256	TTSSEPVDQAEVTSK	S3	1.059	0.167
Wtap	MCG16685, isoform CRA_d	E0CYH0	T298	EGSTPEDDFPSSSGNGNK	T4	1.050	0.475
Wwc2	Protein WWC2	Q6NXJ0	S1017	SDSDSSTLAK	S3	1.028	0.477
Wwc2	Protein WWC2	Q6NXJ0	S1017	SDSDSSTLAKK	S3	0.975	0.678
Wwc2	Protein WWC2	Q6NXJ0	S1017	LNRSDSDSSTLAK	S6	0.905	0.008
Wwc2	Protein WWC2	Q6NXJ0	T999	SQTFSPGER	T3	1.019	0.701
Wwox	WW domain-containing oxidoreductase	Q91WL8	S14	YAGLDDTDSEDELPPGWEER	S9	1.105	0.450
Wwp1	NEDD4-like E3 ubiquitin-protein ligase WWP1	Q8BZZ3	S308	SLIDPDSDSR	S1	1.081	0.256
Wwp1	NEDD4-like E3 ubiquitin-protein ligase WWP1	Q8BZZ3	T178	SSSPPIEIQQNGDALHENGDPATRTTPR	T26	1.030	0.571
Wwp1	NEDD4-like E3 ubiquitin-protein ligase WWP1	Q8BZZ3	Ambiguous	QPEGCVEPLRPQSGNTNTEALPSGWEQR	S/T	0.931	0.671
Wwp1	NEDD4-like E3 ubiquitin-protein ligase WWP1	Q8BZZ3	Ambiguous	SSSPPIEIQQNGDALHENGDPATR	S/T	1.056	0.222
Wwp2	NEDD4-like E3 ubiquitin-protein ligase WWP2	Q9DBH0	S211	TATAASEQSPGAR	S9	0.972	0.756
Xirp1	Xin actin-binding repeat-containing protein 1	E9QQ93	Ambiguous	TEEDLSLPHPSAPEGLPPPPPK	S/T	1.221	0.653
Xpc	DNA repair protein complementing XP-C cells homolog	P51612	S349	ETSVEGPGGSSELSSNSPESHNKPTTSR	S17	1.008	0.927
Xpc	DNA repair protein complementing XP-C cells homolog	P51612	S93	VLKEEALSDGDDFR	S8	0.993	0.930
Xpc	DNA repair protein complementing XP-C cells homolog	P51612	Ambiguous	RKPSCSEGEEAEQK	S	1.025	0.848

Xpnpep3	Xaa-Pro aminopeptidase 3	B7ZMP1	Ambiguous	EMNDIEQICSRTS	S/T	1.043	0.595
Xpr1	Xenotropic and polytropic retrovirus receptor 1	Q9Z0U0	S665	YNQSISLR	S4	1.032	0.832
Xpr1	Xenotropic and polytropic retrovirus receptor 1	Q9Z0U0	T689	VLIEDTDDEANT	T6	0.996	0.935
Xrcc1	DNA repair protein XRCC1	Q60596	S446	TQAAGPSSPPRPPTPK	S8	0.972	0.510
Xrcc1	DNA repair protein XRCC1	Q60596	S446,T452	TQAAGPSSPPRPPTPK	S8,T14	1.004	0.879
Xrcc1	DNA repair protein XRCC1	Q60596	T452,S/T	AKTQAAGPSSPPRPPTPK	T16,S/T	0.948	0.409
Xrcc4	DNA repair protein XRCC4	A0A0R4J024	S320	NSSPEDLFD	S3	0.943	0.701
Xrn1	5'-3' exoribonuclease 1	F8VQ87	S1328	NESPGTSEAQK	S3	1.044	0.585
Xrn1	5'-3' exoribonuclease 1	F8VQ87	S1328	RNESPGTSEAQK	S4	1.060	0.497
Xrn1	5'-3' exoribonuclease 1	F8VQ87	S1382	ENEAQLPHHGEPPDEADLSPQSFAMK	S19	0.974	0.242
Xrn2	5'-3' exoribonuclease 2	Q9DBR1	S499	KAEDSDSEPEPEDNVR	S5	1.004	0.933
Xrn2	5'-3' exoribonuclease 2	Q9DBR1	S499,S501	KAEDSDSEPEPEDNVR	S5,S7	0.987	0.545
Xrn2	5'-3' exoribonuclease 2	Q9DBR1	S499,S501	RKAEDSDSEPEPEDNVR	S6,S8	0.970	0.196
Yae1d1	MCG16556, isoform CRA_a	Q9DAY6	S12	AAPENASPGDQDVFDEEADESLLAQR	S7	0.894	0.702
Yaf2	YY1-associated factor 2	Q99LW6	Ambiguous	SSSPRGEASSLNGESH	S	1.032	0.643
Yap1	Transcriptional coactivator YAP1	P46938	S94	QASTDAGTAGALTPQHVR	S3	1.036	0.806
Yars	Tyrosine--tRNA ligase	A2A7S7	S34	LSAGNRDSGAMGDAPSPEEK	S8	0.841	0.244
Ybx1	Nuclease-sensitive element-binding protein 1	P62960	S163	NYQQNYQNSESGEK	S9	0.982	0.867
Ybx1	Nuclease-sensitive element-binding protein 1	P62960	S163	NYQQNYQNSESGEKNEGSESAPEGQAQQR	S9	0.978	0.752
Ybx1	Nuclease-sensitive element-binding protein 1	P62960	S207	RPQYSNPPVQGEVMEGADNQGAGEQGRPVR	S5	1.347	0.003
Ybx1	Nuclease-sensitive element-binding protein 1	P62960	S207	RPQYSNPPVQGEVMEGADNQGAGEQGRPVR	S5	0.978	0.787
Ybx1	Nuclease-sensitive element-binding protein 1	P62960	S163,S172	NYQQNYQNSESGEKNEGSESAPEGQAQQR	S9,S18	0.901	0.019
Ybx1	Nuclease-sensitive element-binding protein 1	P62960	Ambiguous	AADPPAENSSAPEAEQGGAE	S	1.038	0.617
Ybx3	Y-box-binding protein 3	Q9JKB3; P62960	S126;S100	SVGDGETVEFDVVEGEK	S1	1.332	0.052
Ybx3	Y-box-binding protein 3	Q9JKB3	S328	SRPLNAVSQDGK	S1	1.094	0.057
Ybx3	Y-box-binding protein 3	Q9JKB3	S359	AGEAPTENPAPATEQSSAE	S17	1.083	0.506
Yeats2	YEATS domain-containing protein 2	Q3TUF7	S371	ASAVAQSPEPAAAAPVGEGFPETTEAER	S7	1.105	0.393
Yeats2	YEATS domain-containing protein 2	Q3TUF7	S446	IVPQSQVPNPESPGK	S12	1.022	0.754
Yeats2	YEATS domain-containing protein 2	Q3TUF7	S640	QVISAGEGTTQSPK	S12	0.834	0.203
Yju2	YJU2 splicing factor homolog	Q9D6J3	S211,S213	LLEDSESEDEAPPSRPR	S5,S7	1.078	0.366
Yju2	YJU2 splicing factor homolog	Q9D6J3	Ambiguous	LLEDSESEDEAPPSRPR	S	0.943	0.685
Ykt6	Synaptobrevin homolog YKT6	Q9CQW1	S74	SDSLAGVVIADSEYPSR	S3	0.940	0.245
Ylpm1	YLP motif-containing protein 1	D3YWX2	S902	AAQSNENLSDSQQEPTK	S9	1.071	0.437
Ylpm1	YLP motif-containing protein 1	D3YWX2	Ambiguous	GPASQFYITPNTSLSPR	S/T/Y	0.956	0.821
Ypel5	Protein yippee-like 5	P62700	S118	ESEGFEEHVPSDNS	S11	0.928	0.124
Ythdc1	YTH domain-containing protein 1	E9Q5K9	S309	GISPIVFDR	S3	1.289	0.118
Ythdc1	YTH domain-containing protein 1	E9Q5K9	S146,T148	RAKSPTPDGSER	S4,T6	0.952	0.523
Ythdc2	3'-5' RNA helicase YTHDC2	B2RR83	S1105	ADGIPNDSSDSEMEDR	S9	0.928	0.142
Ythdc2	3'-5' RNA helicase YTHDC2	B2RR83	S1105	ADGIPNDSSDSEMEDR	S9	0.797	0.228
Ythdc2	3'-5' RNA helicase YTHDC2	B2RR83	S1216	KSTADTEFADGSTTGER	S2	1.352	0.009
Ythdc2	3'-5' RNA helicase YTHDC2	B2RR83	S1284	STDSSSYSPSPCASPSPPSSGK	S15	1.033	0.422
Ythdc2	3'-5' RNA helicase YTHDC2	B2RR83	S1444	LPLGEKTTSD	S9	1.576	0.147
Ythdc2	3'-5' RNA helicase YTHDC2	B2RR83	S1278,S1282	STDSSSYSPSPCASPSPPSSGK	S9,S13	0.836	0.019
Ythdc2	3'-5' RNA helicase YTHDC2	B2RR83	Ambiguous	ADGIPNDSSDSEMEDRTTANLAALK	T/S	0.809	0.220
Ythdf2	YTH domain-containing family protein 2	Q91YT7	Ambiguous	LGSTEVASSVPK	S/T	0.935	0.621
Ywhab	14-3-3 protein beta/alpha	Q9CQV8	Ambiguous	DNLTLWTSENQGDEGDAGEGEN	S/T	0.816	0.694
Ywhae	14-3-3 protein epsilon	P62259	Ambiguous	DNLTLWTSDMQGDGEEQNK	S/T	1.598	0.085
Ywhag	14-3-3 protein gamma	P61982	Ambiguous	DNLTLWTSDQQDDDGGEGNN	T/S	1.098	0.434
Ywhah	14-3-3 protein eta	P68510	S235	DNLTLWTSDQQDEEAGEGN	S8	1.069	0.373
Ywhah	14-3-3 protein eta	P68510	S38	AVTELNEPLSNEDR	S10	0.928	0.208
Ywhah	14-3-3 protein eta	P68510; F6VW30; P61982; Q9CQV8	S65;S122;S65;S66	VISSIEQK	S4	0.978	0.443
Ywhaq	14-3-3 protein theta (Fragment)	F6VW30	S290	DNLTLWTSDSAGEECDAAEGAEN	S10	1.103	0.415
Ywhaz	14-3-3 protein zeta/delta	P63101	S64	VVSSIEQK	S4	0.978	0.493
Ywhaz	14-3-3 protein zeta/delta	P63101	Ambiguous	DNLTLWTSDTQGDEAEAGEGGEN	T/S	1.097	0.478
Yy1	Transcriptional repressor protein YY1	Q00899	Ambiguous	DIDHETVVEEQIIGENSPPDYSEYMTGK	S/Y/T	0.830	0.101
Zbed5	MCG130675	B2RPU8	S45	RAPAAQPPAAAAPSAVGSPAAAPR	S18	1.033	0.290
Zbed6	Zinc finger BED domain-containing protein 6	D2EAC2	S383	SESPIPVADQDNPVHAQER	S3	0.976	0.198
Zbp1	Z-DNA-binding protein 1	A2APF7	Ambiguous	AMALGDSSPQTTEPVLR	S/T	1.015	0.873
Zbtb1	Zinc finger and BTB domain-containing protein 1	Q91VL9	T356	VTEKDCNESTDNDELEDEPEEPFYR	T10	1.026	0.718
Zbtb11	MCG130893	G5E8B9	S473	DCPDHSQSPGQPSKDEDTLTEATEK	S8	1.072	0.330
Zbtb11	MCG130893	G5E8B9	Ambiguous	ENVTNASQEDSDTGNDTSPEDIGAK	S/T	1.004	0.970
Zbtb14	Zinc finger and BTB domain-containing protein 14	Q08376	Ambiguous	DVSSPDESNGQSK	S	0.978	0.842
Zbtb17	Zinc finger and BTB domain-containing protein 17	Q60821	Ambiguous	DGTEGQPTLAESPPTAPDCLPPAE	S/T	0.882	0.635
Zbtb18	Zinc finger and BTB domain-containing protein 18	H7BX69	Ambiguous	ELDREDKASDDEMMTPESER	S/T	0.951	0.718
Zbtb20	Zinc finger and BTB domain-containing protein 20	Q8K0L9	S303	YLSTTPETTHCR	S3	1.002	0.973
Zbtb21	Zinc finger and BTB domain-containing 21	E9Q444	S1031	IQPLEPDSPTGLPENPTPATEK	S8	1.098	0.324
Zbtb21	Zinc finger and BTB domain-containing 21	E9Q444	S205	ASPSASVK	S2	1.094	0.063
Zbtb21	Zinc finger and BTB domain-containing 21	E9Q444	S439	SFSASQSTDREEASPVTEVR	S3	1.023	0.537
Zbtb21	Zinc finger and BTB domain-containing 21	E9Q444	S462	TEPSSPLSDPSDIIR	S4	0.947	0.694
Zbtb21	Zinc finger and BTB domain-containing 21	E9Q444	S439,S450	SFSASQSTDREEASPVTEVR	S3,S14	1.178	0.006
Zbtb22	Zinc finger and BTB domain-containing protein 22	Q9Z0G7	S211	ASENQSPSSSNYFSPR	S14	0.994	0.863
Zbtb22	Zinc finger and BTB domain-containing protein 22	Q9Z0G7	S203,S/Y	ASENQSPSSSNYFSPR	S6,S/Y	1.260	0.254
Zbtb22	Zinc finger and BTB domain-containing protein 22	Q9Z0G7	S250,T253,T254,S255	DGGPVFPAPVVGSAGTTSGK	S13,T16,T17,S18	0.997	0.974
Zbtb22	Zinc finger and BTB domain-containing protein 22	Q9Z0G7	Ambiguous	ESTDFSSTSQDAFVASAAGSGNR	T/S	1.000	1.000
Zbtb3	Zinc finger and BTB domain-containing protein 3	Q91X45	S308	VEAIVISDEEADLSEEQPHR	S7	1.086	0.393
Zbtb37	Zinc finger and BTB domain-containing 37	Q8C3U9	S481	QAEEGSPSHEEIVAPGESAQGSVSTTGPD	S6	0.971	0.816
Zbtb38	CtBP-interacting BTB zinc finger protein	Q3LR78	Ambiguous	NTLPSSPAVPVETPSR	S/T	1.193	0.313
Zbtb44	Zinc finger and BTB domain-containing protein 44	A0A1L1SSL7	S161	DGSISPVSSECSAVER	S5	0.992	0.874
Zbtb44	Zinc finger and BTB domain-containing protein 44	A0A1L1SSL7	S194	SYIVMSPESPVK	S9	1.182	0.446
Zbtb5	Zinc finger and BTB domain-containing protein 5	Q7TQG0	S366	IDLSPESSDR	S4	1.013	0.484
Zbtb7a	Zinc finger and BTB domain-containing protein 7A	O88939	S141	QILAADDVGDASQPDGAGPTDQR	S12	0.942	0.478
Zbtb7a	Zinc finger and BTB domain-containing protein 7A	O88939	S331	AGDSDEESRTDDK	S4	0.993	0.916
Zbtb7a	Zinc finger and BTB domain-containing protein 7A	O88939	S331	AGDSDEESRTDDKGVMDYYLK	S4	0.962	0.870
Zbtb7a	Zinc finger and BTB domain-containing protein 7A	O88939	S537	HFKDEEEDEEEASPDGSGR	S13	1.005	0.934
Zbtb9	Zinc finger and BTB domain-containing protein 9	Q8CDC7	S172	SSPFQNPVR	S2	0.963	0.770
Zc2hc1a	Zinc finger C2HC domain-containing protein 1A	Q8BJH1	T242	NTTPPSLAR	T2	0.978	0.689
Zc3h11a	Zinc finger CCCH domain-containing protein 11A	Q6NZF1	S132	LSVQSNPSPQLR	S8	0.953	0.450
Zc3h12a	Endoribonuclease ZC3H12A	Q5D1E7	S103	ECQALTAPSPQPPLVPR	S9	0.958	0.770
Zc3h12c	Probable ribonuclease ZC3H12C	E9Q1I3	S489	SNSVPCSTK	S3	0.980	0.691
Zc3h12c	Probable ribonuclease ZC3H12C	E9Q1I3	S670	SYVSSPDPQLEESLK	S4	1.179	0.117
Zc3h12c	Probable ribonuclease ZC3H12C	E9Q1I3	S772	IDSISDSR	S5	1.002	0.966
Zc3h13	Zinc finger CCCH domain-containing protein 13	A0A2I3BPQ5	S1069	GNLETHEDSQVFSPK	S13	0.947	0.135
Zc3h13	Zinc finger CCCH domain-containing protein 13	A0A2I3BPQ5	S110	NTEEPSSPVRK	S7	0.981	0.654
Zc3h13	Zinc finger CCCH domain-containing protein 13	A0A2I3BPQ5	S1273	LRSPSNDSAHR	S3	1.061	0.172
Zc3h13	Zinc finger CCCH domain-containing protein 13	A0A2I3BPQ5	S198	EVSPEVVR	S3	1.005	0.683
Zc3h13	Zinc finger CCCH domain-containing protein 13	A0A2I3BPQ5	S209	LSPSPSLR	S4	1.046	0.491
Zc3h13	Zinc finger CCCH domain-containing protein 13	A0A2I3BPQ5	S242	AAVVASPLLDQQR	S6	0.999	0.985
Zc3h13	Zinc finger CCCH domain-containing protein 13	A0A2I3BPQ5	S64	FIHGPSPR	S6	0.978	0.741
Zc3h13	Zinc finger CCCH domain-containing protein 13	A0A2I3BPQ5	S77	SPERPTGDLR	S1	0.892	0.290
Zc3h13	Zinc finger CCCH domain-containing protein 13	A0A2I3BPQ5	T363	TLTPSLR	T3	0.968	0.556
Zc3h13	Zinc finger CCCH domain-containing protein 13	A0A2I3BPQ5	T363	TLTPSLRR	T3	0.969	0.645
Zc3h13	Zinc finger CCCH domain-containing protein 13	A0A2I3BPQ5	S1273,S1275	LRSPSNDSAHR	S3,S5	1.045	0.661
Zc3h13	Zinc finger CCCH domain-containing protein 13	A0A2I3BPQ5	S207,S209	SKLSPSPSLR	S4,S6	0.985	0.524
Zc3h13	Zinc finger CCCH domain-containing protein 13	A0A2I3BPQ5	S371,S/T/Y	SASPYPTHCLSSPQR	S3,S/T/Y	1.010	0.844
Zc3h13	Zinc finger CCCH domain-containing protein 13	A0A2I3BPQ5	S921,S924,S929	EHSPDSDTYHSGDDKNEK	S3,S6,S11	1.301	0.389
Zc3h13	Zinc finger CCCH domain-containing protein 13	A0A2I3BPQ5	Ambiguous	NTEEPSSPVR	S/T	0.900	0.091
Zc3h13	Zinc finger CCCH domain-containing protein 13	A0A2I3BPQ5	Ambiguous	SLSPSHLTEDR	S/T	0.978	0.742
Zc3h13	Zinc finger CCCH domain-containing protein 13	A0A2I3BPQ5	Ambiguous	DQRPSSPIR	S	0.996	0.958
Zc3h14	Zinc finger CCCH domain-containing protein 14	Q8BJ05	S515	DLVQPDKPASPK	S10	0.886	0.115
Zc3h14	Zinc finger CCCH domain-containing protein 14	Q8BJ05	S620	NGDECVYHHPISPCK	S12	1.104	0.253
Zc3h14	Zinc finger CCCH domain-containing protein 14	Q8BJ05	Ambiguous	TRTSQEELLAEMVQGQNR	T/S	1.026	0.787
Zc3h18	RIKEN cDNA 5830416A07, isoform CRA_c	G3X8T2	S45	ESGSEQDLDGAGERASDLEEEENATR	S16	1.012	0.824
Zc3h18	RIKEN cDNA 5830416A07, isoform CRA_c	G3X8T2	S45	ASDLEEEENATR	S2	1.218	0.092
Zc3h18	RIKEN cDNA 5830416A07, isoform CRA_c	G3X8T2	S554	LGVSVSPSR	S6	1.002	0.932
Zc3h18	RIKEN cDNA 5830416A07, isoform CRA_c	G3X8T2	S64	VQSQEETRSDEEDRASEPK	S9	1.017	0.850
Zc3h18	RIKEN cDNA 5830416A07, isoform CRA_c	G3X8T2	S795	RRDPSAQPPK	S5	0.963	0.752
Zc3h18	RIKEN cDNA 5830416A07, isoform CRA_c	G3X8T2	S862	YEPSDKDRQSPPAK	S10	1.041	0.486
Zc3h18	RIKEN cDNA 5830416A07, isoform CRA_c	G3X8T2	S871	ANLSPDRGSR	S4	0.995	0.923
Zc3h18	RIKEN cDNA 5830416A07, isoform CRA_c	G3X8T2	S92	GPAGSPCEEGDDVEEDGTSDLR	S5	1.125	0.027
Zc3h18	RIKEN cDNA 5830416A07, isoform CRA_c	G3X8T2	S92	GPAGSPCEEGDDVEEDGTSDLRDEASSVTR	S5	1.017	0.774
Zc3h18	RIKEN cDNA 5830416A07, isoform CRA_c	G3X8T2	S552,S554	LGVSVSPSR	S4,S6	1.024	0.652
Zc3h18	RIKEN cDNA 5830416A07, isoform CRA_c	G3X8T2	S71,T/S	VQSQEETRSDEEDRASEPK	S16,T/S	1.015	0.849
Zc3h18	RIKEN cDNA 5830416A07, isoform CRA_c	G3X8T2	S92,T/S	GPAGSPCEEGDDVEEDGTSDLRDEASSVTR	S5,T/S	1.055	0.178
Zc3h3	Zinc finger CCCH domain-containing protein 3	Q8CHP0	S380	ASSPSASSSSFR	S3	1.289	0.012
Zc3h3	Zinc finger CCCH domain-containing protein 3	Q8CHP0	S851	QMSSGLASGAEAPASPPPSPR	S15	0.964	0.555
Zc3h3	Zinc finger CCCH domain-containing protein 3	Q8CHP0	S851	QMSSGLASGAEAPASPPPSPR	S15	0.992	0.933
Zc3h3	Zinc finger CCCH domain-containing protein 3	Q8CHP0	S851,S855	QMSSGLASGAEAPASPPPSPR	S15,S19	0.967	0.827
Zc3h4	Zinc finger CCCH domain-containing protein 4	Q6ZPZ3	S1115	AAKPCPTEASPPAASPSGDSSPPATAPYDPR	S21	0.984	0.709
Zc3h4	Zinc finger CCCH domain-containing protein 4	Q6ZPZ3	S1270	TGTGSPFAGNSPAR	S5	0.998	0.954
Zc3h4	Zinc finger CCCH domain-containing protein 4	Q6ZPZ3	S1276	TGTGSPFAGNSPAREGEQDAGSLK	S11	1.137	0.299
Zc3h4	Zinc finger CCCH domain-containing protein 4	Q6ZPZ3	S1300	GFDPTASPFCQ	S7	1.025	0.898
Zc3h4	Zinc finger CCCH domain-containing protein 4	Q6ZPZ3	S159	DYSPPYAPSHQQYSSSHNAPLPK	S3	1.054	0.472
Zc3h4	Zinc finger CCCH domain-containing protein 4	Q6ZPZ3	S159	YRDYSPPYAPSHQQYSSSHNAPLPK	S5	1.014	0.843
Zc3h4	Zinc finger CCCH domain-containing protein 4	Q6ZPZ3	S916	AEGSLHSSPAGPSSSK	S7	1.017	0.917
Zc3h4	Zinc finger CCCH domain-containing protein 4	Q6ZPZ3	S1104,S1109	AAKPCPTEASPPAASPSGDSSPPATAPYDPR	S10,S15	0.913	0.488
Zc3h4	Zinc finger CCCH domain-containing protein 4	Q6ZPZ3	S1270,S1276	TGTGSPFAGNSPAREGEQDAGSLK	S5,S11	1.038	0.148
Zc3h4	Zinc finger CCCH domain-containing protein 4	Q6ZPZ3	S1270,S1276	TGTGSPFAGNSPAR	S5,S11	1.030	0.519
Zc3h4	Zinc finger CCCH domain-containing protein 4	Q6ZPZ3	S913,S917	AEGSLHSSPAGPSSSK	S4,S8	0.929	0.252

Zc3h6	Zinc finger CCCH domain-containing protein 6	A2AP88	Ambiguous	EPGQASPTPDEETDDKPLK	S/T	1.239	0.052
Zc3hav1	Zinc finger CCCH-type antiviral protein 1	D3Z5I1	S324	ASQEFLEDGDPDGLFSR	S2	0.950	0.730
Zc3hav1	Zinc finger CCCH-type antiviral protein 1	D3Z5I1	S324	GPSQMRASQEFLEDGDPDGLFSR	S8	0.897	0.076
Zc3hav1	Zinc finger CCCH-type antiviral protein 1	D3Z5I1	S350	TSAAGFPLVAAQR	S2	1.122	0.355
Zc3hav1	Zinc finger CCCH-type antiviral protein 1	D3Z5I1	S490	SSPTGFGIK	S2	1.046	0.449
Zc3hav1	Zinc finger CCCH-type antiviral protein 1	D3Z5I1	S509	EAVYSGVQSLR	S5	0.942	0.214
Zc3hav1	Zinc finger CCCH-type antiviral protein 1	D3Z5I1	S538	LPPSPLSSSTSHR	S4	1.021	0.526
Zc3hav1	Zinc finger CCCH-type antiviral protein 1	D3Z5I1	S553	VAASGSPGK	S6	1.042	0.546
Zc3hav1	Zinc finger CCCH-type antiviral protein 1	D3Z5I1	S344,S350	NRSDSSTSRTSAAGFPLVAAQR	S5,S11	0.970	0.812
Zc3hav1	Zinc finger CCCH-type antiviral protein 1	D3Z5I1	S344,S350	SDSSTSRTSAAGFPLVAAQR	S3,S9	0.970	0.524
Zc3hav1	Zinc finger CCCH-type antiviral protein 1	D3Z5I1	S344,S/T	NRSDSSTSRTSAAGFPLVAAQR	S5,S/T	0.995	0.913
Zc3hav1	Zinc finger CCCH-type antiviral protein 1	D3Z5I1	Ambiguous	VAASGSPGKSSTHASVSPASEPSR	S/T	0.983	0.779
Zc3hc1	Nuclear-interacting partner of ALK	Q80YV2	S334	SQDATVSPGSEQSEK	S7	1.026	0.704
Zc3hc1	Nuclear-interacting partner of ALK	Q80YV2	S343	SQDATVSPGSEQSEKSPGPIVSR	S16	0.953	0.311
Zc3hc1	Nuclear-interacting partner of ALK	Q80YV2	S394	SMGTGDSAGVEVPSSPLR	S15	1.146	0.068
Zc3hc1	Nuclear-interacting partner of ALK	Q80YV2	S394	SMGTGDSAGVEVPSSPLRR	S15	0.995	0.827
Zc3hc1	Nuclear-interacting partner of ALK	Q80YV2	S394	SMGTGDSAGVEVPSSPLRR	S15	1.072	0.215
Zc3hc1	Nuclear-interacting partner of ALK	Q80YV2	S394	SMGTGDSAGVEVPSSPLR	S15	1.024	0.741
Zc3hc1	Nuclear-interacting partner of ALK	Q80YV2	S406	LCSSSSSDTSPR	S3	1.098	0.031
Zc3hc1	Nuclear-interacting partner of ALK	Q80YV2	S62	DTAATFQSVDGSPQAEQSPLESTSK	S12	1.046	0.268
Zc3hc1	Nuclear-interacting partner of ALK	Q80YV2	S334,S343	SQDATVSPGSEQSEKSPGPIVSR	S7,S16	1.190	0.029
Zc3hc1	Nuclear-interacting partner of ALK	Q80YV2	S406,S/T	LCSSSSSDTSPR	S3,S/T	1.161	0.013
Zc3hc1	Nuclear-interacting partner of ALK	Q80YV2	T371,T/S	TRSWESSSPVDRPELEAASPTTR	T21,T/S	1.110	0.448
Zc3hc1	Nuclear-interacting partner of ALK	Q80YV2	Ambiguous	SWESSSPVDRPELEAASPTTR	S/T	0.938	0.219
Zc3hc1	Nuclear-interacting partner of ALK	Q80YV2	Ambiguous	TRSWESSSPVDRPELEAASPTTR	T/S	1.291	0.011
Zc3hc1	Nuclear-interacting partner of ALK	Q80YV2	Ambiguous	SWESSSPVDRPELEAASPTTR	S/T	0.855	0.037
Zc3hc1	Nuclear-interacting partner of ALK	Q80YV2	Ambiguous	DTAATFQSVDGSPQAEQSPLESTSK	S/T	0.897	0.503
Zcchc2	Zinc finger CCHC domain-containing protein 2	Q69ZB8	S212	AEGSRGSVEDEPSGDGEQDAEKDGPGPEGSGCAK	S7	0.950	0.720
Zcchc2	Zinc finger CCHC domain-containing protein 2	Q69ZB8	S59	GPPPPPSPPR	S7	1.050	0.434
Zcchc2	Zinc finger CCHC domain-containing protein 2	Q69ZB8	Ambiguous	YAPPLPPSNDTLDSAD	S/T/Y	1.103	0.619
Zcchc8	Zinc finger CCHC domain-containing protein 8	Q9CYA6	S411	DVFASYLNSNIQSPSMR	S13	0.962	0.807
Zcchc8	Zinc finger CCHC domain-containing protein 8	Q9CYA6	S429	SSSQSSPNSPK	S9	0.917	0.373
Zcchc8	Zinc finger CCHC domain-containing protein 8	Q9CYA6	S450	EGSAAASPADMELDSDVEIPPGSQSSK	S15	0.964	0.596
Zcchc8	Zinc finger CCHC domain-containing protein 8	Q9CYA6	S660	THSPVPDMSK	S3	0.976	0.767
Zcchc8	Zinc finger CCHC domain-containing protein 8	Q9CYA6	Ambiguous	GTPPLTPSDSPQARPAASAMDEDALTLEELEEQQR	S/T	1.140	0.383
Zcchc8	Zinc finger CCHC domain-containing protein 8	Q9CYA6	Ambiguous	EGSAAASPADMELDSDVEIPPGSQSSK	S	0.815	0.182
Zdhhc14	Probable palmitoyltransferase ZDHHC14	Q8BQQ1	S456	MLGAGSPLAHSR	S6	1.016	0.654
Zdhhc14	Probable palmitoyltransferase ZDHHC14	Q8BQQ1	S456	MLGAGSPLAHSR	S6	1.013	0.879
Zdhhc18	Palmitoyltransferase ZDHHC18	Q5Y5T2	S19	DCEYQQISPGAAPPPASPGAR	S17	0.938	0.295
Zdhhc18	Palmitoyltransferase ZDHHC18	Q5Y5T2	S19	MKDCEYQQISPGAAPPPASPGAR	S19	1.096	0.184
Zdhhc18	Palmitoyltransferase ZDHHC18	Q5Y5T2	S19	MKDCEYQQISPGAAPPPASPGAR	S19	1.084	0.342
Zdhhc18	Palmitoyltransferase ZDHHC18	Q5Y5T2	S34	RPGPAAPPAPSPGPAPGAPR	S11	0.973	0.603
Zdhhc18	Palmitoyltransferase ZDHHC18	Q5Y5T2	S53	WSGSGSGSGSLGR	S10	1.130	0.033
Zdhhc20	Palmitoyltransferase ZDHHC20	Q5Y5T1	S320	LVGMDPEQASVANQSDYVR	S10	0.899	0.607
Zdhhc5	Palmitoyltransferase ZDHHC5	Q8VDZ4	S380	GDSLKEPTSIADSSR	S3	1.075	0.085
Zdhhc5	Palmitoyltransferase ZDHHC5	Q8VDZ4	S398	HPSYRSEPSLEPESFR	S6	0.932	0.028
Zdhhc5	Palmitoyltransferase ZDHHC5	Q8VDZ4	S455	SEGTTSTSYK	S8	0.995	0.929
Zdhhc5	Palmitoyltransferase ZDHHC5	Q8VDZ4; Q5Y5T5	S529;S534	EPSPVRYDNLSR	S3	0.950	0.459
Zdhhc5	Palmitoyltransferase ZDHHC5	Q8VDZ4	S621	GLGSPEPGTTAPYLGR	S4	1.053	0.462
Zdhhc5	Palmitoyltransferase ZDHHC5	Q8VDZ4	S636	SISYSSQK	S3	1.496	0.000
Zdhhc5	Palmitoyltransferase ZDHHC5	Q8VDZ4	T348	DSPPTPTMYK	T5	1.037	0.433
Zdhhc5	Palmitoyltransferase ZDHHC5	Q8VDZ4	S398,S/T/Y	HPSYRSEPSLEPESFRSPTFGK	S6,S/T/Y	1.137	0.176
Zdhhc5	Palmitoyltransferase ZDHHC5	Q8VDZ4	S684,S/T	SIGSASPGPGQPPLSSPTR	S6,S/T	0.949	0.214
Zdhhc5	Palmitoyltransferase ZDHHC5	Q8VDZ4	T452,S458	SEGTTSTSYKSLANQTR	T5,S11	1.037	0.621
Zdhhc5	Palmitoyltransferase ZDHHC5	Q8VDZ4	Ambiguous	SIGSASPGPGQPPLSSPTR	S/T	1.033	0.163
Zdhhc8	Probable palmitoyltransferase ZDHHC8	Q5Y5T5	S335	TPRPGSAESALSVQR	S6	1.162	0.133
Zdhhc8	Probable palmitoyltransferase ZDHHC8	Q5Y5T5	S603	YGSRDDLVAGPGFGGAR	S3	0.985	0.910
Zdhhc8	Probable palmitoyltransferase ZDHHC8	Q5Y5T5	S672	QGLPSPPGTPR	S5	1.045	0.338
Zeb2	Zinc finger E-box-binding homeobox 2	A0A0M3HEP2	S1166	AYLQSITPQGYSDSEER	S12	1.013	0.605
Zeb2	Zinc finger E-box-binding homeobox 2	A0A0M3HEP2	S1166	AYLQSITPQGYSDSEERESMPR	S12	0.867	0.033
Zeb2	Zinc finger E-box-binding homeobox 2	A0A0M3HEP2	S1166	AYLQSITPQGYSDSEERESMPR	S12	0.974	0.462
Zeb2	Zinc finger E-box-binding homeobox 2	A0A0M3HEP2	S1180	DGESEKEHEKEGEEGYGK	S4	0.948	0.330
Zeb2	Zinc finger E-box-binding homeobox 2	A0A0M3HEP2	S404	TGSSPNSVSSSPTNSAITQLR	S11	0.954	0.480
Zeb2	Zinc finger E-box-binding homeobox 2	A0A0M3HEP2	S749	VYQYSNSRSPSLER	S9	0.965	0.652
Zeb2	Zinc finger E-box-binding homeobox 2	A0A0M3HEP2	S749	KVYQYSNSRSPSLER	S10	0.987	0.839
Zeb2	Zinc finger E-box-binding homeobox 2	A0A0M3HEP2	S397,S400	TGSSPNSVSSSPTNSAITQLR	S4,S7	1.004	0.942
Zeb2	Zinc finger E-box-binding homeobox 2	A0A0M3HEP2	S828,S/T	SNTPSPLNLSSTSSK	S5,S/T	1.117	0.137
Zeb2	Zinc finger E-box-binding homeobox 2	A0A0M3HEP2	T1161,S1166	AYLQSITPQGYSDSEER	T7,S12	1.001	0.994
Zeb2	Zinc finger E-box-binding homeobox 2	A0A0M3HEP2	Ambiguous	SNTPSPLNLSSTSSK	T/S	1.014	0.822
Zfand2b	AN1-type zinc finger protein 2B	Q91X58	S163	AQGLASTSTAPSPSR	S12	1.033	0.554
Zfand2b	AN1-type zinc finger protein 2B	Q91X58	S173	TLPSSSSPSR	S7	0.990	0.446
Zfand2b	AN1-type zinc finger protein 2B	Q91X58	T178	TLPSSSSPSRATPQLPTR	T12	0.894	0.119
Zfand2b	AN1-type zinc finger protein 2B	Q91X58	T178,S/T	TLPSSSSPSRATPQLPTR	T12,S/T	0.934	0.028
Zfand3	AN1-type zinc finger protein 3	Q497H0	S129	SCGADSQSENEASPVKRPR	S13	1.069	0.415
Zfand3	AN1-type zinc finger protein 3	Q497H0	S129	SCGADSQSENEASPVK	S13	0.970	0.819
Zfand5	AN1-type zinc finger protein 5	O88878	S48	MSPMGTASGSNSPTSDSASVQR	S2	0.962	0.330
Zfand5	AN1-type zinc finger protein 5	O88878	S48	MSPMGTASGSNSPTSDSASVQR	S2	1.017	0.640
Zfand5	AN1-type zinc finger protein 5	O88878	S58	QQNSGRMSPMGTASGSNSPTSDSASVQR	S18	1.037	0.866
Zfc3h1	Zinc finger, C3H1-type-containing	B2RT41	S356	RLSASDIVSEK	S3	1.152	0.298
Zfc3h1	Zinc finger, C3H1-type-containing	B2RT41	S658	AFKPEETSSNSDPPSPPVLNNSQPLSR	S15	1.092	0.617
Zfc3h1	Zinc finger, C3H1-type-containing	B2RT41	S952	LDNSPISSPR	S4	0.952	0.301
Zfc3h1	Zinc finger, C3H1-type-containing	B2RT41	S1304,S/T	KPVSENSCSSDDEQSTGPIK	S7,S/T	0.949	0.677
Zfc3h1	Zinc finger, C3H1-type-containing	B2RT41	S807,S808	TSSSSPANSDVEMDGIGR	S4,S5	1.137	0.411
Zfc3h1	Zinc finger, C3H1-type-containing	B2RT41	S952,S956	LDNSPISSPR	S4,S8	0.994	0.950
Zfc3h1	Zinc finger, C3H1-type-containing	B2RT41	Ambiguous	TSSSSPANSDVEMDGIGR	S/T	1.014	0.747
Zfhx2	Zinc finger homeobox protein 2	Q2MHN3	Ambiguous	EVAVLPAPAGSPEDTSDPPPSCR	T/S	1.173	0.150
Zfhx3	Zinc finger homeobox protein 3	Q61329	T2919	EYDNEGTVDYSETSSLADPCSPSPGASGSAGK	T7	0.966	0.800
Zfp169	Zinc finger protein 169	E9Q3R6	S156	DSSDALLQRTSPTR	S11	0.915	0.525
Zfp217	Zinc finger protein 217	Q3U0X6	S415	TDALSPTMAVDAR	S5	1.080	0.422
Zfp217	Zinc finger protein 217	Q3U0X6	S415	TDALSPTMAVDAR	S5	0.974	0.803
Zfp217	Zinc finger protein 217	Q3U0X6	S415	RTDALSPTMAVDAR	S6	0.972	0.816
Zfp263	Zinc finger protein 263	Q8CF60	S180	LQELLDPSPQR	S8	0.794	0.560
Zfp263	Zinc finger protein 263	Q8CF60	S312	NVESVSPESTHPPVLLPGQAR	S6	0.981	0.922
Zfp280b	Suppressor of hairy wing homolog 2 (Drosophila)	Q505F4	S112	LQPSRPAAPTSEAVVVLPASPAESR	S20	1.037	0.638
Zfp280b	Suppressor of hairy wing homolog 2 (Drosophila)	Q505F4	S221	TPSFDAPNSLSR	S3	1.028	0.758
Zfp280b	Suppressor of hairy wing homolog 2 (Drosophila)	Q505F4	Ambiguous	SSSPQVVPSSSSK	S	0.954	0.526
Zfp292	Zinc finger protein 292	Q9Z2U2	T1325	GGSSENGVFPSQVSSADDFSSTSAQPSTPK	T28	1.050	0.824
Zfp292	Zinc finger protein 292	Q9Z2U2	Ambiguous	LINEDSTNAENQGNTTLK	T/S	0.945	0.716
Zfp36	mRNA decay activator protein ZFP36	P22893	S178	QSISFSGLPSGR	S4	1.260	0.023
Zfp36	mRNA decay activator protein ZFP36	P22893	S52	STSLVEGR	S3	1.388	0.000
Zfp362	Zinc finger protein 362	B1ASA5	S408	TESPGIPVR	S3	1.130	0.201
Zfp36l1	mRNA decay activator protein ZFP36L1	P23950	S92	SFSEGGER	S3	1.309	0.017
Zfp36l2	mRNA decay activator protein ZFP36L2	P23949	S127	SFSENGER	S3	1.201	0.000
Zfp36l2	mRNA decay activator protein ZFP36L2	P23949; P23950	S480;S334	LSISDD	S2	1.025	0.903
Zfp395	Zinc finger protein 395	E9Q5N9	S444	SLSFSEPQQPPPTVK	S3	0.935	0.600
Zfp395	Zinc finger protein 395	E9Q5N9	Ambiguous	SHLIVTSPPR	T/S	0.893	0.070
Zfp407	MCG1556, isoform CRA_b	G3UVV3	S1228	STPCEDEGGSVDDSEEK	S10	1.053	0.018
Zfp444	Zinc finger protein 444	Q3TDV8	S239	GTHPGSPGPALRPLPAR	S6	0.987	0.934
Zfp472	Zinc finger protein 472	B0V2W5	S92	YDYGNQYGETHQQSPEHVANEK	S14	0.889	0.641
Zfp532	Zinc finger protein 532	S4R2I9	S306,S/T	EVNDSPKAADKSPESQNLIDGTK	S5,S/T	0.900	0.401
Zfp638	Zinc finger protein 638	E9QML5	S1099	GSTFSPDLK	S5	1.091	0.222
Zfp638	Zinc finger protein 638	E9QML5	S128	QSSVTQVTEQSPK	S11	1.004	0.966
Zfp638	Zinc finger protein 638	E9QML5	S1400	ATVVSSPK	S6	1.023	0.739
Zfp638	Zinc finger protein 638	E9QML5	S881	SAEESPSGTLEATEKEPVNK	S5	1.063	0.548
Zfp646	Zinc finger protein 646	Q6NV66	S523	STDPGTEGSPSPVKK	S11	0.926	0.335
Zfp654	Zinc finger protein 654	A0A140LI34	S500	RVDQQSVEEDQSTGETDPDDASVVQPK	S12	1.009	0.881
Zfp654	Zinc finger protein 654	A0A140LI34	Ambiguous	VDQQSVEEDQSTGETDPDDASVVQPK	S/T	1.020	0.855
Zfp654	Zinc finger protein 654	A0A140LI34	Ambiguous	CQTLFGFDSDDESA	S/T	1.381	0.573
Zfp655	Zinc finger protein 655	Q9CZP3	S28	GHFQPLENQSECLSPELR	S14	1.116	0.190
Zfp655	Zinc finger protein 655	Q9CZP3	S80	GDCSDGETREENK	S4	1.079	0.067
Zfp655	Zinc finger protein 655	Q9CZP3	S80	EVIRGDCSDGETREENK	S8	1.125	0.201
Zfp655	Zinc finger protein 655	Q9CZP3	S80	EVIRGDCSDGETR	S8	1.092	0.613
Zfp740	Zinc finger protein 740 (Fragment)	D3Z4A3	S44	AGSPDVLR	S3	1.009	0.797
Zfp777	Zinc finger protein 777	B9EKF4	S659	SGYASPERGSAFNPK	S5	0.941	0.592
Zfp869	Zinc finger protein 869	Q9DC47	S155	GGHTEENDSEGNQCEK	S9	1.033	0.893
Zfp91	E3 ubiquitin-protein ligase ZFP91	Q62511	S86,S106	SSPSNRPPDGPGHQPAAAKPPSPAQGK	S2,S22	0.988	0.876
Zfpm1	Zinc finger protein ZFPM1	O35615	S286	AGSPVSATEEKPK	S3	1.088	0.418
Zfpm1	Zinc finger protein ZFPM1	O35615	S681	GSEGSQSPGSSVDDAEDDPSR	S7	0.962	0.601
Zfpm1	Zinc finger protein ZFPM1	O35615	S925	APRSPSPAPENTPSDPADQGAR	S4	0.947	0.672
Zfr	Zinc finger RNA-binding protein	O88532	S1054	RDSDGVDGFEAEGK	S3	1.140	0.014
Zfr	Zinc finger RNA-binding protein	O88532	S1054	RDSDGVDGFEAEGKK	S3	1.042	0.410
Zfr	Zinc finger RNA-binding protein	O88532	S1054	RRDSDGVDGFEAEGK	S4	1.092	0.137
Zfx	Zinc finger X-chromosomal protein	P17012	Ambiguous	ADPGEDDLGGTVDIVESEPENEHGVELLDPNNSIR	S/T	0.659	0.134
Zfyve1	Zinc finger FYVE domain-containing protein 1	Q810J8	S129	VQESLEGEEMDEETK	S4	1.212	0.158
Zfyve1	Zinc finger FYVE domain-containing protein 1	Q810J8	S129	VQESLEGEEMDEETKR	S4	0.971	0.567
Zfyve16	Zinc finger FYVE domain-containing protein 16	Q80U44	S927	SEISQSPTCHTAPVER	S6	0.954	0.788
Zfyve19	Abscission/NoCut checkpoint regulator	Q9DAZ9	S219	TNSQGQASQSLEEEKYK	S3	1.114	0.003
Zfyve19	Abscission/NoCut checkpoint regulator	Q9DAZ9	S219	TNSQGQASQSLEEEK	S3	1.014	0.858

Zfyve19	Abscission/NoCut checkpoint regulator	Q9DAZ9	S280	VTLQDYHLPDSDEDEETAIQR	S11	0.959	0.543
Zfyve19	Abscission/NoCut checkpoint regulator	Q9DAZ9	S69	WSPPQNYK	S2	1.020	0.848
Zfyve26	Zinc finger FYVE domain-containing protein 26	Q5DU37	S1730	RSDSMIHLQEPVHQASDSETLSR	S2	0.988	0.915
Zfyve26	Zinc finger FYVE domain-containing protein 26	Q5DU37	S1732	SDSMIHLQEPVHQASDSETLSR	S3	1.038	0.805
Zfyve26	Zinc finger FYVE domain-containing protein 26	Q5DU37	S605	GPLGLRSPSESPQHIAATER	S7	1.022	0.907
Zfyve26	Zinc finger FYVE domain-containing protein 26	Q5DU37	T1792	AFPQTQPPVEFVPPETPPAR	T16	0.900	0.696
Zfyve26	Zinc finger FYVE domain-containing protein 26	Q5DU37	Ambiguous	SDSMIHLQEPVHQASDSETLSR	S/T	1.012	0.928
Zfyve26	Zinc finger FYVE domain-containing protein 26	Q5DU37	Ambiguous	SSSAEFSAAAAPGSALVR	S	1.018	0.805
Zfyve28	Lateral signaling target protein 2 homolog	Q6ZPK7	S392	LKSGSDEEER	S3	1.118	0.034
Zfyve28	Lateral signaling target protein 2 homolog	Q6ZPK7	S538	SPTSQDSAVAAQEAPGHGTSPLEPR	S20	1.194	0.014
Zgpat	Zinc finger CCCH-type with G patch domain-containing protein	Q8VDM1	S263	EAVVEGDSILPPLRTEATESSDSDTGDASDSSYAR	S8	0.887	0.310
Zgpat	Zinc finger CCCH-type with G patch domain-containing protein	Q8VDM1	T416	LQSQVPGTPDAGVDTPERR	T15	0.858	0.247
Zgrf1	Protein ZGRF1	Q0VGT4	S462	EGSQGADADAALEPEYRPVSPLPEIGHK	S20	0.827	0.133
Zhx1	Zinc fingers and homeoboxes protein 1	P70121	Ambiguous	IEVDESNVGSSKEEPGESSPGDETVAPK	T/S	1.114	0.172
Zkscan3	Zinc finger protein with KRAB and SCAN domains 3	A0A0R4J1L3	S223	MEDVAPVLSPR	S9	1.049	0.753
Zkscan3	Zinc finger protein with KRAB and SCAN domains 3	A0A0R4J1L3	T136	QLDDTPPQVPDDDDGQELLCSK	T5	1.107	0.176
Zmat1	Zinc finger matrin-type protein 1	Q3V0C1	S179	SHSPTQNQSLEEHDQVSPSTCSPK	S3	0.927	0.687
Zmpste24	CAAX prenyl protease 1 homolog	Q80W54	S310	NEGEGDSEEVKAK	S7	1.044	0.542
Zmym2	Zinc finger MYM-type protein 2	Q9CU65	S305	QQGVDSLSPVASLPK	S8	0.995	0.941
Zmym4	Zinc finger MYM-type protein 4	A2A791	S121	AAHQESDNENEIQIQNQLK	S6	0.993	0.953
Zmym4	Zinc finger MYM-type protein 4	A2A791	S1242	CGGGELASASPCSDSLGSAQDHALSQESSEQGCK	S10	0.990	0.964
Zmym6	Zinc finger, MYM-type 6	Q8BS54	S654	SEEPESPPAK	S6	1.002	0.972
Zmynd11	Zinc finger MYND domain-containing protein 11	Q8R5C8	Ambiguous	KEEPEPETEAVSSSQEIPTMPQPIER	S/T	1.039	0.489
Zmynd8	Zinc finger, MYND-type-containing 8	A2A484	S410	LNFDMTASPK	S8	1.070	0.551
Zmynd8	Zinc finger, MYND-type-containing 8	A2A484	S479	STASPASTK	S4	1.027	0.809
Zmynd8	Zinc finger, MYND-type-containing 8	A2A484	S658	SNSPVSEKPDPTPAK	S3	1.021	0.855
Zmynd8	Zinc finger, MYND-type-containing 8	A2A484	S763	QDAIGKPPPSSTSAGNQSPPETPVLTR	S18	1.057	0.139
Znf106	Zinc finger protein 106	O88466	S1041	ATGDGSSPELPSLER	S7	1.082	0.616
Znf106	Zinc finger protein 106	O88466	S1218	QEPMSPEQEGNMNALPQGCASNVSK	S5	1.045	0.455
Znf106	Zinc finger protein 106	O88466	S1339	ETQSPADQPEQQAESTLASAETR	S4	0.771	0.208
Znf106	Zinc finger protein 106	O88466	S431	AGPPGSPSHK	S6	1.011	0.893
Znf106	Zinc finger protein 106	O88466	S878	SLSESSVVMDR	S3	1.141	0.078
Znf106	Zinc finger protein 106	O88466	S878	SLSESSVVMDR	S3	1.043	0.309
Znf106	Zinc finger protein 106	O88466	Ambiguous	RATGDGSSPELPSLER	S/T	0.976	0.635
Znf131	Zinc finger protein 131	Q8K3J5	S231	GQISEDDCASDPISK	S4	1.041	0.693
Znf143	Zinc finger protein 143	O70230	T633	IQQGETPGLDD	T6	0.991	0.899
Znf148	Zinc finger protein 148	Q61624	S306	GGLLTSEEDSGFSTSPK	S15	1.045	0.649
Znf148	Zinc finger protein 148	Q61624	S784	AGMTSSPDATTGQTFG	S6	1.014	0.843
Znf148	Zinc finger protein 148	Q61624	S784	AGMTSSPDATTGQTFG	S6	1.029	0.930
Znf148	Zinc finger protein 148	Q61624	Ambiguous	QPLEQSQTISPLSSYEDSK	T/S/Y	0.858	0.056
Znf219	Zinc finger protein 219	Q6IQX8	S702	APSGETPPSPPLEEEGSPGLSR	S9	0.876	0.173
Znf219	Zinc finger protein 219	Q6IQX8	T687	ADTSPTYVR	T3	1.033	0.610
Znf219	Zinc finger protein 219	Q6IQX8	S702,S710	APSGETPPSPPLEEEGSPGLSR	S9,S17	1.114	0.244
Znf276	Zinc finger protein 276	Q8CE64	S378	QTPQSSDESFEPYPEK	S5	0.925	0.625
Znf318	Zinc finger protein 318	Q99PP2	S167	SPGLCSDSSLEESLR	S1	1.053	0.603
Znf318	Zinc finger protein 318	Q99PP2	S167	RRSPGLCSDSSLEESLR	S3	1.000	0.996
Znf318	Zinc finger protein 318	Q99PP2	S1988	SPTALSEK	S1	1.021	0.775
Znf318	Zinc finger protein 318	Q99PP2	S205	LGSPVDGLQDMDRDDLTDDSVFTR	S3	0.861	0.253
Znf318	Zinc finger protein 318	Q99PP2	S2206	DQVVGGNVSPR	S9	0.998	0.883
Znf318	Zinc finger protein 318	Q99PP2	S41	RPSSPPPPSCSSLR	S4	1.047	0.658
Znf318	Zinc finger protein 318	Q99PP2	S557	SFPDIEDEEK	S1	0.960	0.770
Znf318	Zinc finger protein 318	Q99PP2	T193	ITVGNDHFCVSTPERR	T12	1.005	0.972
Znf318	Zinc finger protein 318	Q99PP2	S2206,S/T	TPPSGSPSRDQVVGGNVSPR	S18,S/T	1.107	0.190
Znf318	Zinc finger protein 318	Q99PP2	S79,S81,S187,S303	RGSPSPPR	S3,S5	0.914	0.419
Znf335	Zinc finger protein 335	A2A5K6	S419	GLVESGVSQSDAENAAPSCQDEADAPPR	S10	1.092	0.227
Znf335	Zinc finger protein 335	A2A5K6	S835	SEGIEAELTSTGGQPSPEDTTPR	S16	1.122	0.716
Znf335	Zinc finger protein 335	A2A5K6	S976	DGSEVLSPTK	S7	0.939	0.663
Znf367	Zinc finger protein 367	Q0VDT2	S300	ADQEQQDPLEYLQSDEEDDEKSGAQR	S14	1.036	0.836
Znf367	Zinc finger protein 367	Q0VDT2	Ambiguous	ADQEQQDPLEYLQSDEEDDEK	Y/S	0.922	0.631
Znf385a	Zinc finger protein 385A	Q8VD12	S160	QPGSPSPPSVPESGQGVTK	S4	1.043	0.403
Znf503	Zinc finger protein 503	Q7TMA2	Ambiguous	TGSPSSSASACSPGGMLPSAGGGPEGK	T/S	1.062	0.726
Znf513	Zinc finger protein 513	Q6PD29	S85	DSEGDSQGARPGLPYGLSDDESGGGR	S18	1.067	0.771
Znf513	Zinc finger protein 513	Q6PD29	S99	ALSAESEVEEPARGPGEAR	S6	1.062	0.359
Znf513	Zinc finger protein 513	Q6PD29	S96,S99	ALSAESEVEEPARGPGEAR	S3,S6	0.775	0.121
Znf516	Zinc finger protein 516	Q7TSH3	S116	VSEGLDGCASPTK	S10	0.963	0.086
Znf516	Zinc finger protein 516	Q7TSH3	S116	VSEGLDGCASPTKSTSACNR	S10	1.019	0.864
Znf516	Zinc finger protein 516	Q7TSH3	S720	RASAPDLVPLDLSMR	S3	0.786	0.290
Znf516	Zinc finger protein 516	Q7TSH3	S919	LAPSPGSGSLSR	S11	1.026	0.528
Znf516	Zinc finger protein 516	Q7TSH3	T923	STTPTPSVITR	T3	0.997	0.874
Znf516	Zinc finger protein 516	Q7TSH3	S912,S	LAPSPGSGSLSR	S4,S	1.123	0.477
Znf516	Zinc finger protein 516	Q7TSH3	Ambiguous	GSSSPLGVTTK	S/T	1.007	0.947
Znf516	Zinc finger protein 516	Q7TSH3	Ambiguous	LAPSPGSGSLSRSTTPTPSVITR	S/T	1.024	0.918
Znf518a	Zinc finger protein 518A	B2RRF6	S515	AAPPACSSPVLAR	S8	1.162	0.061
Znf592	Zinc finger protein 592	Q8BHZ4	S1202	VEAPDSEACSGEEVAMETK	S10	0.963	0.480
Znf592	Zinc finger protein 592	Q8BHZ4	S332	SPRSPLEATR	S4	0.891	0.254
Znf592	Zinc finger protein 592	Q8BHZ4	S366	GSPSVAASSPPAIPK	S9	0.871	0.358
Znf592	Zinc finger protein 592	Q8BHZ4	S39	EAIQAPSEENESPLK	S12	0.987	0.850
Znf593	Zinc finger protein 593	Q9DB42	Ambiguous	LGVPTEVSTDIPEMDTST	S/T	1.012	0.959
Znf608	Zinc finger protein 608	Q56A10	S420	FCESPTSDLEMR	S4	0.948	0.796
Znf608	Zinc finger protein 608	Q56A10	S626	APGSPGAGNPPGTPK	S4	0.929	0.312
Znf608	Zinc finger protein 608	Q56A10	S963	ASSPSDTFSNK	S3	0.970	0.694
Znf609	Zinc finger protein 609	Q8BZ47	S358	FCDSPTSDLEMR	S4	0.807	0.011
Znf609	Zinc finger protein 609	Q8BZ47	S358	FCDSPTSDLEMR	S4	1.103	0.604
Znf609	Zinc finger protein 609	Q8BZ47	S467	TNSMGSATGPLPGTK	S3	1.024	0.477
Znf609	Zinc finger protein 609	Q8BZ47	S491	NCPSPVLIDCPHPNCNK	S4	0.930	0.424
Znf622	Zinc finger protein 622	Q91VY9	S145	AQPSTSPK	S6	1.111	0.318
Znf629	Zinc finger protein 629	Q6A085	Ambiguous	ASTPEDTPSESATLSTNQEDEGEASTPPK	S/T	0.802	0.232
Znf639	Zinc finger protein 639	Q99KZ6	S200	ANSSGLYK	S3	1.161	0.012
Znf639	Zinc finger protein 639	Q99KZ6	S88	NQSYLVPSPVLR	S8	0.981	0.771
Znf652	Zinc finger protein 652	Q5DU09	S203	AASAAAATTSPAPR	S10	0.941	0.423
Znf652	Zinc finger protein 652	Q5DU09	S55	ESGSPYSVLADTK	S2	0.987	0.854
Znf687	Zinc finger protein 687	Q9D2D7	S104	NTVCPEQSESLTGDSGEEETK	S15	0.984	0.808
Znf687	Zinc finger protein 687	Q9D2D7	Ambiguous	QSSDSCSEEPDSTTPPAK	S/T	1.065	0.749
Znf687	Zinc finger protein 687	Q9D2D7	Ambiguous	SSGSAEQSLVGLR	S	1.209	0.247
Znf703	Zinc finger protein 703	P0CL69	S191	DSGSSSVSSTTSSSSSSPGDK	S17	1.654	0.008
Znf768	Zinc finger protein 768	Q8R0T2	S149	YEPKSPGYGSK	S5	1.043	0.635
Znf768	Zinc finger protein 768	Q8R0T2	S191	THSPEFETQSSK	S3	1.081	0.437
Znf768	Zinc finger protein 768	Q8R0T2	S86	FEPESPGFESR	S5	1.025	0.657
Znf768	Zinc finger protein 768	Q8R0T2	Ambiguous	DVHSPNAVGSPEGSLKDPAGNTSENEEGEISQR	T/S	1.085	0.424
Znf768	Zinc finger protein 768	Q8R0T2	Ambiguous	DVHSPNAVGSPEGSLKDPAGNTSENEEGEISQR	S/T	0.839	0.352
Znf800	Zinc finger protein 800	Q0VEE6	S455,S460	QDSESPKSASPSAAGGQQK	S5,S10	1.008	0.943
Znhit6	Box C/D snoRNA protein 1	Q3UFB2	S39	DLDGSPEAGDGEER	S5	1.019	0.856
Znrf2	E3 ubiquitin-protein ligase ZNRF2	Q71FD5	S18	AYSGSDLPSGTGSGGGGADGAR	S3	1.011	0.783
Znrf2	E3 ubiquitin-protein ligase ZNRF2	Q71FD5	S75	SLGGAVGSASGGR	S1	1.041	0.257
Znrf2	E3 ubiquitin-protein ligase ZNRF2	Q71FD5	S75	SRSLGGAVGSASGGR	S3	1.057	0.131
Znrf2	E3 ubiquitin-protein ligase ZNRF2	Q71FD5	S28,S/T/Y	AYSGSDLPSGTGSGGGGADGAR	S13,S/T/Y	1.191	0.138
Znrf2	E3 ubiquitin-protein ligase ZNRF2	Q71FD5	Ambiguous	AAQSAFSIPSAGGGGGPYGSQDSVHSSPEDSVGAR	S/Y	1.053	0.518
Znrf2	E3 ubiquitin-protein ligase ZNRF2	Q71FD5	Ambiguous	AAQSAFSIPSAGGGGGPYGSQDSVHSSPEDSVGAR	S/Y	0.692	0.136
Zranb1	Ubiquitin thioesterase Zranb1	A0A1L1SQ24	S723	QIRPCTSLSDGEEDEDDEDE	S9	1.030	0.674
Zranb2	Zinc finger Ran-binding domain-containing protein 2	Q9R020	S120	ENVEYIEREESDGEYDEFGR	S11	1.146	0.434
Zranb2	Zinc finger Ran-binding domain-containing protein 2	Q9R020	S120	ENVEYIEREESDGEYDEFGRK	S11	1.017	0.575
Zranb2	Zinc finger Ran-binding domain-containing protein 2	Q9R020	S153	EVEDKESEGEEEDEDEDLSKYK	S7	1.070	0.531
Zranb2	Zinc finger Ran-binding domain-containing protein 2	Q9R020	S153	EVEDKESEGEEEDEDEDLSK	S7	1.000	0.998
Zranb2	Zinc finger Ran-binding domain-containing protein 2	Q9R020	S188	LDEDEDEDDADLSKYNLDASEEEDSNK	S20	1.146	0.496
Zranb2	Zinc finger Ran-binding domain-containing protein 2	Q9R020	S188	LDEDEDEDDADLSKYNLDASEEEDSNKK	S20	1.003	0.967
Zranb2	Zinc finger Ran-binding domain-containing protein 2	Q9R020	S188	YNLDASEEEDSNKK	S6	1.084	0.366
Zranb2	Zinc finger Ran-binding domain-containing protein 2	Q9R020	S188	YNLDASEEEDSNK	S6	1.079	0.501
Zranb2	Zinc finger Ran-binding domain-containing protein 2	Q9R020	Ambiguous	TGYGGGFNERENVEYIEREESDGEYDEFGR	Y/S/T	0.941	0.651
Zrsr1	2 small nuclear ribonucleoprotein auxiliary factor 35 kDa subunit-related protein	Q64707	S50	ALEAPPEEDDDVSANEELAER	S13	0.909	0.356
Zscan18	Zinc finger and SCAN domain-containing 18	E9PUD6	S689	ALDAEGEDSSPASPQR	S13	0.911	0.434
Zswim8	Zinc finger SWIM domain-containing protein 8	Q3UHH1	S1094	NVPESSPHSPCEGLPPEAALTPRPEGK	S9	1.010	0.925
Zswim8	Zinc finger SWIM domain-containing protein 8	Q3UHH1	S53	QSAGPNSPTGGGGGGGSGGTR	S7	1.087	0.555
Zswim8	Zinc finger SWIM domain-containing protein 8	Q3UHH1	S53	KQSAGPNSPTGGGGGGGSGGTR	S8	1.040	0.551
Zswim8	Zinc finger SWIM domain-containing protein 8	Q3UHH1	S564	RLSAEGGDK	S3	1.155	0.017
Zswim8	Zinc finger SWIM domain-containing protein 8	Q3UHH1	S701	DDLPSTDDSGSGLHK	S5	0.948	0.533
Zswim8	Zinc finger SWIM domain-containing protein 8	Q3UHH1	S1094,S/T	NVPESSPHSPCEGLPPEAALTPRPEGK	S9,S/T	0.997	0.987
Zswim8	Zinc finger SWIM domain-containing protein 8	Q3UHH1	S1157,S1158	HTGMASIDSSAPETTSDSSPTLSR	S18,S19	1.002	0.985
Zswim8	Zinc finger SWIM domain-containing protein 8	Q3UHH1	Ambiguous	HTGMASIDSSAPETTSDSSPTLSR	S/T	0.980	0.812
Zswim8	Zinc finger SWIM domain-containing protein 8	Q3UHH1	Ambiguous	HTGMASIDSSAPETTSDSSPTLSR	S/T	0.985	0.853
Zw10	Centromere/kinetochore protein zw10 homolog	O54692	S438	EALPDLPSPDADHK	S8	0.985	0.664
Zyx	Zyxin	Q62523	S248	GPLSQAPTPAPK	S4	1.145	0.449
Zyx	Zyxin	Q62523	S272	FSPGAPSGPGPQPNQK	S2	1.090	0.154
Zyx	Zyxin	Q62523	S336	SPGGPGPLTLK	S1	1.174	0.091
Zyx	Zyxin	Q62523	Ambiguous	MVPPDAPSSVSTGSPQPPSFTYAQQK	S/T/Y	0.993	0.904
Zzef1	Zinc finger ZZ-type and EF-hand domain-containing protein 1	A0A140LJ04	S1464	TSSVVEEHFQGSASPTEAATPAAGDR	S3	0.993	0.929
Zzef1	Zinc finger ZZ-type and EF-hand domain-containing protein 1	A0A140LJ04	S1515	LLPSSGPCVAEVSTAEEPSPPSTPTR	S19	1.024	0.600
Zzef1	Zinc finger ZZ-type and EF-hand domain-containing protein 1	A0A140LJ04	S1538	SMEETRPVPTVK	S1	1.232	0.036
Zzef1	Zinc finger ZZ-type and EF-hand domain-containing protein 1	A0A140LJ04	S1538	SMEETRPVPTVK	S1	1.300	0.001

Zzef1	Zinc finger ZZ-type and EF-hand domain-containing protein 1	A0A140LJ04	S1488,T/S	TSSVVEEHFQGSASPTEAATPAAGDRSPALEIQPK	S27,T/S	1.301	0.087
Zzef1	Zinc finger ZZ-type and EF-hand domain-containing protein 1	A0A140LJ04	S1515,S/T	LLPSSGPCVAEVSTAEEPSPPSTPTR	S19,T/S	1.411	0.343
Zzef1	Zinc finger ZZ-type and EF-hand domain-containing protein 1	A0A140LJ04	Ambiguous	QRTSSVVEEHFQGSASPTEAATPAAGDR	S/T	0.947	0.458
Zzef1	Zinc finger ZZ-type and EF-hand domain-containing protein 1	A0A140LJ04	Ambiguous	GDQEEELDRPVSSPGEAEQK	S	1.014	0.844

Supplemental Table S5. List of all mouse qPCR primers used

Gene	Forward primer	Reverse primer
C1qtnf6 (Ctrp6)	AGTCAGGCCGTACATCAACA	TCACCTTTGACACCCTGAGA
Adgre1	CTTTGGCTATGGGCTTCCAGTC	GCAAGGAGGACAGAGTTTATCGTG
Itgam	TGTAAAGCCTGCTGAGTCCA	GCAGTTTGTTCCCAAAGGAG
Cd68	TGTCTGATCTTGCTAGGACCG	GAGAGTAACGGCCTTTTTGTGA
Tnf-a	ATGCTGGGACAGTGACCTGG	CCTTGATGGTGGTGCATGAG
Ccl2	CAGCAAGATGATCCCAATGA	TGTCTGGACCCATTCCTTCT
Il6	TAGTCCTTCCTACCCCAATTTCC	TTGGTCCTTAGCCACTCCTTC
Il1b	TTGACGGACCCCAAAAGATG	AGAGGATGGGCTCTTCTTCA
Itgax	CTGGATAGCCTTTCTTCTGCTG	GCACACTGTGTCCGAACTCA
Nos2	GTTCTCAGCCCAACAATACAAGA	GTGGACGGGTCGATGTCAC
Il10	GCTCTTACTGACTGGCATGAG	CGCAGCTCTAGGAGCATGTG
Mgl2	GCATGAAGGCAGCTGCTATTGGTT	TAGGCCCATCCAGCTAAGCAGATT
Arg1	CTCCAAGCCAAAGTCCTTAGAG	AGGAGCTGTCATTAGGGACATC
Mrc1	CTCTGTTCAGCTATTGGACGC	CGGAATTTCTGGGATTCAGCTTC
Retnla (mouse)	CCAATCCAGCTAACTATCCCTCC	ACCCAGTAGCAGTCATCCCA
Cd3 (mouse)	AGCGGGATTCTGGCTAGTCT	TTGGTTGATCACAGGGGAAG
Cd4 (mouse)	TGAACCTGGTGGTGATGAAA	CTCCTGCTTCAGGGTCAGTC
Cd8 (mouse)	TCTGTCGTGCCAGTCCTTC	GCCGACAATCTTCTGGTCTC
mCd45 (mouse)	ATGTGGAGCCAATCCATTCT	GGCATCTTTGATGGGAAACT

Supplemental Table S6. Primary and secondary antibodies used for Immunoblotting and FACS analysis.

Antibody	Vendor	Catalog#	Dilution
GAPDH	Proteintech	60004-1-IG	1:20000
IκBα	Cell signaling technology	4812	1:1000
Phospho-JNK (Thr183/Tyr185)	Cell signaling technology	9251	1:1000
JNK	Cell signaling technology	9252	1:1000
Phospho-p44/42 MAPK (Thr202/Tyr204)	Cell signaling technology	4370	1:2000
p44/42 MAPK	Cell signaling technology	9102	1:1000
Phospho-p38 MAPK (Thr180/Tyr182)	Cell signaling technology	9216	1:1000
p38 MAPK	Cell signaling technology	9212	1:1000
Phospho-NF-κB p65 (Ser536)	Cell signaling technology	3033	1:1000
NF-κB p65	Cell signaling technology	8242	1:2000
CTRP6	Abcam	Ad36898	1:1000
Alexa Fluor™ 488 Rat Anti-Mouse F4/80	BD Biosciences	567201	1:20
PE Rat Anti-Mouse CD11b	eBioscience	12-0112-82	1:160

Secondary antibodies for Immunoblotting

Antibody	Vendor	Catalog#	Dilution
Anti-rabbit IgG, HRP-linked Antibody	Cell signaling technology	7074S	1:2000
Anti-mouse IgG, HRP-linked Antibody	Cell signaling technology	7076S	1:2000
