# Supplementary material for: CTRP6 promotes the macrophage inflammatory response, and its deficiency attenuates LPS-induced inflammation
Source: J Biol Chem. 2023 Dec 14;300(1):105566. doi: 10.1016/j.jbc.2023.105566 (PMC10789631; doi:10.1016/j.jbc.2023.105566)
Supplement: Supporting Figures S1 and S2 [file mmc2.pdf]

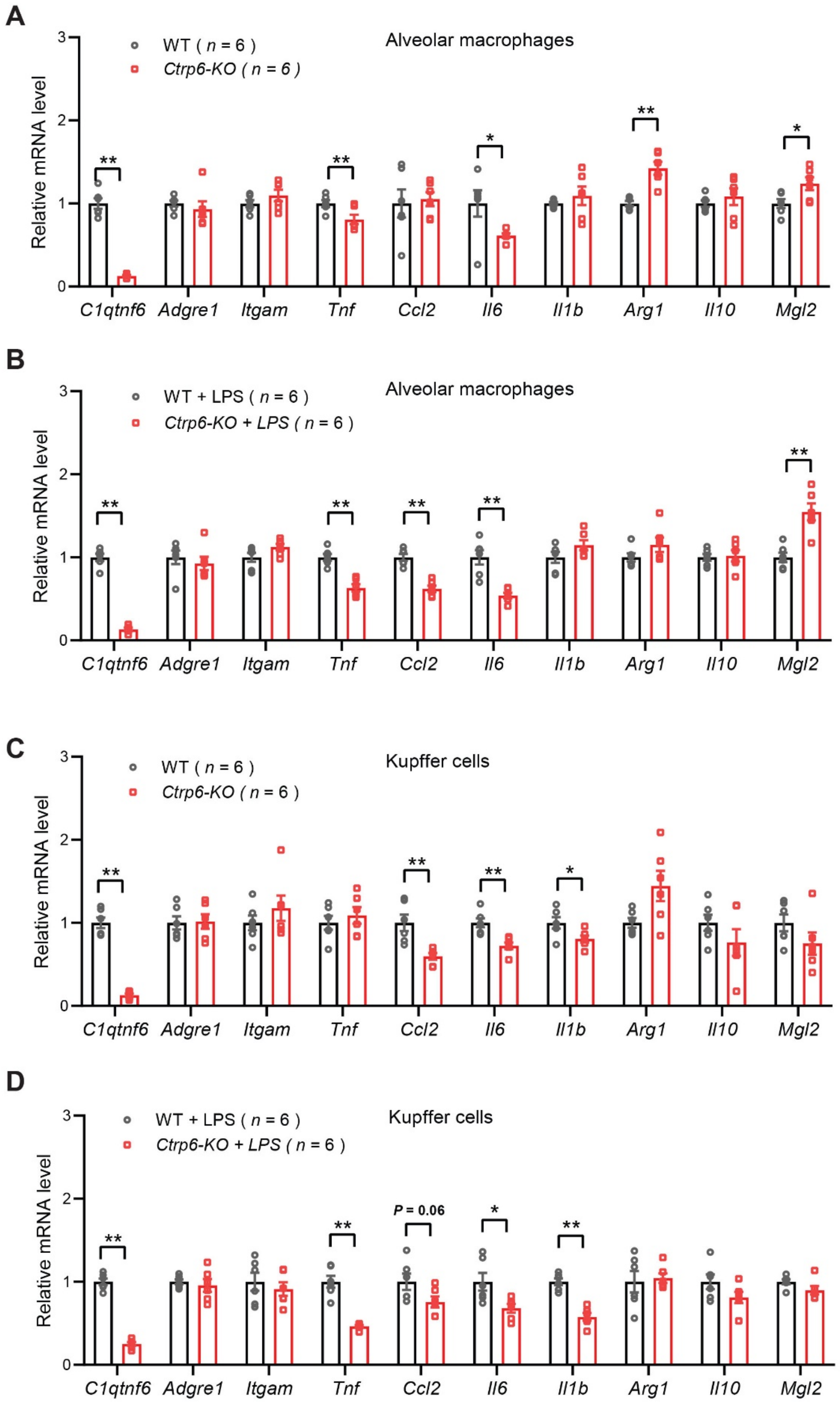

Supplementary Figure 1. Expression of proinflammatory and anti-inflammatory genes in alveolar macrophages isolated from the lung of WT and *Ctrp6*-KO mice at baseline (A) and after LPS treatment (B) for 24 h (n = 6). Expression of proinflammatory and anti-inflammatory genes in Kupffer cells isolated from the liver of WT and *Ctrp6*-KO mice at baseline (C) and after LPS treatment (D) for 24 h (n = 6). Data are shown as the mean ± SEM. Representative of 2 independent experiments. Two-tailed unpaired Student's t-test. * $P < 0.05$, ** $P < 0.01$.

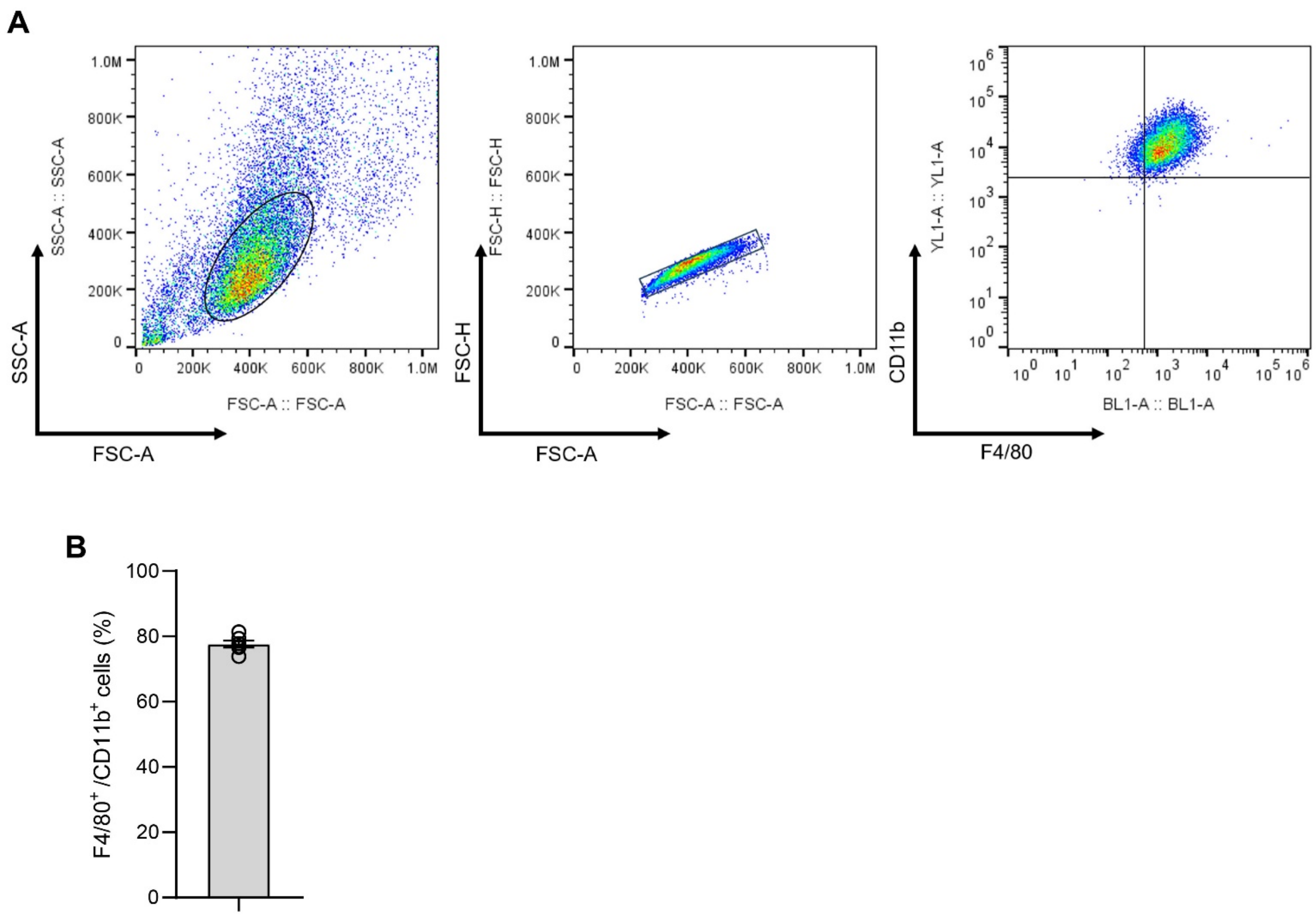

Supplementary Figure 2. Representative flow cytometry plot for expression of F4/80 and CD11b in BMDM after 6 days of differentiation. 20,000 cells were counted per sample. Bar graph shows the quantification of F4/80^{+}/ CD11b^{+} cells. Data are shown as the mean ± SEM. Representative of 2 independent experiments with 6 samples per group.
